# Supplementary material for: SAM-Competitive EZH2-Inhibitors Induce Platinum Resistance by EZH2-Independent Induction of ABC-Transporters
Source: Cancers (Basel). 2023 Jun 3;15(11):3043. doi: 10.3390/cancers15113043 (PMC10252553; doi:10.3390/cancers15113043)
Supplement: Supplementary file 1 [file cancers-15-03043-s001.zip › cancers-2406836-supplementary Table S5.pdf]

Supplemantary Table S5. Total raw count table from RNA sequencing.

	Jurkat n=1				Loucy n=1				DERL2 n=1				HH n=1				Jurkat n=2				Loucy n=2				DERL2 n=2				HH n=2			
Gene ID	untreated	GSK126	Oxaliplatin	Oxaliplatin+ GSK126	untreated	GSK126	Oxaliplatin	Oxaliplatin+ GSK126	untreated	GSK126	Oxaliplatin	Oxaliplatin+ GSK126	untreated	GSK126	Oxaliplatin	Oxaliplatin+ GSK126	untreated	GSK126	Oxaliplatin	Oxaliplatin+ GSK126	untreated	GSK126	Oxaliplatin	Oxaliplatin+ GSK126	untreated	GSK126	Oxaliplatin	Oxaliplatin+ GSK126	untreated	GSK126	Oxaliplatin	Oxaliplatin+ GSK126
ENSG00000223972	0	0	1	1	0	0	0	0	0	1	2	0	1	2	1	0	0	0	0	3	0	0	0	0	0	0	0	0	0	0	2	3
ENSG00000227232	170	130	142	144	226	187	169	243	161	118	480	83	185	171	173	210	64	48	36	112	266	153	167	139	79	142	117	320	80	149	93	167
ENSG00000278267	15	8	10	7	20	11	7	22	18	13	51	10	12	18	10	21	10	4	2	12	28	10	17	25	5	11	7	29	7	17	3	18
ENSG00000243485	1	3	4	3	12	6	3	6	0	1	1	1	1	1	1	2	1	0	1	1	10	8	2	3	0	0	1	0	0	1	0	3
ENSG00000237613	0	0	2	0	0	0	0	0	0	0	0	0	0	0	0	0	0	0	0	0	0	0	0	0	0	0	0	0	0	0	0	0
ENSG00000268020	0	0	0	0	0	0	0	0	0	0	0	0	0	0	0	0	0	0	0	0	0	0	0	0	0	0	0	0	0	0	0	0
ENSG00000240361	0	0	0	0	0	0	0	0	0	0	0	0	0	0	0	0	0	0	0	0	0	0	0	0	0	0	0	0	0	0	0	0
ENSG00000186092	0	0	0	0	0	0	0	0	0	0	0	0	0	0	0	0	0	0	0	0	0	0	0	0	0	0	0	0	0	0	0	0
ENSG00000238009	3	0	0	1	0	0	0	2	2	0	11	0	0	0	0	0	0	1	1	0	3	1	0	1	1	0	1	6	0	2	1	0
ENSG00000239945	0	0	0	0	0	0	0	0	0	0	0	0	0	0	0	0	0	0	0	0	0	0	0	0	0	0	0	0	0	0	0	0
ENSG00000233750	0	0	0	0	0	0	0	0	1	0	3	0	0	0	0	0	0	0	0	1	1	0	1	0	0	0	0	5	0	0	1	0
ENSG00000268903	4	0	0	0	1	0	0	0	0	0	0	0	0	1	0	1	0	0	0	0	0	0	0	0	0	0	0	0	0	0	1	0
ENSG00000269981	7	0	1	5	0	0	0	0	6	0	9	3	9	5	15	6	0	0	0	0	0	0	0	3	0	4	1	3	2	8	2	1
ENSG00000239906	0	0	0	0	0	0	0	0	1	0	0	0	0	0	1	0	0	0	0	0	0	0	0	0	0	0	0	0	0	0	0	1
ENSG00000241860	14	15	13	15	7	10	3	6	5	6	36	3	23	30	23	20	4	4	2	11	2	6	2	3	6	8	10	13	5	22	6	14
ENSG00000222623	0	0	0	0	0	0	0	0	0	0	0	0	0	0	0	0	0	0	0	0	0	0	0	0	0	0	0	0	0	0	0	0
ENSG00000241599	0	0	0	0	0	0	0	0	0	0	0	0	0	0	0	0	0	0	0	0	0	0	0	0	0	0	0	0	0	0	0	0
ENSG00000279928	1	0	0	0	8	3	2	5	1	0	3	0	1	5	3	3	0	1	0	0	6	4	7	0	1	0	0	4	2	0	6	3
ENSG00000279457	201	182	162	142	346	407	266	396	148	128	482	81	110	106	126	162	88	94	69	160	477	256	338	259	100	141	113	258	55	120	58	135
ENSG00000273874	0	0	0	0	0	0	0	0	0	0	2	0	0	0	0	0	0	0	0	0	0	0	1	0	1	0	0	1	0	0	0	0
ENSG00000228463	31	21	19	26	30	20	12	29	5	1	6	0	0	0	1	1	8	12	10	32	17	8	13	10	0	4	2	3	0	0	0	2
ENSG00000241670	0	0	0	0	0	0	0	0	0	0	0	0	0	0	0	0	0	0	0	0	0	0	0	0	0	0	0	0	0	0	0	0
ENSG00000236679	0	0	0	0	0	0	0	0	0	0	0	0	0	0	0	1	0	0	0	2	0	0	0	0	0	1	1	0	0	0	0	0
ENSG00000236743	0	0	0	0	0	0	0	1	0	0	0	0	0	0	0	0	0	0	0	0	0	0	0	0	0	0	0	2	0	0	0	0
ENSG00000236601	0	0	0	0	0	0	0	0	0	0	0	0	0	0	0	0	0	0	0	0	0	0	0	0	0	0	0	0	0	0	0	0
ENSG00000237094	31	18	17	37	7	6	9	5	12	11	62	7	11	9	8	17	1	3	1	16	24	9	7	10	7	7	11	26	5	3	3	10
ENSG00000269732	0	0	0	0	0	0	0	0	0	0	0	0	0	0	0	0	0	0	0	0	0	0	0	0	0	0	0	0	0	0	0	0
ENSG00000278566	0	0	0	0	0	0	0	0	0	0	0	0	0	0	0	0	0	0	0	0	0	0	0	0	0	0	0	0	0	0	0	0
ENSG00000224813	0	0	0	0	0	0	0	0	0	0	0	0	0	0	0	0	0	0	0	0	0	0	0	0	0	0	0	0	0	0	0	0
ENSG00000233653	0	0	0	0	0	0	0	0	0	0	1	0	0	0	0	0	0	0	0	0	0	0	0	0	0	0	0	1	0	0	0	0
ENSG00000250575	1	0	3	0	0	2	0	0	1	1	0	0	0	0	0	0	0	0	0	0	0	0	1	0	0	0	0	0	0	1	0	0
ENSG00000278757	0	0	0	0	0	0	0	0	0	0	0	0	0	0	0	0	0	0	0	0	0	0	0	0	0	0	0	0	0	0	0	0
ENSG00000230021	1	7	8	7	2	3	2	4	3	3	15	2	1	1	3	0	5	1	6	4	14	5	4	6	1	4	1	4	0	7	3	1
ENSG00000235146	0	0	0	1	0	0	0	0	0	0	0	0	0	0	0	0	0	0	0	0	0	0	0	0	0	0	0	0	0	0	0	0
ENSG00000225972	2	1	0	0	0	0	0	0	1	3	6	1	0	1	0	1	0	1	1	0	2	0	4	0	1	1	1	2	0	1	3	0
ENSG00000225630	56	55	57	48	137	129	125	136	74	57	272	45	131	130	115	161	99	57	59	79	310	106	155	91	39	97	52	155	79	172	120	234
ENSG00000237973	788	567	643	753	819	932	557	948	538	354	1831	305	588	625	577	789	1165	541	768	796	2232	639	1151	744	404	593	468	892	452	723	538	904
ENSG00000278791	0	0	0	0	0	0	0	0	0	0	0	0	0	0	0	0	0	0	0	0	0	0	0	0	0	0	0	0	0	0	0	0
ENSG00000229344	3	5	2	2	3348	2967	1907	3127	2	2	6	1	1	3	2	0	10	3	6	3	6992	2027	4006	2724	0	1	4	1	2	2	1	2
ENSG00000240409	2	1	2	0	0	1	2	0	0	0	2	1	0	2	1	3	0	0	0	0	4	1	0	1	0	2	1	2	0	1	0	2
ENSG00000248527	4859	4290	4329	3834	7534	7062	4394	7567	4004	2953	12716	2063	3348	3175	3256	4614	10403	7588	7728	7232	17699	5154	8570	6078	2613	4470	2656	7304	3050	4347	3134	6221
ENSG00000198744	2	3	3	1	7	3	2	1	2	1	9	0	0	1	1	6	2	6	0	2	8	4	6	7	2	0	0	2	2	3	0	2
ENSG00000268663	0	0	0	0	0	0	0	0	0	0	1	0	0	0	0	0	0	1	0	0	0	0	0	0	0	0	0	0	0	0	0	0
ENSG00000273547	0	0	0	0	0	0	0	0	0	0	0	0	0	0	0	0	0	0	0	0	0	0	0	0	0	0	0	0	0	0	0	0
ENSG00000283574	0	0	0	0	0	0	0	0	0	0	0	0	0	0	0	0	0	0	0	0	0	0	0	0	0	0	0	0	1	0	0	0
ENSG00000229376	1	4	4	0	7	0	7	4	19	13	78	11	5	3	6	3	3	0	3	6	7	3	3	2	7	11	19	34	1	7	8	8
ENSG00000228327	352	340	399	295	195	302	249	299	310	319	1213	192	337	313	301	404	76	75	103	308	443	257	254	241	209	331	268	689	110	350	163	366
ENSG00000223181	2	3	2	0	0	1	4	2	0	1	2	1	2	1	4	0	1	0	0	1	1	3	0	2	0	2	0	6	0	0	0	3
ENSG00000229905	1	0	3	0	0	0	2	0	1	1	2	0	1	0	2	0	0	0	0	5	1	0	1	0	4	1	2	3	0	2	0	0
ENSG00000237491	320	226	253	171	144	141	88	152	161	142	714	95	130	113	124	112	99	85	72	286	211	93	87	98	223	248	176	573	29	95	60	95
ENSG00000230092	41	30	52	28	16	18	15	27	37	36	133	28	9	19	8	11	23	23	25	43	37	23	18	20	24	45	29	104	4	14	10	10
ENSG00000177757	37	25	39	30	13	16	6	17	31	37	126	16	9	15	7	9	10	26	14	22	12	11	7	12	18	37	38	97	0	13	3	13
ENSG00000228794	750	602	761	602	479	501	423	580	284	262	1180	187	411	358	426	465	372	474	391	776	1004	425	541	357	277	492	345	824	281	435	376	529
ENSG00000225880	75	60	61	35	44	41	27	39	47	47	228	33	35	40	49	40	17	31	20	40	37	29	22	21	46	80	28	143	13	28	35	44
ENSG00000230368	2	1	2	0	1	0	0	0	0	0	1	0	1	0	1	2	0	0	2	0	0	2	0	0	0	0	0	0	1	0	0	1
ENSG00000234711	0	1	0	0	0	0	0	0	0	0	1	0	0	0	0	0	0	0	0	0	0	0	0	0	0	0	0	0	0	1	0	1
ENSG00000283040	0	0	1	0	0	0	0	0	0	0	0	0	0	0	0	0	0	0	0	0	0	0	0	0	0	0	0	0	0	0	0	0
ENSG00000272438	81	126	132	116	65	89	79	117	55	62	156	32	4	4	5	4	10	23	24	110	118	73	79	68	27	34	42	74	1	2	0	4
ENSG00000230699	410	493	572	613	309	448	398	460	333	397	780	143	13	12	12	23	79	106	79	474	458	274	294	302	100	231	162	453	3	14	2	21
ENSG00000241180	29	30	36	35	25	34	29	28	19	29	66	11	0	3	0	1	4	7	7	30	27	11	13	19	6	16	16	23	0	1	0	1
ENSG00000223764	122	222	192	213	478	620	458	638	1030	1170	4156	678	16	30	21	23	30	30	40	158	622	364	438	396	561	1191	1385	2506	4	25	4	26
ENSG00000187634	9	13	19	14	392	484	310	371	982	1080	3745	749	24	62	37	91	0	5	3	10	443	238	323	180	705	1550	1214	2669	55	126	52	121
ENSG00000188976	2878	2289	2663	2571	2931	3113	2258	3195	3120	2649	10009	1950	3761	3486	4034	4790	876	1354	1102	2520	4537	2201	3577	1961	2599	4629	2208	6365	3244	3974	3371	4109
ENSG00000187961	1279	1332	1273	1376	476	583	410	547	410	363	1517	281	129	141	171	248	665	578	513	1552	715	351	368	300	218	500	390	901	51	166	73	218
ENSG00000187583	9	8	4	11	7	19	5	9	3	11	31	9	2	0	3	0	0	1	1	10	14	4	8	9	8	8	15	16	1	0	0	2
ENSG00000187642	3	5	10	11	10	7	4	9	2	4	18	4	0	0	2	1	0	2	2	9	10	4	6	3	2	9	8	8	0	0	0	0
ENSG00000272512	62	86	89	98	15	21	8	37	1	18	69	14	2	2	3	9	34	36	25	158	24	19	16	14	5	23	16	98	0	0	0	2
ENSG00000188290	1901	1956	1648	1982	683	731	419	459	9	40	145	66	31	50	36	54	658	1789	1099	1922	1222	474	854	264	7	77	38	216	3	20	13	21
ENSG00000187608	697	462	547	422	723	592	450	640	108	131	839	117	532	526	619	691	973	1132	1152	714	1692	557	1161	474	205	271	192	599	881	781	1015	1069
ENSG00000231702	3	2	6	2	6	3	4	2	0	0	3	0	1	0	0	0	0	0	0	1	3	2	4	1	0	0	1	3	0	0	0	1
ENSG00000224969	9	7	10	16	7	12	5	17	0	0	8	0	0	1	2	5	5	11	6	11	13	7	8	11	0	1	0	9	1	1	0	4
ENSG00000188157	1929	2616	2049	2589	2349	2821	1696	2603	1792	2670	17327	2633	1463	1736	1174	2249	307	418	214	1372	2544	1214	1534	1156	1460	3465	4933	10318	431	891	326	1054
ENSG00000242590	0	0	0	0	0	0	0	0	0	0	0	0	0	0	0	0	0	0	0	0	0	0	0	0	0	0	0	0	0	0	0	0
ENSG00000217801	49	70	54	86	27	25	12	28	787	1112	8344	875	371	466	466	612	18	10	17	43	53	19	34	29	873	1756	3702	5465	207	284	272	401
ENSG00000273443	0	14	1	12	9	11	13	18	335	399	3149	353	108	122	110	155	0	1	0	1	14	8	15	8	264	607	1408	2172	22	70	39	124
ENSG00000237330	0	1	0	0	1	0	2	1	61	96	681	40	10	19	22	32	0	0	1	0	1	0	0	0	27	96	250	404	4	10	2	8
ENSG00000131591	689	594	647	613	485	580	374	635	711	831	4138	593	264	327	273	360	297	293	238	612	845	383	585	390	565	1051	1208	2725	201	289	178	376
ENSG00000223823	1	1	1	0	0	0	0	0	1	1	0	0	130	85	139	123	0	0	0	1	0	0	0	3	0	0	0	0	43	76	58	90
ENSG00000207730	0	0	0	0	0	1	0	0	0	0	1	0	4	4	6	8	0	0	0	0	0	0	0	0	0	0	0	0	0	5	1	8
ENSG00000207607	0	0	0	0	0	0	0	0	0	0	0	0	7	3	7	8	0	0	0	0	1	0	0	0	0	0	0	3	0	2	1	3
ENSG00000198976	0	0	0	0	0	0	0	1	0	2	2	0	9	7	9	10	0	0	0	0	0	0	0	0	0	0	1	3	0	5	6	3
ENSG00000272141	0	0	0	0	1	0	1	1	1	0	4	2	12	22	26	35	0	0	0	0	3	3	3	2	0	5	0	3	8	30	13	33
ENSG00000205231	0	0	0	0	0	0	0	0	0	1	3	0	18	17	19	21	0	0	0	0	0	0	0	0	1	7	1	5	2	7	3	14
ENSG00000162571	0	0	0	1	0	0	0	0	14	14	107	9	17	8	14	24	0	0	0	0	0	0	0	0	5	16	12	72	3	19	6	10
ENSG00000186891	0	1	0	1	23	22	22	31	1677	2217	13699	2292	292	276	324	337	0	0	0	0	44	19	33	21	1342	3251	3067	7771	120	192	139	216
ENSG00000186827	74	68	68	104	116	130	92	136	113	198	854	184	1397	1174	1679	1487	48	130	38	147	160	111	138	90	76	130	201	362	589	622	833	781
ENSG00000078808	2100	1848	2008	1734	3231	3494	2318	3523	2216	1969	12397	2003	2348	2178	2645	2879	630	1041	1001	1698	4426	1877	3265	1486	1854	3608	2957	7213	2168	2641	2221	2872
ENSG00000176022	712	599	658	622	619	621	432	633	523	490	2810	457	419	399	381	552	534	937	764	1031	1346	540	1022	420	458	809	618	1493	357	460	304	418
ENSG00000184163	40	39	19	26	17	16	11	22	25	15	152	11	42	46	26	47	9	6	8	27	9	11	18	3	17	28	33	70	6	24	15	40
ENSG00000260179	11	8	9	14	8	9	10	15	22	18	97	10	15	23	20	17	0	2	2	6	17	2	8	9	10	16	19	32	4	17	9	17
ENSG00000160087	1488	1018	1328	1154	1236	1340	962	1373	1068	977	5309	795	1139	937	1293	1423	424	672	666	1110	2164	965	1512	841	1047	1803	1277	3272	972	1202	1055	1303
ENSG00000230415	103	115	121	135	30	45	23	32	32	22	175	26	20	19	15	25	30	25	26	133	32	25	25	22	12	29	57	117	4	7	5	10
ENSG00000162572	562	703	640	685	139	193	139	206	129	177	897	154	107	103	85	168	137	138	143	557	156	94	112	93	56	137	247	466	13	68	32	72
ENSG00000131584	2088	2221	2292	2218	1235	1516	1127	1592	1994	2466	16186	2103	504	514	556	707	747	672	653	2098	1618	932	1234	891	1341	3057	5581	8917	291	610	265	681
ENSG00000278073	0	0	0	0	0	0	0	0	0	0	1	0	0	0	0	0	0	0	0	0	0	0	0	0	0	0	0	0	0	0	0	0
ENSG00000169972	505	378	518	426	303	286	242	309	245	223	1107	253	207	187	187	251	386	602	489	587	568	240	481	214	309	487	344	924	205	241	187	308
ENSG00000127054	2615	2174	2237	2191	2480	2502	1779	2546	2264	2013	8917	1601	1909	1898	1870	2454	938	1148	1035	2036	3409	1552	2412	1388	1792	3019	2289	5576	1320	1858	1374	2058
ENSG00000240731	82	96	96	97	79	82	80	104	103	85	438	65	61	52	53	77	44	44	42	122	148	64	112	59	71	105	117	279	40	58	25	68
ENSG00000224051	225	179	243	188	307	281	196	355	252	221	1268	206	183	147	177	252	79	70	122	148	365	154	296	153	226	377	319	778	144	170	149	199
ENSG00000169962	25	11	26	8	46	34	40	67	20	20	153	7	20	18	17	19	3	7	3	25	60	25	46	28	19	22	43	133	11	18	9	23
ENSG00000107404	2189	1831	1738	1858	1091	1190	796	1182	1212	1140	5636	1088	1028	938	841	1167	991	1419	1024	1870	1721	740	1190	719	872	1827	1636	3393	846	990	677	897
ENSG00000162576	193	210	289	260	217	316	218	302	138	178	1005	116	29	37	26	48	60	76	74	297	344	175	195	209	51	139	274	544	2	32	9	25
ENSG00000175756	1742	1207	1557	1298	1656	1538	1008	1615	1156	884	3801	784	1350	1184	1349	1609	1078	1725	1647	1694	2891	1236	2342	928	1091	1937	880	2661	1509	1482	1621	1757
ENSG00000223663	1	1	0	0	5	0	0	1	0	2	6	0	5	1	0	2	3	0	0	1	5	2	2	2	1	0	2	0	1	0	0	0
ENSG00000221978	4384	3226	3441	3255	4513	4949	3549	5195	2482	2117	9460	1596	1898	1732	1798	2413	1334	1090	881	2319	6031	2909	3433	2860	1788	2980	2422	5486	919	1705	973	1924
ENSG00000224870	814	522	785	660	543	669	502	670	714	578	2936	491	383	368	389	493	371	470	454	658	1004	461	832	420	633	1095	673	1782	338	382	344	410
ENSG00000242485	1866	1206	1492	1298	1082	1075	818	1118	1232	812	4240	766	1220	1024	1336	1399	3158	3855	3708	3170	2988	1167	2157	1226	1224	1836	911	2729	1869	1646	2226	2126
ENSG00000264293	0	0	0	0	0	0	0	0	0	0	0	0	0	0	0	0	0	0	0	0	0	0	0	0	0	0	0	0	0	0	0	0
ENSG00000272455	136	117	115	86	85	86	67	96	121	86	457	68	94	106	114	137	60	71	93	145	206	72	119	79	87	159	82	227	83	113	91	111
ENSG00000235098	2	0	2	0	1	3	0	2	0	0	18	4	29	21	31	52	0	1	0	2	1	3	3	0	0	0	3	3	29	40	23	56
ENSG00000225905	0	1	0	0	1	0	0	1	0	0	0	0	3	0	0	8	0	0	0	0	0	0	0	0	0	0	0	0	0	0	0	3
ENSG00000205116	0	0	1	0	0	0	0	0	0	1	3	0	72	64	30	74	0	0	0	0	0	0	2	0	0	0	0	3	21	22	9	20
ENSG00000225285	4	2	1	3	0	0	1	2	0	0	2	0	6	1	3	1	0	1	0	1	0	0	1	0	0	0	0	0	1	0	0	1
ENSG00000179403	13	14	6	5	0	0	1	0	37	58	100	51	8	24	6	21	0	5	2	2	0	1	1	4	15	39	33	86	2	10	1	2
ENSG00000215915	1	1	1	4	0	1	0	1	0	2	8	1	4	2	0	1	1	3	0	2	0	0	2	0	0	2	6	3	0	0	2	0
ENSG00000160072	897	677	791	728	1087	1227	1056	1318	1359	1037	5378	958	1504	1066	1619	1553	429	546	612	1095	2204	1150	1441	1183	1275	2212	1009	3291	1501	1816	1714	2047
ENSG00000197785	1934	1217	1754	1361	1958	1871	1489	1977	1675	1174	4856	1058	2984	2217	2538	2862	886	1209	1147	1925	3299	1413	2677	1345	1597	2744	946	3672	2620	2698	2058	2662
ENSG00000205090	8	7	7	3	12	1	5	7	14	11	73	11	8	10	11	3	3	1	2	8	14	8	6	13	6	25	3	26	1	14	0	14
ENSG00000160075	1668	1088	1286	1098	1451	1479	1082	1423	1828	1323	6725	1043	1715	1573	1663	1931	1090	1236	1232	1431	2720	1156	2055	1020	1498	2544	1592	4081	1662	2065	1646	2028
ENSG00000215014	41	40	35	40	23	13	7	8	12	16	36	2	13	18	17	13	17	20	15	36	12	6	17	5	11	19	4	15	10	12	12	10
ENSG00000279244	0	1	0	0	0	0	0	0	0	0	4	0	0	0	0	0	0	0	0	0	0	0	0	0	0	0	0	0	0	0	0	0
ENSG00000274481	0	0	0	0	0	0	0	0	0	0	0	0	0	0	0	0	0	0	0	0	0	0	0	0	0	0	0	0	0	0	2	0
ENSG00000228594	99	158	94	131	0	0	0	0	135	179	414	108	155	169	79	189	22	89	45	84	0	0	0	0	112	195	92	263	55	95	41	86
ENSG00000272106	90	44	89	63	73	67	51	86	123	89	450	77	79	54	63	60	25	39	27	54	90	31	76	35	86	119	139	220	46	61	31	61
ENSG00000197530	1044	861	1125	952	1009	1262	831	1236	1443	1208	7733	1172	404	458	499	661	403	529	528	1071	1597	777	1210	712	1145	1958	2010	4194	288	471	320	593
ENSG00000189409	12	4	26	11	6	12	7	7	33	19	196	27	0	2	0	0	5	4	5	20	18	4	10	2	43	54	37	83	0	5	0	1
ENSG00000248333	2313	1531	2020	1662	1398	1429	1178	1584	1196	846	4501	713	1548	1351	1765	1906	776	829	801	1391	2255	1133	1764	949	973	1702	1002	2811	1187	1415	1092	1562
ENSG00000272004	5	0	9	3	0	0	0	1	2	5	18	4	5	0	0	7	2	5	2	5	3	2	5	3	6	7	6	11	0	1	0	1
ENSG00000189339	1897	1517	1891	1606	1332	1367	1004	1469	2447	2215	11229	1747	1584	1420	1311	1834	392	557	495	1139	2009	905	1376	825	1695	2776	3364	6050	929	1239	729	1386
ENSG00000269737	104	105	124	90	86	100	80	128	82	97	500	57	49	42	60	58	31	24	20	119	140	74	78	68	53	99	182	289	46	72	27	77
ENSG00000215914	6	9	7	4	10	18	15	11	2	0	9	2	3	2	4	5	4	1	3	11	21	9	10	8	0	1	1	3	1	2	1	3
ENSG00000008128	665	474	625	521	494	540	434	567	441	336	1776	276	281	225	287	278	309	274	229	511	764	415	483	397	337	581	473	971	173	317	211	341
ENSG00000268575	200	137	201	153	186	177	151	182	159	155	691	92	79	58	57	68	40	37	22	96	187	94	134	95	90	137	173	327	12	57	37	60
ENSG00000227775	440	328	408	327	547	531	413	578	274	251	1327	182	73	77	60	111	111	100	86	264	792	351	471	264	166	274	330	577	34	75	32	75
ENSG00000215790	251	248	247	199	169	201	150	237	325	300	1480	240	81	76	45	89	58	87	49	147	261	117	159	87	205	320	375	713	30	67	29	77
ENSG00000008130	1802	1306	1635	1322	1902	1947	1456	2041	1415	1203	5458	1030	1285	1225	1195	1526	802	911	878	1639	3302	1486	2477	1265	1320	2355	1154	3649	970	1280	854	1337
ENSG00000078369	10873	7856	9954	7967	11572	11181	7827	11049	8615	6846	34034	5731	6001	5206	5669	6670	2510	4439	5020	6290	16780	6619	12257	5619	7946	12611	7811	21220	4282	5290	4276	5537
ENSG00000231050	9	10	13	6	8	8	12	7	3	4	16	5	2	5	3	5	7	7	7	10	19	6	4	8	3	11	0	26	1	3	3	3
ENSG00000169885	6	1	5	5	8	10	11	13	8	5	186	10	12	7	8	9	3	3	2	9	24	14	9	17	1	11	42	50	4	4	3	6
ENSG00000178821	31	62	33	51	0	0	0	1	8	4	18	3	22	36	29	58	15	25	16	40	0	1	0	0	11	12	2	14	16	14	13	35
ENSG00000142609	0	0	0	2	1	1	0	0	2	0	3	1	49	47	30	56	0	0	0	0	0	0	0	0	0	0	0	3	11	31	13	45
ENSG00000233542	0	0	0	0	0	0	0	0	0	0	1	0	0	0	0	0	0	0	0	0	0	0	0	0	0	0	0	0	0	0	0	0
ENSG00000187730	6	3	5	1	0	0	0	0	0	0	1	1	0	0	0	0	5	3	12	8	0	0	0	0	0	1	0	0	0	0	0	0
ENSG00000226969	0	0	0	0	0	0	0	0	0	0	0	1	0	2	0	0	0	0	0	0	0	0	0	0	0	0	0	0	0	1	3	0
ENSG00000067606	4	10	5	10	32	35	23	30	181	198	818	128	146	160	144	219	1	2	3	12	59	31	49	35	136	237	229	557	107	173	118	186
ENSG00000271806	2	1	0	2	1	2	2	1	9	5	35	12	5	4	1	3	0	1	0	0	10	2	5	1	6	9	7	34	1	6	4	3
ENSG00000182873	59	38	32	39	11	24	15	17	53	51	198	43	27	23	19	40	3	16	4	29	40	18	15	18	27	66	47	125	9	28	10	27
ENSG00000162585	1085	893	1040	960	917	1055	652	959	743	620	2840	503	398	342	342	513	551	712	682	1094	1645	746	1195	722	553	913	596	1562	288	380	283	496
ENSG00000234396	0	2	0	0	0	0	0	0	0	0	0	0	0	0	0	0	0	0	0	0	0	0	0	0	0	0	0	0	0	0	0	0
ENSG00000157933	1153	1006	1310	1114	1116	1183	863	1200	1296	1138	4850	849	1027	925	996	1288	288	488	487	832	2029	940	1382	698	1047	2057	1678	3510	771	1029	688	1128
ENSG00000116151	248	193	213	199	91	106	65	102	197	175	644	120	160	136	137	240	75	87	78	187	113	69	96	77	120	192	137	431	52	142	65	138
ENSG00000272161	0	0	0	0	0	1	0	0	3	1	5	0	0	0	0	0	0	0	0	1	0	0	1	0	1	0	0	1	0	0	0	0
ENSG00000269896	7	10	9	11	13	14	7	20	37	46	123	33	36	26	29	44	2	3	5	10	35	20	20	6	27	49	31	92	15	28	2	28
ENSG00000272420	18	7	11	11	3	4	10	6	7	7	26	4	4	7	2	5	1	1	1	4	12	4	2	4	6	12	7	14	1	6	1	6
ENSG00000157916	1748	1224	1503	1251	1627	1739	1442	1817	1308	1116	5418	910	1105	967	1190	1458	1209	1765	1731	1652	3268	1375	2356	1321	1190	1863	1224	3513	1162	1342	1380	1556
ENSG00000157911	396	241	317	268	286	351	283	370	405	291	1111	296	314	313	298	429	248	366	281	454	701	334	582	304	321	496	212	773	366	482	300	442
ENSG00000149527	11	6	4	7	79	93	77	85	37	31	126	24	11	25	7	15	1	2	0	3	133	61	91	83	19	46	35	191	10	19	17	37
ENSG00000224387	0	0	1	1	1	0	2	2	0	0	10	1	0	0	0	0	0	0	0	0	3	3	1	1	0	4	0	3	0	0	0	0
ENSG00000229393	0	1	0	0	0	1	1	2	1	0	10	0	1	0	0	0	0	0	0	0	6	0	1	3	0	0	0	5	0	3	0	1
ENSG00000157881	983	749	796	747	841	886	593	914	489	448	1816	347	442	405	365	494	327	388	369	528	1228	539	942	485	459	773	456	1382	230	356	222	421
ENSG00000197921	0	0	1	0	0	0	0	0	43	37	209	24	0	1	0	0	0	0	0	1	0	0	0	0	46	73	30	149	0	1	0	0
ENSG00000272449	69	51	33	56	42	30	23	42	147	126	639	116	30	36	22	46	29	33	33	27	69	38	50	25	104	192	104	267	38	49	21	47
ENSG00000238164	52	46	69	38	129	151	107	178	208	179	575	110	180	174	227	223	20	12	22	37	160	107	101	103	67	112	86	242	69	167	101	189
ENSG00000157873	440	335	485	267	698	824	562	813	729	725	2775	521	421	480	495	623	194	93	183	301	961	544	661	562	356	670	448	1169	250	439	278	602
ENSG00000225931	4	1	1	0	8	6	8	17	13	11	16	8	27	34	20	26	0	0	1	0	5	4	5	5	3	3	4	7	6	22	4	23
ENSG00000228037	3	5	2	3	4	5	1	5	3	4	21	1	41	33	30	52	3	1	1	9	4	2	5	5	1	2	2	13	12	28	21	41
ENSG00000157870	604	533	630	600	598	660	500	639	1046	919	2666	778	672	721	759	999	167	377	349	657	1082	403	831	413	707	1538	325	1585	553	736	563	903
ENSG00000142606	0	0	1	1	1	1	2	1	26	24	42	24	1	1	0	2	0	0	0	2	2	1	0	0	8	14	1	9	0	1	1	2
ENSG00000237058	0	0	0	0	0	0	0	1	0	0	0	0	0	0	0	0	0	0	0	0	0	0	0	0	0	0	0	0	0	0	0	0
ENSG00000215912	88	75	101	87	29	21	16	30	20	13	46	4	5	9	5	13	49	43	39	114	28	25	23	23	8	19	7	34	5	9	5	9
ENSG00000283259	0	0	0	0	0	0	0	0	0	0	0	0	0	0	0	0	0	0	0	0	0	0	0	0	0	0	0	0	0	0	0	0
ENSG00000233234	0	0	0	0	0	0	0	0	0	0	0	0	0	0	0	0	0	0	0	0	0	0	0	0	0	0	0	0	0	0	0	0
ENSG00000231630	0	0	0	0	0	0	0	0	0	0	0	0	0	0	0	0	0	0	0	0	1	0	0	0	0	0	0	0	0	0	0	0
ENSG00000169717	0	0	0	0	0	0	0	0	0	0	0	0	0	0	0	0	0	0	0	0	0	0	0	0	0	0	0	0	0	0	0	0
ENSG00000177133	0	0	0	0	61	68	54	89	0	0	0	0	0	0	0	0	0	0	0	0	86	50	65	49	0	0	0	0	0	0	0	0
ENSG00000142611	17	28	12	32	517	674	358	713	0	4	1	1	0	0	0	0	2	4	1	4	653	382	440	328	0	0	0	1	0	0	0	0
ENSG00000283572	0	0	0	0	1	1	2	2	0	0	0	0	0	0	0	0	0	0	0	0	0	0	0	1	0	0	0	0	0	0	0	0
ENSG00000226286	0	0	0	0	2	4	3	5	0	0	0	0	0	0	0	0	0	0	0	0	0	0	2	4	0	0	0	0	0	0	0	0
ENSG00000279839	0	0	1	1	4	4	4	10	0	1	0	0	0	0	0	0	0	0	0	0	13	10	7	9	0	0	1	0	0	0	0	0
ENSG00000272235	0	0	0	0	6	29	8	26	0	0	0	0	0	0	0	0	0	0	0	0	20	9	4	13	0	0	0	0	0	0	0	0
ENSG00000130762	2	49	8	51	126	128	121	149	1	5	7	4	4	15	4	18	1	35	4	41	237	123	144	115	0	1	0	4	3	18	1	14
ENSG00000272088	0	0	0	0	4	4	1	4	0	0	0	0	0	0	0	0	0	0	0	0	6	1	1	7	0	0	0	0	0	1	0	0
ENSG00000162591	4	34	5	30	1445	1676	1298	1567	34	33	106	34	85	101	14	119	0	7	0	15	1648	947	1219	978	9	6	12	66	13	65	6	37
ENSG00000207776	0	0	0	0	0	4	0	7	0	0	0	0	0	0	0	0	0	0	0	0	2	1	0	2	0	0	0	0	0	0	0	0
ENSG00000238260	29	24	22	28	9	8	11	8	7	11	90	11	5	6	4	5	8	3	4	15	12	7	5	6	3	9	15	23	3	0	3	9
ENSG00000158109	579	381	528	399	315	342	204	316	278	265	1546	215	284	259	242	367	189	290	298	336	476	223	444	183	285	463	532	962	266	300	246	305
ENSG00000116213	1066	1026	922	915	916	986	759	1030	829	690	3214	589	868	760	647	989	417	605	505	877	1443	687	1051	672	759	1078	758	2069	594	901	417	902
ENSG00000078900	0	1	3	3	0	0	0	0	1797	1323	4942	1030	130	168	71	187	1	0	0	0	0	0	0	0	1369	2312	892	4105	62	129	30	150
ENSG00000227589	0	0	0	0	0	0	0	0	73	45	143	37	3	0	1	2	0	0	0	1	0	0	0	0	37	76	42	113	1	2	0	4
ENSG00000235131	1	0	0	0	0	0	0	0	18	9	54	14	2	0	0	1	0	0	0	0	0	0	0	0	21	13	6	29	0	2	1	5
ENSG00000227372	727	643	738	750	0	0	0	0	10	12	26	3	258	225	256	368	226	228	249	660	0	0	0	0	7	6	5	33	107	219	118	266
ENSG00000276189	0	0	0	0	0	0	0	0	0	0	0	0	0	0	0	0	0	0	0	0	0	0	0	0	0	0	0	0	0	0	0	0
ENSG00000162592	3	1	6	6	0	0	0	0	1	2	1	1	4	5	7	4	2	0	2	1	0	1	0	1	0	0	2	1	0	2	2	0
ENSG00000235169	0	5	1	3	1	1	0	3	4	2	10	2	4	13	3	12	1	0	0	3	0	1	0	0	3	2	1	5	2	3	1	5
ENSG00000130764	1691	1108	1465	1180	1611	1548	1111	1564	1040	879	4407	806	1540	1258	1442	1500	635	788	796	1206	2715	1111	2108	881	954	1578	952	2576	1412	1480	1299	1513
ENSG00000266075	0	3	1	1	2	1	0	2	0	2	3	0	4	1	0	3	0	3	0	0	6	4	5	0	0	2	7	5	1	4	1	2
ENSG00000272153	65	61	78	82	61	40	43	52	47	23	169	36	26	30	54	60	22	26	20	80	95	52	56	61	34	42	29	88	28	45	31	47
ENSG00000116198	2181	1480	1916	1504	883	944	708	942	1029	883	4620	728	1003	941	1068	1146	601	782	736	1338	1346	643	1018	533	957	1540	1178	2894	862	1072	853	1100
ENSG00000169598	585	379	473	448	288	252	184	259	328	285	1202	191	399	322	368	387	225	370	287	461	436	149	296	138	312	486	302	754	422	394	337	395
ENSG00000198912	962	677	871	750	604	655	454	676	665	472	1852	375	550	472	559	633	638	846	702	754	1230	435	772	410	629	893	362	1362	432	528	453	580
ENSG00000236423	4	4	9	6	18	27	21	19	8	8	27	2	14	10	8	12	2	4	5	8	24	16	21	16	5	5	2	18	3	6	7	2
ENSG00000233304	0	0	0	0	1	1	0	1	0	0	0	0	0	0	0	0	0	0	1	0	0	0	0	0	0	0	0	0	0	0	0	0
ENSG00000226374	0	0	0	0	0	0	0	0	0	0	3	0	0	0	0	0	0	0	0	0	1	0	0	0	0	0	0	0	0	0	0	0
ENSG00000229280	0	0	0	0	0	0	0	0	0	0	0	0	0	0	0	0	0	0	0	0	0	0	0	0	0	0	0	0	0	0	0	0
ENSG00000235054	0	0	0	0	0	0	0	0	0	0	0	0	0	0	0	0	0	0	0	0	0	0	0	0	0	0	0	0	0	0	0	0
ENSG00000227169	0	0	0	0	0	0	0	0	0	0	0	0	0	0	0	0	0	0	0	0	0	0	0	0	0	0	0	0	0	0	0	0
ENSG00000232596	0	0	0	0	0	0	0	0	0	0	0	0	0	0	1	0	0	0	0	0	0	0	0	0	0	0	0	0	0	0	0	0
ENSG00000196581	0	0	0	0	0	0	0	0	0	0	0	0	0	0	0	0	0	0	0	0	0	0	0	0	0	0	0	0	0	0	0	0
ENSG00000231510	0	0	0	0	0	0	0	0	0	0	0	0	0	0	0	0	0	0	0	0	0	0	0	0	0	1	0	1	0	0	0	0
ENSG00000260972	0	0	0	0	0	0	0	0	0	0	0	0	0	0	0	0	0	0	0	0	0	0	0	0	0	0	0	0	0	0	0	0
ENSG00000283356	0	0	0	0	0	0	0	0	0	0	0	0	0	0	0	0	0	0	0	0	0	0	0	0	0	0	0	0	0	0	0	0
ENSG00000236948	0	0	0	0	0	0	0	0	0	0	0	0	0	0	0	0	0	0	0	0	0	0	0	0	0	0	0	0	0	0	0	0
ENSG00000264341	0	0	0	0	0	0	0	0	0	0	0	0	0	0	0	0	0	0	0	0	0	0	0	0	0	0	0	0	0	0	0	0
ENSG00000264101	0	1	3	0	0	1	0	0	0	0	1	0	0	1	0	0	0	0	0	0	0	0	0	0	0	0	0	2	0	2	0	0
ENSG00000131697	1820	1709	1882	1747	623	833	530	743	1062	942	2987	644	570	602	528	818	461	563	512	1449	784	438	548	439	644	1233	620	2227	309	506	346	610
ENSG00000069424	18615	15323	16474	15488	8741	8813	6253	9490	6915	5867	18133	4320	6339	5530	4841	6710	3568	5581	4202	9670	8198	3878	6206	3520	3946	7805	4227	13082	3358	4044	2734	4502
ENSG00000116254	15	33	17	26	121	133	123	158	371	267	439	149	98	69	84	110	6	5	12	21	271	112	174	131	68	115	46	179	24	67	34	64
ENSG00000116251	10518	6890	7419	7216	7033	7294	5266	7856	11922	7754	28075	5922	11538	11408	12069	14325	11567	12248	13714	12337	19077	7665	14043	9905	8953	12688	5372	17737	13540	12721	16178	16625
ENSG00000226944	0	0	0	1	0	0	0	1	14	9	32	5	0	2	3	2	0	0	0	0	0	0	0	0	3	7	7	14	1	4	1	2
ENSG00000158286	4	4	9	9	10	9	11	9	509	422	1381	302	32	44	35	49	3	8	5	6	22	5	6	2	204	419	185	599	20	40	16	57
ENSG00000116237	2925	2115	2476	2113	2588	2567	1923	2731	2591	2097	9472	1811	3239	3071	3234	4134	1780	2813	2636	2354	5666	2011	4136	1585	3162	4965	1894	7571	3554	3869	3454	3931
ENSG00000225077	140	107	159	114	33	26	30	41	17	12	79	18	25	34	37	39	72	136	243	163	72	46	88	40	13	26	16	45	30	25	36	37
ENSG00000173673	0	0	0	0	1	1	1	2	0	0	0	0	0	0	0	0	0	0	0	0	2	0	0	0	0	0	0	0	0	2	0	0
ENSG00000158292	509	564	560	559	175	235	134	248	29	63	198	69	373	294	248	386	87	160	141	321	270	110	214	117	14	35	65	235	125	183	89	205
ENSG00000097021	4137	2915	3445	2990	2152	2084	1640	2069	1267	954	3583	880	2584	2177	2213	2800	1084	1804	1819	2613	3382	1405	2543	1146	1323	2177	761	3563	2030	2401	1896	2599
ENSG00000271746	0	0	0	0	0	0	0	0	0	0	0	0	0	0	0	0	0	0	0	0	0	0	0	0	0	0	0	0	0	0	0	0
ENSG00000069812	0	4	1	0	0	0	0	0	0	0	45	15	2	4	3	17	0	1	0	0	0	0	0	0	1	0	5	49	1	3	0	1
ENSG00000187017	17	115	32	92	46	50	35	63	1	1	5	2	685	820	610	1032	5	34	3	45	46	14	23	21	0	2	3	6	250	341	222	365
ENSG00000265392	0	0	0	0	0	0	0	0	0	0	0	0	0	0	0	2	0	0	0	0	0	0	0	0	0	0	0	0	0	0	0	0
ENSG00000231868	1	4	2	0	0	1	0	0	0	0	0	0	3	13	2	6	0	0	0	2	1	0	3	0	0	0	2	0	0	5	2	2
ENSG00000215788	21	26	28	20	490	537	387	514	2	3	46	10	27	26	17	38	3	6	7	24	836	377	594	401	6	11	49	45	8	8	5	10
ENSG00000171680	69	114	84	105	467	615	427	624	23	18	243	25	16	11	12	15	8	35	27	76	752	394	512	411	17	53	188	233	0	4	1	3
ENSG00000162408	1367	955	1269	1075	1407	1374	1085	1510	1205	870	4342	603	1087	951	1206	1209	415	556	586	1172	2610	1064	1901	997	1075	1585	896	2817	981	1386	1059	1322
ENSG00000253022	3	1	1	3	1	1	2	2	0	1	12	0	1	2	4	1	1	2	0	3	3	1	2	5	1	3	3	8	2	0	0	0
ENSG00000229519	19	14	22	20	41	24	25	20	37	25	131	10	16	12	11	15	5	5	4	22	61	23	42	41	18	27	38	80	10	23	13	17
ENSG00000173662	3	2	4	6	1	0	0	0	2	0	10	0	1	0	2	0	0	2	0	2	3	0	2	3	1	0	1	2	0	0	1	0
ENSG00000204859	671	513	625	531	430	503	353	530	524	429	2378	340	323	298	430	457	264	286	289	576	711	315	534	309	347	603	555	1280	280	438	375	461
ENSG00000162413	1769	1226	1633	1254	1160	1100	890	1049	742	567	3843	568	691	618	721	833	515	766	882	1712	2382	919	1598	858	788	1328	1149	2541	721	868	695	1017
ENSG00000116273	1194	922	1105	981	1058	1119	830	1235	474	380	1510	316	288	298	295	404	235	333	330	539	1639	729	1181	576	441	707	372	1286	205	280	168	343
ENSG00000041988	421	341	382	270	244	290	209	274	228	176	754	135	249	232	212	300	330	498	384	328	493	152	316	157	166	254	185	460	192	213	237	216
ENSG00000007923	1723	1250	1641	1266	1627	1773	1340	1813	1378	1018	4395	745	1697	1543	1884	2022	657	867	857	1542	2831	1239	1921	1103	1151	1930	966	2799	1440	1960	1538	2132
ENSG00000228750	0	0	0	0	0	0	0	0	0	0	0	0	0	0	0	1	0	0	0	0	0	0	0	0	0	0	1	0	0	1	0	0
ENSG00000237436	23	18	16	14	9	11	6	6	5	8	34	5	7	11	6	8	7	17	17	15	30	8	12	7	7	12	4	11	6	11	10	12
ENSG00000171735	1051	684	770	763	788	745	561	845	488	371	1415	265	557	710	535	762	853	1120	788	871	1533	604	1129	689	505	635	264	966	449	548	486	674
ENSG00000227950	7	4	1	1	1	1	2	8	4	3	11	0	2	2	1	4	0	0	0	1	0	5	3	7	3	2	1	11	0	0	2	2
ENSG00000237365	0	0	3	0	1	0	0	0	0	0	0	0	0	0	0	0	0	0	0	0	0	0	0	0	0	0	0	0	0	0	0	0
ENSG00000207056	0	0	0	0	0	0	0	0	0	0	0	0	0	0	0	0	0	0	0	0	0	0	0	0	0	0	0	0	0	0	0	0
ENSG00000237402	2	0	3	1	1	2	0	1	0	0	1	0	0	1	0	0	0	0	0	1	1	1	1	2	0	0	0	0	0	0	0	0
ENSG00000237728	0	0	2	1	0	1	0	0	0	0	0	0	0	0	0	0	0	0	0	0	0	0	0	1	0	0	0	0	0	0	0	1
ENSG00000225126	0	0	0	2	0	0	0	0	0	0	0	0	0	1	0	0	0	2	0	0	0	0	1	0	0	0	0	0	0	0	1	0
ENSG00000270171	0	0	1	0	0	0	0	0	0	0	0	0	2	2	1	1	0	0	0	0	0	0	0	0	0	0	0	0	0	0	0	0
ENSG00000270035	0	0	0	0	0	0	0	0	0	0	0	0	0	1	0	0	0	0	0	0	0	0	0	0	0	0	0	0	0	0	0	0
ENSG00000269978	0	0	0	0	0	0	0	0	0	0	0	0	0	2	2	0	0	0	0	0	0	0	0	0	0	0	0	0	0	0	0	0
ENSG00000049245	909	656	733	643	1182	1228	875	1288	325	261	1299	206	731	595	738	814	438	585	527	603	2673	1008	1773	1038	296	435	368	873	640	683	703	849
ENSG00000269925	40	47	44	38	20	23	17	19	18	9	73	4	13	15	9	19	15	6	12	40	35	24	20	33	13	23	25	46	5	12	18	11
ENSG00000049246	204	144	177	157	589	565	506	640	509	412	1885	302	59	54	45	87	79	80	80	157	2840	1100	2173	1156	523	761	488	1215	23	29	28	48
ENSG00000236266	8	1	8	2	0	4	8	11	11	29	86	7	3	1	0	1	1	2	1	4	8	4	2	2	14	20	19	53	0	2	0	0

ENSG00000049247	0	0	0	0	4	3	0	2	0	0	0	0	44	44	35	50	1	0	0	0	15	2	8	3	0	0	0	0	26	27	36	38
ENSG00000049249	0	0	0	0	28	64	25	75	2	2	6	3	862	1052	1134	1440	1	0	2	0	45	47	49	28	1	7	8	7	597	769	1098	1023
ENSG00000270330	0	0	0	0	0	0	0	0	0	0	0	0	0	0	0	0	0	0	0	0	2	1	0	0	0	0	0	0	0	0	0	1
ENSG00000116288	4543	3188	3451	3558	4727	4718	3426	5171	4468	3039	13382	2770	4232	3795	4571	5424	5582	7544	6047	5059	11524	4629	8082	4556	4019	5849	2609	8200	5415	4908	6610	6321
ENSG00000200344	3	3	4	9	4	6	3	10	2	6	15	0	3	5	3	1	1	0	1	5	10	6	4	6	2	12	1	13	0	3	4	6
ENSG00000116285	9	9	9	10	9	15	15	26	99	122	422	94	106	150	128	182	3	0	3	10	24	12	9	16	43	92	69	208	130	181	118	233
ENSG00000238290	0	0	0	0	1	3	4	2	0	0	2	0	0	0	0	0	0	0	0	0	5	0	4	0	0	0	1	0	0	0	0	0
ENSG00000232848	0	0	0	1	0	0	0	0	0	0	0	0	0	0	0	0	0	0	0	0	0	0	0	0	0	0	0	0	1	0	0	0
ENSG00000229305	0	0	0	2	0	0	0	0	0	0	0	0	0	0	0	0	0	0	0	0	0	0	0	0	0	0	0	0	0	0	0	0
ENSG00000227634	0	0	0	0	0	0	0	0	0	0	0	0	0	0	0	0	0	0	0	0	0	0	0	0	0	0	1	1	2	0	0	0
ENSG00000200975	0	0	0	0	0	0	0	0	0	0	0	0	0	0	0	0	0	0	0	0	0	0	0	0	0	0	0	0	0	0	0	0
ENSG00000233645	0	0	0	0	0	0	0	0	0	0	0	0	0	0	0	0	0	0	0	0	0	0	0	0	0	0	0	0	0	0	0	0
ENSG00000251977	0	0	0	0	0	0	0	0	0	0	0	0	0	0	0	0	0	0	0	0	0	0	0	0	0	0	0	0	0	0	0	0
ENSG00000162426	0	1	0	1	1	0	2	0	0	2	6	1	6	11	8	19	0	0	0	2	0	0	0	1	0	1	1	9	12	8	6	7
ENSG00000252254	0	0	0	0	0	0	0	0	1	0	0	0	0	0	0	0	0	0	0	0	0	0	0	0	0	0	0	0	0	0	0	0
ENSG00000142599	1279	1316	1510	1442	2111	2548	1773	2609	1898	1564	5988	996	2923	2663	2803	3315	264	380	329	969	3144	1518	1988	1294	1264	2289	1512	3654	1299	2148	1406	2310
ENSG00000232912	5	1	9	7	4	8	1	2	5	6	15	0	7	12	10	8	2	1	1	3	7	4	3	1	8	8	5	8	1	6	8	10
ENSG00000221083	0	0	3	0	2	0	1	2	2	1	7	3	1	3	0	1	0	0	0	1	1	3	0	1	0	0	0	1	0	0	2	1
ENSG00000270282	2	4	0	0	2	7	2	2	1	1	7	0	1	2	2	1	0	0	1	1	2	0	0	4	3	6	4	11	0	4	6	3
ENSG00000234619	7	0	8	9	4	8	6	4	4	5	29	5	3	2	2	2	0	1	2	5	10	6	9	7	12	22	3	21	0	6	3	6
ENSG00000224315	11	9	6	8	14	9	5	13	8	6	31	3	7	5	11	5	5	3	1	11	23	21	9	17	8	15	7	33	2	3	4	5
ENSG00000222511	1	2	5	3	4	6	3	5	2	4	11	2	2	3	2	2	1	1	2	8	6	3	5	6	2	1	4	15	0	2	2	1
ENSG00000226545	8	9	7	1	14	12	5	7	16	5	30	4	2	5	3	3	3	2	0	6	9	6	6	23	5	5	6	33	0	4	9	4
ENSG00000228423	14	10	15	4	7	16	6	18	4	6	32	4	5	8	7	8	1	4	3	14	23	18	18	15	6	6	9	22	2	0	2	5
ENSG00000229716	0	1	1	0	0	0	0	0	0	0	0	0	0	0	0	0	0	0	0	0	2	1	0	0	0	0	0	0	0	0	0	0
ENSG00000074800	80925	59484	65080	56959	72145	71675	53492	81003	77018	55016	111862	37540	82671	77267	75988	97043	19182	32523	27336	26643	72706	29106	57379	25361	36402	59209	16644	69383	50473	53841	53912	59173
ENSG00000274258	3	1	3	4	4	9	6	7	2	1	6	2	3	2	2	3	0	0	0	7	2	1	0	0	2	4	0	3	5	1	5	1
ENSG00000236269	53	36	40	40	82	64	50	92	67	47	120	16	67	60	49	60	12	2	7	30	70	60	39	48	32	39	26	131	13	56	23	54
ENSG00000230679	8	9	9	8	5	14	3	5	9	4	18	1	4	6	3	4	10	12	10	10	9	8	5	3	3	7	7	13	2	3	0	7
ENSG00000201725	0	0	2	3	0	0	0	2	0	2	0	0	3	1	0	0	1	0	1	2	1	0	0	1	0	0	4	0	1	0	0	0
ENSG00000238249	1	1	2	0	0	0	0	0	1	0	3	0	0	0	0	0	0	0	0	0	0	0	4	1	0	0	0	3	0	0	0	4
ENSG00000232208	0	0	0	0	0	0	0	0	4	0	3	0	0	0	1	0	0	0	0	0	0	0	0	0	0	0	0	1	0	0	0	0
ENSG00000232663	0	0	0	0	0	0	0	0	0	0	0	0	0	0	0	0	0	0	0	0	0	0	0	0	0	0	0	0	0	0	0	0
ENSG00000131686	0	0	0	0	0	0	0	0	300	259	612	152	3	1	3	2	0	0	0	3	0	0	0	0	62	93	139	287	0	1	0	0
ENSG00000265141	0	0	0	0	0	0	0	0	1	0	2	0	0	0	0	0	0	0	0	0	0	0	0	0	0	1	0	0	0	0	0	0
ENSG00000197241	0	0	0	2	0	0	1	1	18	7	22	10	0	0	1	1	0	0	0	0	1	0	0	0	5	4	4	7	0	0	0	0
ENSG00000142583	0	0	0	0	2	1	1	6	0	2	3	0	20	18	12	24	0	0	0	0	2	1	2	3	0	0	1	1	5	6	2	9
ENSG00000252404	0	0	0	0	0	0	0	0	0	1	1	0	0	0	0	0	0	0	0	0	0	0	0	0	0	0	0	0	0	0	0	0
ENSG00000180758	73	146	90	139	119	143	66	132	81	80	150	65	191	238	191	359	13	70	22	113	115	59	81	61	21	59	41	94	128	175	125	190
ENSG00000228526	0	1	0	0	0	0	0	0	212	169	7139	650	16	20	5	35	0	0	0	0	0	0	0	1	79	127	1520	2231	2	4	0	8
ENSG00000234546	0	0	0	0	0	0	0	0	12	16	406	53	0	1	1	3	0	0	0	0	0	2	0	0	7	7	89	173	0	0	1	0
ENSG00000281937	0	0	0	0	0	0	0	0	0	0	27	2	0	1	0	0	0	0	0	0	0	0	0	0	0	1	4	13	0	0	0	0
ENSG00000049239	2152	1894	2110	1729	1710	2026	1031	2056	1156	1019	2951	616	1758	1720	1484	2113	478	558	517	1238	1752	758	922	666	426	855	632	1797	535	912	445	900
ENSG00000171621	272	226	316	254	180	190	161	189	4	4	4	5	164	156	165	223	70	123	160	192	228	114	257	84	2	10	1	37	135	227	176	231
ENSG00000235263	0	0	0	0	0	0	0	0	0	0	0	0	0	0	0	0	0	0	0	0	0	0	0	0	0	0	1	0	0	0	0	0
ENSG00000171612	669	445	566	489	590	592	468	574	387	312	1741	261	506	455	595	517	451	808	708	821	1490	518	1046	488	482	700	387	1151	615	731	694	743
ENSG00000231181	12	10	17	8	13	11	7	19	17	15	114	14	21	17	19	27	5	6	3	20	30	20	14	14	6	35	8	46	7	29	10	33
ENSG00000188807	913	833	954	796	1026	1055	893	1203	809	722	2943	607	705	636	874	848	257	395	422	882	1983	883	1495	804	796	1396	505	2350	630	777	653	885
ENSG00000171608	1988	1517	1836	1583	1007	1142	739	1141	7981	6874	36510	5934	3696	3243	3515	3995	345	506	513	926	1458	652	1072	600	6492	11246	7895	21668	2113	2511	2135	2899
ENSG00000179840	5	6	9	10	4	4	2	2	76	65	310	31	32	28	20	34	2	0	5	10	4	10	11	3	73	104	82	333	10	33	24	42
ENSG00000233268	1	6	6	3	1	1	0	2	12	17	81	8	6	3	10	6	0	1	0	0	1	1	0	2	13	33	18	88	0	4	3	5
ENSG00000231789	79	63	66	52	2	2	2	7	33	26	184	14	9	12	23	8	55	96	67	81	5	1	4	6	32	52	42	129	4	9	14	22
ENSG00000171603	4286	3961	4017	3837	3845	4037	2795	4163	4054	3515	16552	2971	2048	2173	2006	2703	718	1139	873	2007	4996	1997	3400	1740	2938	5093	3554	9465	1133	1775	1120	1899
ENSG00000271264	4	1	1	3	1	0	4	2	2	1	6	2	0	1	3	3	0	0	1	1	0	1	3	1	4	7	4	5	0	3	1	7
ENSG00000280113	0	0	1	0	0	0	0	0	1	0	3	0	0	0	0	0	0	0	0	1	1	0	0	0	0	0	3	4	0	1	0	1
ENSG00000178585	933	674	811	744	989	988	611	977	1444	1201	10552	1559	294	274	256	360	243	487	433	587	1325	574	905	440	1375	1998	1727	6028	179	227	214	266
ENSG00000223989	0	0	0	0	0	0	0	0	0	0	0	0	0	0	0	0	0	0	0	0	0	0	0	0	0	0	0	0	0	0	0	0
ENSG00000162441	1264	735	1082	826	872	989	742	1036	691	498	2619	400	442	436	501	539	1540	1663	1658	1459	1994	835	1529	966	646	856	479	1579	502	533	542	585
ENSG00000228150	19	4	11	6	14	15	7	18	12	16	45	16	25	15	27	21	12	3	9	7	20	15	21	9	12	13	11	17	22	21	36	38
ENSG00000173614	330	232	297	207	188	212	104	176	311	230	1276	202	267	221	262	297	203	266	239	207	220	77	195	62	231	391	312	786	178	210	218	180
ENSG00000202415	6	4	3	2	1	1	3	1	6	3	35	0	8	4	2	9	0	3	0	2	7	1	1	10	8	5	5	13	3	0	0	3
ENSG00000283611	4	2	2	2	4	2	0	3	7	5	31	1	8	3	2	0	3	0	1	3	7	1	3	5	5	5	9	25	0	8	1	5
ENSG00000265521	0	1	0	0	0	2	0	0	0	1	9	0	1	0	2	1	0	0	2	0	0	0	0	0	2	3	0	4	0	2	0	1
ENSG00000241326	0	0	0	0	0	0	0	0	0	0	0	0	0	0	0	0	0	0	0	0	0	0	0	0	0	0	0	0	0	0	0	0
ENSG00000162444	0	0	0	0	0	0	0	0	0	0	4	0	0	0	1	0	1	0	6	1	1	0	0	0	0	0	0	0	0	1	1	0
ENSG00000130939	1761	1328	1365	1255	1456	1475	1081	1601	1539	1326	6034	976	1519	1294	1224	1619	361	570	490	977	2283	1023	1522	832	1225	2095	1356	3811	958	1331	859	1410
ENSG00000224340	3	1	2	2	2	3	3	2	2	0	5	1	1	0	1	2	1	0	0	1	2	4	1	3	1	4	1	12	0	3	4	4
ENSG00000233623	3	4	4	7	6	7	3	6	10	1	24	1	5	6	7	4	2	1	2	4	5	3	5	4	3	8	9	31	0	11	3	11
ENSG00000201746	2	0	0	1	4	4	3	1	5	2	14	1	1	3	1	1	1	0	0	0	3	1	1	1	3	1	3	5	0	1	1	3
ENSG00000054523	1833	1548	1743	1626	2311	2594	1782	2618	1376	1183	6670	944	2237	2256	2148	2851	391	680	544	1192	3053	1334	2072	1090	1297	1972	1412	4043	1265	2130	1502	2362
ENSG00000264881	2	0	0	0	0	1	1	0	0	0	1	0	0	0	0	0	0	0	0	0	1	0	0	2	1	0	1	0	0	0	0	0
ENSG00000199562	2	2	4	0	3	2	2	2	2	0	7	0	4	0	2	0	0	0	0	4	1	1	2	1	3	2	0	4	0	2	1	5
ENSG00000264501	0	0	0	0	0	0	0	0	0	0	0	0	0	0	0	0	0	0	0	0	0	0	0	0	0	0	0	0	0	0	0	0
ENSG00000142657	7867	5215	5532	5124	6457	6399	4803	6741	2333	1614	6965	1378	3724	3252	3465	4351	3344	4895	4048	4298	11789	4523	9222	3842	1946	3291	1413	4635	2753	3000	2929	3231
ENSG00000271989	7	7	13	9	1	0	3	4	1	1	7	7	3	0	3	1	2	3	4	9	3	4	3	5	0	4	0	8	0	5	1	1
ENSG00000251503	35	28	39	42	35	30	31	48	22	14	70	20	25	32	29	38	41	41	44	60	79	27	57	32	28	35	16	43	24	41	35	38
ENSG00000175279	363	249	345	272	164	176	123	206	182	137	420	80	177	190	176	226	363	463	471	442	408	195	303	190	170	218	84	343	196	217	261	237
ENSG00000241563	20	27	41	40	12	17	19	23	14	8	37	6	12	9	12	9	8	7	12	28	27	16	16	10	9	7	8	36	14	13	7	20
ENSG00000160049	3063	2070	2579	2167	2069	2320	1620	2337	2080	1544	6672	1356	4550	3833	4621	4867	1415	1897	1869	1658	2937	1100	2679	767	1386	2494	1383	3969	3607	3692	3671	3232
ENSG00000203469	0	0	0	0	0	0	0	0	0	0	0	0	0	0	0	0	0	0	0	0	0	0	0	0	0	0	0	0	0	0	0	0
ENSG00000142655	586	410	532	395	842	812	539	798	490	385	1590	323	539	545	532	628	186	238	283	340	1166	512	901	435	377	680	317	1017	385	456	446	542
ENSG00000243267	1	0	0	0	6	4	5	4	3	0	8	0	4	0	3	0	0	0	0	4	7	2	3	1	1	1	0	4	1	1	1	3
ENSG00000130940	311	357	370	403	478	550	354	531	29	45	261	32	200	139	157	176	98	129	117	241	687	331	438	247	44	77	51	183	85	112	80	125
ENSG00000272078	38	34	30	40	38	61	48	62	5	8	38	10	3	10	11	19	11	10	6	32	74	53	42	52	10	12	5	28	8	11	14	18
ENSG00000225158	0	0	0	0	0	0	0	0	0	0	2	0	0	0	0	0	0	0	0	0	0	0	0	0	0	0	2	1	0	0	0	0
ENSG00000175262	2	1	2	3	4	2	1	0	0	0	0	0	25	27	16	25	2	0	2	1	2	0	0	2	0	0	1	0	9	16	9	13
ENSG00000215785	0	0	2	0	0	0	0	0	0	0	4	0	0	0	0	0	0	0	0	0	0	0	0	1	0	2	0	0	0	0	0	0
ENSG00000120948	5724	3855	4945	4059	5211	5507	4357	5694	4176	3259	13905	2359	4645	4079	4832	5511	3007	4432	3812	4432	10252	4149	7500	3945	4009	6024	3000	9016	4191	5110	4615	5817
ENSG00000277726	1	0	0	0	3	0	1	6	0	0	11	0	0	0	0	0	0	0	0	0	2	5	3	2	0	0	2	3	0	0	0	0
ENSG00000009724	59	50	65	59	139	162	124	158	90	81	531	73	94	89	65	105	10	13	16	71	248	147	132	110	53	89	162	229	39	69	34	82
ENSG00000271895	49	38	56	34	117	112	72	114	83	73	402	48	46	51	53	82	15	8	17	38	163	74	117	84	60	65	128	194	24	68	18	46
ENSG00000116649	2012	1740	1929	1739	1883	1841	1405	1852	1529	1130	5579	1018	2330	1887	2410	2573	736	1517	1529	1957	2990	1210	2154	1009	1505	2461	1236	3286	2133	2251	2212	2374
ENSG00000171824	2723	1928	2319	1897	2868	2780	2252	3103	1734	1315	5606	1104	1959	1708	2042	2315	2409	2509	2458	2429	6622	2545	4874	2394	1428	2349	1346	3833	1734	2100	2084	2312
ENSG00000226849	27	30	27	22	29	29	21	34	27	13	74	8	14	14	15	12	6	7	13	26	42	25	30	20	15	20	18	54	4	15	3	10
ENSG00000230337	10	1	8	6	10	14	13	14	12	4	29	5	13	10	16	22	5	7	24	8	37	9	22	10	6	4	6	10	23	15	28	18
ENSG00000198793	2879	2104	2649	2202	3453	3652	2531	3679	2057	1596	8652	1441	2584	2212	2756	3002	752	920	878	1808	5393	2528	3744	2153	2082	3799	1861	6553	1671	2430	1841	2560
ENSG00000225602	11	7	6	10	3	17	8	7	6	4	20	0	1	4	4	0	0	3	8	10	20	3	9	16	4	12	2	34	1	5	2	8
ENSG00000253086	0	0	0	0	1	2	0	0	0	0	1	0	0	0	1	0	1	0	0	0	3	1	0	0	4	0	2	4	0	0	4	3
ENSG00000171819	17	12	9	6	6	17	20	12	10	5	21	5	5	4	9	12	5	3	4	20	25	20	19	28	17	25	6	43	4	7	22	13
ENSG00000207451	3	3	0	2	0	1	1	1	0	3	7	0	0	3	0	1	0	0	0	3	5	0	3	4	0	1	0	1	0	3	3	4
ENSG00000238173	0	2	2	0	1	0	0	0	0	3	3	0	0	0	0	1	3	0	0	1	3	4	1	0	1	1	0	10	0	0	0	1
ENSG00000120942	704	462	680	604	793	789	561	815	644	521	2420	483	761	672	930	898	309	543	547	666	1620	647	1262	514	627	1029	583	1805	706	893	808	917
ENSG00000238199	7	6	14	7	7	6	9	2	4	3	20	2	9	11	7	11	0	0	2	7	30	7	15	8	7	4	3	14	6	13	6	9
ENSG00000224338	0	0	0	0	0	0	0	0	0	0	0	0	0	0	0	0	0	0	0	0	0	0	0	0	0	0	0	0	0	0	0	0
ENSG00000204624	67	77	68	78	57	44	26	50	14	19	39	19	7	17	8	16	15	32	20	60	67	42	40	38	5	16	5	40	6	21	5	16
ENSG00000235643	0	0	1	0	0	0	0	0	0	1	0	0	0	0	0	0	0	1	1	0	0	1	0	0	0	0	0	2	0	0	0	0
ENSG00000116661	2	7	5	11	1	1	0	0	6	16	59	9	12	5	7	14	4	3	2	4	1	0	1	0	4	16	13	39	4	8	9	8
ENSG00000132879	438	387	428	422	257	270	153	280	1067	866	5737	868	287	287	237	360	124	211	172	332	191	110	160	112	740	1201	1536	2982	173	253	156	319
ENSG00000116663	87	96	84	91	147	216	115	202	416	335	1981	371	174	183	151	188	42	90	68	87	302	125	223	133	448	685	433	1352	155	253	194	275
ENSG00000116670	2134	1693	1801	1774	1894	1906	1372	1965	2696	2098	8550	1792	1256	1143	1232	1525	998	1589	1469	1496	3151	1252	2522	1137	2829	4336	1496	6663	1251	1260	1315	1558
ENSG00000162490	99	153	91	148	1136	1246	830	1208	4882	4083	22245	3876	153	181	180	287	14	54	29	71	1556	559	927	491	4674	7596	4286	15618	108	199	113	191
ENSG00000177674	402	338	346	339	236	255	154	308	483	399	1881	407	606	651	627	779	156	268	247	307	320	127	222	139	407	647	366	1276	457	571	528	614
ENSG00000215910	2	2	0	0	0	4	2	2	3	0	6	0	3	1	0	5	0	0	0	0	0	0	0	1	0	0	0	2	0	2	0	0
ENSG00000177553	0	0	1	0	0	0	0	0	0	0	0	0	0	0	1	0	0	0	0	0	0	0	0	0	0	0	0	2	0	0	0	0
ENSG00000177000	1535	989	1205	1073	1060	1048	734	1030	557	563	3510	510	575	634	442	829	237	286	258	596	1243	558	808	474	581	912	967	2200	283	413	257	439
ENSG00000011021	2179	1481	1439	1366	1462	1534	1181	1566	453	425	1919	277	815	872	601	971	524	380	296	833	1794	835	859	727	280	547	514	1197	230	479	274	530
ENSG00000242349	84	46	55	50	40	56	49	68	13	15	40	8	40	32	27	47	29	24	10	65	66	41	31	47	12	12	10	31	4	24	11	28
ENSG00000276470	0	0	0	0	0	0	0	0	0	0	0	0	0	0	0	0	0	0	0	0	0	0	0	0	0	0	0	0	0	0	0	0
ENSG00000278852	0	0	0	0	0	0	0	0	0	0	0	0	0	0	0	0	0	0	0	0	0	0	0	0	0	0	0	0	0	0	0	0
ENSG00000175206	33	16	22	12	28	25	21	31	16	5	18	2	11	20	7	14	4	3	7	9	28	10	10	7	6	2	7	8	1	6	8	15
ENSG00000275915	0	0	0	0	0	0	0	0	0	0	0	0	0	0	0	0	0	0	0	0	0	0	0	0	0	0	0	0	0	0	0	0
ENSG00000120937	0	0	0	0	1	0	0	0	0	0	0	0	0	0	0	0	0	0	0	0	0	0	0	0	0	0	0	0	0	0	0	0
ENSG00000224904	2	0	0	0	0	0	0	0	0	0	0	1	0	0	0	0	0	0	0	1	0	0	0	0	0	0	0	0	0	0	0	0
ENSG00000199347	6	1	7	1	4	3	5	1	5	1	1	0	0	1	1	0	14	0	7	13	8	1	5	8	3	0	0	5	2	3	4	1
ENSG00000201801	2	2	6	0	6	4	2	5	1	2	6	0	1	0	1	0	1	0	4	10	6	4	7	1	1	3	9	11	0	3	1	0
ENSG00000116685	1414	1122	1358	1071	1993	1885	1353	1993	858	704	3371	581	1603	1393	1709	1761	593	840	882	992	2945	1071	2036	896	803	1267	686	2015	1616	1504	1808	1684
ENSG00000083444	866	732	970	733	2738	3017	1919	3079	866	658	2727	544	2104	2046	1979	2514	145	203	224	393	1810	840	1253	715	463	849	572	1600	981	1041	947	1228
ENSG00000116688	2039	1400	1715	1444	2855	2930	2178	2960	1976	1581	7815	1413	2091	1721	2281	2427	662	778	924	1447	4450	1960	3258	1711	2041	3434	1668	5452	1914	2259	2040	2507
ENSG00000270914	1	2	0	0	0	0	2	1	0	0	1	0	2	0	0	0	0	0	0	1	2	0	0	0	0	0	2	0	0	1	1	1
ENSG00000116691	1070	752	879	743	761	701	429	724	363	355	1491	322	664	665	858	814	580	641	517	803	1159	474	796	422	359	655	293	933	654	703	765	792
ENSG00000202461	17	13	8	13	10	10	15	13	7	2	50	3	2	5	6	6	2	1	2	3	14	5	9	6	7	5	7	22	1	9	11	7
ENSG00000276869	0	0	0	0	0	0	0	0	0	0	1	0	0	0	0	1	1	0	0	0	0	0	0	0	0	0	0	0	0	0	0	0
ENSG00000243539	0	0	0	0	0	0	0	0	0	0	1	0	0	0	1	0	0	0	0	0	0	0	0	0	1	0	0	0	0	1	0	0
ENSG00000120949	592	572	643	587	167	204	148	231	6	3	3	3	2804	2182	3090	2897	199	456	413	876	337	167	209	134	3	14	0	9	1982	2208	2096	2504
ENSG00000201135	2	0	0	0	0	0	0	0	0	0	2	0	12	9	5	10	0	0	1	4	0	2	0	0	0	0	2	0	4	4	8	6
ENSG00000227875	0	0	4	4	0	0	1	0	0	0	0	0	15	7	8	3	0	5	0	5	0	1	1	1	0	0	0	0	2	8	5	8
ENSG00000028137	13	6	9	2	606	768	476	870	1279	923	3419	816	2119	1829	1820	2508	2	1	6	1	405	238	323	195	716	1295	430	2281	1282	1460	1039	1463
ENSG00000263676	0	0	0	0	0	1	0	0	0	2	1	0	1	0	0	0	0	0	0	0	0	0	0	0	0	0	0	0	0	2	0	0
ENSG00000225196	0	0	0	0	0	0	0	0	0	0	0	0	0	0	0	0	0	0	0	0	2	0	0	0	0	0	0	1	0	0	0	0
ENSG00000252969	0	0	0	0	0	0	0	0	2	0	0	0	0	0	0	0	0	0	0	0	0	0	0	0	0	0	0	0	0	0	0	0
ENSG00000048707	2680	2268	2515	2286	1707	1920	1324	1978	1238	1114	6704	986	1076	1090	1059	1337	449	622	542	1366	2458	1257	1656	1094	1470	2336	1854	5257	653	1154	810	1264
ENSG00000239149	12	19	7	17	1	3	3	11	1	1	6	0	6	3	6	2	1	1	1	14	10	3	10	2	11	3	3	7	0	3	2	6
ENSG00000229484	0	0	1	0	0	1	0	2	0	0	1	2	0	0	0	0	0	0	0	0	0	0	0	0	0	0	2	2	0	0	0	0
ENSG00000162496	181	214	175	197	251	287	195	311	497	397	1782	338	308	371	280	438	23	27	47	65	309	136	290	152	215	354	385	1007	113	191	170	214
ENSG00000221340	1	0	2	2	2	0	1	3	8	1	27	4	0	1	1	1	0	0	0	2	1	1	0	3	3	4	1	9	1	2	3	0
ENSG00000276830	0	1	0	2	0	0	0	0	1	4	8	2	0	0	3	1	0	0	0	4	2	1	0	2	1	1	2	0	0	0	0	0
ENSG00000272482	6	6	13	6	4	8	5	7	11	2	38	5	4	2	3	3	2	0	1	4	7	5	3	6	0	2	5	6	2	1	0	2
ENSG00000204518	0	0	0	0	0	0	0	0	0	0	0	0	0	0	0	0	0	0	0	0	0	0	0	0	2	0	0	0	0	0	0	0
ENSG00000226166	0	0	0	0	0	0	0	0	0	0	0	0	0	0	0	0	0	0	0	0	0	0	0	0	0	0	0	2	0	0	0	0
ENSG00000188984	0	0	0	0	0	0	0	0	0	0	0	0	0	0	0	0	0	0	0	0	0	0	0	0	0	0	0	0	0	0	0	0
ENSG00000157330	0	0	0	0	0	0	0	0	0	0	0	0	0	0	1	0	0	0	0	0	0	0	0	0	0	0	0	0	0	0	0	0
ENSG00000116726	0	0	0	0	0	0	0	0	0	0	0	0	0	0	0	0	0	0	0	0	0	0	0	0	0	0	0	0	0	0	0	0
ENSG00000116721	0	0	0	0	0	0	0	0	0	0	0	0	0	0	0	0	0	0	0	0	0	0	0	0	0	0	0	0	0	0	0	0
ENSG00000228338	0	0	0	0	0	0	0	0	0	0	0	0	0	0	0	0	0	0	0	0	0	0	0	0	0	0	0	0	0	0	0	0
ENSG00000239810	0	0	0	0	0	0	0	0	0	0	0	0	0	0	0	0	0	0	0	0	0	0	0	0	0	0	0	0	0	0	0	0
ENSG00000231103	0	0	0	0	0	0	0	0	0	0	0	0	0	0	0	0	0	0	0	0	0	0	0	0	0	0	0	3	0	0	0	0
ENSG00000179172	0	0	0	0	1	1	0	1	1	0	1	0	1	0	1	2	0	1	0	2	1	1	3	0	0	1	0	0	2	0	1	0
ENSG00000120952	0	0	0	0	0	0	0	0	0	0	0	0	0	0	0	0	0	0	0	0	0	0	0	0	0	0	0	0	0	0	0	0
ENSG00000243073	0	0	0	0	0	0	0	0	0	0	3	0	0	0	0	0	0	0	0	0	0	0	0	0	0	0	0	0	0	0	0	0
ENSG00000187545	0	0	0	0	0	0	0	0	0	0	0	0	0	0	0	0	0	0	0	0	0	0	0	0	0	0	0	0	0	0	0	0
ENSG00000204510	0	0	0	0	0	0	0	0	0	0	0	0	0	0	0	0	0	0	0	0	0	0	0	0	0	0	0	0	0	0	0	0
ENSG00000207434	0	0	0	0	0	0	0	0	0	0	0	0	0	0	0	0	0	0	0	0	0	0	0	0	0	0	0	0	0	0	0	0
ENSG00000234064	0	0	0	0	0	0	0	0	0	0	0	0	0	0	0	0	0	0	0	0	0	0	0	0	0	0	0	0	0	0	0	0
ENSG00000232423	0	0	0	0	0	0	0	0	0	0	0	0	0	0	0	0	0	0	0	0	0	0	0	0	0	0	0	0	0	0	0	0
ENSG00000274178	0	0	0	0	0	0	0	0	0	0	0	0	0	0	0	0	0	0	0	0	0	0	0	0	0	0	0	0	0	0	0	0
ENSG00000274764	0	0	0	0	0	0	0	0	0	0	0	0	0	0	0	0	0	0	0	0	0	0	0	0	0	0	0	0	0	0	0	0
ENSG00000229571	0	0	0	0	0	0	0	0	0	0	0	0	0	0	0	0	0	0	0	0	0	0	0	0	0	0	0	0	0	0	0	0
ENSG00000277058	0	0	0	0	0	0	0	0	0	0	0	0	0	0	0	0	0	0	0	0	0	0	0	0	0	1	0	1	0	0	0	0
ENSG00000277862	0	0	0	0	0	0	0	0	0	0	0	0	0	0	0	0	0	0	0	0	0	0	0	0	0	0	0	0	0	0	0	0
ENSG00000236179	0	0	0	0	0	0	0	0	0	0	0	0	0	0	0	0	0	0	0	0	0	0	0	0	0	0	0	0	0	0	0	0
ENSG00000275774	0	0	0	0	1	0	0	0	0	0	1	0	0	0	0	0	0	0	0	0	1	0	0	0	0	3	1	0	0	0	1	0
ENSG00000229978	0	0	0	0	0	0	0	0	0	0	0	0	0	0	0	0	0	0	0	0	0	0	0	0	0	0	0	0	0	0	0	0
ENSG00000233336	0	0	0	0	0	0	0	0	0	0	0	0	0	0	0	0	0	0	0	0	0	0	0	0	0	0	0	0	0	0	0	0
ENSG00000280267	0	0	0	0	0	0	0	0	0	0	0	0	0	0	0	0	0	0	0	0	0	0	0	0	0	0	0	0	0	0	0	0
ENSG00000179412	0	1	0	0	0	0	1	0	0	0	0	0	0	0	0	0	0	0	0	0	0	0	0	0	0	0	0	0	0	0	0	0
ENSG00000204505	0	0	0	0	0	0	0	0	0	0	0	0	0	0	0	0	0	0	0	0	0	0	0	0	0	0	0	0	0	0	0	0
ENSG00000279169	0	0	0	0	0	0	0	0	0	0	0	0	0	0	0	0	0	0	0	0	0	0	0	0	0	0	0	0	0	0	0	0
ENSG00000279804	0	0	2	0	0	0	0	0	0	0	0	0	0	0	0	0	0	0	0	0	0	0	0	0	0	0	0	0	0	0	0	0
ENSG00000250894	0	0	0	0	0	0	0	0	0	0	0	0	0	0	0	0	0	0	0	0	0	0	0	0	0	0	0	0	0	0	0	0
ENSG00000270601	0	0	0	0	0	0	0	0	0	0	0	0	0	0	0	0	0	0	0	0	0	0	0	0	0	0	0	0	0	0	0	0
ENSG00000231219	0	0	0	0	0	0	0	0	0	0	0	0	0	0	0	0	0	0	0	0	0	0	0	0	0	0	0	0	0	0	0	0
ENSG00000207511	0	0	0	0	0	0	0	0	0	0	0	0	0	0	0	0	0	0	0	0	0	0	0	0	0	0	0	0	0	0	0	0
ENSG00000182330	0	0	0	0	0	0	0	0	0	0	0	0	0	0	0	0	0	0	0	0	0	0	0	0	0	0	0	0	0	0	0	0
ENSG00000204501	0	0	0	0	0	0	0	0	0	0	0	0	0	0	0	0	0	0	0	0	0	0	0	0	0	0	0	0	0	0	0	1
ENSG00000237700	0	0	0	0	0	0	0	0	0	0	0	0	0	0	0	0	0	0	0	0	0	0	0	0	0	0	0	0	0	0	0	0
ENSG00000228229	0	0	0	0	0	0	0	0	0	0	0	0	0	0	0	0	0	0	0	0	0	0	0	0	0	0	0	0	0	0	0	0
ENSG00000204481	0	0	0	0	0	0	0	0	0	0	0	0	0	0	0	0	0	0	0	0	0	0	0	0	0	0	0	0	0	0	0	0
ENSG00000204480	0	0	0	0	0	0	0	0	0	0	0	0	0	0	0	0	0	0	0	0	0	0	0	0	0	0	0	0	0	0	0	0
ENSG00000204479	0	0	0	0	0	0	0	0	0	0	0	0	0	0	0	0	0	0	0	0	0	0	0	0	0	0	0	0	0	0	1	0
ENSG00000204478	0	0	0	0	0	0	0	0	0	0	0	0	0	0	0	0	0	0	0	0	0	0	0	0	0	0	1	0	0	0	0	1
ENSG00000162494	31	42	59	70	0	0	0	0	0	0	0	0	0	0	0	0	4	11	15	35	0	0	0	0	0	0	2	0	0	0	0	0
ENSG00000259961	0	0	0	0	0	0	0	0	0	0	0	0	0	0	0	0	0	0	0	0	0	0	0	0	0	0	0	0	0	0	0	0
ENSG00000215909	0	0	0	0	0	0	0	0	0	0	0	0	0	0	0	0	0	0	0	0	0	0	0	1	0	0	1	0	0	0	0	0
ENSG00000162493	2	0	0	0	0	0	0	0	0	0	1	0	0	0	1	0	0	0	0	0	0	0	0	0	0	0	0	1	0	0	0	0
ENSG00000237445	0	0	0	0	0	0	0	0	0	0	0	0	0	0	0	0	0	0	0	0	0	0	0	0	0	0	0	0	0	0	0	0
ENSG00000253085	0	0	0	0	0	0	1	0	0	0	2	0	0	0	0	3	0	0	0	0	0	0	1	0	1	0	0	0	0	0	0	0
ENSG00000116731	2127	1706	1851	1673	2039	2068	1530	2176	1269	1122	5677	785	3035	2712	3244	3288	598	724	713	1448	3400	1453	2167	1251	1208	1888	1422	3851	1757	2576	2275	2771
ENSG00000189337	535	564	541	593	150	161	100	176	180	152	226	91	738	674	731	942	175	317	204	564	212	82	115	72	62	138	30	190	450	665	477	677
ENSG00000252151	0	0	0	0	0	0	0	0	0	0	0	0	0	0	0	0	0	0	0	0	0	0	0	0	0	0	0	0	0	0	0	0
ENSG00000231606	0	0	0	0	0	0	0	0	0	0	0	0	1	1	1	0	0	0	0	0	0	0	1	0	0	0	0	0	0	0	0	0
ENSG00000234593	0	2	4	1	0	0	0	0	0	0	0	0	0	0	0	0	1	3	0	0	0	0	0	0	0	0	0	0	0	1	1	3
ENSG00000235829	0	0	0	0	0	0	0	0	0	0	0	0	7	9	14	10	0	0	0	0	0	0	0	0	0	0	0	0	1	11	11	23
ENSG00000175147	0	1	0	0	1	0	0	0	0	0	0	0	1	0	0	2	0	0	0	0	0	0	0	0	0	0	0	0	0	2	0	1
ENSG00000171729	0	0	0	1	0	0	0	0	2	1	3	7	9	17	20	11	0	0	0	0	0	0	0	0	1	2	1	1	6	10	12	23
ENSG00000204464	0	0	0	0	0	0	0	0	0	0	0	0	4	0	0	0	0	0	0	0	0	0	0	0	0	0	0	0	3	0	0	0
ENSG00000215720	0	0	0	0	0	0	0	0	0	1	0	0	0	0	0	0	0	0	0	0	0	0	0	0	0	0	0	0	0	0	1	0
ENSG00000278480	0	0	0	0	0	0	0	0	0	0	0	0	0	0	0	0	0	0	0	0	0	0	0	0	0	0	0	0	0	0	0	0
ENSG00000142621	6	6	9	6	2	0	1	7	5	5	8	0	78	91	76	104	4	3	3	8	2	1	2	2	0	0	1	0	13	40	9	34
ENSG00000233485	0	0	0	0	0	0	0	0	0	0	2	0	0	1	0	0	0	0	0	0	0	0	0	0	0	0	3	1	0	0	1	2
ENSG00000236045	0	0	0	0	0	0	0	0	0	0	0	0	0	0	0	0	0	0	0	0	0	0	0	0	0	0	1	0	0	0	1	1
ENSG00000228140	3	0	3	3	3	4	5	0	2	5	13	1	0	7	2	3	0	0	1	4	5	0	4	2	5	3	1	7	0	0	1	2
ENSG00000142634	1898	1458	1862	1676	1826	1974	1374	1800	2804	2257	7292	1750	1869	1803	2349	2477	750	1354	1235	1812	3239	1342	2515	1044	2408	4177	1449	5172	1868	2121	2058	2248
ENSG00000162438	7	4	3	4	0	0	0	1	1	3	10	1	0	2	0	0	0	2	0	2	4	1	0	0	3	1	10	9	0	2	1	1
ENSG00000142615	1	4	2	0	2	0	0	1	0	1	4	0	1	1	1	0	0	0	0	0	0	1	0	2	0	0	2	1	0	0	0	4
ENSG00000215704	3	6	4	6	12	12	16	16	2	4	26	0	3	2	5	0	0	1	2	4	16	3	3	8	3	0	6	6	3	4	2	4
ENSG00000132906	237	201	226	202	320	330	203	328	203	176	828	157	127	137	159	169	91	123	111	136	510	216	397	187	144	230	213	449	114	145	172	179
ENSG00000116138	648	460	556	447	436	438	362	491	419	358	2085	269	451	385	479	506	259	266	258	433	778	321	655	323	390	561	546	1189	296	475	390	509
ENSG00000251866	1	0	0	2	0	0	0	0	1	0	2	0	0	1	0	0	0	0	0	1	0	0	1	1	0	0	0	2	0	0	2	0
ENSG00000272510	0	0	0	0	2	1	0	4	1	3	4	0	7	4	2	4	0	0	0	1	2	1	2	2	0	3	1	3	2	3	2	4
ENSG00000116771	9	3	8	6	85	77	62	74	112	90	369	69	286	264	269	278	1	3	4	8	164	61	120	47	138	184	79	243	270	238	276	242
ENSG00000237301	0	0	3	1	22	21	13	19	9	5	32	0	29	17	12	21	1	4	0	1	33	10	19	11	2	3	16	1	12	16	13	8
ENSG00000235084	0	0	0	0	0	1	1	1	5	2	8	0	1	4	3	4	3	0	1	1	3	1	1	0	0	3	2	4	2	0	3	7
ENSG00000252417	0	0	0	0	0	0	0	0	0	0	0	0	0	0	0	0	0	0	0	0	0	0	0	0	0	0	2	0	0	1	0	0
ENSG00000236500	0	0	0	0	0	0	0	1	1	0	0	0	0	0	0	0	0	0	0	0	0	0	0	0	0	0	0	0	0	0	0	0
ENSG00000197312	2095	1577	2065	1664	1471	1497	1209	1634	1747	1403	7226	1143	1692	1530	2077	2089	648	1050	995	1907	2659	1143	1928	1047	1842	2981	1613	4529	1597	2063	1656	2126
ENSG00000215695	0	0	0	0	0	0	0	0	0	0	0	0	0	0	0	0	0	0	0	0	0	0	0	0	0	0	0	0	0	0	0	0
ENSG00000271742	1	0	0	0	3	1	1	0	0	0	1	0	0	0	0	1	0	0	0	1	1	2	1	3	0	0	0	0	0	1	2	0
ENSG00000116786	1930	1515	1655	1465	1947	1842	1323	2016	1101	966	4557	808	1166	1036	1133	1318	612	837	798	1398	2798	1296	2167	1169	814	1535	1146	2918	850	1103	982	1185
ENSG00000237938	45	42	62	38	30	34	21	37	37	20	148	14	34	28	16	27	16	17	8	39	41	27	37	51	25	34	39	114	13	22	9	38
ENSG00000162461	40	44	29	31	23	21	34	39	25	23	136	13	9	5	4	15	6	10	19	30	27	27	18	23	10	23	25	61	6	7	7	5
ENSG00000224459	0	0	0	0	0	0	0	0	0	0	0	0	0	0	0	0	0	0	0	0	0	0	0	0	0	0	0	0	0	0	0	0
ENSG00000162460	1	0	0	0	0	0	0	0	0	2	0	0	0	0	0	0	0	0	0	0	0	0	0	0	0	0	0	0	0	0	0	0
ENSG00000162458	22	21	26	23	0	0	0	0	0	2	2	2	3	1	5	3	3	6	10	13	0	0	0	0	0	0	0	0	0	0	1	1
ENSG00000224321	0	0	0	0	0	0	0	0	1	0	1	0	0	0	0	0	0	0	0	1	0	0	0	0	0	0	2	2	1	0	0	1
ENSG00000233954	296	199	245	191	236	250	180	273	235	142	598	120	210	174	223	267	347	352	421	371	690	221	466	344	198	258	145	409	257	251	305	289
ENSG00000178715	4	7	6	2	4	4	0	1	8	3	31	3	6	8	5	8	2	2	2	5	4	2	2	1	3	5	0	11	2	1	1	6
ENSG00000179743	186	152	149	164	130	151	97	136	154	139	526	103	160	107	81	146	91	125	130	176	186	100	130	111	124	156	113	283	79	74	71	74
ENSG00000065526	7309	6463	7735	6583	4870	5321	3705	5207	3985	3508	16584	2756	4146	3544	4156	4391	1537	2008	1845	4898	7871	3523	5178	2915	3600	6617	3764	12021	2500	3838	2823	4238
ENSG00000270620	1	3	1	1	1	1	2	3	1	2	8	0	3	0	0	0	0	3	0	0	6	2	1	1	3	6	0	15	0	1	1	1
ENSG00000116809	1373	940	1303	1073	973	931	678	964	431	397	1778	343	500	484	487	586	617	707	804	1096	1545	685	1287	576	313	631	359	1015	339	435	394	542
ENSG00000234607	7	4	3	5	1	5	6	7	4	2	8	3	2	0	1	2	0	1	4	10	4	2	2	4	8	4	1	10	1	1	0	3
ENSG00000231353	0	0	0	0	0	0	0	0	0	0	0	0	0	0	0	0	0	0	0	0	0	0	0	0	0	0	0	0	0	0	0	0
ENSG00000275503	0	0	0	0	0	0	0	0	0	0	0	0	0	0	0	0	0	0	0	0	0	0	0	0	0	0	0	0	0	0	0	0
ENSG00000224966	0	0	0	0	0	0	0	0	0	0	0	0	0	0	0	0	0	0	0	0	0	0	0	0	0	0	0	0	0	0	0	0
ENSG00000183888	0	2	0	1	0	0	0	0	0	0	3	0	0	0	1	0	0	0	0	0	0	0	0	1	0	0	0	0	0	0	0	0
ENSG00000233078	0	0	0	0	0	0	0	0	0	0	0	0	0	0	0	0	0	0	0	0	0	0	0	0	0	0	0	0	0	0	0	0
ENSG00000173641	0	0	0	0	0	0	0	0	0	0	0	0	5	1	0	7	0	0	0	0	0	0	0	0	0	0	0	0	0	0	0	0
ENSG00000186510	0	0	0	0	0	0	0	0	1	0	0	1	3	5	1	6	0	0	0	0	0	0	0	0	0	0	0	0	0	0	0	1
ENSG00000232456	0	0	0	1	0	0	0	0	0	0	0	0	1	1	0	0	0	0	0	0	0	0	0	0	0	0	0	0	0	0	0	0
ENSG00000184908	0	3	2	2	0	0	0	0	2	0	0	0	0	0	0	3	0	0	0	2	0	0	0	0	0	0	0	0	0	1	0	0
ENSG00000185519	0	7	6	10	0	0	0	0	0	0	0	0	4	6	1	7	0	0	0	7	0	0	0	0	0	0	0	0	0	6	1	0
ENSG00000142627	0	0	0	1	101	105	96	92	0	0	5	0	1	4	2	5	0	0	0	0	241	124	165	92	0	0	0	0	3	1	6	4
ENSG00000227959	0	0	0	0	0	0	0	0	0	0	0	0	0	0	1	0	0	0	0	0	0	0	0	0	0	0	0	0	0	0	0	0
ENSG00000224621	0	0	0	0	0	0	0	0	0	0	0	0	0	0	0	1	0	0	0	0	0	0	0	0	0	0	0	0	0	0	0	0
ENSG00000234166	0	0	0	0	0	0	0	0	0	0	0	0	0	0	0	0	0	0	0	0	0	0	0	0	0	0	0	0	0	0	0	0
ENSG00000142632	444	305	383	337	349	335	293	364	234	191	792	129	132	101	170	127	228	325	363	414	821	384	623	361	164	244	198	451	145	162	123	184
ENSG00000237276	5	9	14	12	8	11	5	11	2	5	22	4	19	19	20	20	7	7	1	18	16	2	8	7	9	4	6	19	5	13	5	11
ENSG00000132881	178	122	134	165	60	61	42	68	62	60	215	30	97	61	59	95	72	104	80	131	88	45	68	35	38	65	50	117	53	47	56	73
ENSG00000233929	4	3	1	0	5	0	2	5	6	4	8	3	3	1	2	1	0	1	0	3	12	2	4	3	1	4	1	1	0	4	1	1
ENSG00000037637	1284	1004	1162	924	1143	1075	831	1235	786	628	2630	493	589	576	592	689	343	490	456	692	1693	714	1244	668	595	943	636	1865	360	510	395	622
ENSG00000055070	3900	2722	3735	3324	3983	4177	2838	4270	3607	2879	12092	2373	3182	2745	3263	3613	1151	1666	1804	2679	5791	2508	4371	2066	2973	5038	2488	7666	2394	2745	2330	3044
ENSG00000226457	6	2	5	1	6	4	6	8	2	9	20	1	0	8	6	3	1	2	1	9	6	6	3	8	7	11	5	18	2	4	3	2
ENSG00000187144	8	11	7	8	3	14	6	11	18	2	38	1	8	4	6	11	2	0	4	4	27	6	8	10	3	12	11	27	2	8	11	10

ENSG00000157191	1455	1066	1251	1130	998	1042	707	1083	764	612	3039	509	1575	1441	1787	1920	717	1133	1006	868	1754	739	1341	594	674	1102	716	1836	1638	1557	1832	1687
ENSG00000226029	86	113	120	86	47	61	34	68	66	36	297	27	11	16	17	20	26	22	19	87	59	34	34	28	33	52	101	152	6	20	10	23
ENSG00000080947	371	359	392	328	317	348	214	342	288	310	1989	299	68	84	68	134	76	56	69	261	374	186	197	211	181	250	476	950	42	77	29	93
ENSG00000206652	3	0	0	1	1	0	0	0	0	0	1	1	0	0	0	0	2	1	0	2	1	0	1	1	0	0	0	1	0	0	0	0
ENSG00000261135	29	25	29	27	14	6	10	16	6	3	13	2	10	6	4	9	17	23	13	15	27	16	18	17	5	5	0	4	3	3	3	9
ENSG00000224174	1	2	0	2	0	3	5	0	0	0	1	0	0	0	0	0	3	0	1	0	1	0	3	1	0	0	0	0	0	0	0	0
ENSG00000233421	0	3	0	1	0	0	0	0	0	0	0	0	0	2	1	3	0	0	2	2	0	0	0	0	0	0	0	0	0	1	0	0
ENSG00000277344	0	0	0	0	0	0	0	0	0	0	0	0	0	0	0	0	0	0	0	0	0	0	0	0	0	0	0	0	0	0	0	0
ENSG00000268991	0	0	0	0	0	0	0	0	0	0	0	0	0	0	0	0	0	0	0	0	0	0	0	0	0	0	0	0	0	0	0	0
ENSG00000225710	2	0	0	0	0	0	0	0	0	0	0	0	0	0	0	0	0	0	0	0	0	0	0	0	0	0	0	0	0	0	0	0
ENSG00000219481	1641	1334	1630	1355	970	1034	825	1117	446	463	1654	356	505	591	576	707	518	753	695	1337	1615	706	1133	613	307	642	382	1318	368	594	351	639
ENSG00000271732	11	7	11	9	5	6	3	0	7	5	23	2	7	4	4	4	2	2	1	14	4	2	2	8	4	6	5	24	0	3	3	5
ENSG00000215908	1737	1458	1669	1664	948	954	716	1066	1515	1317	6427	1159	717	628	673	841	558	585	525	1442	1370	710	920	716	982	1776	1419	3830	344	758	444	882
ENSG00000186301	539	453	617	497	267	357	241	349	70	64	308	43	32	40	40	41	221	185	170	458	304	143	183	173	18	22	66	97	15	28	15	34
ENSG00000282143	0	0	0	0	1	0	0	0	0	0	0	0	0	0	0	0	0	0	0	2	1	0	0	0	0	0	1	0	0	0	0	1
ENSG00000207513	1	0	0	0	0	0	0	1	1	0	0	0	0	0	0	0	0	0	0	0	0	0	0	0	0	0	0	0	0	0	0	0
ENSG00000237847	0	0	0	0	0	0	0	0	0	0	0	0	0	0	0	0	0	0	0	0	0	0	0	0	0	0	0	0	0	0	0	0
ENSG00000280114	25	42	26	31	53	64	52	94	0	0	0	0	12	9	6	12	12	6	5	13	77	46	35	39	0	0	0	0	5	6	7	3
ENSG00000236698	23	29	22	25	50	57	46	70	11	4	35	6	19	25	16	32	15	14	14	18	71	39	54	33	8	12	2	23	24	29	13	33
ENSG00000268869	84	93	83	104	151	178	103	123	0	0	1	0	23	31	23	15	12	8	11	53	150	75	79	63	0	0	2	0	5	22	4	11
ENSG00000279151	2	1	0	1	2	1	1	2	0	0	3	0	0	0	1	0	1	0	0	3	2	0	1	0	0	0	0	0	0	0	1	0
ENSG00000279132	0	0	0	0	0	0	0	0	0	0	0	0	0	0	0	0	0	0	0	0	0	0	0	0	0	0	0	0	0	0	0	0
ENSG00000282740	0	0	0	1	0	0	0	0	0	0	0	0	0	0	0	0	0	0	0	0	0	1	0	0	0	0	0	0	0	1	0	0
ENSG00000058453	1174	1162	1268	1371	1586	1793	1211	1873	927	806	4434	683	695	639	591	955	227	366	272	1135	1686	941	1126	837	593	936	982	2414	220	445	235	534
ENSG00000227684	5	3	3	3	0	0	1	0	0	2	4	1	0	0	0	0	1	0	1	0	0	0	1	0	2	2	1	3	0	0	0	0
ENSG00000207389	0	1	0	0	0	0	0	1	0	0	2	0	0	0	0	0	1	0	0	0	0	0	0	0	0	0	0	0	0	0	0	0
ENSG00000186715	157	121	193	128	17	25	26	36	24	7	56	6	0	0	0	2	74	37	39	121	29	20	17	10	7	8	19	35	0	1	2	0
ENSG00000223643	0	0	0	0	0	0	0	0	0	0	0	0	0	0	0	0	0	0	0	2	0	0	0	0	0	0	0	0	0	0	0	0
ENSG00000230239	0	0	0	0	0	0	0	0	0	0	0	0	0	0	0	0	0	0	0	0	0	0	0	0	0	0	0	0	0	0	0	0
ENSG00000263811	0	0	0	0	0	0	0	0	0	0	0	0	0	0	0	0	0	0	0	0	0	0	0	0	0	0	0	0	0	0	1	0
ENSG00000228549	1	3	7	8	0	0	0	0	0	0	0	0	0	1	0	0	0	1	1	3	0	0	0	0	0	0	0	0	0	0	0	0
ENSG00000277234	0	0	0	0	0	0	0	0	0	0	0	0	0	0	0	0	0	0	0	0	0	0	0	0	0	0	0	0	0	0	0	0
ENSG00000238142	0	0	1	0	0	0	0	0	0	0	0	0	5	10	6	9	0	0	1	0	1	0	1	0	0	0	0	2	5	12	9	9
ENSG00000235241	0	0	1	3	1	2	1	0	0	0	2	0	4	4	7	2	0	0	0	0	2	2	1	0	0	0	0	0	5	2	7	7
ENSG00000207005	2	0	0	1	0	0	0	1	0	1	5	4	0	0	0	0	0	1	3	5	1	0	2	1	0	0	1	1	0	1	1	0
ENSG00000272426	0	1	4	0	2	3	1	3	2	1	16	2	3	4	3	7	1	0	2	2	8	8	6	4	1	5	13	10	1	3	1	4
ENSG00000117122	1	1	1	2	5	11	8	7	1	2	9	2	3	3	4	5	1	1	0	1	5	2	3	3	3	5	3	10	1	5	2	5
ENSG00000226526	3	2	2	1	4	2	2	4	1	0	6	0	0	0	5	3	0	1	1	8	7	2	5	2	2	3	3	7	0	2	2	7
ENSG00000159363	1679	1669	1313	1501	2256	2427	1494	2247	755	618	2766	557	615	504	478	679	344	619	401	1047	3900	1779	2525	1465	646	1088	534	1748	458	578	382	573
ENSG00000117118	1639	1119	1361	1012	1267	1271	864	1315	902	636	2811	518	1349	1204	1367	1452	2495	2967	2508	2279	3515	1350	2479	1599	977	1553	571	2004	1844	1633	2168	2044
ENSG00000117115	123	73	135	52	2	2	0	0	8	15	23	3	82	84	96	103	62	64	89	95	2	1	1	3	3	8	6	13	61	77	67	77
ENSG00000204362	0	0	0	0	2	3	0	0	0	0	0	0	0	0	0	0	0	0	0	0	0	1	2	0	0	0	0	0	0	0	0	0
ENSG00000282843	0	0	0	0	0	1	0	0	0	0	0	0	0	0	0	0	0	0	0	0	0	0	0	0	0	0	0	0	0	0	0	0
ENSG00000142623	0	0	0	0	1	0	0	0	0	0	0	0	0	0	1	1	0	0	0	0	1	0	2	0	0	0	0	0	0	0	0	0
ENSG00000142619	0	1	0	0	0	1	0	0	0	0	0	0	0	0	0	0	0	0	0	0	1	0	0	1	0	0	0	1	0	0	0	1
ENSG00000266634	0	0	0	0	0	0	0	0	0	0	0	0	0	0	0	0	0	0	0	0	0	0	0	0	0	0	0	0	0	0	0	0
ENSG00000159339	1	0	1	1	251	287	188	261	0	0	0	0	0	0	0	0	0	2	1	0	570	258	437	215	0	0	0	0	0	0	0	0
ENSG00000280226	0	0	0	0	0	0	0	0	0	0	0	0	0	0	0	0	0	0	0	0	0	0	0	0	1	0	0	0	0	0	0	0
ENSG00000276747	0	0	0	0	0	0	0	0	0	0	1	0	0	1	0	0	0	0	0	0	0	0	0	1	0	0	3	0	0	0	1	0
ENSG00000179051	10599	8606	9574	8940	12617	12650	9124	12309	8134	6723	34719	5961	14281	12403	13052	16132	2097	3579	3346	6534	18175	7846	13345	6461	7356	12549	6635	22748	9310	12400	8854	12541
ENSG00000227751	0	0	0	0	0	0	0	0	0	0	0	0	0	0	0	0	0	0	0	0	0	0	0	0	0	0	0	0	0	0	0	0
ENSG00000074964	0	0	0	0	19	15	17	14	37	53	97	37	332	293	217	357	0	0	0	1	16	4	11	1	12	35	24	130	142	228	113	239
ENSG00000236648	0	0	0	0	0	0	0	0	0	0	0	0	0	0	0	0	0	0	0	0	0	0	0	0	0	0	0	0	0	0	0	0
ENSG00000117148	0	6	0	0	3	0	2	3	5	1	1	2	5	5	9	4	0	0	1	0	6	1	3	1	8	3	0	2	16	10	18	11
ENSG00000261781	0	0	0	0	0	0	0	0	0	0	0	0	0	0	0	0	0	0	0	0	0	0	0	0	0	5	0	0	0	0	0	0
ENSG00000117154	0	0	0	0	0	0	0	0	0	0	0	0	0	0	0	0	0	0	0	0	0	0	0	0	0	0	0	0	0	0	0	0
ENSG00000280222	0	0	0	1	0	0	0	0	0	0	0	0	0	0	0	0	0	2	0	0	0	0	0	0	0	0	0	0	0	0	0	0
ENSG00000230035	0	0	0	0	0	0	0	0	0	0	0	0	0	0	0	0	0	0	0	0	0	0	0	0	0	0	0	0	0	0	0	0
ENSG00000225387	0	0	0	0	0	0	0	0	0	0	0	0	0	0	0	0	0	0	0	0	0	0	0	0	0	0	0	0	0	0	0	0
ENSG00000179023	0	0	0	0	0	0	0	0	0	0	0	0	0	1	0	0	0	0	0	0	0	0	0	0	0	0	0	0	0	0	0	0
ENSG00000235282	0	0	0	0	0	0	0	0	0	0	0	0	0	0	0	0	0	0	0	0	0	0	0	0	0	0	0	0	0	0	0	0
ENSG00000225478	0	0	0	0	0	0	0	0	0	0	0	0	0	0	0	0	0	0	0	0	0	0	0	0	0	0	0	0	0	0	0	0
ENSG00000009709	0	0	2	0	0	0	0	0	0	0	0	0	0	0	0	0	0	0	0	0	0	0	0	0	0	0	0	0	0	0	1	0
ENSG00000179002	0	0	0	0	0	4	0	0	0	0	1	0	0	0	0	0	0	0	1	0	0	0	0	0	0	0	0	0	0	1	2	0
ENSG00000255275	2	2	1	0	4	1	4	1	0	0	2	0	0	0	0	0	0	0	1	1	4	3	1	3	1	3	0	3	0	0	1	0
ENSG00000159423	379	260	347	282	810	741	537	728	154	104	438	94	21	13	26	32	109	167	135	241	1084	416	776	384	124	225	93	320	30	41	23	50
ENSG00000265606	0	0	0	0	0	0	0	0	0	0	0	0	0	0	0	0	0	0	0	0	2	0	0	0	0	0	0	0	0	0	0	0
ENSG00000221662	0	0	1	1	1	1	1	1	2	0	8	0	0	0	0	0	0	0	0	1	3	2	3	0	1	0	1	1	0	0	0	0
ENSG00000169991	103	183	119	195	1304	1326	999	1376	31	39	70	18	674	623	672	804	28	80	66	133	2091	1000	1626	740	29	81	27	121	420	529	420	582
ENSG00000272084	20	22	14	13	31	36	31	39	7	7	45	2	8	5	6	14	7	5	6	9	46	23	23	37	9	16	4	24	2	10	4	9
ENSG00000127481	8659	7762	9320	7865	17602	20348	15122	20789	4376	3968	19538	3108	7660	6507	7447	8346	1608	1979	1912	4482	24616	11862	15951	11016	4076	7649	5237	14917	3402	5736	4323	6330
ENSG00000230424	51	35	49	21	38	40	30	39	37	16	97	13	10	15	19	18	23	10	31	41	28	18	27	25	15	36	13	66	3	15	9	18
ENSG00000127463	2270	1825	2260	1859	2422	2641	2016	2756	1750	1439	6411	1032	1858	1779	2142	2287	561	880	687	1581	3459	1551	2579	1323	1406	2466	1431	4061	1192	1717	1466	1820
ENSG00000053372	1602	999	1337	1103	1472	1544	1173	1562	2149	1316	5291	1187	2543	2117	2756	2811	1608	2408	2119	2167	3694	1416	2750	1274	2048	3051	909	3512	3399	2955	3348	3475
ENSG00000211454	47	52	49	52	25	33	33	34	46	31	156	22	20	22	16	27	26	21	17	84	57	22	31	24	31	58	39	109	11	19	17	29
ENSG00000162482	57	46	56	51	54	40	38	59	44	28	160	23	26	24	16	21	18	20	18	74	62	38	52	40	32	30	43	104	7	20	7	23
ENSG00000270728	89	92	123	75	76	91	77	98	102	84	342	63	49	51	51	70	22	24	37	64	110	60	73	44	64	99	102	250	19	38	28	40
ENSG00000053371	1609	1304	1264	1215	1128	1137	840	1320	1240	984	3512	819	684	712	652	953	1039	1799	1284	1116	2106	823	1609	718	940	1479	721	2508	632	661	624	821
ENSG00000200403	8	8	13	8	3	7	8	14	6	10	31	6	5	1	6	0	3	3	7	11	19	5	15	14	4	9	4	23	5	7	2	7
ENSG00000040487	190	123	169	119	238	234	160	237	89	112	592	89	68	58	55	67	78	86	72	134	400	154	243	145	84	117	166	378	35	35	27	52
ENSG00000077549	8407	6174	7212	6224	8282	8172	6251	8528	4776	3527	15651	2779	4528	3991	4621	5335	1741	3049	3342	4484	12612	5077	9186	4593	4338	6649	3699	10674	3774	4170	3452	4473
ENSG00000240490	0	1	0	0	0	1	1	2	0	1	1	1	2	0	2	0	0	2	0	3	2	2	2	2	2	1	2	5	4	3	1	1
ENSG00000201609	0	0	0	0	0	0	0	0	0	0	0	0	0	0	0	0	0	0	0	0	0	0	0	0	0	0	0	0	0	0	0	0
ENSG00000235185	0	0	0	0	0	0	0	0	0	0	0	0	0	2	0	0	0	0	0	1	2	0	0	0	0	0	0	0	0	0	1	3
ENSG00000173436	882	553	759	647	516	517	456	521	606	463	2174	355	667	552	778	785	1350	1782	1157	1537	1281	596	930	647	729	878	412	1385	914	987	1122	1130
ENSG00000270136	0	1	0	1	1	0	2	2	0	0	2	1	0	0	0	1	0	0	1	1	0	0	1	0	1	0	1	0	1	2	1	3
ENSG00000226396	25	19	13	19	24	26	8	19	54	25	165	34	46	41	47	51	6	5	3	15	37	17	8	13	40	51	41	108	33	45	49	49
ENSG00000158747	9	21	4	26	0	0	0	0	155	116	585	122	77	84	72	97	0	3	0	1	2	1	0	0	146	245	149	475	45	52	48	66
ENSG00000158748	0	0	0	0	1	1	1	3	0	0	2	0	0	2	2	1	0	0	0	0	5	1	4	1	2	3	1	2	0	1	4	3
ENSG00000162542	1	5	3	3	565	568	405	651	177	141	727	120	463	442	480	536	1	1	3	5	947	415	762	341	141	227	175	466	302	432	398	494
ENSG00000178828	0	0	0	0	0	1	0	0	0	0	0	0	0	0	0	0	0	0	0	0	0	0	0	0	0	0	0	0	0	0	0	0
ENSG00000235434	0	0	0	0	0	0	0	0	0	0	0	0	0	0	0	0	0	0	0	0	0	0	0	0	0	0	0	0	0	0	0	0
ENSG00000169914	1685	1426	1355	1476	940	1026	735	1055	706	611	2531	445	913	905	739	1149	375	460	356	1174	1491	698	884	638	477	723	529	1450	340	782	341	786
ENSG00000188784	0	0	0	0	0	0	2	0	0	0	0	0	0	0	0	0	0	0	0	2	1	0	0	0	0	0	0	0	0	1	1	0
ENSG00000242688	0	0	0	0	0	0	0	0	0	0	2	0	0	0	0	0	0	0	0	0	0	0	0	0	0	0	0	1	0	0	1	0
ENSG00000188257	1	1	0	2	0	0	0	0	0	0	0	0	3	1	1	1	0	0	0	0	0	0	0	0	0	0	0	0	0	7	1	3
ENSG00000127472	0	1	0	2	2	8	1	7	0	0	0	1	5	6	11	9	0	0	0	0	4	10	3	6	0	0	0	0	7	13	6	8
ENSG00000117215	8	4	2	15	0	2	0	0	0	0	0	0	0	1	0	0	1	0	0	0	0	0	0	0	0	0	0	0	0	0	0	1
ENSG00000158786	14	17	28	6	0	0	0	0	0	0	0	0	1	6	0	3	0	0	3	13	0	0	0	0	0	1	0	0	0	1	0	2
ENSG00000227066	12	4	19	11	0	0	0	0	0	0	0	0	1	1	0	0	2	0	0	11	0	0	0	0	0	0	0	0	0	0	0	2
ENSG00000187980	57	39	59	47	0	2	3	3	0	0	0	0	2	1	1	2	15	9	28	32	0	2	1	3	0	0	0	0	0	4	3	1
ENSG00000225986	181	110	162	153	5	12	4	5	0	0	0	0	5	14	6	15	169	202	336	185	8	5	7	10	0	0	0	0	15	19	27	29
ENSG00000162543	326	282	284	272	32	69	24	77	0	0	0	0	36	59	52	94	136	215	216	322	66	30	30	36	0	0	0	0	64	116	104	127
ENSG00000228105	0	0	0	0	0	0	0	0	0	0	0	0	0	0	0	0	0	0	0	0	0	0	0	0	0	0	0	0	0	0	0	0
ENSG00000158816	0	0	2	2	0	0	0	0	0	0	0	0	0	0	0	1	0	2	0	1	0	0	0	0	0	0	0	0	0	0	0	0
ENSG00000226664	0	0	0	0	0	0	0	0	0	0	0	0	0	0	0	0	0	0	0	0	0	0	0	0	0	0	0	0	0	0	0	0
ENSG00000236963	0	0	0	0	0	0	0	0	0	0	1	0	1	0	0	0	0	0	0	1	0	0	1	0	0	0	0	0	0	0	0	0
ENSG00000226487	0	0	0	0	0	0	0	0	0	0	0	0	0	0	0	0	0	0	0	0	0	0	0	0	0	0	0	0	0	0	0	0
ENSG00000162545	310	288	273	307	470	489	348	437	13	12	27	8	1	4	2	9	46	90	98	171	688	296	509	253	6	26	5	37	0	1	3	2
ENSG00000090432	471	338	431	331	468	571	381	567	361	348	1717	302	555	433	641	671	211	319	295	385	933	353	814	305	374	641	438	1069	535	577	586	625
ENSG00000215520	0	0	0	0	0	0	0	0	0	0	0	0	0	0	0	0	0	0	0	0	0	0	0	0	0	0	0	0	0	0	0	0
ENSG00000183114	0	1	0	0	0	0	0	0	0	0	0	0	0	2	0	1	0	0	0	0	0	0	0	0	0	0	0	0	0	0	1	0
ENSG00000158825	0	0	0	0	0	0	2	2	0	0	0	0	1	2	0	2	0	0	0	0	1	0	0	0	0	0	0	0	0	4	0	0
ENSG00000158828	299	301	288	347	318	374	206	377	83	81	625	76	75	91	66	132	106	167	183	272	546	233	423	189	119	174	250	432	76	123	91	117
ENSG00000117242	215	181	216	175	231	270	147	259	171	124	549	93	51	71	65	71	84	162	90	207	353	147	238	135	141	181	94	341	62	62	29	59
ENSG00000244038	3767	2595	2921	2701	6636	6891	4287	7041	3440	2761	11463	2244	4282	3855	4529	5562	1483	2601	2128	2510	9984	4101	7259	3149	2746	4786	2459	7488	3739	4355	4135	4741
ENSG00000117245	3	7	11	9	16	19	4	12	1	3	1	0	17	13	18	27	2	2	3	5	19	12	13	3	0	0	0	1	4	6	10	8
ENSG00000235432	0	0	2	0	0	0	0	0	0	0	0	0	0	0	1	0	1	0	0	0	0	0	0	0	1	0	1	0	0	0	0	0
ENSG00000189410	0	3	2	0	43	60	21	52	3	1	2	2	143	134	83	180	0	0	0	2	31	16	24	25	0	1	1	8	33	62	14	54
ENSG00000233069	0	0	0	0	0	2	1	0	2	0	2	0	2	0	1	0	0	1	0	0	2	0	0	0	3	0	0	1	1	1	0	2
ENSG00000127483	11012	7801	10263	8146	9689	9854	7254	10306	7258	6325	29545	4985	3633	3437	4123	4716	2403	2868	3286	5237	14011	6157	10387	5318	6260	9362	5668	17745	2058	3418	2558	4063
ENSG00000075151	3421	2635	3291	2717	2238	2596	1938	2691	2519	2011	10854	1715	2675	2508	3072	3237	1088	1312	1521	2399	3793	1688	2724	1582	2283	3823	2243	6628	1666	2382	2024	2757
ENSG00000251914	0	0	4	0	1	0	0	3	0	0	1	0	0	0	1	1	0	0	0	1	0	0	0	0	0	0	0	1	0	0	0	0
ENSG00000221808	0	1	2	0	0	0	0	0	0	3	3	0	0	0	0	0	1	0	0	4	1	0	0	0	0	2	0	4	0	0	3	1
ENSG00000233072	3	2	1	2	3	0	2	0	8	4	8	2	2	0	2	6	1	0	1	6	6	2	4	6	3	5	0	6	0	8	3	5
ENSG00000236073	3	1	7	0	0	3	1	0	4	1	11	0	2	1	1	1	2	3	0	4	2	2	3	3	2	4	1	11	1	3	1	2
ENSG00000235112	11	10	6	13	26	25	18	34	4	8	39	3	9	12	10	4	2	5	2	12	34	32	30	29	9	18	10	38	1	10	12	11
ENSG00000117298	1791	1767	1660	1730	726	811	537	840	759	724	2613	680	625	696	743	1011	301	563	524	1119	1137	485	734	392	496	1165	526	2024	471	598	460	743
ENSG00000236936	6	13	3	3	1	0	3	4	2	1	11	2	1	4	2	0	0	0	1	17	5	1	1	1	2	2	0	12	0	1	0	2
ENSG00000231105	35	26	26	13	3	4	4	5	6	11	20	3	14	15	14	19	5	2	7	16	8	4	2	3	4	13	5	18	11	9	7	12
ENSG00000236216	0	0	0	0	0	0	0	0	0	0	0	0	0	0	0	0	0	0	0	0	0	0	0	0	0	0	0	0	0	0	0	0
ENSG00000228823	0	0	0	0	0	0	0	0	0	0	0	0	0	0	0	0	0	0	0	0	0	0	0	0	0	0	0	0	0	0	0	0
ENSG00000236009	0	0	0	0	0	0	0	0	0	0	0	0	0	0	0	0	0	0	0	0	0	0	0	0	0	0	0	0	0	0	0	0
ENSG00000227001	1	3	1	4	27	18	17	28	9	14	44	12	18	27	37	27	1	1	1	0	25	11	12	15	16	19	12	32	10	29	9	27
ENSG00000187952	8	3	8	6	27	39	25	24	2	0	0	1	4	2	4	6	1	0	1	3	37	12	30	20	0	1	0	0	2	6	5	8
ENSG00000186543	0	0	0	0	0	0	0	0	0	0	0	0	0	0	0	0	0	0	0	1	0	0	0	0	0	0	0	0	0	0	0	0
ENSG00000142794	14	66	22	52	816	936	576	932	164	197	621	122	487	462	389	579	2	13	9	13	1209	505	772	428	140	299	211	568	241	452	238	460
ENSG00000176378	0	0	0	0	0	2	2	3	0	0	0	0	0	3	0	3	0	0	0	0	0	2	1	4	0	0	0	0	0	2	0	1
ENSG00000162551	0	0	0	0	0	0	0	0	0	7	7	3	0	1	1	5	0	0	0	0	0	0	0	0	0	9	0	5	1	1	0	5
ENSG00000233431	0	0	0	0	0	0	0	0	0	0	0	0	0	2	1	3	0	0	0	0	0	0	0	0	0	0	0	0	0	1	2	1
ENSG00000076864	786	727	731	795	7	2	1	3	4	6	16	10	369	366	309	439	126	256	244	545	4	5	3	2	0	8	1	8	122	211	164	235
ENSG00000090686	3253	2367	2616	2493	2613	2755	1927	2957	3274	2677	11440	1992	2304	2169	2348	2726	1373	1797	1530	2129	4416	1890	3103	1871	2757	4119	2503	7611	1691	2267	2101	2571
ENSG00000231978	7	2	5	3	0	1	1	0	1	3	14	1	0	1	0	1	1	1	0	4	5	2	1	4	3	2	4	11	0	2	2	1
ENSG00000187942	1	4	6	0	0	1	0	0	7	5	24	6	13	10	11	23	0	0	0	2	0	0	0	0	2	1	9	21	1	2	1	6
ENSG00000142798	35	120	58	122	9	8	3	8	88	135	1378	170	2090	2112	1510	2554	1	1	0	12	0	1	2	4	17	40	175	490	336	800	357	985
ENSG00000232037	0	0	0	0	0	0	0	0	1	0	5	0	0	3	0	0	0	0	0	0	0	0	0	0	0	0	0	1	0	2	0	0
ENSG00000283234	0	0	0	0	0	0	0	0	0	0	6	1	9	10	8	16	0	0	0	0	0	0	0	0	0	0	1	1	10	7	12	13
ENSG00000219073	0	0	0	0	0	0	2	0	0	0	0	0	0	0	0	0	0	0	0	0	0	0	1	0	0	0	0	0	0	0	0	0
ENSG00000273610	0	0	0	0	0	0	0	0	0	0	0	0	0	0	0	0	0	0	0	0	0	0	0	0	0	0	0	0	0	0	0	0
ENSG00000201919	0	0	0	0	0	0	0	0	0	0	0	0	0	0	0	0	0	0	0	0	0	0	0	0	0	0	0	0	0	0	0	0
ENSG00000142789	0	0	0	0	0	0	0	0	0	0	0	0	1	0	0	0	0	0	0	0	0	0	0	0	0	0	0	0	0	0	0	0
ENSG00000278124	0	0	0	0	0	0	0	0	0	0	0	0	0	0	0	0	0	0	0	0	0	0	0	0	0	0	0	0	0	0	0	0
ENSG00000201273	0	0	0	0	0	0	0	0	0	0	0	0	0	0	0	0	0	0	0	0	0	0	0	0	0	0	0	0	0	0	0	0
ENSG00000228397	1	5	0	1	6	6	5	4	4	1	17	6	11	4	5	10	3	0	13	1	7	3	10	3	3	8	3	12	7	1	6	11
ENSG00000218510	463	318	422	367	194	194	134	200	432	356	1216	301	215	233	208	295	332	439	370	417	401	153	303	172	268	397	245	684	175	255	231	295
ENSG00000070831	6175	3812	5082	3751	5747	5658	3999	5905	4618	3635	17532	2976	6598	6009	6795	7548	6345	8699	7816	5722	14661	5395	9711	5226	5012	6883	4123	11237	7212	6754	8185	7929
ENSG00000230068	8	2	15	2	7	6	2	9	4	12	23	2	14	7	10	5	3	1	2	12	13	8	3	7	7	7	6	30	1	7	6	10
ENSG00000271428	9	10	10	6	1	4	1	5	6	4	18	3	5	11	12	2	3	1	2	3	12	7	4	3	4	7	6	30	1	4	7	14
ENSG00000271840	4	5	5	2	11	6	4	5	7	8	27	4	8	9	7	10	1	0	4	3	18	5	6	10	10	11	6	24	0	4	9	13
ENSG00000162552	1	3	3	6	5	7	10	11	10	7	29	0	5	10	12	15	0	0	1	3	16	18	11	16	12	15	16	48	6	3	10	12
ENSG00000266564	0	0	0	0	0	0	0	0	0	0	0	0	0	0	0	0	0	0	0	0	0	0	0	0	0	0	0	0	0	0	0	0
ENSG00000234397	0	0	0	0	0	0	0	0	0	0	0	0	0	0	0	0	1	0	0	0	0	0	0	0	0	0	0	0	0	0	0	0
ENSG00000279625	0	0	0	0	0	0	0	0	0	0	0	0	1	1	0	0	0	0	0	0	0	0	0	0	0	0	0	0	0	0	0	3
ENSG00000184677	1765	1518	1762	1525	2233	2307	1837	2452	1052	967	5759	819	881	828	1010	1178	544	638	620	1593	3940	1822	2641	1804	872	1527	1511	3384	484	942	635	1051
ENSG00000237200	17	12	12	13	7	11	5	6	3	7	30	6	6	12	8	15	6	1	2	11	11	7	2	7	3	12	5	31	3	9	5	4
ENSG00000070886	0	0	1	0	1	1	0	2	0	0	4	1	0	0	0	0	0	0	0	0	1	4	2	0	0	2	0	0	0	0	0	0
ENSG00000276835	0	0	0	0	0	0	0	0	0	0	0	0	0	0	0	0	0	0	0	0	0	0	0	0	0	0	0	0	0	0	0	0
ENSG00000173372	0	0	0	0	0	0	0	0	0	0	1	1	0	0	0	0	0	0	0	0	0	0	1	0	0	0	2	3	0	0	0	0
ENSG00000159189	0	0	0	0	0	0	0	0	0	0	0	0	0	0	0	0	0	0	0	0	0	0	1	0	0	0	0	0	1	0	0	0
ENSG00000173369	0	0	0	0	0	0	0	0	0	0	0	0	0	0	0	0	0	0	0	0	0	0	0	0	0	0	0	0	0	0	0	0
ENSG00000133216	724	1128	792	1252	552	611	403	677	0	1	5	1	88	152	52	171	165	444	150	602	566	242	360	197	0	2	1	1	66	162	44	138
ENSG00000265422	1	1	0	0	0	0	0	0	0	0	0	0	0	0	0	0	0	0	0	0	0	2	0	0	0	0	0	0	0	0	0	0
ENSG00000225952	0	6	4	3	1	1	0	1	0	0	0	0	0	0	0	0	0	1	1	2	5	1	3	2	0	0	0	0	0	1	0	0
ENSG00000264014	0	1	0	1	0	0	0	0	0	0	0	0	0	0	0	0	0	0	0	1	0	0	0	0	0	0	0	0	0	0	0	0
ENSG00000215906	1	0	0	0	0	0	0	0	0	0	0	0	1	0	0	0	0	0	0	0	0	0	0	0	0	0	0	0	0	0	0	0
ENSG00000227868	7	10	11	4	3	1	0	3	3	0	6	0	1	1	2	7	3	4	3	6	1	0	1	0	0	0	0	0	0	2	0	0
ENSG00000004487	7838	5918	6531	6104	7704	7330	5381	7529	3224	2591	9320	1839	4284	3816	3924	4942	1868	3245	2728	4441	10209	4227	7352	3707	2429	4094	1864	6001	2701	3595	2588	3850
ENSG00000240553	3	4	5	4	5	6	7	3	4	2	28	0	3	2	6	2	2	0	1	9	7	2	4	5	7	3	13	12	3	3	1	2
ENSG00000263793	1	0	2	0	0	0	1	0	0	1	0	0	0	0	0	1	0	0	0	1	1	0	0	2	0	0	1	4	0	2	0	0
ENSG00000266802	4	4	2	5	3	1	1	4	0	0	2	0	0	6	0	1	0	0	0	7	5	1	0	4	2	3	0	5	0	0	3	1
ENSG00000169641	2070	1880	1767	2008	1709	1827	1236	1823	651	630	2520	514	602	690	635	824	548	777	700	1307	2856	1166	1877	1011	569	1014	520	1945	355	570	405	694
ENSG00000229010	4	3	6	5	4	2	1	6	0	0	0	2	1	3	1	2	1	1	0	4	9	2	3	11	1	4	0	1	0	0	2	3
ENSG00000206935	6	4	0	2	0	2	2	3	3	0	1	0	0	1	2	1	0	3	0	2	4	4	1	3	1	0	0	1	0	0	0	0
ENSG00000252578	0	3	2	7	4	0	1	2	0	3	1	0	2	0	1	1	1	1	0	1	4	2	1	1	1	1	1	3	0	0	0	0
ENSG00000179546	0	1	0	0	0	0	0	0	0	1	2	0	0	0	0	1	0	1	0	0	0	0	1	0	0	0	0	1	0	0	1	2
ENSG00000215381	0	0	0	0	0	0	0	0	0	0	0	0	0	0	0	0	0	0	0	0	0	0	0	0	0	0	0	0	0	0	0	0
ENSG00000261326	710	572	591	524	421	451	342	489	442	380	1424	213	262	269	186	300	105	125	149	437	433	219	239	270	302	454	260	804	58	196	116	260
ENSG00000125944	11964	7801	10156	7961	12379	12603	9656	13147	8909	6663	25773	5143	8716	7950	8840	10370	5272	8632	8283	8743	23138	9029	17923	8095	8645	12900	4600	19073	7862	9091	7761	9390
ENSG00000125945	634	511	574	481	841	837	553	1014	194	185	634	135	223	222	276	295	205	253	279	336	748	373	596	306	186	274	188	498	162	196	178	206
ENSG00000249087	393	357	342	317	367	330	243	421	74	68	212	50	81	78	54	81	61	77	69	104	217	92	114	86	37	63	41	156	23	37	15	35
ENSG00000201405	0	0	0	0	0	0	0	0	0	0	0	0	0	0	0	0	0	0	0	0	0	0	0	0	0	0	0	0	0	0	0	0
ENSG00000271420	0	1	1	1	11	10	12	18	0	0	0	0	0	0	0	0	0	0	0	3	5	1	3	6	1	0	0	0	0	0	0	0
ENSG00000204219	16	11	9	15	1065	1206	886	1386	1	3	3	2	7	11	9	19	9	13	14	15	666	289	506	306	1	0	0	6	0	10	5	1
ENSG00000232482	0	0	0	0	1	3	3	9	0	0	1	0	0	0	0	0	0	0	0	0	1	4	3	2	0	0	0	0	1	0	0	0
ENSG00000088280	58	59	53	96	322	371	254	331	1	11	8	14	3	21	7	24	7	19	8	26	480	232	326	221	2	13	3	21	0	22	5	18
ENSG00000007968	2926	2367	2968	2527	1402	1493	1335	1604	1067	892	2495	628	681	515	631	802	619	754	1024	1753	2814	1109	2098	1071	850	1444	428	2657	332	515	346	576
ENSG00000235052	33	48	62	27	6	17	5	18	4	2	7	1	2	5	2	7	14	5	10	26	8	14	9	3	3	2	0	4	0	1	2	5
ENSG00000117318	3743	2277	3431	2276	361	472	294	542	2075	1850	4951	1334	807	890	865	978	1580	1833	2715	2170	755	397	758	352	1838	2913	1324	4195	1371	1414	1374	1432
ENSG00000197880	1	2	2	1	0	7	1	2	0	0	6	0	6	2	4	4	1	0	0	3	1	1	0	0	0	0	1	1	3	1	1	3
ENSG00000229239	0	0	0	0	0	0	0	0	0	0	0	0	0	0	0	0	0	0	0	0	0	0	0	0	0	0	0	0	0	0	0	0
ENSG00000142676	17861	12295	11796	12746	11819	13110	8845	14470	20730	13844	55569	11395	18973	17961	20877	25780	18611	23025	21703	16784	28746	11914	21430	12209	16111	22819	11240	33891	23556	22315	32339	29394
ENSG00000011007	1722	1227	1804	1365	1423	1429	1196	1545	1167	952	4384	812	1436	1222	1524	1666	785	926	1104	1613	2924	1115	2160	1059	1051	1734	1032	3028	1464	1706	1498	1824
ENSG00000236810	87	61	94	72	27	33	25	26	57	54	159	26	46	24	50	58	90	70	80	66	45	23	38	14	16	27	39	109	31	38	29	37
ENSG00000057757	1671	1128	1492	1219	1284	1399	1007	1319	1206	912	3873	786	1287	1126	1429	1638	808	1217	1217	1310	2367	982	2066	852	1212	1802	821	2683	1209	1305	1371	1429
ENSG00000011009	1343	910	1154	1076	1246	1169	844	1243	1179	930	4255	838	1057	903	1036	1235	1086	1376	1191	1180	2528	923	1811	820	1005	1603	859	2493	1201	1181	1091	1223
ENSG00000117308	735	406	605	490	578	577	408	624	327	242	888	224	661	527	668	691	465	591	618	569	941	396	689	334	253	435	108	551	581	549	585	711
ENSG00000117305	619	479	463	448	613	684	453	691	442	405	1657	313	426	399	416	512	235	445	343	345	711	307	506	263	296	406	386	962	197	266	279	314
ENSG00000179163	339	267	246	238	192	229	106	230	2018	1760	17011	2314	508	426	488	576	102	179	143	202	309	127	252	120	2078	3461	3595	9269	378	458	395	501
ENSG00000188822	112	38	108	50	339	327	265	397	861	780	4885	673	364	312	353	384	59	45	45	81	733	249	498	233	893	1499	1192	2937	408	543	487	616
ENSG00000229106	11	2	9	2	60	56	47	57	86	77	529	70	30	29	41	56	1	5	1	5	98	52	68	38	90	140	130	358	39	88	81	95
ENSG00000232557	2	0	3	0	8	6	8	5	17	8	60	6	8	5	8	7	0	0	0	0	18	7	4	8	10	13	14	59	1	4	8	14
ENSG00000264926	0	0	0	0	0	0	0	0	0	0	1	0	0	0	0	1	0	0	0	0	0	1	0	0	0	0	0	0	0	0	0	1
ENSG00000230228	4	2	1	0	0	0	0	0	0	0	0	0	0	0	0	0	0	1	1	1	0	0	0	1	0	0	0	0	0	0	0	2
ENSG00000189266	4834	3301	4387	3212	2983	2993	2535	3222	3621	3003	11904	2294	2248	2317	2410	2934	3804	5110	4555	3828	7124	2664	5841	2785	3738	5227	2287	8217	2358	2557	2675	2832
ENSG00000188529	8145	5438	6674	5610	8364	8691	6844	9552	7873	6459	23773	4488	5803	5515	6412	7217	5481	6774	6114	7458	18402	7947	12420	8261	7009	10137	4579	16440	4714	6603	5620	7516
ENSG00000215905	0	0	0	0	0	0	0	0	0	0	1	0	0	0	0	0	0	0	0	0	0	0	0	0	0	0	0	0	0	2	0	0
ENSG00000225315	0	0	0	0	2	0	1	1	0	0	2	0	0	1	0	0	0	0	0	0	0	0	0	0	0	0	0	0	1	0	0	0
ENSG00000142661	0	0	0	1	0	0	0	0	0	0	0	0	0	0	0	0	0	3	0	2	1	0	0	0	0	0	1	1	0	0	1	0
ENSG00000230703	0	0	0	1	0	0	0	0	0	0	0	0	0	0	0	0	0	0	0	0	0	0	0	0	0	0	0	0	0	0	0	0
ENSG00000142677	9	32	13	25	0	0	0	0	4	7	17	8	0	1	0	1	0	4	1	6	0	0	0	0	5	13	2	16	0	1	0	0
ENSG00000185436	7	11	8	25	132	150	78	168	7	1	5	1	119	137	164	186	0	15	5	24	170	66	124	49	0	4	0	1	70	90	81	124
ENSG00000230023	0	0	0	2	0	0	0	0	0	0	0	0	0	0	0	0	1	2	0	3	0	0	0	0	0	0	0	0	0	0	0	1
ENSG00000232298	0	0	0	0	0	0	0	0	0	0	0	0	1	0	0	0	0	0	0	0	0	0	1	0	0	0	0	0	0	0	0	0
ENSG00000158055	0	0	0	0	1	1	0	3	0	2	12	3	4	3	1	9	0	0	0	0	0	0	1	0	1	2	0	4	5	13	6	20
ENSG00000001460	24	19	32	29	118	115	71	125	67	61	233	57	280	224	332	364	7	10	22	23	165	67	132	50	74	133	46	193	216	246	258	322
ENSG00000001461	1591	1125	1229	1259	830	834	563	882	1053	861	4699	803	1685	1448	1777	1924	426	620	534	806	1005	435	863	378	894	1217	1128	2986	1203	1479	1522	1661
ENSG00000117602	797	658	769	711	1547	1505	1144	1630	1256	1039	4535	714	2077	1989	2276	2443	214	361	292	589	2518	1066	1962	912	1252	1747	1178	3074	1929	2402	2030	2457
ENSG00000264443	0	0	0	0	0	0	1	0	0	0	0	0	0	0	0	0	0	0	0	0	0	0	0	0	0	0	0	0	0	0	0	0
ENSG00000184454	0	0	1	0	0	0	0	0	0	0	1	3	0	0	2	0	0	1	0	0	0	0	0	0	0	0	1	1	0	0	1	0
ENSG00000227312	0	0	1	0	0	0	0	0	0	0	0	0	0	0	0	0	0	0	0	0	0	0	0	0	0	0	1	0	0	0	0	0
ENSG00000239106	0	0	0	0	0	0	0	0	0	0	0	0	0	0	0	0	0	0	0	0	0	0	0	0	0	0	0	0	0	0	2	0
ENSG00000133226	5576	4105	4818	4134	5713	5285	4427	5699	3412	2727	11486	1941	4031	3516	3934	4160	1355	1718	1566	2837	8598	3601	6071	3112	2983	4584	2333	7995	2661	3472	3018	3667
ENSG00000169504	4474	3146	3692	3107	5456	5314	4187	5750	821	663	2279	446	5113	4584	4860	5795	2113	2654	2153	2798	13879	5104	9609	4992	816	1112	503	1846	3221	3595	3852	4572
ENSG00000238482	3	3	3	3	5	2	3	5	0	0	2	0	3	2	3	2	1	0	4	4	7	4	9	8	1	0	1	6	0	3	1	1
ENSG00000020633	900	944	960	946	2776	2571	1958	2919	7701	6751	26984	5125	5193	4714	5674	5911	203	422	352	733	3762	1513	2588	1338	6126	9904	4823	16603	4733	5089	4121	5539
ENSG00000278034	0	0	0	0	3	1	0	3	3	6	45	7	3	5	5	7	0	0	0	0	6	4	3	3	4	13	4	24	1	4	1	6
ENSG00000229162	8	2	4	3	30	37	14	60	47	52	171	27	8	6	5	7	0	0	0	4	37	22	10	22	46	62	36	219	1	12	7	12
ENSG00000261025	0	2	0	0	1	1	0	0	0	0	0	0	0	0	0	0	0	0	0	0	1	0	0	0	0	0	0	0	0	0	0	1
ENSG00000264371	0	0	0	0	0	0	0	1	1	0	1	0	0	0	0	0	0	0	0	0	1	0	0	0	0	1	0	0	0	0	0	0
ENSG00000233755	0	0	0	0	2	3	1	4	1	1	17	0	1	1	0	2	0	0	0	0	1	1	1	3	1	0	0	3	0	0	0	0
ENSG00000233419	0	0	0	0	0	0	0	0	0	0	0	0	0	1	0	0	0	1	0	0	0	0	0	0	0	0	0	0	0	0	0	0
ENSG00000231953	1	0	0	0	0	0	0	0	0	0	2	0	0	0	0	0	0	0	0	0	0	0	0	1	0	0	2	1	0	0	0	0
ENSG00000117614	1311	707	901	826	840	922	833	1025	1138	865	4566	727	1074	1055	1226	1381	1433	1527	1336	1105	2618	1024	2052	1080	1021	1442	1255	3008	1288	1326	1693	1717
ENSG00000117616	2427	1809	2236	1868	2284	2425	1767	2569	2450	2161	12865	1874	1271	1254	1423	1614	1076	1099	1192	1622	3880	1825	2568	2074	2031	2782	3267	6122	891	1353	1125	1646
ENSG00000272432	22	13	8	6	7	11	4	10	5	6	21	7	5	9	4	5	4	7	4	9	15	12	4	6	9	8	10	18	3	12	1	2
ENSG00000261349	123	82	86	82	49	60	30	33	51	25	145	33	0	0	0	0	35	16	18	44	35	23	25	13	34	53	27	62	0	0	0	0
ENSG00000187010	100	85	109	100	44	63	34	61	68	54	230	33	1	1	1	2	37	25	14	93	58	27	50	42	44	52	79	114	0	0	0	0
ENSG00000224183	25	21	23	10	6	10	3	12	4	8	27	6	0	0	0	0	7	7	6	21	9	3	11	2	6	14	5	10	0	0	0	0
ENSG00000259984	21	12	10	9	9	7	10	6	8	5	28	7	7	9	4	6	0	3	0	2	7	4	10	4	3	5	15	22	3	3	7	8
ENSG00000183726	2247	1493	1695	1491	1759	1857	1254	1960	1863	1394	7524	1210	1826	1741	2235	2381	1726	2202	1931	1569	3915	1504	2817	1396	2033	2911	1963	5043	1806	2121	2154	2457
ENSG00000188672	40	24	18	23	15	14	8	15	5	4	28	5	19	12	7	24	5	9	3	8	25	13	10	9	4	8	6	19	4	5	5	5
ENSG00000238084	0	0	3	0	0	1	1	1	1	1	2	0	1	2	0	1	0	1	1	1	1	0	2	1	3	3	0	1	0	0	1	2
ENSG00000204178	1339	871	1073	865	1008	1032	874	979	707	554	2916	479	846	834	909	1019	537	590	654	800	2627	1012	1800	955	585	1016	791	2045	674	949	842	1070
ENSG00000157978	171	230	194	275	718	810	554	755	61	109	158	73	345	427	431	612	29	106	54	200	1456	687	1077	579	43	131	72	348	327	494	359	562
ENSG00000225643	0	5	2	0	3	4	1	6	0	0	0	1	1	0	1	5	0	0	0	3	4	1	6	2	0	1	2	0	4	2	2	6
ENSG00000117643	1	11	10	8	3	3	4	4	537	695	1880	515	39	45	24	31	0	0	1	4	0	3	3	3	388	1047	732	2256	14	28	15	27
ENSG00000233478	0	2	0	2	0	0	1	0	12	13	24	6	4	0	2	1	0	0	0	1	0	0	0	0	11	16	22	68	0	0	0	3
ENSG00000162430	212	512	248	477	543	568	299	581	163	200	324	141	101	148	74	197	30	166	57	271	471	186	297	153	77	258	133	540	56	130	53	128
ENSG00000255054	0	0	0	0	0	0	0	0	0	0	0	0	0	0	0	0	0	0	0	0	0	0	0	0	0	0	0	0	0	0	0	0
ENSG00000228172	35	38	48	43	11	23	19	25	10	28	91	18	13	10	11	20	4	12	16	43	28	18	12	8	9	27	21	59	8	13	3	10
ENSG00000117640	823	579	775	624	734	626	503	798	688	590	2548	454	422	411	380	493	516	648	543	678	1251	506	891	481	462	684	625	1363	348	406	308	485
ENSG00000272278	0	0	0	0	0	0	0	0	0	0	0	0	0	0	0	0	0	0	0	0	0	0	0	0	0	0	0	0	0	0	0	0
ENSG00000127423	545	365	389	374	280	293	223	320	237	176	666	146	345	296	411	338	313	462	344	410	730	264	556	286	308	456	95	539	302	365	377	417

ENSG00000236528	1	1	2	3	3	0	1	0	0	1	1	0	0	4	0	1	1	2	0	4	1	1	1	2	1	1	0	1	0	1	3	0
ENSG00000182749	102	95	122	86	31	24	13	33	283	246	885	185	92	80	63	76	31	67	57	106	40	23	30	31	142	275	254	486	34	45	36	66
ENSG00000207302	0	0	0	0	0	0	1	0	1	0	1	0	1	0	1	1	0	0	0	1	1	1	1	0	1	2	3	4	0	2	1	5
ENSG00000117632	24998	16205	20764	16261	15032	14939	11037	16143	10982	9073	28951	6724	6439	6818	6470	8358	29875	32758	30706	21983	40334	14194	26025	16539	10346	14372	4090	23731	7545	8049	7641	9956
ENSG00000229786	2	2	3	2	11	6	9	4	3	1	13	2	1	4	6	6	0	0	0	0	7	7	3	12	4	4	5	12	2	2	1	2
ENSG00000223624	10	4	7	4	5	11	3	12	8	5	6	1	6	4	6	2	0	0	0	1	13	9	2	4	0	6	0	10	1	1	3	8
ENSG00000158006	467	295	383	335	334	291	279	305	208	168	652	100	155	122	164	175	137	202	237	308	549	218	419	174	183	247	141	416	95	134	123	134
ENSG00000207237	0	0	0	0	0	0	0	0	0	0	0	0	0	0	1	0	0	0	0	1	2	1	0	1	0	0	1	0	0	1	0	0
ENSG00000252190	0	0	0	0	0	0	0	0	0	0	0	0	0	0	0	0	0	0	0	0	0	0	0	0	0	0	0	0	0	0	0	0
ENSG00000252691	0	0	2	0	0	0	0	0	0	0	0	0	0	0	0	0	0	0	0	0	0	0	0	0	0	0	0	0	0	0	0	0
ENSG00000158008	29	21	32	22	11	9	14	8	1	0	0	0	0	2	0	2	2	7	16	23	8	6	1	5	0	0	0	0	0	0	1	3
ENSG00000158014	1	1	4	3	1	1	1	0	1	0	0	0	0	0	0	0	1	0	5	0	0	1	0	0	0	0	0	0	0	0	1	0
ENSG00000158022	0	0	0	0	0	0	1	0	1	2	5	3	0	0	0	0	0	0	1	0	0	0	1	0	0	2	0	0	0	0	0	0
ENSG00000175087	1374	815	978	729	710	732	550	790	382	331	1270	245	428	397	498	516	778	982	883	953	1447	620	1160	601	451	794	271	1157	381	454	397	522
ENSG00000197245	21	25	16	21	134	151	105	134	0	0	1	0	2	0	0	1	6	9	6	13	299	106	318	107	0	1	0	1	0	2	0	0
ENSG00000282872	0	1	0	1	0	0	0	1	0	0	0	0	0	0	0	0	1	0	0	0	1	0	0	1	0	0	0	0	0	0	0	0
ENSG00000236782	19	17	17	13	11	11	1	6	4	7	48	11	2	5	4	7	13	18	13	16	21	6	15	15	13	10	6	21	10	11	14	18
ENSG00000142684	23	28	21	25	26	22	28	32	22	14	87	17	55	59	57	53	29	47	38	39	57	18	37	20	17	35	18	46	67	66	79	79
ENSG00000142675	74	84	50	63	8	12	8	9	35	26	114	14	7	4	1	4	13	22	15	70	13	5	9	9	9	29	8	57	5	5	4	3
ENSG00000188782	2	0	2	0	0	0	0	0	0	0	0	0	0	0	0	0	0	0	0	7	0	1	0	0	0	0	0	1	0	0	0	1
ENSG00000236155	3	5	4	8	4	0	5	2	1	3	10	0	0	2	6	1	0	2	2	6	4	4	3	3	1	2	2	4	1	3	1	2
ENSG00000278572	0	0	0	0	0	4	2	0	0	0	0	0	0	0	0	0	0	0	0	0	0	0	1	0	0	0	0	1	0	0	0	0
ENSG00000130695	3334	2680	2786	2569	2270	2296	1717	2369	1056	953	3812	761	1437	1290	1377	1622	963	1264	1166	2153	3582	1604	2369	1499	1000	1693	658	3039	836	1190	844	1418
ENSG00000270733	11	10	14	12	15	10	10	13	4	0	13	6	2	2	3	3	1	5	2	11	25	11	14	6	11	9	3	11	1	8	3	3
ENSG00000142669	2884	1881	2707	2005	2897	3010	1958	3143	4882	3424	23917	3465	981	916	956	1213	665	1221	1476	1321	4080	1430	2963	1273	4158	6572	5080	12545	770	789	685	797
ENSG00000158062	860	591	829	605	471	547	417	516	525	453	2101	351	376	335	415	534	503	408	589	684	864	363	686	379	393	618	443	1428	260	393	356	482
ENSG00000169442	133	41	120	39	2467	2098	1776	2594	1041	667	6640	837	274	282	283	373	82	68	153	63	5036	1909	4001	2131	1107	1223	1516	3224	373	245	319	290
ENSG00000176092	2	3	2	6	31	33	34	55	5	7	142	25	5	15	15	15	0	2	1	2	64	34	56	39	7	28	65	185	8	21	13	20
ENSG00000225854	0	0	0	0	0	1	2	1	0	0	3	0	3	0	0	0	0	0	0	0	0	0	0	0	0	0	2	6	0	0	0	0
[illegible]	[illegible]																															

ENSG00000279179	21	36	21	21	21	26	10	27	38	21	115	23	8	8	10	13	8	5	8	36	40	29	22	22	13	35	45	87	1	16	7	31
ENSG00000160094	1004	710	894	719	1175	1239	851	1246	698	633	3036	533	520	528	448	698	226	347	300	589	1695	749	1186	657	495	893	746	1874	338	536	292	531
ENSG00000270115	13	11	13	9	30	44	31	38	6	6	30	6	0	4	3	4	1	3	9	15	58	18	33	49	5	10	9	26	0	5	2	6
ENSG00000184389	0	0	3	0	5	0	3	2	1	3	12	0	0	0	2	1	0	0	2	0	1	1	0	1	3	1	5	5	0	0	0	3
ENSG00000225313	82	57	66	52	51	49	38	67	62	55	267	44	42	29	46	41	31	26	18	72	110	52	44	52	46	58	47	145	21	62	34	57
ENSG00000134686	1794	1448	1614	1423	1374	1342	920	1416	1264	1055	4496	836	1193	1145	1189	1470	421	743	725	1050	2172	878	1549	782	1042	1683	948	2775	935	1132	829	1185
ENSG00000222112	7	10	10	10	9	6	10	5	12	7	16	3	5	6	3	2	1	1	5	13	18	10	5	8	5	11	7	22	3	1	2	7
ENSG00000233246	12	12	9	14	13	31	18	18	0	1	6	1	10	17	7	19	3	2	1	10	17	13	17	5	1	0	2	3	9	15	1	8
ENSG00000234512	0	0	0	0	0	0	0	1	0	0	0	0	0	0	0	0	0	0	0	0	0	0	0	0	0	0	0	0	0	0	0	0
ENSG00000121903	252	168	224	160	134	136	91	136	53	31	165	26	72	67	85	96	68	75	63	178	221	88	164	72	22	48	34	114	28	62	43	73
ENSG00000235907	0	3	2	3	0	1	0	0	0	2	0	0	1	0	0	0	0	0	0	0	0	0	0	0	0	0	0	0	0	0	0	0
ENSG00000121904	4	1	1	1	0	0	1	0	0	2	6	0	0	1	1	0	0	0	0	2	2	0	1	0	0	2	2	2	0	0	1	0
ENSG00000227000	0	0	0	0	0	0	1	0	0	0	0	0	0	0	0	0	0	0	0	0	0	0	0	0	0	0	0	0	0	0	0	0
ENSG00000176256	0	0	0	0	0	1	0	0	0	0	0	0	0	0	0	0	0	0	0	0	0	0	0	0	0	0	0	0	0	0	0	0
ENSG00000231163	0	0	0	0	0	0	0	0	0	0	0	0	0	0	0	0	0	0	0	0	0	0	0	0	0	0	0	0	0	0	0	0
ENSG00000142698	0	0	0	0	1	0	0	0	0	0	0	0	0	0	0	0	0	0	0	0	0	0	0	0	0	0	0	0	0	0	0	0
ENSG00000270241	0	0	0	0	0	0	0	0	0	0	0	0	0	0	0	0	0	0	0	0	0	0	0	0	0	0	0	0	0	0	0	0
ENSG00000207941	0	0	0	0	0	0	0	0	0	0	0	0	0	0	0	0	0	0	0	0	0	0	0	0	0	0	0	0	0	0	0	0
ENSG00000163866	823	552	752	629	703	767	528	787	640	500	2687	450	622	597	693	783	423	579	556	637	1453	610	1132	543	760	1166	587	1737	675	851	735	883
ENSG00000189280	0	0	2	0	0	0	0	0	1	0	1	0	7	5	3	8	0	0	0	0	1	0	0	0	0	2	0	2	0	4	0	2
ENSG00000189433	0	0	0	0	0	1	0	0	0	0	2	1	3	4	2	4	0	0	0	0	0	0	0	0	0	0	0	0	0	1	1	1
ENSG00000255811	0	0	0	0	0	0	0	0	0	0	0	0	2	0	0	3	0	0	0	0	2	0	0	0	0	0	0	0	0	0	0	0
ENSG00000188910	0	0	0	0	0	1	0	1	1	0	1	0	2	3	4	4	0	0	0	0	0	0	0	0	0	0	0	1	0	0	0	1
ENSG00000187513	1	1	0	1	0	1	0	1	3	0	0	1	3	3	0	13	0	0	0	0	1	2	0	0	0	1	0	2	1	1	0	2
ENSG00000230163	34	24	20	14	35	33	45	45	39	32	156	33	42	31	37	53	11	16	5	23	49	24	38	18	44	65	41	116	28	23	15	37
ENSG00000116544	8	7	5	8	60	76	38	84	4	4	10	1	8	15	4	22	2	2	0	1	98	63	74	43	4	5	3	6	4	8	4	12
ENSG00000241014	119	122	119	122	107	121	73	117	103	109	522	92	62	53	37	82	33	21	22	71	125	84	82	66	60	102	130	235	15	38	28	39
ENSG00000271741	16	6	10	7	12	14	12	12	12	14	36	6	11	7	4	11	9	5	8	13	17	4	9	6	18	7	9	22	2	6	11	7
ENSG00000243749	100	59	91	76	114	177	113	135	76	52	167	31	76	69	62	81	104	128	120	96	244	114	222	98	54	88	49	107	64	55	83	86
ENSG00000163867	496	347	361	310	624	653	540	711	438	345	1572	241	343	291	342	390	291	383	321	428	1539	668	993	709	344	451	379	816	243	374	341	415
ENSG00000233940	0	0	1	0	1	0	0	0	0	0	0	0	0	1	1	0	0	0	0	0	0	0	0	0	0	0	0	0	0	0	0	0
ENSG00000197056	1027	747	861	728	787	841	540	870	520	458	1636	293	682	695	714	865	712	888	734	972	1471	597	947	635	513	698	343	1272	425	638	526	747
ENSG00000228348	0	1	0	0	0	0	1	1	0	1	1	0	0	0	0	0	0	0	0	0	1	0	0	0	0	0	0	0	0	0	0	0
ENSG00000116560	13137	9430	11710	9775	12032	13177	9241	13446	14775	12315	51854	9362	8316	8921	10076	11654	4401	6239	6545	13015	24192	10732	17086	9461	13185	20402	9868	32292	8932	13313	9815	14443
ENSG00000201868	0	1	0	0	1	0	3	0	1	1	2	1	0	0	0	0	0	0	0	1	1	0	1	0	1	2	3	3	0	1	0	1
ENSG00000146463	2679	1954	2299	1871	3123	3308	2451	3571	2081	1734	7937	1345	1484	1502	1602	1955	962	1488	1323	2091	5663	2345	3811	2048	1939	2897	1912	5116	1149	1814	1503	1894
ENSG00000240374	0	0	1	4	2	3	3	2	0	6	4	0	0	0	1	1	0	1	0	1	4	2	1	5	0	6	3	4	0	1	0	1
ENSG00000201542	3	0	0	0	1	2	3	0	0	1	3	1	0	1	2	0	0	0	0	0	0	0	0	1	0	0	0	3	1	1	1	2
ENSG00000229994	13	9	10	1	6	18	4	13	10	5	29	5	10	5	13	10	4	1	2	13	19	15	6	13	5	13	12	23	0	7	8	9
ENSG00000227409	9	8	3	2	7	8	5	5	4	6	16	0	2	5	5	1	5	2	1	6	8	8	4	12	3	5	3	26	0	2	4	1
ENSG00000278702	0	0	0	0	0	0	0	0	0	0	0	0	0	0	0	0	0	0	0	0	0	0	0	0	0	0	1	0	0	0	0	0
ENSG00000142687	1352	1029	1245	1157	1302	1579	1143	1617	1149	1010	4564	801	1100	1029	1074	1371	251	291	263	726	1838	938	1258	764	760	1259	1082	2523	536	848	641	949
ENSG00000236274	5	2	5	4	3	2	1	0	3	4	9	0	2	2	1	2	1	0	0	5	3	4	2	4	2	2	3	13	0	7	5	4
ENSG00000020129	658	457	595	493	1181	1143	978	1219	829	681	3369	556	473	481	601	652	190	246	252	549	1757	852	1353	696	688	1256	693	1894	489	650	507	745
ENSG00000239636	39	30	54	28	78	81	57	78	151	152	673	127	10	6	7	15	16	29	42	30	139	64	121	78	107	138	149	347	1	8	3	8
ENSG00000116819	73	56	76	81	82	122	81	119	146	126	855	112	26	37	32	34	7	21	45	61	253	108	174	93	131	217	148	373	8	39	20	48
ENSG00000126067	4371	3048	3926	2943	3607	3426	2532	3672	2755	2143	9090	1702	3547	2940	3419	3934	8551	10133	9159	6694	10673	3968	6957	4286	3188	4668	1849	6592	4830	4496	5951	6160
ENSG00000142686	846	664	764	645	1192	1254	921	1249	1393	1178	5548	1022	655	666	713	888	280	227	284	623	2359	989	1560	878	1477	2249	1277	3392	659	775	633	937
ENSG00000092853	3947	2955	3629	2898	2739	2810	2543	3135	2443	1919	7621	1482	2054	1907	2427	2569	1539	1826	2158	3031	6810	2773	5102	2620	2830	4071	1357	7338	1850	2453	2176	2881
ENSG00000232335	13	5	11	7	11	7	6	14	5	7	26	4	6	4	4	6	2	2	1	14	15	12	13	6	5	4	3	22	2	14	2	7
ENSG00000134698	1184	838	974	824	373	372	261	470	667	606	3107	484	320	363	295	480	272	366	335	569	452	182	290	175	523	804	927	1849	169	284	136	282
ENSG00000092847	2925	2206	2409	2171	2247	2239	1560	2347	1308	1174	5408	869	1473	1317	1389	1782	680	879	891	2013	3424	1530	2318	1456	1134	1825	1283	3405	980	1439	1038	1609
ENSG00000271164	0	0	0	0	0	0	0	0	0	0	0	0	0	0	0	0	0	0	0	0	0	0	0	0	0	0	0	0	0	0	0	0
ENSG00000271914	0	0	0	0	0	0	0	0	0	0	0	0	0	0	0	0	0	0	0	0	0	0	0	0	0	0	0	0	0	0	0	0
ENSG00000126070	2265	1599	2058	1646	1633	1717	1357	1754	1435	1275	6027	868	1340	1349	1366	1643	544	707	777	1826	2852	1272	1816	1234	1205	1785	1552	3534	682	1299	884	1362
ENSG00000271554	11	11	16	7	13	13	9	16	13	9	35	2	4	9	18	17	1	1	7	8	20	9	17	16	9	11	13	25	7	7	8	17
ENSG00000232862	1	2	2	1	0	1	0	1	2	0	4	1	1	1	2	0	0	1	0	1	2	0	4	0	0	2	1	4	0	3	1	0
ENSG00000092850	11	2	13	0	2	2	1	4	4	2	6	1	3	1	3	3	6	2	2	8	6	0	3	2	1	3	1	2	4	3	0	3
ENSG00000116863	903	626	723	666	655	656	435	654	614	428	1576	346	757	642	701	899	348	480	520	603	907	419	777	361	443	625	301	989	655	656	685	771
ENSG00000171812	7	3	3	1	4	2	2	8	3	3	10	3	0	5	1	1	2	2	2	7	13	3	2	4	1	5	1	16	0	3	5	5
ENSG00000054116	1984	1243	1670	1336	736	803	501	763	783	608	3098	602	1036	847	1013	1136	1638	2157	2342	1708	1453	612	1152	551	792	1206	773	2016	1078	976	1240	1228
ENSG00000116871	3702	2432	3614	2856	3031	3070	2392	2942	3986	2963	15240	2658	2984	2696	3252	3380	922	1234	1302	2184	4267	1824	3220	1477	3483	6234	3366	10036	2466	2926	2412	2904
ENSG00000264592	0	0	0	0	0	0	0	0	0	0	0	0	0	0	0	0	0	0	0	0	0	0	0	0	0	1	0	0	0	0	0	0
ENSG00000054118	8359	5389	6520	5520	7262	7083	5488	7575	6578	5016	19212	3731	5229	5016	5241	6250	2284	3047	2872	4961	11411	4964	8331	4126	5422	8165	4141	14056	3906	4939	3875	5337
ENSG00000231849	1	3	1	1	1	2	2	2	0	1	5	1	1	0	0	2	0	0	0	2	3	3	3	0	4	3	2	13	0	0	1	1
ENSG00000214193	759	694	794	725	98	105	76	109	494	445	432	170	97	66	77	117	82	67	77	199	96	71	52	55	77	98	69	253	22	36	31	52
ENSG00000142694	263	248	301	268	146	127	79	86	121	120	278	71	36	21	22	22	38	51	43	85	207	102	130	73	28	57	51	134	5	20	13	38
ENSG00000116883	175	248	203	246	38	43	25	48	222	203	224	83	42	45	30	53	18	26	12	41	35	24	26	28	22	45	34	93	13	18	13	22
ENSG00000196182	1892	1293	1607	1334	1450	1451	1006	1434	945	777	4097	725	1088	963	975	1302	633	980	938	1261	2353	1049	1646	814	948	1709	1046	2766	916	953	845	1152
ENSG00000181817	521	340	461	361	525	434	323	478	352	249	1205	256	458	350	529	551	688	895	824	600	1082	387	895	400	352	644	267	915	770	619	775	679
ENSG00000222821	1	0	0	0	0	0	0	0	1	0	3	0	0	0	2	0	0	0	0	1	1	0	0	0	0	1	1	1	0	0	0	0
ENSG00000116885	84	44	80	52	10	12	6	6	79	63	310	37	57	55	43	59	42	52	65	51	19	14	16	10	50	63	53	160	24	63	49	69
ENSG00000201448	0	0	0	0	0	0	0	1	2	3	12	0	1	0	0	0	0	0	1	1	0	1	0	0	2	2	4	6	0	0	1	3
ENSG00000116898	1434	871	1083	959	924	863	644	920	962	720	3011	561	1623	1357	1630	1889	1649	2007	1679	1492	2219	877	1557	932	912	1445	631	1945	1962	1856	2092	2344
ENSG00000119535	0	0	1	1	0	2	1	3	0	1	3	0	2	1	3	3	0	0	0	0	4	5	0	3	0	0	2	2	0	1	1	4
ENSG00000230214	0	0	0	0	0	0	0	0	0	0	0	0	0	0	0	0	0	0	0	0	0	0	0	0	0	0	0	0	0	0	0	0
ENSG00000234481	0	0	0	0	0	0	0	0	0	0	0	0	0	0	0	0	0	1	0	0	0	0	0	0	0	0	0	0	0	0	0	0
ENSG00000163873	0	0	1	0	0	0	0	0	0	0	0	1	2	0	0	0	0	0	0	0	0	0	0	0	0	0	0	0	0	0	0	0
ENSG00000264698	0	0	0	0	0	0	0	0	0	0	0	0	0	0	0	0	0	0	0	0	0	0	0	0	0	0	0	0	0	0	0	0
ENSG00000207328	0	0	0	0	0	0	0	0	0	0	0	0	0	0	0	0	0	0	0	0	0	0	0	0	0	0	0	0	0	0	0	0
ENSG00000223944	0	0	0	0	0	0	0	0	0	0	0	0	0	0	0	0	0	0	0	0	0	0	0	0	0	0	0	0	0	0	0	0
ENSG00000230451	0	0	0	0	0	0	0	0	0	0	0	0	1	2	1	1	0	0	0	0	0	0	0	0	0	0	0	0	0	0	0	0
ENSG00000233621	55	33	57	33	99	85	59	74	24	16	87	8	60	40	60	94	39	44	50	25	149	53	107	34	17	21	15	38	57	39	67	59
ENSG00000163874	244	181	235	173	213	190	156	210	401	298	1295	243	271	278	346	363	91	96	123	165	341	142	235	154	348	433	344	761	239	311	256	380
ENSG00000163875	1902	1318	1565	1299	1792	1699	1425	1924	1066	825	3944	742	1082	962	1173	1227	1193	1541	1421	1604	4083	1607	2954	1451	1092	1554	962	2701	1198	1363	1267	1451
ENSG00000263675	34	25	18	16	19	18	6	17	8	16	47	9	4	13	15	13	9	6	7	21	16	9	11	16	5	6	18	28	1	7	8	9
ENSG00000163877	626	434	640	476	633	694	525	683	514	488	1775	345	472	453	532	588	392	572	522	481	1268	516	918	430	516	725	487	1318	443	455	490	533
ENSG00000232273	4	2	3	8	8	8	3	9	4	9	26	3	5	6	6	5	0	4	0	4	10	2	7	6	5	2	7	13	1	15	5	12
ENSG00000237749	0	0	0	0	1	3	1	0	1	0	4	0	0	1	0	0	0	0	0	1	1	0	2	0	0	0	0	2	0	0	1	0
ENSG00000163879	7	10	10	8	10	9	9	14	5	3	12	2	4	9	12	9	2	2	3	11	22	9	14	21	2	6	7	11	1	5	8	6
ENSG00000134697	2408	1833	1760	1638	2031	2030	1518	2166	1572	1048	4912	917	2169	1992	2609	2770	2220	3356	2510	2410	4745	1836	3626	1698	1341	2070	1071	3539	2444	2628	3233	3025
ENSG00000169218	1	0	0	0	0	0	0	0	0	0	0	0	0	0	0	0	0	0	0	0	0	0	0	0	0	0	0	0	0	0	0	0
ENSG00000116922	644	512	739	515	553	571	449	584	517	369	1495	256	581	509	671	675	393	563	682	766	1146	490	972	455	461	663	386	1071	704	777	875	841
ENSG00000134690	2385	1743	2253	1889	2398	2498	1645	2538	1828	1500	4768	1199	1387	1337	1546	1852	789	1055	1031	1350	3806	1475	2632	1135	1967	2898	521	3765	1225	1448	1305	1620
ENSG00000183317	1	13	3	7	4	2	1	0	1	1	2	1	8	23	9	42	1	6	0	6	2	1	1	2	1	0	1	1	6	10	3	8
ENSG00000200421	0	0	0	0	0	0	0	0	0	0	0	0	0	0	0	0	0	0	0	0	0	0	0	0	0	0	0	0	0	0	0	0
ENSG00000214141	0	0	0	0	0	0	0	0	0	0	0	0	0	0	0	0	0	0	0	0	0	0	0	0	0	0	0	0	0	0	0	0
ENSG00000227416	0	0	0	0	0	0	0	0	0	0	0	0	0	0	0	0	0	0	0	0	0	0	0	0	0	0	0	0	0	0	0	0
ENSG00000185090	212	193	249	186	385	423	325	439	7	21	89	25	602	589	611	723	90	153	179	240	689	317	539	221	16	30	14	64	494	626	458	589
ENSG00000233728	0	0	0	0	0	0	0	0	0	0	0	0	0	0	0	0	0	0	0	0	0	0	0	0	0	0	0	0	0	0	0	0
ENSG00000196449	803	551	734	623	496	509	366	492	376	287	1456	267	681	582	898	748	540	997	1119	845	1009	363	729	363	387	588	231	820	863	849	1013	910
ENSG00000197982	466	334	445	363	266	279	193	289	249	128	984	172	205	171	274	259	377	588	626	510	643	237	499	255	211	416	202	596	344	288	444	390
ENSG00000188786	849	563	771	587	566	568	373	558	459	379	2046	309	559	451	534	629	277	283	340	577	967	374	678	365	536	810	461	1315	377	583	444	586
ENSG00000235673	0	2	2	1	0	1	0	1	2	0	2	1	0	1	3	1	0	0	1	3	2	1	0	1	2	2	2	2	1	2	1	0
ENSG00000204084	967	619	867	577	1732	1673	1237	1932	887	711	3134	507	590	537	582	680	243	305	330	422	2812	1162	2081	1008	770	1022	968	2233	366	512	369	498
ENSG00000230955	0	0	0	0	0	0	0	0	0	0	0	0	0	0	0	0	0	0	0	0	0	0	0	0	0	0	0	0	0	0	0	0
ENSG00000252448	3	8	2	0	2	2	4	2	6	1	14	1	2	2	2	3	0	0	0	1	19	8	5	1	7	5	5	11	0	0	0	1
ENSG00000222282	1	0	1	2	1	3	0	2	2	0	4	2	0	1	0	0	1	0	0	1	3	0	4	1	1	0	3	1	0	0	3	0
ENSG00000277168	0	0	0	0	0	0	0	0	0	0	0	0	0	0	0	0	0	0	0	0	0	0	0	0	0	0	0	0	0	0	0	0
ENSG00000183431	3929	2697	3746	2898	3972	3909	3451	4449	3049	2319	9106	1724	3659	3183	4029	4064	3361	4811	4657	3497	9465	3434	7755	3262	2811	4212	1903	6421	4245	3843	4693	4385
ENSG00000212541	9	4	6	6	6	9	11	5	4	0	11	0	6	5	2	3	2	1	2	4	9	12	12	5	2	0	1	2	3	6	3	3
ENSG00000183386	292	196	264	191	349	344	258	337	36	31	108	23	159	177	176	211	74	128	125	208	761	305	584	216	22	30	21	50	84	138	97	122
ENSG00000183520	1448	928	1168	935	1382	1438	1202	1570	819	542	2471	454	1309	1105	1515	1452	2439	2784	2425	1830	4200	1531	2993	1683	807	1175	562	1810	1725	1581	2147	1882
ENSG00000185668	0	3	0	0	0	0	0	0	0	0	3	0	5	0	1	3	0	1	0	3	0	0	0	0	0	1	1	2	2	0	0	1
ENSG00000224592	0	0	0	1	0	0	0	1	0	0	1	0	2	0	0	0	0	0	0	0	0	0	0	1	0	0	0	1	0	1	0	2
ENSG00000223589	0	1	0	0	0	0	0	0	0	0	0	0	0	0	0	0	0	0	0	0	0	0	0	0	0	0	0	0	0	0	0	0
ENSG00000265596	0	0	0	0	0	0	0	0	0	0	0	0	0	0	0	0	0	0	0	0	0	0	0	0	0	0	0	0	0	0	0	0
ENSG00000237290	0	0	0	0	0	0	0	0	0	0	0	0	0	0	0	0	0	0	0	0	0	0	0	0	0	0	0	0	0	0	0	0
ENSG00000275350	0	0	0	0	0	0	0	0	0	0	4	0	0	0	0	0	0	0	0	0	0	0	0	0	0	0	0	0	0	0	0	0
ENSG00000200796	0	0	0	0	0	0	0	0	0	0	0	0	0	0	0	0	0	0	0	0	0	0	0	0	0	0	0	0	0	0	0	0
ENSG00000238063	0	0	0	0	0	0	0	0	0	0	0	0	0	1	0	0	0	0	0	0	0	0	0	0	0	0	0	0	0	0	0	0
ENSG00000215895	0	0	0	0	0	0	0	0	1	0	2	0	0	0	0	0	0	0	0	0	0	0	0	0	0	1	0	1	0	0	0	0
ENSG00000116954	436	300	396	299	372	414	287	443	149	131	735	121	189	199	181	247	141	219	239	338	691	267	529	265	206	280	304	670	158	261	167	235
ENSG00000273637	0	0	0	0	2	0	0	0	0	0	0	0	0	0	0	0	0	0	0	0	0	0	0	0	0	0	0	0	0	0	0	0
ENSG00000228436	19	3	12	14	3	4	3	8	5	3	24	8	8	5	2	7	4	7	6	18	6	2	6	7	3	3	6	18	3	2	3	8
ENSG00000214114	1174	706	847	700	795	734	542	757	652	513	1948	378	885	767	865	951	1232	1683	1287	1305	1879	732	1328	853	651	980	383	1469	1024	920	1119	1136
ENSG00000274944	1	2	7	3	1	1	0	2	0	1	13	2	0	0	3	0	1	3	1	5	9	1	1	4	0	2	2	5	4	1	1	5
ENSG00000131233	1	0	4	2	0	0	0	0	0	2	10	1	0	2	2	1	0	0	0	6	6	0	1	1	3	0	1	14	0	5	4	0
ENSG00000158315	9	6	7	0	2	6	3	3	4	8	19	6	0	3	0	1	0	3	3	11	4	3	2	0	4	2	12	16	1	2	3	1
ENSG00000199963	0	0	0	0	0	0	0	0	0	0	0	1	0	0	0	0	0	0	0	0	0	0	0	0	0	0	0	0	0	0	0	0
ENSG00000207466	0	0	0	0	0	0	0	0	0	0	0	0	0	0	0	0	0	0	0	0	0	0	0	0	0	0	0	1	0	0	0	0
ENSG00000235549	0	0	0	0	0	0	0	0	0	0	0	0	0	0	0	0	0	0	0	0	0	0	0	0	0	0	0	0	0	0	0	0
ENSG00000174574	1887	1340	1668	1389	1402	1421	1162	1423	1005	848	4873	648	1538	1402	1873	1770	1327	1916	1799	1564	3496	1267	2478	1076	1157	1778	1174	2860	2085	2109	2279	2449
ENSG00000168653	3292	2045	2477	2044	2622	2723	1828	2915	1287	875	3576	701	2379	2151	2447	2937	3488	3772	4126	4138	7889	3126	5667	4349	1162	1667	769	2260	3464	2963	3639	3878
ENSG00000127603	23041	14117	25867	14563	15234	16256	12021	17010	11849	9879	57810	7617	12994	10748	12059	13367	4747	4122	7061	10820	24110	11001	16644	9821	10242	15079	14474	36705	5028	8395	7043	9747
ENSG00000206654	1	1	3	4	0	1	0	0	0	0	2	2	1	1	1	1	1	0	0	2	3	0	0	3	0	1	2	8	0	2	5	0
ENSG00000270040	6	3	4	4	8	8	12	8	2	5	6	2	1	2	3	0	1	2	1	1	20	6	7	9	3	2	2	24	1	1	5	5
ENSG00000226438	2	1	1	5	6	2	2	7	1	2	5	0	2	5	5	3	0	1	0	6	8	10	4	7	1	1	5	7	3	5	3	6
ENSG00000217897	5	5	2	1	4	0	6	12	3	1	9	0	2	1	3	2	0	0	1	10	11	12	6	7	7	2	5	11	0	4	3	5
ENSG00000183682	25	31	22	34	17	25	6	16	37	44	129	19	16	21	16	13	3	12	4	26	29	9	20	21	13	27	32	69	10	17	7	20
ENSG00000237624	6	4	5	11	0	0	0	0	1	8	6	4	6	4	2	2	0	2	0	7	1	0	0	0	2	4	3	12	1	3	0	3
ENSG00000182109	205	225	216	222	140	173	147	226	254	211	948	138	62	85	94	113	74	55	48	217	246	137	164	157	105	151	253	434	29	103	57	124
ENSG00000243970	110	84	98	127	67	55	53	73	53	63	306	45	39	26	38	31	30	30	26	91	80	38	55	58	49	73	79	147	15	36	29	38
ENSG00000090621	7667	5809	6334	6087	5572	5701	3987	5821	5374	4063	16358	3270	6025	5294	5802	6745	2154	3057	2904	5040	8360	3592	5780	3147	4005	6634	2698	9226	3895	5103	3832	5344
ENSG00000228060	62	36	60	44	32	40	29	36	34	23	96	12	25	19	27	20	14	19	14	53	46	30	29	40	13	36	18	63	1	23	15	29
ENSG00000201457	21	24	30	14	22	28	22	25	12	19	62	16	15	20	20	10	3	3	7	33	40	12	19	16	14	22	16	42	9	24	11	21
ENSG00000163909	75	118	88	126	27	31	24	26	2	1	1	1	37	90	44	71	11	70	29	107	18	5	22	7	0	0	0	1	20	41	7	46
ENSG00000225903	4	0	1	0	0	0	0	1	0	0	0	0	0	1	0	0	0	0	0	0	1	1	0	0	0	0	0	0	0	0	0	0
ENSG00000116981	0	0	0	0	0	0	0	0	0	0	0	0	0	0	0	0	0	0	0	0	0	0	0	0	0	0	0	0	0	0	0	0
ENSG00000116983	33	49	26	52	0	0	0	0	0	0	0	1	14	29	14	39	0	8	3	3	0	0	0	0	1	1	1	0	0	2	5	10
ENSG00000084072	2372	1653	2040	1645	1179	1291	857	1196	1107	834	3103	645	1227	1133	1383	1553	1617	1960	1635	1851	2449	1063	1932	941	862	1318	615	2046	1402	1256	1570	1520
ENSG00000225333	0	0	0	0	0	0	0	0	0	0	0	0	0	0	0	0	0	0	0	0	0	0	0	0	0	0	0	0	0	0	0	0
ENSG00000252413	0	0	0	0	0	0	0	0	0	0	0	0	0	0	0	0	0	0	0	0	0	0	0	0	0	0	0	0	0	0	0	0
ENSG00000116985	126	89	101	89	52	72	46	60	394	349	1748	292	229	272	219	343	28	37	35	117	129	72	74	47	259	406	416	1063	133	239	127	247
ENSG00000198754	5	5	8	4	0	0	1	0	41	48	105	19	24	21	27	37	2	6	3	13	0	0	0	0	21	50	24	79	8	19	16	29
ENSG00000261798	14	17	16	34	0	0	0	0	24	31	100	16	19	32	15	39	10	13	8	31	0	0	0	0	9	24	11	40	13	27	14	24
ENSG00000229213	0	0	0	0	0	0	1	0	0	0	1	0	0	0	0	0	0	0	1	0	0	0	0	0	0	0	0	0	0	0	0	0
ENSG00000043514	1405	904	1126	858	832	821	658	983	844	577	2021	445	1107	914	1229	1277	948	1182	1185	1081	1760	729	1146	704	556	746	360	1077	950	1113	1298	1336
ENSG00000202222	4	5	4	5	0	4	3	3	4	5	11	0	5	4	5	2	2	2	0	6	12	7	5	3	5	9	1	15	0	6	3	3
ENSG00000116990	155	170	134	158	48	72	29	54	2	3	2	2	17	24	15	23	30	105	36	111	54	32	37	13	0	4	2	5	13	25	10	17
ENSG00000236546	1	1	4	2	1	1	0	0	2	0	3	0	0	0	0	1	4	2	19	6	2	1	2	1	1	1	0	1	4	0	2	0
ENSG00000207356	0	0	0	1	0	2	0	0	1	0	0	0	0	0	0	0	0	0	0	1	0	0	0	0	0	0	1	0	0	0	0	0
ENSG00000168389	450	265	372	288	276	260	161	288	34	26	74	22	3	0	0	1	133	206	183	390	531	219	270	202	35	72	14	91	0	1	1	0
ENSG00000228477	14	21	15	18	12	10	8	15	2	2	2	1	0	0	2	4	3	6	6	35	19	20	12	12	1	3	0	1	0	0	0	1
ENSG00000131236	6806	4120	5797	4179	5335	5666	4262	6014	6185	4631	25191	4014	5362	4325	5557	5870	3207	4464	5182	4444	10658	4316	8126	3986	6425	9683	5978	17090	4804	4803	5190	5398
ENSG00000131238	3000	2105	2495	2012	2931	3135	2327	3404	2052	1598	6458	1229	3222	2808	3186	3768	3204	4780	4171	2938	5618	2092	4111	1901	1973	2968	1811	4943	3302	3456	3893	3752
ENSG00000226745	0	0	0	0	0	0	0	0	0	0	1	0	0	1	0	0	0	0	0	0	0	0	0	0	0	0	1	2	0	0	0	0
ENSG00000117000	2119	1838	1731	1585	1442	1510	1030	1588	1118	933	2978	619	981	965	1033	1213	467	532	523	732	2007	830	1344	696	764	1166	700	2053	504	733	551	773
ENSG00000207508	2	1	3	4	6	5	2	3	6	4	9	2	2	2	1	3	0	0	0	4	5	7	3	1	4	6	2	12	0	0	1	4
ENSG00000188800	1	0	0	0	0	0	2	1	0	1	3	2	0	1	0	0	1	0	0	0	0	0	1	3	0	5	3	2	0	0	0	2
ENSG00000259943	237	204	199	183	84	74	70	84	184	114	616	118	169	153	137	242	184	228	223	182	157	68	119	95	156	152	113	339	113	122	92	154
ENSG00000084073	1226	740	1050	708	1383	1384	1130	1519	1049	825	4277	644	1159	1017	1656	1341	1638	2213	2419	1891	4813	1879	3831	1825	1363	2057	1076	3074	2235	2031	2899	2415
ENSG00000231296	6	4	2	5	4	1	6	4	8	5	29	2	4	9	12	6	2	1	4	11	11	15	5	14	7	13	3	11	5	6	5	6
ENSG00000049089	355	311	404	383	649	709	475	726	363	256	1278	225	524	404	542	669	99	175	161	308	733	319	494	288	145	175	256	518	307	327	287	453
ENSG00000227311	0	0	0	0	0	0	0	0	0	0	0	0	0	0	1	0	0	0	0	0	0	0	0	0	0	0	0	0	0	0	0	0
ENSG00000084070	2360	1621	1979	1746	2097	2275	1659	2561	2141	1681	6310	1219	2609	2212	2679	2897	470	684	717	949	2481	1097	1992	954	1784	2758	1571	4289	1419	1752	1619	1956
ENSG00000213172	0	0	0	0	0	0	0	0	0	0	0	0	0	0	0	0	0	0	0	0	0	0	0	0	0	0	0	0	0	0	0	0
ENSG00000187801	346	247	334	230	127	133	100	133	43	39	203	28	127	106	110	113	145	170	180	278	198	85	164	98	46	65	41	151	50	70	58	82
ENSG00000260920	273	193	261	157	70	89	56	79	41	33	141	26	110	84	71	117	85	122	111	203	119	37	61	60	40	47	34	89	31	57	42	67
ENSG00000279667	43	34	39	32	6	3	2	7	5	5	10	4	14	14	13	14	11	10	11	37	12	3	2	3	2	4	8	21	8	10	4	9
ENSG00000187815	53	25	59	40	338	359	265	415	86	81	420	84	111	123	135	144	9	14	21	35	744	291	574	299	98	145	82	267	71	97	111	129
ENSG00000238287	25	21	28	17	30	52	42	40	21	18	115	13	14	26	20	39	16	17	20	23	71	32	39	29	22	22	25	35	12	21	19	19
ENSG00000164002	280	148	236	169	314	331	237	322	300	260	1116	247	186	241	273	292	218	240	325	344	662	282	529	294	334	510	186	809	205	287	273	327
ENSG00000227278	4	1	3	6	1	1	0	4	1	0	3	1	1	0	0	2	2	0	0	3	3	7	2	2	2	0	2	7	1	2	0	3
ENSG00000238186	22	10	23	11	12	14	8	12	16	15	62	11	10	15	9	19	4	16	9	20	32	20	30	14	27	20	14	55	3	4	11	18
ENSG00000117010	195	118	192	125	97	121	98	124	91	69	293	75	76	88	94	122	126	152	185	198	295	122	201	116	99	172	56	248	103	126	133	151
ENSG00000236505	0	0	0	0	0	0	0	0	0	0	0	0	1	1	0	0	0	0	1	0	0	0	0	0	0	0	0	1	0	0	0	0
ENSG00000233910	1	1	1	1	0	0	0	0	0	0	0	0	0	0	0	0	0	0	0	0	0	0	0	0	0	0	0	0	0	0	0	1
ENSG00000117016	2365	1828	1809	1897	1078	1028	663	1027	86	79	328	80	334	299	198	359	404	604	456	914	1543	564	958	587	80	151	83	325	108	167	104	212
ENSG00000237899	12	3	12	8	4	1	8	5	3	7	27	1	4	7	5	7	1	0	8	15	9	1	0	11	1	6	4	7	6	5	1	4
ENSG00000272145	105	98	110	84	64	69	60	104	120	109	342	58	86	87	77	89	26	29	25	71	94	53	63	57	76	74	43	142	44	44	35	71
ENSG00000066136	1217	946	1209	997	1579	1573	1153	1709	1229	984	4503	817	913	830	1019	1085	1158	1547	1366	1538	3776	1466	2587	1343	1054	1614	898	2800	952	1094	1150	1335
ENSG00000198974	1	0	0	0	0	3	0	3	0	0	0	0	0	0	1	0	0	0	0	0	1	0	1	1	2	0	2	4	1	0	1	1
ENSG00000207962	0	0	0	0	0	0	1	0	1	0	10	0	0	1	1	1	1	0	0	0	1	8	0	3	0	0	3	1	0	2	3	2
ENSG00000117013	22	16	15	17	13	13	4	13	8	8	19	4	3	7	3	8	7	8	6	37	34	13	13	7	4	15	4	13	2	2	1	2
ENSG00000264582	0	0	1	2	0	1	0	0	0	0	0	0	0	0	0	2	0	0	0	0	0	0	0	0	0	0	0	0	0	0	0	0
ENSG00000179862	2	6	2	7	0	0	0	0	9	12	33	5	4	8	7	3	0	0	1	0	0	0	0	0	1	18	13	16	2	4	1	4
ENSG00000229528	0	0	0	0	0	0	0	0	0	0	0	0	0	0	0	0	0	0	0	0	0	0	0	0	0	0	0	0	0	0	0	0
ENSG00000228940	0	0	0	0	0	0	0	0	0	0	1	0	0	0	0	0	0	0	0	0	0	0	0	0	0	0	0	0	0	0	0	0
ENSG00000233118	0	0	0	0	0	0	0	0	0	0	0	0	0	0	0	0	0	0	0	0	0	0	0	0	0	0	0	0	0	0	0	0
ENSG00000171793	4009	3097	3757	3185	3453	3520	2769	3883	2574	1866	8940	1572	2492	2274	2708	2952	1346	2304	2378	3685	6418	2701	5067	2572	2845	4585	1620	6774	2059	2695	2173	2965
ENSG00000281207	178	144	170	143	265	319	262	334	115	86	415	63	226	199	248	220	41	29	38	190	534	287	315	302	102	180	83	368	80	283	180	317
ENSG00000171790	66	70	79	75	118	151	100	185	47	28	215	30	132	96	139	134	12	14	16	75	268	125	126	142	52	72	42	179	44	159	103	146
ENSG00000010803	1915	1593	1719	1637	2121	1968	1542	2132	711	642	1873	448	551	569	546	671	546	998	804	1430	3201	1493	2233	1421	519	888	311	1416	300	474	294	484
ENSG00000215893	9	12	14	8	9	13	11	11	13	0	10	2	3	6	1	7	3	4	3	16	28	11	16	15	2	2	2	14	0	5	5	0
ENSG00000235358	4	2	4	2	5	4	3	7	0	0	1	0	3	0	0	1	1	3	1	8	6	7	4	5	1	1	0	0	0	0	1	1
ENSG00000213987	0	0	1	0	0	0	1	0	0	1	1	0	1	0	0	0	0	0	0	0	3	0	0	1	0	0	1	0	0	0	0	1
ENSG00000204060	153	188	162	177	252	248	181	285	10	13	34	8	303	278	323	359	25	67	67	96	316	148	247	122	8	19	10	20	387	463	272	527
ENSG00000229901	3	5	5	13	6	11	6	7	2	0	0	0	8	10	13	7	2	0	5	11	12	6	6	6	1	0	0	0	2	11	9	12
ENSG00000127129	0	0	0	0	0	0	0	0	0	0	16	2	3	3	4	16	0	0	0	0	0	0	0	0	0	0	2	9	4	7	4	1
ENSG00000127124	1028	907	1180	922	292	322	259	345	395	369	4301	616	299	304	605	460	761	1067	1122	1200	740	384	629	321	394	865	608	3540	211	227	429	282
ENSG00000230881	2	1	2	1	0	0	1	1	1	1	21	1	0	0	0	1	1	1	1	2	1	0	1	1	2	2	1	30	0	0	1	1
ENSG00000230638	3	12	7	8	1	7	1	9	3	7	88	6	6	7	7	13	4	6	8	11	11	9	3	2	6	33	12	110	0	6	6	9
ENSG00000227538	0	0	0	0	0	0	0	2	0	0	0	0	0	0	0	0	0	0	0	0	0	0	0	0	0	0	0	0	0	0	0	0
ENSG00000228776	0	0	0	0	0	0	0	0	0	0	1	0	0	0	0	0	0	0	0	0	0	0	0	0	0	0	0	0	0	0	0	0
ENSG00000044012	0	0	0	0	0	0	0	0	0	0	0	0	0	0	0	0	0	0	0	0	0	0	0	0	0	0	0	0	0	0	0	0
ENSG00000197273	0	0	0	0	0	0	1	1	0	0	0	0	0	0	0	0	0	0	0	0	0	0	0	0	0	0	0	0	0	0	0	0
ENSG00000198815	3310	2623	2758	2550	2681	2742	2105	2862	1682	1428	6442	1034	1734	1475	1690	1944	649	1035	874	1950	4459	1940	3034	1789	1655	2528	1437	4170	1150	1620	1200	1751
ENSG00000227527	15	10	5	11	4	7	2	2	1	3	20	3	1	6	1	3	6	4	2	6	11	3	4	3	1	4	3	8	3	3	7	3
ENSG00000177181	1059	1016	845	1034	686	798	493	803	452	399	1264	283	166	273	99	277	335	654	431	795	569	240	386	222	273	418	290	768	107	197	83	220
ENSG00000235002	7	3	5	3	5	5	3	4	0	1	8	0	0	1	1	2	0	3	1	6	6	1	1	4	1	0	0	12	0	2	0	0
ENSG00000066185	18	20	18	22	20	28	16	24	0	13	22	3	30	9	9	21	4	4	4	14	35	16	16	16	9	3	11	10	9	13	7	12
ENSG00000127125	618	398	478	450	765	714	524	852	442	328	1798	338	520	519	563	637	681	793	744	637	1735	675	1191	825	370	499	429	1095	522	526	616	646
ENSG00000186409	65	46	47	33	102	108	70	109	38	46	190	29	52	68	70	79	14	26	31	50	103	53	76	56	32	39	54	112	37	46	57	55
ENSG00000230116	1	0	0	0	0	0	0	1	0	0	1	0	0	0	0	0	0	0	0	2	0	0	1	2	0	1	1	1	0	0	0	1
ENSG00000236876	0	0	0	3	2	0	0	0	2	0	0	0	1	1	1	1	0	0	0	0	1	2	0	0	1	1	0	1	0	0	1	0
ENSG00000200254	0	1	1	1	0	0	0	0	0	0	0	0	0	0	0	2	0	0	0	1	0	0	0	0	0	0	0	1	0	1	0	0
ENSG00000236180	0	0	1	0	0	0	0	0	0	0	1	1	1	1	1	2	0	0	0	1	0	0	0	0	0	0	2	0	0	2	0	0
ENSG00000171960	2034	1304	1567	1350	1199	1102	895	1229	1010	734	2537	580	1346	1159	1454	1432	2796	3319	2999	2572	3084	1146	2006	1286	977	1373	428	1744	1820	1490	1951	1920
ENSG00000234917	14	17	29	4	13	19	14	22	11	7	37	7	10	9	8	9	10	12	19	14	53	23	25	19	12	10	5	23	3	14	11	13
ENSG00000065978	26777	18246	20621	20078	28488	28283	20485	27419	15291	10375	32952	7557	27074	24179	27315	30955	6158	11703	10776	17617	41656	17498	32008	13936	13382	21354	5102	23688	24150	25730	22099	25487
ENSG00000164007	1	1	0	0	1	3	2	3	0	1	3	0	0	0	0	1	0	0	1	0	0	2	1	2	0	1	4	4	0	0	2	0
ENSG00000117385	1856	1562	1600	1543	1101	1191	783	1218	793	706	2863	601	483	493	503	538	923	910	785	1192	1811	825	1206	797	670	1100	650	1883	347	419	388	555
ENSG00000164008	54	40	54	40	36	47	29	53	67	22	186	43	31	47	42	48	15	18	22	46	93	49	71	38	35	51	44	128	22	29	36	26
ENSG00000283580	39	19	20	36	15	15	2	13	14	11	22	7	10	11	8	16	4	7	9	27	21	15	29	7	4	7	11	19	7	10	13	13
ENSG00000228452	53	32	58	49	44	48	26	49	59	50	249	45	51	35	41	35	12	22	22	41	95	49	80	44	45	86	72	183	30	27	27	34
ENSG00000274386	76	87	71	69	24	20	23	32	14	22	77	10	72	60	41	67	21	28	22	63	50	20	28	21	10	23	28	46	17	36	18	40
ENSG00000177868	564	342	389	389	284	260	197	299	278	183	891	156	163	178	201	221	586	680	635	552	659	240	456	272	231	325	233	624	237	209	241	283
ENSG00000164010	240	181	265	227	143	132	80	151	297	245	1151	210	154	151	157	184	105	127	166	274	200	101	145	97	235	352	299	754	92	156	82	179
ENSG00000228192	25	18	33	27	22	19	15	28	16	18	77	16	73	58	64	75	33	25	30	34	51	23	40	20	13	21	15	37	49	49	56	69
ENSG00000164011	367	267	388	274	221	227	189	259	249	219	911	185	194	188	229	267	185	237	214	294	385	160	235	143	182	287	224	528	143	179	145	231
ENSG00000233708	0	1	0	0	0	1	0	2	0	0	0	0	0	0	0	0	0	0	0	3	1	0	0	0	0	0	0	1	0	0	0	0
ENSG00000237090	0	0	0	0	0	0	1	0	0	0	0	0	0	0	0	0	0	0	0	0	0	0	0	0	0	0	0	0	0	0	0	0
ENSG00000225099	0	0	0	0	0	1	0	1	0	0	0	0	0	0	0	0	0	0	0	0	0	0	0	0	0	0	0	0	0	0	0	0
ENSG00000117394	2145	1621	1950	1473	1763	1834	1368	1957	5512	4320	16605	3757	1303	1169	1406	1642	372	506	512	779	1858	791	1338	649	3091	5078	2833	9465	862	1095	1011	1173
ENSG00000227533	58	48	57	51	8	7	7	18	39	22	39	11	3	2	3	8	11	7	3	9	2	4	0	0	9	10	2	22	0	1	2	1
ENSG00000207256	2	0	3	1	1	0	0	0	0	0	0	0	0	0	0	0	0	0	0	0	1	0	0	0	0	0	1	0	0	0	0	0
ENSG00000283136	0	0	0	0	0	0	0	0	0	0	0	0	0	0	0	0	0	0	0	0	0	0	0	0	0	0	0	0	0	0	0	0
ENSG00000277513	0	0	0	0	0	0	0	0	0	0	0	0	0	0	0	0	0	0	0	0	0	0	0	0	0	0	0	0	0	0	0	0
ENSG00000186973	0	0	0	0	0	0	0	0	0	0	4	0	1	2	1	1	0	0	0	0	0	0	0	0	0	0	1	6	0	3	2	3
ENSG00000117395	2159	1594	1900	1955	1062	1096	859	1170	1725	1184	4636	964	2790	2354	2998	3100	3002	4504	3499	3051	2618	1083	2039	957	1550	2387	866	3010	3266	3166	3821	3552
ENSG00000243710	6	3	4	5	5	8	1	3	2	4	71	3	19	26	15	31	7	3	8	11	6	2	5	6	2	10	71	43	16	33	27	32
ENSG00000179178	0	0	0	0	0	0	0	0	0	0	0	0	0	1	0	0	0	0	0	0	0	0	0	0	0	0	0	0	0	0	0	0
ENSG00000253313	1	0	0	1	1	0	0	0	0	1	0	0	0	0	0	0	0	0	0	0	0	0	0	0	0	0	0	1	0	0	0	0
ENSG00000066056	1	1	0	2	92	100	88	103	20	16	136	20	2	4	1	0	0	1	0	1	133	47	86	49	15	13	21	32	1	0	2	0
ENSG00000117400	2	7	7	9	241	251	168	255	3	9	32	3	9	7	5	14	0	0	3	6	182	98	150	90	5	8	5	20	1	12	6	9
ENSG00000234694	17	9	12	12	9	17	8	12	13	14	66	5	13	13	13	24	4	5	10	2	14	11	13	11	15	10	14	37	7	16	9	12
ENSG00000117399	2775	2064	2725	2263	2292	2161	1305	2154	2079	1722	5529	1284	2195	2037	2519	2769	819	1254	1448	1906	2838	1092	2294	1000	2281	3854	613	4137	2095	2464	2083	2547
ENSG00000066322	979	642	802	700	982	1019	664	1004	1023	828	3272	695	891	797	1014	1049	894	1142	966	880	1906	807	1343	614	1020	1611	667	2200	907	1063	1045	1089
ENSG00000159479	529	424	442	422	576	623	436	698	518	408	1943	362	574	555	654	779	638	937	767	620	1387	473	1131	540	538	852	480	1336	750	715	894	808
ENSG00000229431	42	20	23	21	21	18	11	15	19	13	62	12	12	9	12	28	13	14	14	26	32	14	18	13	25	22	20	69	5	15	8	16
ENSG00000198198	1070	1119	1264	1133	1413	1853	1278	1910	1463	1280	6877	954	913	920	1065	1279	223	267	262	868	1868	1157	1467	1030	1038	2015	1641	4381	402	1058	623	1259
ENSG00000229372	0	0	0	1	11	11	9	21	0	0	0	0	0	0	0	0	0	0	0	0	10	4	10	9	0	1	1	9	0	0	1	1

ENSG00000274975	0	0	0	0	0	0	0	0	0	0	0	0	0	0	0	0	0	0	0	0	0	0	0	0	0	0	0	0	0	0	0	0
ENSG00000178922	99	126	143	117	517	529	451	543	101	89	433	76	125	74	136	151	16	26	32	93	784	440	577	363	72	108	104	228	66	130	62	153
ENSG00000229348	13	15	14	18	18	12	12	18	14	4	43	8	14	13	19	34	4	4	3	10	8	5	18	12	6	13	8	23	10	21	5	31
ENSG00000142949	441	1479	436	1623	3830	4438	2583	4608	96	164	412	103	4037	3691	3635	4655	17	162	33	309	2874	1426	2021	1221	23	48	139	278	1348	2032	1372	2221
ENSG00000066135	1915	1488	1585	1486	2240	2406	1665	2446	1041	906	5040	687	1411	1230	1251	1593	403	526	486	931	3613	1566	2723	1314	969	1528	1437	3122	854	1003	831	1152
ENSG00000236200	89	91	106	92	90	107	62	101	72	74	250	54	61	40	55	66	57	57	58	109	167	67	133	67	46	82	56	167	33	67	34	55
ENSG00000126091	610	506	482	490	387	364	255	424	416	370	1418	249	262	221	246	297	121	211	164	381	431	234	302	179	317	581	287	967	96	192	99	223
ENSG00000229444	7	7	10	10	3	5	5	11	4	4	49	9	2	6	3	1	1	0	1	8	15	7	1	15	10	13	6	24	2	5	8	7
ENSG00000283414	4	3	2	3	4	2	2	5	1	0	4	0	3	1	1	2	0	0	0	1	6	0	3	2	5	0	1	4	0	3	0	0
ENSG00000233674	10	13	19	6	8	7	1	9	12	16	31	3	2	2	3	10	2	5	5	15	15	11	8	4	8	12	6	30	1	10	9	3
ENSG00000283477	5	2	3	3	2	3	4	0	3	1	7	2	1	2	0	0	0	1	0	2	5	1	1	6	0	9	1	4	0	0	0	0
ENSG00000224751	11	6	15	7	18	5	7	8	6	16	40	9	2	3	6	4	7	3	3	14	6	7	7	11	15	15	5	52	1	10	5	6
ENSG00000117407	27	28	28	32	19	25	17	20	24	20	83	4	8	15	13	17	6	3	7	26	30	17	14	8	12	25	8	41	2	21	11	20
ENSG00000237950	39	23	27	28	16	16	8	19	44	36	180	20	46	28	40	38	13	12	9	36	16	9	10	5	45	33	34	68	22	44	24	40
ENSG00000117408	996	823	998	875	657	707	447	744	567	421	2691	461	467	424	560	615	405	536	508	950	1151	484	842	474	549	1006	674	1596	404	622	447	771
ENSG00000132768	974	677	869	701	717	679	550	734	871	672	3072	598	1224	942	1143	1204	584	824	757	1035	1424	583	1084	575	826	1488	613	1948	1306	1335	1267	1367
ENSG00000117410	1792	1243	1663	1324	1455	1521	1039	1523	1117	866	4232	824	969	816	1133	1163	1738	3044	2795	2470	3535	1448	2717	1150	1271	1945	989	3104	1849	1611	2102	1697
ENSG00000117411	706	575	557	542	947	962	568	864	717	546	1869	435	978	915	805	1104	471	760	444	741	1444	597	967	484	595	937	235	1241	769	750	606	786
ENSG00000159214	81	89	105	76	74	85	69	98	9	10	110	13	107	71	60	87	37	55	48	82	131	55	85	63	20	34	37	54	46	38	42	60
ENSG00000196517	101	102	50	105	5	4	5	5	0	1	2	0	86	64	52	95	19	66	13	100	5	1	6	3	0	1	0	1	8	24	24	18
ENSG00000230615	6	6	4	9	39	34	29	41	0	0	7	2	6	11	6	6	3	6	0	5	67	36	64	27	2	3	1	5	0	3	3	2
ENSG00000227163	0	0	0	0	0	0	0	2	0	0	2	0	0	0	0	0	0	0	0	0	1	1	0	0	0	0	0	0	0	0	0	0
ENSG00000271647	0	0	0	0	0	0	0	0	0	0	0	0	0	0	0	0	0	0	0	0	0	1	1	0	0	0	0	0	0	0	0	0
ENSG00000239560	0	0	0	0	0	0	0	0	0	0	0	0	0	0	0	0	0	0	0	0	0	0	0	0	0	0	0	0	0	0	0	0
ENSG00000171872	0	0	0	0	5	1	0	3	0	0	2	0	0	0	0	0	0	0	0	0	0	0	1	5	0	0	0	0	0	1	0	0
ENSG00000233514	0	1	0	0	1	0	2	0	0	0	4	1	0	0	1	2	1	0	0	1	0	0	0	0	0	1	2	1	1	0	0	2
ENSG00000283039	0	0	0	0	0	0	0	0	0	0	1	0	0	0	0	0	0	0	0	0	0	0	0	0	0	0	0	0	0	0	0	0
ENSG00000226804	0	0	0	0	0	0	0	0	0	0	0	0	0	0	0	0	1	0	0	0	0	0	0	0	0	1	0	0	0	0	0	0
ENSG00000200171	0	0	0	0	0	0	0	0	0	0	0	0	0	0	0	0	0	0	0	0	0	0	0	0	0	0	0	0	0	0	0	0
ENSG00000238037	0	0	0	0	0	0	0	0	0	0	0	0	0	0	0	0	0	0	0	0	0	0	0	0	0	0	0	0	0	0	0	0
ENSG00000227994	0	0	0	0	0	0	0	0	0	0	0	0	0	0	0	0	0	0	0	0	0	0	0	0	0	0	0	0	0	0	0	0
ENSG00000271329	3	2	0	0	1	0	0	0	0	0	0	0	0	0	1	1	0	3	0	1	2	0	1	0	0	0	0	0	0	0	0	0
ENSG00000178028	1178	847	1117	942	1480	1646	1087	1678	859	631	3072	519	370	441	504	580	587	560	581	1079	2349	1105	1597	1077	557	861	697	1687	387	619	549	781
ENSG00000117419	1252	1092	1089	1070	938	946	701	1067	986	824	2950	617	1022	973	1053	1272	604	1024	813	1060	1468	645	1208	568	783	1255	635	2130	847	1025	957	1190
ENSG00000233602	11	9	22	15	23	18	10	21	19	14	61	12	11	10	15	15	2	6	4	23	20	15	17	11	16	27	12	40	8	10	11	18
ENSG00000199385	0	0	0	0	0	0	0	0	0	0	0	0	0	0	0	0	0	0	0	0	0	0	0	0	0	0	0	0	0	0	0	0
ENSG00000187147	2223	1668	1807	1862	2567	2581	1842	2573	1329	1114	5332	1011	1778	1594	1565	1960	631	1033	894	1467	4026	1686	2963	1477	1190	1974	1190	3279	1382	1651	1333	1743
ENSG00000263381	0	0	0	0	5	3	0	3	0	0	0	0	0	0	0	0	0	0	0	0	2	2	4	3	0	0	0	0	0	0	0	0
ENSG00000126106	185	172	155	136	154	186	98	172	72	46	252	46	53	72	48	94	119	197	151	137	229	116	152	85	52	72	44	138	33	47	36	50
ENSG00000198520	1566	1160	1469	1291	2724	2825	2107	2977	6	7	12	2	9	6	10	12	921	1221	1143	1108	4698	1935	3701	1862	1	1	6	10	1	2	0	8
ENSG00000199377	5	1	3	7	2	2	3	5	1	1	3	1	1	0	3	0	0	2	6	4	9	3	4	7	1	1	1	3	1	0	1	2
ENSG00000200169	4	3	3	1	8	2	3	3	6	1	7	2	1	1	4	1	5	5	4	21	9	3	3	10	2	4	1	18	2	1	4	4
ENSG00000142945	4443	3313	4076	3511	3249	3257	2505	3347	2582	1983	6798	1711	2198	2075	2532	2818	1982	2858	2974	2462	6099	2243	4752	2029	2543	3868	811	5902	1766	2116	2347	2422
ENSG00000225721	46	32	54	27	29	50	26	46	37	16	110	21	17	10	15	17	20	17	36	36	64	43	35	32	17	36	17	83	8	14	9	32
ENSG00000142937	19781	14219	13975	13800	11425	11827	8241	12963	22720	16875	69017	13334	18393	17739	19084	23491	18971	28220	21121	14244	21503	8406	17130	7806	18526	26237	13944	40006	21282	19175	24781	22559
ENSG00000264294	0	0	0	0	0	0	0	0	0	0	0	0	0	0	0	0	0	0	0	0	0	0	0	0	0	0	0	0	0	0	0	0
ENSG00000200913	28	21	18	31	23	24	11	31	15	14	99	13	14	13	15	11	16	27	17	21	26	18	17	25	12	26	12	58	11	14	13	29
ENSG00000202031	0	7	1	5	11	6	3	4	3	3	12	2	3	3	0	4	4	5	9	8	7	6	2	3	2	2	2	6	2	3	5	2
ENSG00000281859	0	0	0	0	0	0	0	0	0	0	0	0	0	0	0	0	0	0	0	0	0	0	0	0	0	0	0	0	0	0	0	0
ENSG00000207421	0	0	0	0	0	0	0	0	0	0	0	0	0	0	0	0	0	0	0	0	0	0	0	0	0	0	0	0	0	0	0	0
ENSG00000234093	2	4	7	5	9	1	10	11	3	4	11	5	6	10	5	3	5	3	1	11	13	3	12	8	4	7	3	7	2	13	6	7
ENSG00000142959	8	1	4	2	5	4	1	6	0	2	12	2	2	1	0	0	0	1	0	8	3	9	8	3	2	0	8	10	3	2	2	3
ENSG00000173846	202	122	251	173	193	205	140	199	131	97	1133	170	136	125	194	221	119	131	149	261	393	225	267	217	124	195	250	584	160	235	209	254
ENSG00000188396	1	4	3	3	1	10	2	6	0	2	11	0	7	2	5	2	1	4	1	3	8	9	7	1	0	1	2	6	1	2	3	7
ENSG00000222009	8	11	16	6	28	36	31	22	1	3	45	5	9	9	25	8	5	1	12	11	41	17	29	17	0	3	15	10	7	20	26	31
ENSG00000117425	46	40	41	48	30	32	25	38	23	28	146	14	13	15	24	31	16	12	9	50	47	26	20	22	16	29	27	51	10	33	14	34
ENSG00000202444	0	0	0	0	0	0	0	0	0	0	0	0	0	0	0	0	0	0	0	0	0	0	0	0	0	0	0	0	0	0	0	0
ENSG00000226499	4	2	5	9	8	1	1	3	1	3	32	2	4	1	4	3	2	0	0	9	9	3	1	2	3	2	4	16	4	4	4	5
ENSG00000070785	436	303	345	291	395	453	334	434	506	385	1500	250	723	663	794	846	494	721	562	557	1192	501	811	470	482	780	296	1192	843	841	1056	1022
ENSG00000229815	1	0	0	0	1	1	3	1	1	5	8	0	0	0	1	0	1	0	0	12	3	2	9	9	2	3	3	6	1	1	8	8
ENSG00000232369	8	8	3	6	2	1	1	0	0	0	1	0	0	1	0	0	4	0	4	9	1	2	1	3	0	0	1	0	0	4	0	0
ENSG00000268949	14	2	23	9	1	3	2	8	3	1	14	0	5	5	3	2	4	5	18	26	17	4	7	7	0	1	0	8	6	5	6	5
ENSG00000126107	995	744	915	725	607	633	426	707	347	335	1863	271	360	311	381	458	263	302	327	546	1025	454	814	424	302	451	601	1071	347	421	315	418
ENSG00000126088	1194	700	979	726	753	757	602	794	609	459	2055	444	443	402	453	502	1400	1578	1666	1276	1742	704	1377	598	514	801	544	1410	603	514	662	694
ENSG00000162415	61	92	69	96	744	765	534	836	22	20	62	18	71	149	85	191	21	82	44	187	1052	486	662	505	2	26	13	42	51	158	58	194
ENSG00000234315	2	0	0	2	5	1	3	8	0	1	0	0	0	0	0	0	0	1	0	0	0	4	4	3	1	0	0	0	0	0	0	1
ENSG00000281912	32	30	25	25	30	31	16	25	28	13	43	8	11	20	10	29	16	17	17	35	33	4	8	15	11	12	8	26	9	12	10	17
ENSG00000186603	8	3	10	3	13	20	12	24	423	359	832	250	920	738	702	956	3	2	3	16	43	16	25	20	348	639	113	612	899	872	741	915
ENSG00000132781	647	491	577	564	859	763	610	808	858	579	2265	505	429	368	446	499	443	627	474	755	1532	706	1289	657	568	933	394	1386	314	441	391	557
ENSG00000132773	620	382	561	424	489	486	408	507	449	320	1388	325	222	213	292	313	416	631	689	700	1007	428	828	421	409	629	217	1000	249	336	344	343
ENSG00000070759	359	303	336	331	224	244	166	235	56	65	283	59	109	122	112	164	120	169	142	251	292	114	204	119	66	96	69	224	51	107	60	82
ENSG00000236872	3	5	3	0	2	5	2	2	0	0	5	0	0	0	0	1	0	1	0	2	2	1	3	1	0	0	0	4	0	2	1	2
ENSG00000280670	156	158	175	156	114	119	94	126	134	108	490	94	28	26	33	47	49	61	58	70	176	75	128	46	106	145	111	270	34	44	35	35
ENSG00000132763	588	408	599	447	398	411	285	437	394	295	1258	248	416	333	426	444	379	502	478	499	712	307	515	270	358	506	216	776	382	499	475	446
ENSG00000117450	12290	8073	9792	8582	14542	16848	10727	17646	4744	3326	12993	2593	9361	8237	10196	10885	16735	23158	22600	14207	31331	14227	20376	11706	5092	7259	3390	10161	10450	9578	14152	11757
ENSG00000234379	1	0	0	0	0	0	0	0	0	0	0	0	0	0	0	2	0	0	0	0	0	0	0	0	0	0	0	0	0	0	0	0
ENSG00000117448	1920	1404	1457	1352	3074	3203	2135	3203	1796	1387	5138	1045	2727	2360	2597	3115	1335	1802	1684	1503	5554	2225	3657	1868	1415	2162	1016	3191	1965	2122	2049	2540
ENSG00000281133	1	3	5	4	1	3	0	1	1	0	0	0	1	3	1	3	1	1	0	4	1	0	2	1	0	2	1	5	2	1	1	0
ENSG00000280836	11	4	9	1	3	3	3	3	2	5	2	5	3	3	4	3	0	2	4	4	4	3	3	3	2	5	0	10	1	2	3	0
ENSG00000132780	11682	8284	9780	8254	8059	8537	6866	9247	8781	6765	25577	5202	6699	6422	6936	8556	7207	8798	8340	8393	18551	7283	13267	6744	7420	10693	4650	18871	6208	7708	7155	9162
ENSG00000281112	4	0	5	1	2	1	2	3	2	1	2	1	1	0	1	1	0	0	1	3	2	1	4	1	1	2	1	12	0	2	2	3
ENSG00000281825	1	0	1	0	2	1	0	0	1	0	5	2	1	0	0	0	0	0	0	0	2	4	1	2	0	3	1	2	2	2	0	1
ENSG00000159588	85	60	93	70	58	95	52	111	42	43	166	18	32	34	34	35	19	12	15	58	87	65	62	80	26	40	46	115	12	37	30	52
ENSG00000159592	5751	3919	4610	3750	2660	2698	2058	2823	2333	1861	9026	1360	2359	2035	2151	2626	2225	2680	2372	3196	4639	1823	3117	1700	2205	3206	2146	5597	1493	1976	1541	2094
ENSG00000225447	30	21	19	18	8	5	15	13	14	10	44	7	6	11	13	19	7	7	2	15	19	5	10	9	11	11	17	29	2	8	7	21
ENSG00000234329	53	33	50	35	23	18	21	21	22	19	101	18	15	23	16	29	17	6	6	37	60	23	30	33	26	40	14	81	5	19	15	28
ENSG00000159596	606	366	529	389	554	585	439	614	606	383	1588	316	623	564	759	768	596	694	571	578	1214	514	884	515	493	657	390	1038	699	701	834	910
ENSG00000197429	936	564	705	640	209	257	162	267	526	362	1881	307	384	355	322	512	495	633	645	602	470	196	362	135	379	606	470	1258	263	340	241	324
ENSG00000230896	72	47	99	46	30	25	18	28	39	41	199	24	34	48	26	45	34	60	55	62	37	10	25	16	26	46	50	136	31	28	24	30
ENSG00000236111	0	0	0	1	0	0	0	0	0	0	1	0	0	0	1	0	1	0	0	0	0	0	0	0	0	0	0	1	0	0	0	0
ENSG00000086015	1644	1555	1707	1521	1682	1759	1198	1748	1005	847	3160	653	810	890	842	1140	298	438	352	1011	2164	1007	1560	910	772	1457	574	2482	504	809	482	793
ENSG00000232467	5	1	10	1	1	1	0	0	0	1	5	1	1	1	0	2	0	1	0	6	3	0	0	2	5	2	0	12	1	5	1	2
ENSG00000117461	2751	1941	2109	1926	1974	1916	1609	2136	554	405	3295	369	348	375	380	531	846	1193	1056	1742	4219	1684	3326	1579	765	1192	743	2051	298	484	337	476
ENSG00000278139	0	0	0	0	0	0	0	0	0	0	0	0	0	0	0	0	0	0	0	0	0	0	0	0	0	0	0	0	0	0	0	0
ENSG00000226957	4	3	6	0	9	6	2	5	4	1	13	1	2	0	4	3	0	2	4	7	7	7	7	19	2	4	2	13	1	1	1	3
ENSG00000233114	4	2	3	4	2	8	3	6	1	0	5	1	1	0	0	3	1	0	1	6	7	8	9	4	2	1	0	16	0	0	1	2
ENSG00000227857	6	15	4	4	1	1	0	0	2	3	9	1	1	0	0	2	7	5	8	9	9	3	2	7	2	0	1	4	2	2	2	2
ENSG00000117472	8	3	11	5	5	10	5	7	8	1	14	2	4	0	8	3	0	2	5	3	9	8	9	8	2	6	2	8	0	3	2	7
ENSG00000250719	0	0	1	0	0	0	0	0	0	0	0	0	0	0	0	0	0	0	0	0	0	0	0	0	0	0	0	0	0	0	0	0
ENSG00000085998	1649	1234	1447	1207	1006	1008	715	1066	855	705	3711	625	733	680	719	919	645	1006	959	1250	1504	653	1098	568	735	1182	811	2023	596	752	636	801
ENSG00000171357	9	14	10	6	1	2	1	5	1	0	2	0	0	0	1	0	1	5	5	9	1	1	1	1	0	0	1	0	0	4	0	0
ENSG00000085999	2860	2319	2605	2197	1806	1942	1638	2136	1285	996	2900	737	1285	1075	1323	1514	756	956	882	1684	2766	1196	2023	1186	981	1631	459	2653	770	1113	824	1254
ENSG00000132128	1745	1308	1640	1370	2115	2273	1629	2309	1524	1268	5822	1000	1341	1266	1509	1658	544	734	688	1319	2758	1284	2017	1182	1190	1937	1284	3559	1094	1431	1265	1553
ENSG00000173660	4713	3057	3423	3188	3666	3752	2663	4313	3786	2575	9278	1934	2934	2899	3290	3820	5368	5894	6435	6141	9566	4005	6910	5298	3049	4649	1667	6200	3796	3202	4483	4365
ENSG00000117481	1087	766	880	754	1085	1090	783	1145	648	522	2118	401	779	657	783	880	408	609	529	723	1768	715	1201	612	549	898	493	1398	536	729	627	803
ENSG00000225779	9	9	4	7	5	8	5	7	3	4	10	1	8	2	1	2	2	3	0	10	11	5	2	3	5	11	6	13	0	2	0	3
ENSG00000117480	95	94	96	79	50	44	27	52	48	42	148	14	40	39	32	41	42	44	19	78	52	20	33	25	19	50	36	84	15	36	22	36
ENSG00000232022	6	9	3	4	2	2	2	4	16	11	31	11	3	7	2	15	4	4	3	7	2	0	3	2	0	2	4	11	2	0	0	7
ENSG00000224863	0	3	0	2	0	1	0	0	4	6	11	3	0	2	1	3	0	1	0	1	0	0	0	0	0	0	1	4	0	1	0	1
ENSG00000197587	0	0	0	0	0	1	0	0	0	0	1	0	0	0	0	0	0	0	0	0	0	0	0	0	0	0	0	1	0	1	0	0
ENSG00000282881	0	0	0	0	0	0	0	0	0	0	1	0	0	0	0	0	0	0	0	0	0	0	0	0	0	0	0	0	0	0	0	0
ENSG00000269956	25	32	19	32	12	16	11	9	11	11	63	6	6	12	6	11	8	1	7	24	22	15	9	11	7	13	22	35	5	10	5	7
ENSG00000162456	0	0	0	0	0	0	0	0	0	0	1	0	0	0	0	0	0	0	0	0	0	0	0	0	0	0	0	1	0	0	0	0
ENSG00000079277	733	575	724	611	472	594	430	562	645	529	2331	438	359	439	362	558	255	325	270	563	856	416	556	376	342	597	504	1245	239	349	311	410
ENSG00000142961	168	116	191	96	245	247	135	230	341	266	1550	219	114	122	129	170	74	50	97	121	319	164	271	157	193	377	411	854	84	122	84	144
ENSG00000123472	979	817	892	824	100	81	70	94	1025	815	3137	616	772	668	738	798	395	915	669	897	201	64	127	72	859	1333	640	2104	554	658	536	672
ENSG00000233647	3	5	2	2	0	3	2	2	2	0	4	0	0	3	0	1	1	0	0	3	2	5	0	4	2	2	2	3	0	3	1	0
ENSG00000186118	6	5	5	5	14	20	15	15	5	2	18	4	2	4	2	7	1	1	1	4	26	8	10	19	6	5	5	23	0	4	2	6
ENSG00000228237	25	16	21	9	23	20	23	25	20	14	62	11	10	12	7	8	2	3	6	24	40	10	16	20	9	16	15	43	6	6	12	16
ENSG00000159658	3113	2197	2676	2139	2231	2248	1629	2379	1608	1356	8033	1275	1568	1412	1634	1890	776	1163	1096	1587	4019	1727	2721	1481	1754	2677	1980	5429	1005	1374	1125	1443
ENSG00000236476	0	0	0	0	0	0	0	0	0	0	0	0	0	0	0	0	0	0	0	0	0	0	0	0	0	0	0	0	0	0	0	0
ENSG00000142973	0	2	2	1	0	0	0	0	0	0	2	0	0	1	2	1	0	0	0	0	0	0	0	0	0	0	3	0	0	1	0	0
ENSG00000154198	0	0	0	0	0	0	0	0	0	0	1	0	0	0	0	0	0	0	0	0	0	0	0	0	0	0	0	0	0	0	0	0
ENSG00000238145	0	0	0	0	0	0	1	0	0	0	0	0	0	0	0	0	0	0	0	0	0	0	0	0	0	0	0	0	0	0	0	0
ENSG00000187048	0	0	0	0	0	0	2	0	0	0	0	0	0	0	0	0	0	0	0	0	0	0	0	0	0	0	0	0	0	0	0	0
ENSG00000259832	0	0	0	0	0	0	0	0	0	0	0	0	0	0	0	0	0	0	0	0	0	0	0	0	0	0	0	0	0	0	0	0
ENSG00000261196	0	0	0	0	0	0	0	0	0	0	0	0	0	0	0	0	0	0	0	0	0	0	0	0	0	0	0	0	0	0	0	0
ENSG00000261593	0	0	0	0	0	0	0	0	0	0	0	0	0	0	0	0	0	0	0	0	0	0	0	0	0	0	0	0	0	0	0	0
ENSG00000261290	0	0	0	0	0	0	0	0	0	0	0	0	0	0	0	0	0	0	0	0	0	0	0	0	0	0	0	0	0	0	0	0
ENSG00000186377	1	1	5	4	4	1	2	3	0	1	2	0	6	1	6	6	1	3	2	4	4	0	0	1	0	1	0	0	0	8	2	3
ENSG00000186160	1	0	0	1	1	1	2	1	1	1	4	0	1	1	0	0	0	1	0	1	5	4	1	2	0	1	3	0	0	2	1	1
ENSG00000225154	0	0	0	0	0	0	0	0	0	0	0	0	0	0	0	0	0	0	0	0	0	0	0	0	0	0	0	0	0	0	0	0
ENSG00000225506	1	4	1	7	37	33	36	22	4	9	33	4	10	11	4	12	0	2	3	2	43	12	32	21	0	2	4	6	1	8	6	5
ENSG00000162365	0	0	1	1	2	5	3	4	1	1	2	0	1	2	2	6	0	0	0	1	8	12	8	6	0	0	1	7	0	1	0	0
ENSG00000225514	0	0	0	0	0	0	1	3	0	0	1	1	0	1	0	0	0	0	0	0	0	1	1	2	0	0	0	2	0	0	0	0
ENSG00000224805	2	1	4	6	12	4	11	5	3	4	8	0	6	6	2	3	3	3	5	4	13	7	15	10	1	0	0	5	2	3	1	3
ENSG00000162366	0	2	3	1	0	0	0	1	0	0	0	0	0	0	0	0	0	0	1	0	0	0	0	0	0	0	0	0	0	0	0	0
ENSG00000162367	4936	3156	4992	3313	0	0	1	0	4	6	6	4	1	5	2	4	1373	1366	1725	2614	5	0	0	3	1	1	0	3	2	0	0	0
ENSG00000226252	292	222	416	221	0	0	0	0	0	0	0	0	0	1	0	0	148	96	122	253	0	0	1	1	0	0	0	0	0	0	0	2
ENSG00000123473	2963	2120	2651	2088	1674	1681	1228	1771	1384	1124	4066	919	886	824	966	1052	1371	1566	1822	1876	3427	1366	2371	1298	1594	2441	695	4158	742	929	1018	1136
ENSG00000162368	3248	2207	2518	1991	2300	2311	1645	2348	2886	2361	11113	1764	3102	2834	3153	3509	2133	2829	2921	2933	5271	2192	3567	1914	3285	4586	2535	7551	2938	3364	3209	3623
ENSG00000225762	13	19	23	13	14	11	3	12	5	4	36	4	5	6	6	11	7	22	12	30	20	7	9	10	5	17	11	29	1	6	3	5
ENSG00000186790	8	15	10	4	3	3	2	2	1	2	2	0	3	1	1	1	3	0	1	6	1	2	1	1	1	3	0	0	1	0	0	2
ENSG00000237424	570	555	455	485	357	327	219	383	225	167	709	131	253	215	173	239	62	72	91	179	485	172	256	154	169	279	121	380	64	102	56	131
ENSG00000186564	260	248	238	225	116	141	101	136	113	79	276	72	68	71	52	60	44	69	70	136	188	93	120	72	70	109	63	209	8	43	18	43
ENSG00000271355	0	0	0	0	0	0	0	0	0	0	0	0	0	0	0	0	0	0	0	0	0	0	0	1	0	0	0	3	0	0	0	0
ENSG00000229554	0	0	0	0	0	0	0	0	0	0	0	0	0	0	0	0	0	0	0	0	0	0	0	0	0	0	0	1	0	0	1	0
ENSG00000227883	0	0	0	0	0	0	0	0	0	0	0	0	0	0	0	0	0	0	0	0	0	0	0	0	0	0	0	0	0	0	0	0
ENSG00000227947	0	0	0	0	0	0	0	0	0	0	3	0	0	0	0	0	0	0	0	0	0	0	0	0	0	0	0	1	0	0	0	0
ENSG00000269113	0	0	0	1	1	5	3	2	0	0	3	0	0	0	0	0	0	3	0	0	16	10	7	4	0	0	0	0	0	0	0	0
ENSG00000223814	0	0	0	0	0	0	0	0	0	0	0	0	0	0	0	0	0	0	0	0	0	0	0	0	0	0	0	0	0	0	0	0
ENSG00000225028	0	0	0	0	0	0	0	0	0	0	0	0	0	0	0	0	0	0	0	0	0	0	0	0	0	0	0	0	0	0	0	0
ENSG00000226133	0	0	0	0	0	0	0	0	0	0	0	0	0	0	0	0	0	0	0	0	0	0	0	0	0	0	0	0	0	0	0	0
ENSG00000231413	0	0	0	0	0	0	0	0	0	0	0	0	0	0	0	0	0	0	0	0	0	0	0	0	0	0	0	0	0	0	0	0
ENSG00000237180	0	0	0	0	0	0	0	0	0	0	0	0	0	0	0	0	0	0	0	0	0	0	0	0	0	0	0	0	0	0	0	0
ENSG00000242267	0	2	0	0	0	0	0	0	0	0	0	0	0	0	0	0	0	0	0	0	0	0	0	0	0	0	0	0	0	0	0	0
ENSG00000279096	0	0	0	0	0	0	0	0	0	0	0	0	0	0	0	0	0	0	0	1	0	0	0	0	0	1	1	0	0	1	0	0
ENSG00000223720	0	1	1	0	0	0	0	0	0	0	0	0	0	0	0	0	0	0	0	0	0	0	0	0	0	0	0	0	0	0	0	0
ENSG00000117834	4	5	2	6	0	0	2	0	0	0	1	1	0	0	0	0	1	0	1	12	1	0	2	0	0	0	2	2	0	0	0	0
ENSG00000272491	1	0	3	0	0	0	0	0	0	0	0	0	0	0	0	0	0	1	0	1	0	0	0	0	0	0	0	1	0	0	0	0
ENSG00000279214	1	0	0	0	0	0	0	0	0	0	0	0	0	0	0	0	0	0	0	0	0	0	0	0	0	0	0	0	0	0	0	0
ENSG00000132122	101	93	104	92	0	0	0	0	24	26	74	18	3	9	5	8	16	37	31	69	0	0	0	0	21	23	35	77	3	8	4	7
ENSG00000224986	1	2	0	0	0	0	0	0	0	0	1	0	0	0	0	0	0	0	0	0	0	0	0	0	0	1	0	1	0	0	0	0
ENSG00000206700	0	0	0	0	0	0	0	0	0	0	0	0	0	0	0	0	0	1	0	0	0	0	0	0	0	0	0	0	0	0	0	0
ENSG00000235105	4	1	0	3	1	0	0	1	0	4	5	0	0	0	0	2	1	3	3	1	2	1	1	0	1	0	0	5	0	1	1	1
ENSG00000223175	4	0	1	0	0	0	0	0	0	0	0	0	0	0	0	0	0	0	0	0	0	0	0	0	0	0	0	2	0	0	0	0
ENSG00000232514	0	0	0	0	0	0	0	0	0	0	0	0	0	0	0	0	1	0	0	0	0	0	0	0	0	0	0	0	0	0	0	0
ENSG00000186094	4	4	6	8	0	1	2	0	3	4	3	1	0	0	0	0	3	1	1	4	2	3	0	1	0	0	0	2	0	0	0	0
ENSG00000162373	499	399	368	325	598	560	435	719	74	60	67	23	0	1	0	0	167	153	115	157	363	125	252	122	3	12	15	34	0	0	0	0
ENSG00000229846	0	0	0	0	0	0	0	0	0	0	0	0	0	0	0	0	0	0	0	0	0	0	0	0	0	0	0	0	0	0	0	0
ENSG00000230114	0	0	0	0	0	0	0	0	0	0	0	0	0	0	0	0	0	0	0	0	0	0	0	0	0	0	0	0	0	0	0	0
ENSG00000225623	0	0	0	0	0	0	0	0	0	0	0	0	0	0	0	0	0	0	0	0	0	0	0	0	0	0	1	0	0	0	0	0
ENSG00000237478	0	0	0	0	0	0	0	0	0	0	0	0	0	0	0	0	0	0	0	0	0	0	0	0	0	0	0	0	0	0	0	0
ENSG00000215887	0	0	0	0	0	0	0	0	0	2	1	0	0	0	0	0	0	0	0	0	0	0	0	0	0	0	0	0	0	0	0	0
ENSG00000279324	0	0	0	0	0	0	0	0	0	0	0	0	0	0	0	0	0	0	0	0	0	0	0	0	0	0	0	0	0	0	0	0
ENSG00000233595	0	0	0	0	0	0	0	0	0	0	0	0	0	0	0	0	0	0	0	0	0	0	0	0	0	0	0	0	0	0	0	0
ENSG00000162374	0	0	0	0	0	0	0	0	0	0	0	0	0	2	0	0	1	0	1	0	0	0	0	0	0	0	1	0	0	0	0	0
ENSG00000230828	0	0	0	0	0	0	0	0	0	0	0	0	0	0	0	0	0	0	0	0	0	0	0	0	0	0	0	0	0	0	0	0
ENSG00000237337	0	0	0	0	0	0	0	0	0	0	0	0	1	0	0	0	0	0	0	0	0	0	0	0	0	0	0	1	0	0	0	1
ENSG00000233407	0	0	0	0	0	0	0	0	0	0	0	0	0	0	0	0	0	0	0	0	0	0	0	0	0	0	0	0	0	0	0	0
ENSG00000234080	0	0	0	0	0	0	0	0	0	1	0	0	0	0	0	1	1	0	0	0	0	0	0	0	0	0	0	0	0	3	0	0
ENSG00000229316	0	0	0	0	0	0	0	0	0	0	0	0	0	0	0	0	0	0	0	0	0	2	0	0	0	0	0	0	0	0	0	0
ENSG00000225671	0	0	0	0	0	2	1	0	0	0	0	0	0	0	0	0	0	0	1	0	0	2	0	0	0	0	0	0	0	0	0	0
ENSG00000142700	12	5	18	13	0	4	2	2	1	0	1	0	0	0	0	0	4	8	13	16	1	2	0	1	2	0	1	2	0	0	0	0
ENSG00000185104	2374	1779	2237	1768	2183	1908	1699	2182	1716	1220	4566	975	2212	1916	2344	2456	1363	1906	1826	1778	4294	1579	3236	1463	1522	2300	991	3707	1549	1836	2028	2161
ENSG00000225767	19	23	13	16	13	18	13	18	14	21	54	5	16	13	16	17	2	1	11	10	43	14	29	13	22	15	13	51	0	15	26	24
ENSG00000207194	2	0	0	1	3	1	4	1	6	1	6	0	2	1	3	1	0	0	1	1	2	0	2	3	1	0	1	3	0	0	1	1
ENSG00000230585	17	13	12	6	6	7	4	5	1	7	26	5	7	10	15	17	6	2	8	11	11	12	12	20	9	10	6	55	6	14	16	13
ENSG00000237130	2	2	4	0	2	4	4	1	1	4	0	2	1	1	1	2	1	1	2	8	9	2	5	5	3	4	1	17	0	3	0	9
ENSG00000123080	3869	2722	3875	3025	5122	5153	3669	5230	3624	2985	11785	2826	459	504	331	669	1167	1786	2347	1853	9328	3665	7149	2876	3938	6185	2030	10850	283	390	276	462
ENSG00000265538	1	0	1	0	1	0	0	0	0	0	0	0	0	0	0	0	0	0	0	0	0	0	0	0	2	0	0	3	0	0	0	0
ENSG00000275816	0	0	0	1	0	0	0	0	4	0	2	0	0	0	0	0	0	0	0	0	0	0	0	0	0	2	0	4	0	0	0	0
ENSG00000204006	0	0	0	0	0	0	0	0	4	2	9	2	0	0	0	0	0	0	1	0	0	0	1	0	0	3	1	1	0	0	0	0
ENSG00000200262	0	0	0	0	0	0	0	1	0	0	0	0	0	0	0	0	0	0	0	0	0	0	0	0	0	0	0	0	0	0	0	0
ENSG00000213478	0	0	1	0	0	0	0	0	0	0	1	0	0	0	0	0	0	1	0	0	1	0	0	0	1	0	0	0	0	0	0	0
ENSG00000203356	0	0	1	0	0	1	0	0	2	2	13	1	0	0	1	0	5	0	0	2	2	0	2	0	2	2	8	3	1	0	0	0
ENSG00000123091	1066	731	969	790	1302	1253	960	1354	705	649	3667	496	520	504	521	631	453	704	761	893	2061	842	1480	724	816	1067	1166	2568	487	586	512	656
ENSG00000233406	6	5	3	2	3	3	0	10	4	2	5	1	1	5	3	2	0	1	0	7	3	2	4	3	4	6	4	6	0	4	1	5
ENSG00000236434	7	4	4	1	3	1	2	8	4	2	8	0	1	0	3	1	0	0	0	5	9	3	3	4	0	3	7	11	0	3	1	5
ENSG00000085831	2	2	6	8	6	1	10	6	2	1	10	2	27	32	18	20	2	5	5	4	13	8	3	4	2	5	6	10	7	9	12	8
ENSG00000261664	0	0	0	0	0	0	0	0	0	0	0	0	1	0	1	0	0	0	0	0	0	0	0	0	0	0	0	0	0	0	0	0
ENSG00000085832	2412	1950	2118	1968	2610	2666	2149	2746	1983	1590	8231	1226	1761	1813	1873	2293	1467	2306	1723	2129	6164	2371	4351	2424	2407	3131	2084	5700	1447	1757	1878	2200
ENSG00000232027	6	7	6	6	9	9	2	6	7	6	16	2	2	1	3	3	0	3	1	5	13	8	9	9	6	12	7	13	0	4	1	7
ENSG00000206595	0	2	1	2	0	1	2	1	0	3	8	1	0	0	0	2	0	0	0	1	2	3	2	0	5	3	1	8	1	2	1	0
ENSG00000238140	2	3	3	2	8	5	3	5	0	8	13	2	2	1	3	1	4	5	2	6	14	7	5	3	5	4	2	11	0	2	6	4
ENSG00000227070	10	14	5	6	12	4	6	6	16	9	79	16	21	19	20	20	4	1	0	9	7	5	7	4	11	16	13	51	16	13	16	19
ENSG00000252032	0	0	0	0	0	0	0	0	0	0	10	0	1	0	0	5	0	0	0	0	0	0	0	0	0	4	2	10	0	6	0	2
ENSG00000227742	1	3	2	2	6	8	7	5	6	2	19	1	11	13	13	16	1	0	0	0	13	4	11	2	6	2	4	23	8	12	17	8
ENSG00000117859	3166	2314	2500	2287	2445	2403	2015	2828	2616	1970	8895	1506	3643	3261	3376	4339	1818	2651	2287	2488	5409	2005	3768	2047	2132	3075	2095	5607	2403	3065	3034	3563
ENSG00000236973	1	0	1	1	3	0	0	0	0	0	3	0	0	0	0	0	0	0	0	3	2	1	1	1	1	0	1	5	0	2	1	3
ENSG00000232846	0	2	1	0	4	1	0	0	0	0	2	0	0	1	0	2	1	0	0	1	1	0	0	0	0	3	1	7	0	0	1	3
ENSG00000212624	1	0	0	1	0	0	2	0	0	0	3	0	1	1	3	0	0	0	3	0	3	0	2	1	0	2	1	6	0	2	2	3
ENSG00000078618	5977	4477	4800	4581	3767	4219	2944	4408	4061	3192	14564	2451	3834	3721	3566	4680	3206	5122	4316	4787	7000	2938	5101	2637	3686	5738	3428	9461	2939	3800	3340	4295
ENSG00000266993	64	43	60	41	45	50	28	46	64	53	204	34	26	31	17	32	16	25	6	53	59	35	36	42	48	53	45	125	8	33	14	27
ENSG00000272175	6	3	5	2	5	3	3	4	3	4	26	0	1	4	0	5	0	1	1	3	2	7	1	3	5	3	7	13	0	0	2	3
ENSG00000230604	5	4	4	2	8	3	4	5	2	1	12	0	0	1	0	0	0	0	0	1	12	4	2	7	6	8	8	9	0	3	1	3
ENSG00000169213	38	102	41	103	8	16	5	21	22	26	55	24	16	54	10	49	4	12	8	34	13	2	2	4	9	17	11	59	2	17	13	23
ENSG00000229032	0	0	1	0	0	0	0	0	0	0	1	0	0	0	0	0	0	0	0	0	0	0	0	0	0	0	0	0	0	0	0	0
ENSG00000264613	0	0	0	0	0	0	0	0	0	0	0	0	0	0	0	0	0	0	0	0	0	0	0	0	0	0	0	0	0	1	0	0
ENSG00000117862	1885	1456	1524	1366	1391	1470	949	1461	1244	937	3413	734	1503	1415	1667	1913	1445	2171	1760	1587	2669	1070	1714	1048	1056	1695	739	2396	1796	1806	2331	2264
ENSG00000198841	288	191	278	195	297	312	234	318	230	195	817	160	354	261	368	406	212	254	284	301	651	256	503	230	207	347	164	466	371	318	396	387
ENSG00000223390	5	5	8	7	3	7	10	6	1	3	13	3	2	0	1	8	2	1	1	5	6	6	9	11	4	4	1	11	0	2	4	2
ENSG00000228369	7	7	18	16	5	13	5	9	6	9	13	3	5	4	6	5	3	1	0	11	16	4	6	5	9	17	5	15	2	9	8	14
ENSG00000134717	1900	1222	1479	1148	1398	1426	1069	1520	983	816	3443	643	1467	1319	1661	1843	1707	1987	1835	1857	3061	1146	2176	1458	980	1363	820	2282	1369	1465	1740	1743
ENSG00000157077	887	750	823	685	519	621	391	631	32	41	71	43	317	333	336	438	318	464	395	653	923	388	643	326	16	56	25	102	180	391	260	346
ENSG00000241745	1	0	3	0	2	0	0	1	0	0	0	0	0	0	0	0	1	0	0	5	0	0	0	2	0	0	0	0	0	0	2	0
ENSG00000228407	6	2	5	2	6	5	1	5	1	0	1	1	3	1	1	1	12	4	9	8	8	0	1	4	0	0	0	1	1	1	6	0
ENSG00000223429	3	1	2	0	2	0	1	6	0	0	4	0	3	1	2	1	1	3	2	2	4	2	4	3	0	0	1	3	0	4	1	2
ENSG00000224255	3	5	5	2	1	3	2	5	0	0	1	0	1	1	0	4	4	0	0	1	5	4	4	1	0	0	2	2	0	1	1	4
ENSG00000236004	2	0	2	2	0	0	0	0	0	0	1	0	2	0	1	0	0	1	0	0	1	1	0	0	0	0	0	0	0	0	3	0
ENSG00000237663	3	2	1	1	0	0	1	4	0	0	1	0	0	0	0	0	0	0	0	4	5	2	1	0	1	0	0	0	1	3	2	2
ENSG00000213735	2	2	0	0	0	1	0	5	0	0	0	0	0	0	2	3	0	1	0	1	1	2	3	3	0	0	2	0	1	3	1	1
ENSG00000154222	1564	1064	1406	1143	920	1115	764	1140	751	619	3680	502	382	366	504	541	618	613	680	1132	1472	749	1086	704	578	964	960	2429	326	528	379	644
ENSG00000272100	16	10	16	14	8	4	8	5	8	4	18	2	2	6	1	2	7	2	3	14	9	8	8	3	5	9	5	13	1	4	6	6
ENSG00000224680	8	8	8	10	17	17	20	16	14	15	39	8	0	7	3	3	1	5	12	11	46	19	41	23	15	15	7	40	8	8	12	7
ENSG00000085840	1224	995	1042	872	1289	1398	1119	1590	1030	784	2290	564	1222	1081	1293	1461	623	824	813	951	3282	1218	2393	1142	880	1391	357	2367	1154	1222	1264	1479
ENSG00000134748	3256	2175	2914	2286	3426	3660	2743	3873	2374	1891	7816	1355	2219	2066	2507	2674	2145	2220	2346	2619	6799	2751	4993	2852	2292	3306	1640	5304	1916	2320	2306	2796
ENSG00000134744	3968	3083	3616	2982	3438	3762	2900	4035	2540	2325	9863	1536	1821	1921	1823	2442	936	1123	1047	2186	5683	2574	3839	2460	1961	3257	2343	5961	991	1962	1210	2117
ENSG00000272371	6	3	3	3	6	2	0	1	4	4	5	1	0	3	0	1	2	0	4	5	4	3	5	4	1	0	3	6	0	2	5	1
ENSG00000236290	0	6	1	3	3	4	2	7	2	2	6	1	1	2	1	3	0	0	0	0	9	2	2	1	0	0	0	5	0	1	0	0
ENSG00000116157	1	3	12	4	898	903	569	955	4	1	4	1	0	2	0	1	5	4	10	5	1697	654	1246	606	0	0	0	1	2	3	0	0
ENSG00000182183	2	0	1	0	4	0	1	1	1	1	5	0	1	1	1	2	0	0	1	1	1	1	0	3	0	0	4	5	0	1	0	0
ENSG00000162377	1096	739	932	781	1393	1402	1115	1492	700	529	2304	444	1444	1332	1736	1678	881	1201	1075	1191	3056	1184	2263	1076	752	1145	418	1573	1678	1802	1855	1995
ENSG00000233664	0	0	0	0	0	0	0	0	0	0	0	0	0	0	0	0	0	0	0	0	0	0	0	0	0	0	0	0	0	0	0	0
ENSG00000239640	0	0	0	0	0	0	0	0	0	0	0	0	0	0	0	0	0	0	0	0	0	0	0	0	0	0	0	0	0	0	0	0
ENSG00000162378	735	618	751	605	1237	1153	802	1162	267	311	887	163	764	776	596	851	266	421	333	593	1831	836	1273	718	203	334	280	748	394	618	457	707
ENSG00000252018	0	1	0	0	0	0	0	0	0	0	0	0	0	0	0	0	0	0	0	0	0	0	0	1	0	0	0	0	0	0	0	0
ENSG00000228929	3	5	7	0	6	1	5	6	9	4	10	7	6	4	3	5	5	2	10	9	18	4	6	6	3	8	1	10	4	3	8	7
ENSG00000206627	2	1	1	0	1	2	3	4	0	0	1	1	0	1	1	0	0	1	1	2	5	2	3	4	0	0	1	0	0	2	0	0
ENSG00000203995	5	9	5	5	0	0	0	1	0	0	3	0	431	388	405	490	0	4	2	8	0	0	0	0	0	0	1	1	329	479	422	572
ENSG00000242391	0	0	0	0	0	0	0	0	0	0	0	0	13	15	15	6	0	0	0	1	0	0	0	0	0	0	0	0	3	10	10	18
ENSG00000121310	3	5	2	6	1	0	3	1	41	45	177	32	719	638	679	877	2	2	0	9	2	0	0	1	43	46	93	136	345	684	376	775
ENSG00000230953	0	0	0	0	0	1	0	0	0	3	6	1	18	14	10	17	0	0	0	1	0	0	0	0	0	2	1	6	3	11	9	14
ENSG00000231866	0	0	0	0	0	0	0	0	0	1	0	0	0	0	0	0	0	0	0	0	0	0	0	0	0	0	1	0	2	0	0	0
ENSG00000116171	2808	1772	2136	1860	2116	2339	1699	2587	1672	1384	5974	1022	1710	1548	1702	2077	2606	3122	2868	2558	5202	2153	3701	2221	1451	2179	1523	4026	1789	1883	2013	2266
ENSG00000264834	1	0	0	2	0	0	0	0	2	0	2	0	1	0	1	2	0	0	0	3	0	1	0	0	0	2	3	0	0	1	1	0
ENSG00000266188	0	0	0	0	0	0	0	0	0	0	0	0	0	0	0	0	0	0	0	0	0	0	0	0	0	0	0	1	0	0	0	0
ENSG00000265815	0	0	1	0	0	0	0	0	0	0	0	0	0	2	0	0	0	0	0	0	0	0	0	0	0	1	0	0	0	0	0	0
ENSG00000237279	0	0	0	0	0	0	0	0	0	0	0	0	1	0	0	0	0	0	0	0	0	0	0	0	0	0	1	0	0	0	0	1
ENSG00000236360	1	1	0	1	0	1	0	4	6	3	16	1	0	1	0	0	0	0	1	0	3	0	1	2	1	10	1	4	0	1	4	0
ENSG00000226147	1	1	2	0	0	0	1	0	1	3	5	0	2	1	1	1	1	0	0	0	4	1	2	0	0	3	3	0	0	1	0	3
ENSG00000279054	1	0	1	0	1	1	2	1	0	0	2	0	1	2	1	1	0	0	0	2	0	1	0	1	0	1	1	0	0	0	3	2
ENSG00000174348	3	1	4	9	0	1	0	0	0	0	2	0	0	0	1	0	0	0	0	0	0	0	0	0	0	0	1	0	0	0	2	0

ENSG00000232993	0	0	0	0	0	0	0	0	0	0	0	0	0	0	0	0	0	0	0	0	0	0	0	0	0	0	0	0	0	0	0	0
ENSG00000227644	0	0	0	0	0	0	0	0	0	0	0	0	0	0	0	0	0	0	0	0	0	0	0	0	0	0	0	0	0	0	0	0
ENSG00000162383	0	2	1	0	3	2	0	0	0	0	0	0	0	0	0	0	1	0	0	0	0	0	0	0	2	0	0	0	0	0	0	0
ENSG00000235563	0	0	0	0	0	0	0	0	0	0	0	0	0	0	0	0	0	0	0	0	0	0	0	0	0	0	0	0	0	0	1	0
ENSG00000277397	0	0	0	0	0	0	0	1	0	0	0	0	0	0	0	0	0	0	0	0	0	0	0	0	0	0	0	0	0	0	0	0
ENSG00000157184	651	447	583	495	491	549	394	520	423	346	1469	265	390	361	334	449	237	411	321	439	810	374	525	282	334	502	292	879	283	359	254	370
ENSG00000236723	12	16	25	10	5	11	7	6	5	12	43	5	6	7	8	13	4	7	6	15	21	9	7	6	8	12	11	22	3	4	8	2
ENSG00000162384	1163	778	1023	876	692	644	494	741	701	543	2451	452	697	668	659	831	1033	1363	1088	1107	1340	567	968	508	529	827	570	1356	648	778	690	868
ENSG00000259818	14	10	14	10	3	5	2	3	3	1	6	1	1	5	4	3	1	1	2	10	2	1	2	2	1	3	0	3	0	2	2	3
ENSG00000162385	1893	1129	1637	1354	1764	1714	1335	1914	1193	819	3586	677	1278	1136	1707	1537	2862	3028	3745	2917	4690	2096	3744	2430	1245	1674	772	2383	2003	1865	2853	2516
ENSG00000226754	7	8	13	1	5	4	0	4	5	4	23	4	8	6	8	5	1	3	2	5	7	6	5	6	8	10	13	24	1	9	11	2
ENSG00000157193	3171	2348	2819	2421	2345	2598	1782	2549	1799	1520	6013	1160	2622	2313	2562	2998	1378	1409	1236	3713	4796	2105	2509	1980	1796	3066	1030	4767	1262	2536	1473	2785
ENSG00000234578	7	3	4	11	8	5	6	6	4	2	22	2	5	3	6	7	2	1	3	2	10	6	8	11	3	6	4	16	2	6	2	3
ENSG00000228838	6	3	11	4	7	14	11	6	12	4	32	7	10	7	7	9	3	1	0	7	19	10	9	16	4	29	6	45	3	27	17	20
ENSG00000225030	7	7	3	1	3	8	2	2	4	0	12	1	8	2	1	2	8	1	0	3	14	4	3	8	1	8	5	9	2	8	4	10
ENSG00000232762	21	17	19	15	25	18	21	38	13	22	44	10	21	22	30	10	7	9	9	33	49	27	26	29	28	35	4	90	9	26	30	44
ENSG00000225675	61	52	90	71	8	15	9	10	17	9	33	6	85	86	67	83	37	34	41	80	19	12	11	6	8	8	4	30	18	44	37	64
ENSG00000226938	1	0	1	1	0	0	0	0	0	0	0	0	3	6	6	5	0	0	0	0	0	0	1	0	0	0	0	0	1	3	0	5
ENSG00000230138	1	3	9	1	0	0	0	1	0	0	2	0	1	4	2	1	5	2	6	3	0	0	0	0	0	1	1	1	1	2	3	2
ENSG00000236253	0	0	0	0	0	0	0	0	0	0	2	0	0	0	0	0	0	0	0	0	0	0	0	0	0	0	0	0	1	0	0	0
ENSG00000143006	0	0	0	3	0	0	2	0	0	0	0	0	0	0	0	0	0	0	0	0	3	0	0	2	0	0	0	0	0	0	0	0
ENSG00000174332	2	0	0	1	6	3	4	0	0	2	1	0	2	0	0	0	0	1	0	1	4	1	1	1	0	1	2	0	0	1	0	0
ENSG00000239007	0	0	0	0	0	0	0	0	0	0	0	0	0	0	0	0	0	0	0	0	0	0	0	0	0	0	0	0	0	0	0	0
ENSG00000058804	3377	2079	2732	2045	3111	2876	2309	3169	2128	1654	5045	1184	2327	2011	2485	2672	3033	4346	3701	3636	6994	2685	4982	2711	2375	3262	721	4335	2502	2680	2840	2987
ENSG00000201003	8	2	6	6	4	4	1	4	2	0	10	0	2	2	5	1	0	2	0	2	3	1	2	0	1	4	0	3	0	2	1	2
ENSG00000229687	0	0	0	0	0	0	0	0	0	0	0	0	0	1	0	0	0	0	0	0	0	0	0	0	0	0	0	0	0	0	0	0
ENSG00000058799	537	377	483	345	302	347	206	332	210	169	955	142	256	242	300	319	438	586	490	393	603	228	383	200	215	335	308	584	300	323	394	411
ENSG00000211452	49	31	42	28	16	18	6	7	17	14	74	8	7	13	10	13	8	2	2	35	20	11	17	14	19	29	19	67	5	9	6	15
ENSG00000081870	1673	1049	1274	1042	1006	1092	806	1141	1349	1014	3546	736	502	484	565	635	915	1010	1201	1142	2285	959	1757	1145	1192	1575	743	2426	584	617	724	782
ENSG00000116212	977	705	887	673	796	725	573	853	607	449	1654	346	266	264	215	321	406	542	632	673	1400	483	991	481	548	822	331	1188	184	231	200	307
ENSG00000219102	10	7	3	7	7	6	3	12	8	2	23	5	3	6	6	8	6	8	2	8	13	1	7	9	3	10	4	23	5	10	4	7
ENSG00000203985	0	0	0	0	0	0	0	0	0	0	0	0	0	0	0	0	0	0	0	0	0	0	1	0	0	0	0	0	0	0	0	0
ENSG00000116209	2008	1296	1594	1306	2422	2461	1800	2586	1499	1281	5549	1002	1153	1171	1199	1504	1452	1895	1756	1515	5153	2021	3850	2193	1328	1892	1457	3489	1243	1278	1567	1521
ENSG00000280378	18	18	15	14	12	14	14	17	9	15	52	13	7	7	11	7	1	1	2	7	15	12	6	17	10	14	31	33	2	3	5	11
ENSG00000116205	1611	1284	1370	1356	891	938	728	996	854	729	2971	495	624	595	530	718	589	776	700	990	1248	542	1007	445	731	968	563	1861	361	501	395	585
ENSG00000225183	3	0	1	0	0	0	0	0	0	0	1	0	0	0	2	1	1	0	0	1	4	0	0	0	0	0	0	0	1	0	0	4
ENSG00000279049	0	0	0	0	0	0	0	0	0	0	0	0	0	0	0	0	0	0	0	0	0	0	0	0	0	0	0	0	0	0	0	0
ENSG00000256407	11	0	4	3	4	3	4	4	3	0	8	0	1	1	1	1	2	4	1	8	9	1	3	4	5	2	1	13	0	1	1	2
ENSG00000157211	2	2	3	0	1	2	3	1	1	2	1	0	0	3	3	0	0	0	1	4	3	0	2	1	2	0	0	1	0	1	0	0
ENSG00000280425	2	5	6	13	6	4	3	3	0	1	3	1	4	2	4	1	0	0	3	4	3	1	0	0	1	2	2	4	0	5	0	1
ENSG00000215883	636	457	582	505	642	611	504	673	506	404	1221	292	446	444	362	529	159	239	300	514	974	399	682	352	365	507	236	957	241	361	219	414
ENSG00000116221	3113	2120	2795	2366	2369	2315	1736	2474	1867	1411	5064	1117	2787	2160	2825	3013	3780	5502	5008	2947	5640	1917	4507	1860	1602	2631	1012	3678	3159	2904	3586	3415
ENSG00000157216	2477	1730	2440	1963	1776	1864	1395	1848	1083	938	3792	772	2660	2456	2767	3312	531	849	819	1321	2745	1205	2045	939	941	1852	1038	2894	2132	2775	1981	2966
ENSG00000198711	81	65	86	83	56	65	48	77	32	47	168	25	61	83	55	82	9	10	20	69	125	42	48	36	33	65	47	132	19	77	49	110
ENSG00000225632	115	65	131	91	0	2	1	0	19	22	39	8	12	9	13	9	78	166	167	154	2	4	1	4	10	10	18	26	6	17	10	8
ENSG00000232245	0	0	0	0	0	0	0	0	0	0	0	0	0	0	0	0	0	0	0	0	0	0	0	0	0	0	0	0	0	0	0	0
ENSG00000235741	0	0	0	0	0	0	0	0	0	0	0	0	0	0	0	0	0	0	0	0	0	0	0	0	0	1	0	2	0	0	0	0
ENSG00000237173	0	0	0	0	0	0	0	0	0	0	0	0	0	0	0	0	0	0	0	0	0	0	0	0	0	0	0	0	0	0	0	0
ENSG00000227453	0	0	0	0	0	0	0	0	0	0	0	1	0	0	0	2	0	0	0	0	0	0	0	0	0	0	0	0	0	0	0	0
ENSG00000162390	48	29	38	40	244	279	232	352	204	158	547	108	101	98	86	148	11	20	16	32	351	132	241	162	125	186	177	369	44	85	77	85
ENSG00000162391	4	0	8	4	32	33	32	35	23	14	76	13	5	8	5	8	0	5	1	5	55	18	24	19	7	14	21	42	4	9	0	3
ENSG00000230728	0	0	0	1	6	3	7	7	0	2	5	1	3	0	1	2	0	0	0	0	2	1	2	2	0	1	2	0	0	1	0	0
ENSG00000184313	2	1	3	3	35	44	39	38	34	27	61	18	8	11	11	14	0	0	0	0	34	16	11	17	3	3	29	25	3	1	1	11
ENSG00000271723	0	2	0	0	1	4	2	0	5	3	4	1	1	1	2	2	0	0	0	0	5	1	1	0	0	1	1	1	0	0	0	0
ENSG00000243725	177	117	192	131	116	131	135	164	110	80	301	42	193	133	196	184	130	204	187	193	309	123	271	112	109	159	90	270	179	192	194	229
ENSG00000162396	223	161	183	160	127	127	91	139	111	104	416	67	228	164	243	215	66	97	76	104	173	71	146	62	108	190	96	267	148	162	168	152
ENSG00000006555	0	0	0	0	0	0	0	0	0	0	0	0	17	14	16	19	0	0	0	0	0	0	0	1	0	0	0	0	10	9	11	16
ENSG00000237453	0	0	0	0	0	0	0	0	0	0	0	0	1	0	0	0	0	0	0	0	0	0	0	0	0	0	0	0	0	0	0	1
ENSG00000162398	12	16	3	6	4	12	2	8	0	3	6	1	41	28	32	35	1	0	0	6	5	4	2	1	3	8	2	9	27	27	35	34
ENSG00000116133	9092	6245	6865	6202	8411	8259	6362	8474	5295	4533	13695	3437	4456	5101	4239	6762	2429	3545	2541	5081	12915	4460	8825	3821	4888	8643	2360	12066	2848	3999	2310	4121
ENSG00000242396	13	11	9	7	8	8	5	11	7	4	16	3	14	12	9	18	6	9	9	8	17	3	7	2	9	5	2	14	6	11	6	14
ENSG00000233203	8	1	3	3	2	2	3	2	4	0	11	0	33	16	5	17	5	4	8	1	10	1	3	1	0	4	3	4	5	19	6	8
ENSG00000143001	0	0	0	0	0	0	0	0	0	0	0	0	0	0	1	0	0	0	0	0	0	0	0	0	0	0	0	0	0	0	0	0
ENSG00000233271	0	0	0	0	0	0	0	0	0	0	0	0	0	0	0	0	0	0	0	0	0	0	0	0	0	0	0	0	0	0	0	0
ENSG00000162399	0	2	0	0	0	0	0	0	1	0	0	0	4	2	0	0	0	0	0	1	0	0	0	0	0	0	0	0	2	1	0	4
ENSG00000169174	0	0	0	1	0	1	0	0	0	3	0	1	0	1	0	0	0	0	0	0	0	0	0	0	0	0	0	2	0	0	0	0
ENSG00000162402	4301	3515	4014	3479	5397	5602	3942	5624	2385	2111	8513	1528	4399	4116	4342	5197	1420	2235	1886	3273	8847	3835	6370	3484	2676	3924	2023	6303	2797	4076	3465	4579
ENSG00000231090	0	0	1	0	0	0	0	0	0	0	0	0	0	0	0	0	0	0	0	0	0	0	0	0	0	0	0	0	0	0	0	0
ENSG00000231095	1	0	0	0	0	0	1	1	0	0	0	0	0	0	0	0	0	0	0	0	1	1	0	0	0	0	0	0	0	0	0	0
ENSG00000265822	0	0	0	0	0	0	0	0	0	0	0	0	0	0	0	0	0	0	0	0	0	0	0	0	0	0	0	0	0	0	0	0
ENSG00000231900	0	0	0	0	0	0	0	0	0	0	0	0	0	0	0	0	0	0	0	0	0	0	0	0	0	0	0	0	0	0	1	0
ENSG00000232198	0	0	0	0	0	0	0	0	0	0	0	0	0	0	0	0	0	0	0	0	0	0	0	0	0	0	0	0	0	0	0	0
ENSG00000199831	0	0	0	0	0	0	0	0	0	0	0	0	0	0	0	0	0	0	0	0	0	0	0	0	0	0	0	0	0	0	0	0
ENSG00000252162	0	0	0	0	0	0	0	0	0	0	0	0	0	0	0	0	0	0	0	0	0	0	0	0	0	0	0	0	0	0	0	0
ENSG00000272051	0	0	0	0	0	0	0	0	0	0	0	0	0	0	0	0	0	0	0	0	0	0	0	0	0	0	0	0	0	0	0	0
ENSG00000234810	0	0	0	0	0	0	0	0	0	0	1	0	0	0	0	0	0	0	0	0	0	0	0	0	0	0	0	0	0	0	1	0
ENSG00000230250	0	0	0	0	0	0	0	0	0	0	0	0	0	0	0	0	0	0	0	0	0	0	0	0	0	0	0	0	0	0	0	0
ENSG00000227104	0	0	0	0	0	0	0	0	0	0	0	0	0	0	0	0	0	0	0	0	0	0	0	0	0	0	0	0	0	0	0	0
ENSG00000233079	0	0	0	0	0	0	0	0	0	0	0	0	0	0	0	0	0	0	0	0	0	0	0	0	0	0	0	0	0	0	0	0
ENSG00000235612	0	0	0	0	0	0	0	0	0	0	1	0	0	0	0	0	0	0	0	0	0	0	0	0	0	0	0	0	0	0	0	0
ENSG00000229871	0	0	0	0	0	0	0	0	0	0	0	0	0	0	0	0	0	0	0	0	0	0	0	0	0	0	0	0	0	0	0	0
ENSG00000260971	0	0	0	0	0	0	0	0	0	0	0	1	0	0	1	1	0	0	0	0	0	1	0	0	0	1	1	0	0	2	0	0
ENSG00000223956	0	0	0	0	0	0	0	0	0	0	0	0	0	0	0	0	0	0	0	0	0	0	0	0	0	0	0	0	0	0	0	0
ENSG00000162407	0	0	0	0	2	7	9	10	10	25	78	15	0	0	0	0	0	0	0	0	13	4	5	1	8	13	9	41	0	0	0	0
ENSG00000236776	0	0	0	0	0	0	0	0	0	0	1	0	0	0	0	0	0	0	0	0	0	0	0	0	0	0	0	0	0	0	0	0
ENSG00000224980	0	0	0	0	0	0	0	0	0	0	0	0	0	0	0	0	0	0	0	0	0	0	0	0	0	0	0	0	0	0	0	0
ENSG00000225475	1	0	0	0	0	0	0	0	0	0	5	0	0	0	0	0	0	0	0	0	0	0	0	0	1	0	0	0	0	0	0	0
ENSG00000162409	0	0	0	1	0	0	0	0	10	3	1	5	0	0	0	0	0	0	0	0	0	0	0	0	0	1	1	0	0	0	0	0
ENSG00000187889	0	0	0	2	0	0	0	0	1	0	2	0	0	4	0	3	0	0	0	0	0	0	1	0	1	0	0	1	0	0	2	0
ENSG00000229913	0	0	0	0	0	0	0	0	1	0	0	0	0	0	0	0	0	0	0	0	0	0	0	0	0	0	0	0	0	0	0	1
ENSG00000157131	0	0	0	0	0	0	0	0	0	0	0	0	0	0	0	0	0	0	0	0	0	0	0	0	0	1	0	0	0	0	0	0
ENSG00000021852	2	0	2	0	0	0	0	1	18	13	20	1	0	0	0	0	0	0	0	0	0	0	1	0	8	6	6	18	0	0	0	0
ENSG00000236341	0	0	0	0	0	0	0	0	0	0	0	0	0	0	0	0	0	0	0	0	0	0	0	0	0	0	0	0	0	0	0	0
ENSG00000173406	19	21	17	21	504	535	365	606	856	734	3867	504	0	0	0	2	8	7	10	8	455	152	303	155	461	829	780	1908	0	0	0	1
ENSG00000227935	0	0	0	0	0	0	0	0	11	12	47	11	0	0	0	0	0	0	0	0	0	0	0	0	10	6	13	40	0	0	0	0
ENSG00000236888	0	0	0	0	0	0	0	0	0	1	0	0	0	0	0	0	0	1	0	0	0	0	0	0	0	0	1	0	0	0	0	0
ENSG00000226759	0	0	0	0	1	0	0	1	0	0	2	0	1	0	0	0	0	0	0	1	0	1	1	0	0	0	0	0	0	0	0	0
ENSG00000229887	0	1	0	0	0	2	0	1	0	0	1	0	1	0	0	0	0	0	0	1	0	1	1	0	0	1	0	0	0	0	0	0
ENSG00000223416	1	0	1	0	0	0	0	0	0	0	1	0	0	1	0	1	1	0	0	0	2	0	0	0	0	0	1	0	0	0	0	0
ENSG00000235038	0	0	0	0	0	1	0	0	0	0	0	0	0	0	0	0	0	0	0	0	0	0	0	1	0	0	0	0	0	0	0	0
ENSG00000230546	0	0	0	0	0	0	0	0	0	0	0	0	0	0	0	0	0	0	0	0	0	0	0	0	0	0	0	0	0	0	0	0
ENSG00000233216	1	1	0	0	0	1	0	1	0	0	0	0	1	2	0	0	0	0	0	0	0	1	0	2	0	0	0	0	0	1	0	0
ENSG00000162600	656	447	521	437	344	397	272	406	555	408	2234	354	397	414	437	503	788	1027	642	804	774	320	547	349	486	670	547	1249	427	496	435	537
ENSG00000270209	0	0	0	0	0	0	0	0	0	0	0	0	0	0	0	0	0	0	0	0	0	0	0	0	0	0	0	0	0	0	0	0
ENSG00000264128	0	0	0	0	0	0	0	0	0	0	0	0	0	0	0	0	0	0	0	0	0	0	0	0	0	0	0	0	0	0	0	0
ENSG00000184292	0	0	0	0	0	1	0	2	0	0	0	0	0	0	0	0	0	0	0	1	3	0	0	0	0	0	0	0	0	0	0	0
ENSG00000185839	1	3	4	6	3	4	6	4	4	1	7	2	4	2	5	3	4	3	2	4	8	3	3	1	2	2	1	6	4	3	8	4
ENSG00000162601	3265	2159	2352	2111	2866	2885	2282	3096	1984	1861	7507	1186	1656	1611	1738	1919	1162	1289	1248	2510	5583	2575	3670	2688	1733	2433	1965	4880	965	1725	1375	1839
ENSG00000283445	1	1	0	0	9	7	10	14	2	0	5	1	0	2	1	0	0	0	0	0	7	5	5	4	0	0	4	2	0	0	0	0
ENSG00000177606	496	440	610	539	603	619	497	687	83	95	340	91	947	1710	1100	2196	121	208	257	312	595	292	548	235	19	58	53	125	579	1311	672	1326
ENSG00000234807	6	1	8	5	1	0	3	1	0	2	0	0	0	2	1	3	3	0	0	5	0	0	1	1	0	2	0	1	0	0	0	1
ENSG00000272226	11	9	8	2	0	1	0	0	0	1	1	0	0	1	1	2	5	2	3	4	0	0	0	0	0	0	0	2	0	0	1	0
ENSG00000231740	0	0	0	1	0	0	0	0	0	0	0	0	0	0	1	0	0	1	0	1	0	0	0	0	0	0	0	0	1	0	0	0
ENSG00000232453	3	0	1	2	1	2	0	3	0	0	1	1	1	0	0	0	0	1	2	0	5	0	0	0	3	0	1	0	0	0	0	0
ENSG00000237352	3	3	3	1	32	31	22	30	11	6	39	4	8	7	3	7	0	2	9	4	31	10	12	16	12	8	4	14	3	4	1	2
ENSG00000228121	0	0	0	1	0	2	0	0	0	0	0	0	0	0	0	0	0	1	0	1	0	0	0	0	0	0	0	0	0	0	0	0
ENSG00000241042	0	0	1	0	1	5	3	1	0	0	0	0	0	0	0	0	0	0	0	0	1	0	1	2	0	0	0	1	0	0	0	0
ENSG00000235215	0	0	1	0	7	1	3	3	2	0	1	0	0	0	0	0	0	0	0	0	1	0	5	2	0	0	1	0	0	0	0	0
ENSG00000224609	0	0	0	1	0	0	0	0	0	0	1	0	0	0	0	0	0	0	0	0	0	0	0	0	0	0	0	0	0	0	0	0
ENSG00000270457	0	0	1	1	0	0	0	1	0	0	0	0	0	0	0	0	0	0	1	1	1	1	1	2	0	0	0	0	0	0	0	0
ENSG00000172456	330	251	306	246	223	235	159	202	145	91	317	65	0	1	0	0	140	255	248	196	411	156	279	129	75	87	40	179	0	0	0	1
ENSG00000266150	0	0	0	0	0	0	0	0	0	0	0	0	0	0	0	0	0	0	0	0	1	0	0	0	0	0	0	1	0	0	0	0
ENSG00000226883	0	2	1	2	2	2	0	4	0	0	1	0	0	0	0	0	0	0	1	4	1	0	1	0	0	0	0	0	0	0	0	0
ENSG00000134709	0	0	4	3	4	3	1	3	739	653	2472	542	0	0	0	0	0	2	1	0	2	1	3	2	729	1081	552	1908	0	0	0	0
ENSG00000134716	4	13	6	11	3	1	0	1	25	21	106	14	1	1	0	4	2	10	6	9	0	0	0	0	8	19	13	44	0	1	1	5
ENSG00000265535	0	0	0	0	0	0	0	0	0	0	0	0	0	0	0	0	0	0	0	0	0	0	0	0	0	0	0	0	0	0	0	0
ENSG00000162598	0	0	0	0	0	0	0	2	0	0	1	0	0	0	0	0	0	0	0	0	0	0	0	0	0	0	0	0	0	0	0	0
ENSG00000229332	0	0	0	0	8	2	2	4	0	0	0	0	0	0	0	0	0	0	0	0	11	1	2	2	0	0	0	0	0	0	0	0
ENSG00000238242	0	0	0	0	1	0	0	0	0	0	0	0	0	0	0	0	0	0	0	0	0	0	0	0	0	0	0	0	0	0	0	0
ENSG00000226476	0	0	0	0	3	6	2	2	0	0	0	0	0	0	0	0	0	0	0	0	6	8	3	4	0	0	0	0	0	0	0	0
ENSG00000231252	0	0	0	0	5	9	13	7	0	0	2	0	0	0	2	0	0	0	0	0	11	10	7	12	0	0	0	0	0	0	0	0
ENSG00000162599	301	343	332	331	3	1	2	0	78	68	146	40	841	753	794	875	86	185	81	264	3	0	1	0	67	46	29	105	423	616	487	656
ENSG00000237928	0	0	0	0	69	67	62	80	0	0	0	0	0	2	1	1	0	0	3	0	206	94	187	138	0	0	0	0	3	1	6	7
ENSG00000270742	4	2	6	8	0	0	0	0	2	1	2	1	8	9	6	9	4	1	0	10	0	0	0	0	0	2	1	4	0	1	8	10
ENSG00000237853	0	6	0	0	0	0	0	0	0	0	0	0	7	1	6	5	0	0	1	2	0	0	0	0	0	0	0	0	1	4	4	7
ENSG00000223920	1	1	1	0	0	0	0	0	0	0	0	0	0	0	0	0	0	0	0	0	0	0	0	0	0	0	0	1	0	0	0	0
ENSG00000162604	860	585	620	599	456	499	367	590	422	323	1623	208	329	370	377	479	595	825	718	855	860	412	543	461	397	468	389	842	342	426	378	468
ENSG00000271200	0	0	0	0	0	0	0	0	2	0	5	2	0	0	0	0	0	0	0	0	0	0	0	0	0	3	0	4	0	2	0	0
ENSG00000132849	1	1	0	1	0	1	1	1	168	156	627	106	0	2	4	3	0	2	2	0	1	0	0	0	145	186	156	443	1	1	0	3
ENSG00000200575	0	0	0	0	0	0	0	0	0	0	1	1	0	0	0	0	0	0	0	0	0	0	0	0	0	0	0	2	0	0	0	0
ENSG00000212360	0	0	0	0	0	0	0	0	0	0	3	0	0	0	0	0	0	0	0	0	0	0	0	0	0	0	0	0	0	0	0	0
ENSG00000236646	0	0	0	0	0	0	0	0	0	0	0	1	0	0	0	0	0	0	0	0	0	0	0	0	2	0	0	0	0	0	0	0
ENSG00000242860	0	0	0	0	0	0	0	0	0	0	0	0	0	0	0	0	0	0	0	0	0	0	0	0	1	0	1	0	0	0	0	0
ENSG00000263908	0	0	0	0	0	0	0	0	0	0	0	0	0	0	0	0	0	0	0	0	0	0	0	0	0	0	0	1	0	0	0	0
ENSG00000283690	0	0	0	0	0	0	0	0	0	0	0	0	0	0	0	0	0	0	0	0	0	0	0	0	0	0	0	0	0	0	0	0
ENSG00000234204	0	0	0	0	0	0	0	0	0	0	0	0	0	0	0	0	0	0	0	0	0	0	0	0	0	0	0	0	0	0	0	0
ENSG00000234088	0	0	1	0	0	0	0	0	0	0	0	0	0	0	0	0	0	0	0	0	0	0	0	0	0	0	0	0	0	0	0	0
ENSG00000240563	0	0	0	0	0	0	0	0	0	0	1	0	0	0	0	0	0	0	0	0	1	0	0	0	0	0	0	0	0	0	0	0
ENSG00000237227	0	0	0	0	0	0	0	0	0	0	0	0	0	0	0	0	0	0	0	0	0	0	0	0	0	0	0	0	0	0	0	0
ENSG00000200174	0	0	0	0	0	0	0	0	0	0	0	0	0	0	0	0	0	0	0	0	0	0	0	0	0	0	0	0	0	0	0	0
ENSG00000132854	8	30	18	39	0	0	0	0	1	2	11	0	0	0	0	0	4	28	5	43	0	0	0	1	2	5	5	13	0	2	0	0
ENSG00000201153	0	0	0	0	0	0	0	0	0	0	0	0	0	0	0	0	0	0	0	0	1	0	0	0	0	0	0	0	0	0	0	0
ENSG00000162607	6870	4766	5972	4961	3644	3823	3254	4488	4745	3633	12287	2591	2879	2901	3229	3868	4021	6215	5984	7225	9425	3746	7462	3704	4732	6711	2187	10830	2888	3748	3433	4067
ENSG00000116641	1268	1084	1101	1070	1967	2119	1376	2287	786	695	2731	466	673	777	674	914	376	728	581	973	2655	1081	1890	1015	738	1103	622	1991	447	711	536	759
ENSG00000270549	1	0	0	0	1	2	0	6	2	1	3	1	1	1	0	0	3	0	0	2	3	1	1	1	0	1	1	1	0	0	0	0
ENSG00000132855	10	15	9	10	21	11	8	18	4	0	13	3	1	6	3	2	7	1	4	13	13	13	12	20	9	9	5	20	2	4	1	2
ENSG00000278967	16	12	7	14	14	12	7	12	6	5	8	1	3	3	0	3	2	3	0	6	12	4	5	7	1	2	1	11	0	1	1	5
ENSG00000213703	1	0	1	3	0	5	1	4	0	1	0	0	0	1	0	2	0	0	0	0	5	0	0	2	1	0	1	2	0	0	0	0
ENSG00000235545	14	8	15	18	13	8	4	11	5	8	26	2	4	2	1	8	2	9	3	11	15	4	2	6	4	6	8	18	0	2	3	0
ENSG00000125703	759	604	665	625	448	518	375	584	401	407	1229	220	349	328	353	469	861	1203	1071	988	1066	433	786	462	357	465	246	790	393	410	461	507
ENSG00000234318	4	0	0	2	1	0	0	0	0	0	1	1	3	0	0	1	1	1	2	2	0	1	0	0	0	1	1	1	1	0	3	0
ENSG00000237163	1	0	0	0	0	0	0	0	0	0	0	0	0	0	0	0	0	0	0	0	0	1	1	2	0	0	0	0	0	0	0	0
ENSG00000229537	0	0	0	0	0	0	0	0	0	0	0	0	0	0	0	0	0	0	0	0	0	0	0	0	0	0	0	0	0	0	0	0
ENSG00000227485	0	0	0	0	0	0	0	0	0	0	0	0	0	0	0	0	0	0	0	0	0	0	0	0	0	0	0	0	0	0	0	0
ENSG00000229225	0	0	0	0	0	0	0	0	0	0	0	0	0	0	0	0	0	0	0	0	0	0	0	0	0	0	0	0	0	0	0	0
ENSG00000203605	0	0	0	0	0	0	0	0	0	0	0	0	0	0	0	0	0	1	0	0	1	0	0	0	0	0	0	0	0	0	0	0
ENSG00000224209	0	1	1	2	1	0	1	0	2	1	2	0	2	2	1	2	0	0	0	3	3	0	2	0	1	0	1	1	1	1	0	3
ENSG00000228734	0	0	0	0	0	0	0	0	0	0	1	0	0	1	0	0	0	0	0	0	0	1	2	0	0	1	0	0	0	0	0	0
ENSG00000223683	0	1	0	0	0	0	1	0	0	0	2	0	0	0	0	0	0	0	0	0	0	0	0	0	0	0	0	0	0	0	0	0
ENSG00000230798	23	21	34	37	0	0	0	0	0	0	4	0	47	50	39	73	9	11	12	26	1	0	0	0	0	1	3	4	38	36	27	47
ENSG00000187140	9	29	22	23	0	0	0	0	1	1	9	2	67	72	56	71	3	8	14	14	0	0	0	0	1	2	1	1	34	54	47	63
ENSG00000275836	0	0	0	0	0	0	0	0	0	0	0	0	0	0	0	0	0	0	0	0	0	0	0	0	0	0	0	0	0	0	0	0
ENSG00000252784	0	0	0	0	0	0	0	0	0	0	0	0	0	0	0	0	0	0	0	0	0	0	0	0	0	0	0	0	0	0	0	0
ENSG00000236674	1	0	2	0	0	0	0	0	0	0	0	0	0	0	0	1	0	0	0	2	0	0	0	0	0	0	0	0	0	0	0	0
ENSG00000088035	1029	700	766	682	522	632	425	642	650	518	1937	367	363	396	392	530	904	1284	1047	1304	1305	599	864	591	520	750	442	1334	303	553	432	615
ENSG00000142856	1580	1177	1224	1097	1215	1291	1025	1408	1081	950	2735	593	843	856	933	1236	897	1098	998	1494	2418	1091	1948	1436	854	1264	446	2169	712	889	766	1113
ENSG00000203965	518	367	441	317	288	291	231	373	185	216	665	138	289	283	267	348	360	384	398	500	537	248	417	266	203	248	167	465	222	332	306	378
ENSG00000264271	1	0	2	0	0	0	0	0	1	0	3	1	1	0	1	1	0	1	0	0	0	0	1	1	0	2	0	3	0	0	0	2
ENSG00000251720	2	0	5	10	1	7	4	14	2	10	10	2	3	1	7	5	1	2	1	1	14	3	3	7	5	2	5	15	4	6	6	7
ENSG00000116652	157	93	138	113	63	69	53	59	47	61	176	29	60	44	44	73	19	30	17	68	79	29	51	40	26	44	44	120	27	70	30	51
ENSG00000276884	0	0	0	0	0	0	0	0	0	0	0	0	0	0	0	0	0	0	0	0	0	0	0	0	0	0	0	0	0	0	0	0
ENSG00000278287	0	0	0	0	0	0	0	0	0	0	0	0	0	0	0	0	0	0	0	0	0	0	0	0	0	0	0	0	0	0	0	0
ENSG00000277565	0	0	0	0	0	0	0	0	0	0	0	0	0	0	0	0	0	0	0	0	0	0	0	0	0	0	0	0	0	0	0	0
ENSG00000275823	0	0	0	0	0	0	0	0	0	0	0	0	0	0	0	0	0	0	0	0	0	0	0	0	0	0	0	0	0	0	0	0
ENSG00000277689	0	0	0	0	0	0	0	0	0	0	0	0	0	0	0	0	0	0	0	0	0	0	0	0	0	0	0	0	0	0	0	0
ENSG00000274199	0	0	0	0	0	0	0	0	0	0	0	0	0	0	0	0	0	0	0	0	0	0	0	0	0	0	0	0	0	0	0	0
ENSG00000079739	1275	1091	1173	1128	3588	3952	2618	4182	1566	1195	3547	841	851	960	832	1150	323	597	406	775	3257	1399	2606	1210	1020	1624	720	2569	476	591	459	632
ENSG00000244256	3	3	4	1	9	8	10	8	9	8	20	6	3	6	2	6	1	0	1	5	12	8	8	7	7	1	7	19	1	1	2	2
ENSG00000185483	1	12	2	11	18	15	17	28	35	58	211	39	3	2	12	6	3	10	6	12	40	20	37	36	34	66	110	158	2	2	3	9
ENSG00000177452	0	0	0	0	0	0	0	0	0	0	2	0	0	0	0	0	0	0	0	0	0	0	0	0	1	0	0	5	0	0	0	0
ENSG00000228056	0	0	0	0	0	0	0	0	0	0	1	1	0	0	0	0	0	0	0	0	0	0	0	1	1	0	0	1	0	0	0	0
ENSG00000207190	0	0	0	0	0	0	0	0	0	0	0	0	0	0	0	0	0	0	0	0	0	0	0	0	0	0	0	0	0	0	0	0
ENSG00000200508	0	0	0	0	0	0	0	0	0	0	0	0	0	0	0	0	0	0	0	0	0	0	0	2	0	0	0	0	0	0	0	0
ENSG00000223949	0	0	0	0	0	0	1	3	5	0	7	0	0	1	2	0	0	0	0	1	5	2	1	1	0	1	10	5	1	0	1	0
ENSG00000177414	0	0	0	0	0	0	0	1	0	0	0	0	0	0	0	0	0	0	0	0	0	0	0	0	0	0	1	0	0	0	0	0
ENSG00000238653	0	0	0	0	0	0	0	0	0	0	0	0	0	0	0	0	0	0	0	0	0	0	0	0	0	0	0	0	0	0	0	0
ENSG00000158966	53	154	63	131	868	935	680	876	316	288	880	193	131	168	120	216	20	106	49	162	1491	645	992	545	204	402	214	637	43	171	61	160
ENSG00000264470	0	0	0	0	0	1	0	1	1	0	0	0	0	0	0	0	0	0	0	0	1	1	0	0	0	0	0	0	0	0	0	0
ENSG00000162437	765	952	742	956	1585	1631	1247	1608	1278	1208	3790	766	627	722	515	897	370	816	529	1151	3204	1251	2000	1257	1266	1811	913	2927	443	872	480	1002
ENSG00000162434	3121	2623	2528	2457	5731	5897	4176	6075	3722	3413	16744	2735	2900	3075	2623	3822	961	1684	1323	1645	10276	3948	7032	3358	3486	5310	5446	11283	1881	2780	2162	2993
ENSG00000234784	2	0	0	0	2	3	6	1	2	2	5	0	1	2	0	1	0	0	0	0	10	2	1	7	1	1	0	4	2	1	2	1
ENSG00000233877	2	1	3	3	4	3	1	10	3	2	5	0	2	2	4	0	0	0	1	3	8	2	1	6	7	5	5	16	0	0	4	4
ENSG00000226891	12	7	6	17	20	14	20	28	24	11	36	7	10	15	1	13	4	5	0	4	50	26	23	35	7	8	14	39	2	7	5	14
ENSG00000185031	1	2	2	4	7	11	4	4	1	0	12	5	3	2	1	4	1	0	0	4	7	4	8	2	2	0	5	9	0	3	1	4
ENSG00000272506	0	1	2	0	4	2	4	5	3	5	8	0	1	1	0	0	0	0	1	2	7	8	4	1	2	2	2	8	0	1	0	2
ENSG00000212257	0	1	0	0	0	1	0	3	0	1	5	0	0	1	0	0	0	0	0	0	1	0	0	0	0	2	2	3	0	2	0	2
ENSG00000265996	4	3	4	0	10	5	8	16	6	9	26	5	6	6	4	11	4	0	0	5	33	16	9	10	11	8	2	16	1	5	3	6
ENSG00000199135	0	0	0	0	0	2	0	3	0	0	2	1	1	1	0	0	0	0	0	0	3	0	3	3	0	4	0	3	0	0	1	2
ENSG00000231485	6	2	0	1	10	6	13	10	3	1	11	1	4	9	2	3	0	0	0	3	11	5	7	17	0	1	4	4	0	4	5	4
ENSG00000235804	0	0	0	0	0	0	0	0	0	0	0	0	0	0	0	0	0	0	0	0	0	0	0	0	0	0	0	0	0	0	0	0
ENSG00000234496	0	0	0	0	0	0	0	0	0	0	0	0	0	0	0	0	0	0	0	0	0	0	0	0	0	0	0	0	0	0	0	0
ENSG00000162433	521	964	594	899	4650	4591	3484	5272	5948	4853	10797	3153	2234	2282	2135	2785	37	103	21	63	4915	1871	3134	1631	2390	3553	1303	5124	1068	1409	1082	1499
ENSG00000231622	0	2	0	3	6	2	2	10	4	1	3	0	2	3	0	1	0	0	0	0	8	1	0	2	1	2	1	2	0	1	1	1
ENSG00000116675	709	705	563	627	674	642	460	644	188	185	672	125	197	263	198	358	216	407	247	581	970	395	723	378	195	338	235	562	130	244	115	208
ENSG00000229294	0	2	1	8	2	1	1	7	2	0	0	0	3	1	0	0	1	0	0	3	1	0	3	3	1	6	0	5	0	1	2	0
ENSG00000234144	4	0	0	0	4	1	1	0	0	0	0	0	1	1	0	1	1	0	0	1	3	0	1	3	0	0	1	0	0	0	0	1
ENSG00000222624	0	0	0	0	0	0	0	0	0	0	0	0	0	0	0	0	1	0	0	0	0	0	0	0	0	0	0	1	0	0	0	0
ENSG00000213625	501	360	423	325	1136	1108	877	1198	716	642	3415	456	457	497	524	661	195	295	207	383	1951	915	1346	836	610	933	876	1837	344	560	440	649
ENSG00000116678	145	126	93	108	205	254	129	242	59	80	247	44	38	61	34	59	55	100	64	69	353	135	181	150	78	84	60	133	20	34	13	25
ENSG00000237852	2	1	0	0	3	1	0	2	1	2	3	2	1	2	0	2	0	0	2	0	6	3	6	2	1	1	2	2	0	1	0	0
ENSG00000224570	0	0	0	0	0	2	0	0	0	0	0	0	0	0	0	0	0	0	0	0	0	0	0	0	0	0	0	1	0	1	0	1
ENSG00000239319	0	0	0	0	0	0	0	0	0	0	2	0	0	0	0	0	0	0	0	0	0	0	0	0	1	1	1	0	0	0	0	0
ENSG00000184588	0	0	0	0	319	484	308	541	886	773	3768	525	1283	1195	1376	1460	0	2	0	1	469	275	286	228	1152	1429	1474	2426	1187	1574	1504	1690
ENSG00000227466	0	0	0	0	0	0	0	0	0	0	0	0	0	0	0	0	0	0	0	0	0	1	1	0	0	0	0	0	0	0	0	0
ENSG00000223152	0	0	0	0	0	0	0	0	0	0	0	0	0	0	0	0	0	0	0	0	0	0	0	0	0	0	0	0	0	0	0	0
ENSG00000118473	0	1	0	0	12	18	23	24	0	0	12	0	3	3	2	3	0	0	6	5	34	10	29	10	0	1	8	2	1	9	0	0
ENSG00000264720	0	0	0	0	0	0	0	0	0	0	0	0	0	0	0	0	0	0	0	0	0	0	0	0	0	0	0	0	0	0	0	0
ENSG00000248458	0	0	0	0	0	0	1	0	0	0	2	0	0	0	0	0	0	0	0	0	0	0	0	0	0	0	0	0	0	0	0	0
ENSG00000152760	0	0	0	0	0	0	0	1	0	0	0	0	2	2	3	3	0	0	0	0	1	0	0	0	0	0	0	1	0	1	3	0
ENSG00000172410	0	0	0	0	0	0	0	0	0	0	0	0	0	2	0	0	0	0	0	0	0	0	0	0	0	0	1	0	0	0	0	1
ENSG00000152763	42	40	50	40	7	12	5	16	22	37	151	25	86	96	88	116	27	56	35	42	21	6	14	8	22	42	27	72	30	90	66	89
ENSG00000231080	0	0	2	0	0	0	0	0	0	0	1	0	0	0	1	1	0	0	0	0	0	0	0	0	0	0	0	1	0	0	0	0
ENSG00000198160	2317	1587	1820	1574	1500	1544	1230	1820	1578	1391	6343	1117	1309	1142	1301	1557	2016	2265	1806	2494	4144	1615	2705	1856	1997	2659	1631	4896	1131	1425	1312	1696
ENSG00000116704	831	631	779	640	1427	1465	1112	1523	1837	1538	10996	1244	686	654	831	864	348	525	481	791	2855	1194	2038	1123	2386	3563	4068	7857	842	1154	1056	1236
ENSG00000203963	0	0	0	0	0	0	0	0	0	0	6	0	0	2	0	1	0	0	0	0	1	0	0	0	0	0	0	0	0	0	0	0
ENSG00000252433	0	0	0	0	0	0	0	0	0	0	0	0	0	0	0	0	0	0	0	0	0	0	0	0	0	0	0	0	0	0	0	0
ENSG00000275678	2	0	0	1	0	0	0	0	0	1	1	0	0	0	0	0	0	0	0	0	0	0	0	1	2	0	2	0	1	0	1	0
ENSG00000162594	4	2	1	6	0	0	0	0	0	1	2	0	1	1	2	1	1	0	0	2	0	0	0	0	0	3	3	6	2	0	1	0
ENSG00000253074	0	0	0	0	0	0	0	0	0	0	0	0	0	0	0	0	0	0	0	0	0	0	0	0	1	0	0	0	0	0	0	0
ENSG00000252116	0	0	0	0	0	0	0	1	0	0	0	0	0	0	0	0	0	0	0	0	4	0	3	2	0	0	0	0	0	0	0	0
ENSG00000228523	0	0	0	0	2	4	5	3	0	0	0	0	0	0	0	0	0	1	0	0	4	2	3	3	0	0	1	0	0	0	0	0
ENSG00000081985	16	3	10	4	65	75	55	73	2816	2787	13591	1924	22	26	30	23	1	2	10	8	123	49	92	45	1938	3953	2792	7065	7	16	15	28
ENSG00000142864	14532	9868	11725	10455	18455	19012	15468	20443	16356	12513	47011	9275	21077	18625	21889	24234	11722	18003	14824	13951	43858	16782	33307	15538	16282	24206	9172	33381	21490	21798	23607	24539
ENSG00000223263	10	6	6	8	12	13	7	4	13	16	33	6	10	18	6	7	3	2	7	11	21	13	20	13	13	14	3	25	7	13	10	9
ENSG00000235200	0	0	0	0	0	0	0	0	0	0	0	0	0	0	0	0	0	0	0	0	0	0	0	0	0	0	0	0	0	0	0	0
ENSG00000207500	0	0	0	0	0	0	0	0	0	0	0	0	0	0	0	0	0	0	0	0	0	0	0	0	0	0	0	0	0	0	0	0
ENSG00000238139	0	0	0	0	0	0	0	0	0	0	0	0	0	0	0	0	0	0	0	0	0	0	0	0	0	0	0	0	0	0	0	0
ENSG00000242482	0	0	0	0	0	0	0	0	0	0	0	0	0	0	0	0	0	0	0	0	0	0	0	0	0	0	0	0	0	0	0	0
ENSG00000232048	0	0	0	0	0	0	0	0	0	0	2	0	0	0	0	0	0	0	0	0	0	0	0	0	0	0	3	2	0	0	0	0
ENSG00000116717	237	261	180	323	1142	1024	911	1170	1261	890	9752	1258	218	325	416	469	58	166	61	164	1826	771	1459	755	950	1396	1921	4499	171	235	227	293
ENSG00000172380	0	1	0	0	0	0	0	0	5	14	47	25	0	1	0	1	0	0	0	1	0	1	0	1	9	35	16	85	0	0	0	0
ENSG00000238778	0	0	0	0	0	0	0	0	0	0	0	0	0	0	0	0	0	0	0	0	0	0	0	0	0	0	0	0	0	0	0	0
ENSG00000232284	0	0	0	0	0	0	0	0	2	4	14	2	0	0	0	0	0	0	0	0	0	0	1	0	2	4	1	7	0	0	0	0
ENSG00000235055	0	0	0	0	0	0	0	0	0	0	1	0	0	0	0	0	0	0	0	0	0	0	0	0	0	0	0	0	0	0	0	0
ENSG00000162595	0	1	0	1	0	0	0	0	0	0	2	2	0	0	0	0	0	3	0	2	0	0	0	0	0	0	2	2	0	0	0	0
ENSG00000228431	0	0	0	0	0	0	0	0	0	0	0	0	0	0	0	0	0	0	0	0	0	0	0	0	1	0	1	0	0	0	0	0
ENSG00000116729	0	2	0	0	6	3	6	2	107	104	401	93	0	0	0	0	0	0	0	0	14	2	5	3	52	108	47	291	0	0	0	0
ENSG00000234383	0	0	0	0	0	0	0	0	0	0	2	4	0	0	0	0	0	0	0	0	0	0	1	0	0	0	3	2	0	0	0	0
ENSG00000221203	0	0	0	0	0	0	0	0	0	0	2	0	0	0	0	0	0	0	0	0	0	0	0	0	1	1	0	0	0	0	0	0
ENSG00000229133	0	0	0	0	0	0	0	0	0	0	2	0	0	0	0	0	0	0	0	0	1	1	0	1	0	0	0	0	0	2	0	0
ENSG00000225242	0	0	0	0	0	0	0	0	0	0	0	0	0	0	0	0	0	0	0	0	0	0	0	0	0	0	0	0	0	0	0	0
ENSG00000237622	0	0	0	0	0	0	0	0	0	0	0	0	0	0	0	0	0	0	0	0	0	0	0	0	0	0	0	0	0	0	0	0
ENSG00000229440	0	0	0	0	0	0	0	0	0	0	0	0	0	0	0	0	0	0	0	0	0	0	0	0	0	0	0	0	0	0	0	0

ENSG00000116745	0	0	0	0	0	0	0	0	0	2	1	0	0	0	0	0	0	0	0	0	0	0	0	0	0	0	0	0	0	0	0	0
ENSG00000024526	1004	818	884	779	2177	2428	1396	2243	1423	1092	4054	756	1266	1299	1457	1681	672	890	772	834	3842	1474	2481	1466	1563	2159	451	3111	1031	1365	1171	1507
ENSG00000233589	39	22	29	19	60	83	30	72	24	14	62	14	42	48	42	41	17	12	7	15	108	57	49	40	42	33	13	56	15	23	24	45
ENSG00000234264	0	0	2	0	1	3	1	2	11	4	34	5	2	4	12	3	2	2	4	4	5	7	8	5	8	7	8	11	4	5	15	11
ENSG00000225167	0	0	0	0	0	0	0	0	0	0	0	0	0	0	0	0	0	0	0	0	0	0	0	0	0	1	0	0	0	0	1	0
ENSG00000223883	0	0	0	0	0	0	0	0	0	0	32	0	0	0	0	0	0	0	0	0	0	0	0	0	0	0	11	12	0	0	0	0
ENSG00000235446	0	0	0	0	0	0	0	0	0	0	1	0	0	0	0	0	0	0	0	0	0	0	0	0	0	0	0	0	2	0	0	0
ENSG00000229639	0	0	0	0	0	0	0	0	0	0	2	0	0	0	0	0	0	0	0	0	0	0	0	0	0	0	0	2	0	0	0	0
ENSG00000033122	136	89	88	91	57	51	59	67	67	52	234	39	56	57	66	94	28	28	18	61	90	39	55	32	47	75	43	111	14	52	39	58
ENSG00000270615	0	0	0	0	0	0	0	0	0	0	0	0	0	0	0	0	0	0	0	0	0	0	0	0	0	0	0	0	0	0	0	0
ENSG00000240692	0	0	0	0	0	0	0	0	0	0	0	0	0	0	0	0	0	0	0	0	0	0	0	0	0	0	0	0	0	0	0	0
ENSG00000229359	0	0	0	0	0	0	0	0	0	0	0	0	0	0	0	0	0	0	0	0	0	0	0	0	0	0	0	0	0	0	0	0
ENSG00000237919	0	0	0	0	0	0	0	0	0	0	0	0	0	0	0	0	0	0	0	0	0	2	2	1	2	0	0	0	0	0	0	0
ENSG00000066557	1274	848	921	808	1076	1124	877	1268	979	808	2821	482	719	698	877	912	1346	1745	1327	1393	3146	1189	2306	1507	1023	1384	588	2150	727	846	1003	1051
ENSG00000244389	3	0	0	0	0	0	2	0	0	0	1	0	0	0	0	1	0	0	0	4	1	1	1	0	0	0	0	1	0	1	1	2
ENSG00000116754	12169	8104	10568	8430	12517	13004	10124	13750	7748	6508	28278	4706	9031	8195	9180	10460	5271	4986	4950	9699	20778	9411	13078	10031	6256	9164	6714	16860	4970	8111	5746	9306
ENSG00000228988	7	5	2	6	1	4	7	7	2	0	7	0	1	3	2	2	0	0	0	3	3	2	4	4	3	3	5	15	2	1	4	0
ENSG00000118454	706	547	609	494	1119	1102	852	1073	1059	878	6626	848	742	638	800	789	256	391	367	530	1622	632	1145	630	1315	1889	1967	4191	598	697	757	818
ENSG00000197568	181	140	137	144	73	85	39	83	118	113	843	106	182	148	160	211	65	89	98	125	78	33	66	29	127	158	218	445	95	134	111	151
ENSG00000226088	6	9	10	4	30	13	18	25	30	10	230	24	29	20	36	22	1	2	0	5	11	2	11	3	16	37	46	88	16	18	15	18
ENSG00000279230	5	5	3	7	2	2	2	2	4	2	32	3	2	0	1	2	2	0	0	2	0	0	1	3	0	5	11	19	0	0	1	7
ENSG00000116761	200	160	142	155	501	486	327	499	136	126	758	103	350	239	275	339	128	173	129	158	637	169	358	180	118	234	184	505	143	141	277	179
ENSG00000278636	0	1	0	0	0	0	1	0	0	0	2	0	0	1	1	1	0	0	0	0	0	1	0	0	0	1	0	0	0	0	0	0
ENSG00000271992	6	3	8	7	25	15	13	13	0	7	22	3	22	15	14	16	2	3	3	5	10	7	6	2	6	7	7	15	4	6	7	6
ENSG00000233020	0	0	0	0	0	0	0	0	0	0	0	0	0	1	0	0	0	0	0	0	0	0	0	0	0	0	0	0	0	0	0	0
ENSG00000237033	0	0	0	0	0	0	0	0	0	0	0	0	0	0	0	0	0	0	0	0	0	0	0	0	0	0	0	0	0	0	0	0
ENSG00000229051	0	0	0	0	0	0	0	0	0	0	0	0	0	0	0	0	0	0	0	1	0	0	0	0	0	0	1	0	0	0	1	0
ENSG00000226208	0	0	0	0	0	0	0	0	0	0	1	0	0	0	0	0	0	0	0	0	0	0	0	0	0	0	0	0	0	0	0	0
ENSG00000050628	43	59	37	54	1004	1116	772	1129	4	8	258	29	14	15	23	31	11	20	14	53	1895	772	1602	823	62	106	189	506	6	7	4	12
ENSG00000235782	0	0	0	0	0	0	0	0	0	0	3	0	0	0	1	0	0	0	0	0	0	0	2	1	0	0	0	3	0	0	0	0
ENSG00000269933	0	0	0	1	8	5	6	7	0	0	1	1	0	0	1	1	0	1	0	0	13	8	15	5	0	0	1	6	0	0	0	0
ENSG00000235079	15	3	12	10	15	8	13	21	17	14	33	10	5	8	10	6	7	0	7	18	29	15	22	15	16	14	15	37	5	8	2	13
ENSG00000132485	7610	4942	6106	4739	6419	7094	5652	7484	4860	3967	17569	2894	4303	3829	4725	5001	6075	6926	6470	7013	15435	6226	10783	7333	4423	6008	3786	10248	3672	4291	4852	5101
ENSG00000207721	27	12	29	16	39	46	45	65	51	35	176	25	12	25	23	23	9	5	8	26	98	50	49	39	25	27	37	89	7	14	6	20
ENSG00000229956	20	12	17	19	12	35	18	40	17	7	67	7	13	17	19	18	8	12	18	26	45	28	56	22	19	20	20	31	6	12	13	15
ENSG00000226324	0	0	0	1	0	0	0	0	0	0	4	0	0	0	0	0	0	0	0	0	0	0	0	0	0	0	0	0	0	0	0	1
ENSG00000172260	39	48	29	38	349	447	349	477	98	51	107	32	0	0	0	0	23	62	35	77	477	207	414	202	49	49	22	70	0	0	0	0
ENSG00000231985	0	0	1	0	0	0	0	1	0	0	0	0	0	0	0	0	0	0	0	0	2	0	2	0	0	1	0	0	0	0	0	0
ENSG00000271618	0	0	0	0	0	0	0	0	0	0	0	0	0	0	0	0	0	0	0	0	0	0	0	0	0	0	0	0	0	0	0	0
ENSG00000228853	0	0	0	0	1	0	0	0	0	0	0	0	0	0	0	0	0	0	0	0	2	0	0	0	0	0	0	0	0	0	0	0
ENSG00000233994	2	3	2	0	5	5	4	7	2	2	2	0	0	0	1	2	2	1	2	3	4	4	9	6	3	2	0	3	1	2	4	0
ENSG00000227207	0	0	0	0	0	0	0	0	0	0	0	0	0	0	0	0	0	0	0	0	0	0	0	0	0	0	0	0	0	1	0	0
ENSG00000212366	0	0	0	0	0	0	0	0	0	0	0	0	0	0	0	0	0	0	0	0	0	0	0	0	0	0	0	0	0	0	0	0
ENSG00000225087	0	0	0	0	0	0	0	0	0	0	0	0	0	0	0	0	0	0	0	0	1	0	1	0	0	0	0	0	0	0	0	0
ENSG00000227016	0	0	0	0	0	0	0	0	0	0	0	0	0	0	0	0	0	0	0	0	0	0	0	0	0	0	0	0	0	0	0	0
ENSG00000280317	0	0	0	0	0	0	0	0	0	0	1	0	0	0	0	0	0	0	0	0	0	0	0	0	0	0	0	0	0	0	0	0
ENSG00000229636	0	0	0	0	0	0	0	0	0	0	0	0	0	0	0	0	0	0	0	0	0	0	0	0	0	0	0	0	0	0	0	0
ENSG00000251825	0	0	0	0	0	0	0	0	0	0	0	0	0	0	0	1	0	0	0	0	0	0	0	0	0	0	0	0	0	1	0	0
ENSG00000233973	0	3	0	0	0	0	0	0	2	1	10	1	3	2	3	2	0	0	0	0	0	0	0	0	0	1	0	4	1	1	6	2
ENSG00000223479	0	0	0	0	0	0	0	0	0	0	0	0	0	0	0	0	0	0	0	2	0	0	0	0	0	0	0	0	0	0	0	0
ENSG00000252005	0	0	0	0	0	0	0	0	0	0	0	0	0	0	0	0	0	0	0	0	0	0	0	0	0	0	0	0	0	0	0	0
ENSG00000162620	187	123	153	109	23	39	34	39	52	55	181	44	107	101	87	129	157	183	239	214	91	32	85	36	55	50	25	136	102	106	93	131
ENSG00000254685	398	253	308	241	229	279	187	306	193	188	619	126	164	178	151	226	490	687	578	565	666	295	462	315	212	253	150	430	143	210	177	214
ENSG00000259030	32	32	29	26	19	13	9	21	14	12	23	2	9	4	4	8	16	12	8	18	13	9	16	6	9	20	8	15	1	3	2	3
ENSG00000116783	5	4	0	0	0	0	0	0	0	0	2	0	0	0	0	0	1	0	0	0	0	2	0	0	0	0	0	0	2	0	0	0
ENSG00000237324	0	1	2	1	0	3	0	1	0	1	4	0	1	0	0	4	0	2	1	0	1	0	2	0	0	1	1	2	0	0	0	0
ENSG00000233894	0	0	0	0	0	0	0	0	0	0	0	0	0	0	0	0	0	0	0	0	1	0	0	0	0	0	1	0	0	0	0	0
ENSG00000162621	0	0	0	1	0	0	1	0	0	0	2	0	0	0	1	0	0	0	0	0	0	0	0	0	0	0	0	0	0	0	0	0
ENSG00000178965	0	0	0	0	3	4	6	7	9	7	33	3	0	0	1	0	0	0	0	0	3	2	3	0	6	9	2	14	0	0	0	1
ENSG00000234497	0	0	0	0	0	0	0	0	2	0	0	0	0	0	0	0	0	0	0	0	0	0	0	0	0	0	0	0	0	0	0	0
ENSG00000272864	7	6	2	8	7	8	2	4	9	0	17	0	0	0	2	1	1	1	0	3	8	2	5	4	2	5	4	6	0	2	0	1
ENSG00000116791	1483	1018	1045	911	1061	1100	823	1213	931	719	2778	454	217	235	201	289	2207	2566	2115	1746	2669	996	1554	1070	817	1277	632	1791	339	365	351	413
ENSG00000162623	1371	810	992	899	1024	1034	862	1140	1050	814	4029	601	713	736	799	875	1832	2148	2078	1739	2912	1185	2095	1377	1010	1546	954	2486	1002	1036	1322	1205
ENSG00000229943	0	0	0	0	0	0	0	0	0	0	0	0	0	0	0	0	0	0	0	0	0	0	0	0	0	0	0	0	0	0	0	0
ENSG00000261213	0	0	0	0	0	0	0	0	0	0	3	0	0	0	0	0	0	0	0	0	0	0	0	0	0	0	0	0	0	0	0	0
ENSG00000224127	0	0	0	0	0	0	0	0	0	0	0	0	0	0	0	0	0	0	0	0	0	0	0	0	0	0	0	0	0	0	0	0
ENSG00000162624	0	0	0	0	0	0	0	0	0	0	0	0	0	0	0	0	0	0	0	0	0	0	0	0	1	1	0	0	0	0	1	0
ENSG00000224149	0	0	0	0	0	0	0	0	0	0	0	0	0	0	0	0	0	0	0	0	0	0	0	0	0	0	0	0	0	0	0	0
ENSG00000206999	0	0	0	0	0	0	0	0	0	0	0	0	0	0	0	0	0	0	0	0	0	0	0	0	0	0	0	0	0	0	0	0
ENSG00000137968	62	72	46	84	223	221	160	227	24	25	122	13	155	188	184	303	11	28	18	50	417	196	291	164	29	63	51	101	65	184	105	209
ENSG00000224493	0	0	2	0	1	0	0	1	1	0	0	0	0	0	0	0	0	0	0	3	3	0	3	5	0	2	1	1	0	0	0	0
ENSG00000252338	0	0	0	0	0	0	0	0	0	0	1	0	0	0	0	0	0	0	0	0	1	0	0	1	0	0	1	0	0	0	0	0
ENSG00000213579	0	0	0	0	1	1	0	1	1	0	0	0	0	0	0	0	0	0	0	2	1	0	0	1	0	0	0	1	0	0	0	0
ENSG00000230863	0	0	0	0	1	0	1	0	0	0	0	0	1	0	0	0	0	0	0	0	5	1	5	2	0	0	0	0	0	0	0	0
ENSG00000178193	2	1	1	4	3	3	2	2	0	1	7	1	0	1	3	1	5	4	1	5	6	5	2	0	1	0	1	2	0	0	0	0
ENSG00000117054	3918	2446	3210	2457	3846	3661	3118	4064	2415	1811	6727	1297	1196	1218	1550	1621	3856	5617	4807	4134	9487	3797	6758	3904	2198	3085	1560	4732	1567	1688	2086	2030
ENSG00000181227	54	53	60	43	58	59	46	63	38	33	154	29	26	22	32	50	18	11	24	72	94	61	62	47	45	65	25	93	16	18	21	42
ENSG00000137955	3589	2368	2641	2508	1997	2114	1590	2277	2633	2043	10212	1587	2777	2657	3172	3507	3412	4023	2868	4268	4129	1962	2606	2152	2337	3594	2158	5865	2509	3414	3285	4136
ENSG00000206620	0	0	0	0	0	0	0	0	0	0	0	0	0	0	0	0	0	0	0	0	0	0	0	0	0	0	0	0	0	0	0	0
ENSG00000207241	0	0	0	0	0	0	0	0	0	0	0	0	0	0	0	0	0	0	0	0	0	0	0	0	0	0	0	0	0	0	0	0
ENSG00000201487	0	0	0	0	0	0	0	0	0	0	0	0	0	0	0	0	0	0	0	0	0	0	0	0	0	0	0	0	0	0	0	0
ENSG00000057468	114	54	138	83	19	23	17	18	7	4	43	3	3	2	9	7	35	41	54	118	16	12	15	20	11	21	18	35	0	4	7	8
ENSG00000154007	0	2	1	0	0	0	0	0	0	0	2	0	0	0	0	0	0	1	0	1	0	0	0	0	0	0	1	1	0	1	0	0
ENSG00000225605	0	0	1	1	0	0	0	0	0	0	0	0	0	0	0	0	0	0	1	1	0	0	0	0	0	1	0	0	0	0	0	0
ENSG00000230027	0	0	1	0	0	0	0	0	0	0	1	0	0	0	0	0	0	0	0	0	0	0	0	0	3	2	3	3	0	0	0	0
ENSG00000184005	0	0	0	0	0	0	0	0	14	18	41	8	0	0	0	0	0	0	0	0	0	1	2	1	14	27	13	26	0	0	0	0
ENSG00000223905	0	0	0	0	0	0	0	0	0	0	0	1	0	0	0	0	0	0	0	0	0	0	0	0	0	0	0	1	0	0	0	0
ENSG00000272855	0	0	0	0	0	0	0	0	0	0	2	0	0	0	0	0	0	0	0	0	0	0	0	0	1	0	0	2	0	0	0	0
ENSG00000226415	17	12	24	14	10	13	8	8	10	17	32	10	11	7	13	15	7	10	3	6	16	4	15	5	8	15	2	18	14	8	11	7
ENSG00000199921	0	0	0	0	0	0	0	0	0	0	0	0	0	0	0	0	0	0	0	0	0	0	0	0	0	0	0	0	0	0	0	0
ENSG00000237552	1	0	0	0	0	0	0	0	0	0	0	0	0	0	0	0	0	0	0	0	0	0	0	0	0	0	0	0	0	0	0	0
ENSG00000117069	0	0	1	0	0	0	0	0	2	1	10	0	31	26	29	29	0	0	0	0	0	0	0	0	1	0	4	5	17	22	19	42
ENSG00000276081	0	0	0	0	0	0	0	0	0	0	0	0	0	0	0	0	0	0	0	0	0	0	0	0	0	0	0	0	0	0	0	0
ENSG00000230498	0	0	0	0	1	0	1	0	0	0	0	0	0	1	0	0	0	0	0	0	1	0	2	0	0	0	0	0	0	0	2	1
ENSG00000142892	937	682	686	649	1251	1399	1052	1544	480	413	1700	296	716	615	782	845	1127	1583	1283	1436	3666	1552	2808	1774	644	1010	331	1237	872	925	1258	1117
ENSG00000226084	22	14	16	22	17	8	11	20	26	7	82	14	22	24	30	33	25	20	29	30	34	12	22	24	18	27	22	45	38	36	47	35
ENSG00000228187	2	2	1	0	2	1	1	0	1	0	0	0	0	2	0	0	1	0	0	1	7	1	2	2	1	1	0	3	0	0	1	1
ENSG00000154027	1	5	0	2	10	5	7	3	31	19	61	14	2	1	5	2	2	4	1	1	10	5	4	2	26	31	17	56	2	4	4	12
ENSG00000233099	0	0	0	0	0	0	0	0	0	0	0	0	0	0	0	0	0	0	0	0	0	0	0	0	0	0	0	0	0	0	0	0
ENSG00000251767	0	0	0	0	0	0	0	0	0	0	0	0	0	0	0	0	0	0	0	0	0	0	0	0	0	0	0	1	0	0	0	0
ENSG00000036549	1183	823	961	785	1016	1148	920	1242	1262	964	4671	734	1256	1145	1277	1435	894	1033	1037	1313	2500	1118	1852	1125	1323	1839	1242	3235	1105	1341	1412	1470
ENSG00000243437	2	1	1	1	0	2	0	2	1	0	4	1	0	0	2	0	0	1	1	1	0	0	1	1	5	3	3	1	0	2	1	0
ENSG00000077254	1968	1413	1653	1404	1815	1940	1443	2049	1574	1288	6688	1030	1341	1117	1382	1499	854	1069	985	1580	3997	1625	2838	1861	1632	2294	1775	4447	987	1421	1360	1547
ENSG00000232672	0	0	0	0	0	0	0	0	0	0	0	0	0	0	0	0	0	0	0	0	0	0	0	0	0	0	0	0	0	0	0	0
ENSG00000180488	597	428	506	364	348	367	271	368	424	349	1586	261	390	369	323	453	278	391	296	461	535	289	412	267	342	471	430	1008	239	357	279	370
ENSG00000219201	7	4	3	1	27	27	30	43	1	2	12	1	4	3	3	1	1	8	1	6	44	25	41	31	2	2	6	7	1	2	5	3
ENSG00000235613	8	6	6	3	4	4	2	4	11	2	16	2	5	4	7	3	1	0	3	10	12	0	8	7	15	5	10	34	0	11	1	3
ENSG00000232015	6	2	1	0	0	1	1	3	0	0	5	1	4	0	1	4	0	0	0	2	0	0	0	2	2	0	1	5	0	0	3	4
ENSG00000235927	4	3	8	3	6	3	2	3	5	4	20	3	7	6	3	8	1	5	4	6	1	2	0	2	3	6	11	9	2	10	2	3
ENSG00000162614	35	30	45	26	40	42	28	37	274	146	619	116	736	617	807	879	40	44	25	35	81	29	41	47	233	308	68	451	688	877	971	1161
ENSG00000162613	7880	5157	5683	4937	5526	5835	4262	5876	5889	4752	21626	3864	4054	3669	3926	4457	3449	4364	4590	5276	10203	3933	6969	3996	6097	8673	4173	15062	2764	4014	3194	4355
ENSG00000162616	522	412	461	411	237	265	185	280	474	393	1972	270	343	479	423	661	257	344	252	351	428	200	300	207	450	477	583	1153	266	459	316	522
ENSG00000137960	2	0	4	2	0	1	0	2	4	6	29	5	6	8	11	8	1	4	0	2	2	2	1	0	7	3	6	18	2	11	12	14
ENSG00000273338	4	11	8	7	11	8	6	6	21	21	102	7	8	7	10	12	4	1	3	6	12	0	3	6	15	21	24	35	7	12	10	6
ENSG00000213561	2	0	0	0	0	1	0	0	0	0	0	0	0	0	0	2	0	0	0	0	0	0	0	0	1	0	0	1	0	0	0	0
ENSG00000251958	0	0	0	0	0	0	0	0	0	0	0	0	0	0	0	0	0	0	0	0	0	0	0	0	0	0	0	0	0	0	0	0
ENSG00000213560	0	0	0	0	0	0	0	0	0	0	0	0	0	0	0	0	0	0	0	0	0	0	0	0	2	1	0	0	0	0	0	0
ENSG00000233333	0	0	0	0	0	0	0	0	1	0	0	0	2	0	0	0	0	0	0	0	0	0	0	0	0	0	0	1	0	2	0	2
ENSG00000237413	0	0	0	0	0	0	0	0	0	0	1	0	0	0	1	0	0	0	0	0	0	0	0	0	0	0	0	0	0	0	0	0
ENSG00000122420	0	0	0	0	0	0	0	0	2	1	5	0	0	0	0	0	0	3	0	1	0	0	0	0	1	2	3	0	0	0	0	0
ENSG00000227556	0	0	0	0	0	0	0	0	0	0	0	0	0	0	0	0	0	0	0	0	0	0	0	0	0	0	0	0	0	0	0	0
ENSG00000137959	0	4	0	0	6	13	0	6	9	6	53	3	38	79	64	101	0	4	0	2	3	2	2	1	8	9	9	17	15	63	33	74
ENSG00000137965	101	58	98	85	412	447	330	455	410	306	1374	256	295	302	324	396	149	142	176	171	743	326	497	363	394	507	269	992	281	362	414	459
ENSG00000238015	0	0	0	0	2	0	0	1	3	0	6	1	1	0	1	0	0	0	0	0	2	1	0	1	3	0	0	3	0	1	0	3
ENSG00000235400	0	0	0	0	2	0	0	0	0	0	0	0	1	0	0	0	0	0	0	0	0	0	1	0	0	1	0	0	0	1	0	0
ENSG00000162618	0	0	0	0	0	0	0	0	0	0	1	0	5	6	7	8	0	0	0	0	0	0	0	0	0	0	0	0	3	8	10	14
ENSG00000230787	4	0	0	0	0	0	0	0	0	0	0	0	4	6	6	7	0	0	0	0	0	0	0	0	0	1	0	2	0	0	5	5
ENSG00000235011	0	0	0	0	0	0	0	0	0	0	0	0	0	0	0	0	0	0	0	0	0	0	0	0	0	0	0	0	0	0	0	0
ENSG00000282898	0	0	0	0	0	0	0	0	0	0	0	0	0	0	0	0	0	0	0	0	0	0	0	0	0	0	0	0	0	0	0	0
ENSG00000232676	0	0	0	0	0	0	0	0	0	0	0	0	0	0	0	0	0	0	0	0	0	0	0	0	0	0	0	0	0	0	0	0
ENSG00000235756	0	0	0	0	0	0	0	0	0	0	0	0	0	0	0	0	0	0	0	0	0	0	0	0	0	0	0	0	0	0	0	0
ENSG00000260322	0	0	0	0	0	0	0	0	0	0	0	0	0	0	0	0	0	0	0	0	0	0	0	0	0	0	0	0	0	0	0	0
ENSG00000277670	0	0	0	0	0	0	0	0	0	0	0	0	0	0	0	0	0	0	0	0	0	0	0	0	0	0	0	0	0	0	0	0
ENSG00000236407	0	0	0	0	0	0	0	0	0	0	0	0	0	0	0	0	0	0	0	0	0	0	0	0	0	0	0	0	0	0	0	0
ENSG00000225598	0	0	0	0	0	0	0	0	0	0	0	0	0	0	0	0	0	0	0	0	0	0	0	0	0	0	0	0	0	0	0	0
ENSG00000213559	0	0	0	0	0	0	0	0	0	0	0	0	0	0	0	0	0	0	0	0	0	0	0	0	0	0	0	0	0	0	0	0
ENSG00000227062	0	0	0	0	0	0	0	0	0	0	0	0	0	0	0	0	0	0	0	0	0	0	1	0	0	0	0	0	0	0	0	0
ENSG00000234108	0	0	0	0	0	0	0	0	0	0	0	0	0	0	0	0	0	0	0	0	0	0	0	0	0	0	0	0	0	0	0	0
ENSG00000224326	0	0	0	0	0	0	0	0	0	0	0	0	0	0	0	0	0	0	0	0	0	0	0	0	0	0	0	0	0	0	0	0
ENSG00000234184	0	0	0	0	0	0	0	0	0	0	1	0	0	0	0	0	0	0	0	0	0	0	0	0	0	0	0	0	0	0	0	0
ENSG00000227937	0	0	0	0	0	0	0	0	0	0	0	0	0	0	0	0	0	0	0	0	0	0	0	0	0	0	0	0	0	0	0	0
ENSG00000229752	0	0	0	0	0	0	0	0	1	0	0	0	0	0	0	0	0	0	0	0	0	0	0	0	0	0	0	0	0	0	0	0
ENSG00000235089	0	0	0	0	0	0	0	0	0	0	0	0	0	0	0	0	0	0	0	0	0	0	0	0	0	0	0	0	0	0	0	0
ENSG00000223026	0	0	0	0	0	0	0	0	0	0	0	0	0	0	0	0	0	0	0	0	0	0	0	0	0	0	0	0	0	0	0	0
ENSG00000117114	0	0	0	1	0	0	0	0	1739	1659	7009	1198	1629	1876	1661	2231	0	1	1	1	4	0	0	1	1834	2560	1825	4792	2217	3047	2392	3241
ENSG00000224494	0	0	0	0	0	0	0	0	0	0	0	0	0	1	0	1	0	0	0	0	0	0	0	0	0	0	0	0	0	0	1	0
ENSG00000227288	0	0	1	1	0	1	0	0	1	0	0	0	0	0	1	0	1	0	0	0	0	3	0	0	0	0	0	3	0	0	0	0
ENSG00000227960	0	0	0	0	0	0	0	0	0	0	0	0	0	0	0	0	0	0	0	0	0	0	0	0	0	0	0	0	0	0	0	0
ENSG00000234953	0	0	0	0	0	0	0	0	0	0	0	0	0	0	0	0	0	0	0	0	0	0	0	0	0	0	0	0	0	0	0	1
ENSG00000242598	0	0	0	0	0	0	0	0	0	0	0	0	0	0	0	0	0	0	0	0	0	0	0	0	0	0	0	0	0	0	0	0
ENSG00000236676	0	0	0	0	0	0	0	0	0	0	0	0	1	0	3	0	0	0	0	0	0	0	0	0	0	0	0	0	0	0	1	0
ENSG00000234683	0	0	0	0	0	0	0	0	0	0	0	0	0	0	0	0	0	0	0	0	0	0	0	0	0	0	0	0	0	0	0	0
ENSG00000215875	0	0	0	0	0	0	0	0	0	0	0	0	0	0	0	0	0	0	0	0	0	0	0	0	0	0	0	0	0	0	0	0
ENSG00000233290	0	0	1	0	0	0	0	0	1	0	0	0	165	173	160	237	0	0	0	0	0	0	0	0	0	0	0	0	161	160	231	207
ENSG00000230817	1	0	0	0	0	0	0	0	0	0	0	0	1	0	0	0	0	0	0	0	0	0	0	0	0	0	0	0	0	0	0	0
ENSG00000236268	0	0	0	0	0	0	0	0	0	0	0	0	0	0	0	0	0	1	0	0	0	0	0	0	0	1	0	0	0	1	0	0
ENSG00000231364	0	0	0	0	0	0	0	0	0	0	0	0	0	1	0	1	0	0	0	0	0	0	0	0	1	3	0	0	0	0	0	1
ENSG00000233008	0	0	0	0	0	0	0	0	4	1	8	1	10	9	7	7	0	0	0	0	0	0	0	0	3	0	6	4	5	4	1	5
ENSG00000237076	0	0	0	0	0	0	0	0	0	0	2	0	0	1	5	0	0	0	0	0	0	0	0	0	1	0	1	1	0	1	0	0
ENSG00000137941	21	58	23	56	0	0	0	0	391	442	1614	278	73	105	75	176	9	19	6	40	0	0	0	0	383	516	441	1238	42	114	32	107
ENSG00000233061	0	2	0	2	0	0	0	0	5	3	13	2	3	1	0	2	0	0	0	0	0	2	0	0	4	3	4	16	1	1	1	4
ENSG00000229486	0	0	0	0	0	0	0	0	0	0	0	0	0	0	0	0	0	0	0	0	0	1	0	1	0	0	0	0	0	0	1	0
ENSG00000271576	153	108	97	107	124	141	86	121	57	47	126	30	37	36	38	68	47	96	73	93	157	60	90	86	29	48	17	73	20	37	22	29
ENSG00000142875	1560	994	1458	967	2946	3038	2626	3495	1993	1451	7864	1064	1930	2001	2056	2489	1428	1556	1862	1710	9647	3822	7164	4802	2000	2309	2095	4307	3098	3701	3903	4033
ENSG00000213540	6	4	5	2	3	6	1	2	1	1	8	0	3	1	1	0	1	3	2	5	5	4	6	7	6	6	3	13	0	3	3	7
ENSG00000226970	1	1	0	4	1	0	0	2	1	1	1	0	0	0	0	0	0	1	1	0	10	1	1	1	1	0	2	2	0	0	3	0
ENSG00000199959	0	0	1	0	0	1	0	0	0	0	0	0	0	0	0	0	0	0	0	2	1	0	0	0	0	0	0	0	0	1	0	1
ENSG00000203943	122	86	79	72	134	94	95	155	23	18	80	11	160	138	133	182	101	108	90	97	181	74	147	67	24	21	13	27	173	153	147	149
ENSG00000249237	1	0	1	0	0	1	2	3	0	0	0	0	4	2	4	8	0	1	1	0	3	0	0	2	0	0	0	0	6	1	3	4
ENSG00000240520	5	2	9	5	5	4	4	4	0	3	16	1	3	4	2	3	2	8	0	10	1	3	2	6	4	0	1	6	2	3	2	4
ENSG00000137976	4	2	1	0	0	2	0	0	0	0	0	0	0	0	4	4	1	1	3	3	1	0	0	0	0	0	0	0	0	0	1	0
ENSG00000117133	1770	1108	1373	1205	1273	1248	1023	1291	899	650	2783	493	1312	1319	1498	1616	2037	2776	2365	1989	3299	1321	2459	1364	891	1317	624	1842	1875	1891	2360	2128
ENSG00000174021	1786	1292	1605	1420	1917	1826	1430	2011	871	673	2807	636	930	808	953	1075	2410	2610	2756	2468	4711	1766	3096	2382	871	1233	577	1958	1011	910	1097	1289
ENSG00000122432	67	36	43	33	38	42	26	44	52	28	163	18	20	25	22	39	15	19	15	46	47	32	25	20	38	40	33	99	7	31	23	25
ENSG00000117151	574	401	421	382	635	659	388	665	316	313	1382	197	445	442	411	533	440	579	499	540	1293	551	802	528	338	356	343	792	330	388	435	481
ENSG00000180869	0	1	0	0	0	0	0	0	0	0	0	0	0	0	0	0	0	0	0	0	1	0	1	0	0	0	0	1	0	1	0	0
ENSG00000232622	0	1	0	0	0	0	0	1	0	0	0	0	1	0	0	0	0	0	0	0	0	0	0	0	0	0	0	1	0	2	0	0
ENSG00000117155	998	848	868	743	590	624	495	696	608	523	2104	394	707	712	792	989	735	1136	876	1176	1318	566	986	652	697	954	408	1641	573	812	761	967
ENSG00000171517	0	0	0	0	0	0	0	0	1902	1828	6279	1294	2	2	1	5	0	1	0	1	0	0	1	0	1501	2624	1228	4680	2	3	2	2
ENSG00000153898	269	268	247	290	0	0	0	0	902	720	2670	444	1260	1066	1202	1353	71	202	83	319	0	0	1	0	564	923	376	1397	629	985	784	1150
ENSG00000162643	39	37	35	29	0	1	1	0	6	11	81	10	12	16	9	11	20	47	16	18	0	0	0	0	3	13	25	23	2	5	10	15
ENSG00000055732	34	32	25	23	0	0	2	0	238	254	987	163	99	173	160	216	5	4	1	33	0	0	0	0	149	299	312	933	65	315	116	311
ENSG00000266110	0	0	1	2	0	0	0	0	0	0	0	0	0	0	0	0	0	0	0	0	0	0	0	0	0	0	0	0	0	0	0	0
ENSG00000280099	84	79	60	74	0	0	0	0	31	19	61	11	68	41	36	77	22	44	9	50	0	0	0	0	33	23	13	26	34	53	27	48
ENSG00000097096	522	404	394	484	0	0	0	0	122	136	385	87	329	341	257	403	146	222	141	376	0	0	0	0	117	224	75	337	171	281	211	271
ENSG00000162642	1090	666	834	695	722	786	539	827	544	452	1917	340	625	616	702	747	1054	1123	1122	1001	1751	642	1225	688	497	717	502	1158	677	763	931	928
ENSG00000223254	3	2	4	1	5	3	2	4	1	0	2	0	1	2	0	0	0	2	2	1	1	1	3	1	2	0	1	1	0	2	1	1
ENSG00000142867	518	391	474	326	342	458	315	454	379	283	1325	238	285	314	367	384	353	475	354	414	918	409	621	362	339	509	354	909	287	340	378	388
ENSG00000223653	0	2	0	0	3	3	0	8	1	1	4	1	12	11	16	9	0	1	2	2	8	9	5	9	0	2	1	1	5	11	10	20
ENSG00000153904	2	1	5	1	821	777	595	870	29	46	52	17	1430	1415	1299	1709	1	3	9	7	1092	463	861	456	10	28	3	49	1007	1389	920	1466
ENSG00000199459	0	0	0	0	0	0	1	0	0	0	0	0	3	1	1	0	0	0	0	0	2	1	0	1	0	0	0	0	0	2	2	0
ENSG00000273264	0	0	0	0	0	0	0	0	0	0	0	0	0	2	0	0	0	0	0	0	1	0	0	0	0	0	0	0	1	0	0	0
ENSG00000282057	0	0	1	2	1	3	2	2	0	0	2	0	1	1	0	5	0	0	3	1	4	2	8	3	0	0	3	0	0	1	0	2
ENSG00000272691	0	1	0	0	1	1	1	2	2	0	0	1	2	0	0	2	0	0	1	0	3	0	0	0	0	0	0	1	0	1	0	0
ENSG00000142871	10	18	16	10	61	49	39	81	2	0	3	0	3	5	18	16	4	10	9	9	83	25	60	27	0	0	1	2	0	6	2	12
ENSG00000199934	0	0	0	0	0	0	0	0	1	0	3	0	0	0	0	0	0	0	0	0	0	1	0	0	0	0	1	0	0	0	1	0
ENSG00000230285	0	0	1	0	1	0	0	0	1	0	0	0	0	0	1	0	0	0	0	0	0	1	0	0	0	0	0	1	0	0	0	1
ENSG00000117174	2098	1366	1768	1595	2014	2078	1658	2129	1129	894	3565	648	2274	2064	2577	2611	1270	1654	1517	1936	4264	1875	3236	1594	1066	1688	757	2357	2317	2650	2831	2898
ENSG00000171502	1	0	4	1	73	77	117	74	9	3	22	4	14	15	13	18	0	4	3	11	112	50	120	47	18	12	9	28	2	6	3	11
ENSG00000229505	0	0	0	0	0	0	0	0	0	0	1	0	0	0	0	0	0	0	0	0	0	0	1	0	0	0	2	0	0	0	0	0
ENSG00000122417	1357	1039	1100	1064	747	822	617	949	1233	1016	4446	695	624	679	727	886	799	889	859	1177	1709	734	1043	831	934	1208	928	2539	423	750	654	968
ENSG00000278281	6	1	2	2	1	1	1	2	0	1	7	0	1	0	0	2	1	1	1	3	3	1	1	1	2	3	2	0	2	0	0	0
ENSG00000231349	0	0	0	0	0	0	0	0	0	0	0	0	0	2	0	1	1	0	0	0	0	0	0	1	0	0	0	2	0	0	0	0
ENSG00000137975	1	0	0	0	0	0	0	0	0	0	159	1	0	0	1	0	0	0	0	3	0	0	0	2	1	0	21	14	0	0	0	0
ENSG00000016490	0	2	0	1	0	0	0	0	0	0	0	0	0	0	0	0	0	1	1	4	0	0	0	0	0	0	0	0	0	0	0	0
ENSG00000016602	0	0	0	0	2	1	1	0	0	0	0	2	0	0	0	1	0	0	0	0	0	0	0	0	0	0	0	0	0	0	0	1
ENSG00000224466	0	0	0	0	0	0	0	0	0	0	0	0	0	0	0	0	0	0	0	0	0	0	0	0	0	0	0	1	0	0	1	0
ENSG00000236915	0	0	0	0	2	0	0	1	1	0	3	0	4	1	0	0	0	0	0	0	1	0	1	1	0	0	0	1	1	0	0	1
ENSG00000153923	6	3	1	3	3	2	0	1	0	2	0	0	2	3	4	1	1	0	0	3	0	1	0	2	0	3	4	1	0	0	0	2
ENSG00000261737	30	19	19	12	34	29	25	33	22	7	57	9	16	16	21	18	26	25	19	35	71	32	48	36	16	16	19	28	15	18	23	12
ENSG00000097033	1917	1572	1577	1474	3392	3546	2727	3891	2035	1695	8732	1410	1682	1623	1702	2066	873	1170	1056	1498	6175	2459	4680	2508	2075	2917	2123	5610	1228	1581	1403	1705
ENSG00000230721	1	1	0	0	0	0	0	0	0	0	0	1	0	0	0	0	0	1	0	0	0	0	1	0	0	0	0	0	0	0	0	2
ENSG00000183291	4876	3229	3874	3147	3832	3891	2955	4056	2955	2275	9890	1660	2641	2582	3140	3357	6738	8697	7486	6281	11070	4396	8007	5459	3183	4289	2261	6307	3840	3425	5091	4391
ENSG00000153936	2524	1766	1998	1726	2090	2274	1755	2382	1444	1226	4440	888	1285	1161	1163	1492	1271	2020	1726	2252	5009	1822	3456	2017	1511	2088	929	3569	970	1297	1043	1370
ENSG00000267734	13	11	10	10	7	10	0	6	9	3	11	1	1	6	1	5	1	6	2	11	21	11	7	8	11	13	4	38	1	7	9	5
ENSG00000267561	1	3	5	1	6	2	2	1	1	0	3	0	2	1	0	0	0	0	3	3	12	2	6	2	1	4	3	7	3	1	1	2
ENSG00000235251	0	0	0	1	2	3	0	1	4	3	3	0	0	3	0	0	0	0	0	6	7	0	2	2	3	2	0	7	1	6	0	0
ENSG00000225568	3	3	0	2	2	2	0	7	0	0	3	0	1	0	0	0	1	0	0	1	6	2	2	3	0	3	1	3	1	2	1	1
ENSG00000267272	2	0	0	3	17	7	10	11	13	14	83	6	7	4	4	6	1	2	1	6	15	6	7	12	19	37	21	30	7	2	4	4
ENSG00000143013	417	405	297	389	717	722	545	758	5722	4606	21291	3222	1243	1311	1162	1654	159	412	216	360	1326	585	1128	505	7231	9568	5236	13704	1867	2369	1751	2416
ENSG00000227290	2	0	2	0	1	1	0	2	6	4	15	2	2	0	0	0	0	0	0	4	0	1	2	0	16	8	5	21	1	0	1	0
ENSG00000279778	0	1	0	0	1	0	0	1	1	0	2	0	16	10	14	17	0	0	0	0	1	1	1	0	0	0	1	0	4	4	3	6
ENSG00000224235	0	0	0	0	0	0	0	0	0	0	0	0	0	0	0	0	0	0	0	0	0	0	0	0	0	0	0	0	0	0	0	0
ENSG00000270507	0	0	0	0	0	0	0	0	0	0	0	0	0	0	0	0	0	0	0	0	0	0	0	0	0	0	0	0	0	0	0	0
ENSG00000239504	0	0	0	0	0	0	0	0	0	0	0	0	0	0	0	0	0	0	0	0	0	0	0	0	0	0	0	0	0	0	0	0
ENSG00000230053	0	0	0	0	0	0	0	0	0	0	1	0	0	0	0	0	0	0	0	0	0	0	0	0	0	0	0	0	0	0	0	0
ENSG00000274321	0	0	0	0	0	0	0	0	0	1	0	0	0	0	0	0	0	0	0	0	0	0	0	0	0	0	0	0	0	0	0	0
ENSG00000237505	4	1	1	2	3	0	1	1	2	0	3	5	2	2	2	0	1	0	3	2	5	0	1	4	2	1	2	10	2	0	3	2
ENSG00000237959	0	0	0	0	0	0	0	0	0	0	0	0	0	0	0	0	0	0	0	0	0	0	0	0	0	0	0	0	0	0	0	0
ENSG00000065243	1830	1217	1411	1248	1417	1445	1078	1579	1235	1001	4940	761	861	889	893	1159	748	798	687	1375	3251	1497	1971	1514	1157	1475	1421	3173	578	1021	818	1139
ENSG00000207234	12	17	5	7	16	6	12	14	9	5	30	3	3	2	1	4	4	1	1	9	20	10	9	12	5	3	11	22	0	9	3	1
ENSG00000241975	52	41	36	44	41	52	36	44	38	37	205	37	23	15	15	41	16	14	5	46	98	52	53	46	20	33	45	107	7	27	13	25
ENSG00000137947	714	449	587	434	558	562	480	604	472	349	1998	308	487	394	566	592	868	1110	1020	934	1792	672	1295	741	456	632	456	1255	635	574	936	791
ENSG00000235308	9	8	9	11	5	9	3	9	4	5	17	3	13	10	7	8	0	2	3	5	9	5	1	4	11	8	9	17	2	4	5	4
ENSG00000137944	872	652	642	613	721	660	535	743	941	761	2726	493	741	640	668	821	587	718	625	727	1476	563	969	651	833	1194	671	1952	552	828	784	917
ENSG00000213516	708	423	615	487	513	534	328	530	996	898	3246	643	408	429	421	586	277	312	338	562	818	380	583	323	892	1351	656	2195	350	478	407	610
ENSG00000117226	291	181	245	161	127	147	115	177	557	436	2375	426	350	320	359	459	160	180	167	155	231	104	147	148	478	704	480	1399	335	455	326	486
ENSG00000202385	1	0	0	0	0	0	0	1	0	0	2	1	0	0	0	0	1	0	0	0	0	0	0	0	1	0	1	3	0	0	0	0
ENSG00000117228	1182	631	1207	629	66	61	58	90	2997	2009	13857	1814	1960	2057	2261	2703	1046	898	1587	857	133	51	114	50	2914	3895	2249	7555	2882	4930	4413	6314
ENSG00000234518	20	13	12	5	9	10	7	13	77	65	347	38	14	17	21	27	11	9	16	13	12	11	10	11	27	50	70	138	11	22	21	41
ENSG00000162645	258	123	327	129	209	275	196	356	1956	1278	7981	1087	345	472	448	719	155	114	359	148	295	215	318	145	916	1308	1412	2877	371	798	716	1057
ENSG00000213512	0	0	0	0	1	0	0	0	3	1	16	1	0	0	0	1	0	0	0	0	0	0	0	0	1	5	7	8	2	1	0	0
ENSG00000162654	0	0	0	0	153	227	112	219	3513	2601	13534	2381	1225	1369	1482	1786	0	0	1	0	173	78	106	65	3744	4997	1413	7716	1344	2632	2142	3031
ENSG00000154451	27	28	16	25	605	808	430	835	17601	14271	56168	10276	1975	2068	2152	2757	32	57	52	64	1244	602	755	591	14419	21428	8253	31359	2178	4433	3446	5959
ENSG00000237568	0	0	0	0	0	0	0	0	0	0	0	0	0	0	0	0	0	0	0	0	0	0	0	0	0	0	0	0	0	0	0	0
ENSG00000238081	0	0	0	0	0	0	0	0	0	0	0	0	3	0	0	0	0	0	0	0	0	0	2	0	0	2	0	1	0	0	0	0
ENSG00000233235	0	0	0	0	0	0	0	0	2	4	5	2	1	1	1	1	0	0	0	0	0	0	0	0	2	3	1	3	0	0	1	0
ENSG00000183347	1	0	0	0	0	0	1	3	93	48	208	51	67	52	48	66	0	0	0	0	1	1	0	0	48	81	13	91	64	76	64	122
ENSG00000215874	0	0	0	0	0	0	0	0	2	1	3	0	3	0	0	0	4	0	0	0	0	0	0	0	0	0	1	2	0	1	0	4
ENSG00000225492	3	0	0	1	1	0	0	0	98	73	439	68	18	32	28	40	0	0	1	6	0	1	0	0	63	81	55	158	27	43	36	64
ENSG00000197147	1132	912	950	813	926	860	632	935	359	321	1726	277	1276	1239	1403	1592	502	843	682	943	1960	698	1256	697	476	746	507	1168	989	1406	1198	1578
ENSG00000231999	91	96	96	89	105	139	78	126	26	38	95	18	47	61	88	91	37	37	58	85	177	67	120	57	25	68	19	72	30	71	67	73
ENSG00000230735	22	14	9	14	20	17	21	24	10	6	26	3	9	11	1	4	0	4	2	11	22	16	28	10	10	9	7	22	10	11	9	12
ENSG00000171488	2269	1956	1809	1828	1583	1576	1252	1747	1015	917	2317	630	1269	1288	1439	1725	886	1584	1092	1661	3125	1182	2174	1218	998	1605	647	2127	1314	1784	1590	1965
ENSG00000271949	0	0	0	0	0	1	0	0	0	0	0	0	0	1	0	0	0	0	0	0	0	1	1	0	1	2	1	0	0	0	0	0
ENSG00000226394	9	3	8	1	3	1	0	2	1	2	2	2	3	2	1	7	3	2	0	4	7	6	2	1	2	4	1	16	0	1	3	4
ENSG00000231613	4	5	4	2	0	0	0	1	2	0	1	0	3	3	0	0	0	1	2	1	1	0	0	1	1	0	0	0	0	2	0	8
ENSG00000272931	47	40	40	39	34	28	19	36	45	36	153	32	30	25	34	44	17	32	14	34	47	21	34	13	41	61	39	93	27	34	24	34
ENSG00000171492	643	493	637	513	1852	1760	1388	1763	1037	768	3865	648	1178	1128	1275	1383	499	857	704	766	4356	1580	3184	1479	1061	1588	850	2381	1074	1313	1371	1490
ENSG00000272672	1	0	1	1	5	5	1	5	2	2	10	0	3	4	3	2	1	0	0	1	3	0	0	1	0	3	4	6	1	1	1	3
ENSG00000252797	1	1	3	1	2	2	0	2	2	2	4	0	2	4	0	0	0	0	2	4	2	1	0	0	0	0	2	0	0	1	0	1
ENSG00000228175	74	54	86	66	25	17	18	29	14	13	46	10	21	22	33	46	42	42	50	55	24	17	27	4	21	18	1	35	11	25	32	28
ENSG00000162664	2960	2153	2231	2106	2022	2242	1608	2410	1764	1477	6696	1128	2466	2381	2676	3159	1824	2157	2085	2529	5314	2387	3992	2258	1685	2295	1503	4266	2342	3100	3053	3571

ENSG00000212459	0	0	0	0	0	0	0	0	0	0	0	0	0	0	0	0	0	0	0	0	0	0	0	0	0	0	0	0	0	0	0	0
ENSG00000229201	0	0	0	0	0	0	0	0	0	0	3	0	0	0	0	0	0	0	0	0	0	0	0	0	0	0	0	0	0	0	0	0
ENSG00000199666	0	0	0	0	0	0	0	0	0	0	7	0	0	0	0	0	0	1	0	0	0	0	0	0	0	0	1	4	0	0	0	2
ENSG00000143032	137	154	153	147	0	0	0	0	0	0	0	0	5	2	10	6	20	73	60	120	0	0	0	0	0	0	0	1	0	2	0	2
ENSG00000233593	135	111	122	110	0	1	1	2	16	26	54	14	15	19	2	16	42	91	48	107	3	4	0	1	12	10	4	27	6	5	4	4
ENSG00000225446	2	1	2	3	0	0	1	0	1	1	1	0	2	0	2	0	1	0	1	3	1	1	0	2	0	1	0	1	0	0	0	1
ENSG00000272094	1	2	1	0	3	1	1	2	6	0	2	1	7	4	9	5	0	0	1	0	2	3	1	3	3	4	0	4	5	4	2	5
ENSG00000232882	5	2	5	6	3	10	3	5	4	7	26	2	3	2	2	5	1	1	0	6	5	3	9	3	1	5	6	12	2	5	3	5
ENSG00000122482	2233	1523	1802	1354	1604	1839	1394	1905	1446	1138	5111	845	1644	1638	1988	2011	2019	2500	2654	2530	5201	2094	3623	2182	1477	2159	1250	3729	1533	2034	2114	2386
ENSG00000232393	0	0	0	0	0	1	0	0	0	0	0	0	0	0	0	0	0	1	0	0	3	0	1	1	0	1	0	0	0	0	1	0
ENSG00000162669	0	1	0	3	5	16	24	22	1	3	4	0	7	13	13	15	1	0	0	2	43	18	24	25	0	2	1	7	6	20	15	19
ENSG00000206817	0	0	0	0	0	0	0	0	0	0	0	0	0	0	0	0	0	0	0	0	0	0	0	0	0	0	0	0	0	0	0	0
ENSG00000215873	0	0	0	0	0	0	0	0	0	0	0	0	0	0	0	1	0	0	0	0	0	0	0	0	0	0	0	0	0	0	0	0
ENSG00000097046	2698	2079	2137	1956	2428	2604	1930	2764	959	802	2269	508	1692	1587	1621	2035	1639	2192	1806	2197	5086	2101	3577	2099	883	1153	402	1996	1209	1487	1400	1715
ENSG00000241318	5	5	11	7	3	5	0	0	3	0	2	0	0	5	7	5	1	5	2	24	6	2	4	2	1	2	1	8	0	5	4	2
ENSG00000229067	0	0	0	0	0	0	0	0	0	0	0	0	0	0	0	0	0	0	0	0	0	0	0	0	0	0	0	2	0	0	4	0
ENSG00000203914	0	0	0	0	0	0	0	0	0	0	0	0	0	0	0	0	0	0	1	0	0	0	0	0	0	0	0	0	0	0	0	0
ENSG00000069702	0	1	0	0	26	57	21	56	92	105	890	131	688	751	742	955	0	2	0	0	46	26	33	12	280	517	694	1522	597	944	655	1112
ENSG00000239794	0	0	0	0	0	0	0	0	0	1	0	0	0	0	0	0	0	0	0	0	0	0	0	0	0	5	0	3	0	0	0	2
ENSG00000137948	0	0	0	0	5	10	1	13	0	0	2	0	1	2	2	3	0	2	0	0	1	2	0	0	0	0	4	6	3	4	2	4
ENSG00000172031	0	0	0	0	49	71	41	72	2	4	7	0	10	28	21	28	0	0	0	0	108	56	91	46	4	5	3	6	13	16	20	19
ENSG00000233228	0	0	0	0	0	0	0	0	0	0	0	0	0	1	0	0	0	0	0	0	0	0	0	0	0	0	0	0	0	0	0	0
ENSG00000230667	1	1	1	0	0	0	0	0	1	1	3	0	0	1	2	0	2	0	1	2	2	2	1	0	0	3	0	0	0	2	3	2
ENSG00000189195	64	61	50	44	47	47	44	48	24	31	117	22	44	65	22	64	24	22	22	33	56	34	41	29	28	28	33	72	21	44	27	40
ENSG00000224678	2	2	1	0	0	1	0	0	0	2	6	2	0	1	0	0	0	0	0	3	2	1	3	1	0	0	0	3	0	0	0	1
ENSG00000233401	1	2	1	2	1	2	0	0	0	1	2	0	1	0	2	3	2	0	0	0	1	2	0	1	5	2	4	0	0	0	1	0
ENSG00000069712	0	0	0	0	0	0	0	0	0	0	0	0	0	0	0	0	0	0	0	0	0	0	0	0	0	0	0	0	0	0	0	0
ENSG00000273487	1	2	0	1	4	3	1	3	3	3	18	4	1	6	2	7	0	0	1	3	5	4	1	0	0	3	5	8	1	5	2	9
ENSG00000226773	0	0	0	0	0	0	0	0	0	0	0	0	0	0	0	0	0	0	0	0	0	0	0	0	0	0	0	0	0	0	0	0
ENSG00000203910	4	7	4	2	7	10	9	11	14	9	25	5	13	10	6	13	2	2	1	2	24	10	5	4	13	13	7	33	5	5	12	10
ENSG00000233125	0	0	0	0	0	0	0	0	0	0	0	0	0	0	0	0	0	1	0	0	0	0	0	0	0	0	0	0	0	0	0	0
ENSG00000174842	705	490	557	506	592	662	473	708	526	405	1779	288	448	467	449	573	587	738	682	827	1590	648	1019	815	494	653	412	1249	491	566	716	730
ENSG00000122484	1556	1125	1358	1093	1096	1215	903	1255	1472	1223	7218	1008	737	773	845	1016	818	1006	871	1472	2201	969	1565	887	1399	1978	1852	4282	702	1112	832	1164
ENSG00000242764	0	0	0	0	0	1	0	0	0	0	2	0	0	0	0	0	0	0	0	0	0	0	0	0	0	0	0	0	0	0	0	1
ENSG00000162676	1027	933	1046	1053	304	419	241	424	897	753	2623	563	467	481	564	607	330	359	428	707	358	176	294	173	799	1198	418	1706	311	387	354	452
ENSG00000067208	5	2	5	2	78	72	47	106	15	23	107	9	264	300	322	321	2	2	4	6	158	62	92	60	13	28	59	119	219	319	279	313
ENSG00000223787	0	0	0	0	0	0	1	0	0	0	0	0	1	0	5	1	0	0	0	0	0	0	1	0	0	0	0	1	0	2	4	0
ENSG00000229992	0	0	0	0	1	0	0	0	0	0	3	0	1	0	1	3	0	0	0	0	1	0	0	0	0	0	1	2	3	3	1	1
ENSG00000201317	0	0	0	0	0	0	0	0	0	0	0	0	0	0	1	0	0	0	0	0	0	0	0	0	0	0	0	0	0	0	0	0
ENSG00000225505	0	0	0	0	0	1	0	1	0	0	2	0	1	3	3	2	0	0	0	0	2	0	1	3	0	0	0	2	1	5	6	1
ENSG00000234202	0	0	0	0	0	0	0	0	0	0	2	0	0	0	0	1	0	0	0	0	0	0	0	0	0	0	0	0	0	0	0	1
ENSG00000223896	0	0	0	1	1	0	0	0	0	0	1	0	1	2	2	1	0	0	0	0	0	0	0	0	0	0	0	1	2	0	2	1
ENSG00000122406	20984	14544	14146	13968	13583	13940	10156	15168	21024	15166	55167	10944	24364	22072	25604	29240	18064	23050	19053	17721	26851	10542	20363	11313	17180	23928	10383	33392	25457	24681	30316	31442
ENSG00000154511	401	274	346	285	284	308	243	333	121	128	372	80	228	201	237	298	289	394	331	392	529	217	362	196	105	166	78	290	187	239	226	293
ENSG00000206680	0	0	0	0	0	0	0	0	0	0	0	0	0	0	0	0	0	0	0	0	0	0	0	0	0	0	0	0	0	0	0	0
ENSG00000207523	27	10	22	14	18	21	22	12	22	15	101	9	13	20	19	27	11	12	10	30	31	19	16	20	22	20	14	36	14	10	12	25
ENSG00000207022	1	3	0	1	1	3	0	1	0	0	4	0	2	0	2	2	2	1	1	3	1	1	0	1	0	0	4	0	0	0	2	0
ENSG00000229052	32	18	23	14	17	14	14	28	11	8	20	0	14	5	10	2	3	7	2	27	23	12	12	21	2	5	4	19	4	7	11	13
ENSG00000252121	0	0	0	0	0	0	0	0	0	0	0	0	0	0	0	0	1	0	0	0	0	0	0	0	0	0	0	0	0	0	0	0
ENSG00000239710	0	0	0	0	0	0	0	0	0	0	0	0	0	0	0	0	0	0	0	0	0	0	0	0	0	0	0	0	0	0	0	0
ENSG00000252752	0	0	0	0	0	0	0	0	0	0	0	0	0	0	0	0	0	0	0	0	0	0	0	0	0	0	0	0	0	0	0	0
ENSG00000222664	0	0	0	0	0	0	0	0	0	0	0	0	0	0	0	0	0	0	0	0	0	0	0	0	0	0	0	0	0	0	0	0
ENSG00000251837	0	0	0	0	0	0	0	0	0	0	0	0	0	0	0	0	0	0	0	0	0	0	0	0	0	0	0	0	0	0	0	0
ENSG00000143033	4137	2804	3009	2848	2982	2941	2219	3251	1425	1097	4676	803	1942	1748	2109	2137	1905	2532	2484	2250	6022	2349	4294	2380	1411	1832	1014	3043	1734	1985	2249	2260
ENSG00000117500	2396	1553	1938	1559	3000	3106	2283	3394	1230	981	4564	724	2343	2177	2586	2626	1883	2191	1996	2323	6970	2969	4753	3076	1477	1839	1010	3060	2327	2464	2740	2786
ENSG00000122483	1316	915	1065	915	1186	1317	1096	1409	1065	845	3605	597	727	659	731	841	541	583	612	993	2541	1183	1664	1265	1149	1396	567	2760	600	850	792	965
ENSG00000237003	1	3	5	2	4	2	2	5	5	2	3	2	2	1	2	1	2	1	2	5	3	4	0	0	3	5	0	6	0	1	3	4
ENSG00000225297	6	8	4	7	7	12	2	6	5	4	20	1	2	2	3	1	0	5	0	4	6	7	4	8	10	9	2	11	2	3	2	5
ENSG00000223745	778	541	702	603	377	465	325	521	1481	1281	5570	763	898	928	806	1222	182	172	154	432	374	252	309	292	595	871	1307	2316	185	481	278	571
ENSG00000229567	6	5	5	3	3	10	1	8	15	10	33	3	5	7	5	6	6	1	2	11	8	1	3	3	16	7	2	26	2	4	8	5
ENSG00000117505	4493	2988	3659	2970	3082	3283	2520	3437	2936	2656	11336	2126	3568	3225	3843	4292	2479	3666	3529	3283	6729	2751	5199	2443	3502	4982	2382	7842	3187	3557	3789	3991
ENSG00000229635	7	1	2	5	2	6	0	3	4	10	13	1	3	3	3	6	1	0	1	4	5	1	4	4	2	4	1	2	0	2	0	1
ENSG00000207379	0	0	0	0	0	1	0	0	0	0	2	1	0	0	0	0	0	0	0	0	0	0	1	1	0	0	0	0	1	0	0	0
ENSG00000137942	1462	1223	1259	1240	815	936	642	1024	61	64	282	46	390	525	433	724	770	1100	850	1110	1531	614	1037	591	15	55	26	102	361	631	449	639
ENSG00000137936	68	65	49	63	31	36	22	55	39	50	186	36	71	65	46	89	18	35	17	45	51	37	29	20	47	126	138	250	27	39	27	55
ENSG00000224093	2	3	2	4	37	27	33	51	2	2	5	0	72	72	112	110	1	0	0	6	52	24	27	34	3	2	1	6	64	88	87	109
ENSG00000230439	35	14	16	27	2	1	2	5	5	2	10	6	19	17	10	13	8	15	15	20	2	3	0	1	3	6	5	8	5	6	7	10
ENSG00000211575	7	3	5	3	1	1	1	0	0	0	4	0	1	1	1	0	0	1	0	3	1	0	0	0	0	0	1	4	1	0	0	0
ENSG00000260464	19	17	11	12	8	7	1	14	2	1	20	0	9	2	6	9	5	3	5	12	8	6	3	8	3	5	7	12	6	7	6	3
ENSG00000067334	1754	1017	1265	1062	1717	1794	1411	2073	1539	1001	5140	845	1330	1295	1659	1697	3345	3746	3185	2386	5707	2045	3768	2287	1227	2112	1218	3428	2040	1996	2777	2418
ENSG00000023909	1385	988	1104	1028	915	984	650	964	589	525	2217	402	1260	1162	1105	1349	467	862	614	1084	1437	657	990	525	645	1110	577	1634	793	1028	784	1083
ENSG00000223490	0	0	0	0	0	2	0	0	1	0	0	0	1	0	0	1	0	0	0	0	2	1	0	0	0	0	1	0	0	0	1	0
ENSG00000234099	0	0	0	0	0	1	1	0	0	1	3	0	2	2	0	0	0	0	0	1	2	0	0	1	0	0	1	0	0	0	0	1
ENSG00000235794	0	0	0	0	0	0	0	0	1	0	0	0	1	0	1	0	0	0	0	0	0	0	0	0	0	0	0	0	0	0	0	0
ENSG00000232752	0	0	2	0	0	0	0	0	0	0	0	0	0	0	0	2	0	0	0	0	0	0	1	0	0	0	1	2	0	0	0	2
ENSG00000229842	0	0	0	0	0	0	0	0	0	0	0	0	2	0	0	0	0	0	0	0	0	0	0	0	0	2	1	2	0	3	0	2
ENSG00000250890	0	0	0	0	0	0	0	0	0	0	0	0	0	0	2	0	0	0	0	0	0	0	0	0	0	0	0	1	0	0	0	0
ENSG00000236689	0	0	0	0	0	0	0	0	0	0	0	0	1	0	0	1	0	0	0	1	0	0	0	0	0	0	0	0	2	1	2	1
ENSG00000226960	0	0	0	0	0	0	0	0	0	0	0	1	0	0	1	0	0	0	0	0	0	0	0	0	0	0	0	0	0	0	0	0
ENSG00000233129	0	0	0	0	0	0	0	0	0	0	2	0	3	3	0	0	0	0	0	0	0	0	0	0	1	0	0	2	0	0	0	1
ENSG00000198691	84	44	120	38	133	165	103	145	0	0	0	0	101	54	56	63	33	8	30	60	50	23	26	17	0	0	0	2	9	22	18	18
ENSG00000233482	0	1	1	0	0	0	0	0	0	0	0	0	0	0	0	0	0	0	0	0	0	0	0	0	0	0	0	0	0	0	0	0
ENSG00000137962	53	30	61	32	19	23	14	15	0	1	0	0	145	145	175	166	21	31	50	54	24	18	19	16	0	0	1	2	30	62	66	97
ENSG00000264963	0	0	0	0	0	0	0	0	0	0	1	0	0	0	0	0	0	0	0	0	1	0	0	0	0	0	0	0	0	0	0	0
ENSG00000226835	0	0	0	0	0	0	0	0	0	0	0	0	0	0	0	1	0	1	0	0	0	0	0	0	0	0	0	0	0	0	0	0
ENSG00000178596	0	0	0	0	0	0	0	0	0	0	0	0	0	0	0	0	0	0	0	0	0	0	0	0	0	0	0	0	0	0	0	0
ENSG00000236098	0	0	0	1	0	0	0	1	0	0	0	0	0	2	0	4	1	0	2	0	0	0	0	0	0	2	0	0	0	0	1	1
ENSG00000232918	1	0	0	0	0	0	0	0	0	0	0	0	0	0	0	0	0	0	0	1	0	2	0	0	0	0	0	0	0	0	0	0
ENSG00000117528	2749	1922	2196	1907	2172	2359	1754	2527	1206	1030	4647	803	1044	1026	1212	1391	1892	2687	2533	2637	5550	2086	3820	2144	1496	2029	1152	3264	990	1232	1086	1432
ENSG00000117525	10	26	19	31	3	9	1	5	2	6	8	2	14	12	3	9	2	12	6	15	3	0	3	0	3	0	0	8	2	5	3	11
ENSG00000223675	0	0	0	0	0	0	0	0	0	0	2	0	0	0	0	0	0	0	0	0	0	0	0	0	0	0	0	0	0	0	0	0
ENSG00000224081	0	0	0	0	5	3	0	7	0	1	2	0	2	15	7	20	0	0	0	0	4	1	4	4	0	0	9	5	0	5	4	6
ENSG00000232857	0	0	0	0	0	0	1	0	0	0	1	0	0	0	0	0	0	0	0	0	0	0	0	0	0	0	0	0	0	0	0	0
ENSG00000263526	0	0	0	0	0	0	0	0	0	0	0	0	0	0	1	0	0	0	0	0	1	0	0	0	0	0	0	0	0	0	0	0
ENSG00000143036	0	0	0	1	4	5	4	7	0	2	22	1	0	2	3	6	0	0	0	0	8	3	6	3	0	3	16	10	0	7	2	6
ENSG00000117519	9	17	12	14	1394	1415	1033	1527	0	0	5	0	1	2	2	2	10	18	1	15	2884	1017	1971	936	0	0	1	5	1	0	2	3
ENSG00000235501	3	12	7	7	55	66	59	74	0	0	2	0	1	3	2	1	3	4	0	12	71	28	41	29	0	0	2	0	0	0	0	1
ENSG00000172339	393	248	348	252	41	44	36	48	78	72	252	58	113	84	109	107	437	557	559	548	131	45	69	52	121	148	64	226	148	153	171	172
ENSG00000230427	0	0	5	5	3	0	1	0	0	0	3	1	0	1	0	1	0	0	1	6	0	0	0	2	0	2	0	2	1	1	2	5
ENSG00000152078	58	109	64	148	127	184	93	215	186	249	997	194	712	743	747	977	15	29	23	42	83	50	58	49	149	246	108	542	361	640	534	769
ENSG00000271092	31	14	26	28	28	31	34	52	35	37	127	21	25	33	44	50	17	30	19	30	82	45	41	53	27	30	28	76	31	35	32	69
ENSG00000231992	1	0	0	1	0	0	0	2	3	3	7	0	0	0	0	1	0	0	0	0	1	0	2	0	0	1	1	3	0	0	0	0
ENSG00000206679	0	2	0	1	0	0	0	2	0	2	4	0	0	0	0	3	0	0	0	0	0	1	0	0	0	0	0	3	0	0	1	0
ENSG00000226026	0	0	2	1	0	2	1	0	2	5	12	0	7	3	6	5	0	0	1	1	0	0	1	0	0	1	3	9	2	4	4	7
ENSG00000122481	218	130	189	142	204	234	150	229	161	161	658	140	255	240	217	277	188	246	208	231	481	225	307	226	144	177	138	397	224	239	241	349
ENSG00000228852	28	33	32	33	24	35	19	37	27	33	106	15	26	51	40	34	9	7	6	20	36	15	23	22	14	24	15	53	5	30	9	18
ENSG00000228504	0	0	0	0	0	0	0	0	0	0	0	0	0	0	0	0	0	0	0	0	0	0	0	0	0	0	0	0	0	0	0	0
ENSG00000230434	0	0	0	0	0	0	0	0	0	0	0	0	0	0	0	0	0	0	0	0	0	0	0	0	0	0	0	0	0	0	0	0
ENSG00000237954	0	0	0	0	0	0	0	0	0	0	0	0	0	0	0	0	0	0	0	0	0	0	0	0	0	0	0	1	0	0	0	0
ENSG00000233907	1	0	0	0	0	0	0	0	0	0	0	0	0	0	0	1	0	0	0	0	0	0	0	0	0	0	0	0	0	0	0	0
ENSG00000228971	0	0	0	0	0	0	0	0	0	0	0	0	0	0	1	0	0	0	0	0	0	0	0	0	0	0	0	0	0	2	0	0
ENSG00000271252	0	0	0	0	0	0	0	0	0	0	0	0	0	0	0	0	0	0	0	0	0	0	0	0	0	0	0	0	0	0	0	0
ENSG00000237435	0	0	0	0	0	0	0	0	0	0	1	0	0	0	0	0	0	0	0	0	1	0	0	0	0	0	0	1	0	0	1	0
ENSG00000200800	0	0	0	0	0	0	0	0	0	0	1	0	0	0	0	0	0	0	0	0	0	0	0	0	0	2	1	3	0	0	0	0
ENSG00000231987	0	0	0	0	0	0	0	0	0	0	0	0	0	0	1	0	0	0	0	0	0	0	0	0	0	0	0	0	0	0	0	0
ENSG00000225923	0	0	0	0	0	0	0	0	0	0	0	0	0	0	0	0	0	0	0	0	0	0	0	0	0	0	0	0	0	0	0	0
ENSG00000234422	0	0	0	0	0	0	0	0	0	0	0	0	0	0	0	0	0	0	0	0	0	0	0	0	0	0	0	0	0	0	0	0
ENSG00000228502	3	1	3	5	1	1	1	1	2	3	9	2	2	5	3	4	2	2	2	0	1	1	0	0	4	1	1	3	4	2	4	3
ENSG00000241992	0	0	0	0	0	0	0	0	0	0	0	0	0	0	0	0	0	0	0	0	0	0	0	0	0	0	0	0	0	0	0	0
ENSG00000234226	0	0	0	0	0	0	0	0	0	0	0	0	0	0	0	0	0	0	0	0	0	0	0	0	0	0	0	0	0	0	0	0
ENSG00000137970	95	57	70	60	66	64	48	66	156	76	297	68	82	93	84	141	118	114	126	142	131	58	106	75	104	135	65	163	83	116	143	120
ENSG00000223229	0	0	0	0	0	0	0	0	0	0	0	0	0	0	0	0	0	0	0	0	0	0	0	0	0	0	0	0	0	0	0	0
ENSG00000117569	1542	1000	1107	985	971	1028	723	1151	748	614	2490	408	540	510	514	652	719	983	842	1241	2211	950	1301	1038	668	832	538	1679	347	656	484	630
ENSG00000188641	520	417	431	379	1218	1202	1012	1346	607	510	1805	351	480	588	474	732	366	549	636	482	2426	870	1948	863	692	788	447	1491	463	702	626	740
ENSG00000232878	11	10	7	3	2	3	4	8	2	3	9	0	3	2	3	4	1	3	1	6	10	10	8	9	9	4	2	10	0	3	1	3
ENSG00000232542	7	1	0	2	4	2	3	2	0	0	5	0	1	0	0	0	2	0	1	0	5	1	2	7	1	1	0	6	0	1	0	1
ENSG00000228057	2	4	6	8	7	6	2	8	1	1	21	1	2	3	9	2	2	2	1	6	13	4	10	7	4	5	3	9	3	2	1	13
ENSG00000226128	1	0	0	2	2	0	0	3	1	0	0	0	0	3	0	1	1	0	1	0	3	0	2	2	3	1	0	5	0	1	1	0
ENSG00000230718	1	0	0	1	0	6	2	4	1	1	1	0	0	0	1	2	0	0	0	0	5	2	1	4	1	0	0	4	0	0	5	3
ENSG00000235777	3	0	0	1	2	4	4	2	1	1	3	1	2	2	0	1	0	2	1	5	14	5	10	7	4	5	2	8	3	3	6	7
ENSG00000270911	3	0	0	0	7	5	0	5	6	0	2	2	1	0	2	2	0	1	2	5	1	1	6	6	2	3	1	10	3	2	2	3
ENSG00000269998	0	0	0	0	0	0	0	0	0	0	0	0	0	0	0	0	0	0	0	0	0	0	0	0	0	0	0	0	0	0	0	0
ENSG00000259946	0	0	0	0	0	0	0	0	0	0	0	0	1	0	0	0	0	0	0	0	0	0	0	1	0	0	0	0	1	2	0	0
ENSG00000225206	0	0	1	0	1	2	1	3	0	0	2	0	1	0	0	2	0	0	0	0	2	6	5	1	0	0	0	0	0	3	0	3
ENSG00000233557	0	0	0	0	0	0	0	0	0	0	0	0	0	0	1	0	0	0	0	0	0	0	0	0	0	0	0	0	0	0	0	1
ENSG00000226053	0	0	0	0	0	0	0	0	0	0	0	0	0	0	0	0	0	0	0	0	0	0	0	0	0	0	0	0	0	0	0	0
ENSG00000280040	0	0	0	0	0	0	0	0	0	0	0	0	0	0	0	0	0	0	0	0	0	0	0	0	0	0	0	0	0	0	0	0
ENSG00000162627	2	0	0	0	0	0	0	0	11	6	30	4	0	1	0	0	0	0	0	0	0	0	0	0	8	11	1	31	0	0	0	0
ENSG00000117598	0	0	0	0	0	0	0	0	1	2	5	0	1	0	0	0	0	1	0	0	0	0	0	0	0	0	6	4	0	0	0	0
ENSG00000232825	0	0	0	0	0	0	0	0	0	0	0	0	0	0	0	0	0	0	0	0	0	0	0	0	0	0	0	0	0	0	1	0
ENSG00000227034	1	0	0	1	0	0	0	1	0	0	0	0	0	0	0	0	0	0	0	0	1	0	0	0	0	0	0	0	0	0	0	0
ENSG00000117600	0	0	0	0	0	0	0	0	0	0	1	0	1	2	1	1	0	0	0	0	0	0	0	0	0	1	0	0	0	1	0	4
ENSG00000233983	0	0	0	0	0	1	0	2	0	0	0	0	0	0	0	0	0	0	0	0	0	0	0	0	0	0	0	0	0	0	0	0
ENSG00000224445	0	0	0	0	0	0	0	0	0	0	0	0	2	0	1	0	0	0	0	0	0	0	0	0	0	0	0	2	0	0	0	0
ENSG00000099260	0	0	0	0	0	1	0	2	0	1	1	0	0	2	0	1	0	0	0	0	1	1	0	0	2	1	2	1	1	1	0	1
ENSG00000223656	0	0	0	0	1	0	0	0	1	0	0	0	0	2	0	0	0	0	0	0	4	0	0	0	1	0	0	1	0	1	1	0
ENSG00000156869	101	71	69	76	118	146	111	163	351	296	780	179	146	135	121	160	60	120	68	126	178	70	135	52	247	412	151	584	135	156	147	175
ENSG00000201491	0	0	0	0	0	0	0	0	0	0	3	0	0	0	0	0	0	0	0	0	0	0	0	0	0	0	0	0	0	0	0	0
ENSG00000202254	0	0	0	0	0	0	0	0	0	0	0	0	0	0	0	0	0	0	0	0	0	0	0	0	0	0	0	0	0	0	0	0
ENSG00000231996	0	0	0	0	0	0	0	0	0	0	0	0	0	0	0	1	0	0	0	0	2	0	0	0	0	0	0	1	0	0	1	0
ENSG00000162688	745	548	669	528	570	593	467	605	410	314	1363	216	463	452	453	604	296	496	429	787	996	431	734	446	343	560	328	817	343	554	405	661
ENSG00000283495	0	0	0	0	0	0	0	0	0	0	0	0	0	0	0	0	0	0	0	0	0	0	0	0	0	0	0	0	0	0	0	0
ENSG00000228084	9	10	18	11	3	8	5	3	13	6	38	4	36	17	25	18	9	9	12	4	7	5	5	3	14	3	5	17	14	12	13	17
ENSG00000117620	1431	1035	1279	1023	1148	1275	980	1254	977	863	3552	619	1101	976	1148	1281	818	1097	1054	1342	2068	924	1423	905	884	1173	753	2201	899	1143	1075	1266
ENSG00000212248	0	1	2	0	0	1	1	2	2	4	2	1	0	0	5	0	0	0	1	0	3	0	0	2	0	3	0	2	0	1	1	1
ENSG00000202259	2	2	3	0	4	1	4	6	0	1	0	0	2	5	1	3	0	0	0	1	9	6	4	3	0	1	3	5	0	1	2	1
ENSG00000156875	1986	1266	1584	1245	1649	1716	1236	1713	1212	997	4512	771	1453	1353	1484	1714	1283	1727	1329	1375	3590	1244	2435	1145	1260	1719	1217	2881	1389	1482	1608	1595
ENSG00000241073	0	0	2	1	1	3	0	1	2	0	4	0	2	1	1	2	0	0	0	2	2	1	1	2	5	2	3	6	2	0	1	1
ENSG00000156876	1297	811	983	714	1296	1278	1080	1366	712	540	1882	379	777	693	793	909	867	854	921	1093	3392	1292	2302	1418	769	1012	327	1690	816	956	976	1127
ENSG00000226952	12	7	11	6	9	8	5	9	4	5	8	3	6	7	8	7	4	4	1	3	18	4	8	9	6	7	2	22	0	7	6	8
ENSG00000122435	1149	718	991	719	805	905	771	868	872	743	3265	564	665	663	846	887	811	836	858	1165	2058	847	1430	946	854	1201	780	1929	658	891	823	1043
ENSG00000122477	141	95	88	107	55	71	52	62	85	70	288	57	25	41	42	46	53	33	34	109	110	48	60	52	55	101	84	152	20	38	24	52
ENSG00000137992	1323	875	1030	805	952	929	686	1078	903	756	3647	460	772	759	802	962	664	850	698	780	1637	624	1077	535	842	1261	941	2110	619	722	706	775
ENSG00000271415	3	1	1	4	0	1	1	1	3	3	8	3	0	1	4	4	1	0	0	3	3	1	0	1	1	2	0	5	0	1	0	3
ENSG00000225169	3	4	1	4	0	4	0	4	5	9	31	2	2	4	5	3	0	1	3	5	6	6	1	1	3	3	6	20	1	5	1	8
ENSG00000230287	1	1	3	2	0	1	1	4	0	0	2	2	0	0	2	1	0	0	1	1	1	1	1	1	0	1	1	7	0	1	0	1
ENSG00000224616	72	36	55	44	34	28	21	25	20	12	79	11	12	9	14	7	27	18	26	36	36	22	23	13	11	17	17	43	4	11	3	20
ENSG00000137996	1279	784	1056	793	992	973	812	1152	987	690	3842	617	828	757	1079	992	1043	1322	1328	1191	2402	950	1561	785	929	1310	819	2410	925	1049	1224	1110
ENSG00000207750	4	0	1	4	1	2	1	10	11	4	19	4	0	2	8	1	0	3	2	6	10	3	3	4	6	4	3	6	1	2	4	2
ENSG00000215871	0	0	0	0	0	0	0	0	0	0	0	0	0	0	0	0	0	0	0	0	0	0	0	1	0	0	0	0	0	0	0	0
ENSG00000223906	0	1	0	0	1	0	0	0	0	0	0	0	1	0	0	0	0	0	0	0	0	0	0	0	0	0	0	2	0	0	0	0
ENSG00000079335	226	167	197	175	599	635	483	636	196	188	772	134	362	394	435	479	46	76	71	171	1036	480	717	455	167	256	253	441	237	346	268	369
ENSG00000234332	10	9	4	1	7	5	4	9	2	2	9	2	7	10	2	2	0	1	2	4	19	7	2	16	6	2	6	15	1	2	11	6
ENSG00000228086	3	1	0	0	3	2	1	1	1	1	4	0	8	2	3	2	0	0	2	3	1	3	2	5	0	0	0	8	1	1	4	1
ENSG00000181656	0	5	2	3	11	12	10	18	6	9	18	9	4	8	6	9	4	4	2	8	14	11	15	5	5	9	11	13	0	8	7	2
ENSG00000213421	0	0	0	0	0	0	0	0	0	0	0	0	0	0	0	0	0	0	0	0	0	0	0	0	0	0	0	0	0	0	0	0
ENSG00000230402	0	0	0	0	1	1	0	0	0	0	0	0	0	0	0	0	0	0	0	0	0	0	0	0	0	0	0	0	0	0	0	0
ENSG00000224685	0	0	0	0	0	0	0	0	0	0	0	0	0	0	0	0	0	0	0	0	0	0	0	0	0	0	0	0	0	0	0	0
ENSG00000162692	0	0	0	0	0	0	0	0	0	0	1	0	968	961	1158	1260	1	0	0	0	0	1	0	0	0	0	0	0	813	967	1320	1263
ENSG00000162694	851	578	759	630	479	454	373	502	556	524	1858	329	402	487	501	592	657	982	743	998	986	404	638	476	549	821	388	1169	472	616	591	791
ENSG00000273204	0	0	0	0	0	0	0	0	0	0	0	0	0	0	0	0	0	0	0	0	0	0	0	0	0	0	0	0	0	0	0	0
ENSG00000162695	1835	1318	1558	1291	792	750	520	843	646	569	3007	416	1009	968	1119	1219	584	728	670	1354	1320	570	889	599	759	1012	801	2036	716	1078	894	1142
ENSG00000230946	17	15	20	3	2	11	7	5	9	6	40	8	3	8	13	3	6	2	2	23	15	9	4	6	10	10	10	38	2	10	7	12
ENSG00000117543	788	515	615	565	316	329	298	406	734	479	2230	359	892	747	908	930	997	1184	892	1081	909	381	623	437	714	981	459	1328	904	990	1087	1229
ENSG00000235795	18	12	19	8	24	25	15	15	18	6	44	4	10	14	16	12	10	10	21	39	23	10	26	18	10	12	12	28	9	21	8	19
ENSG00000233184	144	97	151	105	53	58	44	70	109	69	383	76	219	204	250	246	163	207	193	222	75	44	60	50	119	144	88	226	119	217	196	294
ENSG00000252765	0	0	0	0	0	0	0	0	0	0	0	0	0	0	0	0	0	0	0	0	0	0	0	0	0	0	0	0	0	0	0	0
ENSG00000271578	0	0	0	0	0	0	0	0	0	0	0	0	0	0	0	0	0	0	0	0	0	0	0	0	0	0	0	0	0	0	0	0
ENSG00000225938	1	0	0	0	0	0	0	0	0	1	2	0	0	0	0	0	0	0	0	0	0	0	0	0	0	0	2	1	0	0	0	0
ENSG00000170989	38	41	46	49	0	4	2	7	0	0	0	0	2	3	8	9	7	23	19	23	1	2	8	0	1	0	0	0	5	8	6	13
ENSG00000260940	0	0	0	0	0	0	0	2	0	0	1	0	0	0	0	0	0	0	0	0	0	0	0	1	0	0	0	0	0	0	0	0
ENSG00000228399	0	2	0	0	0	0	0	0	0	0	0	0	0	0	0	1	0	0	0	0	0	0	0	0	0	0	0	1	0	0	0	0
ENSG00000173810	0	0	0	0	0	0	0	0	0	0	0	0	0	0	0	0	0	0	0	0	0	0	0	0	0	0	0	0	0	0	0	0
ENSG00000231671	25	8	33	11	0	1	1	1	0	2	2	0	8	4	6	11	14	11	42	26	0	2	0	0	0	1	0	1	16	27	46	49
ENSG00000226715	0	0	5	0	0	0	1	1	0	0	3	1	0	0	0	0	4	0	1	1	1	1	0	0	1	0	0	1	0	0	0	1
ENSG00000252530	0	0	0	0	0	0	0	0	0	0	0	0	0	0	0	0	0	0	0	0	0	0	0	0	0	0	0	0	0	0	0	0
ENSG00000183298	0	3	2	0	0	0	0	1	1	0	6	2	0	4	3	0	2	7	1	1	6	0	4	1	3	3	1	1	7	2	0	2
ENSG00000118733	0	0	0	0	0	0	0	0	0	0	1	0	0	0	0	0	0	0	1	0	0	0	0	0	1	1	5	0	0	0	0	0
ENSG00000252717	0	0	0	0	0	0	0	0	0	0	0	0	0	0	0	0	0	0	0	0	0	0	0	0	0	0	0	0	0	0	0	0
ENSG00000271277	0	0	0	0	0	0	0	0	0	0	0	0	0	0	0	0	0	0	0	0	0	0	0	0	0	0	0	0	0	0	0	0
ENSG00000162699	0	0	0	0	0	0	0	0	0	0	0	0	0	0	0	0	0	0	0	0	0	0	0	0	0	0	0	0	0	0	0	0
ENSG00000233359	0	1	0	0	0	0	0	0	0	0	2	0	0	0	0	0	1	0	0	0	0	0	0	0	0	0	0	0	0	0	0	0
ENSG00000230864	0	0	0	0	0	0	0	0	0	0	0	0	0	0	0	0	0	0	0	0	0	0	0	0	0	0	0	0	0	0	0	0
ENSG00000060718	0	0	0	0	0	0	1	0	21	9	136	8	0	0	0	0	0	0	0	0	1	1	0	0	34	32	46	45	0	0	0	0
ENSG00000234257	0	0	0	0	0	0	0	0	0	0	0	0	0	0	0	0	0	0	0	0	0	0	0	0	0	0	0	0	0	0	0	0
ENSG00000230759	0	0	0	0	0	0	0	0	0	0	0	0	0	0	0	0	0	0	0	0	0	0	0	0	0	0	0	0	0	0	0	0
ENSG00000224613	4	2	7	1	1	0	0	0	3	0	2	1	1	2	0	1	0	2	1	1	4	0	2	0	0	1	0	4	3	0	3	0
ENSG00000222069	5	1	3	0	0	1	0	0	8	2	9	5	1	0	1	1	0	0	0	1	4	1	0	1	6	0	1	8	0	0	0	2
ENSG00000185946	846	672	642	614	758	833	637	867	768	727	3508	484	492	467	513	668	273	333	294	478	1248	540	693	542	621	847	889	1962	203	485	263	561
ENSG00000240038	120	129	96	98	21	23	15	21	224	226	978	185	262	275	218	366	42	30	31	93	18	16	15	10	127	203	295	504	53	183	83	211
ENSG00000236085	0	0	0	0	0	0	0	0	0	0	0	0	0	0	0	0	0	0	0	0	0	0	0	0	0	0	0	0	0	0	0	0
ENSG00000243480	0	1	0	0	0	0	0	0	0	0	1	0	0	0	0	1	0	0	0	0	0	0	0	0	0	0	0	0	0	0	0	0
ENSG00000237763	0	0	0	0	0	0	0	0	0	0	0	0	0	0	0	0	0	0	0	0	0	0	0	0	0	0	0	0	0	0	0	0
ENSG00000234441	0	0	0	0	0	0	0	0	0	0	0	0	0	0	0	0	0	0	0	0	0	0	0	0	0	0	0	0	0	0	0	0
ENSG00000174876	0	0	0	0	0	0	0	0	0	0	0	0	0	0	0	0	0	0	0	0	0	0	0	0	0	0	0	0	0	0	0	0
ENSG00000227408	0	0	0	0	0	0	0	0	0	0	0	0	0	0	0	0	0	0	0	0	0	0	0	0	0	0	0	0	0	0	0	0
ENSG00000187733	0	0	0	0	0	0	0	0	0	0	0	0	0	0	0	0	0	0	0	0	0	0	0	0	0	0	0	0	0	0	0	0
ENSG00000225191	0	0	0	0	0	0	0	0	0	0	0	0	0	0	0	0	0	0	0	0	0	0	0	0	0	0	0	0	0	0	0	0
ENSG00000215869	0	0	1	0	0	0	0	2	0	0	1	0	0	0	0	0	0	0	0	0	1	0	0	0	0	0	3	1	0	0	0	1
ENSG00000228347	0	0	0	0	0	0	0	0	0	0	0	0	0	0	0	0	0	0	0	0	0	0	0	0	0	0	0	0	0	0	0	0
ENSG00000225036	0	0	0	0	0	0	0	0	0	0	0	0	0	0	0	0	0	0	0	0	0	0	0	0	0	0	0	0	0	0	0	0
ENSG00000230768	0	0	0	0	0	0	0	0	0	0	0	0	0	0	0	0	0	0	0	0	0	0	0	0	0	0	0	0	0	0	0	0
ENSG00000227850	0	0	0	0	0	0	0	0	0	0	3	0	0	0	0	0	0	0	0	0	0	0	0	0	0	0	0	0	0	0	2	0
ENSG00000237897	0	0	0	0	0	0	0	0	0	0	0	0	0	0	0	0	0	0	0	0	0	0	0	0	0	0	0	0	0	0	0	1
ENSG00000232952	1	1	2	0	3	3	0	1	0	0	2	0	0	1	0	2	0	0	2	0	2	2	1	1	0	1	0	0	0	1	0	0
ENSG00000233047	0	0	0	0	0	0	0	0	0	0	0	0	0	0	0	0	0	0	0	0	0	0	0	0	0	0	0	0	0	0	0	0
ENSG00000237480	0	0	0	0	0	0	0	0	0	0	0	0	0	0	0	0	0	0	0	0	0	0	0	0	0	0	0	0	0	0	0	0
ENSG00000230932	0	0	0	0	0	0	0	0	0	0	0	0	0	0	0	0	0	0	0	0	0	0	0	0	0	0	0	0	0	0	0	0
ENSG00000270342	0	0	0	0	0	0	0	0	0	0	0	0	0	0	0	0	0	0	0	0	0	0	0	0	0	0	0	0	0	0	0	0
ENSG00000270976	0	0	0	0	0	0	0	0	0	0	0	0	0	0	0	0	0	0	0	0	0	0	0	0	0	0	0	0	0	0	0	0
ENSG00000233449	0	0	0	0	0	0	0	0	0	0	0	0	0	0	0	0	0	0	0	0	0	0	0	0	0	0	0	0	0	0	0	0
ENSG00000232551	0	0	0	0	0	0	0	0	0	0	0	0	0	0	0	0	0	0	0	0	0	0	0	0	0	0	0	0	0	0	0	0
ENSG00000237460	0	0	0	0	0	0	0	0	0	0	0	0	0	0	0	0	0	0	0	0	0	0	0	0	0	0	1	0	0	0	0	0
ENSG00000198890	869	597	703	628	702	773	494	825	855	671	2983	556	777	774	941	1127	389	642	635	831	1321	632	1191	557	830	1266	600	1755	964	1068	941	1127
ENSG00000162631	0	0	0	0	1	0	0	1	4	3	5	2	5	4	3	13	0	0	0	1	4	0	1	2	0	0	3	7	1	3	1	2
ENSG00000224550	0	0	0	0	0	2	1	1	0	0	0	0	0	0	0	1	2	0	0	1	2	1	0	0	0	1	0	0	0	0	0	0
ENSG00000134215	1923	1745	1662	1675	4862	4776	3891	5272	242	242	1176	206	163	172	168	247	851	1556	1269	2219	10435	4312	7935	4072	347	530	337	1273	128	211	137	198
ENSG00000275455	0	0	0	0	3	0	0	0	0	0	0	0	0	0	0	0	0	0	0	0	0	0	0	0	0	0	0	0	0	0	0	0
ENSG00000230489	7	8	11	5	2	2	2	3	2	0	3	0	0	0	1	1	6	7	17	22	18	5	8	8	0	3	2	2	0	0	0	0
ENSG00000226822	8	1	7	4	31	36	28	30	0	1	1	2	0	0	0	1	7	7	16	20	42	22	47	27	2	0	0	0	0	0	0	0
ENSG00000085491	567	420	534	458	1420	1418	1011	1510	564	488	1974	359	573	643	696	840	382	737	611	647	2868	1177	2103	1067	556	823	514	1459	458	646	611	719
ENSG00000260879	0	0	0	0	0	0	0	0	1	0	0	0	0	0	0	0	0	0	0	0	2	0	0	0	0	0	0	0	0	1	0	1
ENSG00000280186	0	3	1	0	4	2	1	11	1	2	3	0	2	1	1	6	2	1	1	0	5	2	3	1	0	1	7	2	0	9	2	1
ENSG00000196427	7	9	8	12	0	0	0	1	0	0	0	0	10	12	8	15	7	9	9	25	1	0	0	1	0	0	0	0	3	4	1	10
ENSG00000238122	0	0	0	0	0	0	0	0	0	0	0	0	1	0	2	1	0	1	1	3	0	0	0	0	0	0	2	0	0	0	0	0
ENSG00000241361	9	14	8	17	0	0	0	0	0	0	0	0	6	16	2	11	4	14	6	21	0	0	0	0	0	0	0	0	4	10	3	12
ENSG00000243967	2	0	0	0	0	0	0	0	0	0	0	0	1	2	5	6	0	0	0	1	3	0	0	0	0	0	0	0	2	4	0	4
ENSG00000186086	2	0	0	0	0	0	0	0	0	0	0	0	18	17	17	26	0	1	0	0	0	0	0	0	0	0	0	0	5	19	8	14
ENSG00000238118	0	0	0	0	0	0	0	0	0	0	0	0	3	6	4	4	0	0	0	0	0	0	0	0	0	0	0	0	4	6	7	7
ENSG00000224698	0	0	0	0	0	0	0	0	0	0	0	0	0	0	0	0	0	0	0	0	0	0	0	0	0	0	0	0	0	0	0	0
ENSG00000283354	0	0	0	0	0	0	0	0	0	0	0	0	0	0	0	0	0	0	0	0	0	0	0	0	0	0	0	0	0	0	0	0
ENSG00000227832	0	0	0	0	0	0	0	0	0	0	0	0	0	0	0	0	0	0	0	0	0	0	0	0	0	0	0	0	0	0	0	0
ENSG00000226483	0	0	0	0	0	0	0	0	0	0	2	0	0	0	0	0	0	0	0	0	0	0	0	0	0	0	0	0	0	0	0	0
ENSG00000162636	2466	1989	2044	1827	2971	2855	2121	3064	904	923	2452	535	1432	1359	1256	1703	959	1355	1248	1578	4287	1539	2916	1580	798	1117	710	2263	808	1266	909	1395
ENSG00000162639	1770	1225	1453	1261	2418	2342	2009	2526	862	686	2771	470	775	713	760	924	1800	3049	2555	2188	6091	2390	5020	2248	726	900	677	1664	807	834	889	914
ENSG00000134186	6110	3885	4886	4384	4520	4575	4098	4806	4173	3332	14912	2471	3998	3656	4765	4790	2255	2407	2317	5263	8394	4138	6084	4168	3783	5374	3608	8836	3363	4669	3770	5540
ENSG00000143107	7	10	10	7	3	1	1	6	3	5	4	0	6	4	3	5	0	3	5	5	8	2	0	2	2	3	3	1	1	3	1	5
ENSG00000232971	0	0	1	0	1	0	0	0	0	0	0	0	0	0	2	0	0	1	0	0	0	0	0	0	0	0	0	0	0	1	0	0
ENSG00000116266	1003	636	749	656	1302	1363	1020	1393	1069	939	4316	652	1090	1033	1142	1370	869	1015	907	924	3456	1443	2509	1695	1047	1457	1154	2883	1070	1171	1400	1443
ENSG00000228076	2	0	6	2	2	3	0	2	1	2	5	2	0	0	4	0	0	0	0	2	9	1	3	2	1	1	6	9	2	1	0	2
ENSG00000162641	7	2	11	4	2	0	0	0	0	0	4	0	0	0	1	0	2	3	11	4	2	0	2	1	0	0	1	7	0	0	0	0
ENSG00000274068	1	3	4	1	7	1	0	2	1	0	4	1	0	0	0	1	2	0	3	2	3	0	7	4	0	0	2	1	0	0	0	0
ENSG00000203897	0	1	0	0	0	1	1	0	0	0	1	0	0	0	0	0	1	0	0	0	0	0	0	0	0	0	2	2	0	0	0	0

ENSG00000121957	1611	1179	1358	1196	1113	1091	770	1140	717	718	2257	511	700	768	640	955	620	1023	828	1093	1936	660	1265	564	894	1265	260	2043	453	646	497	747
ENSG00000121940	588	529	534	491	611	716	521	817	172	181	610	124	168	204	170	226	258	432	392	512	1101	480	827	465	189	309	170	542	86	144	150	201
ENSG00000085433	794	600	642	531	397	422	292	423	403	333	2032	270	215	247	248	331	527	603	647	600	779	248	488	275	421	702	574	1297	243	278	342	354
ENSG00000237349	3	0	1	0	0	2	0	6	0	0	0	0	1	0	0	0	0	0	1	4	0	0	1	0	0	2	1	2	0	1	2	4
ENSG00000244716	96	59	55	54	45	46	27	64	140	77	335	65	110	91	109	133	122	125	134	99	118	48	73	54	98	143	53	190	90	93	174	152
ENSG00000228665	1	1	1	0	1	0	0	0	4	1	3	0	0	0	0	0	1	0	0	0	2	1	1	0	0	0	2	0	1	0	2	2
ENSG00000228054	1	0	0	0	0	0	0	0	0	0	0	0	0	0	0	0	0	0	0	0	0	0	0	0	0	0	1	0	0	0	0	0
ENSG00000197780	655	360	531	398	460	508	341	481	262	202	1367	136	312	240	394	349	780	918	926	612	1145	376	853	372	288	485	454	853	513	339	617	415
ENSG00000273382	2	0	7	5	3	2	4	4	14	10	90	16	16	8	17	15	4	6	5	9	17	2	3	1	21	16	21	51	12	18	20	13
ENSG00000215717	1146	832	1024	835	921	935	651	906	915	781	3700	570	706	526	718	752	721	961	985	953	1790	672	1312	677	853	1237	1021	2130	593	635	737	742
ENSG00000278249	0	0	0	0	0	0	0	0	0	0	0	0	0	0	0	0	0	0	0	0	0	0	0	0	0	0	0	0	0	0	0	0
ENSG00000270066	2	5	5	0	2	2	0	1	1	0	2	0	2	0	2	4	4	1	0	5	2	0	1	0	1	1	4	3	4	4	2	4
ENSG00000251484	10	10	12	14	15	5	2	4	3	8	18	3	7	13	17	12	2	2	9	36	16	10	13	7	6	7	9	14	7	24	11	21
ENSG00000179902	14	20	16	15	2	3	2	4	9	8	33	5	12	18	18	14	6	16	4	36	20	4	2	13	4	6	12	12	2	27	20	36
ENSG00000116299	26	50	40	47	5	5	0	3	11	9	29	7	264	316	357	453	10	30	19	33	15	5	7	1	8	12	15	27	210	342	314	450
ENSG00000031698	3835	2485	3087	2734	1792	1956	1365	1957	2167	1549	8745	1391	2725	2370	2822	3256	2346	3436	2921	2399	3500	1332	2750	1231	1840	2906	2087	5426	2322	2346	3031	2813
ENSG00000143126	1024	1352	1197	1372	390	534	262	444	909	989	6444	940	351	430	238	560	141	302	185	738	474	208	299	202	652	1325	1655	4384	135	344	118	329
ENSG00000134222	762	592	697	698	559	590	352	629	772	643	1988	522	174	253	211	335	402	414	407	495	678	310	449	258	682	1082	207	1525	128	186	156	257
ENSG00000221986	0	0	2	0	0	0	1	7	1	1	10	1	0	0	0	0	0	0	1	0	6	0	1	0	0	1	4	3	0	0	0	0
ENSG00000134243	2734	2079	2202	1889	2342	2536	1697	2638	1657	1442	8494	1324	172	195	195	246	734	1115	1057	1647	4353	1801	3252	1543	1848	2988	1542	6089	167	260	155	308
ENSG00000143106	3947	2409	3151	2557	2152	2165	1693	2236	2657	1912	9665	1780	2723	2415	2812	3116	6643	7910	7184	5974	6151	2499	4384	2971	2906	4459	1957	6129	3617	3444	4428	4638
ENSG00000143028	4	1	4	2	0	0	1	0	5	5	14	3	2	1	2	3	1	1	5	1	1	3	1	0	4	5	2	7	1	2	1	4
ENSG00000162650	477	348	372	351	343	419	291	427	367	339	1385	260	267	245	243	285	192	206	180	401	555	235	337	281	269	414	258	665	128	243	182	255
ENSG00000174151	496	434	490	387	536	499	361	564	538	464	2361	329	333	284	293	410	174	160	166	428	829	360	493	280	415	678	659	1398	214	281	173	343
ENSG00000181754	38	52	63	41	0	4	0	2	20	37	106	28	33	18	22	46	18	29	12	47	2	1	0	0	9	42	27	70	14	27	5	40
ENSG00000156097	43	54	39	29	4	10	8	12	13	13	34	7	4	4	8	10	21	25	27	28	15	6	15	4	22	18	6	25	7	17	6	4
ENSG00000254942	0	0	0	0	0	0	0	0	0	0	0	0	0	0	0	0	0	0	0	0	0	0	0	0	0	0	0	0	0	0	0	0
ENSG00000065135	2883	1959	2268	1966	2261	2303	1820	2633	2669	1983	7757	1462	2081	2110	2009	2566	1639	2416	2213	2387	5668	2143	3899	2148	2563	3614	1625	5736	1904	2329	2146	2684
ENSG00000206832	1	0	0	0	1	1	5	5	2	1	2	0	0	1	1	0	0	0	0	1	8	2	2	0	1	4	1	7	0	1	1	0
ENSG00000225113	0	0	0	0	0	0	0	0	0	0	0	0	0	0	0	0	0	0	0	0	0	0	0	0	0	0	0	0	0	0	0	0
ENSG00000134183	0	0	0	0	0	0	0	0	1	0	0	0	0	0	0	0	0	0	0	0	0	0	0	0	0	0	0	0	0	0	1	0
ENSG00000116337	1309	1001	1340	1086	1852	1984	1364	1960	1412	1150	5179	888	1783	1433	1775	1898	500	584	590	1257	2546	1061	1872	1092	1091	1785	1114	2988	1345	1751	1332	1900
ENSG00000228703	0	0	0	0	0	0	0	0	0	0	0	0	0	0	0	0	0	0	0	0	0	0	0	0	0	0	0	0	0	0	0	0
ENSG00000235045	9	5	4	3	0	1	1	0	1	2	1	0	3	1	0	0	3	0	0	0	0	1	0	0	0	0	0	1	0	0	0	1
ENSG00000168765	720	602	640	546	376	384	319	435	431	264	1163	242	256	251	204	312	482	689	576	496	797	279	597	244	251	418	311	877	145	202	181	254
ENSG00000213366	221	217	226	215	60	66	50	76	198	205	630	110	74	63	43	87	57	56	54	169	47	28	36	31	68	114	132	294	11	31	12	46
ENSG00000134184	530	398	486	436	228	221	168	268	652	481	1554	393	0	0	0	0	649	1005	707	499	404	133	283	110	511	689	280	1125	0	0	0	0
ENSG00000260246	10	2	9	6	1	0	0	1	9	2	20	4	0	0	0	0	8	4	4	10	5	1	0	1	5	14	8	15	0	0	0	0
ENSG00000134201	24	14	5	25	1	2	0	1	2	5	3	2	1	0	0	0	7	6	6	7	1	0	0	0	1	3	1	2	0	2	0	0
ENSG00000241720	2	2	2	0	0	0	0	0	0	0	0	0	0	0	0	0	1	0	0	0	0	0	0	1	0	0	1	0	0	0	0	0
ENSG00000134202	497	398	399	413	244	298	156	308	4	4	8	1	0	1	0	0	327	610	446	415	405	187	278	209	1	6	4	13	0	0	0	0
ENSG00000198758	2	0	2	0	0	2	0	2	0	0	0	1	0	0	0	0	1	0	3	1	0	0	0	1	1	0	0	0	0	0	0	0
ENSG00000224927	0	0	0	0	9	6	3	7	32	26	74	14	0	0	0	4	0	0	0	0	8	2	8	3	7	5	3	25	0	0	1	0
ENSG00000228420	0	0	0	0	9	21	7	10	91	62	122	29	1	0	0	1	0	0	0	0	5	1	8	3	14	29	2	34	0	1	0	1
ENSG00000235005	0	0	0	0	2	1	0	1	4	1	3	0	0	0	0	0	0	0	0	0	0	1	1	0	1	6	0	0	0	0	0	0
ENSG00000261055	0	0	0	0	0	2	0	1	1	2	0	0	0	0	0	0	0	0	0	0	0	0	0	0	0	2	0	2	0	0	0	0
ENSG00000184371	158	139	159	91	1101	1390	714	1423	11719	8799	29305	5884	91	114	108	168	61	52	64	121	1664	961	1064	724	5208	9516	2386	10252	169	321	182	399
ENSG00000168710	4063	3371	3507	3330	4544	4936	3379	5094	3872	2906	10755	2111	2848	2812	2853	3539	943	1593	1299	2284	6262	2622	4506	2404	2762	4527	1780	6132	1892	2551	1947	2884
ENSG00000143093	1290	986	1241	1003	1334	1429	1013	1465	669	659	3105	503	897	760	878	1008	347	466	400	925	2158	998	1559	952	660	1129	834	1820	604	942	648	975
ENSG00000258634	278	168	235	235	204	198	161	214	118	115	563	72	296	259	256	351	73	61	58	158	312	139	191	155	115	174	135	333	125	265	145	234
ENSG00000156150	2	0	0	1	2	3	0	4	0	0	0	0	1	2	0	0	0	0	0	4	2	0	1	2	0	1	0	0	1	0	0	0
ENSG00000258673	3	4	2	6	8	10	4	5	0	1	11	1	1	0	0	1	1	1	1	2	4	2	4	2	0	2	1	1	0	5	3	1
ENSG00000186150	1	0	0	0	2	1	0	2	0	0	1	0	0	1	0	0	1	1	0	0	1	1	0	1	0	0	0	0	0	1	0	0
ENSG00000197106	0	3	0	2	1	1	0	0	0	1	1	0	0	0	0	0	0	0	0	0	2	2	1	0	0	0	0	0	0	0	0	0
ENSG00000227091	0	0	0	0	0	0	0	0	0	0	0	0	0	0	0	0	0	0	0	0	0	0	0	0	0	0	0	0	0	0	0	0
ENSG00000235526	0	0	0	0	0	0	0	0	0	0	0	0	0	0	0	0	0	0	0	0	0	0	0	0	0	0	0	0	0	0	0	0
ENSG00000224965	7	3	13	2	7	0	6	1	3	1	0	0	6	3	2	5	3	1	9	1	8	4	9	2	4	4	1	0	3	3	6	8
ENSG00000116396	1249	1287	1434	1392	796	954	634	959	280	239	508	154	846	897	613	995	254	321	225	1057	1302	657	732	665	132	285	73	351	247	613	209	631
ENSG00000282852	0	0	0	1	1	0	1	0	0	0	0	0	1	0	0	0	0	0	0	0	1	0	0	0	0	0	0	0	0	0	0	0
ENSG00000200536	2	0	1	1	0	0	2	3	2	0	3	0	1	0	0	0	0	0	0	2	1	0	1	0	0	2	0	0	0	0	0	0
ENSG00000227963	16	23	15	8	20	22	14	24	12	12	48	16	12	11	14	10	5	8	14	28	61	14	21	17	15	34	7	24	12	21	16	21
ENSG00000162775	2106	1736	1784	1839	1313	1441	1000	1547	933	830	2852	612	1337	1154	1124	1423	537	628	632	1469	2190	1056	1416	1045	973	1252	546	2510	632	935	744	1183
ENSG00000224699	409	311	300	277	252	308	219	281	162	159	640	129	112	145	115	151	73	92	58	184	341	130	200	148	167	246	121	543	47	113	78	125
ENSG00000168679	277	201	253	176	200	219	191	230	193	176	665	120	91	81	79	94	37	56	36	130	278	150	141	144	204	219	142	545	30	76	64	83
ENSG00000273373	910	714	664	716	789	753	546	899	537	497	1747	321	214	222	166	269	121	139	125	393	901	423	467	442	399	650	270	1198	66	228	104	252
ENSG00000134248	1959	1194	1541	1423	1436	1378	987	1462	1120	802	4159	754	1229	1081	1162	1256	2538	2595	2597	2465	3309	1403	2420	1727	1122	1618	1028	2531	1609	1347	1642	1710
ENSG00000143125	2	1	1	3	0	0	0	0	1	0	0	0	1	2	3	2	1	0	1	1	0	0	0	0	0	0	3	0	0	1	3	0
ENSG00000270380	0	0	0	0	0	0	0	0	0	0	0	0	0	0	0	0	0	0	0	0	0	0	0	0	0	0	0	0	0	0	0	0
ENSG00000282887	0	0	0	0	0	0	0	0	0	0	0	0	0	0	0	0	0	0	0	0	0	0	0	0	0	0	0	0	0	0	0	0
ENSG00000240194	0	0	0	0	0	0	0	0	0	0	0	0	5	4	5	6	0	0	0	0	0	0	0	0	0	0	0	0	0	3	3	2
ENSG00000235407	0	0	0	0	0	0	1	0	0	0	0	0	23	21	16	28	0	0	0	0	0	0	0	0	0	0	0	0	4	9	8	6
ENSG00000143105	0	0	0	0	0	0	0	0	0	0	0	0	0	1	0	0	0	0	0	0	0	0	0	0	0	0	0	0	0	0	0	0
ENSG00000177301	0	8	4	3	1	7	0	3	3	2	0	0	6	6	10	5	0	0	0	2	8	1	1	5	1	0	0	1	0	2	1	4
ENSG00000259834	1362	1142	1205	1308	115	105	68	87	6	10	11	1	187	241	263	301	232	422	292	698	130	34	77	57	8	13	3	12	75	170	152	250
ENSG00000177272	751	711	650	796	57	67	42	49	8	3	1	3	116	120	153	209	109	182	132	379	61	39	46	37	3	5	0	8	40	104	91	119
ENSG00000199710	0	0	0	0	0	0	0	0	0	0	0	0	0	0	0	0	0	0	0	0	0	0	0	0	0	0	2	0	0	0	0	0
ENSG00000237415	1	0	0	1	0	0	1	0	0	1	2	0	0	0	2	0	0	0	0	0	2	1	0	0	0	0	0	1	0	0	0	0
ENSG00000232791	0	3	0	0	0	0	0	0	0	0	1	0	0	0	0	0	0	0	0	1	0	0	0	0	0	0	0	1	0	0	0	0
ENSG00000143119	2075	1359	1870	1239	1461	1762	1236	1958	2659	2296	9229	1825	1427	1439	1495	1932	1276	1623	1616	1345	2286	973	1893	846	1859	2709	1907	4892	1336	1427	1526	1873
ENSG00000261654	128	99	102	111	36	47	29	45	51	51	234	38	105	99	84	106	32	41	25	84	70	16	29	28	39	68	81	145	27	54	50	72
ENSG00000232811	71	59	63	66	37	45	25	39	36	29	153	24	63	84	80	76	20	22	12	51	47	26	40	19	39	61	45	97	32	41	45	50
ENSG00000121931	1497	856	1254	940	683	742	535	868	875	726	3034	529	738	862	978	1102	1533	1886	1628	1353	1860	688	1238	724	887	1097	908	1979	896	928	1120	1139
ENSG00000273010	22	16	17	17	12	8	13	17	16	12	46	9	11	10	9	17	9	9	13	24	22	14	18	4	15	15	17	25	7	18	7	15
ENSG00000225672	0	0	0	0	0	0	0	0	0	0	1	0	0	0	0	0	0	0	0	0	0	0	1	0	0	0	0	0	0	0	1	0
ENSG00000156171	1160	882	836	801	594	597	426	595	758	690	4222	649	649	680	637	800	1028	1128	846	1008	963	426	653	495	661	956	964	2490	542	603	683	764
ENSG00000134255	1506	993	1146	1079	935	1013	763	1096	973	817	6452	897	765	711	802	860	802	1115	821	1310	2148	948	1378	972	887	1174	1462	3801	602	834	784	972
ENSG00000272982	0	0	0	0	0	0	0	0	0	0	0	0	0	0	0	0	0	0	0	0	0	0	0	0	0	0	0	0	0	0	0	0
ENSG00000273221	0	0	0	0	0	0	0	0	0	0	0	0	0	0	0	0	0	0	0	0	0	0	0	0	0	0	0	0	0	0	0	0
ENSG00000162777	1711	1195	1408	1190	46	47	44	76	507	458	4949	734	203	190	237	212	924	1205	1097	1410	67	44	50	33	270	411	667	2525	162	206	188	202
ENSG00000064886	8367	4791	3559	3852	4	6	5	8	13	5	392	41	10	8	4	5	5056	5656	2259	3247	5	3	4	3	7	24	146	366	5	5	8	13
ENSG00000236040	12	7	10	12	0	0	0	0	0	0	3	2	0	0	0	0	17	6	0	2	0	0	0	0	0	0	2	1	0	0	0	0
ENSG00000203878	1	0	0	3	0	0	0	0	0	0	1	0	0	0	0	0	1	1	0	0	0	0	0	0	0	0	0	0	0	0	0	0
ENSG00000134216	0	0	0	0	0	0	0	0	0	0	0	0	1	0	2	0	0	0	0	1	0	0	0	0	0	0	1	1	0	0	1	0
ENSG00000229283	0	0	0	0	0	0	0	0	0	0	0	0	0	0	0	0	0	0	0	0	0	0	0	0	0	0	0	0	0	0	0	0
ENSG00000232240	0	0	0	0	0	0	0	0	0	0	0	0	0	0	0	0	0	0	0	0	0	0	0	0	0	0	0	0	0	0	0	0
ENSG00000173947	4	14	8	5	0	2	1	0	0	4	76	8	6	14	3	3	2	1	10	11	1	1	0	1	1	3	18	49	6	10	14	12
ENSG00000234020	1	0	1	0	0	0	0	0	0	0	0	0	0	0	0	0	0	0	0	0	0	0	0	0	0	0	1	1	0	0	0	0
ENSG00000236012	0	0	0	0	0	0	0	0	0	0	0	0	0	0	0	0	0	0	0	0	0	0	0	0	0	0	0	0	0	0	0	0
ENSG00000227179	0	3	3	0	0	0	0	0	0	0	1	0	0	1	0	0	0	0	0	1	0	0	0	0	0	0	0	0	0	0	0	0
ENSG00000085465	166	158	132	142	92	68	71	98	38	27	253	20	124	111	80	148	45	55	59	154	109	59	77	87	25	35	67	100	28	71	37	114
ENSG00000260948	117	101	101	126	72	82	55	84	30	26	177	19	60	93	49	95	31	40	22	96	113	45	48	57	25	46	66	120	22	78	29	57
ENSG00000233337	34	23	31	28	24	32	29	37	10	11	123	19	26	29	35	22	16	12	10	28	59	22	17	44	16	52	20	78	9	33	14	37
ENSG00000243960	30	16	27	24	23	20	10	22	9	13	53	7	9	11	17	22	4	13	9	19	43	8	19	24	9	26	14	43	6	21	16	25
ENSG00000116455	1340	877	1183	966	941	997	715	994	1042	833	5342	737	1018	883	1180	1172	702	1021	929	1410	1722	775	1339	747	1285	1939	990	3100	1065	1397	1025	1381
ENSG00000199890	3	2	3	2	1	4	5	5	4	1	7	2	4	2	2	3	2	0	2	6	4	1	3	2	3	6	4	9	1	8	6	5
ENSG00000116459	3010	1962	2338	1906	3065	3048	2304	3449	3287	2357	10364	2024	3346	2942	3796	4007	4121	5182	4555	3480	8381	3221	5731	3703	3360	4930	2388	7737	4682	4100	5702	5322
ENSG00000143110	40	44	21	33	43	36	29	43	31	45	197	34	27	20	21	15	15	12	13	34	47	20	17	25	46	35	46	126	15	11	12	27
ENSG00000121933	0	2	0	0	9	7	8	8	0	0	1	0	0	0	0	0	0	0	0	0	13	8	5	9	0	0	0	0	0	0	0	1
ENSG00000200360	0	0	0	0	0	0	0	1	0	0	0	0	0	0	0	0	0	0	0	0	0	0	0	2	0	0	0	0	0	0	0	0
ENSG00000282608	0	0	0	3	20	14	25	20	2	0	1	0	0	1	0	0	0	0	0	3	33	16	18	10	0	2	3	0	0	0	0	0
ENSG00000116473	1656	1126	1231	1152	2113	2222	1616	2495	1949	1572	6607	1184	1809	1651	1878	2113	1331	1883	1480	1682	4691	1965	3294	1994	1720	2614	1773	4555	1596	1681	1787	2023
ENSG00000231346	0	2	0	3	6	3	5	4	8	5	32	3	7	14	10	7	1	1	0	2	8	9	2	3	3	2	8	14	5	2	4	6
ENSG00000215867	4	10	5	5	11	7	9	15	13	5	36	5	6	5	5	10	1	1	2	14	19	11	10	4	18	13	8	30	2	4	8	9
ENSG00000201028	2	1	3	1	1	3	0	5	5	3	10	1	0	1	2	3	0	2	0	0	5	5	3	3	2	2	4	16	1	0	5	1
ENSG00000197852	214	290	210	290	322	377	227	381	53	58	2262	274	234	320	243	386	58	116	89	226	465	192	359	177	74	113	560	1461	123	220	139	270
ENSG00000227811	0	6	3	2	2	5	3	3	0	1	13	1	1	0	0	3	0	0	1	1	1	2	3	1	2	0	2	3	0	0	0	2
ENSG00000064703	1062	684	885	658	965	909	692	970	729	602	2444	508	749	688	968	932	754	909	922	992	2247	957	1744	841	873	1299	527	1991	868	1028	1107	1164
ENSG00000171385	8	2	3	2	5	5	5	6	1	3	9	2	3	2	1	3	0	2	0	10	15	7	11	9	7	8	3	6	1	3	2	4
ENSG00000232558	0	0	0	0	0	0	0	0	0	0	0	0	0	0	0	0	0	0	0	0	0	0	0	0	0	0	0	0	0	0	0	0
ENSG00000237556	0	0	0	0	0	0	0	0	0	0	0	0	0	0	2	0	0	0	0	0	0	0	0	0	0	0	0	1	0	0	0	0
ENSG00000231437	0	0	2	0	0	0	0	0	0	0	1	0	0	0	0	0	0	0	0	0	0	0	0	0	0	0	2	0	0	0	0	0
ENSG00000231246	0	0	0	0	0	0	0	0	0	0	0	0	0	0	0	0	0	0	0	0	0	0	0	0	0	0	0	0	0	0	0	0
ENSG00000234388	0	0	0	0	0	0	0	0	0	0	0	0	0	0	0	0	0	0	0	0	0	0	0	0	0	0	0	0	0	0	0	0
ENSG00000238761	0	0	0	0	0	0	0	0	0	0	0	0	0	0	0	0	0	0	0	0	0	0	0	0	0	0	0	0	0	0	0	0
ENSG00000143079	379	302	373	304	215	277	176	260	115	96	670	106	1088	1156	1263	1389	156	212	168	207	352	125	242	117	114	159	136	286	695	940	928	1034
ENSG00000283296	0	0	0	0	0	0	0	0	1	0	0	0	0	0	1	3	0	0	0	0	0	2	0	0	0	0	2	1	0	2	1	0
ENSG00000134245	65	100	76	104	39	55	35	58	52	28	202	39	50	72	56	82	23	38	30	106	34	26	21	21	36	33	40	92	19	64	29	51
ENSG00000273483	3	3	1	3	1	3	4	5	0	1	14	1	4	0	3	3	1	1	2	2	1	1	2	0	2	1	3	6	5	2	2	5
ENSG00000007341	391	306	307	285	249	271	196	281	243	179	865	143	174	152	163	177	208	300	261	370	480	226	332	196	213	301	175	557	83	191	130	182
ENSG00000116489	8282	5137	6907	4789	5843	5700	4323	6289	5806	4451	20345	3363	5483	4877	5847	6306	9468	11775	11612	8602	15989	6291	10565	7036	5845	8036	4432	13125	6173	5948	7574	6982
ENSG00000235299	6	2	4	3	4	4	4	4	4	6	8	6	5	8	3	4	0	0	1	6	10	4	7	7	3	9	1	14	0	3	1	3
ENSG00000252750	1	1	3	3	1	2	0	3	1	0	5	0	2	0	1	4	0	0	0	3	2	0	0	0	4	2	2	5	0	1	1	4
ENSG00000155363	2442	1972	2196	2061	2657	3006	2280	3197	1488	1208	5664	1055	889	953	877	1309	612	650	515	1205	3647	1645	2771	1573	1026	1820	1018	3438	622	1052	616	1100
ENSG00000225075	18	26	37	19	25	16	20	20	16	9	63	12	2	7	4	7	5	6	6	7	46	28	18	24	9	16	25	33	6	18	3	15
ENSG00000155366	1890	1373	1427	1397	1472	1523	978	1552	2665	2329	14517	2278	1228	1095	1183	1624	1002	1521	1055	1126	2311	907	1732	832	2621	3937	3431	8209	999	1092	1064	1229
ENSG00000271810	2	0	1	1	0	1	0	0	0	0	4	0	0	1	0	2	0	0	0	3	0	0	0	0	0	1	1	1	0	0	0	0
ENSG00000155367	4	18	4	20	3	7	1	3	7	8	26	6	16	19	6	24	2	8	1	6	3	2	0	1	0	3	3	12	2	8	1	10
ENSG00000261595	0	0	0	2	0	1	1	3	0	1	1	0	0	0	0	0	0	0	0	1	0	0	0	0	0	0	0	3	0	0	1	0
ENSG00000184599	6	2	0	4	2	4	0	0	2	0	1	0	0	4	1	3	0	2	2	5	0	0	0	0	0	0	0	0	0	0	0	0
ENSG00000234790	0	0	0	0	0	0	0	0	0	0	0	0	0	0	0	0	0	0	0	0	0	0	0	0	0	0	0	0	0	0	0	0
ENSG00000215866	0	0	0	1	0	0	0	0	0	0	3	0	0	0	0	0	0	1	1	1	1	0	0	0	0	0	1	0	0	0	0	1
ENSG00000224167	0	0	0	0	0	0	0	0	0	0	1	0	0	0	0	0	0	0	0	0	0	0	0	0	0	0	0	0	0	0	0	0
ENSG00000228040	0	1	1	0	1	0	0	0	1	1	0	0	0	0	0	0	0	2	0	0	0	0	0	0	0	1	0	0	0	0	0	1
ENSG00000155380	4831	3227	3992	3024	2755	2734	2064	2947	2657	1943	6429	1355	7523	6628	7360	8293	5326	7656	6345	5682	6918	2673	4499	2448	2664	3830	1246	4696	6200	5750	7574	6821
ENSG00000229020	17	12	22	16	9	9	3	17	12	10	29	2	29	24	35	21	1	4	3	21	23	10	11	12	16	17	2	13	15	15	15	36
ENSG00000226419	497	350	434	396	199	206	197	248	515	381	1629	327	594	563	645	661	144	165	134	392	290	121	229	128	377	493	337	972	277	557	414	695
ENSG00000238198	30	24	30	27	12	9	4	10	49	27	145	22	50	35	49	65	4	16	11	41	10	5	4	6	29	43	35	108	12	52	27	68
ENSG00000198799	976	671	849	650	551	616	454	682	593	558	2322	347	537	511	524	625	262	302	304	685	1121	458	706	466	533	759	747	1579	302	500	472	584
ENSG00000237278	0	0	0	0	0	0	0	0	0	0	0	0	0	0	0	0	0	0	0	0	0	0	0	0	0	0	0	0	0	0	0	0
ENSG00000233839	0	0	0	0	0	0	0	0	0	0	1	0	0	0	0	0	0	0	0	0	0	0	0	0	0	0	0	0	0	0	0	0
ENSG00000236887	0	0	0	0	0	0	0	0	0	0	0	0	0	0	0	0	0	0	0	0	0	0	0	0	0	0	0	0	0	0	0	0
ENSG00000081026	501	536	522	597	581	680	456	729	648	598	3054	476	197	258	178	364	131	207	164	391	876	396	577	327	753	1086	1801	2939	89	183	80	171
ENSG00000232499	11	12	8	11	8	12	7	9	8	6	25	6	3	4	4	11	5	8	5	10	20	6	10	4	9	14	13	49	6	9	10	18
ENSG00000238256	1	0	0	0	0	2	0	3	1	1	0	0	0	0	0	0	0	1	0	0	1	1	0	0	1	3	0	1	0	0	1	0
ENSG00000116793	20	14	17	9	1485	1438	1231	1743	450	332	1999	312	422	367	464	555	3	0	3	12	2866	1317	2022	1178	517	721	492	1494	341	472	389	566
ENSG00000232450	2	1	3	8	31	21	16	36	8	6	45	4	5	9	6	8	0	0	1	1	29	19	10	22	7	23	16	33	2	11	12	11
ENSG00000081019	2489	1639	1722	1517	1274	1211	944	1321	923	763	2825	559	1002	853	793	984	612	811	746	1079	1806	751	1238	769	810	1124	789	1872	532	754	521	728
ENSG00000231128	9	5	11	5	12	12	15	18	37	30	155	17	34	12	22	25	0	4	5	4	26	16	10	19	33	30	49	81	9	23	9	15
ENSG00000134242	389	119	410	131	978	974	853	1175	2080	1662	10080	1377	1062	938	1179	1292	575	356	728	379	2364	1033	1847	1207	1915	2598	2913	5741	1000	1074	1290	1412
ENSG00000226167	77	36	65	37	78	80	58	68	71	79	393	36	56	44	66	56	18	17	15	53	110	61	51	60	52	88	132	226	16	59	26	57
ENSG00000188761	34	22	22	21	24	25	14	22	27	17	100	16	18	21	15	32	14	11	30	50	60	23	24	35	24	26	20	54	16	25	14	37
ENSG00000134262	908	662	832	671	856	853	652	905	679	575	2610	376	470	412	448	545	447	597	552	716	1389	672	990	643	543	756	639	1472	343	539	403	531
ENSG00000118655	929	658	853	654	896	899	781	1012	715	561	1832	483	656	559	761	746	352	523	634	666	1579	644	1567	611	660	1091	268	1582	611	670	574	717
ENSG00000235527	18	11	16	18	2	3	0	2	14	6	31	2	16	6	5	16	6	5	7	17	5	2	1	1	3	1	10	16	3	6	4	15
ENSG00000163349	3814	2780	3244	2577	3864	3624	2804	3903	3254	2855	13884	2310	3235	3111	3078	3630	699	882	1058	1494	6037	2567	4229	2319	3261	4998	3518	9716	2054	2890	2230	3200
ENSG00000116774	2	2	2	0	4	0	1	3	1	0	4	0	1	0	2	1	0	0	0	1	2	1	3	2	1	2	2	7	0	2	0	1
ENSG00000270780	0	0	0	1	0	0	0	0	0	0	1	0	0	0	0	0	0	0	0	0	0	0	0	0	0	0	0	0	0	0	0	0
ENSG00000282048	0	0	0	0	0	0	0	0	0	0	0	0	0	0	0	0	0	0	0	0	0	0	0	0	0	0	0	0	0	0	0	0
ENSG00000134207	1	0	0	0	0	0	0	0	145	171	644	92	26	17	28	25	1	0	0	0	0	0	0	0	90	127	172	355	15	19	5	16
ENSG00000232895	0	0	0	0	0	0	0	0	17	21	114	22	3	1	6	4	0	0	0	0	0	0	0	0	14	18	29	60	0	3	2	5
ENSG00000273698	0	0	0	0	0	0	0	0	1	1	8	3	0	0	1	1	0	0	0	0	0	0	0	0	3	0	3	2	0	0	0	2
ENSG00000271419	0	0	0	0	0	0	0	0	10	7	40	7	1	1	2	0	0	0	1	1	0	0	0	0	6	9	5	44	0	1	2	0
ENSG00000197323	3801	2659	3231	2624	3266	3196	2573	3498	2622	2104	11272	1636	2157	1895	2137	2266	1112	1713	1732	2647	5890	2329	4259	2445	2795	3928	2621	7256	1355	2098	1675	2356
ENSG00000226984	2	4	2	1	2	5	5	3	6	5	15	1	5	2	5	5	2	1	2	6	14	7	4	11	8	5	2	15	0	6	5	6
ENSG00000225650	1	1	6	0	2	2	2	3	6	1	4	1	1	1	1	3	1	1	0	8	4	2	4	3	7	2	1	14	0	0	5	3
ENSG00000201114	0	2	0	1	0	3	0	0	0	2	7	0	2	0	0	0	0	0	2	0	3	2	3	2	3	1	4	6	2	2	0	0
ENSG00000236480	5	1	6	1	0	0	3	0	1	3	7	0	1	0	0	1	0	1	0	1	3	0	0	0	0	2	0	2	0	1	0	2
ENSG00000116752	1281	781	1075	830	828	901	735	974	830	616	2610	472	821	748	1030	1081	2335	2683	2698	2187	3104	1203	2082	1571	758	1173	566	1699	1305	1244	1713	1609
ENSG00000175984	7	10	6	8	6	0	3	4	25	33	260	22	2	16	2	9	4	3	6	8	6	2	2	6	18	47	74	114	0	3	8	7
ENSG00000116748	0	0	0	1	0	0	0	0	0	0	2	0	1	0	1	0	0	3	0	0	0	0	1	0	0	1	2	1	0	0	0	1
ENSG00000242769	0	0	0	0	0	0	0	0	0	0	1	0	0	0	0	0	0	0	0	0	0	0	0	0	0	0	0	0	0	0	0	0
ENSG00000213281	3007	1996	2705	2093	3452	3446	2747	3546	2381	1945	8313	1385	2746	2508	2794	3114	3317	4249	3772	3027	8189	3107	5857	3018	2512	3506	1888	5452	2454	2578	2781	2957
ENSG00000009307	18518	12889	14316	12916	15725	15824	11458	16647	11310	8552	35950	6377	11115	9886	11119	12796	12013	18150	15919	14541	32003	12438	23277	10807	10456	15225	7891	22893	10435	10874	11625	12457
ENSG00000201900	10	6	14	10	8	4	9	6	4	12	32	5	7	8	7	9	0	1	1	15	23	16	11	14	8	16	10	32	2	6	7	9
ENSG00000052723	3096	2070	2524	2036	1565	1750	1339	1859	1659	1370	5726	995	2141	2050	2169	2667	1824	2212	2097	2906	3652	1619	2699	1590	1810	2391	1340	3779	1724	2158	1910	2454
ENSG00000227970	0	0	0	0	0	0	0	0	0	0	0	0	0	0	0	1	0	0	0	0	0	0	0	0	0	0	0	0	0	0	0	0
ENSG00000198765	1	0	0	0	0	0	0	0	0	0	0	0	1	0	0	0	0	0	0	0	1	0	0	0	0	0	1	0	0	0	0	0
ENSG00000134200	0	0	2	1	6	0	0	1	0	0	0	0	7	4	4	4	1	0	0	1	0	0	1	0	0	0	0	0	3	0	0	1
ENSG00000134198	310	234	261	279	302	303	256	337	4	9	3	2	756	683	666	908	53	172	72	222	446	179	338	180	3	14	1	6	266	385	299	431
ENSG00000233730	0	2	1	1	5	5	4	1	2	0	7	0	0	0	6	2	0	3	0	0	8	3	6	10	2	0	0	5	0	0	1	2
ENSG00000228035	0	0	0	0	0	0	0	0	0	0	0	0	0	0	0	0	0	0	0	0	0	0	0	0	0	0	0	0	0	0	0	0
ENSG00000134259	0	0	0	0	0	0	0	0	0	0	0	0	0	0	0	0	0	0	0	0	0	0	0	0	0	0	0	0	0	0	0	0
ENSG00000226973	0	0	0	1	1	2	1	0	0	0	0	0	0	0	0	0	0	0	0	1	0	1	0	0	0	0	0	0	0	0	0	0
ENSG00000274468	0	0	0	0	0	0	0	0	0	0	0	0	0	0	0	0	0	0	0	2	0	0	0	0	0	0	0	0	0	0	0	0
ENSG00000229101	0	0	1	0	0	0	1	2	0	0	0	0	0	0	0	0	0	0	0	1	1	3	0	0	0	0	0	0	0	0	0	0
ENSG00000232185	0	0	0	0	0	2	0	0	0	0	0	0	0	0	0	0	0	0	0	0	0	1	0	0	0	0	0	0	0	0	0	0
ENSG00000270631	0	0	2	0	0	0	0	0	0	0	0	0	0	0	0	0	0	0	0	0	0	0	0	0	0	0	0	0	0	0	0	0
ENSG00000239984	0	0	0	0	1	0	0	1	0	0	0	0	0	0	0	0	0	0	0	0	0	0	0	0	0	0	0	0	0	0	0	0
ENSG00000207502	0	0	0	0	0	0	0	0	0	0	0	0	0	0	0	0	0	0	0	0	0	0	0	0	0	0	0	0	0	0	0	0
ENSG00000173218	1197	879	1215	860	1523	1509	1140	1555	1197	1038	4083	880	643	569	541	760	459	833	861	1472	2747	1088	1931	861	1056	1814	761	3061	463	591	379	602
ENSG00000118729	0	0	1	0	0	0	0	1	6	3	11	3	0	0	0	0	0	0	0	0	5	0	1	0	0	1	1	5	0	0	0	0
ENSG00000177551	0	0	0	1	0	1	0	1	2	0	23	1	0	0	0	0	0	0	0	0	0	0	0	0	2	0	3	7	0	0	0	0
ENSG00000214204	0	0	0	0	0	0	1	1	0	1	0	0	0	0	0	0	0	0	0	0	1	0	0	0	0	1	0	0	0	0	0	0
ENSG00000271143	0	0	0	0	1	0	0	0	0	1	0	0	0	0	0	0	0	0	0	0	0	0	2	0	0	0	0	0	0	1	0	1
ENSG00000228127	0	0	0	1	0	1	2	0	0	0	11	0	0	4	3	3	0	0	0	0	3	0	1	0	0	0	3	5	1	3	0	2
ENSG00000163393	0	0	0	2	0	0	0	0	269	276	1598	198	183	186	251	232	0	0	0	0	0	0	0	0	230	367	400	872	108	183	136	253
ENSG00000237993	0	0	0	0	0	0	0	0	0	2	12	4	1	1	1	3	0	0	0	0	0	0	0	0	0	4	5	5	2	0	2	1
ENSG00000173212	0	0	0	0	0	0	0	0	6	9	28	2	0	1	2	2	0	0	0	0	0	0	0	0	2	2	8	9	0	2	0	0
ENSG00000235933	0	0	0	0	0	0	0	0	1	0	0	0	0	0	0	0	0	0	0	0	0	0	0	0	0	0	0	4	0	0	0	0
ENSG00000221040	0	0	0	0	0	0	0	0	0	0	0	0	0	0	0	0	0	0	0	0	0	0	0	0	0	0	0	0	0	0	0	0
ENSG00000163399	9081	7133	7369	6986	5837	6310	4443	6706	8268	6857	28639	5527	7591	7069	7300	9098	2370	4202	3587	5700	10068	4238	7420	3529	7525	12646	6601	19808	5649	7636	5876	7783
ENSG00000203865	70	61	70	54	28	51	25	41	101	65	273	57	46	53	30	40	35	43	46	64	67	26	36	23	55	74	67	181	19	43	22	49
ENSG00000212385	3	5	2	3	2	0	2	0	4	0	7	2	5	3	4	1	8	2	5	5	3	3	5	1	2	1	5	4	1	1	5	5
ENSG00000233154	26	20	27	18	3	4	4	1	3	16	29	4	7	10	6	11	5	9	6	8	2	5	0	0	9	6	16	24	0	1	0	3
ENSG00000230381	3	3	6	3	0	0	0	0	0	0	0	1	0	0	0	0	1	0	0	1	0	0	0	0	0	0	0	0	0	0	0	0
ENSG00000200547	2	0	3	0	0	0	1	0	1	0	1	0	0	0	0	0	0	0	0	1	0	0	0	0	0	0	0	0	0	0	0	2
ENSG00000224950	68	46	92	38	55	72	34	57	102	116	660	82	47	48	32	50	28	18	23	92	51	27	24	28	85	138	142	326	19	49	17	48
ENSG00000116815	532	458	411	363	410	445	280	405	358	289	1420	194	172	195	174	217	332	420	430	547	897	380	477	423	279	418	361	847	119	111	120	180
ENSG00000177173	50	55	39	64	61	58	43	57	23	28	112	12	17	9	15	17	18	12	21	65	89	42	66	51	15	46	21	79	6	24	10	22
ENSG00000264419	1	2	0	2	0	2	0	2	0	1	4	0	1	1	0	1	0	1	0	3	3	2	4	0	3	1	0	3	0	1	0	0
ENSG00000143061	97	337	122	290	338	420	221	349	52	95	322	75	101	168	53	191	21	110	24	179	344	173	218	117	62	147	141	352	12	56	12	59
ENSG00000211543	0	0	0	0	0	0	0	0	0	0	0	0	0	0	0	0	0	1	0	0	0	0	0	0	0	0	0	0	0	0	0	0
ENSG00000203864	0	0	0	0	0	0	0	0	0	0	0	0	0	0	0	0	0	0	1	1	0	0	0	0	0	0	0	0	0	0	0	0
ENSG00000231072	0	0	0	0	1	0	0	0	0	0	0	1	1	0	0	0	0	0	0	0	0	0	0	0	0	0	0	0	0	0	0	0
ENSG00000223489	0	0	0	0	0	0	0	0	0	0	1	0	0	0	0	0	0	0	0	0	0	0	0	0	0	0	2	1	0	1	1	0
ENSG00000116824	3384	2451	3223	2130	0	0	2	0	1439	1097	10187	1621	0	1	0	0	1805	1843	2385	2275	0	0	2	0	1576	2064	3490	7547	0	0	0	0
ENSG00000225079	1	0	0	1	0	0	0	0	0	0	15	3	0	0	0	0	0	2	2	0	0	0	0	0	0	3	5	13	0	0	0	0
ENSG00000272715	0	0	0	0	0	0	0	0	0	2	18	2	0	0	0	0	0	0	0	0	0	0	0	0	3	1	12	4	0	0	0	0
ENSG00000134247	8	4	8	4	2	1	1	2	165	154	1342	221	7	17	16	36	3	4	5	1	3	3	3	1	324	533	585	1677	0	20	1	10
ENSG00000134256	6	8	5	5	6	5	6	4	14	13	28	6	2	1	4	6	1	2	2	8	5	3	6	6	14	19	18	38	2	3	4	4
ENSG00000236137	29	26	32	25	9	13	7	18	35	17	84	17	15	21	16	15	31	44	42	52	20	11	9	12	25	38	11	86	6	11	11	20
ENSG00000116830	3506	2352	2932	2588	2558	2571	2075	2746	2758	2158	8947	1767	3034	2822	2971	3682	1276	1667	1525	2464	4369	1940	2931	1822	3018	4547	1302	7586	1714	2801	1945	2964
ENSG00000215930	5	4	1	6	4	2	1	3	4	5	5	0	4	5	5	5	2	4	0	2	7	4	1	2	4	3	1	9	1	8	5	4
ENSG00000134253	74	64	53	87	297	347	255	411	90	70	365	50	71	82	77	115	14	34	25	89	406	235	268	233	56	81	57	235	17	80	46	131
ENSG00000228453	0	0	0	2	0	0	0	0	0	0	0	0	0	0	0	0	0	0	0	0	0	0	0	0	0	0	0	0	0	0	0	0
ENSG00000134258	0	1	0	4	0	0	0	0	2	0	0	0	0	0	0	0	0	0	1	1	0	0	0	0	0	1	0	0	0	0	1	0
ENSG00000277141	0	0	0	0	0	0	0	0	0	0	0	0	0	0	0	0	0	0	0	0	0	0	0	0	0	0	0	0	0	0	0	0
ENSG00000235202	0	0	0	0	0	0	0	0	0	0	0	0	0	0	0	0	0	0	0	0	0	0	0	0	0	0	0	0	0	0	0	0
ENSG00000271427	29	9	12	7	6	13	5	15	9	4	36	3	4	2	1	7	5	10	1	7	13	7	10	9	7	7	5	19	1	2	1	3
ENSG00000198162	2448	1760	1804	1754	2726	2867	1982	3044	1388	1224	6699	1048	1248	1275	1259	1571	908	1256	1104	1827	5152	2130	3413	2011	1587	2257	1749	4584	939	1310	1077	1448
ENSG00000279513	8	4	8	7	10	4	1	7	3	2	23	4	1	2	0	2	2	2	0	4	11	1	5	3	3	3	2	20	0	1	1	0
ENSG00000226755	1	1	1	0	2	0	0	1	0	2	0	0	0	0	0	0	0	1	1	5	2	2	1	0	0	0	0	0	1	3	0	0
ENSG00000236866	0	0	0	0	1	0	0	1	0	1	1	0	1	0	1	0	0	0	1	0	0	0	0	1	0	0	0	0	1	1	0	2
ENSG00000183508	2287	3307	1499	3178	1425	1505	991	1727	2594	2160	5380	1371	2657	2737	2054	3151	858	2441	817	3050	1844	740	1229	631	2075	3045	1099	5067	1091	1675	1119	1736
ENSG00000213262	1	1	0	1	0	1	1	1	2	3	8	5	3	3	5	6	0	0	0	0	1	1	1	1	2	5	0	11	1	2	9	12
ENSG00000212266	0	0	0	0	0	0	0	0	0	0	0	0	2	0	0	0	0	0	0	0	0	0	0	0	0	0	1	0	0	0	1	1
ENSG00000270719	0	1	0	0	0	0	0	0	0	0	3	0	0	0	2	0	0	0	0	0	0	0	0	0	2	0	0	2	1	1	0	1
ENSG00000228217	0	0	0	1	0	1	1	1	1	1	1	2	2	0	0	1	0	0	0	2	3	1	1	2	1	1	0	3	2	2	1	0
ENSG00000196505	915	708	833	654	616	706	490	665	332	333	1650	231	537	516	572	627	440	662	680	712	1245	481	847	490	444	578	423	1169	439	546	577	618
ENSG00000065183	2245	1568	1863	1511	1729	1870	1519	1876	2079	1635	6758	1250	2664	2155	2835	2968	1480	2576	2272	2680	3646	1411	2853	1292	2061	3237	1313	4504	2602	2783	2857	2905
ENSG00000155761	7	7	4	4	3	4	2	6	8	8	9	5	7	3	6	11	4	10	1	12	10	10	5	5	1	8	6	12	2	16	5	3
ENSG00000226126	0	0	0	0	0	0	0	0	0	0	1	0	0	0	0	0	0	0	0	0	0	0	0	0	0	0	1	0	0	0	0	0
ENSG00000229911	0	0	0	0	0	0	0	0	0	0	0	0	0	0	0	0	0	0	0	0	0	0	0	0	0	0	0	0	0	0	0	0
ENSG00000092607	1	0	0	0	1	1	2	1	2	5	18	5	1	0	0	2	0	0	0	1	2	1	2	1	0	3	0	4	0	0	0	0
ENSG00000226172	0	0	0	0	1	0	0	0	0	1	0	0	0	1	0	0	0	0	0	0	0	0	0	0	0	0	0	0	0	0	0	0
ENSG00000239216	0	0	0	0	0	0	0	0	0	0	0	0	0	0	0	0	0	0	0	0	0	0	0	0	0	0	0	1	0	0	0	0
ENSG00000116874	247	207	193	201	321	365	263	372	315	266	961	158	399	393	375	437	318	463	359	333	805	274	627	266	244	302	211	618	409	427	482	457
ENSG00000224238	3	0	3	2	7	4	1	7	8	6	20	1	5	3	3	3	1	0	4	4	10	9	5	1	4	7	4	13	5	6	4	9
ENSG00000223575	0	0	1	1	0	0	0	3	0	4	4	1	0	0	0	1	3	0	0	2	3	0	3	1	0	0	1	6	0	2	0	2
ENSG00000236804	1	2	0	5	2	4	1	0	0	0	2	1	2	0	1	1	1	2	2	1	2	2	6	6	0	2	2	4	4	1	0	3
ENSG00000231365	265	259	291	257	117	157	133	202	332	287	1017	163	365	430	423	529	96	127	118	298	246	148	184	124	178	268	229	582	167	346	238	368
ENSG00000227056	7	2	5	3	1	1	1	2	4	1	9	1	3	2	3	4	1	0	1	6	5	1	2	3	4	8	1	4	1	3	1	7
ENSG00000227712	0	1	0	0	0	0	0	0	0	0	1	0	0	2	2	0	0	0	0	1	0	0	1	0	0	0	0	1	1	3	3	3

ENSG00000232650	0	1	0	0	0	0	0	0	0	0	0	0	1	2	1	1	0	0	1	0	0	0	0	0	0	0	0	0	0	2	0	3
ENSG00000194297	0	0	0	0	0	0	0	0	0	0	0	0	0	0	0	0	0	0	0	0	0	0	0	0	0	1	0	0	0	0	0	0
ENSG00000116882	0	0	0	2	0	0	0	0	0	0	1	0	0	0	1	0	0	0	0	0	0	0	0	0	0	0	0	0	1	0	0	1
ENSG00000230921	0	0	0	0	0	0	0	0	0	0	0	0	0	0	0	0	0	0	0	0	0	0	0	0	0	0	0	0	0	0	0	0
ENSG00000203859	0	0	1	0	0	0	0	0	0	0	0	0	0	0	0	0	0	0	0	0	0	0	0	0	0	0	0	1	0	0	0	0
ENSG00000232780	0	0	0	0	0	0	0	0	0	0	0	0	0	0	0	0	0	0	0	0	0	0	0	0	0	0	0	0	0	0	0	0
ENSG00000203858	0	0	0	0	0	0	0	0	0	0	0	0	0	0	0	0	0	0	0	0	0	0	0	0	0	0	0	0	0	0	0	0
ENSG00000235595	0	0	0	0	0	0	0	0	0	0	0	0	0	0	0	0	0	0	0	0	0	0	0	0	0	0	0	0	0	0	0	0
ENSG00000187481	0	0	0	0	0	0	0	0	0	0	0	0	0	0	0	0	0	0	0	0	0	0	0	0	0	0	0	0	0	0	0	0
ENSG00000229570	0	0	0	0	0	0	0	0	0	0	0	0	0	0	0	0	0	0	0	0	0	0	0	0	0	0	0	0	0	0	0	0
ENSG00000203857	0	0	0	0	0	0	0	0	0	0	0	0	0	0	0	0	0	0	0	0	0	0	0	0	0	0	0	0	0	0	0	0
ENSG00000226443	0	0	0	0	0	1	0	0	0	0	0	0	0	0	0	0	0	0	0	0	0	0	0	0	0	0	0	0	0	0	0	0
ENSG00000249798	0	0	0	0	0	0	0	0	0	0	0	0	0	0	0	0	0	0	0	0	1	0	0	0	0	0	0	0	0	0	0	0
ENSG00000239873	0	0	0	0	0	0	0	0	0	0	0	0	0	0	0	0	0	0	0	0	0	1	0	0	0	0	0	0	0	0	0	0
ENSG00000203855	0	0	0	0	0	1	0	0	0	0	0	0	0	0	0	0	0	0	0	0	0	0	0	0	0	0	0	0	0	0	0	0
ENSG00000240244	0	0	0	0	0	0	0	0	0	0	0	0	0	0	0	0	0	0	0	0	1	0	0	0	0	0	0	0	0	0	0	0
ENSG00000260941	0	0	0	0	0	2	1	4	0	0	1	1	0	1	0	1	0	0	0	0	1	1	3	0	0	0	0	1	0	0	1	0
ENSG00000198857	0	0	0	0	2	2	2	2	0	1	1	0	1	4	1	1	0	0	0	0	3	0	1	1	0	0	0	1	0	0	0	0
ENSG00000143067	0	0	0	0	98	153	70	148	105	116	402	88	71	115	86	136	0	0	0	0	163	77	112	86	71	119	107	308	55	80	68	92
ENSG00000092621	6829	5608	4659	6025	5335	6110	4005	6162	3344	2746	5273	1737	1722	1563	1064	2232	2125	4712	2798	4085	7393	2851	5858	2483	1857	3569	789	4673	656	1030	776	1094
ENSG00000134240	1	1	0	0	1	0	0	1	0	0	0	0	1	0	0	0	0	0	0	0	0	0	0	0	1	1	0	1	0	0	1	0
ENSG00000134193	2	0	0	1	0	0	0	1	79	44	201	33	18	16	16	37	2	1	6	1	1	1	0	0	24	44	45	92	22	13	21	20
ENSG00000215864	0	3	0	0	0	0	0	0	0	0	0	0	0	0	0	0	0	0	0	0	0	0	0	0	0	0	0	0	0	0	0	0
ENSG00000227205	0	0	0	0	0	0	0	0	0	0	0	0	0	0	0	0	0	0	0	0	0	0	0	0	0	0	0	0	0	0	0	0
ENSG00000226446	0	0	0	0	0	0	0	0	1	0	3	0	0	0	0	0	0	0	0	0	0	0	0	0	0	0	0	0	0	0	0	0
ENSG00000134249	0	0	0	0	0	0	0	0	0	0	2	0	0	0	0	0	0	0	0	0	0	0	0	0	0	0	0	1	0	0	0	0
ENSG00000261662	5	0	3	3	14	14	11	15	63	52	289	35	12	6	7	12	4	3	1	5	15	5	6	4	30	57	28	94	4	6	4	11
ENSG00000134250	2701	2898	2710	3075	2355	2559	1747	2655	8975	7865	37042	6535	4871	4741	4969	5880	503	915	617	1809	2533	1118	1842	935	5503	10844	4025	16750	2429	3589	2642	4017
ENSG00000273406	1	0	4	0	0	0	0	0	0	1	7	0	0	0	0	1	0	1	0	3	0	0	0	0	0	0	0	0	2	0	1	0
ENSG00000207149	0	0	0	0	0	0	0	0	0	0	0	0	0	0	0	0	0	0	0	0	0	0	0	0	0	0	0	0	0	0	0	0
ENSG00000265808	2757	1776	2117	1766	2310	2492	1806	2622	1646	1360	6387	1135	1521	1420	1599	1931	2593	3687	2948	2865	5864	2369	4085	2445	1736	2486	1597	4342	1821	1869	2176	2399
ENSG00000274642	0	0	0	0	0	0	0	0	0	0	0	0	0	0	0	1	0	0	0	0	0	0	0	0	0	0	0	0	1	0	1	1
ENSG00000277313	0	0	0	0	0	0	0	0	0	0	0	0	0	0	0	0	0	0	0	0	0	0	0	0	0	0	0	0	0	0	0	0
ENSG00000223804	2	12	9	9	42	53	38	65	9	9	23	6	83	75	84	73	3	8	2	14	86	24	52	32	12	19	2	14	75	107	89	98
ENSG00000273727	0	0	1	0	1	0	2	3	0	0	0	0	1	0	1	3	0	0	0	0	2	0	0	0	0	0	0	0	1	1	2	5
ENSG00000270392	166	157	144	150	91	97	78	122	92	103	216	61	189	180	154	222	27	36	20	123	130	67	65	69	39	97	44	145	48	131	77	163
ENSG00000270231	1451	1158	1307	1234	900	988	652	997	775	777	2208	590	967	899	934	1215	558	549	485	1137	1192	558	724	546	466	793	450	1490	363	775	506	863
ENSG00000275131	326	292	334	289	74	99	97	121	119	152	393	103	530	535	651	706	95	77	96	219	148	66	88	61	88	183	90	260	258	670	467	744
ENSG00000273694	0	2	0	0	0	0	0	0	1	1	0	0	1	0	0	0	0	0	0	0	0	0	0	0	0	2	0	3	0	1	6	3
ENSG00000275933	0	0	0	0	0	0	0	1	0	0	0	0	0	0	0	0	0	0	0	0	0	0	0	0	0	0	0	0	0	1	0	0
ENSG00000273136	497	423	473	495	474	523	410	484	485	495	1379	307	792	769	775	967	118	168	116	378	587	319	425	286	369	632	327	1087	313	682	424	806
ENSG00000275538	2	0	1	0	0	0	0	0	2	3	15	2	0	1	1	0	0	1	1	1	0	0	0	0	11	9	5	27	4	6	3	8
ENSG00000263353	0	1	0	0	0	0	0	0	0	0	0	0	0	0	0	0	0	0	0	0	0	0	0	0	0	0	0	0	0	0	0	0
ENSG00000281741	1	1	0	0	2	1	0	2	20	22	88	18	19	19	30	24	0	0	1	3	1	1	2	0	26	27	16	57	19	28	26	13
ENSG00000226067	18	27	22	35	95	85	71	97	714	548	2461	374	314	305	354	400	4	18	17	13	134	49	91	56	708	982	736	1441	272	373	385	402
ENSG00000277610	3	0	0	3	0	0	5	1	20	16	72	3	15	11	14	4	1	0	0	2	1	2	2	0	21	14	15	40	4	12	12	8
ENSG00000275585	0	0	0	0	0	0	0	0	1	0	7	0	1	5	1	0	0	0	0	0	0	0	0	0	3	1	3	3	0	5	0	5
ENSG00000273516	0	0	0	0	0	0	0	0	0	0	0	0	1	0	0	1	0	0	0	0	1	0	0	0	0	0	0	0	0	0	0	0
ENSG00000275296	0	0	0	0	0	0	0	0	0	0	0	0	0	0	0	0	0	0	0	0	0	0	0	0	0	0	0	0	0	0	0	0
ENSG00000198019	1	0	0	1	1	0	1	0	4	2	1	0	0	2	0	0	0	0	0	1	0	0	0	1	0	1	0	0	0	1	0	0
ENSG00000234998	2	0	0	0	0	0	0	0	0	0	0	0	0	0	0	0	0	0	0	0	0	0	0	0	0	0	0	0	0	0	0	0
ENSG00000233029	1	1	0	0	1	1	4	0	0	7	0	1	0	0	1	2	0	0	2	0	0	0	0	0	0	2	2	3	1	1	0	3
ENSG00000223345	0	0	1	1	0	0	0	0	1	1	0	0	0	0	0	0	0	0	0	0	0	0	0	0	0	1	0	0	0	0	0	1
ENSG00000227193	0	0	0	1	2	0	0	0	0	0	2	1	0	0	0	0	0	0	0	0	0	0	0	0	0	0	0	0	0	1	0	1
ENSG00000213244	0	1	0	0	0	1	0	0	0	0	0	0	0	0	1	2	0	0	0	0	0	0	0	0	0	0	0	0	0	0	0	1
ENSG00000268993	2	2	0	0	0	0	1	0	0	1	4	0	0	0	0	0	0	1	0	0	2	0	0	1	0	0	0	1	0	0	0	0
ENSG00000188610	1346	944	1178	1159	993	1069	703	1074	607	479	1449	333	886	830	988	1143	419	609	495	715	1378	541	983	475	621	947	135	1009	633	744	646	722
ENSG00000171943	349	284	245	312	385	401	279	436	154	125	484	97	117	133	172	187	60	92	76	166	520	268	319	212	172	272	44	433	59	100	89	131
ENSG00000275129	0	0	0	0	0	2	0	2	0	0	2	0	0	0	0	0	0	0	0	1	4	4	2	0	0	0	0	0	0	0	0	0
ENSG00000230806	1	2	1	4	2	5	5	6	0	1	6	0	1	0	0	0	0	0	1	0	5	2	2	3	3	4	0	6	1	2	1	1
ENSG00000227082	0	0	4	0	2	2	0	4	0	0	1	1	0	1	0	1	0	0	0	1	3	4	1	1	2	0	2	2	0	3	2	7
ENSG00000231429	0	0	0	1	0	0	2	1	0	0	2	0	2	1	0	4	0	0	0	0	0	1	0	2	0	0	0	0	0	3	0	0
ENSG00000228826	1	4	0	1	0	1	2	3	0	0	2	0	3	5	0	1	1	1	2	0	0	1	4	2	2	0	0	3	0	3	1	2
ENSG00000233432	6	3	5	2	4	0	2	3	1	0	2	0	4	0	3	6	0	1	0	3	5	0	0	2	1	1	1	2	0	2	0	1
ENSG00000272583	32	25	31	33	10	10	12	10	0	0	0	0	8	10	3	9	19	25	19	28	27	10	18	9	0	0	0	0	5	11	5	5
ENSG00000231752	83	69	60	56	80	89	69	111	19	15	105	27	109	81	60	102	62	68	70	60	218	87	128	63	15	33	31	52	58	85	66	105
ENSG00000224857	0	0	0	1	2	3	2	7	0	0	0	0	1	3	2	0	1	1	1	2	7	1	1	7	0	0	0	0	1	1	2	6
ENSG00000276118	0	0	0	0	0	0	0	0	0	0	0	0	0	0	0	0	0	0	0	0	0	0	0	0	0	0	0	0	0	0	0	0
ENSG00000277095	0	0	0	0	0	0	0	0	0	0	0	0	0	0	0	0	0	0	0	0	0	0	0	0	0	0	0	0	0	0	0	0
ENSG00000277702	1	0	0	0	0	0	0	0	0	0	0	0	0	1	2	1	0	0	0	0	0	0	0	0	0	0	1	0	0	1	0	7
ENSG00000264145	0	0	0	0	0	0	0	0	0	0	0	0	0	0	0	0	0	0	0	0	0	0	0	0	0	0	0	0	0	0	0	0
ENSG00000274927	0	0	0	0	0	0	0	0	0	0	0	0	0	0	0	0	0	0	0	0	0	0	0	0	0	0	0	1	0	0	0	0
ENSG00000242943	0	0	0	0	0	0	0	0	0	0	0	0	0	0	0	0	0	0	0	0	0	0	0	0	0	0	0	0	0	0	0	0
ENSG00000223495	0	0	0	0	0	0	0	0	0	0	0	0	0	0	0	0	0	0	0	0	0	0	0	0	0	0	0	0	0	0	0	0
ENSG00000235887	0	0	0	0	0	0	0	0	0	0	0	0	0	0	0	0	0	0	0	0	0	0	0	0	0	0	0	0	0	0	0	0
ENSG00000203825	0	0	0	0	0	0	0	0	0	0	0	0	0	0	0	0	0	0	0	0	0	0	0	0	0	0	0	0	0	0	0	0
ENSG00000179571	1	3	4	1	1	0	0	0	0	0	0	1	0	0	1	1	0	3	0	2	0	0	0	0	0	0	0	0	0	0	0	1
ENSG00000226028	0	0	0	1	0	0	0	0	0	0	0	0	0	0	0	0	0	0	0	0	0	0	0	0	0	0	0	0	0	0	0	0
ENSG00000202167	0	0	0	0	0	0	0	0	0	0	0	0	0	0	0	0	0	0	0	0	0	0	0	0	0	0	0	0	0	0	0	0
ENSG00000207349	0	0	0	0	0	0	0	0	0	0	0	0	0	0	0	0	0	0	0	0	0	0	0	0	0	0	0	0	0	0	0	0
ENSG00000252826	0	0	0	0	0	0	0	0	0	0	0	0	0	0	0	0	0	0	0	0	0	0	0	0	0	0	0	0	0	0	0	0
ENSG00000206737	0	0	0	0	0	0	0	0	0	0	0	0	0	0	0	0	0	0	0	0	0	0	0	0	0	0	0	0	0	0	0	0
ENSG00000232721	0	0	0	0	0	0	0	1	0	0	0	0	0	0	0	0	0	0	0	0	0	0	0	0	0	0	0	0	0	0	0	1
ENSG00000223779	3	1	0	0	0	0	0	0	1	0	0	0	0	0	0	0	0	0	0	2	0	0	0	0	0	0	0	0	0	0	0	0
ENSG00000242352	0	0	1	0	0	1	0	1	0	0	1	0	3	0	2	0	0	0	0	0	1	2	0	0	0	2	0	0	1	1	0	0
ENSG00000270962	0	0	0	0	0	0	0	0	0	0	0	0	0	0	0	0	0	0	0	0	0	0	0	0	0	0	0	0	0	0	0	0
ENSG00000263590	0	0	0	0	0	0	0	0	0	0	0	0	0	0	0	0	1	0	0	0	0	0	0	0	0	0	0	0	0	0	0	0
ENSG00000252105	0	0	0	0	0	0	0	0	0	0	0	0	0	0	0	0	0	0	0	0	0	0	0	0	0	0	0	0	0	0	0	0
ENSG00000233430	0	0	0	0	0	0	0	0	0	0	0	0	0	0	0	0	0	0	0	0	1	0	0	0	0	0	0	0	0	0	0	0
ENSG00000237503	0	0	0	0	0	0	0	0	0	0	0	0	0	0	0	0	0	0	0	0	0	0	0	0	0	0	0	0	0	0	0	0
ENSG00000265531	0	0	0	0	0	0	0	0	1	0	0	0	0	0	0	0	0	0	0	0	0	0	0	0	0	0	0	0	0	0	0	0
ENSG00000240929	0	0	0	0	0	0	0	0	0	0	0	0	0	0	0	0	0	0	0	0	0	0	0	0	0	0	0	0	0	0	0	0
ENSG00000234571	0	0	0	0	0	0	0	0	0	0	0	0	0	0	0	0	0	0	0	0	0	0	0	0	0	0	0	0	0	0	0	0
ENSG00000273213	0	0	0	0	0	0	0	0	0	0	0	0	0	0	0	0	0	0	0	0	0	0	0	0	0	0	0	0	0	0	0	0
ENSG00000230186	0	0	0	0	0	0	0	0	0	0	0	0	0	0	0	0	0	0	0	0	0	0	0	0	0	0	0	0	0	0	0	0
ENSG00000203818	0	0	0	0	0	0	0	0	0	0	0	0	0	0	0	0	0	0	0	0	0	0	0	0	0	0	0	0	0	0	0	0
ENSG00000268074	1	0	0	0	0	0	0	0	0	0	1	0	0	0	0	0	0	0	1	0	0	1	0	0	0	1	0	0	0	0	0	0
ENSG00000263513	315	227	219	245	211	205	136	205	102	120	288	69	215	191	223	295	92	133	86	163	270	103	219	103	145	169	19	210	145	165	158	179
ENSG00000270872	35	24	39	23	95	130	78	146	20	20	63	15	13	20	14	22	1	12	14	35	149	71	83	53	35	44	10	68	5	5	12	19
ENSG00000276674	0	0	0	0	0	0	0	0	0	0	0	0	0	0	0	0	0	0	0	0	0	0	0	0	0	0	0	0	0	0	0	0
ENSG00000275075	0	0	0	0	0	0	0	0	0	0	0	0	0	0	0	0	0	0	0	0	0	0	0	0	0	0	0	0	0	0	0	0
ENSG00000275767	0	0	0	0	0	0	0	0	0	0	0	0	0	0	0	0	0	0	0	0	0	0	0	0	0	0	0	0	0	0	0	0
ENSG00000232527	0	1	0	2	0	0	0	1	0	0	0	0	2	4	5	5	0	0	0	0	0	0	0	0	0	0	0	0	3	3	4	2
ENSG00000175658	0	0	0	0	0	0	0	0	0	0	0	0	0	0	0	0	0	0	0	0	0	0	0	0	0	0	0	0	0	0	0	0
ENSG00000276756	0	0	0	0	0	0	0	0	0	0	0	0	0	0	0	0	0	0	0	0	0	0	0	0	0	0	0	0	0	0	0	0
ENSG00000271567	0	0	0	0	0	0	0	0	0	0	0	0	0	0	0	0	0	0	0	0	0	0	0	0	0	0	0	0	0	0	0	0
ENSG00000271439	0	0	0	0	0	0	0	0	0	0	0	0	0	0	0	0	0	0	0	0	0	0	0	0	0	0	0	0	0	0	0	0
ENSG00000207205	0	0	0	0	0	0	0	0	0	0	0	0	1	1	2	2	0	0	0	0	0	0	0	0	0	0	0	0	1	1	8	6
ENSG00000237343	0	0	0	0	0	0	0	0	0	0	0	0	1	0	1	2	0	0	0	0	0	0	0	0	0	0	0	0	5	1	0	2
ENSG00000233586	0	0	0	0	0	0	0	0	0	0	0	0	2	0	2	1	0	0	0	0	0	0	0	0	0	0	0	0	1	1	1	1
ENSG00000266338	606	681	608	779	63	72	44	57	59	58	198	38	800	849	831	1146	184	236	240	637	90	36	44	36	40	61	57	133	364	786	456	841
ENSG00000227212	37	33	35	60	0	1	0	2	0	0	0	0	52	59	65	72	4	7	9	36	0	0	0	1	0	0	0	0	17	57	27	58
ENSG00000229002	6	5	11	1	0	0	0	1	0	0	0	0	12	11	19	13	4	2	9	6	0	0	0	0	0	0	0	0	2	13	10	10
ENSG00000275291	0	0	0	0	0	0	0	0	0	0	0	0	4	3	3	5	0	0	0	0	0	0	0	0	0	0	0	0	0	2	5	1
ENSG00000278099	0	0	0	0	0	0	0	0	0	0	0	0	1	0	0	0	0	0	0	0	0	0	0	0	0	0	0	0	0	1	0	0
ENSG00000277918	0	0	0	0	0	0	0	0	0	0	2	0	2	1	0	0	0	0	0	0	0	0	0	0	0	0	0	0	5	0	2	0
ENSG00000279782	0	0	0	0	0	0	0	0	0	0	0	0	0	0	0	0	0	0	0	0	0	0	0	0	0	0	0	0	0	0	0	0
ENSG00000277199	0	0	0	0	0	0	0	0	0	0	0	0	0	0	0	0	0	0	0	0	0	0	0	0	0	0	0	0	0	0	0	0
ENSG00000224363	2	2	0	1	1	0	0	0	4	0	2	0	0	1	0	1	0	0	0	2	3	1	0	1	0	4	0	10	0	0	0	0
ENSG00000278273	0	0	0	0	0	0	0	0	0	0	0	0	0	0	0	0	0	0	0	0	0	0	0	0	0	0	0	0	0	0	0	0
ENSG00000273825	0	0	0	0	0	0	0	0	0	0	0	0	0	0	0	0	0	0	0	0	0	0	0	0	0	0	0	0	0	0	0	0
ENSG00000196369	200	119	134	158	102	114	82	99	135	88	377	81	66	80	85	110	40	45	46	81	151	55	106	46	128	159	81	397	34	49	48	51
ENSG00000271644	1	0	0	0	0	1	0	0	0	0	0	0	0	0	0	0	0	0	0	0	1	1	0	0	2	5	2	1	0	0	0	0
ENSG00000215784	492	335	359	351	303	258	198	261	159	134	429	102	322	285	246	346	133	205	162	249	413	167	270	144	177	262	26	336	203	196	182	255
ENSG00000272419	15	25	10	18	1	0	1	2	127	113	481	58	98	137	135	174	3	6	5	24	1	0	1	3	81	172	143	305	55	139	90	173
ENSG00000277678	4	4	2	3	0	0	0	0	24	21	79	10	17	20	21	17	0	0	0	2	0	0	0	0	9	22	17	32	7	19	9	22
ENSG00000281571	3	5	2	7	9	6	2	5	19	18	58	13	31	27	41	41	0	2	3	9	3	1	2	4	15	29	18	38	20	28	19	39
ENSG00000223612	3	2	7	5	7	5	7	3	4	1	1	0	3	1	4	3	0	1	0	4	12	3	2	1	0	1	0	2	2	0	2	3
ENSG00000256374	0	0	0	0	0	0	0	0	0	0	0	0	0	0	0	0	0	0	0	0	0	0	0	0	0	0	0	0	0	0	0	0
ENSG00000271291	0	0	0	0	0	0	0	0	0	0	0	0	0	0	0	0	0	0	0	0	0	0	0	0	0	0	0	0	0	0	0	0
ENSG00000276216	6	2	4	2	0	0	0	0	4	4	14	7	7	7	8	8	4	2	8	6	0	0	0	0	5	1	0	15	0	6	9	7
ENSG00000207501	0	0	0	0	0	0	0	0	0	0	0	0	0	0	0	0	0	0	0	0	0	0	0	0	0	0	0	0	0	0	0	0
ENSG00000236140	1	0	0	1	0	0	0	0	0	1	4	1	0	0	2	0	0	0	0	1	0	0	0	0	0	1	2	3	0	0	0	2
ENSG00000162825	93	141	110	108	41	47	34	63	13	14	72	7	143	156	150	192	35	51	37	100	56	16	31	20	12	21	49	86	67	137	102	173
ENSG00000234367	6	8	8	15	4	7	1	5	2	3	14	0	24	29	16	21	2	4	1	14	6	8	3	3	2	0	4	13	10	27	14	24
ENSG00000270384	0	0	1	0	0	0	0	0	0	0	0	0	0	0	0	0	0	0	0	0	0	0	0	0	0	0	0	0	0	0	0	0
ENSG00000270722	0	0	0	0	0	0	0	0	0	0	1	0	11	13	20	11	1	0	0	2	0	0	0	0	0	0	0	0	12	13	21	15
ENSG00000278182	0	0	0	0	0	0	0	0	0	0	0	0	0	0	0	0	0	0	0	0	0	0	0	0	0	0	0	0	0	0	0	0
ENSG00000275064	0	2	1	1	0	0	0	0	0	0	0	0	0	0	0	0	0	1	0	0	0	0	0	0	0	0	0	1	0	0	0	1
ENSG00000272150	43	49	32	29	11	7	11	10	10	13	31	2	25	26	23	36	16	35	37	47	27	12	13	18	10	17	15	33	18	39	34	56
ENSG00000117262	278	210	234	220	216	242	186	240	136	113	554	102	157	150	197	210	200	265	233	338	615	247	359	270	154	200	126	394	184	216	256	247
ENSG00000174827	36	41	52	40	51	35	46	66	32	30	79	13	29	33	23	34	13	13	6	48	74	26	38	54	22	38	19	65	10	41	23	31
ENSG00000117281	41	45	48	46	64	71	58	86	42	38	210	24	45	38	37	47	7	14	7	58	118	64	59	67	38	66	53	148	33	61	40	59
ENSG00000265491	1421	1027	1208	997	1566	1570	1177	1630	1277	1039	5300	840	1085	996	1157	1267	632	1007	906	1102	2755	1129	2130	949	1188	1731	1330	3439	980	1255	1144	1207
ENSG00000186141	644	507	531	495	825	692	619	774	584	502	1924	364	896	792	1088	1017	582	763	567	726	1918	661	1368	720	554	855	439	1363	1005	1090	1329	1319
ENSG00000186364	181	133	137	115	113	79	66	104	58	56	196	33	38	39	30	52	85	93	85	135	148	60	81	73	31	61	58	122	25	64	39	51
ENSG00000131788	593	525	575	540	1173	1227	836	1310	521	383	1699	343	318	311	237	426	136	176	178	281	1255	603	936	530	301	583	430	1122	106	234	123	284
ENSG00000278549	0	0	0	0	1	1	0	0	0	0	3	0	0	0	0	0	0	0	0	1	3	0	1	0	0	1	0	0	0	0	0	0
ENSG00000198483	0	0	2	2	8	11	8	11	50	66	177	43	4	0	0	3	0	0	0	0	21	4	15	9	63	94	41	170	0	0	1	1
ENSG00000143127	121	109	147	113	56	74	53	82	89	67	332	56	23	11	19	28	34	35	34	138	86	55	38	61	38	75	75	132	15	41	24	62
ENSG00000244619	0	0	1	0	0	0	0	2	0	0	1	1	0	0	0	0	3	0	0	2	1	0	0	0	0	1	1	2	0	0	0	0
ENSG00000131779	778	400	556	429	584	572	474	608	609	457	2231	345	597	453	662	687	654	881	844	577	1269	473	996	430	539	733	526	1277	581	551	770	714
ENSG00000265241	4590	2935	3855	3161	4589	4542	3431	4928	4631	3256	12563	2578	3846	3665	4447	5089	3667	4888	4636	3617	8360	3296	6597	2799	4208	6067	2510	8219	4245	4790	5378	5422
ENSG00000211451	62	45	42	37	36	26	20	36	41	45	188	33	39	40	35	48	36	36	39	46	58	26	33	28	52	66	44	124	48	39	42	49
ENSG00000234222	52	36	55	41	47	35	30	36	38	26	122	20	15	15	13	13	15	12	12	46	50	29	36	46	26	41	40	86	8	20	13	23
ENSG00000280778	12	9	4	12	6	6	4	4	1	1	1	2	3	4	5	4	2	9	4	4	5	4	7	4	3	0	2	5	0	0	1	3
ENSG00000271601	2432	1737	2166	1826	1577	1641	1126	1624	1718	1433	5643	915	718	680	794	820	438	679	689	1057	2095	879	1617	693	1415	2126	1276	3398	534	580	552	623
ENSG00000272031	203	125	195	106	116	139	97	150	64	46	201	36	77	105	71	130	59	50	59	85	138	73	113	81	45	66	37	128	21	72	57	79
ENSG00000278431	0	0	0	0	0	0	0	0	0	0	0	0	0	0	0	0	0	0	0	0	0	0	0	0	0	0	0	0	0	0	0	0
ENSG00000121851	732	509	700	536	625	641	505	748	624	432	1941	352	597	537	618	741	418	485	533	420	1170	443	1020	494	481	648	444	1163	485	505	509	663
ENSG00000265972	3027	2129	3613	1956	1235	1378	1117	1435	9633	9360	37136	7856	4719	5586	5565	7932	1121	1508	2106	1286	3125	1447	3585	1414	5704	9310	8550	22174	2247	2609	3832	3309
ENSG00000168509	0	0	2	0	0	0	0	0	0	0	1	0	1	0	0	2	0	0	1	0	2	1	0	0	0	0	1	0	0	0	0	0
ENSG00000201558	2	0	2	2	5	3	3	2	0	0	0	0	1	3	3	3	0	1	3	4	2	1	6	5	0	0	0	0	3	5	8	2
ENSG00000233396	28	24	38	32	16	14	14	13	1	2	4	2	31	27	38	47	21	20	27	11	20	12	15	7	0	5	3	7	19	34	33	43
ENSG00000271425	120	113	106	131	115	141	101	167	141	158	717	136	285	211	235	282	31	55	27	99	130	43	68	61	137	214	105	409	94	189	146	226
ENSG00000213240	6	4	6	11	5	3	3	6	5	2	19	4	6	3	10	5	1	1	1	4	2	4	5	3	3	5	3	13	1	5	0	4
ENSG00000264343	80	71	81	85	88	97	75	128	109	80	376	51	160	135	175	161	23	27	27	56	130	51	61	59	64	134	82	276	61	118	90	145
ENSG00000276509	3	4	6	4	0	4	1	1	4	1	14	6	8	9	10	5	1	4	4	5	1	0	4	0	5	2	2	2	8	11	5	2
ENSG00000201789	0	0	0	0	0	0	0	0	0	0	0	0	0	0	0	0	0	0	0	0	0	0	0	0	0	0	0	0	0	0	0	0
ENSG00000271121	0	0	0	0	0	0	0	0	0	0	1	0	0	0	0	0	0	0	0	0	0	0	0	0	0	0	0	0	0	0	0	0
ENSG00000277406	4	15	12	8	0	0	3	0	0	0	6	0	3	3	4	3	5	3	10	6	1	0	0	1	2	1	1	0	0	1	4	4
ENSG00000270339	0	0	0	0	0	0	0	0	0	0	0	0	0	0	0	0	0	0	0	0	0	0	0	0	0	0	0	0	0	0	0	0
ENSG00000273768	2	1	3	1	0	0	0	0	1	0	4	1	0	0	0	0	0	0	1	8	2	2	1	1	0	1	6	6	1	0	0	0
ENSG00000274428	0	2	1	0	2	0	0	1	0	0	0	0	0	0	0	1	2	0	1	0	0	0	1	0	0	0	0	0	0	0	0	0
ENSG00000277655	16	6	20	1	0	0	0	0	0	0	2	3	20	27	24	29	3	1	10	10	0	0	0	0	0	1	0	4	7	22	21	16
ENSG00000277420	5	3	6	5	1	0	0	0	0	0	0	0	0	0	1	1	0	0	0	0	0	0	0	0	0	0	0	2	0	3	0	0
ENSG00000276975	22	21	21	19	0	4	2	1	0	1	4	0	1	2	3	4	8	7	6	8	1	0	1	0	0	0	1	0	3	1	2	1
ENSG00000274450	0	0	0	0	0	0	0	0	0	0	0	0	0	0	0	0	0	0	0	0	0	0	0	0	0	0	2	0	0	0	0	0
ENSG00000268043	804	672	829	639	769	927	680	997	781	693	3382	454	553	474	585	735	219	213	214	594	1064	575	643	580	595	999	1023	2120	282	711	442	798
ENSG00000244371	29	18	27	22	15	20	14	29	31	26	119	11	21	21	18	22	2	5	5	13	26	21	20	18	26	34	46	66	10	43	9	50
ENSG00000234225	0	0	0	0	0	0	0	0	0	0	0	0	0	0	0	0	0	0	0	0	0	0	0	0	0	1	0	0	0	0	1	0
ENSG00000227242	0	0	0	0	2	6	4	0	0	0	0	0	3	5	10	14	0	0	2	0	1	0	3	1	0	0	0	0	19	17	21	11
ENSG00000225603	0	0	0	0	7	0	2	1	0	0	0	0	3	1	0	2	0	0	0	0	0	0	0	0	0	0	0	0	1	0	0	1
ENSG00000201142	0	0	0	0	0	0	0	0	0	0	0	0	0	0	0	1	0	0	0	0	0	0	0	0	0	0	0	0	0	0	0	0
ENSG00000230832	0	0	0	1	0	0	0	1	0	0	1	0	7	14	15	20	0	0	0	0	1	0	0	0	0	2	0	0	7	8	17	9
ENSG00000283342	0	0	0	0	1	0	1	0	0	0	0	0	0	2	0	1	1	0	1	0	2	1	2	1	0	0	0	0	0	1	0	0
ENSG00000131791	1286	1004	989	978	757	811	570	841	464	475	2719	366	480	526	407	587	322	396	319	880	1128	489	654	486	358	531	554	1323	274	471	271	542
ENSG00000237188	20	29	19	22	69	63	35	76	68	45	249	43	34	49	47	61	6	9	4	15	103	51	66	46	40	49	55	165	31	29	23	39
ENSG00000273071	0	0	0	0	0	0	0	0	0	0	2	0	0	0	0	0	0	0	0	0	0	0	0	0	0	1	0	0	0	0	0	0
ENSG00000131781	36	18	30	26	167	200	132	195	73	62	391	42	50	42	41	47	7	9	9	27	212	80	190	85	69	93	72	204	24	45	45	46
ENSG00000180867	49	30	37	31	60	73	51	91	61	50	236	39	42	51	32	71	10	19	7	34	109	37	78	48	49	58	27	122	28	40	27	54
ENSG00000226015	4	2	4	5	31	39	24	44	18	11	82	7	5	9	4	9	5	5	4	4	46	23	38	14	9	14	19	49	5	8	3	7
ENSG00000236806	0	0	0	0	2	1	0	1	0	3	10	2	0	0	0	1	0	0	0	1	2	0	2	0	0	0	4	0	0	0	0	0
ENSG00000131778	2751	2325	2349	2279	3106	3356	2428	3621	1943	1515	5542	1088	2036	1908	2100	2556	1373	2006	1718	2301	5356	2398	4161	2129	1482	2270	1092	3777	1499	2134	1766	2377
ENSG00000278811	187	148	159	133	93	95	78	102	94	119	397	75	88	96	82	85	32	28	20	128	126	65	63	72	97	135	75	307	28	84	56	112
ENSG00000213226	0	0	0	0	0	0	5	0	0	0	2	0	0	0	0	1	0	0	0	1	2	0	0	0	0	0	0	0	0	0	0	0
ENSG00000226653	0	0	0	0	0	0	0	0	0	0	2	0	0	0	0	0	0	0	0	0	0	0	0	0	1	0	1	0	0	1	0	0
ENSG00000207209	0	0	0	0	0	0	0	0	0	0	0	0	0	0	0	0	0	0	0	0	0	0	0	0	0	0	0	0	0	0	0	0
ENSG00000272443	0	0	0	0	0	0	0	0	0	0	0	0	0	0	0	0	0	0	0	0	0	0	0	0	0	0	0	0	0	0	0	0
ENSG00000272480	1	0	0	0	0	0	0	0	0	0	0	0	0	0	0	0	0	0	0	0	0	0	0	0	0	0	0	0	0	0	0	0
ENSG00000116128	1023	854	1050	915	913	1066	669	1028	1417	1451	6914	1021	524	718	608	908	206	304	357	618	1121	485	703	411	1028	2228	1688	4231	430	674	383	772
ENSG00000162836	782	673	645	651	849	918	686	1039	101	95	312	68	557	550	484	616	226	287	252	438	1166	517	838	522	42	75	96	195	278	412	323	492
ENSG00000277762	0	0	0	0	0	0	0	0	0	0	0	0	0	0	0	0	0	1	0	0	0	0	1	0	0	0	0	0	0	0	0	0
ENSG00000227139	0	0	0	0	0	0	0	0	0	0	0	0	0	0	0	0	0	0	0	0	0	0	0	0	0	0	0	0	0	0	0	0
ENSG00000265107	0	0	0	0	10	19	14	10	0	0	4	0	0	0	0	0	0	0	2	0	11	16	20	6	0	1	0	0	0	0	0	0
ENSG00000274415	0	0	0	0	0	0	0	0	0	0	0	0	0	0	0	0	0	0	0	0	0	0	0	0	0	0	0	0	0	0	0	0
ENSG00000234190	0	0	0	0	0	0	0	0	0	0	0	0	0	0	0	0	0	0	0	0	0	0	0	0	0	0	0	0	0	0	0	0
ENSG00000223728	0	0	0	0	0	0	0	0	0	0	0	0	0	0	0	0	0	0	0	0	0	0	0	0	0	0	0	0	0	0	0	0
ENSG00000121634	0	0	0	0	0	0	0	0	0	0	0	0	0	0	0	1	0	1	0	0	0	0	0	0	0	0	1	0	0	0	0	0
ENSG00000188092	352	222	233	239	241	268	205	290	154	141	760	108	162	150	194	180	180	181	179	303	600	247	357	286	162	234	197	458	102	223	194	286
ENSG00000215859	69	61	43	64	49	69	57	70	30	16	105	13	71	59	61	58	11	13	6	48	78	32	46	42	19	39	42	59	18	62	39	56
ENSG00000244211	2	2	3	8	4	3	4	3	5	1	3	1	2	5	2	4	0	1	0	3	7	3	2	4	2	0	2	3	3	4	1	4
ENSG00000273059	2	5	2	2	3	7	3	5	2	2	9	0	5	6	1	2	0	1	0	2	5	5	2	1	1	7	6	4	1	1	0	2
ENSG00000206791	10	7	9	10	4	6	1	5	4	0	3	1	1	4	5	5	0	0	0	1	4	3	1	3	2	3	1	5	1	10	3	3
ENSG00000274372	32	25	23	20	44	33	33	32	22	21	62	8	24	20	15	40	13	21	13	15	30	24	22	23	3	9	12	25	10	30	9	31
ENSG00000206585	0	0	0	0	0	0	0	0	0	0	0	0	0	0	0	1	0	0	0	0	0	0	0	0	0	0	0	0	0	0	0	0
ENSG00000229828	0	0	0	0	0	0	0	0	0	0	2	0	0	0	0	0	0	0	0	0	0	0	0	0	0	0	1	0	0	0	1	0
ENSG00000263956	885	630	766	685	593	767	530	750	573	529	2235	378	420	458	451	641	248	342	234	605	919	459	580	440	370	596	545	1340	256	640	318	715
ENSG00000225080	16	23	20	28	16	32	17	32	23	24	95	17	24	29	26	31	3	8	11	26	28	14	12	11	20	21	22	63	7	32	23	31
ENSG00000198658	22	27	32	28	23	30	13	21	11	11	52	10	11	9	10	18	6	7	2	31	34	25	16	12	8	9	16	39	5	18	7	12
ENSG00000227733	6	7	7	8	0	2	0	0	0	0	0	0	4	1	5	4	3	1	2	10	0	0	0	0	0	0	0	0	1	0	1	4
ENSG00000232265	0	0	0	0	0	0	0	0	0	0	1	0	0	0	1	0	0	0	0	1	0	0	0	0	0	0	0	0	1	0	1	1
ENSG00000224335	0	0	0	0	0	0	0	0	0	0	0	0	0	0	0	0	0	0	0	0	0	0	0	0	0	0	0	0	0	0	0	0
ENSG00000227700	0	0	0	0	0	0	0	0	0	0	0	0	0	0	0	0	0	0	0	0	0	0	0	0	0	0	0	0	0	0	0	0
ENSG00000199879	0	0	0	0	0	0	0	0	0	0	1	0	1	4	1	1	0	0	0	0	0	0	0	0	0	0	0	0	2	6	3	3
ENSG00000234283	0	0	0	0	0	0	0	0	0	0	0	0	1	1	0	1	0	0	0	1	0	0	1	0	0	0	0	0	0	0	1	0
ENSG00000228626	5	12	7	8	1	0	0	2	1	0	3	0	44	32	12	37	9	4	6	10	0	0	0	0	0	0	0	1	1	1	5	8
ENSG00000231196	3	3	8	3	0	0	0	1	0	0	1	2	35	17	18	27	4	16	7	9	0	0	0	0	0	0	1	0	6	2	16	4
ENSG00000238107	2	1	1	0	0	0	0	0	0	2	0	0	7	5	5	8	0	2	0	4	0	0	1	0	0	0	0	1	3	2	2	1
ENSG00000235988	0	0	2	0	0	0	0	0	0	0	0	0	0	0	0	0	0	0	0	0	0	0	0	0	0	0	0	0	0	1	0	0
ENSG00000224481	2	4	3	3	1	1	1	1	1	4	11	1	1	1	0	4	1	4	1	4	0	0	2	1	1	1	2	5	0	2	2	2
ENSG00000252744	1	0	0	0	0	0	0	0	0	0	0	0	1	0	0	0	0	1	0	1	0	0	0	0	0	0	0	1	0	0	0	0
ENSG00000283676	1	0	3	0	0	0	0	0	0	0	0	1	2	0	2	1	0	0	0	0	2	0	0	0	0	0	0	0	0	0	1	0
ENSG00000202408	2	2	2	5	0	0	0	0	0	0	3	1	0	0	1	2	1	0	0	2	0	0	0	0	1	0	4	2	0	1	2	1
ENSG00000272824	1	1	0	3	1	1	0	1	3	6	34	0	0	0	0	0	1	0	0	0	0	0	0	1	4	2	5	9	0	1	2	5
ENSG00000207340	1	4	2	0	0	0	0	0	6	6	36	1	0	1	0	1	0	0	0	0	1	0	0	2	1	1	11	13	0	0	1	0
ENSG00000274020	41	48	41	29	121	128	68	137	302	255	1510	193	106	85	102	109	9	14	7	29	142	85	100	67	240	369	431	716	59	105	90	139
ENSG00000252135	1	0	0	0	0	0	0	0	0	1	9	0	0	0	0	1	0	0	0	0	1	0	0	0	3	0	4	4	0	1	0	0
ENSG00000278596	0	0	0	0	0	0	0	0	0	1	6	0	0	0	1	0	0	1	0	0	0	2	1	0	0	1	4	6	0	0	0	1
ENSG00000238825	0	0	1	0	0	0	0	0	3	0	11	0	0	0	0	1	0	0	0	0	0	0	0	0	1	2	5	3	0	0	0	0
ENSG00000231551	51	54	58	48	64	85	55	75	303	245	1617	179	64	84	86	98	30	23	24	45	85	65	53	64	230	365	508	888	41	115	58	111
ENSG00000201183	7	4	5	3	3	6	0	2	10	6	48	5	5	2	6	4	3	0	1	1	3	4	3	1	10	9	19	27	4	1	6	5
ENSG00000225871	5	4	6	5	27	30	21	31	28	32	201	22	19	20	20	20	0	3	1	3	31	14	22	28	30	47	70	134	1	22	13	15
ENSG00000280649	26	41	13	38	24	25	22	31	83	76	320	46	46	41	25	44	19	13	12	15	23	8	7	7	80	78	93	162	47	39	24	50
ENSG00000236334	0	0	0	0	0	0	0	0	0	2	2	0	0	0	1	0	1	0	0	0	1	0	0	0	0	0	2	0	0	0	0	0
ENSG00000274210	0	2	1	0	0	0	0	0	4	5	21	1	6	2	1	0	2	0	1	6	0	0	0	0	7	7	6	21	5	4	4	4
ENSG00000270629	296	315	277	334	306	369	273	449	354	329	1568	270	534	463	545	659	80	151	80	247	421	178	226	148	296	476	246	863	202	486	289	513
ENSG00000252515	0	0	0	0	0	0	0	0	0	0	0	0	0	0	0	0	0	0	0	0	0	0	0	0	0	0	0	0	0	0	0	0
ENSG00000177144	0	0	0	0	0	0	0	0	0	0	0	0	0	0	1	0	0	0	0	0	0	0	0	0	0	0	0	0	0	0	0	0
ENSG00000275140	0	1	0	0	0	0	0	0	0	0	0	0	0	0	0	0	0	1	0	0	0	0	0	0	0	0	0	0	0	0	0	0
ENSG00000271546	0	0	0	0	0	0	0	0	0	0	0	0	0	0	0	0	0	0	0	0	0	0	0	0	0	0	0	0	0	0	0	0
ENSG00000178104	997	875	957	898	424	503	362	490	383	374	1019	293	3369	3100	3751	3965	531	760	713	886	759	279	497	272	292	504	205	741	2687	3187	3291	3561
ENSG00000252656	0	0	0	0	0	0	0	0	0	0	0	0	0	0	0	0	0	0	0	0	0	0	0	0	0	0	0	0	0	0	0	0
ENSG00000227788	0	0	0	0	0	0	0	0	0	3	0	1	0	0	0	3	0	0	0	0	0	0	0	0	1	1	0	1	0	1	1	1
ENSG00000254913	0	0	0	0	0	0	0	0	0	0	0	0	0	0	0	0	0	0	0	0	0	0	0	0	0	0	0	0	0	0	0	0
ENSG00000255148	0	0	0	0	0	0	0	0	0	0	0	0	0	0	0	0	0	0	0	0	0	0	0	0	0	0	0	0	0	0	1	2
ENSG00000254539	0	0	0	0	0	1	0	1	1	0	1	0	1	1	2	5	0	0	0	0	0	0	0	0	0	0	0	2	0	0	2	0
ENSG00000269713	560	598	541	573	220	311	154	312	67	92	157	54	748	738	769	942	184	277	198	517	201	114	114	90	56	84	39	166	424	911	487	985
ENSG00000274019	8	2	8	0	0	0	0	0	0	0	0	0	28	25	35	43	4	4	5	2	0	0	0	0	0	0	0	0	31	31	29	43
ENSG00000201699	0	0	0	0	0	0	0	0	0	0	0	0	1	0	0	0	0	0	0	0	0	0	0	0	0	0	0	0	0	0	0	0
ENSG00000274265	1	1	0	4	3	9	2	6	49	58	152	37	95	92	111	109	0	0	0	2	2	3	11	4	30	59	41	90	81	91	114	133
ENSG00000215861	0	0	0	0	2	3	0	3	2	4	14	3	7	10	8	10	0	0	0	0	1	0	1	3	2	7	3	7	0	14	11	11
ENSG00000272755	0	0	0	0	0	0	0	0	1	2	3	0	1	0	1	2	0	2	0	0	0	0	0	0	2	0	1	2	2	1	1	1

ENSG00000273591	0	0	0	0	0	0	0	0	0	0	0	0	0	0	0	0	0	0	0	0	0	0	0	0	0	0	0	0	0	0	0	0
ENSG00000274423	0	0	0	0	0	0	0	0	0	0	2	0	1	0	1	0	0	0	0	0	0	0	0	0	0	0	0	0	0	3	0	0
ENSG00000271383	616	617	662	567	607	665	467	694	479	402	1429	229	851	772	847	1051	183	194	162	495	694	342	425	367	351	554	370	1116	328	746	442	815
ENSG00000263464	0	0	1	2	0	0	0	0	0	0	0	0	0	0	0	0	0	1	0	0	1	0	0	0	0	0	0	0	0	0	0	0
ENSG00000277147	120	85	121	110	190	161	115	187	243	177	626	125	155	131	160	158	50	56	57	83	239	110	176	99	206	294	213	395	143	178	114	184
ENSG00000275557	41	46	30	51	38	28	16	31	64	54	236	41	56	40	40	52	14	17	11	23	32	12	16	8	44	76	68	108	42	27	33	39
ENSG00000206828	1	0	2	3	13	6	3	15	3	4	21	3	7	5	11	4	0	4	3	4	15	4	4	11	1	9	16	6	4	16	12	9
ENSG00000234232	0	2	1	0	0	0	0	0	1	1	2	0	1	0	1	3	0	0	0	0	4	0	0	1	3	1	5	1	0	0	2	3
ENSG00000275229	0	0	0	0	0	0	0	0	0	0	0	0	0	0	0	0	0	0	0	0	0	0	1	0	0	0	0	1	0	0	0	0
ENSG00000272058	0	1	0	0	0	0	0	0	0	0	0	0	4	1	0	1	0	0	0	0	0	0	0	0	0	0	0	0	0	5	2	3
ENSG00000226500	0	0	0	0	0	0	0	0	0	0	0	0	0	0	0	0	0	0	0	0	0	0	0	0	0	0	0	0	0	0	0	0
ENSG00000150337	3	0	0	2	4	1	5	3	0	0	1	0	0	0	0	0	0	1	0	1	3	1	1	1	0	0	0	0	0	0	0	2
ENSG00000203814	35	20	37	6	43	29	29	55	19	20	65	10	18	37	26	36	60	45	41	72	121	59	103	65	66	48	17	116	39	49	62	37
ENSG00000233030	1	0	1	1	1	1	1	5	0	0	2	1	1	0	0	1	1	0	0	1	1	1	0	1	0	0	2	2	0	0	0	0
ENSG00000183598	8	4	9	4	9	11	4	9	2	10	16	5	6	0	4	5	6	5	3	24	36	12	30	10	12	9	7	17	13	9	11	14
ENSG00000270882	0	1	0	0	0	2	0	2	2	2	11	4	8	0	4	4	0	0	1	1	4	0	0	0	1	1	1	6	1	3	2	5
ENSG00000203811	0	0	0	0	0	0	0	0	0	0	0	0	0	0	0	0	0	0	0	0	0	0	0	0	0	0	0	0	0	0	0	0
ENSG00000203812	0	0	0	0	0	0	0	0	0	0	0	0	0	0	0	0	0	0	0	0	0	0	0	0	0	0	0	0	0	0	0	0
ENSG00000220323	16	7	8	8	6	6	8	7	19	6	56	13	21	13	13	12	5	2	3	5	14	9	3	2	16	11	12	26	11	7	9	10
ENSG00000261716	330	256	253	207	151	197	129	181	264	214	1417	177	297	263	276	347	81	59	55	101	230	113	123	123	216	239	283	667	116	174	118	266
ENSG00000272993	0	0	0	0	0	0	0	0	0	0	0	0	0	0	0	0	0	0	0	0	0	0	0	0	0	0	0	0	0	0	0	0
ENSG00000203819	18	10	14	13	14	13	5	18	6	9	51	7	13	8	9	14	3	2	1	2	14	3	9	5	12	12	9	43	5	19	7	9
ENSG00000272196	0	0	0	0	0	0	0	0	0	0	0	0	0	0	0	0	0	1	0	0	1	0	0	1	0	0	0	0	0	0	0	0
ENSG00000203852	0	0	0	0	0	0	0	0	0	0	0	0	0	0	0	0	0	0	0	0	0	0	0	0	0	0	0	0	0	0	0	0
ENSG00000270276	0	0	0	0	0	0	0	0	0	0	0	0	0	0	0	0	0	0	0	0	0	0	0	0	0	0	0	0	0	0	0	1
ENSG00000264207	0	0	0	0	0	0	0	0	0	0	0	0	0	0	0	0	0	0	0	0	0	0	0	0	0	0	0	0	1	0	0	0
ENSG00000184678	55	37	45	42	76	88	54	87	64	73	677	41	91	76	92	130	41	22	28	47	196	89	142	93	78	106	187	276	72	86	131	117
ENSG00000184260	11	3	8	1	24	23	19	35	27	22	37	17	23	11	19	10	13	18	8	16	101	60	72	36	45	38	4	57	18	23	18	13
ENSG00000184270	15	10	7	5	6	1	0	4	7	5	11	0	6	1	5	2	10	7	8	15	16	6	9	2	5	1	2	20	4	6	2	11
ENSG00000178096	744	486	568	515	249	311	139	289	241	177	670	176	201	198	173	261	620	895	667	615	433	174	337	171	168	297	178	444	172	170	137	191
ENSG00000159164	2156	1318	1771	1464	3402	3529	2373	3529	2	6	24	5	6	5	10	8	459	524	557	917	3787	1511	2379	1261	5	7	7	16	3	6	3	11
ENSG00000143368	2154	1566	1906	1495	2175	2075	1510	2204	1772	1329	5564	1097	1582	1459	1700	1888	473	822	810	1277	3574	1445	2667	1312	1559	2568	1040	4087	1477	1724	1574	1834
ENSG00000014914	21	18	30	14	10	7	4	10	30	23	76	8	20	36	25	45	6	8	4	11	14	4	7	5	10	18	20	48	10	32	9	45
ENSG00000264522	222	225	248	216	398	415	284	415	742	607	3052	527	832	791	896	907	51	85	62	141	476	253	437	240	704	1131	729	2111	519	802	584	873
ENSG00000223945	3	2	1	1	1	3	3	0	7	4	20	1	10	12	10	10	1	0	0	3	0	1	0	2	4	3	7	3	4	4	8	10
ENSG00000136631	1211	814	1169	791	931	951	806	1059	727	520	2423	414	525	446	606	595	838	916	1138	1016	2064	799	1589	1001	679	950	577	1748	572	561	755	814
ENSG00000023902	1656	1078	1872	1205	2671	2744	2009	2626	2330	1816	7432	1393	882	901	1166	1117	637	950	1529	1712	4508	2010	3742	1669	1895	2835	1735	4442	876	1044	961	1140
ENSG00000266187	0	0	0	0	0	0	0	0	0	0	0	0	1	0	0	0	0	0	0	0	1	0	0	1	0	0	0	0	0	0	0	1
ENSG00000143401	17174	11277	13924	11819	10729	10284	8167	10742	9494	6881	18086	4106	6623	6229	7410	8065	12360	15353	14502	14243	24884	10141	17135	9124	8778	12388	2330	14670	7259	7997	8683	9918
ENSG00000222222	1	5	0	7	3	2	1	4	4	2	12	2	4	2	0	3	0	1	1	0	6	3	2	2	2	0	4	4	1	2	1	6
ENSG00000276110	5	2	2	1	2	0	2	0	0	0	4	0	0	0	0	0	0	0	0	2	1	1	0	0	0	1	1	2	0	0	0	0
ENSG00000118298	66	79	61	61	76	83	69	88	40	46	126	21	62	36	28	32	25	10	17	43	86	63	64	42	42	37	45	113	12	33	22	40
ENSG00000117362	2853	1940	2383	1910	2266	2360	1761	2408	2605	2141	8133	1778	2062	1839	2057	2433	1360	2245	2432	2229	3915	1772	3361	1348	2347	3728	1733	5533	2202	2290	2335	2469
ENSG00000118292	44	27	37	22	21	27	18	24	32	24	139	13	35	22	33	41	22	15	35	35	35	8	26	20	15	23	27	51	13	22	18	30
ENSG00000159208	26	9	17	24	416	396	325	476	4	1	25	6	61	41	47	44	5	1	2	19	110	48	78	59	2	3	6	14	29	31	9	42
ENSG00000266472	1627	1075	1199	1119	1261	1272	897	1365	1422	937	4268	802	1555	1377	1657	1942	1956	2215	2150	1958	2975	1285	2339	1463	1025	1592	951	2565	1789	1810	2489	2316
ENSG00000278939	3	4	4	1	5	8	5	6	6	3	20	3	5	6	7	6	4	2	3	2	1	4	11	2	5	5	2	14	1	12	7	7
ENSG00000117360	1883	1181	1433	1294	1182	1229	959	1371	1534	1177	5517	859	1228	1109	1419	1525	906	1025	1043	1359	2396	1022	1798	1003	1211	1891	1401	3439	1274	1657	1632	1969
ENSG00000163125	1965	1486	1790	1469	2424	2401	1667	2594	1851	1571	6724	1197	1466	1373	1271	1701	445	636	550	1047	3478	1342	2315	1193	1417	2313	1483	4424	792	1213	782	1182
ENSG00000143374	979	663	810	747	1353	1271	1002	1389	849	686	2730	489	1150	1019	1211	1237	296	467	387	489	2101	829	1579	742	627	1068	566	1611	857	1010	883	1076
ENSG00000143369	361	255	460	270	150	142	98	168	22	23	129	20	11	15	19	25	79	73	141	178	83	35	48	33	11	22	26	55	5	23	8	27
ENSG00000228126	16	13	22	19	6	1	0	6	2	2	9	0	2	7	8	9	6	9	6	19	0	1	2	4	1	0	4	3	2	0	2	6
ENSG00000237781	0	0	4	1	5	9	7	10	4	3	33	3	2	1	3	1	0	0	0	6	24	13	12	12	8	2	6	11	0	3	8	2
ENSG00000143382	22	7	22	16	70	103	81	96	19	26	207	13	8	19	21	39	7	3	8	32	164	98	116	126	20	23	77	117	7	42	14	47
ENSG00000264553	0	0	0	0	1	0	0	1	0	0	2	1	0	1	0	1	0	0	0	2	3	3	1	3	0	0	0	0	2	2	1	1
ENSG00000203804	25	11	20	11	29	28	38	37	20	20	87	10	12	9	15	15	5	4	3	13	44	48	30	40	7	30	24	74	7	13	13	31
ENSG00000277452	1	2	1	2	5	12	4	12	2	3	17	0	0	1	3	4	2	0	0	0	11	9	8	8	8	8	6	12	2	1	3	5
ENSG00000274963	3	2	3	3	11	12	11	9	1	3	25	4	2	2	1	5	0	0	1	3	19	2	9	14	1	4	5	17	2	4	2	3
ENSG00000143384	5457	3553	4420	3464	3982	4241	2855	4305	4711	4133	21881	3233	2408	2277	3025	2989	1854	2438	2570	3319	7254	2916	4753	2739	4028	6215	6097	12464	2161	2697	2465	3030
ENSG00000253047	18	12	13	11	5	6	6	2	8	11	24	4	4	11	8	11	2	0	0	0	4	0	0	0	1	9	9	22	0	3	1	6
ENSG00000143420	5478	3576	4562	3791	4542	4583	3450	4958	4102	3161	13326	2663	2911	2957	3445	3975	3648	4306	4625	4906	9345	3820	7409	3524	3942	5680	3054	9430	3459	3542	3979	4589
ENSG00000276103	15	16	16	9	3	4	3	2	11	8	19	7	12	4	10	14	0	2	1	4	4	0	2	2	7	8	4	14	2	6	3	5
ENSG00000143457	879	576	711	589	663	739	481	755	561	441	1768	348	719	672	734	857	479	713	569	571	1044	414	783	331	526	711	460	1180	583	621	640	734
ENSG00000143452	0	0	0	0	1	0	2	0	0	1	15	4	12	4	17	13	0	0	0	2	0	0	0	0	0	1	3	8	5	4	11	15
ENSG00000206931	0	0	0	0	0	0	0	0	0	0	0	0	0	0	0	0	0	0	0	0	0	0	0	0	0	0	0	0	0	0	0	0
ENSG00000163131	51	24	60	32	239	216	167	275	804	575	3424	480	772	727	738	958	12	19	26	17	395	176	316	168	675	1000	663	1904	721	693	806	862
ENSG00000236713	0	0	0	1	0	0	0	0	1	1	2	0	0	0	0	0	0	0	0	0	2	0	1	0	0	0	0	0	0	0	0	0
ENSG00000143387	98	76	84	68	78	92	67	116	99	92	347	57	42	57	28	50	27	24	19	70	119	64	76	72	54	75	111	177	23	58	27	72
ENSG00000224690	2	0	2	0	0	0	3	6	4	0	7	2	0	0	1	0	0	0	0	1	0	0	0	1	3	3	6	2	1	2	1	0
ENSG00000143437	1739	1124	1455	1215	1644	1753	1344	1846	1273	1067	4393	681	743	696	625	852	419	506	620	778	2529	1152	1911	1043	946	1573	1284	2894	420	572	460	670
ENSG00000200175	0	1	0	0	2	0	0	1	0	0	0	1	0	1	0	0	0	0	0	0	0	1	0	1	0	0	0	0	0	1	2	0
ENSG00000224800	0	1	0	0	0	0	0	1	0	0	3	0	1	0	0	0	0	0	0	0	1	0	0	0	0	0	1	2	0	0	0	0
ENSG00000283324	0	0	0	0	0	0	0	0	0	0	2	0	0	0	1	0	0	2	2	0	0	0	0	1	0	0	0	0	0	0	0	0
ENSG00000229357	0	0	0	0	0	0	0	0	0	0	0	0	2	0	0	0	0	0	0	0	0	0	0	0	0	0	0	0	0	0	0	0
ENSG00000143379	1406	985	1258	1047	1938	2008	1455	2078	1509	1216	5404	791	1173	1122	1047	1306	448	431	443	966	2710	1263	1836	1154	925	1523	1206	3115	588	990	694	1089
ENSG00000143418	2981	2251	2584	2297	2859	2760	2138	2926	1940	1603	7725	1470	1512	1379	1791	1858	1237	2136	1679	2423	5044	2128	3824	1702	1987	3281	1813	5367	1519	1697	1715	1828
ENSG00000259357	0	0	0	0	0	0	0	0	0	0	0	0	0	0	0	0	0	0	0	0	0	0	0	0	0	0	0	0	0	0	0	0
ENSG00000231073	10	10	17	18	11	15	8	9	18	9	38	1	8	4	8	4	3	1	2	16	22	14	11	13	7	13	9	32	0	9	3	15
ENSG00000143412	66	43	59	44	154	176	133	216	62	48	224	41	114	93	109	150	11	33	23	30	220	81	175	78	6	39	42	91	80	80	64	96
ENSG00000143409	159	90	135	118	339	405	229	386	130	115	612	89	176	202	181	247	22	33	61	89	487	194	362	175	75	107	120	269	75	134	86	137
ENSG00000143363	952	664	818	618	561	546	345	584	533	466	1736	338	288	307	312	344	351	478	456	521	651	244	544	250	444	751	443	1258	272	319	303	348
ENSG00000200759	1	2	1	0	1	2	1	3	1	2	2	0	0	1	1	0	2	0	2	2	3	1	1	1	0	0	0	0	0	2	0	1
ENSG00000163141	69	56	81	52	20	20	10	13	32	18	100	17	13	33	16	42	12	10	11	42	17	13	9	13	22	30	20	43	7	19	11	23
ENSG00000143443	48	36	37	32	81	65	60	95	30	32	107	16	55	40	45	60	7	11	7	33	91	41	89	52	42	48	43	78	24	41	35	51
ENSG00000197622	2647	1660	2201	1604	2038	2044	1525	2023	2676	2353	13412	2115	1706	1419	1964	1964	966	1230	1226	1509	3116	1333	2322	1218	2521	4014	3370	7962	1569	1620	1819	2011
ENSG00000213190	823	477	725	488	2224	2012	1528	2148	370	293	1291	232	114	90	127	118	500	550	773	638	3758	1396	2554	1354	243	328	303	674	64	108	99	111
ENSG00000143458	2094	1402	1798	1462	80	72	64	93	1032	795	3561	642	829	755	991	1069	745	921	954	1264	183	49	122	59	776	1246	757	2393	755	1044	911	931
ENSG00000233717	1	0	1	0	1	0	0	0	1	0	15	0	0	0	2	0	0	0	0	1	0	0	0	0	0	1	0	6	0	1	0	3
ENSG00000261168	7	10	10	10	0	0	2	0	9	2	23	4	1	2	0	6	5	1	2	6	0	0	1	0	2	6	4	12	2	3	1	2
ENSG00000143434	323	273	332	322	44	81	34	66	130	136	605	93	55	68	51	86	77	65	77	234	38	13	30	21	67	138	207	297	18	44	32	49
ENSG00000163154	36	22	29	18	56	79	49	80	189	110	1005	128	124	111	119	139	8	13	17	26	127	57	113	58	115	169	278	480	77	108	126	133
ENSG00000163156	617	417	605	478	560	587	499	616	558	367	1884	309	707	657	859	854	510	529	652	477	1185	476	1101	461	427	629	468	1178	759	683	997	871
ENSG00000163155	432	329	392	305	231	278	199	244	207	152	767	124	156	119	141	144	157	146	177	269	317	169	284	166	157	238	203	434	101	107	92	103
ENSG00000163157	0	0	0	0	0	0	0	0	0	0	0	0	0	0	0	0	0	0	0	0	0	0	0	0	0	0	0	0	0	0	0	0
ENSG00000163159	1289	853	1023	970	1177	1100	831	1238	796	634	2907	487	764	657	807	997	514	784	703	889	1776	701	1380	618	686	1031	636	1646	640	710	807	760
ENSG00000143398	2635	1819	2430	1881	2121	2160	1642	2255	1350	1135	6109	934	1840	1460	1938	1998	762	1076	1352	1601	3185	1336	2457	1310	1541	2272	1466	4034	1682	1914	1875	2001
ENSG00000159352	4960	3230	3996	3709	3165	3126	2371	3253	3326	2258	10511	1950	3451	3093	3848	4184	4317	5616	5397	4890	6917	2991	5346	2381	2786	4451	2287	6763	4137	3800	4508	4608
ENSG00000232671	14	16	14	12	26	25	12	26	28	22	29	12	14	16	15	14	4	4	7	15	48	14	39	21	16	9	7	34	8	20	17	16
ENSG00000143373	1027	727	791	703	1018	1111	775	1134	932	806	3182	609	561	579	618	775	181	316	280	497	1490	649	1133	582	744	1239	786	2044	347	460	364	529
ENSG00000143393	1568	1110	1320	1108	1681	1714	1283	1817	1298	1107	5613	976	1068	1095	1134	1420	457	651	629	962	2209	1001	1658	914	1090	1805	1344	3261	754	1066	842	1163
ENSG00000265753	2	2	4	3	5	8	5	1	3	3	25	4	2	2	4	3	0	0	0	3	5	3	2	4	6	7	4	20	0	0	1	3
ENSG00000273481	0	0	2	0	0	1	0	0	0	0	6	1	1	0	0	0	3	0	1	0	0	0	0	0	1	0	0	3	0	0	0	0
ENSG00000143390	3589	2565	3083	2777	2844	2915	2249	3151	1702	1310	8120	1138	3154	2818	2996	3651	1852	2517	2017	2689	4597	2045	4062	1899	1878	2703	1742	4738	1919	2195	2064	2679
ENSG00000224645	0	0	0	0	0	0	0	0	0	0	0	0	0	0	0	0	0	0	0	0	0	0	0	0	0	0	0	0	0	0	0	0
ENSG00000237976	32	15	29	13	66	57	48	84	12	3	44	7	50	44	60	66	33	77	68	57	181	74	209	101	12	12	6	23	59	48	71	50
ENSG00000143416	0	1	0	0	0	1	0	3	0	0	0	0	0	0	0	0	0	0	1	1	2	0	4	1	0	0	0	0	0	1	0	0
ENSG00000159377	4749	3309	3745	3196	2637	2711	1842	2765	3042	2338	11038	2144	2833	2639	3055	3547	7081	10071	8312	5545	6891	2640	5106	2445	3077	4747	2322	6791	3899	3475	4648	4450
ENSG00000143442	3347	2672	3082	2643	3015	3097	2291	3276	2254	1820	8024	1348	1440	1468	1570	1749	747	939	819	1918	4296	1876	2913	1759	1800	2719	2032	5461	724	1383	935	1498
ENSG00000238711	10	5	10	6	7	17	7	15	4	10	25	5	3	2	2	3	5	2	1	7	11	7	6	8	9	10	10	31	0	4	3	12
ENSG00000143375	588	572	573	616	211	262	197	246	16	38	30	21	242	249	243	328	177	219	201	453	327	151	238	133	7	25	6	49	133	184	119	222
ENSG00000252840	1	1	6	2	0	0	0	0	0	0	0	0	1	0	0	1	0	0	0	1	0	0	0	0	0	0	0	0	0	2	0	0
ENSG00000143367	14	16	7	32	0	1	0	2	10	11	34	11	195	192	230	272	1	9	1	9	1	0	0	2	10	20	5	37	97	135	150	193
ENSG00000232536	1	1	0	0	0	0	0	0	0	0	0	0	1	3	0	2	0	0	0	1	0	0	0	0	0	0	0	0	1	1	0	0
ENSG00000207606	0	0	0	0	0	0	0	0	0	0	0	0	0	1	1	2	0	0	0	0	0	0	0	0	0	0	0	0	0	4	0	0
ENSG00000223861	0	0	1	0	0	0	0	0	0	0	1	0	1	0	0	1	0	0	0	0	0	0	0	0	0	0	0	0	0	1	2	2
ENSG00000250734	15	22	25	8	18	12	13	5	10	4	19	5	8	2	7	13	5	10	4	15	13	12	6	10	6	5	6	21	8	7	4	14
ENSG00000143376	2099	1496	1692	1455	1102	1094	875	1140	928	694	2995	530	1105	1107	1016	1340	419	660	538	1329	1404	638	1047	622	769	1158	732	1932	822	1128	717	1133
ENSG00000206635	6	3	0	4	3	0	0	4	2	0	4	0	0	0	1	2	0	0	1	1	1	0	2	0	0	2	3	3	1	2	1	2
ENSG00000227045	4	6	3	2	3	8	5	3	2	1	3	2	2	1	1	2	1	0	1	1	7	3	2	1	0	2	1	6	0	0	0	0
ENSG00000159409	5	7	7	14	4	7	3	6	1	0	2	0	4	3	5	7	0	0	1	5	4	4	2	3	1	1	4	3	2	0	4	3
ENSG00000178796	2	1	1	0	0	2	0	1	0	0	0	0	0	0	0	1	0	2	1	0	1	0	0	0	0	0	0	0	0	0	0	0
ENSG00000206980	0	0	0	2	1	1	0	2	0	0	2	0	0	0	0	0	0	1	0	3	1	0	0	0	1	0	0	0	0	0	0	0
ENSG00000269621	4	0	4	0	2	0	0	4	2	0	2	1	0	0	3	1	0	1	0	9	3	0	2	0	3	2	0	1	1	0	2	3
ENSG00000236940	2	0	0	1	1	0	0	0	0	3	7	0	0	0	0	3	0	1	0	2	3	0	0	1	1	1	0	4	0	1	0	3
ENSG00000143436	1584	1066	1426	1202	1447	1375	1076	1545	993	749	3114	595	1380	1313	1400	1626	1199	1638	1552	1392	2828	1241	2276	940	909	1424	659	2015	1364	1406	1467	1573
ENSG00000143450	96	62	66	50	53	34	45	65	25	28	178	27	8	12	13	24	19	30	30	56	67	21	52	25	26	32	41	113	3	14	13	18
ENSG00000249602	48	57	32	38	14	20	8	20	15	17	75	10	4	6	4	4	8	7	6	34	7	6	8	6	12	18	21	55	2	11	2	5
ENSG00000232937	0	0	0	0	0	0	0	0	0	0	0	0	0	0	0	0	0	0	0	0	0	0	0	0	0	0	0	0	0	0	0	0
ENSG00000268288	6	4	2	6	6	1	6	2	0	2	14	1	2	3	0	3	0	0	2	2	0	1	0	3	4	3	8	10	0	0	0	1
ENSG00000182134	1466	1035	1191	1096	438	490	350	572	475	380	2025	376	134	237	160	282	542	580	590	659	528	245	424	203	475	743	504	1516	93	147	88	144
ENSG00000203288	43	39	23	16	11	13	5	11	12	14	97	16	8	4	11	9	7	7	14	14	26	11	16	9	14	19	23	49	2	7	11	5
ENSG00000269489	14	9	9	8	3	3	4	6	1	5	20	3	3	0	0	2	3	2	0	3	5	3	1	1	3	6	2	8	0	3	1	2
ENSG00000213171	6	0	0	2	1	0	0	0	0	3	3	0	0	0	0	0	1	0	4	7	1	0	5	0	0	0	3	3	0	0	1	0
ENSG00000143365	28	17	39	20	20	20	17	22	7	1	10	3	2	1	0	6	26	13	23	32	32	24	16	14	2	0	3	4	1	3	3	1
ENSG00000225556	7	7	14	7	0	1	0	0	0	0	4	0	16	11	22	13	2	3	0	5	0	0	0	0	0	1	0	0	10	11	10	13
ENSG00000201134	1	2	2	2	0	0	0	1	0	0	1	0	3	3	4	2	1	2	4	2	2	1	0	0	0	0	1	0	1	1	1	1
ENSG00000234614	23	5	20	12	0	1	0	1	0	2	4	0	16	12	5	23	8	17	22	14	6	0	0	0	0	0	3	2	6	7	11	12
ENSG00000196407	17	8	15	14	0	0	2	0	0	0	6	0	23	23	20	24	6	8	18	17	3	1	6	2	0	0	1	3	9	12	5	8
ENSG00000159445	1	0	1	1	0	0	0	0	567	452	1943	320	389	380	340	461	0	1	1	0	0	0	0	0	453	653	523	1268	404	508	369	536
ENSG00000223810	0	0	0	0	0	0	0	0	0	0	0	0	0	0	0	0	0	0	0	0	0	0	0	0	0	0	0	1	0	0	0	0
ENSG00000197747	711	896	660	894	842	909	571	1017	1800	1385	4060	905	715	772	630	980	87	220	119	221	769	281	517	282	1169	1474	791	2401	361	435	325	482
ENSG00000229021	1	2	2	0	3	6	3	3	5	6	13	6	4	5	3	0	0	0	0	0	2	2	1	0	1	6	2	12	1	1	5	2
ENSG00000240667	0	0	0	0	0	0	0	0	0	0	0	0	0	0	0	0	0	0	0	0	0	0	0	0	0	0	0	0	0	0	0	0
ENSG00000163191	0	1	0	0	1666	1861	1348	1993	1329	919	7848	1086	1488	1523	1480	2037	1	0	0	0	3183	1238	2237	1392	1562	2138	2044	5717	1744	1850	1792	2426
ENSG00000232678	0	0	0	0	0	0	0	0	0	0	0	0	0	0	0	0	0	0	0	0	0	0	0	0	0	0	0	0	0	0	1	0
ENSG00000182898	0	0	0	0	0	0	1	0	0	0	0	0	0	1	0	0	0	0	0	0	3	4	2	0	0	0	0	0	0	0	0	0
ENSG00000159450	15	24	35	47	0	0	0	0	15	20	34	16	18	32	18	31	14	8	35	35	0	0	0	0	4	13	3	35	27	52	27	92
ENSG00000226716	0	0	0	0	0	0	0	0	0	0	0	0	0	0	0	0	0	0	0	0	0	0	0	0	0	0	0	0	0	0	0	0
ENSG00000233792	0	0	0	0	0	0	1	0	1	2	0	1	2	1	0	2	0	0	0	0	0	0	0	0	0	1	0	4	0	0	1	0
ENSG00000215853	0	0	0	0	0	1	0	0	0	0	2	0	0	0	0	0	0	0	0	0	0	0	0	1	0	0	0	0	0	0	0	0
ENSG00000237975	0	0	0	0	1	0	3	5	50	39	235	19	82	92	75	126	0	0	0	0	1	0	2	0	16	25	37	91	35	81	60	81
ENSG00000236427	0	0	0	0	0	0	0	1	4	3	6	0	4	3	5	4	0	0	0	0	2	0	0	0	1	0	5	4	2	6	4	8
ENSG00000197915	0	0	0	0	1	0	0	2	9	15	86	4	63	75	110	110	0	0	0	0	9	1	6	6	9	12	18	52	36	126	106	165
ENSG00000143631	0	0	0	0	5	4	2	1	71	88	423	63	115	121	99	198	0	0	0	0	6	0	3	2	37	66	64	135	70	163	103	206
ENSG00000143520	0	0	0	0	0	0	0	0	3	9	36	1	7	12	10	24	1	0	0	0	1	0	0	0	1	4	4	7	3	24	5	15
ENSG00000183586	0	0	0	0	0	0	0	0	0	0	0	0	0	0	0	0	0	0	0	0	0	0	0	0	0	0	0	0	0	0	0	2
ENSG00000143536	0	0	0	0	0	0	0	0	0	0	2	0	0	0	0	0	0	0	0	0	0	0	0	0	0	0	0	0	0	0	0	1
ENSG00000186207	0	0	2	0	0	0	0	0	0	0	0	0	0	0	0	0	0	0	0	0	0	0	0	0	0	0	0	0	0	0	0	0
ENSG00000279850	0	0	0	0	0	0	0	0	0	0	0	0	0	0	0	0	0	0	0	0	0	0	0	0	0	0	0	0	0	0	0	0
ENSG00000169509	0	0	0	0	0	0	0	0	0	0	0	0	0	0	0	0	0	0	0	0	0	0	0	0	0	0	0	0	0	0	0	0
ENSG00000185966	0	0	0	0	0	0	0	0	0	0	0	0	0	0	0	0	0	0	0	0	0	0	0	0	0	0	0	0	0	0	0	0
ENSG00000163202	0	0	0	0	0	0	0	0	0	0	0	0	0	0	0	0	0	0	0	0	0	0	0	0	0	0	0	0	0	0	0	0
ENSG00000244057	0	0	0	0	0	0	0	0	0	0	0	0	0	0	0	0	0	0	0	0	0	0	0	0	0	0	0	0	0	0	0	0
ENSG00000187238	0	0	0	0	0	0	0	0	0	0	0	0	0	0	0	0	0	0	0	0	0	0	0	0	0	0	0	0	0	0	0	0
ENSG00000185962	0	0	0	0	0	0	0	0	0	0	0	0	0	0	0	0	1	1	0	0	0	0	0	0	0	0	0	0	0	0	0	0
ENSG00000226947	0	0	0	0	0	0	0	0	0	0	0	0	0	0	0	0	0	0	0	0	0	0	0	0	0	0	0	0	0	0	0	0
ENSG00000176075	0	0	0	0	0	0	0	0	0	0	0	0	0	0	0	0	0	0	0	0	0	0	0	0	0	0	0	0	0	0	0	0
ENSG00000187223	0	0	0	0	0	0	0	0	0	0	0	0	0	0	0	0	0	0	0	0	0	0	0	0	0	0	0	0	0	0	0	0
ENSG00000187180	0	0	0	0	0	0	0	0	0	0	0	0	0	0	0	0	0	0	0	0	0	0	0	0	0	0	0	0	0	0	0	0
ENSG00000159455	0	0	0	0	0	0	0	0	0	0	0	0	0	0	0	0	0	0	0	0	0	0	0	0	0	0	0	0	0	0	0	0
ENSG00000187173	0	0	0	0	0	0	0	0	0	0	0	0	0	0	0	0	0	0	0	0	0	0	0	0	0	0	0	0	0	0	0	0
ENSG00000187170	0	0	0	0	0	0	0	0	0	0	0	0	0	0	0	0	0	0	0	0	0	0	0	0	0	0	0	0	0	0	0	0
ENSG00000198854	0	0	0	0	0	0	0	0	0	0	0	0	0	0	0	0	0	0	0	0	0	0	0	0	0	0	0	0	0	0	0	0
ENSG00000229713	0	0	0	0	0	0	0	0	0	0	0	0	0	0	0	0	0	0	0	0	0	0	0	0	0	0	0	0	0	0	0	0
ENSG00000233819	0	0	0	0	0	0	0	0	0	0	0	0	0	0	0	0	0	0	0	0	0	0	0	0	0	0	0	0	0	0	0	0
ENSG00000203786	0	0	0	0	0	0	0	0	0	0	0	0	0	0	0	0	0	0	0	0	0	0	0	0	0	0	0	0	0	0	0	0
ENSG00000240386	0	0	0	0	0	0	0	0	0	0	0	0	0	0	0	0	0	0	0	0	0	0	0	0	0	0	0	0	0	0	0	0
ENSG00000186226	0	0	0	0	0	0	0	0	0	0	75	2	0	0	0	0	0	0	0	0	0	0	0	0	0	0	2	2	0	0	0	0
ENSG00000172155	0	0	0	0	0	0	0	0	0	0	3	0	0	0	0	0	0	0	0	0	0	0	0	0	0	0	0	0	0	0	0	0
ENSG00000197084	0	0	0	0	0	0	0	0	0	0	5	2	0	0	0	0	0	0	0	0	0	0	0	0	0	0	0	3	0	0	0	0
ENSG00000196734	0	0	0	0	0	0	0	0	0	0	12	0	0	0	0	0	0	0	0	0	0	0	0	1	0	0	0	1	0	0	0	0
ENSG00000186844	0	0	0	0	0	0	0	0	0	0	0	0	0	0	0	0	0	0	0	0	0	0	0	0	0	0	0	0	0	0	0	0
ENSG00000235942	0	0	0	0	0	0	0	0	0	0	0	0	0	0	0	0	0	0	0	0	0	0	0	0	0	0	0	0	0	0	0	0
ENSG00000163206	0	0	0	0	0	0	0	0	0	0	0	0	0	0	0	0	0	0	0	0	0	0	0	0	0	0	0	0	0	0	0	0
ENSG00000163207	0	0	0	0	0	0	0	0	0	0	0	0	0	0	0	0	0	0	0	0	0	0	0	0	0	0	0	0	0	0	0	0
ENSG00000224308	0	0	0	0	0	0	0	0	0	0	0	0	0	0	0	0	0	0	0	0	0	0	0	0	0	0	0	0	0	0	0	0
ENSG00000283227	0	0	0	0	0	0	0	0	0	0	0	0	0	0	0	0	0	0	0	0	0	0	0	0	0	0	0	0	0	0	0	0
ENSG00000184148	0	0	0	0	0	0	0	0	0	0	0	0	0	0	0	0	0	0	0	0	0	0	0	0	0	0	0	0	0	0	0	0
ENSG00000169474	0	0	0	0	0	0	0	0	0	0	0	0	0	0	0	0	0	0	0	0	0	0	0	0	0	0	0	0	0	0	0	0
ENSG00000163209	0	0	0	0	0	0	0	0	0	0	0	0	0	0	0	0	0	0	0	0	0	0	0	0	0	0	0	0	0	0	0	0
ENSG00000252920	0	0	0	0	0	0	0	0	0	0	0	0	0	0	0	0	0	0	0	0	0	0	0	0	0	0	0	0	0	0	0	0
ENSG00000234262	0	0	0	0	0	0	0	0	0	0	0	0	0	0	0	0	0	0	0	0	0	0	0	0	0	0	0	0	0	0	0	0
ENSG00000169469	0	0	0	0	0	0	0	0	0	0	0	0	0	0	0	0	0	0	0	0	0	0	0	0	0	0	0	0	0	0	0	0
ENSG00000163216	0	0	0	0	0	0	0	0	0	0	0	0	0	0	0	0	0	0	0	0	0	0	0	0	0	0	0	0	0	0	1	1
ENSG00000241794	0	0	0	0	0	0	0	0	0	0	2	0	0	0	0	0	0	0	0	0	0	0	0	0	0	0	0	0	0	0	0	0
ENSG00000196805	0	0	0	0	0	0	0	0	0	0	0	0	0	0	0	0	0	0	0	0	0	0	0	0	0	0	0	0	0	0	0	0
ENSG00000203785	0	0	0	0	0	0	0	0	0	0	0	0	0	0	0	0	0	0	0	0	0	0	0	0	0	0	0	0	0	0	0	0
ENSG00000244094	0	0	0	0	0	0	0	0	0	0	0	0	0	0	0	0	0	0	0	0	0	0	0	0	0	0	0	0	0	0	0	0
ENSG00000229035	0	0	0	0	0	0	0	0	0	0	0	0	0	0	0	0	0	0	0	0	0	0	0	0	0	0	0	0	0	0	0	0
ENSG00000159516	0	0	0	0	0	0	0	0	0	0	0	0	1	2	0	4	0	0	0	0	0	0	0	0	0	0	0	0	0	0	0	1
ENSG00000229699	0	0	0	0	0	0	0	0	0	0	0	0	4	3	3	5	2	0	0	0	0	0	0	0	0	0	0	0	0	1	2	2
ENSG00000203784	0	0	0	0	0	0	0	0	0	0	0	0	0	1	0	1	0	0	0	0	0	0	0	0	0	0	0	0	0	1	0	0
ENSG00000203783	0	0	0	0	0	2	1	1	0	0	0	0	1	0	2	1	0	0	0	0	0	1	0	0	0	0	0	2	3	1	0	6
ENSG00000230779	0	0	0	0	0	0	0	0	0	0	0	0	0	2	0	0	0	0	0	0	0	0	0	0	0	0	0	0	0	1	0	0
ENSG00000203782	0	0	0	0	0	0	0	1	0	0	0	0	14	14	11	27	0	0	0	0	0	0	0	0	0	0	0	0	7	16	17	25
ENSG00000159527	3	0	1	0	0	0	0	0	0	0	0	0	69	64	63	55	0	0	1	0	0	0	0	0	0	0	0	0	28	45	47	50
ENSG00000163218	2	6	4	3	2	1	0	3	0	0	0	0	232	169	238	258	1	2	0	2	0	1	0	0	0	0	0	0	109	173	142	190
ENSG00000207321	0	0	0	0	0	0	0	0	0	0	0	0	3	7	0	5	0	0	0	0	0	0	0	0	0	0	0	0	8	10	2	2
ENSG00000163220	0	1	0	2	0	0	0	0	0	0	0	0	0	0	0	0	1	12	6	5	0	0	0	0	0	0	0	0	0	0	0	0
ENSG00000163221	0	0	0	0	0	0	0	0	0	0	0	0	0	0	0	0	0	0	0	0	0	0	0	0	0	0	0	0	0	0	0	0
ENSG00000237008	0	0	0	0	0	0	0	0	0	0	0	1	0	0	0	0	0	0	0	0	0	0	0	0	0	0	0	0	0	0	0	0
ENSG00000143546	0	0	0	0	0	0	0	0	0	0	0	0	0	0	0	0	0	0	0	0	0	0	0	0	0	0	0	0	0	0	0	0
ENSG00000224784	0	0	0	0	0	0	0	0	0	0	0	0	0	0	0	0	0	0	0	0	0	0	0	0	0	0	0	0	0	0	0	0
ENSG00000184330	0	0	0	0	0	0	0	0	0	0	0	0	0	0	0	0	0	0	0	0	0	0	0	0	0	0	0	0	0	0	0	0
ENSG00000203781	0	0	0	0	0	0	0	0	0	0	0	0	0	0	0	0	0	0	0	0	0	0	0	0	0	0	0	0	0	0	0	0
ENSG00000197364	0	0	0	0	0	0	0	0	0	0	0	0	0	0	0	0	0	0	0	0	0	0	0	0	0	0	0	0	0	0	0	0
ENSG00000143556	0	0	2	0	0	0	0	0	0	0	0	0	0	0	0	0	0	0	0	0	0	0	0	0	0	0	0	0	0	0	0	0
ENSG00000263841	0	0	0	0	0	0	0	0	0	0	0	0	0	0	0	0	0	0	0	0	0	0	0	0	0	0	0	0	0	0	0	0
ENSG00000238279	0	0	0	0	8	4	0	4	0	0	0	0	1	4	3	8	0	0	0	0	5	2	5	2	1	0	0	1	0	1	4	3
ENSG00000197956	0	1	0	0	2093	2210	1332	2445	387	290	1076	240	1047	1105	965	1427	0	0	1	0	4258	1638	2849	1798	261	401	126	452	1136	1035	1389	1309
ENSG00000196420	8	9	4	3	15	15	9	23	2	3	11	0	14	6	12	15	5	3	21	15	23	11	14	8	1	1	0	3	5	5	18	9
ENSG00000196154	42	16	51	28	9999	10139	7039	11115	1715	1125	4265	812	4624	4305	4240	5559	48	69	160	69	21707	8958	16218	8649	1518	1965	601	2733	5533	4708	4920	5405
ENSG00000188015	0	0	0	0	4	5	3	3	1	0	1	1	10	9	10	17	0	0	0	0	3	0	2	3	1	0	0	0	1	7	3	4
ENSG00000196754	1	0	0	0	17	21	10	19	3	0	7	0	9	9	13	16	3	1	8	0	29	10	36	13	4	0	1	1	17	12	16	16
ENSG00000188643	1	0	0	0	3	0	0	0	0	0	0	0	1	0	2	1	0	0	0	0	0	0	0	0	0	0	0	0	0	0	4	0
ENSG00000189334	0	0	3	0	0	0	1	0	0	0	1	0	435	328	379	509	1	0	0	2	2	0	0	1	0	0	1	1	327	277	233	290
ENSG00000189171	30	27	49	30	100	126	110	141	121	99	433	79	170	135	133	150	31	17	19	52	200	130	132	117	62	111	75	174	63	116	98	139
ENSG00000271853	4	3	5	3	11	13	6	10	16	6	38	7	20	18	10	13	5	13	20	5	26	13	19	13	6	12	14	32	11	17	14	25
ENSG00000160678	36	29	44	39	87	130	85	132	107	73	349	63	170	151	152	198	16	30	16	78	198	108	139	133	72	108	63	147	74	150	91	190
ENSG00000272030	12	14	12	11	24	54	26	51	42	26	141	13	24	35	39	40	7	4	15	28	56	21	41	32	17	26	21	46	6	39	19	32
ENSG00000160679	3441	2334	2816	2499	2573	2681	1839	2667	2471	2017	8526	1563	3003	2788	3130	3644	2008	2560	2425	2786	4509	1902	3111	1705	2184	3338	1973	5348	2652	2958	2889	3454
ENSG00000143553	603	417	501	390	335	375	240	388	476	380	1574	310	323	274	300	385	684	928	931	693	625	256	519	264	407	587	386	980	344	367	357	427
ENSG00000143621	6012	4264	4778	4163	9131	8980	6657	9234	5024	3697	14369	3015	6600	5700	6732	7479	5397	7362	6259	5624	21054	7631	14975	6872	5450	8092	2543	10397	7733	7517	8317	8596
ENSG00000169418	2	1	0	3	0	0	1	1	0	1	0	1	1	3	1	6	0	0	0	0	0	0	0	0	3	4	1	8	0	2	0	5
ENSG00000274940	0	0	0	0	0	0	0	0	0	0	0	0	0	0	0	0	0	0	0	0	0	0	0	0	0	0	0	1	0	0	0	0
ENSG00000242565	0	0	0	0	0	0	0	0	0	0	0	0	0	0	0	0	0	0	0	0	0	0	0	0	0	0	0	0	0	0	0	0
ENSG00000235015	0	0	0	0	0	0	0	1	0	0	0	0	0	0	0	0	0	0	0	0	0	0	0	0	0	0	0	0	0	0	0	0
ENSG00000199565	0	0	0	0	0	0	0	0	0	0	1	1	0	0	0	0	0	1	0	0	1	0	0	0	0	0	1	0	0	0	0	0
ENSG00000143624	1778	1526	1790	1488	1569	1775	1397	2035	1931	1671	7053	1221	1431	1358	1580	1818	363	412	522	1270	2048	1018	1432	1046	1166	2140	1403	3925	801	1341	942	1482
ENSG00000243613	0	0	1	0	0	0	0	2	2	0	0	0	0	0	0	0	0	0	0	0	0	1	0	0	0	0	0	0	0	0	1	1
ENSG00000233222	1	1	3	2	2	0	1	0	0	0	6	1	0	2	1	0	0	0	0	0	0	0	1	0	0	1	0	3	0	1	1	1
ENSG00000279767	0	0	0	0	0	0	0	0	0	0	0	0	0	0	0	0	0	0	0	0	0	0	0	0	0	0	0	0	0	0	0	0
ENSG00000143554	120	85	147	114	98	126	95	110	665	607	3410	621	84	79	86	148	33	32	47	178	142	102	120	121	665	1107	882	2325	47	111	51	110
ENSG00000207039	0	0	0	0	0	0	0	0	0	0	0	0	0	0	1	0	0	0	0	0	0	0	0	0	0	0	1	0	0	0	0	0
ENSG00000143614	2657	2257	2510	2374	2582	2580	2076	2855	2367	1988	9245	1399	1828	1686	1860	2246	406	514	531	1081	3590	1580	2752	1491	2054	3416	2372	6261	1084	1517	1287	1639
ENSG00000231827	6	13	12	7	9	10	9	12	5	9	36	2	3	2	4	4	4	2	1	7	15	5	5	12	2	19	15	40	3	2	4	3
ENSG00000223599	8	6	28	6	13	8	4	21	32	13	60	9	6	2	9	6	10	4	6	12	21	12	21	18	9	29	23	72	3	9	13	9
ENSG00000236327	2	2	5	1	1	0	4	3	5	2	18	1	4	4	2	4	1	1	1	4	11	5	2	3	7	6	7	28	0	1	0	4
ENSG00000198837	3365	2647	3089	2693	2729	3067	2223	2970	2073	1827	8114	1431	1475	1342	1523	1673	843	863	835	2042	3491	1753	2491	1581	1604	2782	1729	4896	726	1343	839	1490
ENSG00000160741	1080	765	1003	758	1018	1040	793	1029	941	771	3718	609	920	703	1033	1080	340	350	350	562	1471	666	957	627	763	1166	875	2262	717	943	809	1079
ENSG00000143570	1599	1048	1455	1110	1365	1330	1062	1516	1343	1021	3607	825	996	1021	1309	1399	991	1514	1730	1467	2484	1094	1877	823	930	1564	746	2283	1059	1161	1377	1376
ENSG00000276584	4	0	1	0	0	0	0	0	1	0	4	0	0	0	0	0	1	0	0	0	0	0	0	0	0	0	0	0	1	0	0	2
ENSG00000282386	25	18	18	22	6	9	12	12	9	14	55	19	5	8	10	8	5	9	8	31	10	17	15	8	14	28	18	29	3	7	1	10
ENSG00000273026	26	15	16	15	12	7	9	14	10	15	41	11	10	6	9	17	5	6	8	19	30	6	12	11	5	13	8	21	5	7	4	9
ENSG00000143578	410	235	347	274	388	404	250	402	236	208	744	181	171	202	162	278	189	250	216	206	617	255	507	195	141	234	149	454	109	154	108	173
ENSG00000143543	2696	1715	2262	1760	1564	1571	1120	1660	1690	1338	5541	1153	1745	1525	1950	2050	2838	3819	3459	3058	3285	1390	2673	1391	1775	2557	1084	3534	2286	2132	2543	2525
ENSG00000272654	110	53	100	83	28	36	24	38	35	22	114	28	40	38	37	53	45	79	49	61	52	22	34	27	10	40	20	53	35	22	39	34
ENSG00000143545	7	2	9	3	165	241	142	220	0	1	5	0	6	5	9	5	1	0	7	4	239	125	249	88	1	0	0	2	0	1	3	3
ENSG00000177954	13299	8674	9283	8260	5886	5994	3569	6092	15769	12093	55823	10834	10091	10024	10964	12758	15438	18064	17626	15027	15932	6112	11748	8229	13993	18439	13158	31683	13329	11315	17298	15268
ENSG00000143552	77	61	85	59	10	12	16	5	34	33	118	25	16	8	11	10	25	20	18	83	33	16	10	13	29	50	33	88	7	10	14	9
ENSG00000231416	5	1	1	1	3	3	4	2	1	0	3	1	3	0	1	1	0	1	1	2	4	2	2	1	1	3	0	6	1	3	2	2
ENSG00000200220	3	3	8	1	0	0	0	0	0	0	7	3	0	0	0	0	1	0	0	4	0	1	0	0	1	1	0	1	0	0	0	0
ENSG00000231837	0	0	0	1	0	0	0	0	0	0	0	0	0	0	0	0	0	0	0	0	0	0	0	0	0	0	0	0	0	0	0	0

ENSG00000263987	0	0	0	0	0	0	0	0	1	0	0	0	0	0	0	0	0	0	0	0	0	0	0	0	1	0	0	0	0	1	0	0
ENSG00000143549	13121	8686	11017	9073	15932	15779	12328	16962	9189	6667	26765	5115	9087	8146	9824	10812	11923	15315	15155	10869	38624	15092	27170	12749	9352	13864	5187	19125	9927	9539	11543	11624
ENSG00000264384	13	11	8	7	14	13	15	20	14	13	46	4	6	4	9	2	0	0	4	11	45	14	18	14	14	12	4	41	4	3	4	9
ENSG00000215938	0	0	0	0	0	0	0	4	0	0	0	0	0	1	0	0	0	0	0	0	0	0	0	0	0	0	0	1	0	0	0	0
ENSG00000163263	4	4	6	3	9	8	2	9	3	2	10	3	1	0	2	3	6	9	3	11	14	4	5	4	0	1	1	9	1	2	1	1
ENSG00000143612	4123	2824	3307	2885	3403	3321	2415	3469	2605	2056	9587	1812	3165	2946	3340	3886	3537	5585	4829	3604	6195	2496	4816	2077	2513	3838	2112	5926	3338	3224	4059	3813
ENSG00000143569	4856	3332	4512	3640	4939	5416	3922	5643	4238	3574	16118	2809	3798	3451	3991	4516	1283	1626	1623	3324	7526	3451	5089	3172	3636	6257	3621	10937	2663	3777	2797	4169
ENSG00000201129	0	1	0	0	0	3	0	0	0	1	5	0	0	2	0	0	0	0	0	0	1	0	4	1	1	0	1	5	0	2	2	0
ENSG00000143575	2337	1525	1673	1520	1003	1066	701	1121	1189	825	3273	671	1094	1000	995	1261	2084	3309	2551	2045	2026	759	1610	652	1028	1502	719	2206	1005	958	1164	1133
ENSG00000252682	0	0	0	0	0	0	0	0	0	0	2	0	0	0	0	0	0	0	0	0	0	0	0	0	0	0	2	0	0	0	0	0
ENSG00000212292	0	0	0	0	0	0	0	0	0	0	0	0	0	0	0	0	0	0	0	0	0	0	0	0	0	0	0	0	0	0	0	0
ENSG00000222457	0	0	0	0	0	0	0	0	0	0	0	0	0	0	0	0	0	0	0	0	0	0	0	0	0	0	0	0	0	0	0	0
ENSG00000278694	0	0	0	0	0	0	0	0	0	0	0	0	0	0	0	0	0	0	0	0	0	0	0	0	0	0	0	0	0	0	0	0
ENSG00000143595	0	0	1	1	2	0	2	0	0	0	0	0	0	0	0	0	0	0	0	0	1	2	2	1	0	0	0	0	0	2	0	0
ENSG00000143515	2415	1871	2200	1906	1820	1932	1358	1928	567	543	2636	431	171	199	146	285	477	796	691	1503	2848	1276	1845	1106	436	883	825	1835	44	140	64	153
ENSG00000238365	11	19	13	14	8	6	4	5	4	1	15	0	1	0	2	4	2	5	1	6	10	4	4	10	0	2	2	5	0	2	0	2
ENSG00000237920	0	0	0	0	0	0	0	0	0	0	0	0	0	0	0	0	0	0	0	0	0	0	0	0	0	0	0	0	0	0	0	0
ENSG00000226855	0	0	0	0	0	0	0	0	1	1	1	0	0	0	1	0	0	0	0	0	0	1	0	0	0	0	0	0	2	0	0	0
ENSG00000228013	0	0	0	0	0	2	1	0	0	0	1	0	0	0	0	0	0	1	0	0	6	2	0	3	0	0	0	0	0	0	0	0
ENSG00000160712	2	8	3	4	166	206	119	198	0	2	6	1	0	7	8	14	0	2	3	4	230	150	181	102	0	0	0	4	1	3	2	8
ENSG00000228264	0	0	0	0	1	1	0	1	0	0	0	0	0	0	0	0	0	0	0	0	1	2	0	0	0	0	0	0	0	0	0	1
ENSG00000169291	0	0	0	0	1	5	0	4	0	0	2	0	0	0	0	0	0	0	0	0	3	1	3	4	0	0	1	1	0	0	0	0
ENSG00000273110	0	0	0	0	0	0	0	0	0	0	0	0	0	0	0	0	0	0	0	0	0	0	0	0	0	0	0	0	0	0	0	0
ENSG00000163239	0	0	0	1	0	0	0	0	0	0	0	0	0	0	0	0	0	0	0	0	0	0	1	0	0	0	0	0	0	0	0	0
ENSG00000160714	1819	1412	1602	1385	1828	1908	1444	2022	1211	1047	5076	828	1138	1114	1359	1521	752	1215	1239	1491	3000	1288	2152	1230	1079	1789	1164	3106	1158	1380	1220	1625
ENSG00000229780	12	8	6	4	15	11	6	5	3	4	21	6	9	3	7	5	3	2	4	9	20	8	4	10	14	6	3	16	1	6	6	10
ENSG00000160716	35	40	42	44	24	43	19	43	37	25	82	7	28	40	31	37	11	6	5	42	56	41	40	34	33	48	28	104	8	35	29	32
ENSG00000233875	0	0	0	0	0	0	0	0	0	0	0	0	0	0	0	0	0	0	0	0	0	0	0	0	0	0	0	0	0	0	0	0
ENSG00000160710	11016	7955	9659	8073	8343	8519	6033	8838	6664	5532	26789	4600	7643	6816	6978	8328	2445	3749	3321	6314	11508	5124	7975	4315	5995	10683	5937	18914	4676	6530	4455	6992
[illegible]	[illegible]	[illegible]	[illegible]	[illegible]	[illegible]	[illegible]	[illegible]	[illegible]	[illegible]	[illegible]	[illegible]	[illegible]	[illegible]	[illegible]	[illegible]	[illegible]	[illegible]	[illegible]	[illegible]	[illegible]	[illegible]	[illegible]	[illegible]	[illegible]	[illegible]	[illegible]	[illegible]	[illegible]	[illegible]	[illegible]	[illegible]	[illegible]

ENSG00000273933	0	1	0	0	0	0	1	0	0	0	1	0	1	0	0	0	0	0	0	0	0	1	0	0	0	0	0	0	0	0	0	0
ENSG00000177807	73	70	79	71	13	23	15	16	9	8	41	9	11	4	10	11	7	16	5	29	15	13	16	8	3	5	1	18	4	16	12	16
ENSG00000237409	3	0	6	2	1	5	0	5	0	1	1	2	1	1	1	2	0	0	0	2	2	2	2	2	0	0	1	3	0	2	1	1
ENSG00000260766	124	103	114	126	135	141	110	168	131	115	551	93	88	79	83	150	26	24	28	91	219	90	112	86	116	167	116	282	31	95	57	108
ENSG00000143315	788	662	821	718	650	687	520	690	734	606	2852	483	465	437	521	632	349	478	366	729	1077	394	738	434	596	842	565	1591	338	524	393	639
ENSG00000225279	5	5	9	7	0	0	0	2	0	0	2	3	0	4	0	0	0	2	3	4	1	1	3	2	0	2	0	3	1	0	1	0
ENSG00000162728	18	17	16	12	2	2	6	1	11	6	15	0	3	3	4	0	4	1	7	20	8	4	5	6	8	7	5	32	1	6	2	8
ENSG00000162729	1565	1237	1200	1278	843	916	632	957	956	910	3156	689	726	661	659	837	392	701	562	791	1297	525	907	370	792	1341	750	2109	544	610	641	712
ENSG00000018625	0	3	0	0	0	1	0	1	0	0	2	0	0	0	0	2	0	0	0	0	1	0	0	2	0	0	0	0	0	2	1	0
ENSG00000132681	0	0	0	1	1	1	0	1	0	0	5	0	50	27	47	43	0	0	0	0	1	1	2	0	0	0	1	4	44	50	24	55
ENSG00000143318	2	3	4	3	4	3	0	1	1	2	9	1	76	58	56	90	2	0	0	7	2	1	1	1	1	1	0	9	37	77	42	117
ENSG00000227741	34	21	29	33	23	17	11	15	16	11	59	12	25	15	27	26	7	7	8	22	18	16	12	17	10	24	14	34	5	26	13	31
ENSG00000162734	1601	1120	1424	1229	1242	1265	934	1337	1008	753	3893	704	797	718	886	975	712	993	1007	1283	2012	793	1517	689	904	1704	928	3151	663	654	690	784
ENSG00000132716	3275	2398	2587	2421	2528	2710	1842	2781	1805	1510	8020	1094	1485	1374	1409	1774	867	771	639	2006	3852	1793	2120	1892	1289	2260	2063	4382	755	1379	935	1619
ENSG00000258465	2	3	5	2	1	2	3	0	5	6	23	3	1	4	1	4	0	0	0	0	1	1	1	1	2	3	8	11	5	5	6	7
ENSG00000228606	32	33	28	25	15	23	21	32	26	13	96	11	11	12	16	23	21	9	5	27	39	26	24	17	14	40	31	63	8	13	20	21
ENSG00000224261	7	7	2	1	3	1	2	4	6	2	25	1	8	2	7	1	1	1	1	5	0	2	3	2	2	5	8	16	0	4	4	4
ENSG00000162735	1759	1353	1495	1322	1068	1177	841	1279	1026	901	4077	680	1266	1033	1216	1243	1000	1431	1306	1082	1899	735	1380	633	1006	1465	916	2645	990	1091	1196	1287
ENSG00000122218	5885	4451	5791	4641	4810	5091	3921	5569	3676	3078	13782	2365	4782	4302	5031	5523	1562	2514	2829	3551	7665	3223	6735	2988	3139	5022	3290	8887	3444	4065	3957	4530
ENSG00000235082	8	5	11	5	9	15	9	2	7	6	25	1	6	9	6	5	2	1	5	14	10	7	7	8	1	9	7	15	3	2	1	8
ENSG00000202078	5	1	2	2	1	5	2	4	5	0	9	1	2	1	5	0	0	0	0	2	4	1	2	2	6	3	6	12	2	1	0	0
ENSG00000162736	1313	896	1146	968	1442	1525	1078	1580	1099	969	4929	855	976	810	910	1086	334	408	383	752	2103	954	1476	803	913	1576	1114	2776	522	722	546	833
ENSG00000171786	27	27	42	23	8	15	6	9	4	6	23	8	6	2	3	7	12	10	20	64	17	1	2	6	3	5	4	12	2	8	1	8
ENSG00000201608	0	0	0	0	0	0	0	0	0	0	0	0	0	0	0	0	0	0	0	0	0	0	0	0	0	0	0	0	0	0	0	0
ENSG00000162738	552	505	571	513	597	673	461	618	0	0	61	1	0	5	1	4	124	146	107	303	648	263	352	250	0	0	7	16	0	1	2	4
ENSG00000162739	645	416	543	326	39	49	41	44	350	241	1051	148	317	275	309	380	602	720	716	445	88	25	75	26	173	164	82	242	235	211	253	282
ENSG00000234425	0	5	3	1	0	0	0	0	0	0	0	0	0	0	0	0	3	1	2	5	0	0	0	1	0	0	0	0	0	0	0	0
ENSG00000066294	2523	1785	2066	1571	125	165	94	185	2	5	19	0	24	11	27	12	1008	1236	1116	1645	164	54	156	51	10	9	8	18	6	26	29	15
ENSG00000117090	5	2	7	2	1	0	1	1	664	556	3174	539	7	13	31	31	4	7	6	6	0	0	0	0	598	1064	978	2632	3	16	14	24
ENSG00000235101	1	1	3	1	1	0	0	2	11	19	57	7	1	0	0	2	2	1	2	1	0	0	2	0	14	18	13	26	1	4	0	4
ENSG00000228863	19	6	24	12	9	13	5	3	76	54	327	42	17	21	17	20	3	2	9	4	7	7	7	1	91	128	90	314	5	21	21	26
ENSG00000117091	887	425	655	490	405	520	333	552	5283	4327	19784	3689	1992	1634	2129	2271	798	656	825	375	798	343	573	421	4691	7004	4821	12166	2256	1848	3021	2606
ENSG00000026751	22	16	16	21	0	0	0	2	3392	2808	20487	2676	101	78	136	113	5	4	1	2	0	0	0	0	2798	4176	4114	8603	50	60	99	92
ENSG00000275801	0	0	0	0	0	0	0	0	0	0	1	0	0	0	0	0	0	0	0	0	0	0	0	0	0	0	1	0	0	0	0	0
ENSG00000274562	0	0	0	0	0	0	0	0	0	0	0	0	0	0	0	0	0	0	0	0	0	0	0	0	0	0	0	1	0	0	0	0
ENSG00000122224	233	113	256	100	111	96	92	130	21	18	91	18	55	46	60	69	81	81	114	87	136	66	76	68	8	26	24	45	23	32	51	51
ENSG00000122223	85	29	88	22	2373	2384	2089	2564	820	740	3628	661	203	183	166	237	91	51	134	59	5517	2112	4501	1974	559	879	435	1780	162	137	112	142
ENSG00000226003	1	1	1	1	9	3	10	18	8	4	16	1	0	0	0	2	0	0	0	0	17	14	9	9	1	3	5	28	1	2	1	3
ENSG00000179914	2	0	0	0	162	124	78	179	0	2	2	1	2	0	1	0	0	1	1	0	305	103	226	95	0	1	1	2	0	0	0	0
ENSG00000213080	15	10	10	10	4	5	4	8	2	3	5	1	6	5	4	7	5	7	4	11	6	3	3	0	1	0	0	6	1	2	2	1
ENSG00000232188	0	0	0	0	0	0	0	0	0	0	0	0	0	0	0	0	0	0	0	0	0	0	0	0	0	0	0	1	0	0	0	0
ENSG00000198358	0	0	0	0	0	0	1	1	0	2	9	1	0	0	0	0	0	0	0	0	0	0	0	0	1	0	1	3	0	0	0	0
ENSG00000233691	0	0	0	0	0	0	0	0	0	1	1	0	0	0	0	0	0	0	0	0	0	0	0	0	0	0	0	1	0	0	0	0
ENSG00000158764	0	1	0	0	2	0	0	0	0	0	16	3	0	1	0	0	0	0	0	0	2	1	2	3	1	1	5	4	1	1	0	1
ENSG00000158769	736	550	658	568	632	638	490	725	523	535	2279	416	1112	1073	1068	1428	171	238	233	365	666	250	437	264	360	519	396	1128	678	932	791	992
ENSG00000270149	81	61	90	76	1	7	4	2	25	37	81	18	14	17	14	16	16	28	22	46	5	5	1	1	19	26	22	38	5	11	7	19
ENSG00000232879	18	15	20	23	0	0	0	2	14	12	50	11	9	8	5	3	0	3	3	13	0	0	2	0	10	8	16	17	1	5	7	4
ENSG00000215845	562	376	505	373	124	162	114	190	587	448	1537	338	234	215	209	293	465	502	492	391	282	117	218	133	453	537	310	931	147	160	197	207
ENSG00000158773	1782	1130	1397	1181	1285	1384	957	1359	965	737	4133	668	539	488	628	714	937	1134	1172	1108	2352	932	1835	876	904	1451	930	2464	612	663	651	788
ENSG00000186517	6274	3521	5622	3988	6205	6480	4801	6698	9248	7174	35363	6040	5431	4998	5503	6308	2275	2547	3273	4064	8669	3711	6789	3319	7529	12524	8621	23297	3464	4178	3774	4654
ENSG00000278883	1	2	4	0	1	0	3	1	3	1	29	1	1	5	3	1	1	0	1	3	7	2	2	0	4	2	9	21	0	1	3	0
ENSG00000143217	4	2	0	1	4	1	1	7	7	6	1980	92	0	0	0	0	0	0	2	1	2	0	2	3	4	12	294	556	0	2	3	3
ENSG00000228917	2	0	1	0	0	0	0	0	0	0	0	0	0	0	0	0	0	0	0	1	0	0	3	0	1	0	0	0	0	0	0	0
ENSG00000162755	7	5	6	6	0	2	0	2	8	2	34	6	6	9	8	15	5	4	2	5	2	4	2	6	4	11	11	18	6	5	7	11
ENSG00000143256	2759	1710	2090	2218	1371	1481	1183	1503	1033	664	3413	593	1568	1373	1839	1792	3317	4162	3605	2919	3191	1281	2396	1299	989	1690	602	2097	1779	1588	1981	1901
ENSG00000158793	788	603	684	574	451	445	323	500	335	293	1222	261	367	341	400	475	485	562	547	478	575	275	531	250	261	439	301	811	251	256	296	325
ENSG00000158796	958	681	871	696	831	841	626	878	604	473	2011	342	405	387	456	540	379	460	472	671	1324	561	961	537	456	792	485	1314	307	410	334	457
ENSG00000238934	2	2	0	0	0	3	1	6	1	0	7	2	1	2	2	1	0	2	2	3	6	2	1	0	0	0	2	5	1	2	0	2
ENSG00000143222	1971	1311	1545	1236	1575	1585	1158	1657	1450	1049	4796	954	1076	1028	1140	1371	2328	2973	2419	2005	4199	1504	3064	1620	1302	1897	1076	2838	1432	1226	1638	1644
ENSG00000224985	4	3	3	4	0	1	1	2	0	0	4	2	1	2	0	1	1	0	0	6	8	0	1	4	0	3	0	4	0	4	1	0
ENSG00000143258	1161	894	1078	983	830	845	585	845	693	523	2234	404	515	446	488	656	437	437	496	786	1046	485	752	458	493	770	476	1366	366	462	355	516
ENSG00000143224	266	206	248	229	371	401	310	448	441	348	1533	294	263	228	243	332	113	118	114	184	553	265	385	231	272	514	326	781	144	207	166	252
ENSG00000158850	704	496	635	619	876	879	628	962	536	427	1883	363	702	649	695	838	278	530	425	532	1380	596	1023	492	384	712	482	1076	582	737	625	785
ENSG00000158859	11	13	12	15	5	5	8	3	3	4	16	1	33	26	30	30	1	1	2	7	7	4	3	3	1	0	4	5	10	13	13	16
ENSG00000158864	2279	1714	1752	1779	1846	1901	1326	1980	1323	1046	4368	802	1846	1633	1709	2212	1631	2548	2150	1571	3117	1250	2603	1041	1141	1766	1097	2728	1413	1618	1607	1903
ENSG00000158869	8	10	14	13	131	123	113	139	1	2	15	2	9	10	11	16	5	13	7	18	480	201	359	211	4	8	10	7	1	12	7	15
ENSG00000158874	0	0	0	0	1	2	3	5	0	0	0	0	0	0	0	0	0	0	0	1	0	1	3	2	0	0	0	1	0	0	0	0
ENSG00000158882	495	345	474	379	305	305	238	307	299	221	1158	209	225	202	291	277	344	485	479	516	574	267	614	248	309	482	270	783	280	338	339	333
ENSG00000143257	22	13	14	8	12	17	11	17	16	8	57	6	31	23	24	37	4	8	6	13	33	21	15	24	17	18	16	44	11	25	23	41
ENSG00000248485	14	5	15	13	3	0	2	1	11	8	27	1	20	14	13	19	0	0	0	2	0	0	0	0	0	7	7	12	0	6	0	10
ENSG00000158887	109	72	136	101	166	175	121	174	200	130	663	101	278	241	266	319	72	49	96	88	246	103	162	86	141	228	216	433	159	219	251	321
ENSG00000143252	3639	2303	2926	2550	2229	2268	1510	2337	2119	1667	7871	1317	2124	2038	2173	2567	2303	3398	3148	2683	4257	1714	3081	1445	1995	2748	1964	4744	1989	2138	2068	2428
ENSG00000188931	0	0	0	0	0	0	0	0	0	0	0	0	0	0	0	0	0	0	0	0	0	0	0	0	0	0	0	0	0	0	0	0
ENSG00000236484	2	0	1	1	2	0	2	2	0	0	6	1	0	1	0	2	0	0	0	1	4	0	2	1	1	0	0	5	1	2	2	0
ENSG00000283696	26	15	30	17	15	27	18	28	21	17	87	16	15	14	8	19	7	3	7	19	28	14	25	19	12	27	25	51	4	9	2	15
ENSG00000206921	6	4	5	5	4	2	3	6	7	2	23	5	4	2	2	1	2	3	1	4	11	3	6	2	2	1	3	15	0	8	1	4
ENSG00000283360	26	21	22	14	10	14	13	17	14	9	42	3	5	6	1	3	0	3	0	22	19	5	6	18	4	13	12	44	0	6	5	7
ENSG00000215840	7	8	12	11	7	4	5	6	5	4	35	1	4	2	5	1	2	0	1	16	3	7	6	3	4	2	9	21	2	3	0	3
ENSG00000277882	3	2	3	2	2	5	0	1	3	0	14	2	0	0	4	1	1	0	0	4	2	3	1	4	0	4	1	9	2	2	1	2
ENSG00000283317	34	52	37	45	3	6	3	0	8	7	36	8	7	11	6	18	13	21	18	39	3	1	1	1	0	3	7	16	9	11	2	9
ENSG00000143226	2	5	1	3	180	192	168	204	0	1	0	0	12	4	4	4	0	2	0	0	277	133	221	102	1	3	0	0	0	0	2	1
ENSG00000273112	0	0	0	0	3	0	0	2	0	0	0	0	0	0	0	0	0	0	0	0	0	0	1	1	0	0	0	0	0	0	0	0
ENSG00000173110	80	13	23	24	28	32	11	33	0	0	3	0	12	10	12	24	3	7	14	14	16	11	19	11	0	0	0	3	10	8	4	5
ENSG00000224203	0	0	0	0	0	0	0	0	0	0	0	0	0	0	0	0	0	0	0	0	0	0	0	0	0	0	0	0	0	0	0	0
ENSG00000203747	1	0	0	0	0	0	1	0	0	0	0	0	0	0	0	0	0	0	0	0	0	0	0	0	0	0	0	0	0	0	2	1
ENSG00000224515	0	0	0	0	0	0	0	0	0	0	0	0	0	0	0	0	0	0	0	0	0	0	0	0	0	0	0	0	0	0	0	0
ENSG00000244682	0	0	0	0	0	1	0	0	0	0	0	0	0	0	0	0	0	0	0	0	2	0	1	0	0	0	0	0	0	0	0	0
ENSG00000225217	0	0	0	2	1	1	1	0	0	0	0	0	0	1	2	0	0	0	0	2	0	0	1	0	0	1	0	0	0	0	0	0
ENSG00000231586	0	0	0	0	0	0	0	0	0	0	0	0	0	0	0	0	0	0	0	0	0	0	0	0	0	0	0	0	0	0	0	0
ENSG00000162747	0	0	0	0	0	0	0	0	0	0	0	0	0	0	0	0	0	0	0	0	0	0	0	0	0	0	0	0	0	0	0	0
ENSG00000072694	0	0	0	1	0	0	0	0	0	0	1	0	0	1	0	0	0	0	0	0	1	0	0	0	0	0	0	0	0	0	0	0
ENSG00000234211	0	0	0	0	0	0	0	0	0	0	0	2	0	0	0	0	0	0	0	0	0	0	0	0	0	0	0	1	0	0	0	0
ENSG00000213075	0	0	0	0	0	0	0	0	0	0	0	0	0	0	0	0	0	0	0	0	0	0	0	0	0	0	0	1	0	0	0	0
ENSG00000199595	0	0	0	0	0	0	0	0	0	0	0	0	0	0	0	0	0	0	0	0	0	0	0	0	0	0	0	0	0	0	0	0
ENSG00000132185	0	0	0	0	2	0	0	0	0	0	0	0	0	1	0	1	0	0	0	0	0	0	0	0	1	0	0	0	0	0	0	0
ENSG00000162746	3	1	0	2	31	13	24	41	20	15	91	15	28	29	26	28	2	2	0	3	47	22	53	28	20	25	22	60	25	21	25	23
ENSG00000241347	0	0	0	0	0	0	0	0	0	0	0	0	0	0	0	0	0	0	0	0	0	0	0	0	0	0	0	0	0	0	0	0
ENSG00000081721	614	458	493	428	768	772	626	784	422	332	1403	267	482	458	576	555	640	1020	841	738	2116	772	1509	812	468	689	286	888	722	601	915	755
ENSG00000226889	3	3	7	1	9	9	2	5	11	6	12	5	5	5	8	7	2	2	4	4	7	9	8	2	3	1	1	3	9	4	2	6
ENSG00000118217	2492	1612	2023	1626	4860	4735	3668	5145	1486	1181	5759	973	1675	1481	1606	1814	1654	2072	2085	1791	10236	3931	7674	3688	1351	1831	1283	3626	1345	1435	1847	1720
ENSG00000229808	29	10	26	16	26	34	23	44	6	14	30	2	5	6	1	1	4	6	7	9	57	36	38	24	12	19	3	33	5	8	2	10
ENSG00000162745	0	2	3	3	1673	1810	1145	2010	0	0	1	0	0	0	0	0	0	1	1	1	1572	693	1236	605	0	0	2	1	1	0	0	0
ENSG00000227818	0	0	0	0	0	0	0	0	0	0	0	0	0	0	0	0	0	0	0	0	0	0	0	0	0	0	0	0	0	0	0	0
ENSG00000198929	3	29	1	10	61	63	37	43	22	62	350	48	2	0	0	3	0	2	1	5	66	25	28	20	11	47	33	139	0	7	0	0
ENSG00000266144	0	0	0	1	0	0	0	0	0	0	0	0	0	0	0	0	0	0	0	0	0	0	0	0	0	0	0	2	0	0	0	0
ENSG00000227094	0	1	0	0	1	0	1	0	0	1	0	1	0	0	0	0	0	0	0	0	1	1	0	0	0	0	0	3	0	0	0	0
ENSG00000207729	0	0	0	0	0	0	0	0	0	0	1	1	0	0	0	0	0	0	0	0	1	0	0	0	0	0	0	0	0	0	0	0
ENSG00000254706	0	1	0	0	4	2	2	2	0	3	18	4	0	0	0	0	0	0	0	0	4	0	0	0	1	5	0	7	0	0	0	0
ENSG00000239887	1	12	5	21	72	105	69	101	72	82	617	119	3	13	1	8	0	4	0	4	88	43	55	32	20	69	76	214	0	2	0	4
ENSG00000171722	0	0	0	1	4	4	5	2	7	1	41	2	0	0	0	0	0	0	0	0	3	5	4	6	0	5	1	3	0	0	0	1
ENSG00000198574	0	0	0	0	2	3	0	3	234	266	1785	220	0	0	0	0	0	0	0	0	0	0	0	3	170	249	358	677	0	0	0	0
ENSG00000227243	0	0	0	0	0	0	0	0	0	0	1	0	0	0	0	0	0	0	0	0	0	0	0	0	0	0	0	0	0	0	0	0
ENSG00000152332	7296	5409	6007	5199	3848	4196	2737	4226	4169	3990	22368	3052	3478	3138	3246	3824	2454	3327	3384	4826	7329	3152	4815	2738	4650	6785	6514	14295	2113	2954	2321	3091
ENSG00000227941	0	0	0	0	1	0	0	0	0	0	2	0	0	0	1	0	0	0	0	2	0	0	1	1	0	1	0	3	0	0	0	0
ENSG00000259788	1	0	1	0	1	0	0	0	0	0	0	2	0	0	0	0	0	1	1	2	0	0	1	0	0	0	0	2	0	2	0	0
ENSG00000117143	4453	3248	3344	3342	1283	1327	917	1363	1262	933	3807	709	1427	1214	1290	1610	4281	6922	4665	4782	2549	854	1729	878	1105	1592	898	2675	1001	1120	1116	1468
ENSG00000272574	41	33	50	26	9	10	2	3	22	25	118	25	12	15	11	16	0	13	3	49	17	4	5	3	12	20	31	48	4	7	9	12
ENSG00000162733	168	136	105	124	18	19	9	12	28	29	107	13	18	24	16	23	71	63	40	181	38	7	20	13	39	65	38	101	7	20	5	10
ENSG00000243173	0	0	1	0	0	0	0	0	1	0	0	0	0	0	0	2	0	0	0	0	0	0	0	0	0	0	0	1	0	0	0	0
ENSG00000132196	1580	958	794	885	769	650	498	682	461	288	1352	180	630	665	471	800	1087	1055	601	685	828	303	430	348	350	451	193	716	282	359	287	460
ENSG00000185860	1	1	0	0	0	0	2	0	1	1	0	0	0	0	0	0	0	0	0	0	0	0	1	0	0	1	0	0	0	0	0	0
ENSG00000230739	0	2	1	0	0	0	1	0	0	0	0	0	0	1	0	0	0	0	0	0	0	0	0	0	0	0	0	0	0	0	0	0
ENSG00000227667	0	0	0	0	0	0	0	0	0	0	2	0	0	0	0	0	0	0	0	0	0	0	0	0	0	0	0	0	0	0	0	0
ENSG00000117152	0	0	0	2	1	1	0	1	0	0	0	1	0	0	0	0	0	5	0	0	3	2	0	1	0	0	0	0	0	0	0	0
ENSG00000143248	45	26	36	23	27	38	28	25	31	29	77	13	32	22	26	35	31	52	27	35	51	27	43	12	30	38	16	81	17	32	27	36
ENSG00000232892	0	1	0	0	2	0	0	0	1	1	3	0	2	0	0	1	0	0	0	0	0	0	1	1	2	1	1	1	0	6	2	1
ENSG00000225755	2	1	2	2	2	0	1	0	1	0	2	3	3	0	0	0	0	0	0	0	0	1	1	2	1	2	4	3	0	5	2	1
ENSG00000232995	85	70	109	63	57	82	66	83	68	72	278	52	64	69	80	96	35	46	49	93	111	69	87	61	55	81	58	203	42	71	77	72
ENSG00000237756	5	6	9	1	1	1	8	2	0	7	22	1	5	5	6	9	2	1	0	7	7	8	7	7	4	5	8	11	1	5	2	8
ENSG00000143228	2672	1750	1974	1772	1421	1611	1172	1711	1536	1194	4335	919	1206	1112	1325	1475	2132	2376	2040	2389	3193	1347	2145	1780	1593	2336	492	3508	1113	1379	1468	1677
ENSG00000225122	0	0	0	0	0	0	0	0	0	0	0	0	0	0	0	0	0	0	0	0	0	0	0	0	0	0	0	0	1	0	0	0
ENSG00000228289	0	0	0	0	0	0	0	0	0	0	0	0	0	0	0	0	0	0	0	0	0	0	0	0	0	0	0	0	0	0	0	0
ENSG00000212538	0	0	0	0	0	0	0	0	0	0	0	0	0	0	0	0	0	0	0	0	0	0	0	0	0	0	0	0	0	0	0	0
ENSG00000225719	0	0	0	0	1	0	0	0	0	0	0	0	0	0	0	0	0	0	0	0	3	1	0	0	0	0	0	0	0	0	0	0
ENSG00000199849	0	0	0	0	1	1	0	0	0	0	1	0	0	0	0	0	0	0	0	0	0	0	0	3	0	0	0	0	0	0	0	0
ENSG00000213070	0	0	0	0	2	1	0	4	1	0	0	0	0	0	0	0	0	1	0	0	2	0	0	0	0	0	2	1	0	0	0	0
ENSG00000185630	1	8	2	10	226	248	185	288	17	23	63	16	0	1	1	2	0	2	0	3	290	146	209	110	14	23	19	46	0	0	1	3
ENSG00000207082	0	1	0	0	0	1	0	0	1	0	0	0	0	0	0	0	0	0	0	0	0	0	0	0	0	0	0	0	0	0	0	0
ENSG00000269887	0	0	0	0	2	4	0	1	0	2	2	0	0	0	0	0	0	0	0	0	2	1	0	0	0	0	0	0	0	0	0	0
ENSG00000276359	0	0	0	0	0	0	0	0	0	0	0	0	0	0	0	0	0	0	0	0	0	1	0	0	0	0	0	0	0	0	0	0
ENSG00000233693	0	0	0	0	0	0	0	0	0	0	0	0	0	0	0	0	0	0	0	0	1	0	0	1	0	0	1	0	0	0	0	0
ENSG00000271917	0	0	0	0	0	0	0	0	0	0	0	0	0	0	0	0	0	0	0	0	1	0	2	0	0	0	0	0	0	0	0	0
ENSG00000201987	0	0	0	0	0	0	0	0	0	0	0	0	0	0	0	0	0	0	0	0	0	0	0	0	0	0	0	0	0	0	0	0
ENSG00000225487	0	0	0	0	0	0	0	0	0	0	0	0	0	0	0	0	0	0	0	0	0	0	0	0	0	0	0	0	0	0	0	0
ENSG00000201270	0	0	0	0	0	0	0	0	0	0	0	0	0	0	0	0	0	0	0	0	0	0	0	0	0	0	0	0	0	0	0	0
ENSG00000162761	0	0	0	0	0	0	0	0	0	0	0	0	0	0	0	0	0	0	0	0	0	0	0	0	0	0	0	0	0	0	0	0
ENSG00000224702	0	0	0	0	0	0	0	0	0	0	0	0	0	0	0	0	0	0	0	0	0	0	0	0	0	0	0	0	0	0	0	0
ENSG00000238022	0	0	0	0	0	0	0	0	0	0	0	0	0	0	0	0	0	0	0	0	0	0	0	0	0	0	0	0	0	0	0	0
ENSG00000143171	0	0	0	0	0	0	0	0	0	0	0	0	0	0	0	0	0	0	0	0	0	0	0	0	0	0	0	0	0	0	0	1
ENSG00000237463	0	0	0	0	0	0	0	0	0	0	0	0	0	0	0	0	0	0	0	0	0	0	0	0	0	0	0	0	0	0	0	0
ENSG00000230748	0	0	0	0	0	0	0	0	0	0	0	0	0	0	0	0	0	0	0	0	0	0	0	0	0	0	0	0	0	0	0	0
ENSG00000162763	0	0	0	0	0	0	0	0	0	0	0	0	0	0	0	0	0	0	0	0	0	0	0	0	0	0	0	0	0	0	0	0
ENSG00000237783	0	0	0	0	0	0	0	0	0	0	0	0	0	0	0	0	0	0	0	0	0	0	0	0	0	0	0	0	0	0	0	0
ENSG00000236206	0	2	3	1	1	0	0	0	0	1	1	0	0	0	1	0	1	1	1	2	1	0	0	1	0	0	0	0	1	0	0	0
ENSG00000143198	767	602	606	614	615	721	394	678	471	371	1322	290	322	410	303	530	686	1089	949	1099	1304	579	879	533	416	591	290	956	302	442	416	498
ENSG00000143149	1464	997	1155	1037	1685	1650	1258	1778	1006	869	3462	560	1022	936	1061	1213	1005	1431	1276	1260	3554	1435	2566	1251	1028	1500	769	2337	1011	1127	1124	1307
ENSG00000206990	0	0	0	0	0	0	0	0	0	0	0	0	0	0	0	0	0	0	0	0	0	0	0	0	0	0	0	0	0	0	0	0
ENSG00000230175	1	2	3	1	2	5	0	1	0	3	8	2	1	1	2	0	1	0	0	0	2	0	1	5	4	7	1	10	0	1	3	0
ENSG00000225272	2	2	0	3	5	1	2	4	6	0	7	1	2	7	1	1	1	0	0	1	4	1	4	3	3	1	3	5	0	0	2	8
ENSG00000215838	3	9	3	5	4	5	2	5	6	5	9	1	9	11	5	8	3	3	2	11	8	2	6	4	1	3	10	7	1	3	0	5
ENSG00000273365	5	3	1	0	4	0	2	3	1	2	1	0	6	1	1	3	2	1	3	0	5	0	1	0	1	1	2	2	0	2	0	2
ENSG00000143183	1672	1119	1341	968	1515	1576	1259	1737	1231	1005	4443	737	966	809	1058	1133	2749	2940	2916	2295	5051	1824	3417	2263	1363	1817	1183	3033	1466	1385	2202	1842
ENSG00000224358	34	30	41	31	12	16	4	14	18	15	61	9	21	23	13	19	18	19	12	25	14	5	9	10	8	12	10	27	11	13	12	17
ENSG00000230659	0	0	0	0	0	0	0	0	0	0	0	0	0	0	0	1	0	0	0	2	0	0	0	0	0	0	0	0	0	0	0	0
ENSG00000143179	2372	1914	2137	2086	2471	2583	1763	2676	2437	1743	7418	1504	3196	2800	2860	3468	1416	2416	1923	2712	4443	1855	3190	1631	2266	3412	1492	5118	2171	2714	2029	2729
ENSG00000236364	21	24	34	25	24	27	32	33	30	25	148	14	27	25	26	39	12	19	3	38	53	26	27	38	31	54	31	89	14	31	22	37
ENSG00000271527	0	0	0	0	0	0	0	0	0	0	1	0	0	1	1	0	2	0	0	0	0	0	0	0	0	0	0	2	0	3	0	0
ENSG00000226292	0	0	0	0	0	0	0	0	0	0	0	0	0	1	0	0	0	0	0	0	0	0	0	0	0	0	0	0	0	0	0	0
ENSG00000188859	138	172	180	193	0	0	0	0	8	9	39	9	149	210	159	291	34	144	87	211	0	0	0	0	8	11	30	43	95	192	124	170
ENSG00000203307	14	6	16	5	0	0	0	0	0	2	4	0	4	16	12	7	9	5	16	13	0	0	0	2	1	2	0	8	4	6	11	13
ENSG00000230898	5	7	16	5	0	0	0	0	0	0	2	0	2	1	5	6	2	0	2	10	0	0	0	0	0	2	0	2	2	4	6	5
ENSG00000215952	2	2	6	2	0	0	0	0	0	0	2	0	0	3	3	2	0	0	0	6	0	0	0	0	0	0	0	0	0	1	1	1
ENSG00000215835	5	1	3	4	4	4	2	7	4	3	12	1	5	5	8	10	0	0	1	1	8	1	3	2	1	7	5	8	6	5	2	2
ENSG00000229588	1	0	0	1	0	0	0	0	0	0	0	0	1	4	2	2	0	0	0	1	0	0	0	0	0	0	0	0	2	1	4	4
ENSG00000225325	0	0	0	0	0	0	0	0	0	0	3	0	0	0	0	0	0	0	0	0	0	0	1	0	0	0	3	0	0	0	1	0
ENSG00000230231	1	1	1	0	2	0	0	0	14	10	29	6	3	8	1	10	0	1	0	1	1	0	0	0	6	4	5	21	0	14	5	14
ENSG00000234142	3	2	0	1	0	1	0	0	12	7	30	3	17	4	12	20	1	1	1	1	0	3	0	0	1	6	9	10	8	12	5	13
ENSG00000238087	0	0	0	0	0	0	0	0	0	0	2	0	0	0	0	0	0	0	0	0	0	0	0	0	0	0	0	0	0	0	0	0
ENSG00000215834	0	0	0	0	0	0	0	1	0	0	0	0	0	0	0	0	0	0	0	0	0	0	0	0	0	0	0	0	0	0	0	0
ENSG00000234984	0	0	0	0	0	0	0	0	0	0	0	0	0	0	0	0	0	1	0	0	0	0	0	0	0	0	0	0	0	0	0	0
ENSG00000232341	1	0	0	0	0	0	0	0	0	0	0	0	0	0	0	0	0	0	0	0	0	0	0	0	0	0	0	0	0	0	0	0
ENSG00000232148	0	0	0	0	0	0	0	0	0	0	1	0	0	0	0	0	0	0	0	0	0	0	0	0	2	0	0	0	0	0	0	0
ENSG00000232223	0	0	0	0	0	0	0	0	0	0	3	0	0	0	0	2	0	0	0	0	0	0	0	0	0	0	1	2	0	0	0	0
ENSG00000143157	2673	1988	2424	2029	3467	3526	2569	3640	2774	2397	10686	1934	2347	2220	2547	2916	1069	1478	1536	2374	5857	2424	4381	2226	2658	4411	2580	7153	1806	2506	1777	2586
ENSG00000152382	926	683	756	592	642	705	517	743	722	689	2710	483	606	505	509	654	855	1163	907	892	1546	631	1064	678	765	1000	665	1762	470	512	521	622
ENSG00000225171	12	10	11	15	12	20	14	10	20	20	59	6	6	5	0	3	4	1	3	12	18	13	10	14	7	17	16	40	2	4	2	5
ENSG00000143195	1407	1336	1157	1278	621	902	494	938	1412	1352	4872	840	193	280	153	318	332	608	380	1047	922	448	662	446	1220	2015	1557	4265	195	396	166	374
ENSG00000143194	5	5	1	5	5	10	10	13	25	28	112	18	3	12	7	9	1	5	3	10	8	4	7	10	17	13	44	83	2	2	2	4
ENSG00000227907	2	0	3	3	5	3	6	4	0	0	1	0	1	1	4	2	1	0	0	3	9	6	4	2	1	1	2	4	1	2	0	1
ENSG00000143167	488	218	628	251	917	808	682	743	150	106	562	70	164	222	303	349	117	107	223	210	1119	336	893	266	150	119	368	365	176	317	239	311
ENSG00000198842	14	6	10	9	114	84	92	96	0	0	9	0	8	9	13	17	4	5	3	6	87	22	68	19	3	0	3	10	5	7	13	11
ENSG00000213068	0	0	0	0	0	0	0	0	0	0	0	0	0	0	0	0	0	0	0	0	0	0	0	0	0	0	0	0	0	0	0	0
ENSG00000231605	1	1	0	0	0	0	0	0	1	1	3	0	4	0	0	0	0	1	0	0	0	0	0	0	0	0	0	0	0	0	0	0
ENSG00000272205	29	33	48	45	52	35	36	49	13	11	45	6	35	35	18	40	15	11	22	46	66	31	64	22	14	10	12	27	13	32	29	30
ENSG00000143190	3794	3031	3453	3151	3886	3755	3203	4240	2432	1963	7917	1321	2423	2358	2593	2942	648	930	895	2219	6129	2585	4207	2319	2222	3221	1665	5744	1391	2389	1556	2524
ENSG00000272033	50	41	49	51	20	34	28	39	34	37	124	11	28	16	25	7	11	6	12	49	72	45	31	36	35	35	21	113	5	14	21	34
ENSG00000198821	2275	1739	1865	1641	2197	2182	1466	2455	5426	4162	17929	3458	2154	2037	1959	2372	1214	1687	1327	1534	2475	966	1757	880	4505	6869	4497	12351	1491	1651	1436	1810
ENSG00000233411	4	9	16	11	11	25	28	31	58	41	276	35	15	17	19	16	3	5	4	20	27	15	8	25	46	61	60	211	0	33	14	28
ENSG00000273160	28	9	13	8	19	14	10	26	61	55	241	37	15	14	4	19	1	4	3	18	17	11	22	17	47	73	54	196	5	19	14	15
ENSG00000227776	7	3	0	1	0	0	1	4	9	7	30	3	0	3	0	2	0	0	1	4	4	1	0	1	5	5	12	19	0	0	0	0
ENSG00000143162	965	795	679	697	1118	1175	763	1238	771	608	2107	468	1296	1130	1306	1519	1095	1863	1280	1284	2850	1075	1896	1053	560	829	468	1396	1505	1337	2008	1726
ENSG00000231029	0	0	0	0	0	0	0	0	0	0	0	0	0	0	1	1	0	0	0	0	0	0	0	0	0	0	0	0	0	0	0	0
ENSG00000241666	59	44	72	70	107	114	90	118	36	26	114	19	61	48	52	63	23	21	13	45	217	77	123	89	28	48	24	62	21	59	53	80
ENSG00000198771	4620	3242	3992	3315	1615	1551	966	1748	4026	3030	10577	2231	3481	3196	3377	4014	1418	2112	1661	2547	1581	717	1144	577	3067	4407	1619	6674	1964	2235	2019	2552
ENSG00000197965	3749	2688	3000	2687	3035	2908	2262	2977	1045	859	3452	624	1597	1449	1682	1930	969	1465	1174	2022	4232	1580	2832	1359	1078	1665	774	2499	1212	1456	1148	1529
ENSG00000143199	20	15	11	14	5	3	6	4	2	4	16	0	3	2	3	14	0	7	5	9	2	7	3	7	2	1	1	10	2	3	5	1
ENSG00000250762	1	0	0	0	0	0	0	0	0	0	0	0	0	0	0	0	0	0	1	0	0	0	0	0	0	0	0	1	2	3	0	0
ENSG00000232194	0	1	0	0	3	0	0	0	0	0	2	0	0	0	0	1	0	0	0	0	0	0	2	0	0	0	1	0	0	0	0	0
ENSG00000143158	1914	1367	1485	1450	909	935	643	971	726	529	2149	438	629	639	601	778	1458	1888	1800	1661	1881	654	1483	696	722	1071	578	1685	662	648	778	846
ENSG00000143164	1083	737	987	761	1352	1477	1155	1636	895	719	3518	591	750	722	772	908	573	837	799	942	2410	957	1821	952	901	1316	968	2381	700	919	770	1004
ENSG00000221545	0	0	1	0	0	0	0	0	0	0	1	1	0	0	0	0	0	0	0	0	1	0	0	0	0	2	0	1	0	0	3	0
ENSG00000224837	6	1	2	3	1	2	2	3	1	3	7	0	4	1	1	1	6	9	1	7	3	1	6	0	2	1	0	5	2	1	2	2
ENSG00000143147	167	255	176	266	184	212	130	253	66	63	238	55	15	35	18	41	40	108	78	238	227	85	123	80	53	98	67	205	8	18	10	36
ENSG00000143155	2707	1843	1999	1780	1975	2083	1456	2093	1402	1121	5308	882	2335	1856	2310	2491	2686	3645	2780	2763	4567	1767	3117	1782	1540	2210	1196	3349	2092	2201	2666	2695
ENSG00000237049	1	3	2	2	0	0	0	0	0	0	0	0	4	2	3	4	0	0	0	2	0	0	0	1	1	0	0	5	0	2	2	0
ENSG00000237131	2	0	0	0	0	0	1	0	0	0	0	0	15	6	7	3	1	0	0	0	0	0	0	0	0	0	0	0	0	0	1	2
ENSG00000213064	3938	2772	3275	2688	1679	1609	1277	1845	2146	1751	7928	1143	1892	1978	1909	2320	968	1310	1206	2553	3130	1441	2072	1182	2435	3160	2070	6535	1531	2279	1837	2576
ENSG00000214262	0	0	0	0	0	0	0	0	0	0	0	0	0	0	0	0	0	0	0	0	0	0	0	0	0	0	0	0	0	0	0	0
ENSG00000206880	0	0	0	0	0	1	0	0	0	2	1	0	0	0	0	1	0	0	0	1	1	0	1	1	0	0	2	5	0	0	1	0
ENSG00000143178	20	24	6	20	51	65	45	70	73	49	232	25	84	83	33	78	5	12	7	8	93	26	51	57	47	51	55	121	16	49	34	58
ENSG00000227722	0	1	1	0	0	2	2	0	0	0	1	0	0	0	0	2	0	0	0	0	1	0	0	0	0	2	0	1	0	0	0	0
ENSG00000207974	0	0	0	0	0	0	0	0	0	0	0	0	1	0	0	0	0	0	0	0	0	0	0	0	0	0	0	0	0	0	0	0
ENSG00000228697	0	0	3	2	1	3	0	0	0	0	4	0	0	1	2	1	0	0	0	1	3	1	1	0	0	0	0	3	0	0	0	0
ENSG00000283255	0	3	0	0	0	0	0	0	0	0	1	0	0	0	0	0	0	0	0	0	0	0	0	0	0	0	0	0	0	0	0	0
ENSG00000215833	0	0	0	0	0	0	0	0	0	0	1	0	0	0	1	0	0	0	0	0	0	0	0	0	0	0	0	1	0	0	0	0
ENSG00000143185	0	0	1	0	35	31	22	31	817	541	3131	570	45	15	80	32	5	0	1	2	36	13	31	16	839	1351	733	1977	72	47	183	36
ENSG00000227777	0	0	0	0	0	5	1	2	50	51	271	54	2	4	9	1	2	0	0	0	3	1	3	0	57	100	50	99	1	0	5	4
ENSG00000143184	1	0	2	0	13	22	17	24	2177	1721	10533	1781	427	341	861	495	0	0	1	0	15	16	21	22	2420	3600	3082	6881	1082	776	1984	1090
ENSG00000229757	0	0	0	0	0	1	2	0	295	261	1319	254	35	37	93	67	0	0	0	0	2	0	2	0	205	321	239	425	28	31	51	41
ENSG00000143196	0	0	0	0	0	0	0	0	0	0	1	0	0	0	0	0	0	1	0	0	0	0	0	0	0	0	0	1	0	0	1	0
ENSG00000235736	0	0	0	0	0	0	0	0	0	0	0	0	0	0	0	0	0	0	0	0	0	0	0	0	0	0	0	1	0	1	0	0
ENSG00000225826	0	0	1	0	0	0	0	0	5	0	13	1	0	0	0	0	0	0	0	0	0	0	0	0	7	3	11	4	0	0	1	2
ENSG00000229612	0	0	0	0	0	0	0	0	0	0	0	0	0	0	0	0	0	0	0	1	0	0	0	0	0	0	0	0	0	0	0	0
ENSG00000203601	0	0	0	1	0	0	1	0	0	0	2	0	0	0	0	0	0	0	0	0	0	0	0	0	0	0	0	0	0	0	0	0
ENSG00000213063	0	0	0	0	0	0	0	0	0	0	0	0	0	0	0	0	0	0	0	0	0	0	0	0	0	0	0	0	0	0	0	0
ENSG00000237707	0	0	0	0	0	0	0	1	0	0	0	0	0	0	0	0	0	0	0	0	1	0	0	0	0	0	0	0	0	0	0	0
ENSG00000143153	84	166	86	169	327	328	250	323	128	132	573	83	108	141	119	185	34	142	81	148	749	306	550	324	203	294	390	693	90	150	93	191
ENSG00000143156	604	350	444	362	358	349	260	390	310	252	769	169	260	217	226	271	864	934	1004	758	760	281	632	312	218	342	158	460	241	300	336	364
ENSG00000235575	2	2	0	1	6	0	0	0	0	0	0	0	1	0	0	0	1	0	1	0	1	0	3	2	0	1	0	5	0	0	0	0
ENSG00000117475	936	544	623	563	956	969	650	873	406	355	1751	238	309	362	345	400	873	928	839	779	1871	640	1164	702	392	650	416	1135	393	422	552	562
ENSG00000117477	138	97	123	117	19	30	17	25	89	67	208	55	93	91	144	147	189	265	216	233	49	21	25	18	68	111	48	140	134	147	184	217
ENSG00000117479	828	588	580	555	500	531	345	510	317	251	926	153	538	527	583	706	348	664	380	685	856	345	495	324	302	435	175	597	372	545	439	649
ENSG00000234604	17	13	8	11	11	14	9	9	6	3	23	3	4	7	7	7	4	2	2	8	18	6	11	7	7	8	8	12	2	8	4	9
ENSG00000213062	40	26	39	20	28	25	9	21	18	16	55	10	9	11	8	14	7	19	13	27	37	15	29	10	19	22	10	21	4	9	6	3
ENSG00000198734	3	7	2	6	7	5	2	6	0	1	18	6	0	0	1	3	0	3	0	6	1	1	2	3	2	2	2	9	1	3	1	6
ENSG00000174175	8	7	4	3	24	16	16	20	0	0	0	0	1	1	2	0	2	2	5	8	26	7	16	8	0	0	0	0	0	4	1	2
ENSG00000000460	1536	1034	1313	1008	957	838	815	941	706	543	1799	388	532	530	556	615	961	1469	1206	1462	2163	844	1557	946	717	980	295	1735	524	658	597	761
ENSG00000188404	419	306	431	294	5263	4313	4238	4598	21	12	55	4	83	107	106	160	366	747	713	583	11221	3296	8837	3188	8	10	9	11	192	275	227	400
ENSG00000007908	2	2	2	0	1	0	0	3	0	0	0	0	0	0	0	0	1	0	0	2	0	0	0	0	0	0	0	0	0	0	0	0
ENSG00000230704	0	0	0	0	0	0	0	0	0	0	0	0	0	0	0	0	0	0	0	0	0	0	0	0	0	0	0	0	0	0	0	0
ENSG00000171806	433	248	324	279	189	259	198	263	171	163	558	111	180	200	180	218	410	516	485	485	500	209	396	264	150	244	97	370	167	181	248	223
ENSG00000000457	597	376	495	372	655	673	489	662	477	406	1861	326	396	467	472	579	284	336	319	557	1135	528	718	502	551	668	470	1311	335	540	374	526
ENSG00000239494	34	25	28	28	12	22	13	16	27	25	67	10	12	10	16	16	10	7	7	38	28	11	12	14	9	13	12	40	4	11	9	8
ENSG00000075945	1197	938	923	887	510	558	426	557	915	709	3685	517	624	701	705	903	519	733	635	791	1018	437	789	395	877	1257	847	2182	530	688	695	827
ENSG00000243051	1	1	3	2	1	3	1	0	2	0	6	0	0	4	3	3	0	0	0	4	0	1	0	1	0	2	0	4	0	1	0	3
ENSG00000235378	0	0	0	1	0	0	0	0	0	0	1	0	0	0	0	0	1	0	0	1	0	0	0	0	0	0	0	2	0	5	0	1
ENSG00000232959	0	0	2	0	2	0	1	0	0	0	1	0	0	0	1	0	0	0	0	1	2	0	1	2	0	1	1	5	0	1	1	0
ENSG00000224896	0	0	0	0	0	0	0	0	0	0	0	0	0	0	0	0	0	0	0	0	0	0	0	0	0	0	0	0	0	0	0	0
ENSG00000203740	0	0	0	0	0	0	0	0	0	0	0	0	0	0	2	0	0	0	0	0	0	0	0	0	0	0	0	0	0	1	0	0
ENSG00000283340	0	0	0	0	0	0	0	0	0	0	0	0	0	0	0	0	0	0	0	0	0	0	0	0	0	0	0	0	0	0	0	0
ENSG00000263390	0	0	0	0	0	0	0	0	0	0	0	0	0	0	0	0	0	0	0	0	0	0	0	0	0	0	0	0	0	0	0	0
ENSG00000233985	0	0	0	0	0	0	0	0	0	0	0	0	0	0	0	0	0	0	0	0	0	0	0	0	0	0	0	0	0	0	1	0
ENSG00000238051	0	0	0	0	0	0	0	0	0	0	0	0	0	0	0	0	0	0	0	0	0	0	0	0	0	0	0	0	0	0	0	0

ENSG00000224286	0	0	0	0	0	0	0	0	0	0	0	0	0	0	0	0	0	0	0	0	0	0	0	0	0	0	0	0	0	0	0	0
ENSG00000226967	0	0	0	0	0	0	0	0	0	0	0	0	0	0	0	0	0	0	0	0	0	0	0	0	0	0	0	0	0	0	0	0
ENSG00000231407	18	10	23	15	15	15	11	14	8	8	49	2	12	15	8	9	5	3	4	20	26	16	20	12	6	9	11	18	2	4	6	17
ENSG00000120370	473	295	322	323	301	317	243	383	325	248	1038	208	223	230	276	320	400	407	316	422	789	289	531	329	258	391	234	662	190	237	235	353
ENSG00000225545	0	0	0	0	0	0	0	0	0	1	1	0	0	0	0	0	0	0	0	0	0	0	0	0	0	0	0	0	0	1	0	0
ENSG00000235303	0	0	0	0	0	0	0	0	0	0	0	0	0	0	0	0	0	0	1	0	0	0	0	0	0	0	1	0	0	0	0	0
ENSG00000116132	0	0	1	0	0	0	0	1	0	0	2	0	0	0	0	0	0	1	0	0	1	0	0	0	0	0	0	0	0	0	0	0
ENSG00000271811	0	0	0	0	0	0	0	0	0	0	5	0	0	0	0	0	0	0	0	0	1	0	0	0	0	0	0	1	0	0	0	0
ENSG00000117501	0	0	0	0	0	1	0	1	0	0	0	0	0	0	0	0	0	0	0	0	3	0	0	1	0	0	1	1	0	0	1	1
ENSG00000226552	0	0	0	0	0	0	1	0	0	0	0	0	0	0	0	0	0	0	0	0	0	1	2	0	0	0	0	0	0	0	0	0
ENSG00000007933	0	0	0	0	0	0	0	0	0	0	1	1	0	2	0	1	0	0	0	0	2	0	1	0	0	0	1	0	0	1	0	1
ENSG00000221390	0	0	0	0	0	0	0	0	0	0	0	0	0	0	0	0	0	0	0	0	0	0	0	0	0	0	0	0	0	1	0	0
ENSG00000283598	0	0	0	0	0	0	0	0	0	0	0	0	0	0	0	0	0	0	0	0	0	0	0	0	0	0	0	0	0	0	0	0
ENSG00000117507	0	0	0	0	0	0	0	3	0	0	0	1	0	0	0	0	0	0	0	0	0	0	0	0	0	0	1	0	0	0	0	0
ENSG00000094963	0	0	0	0	1	1	0	0	0	0	6	1	0	0	0	1	0	0	1	0	0	0	0	0	0	0	1	1	0	0	0	0
ENSG00000225243	0	0	0	0	0	0	0	0	0	0	0	0	0	0	0	0	0	0	0	0	0	0	0	0	0	0	0	0	0	0	0	0
ENSG00000231424	0	0	0	0	0	2	0	1	0	0	3	1	0	0	0	0	0	0	0	0	1	2	2	2	0	0	0	0	0	2	0	0
ENSG00000010932	6	9	9	5	1	0	1	1	2	1	5	1	1	1	0	1	6	9	9	4	5	0	6	1	1	1	1	3	1	2	3	1
ENSG00000206692	0	0	0	0	0	0	0	0	0	0	0	0	0	0	0	0	0	0	0	0	0	0	0	0	0	0	0	0	0	0	0	0
ENSG00000230547	0	0	0	0	0	0	0	0	0	0	3	0	0	0	0	0	0	0	0	0	0	0	0	0	0	0	0	0	0	0	0	0
ENSG00000076258	65	31	53	39	47	53	44	65	37	44	218	23	68	66	53	68	45	52	53	33	114	58	95	42	24	28	41	97	40	57	68	61
ENSG00000225704	2	4	3	0	3	3	1	1	3	2	3	1	3	1	2	3	0	3	1	1	9	3	2	1	4	0	0	1	0	1	1	3
ENSG00000268062	0	0	0	0	0	0	0	0	0	0	0	0	0	0	0	0	0	0	0	0	0	0	0	0	0	0	0	0	0	0	0	0
ENSG00000239428	0	0	2	0	0	0	0	0	0	0	0	0	0	0	0	0	0	0	0	0	0	0	0	0	0	0	1	0	0	0	0	0
ENSG00000234333	0	0	0	0	0	0	0	0	0	0	0	0	0	0	0	0	0	0	0	0	0	0	0	0	0	0	0	0	0	0	0	0
ENSG00000117523	7712	5724	7278	5882	11477	11629	9134	12294	10444	8287	39314	5939	10476	9664	11287	11571	2023	2274	2303	4844	17045	7472	12355	6765	8983	14449	9372	26632	5950	8965	7004	9906
ENSG00000283683	4	0	0	0	2	3	0	2	0	2	1	1	0	1	3	2	0	1	0	0	3	2	1	0	0	0	1	4	0	1	0	8
ENSG00000034971	0	0	2	0	0	1	0	0	0	0	0	0	0	0	0	1	0	0	0	0	0	0	0	0	0	0	0	0	0	0	0	0
ENSG00000233328	6	7	2	1	9	2	0	3	7	3	30	2	0	4	3	4	0	4	5	3	7	2	4	1	4	6	4	17	1	2	3	9
ENSG00000230364	0	0	0	1	0	1	0	0	1	0	0	0	0	0	1	1	0	1	0	0	0	0	1	0	0	1	0	1	0	1	0	2
ENSG00000117533	751	450	511	413	569	613	401	615	483	458	2093	339	200	185	158	240	406	494	337	462	968	436	745	478	482	738	559	1296	140	232	179	255
ENSG00000232261	0	0	0	0	0	0	0	0	0	0	0	0	0	0	0	0	0	0	0	0	0	2	0	0	0	0	0	0	0	0	1	0
ENSG00000271459	0	0	0	0	0	0	0	0	0	0	0	0	0	0	0	0	0	0	0	0	0	0	0	0	0	0	0	0	0	0	0	0
ENSG00000236741	0	0	0	0	0	0	0	0	0	0	0	0	0	0	0	0	0	0	0	0	0	0	0	0	0	1	0	0	0	0	0	0
ENSG00000253060	0	0	0	0	0	0	0	0	0	0	0	0	0	0	0	0	0	0	0	0	0	0	0	0	0	0	0	0	0	0	0	0
ENSG00000010165	1175	809	1103	928	1498	1632	1325	1674	1338	985	4206	772	1674	1458	1928	1897	429	686	720	1056	2615	1128	2067	1039	1222	1935	800	2791	1631	1902	1764	2058
ENSG00000213060	3	15	9	11	6	3	3	2	0	0	8	0	4	0	0	3	1	4	5	5	4	0	3	1	2	3	4	5	0	1	0	5
ENSG00000224600	0	0	0	0	0	0	0	1	0	0	0	0	0	2	0	0	0	0	0	1	1	0	0	0	1	0	0	0	0	0	0	0
ENSG00000197959	143	164	109	143	361	325	389	422	68	86	815	79	44	54	39	55	61	101	72	189	1162	564	1018	619	87	146	186	560	33	35	48	53
ENSG00000233540	0	1	1	0	0	3	0	0	2	0	4	0	0	0	0	1	1	2	4	0	6	0	2	1	0	2	1	6	0	1	0	4
ENSG00000230630	2	2	2	0	7	9	11	11	3	2	28	2	3	2	1	2	0	1	1	8	28	17	15	33	7	2	15	27	3	1	1	1
ENSG00000283152	0	0	0	0	0	0	0	0	0	0	0	0	0	0	0	0	0	0	0	0	0	0	0	0	0	0	0	0	0	0	0	0
ENSG00000208024	0	0	0	0	0	1	0	0	0	0	0	0	0	0	0	0	0	0	0	0	2	1	1	0	0	0	0	0	0	0	0	0
ENSG00000206684	0	0	0	0	0	0	0	0	0	1	1	0	0	0	0	0	0	0	0	2	1	0	0	0	0	0	2	4	0	0	0	1

[illegible]

ENSG00000231084	3	3	2	4	4	2	2	5	2	7	29	3	4	1	2	2	3	2	2	3	10	4	7	9	2	4	7	28	4	7	4	2
ENSG00000213051	0	0	0	2	0	0	0	0	0	0	1	1	0	0	1	0	0	0	0	0	0	0	0	1	0	0	2	0	0	0	0	0
ENSG00000201421	0	0	0	1	0	0	0	1	0	0	1	0	0	2	0	0	0	0	0	0	0	0	0	0	0	0	0	2	0	0	0	0
ENSG00000206640	0	0	0	0	0	0	0	0	1	0	1	0	0	0	0	0	0	1	0	1	1	1	2	0	0	0	0	1	0	1	0	1
ENSG00000233583	0	2	1	1	0	0	0	3	2	0	2	2	5	0	0	1	0	0	1	1	0	0	0	0	0	0	1	1	0	0	1	0
ENSG00000252612	2	0	1	4	0	0	0	0	0	0	1	0	0	0	0	0	0	0	0	0	1	0	1	0	0	0	0	1	1	0	1	0
ENSG00000238061	9	3	6	9	2	2	7	3	0	0	18	0	0	1	1	1	0	1	0	7	5	3	1	2	0	1	1	13	0	0	1	1
ENSG00000279838	29	28	28	27	40	36	19	18	170	126	515	66	30	38	44	60	11	16	2	40	68	20	28	26	109	94	122	260	14	37	23	35
ENSG00000116679	5726	4144	4663	3969	5498	5633	3896	5887	11011	8483	37756	6606	4326	3743	4225	4880	4859	4873	3703	4785	11196	4624	6351	4756	10181	13600	11814	26268	3084	3784	3610	4640
ENSG00000273004	31	22	32	17	28	23	23	24	14	7	47	8	15	17	31	16	14	7	10	14	34	18	30	16	15	5	13	25	11	12	8	10
ENSG00000261024	20	10	8	7	3	13	6	7	5	4	17	3	9	8	17	6	2	0	2	7	14	2	6	5	1	3	14	16	2	6	10	7
ENSG00000252407	0	0	0	0	0	0	0	0	0	1	1	0	0	0	0	0	0	0	0	0	0	0	0	0	0	1	0	2	0	0	0	0
ENSG00000270920	0	0	0	0	0	0	0	0	0	1	0	0	0	0	0	0	0	0	0	0	0	0	0	0	0	0	0	0	0	0	0	0
ENSG00000279401	0	0	0	2	0	0	0	0	2	0	7	1	0	0	0	0	0	0	0	0	0	0	0	0	0	0	0	8	0	0	0	0
ENSG00000228309	0	0	0	0	0	0	0	0	2	2	14	1	0	0	0	0	0	0	0	0	1	0	0	0	2	1	0	5	0	0	0	0
ENSG00000207108	0	0	0	0	0	0	0	0	0	0	0	0	0	0	0	0	0	0	0	0	0	0	0	0	0	0	0	0	0	0	0	0
ENSG00000201596	0	0	0	0	0	0	0	0	0	0	3	0	0	0	0	0	0	0	0	0	0	0	0	0	0	0	0	0	0	0	0	0
ENSG00000261729	0	0	0	0	0	0	0	0	5	3	15	0	2	0	1	1	0	0	0	0	0	0	0	0	3	5	3	14	2	0	4	3
ENSG00000143341	1	0	0	0	1	0	0	4	97	91	292	54	6	6	26	11	0	0	1	0	1	0	1	0	51	61	70	129	8	16	14	8
ENSG00000224691	0	0	0	0	0	0	0	0	0	1	1	0	0	0	0	0	0	0	0	0	0	0	0	0	0	0	3	0	0	0	0	0
ENSG00000116690	21	12	20	14	25	29	22	28	10	16	46	6	8	20	18	12	6	1	7	21	40	18	12	18	18	18	11	46	5	22	10	18
ENSG00000047410	5917	4257	5390	4465	9426	10336	8149	11001	6974	5438	25216	4250	6404	5931	7187	7759	2595	2425	2791	4516	20120	8040	13470	7655	5871	9192	5574	18040	4696	6591	6660	7861
ENSG00000202025	0	0	0	0	0	0	0	0	0	0	0	0	0	0	0	0	0	0	0	0	0	0	0	0	0	0	0	0	0	0	0	0
ENSG00000157181	549	283	390	308	580	581	470	676	470	413	1764	255	381	387	478	533	367	350	372	494	1409	621	907	639	428	586	474	1078	326	439	483	589
ENSG00000279374	1	1	1	0	0	1	4	0	1	0	4	0	1	1	1	0	2	0	0	1	3	2	3	3	1	8	1	3	1	1	0	2
ENSG00000262180	23	14	18	22	19	27	17	18	29	21	143	15	33	24	21	27	4	5	8	22	36	15	21	21	42	35	27	103	8	32	18	26
ENSG00000229739	27	13	19	16	18	13	12	17	19	13	50	6	11	28	17	28	7	9	4	15	22	8	8	9	15	12	13	29	10	20	11	24
ENSG00000116703	11	14	6	13	11	13	6	11	4	6	27	1	7	17	17	5	3	0	2	12	14	9	14	9	5	2	7	24	3	12	9	22
ENSG00000273198	0	0	0	0	0	1	1	0	0	0	3	0	1	0	0	0	1	1	0	0	1	0	0	0	3	1	2	0	0	0	2	2
ENSG00000228238	3	1	2	1	3	1	1	3	1	0	5	1	4	2	2	0	0	2	1	1	1	1	1	0	3	3	1	2	0	1	1	4
ENSG00000233196	0	0	1	0	2	0	0	0	1	1	5	0	0	0	3	0	0	1	0	6	9	0	2	0	0	0	0	2	1	0	0	2
ENSG00000073756	0	0	0	0	0	0	0	0	17	25	139	14	148	245	398	328	0	0	0	0	0	0	0	0	25	26	186	131	277	606	749	837
ENSG00000273129	0	0	0	0	0	0	0	0	7	4	8	3	8	11	8	17	0	0	0	0	0	0	0	0	6	2	5	3	13	13	13	21
ENSG00000116711	0	0	0	0	0	0	0	0	93	56	188	32	721	720	845	952	0	0	0	0	0	0	0	0	70	102	98	119	1239	1261	1881	1543
ENSG00000236030	12	7	10	4	0	0	2	0	0	0	0	0	20	31	20	36	6	9	7	10	0	0	1	0	0	0	0	0	15	31	35	45
ENSG00000231599	6	6	7	12	0	0	0	0	0	0	0	0	26	26	20	31	4	2	2	2	0	0	0	0	0	0	0	0	10	34	16	15
ENSG00000272827	3	8	2	0	0	0	0	0	0	0	0	0	10	11	6	11	1	1	2	4	0	0	0	0	0	0	0	0	4	14	3	9
ENSG00000271558	0	0	0	0	0	0	0	0	0	0	0	0	0	0	0	0	0	0	0	0	0	0	0	0	0	0	0	0	0	0	0	1
ENSG00000225462	0	0	0	1	1	0	0	0	0	0	0	0	0	0	0	0	0	0	0	0	0	0	0	0	0	0	0	0	0	0	0	0
ENSG00000274702	0	0	0	0	0	0	0	0	0	0	0	0	0	0	0	0	0	0	0	0	0	0	0	0	0	0	0	0	0	0	0	0
ENSG00000230426	0	0	0	0	0	0	0	0	0	0	0	0	0	0	0	0	0	0	0	0	0	0	0	0	0	0	0	0	0	0	0	0
ENSG00000236401	0	0	0	0	0	0	0	0	0	0	0	0	0	0	0	0	0	0	0	0	0	0	0	0	0	0	0	0	0	0	0	0
ENSG00000223847	0	0	0	0	0	0	0	0	0	0	0	0	0	0	0	0	0	0	0	0	0	0	0	0	0	0	0	0	0	0	0	0
ENSG00000150732	0	0	0	0	0	0	0	0	0	0	0	0	0	0	0	0	0	0	0	0	0	0	0	0	0	1	0	0	0	0	0	0
ENSG00000222240	0	0	0	0	0	0	0	0	0	0	0	0	0	0	0	0	0	0	0	0	0	0	0	0	0	0	0	0	0	0	0	0
ENSG00000261182	0	0	0	0	0	0	0	0	0	0	0	0	0	0	0	0	0	0	0	0	0	0	0	0	0	0	0	0	0	0	0	0
ENSG00000224278	1	0	0	0	0	0	0	0	0	0	0	0	0	0	0	0	0	0	0	0	0	0	0	0	0	0	0	0	0	0	0	0
ENSG00000225006	0	0	0	0	0	0	0	0	0	0	0	0	0	0	0	0	0	0	0	0	0	0	0	0	0	0	0	0	0	0	0	0
ENSG00000235083	0	0	0	0	0	0	0	0	0	0	0	0	0	0	0	0	0	0	0	0	0	0	0	0	0	0	0	0	0	0	0	0
ENSG00000231022	0	0	0	0	0	0	0	0	0	0	0	0	0	0	0	0	0	0	0	0	0	0	0	0	0	0	0	0	0	0	0	0
ENSG00000237283	0	0	0	0	0	0	0	0	0	0	0	0	0	0	0	0	0	0	0	0	0	0	0	0	0	0	0	0	0	0	0	0
ENSG00000238054	0	0	0	0	0	0	0	0	0	0	0	0	0	0	0	0	0	0	0	0	0	0	0	0	0	0	0	0	0	0	0	0
ENSG00000226486	0	0	0	0	0	0	0	0	0	0	0	0	0	0	0	0	0	0	0	0	0	0	0	0	0	0	0	0	0	0	0	0
ENSG00000271036	0	0	0	0	0	0	0	0	0	0	0	0	0	0	0	0	0	0	0	0	0	0	0	0	0	0	0	0	0	0	0	0
ENSG00000226196	0	0	0	0	0	0	0	0	0	0	0	0	0	0	0	0	0	0	0	0	0	0	0	0	0	0	0	0	0	0	0	0
ENSG00000232212	0	0	0	0	0	0	0	0	0	0	0	0	0	0	0	0	0	0	0	0	0	0	0	0	0	2	0	0	0	2	0	1
ENSG00000238270	0	0	0	0	0	0	0	0	0	0	0	0	0	0	0	0	0	0	0	0	0	0	0	0	0	0	0	0	0	0	0	0
ENSG00000230987	0	0	0	0	0	0	0	0	0	0	0	0	0	0	0	0	0	0	0	0	0	0	0	0	0	0	0	0	0	0	0	0
ENSG00000162670	0	0	0	0	0	0	0	0	0	1	1	0	0	0	0	0	0	0	0	0	1	0	0	0	0	0	1	1	0	0	0	0
ENSG00000225811	0	0	0	0	0	0	0	0	0	0	0	0	0	0	0	0	0	0	0	0	0	0	0	0	0	0	0	0	0	0	0	0
ENSG00000237457	0	0	0	0	0	0	0	0	1	1	0	0	0	0	0	0	0	0	0	0	0	0	0	0	0	0	2	0	0	0	0	0
ENSG00000241505	0	0	0	0	0	0	0	0	0	0	0	0	0	0	0	0	0	0	0	0	0	0	0	0	0	0	0	0	0	0	0	0
ENSG00000231175	0	0	0	0	0	0	0	0	0	0	0	0	0	0	0	0	0	0	0	0	0	0	0	0	0	0	0	0	0	0	0	0
ENSG00000236025	0	0	0	0	0	0	0	0	0	0	0	0	0	0	0	0	0	0	0	0	0	0	0	0	0	0	0	0	0	0	0	0
ENSG00000238108	0	0	0	0	0	0	0	0	0	0	0	0	0	0	0	0	0	0	0	0	0	0	0	0	0	0	0	0	0	0	0	0
ENSG00000228020	0	0	0	0	0	0	0	0	0	0	0	0	0	0	0	0	0	0	0	1	0	0	0	0	0	0	0	0	0	0	0	0
ENSG00000261642	0	0	0	0	0	0	0	0	0	0	1	0	0	1	0	0	0	0	0	0	0	0	0	0	0	0	0	0	0	0	0	0
ENSG00000271187	0	0	0	0	0	0	0	0	0	0	0	0	0	0	0	0	0	0	0	0	0	0	0	0	0	0	0	0	0	0	0	0
ENSG00000233882	0	0	0	0	0	0	0	0	0	0	0	0	0	0	0	0	0	0	0	0	0	0	0	0	0	0	0	0	0	0	0	0
ENSG00000223344	0	0	0	0	2	0	0	0	0	0	1	0	0	0	0	0	0	0	0	0	2	1	2	0	0	0	0	0	0	0	0	0
ENSG00000228215	0	0	0	0	5	10	10	17	0	0	0	0	0	0	0	0	0	0	0	0	23	8	16	14	0	0	0	0	0	0	0	0
ENSG00000150681	143	82	144	113	962	856	750	855	22	10	37	5	10	1	11	10	197	364	256	293	1974	763	1648	892	25	13	8	30	11	10	14	4
ENSG00000226723	3	5	3	0	2	1	3	4	0	0	1	0	2	0	2	2	3	2	4	1	13	1	6	0	2	0	0	3	2	3	1	0
ENSG00000236792	0	0	0	0	1	0	0	0	0	0	3	1	0	0	2	0	0	1	2	0	2	0	0	0	0	2	0	0	0	2	0	0
ENSG00000253148	0	0	0	0	1	1	0	3	2	1	19	2	3	3	0	1	0	0	0	0	5	2	1	2	8	2	6	11	0	0	0	0
ENSG00000232498	0	0	0	0	36	32	37	46	54	33	217	31	16	16	27	22	0	0	0	0	86	40	59	38	35	60	39	97	26	30	40	36
ENSG00000236069	0	0	0	0	1	7	1	1	7	3	24	1	4	4	5	8	0	0	0	0	5	4	4	4	5	8	6	21	1	4	1	6
ENSG00000090104	0	0	0	0	65	88	64	119	1434	1037	7566	809	3435	2960	5275	4037	0	0	0	0	86	46	39	61	494	711	2924	2797	2428	2996	4991	4331
ENSG00000127074	0	0	0	0	71	64	63	98	42	53	121	19	53	47	125	47	0	0	0	0	49	41	35	43	26	19	47	83	47	59	121	71
ENSG00000231767	14	14	10	5	4	12	2	9	26	17	45	15	20	15	30	10	21	13	16	24	22	4	19	11	17	20	15	41	21	19	21	25
ENSG00000228687	0	0	0	0	0	0	0	0	0	0	1	0	0	0	0	0	0	0	0	0	0	0	0	0	1	0	0	0	0	1	0	0
ENSG00000226814	0	0	0	0	0	0	0	0	0	0	0	0	2	0	1	0	0	0	0	0	0	0	0	0	0	0	0	0	0	4	0	0
ENSG00000116741	31	34	22	41	153	213	153	255	62	43	221	35	59	60	112	101	14	39	33	58	388	203	363	203	34	59	62	126	29	68	81	81
ENSG00000223075	0	0	0	0	0	0	0	0	0	0	0	0	0	0	0	0	0	0	0	0	0	0	0	0	0	0	0	1	0	0	0	0
ENSG00000234871	2	0	3	1	2	2	2	2	0	2	0	0	2	2	1	0	0	0	0	4	2	0	2	3	1	0	1	4	0	1	0	0
ENSG00000234473	17	6	6	4	7	14	6	11	7	2	16	0	9	5	5	3	2	2	2	17	9	15	12	9	10	9	4	27	1	5	5	5
ENSG00000116750	2477	1636	1913	1746	2625	2781	2481	3075	2442	1675	6780	1400	2613	2435	2671	2904	2881	3634	3184	3095	6668	2632	5043	2924	2468	3406	1317	5086	2653	2783	3193	3336
ENSG00000238754	1	0	3	1	2	0	0	1	2	1	5	0	4	1	6	1	3	2	2	5	2	1	2	11	5	0	0	2	1	2	4	7
ENSG00000116747	2804	1918	2047	1918	1883	1922	1551	1982	1832	1633	7393	1172	2825	2462	2722	2982	1183	1633	1524	1992	4049	1567	2818	1423	2135	2784	1900	5522	1982	2356	2387	2482
ENSG00000272579	178	122	169	149	181	173	132	176	198	179	639	94	200	200	234	257	59	112	94	132	331	133	219	139	233	231	176	446	174	199	218	241
ENSG00000023572	134	95	103	111	248	250	226	298	209	118	554	116	145	143	174	221	150	236	167	142	644	265	569	262	242	346	155	395	263	231	247	274
ENSG00000134371	1917	1285	1581	1255	1797	1965	1557	2094	1563	1222	5763	941	1472	1416	1785	1820	1507	2014	1904	1914	4668	1906	3491	1910	1671	2273	1544	4075	1630	1775	2206	2062
ENSG00000221680	0	0	0	0	1	0	0	1	0	1	0	1	0	0	1	0	0	0	0	0	1	0	1	1	0	1	0	0	0	0	0	0
ENSG00000162630	9	6	11	2	13	15	10	12	26	13	365	25	5	19	8	8	2	5	1	20	37	21	15	17	39	44	312	190	8	14	13	22
ENSG00000276735	3	3	3	0	1	2	1	1	3	2	1	2	3	1	0	1	0	0	2	2	1	4	0	1	2	5	1	7	0	0	0	2
ENSG00000232077	0	4	0	5	1	1	0	1	3	0	2	0	3	2	2	5	0	0	1	0	0	0	1	1	3	2	0	6	2	2	3	0
ENSG00000226640	0	0	0	0	0	0	0	0	0	0	0	0	3	3	2	4	0	0	0	0	1	3	0	0	0	0	0	0	4	1	4	4
ENSG00000237011	0	0	0	0	0	0	1	0	0	0	0	0	2	1	6	6	0	0	0	2	0	0	0	0	3	0	0	1	5	5	3	6
ENSG00000237213	0	0	0	0	0	0	0	0	0	0	0	0	1	2	2	1	0	0	0	0	0	0	0	0	0	0	0	0	0	3	1	1
ENSG00000227240	0	0	0	0	0	0	0	2	0	0	0	0	16	15	11	5	0	0	0	0	0	0	0	0	0	1	2	1	2	4	5	16
ENSG00000233057	0	0	0	0	0	2	0	1	1	0	10	1	11	7	18	17	0	1	0	0	1	0	0	0	1	0	1	0	6	3	7	10
ENSG00000231714	0	0	1	0	0	0	0	0	0	0	0	0	0	0	0	0	0	0	0	0	0	0	0	0	0	0	0	0	0	0	0	0
ENSG00000251813	0	0	0	0	0	0	0	0	0	0	0	0	0	0	0	0	0	0	0	0	0	0	0	0	0	0	0	0	0	0	0	0
ENSG00000228167	0	0	0	0	0	0	0	0	0	0	0	0	0	0	0	0	0	0	0	0	0	0	0	0	0	0	0	0	0	0	0	0
ENSG00000227421	0	0	0	0	0	0	0	0	0	0	0	0	0	0	0	0	0	0	0	0	0	0	0	0	0	0	0	0	0	0	0	0
ENSG00000162687	0	0	0	0	0	0	0	0	0	0	0	0	0	1	1	0	0	0	0	0	2	0	0	2	0	0	0	0	2	0	0	2
ENSG00000265986	0	0	0	0	0	0	0	0	0	0	0	0	0	0	0	0	0	0	0	0	0	0	0	0	0	0	0	0	0	0	0	0
ENSG00000000971	0	0	0	0	7	1	3	7	3713	2946	14368	2407	135	171	188	290	0	0	0	0	4	6	3	4	2483	3959	2757	7883	46	108	96	168
ENSG00000116785	1	1	0	1	21	20	17	14	13	17	259	14	3	6	3	10	0	0	0	1	13	9	15	9	4	2	57	72	0	3	5	5
ENSG00000276911	0	0	0	0	0	0	0	0	0	0	0	0	0	0	0	0	0	0	0	0	0	0	0	0	0	0	0	0	0	0	0	0
ENSG00000244414	1	0	0	1	0	1	1	0	0	0	1	0	0	0	0	0	1	0	0	0	0	1	2	1	0	0	3	0	0	0	0	0
ENSG00000134365	0	0	0	0	0	0	0	0	0	0	0	0	1	0	0	0	0	0	0	0	0	0	0	0	0	0	0	0	0	0	1	0
ENSG00000080910	0	0	0	0	0	0	0	0	0	0	0	0	0	0	0	0	0	0	0	0	0	0	0	0	0	0	0	0	0	0	0	0
ENSG00000273844	0	0	0	0	0	0	0	0	0	0	0	0	0	0	0	0	0	0	0	0	0	0	0	0	0	0	0	0	0	0	0	0
ENSG00000134389	0	1	0	0	0	0	0	0	0	0	0	0	0	0	0	0	1	0	0	0	0	1	0	0	0	0	0	0	1	0	0	0
ENSG00000143278	0	0	0	0	0	1	0	0	0	0	4	0	1	0	2	0	2	0	0	0	4	0	0	2	0	1	3	0	0	1	1	1
ENSG00000066279	8850	5993	7886	6176	6555	7025	4617	7129	5948	5446	19175	4135	2966	3122	3521	4225	3447	4126	4491	6516	12566	5278	8042	5394	7497	11101	2309	17333	2024	3603	3287	4194
ENSG00000235748	17	13	13	16	14	11	10	11	10	8	20	7	2	3	6	5	1	4	7	4	24	14	7	18	19	14	0	39	2	7	6	5
ENSG00000177888	1280	886	847	746	296	344	222	390	390	350	1625	269	634	616	541	657	665	930	750	993	732	315	492	367	423	648	417	1170	370	458	461	548
ENSG00000237861	0	1	1	3	0	0	1	0	0	0	0	0	2	1	0	0	1	0	2	1	2	2	0	2	0	3	0	3	0	1	0	0
ENSG00000134376	1	0	4	3	2	2	1	2	3	2	7	4	1	3	0	2	0	2	3	4	2	0	1	0	3	2	4	7	0	0	0	0
ENSG00000221879	0	0	0	0	0	0	0	0	0	0	0	0	0	0	0	0	0	0	0	0	0	0	0	0	0	0	0	0	0	0	0	0
ENSG00000230260	0	0	0	0	0	0	0	0	0	0	0	0	0	0	0	0	0	0	0	0	0	0	0	0	0	0	0	0	0	0	1	0
ENSG00000213047	1634	861	1308	895	1887	1836	1428	1783	891	665	4421	603	2596	2214	2297	2549	925	1017	1343	1585	4283	1679	3179	1798	1530	2049	1333	3609	1988	2605	2174	2849
ENSG00000207139	1	0	0	0	0	2	1	0	0	0	1	0	0	1	0	0	0	0	0	1	0	0	0	2	4	0	0	3	0	1	4	1
ENSG00000213046	8	4	3	1	1	7	2	1	1	1	6	2	1	6	5	10	4	0	0	8	4	1	3	2	10	8	4	15	6	8	8	22
ENSG00000235582	3	0	1	0	4	1	0	3	0	0	0	0	0	2	3	1	1	0	0	4	0	0	2	1	5	2	2	5	0	7	1	2
ENSG00000233040	6	3	4	0	3	3	0	8	1	5	5	0	4	5	3	1	1	0	2	10	7	5	7	4	6	11	3	19	1	8	5	6
ENSG00000224901	2	1	1	3	1	1	5	2	2	0	5	0	3	1	1	3	1	0	0	5	8	3	4	2	8	1	1	8	2	3	1	14
ENSG00000203724	16	19	18	16	45	45	24	40	33	25	159	24	29	38	34	63	31	61	54	58	131	54	67	45	56	74	33	111	68	52	57	85
ENSG00000143355	5	4	8	5	0	0	0	0	2	0	7	0	21	24	21	47	1	8	1	7	0	0	0	0	1	4	4	6	8	15	16	20
ENSG00000151414	5789	3073	4983	3159	2031	2064	1495	1972	2847	2324	11390	1683	1831	1672	1997	2112	4491	4628	5773	4789	4542	1850	3190	1918	3888	5253	2823	8870	2190	2378	2836	2718
ENSG00000232824	5	5	14	10	0	3	1	3	2	1	17	2	3	1	0	2	4	3	3	10	4	4	1	3	11	13	3	30	4	7	7	10
ENSG00000151418	0	0	0	0	0	1	0	0	1	0	1	2	0	0	0	0	0	0	0	0	0	0	0	0	0	2	1	1	1	0	0	0
ENSG00000223881	0	0	0	0	1	1	0	0	5	5	18	3	0	0	0	0	0	0	0	0	1	0	0	0	1	6	0	0	0	0	0	0
ENSG00000081237	16788	8325	14978	8045	16757	16532	14296	18016	12331	10215	38721	5975	3650	3414	3504	4389	14793	12807	17748	13294	43914	17060	31603	17062	13665	17099	11524	27219	3021	3592	3183	4100
ENSG00000261573	20	14	13	1	8	8	4	7	6	10	14	4	1	4	3	2	2	2	1	16	31	22	12	29	28	24	6	33	0	2	5	6
ENSG00000236278	12	5	12	2	3	4	2	4	10	4	21	3	4	6	7	4	3	0	1	7	9	13	8	5	16	15	12	27	5	10	2	11
ENSG00000229989	132	51	110	44	59	77	58	73	36	25	121	19	35	55	55	52	48	37	47	181	198	164	133	132	154	98	31	198	29	83	104	81
ENSG00000207975	8	6	14	6	6	7	2	6	2	1	19	2	5	5	7	4	9	7	5	5	22	19	10	16	6	5	4	10	6	12	5	8
ENSG00000207759	6	1	2	3	0	4	0	1	0	0	2	2	3	3	2	3	3	1	1	4	7	3	8	4	6	3	0	4	0	1	0	3
ENSG00000227747	45	34	62	29	31	23	25	17	0	0	0	0	12	5	13	13	9	6	20	15	39	7	21	24	0	0	0	0	5	8	6	5
ENSG00000225172	101	45	100	50	50	59	52	49	0	0	3	0	20	10	13	12	17	15	25	46	87	70	70	50	0	1	3	0	9	13	12	13
ENSG00000233410	280	201	437	158	125	113	115	124	2	1	0	0	38	21	42	38	106	76	120	197	254	123	181	143	1	1	0	0	17	25	32	33
ENSG00000235492	114	50	123	54	157	145	169	162	0	0	1	0	11	6	11	7	33	24	41	36	322	136	257	195	0	0	0	0	2	8	15	2
ENSG00000231718	4	3	6	3	3	1	0	0	0	0	0	0	0	0	0	3	4	0	4	4	3	0	2	0	0	0	0	0	0	0	1	0
ENSG00000228190	0	0	0	0	0	0	0	0	0	0	0	0	0	0	0	0	0	0	0	0	0	0	0	0	0	0	0	0	0	0	0	0
ENSG00000236468	0	0	0	0	0	0	0	0	0	0	0	0	0	0	0	0	0	0	0	0	1	0	0	0	0	0	0	0	0	0	0	0
ENSG00000231984	0	0	0	0	0	0	0	0	0	0	0	0	0	0	0	0	0	0	0	0	0	0	0	0	0	0	0	0	0	0	0	0
ENSG00000228530	0	0	0	0	0	0	0	0	0	0	0	0	0	0	0	0	0	0	0	0	0	0	0	0	0	0	0	0	0	0	0	0
ENSG00000200139	0	0	0	0	0	0	0	0	0	0	0	0	0	0	0	0	0	0	0	0	0	0	0	0	0	0	0	0	0	0	0	0
ENSG00000229747	0	0	0	0	0	0	0	0	0	0	0	0	0	0	0	0	0	0	0	0	0	0	0	0	0	0	0	0	0	0	0	0
ENSG00000202491	0	0	0	0	0	0	0	0	0	0	0	0	0	0	0	0	0	0	0	0	0	0	0	0	0	0	1	0	0	0	0	0
ENSG00000202329	0	0	0	0	0	0	0	0	0	0	0	0	0	0	0	0	0	0	0	0	0	0	0	0	0	0	0	0	0	0	0	0
ENSG00000116833	0	0	0	0	13	20	8	19	0	0	1	0	305	322	359	400	0	0	0	0	9	1	7	4	1	0	2	1	162	145	242	235
ENSG00000252860	0	0	0	0	0	0	0	0	0	0	0	0	0	0	0	0	0	0	0	0	0	0	0	0	0	0	0	0	0	0	0	0
ENSG00000229220	0	0	0	0	0	0	0	0	0	0	0	0	0	2	0	0	0	0	0	0	0	0	0	0	0	0	0	0	0	0	0	0
ENSG00000233827	0	0	0	0	0	0	0	0	0	0	0	0	0	0	0	0	0	0	0	0	0	0	0	0	0	0	0	0	0	0	0	0
ENSG00000203721	16	23	3	5	8	7	4	4	3	1	11	0	3	18	3	10	0	10	4	6	11	5	5	2	1	8	3	5	2	5	3	4
ENSG00000273093	0	0	4	0	0	0	0	0	0	0	0	0	0	0	0	0	0	0	0	0	1	0	0	0	0	0	0	0	0	0	0	0
ENSG00000213045	1	0	0	0	0	0	0	1	0	0	0	0	0	0	0	0	0	0	0	1	0	0	0	0	0	0	0	2	0	0	0	0
ENSG00000230623	3	2	5	1	1	0	4	0	1	0	3	0	3	3	0	4	0	0	0	6	2	0	1	1	0	0	0	0	0	0	1	2
ENSG00000162702	1854	1364	1515	1397	1360	1405	1064	1392	703	575	2467	424	834	781	868	1040	735	901	829	1337	2650	1066	1771	1025	747	1068	595	1601	724	952	794	1030
ENSG00000282849	0	0	0	0	0	0	0	0	0	0	0	0	0	0	0	0	0	0	0	0	0	0	0	1	0	0	0	0	0	0	0	0
ENSG00000118193	3052	2322	2691	2403	2550	2772	1919	2905	2670	2229	8500	1577	1515	1500	1768	1994	991	1386	1343	1731	3865	1755	2638	1590	3153	4855	871	6703	1043	1556	1438	1792
ENSG00000118197	846	592	678	619	433	474	346	503	542	407	1995	308	417	416	415	474	577	891	838	691	878	357	602	369	476	680	484	1218	311	327	340	384
ENSG00000260088	3	2	2	6	0	5	2	9	6	4	5	2	10	14	6	17	0	2	2	0	5	2	4	0	5	0	1	4	7	8	7	2
ENSG00000118200	44	97	92	128	244	380	237	444	436	418	3031	390	508	547	577	709	20	89	89	130	362	170	292	200	411	674	1094	1981	426	785	648	785
ENSG00000231333	0	0	0	0	0	0	3	0	3	0	5	1	2	2	3	1	0	0	0	0	0	0	0	1	4	1	6	6	1	2	2	6
ENSG00000170128	0	0	0	0	0	2	0	0	20	37	101	21	48	41	73	66	0	0	0	0	0	0	1	0	13	23	19	21	14	20	18	26
ENSG00000163362	0	0	2	0	3	1	1	3	7	2	8	1	256	160	249	225	0	2	2	1	0	2	0	0	1	11	5	6	101	110	88	119
ENSG00000233217	4	2	0	1	2	3	1	6	2	4	3	1	1	7	5	3	0	0	0	2	1	4	0	3	2	2	3	6	0	0	3	2
ENSG00000201032	0	0	0	0	0	0	0	0	0	0	0	0	0	0	0	0	0	0	0	0	0	0	0	0	0	0	0	0	0	0	0	0
ENSG00000116852	4706	3464	3994	3728	2586	2965	1704	2799	2886	2581	9999	1852	4406	3465	3388	4312	999	1385	936	2361	3588	1593	2273	1363	2681	4498	1754	6171	1657	2311	1764	2617
ENSG00000229191	30	42	20	32	21	26	16	26	32	37	65	12	37	22	26	39	16	18	2	17	22	12	22	17	25	34	9	39	7	11	10	18
ENSG00000234132	1	0	0	1	1	1	1	0	0	0	0	0	0	0	0	0	0	0	2	0	0	0	0	0	0	0	0	0	0	0	0	0
ENSG00000081248	2	1	0	0	0	1	1	1	0	0	0	0	0	0	0	0	0	0	0	0	0	0	0	0	0	0	0	0	0	0	1	0
ENSG00000232237	5	5	1	7	4	4	2	4	3	3	12	6	4	5	3	10	0	4	1	0	1	1	3	1	0	2	2	4	3	7	3	9
ENSG00000116857	539	555	547	535	1093	1168	775	1242	98	119	299	66	369	371	356	493	257	441	422	413	1698	718	1263	626	72	114	96	181	317	356	329	413
ENSG00000163395	0	0	0	8	0	0	0	0	0	0	0	0	3	2	2	1	0	1	0	3	1	0	0	0	0	1	0	0	0	0	0	0
ENSG00000229821	0	2	0	0	0	0	0	0	0	0	0	0	0	0	0	0	0	0	0	0	0	0	0	0	0	0	0	0	0	0	0	0
ENSG00000081277	6	3	1	9	0	0	0	0	0	2	2	0	3	1	1	0	0	2	6	1	0	0	0	0	0	0	1	1	2	2	4	1
ENSG00000118194	0	0	0	0	0	0	0	0	0	0	2	0	0	0	0	1	0	0	0	0	0	0	0	1	0	0	1	0	0	0	0	0
ENSG00000159166	0	0	0	0	0	0	0	0	0	0	0	0	0	0	0	0	0	0	0	0	0	0	0	0	0	0	0	0	0	0	0	0
ENSG00000282221	0	0	0	0	0	0	0	0	0	0	0	0	0	0	0	0	0	0	0	0	0	0	0	0	0	0	0	0	0	0	0	0
ENSG00000159173	4	1	1	2	0	0	0	0	0	0	1	0	0	0	0	0	0	0	0	0	0	0	0	0	0	0	0	0	0	0	1	1
ENSG00000227048	0	0	0	0	0	0	0	0	0	0	0	0	0	0	0	0	0	0	0	0	0	0	0	0	0	0	0	0	0	0	1	0
ENSG00000174307	85	129	112	116	133	164	115	168	226	217	5511	746	3	8	6	15	15	50	55	87	225	104	185	90	248	417	1320	2924	5	8	1	10
ENSG00000224818	0	0	0	0	0	0	0	0	0	0	0	0	0	0	0	0	0	0	0	0	0	0	0	0	0	0	0	0	0	0	0	0
ENSG00000159176	1373	1145	1188	1142	2199	2329	1669	2518	1745	1263	6067	1190	1753	1689	1830	2209	443	795	609	1052	3717	1615	2739	1407	1459	2211	1253	3975	1628	1994	1775	2224
ENSG00000224536	192	141	154	134	105	125	70	110	73	64	289	43	95	70	67	95	43	61	37	99	101	44	60	36	65	51	56	156	33	54	40	41
ENSG00000223396	404	322	313	356	167	192	116	184	141	88	604	90	161	136	144	187	107	101	121	207	210	90	130	88	89	117	150	314	45	82	58	95
ENSG00000242150	0	0	0	0	0	0	0	0	0	0	0	0	0	0	0	0	0	0	0	0	0	0	0	0	0	0	0	0	0	0	0	0
ENSG00000134369	282	426	268	445	1398	1560	956	1574	59	46	161	32	1929	1709	1721	2089	33	122	50	184	1494	644	896	609	24	38	53	125	586	943	703	1110
ENSG00000236390	1	0	2	0	2	6	1	3	0	0	0	0	2	6	10	2	0	0	0	1	5	1	2	10	0	0	0	0	1	5	3	5
ENSG00000231871	10	11	12	12	14	19	11	17	24	12	68	9	13	16	23	26	15	8	11	28	69	19	27	26	21	17	12	47	16	25	8	14
ENSG00000264802	0	0	0	0	0	1	2	0	0	0	0	0	0	0	0	0	0	0	0	0	1	0	0	0	0	0	0	0	0	0	1	3
ENSG00000235121	2	3	1	3	8	4	7	9	0	1	2	0	9	7	14	6	4	0	1	3	8	6	5	1	0	0	0	0	2	9	5	13
ENSG00000200942	0	1	0	0	2	3	1	2	0	0	0	0	0	1	0	2	0	1	0	0	4	0	1	0	0	0	0	0	0	0	1	1
ENSG00000221028	0	0	0	0	0	0	0	0	0	0	0	0	0	0	0	0	0	0	0	0	0	0	0	0	0	0	0	0	0	0	0	0
ENSG00000198700	3933	3094	3451	3060	4622	4763	3689	5096	3248	2789	10581	2021	2617	2588	2490	3265	1233	1605	1458	2772	7407	3181	5066	2860	2568	4061	2172	7504	1387	2442	1572	2540
ENSG00000277681	8	19	14	16	6	8	10	11	12	9	30	8	5	4	9	10	2	3	3	10	17	12	6	9	11	7	5	20	3	7	7	13
ENSG00000198892	0	2	2	1	23	10	11	18	2	2	31	7	0	8	5	13	2	1	1	9	39	14	17	11	6	11	5	17	2	10	2	11
ENSG00000223774	0	0	0	0	1	1	1	2	0	0	4	0	1	2	0	0	0	0	0	0	0	0	0	0	0	0	1	1	0	0	0	1
ENSG00000163431	1	1	1	3	7	8	5	7	0	1	4	1	4	0	2	2	1	0	0	1	4	5	4	1	0	0	2	4	0	2	5	4
ENSG00000134375	1842	1196	1580	1363	1811	1969	1474	2109	1318	943	4302	797	1556	1515	1951	1972	2185	2800	2798	2474	5114	2003	3344	2095	1464	2108	876	2917	2504	2328	2972	2819
ENSG00000206637	0	0	0	0	0	0	0	0	0	0	0	0	0	0	0	0	0	0	0	0	0	0	1	1	0	0	0	1	0	0	0	1
ENSG00000238003	0	0	0	0	0	0	0	0	0	1	1	0	5	0	0	0	0	0	0	0	0	0	0	0	0	0	1	0	0	0	0	0
ENSG00000176393	1152	1054	984	1078	2712	3003	1842	2768	1333	1083	5764	914	1558	1451	1443	1826	216	512	388	682	3476	1476	2387	1171	1043	1794	1146	3222	1087	1310	1085	1398
ENSG00000273478	55	50	42	53	65	58	55	64	32	19	158	23	30	38	33	41	9	8	1	29	90	41	65	56	21	35	33	92	4	39	16	44
ENSG00000234678	14	16	19	17	25	35	27	38	18	13	111	10	13	12	17	20	5	1	4	14	52	19	21	20	11	16	22	54	4	12	10	12
ENSG00000275207	0	0	0	0	0	1	1	3	0	0	5	0	0	0	0	0	0	0	0	0	2	1	0	1	0	0	0	2	0	0	0	0
ENSG00000163435	1	8	5	9	15	24	8	29	4	3	35	6	9	44	13	35	1	3	1	3	7	6	8	4	3	1	15	10	5	15	4	7
ENSG00000249007	0	0	1	0	4	0	0	0	0	0	0	0	0	0	0	0	0	0	0	0	0	0	0	0	0	0	0	0	0	0	0	0
ENSG00000232296	0	0	0	0	0	0	0	0	0	0	0	0	0	0	0	0	0	0	0	0	0	0	0	0	0	0	0	0	0	0	0	0
ENSG00000235811	0	0	0	0	0	0	0	0	0	0	0	0	0	0	0	0	0	0	0	0	0	0	0	0	0	0	0	0	0	0	0	0
ENSG00000212512	0	0	0	0	0	0	0	0	0	0	0	0	0	0	0	0	0	0	0	0	0	0	0	0	0	0	0	0	0	0	0	0
ENSG00000227109	0	0	0	0	0	0	0	0	0	0	0	0	0	0	0	0	0	0	0	0	0	0	0	0	0	0	0	0	0	1	0	0
ENSG00000170075	6	3	7	2	17	27	18	22	9	6	39	5	3	6	9	8	2	1	0	6	29	13	15	23	7	24	25	54	2	3	9	11
ENSG00000143862	720	517	586	498	546	569	355	503	362	311	2089	259	298	259	311	344	195	261	316	418	856	380	539	280	356	546	454	1052	212	238	242	278
ENSG00000143851	4521	3392	5496	3474	6827	7246	5997	7976	4007	3132	12139	2504	3597	3496	4916	4771	1851	2215	3434	4931	14015	6951	10706	6338	3254	5429	2239	8386	3741	5404	4413	6052
ENSG00000243323	88	77	115	120	353	583	392	589	307	316	434	106	522	482	421	588	9	16	12	66	359	338	276	278	27	53	49	138	83	285	134	296
ENSG00000133067	8	2	18	5	1	3	1	1	2	1	4	0	1	0	0	0	1	1	2	8	1	1	2	1	0	1	0	0	0	0	0	0
ENSG00000077152	2552	1565	1949	1586	1229	1184	856	1271	1059	732	2487	606	1041	1002	1171	1392	3091	3584	3432	2986	2952	1148	2109	1398	1064	1474	316	2063	1216	1063	1406	1392
ENSG00000077157	1167	1028	976	1024	641	717	475	729	615	548	2199	391	493	535	492	620	243	252	215	734	1086	481	695	549	510	747	489	1545	214	505	274	578
ENSG00000229120	3	4	2	0	3	1	3	1	0	0	3	0	2	0	1	0	0	1	0	2	4	1	2	3	3	2	0	4	0	4	4	1
ENSG00000272262	0	0	0	2	0	0	1	0	0	0	0	0	0	0	0	0	0	0	0	1	2	0	0	1	0	0	0	0	0	0	0	0
ENSG00000232626	7	5	4	8	2	3	0	8	3	3	7	0	5	0	5	5	2	0	1	9	4	7	2	5	2	5	0	13	1	4	1	0
ENSG00000236439	2	2	1	2	1	0	3	0	1	3	7	3	0	0	0	1	2	1	3	6	1	1	1	0	1	2	4	6	1	3	3	1
ENSG00000253042	1	3	2	0	2	5	2	1	1	2	4	0	1	3	0	2	1	1	0	1	2	0	0	2	1	0	9	6	0	0	0	2
ENSG00000143858	25	31	57	25	12	21	24	25	10	10	53	13	6	19	1	13	6	10	5	37	33	29	24	21	9	14	11	50	5	18	9	15
ENSG00000226862	6	4	5	3	4	2	1	1	2	2	16	1	0	1	4	0	1	5	2	5	8	2	1	2	1	3	5	2	0	1	1	4
ENSG00000225620	0	0	0	0	1	0	0	0	0	0	0	0	0	0	0	0	0	0	0	1	0	0	0	0	0	0	0	0	0	0	0	0
ENSG00000117139	4467	3882	4339	3696	7399	6493	5694	7275	557	556	3076	436	47	96	60	123	670	633	709	1206	9027	3643	6836	3251	256	402	1657	1467	18	71	25	60
ENSG00000235449	29	30	26	32	27	25	21	44	2	5	30	1	1	1	0	1	1	2	1	9	49	11	16	20	0	2	10	17	0	1	0	0
ENSG00000226148	17	17	21	6	18	20	15	33	4	5	9	1	0	1	0	0	5	2	0	15	25	35	26	26	6	2	4	14	0	0	0	0
ENSG00000260021	7	4	1	6	2	1	1	2	1	1	0	0	0	0	0	0	1	0	0	0	1	0	0	0	0	0	0	0	0	0	0	0
ENSG00000228288	53	43	29	36	27	30	23	43	1	10	30	3	1	4	0	8	13	4	8	8	24	9	21	2	0	1	7	8	2	1	0	10
ENSG00000184774	3	1	1	2	1	0	0	2	0	0	1	2	0	0	0	0	0	0	0	0	1	1	1	1	0	0	2	0	0	0	0	0
ENSG00000214796	1	0	3	2	0	2	1	0	4	1	4	0	0	0	0	0	2	3	0	0	1	0	0	0	0	0	4	3	1	0	3	0
ENSG00000234996	5	3	3	1	5	8	2	2	8	4	26	8	3	2	2	1	1	0	4	4	7	1	1	7	4	4	7	13	0	3	2	2
ENSG00000183155	423	323	408	324	306	304	285	330	287	253	1506	244	178	234	258	286	411	505	546	472	814	278	621	346	395	488	404	1050	221	283	302	328
ENSG00000117153	2249	1412	1660	1446	1019	982	775	1129	895	766	2776	504	1401	1296	1217	1513	867	1195	1079	1311	1594	661	1163	593	729	1105	716	1737	897	1075	1013	1122
ENSG00000230280	32	34	44	35	17	26	20	19	13	25	89	10	25	9	21	23	11	9	9	52	33	29	11	26	10	12	18	47	14	10	6	17
ENSG00000199471	11	8	3	8	1	2	1	3	8	1	21	0	4	4	12	3	4	5	1	4	8	2	8	4	1	4	4	8	0	0	1	2
ENSG00000159346	2475	1846	2101	1783	1606	1571	1142	1689	1469	1162	5588	953	1651	1568	1749	2092	1164	1841	1725	1513	2617	1023	2001	954	1242	2011	1269	3595	1543	1695	1747	1919
ENSG00000159348	444	329	455	319	39	48	32	31	320	264	1035	184	138	157	111	200	204	270	324	385	44	34	45	27	199	344	271	688	106	139	115	157
ENSG00000234761	0	0	0	0	0	0	0	0	0	0	0	0	0	0	0	0	0	0	0	0	0	0	0	0	1	1	0	0	0	0	0	0
ENSG00000231547	0	0	0	0	0	0	0	0	0	0	0	0	0	0	0	0	0	0	0	0	0	0	0	0	0	0	0	0	0	0	0	0
ENSG00000163444	2703	1711	2166	1677	1897	1842	1240	1930	1192	899	4162	703	1064	957	1092	1233	2697	3517	2535	2374	4375	1614	2925	1696	1250	1758	896	2684	1238	1281	1362	1446
ENSG00000143847	1634	1751	1357	1834	2325	2849	2100	3392	1223	1004	337	374	245	314	145	326	102	66	42	165	547	317	324	312	30	62	9	66	6	25	4	32
ENSG00000122180	2	2	0	0	1	2	5	6	0	3	0	0	0	0	0	0	0	0	0	0	1	0	1	1	0	0	0	0	0	0	0	0
ENSG00000163485	3	22	3	15	30	29	18	44	1	0	1	0	0	0	0	0	0	13	2	13	67	28	58	23	0	0	1	1	0	0	0	0
ENSG00000234775	0	0	0	0	0	0	0	0	0	0	0	0	0	0	0	0	0	0	0	0	0	0	0	0	0	0	0	0	0	0	0	0
ENSG00000224671	0	0	0	1	0	1	0	1	0	0	0	0	0	0	0	0	0	0	0	0	2	0	0	1	0	0	0	0	0	0	0	0
ENSG00000133055	0	0	0	0	2	0	0	1	0	0	0	0	0	1	0	0	0	0	0	1	0	0	0	0	0	0	0	0	0	0	0	0
ENSG00000133048	0	0	0	0	0	0	0	0	0	0	2	0	6	3	8	7	0	0	0	0	0	0	0	0	0	0	1	1	3	7	3	20
ENSG00000133063	0	0	3	1	3	1	1	1	1	0	0	0	0	1	0	0	0	0	0	0	2	0	2	2	0	2	0	0	0	0	4	0
ENSG00000236523	0	0	0	0	0	0	0	0	0	0	0	0	1	0	0	0	0	0	1	0	0	0	0	0	0	0	0	1	0	0	1	0
ENSG00000231507	1	1	5	1	9	11	9	16	0	1	4	1	2	5	3	6	2	6	19	6	22	9	16	8	2	1	1	0	6	2	3	3
ENSG00000233791	12	6	14	7	15	12	12	16	0	0	1	0	0	3	0	1	5	3	3	3	23	1	14	7	1	0	0	1	0	0	0	0
ENSG00000159388	409	326	337	311	1508	1497	1106	1577	709	646	5210	913	272	354	317	504	116	121	126	157	1975	921	1707	776	415	783	1324	2618	141	208	153	285
ENSG00000202300	0	0	0	0	3	0	1	5	0	0	1	1	0	0	0	0	0	0	0	0	0	0	0	0	0	0	0	0	0	0	0	0

ENSG00000122176	0	0	0	2	0	0	0	2	0	0	0	0	0	0	0	0	2	1	3	3	1	0	1	0	0	0	2	0	0	0	0	0
ENSG00000229652	0	0	0	0	0	0	0	0	0	0	0	0	0	0	0	0	0	0	0	0	0	0	0	0	0	0	0	0	0	0	0	0
ENSG00000271588	0	0	0	0	0	0	0	0	0	0	0	0	0	0	0	0	0	0	0	0	0	0	0	0	0	0	0	0	0	0	0	0
ENSG00000188783	0	0	0	0	0	0	1	0	0	0	5	1	1	1	0	1	0	0	0	0	0	0	0	1	0	0	2	2	0	3	1	0
ENSG00000188770	0	0	0	0	0	0	0	0	0	0	0	0	0	0	0	0	0	0	0	0	0	0	0	0	0	0	0	0	0	0	0	0
ENSG00000058668	5893	5024	5462	4898	7458	7426	5448	7664	3218	2900	11666	2175	1352	1445	1308	1877	1096	1655	1276	3224	10322	4355	7223	3739	2404	3908	1418	6525	580	1024	706	1069
ENSG00000236035	8	4	9	11	13	11	15	19	9	0	26	0	1	1	2	4	4	2	0	9	20	17	7	12	4	8	2	9	0	4	5	2
ENSG00000221643	16	22	17	11	18	27	27	15	7	5	28	6	5	3	4	2	3	3	3	19	47	20	30	21	5	8	5	13	1	5	2	0
ENSG00000122188	567	361	547	392	528	626	548	650	170	110	524	102	282	237	315	304	333	357	465	480	1280	604	1111	582	100	154	121	277	395	450	461	454
ENSG00000058673	7575	5431	6102	5248	5572	5802	4192	6336	4348	4012	18238	2974	4230	3872	4342	5173	3246	3957	3823	5117	10584	4284	7076	4012	4186	5954	5098	11072	2762	3852	3613	4535
ENSG00000257315	525	341	411	344	485	533	363	584	282	244	1057	157	177	201	177	209	115	106	106	292	738	359	466	302	312	433	253	855	75	156	108	171
ENSG00000227417	8	5	10	5	6	4	7	10	10	4	17	2	3	1	4	2	2	0	1	7	12	16	5	14	7	13	18	34	4	5	3	7
ENSG00000223505	10	5	7	9	11	4	11	8	5	3	22	1	0	1	3	6	0	1	1	2	8	6	4	2	3	8	3	18	0	7	3	2
ENSG00000182004	2794	1784	1951	1732	2629	2850	2037	2799	2034	1335	5272	974	2188	2094	2240	2598	3176	3412	3506	4160	6739	2955	5231	3827	1820	2453	950	3263	2883	2558	3309	3376
ENSG00000236430	2	1	3	1	9	10	2	8	4	1	12	3	2	6	5	2	2	2	0	4	7	5	8	10	4	7	6	7	1	6	4	7
ENSG00000232917	0	0	0	0	0	0	0	0	0	0	0	0	0	1	0	0	0	0	1	0	0	0	1	0	0	0	0	0	0	0	1	0
ENSG00000237379	0	0	0	0	0	0	0	0	0	0	0	0	0	0	0	0	0	0	0	0	0	0	0	0	0	0	1	1	0	0	0	0
ENSG00000176754	1	0	0	0	0	0	0	0	0	0	0	0	0	0	0	1	0	0	0	0	0	0	0	0	0	0	0	0	0	0	0	0
ENSG00000143842	4	16	7	10	137	136	111	166	290	278	975	200	53	52	58	77	3	3	0	6	211	101	143	100	240	464	214	711	34	47	33	48
ENSG00000261065	0	0	0	0	0	0	0	0	0	0	0	0	0	0	0	0	0	0	0	0	0	0	0	0	0	0	0	0	0	0	0	0
ENSG00000143845	45	78	73	84	13	36	20	25	38	39	39	22	51	74	50	91	16	46	32	59	43	11	18	10	27	46	23	71	23	40	27	51
ENSG00000230550	0	0	2	0	0	1	0	0	1	0	0	0	0	0	0	0	0	0	0	0	0	0	0	0	0	0	0	1	0	0	0	2
ENSG00000143839	0	0	0	0	0	0	0	0	0	0	0	0	98	60	87	92	0	0	0	0	0	0	0	0	0	0	1	0	33	45	35	62
ENSG00000225522	0	0	0	0	0	0	0	0	0	0	0	0	0	0	1	0	0	0	0	0	0	0	0	0	0	0	0	0	0	0	0	0
ENSG00000170498	0	0	0	0	0	0	0	0	0	0	0	0	0	0	0	0	0	0	0	0	0	0	0	0	0	0	0	0	0	0	0	0
ENSG00000174567	0	0	0	0	0	0	0	0	0	0	0	0	2	0	0	0	0	1	0	0	0	0	0	0	0	0	0	0	0	0	1	1
ENSG00000143850	17	13	8	21	35	40	30	26	12	5	16	3	62	120	104	157	3	5	3	27	54	27	37	20	1	8	0	7	123	242	132	295
ENSG00000231691	0	2	0	1	2	1	3	1	0	0	0	0	0	1	0	0	0	0	0	0	1	1	2	0	0	0	0	0	0	0	1	0
ENSG00000219133	1	0	0	0	0	0	2	1	1	0	0	0	0	0	0	0	0	0	0	0	0	0	0	0	1	1	0	0	0	0	0	0
ENSG00000280924	1	2	0	2	0	0	0	0	0	0	1	0	0	0	1	0	0	0	1	2	0	0	0	2	0	0	0	0	0	0	0	0
ENSG00000226330	13	5	9	10	12	15	12	13	2	1	12	1	2	6	5	8	2	2	3	5	32	21	7	10	1	1	1	10	1	7	0	4
ENSG00000237848	2	2	0	1	1	1	1	1	1	0	4	1	1	3	2	0	1	0	0	3	3	0	1	5	0	0	3	10	2	0	1	1
ENSG00000158615	4241	3379	3641	3465	2300	2499	1791	2548	1677	1393	6444	1009	3024	2664	2745	3334	1001	1191	980	2595	3768	1565	2527	1436	1866	2782	1459	4326	1386	2131	1512	2136
ENSG00000133056	1110	769	970	711	1589	1841	1213	1515	308	247	1214	249	316	288	241	377	196	250	226	554	2195	1086	1497	926	277	443	341	807	148	247	151	320
ENSG00000198625	3863	2727	3286	2613	1903	1962	1351	2103	3319	2721	12631	1749	1659	1633	1412	1970	674	754	661	1815	2467	1074	1411	1153	2500	3451	3173	7591	680	1547	805	1493
ENSG00000236779	9	10	6	5	9	5	6	4	6	4	31	2	6	5	0	1	4	0	2	9	13	6	2	7	3	12	7	13	2	6	7	4
ENSG00000240710	1	1	2	0	1	2	2	8	0	1	3	0	0	0	1	2	0	0	0	2	1	0	1	3	0	1	1	5	0	0	0	1
ENSG00000170382	4	2	3	7	205	259	130	243	3	6	20	5	2	3	1	5	0	0	1	1	415	241	241	174	1	6	3	4	1	3	0	4
ENSG00000240219	5	2	3	2	8	13	12	11	2	5	12	1	1	4	1	2	2	3	0	0	30	19	5	20	0	0	3	3	1	2	0	2
ENSG00000228153	0	0	0	0	17	18	13	13	0	0	0	0	0	0	0	0	0	0	0	0	27	24	18	29	0	0	0	0	0	0	0	0
ENSG00000251861	0	0	0	0	0	0	0	0	0	0	0	0	0	0	0	0	0	0	0	0	0	0	0	0	0	0	0	0	0	0	0	0
ENSG00000163531	8	17	6	10	1566	1658	1237	1933	2	3	13	1	22	16	13	13	2	1	0	7	1936	1050	1533	970	0	0	0	3	5	14	10	21
ENSG00000229657	0	0	0	0	0	2	0	0	0	0	0	0	0	0	0	0	0	0	0	0	0	0	0	1	0	0	0	0	0	0	0	0
ENSG00000184144	19	13	14	13	15	13	10	15	10	2	8	2	7	12	13	8	2	0	3	12	6	10	9	7	8	4	1	19	4	9	16	26
ENSG00000174529	84	73	59	71	77	72	49	81	37	34	164	37	33	33	35	49	22	30	21	28	98	28	49	21	51	65	19	84	21	21	26	22
ENSG00000117222	1355	1036	1208	1170	922	915	786	1100	738	617	2817	486	814	813	1063	1041	491	821	680	937	1796	688	1371	647	868	1168	627	2045	725	865	876	1007
ENSG00000271580	29	20	32	19	10	14	15	17	16	16	50	11	12	12	15	8	2	9	6	16	23	16	10	15	13	14	4	39	2	23	22	18
ENSG00000236108	0	1	2	2	0	0	0	0	1	0	1	0	1	0	0	1	0	0	1	2	1	0	0	0	1	0	0	0	0	0	1	0
ENSG00000133059	593	610	532	616	844	938	611	972	405	327	1716	287	352	411	376	523	161	297	228	458	1262	522	875	469	299	477	542	1101	222	403	233	410
ENSG00000213041	4	7	14	1	3	5	2	7	5	3	14	1	2	0	1	1	0	0	0	4	6	3	5	4	2	8	1	7	2	0	3	2
ENSG00000133069	197	210	191	223	250	327	181	283	0	3	6	2	7	20	20	35	61	85	111	146	345	152	267	135	1	7	0	7	22	30	14	24
ENSG00000225063	1	1	1	1	5	3	2	10	0	0	0	2	0	0	0	1	1	0	0	0	1	2	0	4	0	0	0	0	0	0	0	0
ENSG00000163545	0	0	0	0	5	10	3	9	0	0	1	0	24	44	30	54	0	0	0	0	8	6	8	5	0	0	0	0	13	25	8	25
ENSG00000162873	0	0	0	0	0	0	0	1	0	0	0	0	1	0	3	2	0	0	0	0	0	0	0	0	0	0	0	0	0	0	0	1
ENSG00000235363	119	80	108	86	83	90	58	86	97	41	212	55	76	64	114	112	173	160	290	220	250	108	227	144	85	144	34	155	117	104	177	158
ENSG00000226235	0	0	0	0	0	0	0	1	0	0	0	0	7	1	4	8	0	0	0	0	0	0	0	0	0	0	0	0	2	1	0	4
ENSG00000186007	0	2	1	0	19	18	16	18	0	0	0	0	58	80	89	94	0	0	1	2	30	13	13	6	0	0	0	0	34	74	43	116
ENSG00000281406	1	4	1	0	8	30	17	17	1	2	0	0	104	114	119	167	1	0	0	3	24	14	13	12	0	0	0	1	66	114	91	184
ENSG00000199059	0	0	0	0	0	2	0	0	0	0	0	0	2	1	2	2	0	0	0	0	2	0	0	2	0	0	0	0	0	4	0	2
ENSG00000224717	0	0	0	0	30	36	24	55	0	0	0	0	5	2	2	5	1	0	0	0	50	22	32	18	0	0	0	0	3	7	0	2
ENSG00000117266	938	1052	748	954	756	838	495	811	81	87	259	82	938	885	882	1154	151	254	91	366	649	296	382	327	32	66	23	126	300	518	375	647
ENSG00000253097	0	0	0	0	0	0	0	0	0	0	0	0	0	0	0	0	0	0	0	0	0	0	0	0	0	0	0	0	0	0	0	0
ENSG00000174514	83	51	51	46	54	75	35	72	34	33	255	33	66	65	51	62	23	31	18	67	106	55	73	56	36	35	65	151	26	45	34	52
ENSG00000206762	16	8	13	12	11	10	7	9	13	8	54	2	6	12	6	8	6	5	5	16	10	6	11	6	5	7	3	26	1	4	1	9
ENSG00000158711	4728	3952	3859	3782	3087	3330	2396	3644	3092	2710	16661	2139	3468	3388	3258	3914	1196	1470	1264	3131	5645	2391	3795	2194	3706	5110	4108	11598	1706	2863	2101	3014
ENSG00000236942	20	16	19	8	22	9	3	16	23	18	85	7	9	3	8	15	3	1	4	15	25	14	17	15	32	36	24	64	1	11	13	10
ENSG00000158715	283	334	319	361	1028	1085	758	1126	29	21	31	18	44	66	54	80	117	243	205	353	1564	607	1046	528	11	22	4	22	43	82	42	63
ENSG00000275213	0	0	0	1	0	0	0	0	0	0	0	0	0	0	0	0	0	0	0	0	4	0	0	0	0	0	0	0	0	0	0	0
ENSG00000069275	15511	10565	12558	11334	15043	15501	10857	16160	16315	12840	47992	9653	15990	15799	16856	19373	5829	9047	8204	10461	22936	9611	17499	8316	17016	23341	8529	38726	12307	15865	13438	17424
ENSG00000201944	8	7	9	6	12	8	5	14	3	6	25	1	6	10	11	3	5	5	1	11	15	8	11	14	13	15	2	20	1	4	10	12
ENSG00000117280	889	621	772	702	728	775	563	839	564	462	1889	344	470	480	451	596	410	683	595	854	1206	510	965	505	508	848	419	1236	431	466	355	536
ENSG00000133065	2090	1554	1707	1546	1576	1599	1059	1689	250	275	872	240	467	478	514	659	521	728	689	1254	1793	776	1187	705	220	384	210	713	302	556	343	573
ENSG00000162877	4	8	5	6	2	2	0	3	12	8	26	4	3	2	1	8	3	2	1	7	1	2	1	6	13	10	1	35	0	0	0	2
ENSG00000174502	0	1	0	0	2	0	0	0	0	0	2	0	28	27	60	51	0	3	2	0	0	0	0	0	0	0	0	3	27	22	32	66
ENSG00000227687	0	0	0	0	0	0	0	0	0	0	0	0	0	0	0	0	0	0	0	0	0	0	0	0	0	0	0	0	0	0	0	0
ENSG00000276600	8	5	8	7	9	3	1	3	0	1	0	0	1	0	0	0	0	4	2	2	9	0	2	1	0	0	0	0	0	0	0	1
ENSG00000196188	4	0	1	1	15	11	19	14	0	0	0	0	0	0	0	1	1	3	6	1	37	23	43	12	0	0	0	1	0	0	0	1
ENSG00000263961	81	76	130	123	9005	8568	6656	8573	0	0	2	0	15	30	22	45	27	47	55	91	17002	6384	11175	6181	0	0	0	0	45	75	54	86
ENSG00000252692	0	0	0	0	7	0	2	2	0	0	0	0	0	0	0	0	0	0	0	0	5	0	2	4	0	0	0	0	0	0	0	0
ENSG00000198049	1	0	1	0	28	15	7	35	0	0	0	0	0	0	0	0	0	0	0	1	11	11	7	7	0	0	0	0	0	1	0	0
ENSG00000226780	0	0	0	0	4	10	8	2	0	0	0	0	0	0	0	0	0	0	0	0	8	6	2	1	0	0	0	0	0	0	1	0
ENSG00000225483	2	0	6	5	2	1	1	3	1	1	0	2	2	0	3	2	2	1	1	3	3	0	1	0	2	0	0	5	0	1	0	2
ENSG00000236889	6	5	8	4	3	3	1	1	2	0	3	1	1	1	0	3	1	0	1	3	1	0	0	0	1	1	1	2	0	1	2	0
ENSG00000196550	607	448	529	498	340	298	202	342	281	270	659	158	447	380	429	472	183	286	214	295	479	221	313	141	312	467	79	576	264	366	270	331
ENSG00000266028	1138	967	1078	959	1891	1897	1321	2027	1058	1067	4878	831	433	433	563	695	225	349	270	613	2137	983	1390	850	1220	2092	1263	3923	204	445	277	521
ENSG00000229509	0	0	0	0	0	2	1	0	0	0	2	0	1	0	0	0	0	0	0	2	0	2	3	2	2	0	0	5	0	1	0	1
ENSG00000263528	420	322	434	299	973	1006	824	1097	644	547	2000	399	1185	1037	1372	1528	216	207	248	390	1746	770	1424	689	553	771	419	1247	1239	1508	1397	1724
ENSG00000278465	1	0	0	0	0	0	0	0	0	0	1	0	0	0	0	1	0	0	0	0	1	0	1	1	0	0	0	1	0	0	0	1
ENSG00000162888	22	22	28	25	33	27	30	30	34	50	120	13	62	38	40	52	12	13	6	15	64	23	52	37	44	29	17	74	24	85	58	100
ENSG00000261000	6	2	4	1	11	13	13	17	2	7	24	0	13	11	14	16	2	0	2	0	24	6	15	8	4	5	7	17	10	17	7	18
ENSG00000266094	2900	1456	2797	1651	2324	2404	1704	2431	3639	2924	14234	2299	3732	3062	4186	3951	1073	1174	1894	1913	4208	1680	2897	1478	3461	5453	3664	8725	3498	3760	3526	4085
ENSG00000234981	1	5	3	1	8	6	2	9	4	4	15	4	2	9	3	3	0	0	1	1	5	1	1	3	2	7	0	8	0	4	1	3
ENSG00000279946	1	0	0	0	2	3	5	1	2	0	15	1	8	5	9	5	0	0	0	0	2	2	0	0	4	3	2	11	0	2	5	7
ENSG00000143486	1949	1340	1645	1316	1434	1427	1174	1527	1190	1008	3719	810	1525	1401	1494	1798	1194	1773	1578	1330	2769	1077	2311	1007	918	1338	806	2292	1234	1421	1504	1580
ENSG00000237605	8	5	8	5	21	19	14	15	2	0	4	1	6	2	3	9	4	11	7	9	41	15	36	15	1	1	1	1	2	7	5	4
ENSG00000143479	601	490	483	469	1353	1351	1027	1304	232	192	503	152	517	485	615	613	252	417	341	521	2715	1027	2155	877	177	275	113	358	328	396	410	491
ENSG00000162889	2294	1804	2001	1707	3850	3508	2717	3557	1548	1290	6912	1093	3220	2574	3312	3297	628	951	943	1284	6649	2587	4722	2275	1511	2577	1469	4201	2571	2942	2748	3196
ENSG00000224114	6	2	4	5	4	11	5	6	1393	481	4049	578	560	553	725	1046	3	1	6	2	15	7	12	14	416	1143	744	2012	732	678	1274	1163
ENSG00000199349	0	0	1	0	2	5	2	5	0	0	0	0	6	5	5	1	0	0	0	0	1	1	2	2	0	0	0	1	3	2	3	6
ENSG00000136634	1	3	0	0	3	0	0	0	0	0	0	0	861	607	893	789	0	0	0	0	1	1	1	2	0	0	0	0	568	602	554	715
ENSG00000142224	0	0	0	0	26	9	14	17	0	0	0	0	8	2	3	2	0	0	0	0	31	12	26	15	0	0	0	0	1	1	6	3
ENSG00000162891	0	0	0	0	1	0	0	0	0	0	0	0	0	0	1	0	0	1	0	0	2	0	0	0	0	0	0	0	0	0	0	0
ENSG00000162892	36	25	40	17	0	0	1	2	1	0	3	0	37	43	34	45	15	4	18	8	4	1	2	1	0	2	1	4	28	35	25	37
ENSG00000162894	176	106	204	91	24	25	6	35	4	4	17	5	190	141	206	235	74	89	125	117	26	9	21	13	0	3	3	12	148	177	164	194
ENSG00000271680	4	0	3	0	0	0	0	0	0	0	0	0	0	1	0	0	0	0	0	0	0	0	0	1	0	0	0	0	0	4	0	2
ENSG00000226945	2	0	0	0	0	0	0	0	0	0	0	0	3	2	0	0	0	0	0	0	1	0	0	0	0	0	1	0	2	0	1	4
ENSG00000162896	0	0	0	0	0	0	0	0	0	0	0	0	1	1	0	4	0	0	0	0	0	0	0	2	0	0	0	1	0	0	0	3
ENSG00000162897	0	0	0	0	0	0	0	0	0	0	0	0	4	4	7	8	0	0	0	0	0	0	0	0	0	0	0	0	4	1	6	4
ENSG00000182795	2	10	8	7	0	0	0	2	0	0	7	0	1	2	1	1	1	0	0	1	2	4	2	0	0	0	0	3	1	0	0	1
ENSG00000123836	789	566	664	603	45	68	59	98	155	163	909	117	255	275	279	340	212	243	287	569	91	57	69	73	198	360	353	716	197	282	172	360
ENSG00000180667	2880	2204	1840	2046	351	383	279	360	316	293	1337	263	881	888	916	1019	1101	1320	1021	1976	774	350	517	350	498	655	405	1179	734	944	911	1089
ENSG00000123843	1	0	0	0	0	0	0	0	0	0	0	0	0	1	0	0	0	0	0	0	0	0	0	0	0	0	0	0	0	0	0	0
ENSG00000123838	0	0	0	0	0	0	0	0	0	0	0	0	0	1	0	0	0	0	0	0	0	0	0	0	1	0	0	0	0	0	0	1
ENSG00000275392	0	0	0	0	0	0	0	0	0	0	0	1	0	1	0	1	0	0	0	0	0	0	0	0	0	0	0	0	0	0	0	0
ENSG00000226565	0	0	0	0	0	0	0	0	0	0	0	0	0	1	0	0	0	0	0	0	0	0	0	0	0	0	0	1	0	0	0	0
ENSG00000224462	0	0	0	0	0	0	0	0	0	0	1	0	1	1	0	3	0	0	0	0	0	0	0	0	0	0	0	0	0	0	0	0
ENSG00000243636	0	0	0	0	0	0	0	0	0	0	0	0	0	0	0	0	0	0	0	0	0	0	0	0	0	0	0	0	0	0	0	0
ENSG00000232621	0	0	0	0	0	0	0	0	0	1	0	0	0	0	1	0	1	0	0	0	0	0	0	0	0	0	0	0	1	0	0	0
ENSG00000237074	0	0	2	0	0	0	0	0	0	0	0	0	0	3	0	2	0	0	0	0	0	0	0	0	1	0	0	3	1	2	0	2
ENSG00000196352	91	148	67	119	669	706	536	771	468	389	2092	340	941	969	1031	1285	44	207	57	165	1039	454	698	397	421	754	527	1214	722	972	958	1060
ENSG00000274245	0	0	0	0	5	2	3	5	5	3	6	0	2	1	0	9	0	0	0	2	3	2	2	6	3	1	1	2	0	2	0	1
ENSG00000283044	0	0	0	0	0	0	0	0	0	0	0	0	0	0	0	0	1	0	0	0	0	0	0	0	0	0	0	0	0	0	0	0
ENSG00000117322	14	49	10	38	0	0	0	0	0	0	0	0	1	5	0	4	7	7	0	16	0	0	0	0	0	0	0	0	0	1	0	1
ENSG00000203710	0	0	0	0	0	0	0	0	0	1	5	0	0	0	0	0	0	0	0	0	0	0	0	0	0	0	0	0	0	0	0	0
ENSG00000236911	0	0	0	0	0	0	0	0	0	0	0	0	0	0	0	0	0	0	0	0	0	0	0	0	0	0	0	0	0	0	0	0
ENSG00000197721	1	0	0	0	0	0	0	0	0	0	0	0	0	1	0	1	0	0	0	4	0	0	0	0	0	0	0	0	0	0	0	0
ENSG00000244703	0	0	0	0	0	0	0	0	0	0	1	0	0	0	0	0	0	0	0	0	0	0	0	0	0	0	0	0	0	0	0	0
ENSG00000226289	0	0	0	0	0	0	0	0	0	0	0	0	0	0	0	0	0	0	0	0	0	0	0	0	0	0	0	0	0	0	0	0
ENSG00000117335	7521	4956	5587	4415	3640	3685	2703	3879	3588	3149	12785	2227	4955	4558	4529	5622	3519	3694	3379	4542	6926	2946	4713	3164	3009	4106	2968	7656	2866	3601	3513	4486
ENSG00000234219	11	0	14	4	5	11	6	8	3	4	8	2	4	2	4	7	1	0	0	12	17	4	8	4	2	6	1	11	2	6	3	2
ENSG00000203709	490	406	601	388	381	411	267	481	125	89	496	53	100	99	72	100	190	164	194	711	841	488	519	514	111	100	144	342	23	87	51	124
ENSG00000174059	421	383	549	389	13	14	15	9	1	0	0	0	2	0	0	0	180	360	407	559	12	7	9	7	0	0	0	0	0	0	1	0
ENSG00000076356	0	0	2	2	8	10	8	14	3	3	219	10	0	0	0	4	0	2	1	1	8	6	9	2	0	1	32	52	0	0	0	0
ENSG00000226843	0	0	0	0	0	0	0	0	0	0	0	0	0	0	0	0	0	0	0	0	0	0	0	0	0	0	0	0	0	0	0	0
ENSG00000261453	0	0	0	0	0	0	0	0	0	0	0	0	0	0	0	0	0	0	0	0	0	0	0	0	0	0	0	0	0	0	0	0
ENSG00000259815	0	0	0	0	0	0	0	0	0	0	0	0	0	0	0	0	0	0	0	0	0	0	0	0	0	0	0	0	0	0	0	0
ENSG00000227887	0	0	0	0	0	0	0	0	0	0	1	0	0	0	0	0	0	0	0	0	0	0	0	0	0	0	0	0	0	0	0	0
ENSG00000232812	0	0	0	0	0	0	0	0	0	0	0	0	0	0	0	0	0	0	0	0	0	0	0	0	0	0	1	0	0	0	0	0
ENSG00000236950	0	0	0	0	0	0	0	0	0	0	0	0	0	0	0	0	0	0	0	0	0	0	0	0	0	0	0	0	0	0	0	0
ENSG00000278684	0	0	0	0	0	0	0	0	0	0	0	0	0	0	0	0	0	0	0	0	0	0	0	0	0	0	0	0	0	0	0	0
ENSG00000232537	0	0	0	0	0	0	0	0	0	0	0	0	0	0	0	0	0	0	0	0	0	0	0	0	0	0	0	0	0	0	0	0
ENSG00000228081	0	0	0	0	0	0	0	0	0	0	0	0	0	0	0	0	0	0	0	0	0	0	0	2	0	0	0	0	0	0	0	0
ENSG00000224540	0	0	0	0	0	0	0	0	0	0	0	0	0	0	0	0	0	0	0	0	0	0	0	0	0	0	0	0	0	0	0	0
ENSG00000225712	0	0	0	0	0	0	0	0	0	0	0	0	0	0	0	0	0	0	0	0	0	0	0	0	0	0	0	0	0	0	0	0
ENSG00000227940	0	0	0	0	0	0	0	0	0	0	0	0	0	0	0	0	0	0	0	0	0	0	0	0	0	0	0	0	0	0	0	0
ENSG00000231648	0	0	0	0	0	0	0	0	0	0	0	0	0	0	0	0	0	0	0	0	0	0	0	0	0	0	0	0	0	0	1	0
ENSG00000230937	0	0	0	0	0	0	0	0	0	0	4	2	0	0	1	0	0	0	0	0	0	0	0	0	0	0	0	3	1	0	1	0
ENSG00000224260	0	0	0	0	0	0	0	0	0	0	1	0	0	0	0	0	0	0	0	0	0	0	0	1	0	0	0	0	0	0	0	0
ENSG00000008118	0	0	0	0	0	0	0	0	0	0	0	0	1	4	15	9	0	0	0	0	0	0	0	0	0	0	0	0	2	2	4	2
ENSG00000196878	3	6	10	4	4	9	3	4	7	3	14	4	105	109	129	147	1	5	2	4	3	4	5	0	1	3	2	6	17	18	37	33
ENSG00000227591	0	0	4	5	1	0	0	2	2	1	7	2	3	3	3	0	1	0	9	3	0	0	1	2	0	1	0	2	3	2	3	4
ENSG00000123689	0	0	0	0	0	0	0	0	0	0	1	0	0	3	5	0	0	0	0	0	0	1	0	0	0	0	0	0	0	0	0	0
ENSG00000117594	1	0	1	0	0	0	0	0	0	0	2	0	0	0	0	0	0	0	0	0	2	0	0	1	1	0	0	1	0	0	1	1
ENSG00000236136	0	0	0	0	0	0	0	0	0	1	0	0	0	0	0	0	0	0	0	2	0	0	0	0	0	0	0	1	0	0	0	1
ENSG00000009790	1480	795	1561	822	1816	1917	1567	2025	3095	2412	9890	1689	1426	1279	1605	1794	773	747	1151	1104	3170	1323	2638	1288	1929	2691	2021	4769	1351	1633	1549	1922
ENSG00000162757	328	293	329	332	167	178	117	193	190	144	639	130	166	187	175	245	189	257	199	260	274	136	228	116	177	260	141	412	96	159	145	200
ENSG00000117595	0	0	0	1	0	0	0	0	11	7	33	9	34	34	32	30	0	0	0	3	1	0	0	0	2	8	9	28	17	15	15	19
ENSG00000117597	630	409	532	421	719	794	577	808	741	643	2657	482	1006	965	1033	1115	433	630	466	592	1512	716	1141	616	774	1111	556	1742	905	1104	991	1183
ENSG00000143469	0	0	0	0	0	0	0	0	1	0	6	0	2	10	1	6	0	0	0	0	0	0	0	0	0	1	0	0	0	2	1	3
ENSG00000283619	0	0	0	0	0	0	0	0	0	0	0	0	0	0	0	0	0	0	0	0	0	0	0	0	0	0	0	0	0	0	0	0
ENSG00000203706	0	0	0	0	0	0	0	0	0	0	0	0	2	8	6	3	0	0	0	0	0	0	0	0	0	0	0	0	2	5	2	4
ENSG00000082497	0	1	0	0	0	0	0	0	0	0	1	0	8	24	22	22	0	0	0	0	0	0	0	1	0	0	1	0	8	19	11	16
ENSG00000228110	2	0	1	1	0	0	1	0	0	0	1	0	0	0	0	1	3	0	1	0	2	0	2	1	0	0	1	1	0	4	0	1
ENSG00000233455	0	0	0	0	0	0	0	0	0	0	0	0	0	0	0	0	0	0	0	0	0	0	0	0	0	0	0	0	0	0	0	0
ENSG00000054392	105	104	92	92	103	148	84	166	54	52	259	50	176	195	165	220	39	52	58	67	193	85	147	71	26	29	53	113	102	196	177	192
ENSG00000200972	4	1	2	0	1	0	0	0	0	0	0	0	0	0	0	0	2	2	2	1	3	0	1	1	0	0	0	0	0	1	0	2
ENSG00000270936	2	0	0	1	0	0	0	0	0	0	0	0	0	0	0	0	0	0	0	0	1	0	0	0	0	0	0	0	0	0	1	0
ENSG00000279333	0	0	0	0	0	0	0	0	0	0	0	0	0	0	0	0	0	0	0	0	0	0	0	0	0	0	0	0	0	0	0	0
ENSG00000143473	3	4	3	0	3	3	1	5	0	3	4	1	7	3	6	8	1	3	1	3	5	1	10	1	0	4	2	1	1	7	8	9
ENSG00000224668	0	0	0	0	0	1	0	0	1	0	3	0	0	0	0	0	0	1	0	0	1	0	2	0	0	0	0	0	0	0	0	0
ENSG00000234233	0	0	0	0	0	0	0	0	0	0	0	0	1	0	0	2	0	0	0	0	0	0	0	0	1	0	0	0	0	4	2	1
ENSG00000234004	0	0	0	0	0	0	0	0	0	0	1	0	0	0	0	0	1	1	0	0	0	0	0	0	0	0	0	0	0	0	0	0
ENSG00000223562	0	0	0	0	0	0	0	0	0	0	0	0	0	0	0	0	0	0	0	0	0	0	0	0	0	0	0	0	0	0	0	0
ENSG00000226986	0	0	0	0	0	0	0	0	0	1	1	0	0	0	0	0	0	0	0	0	1	0	0	0	0	0	0	0	0	0	1	0
ENSG00000117625	1160	922	1040	878	1538	1556	1178	1672	1109	980	4929	752	872	826	829	1068	426	473	535	883	2707	1204	1842	1188	953	1316	1404	3053	458	947	630	1012
ENSG00000082512	2384	1750	2121	1689	4224	4690	3185	4990	1890	1545	8970	1223	1044	901	980	1199	604	634	676	1195	7059	3200	4446	2989	1660	2272	2004	5251	478	980	711	1086
ENSG00000153363	103	74	75	74	195	184	139	203	114	103	420	65	184	210	189	273	44	49	40	129	348	172	166	190	111	148	126	297	142	179	218	225
ENSG00000236809	1	1	1	0	0	5	2	3	8	8	39	6	0	6	5	0	1	0	0	3	15	2	4	2	6	11	1	18	0	2	1	0
ENSG00000238137	1	0	0	0	1	0	0	5	0	1	7	0	0	0	1	1	0	0	1	0	1	3	0	0	1	0	1	1	0	0	0	1
ENSG00000198570	0	1	0	0	28	22	26	32	0	0	0	0	0	0	0	0	0	0	0	0	26	6	18	7	0	0	1	2	2	0	0	1
ENSG00000223649	0	0	0	0	0	0	0	0	0	0	0	0	0	0	0	0	0	0	0	0	0	0	0	0	0	0	0	0	0	0	0	0
ENSG00000170385	813	752	585	674	662	786	543	776	658	590	3615	529	624	656	767	765	261	436	365	721	1698	717	1290	749	1109	1503	1308	2651	734	875	833	916
ENSG00000261314	0	0	0	0	0	0	2	3	1	1	2	0	0	0	0	0	0	3	0	0	0	0	2	0	0	0	2	0	0	0	0	1
ENSG00000228792	0	0	0	0	0	0	0	0	0	0	0	0	0	0	0	0	0	0	0	0	0	0	0	0	0	0	0	0	0	0	0	0
ENSG00000227764	0	0	0	0	0	0	0	0	0	0	0	0	0	0	0	0	0	0	0	0	0	0	0	0	0	0	0	0	0	0	0	0
ENSG00000117650	1363	906	1273	947	960	1075	725	1089	882	730	3047	608	635	591	675	833	839	974	1164	875	1854	749	1316	679	1147	1585	349	2542	495	539	745	700
ENSG00000224763	16	10	10	5	6	6	3	6	9	2	17	3	0	4	1	7	3	2	7	6	14	9	11	1	4	2	1	23	0	0	3	3
ENSG00000231057	2	5	3	5	0	0	0	0	3	0	12	0	2	0	2	1	5	4	8	2	0	0	0	0	0	1	5	1	5	5	0	1
ENSG00000226868	0	2	0	0	0	0	0	0	0	0	0	0	0	0	0	0	0	0	0	0	0	0	0	0	0	0	0	0	0	0	0	0
ENSG00000123684	2635	2277	2376	2099	2412	2508	1886	2539	1513	1403	5804	1083	1204	1184	1304	1433	767	1312	1294	1952	3993	1632	2871	1495	1587	2466	1310	4324	801	1306	1026	1326
ENSG00000241395	0	3	1	1	4	0	1	2	1	0	4	1	1	0	0	0	0	0	0	2	2	3	0	2	3	0	1	4	1	0	3	0
ENSG00000212205	7	5	4	3	8	7	7	3	6	5	19	5	1	3	1	1	1	0	2	3	13	10	7	10	5	2	6	18	0	4	0	1
ENSG00000229258	0	0	0	0	0	0	0	0	0	0	0	0	0	1	0	1	1	2	0	1	0	0	0	0	1	0	0	0	0	0	0	1
ENSG00000233626	0	0	6	1	0	3	2	3	1	0	12	0	0	0	0	4	1	3	3	5	5	0	3	2	3	4	2	5	0	0	0	4
ENSG00000143493	1413	1159	1514	1167	1138	1222	1053	1331	1194	929	4084	754	1078	987	1160	1224	1528	2046	1970	1649	3368	1269	2519	1316	1188	1847	856	3190	953	1078	1200	1348
ENSG00000212187	0	2	3	0	0	0	0	2	1	2	3	0	0	0	1	0	0	0	0	0	1	0	0	0	1	2	1	2	2	0	1	0
ENSG00000143476	4039	2865	3252	2628	2009	2051	1708	2315	2715	2278	7696	1648	2577	2015	2218	2665	1902	2473	2514	2440	4679	1724	3613	1601	2459	3588	1320	7098	1856	2351	1817	2558
ENSG00000220749	20	8	10	15	6	4	7	9	23	10	60	8	14	12	14	22	19	23	38	26	31	8	20	21	22	31	8	40	22	18	20	21
ENSG00000264358	6	1	5	0	4	0	0	1	2	1	5	0	0	0	1	7	2	2	1	8	9	1	0	1	4	4	0	4	5	3	1	3
ENSG00000252879	4	4	1	0	3	0	0	0	4	3	2	7	1	1	1	2	3	0	0	0	4	2	1	0	2	1	2	5	0	2	0	1
ENSG00000229983	5	10	6	5	0	0	0	1	9	4	12	1	2	0	0	2	0	0	1	2	0	1	0	0	6	5	0	10	3	0	0	0
ENSG00000226251	11	2	9	1	8	7	10	14	2	4	3	0	0	0	0	0	4	5	1	1	14	4	11	5	2	2	3	3	0	0	0	0
ENSG00000066027	1753	1207	1622	1224	1997	2111	1522	2088	1448	1155	5789	922	1281	1183	1303	1409	960	1356	1273	1353	3715	1481	2987	1385	1562	2164	1347	3767	1091	1125	1185	1324
ENSG00000230063	8	1	2	4	3	4	0	2	0	3	8	0	3	4	3	0	1	2	0	4	8	3	3	5	1	4	4	17	0	0	0	0
ENSG00000234915	6	6	2	7	3	5	2	5	4	1	6	6	3	0	3	2	0	0	1	8	1	2	2	5	5	3	11	13	0	0	1	0
ENSG00000225338	3	3	9	5	3	3	2	5	0	2	6	0	2	3	0	1	2	4	0	2	4	3	2	2	10	3	5	14	1	3	0	1
ENSG00000201544	4	10	4	10	2	2	2	14	2	8	14	6	2	0	1	4	0	1	1	8	18	9	4	2	4	8	7	11	1	3	3	5
ENSG00000229832	11	9	11	5	8	17	7	11	9	15	38	5	5	1	7	5	4	8	4	17	15	13	13	10	7	11	2	11	4	5	6	6
ENSG00000065600	1005	752	865	769	958	895	651	882	121	126	432	75	397	347	444	455	946	1380	1101	990	1952	755	1257	635	130	173	124	294	535	492	648	573
ENSG00000117691	675	551	557	558	547	585	295	550	288	278	830	221	291	292	262	360	328	514	601	650	673	307	530	239	182	339	188	532	242	287	234	347
ENSG00000224535	0	0	1	0	0	0	0	0	1	0	0	0	0	0	0	0	0	0	0	0	0	0	0	0	0	0	0	0	0	0	0	0
ENSG00000228067	2	0	0	0	0	0	0	0	0	0	0	0	0	0	0	0	0	0	0	1	0	0	1	0	0	0	0	0	0	0	0	0
ENSG00000260805	38	32	31	25	7	5	8	12	0	0	5	4	8	7	5	6	9	13	11	24	12	13	10	6	0	2	3	4	4	3	3	8
ENSG00000162772	337	363	297	398	333	349	245	367	15	15	195	31	423	498	367	754	118	242	124	239	391	170	327	171	5	8	15	143	69	157	137	210
ENSG00000235862	0	0	0	0	0	0	0	0	0	0	0	0	0	0	0	0	1	0	0	0	0	0	0	0	0	0	0	0	0	0	0	0
ENSG00000162771	0	0	0	0	0	1	0	0	0	0	0	1	0	0	0	0	0	0	0	0	0	0	0	1	0	0	0	0	0	0	0	0
ENSG00000226036	0	0	0	0	0	0	0	0	0	1	0	0	0	0	0	0	0	0	0	0	0	0	0	1	0	0	0	0	0	0	0	0
ENSG00000237980	0	0	2	0	5	2	2	2	0	0	0	0	0	0	0	0	0	0	1	0	1	0	0	0	0	0	0	1	0	1	0	0
ENSG00000123685	545	438	513	552	489	563	367	505	322	286	704	229	196	165	197	206	379	824	600	925	951	472	746	384	137	406	54	428	157	209	200	226
ENSG00000117697	1440	912	1146	892	1164	1089	917	1185	695	513	2019	426	499	507	528	670	1364	1988	1631	1231	2776	1039	2214	1015	567	940	461	1469	694	657	743	821
ENSG00000203705	824	464	571	479	409	357	286	366	263	204	801	139	265	253	272	307	706	878	743	728	810	314	579	353	251	372	177	526	210	238	216	280
ENSG00000236905	6	8	3	0	1	3	1	1	4	2	7	1	1	0	0	2	1	2	0	7	1	0	0	1	0	0	1	6	0	0	2	3
ENSG00000185523	5	1	3	4	8	5	1	1	1	4	20	3	6	2	3	5	1	4	5	10	12	1	2	0	2	3	2	13	4	1	5	4
ENSG00000198468	104	84	72	60	103	123	83	94	140	114	399	73	115	101	84	140	43	50	43	66	138	64	135	69	110	143	85	261	69	77	100	119
ENSG00000235182	0	0	0	0	0	0	0	0	0	0	0	0	0	0	0	0	0	0	0	0	0	0	0	0	0	0	0	0	0	0	0	0
ENSG00000236317	8	4	11	5	2	2	3	3	7	3	10	2	3	2	1	2	2	1	0	3	3	6	1	1	1	6	3	6	1	1	2	2
ENSG00000162769	1411	901	949	908	1796	1641	1258	1798	597	484	1890	322	699	700	634	832	364	566	357	815	2218	1013	1415	949	463	664	347	1189	463	602	469	738
ENSG00000282718	0	0	0	0	0	0	0	0	0	0	2	0	0	0	0	0	0	0	0	0	0	0	0	0	0	0	2	0	0	0	0	0
ENSG00000143494	1726	1313	1495	1293	1034	908	696	1017	116	129	392	93	35	41	24	40	319	463	426	828	1049	409	686	427	72	124	65	235	8	22	16	34
ENSG00000174606	1318	915	1005	1001	693	764	490	795	1011	935	4634	642	856	867	839	1061	561	766	695	1073	1219	538	856	525	968	1362	1217	2799	587	794	667	901
ENSG00000136643	509	350	377	353	368	441	295	481	360	231	1403	231	234	213	214	331	417	556	393	531	839	380	601	338	405	488	361	1004	245	316	289	385
ENSG00000228646	0	0	0	0	0	0	0	0	0	0	0	0	0	0	0	0	0	0	0	0	0	0	0	0	0	0	0	0	0	0	0	0
ENSG00000225233	0	0	0	0	0	0	0	0	0	0	0	0	0	0	0	0	0	0	0	0	0	0	0	0	0	0	0	0	0	0	0	0
ENSG00000228255	0	0	0	0	0	0	0	0	0	0	0	0	0	0	0	0	0	0	0	0	0	0	0	0	0	0	0	0	0	0	0	0
ENSG00000230461	72	76	63	80	0	0	0	0	0	0	0	0	0	1	0	0	21	39	35	45	0	0	0	0	0	0	1	0	0	0	0	0
ENSG00000281664	5	9	4	3	0	0	0	0	0	0	0	0	0	0	0	0	0	1	2	0	0	0	0	0	0	0	0	0	0	0	0	0
ENSG00000117707	264	303	342	328	0	0	0	1	0	2	3	0	0	9	1	0	44	72	70	136	0	0	2	0	0	0	1	0	1	2	1	2
ENSG00000274895	14	20	25	13	0	0	0	0	0	0	0	0	0	0	0	0	1	1	5	7	0	0	0	0	2	0	0	0	0	0	0	0
ENSG00000272167	0	0	1	0	0	0	0	0	0	0	0	0	0	0	0	0	0	0	0	0	0	0	0	0	0	0	0	0	0	0	0	0
ENSG00000283518	0	0	0	0	1	1	0	0	1	0	1	0	1	0	0	0	0	0	0	1	0	0	0	0	0	0	1	0	0	0	0	0
ENSG00000143499	1721	1477	1432	1470	2067	2113	1542	2311	1598	1297	4716	951	953	1026	974	1304	1257	2343	1598	1770	3297	1234	2305	1201	1338	2054	957	2980	1016	1196	949	1350
ENSG00000228470	2	4	6	4	0	6	5	9	9	9	61	11	3	3	1	2	2	0	0	2	8	4	4	7	8	13	6	33	1	3	2	3
ENSG00000152104	620	741	752	693	2191	2862	1699	3151	6991	5523	40693	5090	514	564	455	691	94	221	190	435	1877	1029	1472	825	6460	9811	8745	23944	105	225	99	221
ENSG00000213036	0	0	1	2	2	2	0	1	6	8	36	1	0	0	0	0	1	0	0	2	1	1	0	1	9	9	2	24	0	0	0	0
ENSG00000215819	0	0	0	0	0	4	0	2	3	2	15	1	1	0	0	2	0	0	0	0	0	3	1	0	2	6	6	11	0	0	0	0
ENSG00000117724	11895	9111	10478	9390	12345	13529	9034	14106	13739	11961	41711	8369	7411	7456	6903	9852	6837	7023	7658	8920	28382	10881	17276	9258	14216	22562	5075	35533	4874	7306	6277	9005
ENSG00000250536	1	3	5	3	1	2	3	4	5	3	16	5	0	0	3	5	3	1	3	12	7	7	6	8	5	11	2	26	0	11	0	4
ENSG00000224584	1	3	2	2	0	1	1	4	1	0	10	0	1	1	0	2	0	0	0	1	4	3	0	8	3	3	1	8	4	0	1	1
ENSG00000185221	0	0	0	0	0	0	0	0	0	0	0	0	0	0	0	0	0	0	0	0	0	0	0	0	0	0	0	0	0	0	0	0
ENSG00000223869	0	0	0	0	0	0	0	0	0	0	0	0	0	0	0	0	0	0	0	0	0	0	0	0	0	0	0	0	0	0	0	0
ENSG00000082482	0	0	0	0	0	0	0	0	0	0	0	0	0	0	0	0	0	0	0	0	0	0	0	0	0	0	0	0	0	0	0	0
ENSG00000232809	0	0	0	0	0	0	0	0	0	0	1	0	0	0	0	1	0	0	0	0	0	0	0	0	0	0	0	0	0	0	0	0
ENSG00000282265	0	0	0	0	0	0	0	0	0	0	0	0	0	0	0	0	0	0	0	0	0	0	0	0	0	0	0	0	0	0	0	0
ENSG00000136636	891	846	666	757	1082	1216	831	1269	2435	2022	6716	1424	794	915	875	1062	611	1144	675	1124	2166	890	1530	808	1812	2887	937	4287	657	1003	804	1028
ENSG00000042781	0	1	2	3	2	9	4	14	21	4	29	4	1	4	4	6	3	2	0	5	5	5	4	4	10	17	17	28	0	8	1	1
ENSG00000202498	0	0	0	0	0	0	0	0	0	0	0	0	0	0	0	0	0	0	0	0	0	0	0	0	0	0	2	0	1	0	0	1
ENSG00000229242	0	0	0	0	0	0	0	0	0	0	0	0	0	0	0	0	0	0	0	0	0	0	0	0	0	0	1	0	0	0	0	0
ENSG00000233620	0	0	0	0	0	0	0	0	0	0	0	0	0	0	0	0	0	0	0	0	0	0	0	0	0	0	0	0	0	0	0	0
ENSG00000236292	0	0	0	0	0	0	0	0	0	0	0	0	0	0	0	0	0	0	0	0	0	0	0	0	0	0	0	0	0	0	0	0
ENSG00000226013	0	0	0	0	0	0	0	0	0	0	0	0	0	0	0	0	0	0	0	0	0	0	0	0	0	0	0	0	0	0	0	0
ENSG00000196482	0	0	0	0	0	0	0	0	0	0	9	0	0	0	0	0	0	0	0	0	0	0	0	0	0	2	0	0	0	0	0	2
ENSG00000092978	662	463	487	442	294	306	267	372	343	291	1483	232	353	261	354	393	410	540	471	525	708	320	529	280	359	530	360	956	268	354	335	385
ENSG00000162814	5	13	12	6	1	0	0	3	1	7	4	3	5	19	24	22	16	18	16	24	1	0	2	0	1	2	1	4	11	16	10	19

ENSG00000234070	0	0	0	0	0	0	0	0	0	0	0	0	0	0	1	0	0	0	0	0	0	0	0	0	0	0	0	0	1	0	2	0
ENSG00000228247	0	0	0	2	0	0	1	0	0	0	1	0	0	2	3	2	0	0	0	1	3	1	3	1	0	1	0	1	0	0	0	1
ENSG00000231814	0	0	0	0	0	0	0	0	0	0	0	0	0	0	0	0	0	0	0	0	0	0	0	0	0	0	0	0	0	0	0	0
ENSG00000230714	0	0	0	0	0	0	0	0	0	0	0	0	0	0	0	0	0	0	0	0	0	0	0	0	0	0	0	0	0	0	0	0
ENSG00000232100	0	0	0	0	0	0	0	0	0	0	2	0	0	0	0	0	0	0	0	0	0	0	0	0	0	0	0	0	0	0	0	0
ENSG00000201493	0	0	0	0	0	0	0	0	0	0	0	0	0	0	0	0	0	0	0	0	0	0	0	0	0	0	1	0	0	0	0	0
ENSG00000067533	942	684	810	735	1291	1287	996	1358	748	568	2323	379	789	692	876	920	1050	1398	1340	1428	3188	1259	2288	1300	703	1023	486	1581	908	937	1059	1123
ENSG00000229016	1	0	0	0	1	1	0	1	1	0	0	0	1	0	0	0	0	0	0	0	0	1	3	3	2	2	0	2	0	3	0	0
ENSG00000223375	0	0	0	1	0	0	0	0	1	0	3	0	2	1	0	0	0	0	0	0	0	2	0	0	2	3	0	4	0	1	0	1
ENSG00000232480	0	0	0	0	6	13	14	22	1	0	0	0	0	0	2	0	2	0	1	0	12	11	25	7	1	0	3	3	0	0	4	6
ENSG00000092969	0	4	2	2	84	104	66	83	0	0	7	0	1	1	0	1	3	5	2	5	110	50	90	46	1	0	2	2	1	1	3	1
ENSG00000281453	0	0	0	0	0	0	0	0	0	0	0	0	0	0	0	0	0	0	0	0	0	0	0	0	0	0	0	0	0	0	0	0
ENSG00000228208	0	0	0	0	0	0	0	0	0	0	1	0	0	0	0	0	0	2	0	0	1	0	0	2	0	0	0	0	1	0	0	0
ENSG00000212610	0	0	0	0	0	0	0	0	0	0	0	0	0	0	0	0	0	0	0	0	0	0	0	0	0	0	0	0	1	0	0	0
ENSG00000226693	0	0	0	0	0	0	0	0	0	0	0	0	0	0	0	0	0	0	0	0	1	1	0	0	0	0	0	0	0	0	0	0
ENSG00000225561	0	0	0	0	0	0	0	0	0	0	0	0	0	0	0	0	0	0	0	0	0	0	0	0	0	2	0	0	0	0	0	0
ENSG00000228063	12	1	6	8	5	9	5	3	3	6	8	1	2	0	0	3	10	7	15	6	15	12	20	5	1	3	3	1	0	1	0	2
ENSG00000143353	469	332	376	348	268	266	195	257	286	225	979	162	277	263	301	326	548	679	575	660	595	271	418	328	232	277	217	536	299	338	371	397
ENSG00000225920	51	32	51	56	46	46	36	42	31	25	163	22	28	15	12	23	5	10	9	38	44	27	28	28	25	30	30	75	4	24	14	27
ENSG00000223842	6	3	7	5	5	7	3	5	3	4	22	2	1	5	4	3	2	2	2	2	6	0	0	2	2	5	7	6	2	3	3	3
ENSG00000277007	18	20	27	22	12	14	9	18	17	13	61	10	8	15	10	14	7	2	1	10	12	6	5	11	11	10	7	26	4	9	2	5
ENSG00000228536	0	1	0	3	3	3	2	4	1	0	1	2	0	0	0	0	0	0	0	1	4	0	2	2	1	0	1	2	0	0	0	0
ENSG00000230024	3	4	0	2	5	8	3	5	0	1	1	0	0	1	0	0	1	0	1	1	8	3	3	4	1	0	0	0	0	0	0	1
ENSG00000238232	0	0	0	0	0	0	0	1	0	0	0	0	0	0	0	0	0	0	0	0	0	0	1	0	0	0	0	0	0	0	0	1
ENSG00000215817	6	1	5	6	22	24	16	37	0	0	5	0	0	1	0	0	1	2	2	2	58	39	47	22	0	0	0	0	0	0	0	1
ENSG00000196660	0	1	0	0	25	56	43	62	0	0	0	0	0	0	0	0	1	0	0	0	95	65	111	70	0	0	0	1	0	0	0	0
ENSG00000221673	0	0	0	0	0	0	0	0	0	0	0	0	0	0	0	0	0	0	0	0	0	0	0	0	0	0	0	0	0	0	0	0
ENSG00000136628	6156	4160	4566	4139	4470	4807	3365	5075	5009	3561	13864	2605	5334	4432	4623	5545	2916	4820	4080	4189	8194	3223	6262	2663	4488	6629	3073	9840	4182	4143	4439	4307
ENSG00000162813	1398	772	1129	770	688	754	567	837	620	494	2443	406	767	782	950	956	1397	1818	1903	1545	1570	637	1293	575	711	861	578	1440	1184	1108	1397	1231
ENSG00000067704	4430	2850	3688	2876	3703	3659	2683	3999	2817	2006	8079	1483	4298	3470	3760	4399	3510	4667	4231	3536	6941	2533	4911	2324	2633	3798	1789	5242	3723	3860	3730	4318
ENSG00000207590	9	3	2	5	1	4	3	5	2	2	5	0	1	3	1	7	0	3	1	2	6	3	3	3	3	3	0	9	1	9	2	4
ENSG00000207624	1	1	3	0	1	0	1	5	0	2	8	0	1	0	3	0	2	0	0	1	4	1	0	1	2	2	4	6	0	1	0	1
ENSG00000232134	7	5	3	1	6	4	1	6	3	6	6	0	3	0	2	1	3	0	1	0	5	3	0	2	0	5	4	4	1	5	1	4
ENSG00000118873	1865	1326	1536	1218	1942	1922	1493	2094	2041	1598	7309	1170	2157	1947	2020	2354	583	775	739	1267	3242	1345	2456	1345	1747	2479	1877	4797	1224	1708	1373	1839
ENSG00000281696	0	1	3	1	1	0	0	0	0	0	1	0	0	0	0	0	0	1	0	0	1	1	0	0	0	0	0	0	0	0	0	1
ENSG00000222370	3	3	1	4	0	1	2	0	1	0	4	0	7	0	0	3	0	0	0	2	0	3	0	2	1	1	2	3	0	1	0	2
ENSG00000270585	18	21	12	23	9	9	11	23	13	13	31	6	16	10	15	14	8	3	0	19	21	7	12	14	9	30	12	36	1	14	8	21
ENSG00000270708	2	4	0	0	2	1	0	0	0	1	3	1	3	0	5	1	0	0	1	1	1	1	3	0	6	3	1	9	0	1	1	1
ENSG00000218283	300	183	246	225	396	421	334	476	253	140	680	139	144	145	133	174	310	303	260	228	924	382	690	354	185	257	191	471	146	146	163	182
ENSG00000213033	6	4	1	0	4	11	9	5	4	8	24	2	1	1	3	1	0	1	0	13	22	2	8	5	6	10	1	17	0	7	5	2
ENSG00000230318	0	0	0	0	0	0	0	0	0	0	0	0	0	0	0	0	0	0	0	0	1	0	0	0	0	0	0	0	0	0	0	0
ENSG00000232883	0	0	0	0	0	0	0	0	0	0	2	0	0	0	0	0	0	0	0	0	0	0	0	0	0	0	0	0	0	0	0	0
ENSG00000226927	0	0	0	0	0	0	0	0	0	0	0	0	0	0	0	0	0	0	0	0	0	0	0	0	0	0	0	0	0	0	0	0
ENSG00000260505	0	0	0	0	0	0	0	0	0	0	0	0	0	0	0	0	0	0	0	0	0	0	0	0	0	0	0	0	0	0	0	0
ENSG00000235163	0	0	0	0	0	0	0	0	0	0	0	0	0	0	0	0	0	0	0	0	0	0	0	0	0	0	0	0	0	0	0	0
ENSG00000234863	0	0	0	0	0	0	0	0	0	0	0	0	0	0	0	0	0	0	0	0	0	0	0	0	0	0	0	0	0	0	0	0
ENSG00000226226	0	0	0	0	0	0	0	0	0	0	2	0	0	0	0	0	0	0	0	0	0	0	0	0	0	0	0	0	0	0	0	0
ENSG00000231529	0	0	0	0	0	0	0	0	0	0	0	0	0	0	0	0	0	0	0	0	0	0	0	0	0	0	0	0	0	0	0	0
ENSG00000116141	0	0	0	1	1	0	0	0	0	4	17	4	6	9	5	12	0	0	0	0	0	0	0	0	1	3	5	12	1	7	2	10
ENSG00000243872	0	0	0	0	0	0	0	0	0	0	0	0	0	0	1	0	0	0	0	0	0	0	0	0	0	0	0	0	0	0	0	0
ENSG00000233012	0	0	0	0	0	0	0	0	0	0	0	0	0	0	0	0	0	0	0	0	0	0	0	0	0	0	0	0	0	1	3	0
ENSG00000162817	0	0	0	0	0	0	0	0	1	8	8	2	11	31	4	35	0	0	0	0	0	0	0	0	4	10	1	11	0	7	2	4
ENSG00000117791	0	0	0	0	0	0	0	0	27	15	73	12	0	0	0	0	0	0	0	0	0	0	0	0	33	38	26	69	0	0	0	0
ENSG00000186205	0	0	0	0	0	0	0	0	2	0	4	0	14	26	14	36	0	0	0	0	1	0	0	1	4	0	0	0	1	14	3	4
ENSG00000221571	0	0	0	0	0	0	0	0	0	0	0	0	0	0	0	0	0	0	0	0	0	0	0	0	0	0	0	0	0	0	0	0
ENSG00000276642	0	0	0	0	0	0	0	0	0	0	0	0	0	0	0	0	0	0	0	0	0	0	0	0	0	0	0	0	0	0	0	0
ENSG00000272823	0	0	0	0	0	0	0	0	4	3	1	0	0	0	0	0	0	0	0	0	0	0	2	0	0	0	0	0	0	0	0	0
ENSG00000238078	0	0	0	0	4	5	8	0	6	6	8	1	0	1	1	0	0	0	0	0	7	3	2	1	0	0	0	0	0	0	0	0
ENSG00000257551	0	0	0	0	0	0	0	0	0	2	3	0	0	0	1	0	0	0	0	0	3	0	4	0	0	0	0	0	0	1	2	0
ENSG00000136630	0	0	0	0	55	61	62	63	159	121	308	95	42	35	38	47	0	0	0	0	103	31	61	38	15	15	3	13	12	23	13	21
ENSG00000226211	0	0	0	0	0	0	0	0	0	0	0	0	0	0	0	0	0	0	0	0	0	0	0	0	0	0	0	0	0	0	0	0
ENSG00000234754	0	0	0	0	0	0	0	1	0	0	0	0	0	0	0	1	0	0	0	0	0	0	0	0	0	0	0	0	0	0	0	0
ENSG00000232436	0	0	0	0	0	0	0	0	0	0	0	0	0	0	0	0	0	0	0	0	0	0	0	0	0	0	0	0	0	0	0	0
ENSG00000227585	0	0	1	0	0	0	0	0	0	0	0	0	0	0	0	0	0	0	0	0	0	0	0	0	0	0	0	0	0	0	0	0
ENSG00000143507	116	88	104	103	701	806	606	846	515	480	2267	333	1003	917	1188	1298	19	26	42	41	1411	693	1233	664	478	717	606	1495	597	928	771	1001
ENSG00000227925	0	0	0	0	4	1	0	0	0	0	2	0	0	0	0	2	0	0	0	1	2	0	1	0	0	0	2	2	0	0	1	0
ENSG00000200033	0	0	0	0	1	1	1	3	0	0	0	0	0	0	0	0	0	0	0	0	1	0	5	0	0	0	0	0	0	0	0	0
ENSG00000238042	0	0	0	0	8	4	3	11	0	0	0	0	1	1	0	0	0	0	0	0	13	2	4	2	0	0	0	0	0	0	0	1
ENSG00000228437	0	0	0	0	0	0	0	1	0	0	0	0	0	0	0	0	0	0	0	0	0	1	0	0	0	0	0	0	0	0	0	0
ENSG00000232679	0	0	0	2	0	0	0	0	0	0	0	0	1	0	0	0	0	0	0	0	0	0	0	0	0	0	0	0	0	0	1	0
ENSG00000236230	0	0	0	0	2	1	2	0	0	1	2	0	0	0	1	0	0	0	0	0	0	0	0	0	0	0	0	0	0	1	0	0
ENSG00000227613	0	0	0	0	0	0	0	0	0	0	0	0	0	0	0	0	0	0	0	0	0	0	0	0	0	0	0	0	0	0	0	0
ENSG00000226643	0	0	0	0	0	0	1	0	0	0	0	0	2	0	1	0	0	0	0	0	0	3	0	0	0	0	0	1	0	0	0	0
ENSG00000234419	0	0	0	0	0	1	0	1	1	0	1	0	1	0	0	0	0	0	0	0	0	0	0	0	0	0	0	1	0	5	0	0
ENSG00000276997	15	23	21	17	21	13	17	17	21	13	49	4	15	20	10	34	3	0	4	30	25	17	17	16	12	11	15	38	5	21	10	20
ENSG00000222399	0	0	0	0	0	0	0	0	0	0	0	0	0	0	0	0	0	0	0	0	0	0	0	0	0	0	0	0	0	0	0	0
ENSG00000267305	3	1	1	6	0	0	0	0	0	3	3	0	0	3	1	1	0	0	0	3	0	0	0	0	0	0	1	3	0	2	0	1
ENSG00000143512	6	2	5	5	13	4	6	9	10	4	31	7	7	5	13	10	3	5	1	7	16	14	18	20	4	6	3	16	6	15	9	13
ENSG00000143498	480	306	389	311	282	336	290	360	358	244	722	167	400	313	387	428	475	597	512	539	914	314	579	328	346	492	111	559	424	431	528	473
ENSG00000225265	32	18	33	24	9	12	14	14	34	16	83	21	41	27	29	42	22	30	37	41	47	23	26	24	15	15	11	56	38	44	74	74
ENSG00000154305	2434	1813	1901	1762	2212	2426	1874	2471	1452	1323	5687	992	1524	1518	1584	2003	819	803	693	1380	3741	1588	2522	1557	1564	2448	1637	4207	886	1555	1044	1666
ENSG00000229399	6	5	9	7	1	4	5	3	1	5	6	1	1	0	0	8	1	2	1	14	9	1	3	5	5	5	2	4	1	2	3	4
ENSG00000272750	56	27	37	34	54	65	54	57	39	34	149	18	37	39	23	33	21	10	9	38	63	39	35	63	44	63	37	110	17	23	32	36
ENSG00000186063	1571	1109	1276	1155	1019	1036	827	1086	961	827	3256	626	1055	927	979	1193	1023	1425	1151	1070	2062	775	1614	764	1146	1476	771	2322	1067	1095	1114	1177
ENSG00000162819	954	639	775	557	1071	1156	956	1240	741	652	3052	465	1316	1104	1315	1518	593	702	741	703	2198	892	1695	997	734	1057	856	2132	1039	1219	1302	1336
ENSG00000197520	0	0	4	5	4	8	6	7	1	0	3	2	1	1	0	1	0	2	2	2	7	0	0	0	0	0	1	7	0	0	2	2
ENSG00000228106	90	72	78	103	113	108	58	107	66	50	269	50	59	74	67	89	97	160	148	174	227	119	175	151	68	100	62	172	100	100	129	132
ENSG00000278610	0	3	0	1	3	0	3	0	0	0	5	1	0	1	0	1	0	0	0	2	2	1	1	6	1	1	0	2	1	3	1	3
ENSG00000154309	55	65	67	71	75	102	98	116	37	39	209	50	64	65	62	96	68	86	65	72	198	74	167	89	45	44	54	142	39	71	47	94
ENSG00000234523	0	2	1	4	1	0	2	1	3	2	6	0	1	0	0	2	0	0	1	0	3	3	2	0	0	1	1	4	0	1	0	2
ENSG00000187554	46	46	41	51	11	24	8	21	0	2	3	0	191	214	205	302	25	29	38	45	15	10	16	4	0	0	0	2	131	136	121	177
ENSG00000236846	1	0	0	0	0	1	0	1	0	0	0	0	4	1	3	3	1	1	0	0	5	0	3	2	0	0	0	0	5	5	10	7
ENSG00000226601	0	0	0	0	0	0	0	0	0	0	0	0	0	0	1	1	0	0	0	0	0	0	0	0	0	0	0	0	2	1	2	2
ENSG00000143502	474	415	440	386	422	520	320	498	0	2	6	1	620	590	632	696	215	327	310	473	676	254	531	284	2	2	7	13	771	944	771	1134
ENSG00000251789	0	0	0	0	0	0	0	0	0	0	0	0	0	0	0	0	0	0	0	0	0	0	0	0	0	0	0	0	0	0	0	0
ENSG00000178395	0	0	0	0	0	0	0	0	0	0	0	0	0	0	0	0	0	0	0	0	0	1	0	0	0	0	0	0	0	0	0	0
ENSG00000203697	0	0	0	0	0	0	0	0	10	3	23	0	0	1	0	1	0	2	0	3	0	0	1	0	3	4	2	5	0	2	0	2
ENSG00000227002	0	0	0	0	0	0	0	0	0	0	0	0	0	0	0	0	0	0	0	0	0	0	0	0	0	0	0	0	0	0	0	0
ENSG00000212398	0	0	0	0	0	0	0	0	0	0	1	0	0	0	0	1	0	0	0	0	0	0	0	0	0	0	0	0	0	0	0	0
ENSG00000162909	22	14	24	24	1442	1495	1037	1542	2491	2069	11437	1895	2144	2152	1987	2746	8	4	11	31	1827	790	1363	656	2011	3340	2368	6356	1565	2005	1439	2134
ENSG00000143514	1734	1565	1458	1504	1474	1452	1136	1472	1193	1030	4890	858	1131	1096	1307	1543	620	1017	953	1387	2776	1084	2017	952	1267	1902	1260	3440	861	1146	950	1129
ENSG00000227621	0	0	0	0	0	0	0	0	0	0	1	0	0	1	1	1	0	0	0	0	0	0	0	0	0	0	0	1	0	0	0	1
ENSG00000188460	0	1	0	0	0	0	0	0	0	0	0	0	0	0	0	0	0	0	0	0	0	0	0	0	0	0	0	0	0	0	0	0
ENSG00000233771	2	2	4	1	0	4	1	0	2	1	3	1	1	1	2	3	2	0	4	6	3	3	0	2	5	0	0	4	0	0	3	8
ENSG00000272645	466	391	510	366	189	264	202	239	288	261	1098	173	254	254	236	287	165	124	111	393	376	207	204	195	156	242	249	560	101	236	162	300
ENSG00000212157	1	0	0	0	1	0	0	1	0	1	1	0	0	1	0	0	0	0	0	0	0	0	0	0	0	0	0	5	0	2	1	0
ENSG00000185495	250	187	213	157	143	145	92	183	259	228	1426	157	308	269	296	323	42	55	78	236	237	118	128	144	227	314	338	870	77	244	119	228
ENSG00000278467	29	20	20	23	27	29	7	25	21	22	139	9	38	24	35	42	4	3	6	31	31	16	17	16	34	42	36	139	11	27	11	31
ENSG00000229930	9	9	9	16	1	6	4	11	5	2	30	5	0	1	1	0	6	7	2	12	8	5	1	3	3	7	7	14	0	1	0	1
ENSG00000252484	0	0	0	0	0	0	0	0	0	0	0	0	0	0	0	0	0	1	0	0	0	0	0	0	0	1	0	2	0	0	0	0
ENSG00000143756	1069	851	944	838	777	852	622	858	714	637	3201	470	715	673	833	843	743	1210	1126	1142	2038	776	1318	750	834	1179	857	2223	756	778	958	1008
ENSG00000236773	5	1	3	5	1	1	4	0	0	1	6	0	0	3	1	1	2	3	2	5	1	1	0	0	0	0	0	3	0	0	0	0
ENSG00000143753	898	811	790	803	1239	1268	912	1446	1071	810	4396	764	1098	942	1238	1271	346	595	465	571	2423	946	1736	835	1257	1764	1207	3150	1136	1262	1247	1368
ENSG00000201898	0	0	0	0	0	0	1	0	1	0	0	0	1	1	0	0	0	0	0	0	1	0	0	0	0	0	1	0	0	1	0	0
ENSG00000232628	6	3	5	8	6	3	6	6	3	8	31	4	3	2	0	1	0	0	1	3	3	3	1	0	7	5	12	13	1	2	1	2
ENSG00000237101	11	18	17	10	1	3	5	8	4	9	39	6	1	4	2	2	4	5	0	12	4	6	4	5	5	4	8	19	3	2	6	4
ENSG00000143748	1069	710	940	739	883	944	766	1073	748	571	2639	506	835	669	1000	904	808	1050	1069	931	2365	957	1864	897	757	1095	617	2004	969	1028	1284	1261
ENSG00000221406	9	4	7	12	4	7	7	9	8	4	17	2	1	2	7	10	0	0	2	3	8	5	5	6	6	13	3	17	4	11	3	7
ENSG00000229742	4	2	2	0	0	1	0	4	0	1	3	0	1	1	1	1	0	0	2	2	9	2	4	3	1	2	1	7	2	2	2	2
ENSG00000206887	0	1	0	0	0	0	1	1	0	0	2	0	1	0	1	0	0	0	0	4	0	0	0	1	4	0	0	6	0	0	0	0
ENSG00000143771	1348	1062	1083	1123	517	540	326	564	513	431	1558	279	410	401	425	510	940	1442	1104	1354	1114	487	758	569	457	662	340	1073	453	480	441	488
ENSG00000162923	4411	3914	3823	4003	3588	3824	2905	4298	2702	2435	12413	1813	2424	2306	2540	2878	834	1286	1105	2170	4923	2155	3376	2207	2540	3711	3330	7850	1815	2370	2042	2618
ENSG00000266618	1	0	1	2	1	4	2	3	0	2	4	2	0	1	2	0	0	0	0	0	1	0	0	2	3	2	2	10	0	0	1	5
ENSG00000143786	62	67	44	50	162	186	110	205	35	31	209	34	44	50	47	53	13	30	30	47	334	197	277	117	27	46	96	168	21	28	35	46
ENSG00000229991	0	0	1	0	1	0	1	0	0	0	1	0	0	0	0	0	0	0	0	0	1	0	0	0	0	0	0	1	0	0	0	0
ENSG00000233384	2	7	8	6	32	43	35	39	5	1	12	1	9	15	8	17	3	9	5	8	77	35	68	36	2	12	4	11	1	5	4	7
ENSG00000234247	0	0	0	1	5	1	0	1	0	0	2	0	0	1	0	0	0	0	0	1	3	0	1	6	1	1	1	11	0	0	0	0
ENSG00000229400	0	0	0	0	1	4	2	3	0	0	0	2	0	0	0	0	0	0	0	0	6	2	2	2	0	0	0	2	0	0	0	0
ENSG00000225334	0	0	0	0	0	1	3	1	0	0	1	0	0	0	0	0	0	0	0	0	1	3	0	3	0	0	0	3	0	0	0	0
ENSG00000185842	371	337	330	305	760	772	573	841	145	115	513	74	521	515	531	678	304	465	284	527	1620	713	938	761	132	189	103	420	325	593	493	741
ENSG00000143815	7971	5205	6498	5078	7125	6911	5256	7473	4845	4124	15137	2864	4210	3893	4224	5049	5427	7427	6374	6405	16228	6299	11320	5318	5340	7877	2681	10960	3598	4310	3743	4915
ENSG00000234476	7	3	6	7	13	5	4	4	14	10	95	10	1	1	7	7	4	0	2	0	7	4	11	1	11	14	9	31	2	1	3	2
ENSG00000282418	1	0	2	0	1	5	0	1	3	5	37	2	1	0	0	0	0	0	0	1	2	4	1	0	3	5	10	21	0	0	0	2
ENSG00000154380	1170	1295	1133	1391	2498	2547	1726	2553	1452	1335	5715	987	106	266	79	248	294	677	423	1178	4031	1760	2781	1631	1136	2044	954	3378	36	147	41	140
ENSG00000227496	0	0	0	2	1	0	0	0	0	0	3	0	0	1	1	0	0	0	0	0	0	0	0	0	0	0	0	0	0	0	0	0
ENSG00000226349	0	0	0	0	1	0	1	0	0	0	0	0	0	0	0	0	0	0	0	0	0	0	0	0	0	0	0	1	0	0	1	0
ENSG00000223306	0	0	0	0	0	0	0	0	0	0	1	0	0	0	0	0	0	0	0	0	0	0	0	0	0	0	0	0	0	0	0	0
ENSG00000143742	7695	4896	6010	4559	6268	6144	4906	6647	5932	4661	18016	3475	4454	3929	4741	5052	9787	12481	11067	8631	17791	6470	12173	8053	6463	8549	3723	12786	5859	5054	6887	6387
ENSG00000234112	1	1	0	1	1	0	1	0	1	0	3	0	0	0	0	2	0	0	1	0	0	1	0	0	1	0	3	3	0	0	0	0
ENSG00000143819	339	295	402	272	264	317	246	364	151	164	775	132	98	124	119	180	80	132	152	207	436	189	368	193	128	237	341	740	52	106	54	118
ENSG00000242861	64	37	87	70	71	75	57	79	62	59	305	43	32	42	46	81	12	5	13	31	96	34	56	41	33	55	102	153	18	33	22	51
ENSG00000196187	1676	1426	1764	1396	1453	1709	1131	1780	985	936	5262	824	709	675	697	917	376	332	300	934	1934	954	1257	888	736	1338	1490	3545	322	729	444	835
ENSG00000243709	0	1	2	2	0	4	1	1	0	1	3	1	1	0	1	0	1	0	1	1	5	0	0	2	1	1	3	1	1	0	0	5
ENSG00000255835	0	0	0	0	0	0	0	0	0	0	0	0	0	0	0	0	0	0	0	0	0	0	0	0	0	0	0	0	0	0	0	1
ENSG00000143811	2632	2119	2321	2079	1775	1745	1380	2014	2013	1583	7258	1429	1681	1378	1607	1983	866	1379	1216	1888	2579	1062	1968	958	1869	2962	1668	5092	1158	1502	1168	1527
ENSG00000248322	0	0	0	0	0	0	0	0	0	0	0	0	0	0	0	0	0	0	0	0	0	0	0	0	0	0	0	0	0	0	0	0
ENSG00000143768	0	0	0	0	1	0	1	0	0	0	16	1	0	0	0	0	0	0	0	0	0	0	0	0	2	1	35	25	0	0	0	1
ENSG00000213032	0	0	0	0	0	0	0	0	0	0	0	0	0	0	0	0	0	0	0	0	0	0	0	0	0	0	0	0	0	0	0	0
ENSG00000143751	1793	1168	1418	1199	1540	1506	1293	1608	835	713	3484	544	1008	906	1261	1116	657	962	941	1048	3535	1429	2738	1252	953	1410	837	2300	979	1126	1209	1222
ENSG00000272562	24	24	24	14	17	19	10	15	17	24	119	10	19	15	11	22	10	9	8	9	18	9	14	7	12	16	35	54	5	4	3	6
ENSG00000163041	6365	4597	5373	4608	11690	10979	7807	10912	5643	4352	18252	3805	4464	4170	4363	5153	5279	7451	7263	5277	27106	9662	17729	9591	5556	7348	3716	11265	5432	5114	5621	5709
ENSG00000225518	8	18	20	15	0	0	0	0	5	6	3	1	0	0	2	0	5	6	5	14	0	0	1	1	1	4	0	3	0	0	0	0
ENSG00000270598	0	0	0	0	0	0	0	0	0	0	1	0	0	1	0	0	0	0	0	0	0	0	0	0	0	0	0	0	0	1	0	0
ENSG00000182827	1618	1057	1306	1066	1255	1305	972	1299	921	712	3724	581	1067	1021	1291	1236	1145	1362	1347	1263	2644	1022	1959	976	918	1431	919	2375	1151	1256	1483	1375
ENSG00000234478	9	7	10	16	6	9	8	5	3	3	47	8	11	2	9	5	3	4	2	11	17	9	10	18	10	13	8	28	3	8	13	5
ENSG00000223570	1	0	0	0	2	0	1	0	0	0	0	0	2	2	0	0	0	0	0	0	0	0	0	3	0	0	0	0	0	0	0	0
ENSG00000185155	0	5	0	1	5	4	8	5	4	0	4	1	0	1	3	1	0	2	0	3	7	3	5	12	2	1	1	6	0	1	0	2
ENSG00000183814	630	432	470	429	869	886	661	901	445	334	1309	287	348	344	373	401	731	795	759	782	2245	837	1539	1083	540	692	229	1160	360	421	430	470
ENSG00000236994	0	0	0	0	0	0	0	0	0	0	0	0	0	0	0	0	0	0	0	0	0	0	0	0	0	0	0	0	0	0	0	0
ENSG00000275406	0	0	0	0	0	0	0	0	0	0	0	0	0	0	0	0	0	1	0	0	2	0	0	0	0	0	0	0	0	0	0	0
ENSG00000143799	12376	8721	9924	9176	18455	18996	14387	19321	9065	6951	25312	5288	11362	10039	11750	13649	3044	5055	4944	6986	26658	11029	21418	9660	7677	12545	5030	18113	8469	10176	9209	11184
ENSG00000270287	0	0	0	0	0	0	0	0	0	0	0	0	0	0	0	0	0	0	0	0	0	0	0	0	0	0	0	0	0	0	0	0
ENSG00000229965	0	0	0	0	0	0	0	0	0	0	0	0	0	0	0	0	1	0	0	0	0	0	0	0	0	0	0	0	0	0	0	0
ENSG00000223282	0	0	0	0	0	0	0	0	0	0	0	0	0	0	0	0	0	0	0	0	0	1	0	0	0	0	0	0	0	0	0	0
ENSG00000177770	0	0	0	0	0	0	0	0	0	0	0	0	0	0	1	0	0	0	0	0	0	0	0	0	0	0	0	0	0	0	0	0
ENSG00000203685	1	5	3	9	0	2	0	1	11	12	22	2	1	0	0	0	0	0	0	4	3	0	0	1	3	5	8	11	0	0	0	0
ENSG00000143772	1168	995	1255	983	1964	1858	1358	1941	657	593	2767	464	610	606	587	701	286	412	468	787	3368	1409	2570	1282	545	936	689	1657	412	592	435	640
ENSG00000228382	11	1	4	4	7	6	3	0	5	3	18	5	0	4	6	3	4	1	3	5	18	8	10	10	5	8	6	18	3	8	6	5
ENSG00000228548	7	1	4	3	1	3	5	2	9	1	4	0	7	2	3	4	1	1	2	3	5	9	6	5	1	5	0	16	0	2	1	11
ENSG00000238183	0	0	0	0	0	0	0	0	0	1	0	0	0	0	0	0	0	0	0	0	0	0	0	0	0	0	0	0	0	0	0	0
ENSG00000143801	0	0	1	0	81	80	69	84	72	58	498	74	201	180	226	225	0	0	0	0	31	7	19	12	85	145	110	336	178	242	245	309
ENSG00000163050	1429	1183	1160	1243	755	711	562	766	1059	917	4388	810	2191	1977	1690	2328	351	625	428	946	1035	424	748	377	762	1267	948	2491	1241	1595	1116	1610
ENSG00000228525	6	2	3	1	1	2	2	0	1	2	4	0	2	0	3	2	0	0	0	1	4	2	0	1	1	2	2	8	0	1	0	6
ENSG00000143776	2488	2111	2510	2261	0	0	0	2	2918	2653	15128	2210	794	977	743	1198	654	1098	1186	2442	1	0	0	0	3225	4899	4097	11041	441	916	547	1016
ENSG00000233706	0	0	0	0	0	0	0	0	0	0	0	0	0	0	0	0	0	0	0	0	0	0	0	0	0	0	0	0	0	0	0	0
ENSG00000228625	1	2	2	0	0	0	0	0	2	1	8	0	1	0	1	0	2	0	0	15	0	0	0	0	5	9	1	27	0	1	0	5
ENSG00000228729	8	0	2	0	0	0	0	0	4	2	16	1	3	0	2	2	1	0	1	9	0	0	0	0	6	9	5	31	1	3	0	2
ENSG00000236636	1	2	2	0	0	0	0	0	0	1	3	1	0	1	0	0	1	0	0	6	0	0	0	0	2	4	0	17	0	2	0	1
ENSG00000234277	0	0	0	0	0	0	0	0	1	2	0	0	0	0	0	0	0	0	0	1	0	0	0	0	0	0	0	0	1	0	1	0
ENSG00000232864	0	0	0	0	0	0	0	0	0	0	1	0	0	0	0	0	0	0	0	0	0	0	0	0	0	0	0	0	0	0	0	0
ENSG00000242757	0	0	0	0	0	0	0	0	0	0	0	0	0	0	0	0	0	0	0	0	0	0	0	0	0	0	0	0	0	0	0	0
ENSG00000224834	0	0	0	0	0	0	0	0	0	0	0	0	0	0	0	0	0	0	0	0	0	0	0	0	0	0	0	0	1	1	0	0
ENSG00000237193	0	0	0	0	0	0	0	0	0	0	0	0	0	0	0	0	0	0	0	0	0	0	0	0	0	0	0	0	0	0	0	0
ENSG00000278180	0	0	0	0	0	0	0	0	0	0	0	0	0	0	0	0	0	0	0	0	0	0	0	0	0	0	0	0	0	0	0	0
ENSG00000237469	0	0	0	0	0	0	0	0	0	0	0	0	0	0	0	0	0	0	0	0	0	0	0	0	0	0	0	0	0	0	0	0
ENSG00000235827	0	0	0	0	0	0	0	0	0	0	0	0	0	0	0	0	0	0	0	0	0	0	0	0	0	0	0	0	0	0	0	0
ENSG00000227711	0	1	0	0	0	0	0	0	0	0	0	0	0	0	0	0	0	0	0	0	0	0	0	0	0	0	0	0	0	0	0	0
ENSG00000181450	1279	815	905	796	723	818	576	851	296	254	964	171	502	519	545	544	730	724	725	1034	1750	661	1166	786	308	395	214	749	324	395	472	539
ENSG00000225934	8	1	5	0	2	1	0	3	0	0	6	0	0	0	2	0	0	3	1	3	2	4	3	5	4	4	5	13	1	3	2	3
ENSG00000215812	11	7	7	9	4	6	5	4	0	0	1	0	0	0	0	1	0	2	2	7	3	3	3	0	1	0	0	1	0	1	0	2
ENSG00000143740	627	454	609	541	579	597	448	636	550	475	1961	371	463	428	484	583	322	480	472	522	944	374	722	327	379	610	468	1297	383	452	455	453
ENSG00000081692	369	244	327	220	152	166	95	165	174	187	767	173	159	154	206	206	238	393	389	406	268	151	253	148	178	297	155	547	193	202	192	216
ENSG00000230005	2	2	3	1	2	2	3	2	3	0	24	1	2	0	0	1	1	0	0	2	2	6	1	6	8	2	12	20	1	1	7	6
ENSG00000185888	0	0	0	0	0	0	0	0	0	0	0	0	0	0	0	0	0	0	0	0	0	0	0	0	0	0	0	0	0	0	0	0
ENSG00000143816	0	0	0	2	6	18	14	29	18	17	147	23	0	0	0	0	0	0	0	0	18	8	11	7	4	10	12	34	0	0	0	0
ENSG00000264483	0	0	0	0	0	0	0	0	0	0	0	0	0	0	0	0	0	0	0	0	0	0	0	0	0	0	2	2	0	0	0	0
ENSG00000233003	1	0	0	0	0	0	0	0	0	0	3	0	0	1	0	0	0	0	0	0	5	0	0	0	0	1	0	0	0	0	0	0
ENSG00000274886	0	0	0	0	0	0	0	0	0	0	0	0	0	0	0	0	0	0	0	0	0	0	0	0	0	0	0	0	0	0	0	0
ENSG00000154342	0	0	0	0	1	0	0	3	0	0	2	0	0	0	0	0	0	0	0	1	4	2	3	3	0	0	3	2	0	0	0	0
ENSG00000276255	19	19	41	8	19	11	13	20	16	17	64	7	9	18	12	18	7	5	10	47	37	15	26	24	13	29	27	50	4	9	8	14
ENSG00000143761	7173	5172	6386	5148	5586	5745	3833	5653	4863	3805	17167	3129	4827	4185	4501	5647	2172	3735	3887	4393	8106	3307	5860	2707	4486	7464	3888	11410	4049	4046	3809	4454
ENSG00000143793	1288	886	1075	992	623	666	510	702	716	520	1890	468	791	745	838	1030	666	676	693	971	1080	527	1029	459	549	874	453	1378	731	887	779	939
ENSG00000162910	589	395	407	400	430	435	312	445	315	241	866	196	337	265	382	406	703	912	791	601	817	334	610	324	278	367	201	537	402	383	468	550
ENSG00000224566	10	9	9	6	4	1	1	1	406	329	1244	194	0	4	6	10	1	2	1	6	0	0	2	0	286	322	180	573	0	6	1	2
ENSG00000280157	47	36	39	41	4	10	1	12	7	3	22	3	3	8	5	14	4	1	7	21	5	1	1	0	6	2	11	11	0	6	3	5
ENSG00000227625	1	0	1	0	0	0	0	0	1	0	1	0	0	0	0	0	2	0	0	0	0	0	0	0	0	0	0	0	0	0	0	0
ENSG00000143774	4869	3365	3998	3723	1734	1666	1176	1891	2551	1892	8509	1673	1398	1316	1395	1692	3557	4160	3900	3537	3321	1327	2288	1207	2081	3127	1722	5122	1701	1626	1888	2054
ENSG00000198835	19	8	35	15	8	11	10	8	17	14	99	14	8	9	17	14	7	17	10	13	22	12	18	15	8	18	33	53	23	22	22	23
ENSG00000203684	10	10	10	9	7	5	11	4	12	5	40	2	8	13	8	4	3	3	3	8	13	3	2	5	2	8	7	20	3	2	5	12
ENSG00000181873	478	381	487	391	572	569	389	582	372	361	1675	259	283	326	352	445	141	232	240	450	1073	534	768	406	340	604	382	1149	262	354	292	445
ENSG00000162913	73	55	57	80	35	63	46	78	59	60	132	40	20	19	12	29	20	18	19	59	83	42	56	54	37	45	58	105	5	23	9	31
ENSG00000154358	2635	2757	3032	2760	3323	4463	2921	4592	2478	2246	5649	1226	1244	1358	1431	1923	372	414	368	1508	3494	2123	2475	2054	815	1439	1068	3241	686	1250	1001	1478
ENSG00000269890	41	46	38	45	36	66	47	74	31	47	80	16	16	15	23	10	5	5	5	43	60	36	39	24	7	24	24	61	2	9	8	14
ENSG00000269934	3	6	11	4	3	5	4	13	1	4	13	1	3	3	6	2	1	1	0	8	7	4	3	3	0	5	1	11	2	2	1	2
ENSG00000270110	43	40	48	36	16	34	32	31	53	31	75	9	16	19	28	24	2	1	4	21	31	27	20	25	21	27	13	43	1	11	9	17
ENSG00000270104	11	1	10	7	3	2	5	4	1	2	6	0	4	2	2	3	1	1	0	5	3	1	2	3	0	3	2	6	0	2	0	6
ENSG00000154370	2061	1423	1816	1446	1055	1035	822	1128	1013	809	4161	780	979	840	1115	1103	615	650	563	1296	1779	923	1366	818	872	1468	867	2678	760	1042	919	1092
ENSG00000270094	0	0	0	0	0	0	0	0	0	0	0	0	0	0	0	0	0	0	0	0	0	0	0	0	0	0	0	0	0	0	0	0
ENSG00000231563	11	10	17	14	4	9	8	8	31	16	97	19	8	5	3	7	6	6	4	12	15	3	9	9	22	21	26	53	6	2	1	6
ENSG00000162931	79	75	107	64	33	50	30	48	286	231	681	150	31	37	32	52	11	18	12	57	50	31	28	33	102	243	133	414	4	12	7	26
ENSG00000168148	5	3	5	13	3	5	2	8	2	6	15	1	10	2	1	10	0	0	1	3	4	2	2	5	4	0	0	6	0	5	0	1
ENSG00000230841	1	0	1	0	0	0	0	0	1	0	0	0	0	1	0	0	0	0	0	0	0	2	0	0	0	0	0	1	0	0	0	0
ENSG00000181218	68	40	36	35	0	0	0	0	189	132	782	143	122	174	130	169	37	52	49	72	0	0	0	0	173	282	208	392	243	212	250	232
ENSG00000196890	29	16	27	16	0	0	0	0	24	9	52	4	13	10	8	6	28	18	21	25	0	0	0	0	15	19	19	51	12	20	16	22
ENSG00000266174	0	2	2	1	0	0	0	0	1	3	7	2	2	1	2	0	0	0	0	0	0	0	0	0	3	4	0	7	3	1	0	2
ENSG00000181201	16	2	6	2	0	0	0	0	18	6	35	12	0	8	12	12	2	6	2	3	0	0	0	0	10	27	9	30	6	11	5	9
ENSG00000279306	14	7	8	10	8	14	6	9	13	2	28	4	13	9	4	10	10	7	10	8	14	6	2	3	4	6	4	13	6	8	3	3
ENSG00000168159	2852	2020	2502	2146	2872	2895	2089	2982	2402	1871	8050	1628	1889	1700	1778	2205	661	1025	1208	1572	4005	1715	3038	1416	1958	3257	1956	5135	1327	1606	1327	1681
ENSG00000215811	19	5	8	12	19	24	11	26	1	5	16	5	4	1	5	6	10	7	5	10	34	7	27	3	4	3	14	10	1	3	4	3
ENSG00000183929	69	58	67	46	26	24	14	38	3	4	1	2	81	61	61	69	9	9	8	43	8	8	1	8	0	3	0	5	25	43	19	53
ENSG00000206878	11	12	8	7	3	9	2	4	2	0	0	0	7	12	10	12	2	1	3	5	4	1	1	2	0	0	0	1	1	8	2	10
ENSG00000234975	12	5	12	4	6	5	4	3	3	1	8	0	5	4	4	4	0	4	1	1	13	1	5	5	0	0	1	5	2	4	1	5
ENSG00000116574	274	417	278	439	106	146	103	131	64	90	172	73	301	284	212	356	123	503	297	748	246	78	212	85	35	104	45	231	77	117	64	136
ENSG00000271475	0	0	0	0	0	0	0	0	0	0	0	0	0	0	0	0	0	0	0	0	0	0	0	0	0	0	0	0	0	0	0	0
ENSG00000271399	0	0	0	0	0	0	0	0	0	0	0	0	0	0	0	0	0	0	0	0	0	0	0	0	0	0	2	1	0	0	0	0
ENSG00000282111	0	0	0	0	0	0	0	0	0	0	1	0	0	0	0	0	0	0	0	0	1	0	0	0	0	0	0	0	1	0	0	0
ENSG00000229840	0	0	0	0	0	0	0	0	0	0	0	0	0	0	0	0	0	0	0	0	0	0	0	0	0	0	0	0	0	0	0	0
ENSG00000236975	4	2	5	13	1	0	0	0	0	0	0	0	0	0	0	0	2	4	2	12	0	0	0	0	0	0	0	0	0	0	0	0
ENSG00000233920	0	0	0	0	0	0	0	0	0	0	0	0	0	0	0	0	0	0	0	0	0	0	0	0	0	0	0	0	0	0	0	0
ENSG00000177800	2	1	1	1	1	0	2	1	0	0	1	0	0	0	3	2	0	0	0	0	0	0	2	0	1	2	0	2	0	1	2	2
ENSG00000177788	38	19	27	24	24	23	28	33	31	23	107	14	35	37	48	44	5	13	9	27	67	19	28	18	16	27	19	64	12	23	26	32
ENSG00000240524	4	0	3	3	3	1	3	1	2	0	5	1	4	0	10	0	0	0	3	1	2	1	3	1	2	7	0	3	2	1	6	2
ENSG00000168118	666	457	553	503	532	606	482	608	572	485	1945	353	708	682	734	897	493	671	491	556	1352	478	883	469	529	737	411	1268	744	746	706	818
ENSG00000213029	0	0	0	0	0	0	0	0	0	0	0	0	0	0	0	0	0	0	0	0	0	0	0	0	0	0	0	0	0	0	0	0
ENSG00000237481	8	4	6	4	10	4	3	5	5	6	24	5	7	4	7	3	5	3	5	6	12	10	10	4	6	11	8	30	0	14	5	8
ENSG00000154429	2081	1775	1756	1707	1036	1172	796	1125	544	493	1683	319	1583	1486	1783	1841	677	924	779	1057	1138	450	765	403	609	831	315	1565	1196	1520	1598	1658
ENSG00000252506	0	0	0	0	0	0	0	0	0	0	0	0	0	0	0	0	0	0	0	0	0	0	0	0	0	0	0	0	0	0	0	0
ENSG00000252051	0	0	0	0	0	0	1	0	0	0	0	0	0	0	0	1	0	0	0	0	0	0	0	0	0	0	0	0	0	0	0	0
ENSG00000230331	0	0	0	0	0	0	0	0	0	0	0	0	0	0	0	0	0	0	0	0	0	0	0	0	0	0	0	0	0	0	1	0
ENSG00000143632	3	0	9	2	3	8	5	5	0	2	4	1	2	4	1	5	0	0	1	3	11	11	2	6	1	4	2	7	1	10	0	7
ENSG00000069248	2811	1900	2198	1869	1917	1923	1429	2055	1612	1312	5288	987	2245	2092	2342	2654	1770	2451	2227	2166	4138	1543	3004	1422	1654	2305	1230	4151	2310	2310	2611	2682
ENSG00000226920	0	0	0	0	0	0	0	0	0	0	0	0	0	0	0	0	0	0	0	0	0	0	0	0	0	0	0	0	0	0	0	0
ENSG00000223635	0	0	2	0	1	0	2	0	0	0	5	1	0	0	0	0	0	1	0	1	0	1	0	1	2	0	0	0	0	2	0	0
ENSG00000135776	2265	1701	1657	1547	881	911	632	1019	1076	948	3652	758	1144	1053	1154	1225	997	1834	1056	1703	812	270	671	283	1123	1858	918	3080	955	1049	920	1120
ENSG00000199672	14	4	11	16	3	3	1	6	8	3	11	4	3	7	3	1	4	2	3	8	3	0	3	4	2	6	1	15	1	7	6	6
ENSG00000229367	1	0	0	0	0	1	0	0	1	0	0	0	0	0	0	0	0	0	0	0	0	0	0	0	0	0	0	0	0	1	0	0
ENSG00000135801	1275	938	1089	1023	1220	1242	828	1282	872	765	3145	630	846	783	981	959	571	817	743	1165	2063	841	1572	719	936	1501	705	2235	873	1046	923	1133
ENSG00000135763	1099	817	1066	845	948	893	722	965	751	642	2789	538	920	733	1044	953	474	832	759	1248	1725	779	1338	746	943	1553	570	2281	844	1104	1006	1157
ENSG00000213028	0	0	0	0	1	4	3	3	0	1	5	1	0	1	8	0	0	1	0	2	8	4	10	3	0	0	1	2	0	7	3	10
ENSG00000225984	0	0	0	0	0	0	0	0	0	0	0	0	0	0	0	1	0	0	0	0	1	0	0	1	0	0	0	1	1	0	0	1
ENSG00000227043	0	0	0	0	0	0	0	0	0	0	0	0	0	0	0	0	0	0	0	0	0	0	0	0	0	0	0	0	0	0	0	0
ENSG00000228058	0	0	0	0	0	0	0	0	0	0	0	0	0	0	0	0	0	0	0	0	0	0	0	0	0	0	0	0	0	0	0	0
ENSG00000143641	3956	3662	3187	3855	4625	4517	3324	4964	913	731	4117	707	1118	1331	1129	1680	761	1632	1020	2580	5440	2236	3862	1752	899	1507	1415	2743	775	1080	622	1081

ENSG00000227006	27	34	25	28	21	25	32	27	6	8	41	6	0	3	2	5	8	5	3	36	29	23	21	17	11	15	12	28	0	10	4	10
ENSG00000224407	0	0	0	0	0	0	0	0	0	0	0	0	0	0	0	0	0	0	0	0	0	0	0	0	0	0	0	0	0	0	0	0
ENSG00000177614	33	136	34	111	0	0	0	2	105	182	1107	164	144	182	136	259	6	56	24	93	1	0	0	0	46	122	221	422	138	234	126	288
ENSG00000282564	0	0	0	0	0	0	0	0	0	0	2	0	0	0	0	0	0	0	0	0	0	0	0	0	0	0	0	0	0	0	0	0
ENSG00000232964	0	0	0	0	0	0	0	0	0	0	0	0	0	0	0	0	0	0	0	0	0	0	0	0	0	0	0	0	0	0	0	0
ENSG00000235817	0	0	0	0	0	0	0	0	0	0	0	0	0	0	0	0	0	0	0	0	0	0	0	0	0	0	0	0	0	0	0	0
ENSG00000135775	1006	729	891	761	1178	1218	963	1315	772	695	3624	544	766	725	765	900	1399	1746	1358	1321	3061	1248	2184	1219	901	1247	896	2321	743	864	870	952
ENSG00000135744	0	0	0	0	0	0	0	0	1	0	3	0	0	0	0	0	0	0	0	0	0	0	0	0	1	0	0	0	0	0	0	0
ENSG00000244137	0	1	2	0	0	1	0	0	0	0	2	0	0	0	0	0	0	0	0	0	0	0	0	0	0	0	2	0	0	0	0	0
ENSG00000135773	3	3	6	6	0	0	0	0	6	1	13	8	0	0	0	0	0	3	0	4	0	0	0	1	4	3	1	10	0	1	1	0
ENSG00000225656	0	0	0	0	0	1	0	0	0	0	0	0	0	0	0	0	0	0	0	0	0	0	0	0	0	0	0	0	0	0	0	0
ENSG00000119280	975	816	905	918	1487	1395	1112	1564	411	368	1457	319	799	762	825	987	226	540	418	593	2586	927	1789	828	433	713	301	1210	574	809	570	821
ENSG00000223393	1	0	0	1	7	8	3	11	2	0	3	0	0	2	5	5	0	1	2	1	18	9	17	8	0	1	1	1	0	1	6	2
ENSG00000240502	0	0	0	0	0	0	0	0	0	0	0	0	0	0	0	0	0	0	0	0	1	0	0	0	0	2	1	0	0	0	0	0
ENSG00000143643	676	475	493	463	389	430	321	450	588	497	3473	500	706	606	736	897	234	348	295	444	889	378	641	446	753	1003	1061	2214	385	689	485	778
ENSG00000173409	525	319	385	384	265	303	203	297	370	313	1209	237	335	334	377	424	597	708	669	680	676	299	552	328	386	530	274	773	536	548	604	638
ENSG00000182118	281	341	289	359	118	157	96	149	136	111	395	89	341	362	413	433	151	352	239	390	254	127	160	85	129	227	86	277	268	382	297	359
ENSG00000227934	1	13	1	2	3	1	2	2	2	3	12	0	0	1	1	3	0	3	0	6	3	1	0	5	3	1	4	4	0	3	1	2
ENSG00000229595	0	0	0	0	0	0	0	0	0	0	0	0	0	0	0	0	0	0	0	0	0	0	0	0	0	0	0	0	0	0	0	0
ENSG00000119283	7	7	3	4	3	1	1	3	2	0	7	1	4	2	0	0	3	4	2	14	5	2	10	3	1	1	8	4	3	1	1	6
ENSG00000235710	0	0	0	0	0	0	0	0	0	0	0	0	0	0	0	0	0	0	0	0	0	0	0	0	0	0	0	0	0	0	0	0
ENSG00000143633	748	469	545	461	351	433	309	411	471	417	1778	353	533	444	558	595	1069	1343	1163	933	1029	422	723	444	498	727	353	1190	774	603	837	803
ENSG00000116906	2207	1748	1852	1656	1746	1770	1374	1989	1191	893	3504	732	1389	1356	1411	1601	2010	2687	2219	2239	3746	1517	2556	1421	1068	1452	726	2367	1317	1485	1397	1697
ENSG00000116903	1346	995	1104	999	667	779	505	790	468	408	2044	310	618	645	750	806	358	420	432	876	1173	528	820	483	578	890	608	1428	426	614	422	621
ENSG00000010072	617	363	462	406	423	469	319	498	283	232	1129	194	382	336	529	431	504	723	702	658	1111	432	865	421	357	515	240	809	545	575	690	676
ENSG00000135766	3068	2554	2424	2311	35	26	13	21	1468	1453	2993	887	1154	1161	1273	1402	535	929	698	908	25	15	16	10	729	1210	605	1927	669	802	835	858
ENSG00000223982	2	0	1	0	0	0	0	0	0	0	0	0	0	0	0	0	0	0	0	0	0	0	0	0	0	0	0	2	0	0	0	0
ENSG00000233461	62	69	98	55	53	49	29	67	29	18	70	14	25	30	21	43	99	113	107	83	74	30	60	34	11	26	8	23	35	23	33	32
ENSG00000270106	2	0	0	0	0	0	1	2	0	0	0	0	1	0	0	1	1	0	0	1	1	1	5	2	0	0	0	0	0	0	1	1
ENSG00000116918	1113	851	974	868	1684	1895	1276	1921	817	711	3530	553	919	837	1098	1072	1034	1419	1009	1037	3170	1397	2439	1563	991	1229	794	2269	852	873	1145	1068
ENSG00000229228	0	0	0	1	1	0	1	0	0	0	2	0	0	2	0	0	1	1	0	0	1	0	1	3	0	0	1	0	0	2	0	0
ENSG00000162946	210	175	177	152	493	508	362	557	45	41	98	24	139	111	115	133	55	58	50	147	725	279	495	256	12	32	19	78	52	70	51	68
ENSG00000222986	2	1	0	0	1	1	4	0	0	0	0	0	0	1	0	0	0	0	0	0	0	1	0	0	0	0	0	0	0	1	0	0
ENSG00000274121	0	0	1	0	0	0	1	6	0	0	0	0	0	0	0	0	2	0	0	0	2	1	0	0	0	0	0	1	0	0	0	0
ENSG00000226758	0	2	1	3	8	5	3	5	0	2	1	0	0	3	0	2	0	0	1	5	8	2	5	7	0	0	1	0	1	0	0	0
ENSG00000235152	0	0	0	0	0	0	0	0	0	0	0	0	0	0	0	0	0	0	0	1	0	0	0	0	0	0	0	0	0	0	0	0
ENSG00000236372	0	0	0	0	0	0	0	0	0	0	0	0	0	0	0	0	0	0	0	0	0	2	0	0	0	0	0	0	0	0	0	0
ENSG00000224436	0	0	0	0	0	0	0	0	0	0	0	0	0	0	0	0	0	0	0	0	0	0	0	0	0	0	0	0	0	0	0	0
ENSG00000242794	0	0	0	0	0	0	0	0	0	0	0	0	0	0	0	0	0	0	0	0	2	0	0	0	0	0	0	0	0	0	0	0
ENSG00000116991	1192	1055	1569	1129	751	719	477	738	3	1	41	10	9	19	7	8	472	631	1153	1670	1278	519	646	429	4	5	8	31	5	18	10	19
ENSG00000238382	0	0	0	0	0	0	0	0	0	0	0	0	0	0	0	0	0	0	0	0	0	0	0	0	0	0	0	0	0	0	0	0
ENSG00000225919	0	0	0	0	0	0	0	0	0	0	0	0	0	0	0	0	0	0	0	0	0	0	0	0	0	0	0	0	0	0	0	0
ENSG00000236497	0	0	0	0	2	1	2	2	0	0	1	0	0	0	0	0	0	0	0	0	10	3	1	2	0	0	0	0	0	0	0	0
ENSG00000212916	0	0	1	0	642	729	473	754	166	142	692	114	46	68	60	105	0	0	1	3	1380	571	930	523	152	171	217	403	38	51	38	38
ENSG00000206835	0	0	0	0	2	1	0	1	0	0	0	0	0	0	0	0	0	0	0	0	4	1	0	0	0	0	2	0	0	0	0	0
ENSG00000232175	0	0	0	1	0	0	0	1	1	3	5	1	0	0	0	0	0	0	3	0	1	0	0	0	1	0	0	0	0	0	0	0
ENSG00000135778	1891	1243	1992	1335	1025	1106	663	1166	436	397	1936	264	630	611	551	774	1014	1075	1548	1518	1332	614	894	485	336	429	417	876	381	520	472	631
ENSG00000135749	1018	724	1041	882	623	688	470	712	440	448	2541	372	1243	1133	1360	1499	301	324	452	1038	888	468	679	458	367	534	580	1372	605	1227	853	1358
ENSG00000231940	23	15	16	15	12	14	12	9	9	3	29	6	12	17	8	24	11	12	15	8	15	7	10	8	9	12	6	18	12	19	9	19
ENSG00000251508	3	1	3	0	0	1	2	4	1	0	6	1	5	4	2	2	0	0	3	4	8	5	2	2	0	1	1	7	0	2	3	2
ENSG00000143674	849	826	704	817	804	835	591	941	453	454	1207	252	2425	2282	2601	2864	226	465	259	668	964	409	653	411	341	515	240	832	1512	2099	1723	2335
ENSG00000252501	0	0	0	0	0	0	0	0	0	0	0	0	0	0	0	0	0	0	0	0	0	0	0	0	0	0	0	0	0	0	0	0
ENSG00000135750	241	234	184	242	0	0	0	0	0	0	0	0	403	376	396	484	106	199	156	243	0	0	0	0	0	0	0	0	265	320	328	392
ENSG00000265744	1	1	1	2	0	0	0	0	0	0	0	0	1	0	3	2	0	0	0	2	0	0	0	0	0	0	0	0	0	0	2	0
ENSG00000225486	0	0	1	0	0	0	0	0	0	0	0	0	1	0	0	0	1	0	0	0	0	0	0	0	0	0	0	0	0	0	0	0
ENSG00000183780	12	25	15	24	26	29	15	45	0	0	2	0	239	243	217	326	4	28	13	20	10	8	17	6	0	1	0	0	155	222	143	267
ENSG00000236101	0	0	0	0	0	0	0	0	0	0	0	0	0	1	0	1	0	0	0	0	0	0	0	0	0	0	0	0	0	2	0	0
ENSG00000233332	0	1	0	0	0	0	1	2	0	0	0	0	2	1	2	5	0	1	0	1	0	0	0	0	0	0	0	0	3	12	4	5
ENSG00000231272	0	2	1	0	1	1	2	1	0	0	0	0	7	6	5	9	1	0	0	1	1	1	1	0	0	0	0	0	3	4	2	13
ENSG00000236244	2	2	3	0	1	0	2	4	0	0	0	0	2	4	9	6	0	0	0	1	2	0	2	0	0	0	0	0	0	12	3	9
ENSG00000232166	0	0	2	1	1	2	0	2	0	0	0	0	4	4	1	9	0	0	0	0	3	0	0	0	0	0	0	0	2	10	6	13
ENSG00000264377	0	0	0	0	0	1	0	1	0	0	0	0	0	3	1	5	0	0	0	0	0	0	0	0	0	0	0	0	0	2	4	1
ENSG00000235605	12	16	17	18	3	1	0	0	5	5	8	1	7	5	12	9	1	3	14	10	8	1	3	0	5	7	8	6	7	6	24	12
ENSG00000236358	113	102	110	101	0	0	0	0	30	22	79	12	24	29	36	41	18	11	10	38	0	0	0	0	9	7	23	39	9	19	14	31
ENSG00000273367	66	52	62	37	1	0	0	0	15	16	44	6	27	16	12	20	18	8	3	21	1	1	0	2	3	12	15	15	11	17	3	9
ENSG00000231663	34	23	15	14	70	39	38	46	43	29	150	33	23	26	20	32	14	22	12	18	79	40	53	52	32	39	40	66	24	38	23	27
ENSG00000168275	668	392	473	338	341	375	263	441	315	295	1020	209	444	407	496	529	912	1152	930	868	910	326	684	450	348	495	210	606	679	581	669	662
ENSG00000059588	3824	3222	3075	3388	3508	3914	2729	3993	1566	1430	5682	898	2435	2041	1889	2380	1023	1674	1242	2936	4864	2470	2903	2384	1081	1666	1285	3127	802	1578	878	1668
ENSG00000231768	1	0	2	1	0	0	0	0	0	0	1	0	4	3	3	5	0	3	0	0	0	0	0	2	0	0	0	0	1	2	0	2
ENSG00000230404	2	0	0	0	0	0	0	0	0	0	0	0	0	1	0	0	0	0	0	0	0	0	0	0	0	0	0	0	0	0	0	0
ENSG00000212144	0	0	0	0	1	0	0	0	0	0	0	0	0	0	0	1	0	1	0	0	0	0	0	0	1	0	0	3	0	0	0	0
ENSG00000168264	3604	3377	2980	3333	3217	3128	2318	3253	3074	2647	7808	1651	3986	3369	3831	4088	798	1210	807	1600	3860	1669	3025	1439	2279	3700	2337	5239	1887	2210	2043	2398
ENSG00000228830	0	0	0	0	0	0	0	0	0	0	0	0	0	0	0	0	0	0	0	0	0	0	0	0	0	0	0	0	0	0	0	0
ENSG00000224939	3	0	0	0	0	0	0	1	2	1	6	1	0	0	0	1	0	0	3	0	2	1	2	0	0	2	1	2	0	0	0	0
ENSG00000272362	0	0	0	0	0	0	0	0	0	0	2	0	0	0	0	0	0	0	0	0	0	0	0	0	0	0	0	0	0	0	0	0
ENSG00000228044	0	1	0	3	0	0	0	0	0	2	0	0	1	1	0	0	0	0	0	0	0	0	0	0	0	0	0	0	4	6	3	4
ENSG00000241475	0	0	0	0	2	0	0	0	0	0	0	0	0	0	0	0	0	0	0	0	0	0	0	0	0	0	0	0	0	2	1	6
ENSG00000230628	0	0	1	0	0	2	0	0	0	0	0	0	3	2	2	5	0	0	0	0	1	0	0	0	0	0	0	0	1	5	1	2
ENSG00000282097	0	0	1	0	0	0	0	0	0	0	0	0	0	0	0	0	0	0	0	0	0	0	0	0	0	0	0	0	0	1	0	0
ENSG00000227630	3	2	4	1	293	255	213	304	126	68	187	43	22	17	28	24	1	1	2	3	628	205	512	200	80	93	38	144	14	19	17	22
ENSG00000279261	1	0	0	0	3	0	1	0	0	0	1	0	0	0	1	2	0	0	0	2	2	0	0	0	0	0	0	0	0	0	0	0
ENSG00000201638	0	0	0	0	0	0	0	0	0	0	0	0	0	0	0	0	0	0	0	0	0	0	0	0	0	0	0	0	0	0	0	0
ENSG00000239690	0	0	0	0	0	0	0	0	0	0	0	0	0	0	0	0	0	0	0	0	0	0	0	0	0	0	0	0	0	0	0	0
ENSG00000237520	0	0	0	0	12	11	12	25	0	0	0	0	10	1	1	3	0	0	0	0	10	5	3	3	1	0	1	0	0	0	1	1
ENSG00000238005	0	0	0	4	110	124	100	140	2	2	7	2	12	10	29	27	2	2	0	3	109	41	62	46	3	1	3	5	10	10	13	8
ENSG00000258082	5	4	8	2	35	63	33	37	4	4	5	0	1	0	4	5	2	0	3	4	50	27	31	18	0	0	1	2	0	2	2	2
ENSG00000280587	0	1	4	0	2	2	0	1	2	2	4	0	0	2	1	5	0	0	0	0	2	0	0	1	0	0	0	0	0	1	0	1
ENSG00000233018	0	0	0	0	0	0	1	0	0	0	0	0	0	0	0	2	0	0	0	0	0	0	0	0	0	0	0	0	0	0	0	0
ENSG00000273416	3	0	2	1	1	1	1	1	2	2	6	0	2	1	2	7	1	0	0	2	5	1	4	4	1	0	0	0	0	0	1	2
ENSG00000173726	5439	4137	4245	3889	4229	4094	3132	4324	3945	3250	13080	2274	6585	5438	6083	6788	3292	4803	3817	4192	7727	3074	5692	2742	3958	5704	2908	8794	5071	5345	5182	6239
ENSG00000207181	12	8	6	4	5	4	4	7	4	3	16	0	19	7	11	11	11	3	8	12	18	8	8	7	4	3	3	14	10	12	21	22
ENSG00000188739	100	76	106	87	92	111	101	133	73	64	333	66	62	67	66	96	48	71	62	97	260	113	154	103	88	117	98	193	57	96	74	107
ENSG00000054267	3245	1966	2705	1984	1907	2102	1657	2124	1900	1474	8211	1173	2708	2474	2914	2984	977	1005	1230	1781	3496	1505	2522	1474	1802	2444	2371	5062	1614	2443	2302	2805
ENSG00000263439	3	0	0	0	0	0	0	0	0	0	4	1	1	1	1	0	0	0	0	0	0	2	0	0	0	0	1	2	1	0	2	0
ENSG00000236863	4	3	7	1	1	1	0	2	7	6	10	1	2	8	8	2	2	4	2	4	6	8	0	6	2	5	4	19	2	13	9	5
ENSG00000152904	1555	1149	1304	1113	893	930	687	959	762	620	2559	432	1158	1141	1075	1337	1510	1891	1709	1529	2010	813	1345	784	712	1001	637	1583	891	992	980	1130
ENSG00000227962	7	10	10	6	3	8	8	7	2	3	22	1	2	6	4	9	1	2	4	4	17	4	10	7	6	3	2	17	3	2	2	4
ENSG00000230026	0	0	0	0	0	2	2	2	2	0	2	0	0	1	1	1	0	0	0	2	1	0	1	1	1	1	3	0	0	0	1	2
ENSG00000116957	1190	849	981	821	959	946	747	1015	648	521	2229	398	1271	1054	1264	1399	669	779	726	835	1722	680	1294	607	663	879	475	1315	1065	1152	1206	1308
ENSG00000229795	7	3	7	2	4	4	1	7	1	2	2	0	4	2	2	1	2	2	2	1	4	3	2	4	1	8	6	9	0	5	3	7
ENSG00000162885	1399	1007	1176	1120	746	794	556	827	428	363	1627	278	561	450	606	630	346	445	487	760	1075	539	728	441	410	596	312	1041	355	507	480	569
ENSG00000238236	0	0	0	0	0	0	0	0	0	0	0	0	0	0	0	0	0	0	0	0	0	0	0	0	0	0	0	0	0	0	1	1
ENSG00000235638	0	0	0	0	0	0	0	0	0	0	1	0	0	0	0	0	0	0	0	0	0	0	0	0	0	0	0	0	0	0	0	0
ENSG00000231199	0	0	1	0	0	0	0	0	0	0	0	0	0	1	0	0	0	0	0	1	0	0	0	0	0	0	0	0	0	0	0	1
ENSG00000226663	0	0	0	0	0	0	0	0	0	0	0	0	0	0	0	1	0	0	0	0	0	0	0	0	0	0	0	0	0	0	0	0
ENSG00000231582	0	0	0	0	0	0	0	0	0	0	0	0	0	0	0	0	0	0	0	0	0	0	0	0	0	0	0	0	0	0	0	0
ENSG00000232714	0	0	0	0	0	0	0	0	0	0	0	0	0	0	0	0	0	0	0	0	0	0	0	0	0	0	0	0	0	0	0	0
ENSG00000235761	0	0	0	0	0	0	0	0	0	0	0	0	0	0	0	0	0	0	0	0	0	0	0	0	0	0	0	0	0	0	0	0
ENSG00000168243	241	313	275	298	1	1	1	0	1	4	2	2	1557	1538	1375	1950	31	139	93	183	0	3	0	2	0	0	0	0	938	1008	732	970
ENSG00000270710	0	1	1	0	0	0	0	0	0	0	0	0	6	4	4	5	0	0	0	0	0	0	0	0	0	0	0	0	1	4	2	6
ENSG00000227236	2	1	2	3	0	0	0	0	0	0	0	0	22	10	14	11	1	2	1	5	0	0	0	0	0	0	0	0	5	15	12	13
ENSG00000143669	593	428	556	488	238	331	230	366	271	274	1321	206	1111	1082	1533	1364	202	303	359	547	427	191	326	176	327	529	445	989	678	1091	1245	1445
ENSG00000235674	0	2	1	0	0	0	1	0	3	1	5	0	5	0	3	3	0	0	0	4	0	1	0	1	4	0	0	6	5	3	4	11
ENSG00000229463	2	2	1	3	2	4	2	2	0	5	4	1	3	2	3	3	0	1	1	0	4	1	1	2	2	0	2	4	5	3	5	1
ENSG00000222831	1	2	1	1	3	0	0	0	0	0	0	0	0	0	1	1	0	0	2	0	1	0	0	0	0	0	1	0	1	0	4	0
ENSG00000206803	0	0	0	0	0	0	0	0	0	0	0	0	0	0	0	0	0	0	0	0	0	0	0	0	0	0	0	0	0	0	0	0
ENSG00000237845	0	0	0	0	0	0	0	0	0	0	0	0	0	1	0	0	0	0	0	0	0	0	0	0	0	0	0	0	0	0	0	0
ENSG00000229291	0	0	1	1	0	0	2	0	0	0	0	0	1	0	2	1	2	0	2	0	0	0	0	0	0	0	0	0	0	0	0	0
ENSG00000116962	0	0	0	0	1	1	0	0	0	1	40	1	0	0	0	2	0	0	0	0	0	0	0	0	0	0	8	8	0	2	0	0
ENSG00000252822	0	0	0	0	0	0	0	0	0	0	3	0	0	0	0	0	0	0	0	0	0	0	0	0	0	0	0	0	0	0	0	0
ENSG00000235371	0	2	0	0	0	0	0	0	0	0	0	0	0	0	0	0	0	0	0	0	0	0	0	0	0	0	1	0	0	0	0	0
ENSG00000077585	247	234	196	224	2	6	1	2	131	115	656	132	305	290	348	395	98	157	85	196	4	4	1	1	125	155	198	443	259	347	301	366
ENSG00000086619	565	427	455	435	303	316	213	348	169	185	897	136	368	365	369	451	199	268	235	294	347	124	186	111	172	252	347	586	246	330	306	413
ENSG00000222650	1	1	0	1	0	0	0	3	0	1	3	1	0	0	0	0	0	0	1	1	1	0	0	0	1	0	2	0	1	2	0	0
ENSG00000237922	0	0	0	0	0	0	0	0	0	0	0	0	0	1	0	0	0	1	0	1	0	2	0	0	0	0	0	1	0	0	0	0
ENSG00000186197	9	35	21	42	0	1	0	0	45	41	160	52	92	102	96	112	2	21	11	30	1	1	0	0	35	51	55	127	88	129	105	167
ENSG00000244457	0	0	0	0	0	0	0	0	0	0	0	0	0	0	0	0	0	0	0	0	0	0	0	0	0	0	0	0	0	0	0	0
ENSG00000116977	1742	1272	1519	1148	543	471	369	567	1430	1299	5361	990	1001	921	950	1225	745	978	888	1044	799	342	487	329	1212	1679	1516	3492	838	991	874	1143
ENSG00000223776	8	10	3	17	0	0	1	2	4	1	10	1	5	7	1	14	5	8	1	12	1	0	0	0	3	3	4	5	2	2	1	2
ENSG00000273058	16	14	18	13	8	13	8	10	24	15	73	12	5	4	11	17	5	14	12	34	54	6	22	9	17	26	25	55	6	9	11	12
ENSG00000230325	30	32	19	28	10	8	9	9	26	11	64	12	15	6	9	11	6	6	5	8	16	7	5	5	19	21	11	30	3	6	8	7
ENSG00000119285	3323	2196	2821	2275	2832	2913	2178	2887	2229	1928	7449	1377	4303	3888	4112	4515	2160	3090	2682	3352	6421	2610	4665	2309	2560	3814	1626	5424	3713	4536	4249	4854
ENSG00000077522	2	3	1	12	10	12	10	7	3	3	4	0	13	20	9	23	0	8	4	7	19	14	11	13	0	3	0	4	5	9	3	16
ENSG00000116984	4929	4119	4213	4182	4189	4559	3336	5001	3332	2593	9681	1850	3973	3916	3579	4675	798	1172	1013	2426	6291	2871	4732	2573	2639	3869	2121	6676	2040	3205	2155	3433
ENSG00000226498	7	5	8	4	3	3	4	1	2	4	6	4	5	3	1	2	1	3	2	6	5	3	2	5	7	10	5	12	2	3	2	6
ENSG00000237991	1	0	1	3	0	0	0	0	1	1	3	0	2	0	0	2	1	0	0	0	2	1	0	0	0	1	0	0	0	0	0	2
ENSG00000244020	0	0	0	0	0	0	0	0	0	0	0	0	0	0	0	0	0	0	0	0	0	0	0	0	0	0	0	0	0	0	0	0
ENSG00000198626	3	0	1	1	1541	1491	1287	1641	34	7	75	7	659	606	597	667	0	1	1	0	2039	967	1576	836	12	11	21	49	136	261	177	304
ENSG00000252396	0	0	0	0	0	0	0	0	0	0	0	0	0	0	0	0	0	0	0	0	0	0	0	0	0	0	0	0	0	0	0	0
ENSG00000266262	1	0	0	0	3	5	2	14	0	0	0	0	0	0	1	2	0	0	0	0	7	5	6	4	0	0	0	0	0	0	1	2
ENSG00000252290	0	0	0	0	1	0	1	0	0	0	0	0	1	0	0	0	0	0	0	0	2	3	1	0	0	0	0	0	0	0	1	0
ENSG00000237250	0	0	0	0	0	0	0	0	0	0	0	0	0	0	0	0	0	0	0	0	0	0	0	0	0	0	0	0	0	0	0	0
ENSG00000116996	0	0	0	0	0	0	0	0	0	0	0	0	0	0	0	0	0	0	0	0	0	0	0	0	0	0	0	0	0	0	0	0
ENSG00000243781	0	0	0	0	0	0	0	0	0	0	0	0	0	0	0	0	0	0	0	0	0	0	0	0	0	0	0	0	0	0	0	0
ENSG00000283174	0	0	0	0	0	0	0	0	0	0	0	0	0	0	0	0	0	0	0	0	0	0	0	0	0	0	0	0	0	0	0	0
ENSG00000283166	0	0	0	0	0	0	0	0	0	0	0	0	0	0	0	0	0	0	0	0	0	0	0	0	0	0	0	0	0	0	0	0
ENSG00000283377	0	0	0	0	0	0	0	0	0	0	0	0	0	0	0	0	0	0	0	0	0	0	0	0	0	0	0	0	0	0	0	0
ENSG00000270188	0	0	0	0	0	0	0	0	0	0	0	0	0	0	0	0	0	0	0	0	0	0	0	0	0	0	0	0	0	0	0	0
ENSG00000232154	0	0	0	0	0	0	0	0	0	0	0	0	0	0	0	0	0	0	0	0	0	0	0	0	0	0	0	0	0	0	0	0
ENSG00000225723	0	0	0	0	0	0	0	0	0	0	0	0	0	0	0	0	0	0	0	0	0	0	0	0	0	0	0	0	0	0	0	0
ENSG00000227860	0	0	0	0	0	0	0	0	0	0	0	0	0	0	0	0	0	0	0	0	0	0	0	0	0	0	0	0	0	0	0	0
ENSG00000230019	0	0	0	0	0	0	0	0	0	0	1	0	0	0	0	0	0	0	0	0	0	0	0	0	0	0	0	0	0	0	0	0
ENSG00000232989	0	0	0	0	0	0	0	0	0	0	0	0	0	0	0	0	0	0	0	0	0	0	0	0	0	0	0	0	0	0	0	0
ENSG00000234464	0	0	0	0	0	0	0	0	0	0	0	0	0	0	0	0	0	0	0	0	0	0	0	0	0	0	0	0	0	0	0	0
ENSG00000252371	0	0	0	0	0	0	0	0	0	0	4	0	0	0	0	0	0	0	0	0	0	0	0	0	0	0	1	0	0	0	0	0
ENSG00000215808	0	0	0	0	0	0	0	0	0	0	0	0	0	0	0	0	0	0	0	0	0	0	0	0	0	2	0	1	0	0	0	0
ENSG00000231877	0	0	0	0	0	0	0	0	0	1	3	0	0	0	0	0	0	0	0	0	0	0	0	0	0	0	0	1	0	0	0	0
ENSG00000215149	0	0	0	0	0	0	0	0	0	0	0	0	0	0	0	0	0	0	0	0	0	0	0	0	0	0	0	1	0	2	0	0
ENSG00000224783	0	0	0	0	0	0	0	0	0	0	3	0	0	0	0	0	0	0	0	0	0	0	0	0	0	0	0	0	0	0	0	0
ENSG00000227854	0	0	0	0	0	0	0	0	0	0	0	0	0	0	0	0	0	0	0	0	0	0	0	0	0	0	0	0	0	0	0	0
ENSG00000259776	0	0	0	0	1	0	0	0	0	0	0	0	0	0	0	0	0	0	0	0	0	0	0	0	0	0	0	0	0	0	0	0
ENSG00000133019	0	1	0	0	1	0	2	1	14	20	84	13	248	284	229	358	0	1	0	1	4	4	3	3	9	18	10	28	149	208	178	292
ENSG00000233355	32	19	23	20	5	2	3	6	241	193	933	103	68	41	72	59	15	27	1	18	22	5	12	5	194	252	265	506	23	44	53	35
ENSG00000234601	0	0	0	0	0	0	0	0	0	0	0	0	2	1	3	2	0	0	0	0	0	0	0	0	0	0	0	0	1	5	2	6
ENSG00000231979	0	0	0	0	0	0	0	0	0	0	0	0	0	0	0	0	0	0	0	0	0	0	0	0	0	0	0	0	0	0	0	0
ENSG00000215805	0	0	0	0	0	0	0	0	0	0	0	0	0	0	0	0	0	0	0	0	0	0	0	0	0	0	0	0	0	0	0	0
ENSG00000217327	0	0	0	0	0	0	0	0	0	0	0	0	0	0	0	0	0	0	0	0	0	0	0	0	0	0	0	0	0	0	0	0
ENSG00000155816	0	4	1	6	0	0	0	0	0	0	1	0	0	0	0	0	1	0	0	3	0	0	1	0	3	0	1	2	0	0	0	1
ENSG00000228818	0	0	0	0	0	0	0	0	0	0	1	0	0	0	0	0	0	0	0	2	3	0	0	0	0	2	0	0	0	0	0	1
ENSG00000202041	0	0	0	0	0	0	0	0	0	0	0	0	0	0	0	0	0	0	0	0	0	0	0	0	0	0	0	0	0	0	0	0
ENSG00000223694	0	0	0	0	0	0	0	0	0	0	0	0	0	0	0	0	0	0	0	0	0	0	0	0	0	0	0	0	0	1	0	0
ENSG00000233735	0	0	0	0	0	0	0	0	0	0	0	0	0	0	0	0	0	0	0	0	0	0	0	0	0	0	0	0	0	0	0	0
ENSG00000252317	0	0	0	0	0	0	0	0	0	0	0	0	0	0	0	0	0	0	0	0	0	0	0	0	0	0	0	0	0	0	0	0
ENSG00000233519	0	0	0	0	0	0	0	0	0	0	4	0	0	0	0	0	0	0	0	0	0	0	0	0	0	0	0	0	0	0	0	0
ENSG00000180875	0	0	0	1	0	1	0	0	0	0	0	0	0	1	0	0	0	0	0	0	0	2	0	0	0	0	0	1	0	0	0	0
ENSG00000224359	0	0	0	0	0	0	0	0	0	0	0	0	0	0	0	0	0	0	0	0	0	0	0	0	0	0	0	0	0	0	0	0
ENSG00000226014	0	0	0	0	0	0	0	0	0	0	0	0	0	0	0	0	0	0	0	0	0	0	0	0	0	0	0	0	0	1	0	0
ENSG00000231440	0	0	0	0	0	0	0	0	0	0	0	0	0	0	0	0	0	0	0	0	0	0	0	0	0	0	0	0	0	0	0	0
ENSG00000228844	0	0	0	0	0	0	0	0	0	0	0	0	0	0	0	0	0	0	0	0	0	0	0	0	0	0	0	0	0	0	0	0
ENSG00000251750	0	0	0	0	0	0	0	0	0	0	0	0	0	0	0	0	0	0	0	0	0	0	0	0	0	0	0	0	0	0	0	0
ENSG00000234872	0	0	0	0	0	0	0	0	0	0	0	0	0	0	0	1	0	0	0	0	0	0	0	0	0	0	0	0	0	0	0	0
ENSG00000199241	0	0	0	0	0	0	0	0	0	0	0	0	0	0	0	0	0	0	0	0	0	0	0	0	0	0	0	0	0	0	0	0
ENSG00000230015	0	0	0	0	0	0	0	0	0	0	0	0	0	0	0	0	0	0	0	0	0	0	0	0	0	0	0	0	0	0	0	0
ENSG00000226919	0	0	0	0	0	0	0	0	0	0	0	0	0	0	0	0	0	0	0	0	0	0	0	0	0	0	0	0	0	0	0	0
ENSG00000223963	0	0	0	2	0	0	0	0	0	0	0	0	0	0	0	0	0	0	0	0	0	0	0	0	0	0	0	0	0	0	0	0
ENSG00000182901	0	1	0	0	1	1	0	1	0	0	0	0	0	0	1	0	0	0	0	0	0	0	0	0	0	0	0	2	0	0	0	0
ENSG00000215802	0	0	0	0	0	0	0	0	0	0	0	0	0	0	0	0	0	0	0	0	0	0	0	0	0	0	0	0	0	0	0	0
ENSG00000233615	0	0	0	0	0	0	0	0	0	0	0	0	0	0	0	2	1	0	0	0	0	1	0	0	0	0	0	0	0	0	0	0
ENSG00000238085	0	0	0	0	0	0	0	0	0	0	0	0	0	0	0	0	0	0	1	0	0	0	0	1	0	0	0	0	0	0	2	0
ENSG00000265831	0	0	0	0	0	0	0	0	0	0	0	0	0	0	0	0	0	0	0	0	0	0	0	0	0	0	0	0	0	0	0	0
ENSG00000224348	0	0	0	0	0	0	0	0	0	0	0	0	0	0	0	0	0	0	0	0	0	0	0	1	0	0	0	0	0	0	0	0
ENSG00000225554	0	0	0	0	0	0	0	0	0	0	0	0	0	0	0	0	0	0	0	0	0	0	0	0	0	0	0	0	0	0	0	0
ENSG00000091483	2152	1477	1552	1524	1772	1667	1362	1808	1663	1131	4285	911	2092	1677	2015	2175	3294	4790	3327	2863	5651	2022	3705	2244	1801	2558	844	3480	2747	2389	2978	3106
ENSG00000117009	23	25	15	17	1	1	0	1	4	5	13	3	15	7	4	18	3	9	10	11	1	1	0	1	1	6	5	4	1	9	0	6
ENSG00000054277	360	260	278	279	32	54	33	39	85	118	280	60	195	235	143	242	151	247	172	179	40	25	30	16	51	91	55	126	74	111	53	92
ENSG00000203668	2262	1597	1983	1609	679	648	446	719	1866	1587	5464	1055	1611	1468	1676	1763	1270	1749	1917	2444	1222	514	863	493	1690	2361	1133	3778	1483	1927	1518	2090
ENSG00000162843	3	0	0	0	0	2	0	0	0	0	14	0	3	1	0	1	2	1	0	1	2	0	0	0	0	2	1	2	0	3	0	2
ENSG00000229022	1	0	2	2	0	0	0	0	0	0	0	0	1	0	0	0	0	0	0	0	0	0	0	0	1	0	0	0	0	0	0	0
ENSG00000174371	1666	1291	1489	1261	1162	1260	1022	1381	1057	805	2578	661	1026	862	1109	1202	790	1378	1250	1390	2562	1109	2078	1025	950	1358	424	2421	903	1133	1030	1206
ENSG00000235990	11	7	7	8	5	8	7	4	7	10	49	6	5	7	6	1	1	1	4	10	14	7	5	4	2	9	6	34	3	9	4	13
ENSG00000196289	0	1	2	0	0	1	0	0	3	0	3	0	3	6	2	16	0	0	0	2	0	0	1	0	1	0	0	0	1	7	5	5
ENSG00000213026	0	0	0	0	0	0	0	0	0	0	0	0	0	0	0	0	0	0	0	0	0	0	0	0	0	0	0	1	0	0	0	0
ENSG00000197769	0	0	0	0	0	0	0	0	0	0	0	0	0	2	0	0	0	0	0	0	0	0	0	1	0	0	1	0	0	0	0	0
ENSG00000224625	0	0	0	0	0	0	0	0	0	0	0	0	0	0	0	0	0	0	0	0	0	0	0	0	0	0	0	1	0	0	0	0
ENSG00000277099	0	0	0	0	0	0	0	0	0	0	0	0	0	0	0	0	0	0	0	0	0	0	0	0	0	0	0	0	0	0	0	0
ENSG00000180287	0	0	0	0	0	0	0	0	0	0	0	0	0	1	0	1	0	0	0	0	0	0	0	0	0	0	0	1	0	0	0	0
ENSG00000272865	0	0	0	0	0	0	0	0	0	0	0	0	0	0	0	0	0	0	0	0	0	0	0	0	0	0	0	0	1	0	0	5
ENSG00000252084	0	0	0	0	0	0	0	0	0	0	0	0	0	0	0	0	0	0	0	0	0	0	0	0	0	0	0	0	0	0	0	0
ENSG00000224525	0	0	0	0	0	0	0	0	0	0	0	0	0	0	0	0	0	0	0	0	0	0	0	0	0	0	0	0	0	0	0	0
ENSG00000226750	0	0	0	0	0	0	0	0	0	0	0	0	0	0	0	0	0	0	0	0	0	0	0	0	0	0	0	0	0	0	0	0
ENSG00000235467	0	0	0	0	0	0	0	0	0	0	0	0	0	0	0	0	0	0	0	0	0	0	0	0	0	0	0	0	0	0	0	0
ENSG00000213690	0	0	0	0	0	0	0	0	0	0	0	0	0	0	0	0	0	0	0	0	0	0	0	0	0	0	0	0	0	0	0	0
ENSG00000270859	0	0	0	0	0	0	0	0	0	0	0	0	0	0	0	0	0	0	0	0	0	0	0	0	0	0	0	0	0	0	0	0
ENSG00000215800	0	0	0	0	0	0	0	0	0	0	0	0	0	0	0	0	0	0	0	0	0	0	0	0	0	0	0	0	0	0	0	0
ENSG00000270818	0	0	0	0	0	0	0	0	0	0	0	0	0	0	0	0	0	0	0	0	0	0	0	0	0	0	1	0	0	0	0	0
ENSG00000277704	0	0	0	0	0	0	0	0	0	0	0	0	0	0	0	0	0	0	0	0	0	0	0	0	0	0	0	0	0	0	0	0
ENSG00000230199	1	0	0	0	0	0	0	1	1	1	1	0	0	0	0	0	0	0	0	1	0	0	0	0	0	0	0	0	0	0	0	0
ENSG00000234116	0	0	0	0	0	0	0	0	0	0	0	0	0	0	0	0	0	0	0	0	0	0	0	0	0	0	0	0	0	0	0	0
ENSG00000231512	0	1	0	0	0	0	0	0	0	0	1	0	0	0	0	0	0	0	0	0	0	0	0	0	0	0	1	0	0	0	0	0
ENSG00000282831	0	0	0	0	0	0	0	0	0	0	0	0	0	0	0	0	0	0	0	0	0	0	0	0	0	0	0	0	0	0	0	0
ENSG00000278455	0	0	0	0	0	0	0	0	1	0	5	1	0	0	0	0	0	0	0	0	0	0	0	0	1	0	0	2	0	0	0	0
ENSG00000253326	1	0	1	0	1	1	0	1	17	13	59	8	6	0	1	4	0	0	0	1	0	0	0	0	5	12	23	35	1	0	2	2
ENSG00000214837	41	40	46	30	32	31	24	42	63	69	360	42	22	15	12	35	10	9	10	33	50	21	29	22	52	64	79	201	6	25	14	23
ENSG00000212230	0	0	1	1	0	0	0	0	0	0	0	0	0	0	0	0	0	0	0	0	0	0	1	0	0	0	0	2	0	0	1	0
ENSG00000143702	1307	1267	1407	1269	2877	3214	2367	3164	1747	1467	5845	996	1187	1166	1352	1428	518	879	840	1400	4808	1892	3274	1800	1642	2155	1447	4095	913	1282	1154	1452
ENSG00000227230	9	15	9	14	17	30	20	15	21	17	59	6	10	6	7	7	2	11	2	20	36	14	12	17	5	12	16	34	3	8	3	8
ENSG00000232085	0	0	0	0	0	0	4	0	0	3	2	0	3	2	1	0	0	1	0	2	3	1	2	2	6	0	3	10	0	1	2	1
ENSG00000054282	530	442	525	476	494	526	446	626	428	313	1698	235	518	447	578	578	335	487	390	457	910	395	644	427	384	505	512	1105	366	453	533	580
ENSG00000224727	5	4	1	5	7	10	10	5	4	4	18	3	4	7	4	6	1	0	0	4	11	5	6	11	5	8	11	21	1	3	8	5
ENSG00000265201	1	0	0	1	2	4	2	3	1	2	5	5	1	0	1	0	1	1	1	2	3	3	8	3	2	2	1	3	1	1	1	4
ENSG00000117020	1295	1312	1120	1364	5	12	11	11	675	593	2837	471	1057	1195	946	1397	324	646	407	827	11	10	3	6	709	1027	799	2195	549	1002	556	1082
ENSG00000236031	3	3	2	9	0	1	1	1	3	4	17	0	1	7	10	13	0	4	0	6	6	0	1	0	5	8	8	6	1	11	4	5
ENSG00000226766	1	2	0	2	1	0	0	0	0	2	3	1	2	0	2	3	0	1	2	2	0	0	0	0	4	6	6	4	0	3	0	5
ENSG00000232184	2	0	2	1	0	0	0	0	0	1	5	3	5	5	3	0	2	0	0	3	0	0	0	0	0	2	4	9	1	8	1	3
ENSG00000228939	10	9	3	4	0	0	0	0	6	4	29	1	11	7	1	13	0	3	1	4	0	0	0	0	3	8	8	25	2	13	6	9
ENSG00000226828	4	3	2	3	2	2	0	1	0	0	4	0	0	1	0	1	0	1	3	4	3	1	2	5	0	0	1	0	0	0	0	0
ENSG00000179456	1651	1298	1465	1359	881	966	661	945	249	325	1423	271	266	342	263	420	474	664	639	1129	1331	531	967	573	325	535	474	1211	175	298	180	308
ENSG00000279774	0	0	0	0	0	0	0	0	0	0	1	0	0	0	1	0	0	0	1	0	0	0	0	0	0	0	0	0	0	0	0	0
ENSG00000229960	0	0	0	0	0	0	0	0	0	0	0	0	0	0	0	0	0	0	0	0	0	0	0	0	0	0	1	0	0	0	0	0
ENSG00000244066	0	0	0	0	0	0	0	0	0	0	0	0	0	0	0	0	0	0	0	0	0	0	0	0	0	0	0	0	0	0	0	0
ENSG00000237759	0	0	0	0	0	0	0	0	0	0	0	0	0	0	0	0	0	0	0	0	0	0	0	0	0	0	0	0	0	0	0	0
ENSG00000173728	2	0	7	1	6	5	7	8	9	5	38	6	0	0	0	1	1	0	4	10	9	2	8	6	6	13	7	21	0	1	1	4
ENSG00000240963	1	0	0	0	1	2	0	1	0	1	4	1	0	0	0	0	0	0	1	0	4	0	0	3	0	0	1	6	0	0	0	1
ENSG00000225401	3	2	4	7	7	7	1	5	0	0	26	0	2	2	0	2	4	0	1	3	19	8	7	4	7	4	7	27	1	0	1	3
ENSG00000035687	2895	1897	2273	1979	1913	2008	1660	2169	2005	1508	7641	1235	3268	2928	3455	3636	2104	3256	2737	3034	4807	1973	3665	2013	2400	3301	1553	5223	3451	3626	3862	4171
ENSG00000179397	36	16	49	22	32	35	32	36	27	13	75	10	10	12	14	16	8	19	15	51	49	28	30	28	9	16	12	34	7	12	9	16
ENSG00000227735	0	0	0	0	0	0	0	0	0	0	0	0	0	0	0	0	0	0	0	0	1	0	0	0	0	0	0	1	0	0	0	1
ENSG00000121644	2809	2190	2465	2264	2250	2089	1566	2424	1649	1339	7342	1178	1658	1375	1612	1865	2150	3370	2648	3097	5534	2159	3836	1948	2025	2848	1761	5635	1653	1779	1774	1855
ENSG00000232059	3	3	8	4	2	4	3	9	4	4	26	8	7	6	5	3	2	1	0	2	7	3	7	2	8	6	2	23	0	4	4	1
ENSG00000282317	0	0	0	0	0	0	0	2	0	2	5	0	0	0	0	0	0	0	0	0	0	1	0	0	0	1	0	0	0	0	0	0
ENSG00000203667	271	194	249	188	188	187	166	227	187	164	720	140	134	137	102	158	318	295	348	355	414	219	302	256	194	275	133	477	118	153	157	179
ENSG00000153187	29635	21240	26404	22057	28823	29941	23474	32443	24640	19311	70622	13645	25916	24072	25871	30320	8862	14614	15027	21367	46154	19960	36263	17926	21829	34007	14349	50167	20504	26289	20991	27357
ENSG00000273175	0	2	2	0	3	2	0	3	2	0	2	0	0	1	1	0	2	4	1	3	10	2	6	2	1	2	0	5	3	1	1	1
ENSG00000201758	0	0	0	1	0	0	0	0	0	0	0	0	0	0	0	0	0	0	0	0	0	0	0	0	0	0	0	1	0	0	0	0
ENSG00000252073	0	0	0	0	0	0	0	0	0	0	0	0	0	0	0	0	0	0	0	0	0	0	0	0	0	0	0	0	0	0	0	0
ENSG00000272195	7	11	13	14	12	20	22	23	18	20	53	12	15	24	14	13	4	3	3	17	34	22	22	13	8	15	19	33	5	16	5	27
ENSG00000203666	70	52	43	62	121	209	143	177	261	191	687	109	59	57	81	88	48	50	50	83	372	159	241	200	185	281	138	394	50	70	77	82
ENSG00000252282	0	0	0	0	0	0	0	0	0	0	2	0	0	3	0	0	0	0	0	0	2	0	1	1	1	0	0	2	0	0	0	0
ENSG00000251754	0	0	0	0	0	2	0	0	0	0	2	0	0	0	0	0	0	0	0	0	1	0	0	0	0	0	0	1	0	0	0	0
ENSG00000223353	0	0	0	0	0	0	0	0	0	0	0	0	0	0	0	0	0	0	0	0	0	0	0	0	0	0	0	0	0	0	0	0
ENSG00000201170	0	0	0	0	0	1	0	0	0	2	0	0	0	0	0	0	0	0	0	0	0	0	1	1	0	0	0	0	0	0	0	0
ENSG00000162849	10	10	11	9	47	58	45	63	22	32	103	16	7	13	6	15	1	15	10	21	102	61	43	36	17	30	33	84	5	17	6	16
ENSG00000232192	0	0	0	0	0	0	1	1	1	1	3	0	0	1	0	0	0	0	0	2	0	1	0	0	0	0	0	5	0	0	0	0

ENSG00000232793	0	0	0	0	0	0	0	0	0	0	1	0	0	0	0	0	0	0	0	0	0	2	0	2	0	0	0	0	0	0	0	0
ENSG00000238224	0	0	3	0	4	3	2	2	0	0	1	0	0	1	0	0	0	0	0	0	1	3	5	0	0	0	0	0	0	1	0	0
ENSG00000231612	4	0	1	2	15	9	9	11	1	1	4	1	1	1	0	1	0	2	0	0	14	4	9	10	4	0	1	1	0	1	1	0
ENSG00000185420	915	696	745	627	2873	2941	2139	3164	385	299	1052	200	387	399	418	550	693	1137	791	737	6294	2210	3932	2203	320	428	209	759	337	335	404	414
ENSG00000235096	10	5	7	3	26	29	17	39	11	4	38	1	2	2	4	2	7	4	0	3	35	19	22	28	7	11	5	13	0	2	2	16
ENSG00000226876	14	9	14	15	63	81	60	89	16	8	44	3	8	8	4	11	8	2	4	24	100	67	59	80	7	7	5	31	0	10	10	23
ENSG00000225222	7	3	8	3	18	27	16	32	4	1	10	0	2	1	4	3	3	1	0	12	46	16	19	23	5	2	0	9	0	3	6	6
ENSG00000202184	8	0	7	5	19	11	4	16	2	3	7	1	0	1	2	5	0	0	1	9	19	15	12	13	6	0	1	9	1	2	3	3
ENSG00000230184	18	12	12	13	44	37	29	28	16	20	92	18	8	8	11	11	4	3	1	22	49	33	26	13	20	13	16	48	3	13	12	12
ENSG00000207326	0	2	2	0	3	2	1	3	0	2	3	0	0	0	3	1	0	0	0	6	2	2	0	2	0	0	0	6	0	1	4	2
ENSG00000227728	0	0	0	0	0	0	0	0	0	0	0	0	0	0	0	0	0	0	0	1	0	0	0	1	0	0	0	0	0	0	0	0
ENSG00000242042	1	0	2	1	3	3	0	2	0	0	3	0	1	0	0	0	0	0	0	0	1	0	0	1	0	0	0	1	0	0	0	1
ENSG00000230813	0	0	0	0	1	1	0	0	0	0	4	0	0	0	0	0	0	0	0	0	1	1	1	2	0	1	1	0	0	0	0	0
ENSG00000229112	0	0	0	0	1	0	0	0	0	0	4	0	0	0	0	0	0	0	0	0	1	0	0	0	0	0	0	0	0	0	0	2
ENSG00000162851	408	231	329	242	429	412	341	440	521	363	1682	277	646	521	710	647	497	662	511	589	1172	485	869	501	560	724	358	1104	814	810	885	872
ENSG00000162852	1076	672	907	693	1625	1628	1246	1804	530	430	1975	372	613	475	608	651	503	692	705	791	3963	1706	2918	1578	536	856	487	1360	527	713	735	796
ENSG00000225300	11	8	9	3	19	7	12	14	6	5	21	3	4	6	3	1	5	1	1	10	33	16	17	17	5	10	4	17	2	8	3	5
ENSG00000228955	4	4	6	3	9	9	11	12	5	2	5	0	2	0	1	1	3	3	0	4	30	22	10	18	0	0	1	3	1	2	4	3
ENSG00000260698	5	3	10	4	14	14	17	12	7	7	20	9	2	2	11	2	3	4	3	16	33	15	28	22	5	7	6	16	4	3	2	6
ENSG00000235021	0	0	2	0	0	1	0	0	0	2	0	0	0	0	0	0	0	2	0	1	0	0	0	2	0	0	0	4	0	1	0	0
ENSG00000143653	2378	1711	1977	1738	3139	2921	2425	3340	1603	1257	4483	920	1590	1640	1615	2026	2305	3581	3055	2371	7324	2718	5477	2468	1284	1994	928	3171	1641	1546	1926	1915
ENSG00000228879	2	4	2	1	6	2	2	4	2	0	9	0	1	0	2	1	0	3	1	2	10	5	3	2	2	5	3	9	0	3	3	4
ENSG00000223519	30	9	24	21	110	124	92	115	28	22	81	18	12	18	11	13	8	6	6	33	164	78	81	84	11	18	22	46	6	12	7	23
ENSG00000260855	51	44	52	32	208	224	210	263	48	60	211	31	34	35	35	55	13	13	17	64	265	150	207	155	44	36	42	164	11	38	26	35
ENSG00000227953	25	28	56	33	160	187	141	245	30	26	144	27	14	29	17	28	8	19	19	66	284	140	149	137	32	25	32	75	9	32	6	29
ENSG00000235386	0	0	0	0	0	0	0	0	0	0	0	0	0	0	0	0	0	0	0	0	0	0	0	0	0	0	0	0	0	0	0	0
ENSG00000252011	0	0	0	0	0	0	0	0	0	0	0	0	0	0	0	0	0	0	0	0	0	0	0	0	0	0	0	0	0	0	0	0
ENSG00000153207	4416	2878	3777	2975	3082	3294	2651	3313	1868	1536	7095	1267	3007	2542	3285	3228	1436	2047	2001	3259	6050	2565	4279	2490	2267	3302	1554	5592	2249	2986	2750	3184
ENSG00000197472	259	177	228	174	187	224	194	250	0	0	2	0	2	5	2	7	151	132	135	179	446	174	271	172	0	0	0	3	2	1	3	0
ENSG00000135747	36	18	29	21	37	33	22	24	4	0	9	1	1	2	2	7	29	14	16	35	35	24	22	20	0	2	3	7	2	4	3	7
ENSG00000236775	1	4	0	4	0	0	0	0	0	0	0	0	0	2	0	1	0	0	0	0	0	1	0	0	0	0	0	0	0	0	1	0
ENSG00000277462	279	199	235	186	152	150	111	161	101	83	313	63	190	162	178	169	314	366	419	321	536	230	316	257	97	164	66	216	151	146	220	195
ENSG00000215796	7	5	5	4	5	2	2	4	2	5	12	0	2	3	2	4	1	0	3	4	8	5	4	7	3	4	7	11	3	2	1	4
ENSG00000228982	0	0	0	0	0	0	0	1	0	0	0	0	0	0	0	0	0	0	0	1	0	2	1	0	0	0	0	0	0	0	0	0
ENSG00000188295	472	308	377	281	307	271	212	296	159	126	677	93	153	181	216	208	389	444	395	289	763	260	526	213	136	208	174	414	226	196	230	210
ENSG00000221953	8	5	8	2	7	14	21	19	0	0	1	0	0	0	1	1	4	4	1	3	10	9	5	4	1	0	1	1	0	0	0	0
ENSG00000226164	0	0	2	0	0	0	0	0	0	0	0	0	1	0	0	0	0	0	0	0	0	0	1	0	0	0	0	0	0	0	0	1
ENSG00000196418	463	304	426	321	399	437	329	412	289	213	958	145	234	206	255	271	263	329	348	459	994	374	592	368	319	374	249	693	190	279	297	314
ENSG00000224014	7	3	5	6	3	5	4	3	6	2	18	0	1	3	3	5	1	2	5	4	9	5	2	3	2	1	6	10	0	5	1	2
ENSG00000215795	12	17	10	8	14	8	9	16	0	4	15	2	11	11	8	8	4	3	0	10	28	14	8	12	2	8	7	8	2	9	11	11
ENSG00000259865	343	284	284	257	364	410	341	405	365	307	1328	190	232	205	286	265	76	95	98	210	609	300	325	337	288	340	204	715	119	239	139	266
ENSG00000227671	1816	1154	1602	1190	1929	2041	1652	2214	1552	1486	6940	1084	871	790	984	1088	497	486	519	1140	2780	1286	1765	1439	1242	1646	1358	3625	405	803	606	958
ENSG00000232347	1	1	2	1	8	0	4	2	0	1	6	0	7	0	0	0	0	1	0	1	4	2	1	0	0	0	1	9	0	1	1	4
ENSG00000214144	0	0	0	1	1	0	0	1	0	0	1	1	0	0	0	0	0	0	0	1	2	0	0	0	0	0	0	0	1	0	0	1
ENSG00000235818	0	0	0	0	0	0	0	2	0	0	0	0	0	0	0	0	0	0	0	0	0	0	0	0	0	0	0	0	0	0	0	0
ENSG00000197617	0	1	1	0	3	4	0	3	0	1	5	1	0	1	0	0	0	2	0	1	7	3	5	2	0	0	0	2	1	4	1	0
ENSG00000202079	2	1	2	4	6	7	9	5	6	5	19	5	2	2	4	3	0	1	1	4	9	7	3	8	9	3	6	15	0	1	0	0
ENSG00000162714	1988	1562	1798	1522	2726	2675	1873	2709	1483	1210	4571	944	1286	1158	1224	1479	420	605	595	1039	3603	1596	2482	1474	1127	1850	925	3159	724	1027	830	1127
ENSG00000162711	0	1	0	1	230	295	200	299	7	12	86	18	0	1	0	0	0	1	3	1	432	200	329	222	18	24	33	62	0	0	0	0
ENSG00000177535	0	0	0	0	0	0	2	0	0	0	3	0	0	0	0	0	0	0	0	0	7	1	1	2	0	0	0	0	0	0	0	0
ENSG00000203664	0	0	0	0	0	0	0	0	0	0	0	0	0	0	0	0	0	0	0	0	0	0	0	0	0	0	0	0	0	0	0	0
ENSG00000169224	0	0	0	0	4507	4549	3588	5375	0	0	0	0	1	0	0	0	0	0	1	1	8789	3306	6009	4030	0	0	1	2	0	0	0	0
ENSG00000227135	0	0	0	0	0	0	0	0	0	0	0	0	0	0	0	0	0	0	0	0	0	0	2	0	0	0	0	0	0	0	0	0
ENSG00000196242	0	0	0	0	0	1	0	1	0	0	0	0	0	0	0	0	0	0	0	0	2	0	0	0	0	0	0	0	0	0	0	0
ENSG00000236817	0	0	0	0	3	4	6	2	0	0	0	0	0	0	0	0	0	0	0	0	11	7	0	3	0	0	0	0	0	0	0	0
ENSG00000177489	0	0	0	0	4	9	5	7	0	0	0	0	0	0	0	0	0	0	0	0	10	4	0	9	0	0	0	0	0	0	0	0
ENSG00000177476	0	0	0	0	8	10	5	9	0	0	0	0	0	0	0	0	0	0	0	0	4	7	2	3	0	0	0	0	0	0	0	0
ENSG00000283369	0	0	0	0	4	1	2	3	0	0	0	0	0	0	0	0	0	0	0	0	0	0	0	1	0	0	0	0	0	0	0	0
ENSG00000198452	0	0	0	0	78	94	57	86	0	0	0	0	0	0	0	0	0	0	0	0	83	31	68	25	0	0	0	0	0	0	0	0
ENSG00000235749	0	0	0	0	5	13	2	11	0	0	0	0	0	0	0	0	0	0	0	0	6	3	4	3	0	0	0	1	0	0	0	0
ENSG00000230411	0	0	0	0	2	3	2	6	0	0	0	0	0	0	0	0	0	0	0	0	1	2	1	1	0	0	0	0	0	0	0	0
ENSG00000197437	0	0	0	0	0	1	2	3	0	0	0	0	0	0	0	0	0	0	0	0	0	0	1	1	0	0	0	0	0	0	0	0
ENSG00000239395	0	0	0	0	0	0	1	0	0	0	0	0	0	0	0	0	0	0	0	0	0	0	0	0	0	0	0	0	0	0	0	0
ENSG00000169214	0	0	0	0	1	1	0	2	0	0	0	0	0	0	0	0	0	0	0	0	1	0	0	0	0	0	0	0	0	0	0	0
ENSG00000241128	0	0	0	0	1	1	0	3	0	0	0	0	0	0	0	0	0	0	0	0	0	0	0	0	0	0	0	0	0	0	0	0
ENSG00000153230	0	0	0	0	5	5	0	7	0	0	0	0	0	0	0	0	0	0	0	0	1	0	2	2	0	0	2	0	0	0	0	0
ENSG00000221888	0	0	0	0	1	0	1	1	0	0	0	0	0	0	0	0	0	0	0	0	1	0	0	0	0	0	0	0	0	0	0	0
ENSG00000228336	0	0	0	0	0	5	1	1	0	0	0	0	0	0	0	0	0	0	0	0	0	0	0	0	0	0	0	0	0	0	0	0
ENSG00000196772	0	0	0	0	0	0	0	0	0	0	0	0	1	0	0	0	0	0	0	0	0	0	0	0	0	0	0	0	0	0	0	0
ENSG00000232908	0	0	0	0	0	0	0	0	0	0	0	0	0	0	0	0	0	0	0	0	0	0	0	0	0	0	0	0	0	0	0	0
ENSG00000230576	0	0	0	0	0	0	0	0	0	0	0	0	0	0	0	0	0	0	0	0	0	0	0	0	0	0	0	0	0	0	0	0
ENSG00000197591	0	0	0	0	0	0	0	0	0	0	0	0	0	0	0	0	0	0	0	0	0	0	0	0	0	0	0	0	0	0	0	0
ENSG00000162722	0	0	0	0	1	1	1	3	0	0	0	0	0	0	2	0	0	0	0	0	0	1	0	0	0	0	1	0	0	0	0	0
ENSG00000238243	0	0	0	0	0	0	0	0	0	0	0	0	0	0	0	0	0	0	0	0	0	0	1	0	0	0	0	0	0	0	0	0
ENSG00000177462	0	0	0	0	0	0	0	0	0	0	0	0	0	0	0	0	0	0	0	0	0	1	1	0	0	0	0	0	0	0	0	0
ENSG00000177275	0	0	0	0	0	0	0	0	0	0	0	0	0	0	0	0	0	0	0	0	0	0	0	0	0	0	0	0	0	0	0	0
ENSG00000226191	0	0	1	1	0	0	0	0	0	0	0	0	0	0	0	0	0	4	1	1	0	0	0	0	0	0	0	0	0	0	0	0
ENSG00000224830	0	0	0	0	0	0	0	0	0	0	0	0	0	0	0	0	0	0	0	0	0	0	0	0	0	0	0	0	0	0	0	0
ENSG00000279263	0	0	0	0	0	0	0	0	0	0	0	0	0	0	0	0	0	0	0	0	0	0	0	0	0	0	0	0	0	0	0	0
ENSG00000187080	0	2	0	1	0	0	0	0	0	0	0	0	0	0	0	0	0	0	1	1	0	0	0	0	0	0	0	0	0	0	0	0
ENSG00000237492	0	1	0	0	0	0	0	0	0	0	0	0	0	0	0	0	0	2	0	1	0	0	0	0	0	0	0	0	0	0	0	0
ENSG00000224227	1	5	1	2	0	0	0	0	0	0	0	0	0	0	0	0	2	0	0	1	0	0	0	0	0	0	0	0	0	0	0	0
ENSG00000199442	0	0	0	0	0	0	0	0	0	0	0	0	0	0	0	0	0	0	0	0	0	0	0	0	0	0	0	0	0	0	0	0
ENSG00000232215	0	0	2	0	0	0	0	0	0	0	0	0	0	0	0	0	0	0	0	0	0	0	0	0	0	0	0	0	0	0	0	0
ENSG00000197454	0	1	0	0	0	0	0	0	0	0	0	0	0	0	0	0	0	0	0	0	0	0	0	0	0	0	0	0	0	0	0	0
ENSG00000200085	0	0	0	0	0	0	0	0	0	0	0	0	0	0	0	0	0	0	0	0	0	0	0	0	0	0	0	0	0	0	0	0
ENSG00000203663	0	0	0	1	0	0	0	0	0	0	0	0	0	0	0	1	0	0	0	0	0	0	0	0	0	0	0	0	0	0	0	0
ENSG00000200982	0	0	0	0	0	0	0	0	0	0	0	0	0	0	0	0	0	0	0	0	0	0	0	0	0	0	0	0	0	0	0	0
ENSG00000198128	0	0	0	0	0	0	0	0	0	0	0	0	0	0	0	0	0	0	0	0	0	0	0	0	0	0	0	0	0	0	0	0
ENSG00000197067	0	0	0	0	0	0	0	0	0	1	0	0	0	0	0	0	0	0	0	0	0	0	0	0	0	0	0	0	0	0	0	0
ENSG00000196071	0	0	0	0	0	0	0	0	0	0	0	0	0	0	0	0	0	0	0	1	0	0	0	0	0	0	0	0	0	0	0	0
ENSG00000177233	0	0	0	0	0	0	0	0	0	0	0	0	0	0	0	0	0	0	0	0	0	0	0	0	0	0	0	0	0	0	0	0
ENSG00000162727	0	0	0	0	0	0	1	0	0	0	0	0	0	0	0	0	0	0	0	0	0	0	0	0	0	0	0	0	0	0	0	0
ENSG00000198601	0	0	0	0	0	0	0	0	0	0	0	0	0	0	0	0	0	0	0	0	0	0	0	0	0	0	0	0	0	0	0	0
ENSG00000228198	0	0	0	0	0	0	0	0	0	0	0	0	0	0	0	0	0	0	0	0	0	0	0	0	0	0	0	0	0	0	0	0
ENSG00000171180	0	0	0	0	0	0	0	0	0	0	0	0	0	0	0	0	0	0	0	0	0	0	0	0	0	0	0	0	0	0	0	0
ENSG00000177212	0	0	0	0	0	0	0	0	0	0	0	0	0	0	0	0	0	0	0	0	0	0	0	0	0	0	0	0	0	0	1	0
ENSG00000177201	0	0	0	0	0	0	0	0	0	0	0	0	0	0	0	0	0	0	0	0	0	0	0	0	0	0	0	0	0	0	0	0
ENSG00000177186	0	0	0	0	0	0	0	0	0	0	0	0	0	0	0	0	0	0	0	0	0	0	0	0	0	0	0	0	0	0	0	0
ENSG00000177174	0	0	0	0	0	0	0	0	0	0	0	0	0	0	0	0	0	0	0	0	0	0	0	0	0	0	0	0	0	0	0	0
ENSG00000196944	0	0	0	1	0	0	0	0	0	0	0	0	0	0	0	0	0	0	0	0	0	0	0	0	0	0	0	0	0	0	0	0
ENSG00000198104	0	0	0	0	0	0	0	0	0	0	0	0	0	0	0	0	0	0	0	0	0	0	0	0	0	0	0	0	0	0	0	0
ENSG00000175143	0	0	0	0	0	0	0	0	0	0	0	0	0	1	0	0	0	0	0	0	0	0	0	0	0	0	0	0	0	0	0	0
ENSG00000227152	0	0	0	0	0	0	0	0	0	0	0	0	0	0	0	0	0	0	0	0	0	0	0	0	0	0	0	0	0	0	0	0
ENSG00000196240	0	0	0	0	0	0	0	0	0	0	0	0	0	0	0	0	0	0	0	0	0	0	0	0	0	0	0	0	0	0	0	0
ENSG00000196539	0	0	0	0	0	0	0	0	0	0	0	0	0	2	0	0	0	0	0	0	0	0	0	0	0	0	0	0	0	1	0	0
ENSG00000229255	0	0	0	0	0	0	0	0	0	0	2	0	3	1	3	2	0	0	0	0	0	0	0	0	0	0	1	2	0	1	3	2
ENSG00000203661	0	0	0	0	0	0	0	0	0	0	0	0	0	0	0	0	0	0	0	0	0	0	0	0	0	0	0	0	0	0	0	0
ENSG00000271934	0	0	0	0	0	0	0	0	0	0	0	0	0	0	0	0	0	0	0	0	0	0	0	0	0	0	0	0	0	0	0	0
ENSG00000188558	0	0	0	0	0	0	0	0	0	0	0	0	0	0	0	0	0	0	0	0	0	0	0	0	0	0	0	0	0	0	1	0
ENSG00000224521	0	0	0	0	0	0	0	0	0	0	0	0	0	0	0	1	0	0	0	0	1	0	0	0	0	0	0	0	0	2	0	3
ENSG00000227102	0	0	0	0	0	0	0	0	0	0	0	0	0	0	0	0	0	0	0	0	0	0	0	0	0	0	0	0	0	0	0	0
ENSG00000182783	0	0	0	0	0	0	0	0	0	0	0	0	0	0	0	0	0	0	0	0	0	0	0	0	0	0	0	0	0	0	0	0
ENSG00000183310	0	0	0	0	0	0	0	0	0	0	0	0	0	0	0	0	0	0	0	0	0	0	0	0	0	0	0	0	0	0	0	0
ENSG00000184022	0	0	0	0	0	0	0	0	0	0	0	0	0	0	0	0	0	0	0	0	0	0	0	0	0	0	0	0	0	0	0	0
ENSG00000201602	0	0	0	0	0	0	0	0	0	0	0	0	0	0	0	0	0	0	0	0	0	0	0	0	0	0	0	0	0	0	0	0
ENSG00000271701	0	0	0	0	0	0	0	0	0	0	0	0	0	0	0	0	0	0	0	0	0	0	0	0	0	0	0	0	0	0	0	0
ENSG00000279301	0	0	0	0	0	0	0	0	0	0	0	0	0	0	0	0	0	0	0	0	0	0	0	0	0	0	0	0	0	0	0	0
ENSG00000177151	0	0	0	0	0	0	0	0	0	0	0	0	0	0	0	0	0	0	0	0	0	0	0	0	0	0	0	0	0	0	0	0
ENSG00000187701	0	0	0	0	0	0	0	0	0	0	0	0	0	0	0	0	0	0	0	0	0	0	0	0	0	0	0	0	0	0	0	0
ENSG00000189181	0	0	0	0	0	0	0	0	0	0	0	0	0	0	0	0	0	0	0	0	0	0	0	0	0	0	0	0	0	1	0	0
ENSG00000229703	0	0	0	0	0	0	0	0	0	0	0	0	0	0	0	0	0	0	0	0	0	0	0	0	0	0	0	0	0	0	0	0
ENSG00000276520	0	0	0	0	0	0	0	0	0	0	0	0	0	0	0	0	0	0	0	0	0	0	0	0	0	0	1	0	0	0	0	0
ENSG00000259823	1	1	1	3	0	0	0	0	0	0	5	0	0	0	0	0	1	4	4	3	0	0	0	0	0	0	2	0	0	0	0	0
ENSG00000242529	0	0	0	0	0	0	0	0	0	0	0	0	0	0	0	0	0	0	0	0	0	0	0	0	0	0	0	0	0	0	0	0
ENSG00000229663	0	0	0	0	0	0	0	0	0	0	1	0	0	1	0	0	0	0	0	0	0	0	0	0	0	1	0	0	0	0	0	0
ENSG00000175137	1161	908	988	949	1107	1164	769	1218	769	683	3146	627	584	610	556	747	374	615	519	738	1640	702	1224	589	751	1254	739	2257	514	622	476	612
ENSG00000264500	0	0	0	0	0	0	0	0	0	0	0	0	0	0	0	0	0	0	0	0	0	0	0	0	0	0	0	0	0	0	0	0
ENSG00000171161	1309	1068	1232	1054	1308	1405	953	1423	785	708	2951	615	524	577	647	778	409	581	589	743	1980	870	1654	771	661	1212	567	2176	460	537	415	640
ENSG00000171163	1753	1586	1648	1662	1957	2129	1613	2215	1215	913	3539	669	927	918	916	1175	1077	1061	822	1773	2450	1190	1689	1276	606	1081	609	1633	530	924	485	931
ENSG00000227237	2	2	4	2	3	2	1	2	0	0	4	0	2	2	0	4	3	0	1	3	3	2	3	1	0	0	2	1	1	6	1	3
ENSG00000185220	120	115	137	118	322	311	227	335	261	215	1075	162	146	182	233	248	84	110	90	150	589	213	389	201	185	228	203	564	176	252	206	244
ENSG00000200495	0	0	2	0	2	0	0	2	1	1	2	0	0	1	2	1	0	0	0	1	0	1	1	0	0	3	0	3	0	1	1	0
ENSG00000233084	1	0	3	2	5	3	6	0	3	0	11	1	1	5	4	5	1	3	1	1	13	1	5	2	0	1	1	4	5	7	3	6
ENSG00000184731	888	717	703	722	0	0	0	0	0	0	4	2	0	0	0	0	188	299	258	540	1	1	1	1	0	0	0	0	1	0	0	0
ENSG00000227061	0	0	0	0	0	0	0	0	0	0	1	0	0	0	0	0	0	0	0	0	0	0	0	0	0	0	0	0	0	0	2	0
ENSG00000035115	1535	1173	1410	1209	319	357	246	355	1050	944	3491	471	573	588	574	772	885	1192	1063	1718	564	237	328	222	751	1089	809	1899	270	441	338	536
ENSG00000143727	4707	3362	3621	3279	1643	1716	1212	1884	2546	1973	7406	1439	4396	4063	4523	5187	5065	6568	6095	5080	4644	1755	3243	2020	2386	3211	1546	4821	4962	4874	6127	6180
ENSG00000189292	9	9	8	10	13	16	6	21	4	0	22	0	4	15	5	8	5	6	4	6	24	11	12	9	6	3	2	17	7	3	5	4
ENSG00000228643	14	16	22	11	11	11	11	22	2	2	14	0	2	4	0	3	10	11	8	15	27	15	22	16	1	2	0	4	2	2	3	3
ENSG00000235779	4	5	12	11	7	7	2	7	0	0	4	0	1	1	0	1	4	0	2	5	4	4	3	0	0	0	0	0	0	0	0	0
ENSG00000277997	0	1	1	3	0	0	1	0	0	0	1	0	2	0	0	0	0	0	0	1	2	0	0	0	0	0	0	0	0	0	0	0
ENSG00000233684	3	4	3	1	3	1	1	5	2	1	1	1	2	1	0	2	0	0	0	1	1	1	2	0	0	0	0	3	0	1	0	0
ENSG00000236856	1	2	10	2	0	0	0	0	0	0	0	0	3	1	1	0	2	2	4	10	0	0	0	1	0	0	0	0	1	2	0	3
ENSG00000226277	1	0	3	0	0	0	0	0	0	0	0	0	0	0	0	0	3	0	0	0	0	0	0	0	0	0	0	0	0	0	0	0
ENSG00000223985	0	0	0	0	0	0	0	0	0	0	0	0	0	0	0	0	0	0	0	0	0	0	0	0	0	0	0	0	0	0	0	0
ENSG00000225942	0	0	0	0	0	0	0	0	0	0	0	0	0	0	0	0	0	0	0	0	0	0	0	0	0	0	0	0	0	0	0	0
ENSG00000233633	0	0	0	0	0	0	0	0	0	0	0	0	0	0	0	0	0	0	0	0	0	0	0	0	0	0	0	0	0	0	0	0
ENSG00000151353	1880	1567	1505	1567	641	661	574	790	595	520	1899	393	811	813	775	1025	1768	2296	1829	1846	1500	605	1130	601	565	795	408	1310	797	880	775	1039
ENSG00000233296	37	27	31	37	15	24	15	27	13	5	35	5	41	25	16	24	18	23	23	40	29	11	17	9	5	11	4	17	7	16	18	24
ENSG00000280360	1	0	1	0	0	0	0	2	1	0	1	0	2	4	4	4	0	0	0	2	0	0	0	0	0	0	0	0	1	0	0	3
ENSG00000233970	1	0	0	0	0	0	0	0	0	0	0	0	3	0	0	0	0	0	0	1	0	0	0	0	0	0	0	0	0	0	0	0
ENSG00000227713	0	0	0	0	0	0	0	0	0	0	0	0	0	0	0	0	0	0	0	0	0	0	0	0	0	0	1	0	0	0	0	0
ENSG00000272342	5	3	4	7	0	0	0	0	0	0	0	0	0	0	0	0	3	1	0	5	0	0	0	0	0	0	0	0	0	0	0	0
ENSG00000231173	0	1	0	1	0	0	0	0	0	0	0	0	0	0	0	0	0	0	2	0	0	0	0	0	0	0	0	0	0	0	0	0
ENSG00000223751	7	4	0	4	0	0	0	0	0	0	0	0	0	0	0	0	1	1	1	0	0	0	0	0	0	0	0	0	0	0	0	0
ENSG00000237667	34	20	51	26	2	3	0	1	0	0	0	0	0	0	0	0	16	12	20	37	10	1	0	0	0	0	0	0	0	0	0	0
ENSG00000228799	16	15	18	9	0	0	0	0	0	0	0	0	0	0	0	0	17	26	17	36	0	0	0	0	0	0	0	0	0	0	0	0
ENSG00000234796	11	13	13	11	0	0	0	0	0	0	0	0	0	0	0	0	2	7	0	23	0	0	0	0	0	0	0	0	0	0	0	0
ENSG00000235688	461	434	436	508	10	12	12	16	0	0	0	0	0	0	0	0	105	159	84	257	7	5	6	10	0	0	0	0	0	0	0	0
ENSG00000172554	122	152	154	164	1	1	1	2	0	0	1	0	0	0	0	0	32	75	69	103	1	0	4	2	0	0	0	0	1	2	0	0
ENSG00000235403	1	4	0	1	0	0	0	0	0	0	0	0	0	0	2	0	1	0	0	2	0	0	0	0	0	0	0	0	0	0	0	0
ENSG00000236665	11	9	16	11	0	0	0	0	0	0	0	0	0	0	0	0	2	8	6	13	0	0	0	0	0	0	0	0	0	0	0	0
ENSG00000233553	1	0	0	2	0	0	0	0	0	0	0	0	0	0	0	0	0	0	1	0	0	0	0	0	0	0	0	0	0	0	0	0
ENSG00000115705	59	48	48	48	6	7	6	8	0	0	3	2	0	0	0	0	6	13	8	21	9	4	5	1	0	1	0	0	0	0	0	0
ENSG00000228613	1	2	2	0	3	11	7	7	0	0	0	0	0	0	0	0	0	0	2	0	6	1	1	2	0	0	0	0	0	0	0	0
ENSG00000231482	0	0	0	0	0	0	0	0	0	0	0	0	0	0	0	0	0	0	0	0	0	0	1	0	0	0	0	0	0	0	0	0
ENSG00000203635	0	0	1	0	13	11	7	17	0	0	0	0	0	0	0	0	0	0	1	0	10	4	8	1	0	0	0	0	0	0	0	0
ENSG00000130508	3034	2921	2898	2970	6265	6300	4234	6457	1429	1232	4957	913	2	0	0	0	483	914	678	1593	7619	3096	5060	2495	1081	1820	863	2683	1	0	0	0
ENSG00000186487	14	6	23	3	201	160	185	193	12	10	42	15	0	0	0	0	9	14	24	19	388	131	264	160	29	24	4	15	1	0	0	0
ENSG00000232057	3	0	0	1	0	0	0	0	0	0	0	0	0	0	0	0	0	2	0	1	0	0	1	0	0	0	0	0	0	0	0	0
ENSG00000225619	0	0	0	0	0	0	0	0	0	0	0	0	0	0	0	0	0	0	0	0	0	0	0	0	0	0	0	0	0	0	0	0
ENSG00000234929	0	0	0	0	0	0	0	0	0	0	0	0	0	0	0	0	0	0	0	0	0	0	0	0	0	0	0	0	0	0	0	0
ENSG00000227364	0	0	0	0	0	0	0	0	0	0	0	0	0	0	0	0	0	0	0	0	0	0	0	0	0	0	0	0	0	0	0	0
ENSG00000237720	0	0	0	0	0	0	0	0	0	0	0	0	0	0	0	0	0	0	0	0	0	0	0	0	0	0	0	0	0	0	0	0
ENSG00000228391	0	0	0	0	0	0	0	0	0	0	0	0	0	0	0	0	0	0	0	0	0	0	0	0	0	0	0	0	0	0	0	0
ENSG00000234423	3	0	0	1	1	3	1	3	0	0	2	0	2	2	4	0	0	0	0	0	0	0	0	0	0	0	0	2	2	1	1	4
ENSG00000236760	0	0	4	1	4	3	3	2	1	0	3	1	6	4	7	3	0	0	2	2	0	0	3	1	0	1	1	4	0	4	3	5
ENSG00000226649	6	5	4	2	2	1	2	6	2	3	12	0	11	5	9	9	4	0	3	4	2	3	1	0	1	9	4	6	3	2	12	5
ENSG00000032389	1521	1071	1231	1047	896	933	644	915	753	566	2260	475	1710	1502	1755	2047	1019	1630	1531	1243	1843	713	1496	644	646	935	371	1483	1764	1688	1953	1845
ENSG00000224885	8	4	14	7	4	3	3	5	5	7	23	2	10	14	8	7	10	3	5	22	9	10	9	8	9	8	3	20	4	18	11	10
ENSG00000171853	1596	1157	1466	1212	934	954	716	1012	730	605	2868	515	1622	1434	1724	1865	483	499	556	1205	1419	724	985	607	616	1100	749	1984	1044	1577	1256	1730
ENSG00000225234	49	28	46	37	35	28	31	25	14	15	90	19	24	21	23	26	14	12	9	30	32	35	28	18	8	25	15	48	15	29	26	35
ENSG00000271868	0	5	11	4	4	8	1	4	1	6	10	1	6	9	8	4	0	0	2	4	10	8	10	1	4	5	4	3	3	2	5	3
ENSG00000182551	1576	1068	1517	1200	1670	1752	1268	1721	1145	911	4748	754	3296	3008	3223	3971	1611	2151	2515	2206	4305	1757	3329	1605	1183	1456	1070	2833	3170	3584	3532	3875
ENSG00000235078	72	53	78	60	10	13	7	8	6	4	37	7	24	27	23	41	56	96	76	111	22	5	12	3	9	14	13	18	21	29	23	22
ENSG00000242282	85	75	71	82	107	102	73	111	16	20	79	15	111	94	91	96	53	60	55	95	193	107	153	73	24	31	19	56	66	79	84	114
ENSG00000255767	0	0	0	0	0	0	1	0	0	0	0	0	0	0	0	0	0	0	0	2	0	0	0	0	0	0	0	0	0	0	0	0
ENSG00000171865	2236	1409	1844	1515	733	765	606	802	786	552	2631	414	2189	1741	2156	2222	1240	1471	1378	1750	1736	684	1259	671	774	1235	495	1751	1948	2236	1967	2298
ENSG00000234171	241	146	246	210	105	78	84	100	239	145	623	125	503	530	520	690	204	266	238	342	242	111	193	79	190	216	111	359	617	542	603	603
ENSG00000171863	20748	14029	14536	13359	9233	8826	6508	9416	14947	10659	42152	8476	31716	27846	31671	36668	27305	29689	25304	27426	24165	9016	16009	12711	13156	18694	8269	27241	41460	34687	45837	47700
ENSG00000118004	1	3	5	7	11	10	10	12	7	7	19	9	68	58	64	93	3	1	6	5	8	8	16	12	3	7	5	22	47	68	77	75
ENSG00000237370	0	0	0	0	0	0	1	3	0	0	0	0	7	2	0	2	0	0	0	1	0	0	0	0	0	0	0	0	0	0	0	2
ENSG00000188765	2	0	1	0	0	0	0	0	0	0	1	0	0	0	2	1	1	0	2	0	0	2	0	0	0	0	0	3	0	0	0	1
ENSG00000151360	0	0	2	0	0	1	0	1	0	0	2	0	1	2	0	1	0	0	0	0	0	0	0	0	0	0	0	0	0	0	1	0
ENSG00000232718	0	0	0	0	0	0	0	0	0	0	0	0	0	0	0	0	0	0	0	0	0	0	0	0	0	0	0	0	0	0	0	1
ENSG00000224661	0	0	0	0	0	0	0	0	0	0	0	0	0	0	0	0	0	0	0	0	0	0	0	0	0	0	0	0	0	0	0	0
ENSG00000214866	0	0	0	0	0	0	0	0	0	0	0	0	0	0	0	0	0	0	0	0	2	0	0	1	0	0	0	0	0	0	0	0
ENSG00000227363	0	0	0	0	0	0	0	2	0	0	0	0	0	0	0	0	0	0	0	0	0	0	0	0	0	0	0	0	0	0	0	0
ENSG00000237401	0	0	0	0	0	0	0	0	0	0	4	0	0	0	0	0	0	0	0	0	0	0	2	0	0	0	2	0	0	0	0	0
ENSG00000229550	0	0	0	0	0	0	0	0	0	0	0	0	0	0	0	0	0	0	0	0	0	0	0	0	0	0	0	0	0	0	0	0
ENSG00000231821	0	0	0	0	0	0	0	0	0	0	0	0	0	0	0	0	0	0	0	0	0	0	0	0	0	0	0	0	0	0	0	0
ENSG00000231532	5	1	0	0	0	0	0	0	0	0	1	0	1	0	0	0	0	0	0	1	0	0	0	0	0	0	0	0	0	0	0	0
ENSG00000252238	0	0	0	0	0	0	0	0	0	0	0	0	0	0	0	0	0	0	0	0	0	0	0	0	0	0	0	0	0	0	0	0
ENSG00000207192	0	0	0	0	0	0	0	0	0	0	0	0	0	0	0	0	0	0	0	0	0	0	0	0	0	0	0	0	0	0	0	0
ENSG00000228585	0	0	0	0	0	0	0	0	0	0	0	0	0	0	0	0	0	0	0	0	0	0	0	0	0	0	0	0	0	0	0	0
ENSG00000232835	0	0	0	0	0	0	0	0	0	0	0	0	0	0	0	0	0	0	0	0	0	0	0	0	0	0	0	0	0	0	0	0
ENSG00000224128	1	0	3	0	0	0	0	0	0	0	0	0	0	0	0	0	0	1	0	3	0	0	0	0	0	0	0	0	0	0	0	0
ENSG00000230090	2	0	1	0	0	0	0	0	0	0	0	0	0	0	0	0	0	0	3	6	0	0	0	0	0	0	0	0	0	0	0	0
ENSG00000176887	250	172	206	166	0	0	0	0	0	0	3	0	1	0	0	0	119	80	101	262	0	0	0	0	0	0	4	0	0	0	0	0
ENSG00000242540	56	24	41	25	0	0	0	0	0	0	0	0	0	0	0	0	38	43	61	80	0	0	0	0	0	0	0	0	0	0	0	0
ENSG00000236106	14	6	16	6	0	0	0	0	0	0	0	0	0	0	0	0	2	14	4	14	0	0	0	0	0	0	0	0	0	0	0	0
ENSG00000237266	0	0	0	0	0	0	0	0	0	0	0	0	0	0	0	0	0	0	0	0	0	0	0	0	0	0	0	0	0	0	0	0
ENSG00000232044	0	0	0	0	0	0	0	0	0	0	1	0	0	0	0	0	0	0	4	1	0	0	0	0	0	0	1	0	0	1	0	0
ENSG00000276733	0	0	0	0	0	0	0	0	0	0	0	0	0	0	0	0	0	0	0	0	0	0	0	0	0	0	0	0	0	0	0	0
ENSG00000234275	0	0	0	0	0	0	0	0	0	0	0	0	0	0	0	0	0	0	0	0	0	0	0	0	0	0	0	0	0	0	0	0
ENSG00000227007	0	0	0	0	0	0	0	0	0	1	2	0	1	0	0	1	0	0	0	0	0	0	0	0	0	0	0	0	0	0	1	0
ENSG00000231610	0	0	0	0	0	0	0	0	0	0	0	0	0	0	0	0	0	0	0	0	0	0	0	0	0	0	0	0	0	0	0	0
ENSG00000226488	0	0	0	0	0	0	0	0	0	0	0	0	0	0	0	0	0	0	0	0	0	0	0	0	0	0	0	0	0	0	0	0
ENSG00000236172	0	0	0	0	0	0	0	0	0	0	0	0	0	1	0	0	0	0	0	0	0	0	0	0	0	0	0	0	0	0	0	0
ENSG00000283394	0	0	0	0	0	0	0	0	0	0	0	0	0	0	0	0	0	0	0	0	0	0	0	0	0	0	0	0	0	0	0	0
ENSG00000205837	0	0	0	0	1	0	0	0	1	0	0	0	0	0	0	0	0	0	0	0	3	0	1	0	0	2	2	1	2	0	1	0
ENSG00000225964	11	21	21	8	13	14	9	23	12	15	72	7	9	11	6	16	2	6	4	21	33	15	24	20	18	24	25	55	9	16	10	26
ENSG00000134326	371	299	304	334	331	308	243	316	668	632	2382	443	845	882	744	996	214	447	429	470	811	295	604	299	1023	1670	828	2542	1448	2051	1341	1963
ENSG00000134321	107	102	91	105	44	42	41	51	320	337	1853	193	198	213	237	306	62	108	64	125	87	45	54	42	514	655	1274	1616	177	209	367	302
ENSG00000271947	28	11	9	6	0	0	0	0	120	128	488	68	42	46	51	55	6	4	2	11	0	0	0	0	112	130	140	313	34	71	59	66
ENSG00000228203	116	80	101	94	4	0	0	0	952	833	4272	655	256	256	306	359	33	51	34	65	0	1	2	0	867	1321	1281	2980	313	471	321	523
ENSG00000151692	1095	1091	859	1053	402	454	284	513	3142	2808	15005	2238	1781	1802	1401	2195	246	431	309	663	545	255	395	229	3680	5045	6019	11685	1422	2141	1317	2143
ENSG00000223884	5	6	5	7	2	7	6	7	18	16	131	17	24	16	8	17	1	1	0	5	7	8	8	4	34	27	59	112	8	22	8	35
ENSG00000223145	2	0	1	1	0	0	0	0	0	0	4	0	2	3	0	1	1	0	0	1	2	0	1	0	3	2	11	13	0	4	1	6
ENSG00000272002	2	2	1	3	3	1	1	0	9	8	34	2	6	3	5	3	2	0	4	5	4	0	0	1	10	18	22	37	3	7	1	5
ENSG00000213774	0	0	1	0	0	0	0	0	0	0	0	0	2	0	0	0	0	0	0	0	0	0	0	0	0	0	3	0	0	0	0	1
ENSG00000229727	3	4	0	2	2	2	2	1	9	9	34	2	0	2	0	3	1	1	0	2	1	0	1	0	8	14	6	20	0	0	0	1
ENSG00000221255	0	0	0	0	0	0	0	0	0	0	0	0	0	0	0	0	0	0	0	0	0	0	0	0	0	0	0	0	0	0	0	0
ENSG00000230515	0	0	0	0	0	0	0	0	5	3	27	4	0	0	0	0	0	0	0	0	0	0	0	0	14	9	0	18	0	0	0	0
ENSG00000235576	0	0	0	0	0	0	0	0	766	488	7207	873	0	0	0	0	0	0	0	0	0	0	0	0	907	1255	1708	4247	0	0	0	0
ENSG00000232979	0	0	0	0	0	0	0	0	11	5	76	8	0	0	0	0	0	0	0	0	0	0	0	0	19	14	27	57	0	0	0	0
ENSG00000229405	0	0	0	0	0	0	0	0	15	12	79	14	0	0	0	0	0	0	0	0	0	0	0	0	20	14	31	73	0	0	0	0
ENSG00000226506	0	0	0	0	0	0	0	0	0	0	0	0	0	2	0	0	0	0	0	1	0	0	1	0	0	0	2	0	0	0	0	0
ENSG00000235665	0	0	0	0	3	1	1	1	3	2	11	2	0	0	1	2	0	0	0	0	0	1	0	0	1	10	1	10	1	1	0	1
ENSG00000236790	0	0	0	0	5	1	3	0	39	66	197	23	7	15	8	10	0	1	0	1	0	3	3	0	36	38	34	100	5	11	8	11
ENSG00000229740	0	0	0	0	0	0	0	0	0	0	1	0	0	0	0	0	0	0	0	0	0	0	0	0	0	0	0	4	0	1	0	2
ENSG00000231435	0	0	1	0	7	1	0	0	7	5	26	4	1	0	0	1	0	0	0	0	4	2	3	4	1	7	13	25	0	1	0	1
ENSG00000236008	2	0	1	0	3	10	1	8	26	18	141	22	5	1	6	5	0	0	0	0	2	7	3	3	17	31	30	119	0	1	5	1
ENSG00000231083	0	0	0	0	0	1	0	0	0	3	23	4	0	0	0	0	0	0	0	0	0	1	0	0	4	7	9	17	0	0	0	1
ENSG00000225642	0	0	0	0	0	0	0	0	0	1	6	0	0	1	0	0	0	0	0	0	0	0	0	0	0	0	2	3	0	0	0	0
ENSG00000235092	25	10	38	14	13	28	14	30	8	6	62	5	33	18	16	31	7	9	10	8	23	16	17	13	7	13	9	43	7	18	12	20
ENSG00000115738	113	125	151	171	734	736	493	865	4473	4916	28968	4777	437	479	521	631	100	193	259	184	2000	656	1324	948	6369	9768	7909	21081	791	792	982	1068
ENSG00000260837	15	9	17	24	24	31	16	35	15	9	59	8	21	35	16	54	0	1	2	7	20	8	5	9	6	14	37	12	8	19	7	16
ENSG00000134313	2461	2082	2290	2133	2849	2887	2234	3200	1962	1862	9906	1527	2536	2614	2819	3425	706	1121	1053	1924	5169	2048	3725	1822	1916	2882	2931	6253	1795	2910	2299	3252
ENSG00000143797	1161	1249	1077	1177	1872	1959	1556	2143	2340	2036	7712	1449	1054	1496	1063	1875	507	925	775	1123	4966	2128	3428	1966	2166	3105	1898	5345	670	1338	702	1477
ENSG00000230886	0	0	0	0	1	0	0	0	0	0	1	0	0	1	0	0	0	0	0	0	0	0	0	0	0	0	0	1	0	0	0	0
ENSG00000223640	0	0	0	0	0	0	0	0	0	0	2	0	0	0	0	1	0	0	0	0	0	0	0	0	0	0	0	0	0	0	0	0
ENSG00000242136	0	0	1	0	0	0	0	0	0	0	0	0	0	0	0	0	0	0	0	0	0	0	0	0	0	0	0	0	0	0	0	0
ENSG00000261104	0	0	0	0	0	0	0	0	0	0	0	0	0	0	0	0	0	0	0	0	1	0	0	0	0	0	0	0	0	0	0	0
ENSG00000244310	0	0	0	1	0	0	0	0	0	0	0	0	0	0	0	0	0	0	0	0	0	0	0	0	0	0	0	0	0	1	0	0
ENSG00000240980	0	0	0	0	0	0	0	0	0	0	0	0	0	0	0	0	0	0	0	0	0	0	0	0	0	0	1	0	0	0	0	0
ENSG00000151693	258	366	224	333	980	943	683	980	484	427	2257	388	14	68	21	74	63	101	43	249	1575	696	926	597	334	636	536	1590	6	38	9	38
ENSG00000213772	0	0	0	1	2	1	0	1	0	1	3	1	0	0	0	0	0	0	1	1	1	4	1	1	0	1	0	2	0	0	0	1
ENSG00000119185	1497	1007	1268	1140	1072	1038	862	1178	1406	1017	4096	819	2073	1932	2193	2740	1446	1819	1953	1766	2476	1016	1871	996	1251	1893	852	2797	2361	2433	2626	2760
ENSG00000119203	2284	1689	1877	1590	1664	1730	1260	1827	1478	1058	4570	857	3435	3056	3519	3905	2189	2789	2555	1983	4220	1525	2959	1417	1408	2123	913	3252	3473	3478	4178	4184
ENSG00000134330	883	549	728	589	594	609	428	705	593	479	2055	371	1056	939	970	1170	894	1218	1102	894	1312	533	924	521	524	781	549	1408	986	987	1096	1147
ENSG00000151694	1260	828	980	857	1030	1143	839	1194	797	687	3182	520	1178	1269	1365	1408	595	614	561	737	2299	917	1624	811	764	1195	707	2079	915	1228	1169	1361

ENSG00000239300	19	11	14	15	22	16	9	12	12	19	83	5	18	26	20	22	3	4	8	23	16	10	14	8	22	21	16	42	5	26	7	21
ENSG00000271855	39	29	42	19	19	15	2	19	26	15	85	9	27	25	25	32	18	17	18	17	19	14	6	5	21	20	15	39	20	19	14	23
ENSG00000134308	10696	7546	9242	7663	11757	11048	8371	12012	8060	6504	24554	4955	15794	14207	16003	18606	7060	10499	9863	9764	24788	8772	17318	8038	7744	11306	5243	17139	16109	17494	17121	20632
ENSG00000207086	4	11	4	3	1	6	4	4	4	1	16	1	3	4	7	3	0	0	2	3	12	5	6	6	8	5	11	15	1	5	6	4
ENSG00000240687	0	0	0	0	0	0	0	0	0	0	0	0	0	0	0	2	0	0	0	0	0	0	0	0	0	0	0	0	0	0	0	0
ENSG00000244260	0	0	0	0	0	0	0	0	0	0	0	0	0	2	2	1	0	0	0	0	0	0	0	0	0	0	0	0	2	0	1	0
ENSG00000200034	0	0	0	0	0	0	0	0	0	0	0	0	1	0	1	0	0	0	0	0	0	0	0	0	0	0	0	0	0	0	0	0
ENSG00000240960	0	0	0	0	0	0	0	0	0	0	0	0	0	0	1	0	0	0	0	0	0	0	0	0	0	0	0	0	0	0	0	0
ENSG00000243491	0	0	1	0	10	1	3	2	3	3	4	0	16	18	4	22	0	0	1	0	13	2	9	2	0	0	0	0	2	3	3	2
ENSG00000115750	970	681	763	656	509	560	425	615	509	409	1930	362	1110	1083	1277	1406	738	948	836	901	1198	480	765	457	597	864	452	1590	1121	1245	1339	1506
ENSG00000269973	100	67	86	80	81	78	47	76	86	67	306	44	135	161	149	193	52	49	61	71	96	54	77	40	84	113	72	253	112	167	114	136
ENSG00000134317	194	226	149	212	177	200	134	193	7	6	44	11	234	251	194	301	67	111	80	169	372	134	200	178	10	21	5	69	115	276	132	303
ENSG00000188525	0	0	1	2	0	1	0	0	0	0	0	0	1	1	1	2	0	0	0	0	1	0	1	0	0	0	0	0	0	0	1	0
ENSG00000260476	0	0	0	0	0	0	0	0	0	0	3	0	0	0	0	0	0	0	0	0	0	0	0	0	0	0	1	0	0	0	0	0
ENSG00000260077	16	34	13	40	0	0	0	0	4	7	15	4	99	101	53	105	7	5	0	22	0	0	0	0	3	2	2	17	17	22	19	29
ENSG00000172059	177	179	165	183	0	0	0	0	99	90	310	70	1385	1153	1063	1353	45	103	67	144	0	0	0	0	70	136	51	275	545	687	493	680
ENSG00000271787	1	0	0	0	0	0	0	0	0	0	1	0	0	2	2	2	0	0	0	0	0	0	0	0	0	0	0	1	0	1	0	0
ENSG00000205795	0	0	1	0	0	0	0	2	7	17	27	16	88	139	97	203	0	0	0	0	2	0	0	0	0	32	3	69	55	135	102	166
ENSG00000233502	0	0	0	0	0	0	0	0	0	0	0	0	0	2	0	2	0	0	0	0	1	0	0	0	0	0	0	1	1	0	2	1
ENSG00000212558	0	0	0	0	0	0	0	0	0	0	0	0	2	0	0	0	0	0	0	0	0	0	0	0	0	0	0	0	0	0	0	0
ENSG00000272524	26	23	30	35	33	22	15	22	16	10	45	13	36	40	46	57	19	36	28	41	41	10	26	15	14	24	1	28	30	44	40	64
ENSG00000171848	9956	7998	9601	7936	3936	4062	3625	4555	9654	8378	25057	6073	13128	12830	14790	17845	3936	5977	7286	8157	8653	3321	6748	3034	9827	15116	3359	26560	11734	14768	13980	16954
ENSG00000163009	564	493	445	594	585	601	422	578	244	153	502	126	365	327	340	431	98	116	85	369	672	331	337	348	159	200	55	469	120	313	206	384
ENSG00000206647	39	36	27	22	24	35	24	57	19	22	55	13	39	32	37	29	13	6	5	16	53	37	38	25	12	17	8	56	13	24	38	40
ENSG00000265418	19	16	12	25	28	36	26	35	8	6	32	7	12	25	14	19	1	0	0	11	37	20	14	33	5	11	3	31	4	10	8	16
ENSG00000264030	0	0	0	0	0	0	0	0	0	0	1	0	0	0	0	0	0	0	0	0	0	0	0	0	0	0	0	0	0	0	0	0
ENSG00000206898	1	0	1	0	1	3	0	2	0	0	0	0	0	0	0	0	0	0	0	3	1	0	0	1	1	0	2	0	0	0	0	0
ENSG00000115756	1265	1135	1112	1195	1434	1396	1008	1409	2288	1745	4572	1190	1551	1848	1360	2204	215	461	335	715	1380	599	967	510	982	1632	673	2275	718	944	630	1032
ENSG00000115758	7032	5202	6052	5595	4791	4525	3666	4701	4665	3413	12720	2509	15498	12117	19018	16389	7836	14432	11615	9978	14175	5421	10768	4669	4517	6826	1911	7678	21042	19176	26465	21355
ENSG00000206633	6	0	3	8	7	2	3	5	5	6	15	4	12	12	7	10	0	0	0	4	10	1	7	4	0	7	4	9	6	9	10	12
ENSG00000257135	70	57	66	71	37	42	30	42	10	5	16	8	96	93	73	126	26	50	27	63	72	29	55	35	0	3	11	6	62	80	51	93
ENSG00000217258	9	4	10	13	5	5	7	9	2	0	0	0	21	18	32	26	0	3	2	8	10	9	4	6	0	0	0	1	10	22	16	15
ENSG00000115761	1220	734	1085	835	823	813	699	880	822	654	2536	503	2760	2431	2919	2909	1280	1974	1657	1569	2181	840	1651	787	937	1412	586	2031	3564	3295	3623	3841
ENSG00000234818	0	3	2	5	3	1	1	2	3	3	7	1	6	5	11	11	1	1	1	4	8	10	2	3	7	12	2	16	2	4	8	7
ENSG00000243819	24	9	31	21	7	6	7	11	20	13	101	15	35	28	38	30	16	17	10	26	14	2	12	13	12	18	13	21	27	38	30	51
ENSG00000277446	0	0	0	0	0	0	0	0	0	0	0	0	0	0	0	0	0	0	0	0	0	0	0	0	0	0	0	0	0	0	0	0
ENSG00000236162	0	0	0	0	0	0	0	1	0	0	0	1	0	0	0	0	0	0	0	0	0	0	0	0	0	0	0	0	0	0	0	0
ENSG00000143882	76	86	60	49	147	156	98	173	113	82	261	53	174	184	153	210	35	58	41	55	250	94	116	113	72	102	47	178	92	138	92	206
ENSG00000239053	0	0	0	0	0	0	0	0	0	0	0	0	0	0	0	0	0	0	0	0	0	0	0	0	0	0	0	0	0	0	0	0
ENSG00000272275	1	1	1	5	7	7	13	9	0	1	2	4	36	32	23	42	0	2	0	5	21	4	11	6	2	4	0	2	17	32	16	33
ENSG00000143870	3798	3015	2891	3112	9045	9470	6041	10427	3850	3149	11529	2389	7872	7780	7520	9591	1916	3395	2308	2977	13060	4781	8083	3826	3626	5776	2270	8551	6344	7153	6551	8048
ENSG00000238962	0	0	0	0	0	0	0	0	0	0	0	0	0	0	0	0	0	0	0	0	0	0	0	0	0	0	0	0	0	0	0	0
ENSG00000232056	0	0	0	0	0	0	0	0	0	0	0	0	0	0	0	0	0	0	0	0	0	0	0	0	0	0	0	0	0	0	0	0
ENSG00000270488	0	0	0	0	0	0	0	0	0	0	0	0	0	0	0	0	0	0	0	0	0	0	0	0	0	0	0	0	0	0	0	0
ENSG00000271952	0	0	0	0	0	0	0	0	0	0	0	0	0	1	0	1	0	0	0	0	0	0	0	0	0	0	0	0	0	0	0	0
ENSG00000162975	0	0	0	0	0	0	0	0	0	0	0	0	2	17	7	18	0	0	0	0	0	0	0	0	0	0	0	0	0	4	3	7
ENSG00000226080	0	0	0	0	0	0	0	1	0	0	0	0	0	0	0	0	0	1	0	0	0	0	0	0	0	0	0	0	0	0	0	0
ENSG00000145063	3	0	2	1	2	2	0	4	0	0	0	0	0	3	1	6	0	1	0	1	1	0	4	0	0	0	0	0	0	1	0	2
ENSG00000150873	9	4	6	5	6	5	3	2	0	1	1	0	5	8	9	11	1	5	4	10	3	5	2	4	0	1	0	1	7	7	4	5
ENSG00000162976	272	288	202	250	242	262	153	286	399	380	1671	239	462	537	395	642	162	270	169	194	480	191	342	188	336	499	414	1028	343	397	365	475
ENSG00000134318	1559	1378	1345	1335	2019	2043	1677	2273	1459	1360	5038	976	1900	2146	1936	2540	512	869	702	1443	3352	1418	2496	1400	1586	2098	1100	3861	1374	2197	1495	2349
ENSG00000207267	0	0	0	0	2	1	2	0	0	0	0	0	3	1	0	1	0	1	0	0	0	1	0	0	1	1	0	2	0	1	0	0
ENSG00000230154	2	3	4	2	0	3	1	1	0	5	7	2	0	3	4	0	0	0	1	3	5	0	1	3	4	2	0	16	1	2	2	1
ENSG00000226961	0	0	0	0	0	0	0	0	0	0	0	0	0	0	0	0	0	0	0	0	0	0	0	0	0	0	1	0	0	0	0	0
ENSG00000230952	0	1	0	0	0	0	0	0	0	0	0	0	0	0	0	0	0	0	0	0	0	0	0	0	0	0	0	0	0	0	0	0
ENSG00000231403	1	1	0	0	1	0	0	0	1	0	0	1	0	0	0	0	0	0	0	2	0	1	0	3	3	0	0	6	0	0	0	0
ENSG00000224177	5	3	0	1	4	2	4	3	0	2	9	0	0	0	1	0	1	0	0	4	0	1	0	1	4	2	1	11	0	2	1	1
ENSG00000169016	851	630	712	628	873	957	705	933	437	380	1600	336	969	914	1026	1161	626	922	818	997	2297	953	1447	937	510	686	326	1160	852	1131	970	1239
ENSG00000196208	338	307	405	275	75	64	65	89	146	145	428	95	243	230	225	406	116	144	142	291	58	52	66	32	116	254	90	417	90	179	107	199
ENSG00000264010	0	1	0	0	0	1	0	0	0	0	1	0	0	0	0	0	0	0	0	1	0	4	0	1	0	0	0	0	0	0	0	0
ENSG00000251718	1	1	0	0	0	0	0	0	0	0	0	0	0	1	0	1	0	1	1	0	0	0	0	0	1	0	0	0	0	2	1	0
ENSG00000239899	3	1	3	3	2	4	0	0	6	2	1	0	1	3	3	1	2	2	0	2	0	0	0	1	0	2	0	1	1	1	2	3
ENSG00000169006	0	0	0	0	0	0	0	0	0	0	0	0	0	0	0	0	0	0	0	0	0	0	0	0	0	0	0	0	0	0	0	0
ENSG00000230044	0	0	0	0	0	0	0	0	0	0	0	0	0	0	0	0	0	0	0	0	0	0	0	0	0	0	0	0	0	0	0	0
ENSG00000276411	0	0	0	0	0	0	0	0	0	0	0	0	0	0	0	0	0	0	0	0	0	0	0	0	0	0	0	0	0	0	0	0
ENSG00000134324	1360	1167	1103	1168	1418	1436	1044	1462	2596	2335	11175	1788	5634	5359	4748	6629	251	466	380	811	1986	856	1422	770	2395	3895	3488	7101	2806	3937	3093	4473
ENSG00000228496	4	9	5	6	1	2	1	2	4	0	8	1	6	5	5	7	2	3	0	0	1	1	0	0	3	2	3	5	0	0	1	4
ENSG00000203643	0	0	0	0	0	0	0	0	0	0	0	0	2	0	0	3	0	0	0	0	0	0	0	0	0	0	0	0	0	0	0	0
ENSG00000230790	2	0	0	2	0	0	0	0	0	0	2	0	6	1	3	3	0	2	1	1	0	0	0	0	1	0	0	4	0	5	2	13
ENSG00000265056	0	0	1	0	0	1	0	0	1	0	3	1	0	2	1	0	0	0	0	0	0	1	0	0	2	0	4	4	1	0	0	4
ENSG00000265172	0	0	0	0	0	0	0	0	0	0	0	0	0	0	0	1	0	0	0	0	0	0	0	0	0	1	0	0	0	0	0	0
ENSG00000224184	1	0	0	0	3	3	3	4	0	0	0	0	353	280	343	403	0	0	0	1	6	0	6	0	0	0	0	0	359	429	548	572
ENSG00000238503	0	0	0	0	0	0	0	0	0	0	0	0	3	2	6	3	0	0	0	0	0	0	0	0	0	0	0	0	1	7	7	12
ENSG00000264089	0	0	0	0	0	0	0	0	0	0	0	0	1	1	3	3	0	0	0	0	0	0	0	0	0	0	0	0	2	8	5	7
ENSG00000207183	0	0	0	0	0	0	0	0	0	0	0	0	1	3	5	0	0	0	0	0	0	0	0	0	0	0	0	0	0	9	5	6
ENSG00000223360	0	0	0	1	0	0	0	0	0	0	0	0	0	3	0	0	0	0	0	0	0	0	0	0	0	0	0	0	0	1	0	0
ENSG00000261117	3	0	0	2	0	1	3	2	0	0	2	0	0	2	0	1	1	1	0	4	6	5	4	1	0	2	1	0	0	0	1	1
ENSG00000071575	827	1201	731	1280	2422	2531	2109	2719	1500	1067	4328	752	2053	2324	2219	3228	229	650	272	993	3093	1347	2566	1258	935	1440	1373	2608	1602	3075	1559	3215
ENSG00000264370	3	0	0	0	0	2	4	5	0	2	2	1	3	2	0	8	0	0	0	1	4	0	2	2	1	0	1	3	2	6	1	6
ENSG00000225649	0	0	0	0	0	0	0	0	1	1	23	2	0	0	0	1	0	0	0	0	0	0	1	0	0	2	5	6	0	0	0	0
ENSG00000229370	0	0	0	0	0	0	0	0	0	0	0	0	0	0	0	0	0	0	0	0	0	0	0	0	0	0	0	0	0	0	0	0
ENSG00000230448	0	0	0	0	0	0	0	0	0	0	0	0	0	1	0	0	0	0	0	0	0	0	0	0	0	0	0	0	0	0	0	0
ENSG00000227718	0	0	0	0	0	0	0	0	0	0	0	0	0	0	0	0	0	0	0	0	0	0	0	0	0	0	0	0	0	0	0	0
ENSG00000251859	0	0	0	0	0	0	0	0	0	0	0	0	0	0	0	0	0	0	0	0	0	0	0	0	0	0	0	0	0	0	0	0
ENSG00000237261	0	0	0	0	0	0	0	0	0	0	0	0	0	0	0	0	0	0	0	0	0	0	0	0	0	0	0	0	0	0	0	0
ENSG00000228505	0	0	0	0	0	0	0	0	0	0	0	0	0	0	0	0	0	0	0	0	0	0	0	0	0	0	0	0	0	0	0	0
ENSG00000162981	1	3	0	0	0	1	1	0	0	1	2	0	2	0	0	0	0	0	0	1	0	0	0	0	0	0	1	0	0	0	1	0
ENSG00000235127	0	0	0	0	0	0	0	0	0	0	0	0	0	0	0	0	0	0	0	0	1	0	1	0	0	0	0	1	0	0	0	0
ENSG00000151779	1942	1520	1652	1377	3684	3360	2665	3505	864	749	2813	616	2025	1859	1751	2294	599	945	825	1114	5836	2197	4621	2028	904	1337	634	2300	1212	1569	1544	1598
ENSG00000229087	2	0	0	2	0	0	0	0	0	0	1	0	0	0	3	0	0	0	0	1	4	2	6	0	0	0	1	0	0	1	0	0
ENSG00000234022	0	0	1	1	3	3	6	3	0	2	2	0	1	2	4	1	2	3	0	0	7	2	6	7	0	2	0	1	0	2	4	7
ENSG00000079785	3603	2530	2693	2572	3661	3654	3208	4390	2852	1861	8166	1583	4420	3898	5360	5495	4522	6734	5554	4823	11464	4497	8804	4392	2526	3807	1998	5510	6232	6227	7884	7679
ENSG00000224194	0	0	0	0	0	0	0	0	1	0	0	0	0	0	0	0	0	0	0	0	0	0	0	0	0	1	0	0	0	0	0	0
ENSG00000231031	0	0	0	0	0	0	0	0	0	0	3	0	1	0	0	1	0	0	0	0	0	0	0	0	0	0	1	0	0	0	1	0
ENSG00000237326	0	0	0	0	0	0	0	0	0	0	0	0	0	0	0	0	0	0	0	0	0	0	0	0	0	0	0	0	0	0	0	0
ENSG00000202160	0	0	0	0	0	0	0	0	0	0	0	0	0	0	0	0	0	0	0	0	0	0	0	0	0	0	0	0	0	0	0	0
ENSG00000214657	0	0	0	0	0	0	0	0	0	1	0	0	0	0	0	0	0	0	0	0	0	0	0	0	0	0	0	0	0	0	0	0
ENSG00000238371	0	0	0	0	0	0	0	0	0	0	0	0	0	0	0	0	0	0	0	0	0	0	0	0	0	0	0	0	0	0	0	0
ENSG00000223850	0	0	0	0	0	2	0	1	0	0	0	0	0	0	0	0	0	0	0	0	2	0	0	0	0	0	0	0	0	0	0	0
ENSG00000233718	0	1	1	2	132	104	91	94	0	0	0	0	16	24	28	16	3	13	0	24	156	65	124	79	0	0	0	0	4	8	3	17
ENSG00000134323	8	29	7	18	1484	1362	1039	1398	0	0	0	2	151	168	178	194	3	69	12	126	2804	1108	2194	1062	0	0	0	0	64	97	79	126
ENSG00000243541	0	0	0	1	1	0	0	0	0	0	0	0	0	0	0	0	0	0	0	0	1	0	0	2	0	0	2	0	0	0	0	0
ENSG00000226764	0	0	0	0	0	1	1	0	0	0	0	0	2	1	0	0	0	0	0	0	1	0	0	2	0	0	0	0	0	0	1	0
ENSG00000236289	0	0	0	0	6	3	6	10	0	0	1	0	2	6	4	2	0	0	0	0	4	2	3	1	0	0	0	0	0	0	0	1
ENSG00000236989	0	0	0	0	0	0	0	4	0	0	0	0	0	0	0	0	0	0	0	0	0	0	0	1	0	0	0	0	0	0	0	0
ENSG00000271315	0	0	0	0	0	0	0	0	0	0	0	0	0	0	0	0	0	0	0	0	0	0	0	0	0	0	0	0	0	0	0	0
ENSG00000251704	0	0	0	0	0	0	0	0	0	0	0	0	0	0	0	0	0	0	0	0	0	0	1	0	0	0	1	0	0	0	0	0
ENSG00000226041	0	0	0	0	1	0	0	0	0	0	0	0	0	0	0	0	0	0	0	0	0	0	0	0	0	0	0	0	0	0	0	0
ENSG00000228876	1	0	0	0	0	0	0	0	0	0	0	0	0	0	0	0	2	1	0	1	0	0	0	0	0	0	0	0	0	0	0	0
ENSG00000231266	0	0	0	0	0	0	0	0	0	0	0	0	0	0	0	0	0	0	0	0	0	0	0	0	0	0	0	0	0	0	0	0
ENSG00000232444	0	0	0	0	15	9	8	17	0	0	0	0	0	0	0	0	0	0	0	2	8	8	13	2	0	0	0	0	0	0	0	0
ENSG00000224400	1	2	0	0	219	216	214	224	0	0	0	0	0	0	0	0	3	4	1	27	628	290	311	281	0	0	0	0	0	0	0	0
ENSG00000237633	0	0	0	0	0	0	0	0	0	0	0	0	0	0	0	0	0	0	0	0	0	0	0	0	0	0	0	0	0	0	0	0
ENSG00000279663	0	0	0	0	0	0	0	0	0	0	1	0	0	0	0	0	0	0	0	0	0	0	0	0	0	0	0	0	2	2	0	0
ENSG00000197872	0	2	0	0	154	135	119	151	1	5	27	10	0	4	0	2	2	3	0	2	233	67	180	80	0	0	0	11	0	2	0	0
ENSG00000223536	0	0	0	0	0	0	0	0	0	0	0	0	0	0	0	0	0	0	0	0	0	0	0	0	0	0	0	0	0	0	0	0
ENSG00000224604	0	0	0	0	0	0	0	0	0	0	0	0	0	0	0	0	0	0	0	0	0	0	0	0	0	0	0	0	0	0	0	0
ENSG00000237670	0	0	0	0	0	0	0	0	0	0	0	0	0	0	0	0	0	0	0	0	0	0	0	0	0	0	0	0	0	0	0	0
ENSG00000222842	0	0	0	0	0	0	0	0	0	0	0	0	0	0	0	0	0	0	0	0	0	0	0	0	0	0	0	0	0	0	0	2
ENSG00000214843	0	0	0	0	0	0	0	0	0	0	0	0	0	0	0	0	0	0	0	0	0	0	0	0	0	0	0	0	0	0	0	0
ENSG00000236348	0	0	0	0	0	0	0	0	0	0	0	0	0	0	0	0	0	0	0	0	0	0	0	0	0	0	1	0	0	0	0	0
ENSG00000214842	0	0	1	0	0	0	0	0	0	0	0	0	0	0	0	0	0	0	0	0	0	0	0	0	0	0	0	0	0	0	0	0
ENSG00000163032	11	5	2	1	3	5	2	4	1	2	11	4	4	2	1	3	1	1	0	4	4	5	0	3	5	3	2	12	4	4	7	2
ENSG00000163029	2116	1578	1645	1513	1484	1705	1372	1894	1439	1174	4251	857	4233	4107	4187	5263	2011	2569	2023	2128	4151	1763	2975	1947	1395	2006	970	3538	3801	4372	4780	5460
ENSG00000178295	2043	1488	1891	1516	1930	2063	1552	2132	1609	1387	5683	997	2340	2294	2728	3033	780	1098	1066	2197	4111	1834	2521	1887	1659	2162	935	4036	1566	3044	2074	3625
ENSG00000151379	0	0	0	0	0	0	0	0	0	0	0	0	0	0	0	1	0	0	0	0	0	0	0	0	0	0	0	0	0	1	2	2
ENSG00000170745	0	0	0	3	0	0	0	0	0	1	0	1	10	19	21	38	0	2	2	0	0	0	0	0	0	0	0	2	44	102	82	146
ENSG00000212455	0	0	0	0	0	0	0	0	0	0	0	0	0	0	0	0	0	0	0	0	0	0	0	0	0	0	0	0	0	0	0	0
ENSG00000260331	9	1	9	2	4	7	4	4	3	6	9	2	1	1	7	2	0	2	3	2	3	1	3	2	4	2	1	4	0	2	6	2
ENSG00000240857	341	248	287	250	306	307	238	328	307	235	1204	201	643	659	806	861	222	440	381	330	654	327	620	242	253	462	292	681	731	812	891	802
ENSG00000250741	0	0	0	0	0	0	0	0	0	0	0	0	0	0	0	0	2	0	0	0	0	0	0	0	0	0	0	0	0	1	0	0
ENSG00000185013	8	3	7	5	5	5	7	5	9	5	32	12	13	16	26	28	1	4	3	8	27	4	10	7	6	9	5	18	10	40	23	24
ENSG00000207170	0	1	0	0	1	5	0	2	0	0	2	0	2	1	3	1	0	0	0	0	3	0	2	1	1	0	0	4	0	3	0	2
ENSG00000224626	0	0	0	0	0	0	0	1	0	1	0	1	0	0	1	1	0	0	0	0	1	0	0	1	0	0	0	2	0	8	2	4
ENSG00000236204	17	11	14	11	13	12	11	14	12	7	26	11	11	21	17	19	2	2	4	6	11	7	8	2	9	7	12	17	10	10	9	16
ENSG00000266738	0	0	0	0	0	0	0	0	0	0	0	0	0	0	0	0	0	0	0	0	0	0	0	0	0	0	0	0	0	0	0	0
ENSG00000143867	0	0	0	0	0	0	0	0	0	0	0	0	0	1	0	0	0	0	4	0	0	0	0	0	1	0	0	0	0	0	0	1
ENSG00000234597	0	0	0	0	0	0	0	0	0	0	2	0	0	0	0	0	0	0	0	0	0	0	0	0	0	0	0	0	0	0	0	0
ENSG00000237992	0	0	0	0	0	0	0	0	1	0	0	0	0	0	0	0	0	0	0	0	0	0	0	0	0	0	0	0	0	0	0	0
ENSG00000235911	0	0	0	0	0	0	0	0	0	0	0	0	0	0	0	0	0	0	0	0	0	0	0	0	0	0	0	0	0	0	0	0
ENSG00000213403	1	0	0	0	0	0	0	0	0	1	5	0	0	0	0	0	0	0	0	2	0	0	0	0	0	0	0	1	0	0	0	1
ENSG00000228784	1	0	0	2	0	1	0	0	3	9	23	8	11	15	9	6	0	1	0	3	1	1	0	2	12	17	8	14	3	15	3	21
ENSG00000183891	340	237	308	292	229	178	160	261	305	236	922	189	523	494	490	676	225	231	236	285	370	182	238	165	205	323	182	593	390	453	550	602
ENSG00000271991	1	4	7	1	1	3	2	0	1	1	10	2	5	8	10	2	5	6	2	7	3	0	6	1	5	2	7	6	4	16	6	7
ENSG00000118965	476	307	434	317	80	259	81	276	712	558	2139	381	2172	1769	2638	2384	275	428	321	461	37	50	31	34	562	839	490	1366	1581	1950	2376	2373
ENSG00000227210	2	2	2	1	0	0	0	0	0	1	3	1	8	8	3	5	0	1	2	1	0	0	0	0	0	3	0	1	0	6	1	3
ENSG00000132031	0	1	1	2	0	0	0	0	0	2	1	1	4	3	2	1	3	0	0	0	1	0	0	0	0	1	0	2	1	0	4	3
ENSG00000068697	1428	950	1200	1058	1962	2201	1487	2296	857	603	3112	593	1741	1746	2155	2436	1404	2165	1628	1370	4532	1880	2983	1734	730	1118	804	1847	2818	2718	4403	3591
ENSG00000223734	2	3	4	3	1	0	0	0	0	0	2	0	4	1	0	0	3	1	1	2	0	0	1	1	2	0	0	0	2	2	2	1
ENSG00000234378	0	0	0	0	0	0	0	0	0	0	0	0	0	0	0	0	0	0	0	0	0	0	0	0	0	0	0	0	0	0	0	0
ENSG00000213729	0	0	0	0	0	0	0	0	0	0	0	0	0	0	0	0	0	0	0	0	0	0	0	0	0	0	0	0	0	0	0	0
ENSG00000266059	0	0	0	0	0	0	0	0	0	0	1	0	0	0	0	0	0	0	0	0	0	0	0	0	0	0	1	0	0	0	0	0
ENSG00000200763	0	0	0	0	0	0	0	0	0	0	1	0	0	0	0	0	0	0	0	0	0	0	0	0	0	0	0	0	0	0	0	0
ENSG00000115884	0	0	1	0	3	2	0	4	12	12	386	69	1304	1091	1168	1377	0	0	0	0	9	6	10	8	3	22	59	296	726	886	779	1000
ENSG00000235411	0	0	0	0	1	0	3	2	0	0	2	2	1	0	0	3	0	1	2	0	3	1	2	0	0	4	0	2	0	2	5	4
ENSG00000238735	1	0	0	0	1	4	0	3	0	2	5	0	1	2	0	0	0	0	0	3	5	1	0	3	0	0	1	9	2	3	4	7
ENSG00000055917	5587	3690	4658	3535	4658	4671	3641	5110	4381	3878	16882	2923	7180	6719	7460	8207	3512	4876	4360	4868	10993	4300	7365	4215	5050	7043	3945	12084	6076	7829	6930	8829
ENSG00000232800	0	0	0	0	0	0	0	0	0	1	0	0	0	0	0	1	0	0	1	0	0	0	0	0	0	0	0	0	0	0	0	0
ENSG00000143878	0	2	2	2	0	1	0	0	417	463	2196	486	384	530	526	778	0	0	0	1	0	0	0	0	227	613	235	1199	142	240	133	257
ENSG00000228950	0	0	0	0	0	0	0	0	0	0	0	0	0	0	0	0	0	0	0	0	0	0	0	0	0	0	0	0	0	0	0	0
ENSG00000200829	0	0	0	0	0	0	0	0	0	0	0	0	0	0	0	0	0	0	0	0	0	0	0	0	0	1	2	0	0	0	0	0
ENSG00000227047	0	0	0	0	0	0	0	0	0	0	0	0	0	0	0	0	0	0	0	0	0	0	1	0	0	0	0	0	0	0	0	0
ENSG00000279307	5	7	1	2	1	2	3	1	4	3	7	1	5	9	7	7	5	14	4	11	11	2	3	2	3	2	0	6	4	16	12	16
ENSG00000118960	243	265	236	267	345	386	256	338	177	193	752	154	401	432	307	627	101	174	106	204	482	204	387	164	140	234	230	636	238	311	192	305
ENSG00000269976	4	0	0	1	2	0	1	2	4	0	9	0	5	7	8	4	0	0	0	0	1	0	1	1	2	4	3	1	1	4	2	2
ENSG00000231948	1	3	0	1	3	1	3	5	0	1	8	1	2	2	0	2	1	0	0	0	1	3	0	1	0	0	3	3	0	0	2	0
ENSG00000233416	4	4	6	3	2	2	1	0	1	2	1	2	0	0	0	0	0	0	0	1	2	1	0	1	0	2	4	8	0	1	0	0
ENSG00000143869	5	13	12	7	15	11	4	16	11	12	39	6	23	20	13	27	3	0	4	10	12	3	9	11	5	14	9	44	8	15	11	32
ENSG00000270100	0	0	0	0	0	0	0	0	0	0	0	0	0	0	0	0	0	0	0	0	0	0	0	0	0	0	0	0	0	0	0	0
ENSG00000118961	924	692	817	645	450	482	362	474	859	687	2925	576	1984	1927	1910	2476	1201	1636	1325	1329	1046	372	726	432	844	1185	738	2076	2003	2119	2410	2781
ENSG00000236436	0	0	0	0	0	0	0	1	0	0	0	0	0	0	0	0	0	0	0	0	0	0	0	0	0	1	0	0	0	0	0	0
ENSG00000261012	0	0	0	0	0	0	0	0	0	0	0	0	0	0	0	0	0	0	0	0	0	0	0	0	0	0	0	0	0	0	0	0
ENSG00000084674	0	0	1	1	2	2	0	0	0	0	1	0	4	0	0	3	0	0	0	1	1	1	2	0	0	0	0	2	0	1	1	0
ENSG00000280390	0	0	0	0	0	2	0	0	0	0	0	0	0	0	0	0	0	0	0	0	0	0	0	0	0	0	0	0	0	0	0	0
ENSG00000218819	0	0	0	0	0	0	1	0	0	0	0	0	0	2	0	1	0	0	0	0	1	0	2	1	0	0	0	0	0	0	0	4
ENSG00000271629	0	0	0	0	0	0	0	0	0	0	0	0	0	0	0	0	0	0	0	0	0	0	0	0	0	0	0	0	1	0	0	0
ENSG00000233005	0	0	0	0	1	0	0	0	0	0	0	0	0	0	0	0	0	0	0	0	0	0	0	0	0	0	0	0	0	0	0	0
ENSG00000231204	0	0	0	0	0	0	0	0	0	0	0	0	0	0	0	0	0	0	0	0	0	0	0	0	0	0	0	0	0	0	0	0
ENSG00000270654	0	0	0	0	0	0	0	0	0	0	0	0	0	0	0	0	0	0	0	0	0	0	0	0	0	0	0	0	0	0	0	0
ENSG00000235537	0	0	0	0	0	0	0	0	0	0	0	0	1	4	0	0	0	0	0	1	0	0	0	0	0	0	0	0	1	1	0	0
ENSG00000228538	0	0	0	0	0	0	0	0	0	0	0	0	1	0	0	1	0	0	0	0	0	0	0	0	0	0	0	0	0	0	0	0
ENSG00000229621	0	0	0	0	0	0	0	0	0	0	0	0	10	32	4	28	0	0	0	4	0	0	0	0	0	0	0	0	3	9	3	13
ENSG00000264192	0	0	0	0	0	0	0	0	0	0	0	0	0	0	0	0	0	0	0	0	0	0	0	0	0	0	0	0	0	0	0	0
ENSG00000231200	0	0	0	0	0	0	0	0	0	0	0	0	0	0	0	0	0	0	0	0	0	0	0	0	0	0	1	0	0	0	0	0
ENSG00000228999	0	0	0	1	0	0	0	0	0	0	0	0	0	0	0	0	0	0	0	0	0	0	0	0	0	0	0	0	0	0	1	0
ENSG00000223691	0	0	0	0	0	0	0	0	0	0	0	0	0	0	0	0	0	0	0	0	0	0	0	0	0	0	0	0	0	0	0	0
ENSG00000234189	0	0	0	0	0	0	0	0	0	0	0	1	0	0	0	0	0	0	0	0	0	0	0	0	0	0	0	0	0	0	0	0
ENSG00000234207	0	0	0	0	0	0	0	0	0	0	0	0	0	0	0	0	0	0	0	0	0	0	0	0	0	0	0	0	0	0	0	0
ENSG00000233587	0	0	0	0	0	0	0	0	0	0	0	0	0	0	0	0	0	0	0	0	0	0	0	0	0	0	0	0	0	0	0	0
ENSG00000222616	0	0	0	0	0	0	0	0	0	0	0	0	0	0	0	0	0	0	0	0	0	0	0	0	0	0	0	0	0	0	0	0
ENSG00000232451	0	0	0	0	0	0	0	0	0	0	0	0	1	1	0	3	0	0	0	0	0	0	0	0	0	1	0	0	0	0	0	0
ENSG00000223530	0	0	0	0	0	0	0	0	0	0	0	0	0	0	0	0	0	0	0	0	0	0	0	0	0	0	0	0	0	0	0	0
ENSG00000233714	0	1	0	0	0	0	0	0	0	0	0	0	0	0	0	0	0	0	2	0	0	0	0	0	0	0	0	0	0	0	0	0
ENSG00000223634	0	1	0	1	0	0	0	0	0	0	0	0	0	0	0	0	0	0	0	0	0	0	0	0	0	0	0	0	0	0	1	0
ENSG00000235497	1	1	0	1	0	0	0	0	0	0	0	0	1	0	0	0	0	0	1	3	0	0	1	0	0	0	0	0	2	0	0	0
ENSG00000119771	325	487	377	514	130	166	103	159	22	13	97	6	21	21	10	42	91	189	139	427	161	102	112	86	12	18	40	54	15	37	27	41
ENSG00000224361	0	2	3	2	0	2	0	0	0	0	0	0	0	0	0	0	0	0	0	0	0	1	0	0	0	0	0	0	0	0	0	0
ENSG00000279526	0	7	2	0	0	2	0	2	0	0	0	0	0	0	0	0	2	0	0	4	0	0	0	0	0	0	0	0	0	0	0	0
ENSG00000283031	19	16	23	27	14	12	18	21	2	0	4	1	4	2	0	2	9	2	5	24	30	12	14	18	4	5	2	3	0	2	0	0
ENSG00000119778	1545	1105	1260	1007	1278	1285	1003	1342	864	741	3966	590	1570	1397	1714	1691	471	624	566	1014	2696	1157	1950	1184	1029	1609	1090	3012	1072	1662	1417	1890
ENSG00000224464	0	2	3	2	2	2	4	9	2	3	11	1	2	8	4	10	0	0	0	10	8	5	4	5	3	8	6	11	2	3	8	3
ENSG00000238111	2	1	2	0	0	1	3	0	0	0	6	0	0	1	2	2	0	0	2	0	2	1	0	2	2	7	3	5	1	0	2	2
ENSG00000173960	997	807	995	834	1158	1134	887	1240	694	548	2405	459	1259	1234	1394	1601	734	1101	1035	801	2541	860	1802	697	659	1024	509	1735	1207	1236	1152	1301
ENSG00000234946	5	5	1	4	4	0	2	7	1	3	10	0	2	3	5	1	4	2	0	1	7	3	1	4	1	2	1	6	1	9	2	4
ENSG00000243847	2	0	4	4	0	1	0	1	2	0	3	0	1	2	0	0	1	0	0	3	2	3	0	1	0	2	0	2	1	3	2	3
ENSG00000205639	40	29	47	38	23	9	8	23	5	5	18	9	20	16	13	16	10	8	8	19	27	14	18	17	10	9	7	22	2	11	4	23
ENSG00000163026	1161	780	924	766	580	578	432	581	497	458	1397	320	797	879	923	1174	872	1223	1068	1102	1201	441	908	377	480	678	279	1032	923	1021	998	1210
ENSG00000222940	0	0	1	1	1	1	2	1	0	0	0	0	1	0	0	2	0	1	0	3	0	0	0	0	0	0	1	1	2	2	2	0
ENSG00000119782	48	27	36	47	0	1	0	3	2	6	12	4	5	5	0	14	20	23	30	21	1	0	0	0	3	7	6	3	7	5	11	10
ENSG00000115128	1447	852	985	824	1087	1051	757	1089	922	650	2908	540	2194	1973	2442	2534	1592	1751	1679	1844	2624	1166	1878	1546	982	1255	642	1918	2821	2460	3313	3080
ENSG00000219626	56	33	61	35	124	93	72	127	240	170	886	132	140	136	120	165	18	25	24	36	119	56	73	48	145	226	228	436	45	93	63	115
ENSG00000115129	155	102	136	90	479	472	322	505	19	26	676	37	164	181	147	207	114	129	117	103	856	415	642	335	17	22	85	134	136	183	175	187
ENSG00000176732	9	9	6	11	8	11	7	19	11	12	52	14	17	18	8	21	3	10	5	14	16	8	17	7	3	15	17	32	2	9	7	16
ENSG00000276087	0	0	0	0	0	0	1	0	0	0	2	0	0	0	0	0	0	0	0	0	0	0	1	1	0	1	2	2	0	0	1	1
ENSG00000232642	2	0	0	0	1	0	0	0	1	1	8	0	1	3	0	3	1	0	0	0	1	0	1	0	0	1	2	9	0	0	0	1
ENSG00000186453	0	1	3	3	0	1	0	1	2	5	9	2	2	2	0	0	1	0	0	0	2	1	0	1	1	7	6	6	1	0	0	1
ENSG00000223754	1	2	0	3	1	1	0	3	1	0	12	4	1	0	1	0	0	0	0	0	2	1	1	1	1	1	1	5	0	0	0	1
ENSG00000198399	1932	1400	1771	1448	2551	2623	2059	2726	1414	1202	7145	1059	3159	3209	3362	3851	671	980	961	1581	4845	1962	3323	1874	1633	2499	1834	5059	2343	3338	2827	3761
ENSG00000242628	4	0	1	1	0	1	1	4	1	2	7	0	0	2	0	1	0	0	0	2	3	1	0	2	1	3	4	7	0	5	2	3
ENSG00000251805	1	1	0	0	1	1	0	4	3	1	2	1	0	1	1	0	0	2	0	0	2	1	2	1	0	3	1	10	1	1	3	1
ENSG00000232963	1	0	0	0	2	3	1	2	0	0	6	1	0	1	3	2	0	2	0	2	2	1	3	1	3	1	6	1	3	3	3	1
ENSG00000233747	0	0	0	0	0	3	0	1	1	2	9	2	2	0	3	2	0	0	0	3	4	1	0	1	2	3	2	2	1	1	0	0
ENSG00000084676	1058	895	981	845	1464	1441	1091	1555	1450	1238	6158	983	670	831	733	1123	252	418	414	731	2540	992	1926	920	1522	2394	1591	4516	518	918	513	1080
ENSG00000206732	0	0	0	0	0	1	0	0	0	0	5	0	0	1	1	2	0	0	0	0	0	1	0	1	0	2	0	3	0	0	0	0
ENSG00000184924	306	241	254	233	300	294	193	333	334	285	1159	262	502	564	595	745	484	645	513	477	636	255	453	279	324	417	249	672	789	727	886	899
ENSG00000138092	1682	1215	1603	1299	1452	1433	1229	1529	1137	863	2959	632	1005	1019	1324	1479	578	712	818	1225	2531	1039	1949	963	1040	1651	468	2651	896	1348	1043	1549
ENSG00000138031	1265	1170	1229	1140	2726	2783	2325	2902	1489	1220	5261	994	3028	2685	3177	3650	322	465	369	1142	4636	2145	3576	2022	1269	2354	1327	4089	1967	2712	2177	2951
ENSG00000271936	167	124	156	129	154	166	96	167	129	92	365	74	117	132	104	134	44	46	58	113	230	94	129	92	103	156	62	293	58	94	75	97
ENSG00000115137	331	227	276	236	261	229	134	250	182	181	742	116	327	298	313	372	205	164	185	270	384	212	267	192	180	221	171	433	244	348	254	370
ENSG00000237953	0	0	0	0	0	6	1	2	0	0	1	2	0	1	0	3	0	0	2	0	0	1	1	0	0	0	3	4	0	0	1	1
ENSG00000202479	0	0	1	0	2	0	0	1	1	0	2	0	0	0	0	1	0	0	1	0	0	0	0	0	1	1	1	1	0	1	0	1
ENSG00000224165	48	31	35	41	15	10	11	23	14	15	39	6	23	19	25	23	17	18	22	27	26	9	12	7	5	5	11	18	15	5	12	18
ENSG00000207069	0	1	0	0	0	0	0	0	0	0	0	0	0	1	0	0	0	0	0	0	0	1	0	0	0	0	0	0	0	0	1	0
ENSG00000084710	5	24	3	19	1007	1234	781	1143	6	19	10	13	20	24	9	44	2	5	3	14	1184	589	800	549	3	14	8	22	2	11	2	11
ENSG00000276653	0	0	0	0	7	2	1	4	0	1	0	0	0	0	0	0	0	0	0	0	6	3	3	3	0	0	0	0	0	0	0	0
ENSG00000229593	0	0	0	0	6	4	3	2	0	0	0	0	0	0	0	1	0	0	0	0	3	0	5	4	0	0	0	0	0	0	0	0
ENSG00000115138	0	0	0	0	0	1	0	2	1	1	0	0	4	1	1	2	0	0	0	0	3	2	4	2	1	3	0	2	0	2	0	0
ENSG00000230452	0	0	0	0	0	0	0	0	0	0	0	0	0	0	0	0	0	0	0	0	0	0	1	0	0	0	0	0	0	0	0	0
ENSG00000119772	3869	2817	3602	2903	4855	4719	3685	5098	342	308	2065	307	1865	1600	2233	2263	1059	1341	1395	2607	7446	3307	5131	2915	323	579	695	1404	977	1483	1393	1738
ENSG00000221445	0	3	7	0	2	4	3	3	1	1	6	2	2	3	3	3	1	0	2	2	2	2	3	0	0	3	3	5	0	2	1	2
ENSG00000235072	6	2	9	9	9	19	7	15	12	14	28	6	8	22	12	12	4	2	2	11	27	9	18	14	5	13	4	34	2	15	4	19
ENSG00000138101	601	529	501	548	573	594	391	608	243	209	638	173	473	559	487	717	176	257	156	444	847	388	630	368	169	300	167	576	323	599	364	611
ENSG00000224220	12	9	15	12	2	4	10	2	2	4	3	2	3	8	14	3	9	1	3	12	12	2	1	8	4	10	5	13	4	13	11	6
ENSG00000201160	0	0	0	0	0	0	0	0	0	0	0	0	0	0	0	0	0	0	0	0	0	0	0	0	0	0	0	0	0	0	0	0
ENSG00000143970	2473	1772	2016	1840	2385	2340	1609	2436	1637	1553	8353	1265	3644	3125	3436	3985	454	680	576	1222	3457	1650	2604	1320	1969	3164	2170	6182	2023	2908	2248	3071
ENSG00000187536	3	2	1	2	3	3	2	3	0	5	6	2	4	3	2	4	2	0	0	3	4	6	2	5	8	3	4	22	2	6	8	8
ENSG00000217643	0	0	3	0	0	3	3	2	3	0	8	1	5	4	3	1	1	2	0	3	9	5	3	2	3	4	3	11	3	12	7	6
ENSG00000218682	2	4	2	1	0	4	1	7	0	0	9	0	8	3	1	2	0	1	0	5	8	6	2	1	2	3	2	7	3	6	8	1
ENSG00000217718	0	0	0	0	0	0	0	0	0	0	0	0	0	0	0	0	0	0	0	0	0	0	0	0	0	0	0	0	0	1	0	0
ENSG00000084731	617	466	537	471	929	972	685	962	345	301	1217	192	405	512	381	647	167	221	217	372	1180	510	795	471	240	398	336	745	284	382	230	416
ENSG00000237629	0	0	0	0	0	0	0	0	0	0	0	0	0	0	0	0	0	0	0	0	0	0	0	0	0	0	0	0	0	0	0	0
ENSG00000268412	1	0	0	0	2	0	0	0	0	1	1	0	1	0	0	0	0	0	0	1	4	0	0	0	0	0	0	1	2	1	0	1
ENSG00000084733	3129	2158	2517	2276	3719	3693	2703	3896	3552	3035	11975	2320	6368	6127	6524	7610	1731	3053	2466	2567	7373	3059	5473	2619	3840	5882	2754	8700	6282	6497	6907	7341
ENSG00000199872	3	0	2	3	5	4	5	3	3	1	10	2	3	3	5	4	1	3	1	0	7	9	3	8	5	5	1	11	0	9	4	6
ENSG00000235440	7	7	3	4	5	3	4	9	4	5	19	2	10	8	13	6	2	2	0	6	8	5	6	10	2	10	6	9	1	6	12	16
ENSG00000231655	1	0	1	0	1	4	0	6	4	5	20	4	4	4	8	3	2	0	1	3	4	4	2	5	3	4	6	11	5	8	8	3
ENSG00000214465	5	2	6	2	5	7	2	1	3	4	23	2	1	3	1	2	0	2	1	4	7	2	1	3	5	1	5	10	0	0	1	0
ENSG00000227133	4	0	4	1	0	0	0	1	1	0	5	0	1	1	1	0	0	0	3	1	6	1	1	0	3	2	1	3	1	4	0	0
ENSG00000237951	1	0	0	0	0	2	0	0	2	0	6	0	0	0	0	0	0	0	0	0	0	4	0	1	1	0	1	3	0	0	0	0
ENSG00000157833	296	286	316	291	3	4	1	2	167	138	584	121	199	195	178	289	57	148	91	208	1	3	0	1	115	165	165	403	84	170	78	183
ENSG00000084754	4805	3627	3724	3574	5257	5113	3943	5394	3553	2878	10492	2095	9576	8436	9089	10925	2180	4028	3077	2843	8763	3295	7015	2807	3373	5083	2349	7720	8457	9181	9369	10344
ENSG00000138029	1968	1387	1582	1424	2003	2040	1593	2098	1286	989	3843	664	3155	2725	3366	3578	2167	2845	2383	1626	4575	1708	3196	1630	943	1443	898	2517	4173	3613	5269	4677
ENSG00000173567	124	99	134	87	119	149	110	143	124	92	292	43	96	121	133	141	43	37	57	115	200	106	164	124	64	137	42	253	50	120	86	136

ENSG00000138018	3124	2192	2574	2132	2232	2455	1931	2523	1754	1600	6178	1097	4954	4462	5372	5590	1910	2882	2482	3405	5254	2152	3498	2093	2044	3182	1052	4615	4617	5806	5932	6710
ENSG00000157856	0	1	3	2	0	3	2	2	1	1	5	0	5	5	3	1	2	1	3	0	3	2	4	0	0	1	1	2	5	5	7	6
ENSG00000115155	23	24	30	18	5	5	5	5	5	3	7	1	0	1	0	0	2	1	3	15	5	2	4	3	0	0	0	5	0	1	0	0
ENSG00000173557	0	1	1	2	1	8	1	3	0	1	2	0	0	0	0	0	0	2	0	1	3	2	0	2	0	0	0	0	0	0	0	0
ENSG00000157884	0	0	0	3	0	0	0	0	0	0	0	0	0	0	0	0	0	0	0	0	0	0	0	0	0	0	0	0	0	0	0	0
ENSG00000225378	0	0	0	0	0	0	0	0	0	0	0	0	0	0	0	0	0	0	0	0	0	0	0	0	0	0	0	0	0	0	0	0
ENSG00000225024	0	0	0	0	0	0	0	0	0	0	0	0	0	0	0	0	0	0	0	0	0	0	0	0	0	0	0	0	0	0	0	0
ENSG00000171303	0	0	0	1	0	0	0	1	0	0	0	0	0	0	0	0	0	0	0	0	0	0	0	0	0	0	0	0	0	0	0	0
ENSG00000213699	717	485	672	458	496	536	386	542	333	245	1702	277	607	564	686	745	139	235	277	491	726	332	542	260	370	527	396	1118	565	622	551	734
ENSG00000115163	1025	765	961	912	935	924	611	896	579	486	1626	406	1053	959	1101	1320	382	613	650	678	1421	558	1021	481	709	1130	163	1353	914	1120	959	1208
ENSG00000145835	0	0	0	1	0	0	0	0	0	0	0	0	0	0	0	0	0	0	0	0	0	0	0	0	0	0	0	0	0	0	0	0
ENSG00000157851	0	0	0	0	0	0	0	0	0	0	0	0	0	2	0	9	0	2	0	0	0	0	0	0	0	0	0	0	0	1	0	0
ENSG00000230286	0	0	1	0	0	0	0	0	0	0	1	0	0	0	0	0	0	0	0	0	0	0	0	0	0	0	0	0	0	0	0	0
ENSG00000084764	85	82	69	106	19	26	18	24	5	14	52	16	46	68	37	91	19	37	24	89	26	15	9	15	8	10	12	48	19	34	24	57
ENSG00000205500	3	2	4	3	0	1	0	1	0	3	3	2	0	3	0	4	1	1	0	11	3	0	1	0	1	0	3	6	2	1	0	5
ENSG00000119777	1223	1001	962	906	1109	1156	804	1275	1047	913	3760	734	1755	1550	1933	2150	363	477	417	801	1434	738	1154	694	853	1527	871	2754	1324	1791	1374	1932
ENSG00000084693	1906	1394	1759	1491	1842	1889	1447	1927	800	639	2534	515	1387	1205	1188	1582	580	841	898	1243	2547	997	1952	890	533	879	434	1551	921	1217	777	1275
ENSG00000231636	5	3	2	2	8	5	4	11	2	2	4	1	2	3	0	6	1	2	1	4	5	2	8	1	1	3	2	7	3	7	2	2
ENSG00000272056	12	10	16	13	15	22	11	8	7	7	20	6	11	10	6	7	4	0	10	8	20	11	9	11	2	7	8	18	1	4	4	18
ENSG00000229122	14	10	12	15	14	18	17	17	17	9	75	14	14	16	15	27	5	0	2	8	28	2	9	8	16	17	29	49	5	29	19	19
ENSG00000272148	18	12	13	8	14	34	12	19	22	10	58	3	17	22	12	26	2	2	5	16	25	18	18	11	14	8	21	45	8	20	7	25
ENSG00000228474	1538	1042	1208	973	874	910	658	1059	1161	878	3898	787	1282	1153	1355	1573	2245	2366	2432	1779	1949	745	1615	893	949	1195	997	2418	1758	1527	1990	2144
ENSG00000138080	82	62	109	63	400	379	310	378	47	31	117	23	30	35	33	49	49	61	98	99	774	348	551	284	27	64	19	70	45	34	23	57
ENSG00000138030	301	278	236	252	336	250	264	310	252	206	633	159	347	311	318	449	107	248	186	284	452	226	384	154	193	277	152	428	225	296	168	288
ENSG00000138028	44	46	38	51	9	13	11	11	618	534	1326	361	414	452	338	536	15	45	12	50	23	15	9	13	263	499	235	796	161	222	143	225
ENSG00000143994	83	48	77	62	48	51	39	77	23	21	50	9	45	52	50	73	13	36	17	53	66	29	46	38	23	27	19	63	18	46	23	59
ENSG00000138073	2093	1524	1732	1397	1121	1113	737	1131	910	745	3151	667	2009	1821	2010	2403	1023	1597	1403	1615	1650	778	1461	734	826	1350	764	2135	2045	2129	1958	2313
ENSG00000186143	2	0	1	1	0	0	0	0	0	0	0	0	0	0	0	0	0	0	0	0	1	0	0	0	0	0	0	0	0	0	0	0
ENSG00000163792	0	2	1	2	1	0	0	0	0	0	0	0	0	1	0	0	0	0	1	0	0	0	0	0	0	0	1	1	0	0	1	1
ENSG00000236099	0	0	0	0	0	0	0	0	0	0	0	0	0	0	0	0	0	0	0	0	0	0	0	0	0	0	0	0	0	0	0	0
ENSG00000138074	1229	887	1199	947	2070	2171	1624	2080	1328	1041	4325	788	3627	3145	4178	4204	666	883	878	1458	4234	1828	2834	1626	1158	2139	771	2458	3976	5143	4124	5203
ENSG00000138085	1408	978	1099	922	1352	1392	996	1439	839	647	2472	554	1421	1315	1484	1694	911	1386	1191	1093	2459	1009	2004	950	658	1040	477	1694	1362	1377	1597	1665
ENSG00000084774	4024	3543	4181	3683	5694	6124	4559	5916	3554	2873	11044	2142	15989	12925	13083	15977	1142	1393	1280	3396	7091	3576	5867	3100	2724	5319	1837	7521	6981	10133	6616	10153
ENSG00000115194	416	446	384	500	4	9	3	8	3	5	19	9	23	41	16	74	68	154	69	242	0	0	2	2	0	1	3	17	6	28	9	27
ENSG00000163793	220	225	233	235	0	0	0	0	0	0	2	0	17	17	12	26	50	54	35	232	0	0	0	0	1	0	1	0	4	17	3	21
ENSG00000138100	215	229	213	265	4	1	3	5	3	4	15	3	9	22	17	43	31	49	38	186	2	3	2	5	1	3	1	10	1	15	5	33
ENSG00000163794	47	46	55	52	66	96	57	100	59	59	261	48	49	52	49	75	10	19	13	34	54	39	64	24	28	62	36	118	20	46	37	55
ENSG00000115204	731	564	668	543	830	901	674	1013	968	737	3527	588	1294	1174	1248	1511	556	846	635	736	1454	581	1051	605	693	1060	826	2102	1103	1276	1241	1531
ENSG00000115207	1486	1079	1258	1076	1979	1968	1482	2070	1620	1287	5443	989	2970	2508	3105	3101	552	799	619	987	3243	1358	2375	1247	1361	2279	1134	3424	2179	2782	2386	2876
ENSG00000234945	32	29	30	26	29	40	34	45	50	35	160	23	53	49	47	52	12	16	10	38	74	42	38	40	34	45	28	113	11	50	47	73
ENSG00000234072	239	232	258	245	337	302	242	325	381	312	1298	188	692	684	747	914	77	72	73	189	412	248	278	189	268	370	216	590	432	836	532	912
ENSG00000115211	876	642	838	689	876	933	795	932	616	452	2386	399	1124	1136	1385	1469	530	686	725	810	1756	747	1509	733	553	936	674	1480	1210	1358	1486	1622
ENSG00000115234	2226	1511	1978	1488	1669	1680	1270	1790	2199	1667	7665	1411	2868	2700	3021	3387	878	1688	1573	1582	2547	1129	2096	1059	2024	3024	1821	5219	2371	2545	2408	2891
ENSG00000163795	346	218	336	269	248	277	203	281	245	220	1245	212	94	90	125	146	165	196	183	250	381	206	290	193	177	309	351	677	110	168	120	198
ENSG00000115241	5357	3532	4311	3669	5094	4919	3908	5224	4084	2743	12325	2398	9582	7849	9756	10603	2032	2816	2526	3404	8896	3571	6992	3230	3566	5832	2275	8791	8720	9386	8560	10222
ENSG00000213453	20	10	12	6	12	12	12	16	13	7	45	9	25	15	32	17	5	7	4	16	25	10	17	19	15	13	9	38	9	16	31	44
ENSG00000115216	1464	957	1225	893	1836	1901	1307	1914	1324	1021	4973	844	1472	1408	1490	1716	951	1246	1080	987	3758	1368	2443	1250	1208	1711	1337	3155	1745	1799	1823	2001
ENSG00000157992	54	37	52	43	52	40	35	54	41	29	131	16	24	24	30	30	15	11	17	30	49	26	23	31	11	29	29	58	13	27	24	39
ENSG00000138002	519	560	665	558	238	298	204	345	607	549	2027	327	758	762	807	1045	152	168	146	415	255	128	259	135	383	686	528	1311	321	602	465	787
ENSG00000235267	0	1	1	1	1	0	0	1	4	5	4	0	3	2	1	2	1	1	1	0	1	3	2	2	1	2	3	5	0	1	2	1
ENSG00000200003	1	1	1	4	0	3	0	0	3	0	3	0	1	0	0	1	0	0	0	0	0	0	1	0	0	1	4	0	0	2	1	2
ENSG00000115226	1	1	0	1	1	1	0	1	0	0	0	0	2	2	0	1	0	0	2	0	0	0	0	0	0	2	0	1	0	0	0	0
ENSG00000084734	2	1	0	2	1	0	0	0	1	0	0	0	0	0	0	1	0	0	0	0	0	0	0	0	0	0	1	0	0	1	0	0
ENSG00000233438	4	0	3	5	9	3	5	4	4	0	5	2	0	2	1	1	1	1	4	3	4	3	8	4	1	1	1	2	0	1	3	1
ENSG00000280034	0	0	0	0	0	0	0	0	0	0	0	1	0	0	0	0	0	0	0	0	0	0	0	0	0	0	0	0	0	0	0	0
ENSG00000221843	51	32	77	52	35	53	32	41	28	8	170	19	14	13	30	33	17	10	30	48	52	27	28	23	13	26	37	59	13	18	22	22
ENSG00000243943	2109	1479	1961	1454	1535	1537	1347	1750	1034	845	3766	588	1424	1235	1460	1686	1096	1418	1228	1455	2875	1155	2250	1057	836	1154	923	2137	950	1360	1244	1577
ENSG00000259080	0	0	0	2	0	0	0	0	0	0	4	0	0	1	1	1	0	1	0	4	0	0	0	1	0	0	0	1	0	6	0	2
ENSG00000176714	46	42	30	33	22	29	24	33	28	36	118	14	85	81	71	100	32	42	30	46	60	23	37	16	28	35	41	91	65	69	76	101
ENSG00000198522	1205	797	1013	791	1051	1034	780	1129	774	599	2678	493	2069	1686	2170	2270	1278	1708	1300	1277	2665	1019	1911	1121	791	1032	616	1640	2308	2273	2868	2709
ENSG00000206731	1	1	4	0	3	0	0	2	4	0	3	0	2	1	3	1	0	0	0	3	2	0	1	2	0	2	1	2	3	2	1	1
ENSG00000119760	1408	880	1180	900	1321	1262	1029	1321	1020	900	4800	788	1515	1276	1789	1807	617	557	693	763	2478	967	1743	964	833	1356	1089	2594	1171	1601	1466	1813
ENSG00000163798	1244	698	983	708	893	840	683	961	783	609	2880	458	1521	1310	1743	1692	681	827	859	853	1665	649	1301	641	790	1134	771	1901	1450	1609	1709	1827
ENSG00000205334	0	0	0	0	0	0	0	0	1	0	0	0	0	2	0	0	0	0	0	0	1	0	0	1	0	0	0	0	0	0	0	0
ENSG00000243147	640	436	471	467	528	520	358	576	617	379	2041	326	699	655	840	924	788	1121	1086	991	1331	468	903	615	521	815	515	1269	1093	947	1375	1309
ENSG00000171174	79	36	49	53	21	11	7	27	42	33	121	15	95	92	152	112	55	89	71	46	33	13	21	13	19	36	27	72	118	132	228	179
ENSG00000158019	420	323	358	297	518	574	380	587	632	480	1900	319	868	914	930	1068	374	429	372	351	926	377	639	325	447	723	387	1107	806	895	913	1076
ENSG00000270846	4	0	3	0	0	0	0	2	5	1	6	1	4	4	2	2	2	2	2	4	3	3	3	2	4	1	2	14	1	2	3	1
ENSG00000265321	7	6	6	6	3	3	3	7	5	5	18	1	3	9	11	6	1	9	3	6	12	4	6	1	5	7	4	5	6	12	6	9
ENSG00000270528	2	2	2	2	6	4	0	2	1	3	9	1	2	2	3	1	1	0	0	3	1	0	1	3	6	6	4	6	1	3	2	0
ENSG00000277035	3	5	1	1	0	4	0	2	2	3	13	3	4	1	2	2	0	0	0	0	4	1	2	5	7	3	1	14	1	6	3	6
ENSG00000223522	35	14	16	19	18	23	17	39	28	31	152	11	49	41	36	51	8	0	2	15	40	19	11	21	19	31	37	113	15	46	24	69
ENSG00000225991	0	0	0	0	0	0	0	0	0	0	0	0	0	0	0	0	0	0	0	0	0	0	0	0	0	0	0	0	0	0	0	0
ENSG00000229951	1	0	0	0	10	22	10	11	2	3	5	0	3	3	1	3	0	0	0	2	18	3	7	4	0	1	0	4	0	0	0	0
ENSG00000075426	703	668	763	729	2239	2221	1771	2490	913	856	2009	608	440	691	571	897	122	201	200	377	3041	1266	1900	1137	477	1032	243	1440	253	468	252	487
ENSG00000270640	0	0	1	0	9	9	3	10	0	1	1	0	0	1	0	1	0	0	0	1	11	2	3	4	2	5	1	4	0	0	1	3
ENSG00000270210	0	1	1	0	3	0	4	2	1	0	3	0	0	0	0	1	0	0	0	1	4	3	0	1	0	0	0	2	0	0	0	0
ENSG00000227938	0	0	0	0	0	0	0	0	0	0	0	0	0	0	0	0	0	0	0	0	0	0	1	2	0	0	0	0	0	0	0	0
ENSG00000163803	1	0	1	1	31	38	17	37	13	8	29	5	11	12	21	28	0	0	2	0	37	18	18	16	0	3	6	9	8	7	11	14
ENSG00000242915	0	0	0	0	0	0	0	0	0	0	0	0	0	0	0	0	0	0	0	0	0	0	0	0	0	0	0	0	0	0	0	0
ENSG00000230730	0	0	0	0	0	0	2	1	0	0	0	0	0	3	0	0	0	0	0	0	0	0	0	0	0	0	0	0	1	0	0	0
ENSG00000226833	11	15	4	17	4	8	1	5	2	7	20	3	11	14	9	30	3	3	3	11	9	4	2	2	4	5	4	3	5	15	6	7
ENSG00000213639	6231	4029	4633	3919	6486	6729	5201	7115	5327	4448	16162	3257	9779	9333	9931	11582	5419	7610	5883	5876	14796	5847	10397	6675	5079	7484	3655	11499	9832	10828	11467	12216
ENSG00000163806	66	61	42	57	54	59	39	48	66	45	195	31	74	66	76	125	14	19	14	65	71	41	42	45	56	56	54	121	28	103	78	107
ENSG00000273233	1	3	2	0	2	2	0	0	3	1	3	0	2	3	1	3	0	0	0	1	0	1	1	1	0	3	2	3	1	7	3	4
ENSG00000171103	541	343	439	380	362	365	244	397	344	299	1122	199	702	612	738	787	367	588	467	479	670	304	559	280	305	441	249	738	670	730	761	799
ENSG00000163811	2017	1291	1784	1366	2197	2292	1831	2452	2257	1630	8512	1396	6515	5528	7222	7045	2646	3632	3442	3353	6317	2448	4698	2513	2420	3646	1716	5475	7796	8010	9806	9189
ENSG00000264994	2	0	0	1	0	1	1	0	0	0	2	1	5	2	3	2	3	0	1	7	1	0	2	0	2	0	0	4	0	0	0	4
ENSG00000265145	8	5	1	2	2	3	0	5	3	1	10	2	11	6	5	7	0	1	0	2	3	3	0	4	4	2	0	3	0	5	5	7
ENSG00000222224	2	6	2	4	2	2	1	4	2	0	7	2	12	10	8	6	0	1	1	2	2	1	3	0	0	4	0	5	9	7	10	19
ENSG00000265706	9	4	5	9	5	3	3	3	7	1	25	4	33	17	11	21	7	3	4	9	7	10	5	4	8	6	11	16	11	13	13	25
ENSG00000189350	75	50	68	36	17	39	30	32	201	195	630	141	4	2	8	9	38	20	27	44	58	32	51	30	115	224	51	240	3	4	10	6
ENSG00000200283	0	0	0	0	0	0	0	0	0	0	0	0	0	0	0	0	0	0	0	0	0	0	0	0	0	0	0	0	0	0	0	0
ENSG00000179270	0	0	0	0	0	1	0	1	0	0	0	0	0	0	0	0	3	0	0	0	0	0	0	0	1	3	2	1	0	0	0	1
ENSG00000229224	0	0	0	0	0	0	0	0	1	0	0	0	0	0	0	0	0	0	0	2	0	0	0	0	0	0	0	0	0	0	0	0
ENSG00000115295	1105	814	856	767	0	0	0	0	406	377	1613	311	231	263	265	342	395	496	445	711	2	0	0	0	403	590	519	1189	142	232	150	290
ENSG00000171094	0	2	0	1	4	5	4	1	1	0	6	1	1	3	1	2	2	2	3	5	9	2	0	0	1	6	8	14	0	5	3	0
ENSG00000242699	0	0	0	0	0	0	0	0	0	0	0	0	0	0	0	0	2	0	0	0	0	0	0	0	0	6	0	0	0	0	0	0
ENSG00000230737	0	0	0	0	0	0	0	0	0	0	0	0	0	0	0	0	0	0	0	0	0	0	0	0	0	0	0	0	0	0	0	0
ENSG00000197644	0	0	0	0	0	0	0	0	0	0	1	0	0	0	0	0	0	0	0	0	0	0	0	0	0	0	0	0	0	0	1	0
ENSG00000233862	2	1	2	1	2	2	2	0	0	2	7	0	0	0	0	2	0	1	0	1	2	1	1	0	0	0	2	1	0	0	0	0
ENSG00000225943	0	0	0	0	0	0	0	0	0	0	0	0	0	0	0	0	0	0	0	0	0	0	0	0	0	0	0	0	0	0	0	0
ENSG00000119801	376	249	280	241	650	676	540	774	472	366	3467	364	359	455	264	630	196	211	249	156	1321	430	902	493	367	483	1241	1835	226	291	272	342
ENSG00000207187	0	0	0	0	0	0	0	0	0	0	0	0	0	0	0	1	0	0	0	0	0	0	0	0	0	0	0	0	0	0	2	0
ENSG00000231712	0	0	0	0	0	0	0	0	0	0	1	0	0	0	0	0	0	0	0	0	0	0	0	0	0	0	0	0	0	0	0	0
ENSG00000213626	764	618	696	617	1354	1581	1129	1737	507	499	1165	344	1120	1485	1368	2169	242	500	495	435	3626	1515	2855	1273	252	418	216	688	1072	1699	1502	2020
ENSG00000235997	1	0	0	0	0	0	0	2	0	0	1	0	1	0	1	0	0	0	0	0	1	3	1	0	0	0	0	0	0	0	1	2
ENSG00000280154	0	0	0	0	0	0	0	0	0	0	0	0	0	0	0	0	0	0	0	0	0	0	0	0	0	0	0	0	0	0	0	0
ENSG00000172954	446	298	422	304	689	728	655	870	525	463	1495	287	1117	1085	1410	1458	303	360	408	435	2033	746	1591	744	597	781	318	1161	1314	1632	1873	1913
ENSG00000278908	0	0	0	0	0	0	0	0	0	0	0	0	0	0	0	0	0	0	0	0	0	0	0	0	0	0	0	0	0	0	0	0
ENSG00000162949	3	1	0	0	0	0	0	0	0	0	0	0	0	0	0	1	0	2	0	0	0	0	0	0	0	0	0	0	0	6	0	1
ENSG00000230118	0	0	0	0	0	0	0	0	0	0	0	0	0	0	0	0	0	0	0	0	0	0	0	0	0	0	0	0	0	0	0	0
ENSG00000158089	66	122	59	123	0	0	0	0	1	0	2	3	1	1	4	6	27	105	23	110	0	0	0	2	0	0	0	2	0	2	0	1
ENSG00000234579	0	0	0	0	0	0	0	0	0	0	0	0	0	0	0	0	0	0	0	0	0	0	0	0	0	0	0	0	0	0	0	0
ENSG00000214711	74	60	69	61	0	0	0	0	1	7	9	1	15	8	10	22	18	28	30	41	0	0	0	0	1	0	1	6	1	2	2	6
ENSG00000013016	9	9	18	13	0	0	0	0	7	8	47	3	4	9	5	6	6	9	25	19	0	0	0	0	6	1	5	21	5	16	5	16
ENSG00000213620	0	0	0	0	0	0	0	0	0	0	0	0	0	0	0	0	0	0	0	0	0	0	0	0	0	0	0	0	0	0	0	0
ENSG00000158125	0	0	0	1	0	0	0	0	0	0	0	0	0	0	0	0	0	0	0	0	0	0	0	0	0	0	0	0	0	0	3	0
ENSG00000277893	0	1	0	0	1	1	2	0	0	0	2	0	1	0	0	1	0	0	0	0	2	2	1	1	0	0	1	0	1	0	1	0
ENSG00000228563	1	0	0	0	0	1	0	0	0	0	0	0	0	0	0	0	0	0	0	0	0	0	0	0	0	0	0	0	0	0	0	0
ENSG00000270422	0	0	0	0	0	0	0	1	0	0	0	0	0	0	0	0	0	0	0	0	0	0	0	0	0	0	0	0	0	0	0	0
ENSG00000223647	0	0	0	0	0	0	0	0	0	0	0	0	0	0	0	0	0	0	0	0	0	0	0	5	0	0	0	0	0	0	0	0
ENSG00000237007	0	0	0	0	0	1	2	2	1	0	3	2	0	1	1	1	0	0	0	0	5	3	2	2	0	1	0	3	0	8	2	2
ENSG00000242272	0	0	0	0	1	2	1	3	0	0	1	0	1	1	1	1	0	0	0	0	7	1	3	3	1	0	0	2	1	4	1	3
ENSG00000273165	2	5	3	1	8	11	10	8	10	11	54	7	16	12	11	15	1	0	2	2	14	11	7	8	10	15	21	25	9	13	28	23
ENSG00000162959	194	142	199	137	373	342	277	347	225	181	958	164	484	425	462	505	77	139	126	177	662	354	459	272	261	388	237	591	429	495	511	522
ENSG00000162961	558	341	410	338	710	754	570	826	572	374	1550	270	1323	1325	1279	1553	685	804	796	615	1593	632	1134	740	470	682	355	974	1626	1524	1846	1770
ENSG00000271228	1	0	5	1	9	4	9	4	10	2	8	2	7	7	9	23	1	1	2	3	5	1	5	7	4	2	2	11	4	5	1	4
ENSG00000021574	594	387	591	435	1163	1179	949	1269	804	665	3080	480	1318	1259	1341	1549	327	437	445	570	2570	897	1935	937	868	1201	836	1992	1286	1497	1412	1624
ENSG00000272716	11	9	10	11	9	9	5	15	12	14	29	6	36	27	53	36	3	5	4	19	23	2	1	3	9	13	6	17	22	38	20	32
ENSG00000152683	529	325	450	354	873	904	693	927	1019	833	3457	602	1538	1458	1901	1945	325	511	549	478	2060	825	1552	867	1125	1487	865	2339	1591	1706	2226	1968
ENSG00000229816	18	14	21	21	15	12	12	25	24	28	91	12	38	41	42	35	9	3	1	19	26	12	10	15	34	12	35	66	14	50	46	43
ENSG00000201113	0	1	3	0	2	0	0	1	3	1	2	0	3	2	2	3	0	0	0	0	0	3	0	2	0	0	0	7	0	0	5	5
ENSG00000091106	11	18	8	9	23	30	15	44	6	9	17	3	21	18	14	22	2	3	7	12	33	24	27	18	2	15	5	24	7	26	13	21
ENSG00000119820	1033	616	736	677	1267	1376	1001	1390	1547	1235	7486	944	2678	2453	2730	3100	527	677	543	572	2271	992	1781	800	1313	2071	1883	4060	2100	2343	2474	2432
ENSG00000272754	3	1	4	6	7	5	3	9	14	6	52	6	17	11	20	21	2	3	2	7	15	4	6	6	7	7	1	31	10	15	10	13
ENSG00000115760	2134	1586	2004	1573	6104	6644	4360	6550	4095	3696	20720	3084	8259	7553	8760	8943	754	1031	1064	1510	11320	4809	7370	4445	4753	7406	5712	15612	4939	8030	6965	8725
ENSG00000230046	4	0	6	3	10	4	9	10	4	4	14	1	6	8	5	10	1	0	3	1	21	10	3	11	7	8	6	16	4	9	19	8
ENSG00000276334	25	16	23	20	39	45	46	54	41	45	199	32	85	58	62	98	7	8	14	18	70	43	44	37	35	65	65	151	20	59	48	57
ENSG00000276517	22	15	19	12	81	84	65	106	46	34	245	22	63	44	62	75	2	8	2	24	152	73	82	86	62	61	68	167	32	89	61	81
ENSG00000207653	1	1	1	0	2	2	1	4	1	1	4	0	6	0	1	6	1	0	0	1	1	3	4	3	3	2	0	2	0	4	2	2
ENSG00000274159	2	1	1	1	2	5	4	4	1	1	15	0	1	2	4	5	0	0	1	2	3	4	3	3	2	2	0	5	1	1	2	7
ENSG00000279897	5	7	7	4	14	23	6	22	9	8	69	3	25	19	22	28	1	0	0	1	29	15	16	20	14	16	22	70	6	24	25	31
ENSG00000279544	20	13	17	12	33	44	24	36	31	18	116	11	50	56	46	52	7	2	1	16	63	50	23	35	34	47	44	112	10	55	54	60
ENSG00000018699	507	337	398	332	263	284	199	273	542	435	1352	265	1952	1724	1774	2134	389	608	479	477	648	241	566	208	500	784	300	954	1770	1808	1874	1988
ENSG00000265057	1	0	0	0	0	0	0	0	0	0	0	0	0	0	0	0	0	0	0	0	0	0	0	0	0	1	0	0	1	0	0	1
ENSG00000230876	0	1	0	0	0	2	0	0	0	3	9	1	1	5	2	1	0	0	0	0	0	0	1	1	0	1	0	4	0	0	0	3
ENSG00000236854	0	0	0	0	0	0	0	0	0	0	0	0	0	0	0	1	0	0	0	0	0	0	0	0	0	0	1	0	0	0	0	1
ENSG00000206951	1	0	0	0	0	0	0	0	2	0	3	1	0	0	0	0	0	0	0	0	0	0	0	0	0	2	0	4	0	0	0	0
ENSG00000049323	278	332	327	397	849	1040	628	1069	424	429	2259	331	1069	1395	1199	1776	42	101	67	142	1270	557	965	428	367	629	839	1312	688	1290	872	1444
ENSG00000223951	1	1	0	0	2	0	0	0	0	0	1	0	1	0	0	0	2	0	0	0	2	1	0	3	1	0	0	3	1	0	2	4
ENSG00000283591	0	0	0	0	0	0	0	0	0	0	0	0	0	0	0	0	0	0	0	0	0	0	0	0	0	0	0	0	0	0	0	0
ENSG00000152689	14	13	19	24	35	37	47	30	344	280	2409	215	144	177	209	239	14	23	18	39	57	23	57	21	318	432	958	1250	122	244	216	267
ENSG00000237133	3	0	6	1	2	2	0	1	3	0	10	0	10	8	4	2	1	0	0	2	3	0	1	5	5	3	2	3	0	2	0	7
ENSG00000119812	833	631	709	676	1157	1151	800	1120	1094	820	4168	698	1706	1490	1821	1847	790	1217	1004	996	2266	984	1658	872	1221	1730	814	2511	2273	2237	2360	2501
ENSG00000238038	4	4	5	3	3	1	0	0	0	0	2	2	5	3	2	4	0	0	0	3	3	1	0	1	1	1	0	2	1	2	1	1
ENSG00000187999	5	3	0	4	0	0	3	1	5	1	19	1	7	10	11	11	1	1	1	4	0	0	0	0	3	3	2	16	0	5	3	5
ENSG00000203386	3	3	1	4	0	0	0	0	2	0	0	0	0	1	0	0	0	2	4	4	0	0	0	0	0	3	0	1	0	1	0	0
ENSG00000239649	1	1	1	0	0	0	0	0	0	0	3	0	2	0	0	0	1	0	0	0	0	0	2	0	0	0	1	1	0	1	0	0
ENSG00000235064	0	0	0	0	0	0	0	0	0	0	0	0	0	0	1	0	0	0	0	0	0	0	0	0	0	0	0	0	0	0	0	1
ENSG00000237790	0	0	0	0	0	0	0	0	0	0	0	0	0	0	1	0	0	0	0	0	0	0	0	0	0	0	0	0	0	0	0	0
ENSG00000226785	0	0	0	0	0	0	0	0	0	0	0	0	0	0	0	0	0	0	1	0	0	0	0	0	0	0	0	0	0	0	0	0
ENSG00000228262	1	0	0	0	0	0	0	0	0	0	1	0	0	0	0	0	0	0	0	0	0	1	0	0	0	0	1	1	0	0	0	0
ENSG00000272027	0	0	0	0	0	0	0	0	0	0	1	0	0	0	0	0	0	0	0	0	0	0	0	0	0	0	0	0	0	0	0	0
ENSG00000232153	0	0	0	0	0	0	0	0	0	0	0	0	0	0	0	0	0	0	0	0	0	0	0	0	0	0	0	0	0	0	0	0
ENSG00000226994	0	0	0	0	0	0	0	0	0	0	1	0	0	0	0	0	0	0	0	0	0	1	0	0	0	0	0	0	0	0	0	0
ENSG00000264352	0	0	0	0	0	0	0	0	0	0	0	0	0	0	0	0	0	0	0	0	0	0	0	0	0	0	0	0	0	0	0	0
ENSG00000237320	0	0	0	0	0	0	0	0	0	0	0	0	0	0	0	0	0	0	0	0	0	0	0	0	0	0	0	0	0	0	0	0
ENSG00000196085	0	0	0	0	0	0	0	0	0	0	0	0	0	0	0	0	0	0	0	0	0	0	0	0	0	0	0	0	0	0	0	0
ENSG00000265301	0	0	0	0	0	0	0	0	0	0	0	0	0	0	0	0	0	0	0	0	0	0	0	0	0	0	0	0	0	0	0	0
ENSG00000207255	0	0	0	0	0	0	0	0	0	0	0	0	0	0	0	0	0	0	0	0	0	0	0	0	0	0	0	0	0	0	0	0
ENSG00000229013	0	0	0	0	0	0	0	0	0	0	0	0	0	0	0	0	0	0	0	0	0	0	0	0	0	0	0	0	0	0	0	0
ENSG00000234587	0	0	0	0	0	0	0	0	0	0	0	0	0	0	0	0	0	0	0	0	0	0	0	0	0	0	0	0	0	0	0	0
ENSG00000233752	0	0	0	0	0	0	0	0	0	0	0	0	0	0	0	0	0	0	0	0	0	0	0	0	0	0	0	0	0	0	0	0
ENSG00000260025	0	0	0	0	2	2	0	3	0	1	1	1	0	0	0	0	0	0	0	0	0	1	1	0	2	1	1	2	0	0	0	0
ENSG00000150938	7	6	10	4	531	660	354	737	706	601	2364	481	10	20	12	19	8	6	6	15	803	404	552	376	622	1007	593	1584	11	27	26	19
ENSG00000273090	2	0	0	0	7	1	0	1	2	1	2	0	0	0	0	0	0	0	0	1	0	0	0	0	0	3	0	0	0	0	0	0
ENSG00000171055	406	339	302	309	807	871	590	918	478	386	2190	325	490	550	581	707	223	329	274	303	1399	607	950	600	402	584	629	1412	441	546	472	657
ENSG00000270677	0	0	0	0	0	0	0	0	0	0	0	0	0	0	0	0	0	0	0	0	0	0	0	0	0	0	0	0	0	0	0	0
ENSG00000205221	0	0	0	0	0	0	0	0	0	0	0	0	0	0	0	0	0	0	0	0	1	0	2	0	0	0	0	0	0	0	0	0
ENSG00000279519	65	47	81	48	157	203	123	182	79	87	284	59	99	76	54	97	21	23	15	42	222	107	152	110	74	122	62	198	30	87	21	84
ENSG00000115808	1459	1119	1311	1096	2113	2163	1638	2260	1377	1222	4772	954	1858	1857	1882	2228	505	743	726	975	4038	1731	3062	1637	1587	2203	1112	4022	1341	1912	1531	2023
ENSG00000252756	2	0	2	0	1	4	0	1	0	0	1	2	1	0	0	1	0	0	0	2	5	1	2	0	4	2	0	3	0	2	0	4
ENSG00000008869	838	523	690	593	1629	1706	1308	1870	1062	928	4708	742	1201	1146	1117	1414	374	434	451	621	3465	1401	2394	1271	1101	1513	1505	3242	912	1262	1172	1388
ENSG00000152133	598	351	532	404	710	758	639	801	671	531	2200	405	554	478	641	663	404	594	562	417	1605	664	1168	627	624	935	506	1632	555	651	723	684
ENSG00000055332	1922	1454	1697	1484	3037	3093	2447	3405	2707	2297	9943	1708	4511	4302	4486	5077	633	910	860	1421	5499	2098	3734	1753	2928	4210	2009	7189	3086	4600	3434	4694
ENSG00000224891	4	1	0	1	2	9	7	8	8	6	22	0	5	4	4	1	0	3	3	2	23	10	4	2	7	6	5	9	5	6	9	10
ENSG00000138068	0	0	0	0	0	0	0	0	0	0	0	0	1	0	0	0	0	0	0	0	0	0	0	0	0	0	0	3	0	1	0	0
ENSG00000233159	0	0	0	0	0	0	0	0	0	0	1	0	0	0	1	0	0	2	0	0	0	0	0	0	0	0	0	0	0	0	1	1
ENSG00000218739	1105	735	848	680	2028	1939	1570	2169	1509	1188	5088	828	2038	1697	2074	2214	706	873	728	796	3640	1347	2672	1405	1450	1901	1159	3136	1373	1510	1559	1925
ENSG00000115816	1410	924	963	855	1649	1902	1487	1990	1789	1309	5019	921	2424	2238	2737	2875	2063	2805	1963	1882	5277	2038	3434	2243	1510	2343	1162	3761	3178	3150	3956	3693
ENSG00000272054	106	50	63	39	111	177	115	175	87	82	453	70	92	104	105	115	26	16	30	51	186	101	120	110	83	126	115	269	46	101	76	126
ENSG00000003509	392	263	337	258	476	510	378	514	384	284	1268	191	494	537	655	639	230	341	254	338	863	405	588	440	332	465	302	834	527	553	600	719
ENSG00000115825	1545	1289	1299	1221	3577	3715	2934	3978	2228	2028	7249	1380	3040	2975	3402	3862	906	1619	1106	1526	7827	3165	5902	3056	2199	3131	1798	5467	2579	3668	3814	4088
ENSG00000232028	2	2	1	0	0	0	1	0	0	0	0	0	0	1	0	0	0	0	0	0	2	0	1	0	0	0	0	0	1	1	4	2
ENSG00000207364	1	0	0	0	0	0	0	0	0	0	0	0	0	0	0	0	0	0	0	0	0	0	0	0	0	0	0	0	0	0	0	0
ENSG00000115828	0	1	0	0	1	0	2	1	0	0	0	0	3	3	2	2	0	0	0	0	0	1	1	0	0	0	0	1	4	2	13	5
ENSG00000253078	0	0	0	0	0	0	0	0	0	0	0	0	0	0	0	0	0	0	0	0	0	0	0	0	0	0	1	0	0	0	0	0
ENSG00000236213	0	0	0	0	0	0	0	1	0	0	0	0	0	0	1	0	0	0	0	1	0	0	0	2	0	0	0	0	0	0	0	0
ENSG00000163171	275	245	261	274	1405	1649	1170	1674	219	174	1168	156	271	364	297	420	67	141	91	254	2421	1063	1919	1039	155	222	230	567	152	386	235	398
ENSG00000236572	0	0	0	0	0	0	0	0	0	0	0	1	0	0	0	0	0	0	0	0	0	0	0	0	0	0	0	1	0	0	0	0
ENSG00000225402	0	0	0	0	0	0	2	0	0	0	0	0	0	0	0	0	0	0	0	0	2	0	1	0	0	0	0	0	0	0	0	0
ENSG00000237803	0	0	0	0	7	9	13	11	0	0	1	0	0	0	0	0	0	0	0	0	13	3	6	5	0	0	2	2	0	0	0	0
ENSG00000115841	25	22	20	19	0	0	0	0	77	57	352	54	29	50	54	50	28	45	57	45	0	0	0	0	87	118	90	247	40	43	71	54
ENSG00000235848	0	3	0	0	0	0	0	0	3	2	10	0	1	3	5	2	2	0	0	3	0	0	0	0	1	5	8	7	1	1	4	2
ENSG00000138061	30	40	27	42	0	0	1	0	5	0	11	1	1	0	0	1	4	31	9	40	0	0	0	0	5	6	6	23	0	1	2	0
ENSG00000232973	4	5	12	7	0	0	0	0	4	0	15	6	12	3	6	17	0	6	1	9	0	0	0	0	6	6	3	11	3	7	11	8
ENSG00000280276	0	0	0	0	0	0	0	0	0	1	0	1	1	2	0	4	0	0	0	0	0	0	0	0	0	0	0	0	0	2	0	0
ENSG00000229160	0	0	0	0	0	0	0	0	0	0	0	0	4	0	0	1	0	0	0	0	1	0	0	0	0	1	0	2	2	0	0	0
ENSG00000199603	0	0	0	0	0	0	0	0	0	0	0	0	0	0	0	0	0	0	0	0	0	0	0	0	0	0	0	0	0	1	0	1
ENSG00000273006	0	0	0	0	0	0	0	0	0	0	0	0	0	1	0	1	0	0	0	0	0	0	0	0	0	0	0	0	0	0	0	0
ENSG00000227292	0	0	0	0	0	0	0	0	0	0	0	0	2	0	0	1	0	0	0	0	0	0	0	0	0	0	0	0	2	0	0	0
ENSG00000227770	0	0	0	0	0	0	0	0	0	0	0	0	0	0	0	0	0	0	0	0	0	0	0	0	0	0	0	0	0	0	0	0
ENSG00000213594	0	0	0	0	0	0	1	0	0	0	2	0	0	0	0	0	0	0	0	0	0	1	2	0	0	0	0	0	0	0	0	2
ENSG00000119787	1283	849	1044	828	1869	1897	1372	1991	1106	1054	3565	731	2924	2877	3007	3825	619	673	652	964	3576	1404	2533	1480	1235	1920	678	2715	2160	3050	2338	3395
ENSG00000231367	0	0	0	0	0	0	1	0	0	0	1	0	7	12	11	13	0	0	0	0	0	0	0	0	0	0	0	0	5	18	5	28
ENSG00000235009	0	0	0	0	0	0	0	0	0	0	2	0	1	1	1	0	0	0	0	0	0	0	0	0	0	0	0	0	0	2	2	1
ENSG00000213553	85	74	48	56	75	72	37	66	71	60	144	48	39	57	27	69	82	80	47	66	104	62	83	42	48	75	41	110	67	50	51	69
ENSG00000271443	0	0	0	0	0	0	0	0	0	0	0	0	0	0	0	0	0	0	0	0	0	0	0	0	0	0	0	0	0	0	1	1
ENSG00000143889	1637	1362	1255	1376	1340	1381	1002	1390	0	0	1	0	988	915	1188	1128	732	1542	1064	1350	2488	945	1902	965	0	2	0	3	993	1092	1501	1305
ENSG00000235586	4	4	2	3	10	13	6	11	0	0	0	0	4	0	2	1	4	1	2	4	21	5	14	5	0	0	0	0	8	3	0	9
ENSG00000143891	4	1	6	2	36	69	32	75	9	6	17	3	456	341	541	474	2	1	3	9	57	25	36	23	13	17	9	22	446	352	646	470
ENSG00000232518	0	0	0	1	1	0	0	0	0	0	0	0	3	0	0	4	0	0	0	1	0	0	0	1	0	0	0	0	0	3	4	4
ENSG00000115875	4808	3281	3950	3631	5979	6186	4465	6459	6592	5025	17947	3756	7339	6387	7679	8432	6808	9184	7853	6598	15553	6497	10240	6406	6186	8757	3250	12152	7492	7576	8685	9210
ENSG00000152147	320	214	288	208	499	478	356	536	483	320	1491	256	759	658	897	891	280	357	441	428	888	401	857	416	409	605	284	905	1042	929	1191	1119
ENSG00000227238	0	0	0	0	0	0	0	0	0	0	0	0	1	0	0	0	0	0	0	0	0	0	0	0	0	0	0	0	0	0	1	0
ENSG00000232114	0	0	0	0	0	0	0	0	0	0	2	0	0	0	0	0	0	0	0	0	0	0	0	0	0	0	0	0	0	0	0	0
ENSG00000163214	471	335	480	381	660	782	524	801	610	485	2210	395	738	725	757	913	191	256	307	423	1252	528	860	423	491	802	565	1336	617	838	656	975
ENSG00000223922	8	1	1	1	5	4	3	1	7	4	15	0	9	4	2	4	0	1	0	3	5	4	5	5	3	9	7	8	0	10	4	11
ENSG00000225284	0	0	0	0	1	2	0	0	1	2	5	0	2	1	0	0	0	0	0	0	3	0	0	3	2	0	0	0	0	0	0	1
ENSG00000188010	49	36	50	33	17	16	15	24	55	22	162	27	100	64	100	90	83	79	117	96	28	17	22	19	39	58	41	94	112	71	159	116
ENSG00000207295	0	0	0	1	0	1	0	0	0	0	0	0	0	0	2	0	0	0	0	1	0	0	0	0	0	0	0	0	0	0	0	0
ENSG00000214694	3	2	7	7	9	7	2	4	9	4	24	1	0	6	2	3	1	0	2	2	13	8	9	4	12	12	8	25	0	1	2	1
ENSG00000265905	0	0	0	0	0	0	0	0	0	0	0	0	0	0	0	0	0	0	0	0	0	0	0	0	0	0	0	0	0	0	0	0
ENSG00000269210	0	0	1	0	0	0	0	0	0	0	0	0	0	0	0	0	0	0	0	1	2	0	0	0	0	0	0	0	0	0	0	0
ENSG00000115904	1239	1096	1080	912	2085	2141	1627	2186	7010	5714	37006	4387	2063	1958	1832	2254	334	425	370	770	3552	1589	2306	1571	9888	13102	12339	26309	1263	2050	1511	2182
ENSG00000229692	68	67	67	50	200	177	132	213	424	385	2370	268	94	98	69	92	21	27	15	57	285	125	182	126	585	784	736	1588	61	121	59	131
ENSG00000206985	0	0	0	0	0	2	0	2	6	6	28	0	2	3	1	2	1	0	0	2	2	5	0	1	8	12	17	28	1	3	3	2
ENSG00000225900	2	2	1	0	2	3	3	2	16	7	80	7	3	6	4	5	1	0	0	0	5	6	4	1	21	36	41	90	2	7	5	6
ENSG00000202309	0	0	0	0	0	0	0	0	0	0	2	0	0	1	1	0	0	0	0	0	0	0	0	0	0	0	0	0	0	0	0	0
ENSG00000205111	2	0	2	0	1	0	2	1	11	15	73	7	12	9	10	6	0	3	1	0	3	1	6	1	27	21	23	42	7	14	11	11
ENSG00000011566	357	299	331	281	201	237	152	251	319	326	1508	221	332	366	432	516	118	153	138	299	474	200	289	186	291	433	422	1013	284	467	296	490
ENSG00000252473	1	8	1	9	2	2	5	7	2	8	22	2	6	5	4	4	3	0	2	2	5	5	1	5	1	2	2	12	1	2	1	2
ENSG00000273035	7	5	3	2	1	0	0	3	0	0	13	0	7	9	3	3	0	1	0	4	1	0	0	1	0	1	1	8	4	7	4	7
ENSG00000231312	84	74	66	62	2	4	6	9	46	30	114	14	132	99	89	120	56	57	38	63	11	7	8	4	28	33	30	81	70	64	76	101
ENSG00000152154	11	21	11	23	0	0	1	0	10	4	8	0	17	3	2	13	3	0	0	8	0	0	0	0	0	1	1	2	0	4	1	0
ENSG00000138050	274	225	223	197	626	647	528	731	380	271	1210	238	370	379	366	468	277	421	306	424	1633	594	995	716	273	389	239	710	397	491	395	585
ENSG00000227028	1	2	0	2	0	0	1	0	8	7	37	3	0	0	1	1	1	0	0	1	0	1	2	0	9	2	6	15	0	0	1	0
ENSG00000235653	0	0	0	0	0	0	0	0	0	0	1	0	0	0	0	0	0	0	0	1	0	0	0	0	0	0	0	6	0	0	0	0
ENSG00000183023	0	0	4	0	2	2	2	1	76	56	122	29	0	1	1	1	0	0	0	0	2	0	0	0	43	71	44	140	0	2	0	0
ENSG00000233128	0	0	0	0	0	0	0	0	0	0	0	0	0	0	0	0	0	0	0	0	0	0	0	0	0	0	0	0	0	0	0	0
ENSG00000237442	0	0	0	0	0	0	0	0	0	0	0	0	0	0	0	0	0	0	0	0	0	0	0	0	0	0	0	0	0	0	0	0
ENSG00000236090	1	1	0	0	1	0	1	2	3	2	1	0	0	2	2	3	0	0	1	0	0	3	0	0	1	0	0	2	1	0	2	2
ENSG00000236533	1	0	0	0	0	0	0	0	0	0	0	0	0	0	0	0	0	0	0	0	0	0	0	0	0	2	1	0	0	0	0	1
ENSG00000207218	0	0	0	0	0	0	0	0	0	0	0	0	0	0	0	0	0	0	0	0	0	0	0	0	0	0	0	0	0	0	0	0
ENSG00000223245	0	0	0	0	0	0	0	0	0	0	0	0	0	0	0	0	0	0	0	0	0	0	0	0	0	0	0	0	0	0	0	0
ENSG00000214691	0	0	0	0	0	0	0	0	1	0	0	0	0	0	0	0	0	0	0	0	0	0	0	0	0	0	0	0	0	0	0	0
ENSG00000234362	0	0	0	0	0	0	1	0	0	1	0	0	0	0	0	0	0	0	0	0	0	0	0	0	0	0	0	0	0	0	0	0
ENSG00000205086	0	1	0	0	0	0	0	0	0	0	3	0	0	0	0	0	0	0	0	0	0	0	1	0	0	0	4	0	0	0	1	0
ENSG00000226398	0	0	0	0	1	0	2	1	0	0	0	0	0	0	0	0	0	0	0	0	3	2	1	1	0	0	1	0	0	0	0	0
ENSG00000162878	0	0	0	0	0	0	0	0	0	0	3	0	5	4	3	6	0	0	0	0	0	0	0	0	1	2	1	21	0	0	0	2
ENSG00000224875	3	5	5	0	1	5	1	3	10	6	94	22	1	2	4	4	1	2	0	4	2	2	2	5	25	20	35	99	0	4	11	4
ENSG00000143924	1324	915	1012	943	1664	1764	1286	1870	4951	3909	23462	3311	3125	3058	3225	4023	462	760	655	988	3513	1487	2511	1408	5337	7877	6932	17117	2448	3619	2836	4143

ENSG00000115944	1220	743	875	768	887	946	675	914	1534	1094	6055	1075	1594	1399	1740	1824	1296	1669	1248	1020	2198	813	1512	785	1512	2199	1530	3791	1970	1725	2399	2107
ENSG00000221300	15	10	8	8	0	0	0	0	0	0	0	0	0	1	0	0	2	4	0	5	0	0	0	0	0	0	0	0	0	0	0	0
ENSG00000171126	351	260	329	279	0	0	0	0	2	2	12	3	19	23	7	13	95	222	266	325	0	0	0	0	4	2	2	14	10	16	8	27
ENSG00000232526	22	25	11	12	0	0	0	0	0	2	1	0	0	1	3	0	2	3	2	16	0	0	0	0	0	0	0	0	0	0	0	2
ENSG00000236913	6	12	8	10	0	0	0	0	0	0	1	0	0	0	0	0	0	3	2	8	0	0	0	0	0	0	0	0	0	0	0	0
ENSG00000057935	2898	2454	2809	2371	1051	1108	855	1303	520	445	1740	270	665	662	656	953	904	1367	1630	1913	1530	644	1114	635	369	600	469	1145	465	656	510	795
ENSG00000273913	3	4	2	0	0	0	0	0	0	0	1	0	0	0	0	0	0	1	0	1	0	0	0	0	0	0	0	0	0	0	0	0
ENSG00000215263	1	2	2	2	0	0	0	0	1	0	2	0	1	0	0	0	0	4	1	13	0	0	0	0	1	0	1	2	0	1	0	1
ENSG00000226523	5	1	1	0	0	0	0	0	1	0	1	0	0	1	0	1	0	0	0	4	0	1	1	1	1	2	0	3	0	0	2	1
ENSG00000200550	9	11	6	7	3	2	2	3	2	2	6	0	2	2	2	3	3	2	1	4	7	4	4	3	2	2	1	2	0	7	0	6
ENSG00000162881	4	6	8	1	2	0	2	7	10	6	19	0	11	3	3	9	1	1	3	9	3	9	1	2	3	3	7	12	5	2	6	6
ENSG00000162882	5	4	8	2	8	9	1	6	4	5	34	3	8	8	3	13	2	2	2	11	7	7	4	9	4	11	9	29	1	2	5	12
ENSG00000226491	3	1	6	5	3	2	5	6	4	4	15	4	2	1	2	6	2	2	1	3	3	2	3	0	2	1	4	11	1	7	1	7
ENSG00000232202	0	1	0	0	5	9	6	5	2	5	6	0	6	9	5	8	1	0	0	0	9	10	6	4	0	0	1	1	5	2	2	5
ENSG00000224739	2	5	1	0	0	0	1	1	0	0	1	0	2	8	1	2	0	0	0	0	1	2	1	1	0	0	0	0	0	0	0	0
ENSG00000231826	7	7	6	13	1	2	1	0	1	0	1	0	20	21	3	24	0	1	0	0	0	0	0	0	0	0	0	0	1	2	0	2
ENSG00000233978	1	3	3	0	0	0	0	0	0	0	0	0	1	3	1	4	0	0	0	0	0	0	0	0	0	0	1	0	0	0	0	0
ENSG00000207087	6	5	10	4	1	0	1	0	2	2	0	0	4	10	8	10	0	0	0	0	0	0	0	0	0	0	0	0	2	1	2	1
ENSG00000230587	19	20	32	11	0	1	5	7	6	5	4	2	53	40	40	52	2	9	4	2	1	0	0	1	0	1	0	3	9	15	11	11
ENSG00000152518	7789	5976	7000	5535	1356	1253	943	1252	3863	3134	10075	2191	6840	6080	5648	6824	1223	1843	1351	2332	2023	821	1367	644	3327	4833	2529	7411	3325	4354	3014	4448
ENSG00000279873	14	7	8	14	76	100	80	111	34	43	151	25	38	39	41	67	4	3	2	6	123	58	71	69	24	35	40	81	14	25	19	32
ENSG00000234936	10	5	12	5	46	48	47	34	15	19	77	10	37	25	21	30	1	8	0	11	67	35	43	50	15	6	11	51	13	16	13	19
ENSG00000115970	719	540	681	513	873	970	695	1024	1038	831	3273	593	1430	1341	1577	1653	357	493	470	653	1741	749	1331	763	1010	1463	743	2516	1011	1589	1338	1582
ENSG00000252804	0	0	1	0	0	1	0	1	0	0	3	0	2	0	0	0	0	0	1	0	1	0	1	0	0	1	0	2	1	0	0	0
ENSG00000264071	1	0	1	0	1	1	0	2	0	1	1	1	0	0	1	1	0	0	0	0	1	0	0	0	1	1	1	2	0	1	2	1
ENSG00000207411	0	0	0	0	0	0	0	0	0	0	0	0	0	0	0	0	0	0	0	0	0	0	0	0	0	0	0	0	0	0	0	0
ENSG00000152527	96	134	86	157	0	0	0	0	19	25	46	24	19	43	19	57	14	43	15	64	0	0	0	0	11	7	8	16	7	32	14	26
ENSG00000223658	0	0	0	0	0	0	0	0	0	0	0	0	0	0	0	0	0	0	0	0	0	0	0	0	0	0	0	0	0	0	0	1
ENSG00000229695	0	1	1	0	0	0	0	0	0	0	0	0	0	0	0	0	0	0	0	0	0	0	0	0	0	0	0	1	1	0	0	2
ENSG00000252490	0	0	0	0	0	0	0	0	0	0	0	0	0	0	0	0	0	0	0	0	0	0	0	0	0	0	0	0	0	0	0	0
ENSG00000138036	206	143	153	145	164	177	120	203	142	115	449	74	363	333	427	464	190	198	221	167	311	154	228	194	68	100	120	225	276	333	412	456
ENSG00000138075	2	3	4	1	2	1	0	0	2	1	2	0	0	1	0	1	2	2	1	1	1	1	1	0	0	0	2	1	0	0	0	3
ENSG00000143921	0	0	1	0	0	0	0	0	0	0	4	1	0	0	0	0	0	0	1	0	0	0	0	2	0	1	0	0	0	0	1	0
ENSG00000138095	4110	2830	3419	2837	7240	6853	5920	7438	7103	5446	21640	3936	10528	9086	11103	11468	2706	3580	3462	4243	16356	6371	12092	6659	6656	9740	4562	15100	9385	11533	12261	13330
ENSG00000200924	4	6	11	2	3	11	15	5	8	13	46	9	17	5	5	13	0	3	2	11	17	2	4	15	16	14	9	25	12	17	8	26
ENSG00000264047	6	1	0	0	0	0	2	2	1	1	0	0	1	0	3	0	1	0	0	0	3	2	2	0	5	2	0	3	3	1	5	3
ENSG00000219391	0	1	0	0	0	0	0	0	0	0	0	0	0	0	0	0	0	0	0	0	0	0	0	0	0	0	0	0	0	0	0	0
ENSG00000252599	0	0	0	0	0	0	0	0	0	0	0	0	0	0	0	0	0	0	0	0	0	0	0	0	0	0	0	0	0	0	0	0
ENSG00000224637	2	0	0	0	2	3	5	1	0	1	0	0	1	0	0	2	0	0	0	0	3	0	1	0	1	0	0	0	2	0	1	1
ENSG00000273106	4	3	0	0	2	2	3	5	2	1	11	0	3	5	2	1	3	0	0	4	4	5	5	4	1	2	2	1	1	1	6	0
ENSG00000138032	1116	726	931	736	2774	3023	2162	3211	1477	1244	6328	946	1446	1317	1602	1716	305	408	449	561	4834	1957	3585	1891	1618	2448	1464	4404	1050	1494	1247	1680
ENSG00000226972	0	0	0	0	0	2	0	0	0	0	0	0	0	0	0	0	0	0	0	0	0	0	1	0	0	1	0	0	0	0	0	0
ENSG00000138079	9	16	9	2	9	13	5	23	12	6	58	8	9	27	6	14	2	0	4	10	14	14	8	9	6	7	19	31	2	12	8	5
ENSG00000138078	1194	878	973	863	985	1092	748	1076	1498	1236	5577	933	1841	1839	1940	2221	930	1358	1147	1135	1286	546	1045	510	1290	1957	1405	3472	1362	1866	1960	2155
ENSG00000143919	172	147	155	138	137	150	119	145	94	101	325	56	359	384	433	453	83	107	88	153	229	79	163	68	104	143	105	243	262	350	326	381
ENSG00000259439	1	0	2	0	0	0	0	0	5	7	18	3	1	1	1	0	0	0	0	0	0	0	0	0	6	16	4	24	0	0	0	0
ENSG00000278957	0	0	0	0	0	0	0	0	0	0	0	0	0	0	0	0	0	0	0	0	0	0	0	0	0	0	0	2	0	0	0	0
ENSG00000274520	0	0	0	0	0	0	0	0	0	2	2	0	0	0	0	0	0	0	0	0	0	0	0	0	0	0	2	1	0	0	0	0
ENSG00000277311	0	0	0	0	0	0	0	0	0	0	4	0	0	0	0	0	0	0	0	0	0	0	0	0	0	0	0	3	0	0	0	0
ENSG00000276229	0	0	0	0	0	0	0	0	0	0	0	0	0	0	0	0	0	0	0	0	0	0	0	0	0	0	0	0	0	0	0	0
ENSG00000277809	0	0	0	0	0	0	0	0	0	0	0	0	0	0	0	0	0	0	0	0	0	0	0	0	0	0	0	0	0	0	0	0
ENSG00000276048	0	0	0	0	0	0	0	0	0	0	1	2	0	0	0	0	0	0	0	0	0	0	0	0	0	0	1	5	0	0	0	0
ENSG00000274547	0	0	0	0	0	0	0	0	0	0	0	0	0	0	0	0	0	0	0	0	0	0	0	0	0	0	0	0	0	0	0	0
ENSG00000276670	0	0	0	0	0	0	0	0	0	0	0	0	0	0	3	0	0	0	0	0	0	0	0	0	0	0	1	0	0	0	0	0
ENSG00000236502	41	53	28	58	0	0	0	0	4	2	13	1	0	4	2	6	1	23	5	29	0	0	0	0	1	7	2	8	0	5	1	3
ENSG00000138083	215	468	239	486	0	0	0	1	26	21	82	25	14	28	33	48	25	90	39	163	0	0	0	0	14	33	16	76	10	20	8	28
ENSG00000225156	6	17	7	8	0	0	0	1	1	2	11	2	3	2	1	2	2	1	0	11	0	0	0	0	1	7	5	7	1	0	0	1
ENSG00000231848	0	0	0	0	0	1	0	0	0	0	1	0	0	0	1	0	0	0	0	1	0	0	1	0	0	0	0	0	0	0	0	0
ENSG00000170577	0	4	0	1	0	0	0	0	0	0	1	0	0	0	0	0	0	0	0	0	0	0	0	0	0	1	0	0	0	0	0	0
ENSG00000231156	0	0	0	0	0	0	0	0	0	0	0	0	0	0	0	0	0	0	0	0	0	0	0	0	0	0	0	0	0	0	0	0
ENSG00000205054	0	2	8	1	4	1	0	0	0	0	2	0	0	0	0	0	0	3	0	3	0	0	0	1	0	0	0	2	0	0	1	0
ENSG00000231054	0	0	4	1	0	0	0	0	0	0	1	0	0	0	0	0	2	0	0	0	0	0	0	0	0	0	0	1	0	0	0	0
ENSG00000068784	321	271	270	255	1184	1226	1062	1414	819	594	2716	509	960	976	1089	1289	192	313	283	283	2806	1081	2188	1221	758	1075	659	1899	1053	1204	1386	1417
ENSG00000239396	1	0	0	2	0	1	2	0	1	3	3	0	0	0	2	1	1	0	0	0	2	1	4	3	0	4	0	7	0	1	7	4
ENSG00000171132	303	177	356	181	990	1237	761	1339	614	458	2279	352	682	645	767	885	325	339	784	567	1558	741	965	680	503	729	299	1195	488	815	747	972
ENSG00000228481	1	0	4	3	3	1	8	12	3	1	16	1	6	6	4	5	2	0	0	0	9	5	1	3	3	4	1	21	0	6	5	5
ENSG00000233829	3	0	0	0	0	0	0	1	1	0	1	0	3	0	0	0	0	0	0	0	2	0	1	0	2	0	0	3	0	0	2	2
ENSG00000232696	0	0	1	0	1	0	0	2	1	0	5	0	4	1	1	0	0	0	2	1	2	1	0	0	1	0	2	3	0	3	4	2
ENSG00000231336	2	1	1	1	2	4	0	3	1	2	7	1	2	5	3	1	0	1	0	4	4	1	1	5	3	1	0	7	0	4	0	2
ENSG00000233335	0	0	0	0	0	0	0	0	0	0	0	0	0	0	0	0	0	0	0	0	0	0	0	0	0	0	0	0	0	0	0	0
ENSG00000278764	0	0	0	0	0	0	0	0	0	0	0	0	0	0	0	0	0	0	0	0	0	0	0	0	0	0	0	0	0	0	0	0
ENSG00000116016	191	301	222	308	527	633	282	621	917	806	6449	775	213	250	386	405	42	79	97	189	1580	867	1060	641	992	1815	1610	3784	231	491	570	672
ENSG00000228100	3	2	2	0	0	0	0	0	0	1	0	0	0	0	0	1	1	0	0	2	0	0	0	0	0	1	0	0	0	0	0	1
ENSG00000187600	0	2	4	0	0	0	1	0	0	0	0	0	1	2	1	0	1	1	0	0	0	0	1	1	0	4	0	0	1	0	0	0
ENSG00000241791	0	0	0	0	0	0	0	0	0	0	0	0	0	0	0	0	0	0	0	0	0	0	0	0	0	0	0	0	0	0	0	0
ENSG00000250565	167	167	153	187	212	259	155	243	96	68	246	53	277	251	227	298	130	134	90	173	259	120	199	142	46	101	30	139	118	165	174	195
ENSG00000253515	1	0	0	1	0	0	0	0	0	2	2	0	1	3	1	2	0	0	0	0	0	0	0	0	0	0	0	0	0	2	0	1
ENSG00000119729	267	217	226	224	889	984	638	916	362	356	1375	253	530	498	432	587	102	142	88	242	1332	533	840	534	281	429	236	719	175	345	211	412
ENSG00000250116	7	7	13	15	44	38	25	52	34	19	51	12	13	17	19	19	2	4	0	21	43	27	21	32	8	12	14	23	2	7	4	19
ENSG00000151665	405	292	287	308	525	598	397	621	359	266	1209	184	432	389	351	437	358	426	348	388	1137	484	754	601	268	398	259	710	257	372	292	323
ENSG00000119878	385	233	275	264	296	380	270	343	365	273	1399	258	438	435	459	608	395	485	377	429	657	299	472	381	343	447	311	796	534	497	662	673
ENSG00000279254	3	4	2	4	3	1	2	5	0	2	4	2	8	4	5	5	1	0	0	1	4	2	3	1	0	1	5	3	0	7	3	5
ENSG00000171150	468	383	423	319	550	559	412	589	535	462	2214	332	527	580	490	646	200	332	272	391	1110	465	780	441	466	759	593	1382	348	499	344	542
ENSG00000260977	2	2	1	1	11	14	7	11	1	7	24	5	10	12	8	14	0	0	0	4	16	7	12	12	3	4	11	17	7	4	5	10
ENSG00000222005	1	0	0	1	0	6	8	1	2	1	5	1	5	5	2	4	0	0	0	0	11	3	4	4	0	1	5	9	0	1	7	2
ENSG00000239332	0	2	0	2	13	10	3	6	3	4	2	2	0	3	5	2	0	2	0	1	17	6	10	4	2	3	2	5	2	4	4	8
ENSG00000226548	1	0	0	0	0	0	1	0	0	0	1	1	0	1	0	1	0	1	0	0	0	2	0	2	0	0	1	2	0	3	2	0
ENSG00000228925	12	11	10	10	19	34	18	32	16	13	65	4	15	13	14	15	0	7	4	15	45	25	17	22	13	15	7	42	5	19	16	18
ENSG00000180398	1204	799	944	744	1867	1906	1263	1877	1385	1155	4720	947	2061	1949	2216	2477	622	1000	794	932	3272	1303	2400	1174	1449	1938	1046	3258	2007	2241	2222	2357
ENSG00000068724	937	736	833	712	648	688	471	787	581	478	1454	363	1608	1405	1532	1806	199	304	279	452	1027	442	796	394	504	845	365	1222	1244	1432	1207	1550
ENSG00000272814	2	1	0	1	0	2	0	3	0	1	5	1	1	2	4	0	0	0	0	0	4	2	0	4	0	0	1	3	1	0	0	3
ENSG00000233845	4	2	1	0	6	4	2	0	2	3	8	0	5	2	2	6	1	1	0	4	6	3	3	8	6	3	5	14	1	10	3	8
ENSG00000239605	6	3	3	0	5	2	4	4	2	6	16	5	5	5	3	6	0	1	0	7	4	4	4	1	2	3	6	14	2	8	5	10
ENSG00000273269	0	0	0	0	0	0	0	0	0	1	1	1	0	0	0	0	0	0	0	0	0	0	0	0	0	0	0	0	0	0	0	0
ENSG00000225187	2	3	1	4	2	7	3	2	6	1	4	0	1	3	7	7	0	0	0	2	8	3	2	1	3	6	0	7	3	6	5	7
ENSG00000143933	6449	4363	5417	4660	10587	11282	7807	11753	8655	6472	28990	5478	8652	8294	9710	11156	5083	5899	6149	7748	24754	11087	18189	15278	9614	13481	5157	20378	9208	9237	11044	11363
ENSG00000234690	0	0	1	0	0	0	0	0	0	0	2	0	0	0	1	0	0	0	0	0	0	0	0	0	0	0	1	0	0	0	0	0
ENSG00000226087	0	0	0	0	0	0	0	0	0	0	0	0	0	0	0	0	0	0	0	0	0	0	0	0	0	0	0	1	0	0	0	0
ENSG00000236824	104	130	71	220	14	15	8	11	116	81	194	58	745	864	968	1129	147	246	182	209	12	10	11	6	83	121	16	79	567	650	1042	803
ENSG00000119888	0	0	0	0	0	0	0	0	206	182	514	113	2830	2456	3356	3252	0	0	0	0	0	0	0	0	154	232	110	326	3196	3231	3547	3576
ENSG00000222685	0	0	0	0	0	0	0	0	0	0	0	0	0	0	0	0	0	0	0	0	0	0	0	0	0	0	0	0	0	0	0	0
ENSG00000207923	0	0	0	0	0	0	0	0	0	2	1	0	4	4	5	8	0	0	0	0	0	0	0	0	0	0	1	0	1	8	4	5
ENSG00000277396	0	0	0	0	0	0	0	0	0	0	3	0	2	7	3	0	0	0	0	0	0	0	0	0	0	0	0	0	0	1	2	8
ENSG00000095002	357	234	249	223	3896	4006	3310	4558	2514	2003	6740	1506	3056	2877	3357	3787	156	217	227	300	11366	4196	8725	4045	2425	3443	1287	5760	3834	3787	4921	4465
ENSG00000184261	11	9	6	11	21	18	19	26	19	8	49	12	27	13	13	23	4	10	6	18	55	17	31	17	8	9	18	28	5	11	2	14
ENSG00000235760	9	2	2	4	6	2	3	11	4	5	11	4	3	8	1	0	2	0	1	3	10	8	10	9	0	0	1	3	5	3	0	5
ENSG00000230979	5	0	3	1	1	1	1	4	3	2	11	3	6	5	6	7	1	3	4	2	2	1	3	0	2	5	3	6	7	4	4	4
ENSG00000116062	2521	1955	2086	1901	4920	5512	3972	5972	4352	3548	11262	2389	4685	4296	4219	5487	1175	1773	1774	1891	10624	3954	7647	3727	3923	5734	2093	9161	3402	4476	3524	4834
ENSG00000224443	2	0	0	0	0	1	0	0	0	0	0	0	1	0	0	0	0	0	0	0	0	0	0	0	0	0	0	0	0	0	0	0
ENSG00000224058	0	0	0	0	0	0	0	0	0	0	0	0	0	0	0	0	0	0	0	0	0	0	0	0	0	0	0	0	0	0	0	0
ENSG00000283502	0	0	0	0	0	0	2	1	0	0	0	0	0	1	0	0	0	0	0	0	0	0	0	0	0	0	0	1	0	0	0	0
ENSG00000138081	883	674	708	669	1603	1672	1213	1697	979	830	4078	633	1228	1025	1217	1354	191	270	224	476	2429	1186	1737	1036	983	1410	1162	2571	720	992	876	1195
ENSG00000235369	2	1	3	4	8	8	5	13	10	12	51	2	11	10	11	7	2	1	0	3	22	7	8	6	8	8	11	33	0	14	13	7
ENSG00000236549	3	1	3	3	13	15	2	12	6	7	37	6	5	5	9	4	0	0	0	3	16	12	12	7	11	7	3	22	2	6	11	13
ENSG00000233230	62	65	60	63	145	162	119	162	76	63	303	56	90	79	88	108	14	20	5	38	151	94	121	69	33	98	72	160	44	70	72	66
ENSG00000226473	0	0	0	0	0	0	1	0	0	0	0	0	0	0	0	0	0	0	0	0	0	0	0	0	0	0	0	0	0	0	0	0
ENSG00000230773	2	1	0	0	4	2	5	2	1	1	9	0	5	5	2	5	0	0	0	0	10	5	11	3	1	1	0	6	2	5	4	4
ENSG00000275423	0	0	0	0	0	2	0	0	0	0	0	0	0	0	0	0	0	0	0	0	0	0	0	0	0	0	0	0	0	1	0	0
ENSG00000201010	0	0	0	0	0	0	0	0	0	0	0	0	0	1	1	0	0	0	0	0	0	0	0	0	0	0	0	0	0	0	0	0
ENSG00000170802	1050	722	754	670	1219	1270	899	1282	1345	1253	6073	948	1735	1604	1725	1986	992	1326	1056	1256	3178	1228	2201	1473	1876	2346	1563	4317	1678	1879	2040	2136
ENSG00000251889	1	1	0	2	1	2	0	0	0	1	10	1	1	4	0	1	0	0	0	0	0	3	1	1	3	1	4	7	3	3	1	0
ENSG00000272663	2	2	1	1	1	0	1	0	2	1	5	1	1	1	0	0	2	2	1	1	3	3	5	2	3	1	0	1	2	0	0	1
ENSG00000162869	324	204	255	212	740	679	640	775	927	711	2896	474	809	694	680	865	127	159	140	225	1847	681	1373	906	606	909	808	1628	436	642	519	695
ENSG00000202227	0	2	4	1	0	0	3	5	0	4	7	0	6	3	2	4	2	0	1	2	0	5	1	1	5	2	2	14	0	2	1	8
ENSG00000243244	12	24	25	19	235	296	204	358	53	49	125	17	52	30	31	63	4	9	2	27	236	107	164	106	29	37	20	87	21	39	25	46
ENSG00000068781	0	0	0	0	0	1	2	2	0	0	0	0	0	0	0	0	0	0	0	2	1	0	0	0	0	0	0	0	0	0	1	0
ENSG00000242441	0	0	0	0	1	0	0	1	0	0	1	0	0	0	0	0	0	0	0	0	0	0	0	0	0	0	0	0	0	0	0	0
ENSG00000279956	0	0	0	0	0	0	0	0	0	0	1	0	0	0	0	0	0	0	0	0	1	0	0	0	0	0	0	0	0	0	0	0
ENSG00000239995	0	0	0	0	0	0	0	0	0	0	0	0	0	0	0	0	0	0	0	0	0	0	0	0	0	0	0	0	0	0	0	0
ENSG00000138039	0	0	0	0	0	0	0	0	0	0	0	0	0	0	0	0	0	0	0	0	0	0	0	0	0	0	1	0	0	0	0	0
ENSG00000232285	0	0	0	0	0	0	0	0	0	0	0	0	0	0	0	0	0	0	0	0	0	0	0	0	0	0	0	0	0	0	0	0
ENSG00000282890	0	0	0	0	0	0	0	0	0	0	0	0	0	0	0	1	0	0	0	0	0	0	0	0	0	0	3	0	0	0	1	0
ENSG00000214602	0	0	0	0	0	0	0	0	0	0	0	0	0	0	0	0	0	0	0	0	0	0	0	0	0	0	0	0	0	0	0	0
ENSG00000170820	0	0	0	0	0	0	0	0	0	0	0	0	0	0	0	0	0	0	0	0	0	0	0	0	0	0	0	0	0	0	1	0
ENSG00000212036	0	0	0	0	0	0	0	0	0	0	0	0	0	0	0	0	0	0	0	0	0	0	0	0	0	0	0	0	0	0	0	0
ENSG00000282998	0	0	0	0	0	0	0	0	0	0	0	0	0	0	0	0	0	0	0	0	0	0	0	0	0	0	1	0	0	0	0	0
ENSG00000206915	0	0	0	0	0	0	0	0	0	0	0	0	0	0	0	0	0	0	0	0	0	0	0	0	0	0	0	0	0	0	0	0
ENSG00000282828	0	0	0	0	0	0	0	0	0	0	0	0	0	0	0	0	0	0	0	0	0	0	0	0	0	0	0	0	0	0	0	0
ENSG00000234253	0	0	0	0	0	0	0	0	0	0	0	0	0	0	0	0	0	0	0	0	2	0	0	0	0	0	0	0	0	0	0	0
ENSG00000212580	0	0	0	0	0	0	0	0	0	0	0	0	0	0	0	0	0	0	0	0	0	0	0	0	0	0	0	0	0	0	0	0
ENSG00000179915	0	0	0	0	0	0	0	0	0	2	4	1	0	15	4	13	0	0	0	0	1	2	1	0	0	2	11	2	1	3	2	16
ENSG00000230327	0	0	0	0	0	0	0	0	0	0	0	0	0	0	0	0	0	0	0	0	0	0	0	0	0	0	0	0	0	0	0	0
ENSG00000283058	0	0	0	0	0	0	0	0	0	0	1	0	0	0	0	0	0	0	0	0	0	0	0	0	0	0	0	0	0	0	0	0
ENSG00000216191	0	0	0	0	0	0	0	0	0	0	0	0	0	0	0	0	0	0	0	0	0	0	0	0	0	0	0	0	0	0	0	0
ENSG00000238165	0	0	1	0	0	0	0	0	0	0	0	0	0	1	0	0	0	0	0	0	0	0	0	0	0	1	0	0	0	1	0	0
ENSG00000231918	0	0	0	0	0	0	0	0	0	0	1	0	0	1	1	1	0	0	0	0	0	0	1	0	0	0	1	1	0	0	1	0
ENSG00000224344	0	0	0	0	0	0	0	0	0	0	0	0	0	0	0	0	0	0	0	0	0	0	0	0	0	0	0	0	0	0	0	0
ENSG00000223710	0	0	0	0	0	0	0	0	0	0	0	0	0	1	0	0	0	0	0	0	0	0	0	0	0	0	0	0	0	0	0	0
ENSG00000230840	0	0	0	0	0	0	0	0	0	0	0	0	0	0	0	0	0	0	0	0	0	0	0	0	0	0	0	0	0	0	0	0
ENSG00000226366	0	0	0	0	0	0	0	0	0	0	0	0	0	0	0	0	0	0	0	0	0	0	0	0	0	0	0	0	0	0	0	0
ENSG00000236538	0	0	0	0	0	0	0	0	0	0	0	0	0	0	0	0	0	0	0	0	0	0	0	0	0	0	0	3	0	0	0	0
ENSG00000202195	0	0	0	0	0	0	0	0	0	0	0	0	0	0	0	0	0	0	0	0	0	0	0	0	0	0	0	0	0	0	0	0
ENSG00000225444	0	0	0	0	0	0	0	0	0	0	0	0	0	0	0	0	0	0	0	0	0	0	0	0	0	0	0	0	0	0	0	0
ENSG00000225842	0	0	0	0	0	0	0	0	0	0	0	0	0	0	0	0	0	0	0	0	0	0	0	0	0	0	0	0	0	0	0	0
ENSG00000236837	0	0	0	0	0	0	0	0	0	0	0	0	0	0	0	0	0	0	0	0	0	0	0	0	0	0	0	0	0	0	0	0
ENSG00000204993	0	0	0	0	0	0	0	0	0	0	4	1	0	0	0	0	0	0	0	0	0	0	0	0	0	0	0	0	0	0	0	0
ENSG00000233083	0	0	0	0	0	0	0	0	0	0	0	0	0	0	0	0	0	0	0	0	0	0	0	0	0	0	0	0	0	0	0	0
ENSG00000264975	0	0	0	0	0	0	0	0	0	0	0	0	0	0	0	0	0	0	0	0	0	0	0	0	0	0	0	0	0	0	0	0
ENSG00000228033	0	0	0	0	0	0	0	0	0	0	0	0	1	0	0	0	0	0	0	0	0	0	0	0	0	0	0	0	0	0	0	0
ENSG00000232604	0	0	0	0	0	0	0	0	0	0	1	0	0	0	0	0	0	0	0	0	0	0	0	0	0	0	0	0	0	0	0	0
ENSG00000232668	0	0	0	0	0	0	0	0	0	0	0	0	0	0	0	0	0	0	0	0	0	0	0	0	0	0	0	0	0	0	0	0
ENSG00000251942	0	0	0	0	0	0	0	0	0	0	0	0	0	0	0	0	0	0	0	0	0	0	0	0	0	0	0	0	0	0	0	0
ENSG00000223897	0	0	0	0	0	0	0	0	0	0	0	0	0	0	0	0	0	0	0	0	0	0	0	0	0	0	0	0	0	0	0	0
ENSG00000115239	191	134	169	138	140	138	138	131	123	128	676	92	147	146	195	157	126	177	183	176	369	137	290	121	94	138	141	401	133	147	218	219
ENSG00000207456	0	0	0	0	0	0	0	0	0	0	0	0	0	0	0	0	0	0	0	0	0	0	0	0	0	0	0	0	0	0	0	0
ENSG00000270898	7	6	9	7	9	6	3	8	8	3	38	7	9	3	9	7	1	1	6	14	18	5	9	3	8	11	8	19	2	6	7	10
ENSG00000143942	529	273	402	293	232	214	182	253	245	169	717	154	393	343	449	495	695	831	692	669	640	210	477	270	275	440	110	525	583	505	713	678
ENSG00000068912	1128	763	925	731	842	910	676	947	656	566	2971	451	1024	932	1025	1315	557	774	683	787	1527	629	1142	587	735	997	746	1958	851	1021	924	1157
ENSG00000265452	4	6	7	3	4	10	6	6	15	10	50	8	8	3	1	5	3	0	2	12	10	4	12	10	10	11	12	28	7	5	1	10
ENSG00000119737	46	40	45	22	55	45	32	52	63	59	330	34	26	38	32	43	20	34	22	90	113	43	81	39	61	120	88	209	36	72	32	60
ENSG00000068878	2928	2508	2692	2449	3257	3361	2567	3401	1781	1435	7704	1212	3289	2970	3657	3726	992	1658	1424	2654	6230	2644	4311	2523	2419	3597	2078	5983	2491	3451	3093	3906
ENSG00000170634	213	173	192	171	172	170	115	182	142	112	635	90	194	207	200	263	180	251	222	218	352	173	252	188	141	192	177	393	164	202	195	233
ENSG00000235937	0	1	2	3	1	1	1	2	2	7	16	2	0	0	1	0	0	0	1	0	0	1	0	0	6	4	9	7	2	4	3	1
ENSG00000233266	13	5	9	10	1	9	4	2	1	5	26	1	3	8	10	9	9	6	13	13	6	2	3	4	1	6	9	13	3	4	8	4
ENSG00000241114	0	0	0	0	0	0	0	0	0	0	0	0	0	0	0	0	0	0	0	0	0	0	0	0	0	0	0	0	0	0	0	0
ENSG00000272156	6	3	10	1	1	6	3	3	5	3	46	6	6	6	5	12	3	1	0	2	3	0	3	0	6	6	25	20	3	3	2	7
ENSG00000252934	0	0	1	0	0	0	0	1	0	0	1	0	0	0	0	0	0	0	0	1	0	1	0	0	0	0	4	0	0	1	0	0
ENSG00000178021	7	4	7	4	0	3	3	1	3	1	11	1	1	8	6	3	4	0	1	6	2	3	4	9	4	5	3	11	3	2	1	2
ENSG00000177994	1	0	1	0	0	1	0	0	0	0	2	0	7	6	8	5	0	0	0	1	1	0	0	0	2	0	0	2	0	2	1	0
ENSG00000115306	18616	17496	17743	17745	10265	11146	7713	11569	6757	6385	26526	5145	8143	7995	7468	10872	4058	8190	6044	12336	15462	6792	11322	5526	6828	12437	5071	19905	4853	7196	5351	8239
ENSG00000228108	0	0	0	1	1	0	0	0	0	0	4	0	0	1	1	3	0	0	0	5	0	0	0	3	2	1	5	6	1	2	1	1
ENSG00000237887	0	0	0	1	0	1	0	0	0	0	10	0	0	3	0	2	0	0	0	0	0	0	1	0	3	3	1	6	0	0	0	1
ENSG00000234943	3	7	5	11	1	2	0	0	2	2	3	0	2	6	1	6	1	0	2	3	0	0	0	1	2	3	6	5	1	3	1	4
ENSG00000238018	105	97	97	114	42	47	42	41	38	38	163	30	23	34	30	48	17	19	20	109	89	48	34	49	26	53	26	89	7	37	15	42
ENSG00000214595	1270	1278	1149	1199	513	725	516	710	115	140	382	104	187	237	147	296	357	354	291	946	768	390	553	443	115	222	153	479	80	222	125	211
ENSG00000231334	5	6	2	7	1	2	3	1	0	0	2	0	1	1	1	4	0	0	1	6	8	2	2	4	1	2	3	3	0	1	0	5
ENSG00000252507	1	0	0	1	0	0	0	0	0	0	0	0	1	0	0	0	0	0	0	0	0	0	0	0	0	0	0	0	0	0	0	0
ENSG00000115310	7035	5243	5162	5181	3826	3692	2481	3854	3170	2575	10427	1877	4748	4446	4212	5747	1315	2142	1762	3059	5009	2079	3496	1771	2808	4304	2579	7073	3128	3743	2943	4090
ENSG00000200086	4	8	3	1	1	0	0	2	1	3	3	0	5	0	2	0	3	0	1	1	1	3	0	2	4	1	0	9	1	6	1	0
ENSG00000162994	226	156	229	156	122	141	134	122	262	250	1120	154	295	260	266	366	126	130	148	204	252	111	156	126	183	275	257	631	157	327	268	382
ENSG00000203327	17	10	5	9	12	8	6	14	23	21	114	12	24	24	22	25	2	3	2	10	23	9	10	14	9	14	22	59	4	31	15	26
ENSG00000227799	67	50	55	50	22	39	15	32	48	35	163	20	25	30	32	36	12	18	11	28	50	16	26	20	24	42	35	101	15	24	32	44
ENSG00000143947	15350	9884	10072	9230	7349	7633	5198	8059	15526	10693	44703	9274	15259	13906	16404	18112	18819	20397	19550	18195	19322	7757	13905	10823	13699	18689	9111	27837	19376	16154	24027	22629
ENSG00000085760	994	674	869	694	725	833	705	965	1239	963	3738	655	2148	1766	2402	2434	837	1104	1011	1129	2257	908	1903	999	1040	1564	886	2451	2598	2364	3145	2956
ENSG00000162997	68	50	65	61	53	69	48	71	116	73	412	56	75	85	74	101	16	16	14	81	104	62	46	54	47	79	107	223	36	51	48	71
ENSG00000240401	107	71	100	75	59	58	51	94	33	31	129	18	24	20	22	25	20	18	16	76	127	57	89	77	28	46	33	90	9	35	24	20
ENSG00000206964	6	9	12	2	2	5	4	5	5	2	11	0	3	3	0	1	1	4	0	3	8	2	4	0	4	1	7	7	1	1	1	3
ENSG00000115355	9218	6026	8533	6590	7639	7730	6043	7911	2470	2144	7474	1482	1672	1868	1729	2356	2708	3314	3628	6564	13646	5762	8697	6062	2010	2881	1224	5006	824	1713	994	1951
ENSG00000233594	0	0	0	0	0	0	0	2	0	0	0	0	0	0	1	0	0	0	0	2	1	0	1	0	1	0	1	1	0	0	0	0
ENSG00000238619	0	0	0	0	0	0	0	0	0	0	0	0	0	0	0	0	0	0	0	0	0	0	0	0	0	0	0	0	0	0	0	0
ENSG00000238493	0	0	0	0	0	0	0	0	0	0	0	0	0	0	0	0	0	0	0	0	0	0	0	0	0	0	0	0	0	0	0	0
ENSG00000239189	0	0	0	0	0	0	0	0	0	0	0	0	0	0	0	0	0	0	0	1	0	0	0	0	0	0	0	0	0	0	0	0
ENSG00000163001	860	593	710	556	264	273	185	324	653	454	1550	268	673	613	690	852	835	904	927	751	451	163	343	188	399	563	351	942	781	680	885	926
ENSG00000275052	2858	1850	2421	1965	3442	3453	2709	3646	2667	2253	10149	1784	3714	3273	3857	3940	1747	2293	2266	2267	7182	2762	5065	2602	2690	3948	2441	6660	3087	3379	3609	3804
ENSG00000212175	14	6	7	9	18	13	12	8	3	5	38	5	8	6	11	12	2	4	1	11	15	11	11	6	6	17	8	14	1	9	4	6
ENSG00000272606	45	47	38	23	15	21	11	10	31	21	134	13	28	42	36	44	14	14	19	29	15	13	15	17	18	30	24	61	14	20	31	33
ENSG00000138035	2156	1465	1604	1341	2262	2135	1748	2267	1285	980	4637	774	2696	1992	2960	2729	2693	3701	2910	2938	5470	2158	3597	2169	1670	2331	952	2989	3053	3159	3719	3678
ENSG00000115380	21	35	25	46	42	34	69	50	0	0	0	0	1	1	6	8	26	104	38	105	208	64	179	67	0	1	0	0	1	6	4	13
ENSG00000202344	0	0	0	0	0	0	0	0	0	0	0	0	0	0	0	0	0	2	0	1	0	0	0	1	0	0	0	0	0	0	0	0
ENSG00000272180	0	0	0	0	0	0	0	0	42	22	17	6	0	0	0	0	0	0	1	0	0	0	0	0	14	40	4	29	0	0	0	0
ENSG00000226702	0	0	0	0	0	0	0	0	0	0	0	0	0	0	0	0	0	0	0	0	0	0	0	0	0	0	0	0	0	0	0	0
ENSG00000207548	0	0	0	0	0	0	0	0	0	0	0	0	0	0	0	0	0	0	0	0	0	0	0	0	0	0	0	0	0	0	0	0
ENSG00000207798	0	0	0	0	0	0	0	0	0	0	1	0	0	0	0	0	0	0	0	0	0	0	0	0	0	0	0	0	0	0	0	0
ENSG00000211520	0	0	0	0	0	0	0	0	0	0	0	0	0	0	0	0	0	0	0	0	0	0	0	0	0	0	0	0	0	0	0	0
ENSG00000229805	0	0	1	0	0	0	0	0	0	0	0	0	0	0	0	0	0	0	0	0	0	0	0	0	0	0	0	0	0	0	0	0
ENSG00000271894	0	0	0	0	0	0	0	0	0	0	0	0	0	0	0	0	0	0	0	0	0	0	0	0	0	0	0	0	0	0	0	0
ENSG00000233251	1	0	2	0	0	0	0	0	312	225	172	59	0	0	0	0	0	0	0	0	0	0	0	0	200	335	57	232	0	0	0	2
ENSG00000055813	2	0	0	0	0	0	0	0	157	121	174	56	0	0	0	0	0	0	0	1	0	0	0	0	144	274	54	192	0	0	0	1
ENSG00000231570	0	0	0	0	0	0	0	0	0	0	0	0	0	0	0	0	0	0	0	0	0	0	0	0	0	0	0	0	0	0	0	0
ENSG00000271115	0	0	0	0	0	0	0	0	0	0	0	0	0	0	0	0	0	0	0	0	0	0	0	0	0	0	0	0	0	0	0	0
ENSG00000270569	0	0	0	0	0	0	2	0	1	1	1	0	1	0	0	0	0	0	0	0	0	0	0	0	0	0	0	0	0	0	0	0
ENSG00000212168	0	0	0	0	0	0	0	0	0	0	0	0	0	0	0	0	0	0	0	0	0	0	0	0	0	0	0	0	0	0	0	0
ENSG00000271615	10	0	6	4	0	0	0	0	1	0	0	0	1	0	1	0	0	1	3	3	0	0	0	0	0	0	0	1	0	0	0	1
ENSG00000028116	295	229	218	219	787	752	624	864	544	452	1972	342	543	574	600	801	318	562	424	473	2147	807	1560	844	566	840	477	1535	679	765	739	877
ENSG00000115392	919	733	816	759	1060	1086	837	1232	1049	864	2609	581	961	1050	1001	1472	556	696	700	964	2601	1052	1762	1190	895	1200	466	1987	783	1095	1016	1350
ENSG00000273063	3	2	1	0	6	3	0	10	0	0	4	0	1	1	0	3	2	2	6	3	5	5	2	0	2	0	1	0	10	5	4	4
ENSG00000233426	52	43	58	51	11	12	9	20	23	11	95	19	9	14	12	23	15	26	18	24	23	8	10	8	15	34	23	60	9	23	19	17
ENSG00000225226	0	0	0	0	0	0	0	0	0	0	0	0	0	0	0	0	0	0	0	0	0	1	0	0	0	0	0	0	0	0	0	0
ENSG00000233723	0	0	0	0	54	34	48	58	3	2	11	1	4	1	2	4	0	0	0	0	146	49	75	36	1	1	3	6	3	2	7	5
ENSG00000231043	1	0	1	1	2	1	1	0	1	0	7	0	2	0	0	2	3	0	2	0	6	2	1	1	0	2	1	1	0	3	3	3
ENSG00000222030	1	0	3	1	0	0	1	0	0	0	3	0	0	0	0	0	1	0	0	1	0	0	0	0	0	0	0	0	0	0	0	0
ENSG00000271955	19	15	20	13	0	3	0	0	0	0	0	0	0	0	0	0	3	4	5	10	0	0	0	1	0	0	0	0	0	0	0	0
ENSG00000233891	12	5	8	1	1	0	0	0	0	0	0	0	0	0	0	0	5	1	2	3	0	0	0	1	0	0	0	0	0	0	0	0
ENSG00000231815	4	1	1	1	0	0	0	0	0	0	0	0	0	0	0	0	0	0	0	1	0	0	0	0	0	0	0	0	0	0	0	0
ENSG00000270447	0	0	0	0	0	0	0	0	0	0	0	0	0	0	0	0	0	0	0	0	0	0	0	0	0	0	0	0	0	0	0	0
ENSG00000200101	0	0	1	0	0	0	0	0	0	0	0	0	0	0	0	0	0	0	0	0	0	0	0	0	0	0	0	0	0	0	0	0
ENSG00000228590	0	0	0	0	0	0	0	0	0	0	0	0	0	0	0	0	0	0	0	0	0	0	0	0	0	0	0	0	0	0	0	0
ENSG00000223929	0	0	0	0	0	0	0	0	0	0	6	0	0	0	0	0	0	0	0	3	0	0	0	0	0	1	3	0	0	0	0	0
ENSG00000200807	0	0	0	0	0	0	0	0	0	0	0	0	0	0	0	0	0	0	0	0	0	0	0	0	0	0	0	0	0	0	0	0
ENSG00000266078	0	0	0	0	0	0	0	0	0	0	0	0	0	0	0	0	0	0	0	0	0	0	0	0	0	0	0	0	0	0	0	0
ENSG00000119866	2094	1551	1991	1686	832	774	525	835	0	1	8	0	1	2	0	4	584	834	766	1317	1219	531	743	490	0	0	2	0	0	1	0	1
ENSG00000233953	20	23	28	27	13	19	24	36	0	0	0	0	0	0	0	0	2	4	2	26	27	16	12	14	0	0	0	0	0	0	0	0
ENSG00000242158	0	0	0	0	0	0	0	0	0	0	0	0	0	0	0	0	0	0	0	0	0	0	0	0	0	0	0	0	0	0	0	0
ENSG00000271134	0	0	0	0	0	0	0	0	0	0	0	0	0	0	0	0	0	0	0	0	0	0	0	1	0	0	0	0	0	0	0	0
ENSG00000213495	0	0	0	1	0	0	0	0	0	0	0	0	0	0	0	0	0	0	0	0	1	0	0	0	0	0	0	0	0	0	0	0
ENSG00000252718	0	0	0	0	0	0	0	0	0	0	0	0	0	0	0	0	0	0	0	0	0	0	0	0	0	0	0	0	0	0	0	0
ENSG00000271707	0	0	0	1	0	0	0	0	0	0	0	0	0	2	0	1	0	0	0	0	1	0	0	0	0	0	0	0	0	0	0	0
ENSG00000115421	1037	805	820	701	777	813	619	828	534	489	2659	435	778	692	771	975	519	691	610	749	1465	572	1008	549	715	1009	660	1638	609	767	677	874
ENSG00000228414	3	1	1	1	3	0	6	2	0	0	2	0	2	2	3	1	0	3	1	2	3	1	3	3	0	2	1	1	5	3	5	4
ENSG00000241524	0	0	0	0	0	0	0	0	0	0	0	0	0	0	0	0	0	0	0	0	0	0	0	0	0	0	0	0	0	0	0	0
ENSG00000237024	0	0	0	0	0	0	0	0	0	0	0	0	0	0	1	0	0	0	0	0	0	0	0	0	0	0	0	0	1	0	2	0
ENSG00000162924	1284	1001	978	1011	780	831	701	895	640	589	1953	312	1521	1519	1778	1891	252	320	315	593	1365	584	1045	505	616	900	515	1645	874	1257	1226	1580
ENSG00000201076	0	5	1	1	0	0	0	1	2	0	1	1	0	0	2	7	0	2	0	2	3	0	0	0	3	0	0	1	0	0	1	4
ENSG00000267520	0	0	0	0	0	0	0	0	0	0	0	0	0	0	0	0	0	0	0	0	0	0	0	0	0	0	0	0	0	0	0	0
ENSG00000237522	120	127	124	114	73	82	65	57	49	41	156	32	143	153	160	219	14	16	12	79	70	55	50	33	35	57	48	88	49	101	72	153
ENSG00000232713	57	70	49	55	25	40	30	44	24	24	75	19	96	104	83	119	8	18	4	31	45	32	26	21	12	27	22	40	31	56	31	92
ENSG00000162927	511	396	380	340	381	371	272	452	223	205	953	143	400	405	387	543	326	387	325	392	775	280	469	327	196	267	267	590	266	411	347	471
ENSG00000162928	1039	795	796	778	662	798	517	761	468	314	1682	299	828	636	791	846	563	780	579	678	1343	566	952	509	449	613	423	1092	719	737	805	879
ENSG00000162929	654	508	556	490	378	446	357	467	148	148	700	129	113	161	103	173	182	225	218	339	814	304	547	330	190	276	193	654	87	165	94	156
ENSG00000274769	9	12	12	11	2	3	2	0	3	3	9	0	1	3	2	1	2	0	5	1	3	2	4	0	0	0	5	11	0	3	1	3
ENSG00000212978	1	1	0	3	0	2	3	0	288	253	1097	194	364	350	438	483	0	1	0	1	1	0	0	0	307	360	238	655	305	347	384	396
ENSG00000237651	0	3	4	5	0	0	0	0	131	104	421	68	172	143	169	153	0	2	0	0	1	1	0	1	102	139	108	289	189	183	242	233
ENSG00000271889	1	3	1	2	0	0	1	0	19	11	103	16	15	11	12	14	0	2	1	0	0	0	0	0	9	17	25	47	6	17	6	17

ENSG00000173209	4673	3831	4294	3941	2952	3511	2468	3623	2650	2315	10488	1637	1757	1762	1783	2467	1483	1143	1146	2863	3684	1928	2267	1962	1422	2262	2178	5090	533	1407	629	1649
ENSG00000115464	4632	3333	4115	3212	4128	4323	3318	4538	2792	2493	11610	1904	4157	3748	4313	4555	1415	1817	1874	3349	7930	3322	5434	3202	3061	4439	3024	8861	2624	4031	3586	4518
ENSG00000273302	33	27	34	17	34	36	28	42	30	23	165	23	38	33	34	37	5	10	4	19	42	25	24	26	27	38	37	93	10	30	14	30
ENSG00000271146	2	2	5	1	1	2	1	3	2	4	14	1	3	1	0	1	0	2	2	4	3	3	3	5	3	3	3	11	0	4	2	5
ENSG00000234624	1	0	2	1	1	2	0	4	2	1	4	1	1	0	0	0	0	1	0	3	0	2	1	2	5	2	0	6	0	0	1	1
ENSG00000206937	1	3	1	0	1	0	0	1	0	0	5	0	0	2	0	2	1	0	0	0	0	0	0	1	2	2	0	8	1	2	1	2
ENSG00000270820	97	82	78	67	43	58	48	61	73	36	201	29	55	61	49	76	27	30	46	57	105	27	81	59	41	57	58	125	43	67	44	68
ENSG00000082898	14754	9756	12706	9812	13402	13628	10139	14484	10707	9326	33764	6268	10116	8985	10357	11562	7927	10716	10568	12615	27971	11463	19143	10973	10364	14879	6997	23689	7981	10238	9672	11717
ENSG00000271243	0	0	0	0	0	0	0	0	0	0	3	0	0	0	0	0	0	0	0	0	0	0	0	0	0	0	0	0	0	0	0	0
ENSG00000226884	0	0	0	0	0	0	0	0	0	0	0	0	0	0	0	0	0	0	0	0	0	0	0	0	0	0	0	0	0	0	0	0
ENSG00000206973	0	0	0	0	0	0	0	0	0	0	1	2	0	0	0	0	0	0	0	0	0	0	0	0	0	0	0	0	0	0	0	0
ENSG00000213486	0	0	0	0	0	0	0	0	0	0	0	0	0	0	0	0	0	0	0	0	0	0	0	0	0	0	0	0	1	0	0	0
ENSG00000227361	0	0	0	0	0	0	0	0	0	0	0	0	0	0	0	0	0	0	0	0	0	0	0	0	0	0	0	0	0	0	0	0
ENSG00000229831	0	0	0	0	0	0	0	0	0	0	0	0	0	0	0	1	0	0	0	0	0	0	0	0	0	0	0	0	0	0	0	0
ENSG00000170264	488	374	514	370	168	195	148	229	387	356	899	191	411	529	436	652	401	514	452	467	304	119	235	141	256	335	162	510	330	543	352	630
ENSG00000230702	0	1	2	0	2	2	0	3	0	0	9	0	1	0	0	2	0	0	0	0	3	0	0	2	0	2	1	2	0	2	0	0
ENSG00000277498	0	1	3	0	4	3	1	1	2	0	7	0	0	0	4	3	0	0	0	0	7	3	7	7	0	1	1	8	3	2	3	5
ENSG00000115484	7370	5361	5749	5077	5870	5804	4413	6432	4284	3234	12080	2313	9561	8486	9275	10748	4597	6113	5844	6393	10966	4185	7759	4152	4367	6359	2516	8679	8661	9635	9156	10465
ENSG00000236498	43	29	31	19	16	24	23	20	24	11	82	18	46	35	29	34	11	7	19	40	38	20	32	20	10	35	12	41	25	45	20	35
ENSG00000173163	256	181	245	230	316	279	278	330	176	167	575	124	246	207	247	319	411	558	458	349	639	240	463	219	168	264	135	374	269	288	336	318
ENSG00000278523	0	0	1	0	2	1	0	1	3	0	0	1	0	0	0	0	0	0	2	2	1	2	0	2	4	0	0	8	0	2	2	2
ENSG00000280544	1	2	5	0	1	0	2	1	1	3	4	0	1	1	0	1	0	0	0	2	4	3	0	2	0	0	2	7	0	1	3	3
ENSG00000229839	17	8	15	13	97	116	90	137	14	10	44	7	9	9	20	5	4	5	5	23	135	63	72	82	9	12	13	38	5	6	2	14
ENSG00000242735	0	0	0	0	0	0	0	0	0	0	0	0	0	0	0	0	0	0	0	0	0	0	0	0	0	0	0	0	0	0	0	0
ENSG00000271657	0	0	2	0	2	3	5	6	2	0	0	3	0	0	0	0	0	1	0	1	4	2	0	0	1	0	1	8	0	1	1	0
ENSG00000170340	1204	896	1057	827	5406	5003	3752	5385	406	351	1959	288	767	653	851	947	561	869	818	760	8491	3292	6138	2841	534	685	512	1326	832	872	934	1057
ENSG00000266097	0	1	7	3	11	24	12	18	4	0	2	1	1	0	2	1	0	0	0	1	20	7	8	12	2	3	0	3	0	0	3	3
ENSG00000239958	0	0	0	0	0	1	1	2	0	0	0	0	0	0	0	0	0	0	0	0	0	0	0	1	0	0	1	0	0	1	1	0
ENSG00000270335	0	0	0	0	1	2	3	3	0	0	3	1	1	0	0	0	0	0	0	0	0	1	1	0	1	1	0	0	0	0	1	0
ENSG00000228541	0	1	2	0	3	4	6	6	0	1	1	0	4	1	7	12	0	2	0	1	10	0	5	5	0	0	0	0	1	5	2	7
ENSG00000241625	0	0	0	0	0	0	0	0	0	0	1	0	0	1	0	0	0	0	0	0	0	0	0	0	0	0	0	0	0	0	0	0
ENSG00000186889	44	38	41	32	16	8	7	6	40	26	113	16	6	11	14	12	71	63	64	69	25	11	14	12	18	36	34	79	2	8	6	13
ENSG00000233702	0	0	0	0	0	1	1	1	0	0	0	0	0	0	0	0	0	0	0	0	0	0	0	0	0	2	0	0	0	0	0	0
ENSG00000229503	0	0	0	0	0	0	0	0	0	1	1	0	0	0	0	0	0	0	0	0	1	0	1	0	0	0	1	0	0	0	0	0
ENSG00000232144	0	0	0	0	1	0	0	1	0	0	0	0	0	0	0	0	0	0	0	0	1	0	1	1	0	0	0	0	0	0	0	0
ENSG00000230595	0	0	0	0	2	0	0	0	0	0	0	0	0	0	0	0	0	0	0	0	0	1	0	0	0	0	0	0	0	0	0	0
ENSG00000226622	0	0	0	1	0	0	0	4	0	0	0	0	0	0	1	0	0	0	0	0	0	1	0	1	0	0	0	0	0	0	0	0
ENSG00000115504	953	794	797	853	1007	1086	729	1125	848	695	2605	490	750	804	783	917	525	738	591	873	1698	669	1218	701	732	1181	614	1901	519	820	669	1000
ENSG00000252436	0	2	1	0	2	1	0	1	0	0	0	0	1	3	2	0	0	0	1	1	0	0	0	0	1	1	0	2	0	2	1	1
ENSG00000226605	1	0	0	0	0	0	2	0	0	0	0	0	1	0	0	0	0	0	0	1	1	0	0	0	0	1	0	1	0	0	1	0
ENSG00000237162	3	0	0	0	1	0	0	7	2	0	5	2	2	5	1	3	0	0	0	3	4	2	1	4	3	7	0	5	0	1	1	1
ENSG00000231609	30	26	29	24	18	31	13	32	23	19	105	15	20	6	24	29	9	8	9	18	39	19	14	10	23	25	42	61	3	24	7	21
ENSG00000115507	236	168	218	241	94	91	69	107	78	58	233	41	128	153	149	203	44	76	52	156	122	56	81	44	40	102	38	123	81	110	71	118
ENSG00000270437	2	1	2	1	0	0	0	0	0	0	1	0	0	2	0	0	0	1	0	2	1	0	0	0	0	0	1	0	0	0	0	0
ENSG00000232403	0	1	1	2	6	3	0	1	0	0	2	1	0	1	0	0	2	4	1	1	1	2	1	2	2	4	1	0	0	0	0	1
ENSG00000242412	4	12	7	5	10	7	10	9	9	3	36	5	7	7	5	11	1	5	2	6	14	2	7	3	2	3	3	23	2	4	4	3
ENSG00000143951	367	279	326	262	282	303	226	330	204	163	730	100	208	217	218	240	144	176	180	239	506	171	332	200	233	264	168	523	157	242	218	244
ENSG00000283636	3	3	2	3	7	7	3	3	3	1	9	4	4	3	0	5	0	0	0	4	2	2	0	9	3	2	1	5	1	2	1	3
ENSG00000233716	7	10	12	10	4	2	1	6	4	0	23	0	2	4	4	4	4	5	3	23	9	4	9	2	6	2	2	11	2	5	3	6
ENSG00000279485	4	0	1	4	0	0	2	4	0	0	2	0	1	1	2	1	0	0	0	3	4	1	1	4	3	3	2	8	2	5	1	4
ENSG00000014641	4261	2806	3422	2765	3877	3770	2822	4047	2701	1954	7913	1616	5047	4450	5563	5752	4938	6576	5669	4566	9239	3700	6475	3994	2442	3540	1612	5579	6138	5171	7413	6928
ENSG00000281071	0	0	0	0	0	1	0	0	0	0	2	0	0	1	1	1	0	1	0	3	1	0	0	0	0	0	0	0	0	0	0	0
ENSG00000228305	1	2	2	3	5	0	1	0	1	1	4	1	0	0	2	1	1	0	2	1	1	0	2	2	0	2	0	4	0	2	3	1
ENSG00000229920	2	2	4	6	3	3	3	2	11	5	14	1	2	4	1	9	1	3	4	7	2	2	4	2	2	5	2	18	1	3	1	4
ENSG00000234488	0	0	0	0	0	0	0	0	0	0	2	0	0	0	0	0	2	0	0	0	0	0	0	0	0	0	0	0	0	0	0	0
ENSG00000228872	1	0	2	1	0	0	0	0	0	0	2	0	0	1	0	0	0	0	0	0	0	0	0	0	1	0	0	1	0	0	0	0
ENSG00000229221	1	1	3	4	0	1	1	0	1	2	6	2	0	0	1	1	0	1	0	4	1	1	0	1	2	3	0	3	3	1	2	1
ENSG00000169764	2055	1579	1692	1493	2051	2314	1477	2301	4402	3502	14232	2664	3376	3089	3450	4068	993	1524	1334	1507	4803	1870	3251	1867	4634	6492	3044	10691	4417	4677	5096	5587
ENSG00000251775	20	15	33	19	3	2	4	5	12	11	34	8	5	6	5	5	7	7	9	18	6	4	3	4	7	5	6	19	3	5	4	11
ENSG00000143952	693	534	564	480	836	819	581	852	491	400	1786	289	770	751	765	982	331	559	455	618	1173	461	731	480	447	694	452	1168	593	732	604	895
ENSG00000228079	0	0	3	0	0	0	0	0	1	0	0	0	1	0	1	2	1	0	0	0	0	0	0	0	0	1	0	0	0	1	0	4
ENSG00000197329	864	722	737	735	467	479	373	510	330	308	1004	203	1758	1689	1762	2083	342	441	382	494	771	301	543	268	210	318	256	612	815	1123	954	1383
ENSG00000225889	57	49	29	48	54	88	63	95	5	3	3	4	55	49	42	63	12	32	10	26	66	26	24	31	2	3	1	10	10	17	13	35
ENSG00000230923	0	0	0	0	0	0	0	0	0	0	0	0	2	1	0	1	0	0	0	0	0	0	0	0	0	0	1	0	0	1	0	1
ENSG00000270354	0	0	0	0	0	0	0	0	0	0	0	0	0	0	0	0	0	0	0	0	0	0	0	0	0	0	0	0	0	0	0	0
ENSG00000238012	0	0	0	0	0	0	0	0	0	0	0	0	0	0	0	0	0	0	0	0	0	0	0	0	0	0	0	0	0	0	0	0
ENSG00000238201	0	0	0	0	0	0	0	0	0	0	0	0	0	0	0	0	0	0	0	0	0	0	0	0	0	0	0	0	0	0	0	0
ENSG00000264297	0	0	0	0	0	0	0	0	0	0	0	0	0	0	0	0	0	0	0	0	0	0	0	0	0	0	0	0	0	0	0	0
ENSG00000283222	0	0	0	0	0	0	0	0	0	0	0	0	0	0	0	0	0	0	0	0	0	0	0	0	0	0	0	0	0	0	0	0
ENSG00000236148	0	0	0	0	0	0	0	0	0	0	0	0	0	0	0	0	0	0	0	0	0	0	0	0	0	0	0	0	0	0	0	0
ENSG00000223935	1	0	0	2	0	0	0	0	0	1	0	0	2	6	3	6	1	0	2	5	0	0	0	0	0	0	2	2	2	6	5	6
ENSG00000237217	0	3	1	3	0	0	0	0	0	0	1	0	1	0	1	0	0	0	0	1	0	0	0	0	0	0	0	3	2	1	1	1
ENSG00000119862	465	510	431	502	9	12	2	9	28	33	109	14	73	96	86	140	213	436	274	486	3	0	4	3	22	40	32	68	87	200	104	178
ENSG00000223863	0	0	0	0	0	0	0	0	0	0	0	0	0	0	0	0	0	0	0	0	0	0	0	0	0	0	0	0	0	0	0	0
ENSG00000260101	30	8	40	16	10	26	22	21	4	4	20	3	10	10	17	18	19	27	50	26	36	38	31	30	8	5	10	16	16	8	19	19
ENSG00000119844	1347	860	1005	897	979	855	721	1021	804	573	3156	481	849	836	843	992	660	974	866	857	1832	765	1185	696	729	1057	774	2036	726	892	796	914
ENSG00000283204	6	5	5	2	3	2	2	0	2	1	9	0	3	1	2	3	0	0	0	3	3	3	3	3	1	2	2	6	0	3	2	5
ENSG00000279644	12	7	8	5	6	9	6	12	3	8	35	4	6	11	8	7	2	4	7	12	13	7	7	4	7	11	10	14	1	10	3	5
ENSG00000252414	3	4	4	1	4	2	2	1	7	3	19	8	6	4	3	10	0	1	1	2	5	7	3	5	1	2	9	12	0	4	5	7
ENSG00000233694	0	0	0	0	0	0	0	1	2	0	4	0	8	5	2	8	0	0	0	0	0	1	0	1	0	0	1	2	1	3	2	5
ENSG00000179833	1929	1931	1651	1984	1814	1851	1435	1886	1595	1320	6831	1121	4956	4650	4662	5688	386	600	417	1128	2590	950	1946	933	1390	2424	1391	4501	2841	4262	3081	4324
ENSG00000226756	4	4	2	3	1	3	2	3	6	3	35	11	6	8	5	9	2	1	2	5	4	3	1	3	7	7	13	27	7	19	9	15
ENSG00000227791	0	1	0	0	0	0	2	3	0	0	2	0	2	1	1	1	0	0	0	0	2	0	1	1	0	0	0	5	0	1	5	1
ENSG00000275588	0	0	0	0	0	0	0	0	0	0	0	0	0	0	0	0	0	0	0	0	0	0	0	0	0	0	0	0	0	0	0	0
ENSG00000199964	0	0	0	0	0	0	0	0	0	0	0	0	0	0	0	0	0	0	0	0	0	0	0	0	0	0	0	0	0	0	0	0
ENSG00000234572	0	0	0	1	0	1	0	0	1	0	2	0	4	0	1	3	0	1	0	1	0	0	0	0	0	0	0	0	3	2	6	12
ENSG00000237638	2	5	2	3	0	0	2	0	1	1	5	2	18	17	12	10	0	2	3	5	0	0	0	0	1	0	0	3	11	18	9	19
ENSG00000244534	0	2	0	0	0	0	0	0	0	0	0	0	2	2	0	0	0	1	0	0	0	0	0	0	0	0	0	0	1	4	2	0
ENSG00000227394	7	8	6	11	1	2	1	1	6	3	27	6	3	6	4	13	1	2	1	6	6	1	0	0	3	7	5	13	4	6	6	11
ENSG00000115902	3283	2732	3138	2733	1622	1772	1093	1629	4870	4061	17567	3410	3093	2842	2817	3767	1081	1645	1431	1984	2575	997	1687	819	3904	6616	4316	10992	1965	2377	2327	2477
ENSG00000252892	26	37	20	20	3	10	2	6	9	11	35	3	12	3	10	5	4	9	1	10	7	6	5	4	10	18	8	25	4	4	5	5
ENSG00000232613	19	11	18	13	7	4	3	7	4	4	24	3	11	11	6	14	2	4	3	13	8	1	3	5	8	14	10	20	3	10	8	9
ENSG00000011523	1336	1023	1041	1016	1057	1099	719	1193	1037	978	3931	693	1399	1341	1225	1639	280	367	297	536	1490	591	1099	594	784	1199	1047	2486	751	973	742	1086
ENSG00000138069	2041	1506	1660	1559	1819	1949	1441	2058	1754	1287	6746	967	2044	1906	1978	2559	1523	2001	1657	1555	3529	1471	2323	1212	1606	2278	1519	3996	1865	1953	1937	2371
ENSG00000272025	0	0	0	0	0	0	0	0	0	0	0	0	0	0	0	0	0	0	0	0	0	0	0	0	0	0	0	0	0	0	0	0
ENSG00000273763	9	3	5	7	1	4	1	2	5	3	37	3	6	11	12	4	1	3	2	10	5	1	0	0	2	6	0	19	5	7	4	5
ENSG00000172974	39	29	27	17	22	23	20	18	35	12	85	7	17	18	16	31	14	25	24	23	43	19	27	15	16	27	19	39	18	20	25	26
ENSG00000138071	19784	12894	17098	13621	16217	17317	12529	18655	13999	11024	56392	8556	18104	15492	19005	20733	10777	13720	15882	13131	36780	14346	27334	12315	15596	22523	12551	38053	15068	16130	17685	18568
ENSG00000198369	1475	1097	1537	1147	921	1095	615	1014	203	264	508	171	1323	1395	1428	1710	333	495	585	867	1471	644	812	625	160	381	70	422	986	1267	899	1427
ENSG00000232693	7	1	9	0	5	3	1	5	2	2	6	3	6	8	5	14	0	0	4	9	3	3	2	12	4	5	0	8	0	7	10	9
ENSG00000204929	3	0	0	0	0	0	0	0	0	0	0	0	4	4	8	9	0	1	0	0	0	0	0	0	0	0	6	0	1	2	1	3
ENSG00000234255	3	0	1	1	0	0	0	0	0	0	5	0	3	1	8	3	0	0	0	1	0	0	0	0	0	1	2	1	0	2	4	1
ENSG00000237979	0	0	0	1	0	0	0	1	0	0	1	0	1	1	1	1	0	0	0	0	0	0	0	0	0	0	1	1	0	0	3	0
ENSG00000223739	0	0	0	0	0	0	0	0	0	0	0	0	1	0	0	0	0	0	0	0	0	0	0	0	0	0	2	0	0	0	0	0
ENSG00000243029	0	0	0	0	0	0	0	0	0	0	0	0	0	0	0	0	0	0	0	0	0	0	0	0	0	0	1	0	0	0	0	0
ENSG00000251900	0	0	0	0	0	0	0	1	0	0	2	0	0	0	0	4	0	0	0	0	0	0	0	0	0	0	2	1	0	0	0	0
ENSG00000235725	0	0	0	0	0	0	0	0	0	0	0	0	0	0	0	0	0	0	0	0	0	0	0	0	0	0	0	0	0	0	0	0
ENSG00000281920	0	0	0	0	0	0	0	0	0	0	1	0	2	1	0	0	0	0	0	0	2	1	0	0	0	0	0	0	0	0	0	2
ENSG00000214533	0	0	0	1	0	0	0	0	0	0	0	0	0	0	0	1	0	0	0	0	0	0	1	0	0	0	0	0	0	0	0	0
ENSG00000280257	0	0	0	0	0	0	0	0	0	0	0	0	0	0	0	0	0	0	0	0	0	0	0	0	0	0	0	0	0	0	0	0
ENSG00000227293	0	0	0	0	0	0	0	0	0	0	0	0	0	0	0	0	0	0	0	0	0	0	0	0	0	0	0	0	0	0	0	0
ENSG00000225815	0	0	0	0	0	0	0	0	0	0	0	0	0	0	0	0	0	0	0	0	0	0	0	0	0	0	0	0	0	0	0	0
ENSG00000264525	0	0	0	0	0	0	0	0	0	0	0	0	0	0	0	0	0	0	0	0	0	0	0	0	0	0	0	0	0	0	0	0
ENSG00000232164	0	0	0	0	0	0	0	0	0	0	0	0	0	0	0	0	0	0	0	0	0	0	0	0	0	0	0	0	0	0	0	0
ENSG00000226819	0	0	0	0	0	0	0	0	0	0	0	0	0	0	0	0	0	0	0	0	0	0	0	1	0	0	1	0	0	0	0	1
ENSG00000143995	0	0	0	1	3	4	2	8	6	9	19	3	80	78	59	108	1	1	1	3	6	0	4	1	1	3	5	11	31	80	37	85
ENSG00000230749	0	0	0	0	0	0	0	0	0	0	2	0	0	2	1	0	0	0	0	0	0	0	0	0	0	0	0	0	0	0	0	2
ENSG00000232046	0	0	0	0	0	0	0	0	0	1	1	0	0	0	0	2	0	0	0	0	0	1	0	0	0	0	0	0	0	0	0	0
ENSG00000237179	0	0	0	0	0	0	0	0	0	0	1	0	0	0	0	0	0	0	0	0	0	0	0	0	0	0	0	0	0	0	0	0
ENSG00000224071	0	0	0	0	0	0	0	0	0	0	0	0	0	0	0	0	0	0	0	0	0	0	0	0	0	0	1	0	0	0	0	0
ENSG00000230355	0	0	0	0	0	0	0	0	0	0	0	0	0	0	0	0	0	0	0	0	0	0	0	0	0	0	0	0	0	0	0	0
ENSG00000230525	0	0	0	0	0	0	0	0	0	0	0	0	0	0	0	0	0	0	0	0	0	0	0	0	0	0	0	0	0	0	0	0
ENSG00000232688	0	0	0	0	0	0	0	0	0	0	0	0	0	0	0	0	0	0	0	0	0	0	0	0	0	0	0	0	0	0	0	0
ENSG00000223859	0	0	0	0	0	0	0	0	0	0	0	0	0	0	0	0	0	0	0	0	0	0	0	0	0	0	0	0	0	0	0	0
ENSG00000235885	0	0	0	0	1	0	0	0	0	0	2	0	0	0	0	1	1	0	0	1	0	0	2	0	1	1	2	2	0	0	1	0
ENSG00000236780	5	4	2	3	0	2	0	0	2	0	5	1	0	2	2	1	0	0	1	1	0	0	0	0	1	0	1	1	0	0	0	0
ENSG00000236605	0	0	0	2	0	0	0	0	0	0	0	0	0	0	0	0	0	0	0	0	0	0	0	0	0	0	0	2	0	0	0	0
ENSG00000143971	1073	775	784	734	676	677	570	788	502	445	1681	288	640	615	583	775	651	885	786	890	1550	653	1061	685	426	572	395	1066	466	606	576	705
ENSG00000224173	0	0	0	0	0	0	0	0	0	0	0	0	0	0	0	0	0	0	0	0	0	0	0	0	0	0	0	0	0	0	0	0
ENSG00000235495	0	0	0	0	0	0	0	0	0	0	2	0	0	0	0	0	0	0	0	0	0	0	0	0	0	0	0	0	0	0	0	0
ENSG00000237013	0	0	0	0	0	0	0	1	0	1	0	0	0	0	0	0	0	0	0	0	0	0	0	0	0	0	1	0	0	0	0	0
ENSG00000235968	0	0	0	0	0	0	0	0	0	0	0	0	0	0	0	0	0	0	0	0	0	0	0	0	0	0	0	1	0	0	0	0
ENSG00000197223	907	562	680	526	668	691	535	699	560	478	2080	355	677	693	826	885	914	1135	1059	1207	1724	717	1249	912	615	795	510	1486	977	899	1184	1125
ENSG00000243667	268	205	263	162	193	157	127	203	217	179	699	120	384	290	352	406	139	197	170	168	281	129	216	106	209	300	146	449	322	383	343	400
ENSG00000236259	1	1	3	2	2	1	0	3	0	0	4	0	2	0	2	1	5	1	1	5	3	2	1	2	0	1	1	6	1	3	0	0
ENSG00000273398	7	4	6	4	1	0	0	0	1	5	4	0	4	4	1	1	1	1	3	4	3	0	0	1	5	6	0	6	0	2	0	2
ENSG00000115946	833	508	704	514	541	508	436	569	574	424	1943	336	1206	1035	1458	1250	1025	1542	1348	1219	1361	555	970	505	741	1037	359	1332	1747	1609	2011	1790
ENSG00000221823	2389	1694	2234	1722	1895	1845	1335	1782	1618	1241	5628	975	2406	2112	2660	2767	768	1304	1253	1728	3299	1393	2422	1229	1761	2444	1261	3848	1897	2567	2305	2681
ENSG00000273275	0	0	0	0	0	0	0	0	0	0	0	0	0	0	0	0	0	0	0	0	0	0	0	0	0	0	0	0	0	0	0	0
ENSG00000273064	27	25	30	20	18	14	14	25	19	16	69	14	21	25	27	47	27	17	26	39	39	11	25	17	10	24	11	41	18	29	17	40
ENSG00000119865	1	0	2	0	0	1	3	1	8	12	19	8	1	7	2	3	0	1	0	6	2	5	3	4	3	8	5	17	0	5	2	4
ENSG00000203395	1	2	0	1	0	0	0	0	0	0	0	1	4	4	3	4	1	0	0	1	0	0	0	0	0	0	0	1	2	1	3	0
ENSG00000115956	1306	1184	940	1191	322	327	277	380	398	334	970	239	1825	1417	2160	1870	588	1911	861	1134	629	229	446	226	371	513	159	647	1043	944	1763	1263
ENSG00000229462	0	1	0	0	0	1	0	0	0	0	0	0	0	0	0	0	0	0	0	0	0	0	0	0	0	0	0	0	0	0	0	0
ENSG00000204923	804	565	654	567	112	119	81	120	179	148	484	125	256	242	186	300	231	289	239	324	166	70	119	75	123	193	90	395	134	191	144	186
ENSG00000169621	180	103	139	123	87	91	73	96	132	86	391	79	203	175	167	238	106	95	82	104	173	87	107	86	129	155	118	235	156	209	206	258
ENSG00000214525	2	2	4	4	2	3	0	2	1	2	6	0	2	0	1	3	0	1	1	0	4	2	0	2	2	3	1	4	0	3	1	1
ENSG00000169618	0	3	0	0	0	1	0	0	1	1	7	0	0	1	0	2	2	2	0	6	1	0	0	0	0	1	0	3	0	0	0	2
ENSG00000163219	682	453	590	466	1049	965	859	1045	552	357	2505	327	471	441	615	724	203	272	258	321	1645	617	1353	628	418	509	634	1410	438	479	556	619
ENSG00000237576	0	0	0	0	0	0	1	0	0	0	3	0	0	0	0	0	0	0	0	2	1	1	1	0	0	0	0	0	1	0	0	0
ENSG00000228329	4	0	1	1	2	1	0	1	0	1	1	0	0	0	0	0	0	0	0	0	0	0	1	3	0	0	0	1	0	0	0	0
ENSG00000163217	10	7	28	15	1	2	0	7	4	2	25	2	0	0	0	1	6	10	9	29	4	1	2	0	0	2	4	0	0	3	1	1
ENSG00000283586	0	0	1	0	1	0	0	0	0	0	0	0	0	0	2	1	0	0	0	0	0	0	1	1	0	1	0	0	0	0	0	0
ENSG00000183607	0	0	0	0	0	0	0	0	0	0	0	0	0	0	0	0	0	0	0	0	0	0	0	0	0	0	0	0	0	0	0	0
ENSG00000169605	0	0	0	0	0	0	0	0	0	0	0	0	0	0	0	0	0	0	1	0	0	0	0	0	0	0	0	0	0	0	0	0
ENSG00000169604	1152	1129	1224	1258	681	905	535	918	1392	1140	5345	894	163	308	264	443	283	567	517	1125	937	514	742	418	898	1443	1160	2858	183	497	337	579
ENSG00000266649	0	0	0	1	4	2	0	1	0	0	2	0	0	0	1	0	1	0	0	1	0	0	0	0	0	0	1	5	0	0	0	0
ENSG00000199460	0	0	1	0	0	0	0	0	0	0	5	0	0	1	0	0	0	0	0	1	1	0	0	0	0	0	1	2	0	0	0	2
ENSG00000271597	0	1	1	1	0	0	0	0	1	0	3	0	0	0	0	0	0	0	0	0	0	0	0	1	1	1	1	4	0	0	1	0
ENSG00000198380	3635	2713	2828	2777	2243	2191	1650	2336	1537	1302	5801	974	3417	3169	3955	4064	1064	1569	1283	2289	4032	1570	2499	1481	1612	2388	1137	4001	2413	3051	2998	3487
ENSG00000252250	3	2	1	1	1	1	0	3	2	1	5	0	0	2	1	1	1	0	0	1	2	2	0	0	0	0	0	0	0	0	0	0
ENSG00000169599	268	140	202	170	353	376	274	368	323	215	1018	201	634	504	558	652	460	491	426	366	1035	406	818	548	285	420	236	636	769	663	830	851
ENSG00000115977	1823	1474	1989	1502	2015	1947	1531	2309	3194	2512	14934	2083	1033	1147	1238	1480	482	695	804	1135	2382	961	1618	736	2986	4359	4185	11024	1157	1801	1456	1937
ENSG00000244236	0	0	0	0	0	0	3	2	1	0	7	0	0	0	0	0	1	0	0	0	0	1	0	0	0	0	0	1	0	0	0	1
ENSG00000207016	0	0	7	0	1	0	0	2	2	3	1	1	0	1	0	0	0	0	0	0	1	0	0	0	1	2	1	0	0	1	2	2
ENSG00000232228	3	2	3	6	1	2	2	7	4	0	16	3	3	1	7	1	2	0	1	2	11	2	0	3	8	7	4	23	1	1	1	11
ENSG00000229948	4	3	3	3	5	1	5	2	10	4	34	3	4	6	4	1	0	0	2	12	8	2	3	5	14	9	10	58	1	8	6	5
ENSG00000196975	608	519	503	474	392	431	241	463	828	642	4486	657	1166	1129	1265	1570	406	723	558	522	557	255	378	210	690	1066	863	2108	1035	1101	1129	1349
ENSG00000231024	1	3	5	0	0	0	0	1	1	0	5	1	0	0	1	1	0	1	0	0	0	0	0	0	0	0	0	0	0	1	0	0
ENSG00000087338	2271	1574	1749	1607	1654	1682	1187	1784	1556	1312	5529	914	1830	1660	1679	1963	1031	1411	1125	1593	2933	1153	1945	1112	1583	2257	1187	4076	1430	1622	1480	1740
ENSG00000275381	13	12	8	10	3	2	9	10	16	9	47	5	9	6	7	16	8	2	3	25	14	3	8	13	6	15	6	39	1	9	7	9
ENSG00000270723	6	3	2	3	7	4	4	0	5	1	13	1	6	2	4	6	0	6	2	3	8	2	4	6	1	3	2	15	3	3	1	7
ENSG00000124380	1108	734	981	711	696	637	549	697	622	490	2320	434	710	610	830	764	1072	1147	1081	1009	1542	586	1015	646	618	808	552	1633	849	879	962	1100
ENSG00000059728	880	571	564	522	178	188	132	222	243	171	1504	182	562	566	741	752	140	113	112	268	289	133	205	144	233	356	495	914	426	719	685	813
ENSG00000279229	2	0	4	1	2	2	2	3	2	0	9	0	0	0	1	1	0	0	0	0	1	2	3	3	1	1	0	4	0	0	3	0
ENSG00000244617	133	100	138	125	85	89	54	115	38	32	132	22	84	75	83	97	22	48	59	81	106	38	105	52	26	37	43	72	39	58	40	58
ENSG00000179818	1218	971	1335	942	2168	2100	1660	2442	805	660	3116	439	649	617	636	772	322	389	481	1100	2960	1381	1914	1563	584	809	775	1734	348	650	509	802
ENSG00000263669	0	2	2	0	1	1	1	1	0	0	4	0	0	1	1	1	0	0	0	1	0	0	0	0	0	2	0	1	0	0	0	1
ENSG00000169564	4467	3553	3658	3539	4337	4418	2750	4265	3763	2916	11267	2531	5227	4953	5131	6359	937	1803	1459	2487	5301	2202	3735	1774	2991	5077	2338	7676	3885	4679	3702	4515
ENSG00000233060	1	1	2	0	3	1	0	2	0	0	0	1	1	0	1	0	0	0	0	3	2	0	0	1	2	1	1	4	2	1	0	4
ENSG00000225392	0	1	0	2	0	0	0	0	0	0	1	0	0	0	0	0	0	0	0	0	0	0	0	0	0	0	0	1	0	0	0	0
ENSG00000231327	9	8	17	19	12	21	2	21	10	2	17	0	13	7	15	20	2	3	7	15	37	13	14	15	2	4	7	21	8	16	8	11
ENSG00000115998	276	171	269	200	217	219	180	239	239	201	1162	184	180	149	173	198	153	178	190	223	380	151	287	153	220	343	327	743	145	177	175	205
ENSG00000116001	4860	3046	3857	2976	4644	4617	3510	5103	2738	2340	11235	1833	3212	3035	3379	3787	2698	2808	3186	3704	9058	3834	6279	3985	2395	3517	2686	6491	2965	3602	3488	4152
ENSG00000221238	0	0	0	0	0	0	0	0	0	0	0	0	0	0	0	0	0	0	0	0	0	0	0	0	0	0	0	0	0	0	1	0
ENSG00000231414	2	0	0	0	0	0	0	0	0	1	2	1	0	0	1	0	0	0	0	0	0	0	1	0	0	0	0	0	1	0	1	1
ENSG00000116005	1233	896	1011	899	967	997	804	1073	906	811	3340	602	1004	1045	894	1309	479	686	612	746	1975	673	1301	616	729	1093	838	2042	703	827	705	874
ENSG00000143977	2536	1606	2124	1729	1800	1806	1308	1900	1492	1030	4663	860	1744	1563	2157	2011	2692	3163	3714	3612	4929	1982	3913	2867	1719	2140	814	3146	2456	2148	2996	2797
ENSG00000035141	2303	1574	1963	1720	1968	2040	1494	2141	1914	1370	5796	1085	2502	2241	2736	2969	2919	3608	3539	3025	5075	2046	3963	2091	1835	2808	1120	3497	3425	3376	3988	4026
ENSG00000233849	0	0	0	0	0	0	0	0	0	0	0	0	0	0	0	0	0	0	0	0	0	0	0	0	0	0	0	0	0	0	0	0
ENSG00000235289	0	0	0	0	0	0	0	0	0	0	0	0	0	0	0	0	0	0	0	0	0	0	0	0	0	0	0	0	0	0	0	0
ENSG00000229229	0	0	1	0	0	0	0	0	1	0	0	0	0	0	0	0	0	0	0	1	0	0	0	0	0	0	0	0	0	2	0	0
ENSG00000163235	64	128	66	126	435	563	352	628	219	161	4931	381	0	0	1	0	16	53	39	84	726	377	540	338	260	387	813	1607	0	2	1	3
ENSG00000224606	0	0	0	0	0	0	2	0	0	0	6	1	0	0	0	0	0	0	0	0	3	1	0	1	0	1	2	1	0	0	0	0
ENSG00000075340	30	153	34	169	0	7	5	2	258	298	642	201	1783	2035	1664	2538	2	32	8	52	0	4	1	3	78	183	29	258	773	1538	834	1717
ENSG00000235035	0	0	0	0	0	0	0	0	0	2	0	0	0	1	1	2	0	0	0	0	0	0	0	0	0	0	0	0	0	3	2	1
ENSG00000183733	2	0	0	0	0	0	0	0	0	0	0	0	0	0	0	0	0	0	0	0	0	0	0	0	0	0	0	0	0	0	0	0
ENSG00000228034	0	0	0	0	0	0	0	0	0	0	0	1	0	0	0	0	0	0	0	0	0	0	0	0	0	0	0	1	0	0	0	0
ENSG00000152672	6	1	3	12	0	0	1	0	0	0	0	0	0	0	0	0	0	0	0	3	2	0	0	0	0	0	0	0	0	0	0	0
ENSG00000231386	0	0	0	0	0	0	0	0	0	0	0	0	0	0	0	0	0	0	0	0	0	0	0	0	0	0	0	0	0	0	0	0
ENSG00000116031	4	1	2	3	0	0	0	3	0	0	0	0	1	0	0	0	0	3	0	2	1	0	0	0	0	0	0	0	0	0	0	0
ENSG00000237751	0	0	0	0	0	0	0	0	0	0	3	0	0	0	0	0	0	0	0	2	0	0	0	0	0	0	0	0	0	0	0	0
ENSG00000277764	0	1	1	0	0	0	0	0	0	0	0	0	0	0	0	0	0	0	0	0	0	0	0	0	1	0	0	0	0	0	0	0
ENSG00000116035	26	34	28	32	10	9	5	5	0	0	0	1	3	3	2	0	1	11	8	29	3	4	1	3	0	0	0	0	1	4	1	0
ENSG00000116039	31	33	37	37	18	22	5	20	0	1	3	0	7	11	3	17	14	7	16	39	4	7	1	5	2	1	0	1	2	4	5	6
ENSG00000258881	144	145	129	146	47	75	50	71	0	1	8	1	20	33	18	37	58	67	68	175	58	38	42	31	6	0	1	3	4	15	13	8
ENSG00000239322	51	50	49	40	18	49	16	31	0	2	1	0	20	16	13	19	53	70	51	71	34	17	24	11	0	1	0	2	21	14	14	25
ENSG00000226186	1	1	0	0	0	0	0	0	0	0	0	0	0	0	0	0	0	0	0	1	0	0	0	0	0	0	0	1	0	0	0	0
ENSG00000241159	0	0	1	1	0	0	0	0	0	0	0	0	0	1	1	0	0	0	0	0	0	0	1	0	0	0	0	0	0	0	0	0
ENSG00000144031	26	16	18	14	8	25	17	19	6	5	25	1	7	8	4	14	3	1	0	12	20	15	10	17	1	2	6	23	3	4	10	18
ENSG00000144043	1931	1472	1871	1456	1582	1527	1380	1679	1426	1185	5379	969	1101	966	1304	1391	957	1438	1465	1845	3293	1323	2516	1159	1387	2215	1154	3581	1221	1512	1363	1668
ENSG00000228384	6	5	2	5	3	11	4	4	3	1	7	4	1	1	3	3	2	4	3	7	7	3	1	12	2	1	2	5	2	2	9	7
ENSG00000236469	0	0	0	0	0	0	0	0	0	0	0	0	0	0	2	1	0	0	0	0	0	0	0	0	0	0	0	0	1	0	0	0
ENSG00000205847	0	0	0	0	0	0	0	0	0	0	0	0	0	0	0	0	0	0	0	0	0	0	2	0	0	0	0	0	0	0	0	0
ENSG00000234485	0	0	0	0	0	0	0	0	0	0	1	0	0	0	0	0	0	0	0	0	0	0	0	0	0	0	2	0	1	0	0	0
ENSG00000234896	0	0	0	0	1	0	0	0	0	0	0	0	0	0	0	0	0	0	0	0	0	0	0	0	0	0	0	0	0	0	0	0
ENSG00000124357	1183	896	1172	960	819	966	690	1051	729	570	3113	539	596	579	770	886	525	739	809	1155	1645	857	1216	763	438	879	776	1891	584	962	760	1140
ENSG00000272735	5	3	12	9	3	0	1	1	1	0	1	0	4	6	1	2	0	1	1	3	6	3	3	0	1	0	1	2	7	1	0	2
ENSG00000124370	307	213	221	199	232	237	176	256	162	146	476	101	309	227	242	300	218	242	166	290	420	219	350	246	129	205	123	296	159	234	209	326
ENSG00000124383	1337	808	1102	770	1423	1376	1188	1588	1108	797	3645	548	1242	1153	1525	1547	1990	2165	1947	1686	4160	1712	2948	1854	1039	1605	855	2468	1934	2038	2491	2410
ENSG00000233870	0	0	0	0	0	0	0	0	1	0	0	0	0	0	0	0	0	0	0	1	0	1	0	0	0	1	0	0	0	0	0	0
ENSG00000124374	913	737	689	770	174	174	118	185	265	200	640	139	265	367	242	513	256	359	261	369	169	64	116	58	150	224	126	397	91	242	85	245
ENSG00000075292	4078	2983	3372	3022	3199	3448	2632	3693	3225	2657	12335	2078	2203	2343	2194	2888	2136	2652	2548	3473	6889	2844	4375	2822	2983	4196	3231	8290	1622	2612	2128	3187
ENSG00000281195	101	80	115	91	86	94	80	113	121	111	606	59	59	52	51	55	26	25	25	108	198	121	84	109	130	140	176	444	16	107	47	116
ENSG00000200779	0	0	0	0	0	0	0	0	0	0	0	0	0	0	0	0	0	0	0	0	0	0	0	0	0	0	0	0	0	0	0	0
ENSG00000135636	55	81	72	76	45	57	30	68	688	493	2086	377	3	8	12	5	11	33	39	83	42	18	36	16	401	602	345	1189	8	4	10	8
ENSG00000233971	0	0	0	0	0	0	0	0	0	0	1	0	0	0	0	0	0	0	0	2	0	0	0	0	0	0	0	0	0	0	0	0
ENSG00000003137	25	95	36	96	0	0	0	2	2	0	0	0	0	1	3	1	1	30	5	57	1	1	1	5	0	1	5	1	0	3	3	2
ENSG00000144036	915	754	809	758	277	292	199	329	371	341	1371	254	460	582	466	721	326	545	459	615	465	216	419	155	375	486	397	992	347	589	484	629
ENSG00000228568	0	0	1	1	0	2	0	1	0	0	1	0	2	0	0	4	0	0	1	1	0	0	0	0	0	3	0	7	0	1	0	0
ENSG00000222536	1	0	0	1	0	0	0	0	0	0	0	0	0	0	0	0	0	0	0	0	0	0	0	0	0	0	2	1	0	0	0	0
ENSG00000212378	0	0	0	1	0	0	0	0	0	0	0	0	1	0	0	0	0	0	0	0	0	0	2	0	0	0	0	0	0	0	1	1
ENSG00000116096	674	573	548	683	0	0	0	1	122	106	277	98	506	519	448	704	224	393	338	537	0	0	0	0	82	185	26	194	395	462	347	473
ENSG00000135638	1	0	0	0	7	7	10	22	4	14	45	18	58	142	43	172	0	0	1	3	10	11	12	18	1	14	7	35	34	60	51	106
ENSG00000278060	1	0	0	0	0	0	1	0	2	1	1	1	1	0	1	4	0	0	0	0	0	0	0	0	0	1	0	3	0	1	4	3
ENSG00000144040	426	409	469	385	657	678	588	798	563	452	3186	469	135	170	151	198	134	176	216	442	1517	724	994	629	544	868	721	2223	154	240	160	267
ENSG00000135631	570	585	445	579	972	918	576	1007	28	19	54	12	246	348	233	449	85	187	120	272	867	379	573	318	9	18	11	43	152	278	135	277
ENSG00000272702	16	17	15	16	14	31	9	20	1	0	4	0	11	11	3	19	5	6	3	14	18	13	19	8	2	1	0	4	5	10	5	9
ENSG00000214513	0	0	0	1	0	0	0	4	0	0	1	0	1	2	0	0	1	0	0	3	0	0	0	3	0	0	1	0	0	0	0	0
ENSG00000135632	1241	846	1104	903	659	678	480	699	914	671	2284	489	1276	1188	1152	1497	386	660	589	938	1065	477	845	431	624	1032	387	1360	916	1078	839	1142
ENSG00000135617	596	394	419	392	357	390	303	402	168	160	636	152	160	158	203	178	385	640	583	600	638	276	560	229	238	341	121	578	287	229	300	254
ENSG00000135624	14972	10453	11679	11219	9032	9325	6421	9511	8006	5341	22287	4481	14842	12900	15326	17312	9901	15918	14553	11126	16200	6235	13460	5413	7164	11427	4179	14447	14834	14753	16844	16853
ENSG00000163013	3602	2907	3290	3016	3514	3490	2440	3584	2246	1900	7767	1405	3024	2585	2911	3188	957	1215	1002	2681	4159	2086	2745	2253	1518	2780	1415	4321	1638	2622	1813	2676
ENSG00000135625	46	29	47	18	0	0	0	0	0	0	4	0	1	5	3	5	5	11	11	17	1	0	0	0	1	0	0	1	0	5	1	0
ENSG00000252988	0	0	0	0	0	0	0	0	0	0	0	0	0	0	0	0	0	0	0	0	0	0	0	0	0	0	0	0	0	0	0	0
ENSG00000214484	0	0	0	0	0	0	0	0	0	0	0	0	0	0	0	0	0	0	0	0	0	0	0	0	0	0	0	0	0	0	0	0
ENSG00000116127	2594	2068	2194	1918	2187	2318	1639	2369	1173	1053	4195	796	1360	1442	1431	1790	880	1236	1118	1873	4468	1749	2967	1553	1365	1955	997	3971	1080	1644	1426	1955
ENSG00000230002	180	123	94	115	105	87	62	85	42	31	134	17	31	27	29	29	38	20	28	47	175	74	68	65	44	61	16	87	11	13	25	22
ENSG00000144035	0	0	0	0	0	4	2	5	0	0	5	0	0	1	1	0	0	0	0	0	6	4	4	4	2	3	0	3	0	4	1	0
ENSG00000163016	0	1	0	0	10	14	8	7	1	4	9	0	5	2	6	5	0	0	0	0	25	7	15	3	1	0	3	8	2	3	0	6
ENSG00000204872	0	0	0	0	1	1	0	2	0	0	0	1	0	0	0	0	0	0	0	0	1	1	0	0	0	0	0	2	0	0	0	0
ENSG00000144034	1396	858	1189	904	840	859	691	938	889	604	2214	431	805	807	1014	1038	1292	1468	1672	1600	2124	923	1760	1174	807	1184	422	1553	1156	1074	1288	1269
ENSG00000273245	18	10	17	4	18	18	7	17	10	7	29	4	16	8	18	15	7	2	2	14	18	12	10	8	9	19	8	17	7	23	17	25
ENSG00000280037	13	9	5	4	14	3	13	14	10	7	28	1	14	7	14	8	3	4	1	9	19	7	4	11	4	6	11	26	2	14	13	15
ENSG00000144048	565	356	541	349	511	507	432	534	518	437	1744	318	751	701	916	851	681	770	822	636	1407	610	1155	629	543	739	413	1300	1120	986	1416	1178
ENSG00000187833	2	0	0	0	0	0	0	0	2	1	7	2	10	10	8	22	0	0	0	1	0	0	0	0	5	1	0	6	1	12	4	2
ENSG00000124356	1533	995	1253	1013	1379	1478	1143	1547	1497	1192	4443	848	2093	1822	1901	2283	1166	1643	1660	1280	3036	1273	2423	1240	866	1467	1053	2611	1334	1478	1578	1741
ENSG00000271536	0	0	0	0	0	2	0	0	0	0	1	0	0	0	0	1	0	0	1	1	0	0	0	0	0	0	0	0	0	0	0	0
ENSG00000163017	548	174	1097	234	111	98	85	98	2884	1681	15596	1511	1337	1104	1694	1449	426	201	1149	323	560	192	401	195	3111	3783	3861	8682	337	419	671	683
ENSG00000114956	1798	1291	1544	1224	908	945	654	1096	1108	748	3194	573	1019	916	1108	1241	1757	2056	1986	1765	2058	830	1480	917	1029	1453	646	2029	985	1086	1235	1358
ENSG00000237883	70	52	95	61	71	99	69	89	45	45	213	19	39	35	41	50	51	66	76	108	151	69	106	73	33	44	43	117	45	65	54	55
ENSG00000235499	62	39	51	61	59	61	58	82	85	54	211	38	52	59	77	67	10	13	25	65	130	59	109	60	46	68	46	135	38	38	43	60
ENSG00000187605	2189	1823	2523	1830	2483	2718	2128	2736	1621	1527	6167	1134	2553	2348	2844	2965	520	686	699	1712	4514	1972	2962	1839	1180	1956	1381	3683	1592	2258	1869	2595

ENSG00000279181	2	2	6	3	5	5	4	13	1	2	13	3	0	2	5	5	3	0	2	8	7	7	5	7	1	4	4	3	3	2	5	9
ENSG00000257800	181	146	193	175	3	2	6	1	165	120	620	101	211	204	242	271	45	33	42	119	7	4	3	6	112	117	133	292	89	183	119	208
ENSG00000217702	10	6	10	5	3	2	1	2	3	3	11	3	2	5	4	6	0	1	0	7	3	1	2	0	0	3	6	8	6	1	7	3
ENSG00000163170	628	435	521	399	277	351	229	329	364	223	1002	210	465	446	576	557	989	1183	1163	968	916	370	653	438	332	523	146	667	656	554	835	749
ENSG00000225439	89	59	71	48	35	44	34	42	39	21	87	27	10	21	22	19	35	46	31	49	55	20	43	31	23	17	16	51	1	3	13	15
ENSG00000114978	7946	4893	6254	4900	6977	7034	4731	7198	7429	6323	27720	4759	6102	5621	6560	7168	3592	4645	4729	4906	13167	5098	8999	4483	8015	11543	5863	19146	5705	6465	5979	7378
ENSG00000279201	0	0	0	0	0	0	0	1	1	0	1	0	0	0	0	0	0	0	0	0	0	0	0	0	0	0	2	3	0	1	2	1
ENSG00000279070	5	2	1	2	1	2	0	2	5	0	6	3	3	0	1	1	0	1	2	4	3	1	1	1	0	2	1	3	0	0	0	0
ENSG00000065911	9843	6558	6779	6540	4358	4648	3454	4691	5490	4059	21833	4007	9230	7583	8471	9909	6367	9389	5660	6612	9004	3460	6584	3390	5818	8378	3692	16368	6479	6483	7949	8059
ENSG00000264324	3	8	8	6	4	15	5	10	5	9	20	3	2	5	6	1	2	1	5	3	16	11	12	10	2	2	8	18	2	4	4	4
ENSG00000188687	48	34	40	28	47	51	39	79	35	49	152	19	69	60	54	97	10	14	5	34	74	46	54	53	31	50	43	135	9	45	41	45
ENSG00000229798	0	0	0	1	0	0	1	0	0	0	2	0	3	0	1	4	0	0	0	0	1	1	1	2	0	0	3	2	0	0	1	0
ENSG00000252214	0	0	0	1	0	0	1	0	0	0	1	2	0	0	0	0	0	0	0	0	1	1	1	0	0	0	0	0	0	0	0	1
ENSG00000234632	1	1	1	3	4	8	6	3	5	2	4	2	0	1	0	2	1	0	0	0	6	5	3	4	2	5	1	8	3	4	1	3
ENSG00000225658	2	0	0	1	6	2	2	1	1	1	4	0	1	1	1	0	0	0	0	1	2	0	0	2	0	2	0	1	0	0	0	3
ENSG00000204843	5164	3677	4615	4233	3155	3270	2305	3424	3554	2780	12332	2309	3335	3277	3600	4346	1406	1953	1860	3285	4631	1986	3537	1638	2616	4874	2741	7615	2337	3217	2553	3422
ENSG00000237737	27	6	15	15	4	16	7	6	7	4	33	2	9	7	19	12	6	3	1	19	14	7	9	12	9	13	9	20	3	8	10	15
ENSG00000159239	127	135	148	118	67	73	50	68	174	142	534	89	260	236	246	307	35	49	52	92	77	28	75	29	63	100	98	191	132	167	157	215
ENSG00000234521	2	0	0	0	1	0	0	1	0	3	2	0	0	0	0	0	0	0	0	0	0	0	0	0	0	1	0	0	0	0	0	1
ENSG00000233155	37	20	19	20	7	5	12	14	25	25	79	8	49	52	50	67	5	10	6	21	9	7	5	4	18	12	19	38	26	30	33	44
ENSG00000005448	1615	1270	1237	1260	971	1069	731	1219	1285	937	2291	632	1191	1105	1213	1432	416	577	520	620	904	407	805	383	573	918	332	1275	728	844	805	1015
ENSG00000114993	32	33	39	36	388	335	227	383	194	143	627	99	18	15	17	24	5	15	15	37	497	208	319	192	104	192	194	354	3	13	12	14
ENSG00000115274	111	82	86	74	71	67	63	65	61	81	350	62	90	82	130	128	52	68	64	104	114	49	74	47	77	91	72	177	100	144	128	156
ENSG00000274049	3	1	5	0	2	3	7	4	3	1	7	1	5	4	9	10	1	2	3	3	3	2	4	1	1	4	1	3	9	5	10	7
ENSG00000239779	830	583	715	714	575	641	475	646	465	325	2047	315	562	522	532	726	339	419	415	487	839	363	638	379	249	478	549	953	310	430	416	540
ENSG00000115275	1927	1535	1591	1548	1470	1646	1166	1592	1365	1236	4874	938	1392	1155	1215	1578	536	581	531	1050	1687	866	1211	862	1093	1968	1070	2780	958	1199	788	1334
ENSG00000204822	222	108	198	137	91	105	86	101	135	116	611	86	99	93	110	121	143	136	143	219	172	72	110	96	135	187	125	337	94	118	116	136
ENSG00000135637	378	243	407	319	221	234	214	249	259	224	1263	243	141	153	193	195	123	151	203	378	412	188	291	214	231	347	333	784	138	282	174	273
ENSG00000115282	954	711	862	747	1275	1392	948	1378	888	740	2861	611	604	575	621	767	417	496	430	667	1709	781	1303	752	644	1057	688	1856	331	586	359	711
ENSG00000179528	80	94	87	82	163	188	147	184	110	81	358	67	42	61	48	55	15	13	10	47	157	81	111	110	45	93	92	208	8	34	18	42
ENSG00000272183	24	21	21	21	29	60	26	38	16	11	61	10	10	15	11	12	3	6	0	7	32	27	18	18	9	25	25	39	3	11	0	6
ENSG00000257702	38	46	36	46	61	86	56	59	65	49	190	55	21	34	20	32	8	3	1	26	86	42	71	43	34	73	51	109	10	13	7	19
ENSG00000115289	391	241	254	276	205	206	162	225	286	239	1063	204	307	270	379	412	344	357	361	364	399	151	233	153	241	390	301	584	343	398	390	438
ENSG00000115297	158	111	182	113	24	32	16	37	4	1	6	0	0	1	0	4	85	132	161	232	37	24	23	12	0	0	0	5	0	3	2	4
ENSG00000144045	74	62	92	58	33	31	28	39	7	4	59	2	6	7	4	14	29	25	49	93	46	22	25	29	5	5	2	12	5	8	3	11
ENSG00000115307	1886	1431	1620	1423	2325	2293	1705	2509	1903	1415	6321	1187	1935	1603	2264	2308	1055	1452	1350	1740	4009	1661	3147	1626	1542	2385	1360	3787	1905	2162	2134	2401
ENSG00000115317	1469	1018	1241	1067	829	865	613	855	704	568	2596	563	866	711	908	926	953	1466	1354	1400	1411	616	1162	512	646	1044	642	1714	841	786	916	888
ENSG00000115318	67	63	76	67	198	253	173	254	47	51	240	23	40	36	39	53	23	29	30	53	319	147	168	156	41	57	53	128	22	34	33	40
ENSG00000115325	606	470	588	453	896	956	615	894	608	560	3084	523	275	324	354	412	234	390	368	552	1383	530	1162	482	547	939	786	1613	265	339	271	385
ENSG00000159374	12	6	11	8	22	24	15	31	5	8	38	5	2	1	7	2	2	7	6	8	45	16	14	24	9	11	13	30	3	3	3	3
ENSG00000233872	0	0	0	0	0	0	0	0	0	0	0	0	0	0	0	0	0	0	0	0	0	0	0	0	0	0	0	1	0	0	0	0
ENSG00000108442	0	0	0	0	0	0	0	0	0	0	0	0	0	0	0	0	0	0	0	0	0	0	0	0	0	0	0	0	0	0	0	0
ENSG00000135622	465	427	386	408	285	367	176	379	236	200	914	181	224	297	193	369	139	168	131	328	274	129	191	108	166	267	231	541	141	240	98	233
ENSG00000224646	0	0	0	0	0	4	0	1	0	0	0	0	0	0	0	0	0	0	0	0	1	0	1	1	0	0	0	0	0	0	0	0
ENSG00000272711	140	116	167	127	144	144	86	130	130	82	427	63	134	192	225	250	50	112	95	155	236	82	153	76	130	175	61	261	185	196	194	251
ENSG00000159399	4419	3841	3848	3776	5668	5355	4153	5934	3913	3142	7593	2024	6083	5612	5530	7013	605	942	762	1545	4844	2230	3633	1841	2212	3788	1146	4632	2518	3363	2630	3604
ENSG00000204792	0	0	0	0	0	0	0	1	236	208	693	158	258	241	246	320	0	0	0	0	0	0	0	0	216	311	180	552	157	241	193	250
ENSG00000236209	0	0	0	0	0	0	0	0	8	3	32	6	16	8	13	20	0	0	0	0	0	0	0	0	7	12	6	12	4	6	1	4
ENSG00000230836	0	0	0	0	0	0	0	0	16	8	50	4	19	13	7	13	0	0	0	0	0	0	1	0	12	19	7	38	1	11	5	14
ENSG00000115350	227	164	250	176	380	401	265	406	328	225	784	165	464	442	510	582	296	349	307	266	885	361	634	366	278	412	175	625	608	456	658	639
ENSG00000115353	9	3	7	3	9	14	18	18	7	7	24	6	3	5	4	13	0	0	1	11	34	21	20	24	7	8	4	23	3	20	18	22
ENSG00000263909	0	0	0	0	0	0	0	0	3	0	1	0	0	1	0	0	0	0	0	0	4	1	0	2	0	0	1	1	0	3	1	2
ENSG00000270571	3	0	5	6	10	11	5	8	6	3	14	4	12	13	11	17	2	2	5	5	22	11	9	17	5	5	6	7	6	6	5	11
ENSG00000236167	0	0	0	0	0	0	0	0	0	0	1	0	0	0	0	0	0	0	0	0	0	0	0	0	0	0	0	0	0	0	0	0
ENSG00000115363	0	0	1	0	1	4	3	3	0	0	1	0	1	1	0	0	1	0	1	0	2	1	0	0	0	2	2	3	0	1	0	0
ENSG00000237293	1	0	1	1	0	2	0	4	1	0	0	0	0	0	0	0	0	0	0	1	0	0	2	0	0	0	0	0	0	0	0	0
ENSG00000238410	0	0	0	0	0	0	0	1	0	0	0	0	0	0	0	0	0	0	0	0	0	0	1	0	0	0	0	0	0	0	0	0
ENSG00000231172	0	0	0	0	0	0	0	0	0	0	0	0	0	0	0	0	0	0	0	0	0	0	0	0	0	0	0	0	0	0	0	0
ENSG00000230477	0	0	0	0	0	0	0	0	0	0	0	0	0	0	0	0	0	0	0	0	0	0	0	0	0	0	0	0	0	0	0	0
ENSG00000221638	0	0	0	0	0	0	0	0	0	0	0	0	0	0	0	0	0	0	0	0	0	0	0	0	0	0	0	0	0	0	0	0
ENSG00000115364	1377	971	1032	865	1302	1285	948	1282	910	689	3073	580	1789	1542	1751	1806	1085	1417	1281	1397	3135	1153	2256	1094	1048	1376	696	2220	2024	2091	2221	2215
ENSG00000226247	1	3	0	1	0	1	1	3	0	0	1	0	0	1	0	4	0	0	0	2	6	2	2	0	1	3	0	1	0	1	2	1
ENSG00000005436	1367	986	989	928	901	936	664	997	652	551	2358	381	1034	1052	1004	1228	1023	1517	1140	1435	1814	730	1179	808	568	760	623	1482	934	1058	999	1180
ENSG00000270696	292	226	235	182	161	146	140	162	153	121	492	94	357	298	355	350	120	169	109	240	300	127	166	111	178	211	141	346	240	342	249	362
ENSG00000271452	35	31	24	22	11	14	7	15	23	18	71	10	29	27	27	39	9	11	20	44	32	18	19	10	23	22	20	61	27	47	24	35
ENSG00000270462	8	8	1	10	5	10	1	11	5	2	19	0	8	10	6	7	1	0	1	11	9	4	9	5	5	5	3	12	2	2	1	6
ENSG00000270996	5	8	6	2	0	1	0	2	0	1	12	0	4	2	1	6	1	1	1	1	2	0	1	0	0	0	0	0	1	2	5	1
ENSG00000233107	0	0	0	0	0	0	0	0	0	0	0	0	0	0	0	0	0	0	0	0	0	0	0	0	0	0	0	0	0	0	0	0
ENSG00000228209	0	0	0	0	0	0	0	0	0	0	0	0	0	0	0	0	0	0	0	0	0	0	0	0	0	0	0	0	0	0	0	0
ENSG00000271356	0	0	0	0	0	0	0	0	0	0	0	0	0	0	0	0	0	0	0	0	0	0	0	0	0	0	0	0	0	0	1	0
ENSG00000200488	0	0	0	0	2	0	0	0	0	0	1	0	0	0	0	0	0	0	0	0	0	0	0	0	1	0	0	0	0	0	0	0
ENSG00000251947	0	0	0	0	0	0	0	0	0	0	0	0	0	0	0	0	0	0	0	0	0	0	0	0	0	0	0	0	0	0	0	0
ENSG00000176204	0	0	0	0	0	0	0	0	0	1	10	0	0	0	0	0	0	0	1	1	0	0	1	0	1	0	7	7	0	0	0	0
ENSG00000234653	0	0	0	0	0	0	0	0	0	0	0	0	0	0	0	0	0	0	0	0	0	0	0	0	0	0	0	0	0	0	0	0
ENSG00000227088	0	0	0	0	0	0	0	0	0	0	0	0	0	0	0	0	0	0	0	0	0	0	0	0	0	0	0	0	0	0	0	0
ENSG00000230968	0	0	0	0	2	0	1	0	0	0	0	0	0	0	0	0	0	0	0	0	1	0	0	0	0	0	0	0	0	0	0	0
ENSG00000237077	0	0	0	0	0	0	0	0	0	0	0	0	0	0	0	0	0	1	0	0	0	0	0	0	0	0	0	2	0	0	0	0
ENSG00000233605	0	0	0	0	0	0	0	0	0	0	0	0	0	0	0	0	0	0	0	0	0	0	0	0	0	0	0	0	0	0	0	0
ENSG00000229494	0	0	0	0	0	0	0	0	0	0	3	0	0	0	0	1	0	0	0	0	0	0	0	0	0	0	0	0	0	0	0	2
ENSG00000214429	1	0	0	0	0	0	0	0	0	0	0	0	4	3	0	0	0	0	0	0	0	0	0	0	0	0	0	0	0	1	0	3
ENSG00000270470	0	0	0	0	0	0	0	0	0	0	0	0	0	0	0	0	0	0	0	0	0	0	0	0	0	0	0	0	0	0	0	0
ENSG00000234877	0	0	0	0	0	0	0	0	0	0	0	0	0	0	0	0	0	0	0	0	0	0	0	0	0	0	0	0	0	0	0	0
ENSG00000202423	0	0	0	0	0	0	0	0	0	0	0	0	0	0	0	0	0	0	0	0	0	0	0	0	0	0	0	0	0	0	0	0
ENSG00000200048	0	0	0	0	0	0	0	0	0	0	0	0	0	0	0	0	0	0	0	0	0	0	0	0	0	0	0	0	0	0	0	0
ENSG00000143954	0	0	0	0	0	0	0	0	0	0	1	0	0	0	0	0	0	1	0	0	0	0	0	0	0	0	0	0	0	0	0	0
ENSG00000172023	0	0	0	0	0	0	0	0	0	0	0	0	0	0	0	0	0	0	0	0	0	0	0	0	0	0	0	0	0	0	0	0
ENSG00000115386	0	1	0	0	0	0	0	0	0	0	0	0	0	0	0	0	0	0	0	0	0	0	0	0	0	0	0	1	0	0	1	0
ENSG00000204787	0	0	0	0	0	0	0	0	0	0	0	0	0	0	0	0	0	0	0	0	0	0	0	0	0	0	0	0	0	0	0	0
ENSG00000172016	0	0	0	0	0	0	0	0	0	0	0	0	0	0	0	0	0	0	0	0	0	0	0	0	0	0	0	0	0	0	0	0
ENSG00000224879	0	0	0	0	0	0	0	0	0	0	0	0	0	0	0	0	0	0	0	0	0	0	0	0	0	0	0	0	0	0	0	0
ENSG00000066032	2	2	9	4	1	1	3	1	0	0	0	0	3	2	3	2	1	4	5	1	0	1	0	0	0	0	0	0	1	1	0	3
ENSG00000229385	0	0	0	0	0	0	0	0	0	0	0	0	0	0	0	0	0	0	0	0	0	0	0	0	0	0	0	0	0	0	0	0
ENSG00000277171	1	0	0	0	0	0	0	0	0	0	0	0	0	0	0	0	0	0	0	0	0	0	0	0	0	0	0	0	0	0	0	0
ENSG00000230237	0	0	0	0	0	0	0	0	0	0	0	0	0	0	0	0	0	0	0	0	0	0	0	0	0	0	0	0	0	0	0	0
ENSG00000252031	0	0	0	0	0	0	0	0	0	0	0	0	0	0	0	0	0	0	0	0	0	0	0	0	0	0	0	0	0	0	0	0
ENSG00000266436	0	0	0	0	0	0	0	0	0	0	0	0	0	0	0	0	0	0	0	0	0	0	0	0	0	0	0	0	0	0	0	0
ENSG00000276917	0	1	0	0	0	0	0	0	0	0	0	0	0	0	0	0	0	0	0	0	0	0	0	0	0	0	0	0	0	0	0	0
ENSG00000224731	1	0	0	0	0	0	0	0	0	0	0	0	2	1	3	4	0	0	0	0	0	0	0	0	0	0	0	0	5	1	1	0
ENSG00000223427	0	0	0	0	0	0	0	2	0	0	1	1	0	0	0	3	0	0	0	0	1	1	0	0	0	0	0	0	0	1	0	0
ENSG00000162951	0	0	0	0	0	0	0	0	0	0	0	0	0	0	0	1	0	0	0	0	0	0	0	0	0	0	1	1	0	0	0	0
ENSG00000237031	0	1	0	0	0	0	0	0	0	0	1	0	0	0	1	0	0	0	1	0	0	0	0	0	0	0	0	0	0	0	0	0
ENSG00000230975	0	0	0	0	0	0	0	0	0	0	0	0	0	0	0	0	0	0	0	0	0	0	0	0	0	0	0	0	0	0	0	0
ENSG00000234429	0	0	0	0	0	0	0	0	0	1	0	0	0	0	0	0	0	0	0	0	0	0	0	0	0	0	0	0	0	0	0	0
ENSG00000231722	0	0	0	0	0	0	0	0	0	0	0	0	0	0	0	0	0	0	0	0	0	0	0	0	0	0	0	0	0	0	0	0
ENSG00000235519	0	0	0	0	0	0	0	0	0	0	0	0	0	0	0	0	0	0	0	0	0	0	0	0	0	0	0	0	0	0	0	0
ENSG00000233444	0	0	0	0	0	0	0	0	0	0	0	0	0	0	0	0	0	0	0	0	0	0	0	0	0	0	0	0	0	0	0	0
ENSG00000242118	0	0	0	0	0	0	0	0	0	0	0	0	0	0	0	0	0	0	0	0	0	0	0	0	0	0	0	0	0	0	0	0
ENSG00000231781	0	0	0	0	0	0	0	0	0	0	0	0	0	0	0	0	0	0	0	0	0	0	0	0	0	0	1	0	0	0	0	0
ENSG00000229560	0	0	0	0	0	0	0	0	0	0	0	0	0	0	0	1	0	0	0	0	0	0	0	0	0	0	0	0	0	0	0	0
ENSG00000252897	0	0	0	0	0	0	0	0	0	0	0	0	0	0	0	0	0	0	0	0	0	0	0	0	0	0	0	0	0	0	0	0
ENSG00000201311	0	0	0	0	0	0	0	0	0	0	0	0	0	0	0	0	0	0	0	0	0	0	0	0	0	0	0	0	0	1	0	0
ENSG00000237498	0	0	0	0	0	0	0	0	0	0	0	0	0	0	0	0	0	0	0	0	0	0	0	0	0	0	0	0	0	0	1	0
ENSG00000226860	0	0	0	0	0	0	0	0	0	0	0	0	0	0	0	0	0	0	0	0	0	0	0	0	0	0	0	0	0	0	0	0
ENSG00000226700	0	0	0	0	0	0	0	0	0	0	0	0	0	0	0	0	0	0	0	0	0	0	0	0	0	0	0	0	0	0	0	0
ENSG00000225045	0	0	0	0	0	0	0	0	0	0	0	0	0	0	0	0	0	0	0	0	0	0	0	0	0	0	0	0	0	0	0	0
ENSG00000224695	0	0	0	0	0	0	0	0	0	0	0	0	0	0	0	0	0	0	0	0	0	0	0	0	0	0	0	0	0	0	0	0
ENSG00000227789	0	0	0	0	0	0	0	0	0	0	0	0	0	0	0	0	0	0	0	0	0	0	0	0	0	0	0	0	0	0	0	0
ENSG00000168129	0	0	0	0	0	0	0	0	0	0	0	0	0	0	0	0	0	0	0	0	0	0	0	0	0	0	0	0	0	0	0	0
ENSG00000223977	0	0	0	0	0	0	0	0	0	0	0	0	0	0	0	0	0	0	0	0	0	0	0	0	0	0	0	0	0	0	0	0
ENSG00000232548	0	0	0	0	0	0	0	0	0	0	0	0	0	0	0	0	0	0	0	0	0	0	0	1	0	0	0	0	0	0	0	0
ENSG00000224627	0	0	0	0	0	0	0	0	0	0	0	0	0	0	0	0	0	0	0	0	0	0	0	0	0	0	0	0	0	0	0	0
ENSG00000199295	0	0	0	0	0	0	0	0	0	0	0	0	0	0	0	0	0	0	0	0	0	0	0	0	0	0	0	0	0	0	0	0
ENSG00000228225	0	0	0	0	0	0	0	0	0	0	0	0	0	0	0	0	0	0	0	0	0	0	0	0	0	0	0	0	0	0	0	0
ENSG00000228902	0	0	0	0	0	0	0	0	0	0	0	0	0	0	0	0	0	0	0	0	0	0	0	0	0	0	0	0	0	0	0	0
ENSG00000182814	0	0	1	0	0	0	0	0	0	0	0	0	0	0	0	0	0	0	2	0	0	0	0	0	0	0	0	0	0	0	0	0
ENSG00000228272	0	0	0	0	0	0	0	0	0	0	0	0	0	0	0	0	0	0	0	0	0	0	0	0	0	0	1	0	0	0	0	0
ENSG00000163541	1839	1334	1440	1295	1249	1369	954	1368	1365	1000	4359	796	1801	1623	1873	2322	2257	3003	2473	2163	3381	1291	2377	1477	1334	1905	902	2938	2504	2407	3082	2991
ENSG00000115423	153	81	95	77	4	10	5	13	13	8	21	4	92	123	96	138	22	34	23	60	7	6	5	2	5	12	7	19	34	59	38	80
ENSG00000259056	2	1	1	1	0	0	1	0	0	1	0	0	2	3	2	2	0	3	0	0	0	0	0	0	0	0	0	0	0	1	0	0
ENSG00000235847	1	0	1	0	2	0	0	0	1	1	2	0	1	2	0	6	1	0	1	1	2	0	0	0	2	2	0	0	0	0	0	1
ENSG00000186854	5	19	10	10	25	17	16	23	40	43	45	19	264	325	332	412	7	10	9	6	38	17	22	18	22	51	14	64	173	279	227	374
ENSG00000276362	0	0	0	0	0	1	2	0	0	0	2	0	0	4	0	2	0	0	0	0	0	1	0	0	0	2	0	0	0	2	1	2
ENSG00000213400	0	0	0	0	0	0	0	0	1	0	0	0	0	1	5	0	0	0	2	0	0	0	0	0	0	1	0	0	1	0	0	0
ENSG00000279992	0	0	2	1	1	0	1	2	0	0	0	0	0	0	0	0	0	0	0	0	2	0	0	0	0	0	0	0	0	0	0	0
ENSG00000034510	10786	8209	9939	9243	11018	10541	6886	11528	13576	9729	31915	7486	16402	16840	16202	22946	4231	6039	6067	5162	15519	5300	10856	4854	8382	13520	5556	18045	14617	17911	15922	22262
ENSG00000213399	4	1	1	0	3	0	1	3	3	0	11	1	3	0	4	2	1	2	0	5	2	1	0	0	2	3	0	4	1	7	0	2
ENSG00000176407	1506	1048	1160	954	1147	1044	856	1116	921	810	3629	652	1251	1125	1336	1435	1117	1537	1552	1330	2561	975	1813	878	1017	1579	836	2371	1450	1553	1623	1674
ENSG00000260840	0	0	0	0	0	0	0	0	0	0	1	0	0	0	0	0	0	0	0	0	1	0	0	1	0	2	1	0	0	0	0	0
ENSG00000235463	0	0	0	0	0	0	0	0	0	0	0	0	0	0	0	0	0	0	0	0	0	0	0	1	0	0	0	0	0	0	0	0
ENSG00000225787	0	0	0	0	0	0	0	0	0	0	0	0	0	0	0	0	0	0	0	0	0	0	0	0	0	0	0	0	0	0	0	0
ENSG00000152284	67	56	73	64	67	79	28	63	25	20	62	9	31	22	26	32	7	4	9	33	36	20	16	20	22	17	24	43	4	11	7	24
ENSG00000231134	0	0	0	0	0	0	0	0	0	0	0	0	0	0	0	0	0	0	0	0	0	0	0	0	0	0	0	0	1	0	0	0
ENSG00000200701	0	0	0	0	0	0	0	0	0	0	0	0	0	0	0	0	0	0	0	0	0	0	0	0	0	0	0	0	0	0	0	0
ENSG00000230299	0	0	0	0	0	0	0	0	0	0	0	1	0	0	0	0	0	0	0	0	0	0	0	0	0	0	0	0	0	1	0	0
ENSG00000246575	115	111	122	108	65	86	73	92	55	55	282	35	67	57	36	64	17	21	9	56	75	42	41	38	33	47	56	126	16	51	18	49
ENSG00000152291	4583	3412	4408	3536	5245	5300	4226	5684	4676	4172	19016	3008	4883	4211	5011	5536	1465	1513	1933	3141	8779	3591	5984	2889	3752	5929	4475	11363	3580	4803	4189	5353
ENSG00000239143	0	2	0	0	1	0	0	0	0	0	1	1	0	0	1	0	0	0	0	1	2	0	0	4	0	0	2	0	2	1	0	1
ENSG00000270532	3	0	2	0	4	1	2	0	0	0	4	0	0	2	2	0	0	1	2	1	2	5	0	2	1	0	0	4	0	0	2	4
ENSG00000042445	988	743	814	742	599	605	441	692	676	593	3648	566	875	832	811	1091	372	403	389	733	930	425	702	391	585	930	982	2060	614	755	648	973
ENSG00000115459	609	447	593	436	416	454	353	531	345	281	1688	263	250	236	285	303	303	317	347	461	624	297	452	341	252	465	454	1002	170	321	211	340
ENSG00000271014	2	7	1	1	1	2	2	0	1	1	3	0	5	3	0	1	0	1	0	0	1	0	2	3	1	0	3	4	0	0	0	3
ENSG00000202382	9	2	2	3	0	4	1	1	2	5	19	3	5	4	1	3	4	3	0	4	7	6	4	7	2	5	3	12	0	7	3	2
ENSG00000278590	10	3	6	7	1	3	6	5	5	3	17	7	4	5	6	2	3	1	4	11	4	5	4	5	4	2	3	6	4	5	2	12
ENSG00000273196	9	5	9	4	11	6	1	6	2	0	21	1	1	0	0	5	4	1	3	9	5	2	3	4	6	6	7	12	4	1	4	1
ENSG00000042493	2921	1828	2669	1813	4903	5165	3361	5378	1189	1073	7206	1234	1864	1720	1962	2345	1668	2555	2837	2085	8838	3885	7371	2905	1061	2051	1608	5328	2156	2208	2599	2546
ENSG00000152292	57	51	76	42	15	8	9	17	13	11	44	4	10	15	13	21	33	16	28	62	17	10	5	6	5	15	7	25	1	4	6	8
ENSG00000207207	0	1	3	4	0	0	0	0	0	0	2	0	0	0	0	0	1	0	1	0	0	0	2	0	0	0	1	0	0	0	0	0
ENSG00000244399	0	0	0	0	0	0	0	1	0	0	0	0	0	0	0	0	0	0	0	0	0	0	0	0	0	0	0	0	0	0	0	0
ENSG00000199936	0	0	0	0	0	0	0	0	0	0	0	0	0	0	0	0	0	0	0	0	0	0	0	0	0	0	0	0	0	0	0	0
ENSG00000229648	0	0	0	0	0	0	0	0	0	0	0	0	0	0	0	0	0	0	0	0	2	0	0	0	0	0	0	0	0	0	0	0
ENSG00000168906	12882	9321	12050	9398	10619	10840	8551	11328	12007	9714	40037	7697	12660	11020	12313	14385	8071	8273	9077	13785	22332	9283	13753	9487	11048	16427	6874	24581	8268	13067	9377	14579
ENSG00000115486	726	555	674	587	431	415	301	529	383	312	1343	281	390	363	464	509	263	328	304	597	581	283	358	243	327	465	357	928	276	476	411	534
ENSG00000118640	9	11	5	3	1690	1558	1215	1635	974	688	3600	613	1903	1618	1953	2197	1	8	12	14	3543	1321	2584	1178	923	1199	696	1886	2253	1830	2477	2351
ENSG00000276281	0	0	0	1	0	0	0	0	0	0	0	0	0	0	0	1	0	0	0	0	0	0	0	0	0	0	0	0	0	0	0	0
ENSG00000281331	0	0	0	1	0	0	0	0	0	0	3	0	0	0	0	0	0	0	2	0	0	0	0	0	0	0	0	0	0	0	0	0
ENSG00000168899	643	439	622	457	504	508	354	487	23	12	68	10	56	47	28	41	726	750	815	619	956	353	734	337	13	40	15	57	40	38	33	55
ENSG00000168894	878	603	783	616	578	622	443	687	501	442	1428	333	715	683	798	831	977	1002	1112	788	1211	547	939	603	372	662	337	974	823	744	1025	1060
ENSG00000168890	41	36	35	25	30	28	33	35	36	28	162	27	71	65	114	101	16	15	11	32	77	35	52	45	21	27	53	88	149	173	139	156
ENSG00000168883	2904	1998	2526	1992	2098	2037	1586	2224	1433	1006	4549	870	2739	2467	2915	3232	1511	2388	2351	2111	4211	1539	3277	1506	1669	2348	979	3335	2801	2974	2950	3162
ENSG00000168887	1051	831	1005	824	859	934	698	908	804	699	2846	505	1018	1086	1044	1329	500	609	515	651	1119	516	882	449	598	877	666	1726	606	886	742	1080
ENSG00000168878	13	17	16	8	4	8	14	8	6	4	18	3	4	11	9	12	3	5	4	6	13	11	10	10	10	13	4	14	1	6	2	8
ENSG00000115523	19	6	8	12	18	17	7	21	1671	2234	1286	1463	111	120	112	140	5	3	6	7	3	6	6	8	12	16	4	42	136	170	116	230
ENSG00000203363	1	0	1	1	0	1	0	0	0	1	0	0	0	1	3	2	0	0	0	0	2	0	0	0	0	0	0	0	0	2	1	4
ENSG00000199687	0	0	0	0	0	0	0	0	0	0	0	0	0	0	0	0	0	0	0	0	0	0	0	0	0	0	0	0	0	0	0	0
ENSG00000168874	0	1	0	0	38	52	26	54	1	2	8	0	0	1	2	2	0	0	0	0	93	33	77	37	2	3	0	8	0	0	0	3
ENSG00000278783	0	0	0	0	0	0	0	0	0	0	0	0	0	0	0	0	0	0	0	0	0	0	0	0	0	0	0	0	0	0	0	0
ENSG00000229498	2	0	0	0	0	0	0	0	0	1	2	0	0	1	0	1	0	1	0	0	0	0	1	0	0	0	3	2	0	0	0	0
ENSG00000252321	0	0	0	0	0	0	0	0	0	0	0	0	0	0	0	0	0	0	0	0	0	0	0	0	0	0	0	0	0	0	0	0
ENSG00000115525	668	438	538	471	470	496	357	536	517	491	2232	331	271	251	264	368	260	401	339	392	716	315	579	290	465	655	746	1288	184	270	193	351
ENSG00000232504	14	2	10	7	1	6	2	3	7	1	21	8	3	2	3	1	1	2	8	1	6	2	6	3	1	1	16	9	1	0	6	3
ENSG00000272564	1	1	0	3	0	2	0	0	0	0	5	0	0	1	0	1	1	3	0	0	0	0	1	0	0	0	1	1	0	0	1	0
ENSG00000068654	3073	2238	3042	2413	3890	3784	2847	3877	3091	2455	11119	1842	4111	3646	4398	4641	758	1104	1020	2417	5907	2785	4328	2383	2713	4432	2143	7158	2773	3986	3037	4205
ENSG00000132300	3884	2688	2965	2255	2966	2939	2397	3095	2329	1981	7882	1388	3776	3007	3705	3989	2693	2786	2560	2839	6617	2611	4427	2788	2110	3147	1712	5094	2906	3334	3573	3998
ENSG00000208772	35	11	22	12	25	25	24	30	17	21	66	14	28	26	30	27	12	2	10	20	63	30	32	38	14	13	11	44	13	30	20	24
ENSG00000132305	3528	2716	2911	2553	3147	2993	2202	3193	2415	1736	7180	1329	4093	3467	3796	4515	2455	3717	2838	2504	5972	2192	4271	2051	2276	3254	1448	5082	3564	3729	4092	4164
ENSG00000200241	6	1	2	0	2	3	1	1	1	1	2	1	1	1	0	1	0	0	0	1	2	0	1	2	2	1	2	7	0	1	1	4
ENSG00000265420	0	0	0	0	0	1	1	0	0	3	2	0	0	0	0	3	0	0	0	0	1	0	0	0	0	1	0	6	0	1	0	2
ENSG00000273080	38	21	40	30	6	4	7	6	19	23	102	18	23	13	22	26	25	33	31	21	12	5	12	14	19	28	16	51	14	33	19	28
ENSG00000132313	1273	878	1109	830	1070	988	769	1031	1017	738	3289	560	1515	1304	1711	1684	1184	1500	1320	1586	2573	952	1794	1190	1114	1573	723	2275	1885	1819	2195	2278
ENSG00000068615	16	72	21	57	0	3	0	2	10	12	13	7	37	33	14	41	10	66	22	70	5	4	1	1	9	11	3	22	1	23	5	17
ENSG00000202537	0	0	0	0	0	0	0	0	0	0	0	0	0	0	0	0	0	0	0	0	0	0	0	0	0	0	0	0	0	0	0	0
ENSG00000251974	0	0	0	0	0	0	0	0	0	0	0	0	0	0	0	0	0	0	0	0	0	0	0	0	0	0	0	0	0	0	0	0
ENSG00000115548	4061	3130	3589	2711	2931	2804	2144	3255	2502	2145	7207	1445	2955	2930	2659	3495	1109	1275	1346	1530	3762	1586	2502	1464	1569	2046	1813	4355	1186	1752	1396	2005
ENSG00000115561	833	585	741	548	752	757	612	841	854	660	3515	565	744	795	792	1068	539	868	685	746	1805	701	1343	661	887	1283	827	2316	754	945	840	1040
ENSG00000249884	17	12	12	5	8	16	19	24	6	3	23	4	8	9	18	13	14	6	8	22	35	11	20	15	9	13	11	18	4	11	15	12
ENSG00000200563	0	0	2	1	0	2	0	0	3	4	6	0	0	0	1	1	0	0	0	3	0	0	0	0	1	2	3	9	0	1	1	1
ENSG00000228363	64	32	48	35	40	54	42	55	39	30	171	30	54	43	39	64	5	7	5	29	67	45	42	45	22	52	38	99	9	33	23	43
ENSG00000239305	804	631	734	610	514	609	457	630	380	312	1873	232	572	551	516	696	231	323	255	421	1140	437	733	463	370	495	580	1113	253	486	296	562
ENSG00000153561	4890	3511	4059	3576	3819	3927	2848	3929	2628	2152	11407	1831	6593	5863	6607	6850	2495	4078	3666	3650	7938	3216	5833	2628	3193	4274	3005	7817	5393	6525	6728	7191
ENSG00000153563	28	33	32	47	10	18	11	26	7130	5943	32048	5439	22	33	35	36	3	19	10	35	22	11	14	10	7660	11472	9118	23722	18	24	9	25
ENSG00000172116	4	2	1	1	3	0	0	1	276	234	1362	260	1	1	0	0	0	4	3	2	0	0	0	1	234	326	334	902	0	3	1	0
ENSG00000233673	1	4	3	7	5	1	1	1	14	7	23	6	6	1	3	0	1	1	3	3	3	2	4	1	3	12	7	17	0	1	2	1
ENSG00000213605	0	0	0	0	0	0	0	0	0	0	2	0	0	0	0	0	0	0	0	0	0	0	0	0	0	0	0	1	0	0	0	0
ENSG00000187627	12	4	8	6	7	2	6	8	9	8	39	1	6	11	9	11	9	11	12	15	12	3	4	6	7	17	8	13	12	8	10	14
ENSG00000231351	0	0	0	1	0	2	0	0	0	0	0	0	0	0	0	0	0	0	0	0	0	0	0	0	0	0	0	0	0	0	0	0
ENSG00000226114	0	0	0	0	0	0	0	0	1	0	1	0	0	0	0	0	0	0	0	0	0	0	0	0	0	0	0	0	0	0	0	0
ENSG00000183281	6	2	1	4	23	39	26	37	44	42	280	26	32	31	29	46	1	3	3	4	42	22	27	26	44	57	66	127	12	37	12	43
ENSG00000231259	220	201	191	195	218	236	180	289	181	144	694	107	368	305	290	395	128	199	121	127	500	198	330	192	145	211	177	457	219	264	260	270
ENSG00000266931	26	20	30	33	11	25	16	17	13	16	43	5	42	24	25	28	13	20	20	7	42	28	20	11	14	13	10	33	13	21	37	16
ENSG00000230395	0	1	0	0	0	0	0	2	0	0	2	0	0	0	0	0	0	0	0	2	4	0	2	0	0	3	0	0	0	0	0	0
ENSG00000204745	94	57	93	69	83	88	73	94	95	67	206	45	93	71	93	106	40	78	67	91	182	75	149	81	66	95	44	180	53	103	67	76
ENSG00000264793	0	0	0	0	0	0	0	0	0	0	0	0	0	0	0	0	0	0	0	0	0	0	0	0	0	0	0	0	0	0	0	0
ENSG00000233671	0	0	0	0	0	0	0	0	0	0	3	0	0	0	0	0	0	0	0	0	1	0	0	0	1	0	1	1	1	0	0	0
ENSG00000281477	0	0	0	0	0	0	0	0	0	0	0	0	0	0	0	0	0	0	0	0	0	0	0	1	0	0	0	0	0	0	0	0
ENSG00000271097	0	0	0	0	0	0	0	0	0	0	0	0	0	0	0	0	0	0	0	0	0	0	0	0	0	0	0	1	0	0	0	0
ENSG00000233999	0	0	0	0	0	0	0	1	0	0	0	0	0	0	0	0	0	0	0	0	2	0	0	0	0	0	0	0	0	0	0	0
ENSG00000224881	0	0	1	0	0	1	0	0	0	0	0	0	0	0	0	0	0	0	0	0	0	1	0	0	0	0	0	0	0	0	0	0
ENSG00000280721	7	3	2	2	2	2	3	8	18	28	70	19	4	7	15	8	4	3	0	8	22	12	2	6	23	40	16	83	7	13	13	17
ENSG00000222041	96	106	118	102	183	196	146	251	692	508	1873	365	680	587	731	743	213	300	214	255	549	192	423	288	551	788	379	1261	902	832	1214	1151
ENSG00000273445	1	5	8	0	1	6	3	3	22	18	44	4	13	8	18	10	0	3	0	3	4	3	1	6	14	19	9	48	9	22	12	38
ENSG00000214354	0	1	0	0	0	1	0	0	4	2	7	1	1	1	1	1	0	1	0	2	2	0	0	1	5	8	3	6	0	1	4	3
ENSG00000265507	0	0	0	0	1	1	0	4	2	2	3	3	3	1	0	0	0	0	0	0	0	2	1	1	7	8	2	13	0	2	1	0
ENSG00000225933	0	0	0	1	1	2	0	0	4	3	25	1	0	2	1	1	0	0	0	1	2	2	1	0	3	4	5	11	0	1	0	1
ENSG00000234231	121	78	71	86	18	16	16	19	136	113	342	90	38	33	24	39	61	102	66	98	36	23	39	20	100	174	84	263	25	25	29	48
ENSG00000125551	10	17	8	8	8	8	9	11	20	24	102	9	2	7	3	2	4	8	1	9	13	11	8	8	13	14	29	62	0	3	1	2
ENSG00000185304	9	5	15	2	43	37	34	35	10	13	87	8	57	58	47	64	21	29	16	22	32	29	34	24	17	19	28	52	36	59	52	76
ENSG00000229604	0	0	0	0	0	1	0	0	1	0	0	0	2	0	1	0	0	0	0	0	2	2	0	1	0	0	0	0	1	3	0	2
ENSG00000225428	0	0	0	0	0	0	0	0	0	0	0	0	0	0	0	0	0	1	0	0	0	0	0	0	0	0	0	0	0	0	0	2
ENSG00000233205	0	0	2	1	0	3	0	0	0	2	0	0	0	1	0	1	0	0	2	1	1	0	1	0	0	0	1	0	0	0	0	1
ENSG00000275597	0	0	1	0	0	0	0	0	0	0	1	0	0	0	0	0	0	0	1	1	0	1	0	0	0	0	0	0	0	0	0	0
ENSG00000222724	0	0	0	1	0	0	0	0	0	0	2	0	0	1	0	0	0	0	0	0	0	0	0	0	3	0	0	5	0	1	0	3
ENSG00000172086	821	525	631	477	97	111	71	140	597	485	2396	358	541	606	601	787	984	1223	920	1028	277	133	214	162	576	811	716	1685	629	665	757	885
ENSG00000115593	0	0	0	0	0	0	0	0	1	2	0	0	0	0	0	0	0	1	1	0	2	0	1	0	0	0	0	0	0	0	0	0
ENSG00000265003	0	0	0	0	0	0	0	0	0	0	0	0	0	0	0	0	0	0	0	0	0	0	0	0	0	0	0	0	0	0	0	0
ENSG00000163586	0	0	0	0	0	0	0	0	0	0	0	0	0	0	0	0	0	0	0	0	0	0	2	0	0	0	0	0	0	0	0	0
ENSG00000144115	0	0	0	0	0	0	1	2	0	0	0	0	0	0	0	2	1	0	0	0	1	0	1	0	0	0	1	0	0	1	0	1
ENSG00000232508	0	0	0	0	0	0	0	0	4	0	1	5	31	30	39	35	0	1	0	0	0	0	0	0	2	1	1	3	15	19	22	34
ENSG00000283262	0	0	0	0	0	0	0	0	0	0	0	0	0	0	0	0	0	0	0	0	0	0	0	0	0	0	0	0	0	0	0	0
ENSG00000212133	0	0	0	0	0	0	0	0	0	0	0	0	0	0	0	0	0	0	0	0	0	0	0	0	0	0	0	0	0	0	0	0
ENSG00000252395	0	0	0	0	0	0	0	0	0	0	0	0	0	0	0	0	0	0	0	0	0	0	0	0	0	0	0	0	0	0	0	0
ENSG00000224291	0	0	0	0	0	0	0	0	0	0	0	0	0	0	0	0	0	0	0	0	0	0	0	0	0	0	0	0	0	0	0	0
ENSG00000214336	0	0	0	0	0	0	0	0	0	0	0	0	0	0	0	0	0	0	0	0	0	0	0	0	0	0	0	1	0	0	0	0
ENSG00000172073	0	0	1	0	0	0	0	0	0	0	58	8	0	0	0	0	0	0	0	0	0	0	0	0	0	0	7	22	0	0	0	0
ENSG00000225420	3	0	2	4	2	2	0	2	1	1	23	2	2	2	3	7	0	2	0	0	4	3	6	2	2	14	2	15	2	3	2	1
ENSG00000172071	591	521	528	506	636	576	481	672	656	626	2125	498	472	509	499	605	204	293	238	362	1170	542	857	489	585	957	512	1850	365	502	459	575
ENSG00000234028	207	206	206	199	50	63	56	62	65	59	146	42	61	66	57	87	48	51	59	109	70	32	49	22	39	52	25	110	23	43	24	38
ENSG00000153574	2377	1476	1772	1547	1929	1781	1379	1865	1145	859	3268	685	2682	2209	2747	2893	2069	2955	2608	2242	3941	1512	2935	1272	1120	1671	610	2268	3052	2708	3074	3057
ENSG00000230006	2	1	4	4	0	0	0	0	0	1	1	0	1	1	0	1	1	2	1	9	2	0	1	1	0	0	0	3	0	5	0	6
ENSG00000283270	0	0	0	0	0	0	0	0	0	0	0	0	0	0	0	0	0	0	0	0	0	0	0	0	0	0	0	0	0	0	0	0
ENSG00000240040	0	0	0	0	0	0	0	0	0	0	0	0	0	0	0	0	0	0	0	0	0	0	0	0	0	0	0	0	0	0	0	0
ENSG00000265510	0	0	0	0	0	0	0	0	0	0	0	0	0	0	0	0	0	0	0	0	0	0	0	0	0	0	0	0	0	0	0	0
ENSG00000211592	0	0	1	0	51	60	42	57	1	0	0	0	1488	1223	1818	1692	0	0	0	0	8	6	12	5	0	0	0	0	1355	1355	1936	1595
ENSG00000211593	0	0	0	0	2	0	0	1	0	0	0	0	29	27	39	33	0	0	0	0	0	0	0	0	0	0	0	0	24	28	29	36
ENSG00000211594	0	0	0	0	3	2	0	3	0	0	0	0	33	25	48	49	0	0	0	0	1	0	0	0	0	0	0	0	17	26	28	33
ENSG00000211595	0	0	0	0	1	1	0	3	0	0	0	0	49	43	49	50	0	0	0	0	0	1	0	0	0	0	0	0	35	31	49	45
ENSG00000211596	0	0	0	0	0	0	0	0	0	0	0	0	0	0	0	0	0	0	0	0	0	0	0	0	0	0	0	0	0	0	0	0
ENSG00000211597	0	0	0	0	9	12	7	8	0	0	0	0	177	118	144	169	0	0	0	0	1	2	3	0	0	0	0	0	80	86	102	97
ENSG00000211598	0	0	0	0	0	1	0	0	0	0	0	0	2	1	2	2	0	0	0	0	0	0	0	2	0	0	0	0	0	0	0	1
ENSG00000211599	0	0	0	0	0	0	0	0	0	0	0	0	11	7	3	7	0	0	0	0	0	0	0	0	0	0	0	0	2	5	2	6
ENSG00000197794	0	0	0	0	0	0	0	0	0	0	0	0	0	1	0	0	0	0	0	0	0	0	0	0	0	0	0	0	0	0	0	0
ENSG00000225136	0	0	0	0	0	0	0	0	0	0	0	0	0	0	0	0	0	0	0	0	0	0	0	0	0	0	0	0	0	0	0	0
ENSG00000253435	0	0	0	0	0	0	0	0	0	0	0	0	0	0	0	0	0	0	0	0	0	0	0	0	0	0	0	0	0	0	0	0
ENSG00000243466	0	0	0	0	0	0	0	0	0	0	0	0	0	0	0	0	0	0	0	0	0	0	0	0	0	0	0	0	0	0	0	0

ENSG00000239855	0	0	0	0	0	0	0	0	0	0	0	0	0	0	0	0	0	0	0	0	0	0	0	0	0	0	0	0	0	0	0	0
ENSG00000243063	0	0	0	0	0	0	0	0	0	0	0	0	0	0	0	0	0	0	0	0	0	0	0	0	0	0	0	0	0	0	0	0
ENSG00000240671	0	0	0	0	0	0	0	0	0	1	0	0	0	0	0	0	0	0	0	0	0	0	0	0	0	0	0	0	0	0	0	0
ENSG00000241755	0	0	0	0	0	0	0	0	0	0	0	0	0	0	0	0	0	0	0	0	0	0	0	0	0	0	0	0	0	0	0	0
ENSG00000253278	0	0	0	0	0	0	0	0	0	0	0	0	0	0	0	0	0	0	0	0	0	0	0	0	0	0	0	0	0	0	0	0
ENSG00000241351	0	0	0	0	0	0	0	0	0	0	0	0	0	0	0	0	0	0	0	0	0	0	0	0	0	0	0	0	0	0	0	0
ENSG00000243290	0	0	0	0	0	0	0	0	0	0	0	0	0	0	0	0	0	0	0	0	0	0	0	0	0	0	0	0	0	0	0	0
ENSG00000253497	0	0	0	0	0	0	0	0	0	0	0	0	0	0	0	0	0	0	0	0	0	0	0	0	0	0	0	0	0	0	0	0
ENSG00000253265	0	0	0	0	0	0	0	0	0	0	0	0	0	0	0	0	0	0	0	0	0	0	0	0	0	0	0	0	0	0	0	0
ENSG00000244437	0	0	0	0	0	0	0	0	0	0	0	0	0	0	0	0	0	0	0	0	0	0	0	0	0	0	0	0	0	0	0	0
ENSG00000240864	0	0	0	0	0	0	0	0	1	0	0	0	0	0	0	0	0	0	0	0	0	0	0	0	0	0	0	0	0	0	0	0
ENSG00000240382	0	0	0	0	0	0	0	0	0	0	0	0	0	0	0	0	0	0	0	0	0	0	0	0	0	0	0	0	0	0	0	0
ENSG00000254157	0	0	0	0	0	0	0	0	0	0	0	0	0	0	0	0	0	0	0	0	0	1	1	0	0	0	0	0	0	0	0	0
ENSG00000253732	0	0	0	0	0	0	0	0	0	0	1	0	0	0	0	0	0	0	0	0	0	0	0	0	0	0	0	0	0	0	0	0
ENSG00000239951	0	0	0	0	0	0	0	0	0	0	0	0	0	0	0	0	0	0	0	0	0	0	0	0	0	0	0	0	0	0	0	0
ENSG00000211611	0	0	0	0	0	0	0	0	0	0	0	0	0	0	0	0	0	0	0	0	0	0	0	0	0	0	0	0	0	0	0	0
ENSG00000253578	0	0	0	0	0	0	0	0	0	0	0	0	0	0	0	0	0	0	0	0	0	0	0	0	0	0	0	0	0	0	0	0
ENSG00000253625	0	0	0	0	0	0	0	0	0	0	0	0	0	0	0	0	0	0	0	0	0	0	0	0	0	0	0	0	0	0	0	0
ENSG00000241294	0	0	0	0	0	0	0	0	0	0	0	0	0	0	0	0	0	0	0	0	0	0	0	0	0	0	0	0	0	0	0	0
ENSG00000253202	0	0	0	0	0	0	0	0	0	0	0	0	0	0	0	0	0	0	0	0	0	0	0	0	0	0	0	0	0	0	0	0
ENSG00000254098	0	0	0	0	0	0	0	0	0	0	0	0	0	0	0	0	0	0	0	0	0	0	0	0	0	0	0	0	0	0	0	0
ENSG00000277852	0	0	0	0	0	0	0	0	0	0	0	0	0	0	0	0	0	0	0	0	0	0	0	0	0	0	0	0	0	0	0	0
ENSG00000244575	0	0	0	0	0	0	0	0	0	0	0	0	0	0	0	0	0	0	0	0	1	0	0	0	0	0	0	0	0	0	0	0
ENSG00000244116	0	0	0	0	0	0	0	0	0	0	0	0	0	0	0	0	0	0	0	0	0	0	0	0	0	0	0	0	0	0	0	0
ENSG00000253998	0	0	0	0	0	0	0	0	0	0	0	0	0	0	0	0	0	0	0	0	0	0	0	0	0	0	0	0	0	0	0	0
ENSG00000243238	0	0	0	0	0	0	0	0	0	0	0	0	0	0	0	0	0	0	0	0	0	0	0	0	0	0	0	0	0	0	0	0
ENSG00000253158	0	0	0	0	0	0	0	0	0	0	0	0	0	0	0	0	0	0	0	0	0	0	0	0	0	0	0	0	0	0	0	0
ENSG00000253870	0	0	0	0	0	0	0	0	0	0	0	0	0	0	0	0	0	0	0	0	0	0	0	0	0	0	0	0	0	0	0	0
ENSG00000242076	0	0	0	0	0	0	0	0	0	0	0	0	0	0	0	0	0	0	0	0	0	0	0	0	0	0	0	0	0	0	0	0
ENSG00000253860	0	0	0	0	0	0	0	0	0	0	0	0	0	0	0	0	0	0	0	0	0	0	0	0	0	0	0	0	0	0	0	0
ENSG00000253461	0	0	0	0	0	0	0	0	0	0	0	0	0	0	0	0	0	0	0	0	0	0	0	0	0	0	0	0	0	0	0	0
ENSG00000253487	0	0	0	0	0	0	0	0	0	0	0	0	0	0	0	0	0	0	0	0	0	0	0	0	0	0	0	0	0	0	0	0
ENSG00000239862	0	0	0	0	0	0	0	0	0	0	0	0	0	0	0	0	0	0	0	0	0	0	2	0	0	0	0	0	0	0	0	0
ENSG00000253592	0	0	0	0	0	0	0	0	0	0	0	0	0	0	0	0	0	0	0	0	0	0	0	0	0	0	0	0	0	0	0	0
ENSG00000242371	0	0	0	0	0	0	0	0	0	0	0	0	0	0	0	0	0	0	0	0	0	0	0	0	0	0	0	0	0	0	0	0
ENSG00000273962	0	0	0	0	0	0	0	0	0	0	0	0	0	0	0	0	0	0	0	0	0	0	0	0	0	0	0	0	0	0	0	0
ENSG00000283427	6	12	2	8	0	3	0	0	0	0	0	0	0	1	1	0	1	1	1	4	0	0	0	0	1	0	1	1	0	0	0	2
ENSG00000283196	157	132	155	158	62	57	51	63	54	40	194	34	55	53	55	61	118	170	134	160	98	45	59	40	37	51	39	94	55	67	45	81
ENSG00000283132	0	0	0	0	0	0	0	0	0	0	1	0	0	0	0	0	0	0	0	0	0	0	0	0	0	0	1	0	0	0	0	0
ENSG00000283214	0	0	0	0	0	0	0	0	0	0	0	0	0	0	0	0	0	0	0	0	0	0	0	0	0	0	0	0	0	0	0	0
ENSG00000283659	0	0	0	0	0	0	0	0	0	0	0	0	0	0	0	0	0	0	0	0	0	0	0	0	0	0	0	0	0	0	0	0
ENSG00000251039	0	0	0	0	0	0	0	0	0	0	0	0	0	0	0	0	0	0	0	1	1	0	0	0	0	0	0	0	0	0	0	0
ENSG00000251546	0	0	0	0	0	0	0	0	0	0	0	0	0	0	0	0	0	0	0	0	0	0	0	0	0	0	0	0	0	0	0	0
ENSG00000254009	0	0	0	0	0	0	0	0	0	0	0	0	0	0	0	0	0	0	0	0	0	0	0	0	0	0	0	0	0	0	0	0
ENSG00000250036	0	0	0	0	0	0	0	0	0	0	0	0	0	0	0	0	0	0	0	0	0	0	0	0	0	0	0	0	0	0	0	0
ENSG00000253127	0	0	0	0	0	0	0	0	0	0	0	0	0	0	0	0	0	0	0	0	0	0	0	0	0	0	0	0	0	0	0	0
ENSG00000232747	0	0	0	0	0	0	0	0	0	0	0	0	0	0	0	0	0	0	0	0	0	0	0	0	0	0	0	0	0	0	0	0
ENSG00000253501	0	0	0	0	0	0	0	0	0	0	0	0	0	0	0	0	0	0	0	0	0	0	0	0	0	0	0	0	0	0	0	0
ENSG00000239975	0	0	0	0	0	0	0	0	0	0	0	0	0	0	0	0	0	0	0	0	0	0	0	0	0	0	0	0	0	0	0	0
ENSG00000253191	0	0	0	0	0	0	0	0	0	0	0	0	0	0	0	0	0	0	0	0	0	0	0	0	0	0	0	0	0	0	0	0
ENSG00000253999	0	0	0	0	0	0	0	0	0	0	0	0	0	0	0	0	0	0	0	0	0	0	0	0	0	0	0	0	0	0	0	0
ENSG00000239571	0	0	0	0	0	0	0	0	0	0	0	0	0	0	0	0	0	0	0	0	0	0	0	0	0	0	0	0	0	0	0	0
ENSG00000243264	0	0	0	0	0	0	0	0	0	0	0	0	0	0	0	0	0	0	0	0	0	0	0	0	0	0	0	0	0	0	0	0
ENSG00000242534	0	0	0	0	0	0	0	0	0	0	0	0	0	0	0	0	0	0	0	0	0	0	0	0	0	0	0	0	0	0	0	0
ENSG00000224607	0	0	0	0	0	0	0	0	0	0	1	0	0	0	0	0	0	0	0	0	0	0	0	0	0	0	0	0	0	0	0	0
ENSG00000211623	0	0	0	0	0	0	0	0	0	0	1	0	0	0	0	0	0	0	0	0	0	0	0	0	0	0	0	0	0	0	0	0
ENSG00000254097	0	0	0	0	0	0	0	0	0	0	0	0	0	0	0	0	0	0	0	0	0	0	0	0	0	0	0	0	0	0	0	0
ENSG00000241566	0	0	0	0	1	0	0	0	0	0	0	0	0	0	0	0	0	0	0	0	1	0	0	0	0	0	0	0	0	0	0	0
ENSG00000254345	0	0	0	0	0	0	0	0	0	0	0	0	0	0	0	0	0	0	0	0	1	0	0	0	0	0	0	0	0	0	0	0
ENSG00000253365	0	0	0	0	0	0	0	0	0	0	0	0	0	0	0	0	0	0	0	0	2	0	0	0	0	0	0	0	0	0	0	0
ENSG00000225523	0	0	0	0	0	0	0	0	0	0	0	0	0	0	0	0	0	0	0	0	0	0	0	0	0	0	0	0	0	0	0	0
ENSG00000211625	0	0	0	0	0	0	0	0	0	0	0	0	0	0	0	0	0	0	0	0	0	0	0	0	0	0	0	0	0	0	0	0
ENSG00000253765	0	0	0	0	0	0	0	0	0	0	0	0	0	0	0	0	0	0	0	0	0	0	0	0	0	0	0	0	0	0	0	0
ENSG00000254220	0	0	0	0	0	0	0	0	0	0	0	0	0	0	0	0	0	0	0	0	0	0	0	0	0	0	0	0	0	0	0	0
ENSG00000211626	0	0	0	0	0	0	0	0	0	0	0	0	0	0	0	0	0	0	0	0	0	1	0	1	0	0	0	0	0	0	0	0
ENSG00000242766	0	0	0	0	0	0	0	0	0	0	0	0	0	0	0	0	0	0	0	0	0	0	0	0	0	0	0	0	0	0	0	0
ENSG00000241244	0	0	0	0	0	0	0	0	0	0	0	0	0	0	0	0	0	0	0	0	0	0	0	0	0	0	0	0	0	0	0	0
ENSG00000224041	0	0	0	0	0	0	0	0	0	0	0	0	0	0	0	0	0	0	0	0	0	0	0	0	0	0	0	0	0	0	0	0
ENSG00000254292	0	0	0	0	0	0	0	0	0	0	0	0	0	0	0	0	0	0	0	0	0	0	0	0	0	0	0	0	0	0	0	0
ENSG00000276566	0	0	0	0	0	0	0	0	0	0	0	0	0	0	0	0	0	0	0	0	0	0	0	0	0	0	0	0	0	0	0	0
ENSG00000278857	0	0	0	0	0	0	0	0	0	0	0	0	0	0	0	0	0	0	0	0	0	0	0	0	0	0	0	0	0	0	0	0
ENSG00000211632	0	0	0	0	0	0	0	0	0	0	0	0	0	0	0	0	0	0	0	0	0	0	0	0	0	0	0	0	0	0	0	0
ENSG00000253906	0	0	0	0	0	0	0	0	0	0	0	0	0	0	0	0	0	0	0	0	0	0	0	0	0	0	0	0	0	0	0	0
ENSG00000211633	0	0	0	0	0	0	0	0	0	0	0	0	0	0	0	0	0	0	0	0	0	0	0	0	0	0	0	0	0	0	0	0
ENSG00000242580	0	0	0	0	0	0	0	0	0	0	0	0	0	0	0	0	0	0	0	0	0	0	0	0	0	0	0	0	0	0	0	0
ENSG00000239819	0	0	0	0	0	0	0	0	0	0	0	0	0	0	0	0	0	0	0	0	0	0	0	0	1	0	0	0	0	0	0	0
ENSG00000228325	0	0	0	0	0	0	0	0	0	0	0	0	0	0	0	0	0	0	0	0	0	0	0	0	0	0	0	0	0	0	0	0
ENSG00000275490	0	0	0	0	0	0	0	0	0	0	0	0	0	0	0	0	0	0	0	0	0	0	0	0	0	0	0	0	0	0	0	0
ENSG00000270999	0	0	0	0	0	0	0	0	0	0	0	0	0	0	0	0	0	0	0	0	0	0	0	0	0	0	0	0	0	0	0	0
ENSG00000265897	0	0	0	0	0	0	0	0	0	0	0	0	0	0	0	0	0	0	0	0	0	0	0	0	0	0	0	0	0	0	0	0
ENSG00000281904	0	0	0	0	0	0	0	0	0	0	0	0	0	0	0	0	0	0	0	0	0	0	0	0	0	0	0	0	0	0	0	0
ENSG00000230964	0	0	0	0	0	0	0	0	0	0	0	0	0	0	0	0	0	0	0	0	0	0	0	0	0	0	0	0	0	0	0	0
ENSG00000261600	1	2	0	2	0	0	0	0	0	0	0	0	0	0	0	0	1	0	0	0	0	0	0	0	0	0	0	0	0	0	0	0
ENSG00000278131	13	10	9	10	1	0	0	0	0	0	0	0	0	1	0	1	0	1	1	4	0	0	0	0	0	0	0	0	0	0	0	0
ENSG00000143429	125	105	104	105	42	38	32	53	17	9	73	13	61	69	71	104	73	99	75	121	73	31	53	26	16	15	25	53	65	68	65	93
ENSG00000184303	0	0	0	0	0	0	0	0	0	0	0	0	0	0	0	0	0	0	0	0	0	0	0	0	0	0	0	0	0	0	0	0
ENSG00000230572	0	0	0	0	0	0	0	0	0	0	1	0	0	0	0	0	0	0	1	0	0	0	0	0	0	0	1	1	0	0	0	0
ENSG00000271627	0	0	0	0	0	0	0	0	0	0	0	0	0	0	0	0	0	0	0	0	0	0	0	0	0	0	0	0	0	0	0	0
ENSG00000223703	0	0	0	0	0	0	0	0	0	0	0	0	0	0	0	0	0	0	0	0	0	0	0	0	0	0	0	0	0	0	0	0
ENSG00000232531	1	0	3	1	1	0	1	1	4	1	5	0	3	1	0	1	0	0	0	1	1	2	0	0	2	0	1	3	3	0	1	1
ENSG00000236969	0	0	0	0	0	0	0	0	0	0	0	0	0	0	0	0	0	0	0	0	0	0	0	0	0	0	0	0	0	0	0	0
ENSG00000235235	0	0	0	0	0	0	0	0	0	0	0	0	0	0	0	0	0	0	0	0	0	0	0	0	0	0	0	0	0	0	0	0
ENSG00000214330	0	0	0	0	0	0	0	0	0	0	0	0	0	0	0	0	0	0	0	0	0	0	0	0	0	0	0	0	0	0	0	0
ENSG00000237085	0	0	0	0	0	0	0	0	0	0	0	0	0	0	0	0	0	0	0	0	0	0	0	0	0	0	0	0	0	0	0	0
ENSG00000227497	0	0	0	0	0	0	0	0	0	0	0	0	0	0	0	0	0	0	0	0	0	0	0	0	0	0	0	0	0	0	0	0
ENSG00000214329	0	0	0	0	0	0	0	0	0	0	0	0	0	0	0	0	0	0	0	0	0	0	0	0	0	0	0	0	0	0	0	0
ENSG00000226481	0	0	0	0	0	0	0	0	0	0	0	0	0	0	0	0	0	0	0	0	0	0	0	0	0	0	0	0	0	0	0	0
ENSG00000234898	0	0	0	0	0	0	0	0	0	0	0	0	0	0	0	0	0	0	0	0	0	0	0	0	0	0	0	0	0	0	0	0
ENSG00000235147	0	0	0	0	0	0	0	0	0	0	0	0	0	0	0	0	0	0	0	0	0	0	0	0	0	0	0	0	0	0	0	0
ENSG00000223816	0	0	0	0	0	0	0	0	0	0	0	0	0	0	0	0	0	0	0	0	0	0	0	0	0	0	0	0	0	0	0	0
ENSG00000261522	0	0	0	0	0	0	0	0	0	0	1	0	8	8	10	13	0	0	0	0	0	0	0	0	0	0	0	0	1	2	1	2
ENSG00000259916	0	0	0	0	0	0	0	0	0	0	0	0	0	0	0	0	0	0	0	0	0	0	0	0	0	0	0	0	0	0	0	0
ENSG00000237383	0	0	0	0	0	0	0	0	0	0	0	0	0	0	0	0	0	0	0	0	0	0	0	0	0	0	0	0	0	0	0	0
ENSG00000227265	0	1	0	0	0	0	0	0	0	0	0	0	0	0	0	0	0	0	0	0	0	0	0	0	0	0	0	0	0	0	0	0
ENSG00000213355	0	0	0	0	0	0	0	0	0	0	0	0	0	0	0	0	0	0	0	0	0	0	0	0	0	0	0	0	0	0	0	0
ENSG00000234837	0	0	0	0	0	0	0	0	0	0	0	0	0	0	0	0	0	0	0	0	0	0	0	0	0	0	0	0	0	0	0	0
ENSG00000223564	1	7	0	2	0	0	0	0	0	0	0	0	0	0	0	0	0	0	0	0	0	0	0	0	0	0	0	0	0	0	0	0
ENSG00000232502	0	0	0	0	0	0	0	0	0	0	0	0	0	0	0	0	0	0	0	1	0	0	0	0	0	0	0	0	0	0	0	0
ENSG00000235571	0	1	0	2	0	0	0	0	0	0	0	0	1	0	0	0	1	0	0	0	0	0	0	0	0	0	0	0	0	0	0	0
ENSG00000204717	0	0	0	1	0	0	0	0	0	0	0	0	0	0	0	0	0	0	0	1	0	0	0	0	0	0	0	0	0	0	0	0
ENSG00000229089	1	3	2	2	0	0	0	0	0	0	0	0	0	0	1	0	0	0	0	4	0	0	0	0	0	0	0	0	2	0	1	0
ENSG00000212140	0	0	0	0	0	0	0	0	0	0	0	0	0	0	0	0	0	0	0	0	0	0	0	0	0	0	0	0	0	0	0	0
ENSG00000278631	0	0	0	0	0	0	0	0	0	0	0	0	0	0	0	0	0	0	0	1	0	0	0	0	0	0	0	0	0	0	0	0
ENSG00000259848	0	2	0	3	0	0	0	0	0	0	0	0	0	1	0	0	0	0	0	0	0	0	0	0	0	0	0	0	0	0	0	1
ENSG00000163060	0	2	0	0	0	0	0	0	0	0	0	0	0	0	0	0	0	0	0	0	0	0	0	0	0	0	0	0	0	0	0	1
ENSG00000237055	0	0	0	0	0	0	0	0	0	0	0	0	0	0	0	0	0	0	0	0	0	0	0	0	0	0	0	0	0	0	0	0
ENSG00000232128	0	0	0	0	0	0	0	0	0	0	0	0	0	0	0	0	0	0	0	0	0	0	0	0	0	0	0	0	0	0	0	0
ENSG00000233158	0	0	0	0	0	0	0	0	0	0	0	0	0	1	0	0	0	0	0	0	0	0	0	0	0	0	0	0	0	0	0	0
ENSG00000231331	0	0	0	0	0	0	0	0	0	0	0	0	0	0	0	0	0	0	0	0	0	0	0	0	0	0	0	0	0	0	0	0
ENSG00000232594	0	0	0	0	0	0	0	0	0	0	0	0	0	0	0	0	0	0	0	0	0	0	0	0	0	0	0	0	0	0	0	0
ENSG00000233105	0	0	0	0	0	0	0	0	0	0	0	0	0	0	0	0	0	0	0	0	0	0	0	0	0	0	0	0	0	0	0	0
ENSG00000224585	0	0	0	0	0	0	0	0	0	0	0	0	0	0	0	0	0	0	0	0	0	0	0	0	0	0	0	0	0	0	0	0
ENSG00000233447	0	0	0	0	0	0	0	0	0	0	0	0	0	0	0	0	0	0	0	0	0	0	0	0	0	0	0	0	0	0	0	0
ENSG00000235186	0	0	0	0	1	1	0	0	1	3	5	1	0	1	3	1	0	1	0	0	0	0	0	0	0	0	0	1	0	1	0	0
ENSG00000277591	0	0	0	0	0	0	0	0	0	4	1	1	0	0	0	0	0	0	0	0	0	0	0	0	1	2	0	2	1	0	0	1
ENSG00000233850	3	1	2	3	0	0	0	0	60	65	123	41	0	0	0	0	0	0	0	2	0	0	0	0	20	51	3	37	0	0	1	0
ENSG00000172005	15	26	14	22	0	0	0	0	2595	2436	3604	1575	2	5	2	6	1	8	0	2	0	0	0	0	515	1404	95	1184	2	10	4	18
ENSG00000231062	0	1	4	0	0	0	0	0	79	60	113	38	0	1	0	2	1	0	0	0	0	0	0	0	10	24	4	29	0	0	1	1
ENSG00000144029	2700	1968	2327	2307	1808	1866	1323	2017	1489	1190	4183	831	1764	1719	1844	2280	1520	2168	1919	2095	3185	1327	2398	1162	1179	1898	819	2576	1700	1920	1961	2323
ENSG00000144026	368	237	388	258	339	452	345	527	407	357	1588	292	130	119	95	173	123	105	126	285	485	235	290	233	285	366	486	936	45	79	71	91
ENSG00000275111	266	162	205	168	134	129	115	155	110	93	442	75	145	138	129	164	89	123	109	181	206	96	164	77	121	169	141	316	116	125	129	131
ENSG00000275768	0	0	0	0	0	0	0	0	0	0	0	0	0	0	0	0	0	0	0	0	0	0	0	0	0	0	0	2	0	0	0	0
ENSG00000233757	140	88	100	80	157	155	120	139	20	10	30	10	3	2	1	5	28	46	41	84	290	118	174	103	5	18	15	37	3	0	2	0
ENSG00000155066	7	10	7	8	46	56	42	45	1	2	10	0	0	0	2	0	1	3	4	15	46	41	19	23	1	0	2	2	0	0	0	0
ENSG00000115041	129	106	105	151	180	171	109	195	0	0	5	1	1	1	0	1	33	58	39	104	239	107	196	91	0	0	0	5	1	0	0	0
ENSG00000235129	0	0	0	0	0	0	0	3	0	0	0	0	0	0	0	0	0	0	0	0	1	1	1	0	0	0	0	0	0	0	0	0
ENSG00000115042	1261	905	1060	928	645	694	452	657	516	419	1899	324	676	710	633	905	544	709	465	673	889	391	589	362	411	576	457	1244	431	519	503	617
ENSG00000248821	0	0	0	0	0	0	0	0	0	0	0	0	0	0	0	0	0	0	0	0	0	0	0	0	0	0	0	0	0	0	0	0
ENSG00000233275	0	0	0	0	0	0	0	0	0	0	0	0	0	0	0	0	0	0	0	0	0	0	0	0	0	0	0	0	0	0	0	0
ENSG00000227120	0	0	0	0	0	0	0	0	0	0	0	0	0	0	0	0	0	0	0	0	0	0	0	0	0	0	0	0	0	0	0	0
ENSG00000204705	0	0	0	0	0	0	0	0	0	0	0	0	0	0	0	0	0	0	0	0	0	0	0	0	0	0	0	0	0	0	0	0
ENSG00000277747	0	0	0	0	0	0	0	0	0	0	0	0	0	0	0	0	0	0	0	0	0	0	0	0	0	0	0	0	0	0	0	0
ENSG00000234903	0	0	0	0	0	0	0	0	0	0	0	0	0	0	0	0	0	0	0	0	0	0	0	0	0	0	0	0	0	0	0	0
ENSG00000144010	0	0	0	0	0	0	0	0	0	0	0	0	0	0	0	0	0	0	0	0	0	0	0	0	0	0	0	0	0	0	0	0
ENSG00000238162	0	0	0	0	0	0	0	0	0	0	0	0	0	0	0	0	0	0	0	0	0	0	0	0	0	0	0	0	0	0	0	0
ENSG00000223917	0	0	0	0	0	0	0	0	0	0	0	0	0	0	0	0	0	0	0	0	0	0	0	0	0	0	0	0	0	0	0	0
ENSG00000237308	0	0	0	0	0	0	0	0	0	0	0	0	0	0	0	0	0	0	0	0	0	0	0	0	0	0	0	0	0	0	0	0
ENSG00000224719	0	0	0	0	0	0	0	0	0	0	0	0	0	0	0	0	0	0	0	0	0	0	0	0	0	0	0	0	0	0	0	0
ENSG00000226185	0	0	0	2	0	1	0	2	0	0	0	0	0	0	1	0	1	0	0	0	2	0	0	0	0	0	0	0	0	0	0	0
ENSG00000272913	110	94	94	103	72	80	62	118	18	21	134	24	0	0	0	0	60	89	56	113	125	59	107	69	23	38	38	93	0	0	0	0
ENSG00000229689	88	64	85	55	76	90	69	100	79	81	397	70	0	0	0	0	21	21	29	98	136	80	86	50	59	127	84	218	0	0	0	0
ENSG00000236431	17	11	15	8	11	5	6	17	11	9	60	6	0	0	0	0	2	2	4	6	14	10	11	4	7	17	18	38	0	0	0	0
ENSG00000273305	21	17	24	8	10	15	8	5	19	14	77	8	0	0	0	0	1	5	8	7	20	11	9	8	13	14	16	35	0	0	0	0
ENSG00000233037	0	0	0	0	0	0	0	0	0	1	3	0	0	0	0	0	0	0	0	0	1	0	0	1	4	2	0	0	0	0	0	0
ENSG00000168992	0	0	1	0	2	0	0	0	0	0	1	0	0	0	0	0	0	1	0	0	0	0	0	2	0	0	0	3	0	0	0	0
ENSG00000232717	0	0	0	0	0	0	0	0	0	0	0	0	0	0	0	0	0	0	0	0	0	0	0	0	0	0	0	0	0	0	0	1
ENSG00000274028	0	0	0	0	0	0	0	0	0	0	0	0	0	0	0	0	0	0	0	0	0	0	0	0	0	0	0	0	0	0	0	0
ENSG00000230083	0	0	0	0	0	0	0	0	0	0	0	0	0	0	0	0	0	0	0	0	0	0	0	0	0	0	0	0	0	0	1	0
ENSG00000144015	0	0	0	0	0	0	0	0	0	0	2	0	0	0	0	0	0	0	0	0	0	0	0	0	0	0	0	0	0	0	0	0
ENSG00000236026	0	0	0	0	0	0	0	0	0	0	0	0	0	0	0	0	0	0	0	0	0	0	0	0	0	0	0	0	0	0	0	0
ENSG00000271003	0	0	0	0	0	0	0	0	0	0	0	0	0	0	0	0	0	0	0	0	0	0	0	0	0	0	0	0	0	0	0	0
ENSG00000270193	0	0	0	0	0	0	0	0	0	0	0	0	0	0	0	0	0	0	0	0	0	0	0	0	0	0	0	0	0	0	0	0
ENSG00000204699	0	0	0	0	0	0	0	0	0	0	0	0	0	0	0	0	0	0	0	0	0	0	0	0	0	0	0	0	0	0	0	0
ENSG00000275497	0	0	0	0	0	0	0	0	0	0	0	0	0	0	0	0	0	0	0	0	0	0	0	0	0	0	0	0	0	0	0	0
ENSG00000235959	0	0	0	0	0	0	0	0	0	0	0	0	0	0	0	0	0	0	0	0	0	0	0	0	0	0	0	0	0	0	0	0
ENSG00000236750	0	0	0	0	0	0	0	0	0	0	0	0	0	0	0	0	0	0	0	0	0	0	0	0	0	0	0	0	0	0	0	0
ENSG00000235584	0	0	0	0	0	0	0	0	0	0	0	0	0	0	0	0	0	0	0	0	0	0	0	0	0	0	0	0	0	0	0	0
ENSG00000237510	0	0	1	0	5	0	0	0	1	0	5	0	0	0	0	0	0	0	0	0	3	0	1	3	0	0	0	2	0	0	0	1
ENSG00000232931	1542	1161	1694	1281	1309	1459	1162	1571	1092	937	4070	710	318	375	338	461	387	360	363	1440	2136	1058	1102	1107	605	932	730	2328	181	511	239	644
ENSG00000174501	1675	1218	1502	1094	1430	1482	1261	1620	954	830	3229	576	651	694	631	702	550	535	489	1568	2322	1180	1546	1283	864	966	560	3017	310	741	535	825
ENSG00000275961	0	0	1	0	0	0	0	0	0	0	0	0	0	0	0	0	0	0	0	1	0	0	0	0	0	0	0	0	0	0	0	0
ENSG00000231584	236	172	206	138	118	137	82	144	89	81	504	87	188	157	195	249	99	175	89	204	139	66	64	71	58	78	97	290	132	161	139	192
ENSG00000186281	13	16	15	27	6	10	10	14	1	2	80	9	19	12	12	14	6	4	4	11	5	6	5	4	5	5	16	51	2	9	12	17
ENSG00000274286	3	5	2	2	0	2	1	4	10	15	1975	253	7	6	12	15	0	0	0	3	0	0	2	0	20	36	142	1080	0	3	2	7
ENSG00000188886	5	5	12	10	8	10	5	3	4	3	22	5	39	32	38	32	4	4	5	23	13	3	4	3	3	6	4	9	2	20	11	21
ENSG00000158050	619	428	642	497	296	299	208	236	164	179	983	172	937	804	1319	1060	356	569	585	593	589	300	436	215	145	272	129	457	903	915	1185	872
ENSG00000228873	3	2	1	2	5	0	2	1	0	0	2	0	1	1	4	2	0	0	0	1	1	1	0	1	2	0	0	0	3	1	3	3
ENSG00000084090	4766	3191	4274	3299	4338	4190	3442	4645	4798	3681	16121	2968	5588	5094	5843	6402	2547	4500	4104	4321	8812	3531	6780	3192	4878	7295	3205	11269	5781	6427	6108	6914
ENSG00000204685	119	108	130	164	128	138	72	164	87	104	473	79	106	104	111	113	44	54	49	106	206	103	146	96	102	128	112	281	62	138	57	108
ENSG00000135956	770	575	759	549	849	873	579	867	699	638	4076	567	804	733	843	1012	186	271	341	547	1392	634	990	483	720	1158	1073	2418	630	790	686	888
ENSG00000144021	2752	1964	2406	2011	1970	2038	1512	2087	2329	1944	9254	1610	2589	2445	2790	3153	1599	2164	1971	2378	3158	1363	2653	1226	1835	3094	1996	5411	2028	2581	2257	2992
ENSG00000144028	11999	9456	11210	9496	12973	13464	10356	14373	10223	8548	36192	6616	14556	12793	14816	16877	2509	3518	3439	7064	18557	8166	14499	7425	8198	14620	7647	24212	8348	12160	9670	13543
ENSG00000230747	6	4	6	0	58	66	34	73	89	48	243	61	64	48	45	77	4	1	3	7	82	40	52	45	43	60	44	113	33	43	34	37
ENSG00000198885	979	701	1019	773	739	772	660	854	984	737	2455	564	722	642	878	917	443	692	680	860	1403	568	1112	506	862	1242	317	1573	604	724	603	802
ENSG00000121152	2483	1776	2237	1863	2682	2544	2083	2842	1695	1324	4594	1128	2115	1897	2186	2698	1182	1498	1456	1566	4742	1798	3725	1720	2033	3080	572	4586	1574	2032	1862	2248
ENSG00000163121	0	0	0	0	0	0	0	0	0	0	0	0	2	1	2	5	0	0	0	0	0	0	1	0	0	0	0	2	1	0	0	2
ENSG00000235480	0	1	0	0	2	0	0	0	1	0	0	0	2	1	1	0	0	0	0	0	0	0	0	1	0	0	2	0	1	0	0	0
ENSG00000196843	494	441	522	410	388	412	270	416	1108	989	4986	816	1312	1408	1567	1775	189	277	386	434	754	322	569	272	950	1674	1440	3315	922	1195	1174	1455
ENSG00000114982	3009	2763	2797	2648	1623	1658	1272	1770	2477	2001	7295	1363	1457	1421	1369	1866	750	1203	1010	2142	2589	1051	1854	1033	1663	2989	1584	4761	719	1146	805	1297
ENSG00000249715	12	2	18	10	2	6	8	9	6	4	6	3	17	14	10	17	0	2	2	6	2	2	2	2	5	7	4	18	7	15	11	23
ENSG00000114988	590	399	484	409	407	408	344	426	293	214	896	189	398	399	518	543	225	400	374	363	512	197	487	209	205	342	234	480	437	455	521	551
ENSG00000158158	327	350	361	332	192	226	139	216	538	444	1639	347	325	368	314	491	63	156	95	255	202	97	144	63	305	586	390	1057	203	298	207	341
ENSG00000264157	3	0	5	1	0	0	1	1	5	3	15	0	2	0	0	2	2	0	0	6	0	0	0	0	0	1	1	1	0	1	1	1
ENSG00000273265	34	18	24	25	3	4	2	3	21	8	35	9	21	14	12	16	9	9	9	12	4	0	2	1	6	9	8	30	13	20	9	20
ENSG00000168763	934	825	944	866	889	1038	692	982	920	826	3756	683	967	855	878	1142	207	333	294	707	1402	618	947	525	671	1236	921	2398	712	891	609	892
ENSG00000163126	67	54	57	63	46	44	39	59	89	51	353	64	38	39	37	48	23	21	15	92	70	37	25	24	45	77	76	195	21	51	25	52
ENSG00000213337	618	500	552	498	243	292	187	327	390	284	1369	284	281	249	347	289	470	718	642	649	491	206	367	196	329	537	330	893	337	362	479	431
ENSG00000168758	190	368	192	337	803	890	565	912	2624	2091	10365	1749	603	729	783	967	34	74	45	145	1280	583	782	560	1849	3330	2404	5533	333	559	493	777
ENSG00000168754	5	6	6	18	2	0	0	0	29	23	67	7	6	11	8	7	0	0	1	4	4	2	3	1	12	20	13	35	3	2	4	9
ENSG00000274206	0	0	0	0	0	0	0	0	0	0	0	0	0	0	0	0	0	0	0	0	0	0	0	0	0	0	0	0	0	0	0	0
ENSG00000275094	0	0	0	0	0	0	0	0	0	0	1	0	0	0	0	0	0	0	0	0	0	0	0	0	0	0	0	0	0	0	0	0
ENSG00000273634	0	0	0	0	0	0	0	0	0	0	0	0	0	0	0	0	0	0	0	0	0	0	0	0	0	0	0	0	0	0	0	0
ENSG00000248134	0	0	0	0	0	0	0	0	0	0	0	0	0	0	0	0	0	0	0	0	0	0	0	0	0	0	0	0	0	0	0	0
ENSG00000144188	0	0	0	1	0	0	0	0	5	0	5	0	0	0	0	0	0	0	0	1	0	0	0	0	0	0	2	3	0	0	2	0
ENSG00000230343	0	0	0	0	0	0	0	0	0	0	0	0	0	0	0	0	0	0	0	0	0	0	0	0	0	0	0	0	0	0	0	0
ENSG00000236847	0	0	0	0	0	0	0	0	0	0	0	0	0	0	0	0	0	0	0	0	0	0	0	0	0	1	0	0	0	0	0	0
ENSG00000270748	0	0	0	0	0	0	0	0	0	1	0	0	0	0	0	0	0	0	0	0	0	0	0	0	0	0	0	1	0	0	0	0
ENSG00000271402	0	0	0	0	0	0	0	0	0	0	1	0	0	0	0	0	0	0	0	0	0	0	0	0	0	0	0	0	0	0	0	0
ENSG00000204670	0	0	0	0	0	0	0	0	0	0	0	0	0	0	0	0	0	0	0	0	0	0	0	0	0	0	0	0	0	0	0	0
ENSG00000188383	44	38	43	34	3	2	4	0	16	21	64	18	12	14	8	18	21	11	14	66	3	2	4	4	22	23	19	46	6	4	1	17
ENSG00000144199	599	474	476	503	73	96	57	83	436	389	1363	270	439	386	506	515	301	389	261	390	92	41	83	43	355	601	420	1039	305	372	340	414
ENSG00000279791	23	9	14	6	0	2	4	1	8	3	8	2	4	9	2	3	10	6	10	9	0	0	4	0	3	8	4	12	0	5	0	4
ENSG00000275655	0	0	3	1	0	0	0	0	0	1	1	0	1	0	0	1	1	0	0	4	0	0	0	0	0	0	0	0	0	0	0	0
ENSG00000135976	2201	1783	1999	1631	1282	1423	1119	1445	1186	1080	4047	699	1269	1286	1106	1362	547	549	433	1571	2019	1111	1314	1074	1218	1436	855	3336	499	1284	776	1294
ENSG00000277701	4	0	0	2	1	2	0	1	5	0	2	0	2	2	0	1	1	0	0	0	6	2	4	2	0	1	0	2	0	1	0	1
ENSG00000237837	0	0	0	0	0	0	0	0	0	0	0	0	0	0	0	0	0	0	0	0	0	0	0	0	0	0	0	0	0	0	0	0
ENSG00000271351	0	0	0	0	0	0	0	0	0	0	0	0	0	0	0	0	0	0	0	0	0	0	0	0	0	0	0	0	0	0	0	0
ENSG00000276050	0	0	0	0	0	0	0	0	0	0	0	0	0	0	0	0	0	0	0	0	0	0	0	0	0	0	0	0	0	0	0	0
ENSG00000271015	0	0	0	0	0	0	0	0	0	0	0	0	0	0	0	0	0	0	0	0	0	0	0	0	0	0	0	0	0	0	0	0
ENSG00000270252	0	0	0	0	0	0	0	0	0	0	0	0	0	0	0	0	0	0	0	0	0	0	0	0	0	0	0	0	0	0	0	0
ENSG00000271569	0	0	0	0	0	0	0	0	0	0	0	0	0	0	0	0	0	0	0	0	0	0	0	0	0	0	0	0	0	0	0	0
ENSG00000270450	0	0	0	0	0	0	0	0	0	0	0	0	0	0	0	0	0	0	0	0	0	0	0	0	0	0	0	0	0	0	0	0
ENSG00000278537	0	0	0	0	0	0	0	0	0	0	0	0	0	0	0	0	0	0	0	0	0	0	0	0	0	0	0	0	0	0	0	0
ENSG00000270187	0	0	0	0	0	0	0	0	0	0	0	0	0	0	0	0	0	0	0	0	0	0	0	0	0	0	0	0	0	0	0	0
ENSG00000230606	44	44	48	52	84	90	89	110	94	70	391	52	33	30	56	49	17	12	11	55	119	47	83	64	39	62	70	184	20	50	25	56
ENSG00000278766	0	5	1	0	5	5	8	2	1	0	8	0	0	0	0	0	2	0	2	3	7	6	3	5	0	0	0	5	0	1	1	1
ENSG00000196912	940	699	797	746	784	772	554	872	462	483	2095	392	314	311	259	365	269	225	207	645	1170	552	792	617	465	615	392	1509	133	298	184	362
ENSG00000235833	2	6	4	4	8	8	3	9	4	0	9	3	0	0	0	0	0	0	2	5	7	7	10	9	8	7	3	7	0	0	0	0
ENSG00000228970	1	1	1	0	0	0	0	0	0	0	0	0	0	1	0	0	0	0	0	3	1	1	0	0	0	0	0	0	0	0	0	0
ENSG00000135940	1983	1357	1586	1370	1269	1278	894	1453	1863	1301	5589	1111	1935	1802	1921	2402	2541	2538	2933	2721	3191	1371	2225	1747	1516	2462	1252	3667	2801	2240	3116	3000
ENSG00000115073	2530	1960	1901	2001	1665	1802	1057	1888	1742	1379	5606	1174	1720	1773	1337	2235	684	909	741	1231	1756	810	1300	780	1181	1761	1230	3340	804	1176	736	1216
ENSG00000228486	109	73	98	98	199	245	162	279	127	129	580	69	148	188	157	236	21	29	32	112	287	158	176	166	81	94	114	262	51	139	77	159
ENSG00000201806	1	0	6	2	6	6	1	9	2	4	11	0	2	3	1	2	1	0	1	5	13	6	3	2	4	4	1	7	2	0	1	3
ENSG00000115085	11260	7462	11813	8118	4498	4763	3435	4882	5717	4689	28351	4166	4234	3731	4459	5024	3906	3529	4825	7678	6790	3185	4768	3161	3986	7013	7201	14623	2829	3932	3060	4551
ENSG00000075568	1967	1529	1904	1525	2033	2256	1594	2442	2557	2194	13526	1828	2193	2165	2449	2793	424	534	502	1176	3026	1325	2193	1137	1910	3293	2922	7366	1519	2417	1773	2509
ENSG00000235734	5	3	4	3	34	48	28	28	3	2	14	0	6	11	12	15	2	4	1	3	63	33	48	35	0	4	7	24	14	13	21	21
ENSG00000238719	4	0	0	1	0	0	0	1	6	1	7	0	0	1	0	0	0	1	0	6	2	1	0	1	1	6	2	14	1	4	3	5
ENSG00000168658	1	2	6	1	3	4	0	7	2	0	12	1	9	8	9	12	0	0	2	0	4	5	2	4	0	2	2	3	3	4	5	11
ENSG00000231635	0	0	0	0	0	2	0	1	0	0	0	0	0	1	0	0	0	0	0	0	0	0	0	0	0	0	0	0	0	0	0	0
ENSG00000222000	0	0	0	0	0	0	0	0	0	0	3	0	0	1	0	0	0	0	0	0	0	0	2	0	0	0	1	0	0	0	1	0
ENSG00000144191	0	1	0	0	0	0	0	0	0	0	0	0	0	0	0	0	0	0	0	0	0	0	0	0	0	0	0	0	0	0	0	0
ENSG00000227987	0	0	0	0	0	0	0	0	0	0	0	0	0	0	0	0	0	0	0	0	0	0	0	0	0	0	0	0	0	0	0	0
ENSG00000040933	3447	2965	3341	3143	972	1111	770	1077	5280	4726	24558	3831	1532	1380	1489	1716	830	1064	1002	2194	1613	799	1278	726	4914	8028	6397	16465	993	1565	1140	1587
ENSG00000183513	1026	772	856	837	618	650	442	709	797	683	3164	510	581	525	592	732	718	792	601	842	1081	430	750	471	644	914	868	1723	439	543	544	704
ENSG00000115446	960	717	835	790	719	759	583	805	535	468	2678	366	422	387	533	575	665	856	889	973	1599	695	1032	720	595	880	736	1582	433	499	565	651
ENSG00000071073	4997	4788	3993	4704	377	444	341	492	1759	1513	10145	1213	1117	989	991	1268	1926	2982	2113	3012	819	350	635	353	2430	3084	4063	7039	624	808	586	951
ENSG00000236564	9	3	4	5	1	2	1	0	2	2	31	2	1	0	1	2	4	2	0	3	1	0	0	0	3	8	14	26	2	3	1	1
ENSG00000226791	151	128	185	127	0	1	1	1	12	14	62	7	40	28	58	48	104	113	149	127	1	0	3	1	12	26	38	45	35	37	69	62
ENSG00000201070	0	0	0	2	0	0	0	0	0	0	0	0	0	0	0	0	0	0	0	0	0	0	0	0	0	0	0	0	0	0	0	0
ENSG00000196872	1	0	2	3	0	0	0	0	1	1	6	1	4	2	4	3	7	1	2	6	0	0	0	0	0	0	1	5	0	1	1	0
ENSG00000238830	0	0	0	0	0	0	0	0	0	0	0	0	0	0	0	0	0	0	0	0	0	0	0	0	0	0	0	0	0	0	0	0
ENSG00000135951	292	237	245	227	86	116	70	116	218	224	1182	180	261	201	246	285	126	183	182	258	162	94	105	89	184	309	352	722	149	260	230	276
ENSG00000231822	42	27	29	35	7	11	13	8	23	19	124	7	30	24	14	37	15	14	13	25	21	5	14	9	30	22	23	74	21	33	9	35
ENSG00000241962	60	23	42	24	14	8	16	16	24	16	71	13	13	12	12	8	35	39	35	54	38	14	32	14	16	23	29	55	7	10	4	16
ENSG00000273045	94	102	75	72	21	31	22	27	50	31	114	9	69	55	60	57	84	91	78	77	47	17	19	25	29	33	22	42	63	45	61	50
ENSG00000144182	172	152	157	133	97	108	105	121	149	114	553	85	91	106	98	123	217	251	216	277	224	80	133	100	137	182	104	289	87	90	89	116
ENSG00000273155	8	4	6	6	11	6	10	3	6	11	16	1	14	6	4	6	5	11	6	1	8	7	9	13	3	7	4	2	6	3	6	3
ENSG00000158411	938	635	745	562	486	516	390	574	576	425	1792	298	466	458	451	557	881	877	867	908	978	439	661	496	524	673	447	1182	415	545	473	565
ENSG00000185414	1141	778	784	742	777	935	652	909	837	603	2591	436	1128	951	1157	1253	990	1430	1134	1205	2180	829	1522	820	846	1154	559	1864	1417	1246	1503	1537
ENSG00000185674	29	13	30	25	9	11	8	15	24	28	178	16	6	4	4	24	13	5	12	48	20	13	11	9	20	35	26	110	10	16	6	13
ENSG00000144214	112	60	114	86	12	14	14	27	14	19	90	18	23	14	27	27	59	63	39	113	17	15	13	22	22	16	19	45	11	14	19	24
ENSG00000115514	631	412	404	356	521	514	430	597	437	399	1870	350	497	486	600	570	615	737	574	787	1517	620	1038	856	600	835	400	1406	755	680	812	854
ENSG00000158417	9777	5818	7973	6452	5754	5515	5042	6138	5095	3427	14997	2583	6967	5808	7133	7202	6809	8965	8009	8245	13223	5182	9764	4812	4896	6983	3254	10476	6397	6793	7038	7947

ENSG00000135945	1752	1236	1415	1245	1245	1433	1048	1468	822	704	3008	546	899	804	915	998	572	780	744	1323	2639	1110	1759	1133	748	1118	762	2228	569	894	700	1018
ENSG00000273306	39	60	46	23	23	16	17	28	34	23	105	17	29	27	27	28	15	21	15	37	50	18	28	26	26	23	15	80	25	39	30	39
ENSG00000144218	1829	1755	1806	1783	1256	1338	969	1521	10	24	52	16	0	1	1	0	344	688	534	1236	1572	697	1272	604	7	21	12	46	0	0	0	1
ENSG00000230393	28	13	29	21	7	9	4	8	0	0	1	0	0	0	0	0	3	11	10	16	20	7	8	16	0	1	0	0	0	0	0	0
ENSG00000232084	0	0	0	0	0	0	0	1	0	0	1	0	0	0	0	1	0	0	0	0	0	0	1	0	0	0	0	0	0	0	0	0
ENSG00000170500	25	17	42	14	279	350	250	402	0	2	2	0	2101	2178	1998	2593	11	11	11	36	355	215	221	180	1	0	0	2	885	1775	1002	1808
ENSG00000269383	0	0	0	0	0	0	0	0	0	0	0	0	0	0	0	0	0	0	0	0	0	0	0	0	0	0	0	0	0	0	0	0
ENSG00000270699	0	0	0	0	0	0	0	0	0	0	0	0	0	0	0	0	0	0	0	0	0	0	0	0	0	0	0	0	0	0	0	0
ENSG00000115526	487	461	445	415	516	474	369	558	246	225	1044	194	435	505	462	633	270	503	358	526	834	411	650	342	259	394	255	768	341	446	319	463
ENSG00000238029	0	0	0	1	0	0	0	0	0	0	0	0	0	0	0	0	1	0	0	0	0	0	0	0	0	0	0	0	0	0	0	0
ENSG00000204640	0	0	0	0	0	0	0	0	0	0	0	0	0	0	0	0	0	0	0	0	0	0	0	0	0	0	0	0	0	0	0	0
ENSG00000204637	0	0	0	0	0	0	0	0	0	0	0	0	0	0	0	0	0	0	0	0	0	0	0	0	0	0	0	0	0	0	0	0
ENSG00000115539	894	543	772	607	809	756	661	814	534	380	1952	321	995	962	1189	1166	1293	1586	1493	1125	2220	822	1641	868	546	795	396	1192	1257	1276	1552	1430
ENSG00000227259	0	0	0	0	0	0	0	0	0	0	0	0	0	0	0	0	0	0	0	0	0	0	0	0	0	0	0	0	0	0	0	0
ENSG00000234988	0	0	0	0	0	0	0	0	0	0	0	0	0	0	0	0	0	0	0	0	0	0	0	0	0	0	0	0	0	0	0	0
ENSG00000226034	0	0	0	0	0	0	0	0	0	0	0	0	0	0	0	0	0	0	0	0	0	0	0	0	0	0	0	0	0	0	0	0
ENSG00000234169	0	0	0	0	0	0	0	0	0	0	0	0	0	0	0	0	0	0	0	0	0	0	0	0	0	0	0	0	0	0	0	0
ENSG00000228488	0	0	0	0	0	0	0	0	0	0	0	0	0	0	0	0	0	0	0	0	0	0	0	0	0	0	0	0	0	0	0	0
ENSG00000170485	219	337	264	318	5	11	1	9	1	0	2	0	49	71	37	88	50	135	58	231	7	2	3	10	0	0	2	4	21	65	24	65
ENSG00000232034	3	8	3	8	0	0	0	0	0	0	2	0	1	0	1	1	0	0	0	1	0	0	0	0	0	0	0	0	0	4	0	1
ENSG00000230140	1	1	2	2	1	1	0	2	0	0	0	0	0	1	0	0	0	0	1	0	1	0	0	0	0	0	0	2	0	0	2	3
ENSG00000223947	6	12	8	6	2	0	2	1	0	0	0	0	4	2	1	7	0	1	5	10	1	2	1	1	0	0	0	0	0	5	0	8
ENSG00000071082	13695	9056	8568	8404	3220	3144	2159	3489	14572	10697	46326	9552	23940	23216	24724	30569	12528	14381	12941	14299	6781	2938	5359	3689	11548	16938	10598	26717	26613	23429	29761	31265
ENSG00000204634	431	488	409	430	120	169	109	163	74	60	383	53	632	605	592	866	141	194	118	451	198	106	133	103	63	105	64	227	384	729	421	845
ENSG00000271532	0	3	0	0	0	0	0	0	0	0	0	0	1	3	2	1	0	0	0	1	0	0	0	1	0	0	0	0	0	1	3	8
ENSG00000272902	12	19	15	21	18	23	16	24	1	3	4	0	10	10	19	15	12	6	3	15	30	17	27	9	0	0	0	1	6	12	11	12
ENSG00000158435	1681	1108	1396	1146	1986	1808	1498	1969	1429	1169	5256	909	3451	3047	3528	3925	807	1006	959	1196	3215	1240	2503	1196	1306	2079	1098	3094	2705	3116	2817	3304
ENSG00000163162	1668	1175	1404	1179	1029	1084	784	1070	838	687	3148	497	1969	1875	2003	2380	593	688	742	1150	1383	632	1002	557	699	936	779	1909	1163	1759	1382	1786
ENSG00000212283	31	16	15	12	13	12	7	9	14	5	36	4	30	30	20	20	11	5	3	10	12	5	8	8	6	11	8	21	8	14	19	28
ENSG00000264857	0	0	0	1	0	1	0	0	0	0	0	0	0	2	0	1	0	0	0	0	3	0	0	0	0	0	0	0	0	0	0	1
ENSG00000175874	2	1	2	3	18	31	15	25	2	0	3	1	2	6	1	3	3	0	3	8	23	10	17	2	0	0	0	1	1	0	2	4
ENSG00000226058	0	0	0	0	0	0	0	0	0	0	0	0	0	0	0	0	0	0	0	0	1	0	0	0	0	0	0	0	0	0	0	0
ENSG00000196460	492	560	596	545	273	316	226	327	0	0	0	0	73	77	69	89	216	262	208	625	340	156	281	197	0	0	0	0	23	55	27	70
ENSG00000223826	0	0	1	0	0	0	0	0	1	0	0	0	5	1	2	3	0	0	0	0	0	0	3	0	0	0	0	0	0	2	1	1
ENSG00000237549	0	0	0	0	1	0	1	0	0	0	0	0	27	30	26	25	0	0	0	0	0	0	1	1	0	0	0	0	18	15	36	35
ENSG00000232556	0	0	0	0	1	0	0	0	0	0	0	0	0	1	2	1	0	0	0	0	0	0	0	0	0	0	0	0	0	1	1	3
ENSG00000071054	10958	8336	10787	9187	8361	8526	6492	9070	2707	2236	18480	2569	10761	11145	12327	14505	4338	5855	6419	8949	13110	5453	10364	4650	3191	5021	4702	12967	7768	11238	9345	12628
ENSG00000281162	0	1	0	1	0	1	0	0	0	0	1	0	0	0	0	0	0	0	0	0	1	0	0	0	0	1	2	0	0	0	0	0
ENSG00000115590	2	3	2	4	1	0	0	0	0	0	0	0	0	0	0	0	5	10	2	10	0	0	0	0	0	0	0	0	0	0	0	2
ENSG00000115594	0	0	0	0	0	0	0	1	0	0	1	0	0	0	2	3	0	1	0	0	0	0	0	3	0	1	0	0	0	2	0	0
ENSG00000226925	0	0	0	0	0	0	0	0	0	0	0	0	0	0	0	0	0	0	0	0	0	0	0	0	0	0	0	0	0	1	0	0
ENSG00000115598	0	0	0	0	0	0	0	0	0	0	0	0	0	0	0	0	0	0	0	0	0	2	0	0	0	0	0	0	0	0	1	0
ENSG00000205716	0	0	0	0	0	0	0	0	0	0	0	0	0	0	0	0	0	0	0	0	0	0	0	0	0	0	0	0	0	0	0	0
ENSG00000115602	0	0	0	0	603	537	393	480	14	24	20	10	7	0	4	4	0	0	0	0	1061	417	516	400	10	9	0	9	2	0	0	2
ENSG00000115604	0	0	0	0	1133	1174	802	1219	848	664	1915	428	76	121	141	145	0	2	0	3	1952	783	1029	763	672	911	441	1294	52	129	114	184
ENSG00000236785	0	0	0	0	0	1	2	1	0	0	1	0	0	1	0	2	0	0	0	0	2	2	0	0	0	2	2	0	0	0	0	1
ENSG00000115607	0	0	0	0	3801	3307	2915	3503	189	155	618	91	18	13	11	18	0	0	0	0	9367	3517	6153	3554	205	186	227	326	23	27	47	42
ENSG00000264764	0	0	0	0	20	17	10	23	3	3	7	0	0	0	0	0	0	0	0	0	42	25	20	16	0	1	1	4	0	0	0	0
ENSG00000236525	0	0	0	0	229	156	194	206	19	11	54	3	1	1	2	0	0	0	0	0	384	190	217	225	6	4	9	18	1	2	0	1
ENSG00000234389	0	0	0	0	552	459	482	513	27	25	134	5	2	1	2	3	0	0	0	0	937	450	475	448	27	19	35	56	1	1	1	4
ENSG00000180251	0	0	0	0	1	0	0	0	0	0	0	0	0	0	0	1	0	1	0	0	0	1	0	0	0	0	0	0	0	3	0	1
ENSG00000115616	6	8	9	10	1	4	1	2	0	1	6	0	82	86	67	113	4	4	3	9	2	2	0	0	1	2	5	1	67	129	74	174
ENSG00000135953	880	755	786	712	414	377	240	438	405	334	1527	277	190	227	203	288	262	349	300	676	628	287	459	270	255	491	349	876	162	231	158	275
ENSG00000170417	336	238	232	218	207	245	199	259	145	117	386	85	205	171	197	242	245	438	361	372	571	196	504	219	99	157	49	227	178	205	283	235
ENSG00000277527	2	1	3	1	0	0	0	3	0	2	1	0	2	1	1	1	1	2	0	1	4	4	1	0	0	0	0	3	0	2	3	1
ENSG00000224095	3	1	2	1	0	0	0	0	2	0	6	0	2	0	0	2	0	1	0	0	0	0	0	0	0	0	0	0	0	0	0	0
ENSG00000227680	2	0	2	0	0	0	0	2	0	0	0	0	1	0	0	0	3	0	0	1	0	1	0	0	0	0	0	0	0	0	0	0
ENSG00000224850	0	1	0	1	0	0	0	0	2	0	0	0	0	2	0	0	0	0	0	0	1	0	0	0	0	0	0	0	0	0	0	0
ENSG00000229209	0	0	0	2	0	0	0	0	0	0	0	0	0	0	0	0	0	0	0	0	0	0	0	0	0	0	0	0	0	0	0	0
ENSG00000227623	0	0	0	0	0	0	0	0	0	0	0	0	0	0	0	0	0	0	0	0	0	0	0	0	0	0	0	0	0	0	0	0
ENSG00000236596	0	0	0	0	0	0	0	0	0	0	0	0	0	0	0	0	0	0	0	0	0	0	0	0	0	0	0	0	0	0	0	0
ENSG00000221849	0	0	0	0	0	0	0	0	0	0	0	0	1	0	0	0	0	0	0	0	0	0	0	0	1	0	1	0	0	1	0	0
ENSG00000231723	0	0	0	0	0	0	0	0	0	0	0	0	0	0	0	0	0	0	0	0	0	0	0	0	0	0	0	0	0	0	0	0
ENSG00000232597	0	0	0	0	0	0	0	0	0	0	0	0	0	0	0	0	0	0	0	0	0	0	0	0	0	0	0	0	0	0	0	0
ENSG00000236141	0	0	0	0	0	0	0	0	0	0	0	0	0	0	0	0	0	0	0	0	0	0	0	0	0	0	0	0	0	0	0	0
ENSG00000256637	0	0	0	0	0	0	0	0	0	0	0	0	0	0	0	0	0	0	0	0	0	0	0	0	0	0	0	1	0	0	0	0
ENSG00000273515	0	0	0	0	0	0	0	0	0	0	0	0	0	0	0	0	0	0	0	0	0	0	0	0	0	0	0	0	0	0	0	0
ENSG00000228968	0	0	0	0	0	0	0	0	0	0	0	0	0	0	0	0	0	0	0	0	0	0	0	0	0	0	0	0	0	0	0	0
ENSG00000233955	0	0	0	0	0	0	0	0	0	0	0	0	0	0	0	0	0	0	0	0	0	0	0	0	0	0	0	0	0	0	0	0
ENSG00000227157	0	0	0	0	0	0	0	0	0	0	0	0	0	0	0	0	0	0	0	0	0	0	0	0	0	0	0	0	0	0	0	0
ENSG00000225765	0	0	0	0	0	0	1	0	0	0	0	0	0	0	0	0	0	0	0	0	0	0	0	0	0	0	0	1	0	0	0	0
ENSG00000235597	0	0	0	0	0	0	0	0	0	0	1	0	0	0	0	0	0	0	0	0	0	0	0	0	0	0	1	2	0	0	1	0
ENSG00000234781	0	0	0	0	0	0	0	0	0	0	0	0	0	0	0	0	0	0	0	0	0	0	0	0	0	0	0	0	0	1	0	0
ENSG00000231626	0	0	0	0	0	0	0	0	0	0	0	0	0	0	0	0	0	0	0	0	0	0	0	0	0	0	0	0	0	0	0	0
ENSG00000236109	0	0	0	0	0	0	0	0	0	0	0	0	0	0	0	0	0	0	0	0	0	0	0	0	0	0	0	0	0	0	0	0
ENSG00000230690	0	0	0	0	7	1	20	13	0	0	0	0	0	0	0	0	0	0	0	0	36	7	27	11	0	0	0	0	0	0	0	0
ENSG00000225669	0	0	0	0	0	0	0	0	0	0	0	0	0	0	0	0	0	0	0	0	0	0	0	0	0	0	0	0	0	0	0	0
ENSG00000228528	0	0	0	0	0	0	0	0	0	0	15	0	0	1	0	2	0	0	0	0	0	0	0	0	0	0	1	3	0	0	0	0
ENSG00000279904	0	0	0	0	0	1	0	0	0	0	1	0	0	1	0	0	0	0	0	0	0	0	0	0	0	0	0	0	0	0	0	0
ENSG00000207249	0	0	0	0	0	0	0	0	0	0	0	0	0	0	0	0	0	0	0	0	0	0	0	0	0	0	0	0	0	0	0	0
ENSG00000234177	0	0	0	0	3	3	6	1	0	0	0	0	0	0	0	0	0	0	0	0	12	4	13	7	0	0	0	0	0	0	0	0
ENSG00000213301	0	0	0	0	0	0	0	0	0	0	0	0	0	0	0	0	0	0	0	0	0	0	0	0	0	0	0	0	0	0	0	0
ENSG00000233639	0	5	1	2	0	1	0	1	0	0	0	0	0	2	0	0	0	0	0	0	0	4	0	0	0	0	0	0	0	0	0	1
ENSG00000269707	0	2	0	3	0	1	1	0	0	0	0	0	0	0	0	0	0	0	0	1	0	0	1	0	0	0	0	0	0	0	0	0
ENSG00000198914	44	124	52	123	0	3	2	7	0	0	2	0	4	4	9	9	14	48	21	74	6	4	6	3	0	0	1	0	1	2	1	4
ENSG00000239587	0	0	0	0	1	1	2	0	0	0	0	0	0	0	0	0	0	0	0	0	1	0	0	0	0	0	0	0	0	0	0	0
ENSG00000229743	0	1	2	3	28	32	21	28	0	0	0	0	0	0	0	0	0	1	0	4	46	30	53	23	0	0	0	0	0	0	0	0
ENSG00000224509	2	1	3	4	2	0	6	6	2	0	6	1	4	3	4	7	1	2	1	2	2	3	3	6	0	1	2	4	1	2	1	3
ENSG00000135972	1276	879	1053	970	682	701	581	818	541	387	1756	285	1574	1449	1484	1856	974	1322	1188	1228	1612	632	1235	578	454	794	432	1263	1487	1613	1637	2008
ENSG00000231851	21	22	18	17	5	12	9	13	12	10	45	13	16	20	13	21	6	10	8	29	19	8	14	19	9	11	7	34	15	33	12	22
ENSG00000226508	0	4	0	4	0	0	0	0	8	13	7	9	1	2	0	1	0	1	0	2	0	0	0	0	0	6	3	12	0	1	0	1
ENSG00000135973	10	4	4	1	1	7	0	7	1	4	7	0	3	1	1	0	0	0	0	5	1	1	1	1	0	0	2	2	0	0	0	0
ENSG00000272861	1	1	0	2	1	8	2	1	1	0	9	1	3	2	3	1	1	3	1	1	2	0	2	1	3	2	2	8	2	2	1	0
ENSG00000135966	1372	1009	1365	1110	1138	1053	836	1131	1119	880	4482	796	2878	2474	2900	3016	519	775	705	1202	1916	760	1345	704	1056	1641	1025	2968	2148	2655	2124	2752
ENSG00000235319	13	13	3	2	2	1	0	6	5	2	10	1	8	5	3	9	4	4	4	6	12	5	5	4	6	2	3	6	2	7	9	8
ENSG00000272994	178	143	143	151	149	173	114	159	135	137	501	87	193	190	211	244	107	75	107	226	293	130	195	141	110	164	116	240	141	217	149	336
ENSG00000135974	1093	770	992	809	731	711	612	778	669	577	2922	407	1082	998	1167	1251	614	687	678	771	1571	598	1010	619	567	867	725	1598	803	971	808	1140
ENSG00000115641	716	487	739	460	573	547	453	603	284	221	1195	203	642	684	854	990	452	788	994	802	1835	628	1279	597	252	421	211	727	733	1017	929	1105
ENSG00000238273	9	18	15	11	15	23	10	11	12	6	72	15	11	23	21	32	6	5	7	37	52	19	17	24	9	22	15	43	11	26	18	20
ENSG00000071051	993	771	914	755	790	789	521	780	560	452	1924	367	1965	1599	2056	2168	302	448	400	640	1194	482	808	398	510	827	531	1252	1427	1682	1534	1942
ENSG00000235522	10	3	30	3	11	3	8	9	15	3	29	1	20	24	20	21	5	4	2	19	13	9	9	11	7	9	8	39	14	29	28	31
ENSG00000234162	0	0	0	0	0	0	0	0	0	0	0	0	0	0	0	0	0	0	0	0	0	0	0	0	0	0	0	4	0	0	2	0
ENSG00000119147	1	7	1	4	1	1	0	3	1	1	3	0	3	3	1	1	3	5	1	5	2	1	2	0	1	0	1	0	0	4	0	1
ENSG00000115652	885	636	673	666	930	917	672	968	632	561	2072	376	1701	1579	1729	2059	502	784	646	781	2040	718	1444	689	625	950	456	1483	1409	1671	1483	1894
ENSG00000233339	4	6	8	7	2	3	3	3	3	5	11	2	7	7	7	9	4	0	2	9	8	2	11	7	3	5	1	14	4	9	9	8
ENSG00000273595	0	1	0	0	0	0	0	0	0	0	0	0	0	0	0	1	0	0	0	1	0	0	0	0	0	0	0	0	0	0	0	0
ENSG00000213295	0	0	0	0	0	0	0	0	0	0	0	0	0	0	0	0	1	0	0	0	0	0	0	0	0	0	0	0	0	0	0	0
ENSG00000224061	0	0	0	0	0	0	0	1	1	0	0	0	0	0	1	0	1	1	0	0	0	0	0	0	0	0	0	0	0	0	0	0
ENSG00000230353	0	0	0	0	0	0	0	0	0	0	3	0	0	0	0	0	0	0	0	0	0	0	0	0	0	0	0	0	0	0	0	0
ENSG00000233939	0	0	0	0	0	0	0	0	0	0	0	0	0	0	0	0	0	0	0	0	0	0	0	0	0	0	0	0	0	0	0	0
ENSG00000227513	0	0	0	0	0	0	0	0	0	0	0	0	0	0	0	0	0	0	0	0	0	0	0	0	0	0	0	0	0	0	0	0
ENSG00000229682	1	0	0	0	0	0	0	0	1	0	1	0	0	0	0	0	0	0	0	1	0	0	3	2	0	3	0	4	0	1	0	1
ENSG00000235486	0	0	0	1	0	0	0	0	0	0	0	0	0	0	0	0	0	1	0	0	1	0	0	0	0	0	0	0	0	0	0	0
ENSG00000240935	0	0	0	0	1	0	0	0	0	0	1	0	0	0	0	0	0	0	0	0	0	1	0	0	0	0	0	0	0	0	0	0
ENSG00000153165	12	14	28	17	11	9	5	9	10	6	37	14	21	18	14	10	14	19	20	19	18	5	9	7	11	14	0	28	27	18	9	38
ENSG00000237666	0	0	0	0	0	0	0	0	0	0	1	0	0	1	0	0	0	0	0	0	0	0	0	0	0	0	0	1	0	0	1	1
ENSG00000254126	0	0	0	0	0	0	0	0	0	0	4	3	4	3	2	7	0	0	0	0	0	0	0	0	2	1	0	5	3	6	2	7
ENSG00000231505	0	0	0	0	0	0	0	0	0	0	0	0	0	0	0	0	0	0	0	0	0	0	0	0	0	0	0	0	0	0	1	1
ENSG00000232001	0	0	0	0	0	0	0	0	0	0	0	0	1	0	0	0	0	0	0	0	0	0	0	0	0	0	0	0	0	0	1	0
ENSG00000214199	2	1	0	1	0	2	0	1	3	3	18	0	4	2	3	5	2	2	1	1	4	1	0	0	4	1	0	3	3	1	5	2
ENSG00000144057	0	0	0	0	0	0	0	0	0	0	1	0	0	0	0	0	0	0	0	0	0	0	1	0	0	0	0	2	0	0	2	0
ENSG00000238250	0	0	0	0	0	0	0	0	0	0	0	0	0	0	0	0	0	0	0	0	0	0	0	0	0	0	0	0	0	0	0	0
ENSG00000227294	0	0	0	0	0	0	0	0	0	0	0	0	0	0	0	0	0	0	0	0	0	0	0	0	0	0	0	0	0	0	0	0
ENSG00000229360	0	0	0	0	0	0	0	0	0	0	0	0	0	0	0	0	0	0	0	0	0	0	0	0	0	0	0	0	0	0	0	0
ENSG00000226905	0	0	0	0	0	0	0	0	0	0	0	0	0	0	0	0	0	0	0	0	0	0	0	0	0	0	0	0	0	0	0	0
ENSG00000229457	0	0	0	0	0	0	0	0	0	0	0	0	0	0	0	0	0	0	0	0	0	0	0	2	0	0	3	1	0	0	0	0
ENSG00000225588	0	0	0	0	0	0	0	3	0	0	0	0	0	0	0	0	0	0	0	0	0	0	0	0	0	0	0	0	0	0	0	0
ENSG00000237880	0	0	0	0	1	0	0	2	0	0	0	0	0	0	0	0	0	0	0	0	0	0	3	0	0	0	0	0	0	0	0	0
ENSG00000224568	0	0	0	0	0	0	1	0	0	0	0	0	0	0	1	0	0	0	0	0	0	0	0	0	0	1	2	0	0	0	0	0
ENSG00000232991	0	0	0	0	0	3	0	0	0	0	0	0	0	0	0	0	0	0	0	1	0	0	0	1	0	0	0	0	0	0	0	0
ENSG00000230651	0	0	0	0	0	0	0	0	0	0	0	0	0	0	0	0	0	0	0	0	0	0	0	0	0	0	1	0	0	0	0	0
ENSG00000196862	0	1	0	2	2	7	0	5	0	0	1	0	0	0	1	0	0	0	0	0	3	2	3	1	1	0	0	1	0	0	0	0
ENSG00000233494	0	0	0	0	0	0	0	0	0	0	0	0	0	0	0	0	0	0	0	3	0	0	0	0	1	0	0	0	0	0	0	0
ENSG00000227270	0	0	0	0	0	0	0	0	0	0	0	0	0	0	0	0	0	0	0	0	0	0	0	0	0	0	0	1	0	0	0	0
ENSG00000237745	0	0	0	0	0	0	0	0	0	0	0	0	0	0	0	0	0	0	0	0	0	0	0	0	0	0	0	0	0	0	0	0
ENSG00000235325	0	0	0	0	0	0	0	0	0	0	0	0	0	0	0	2	0	0	0	0	0	0	0	0	0	0	0	0	0	0	0	0
ENSG00000115665	0	0	0	0	0	0	0	0	0	0	0	0	0	0	0	0	0	0	0	0	0	2	0	0	0	0	0	0	0	0	0	0
ENSG00000231221	0	1	0	0	1	0	2	0	0	0	0	0	0	0	0	0	0	1	0	0	0	0	0	0	0	0	0	0	0	0	0	0
ENSG00000229890	0	0	0	0	0	0	0	0	0	0	0	0	0	0	0	0	0	0	0	0	0	0	0	0	0	0	0	0	0	0	0	0
ENSG00000225328	0	0	0	1	0	0	0	0	0	0	0	0	0	0	0	0	1	1	0	1	2	1	0	0	0	0	0	0	0	0	0	0
ENSG00000236877	0	0	0	0	0	0	0	0	0	0	0	0	0	0	0	0	0	0	0	0	0	0	0	0	0	0	0	0	0	0	0	0
ENSG00000196228	0	0	1	0	0	0	0	2	3	0	0	0	0	0	0	0	0	0	0	0	2	1	1	0	0	0	0	0	0	0	0	0
ENSG00000227597	0	0	0	0	1	0	0	5	0	0	0	0	0	0	0	0	0	0	0	0	0	0	0	0	0	0	0	1	0	0	0	0
ENSG00000198203	1	1	0	2	1	0	2	5	1	1	4	0	0	1	1	0	1	0	0	0	5	4	10	1	0	2	0	1	0	1	0	2
ENSG00000261209	0	0	0	0	0	0	0	0	0	0	0	0	0	1	0	0	0	0	0	0	0	0	0	0	0	0	0	0	0	0	0	0
ENSG00000237223	1	0	0	0	1	0	0	1	0	0	0	0	0	1	1	0	0	0	0	0	4	0	0	1	0	1	0	0	0	0	0	0
ENSG00000271070	0	0	0	0	3	0	0	0	0	0	0	0	0	0	0	0	0	0	0	0	0	0	0	1	0	0	0	0	0	0	0	0
ENSG00000198075	0	0	0	0	0	2	1	0	0	1	3	0	1	0	0	1	0	1	0	0	3	2	3	1	0	2	2	0	0	0	0	0
ENSG00000271284	0	0	2	1	0	0	0	0	0	0	0	0	0	0	0	0	0	0	0	2	0	0	0	0	0	0	0	1	0	0	0	0
ENSG00000135968	1977	1343	1530	1331	1142	1242	946	1314	1495	1400	6866	915	1158	1121	1174	1370	878	873	881	1373	2055	861	1331	916	1116	1522	2078	3817	669	1143	1005	1316
ENSG00000214184	18	21	29	20	44	32	20	40	15	17	50	4	12	16	15	22	7	9	13	14	48	19	35	14	6	16	14	18	6	12	11	9
ENSG00000169756	1799	1417	1491	1516	4687	4528	3795	4999	1631	1409	5593	1048	1801	1658	1909	2229	598	1257	900	1330	9759	3665	7432	3300	1613	2551	1104	4124	1417	1773	1411	1883
ENSG00000228763	15	5	4	6	33	20	12	19	8	5	26	6	2	8	4	2	4	3	3	10	31	11	11	3	10	6	1	11	4	8	0	5
ENSG00000233648	0	0	0	0	0	0	0	0	0	0	0	0	0	2	0	0	0	0	0	0	0	0	0	0	1	0	3	0	0	0	0	1
ENSG00000235644	0	0	0	0	0	0	0	0	0	0	0	0	0	0	0	0	0	0	0	0	0	0	0	0	0	0	0	0	0	0	0	0
ENSG00000153201	6625	4848	5799	4475	6015	6558	5055	7340	4325	3563	17608	2865	5183	4394	5782	5732	2993	3511	3554	5727	15471	6740	10630	5767	4730	7283	4544	13245	4278	5729	5811	6361
ENSG00000163006	961	634	741	596	866	878	738	935	323	233	810	191	310	286	390	363	862	1065	1157	1219	2192	954	1680	1046	300	418	124	613	331	409	466	460
ENSG00000227470	34	15	38	20	44	30	34	22	4	8	21	6	10	6	18	17	11	14	14	23	85	23	80	32	10	9	1	18	11	10	11	16
ENSG00000135960	10	7	5	9	3	7	8	6	0	1	5	0	5	3	4	13	1	2	1	4	11	4	8	2	3	6	0	2	33	58	52	85
ENSG00000277628	0	0	0	0	0	0	0	0	0	0	0	0	0	0	0	0	0	0	0	0	1	0	0	0	0	0	0	0	0	0	0	0
ENSG00000259863	0	4	0	1	78	107	63	77	0	0	0	0	0	0	0	0	0	2	0	1	121	54	88	39	0	0	0	0	0	1	1	1
ENSG00000172985	3	7	2	10	534	597	455	584	0	1	0	0	0	1	0	2	0	2	1	6	825	427	648	341	0	0	0	0	0	4	1	1
ENSG00000264934	0	0	0	0	0	2	1	2	0	0	0	0	0	0	0	0	0	0	0	1	1	0	2	1	0	0	0	0	0	0	0	0
ENSG00000228551	0	0	0	0	0	3	0	1	0	0	0	0	0	0	0	0	0	0	1	0	1	3	5	2	0	0	0	0	0	0	0	0
ENSG00000265965	0	0	0	0	1	0	0	1	0	0	0	0	0	0	0	0	0	0	0	0	0	0	0	0	0	0	0	0	0	0	0	0
ENSG00000279515	0	0	0	0	0	1	0	1	0	0	0	0	0	0	0	0	0	0	0	0	2	0	0	0	0	0	0	0	0	0	0	0
ENSG00000186522	0	0	0	0	0	0	0	0	3	0	0	1	0	1	1	3	0	0	0	0	0	0	0	0	0	0	0	3	2	2	0	1
ENSG00000237916	0	0	0	0	0	0	0	0	0	0	0	0	0	0	0	0	0	0	0	0	0	0	0	0	0	0	0	0	0	0	0	0
ENSG00000230696	0	0	0	0	0	0	0	0	0	0	0	0	0	0	0	0	0	0	0	0	0	0	0	0	0	0	0	0	0	0	0	0
ENSG00000198142	0	0	0	0	0	0	0	0	3	7	4	4	5	2	1	3	1	0	0	0	0	0	0	0	1	1	0	2	1	0	3	3
ENSG00000230603	0	0	0	0	0	0	0	0	0	0	4	0	0	0	0	0	0	0	0	0	0	0	0	0	0	0	1	0	0	0	0	0
ENSG00000231099	1	0	0	0	0	0	0	0	0	0	0	0	0	0	0	0	0	0	0	0	0	0	0	0	0	0	0	0	0	0	0	0
ENSG00000233307	0	0	0	0	0	0	0	0	0	0	1	0	0	1	0	0	0	0	0	0	0	0	0	0	0	0	0	0	0	0	0	0
ENSG00000233707	0	0	0	0	0	0	0	0	0	0	0	0	0	0	0	0	1	0	1	0	0	0	0	0	0	0	0	2	0	0	0	0
ENSG00000282033	0	0	0	0	0	0	0	0	0	0	0	0	0	0	0	0	0	0	0	0	0	0	0	0	0	0	0	2	0	0	0	0
ENSG00000015568	0	0	0	1	0	0	0	1	0	0	0	0	0	0	0	0	0	0	0	0	0	0	0	0	0	1	0	2	0	1	0	0
ENSG00000256977	0	0	0	0	0	0	0	1	0	0	0	0	0	0	0	0	0	0	0	0	1	1	0	0	0	0	0	0	0	0	0	0
ENSG00000186148	0	0	0	0	0	1	1	0	0	0	0	0	0	0	0	0	0	0	0	0	1	0	1	0	0	0	0	0	0	0	0	0
ENSG00000283684	0	0	0	0	0	0	0	2	0	0	0	0	0	0	0	0	0	0	0	0	0	0	0	0	0	0	0	0	0	0	0	0
ENSG00000240069	0	0	0	0	0	0	0	0	0	0	0	0	0	0	0	0	0	0	0	0	0	0	0	0	0	0	0	0	0	0	0	0
ENSG00000225108	0	0	0	0	0	0	0	0	0	0	0	0	0	0	0	0	0	0	0	0	0	0	0	0	0	0	0	0	0	0	0	0
ENSG00000204588	0	2	0	0	0	0	0	0	0	0	0	0	0	0	0	0	0	0	0	0	0	0	0	0	0	0	0	0	0	0	0	0
ENSG00000235721	0	0	0	0	0	0	0	0	0	0	0	0	0	0	0	0	0	0	0	0	0	0	0	0	0	0	0	0	0	0	0	0
ENSG00000229730	0	0	0	0	0	0	0	0	0	0	0	0	0	0	0	0	0	0	0	0	0	0	0	0	0	0	0	0	0	0	0	0
ENSG00000283283	0	0	0	0	0	0	0	0	0	0	0	0	0	0	0	0	0	0	0	0	0	0	0	0	0	0	0	0	0	0	0	0
ENSG00000283331	0	0	0	0	0	0	0	0	0	0	0	0	0	0	0	0	0	0	0	0	0	0	0	0	0	0	0	0	0	0	0	0
ENSG00000265682	0	0	0	0	0	0	0	0	0	0	0	0	0	0	0	0	0	0	0	0	0	0	0	0	0	0	0	0	0	0	0	0
ENSG00000144063	0	0	0	0	1	0	0	0	0	0	0	0	0	0	0	0	0	0	0	0	0	0	0	0	0	0	0	0	0	0	0	0
ENSG00000264979	0	0	0	0	0	0	0	0	0	0	0	0	0	0	0	0	0	0	0	0	0	0	0	0	0	0	0	0	0	0	0	0
ENSG00000144061	76	18	40	49	3	11	5	6	16	9	38	4	77	72	76	78	40	69	71	58	4	3	6	4	2	5	9	26	50	63	65	90
ENSG00000233370	0	1	0	0	0	0	0	0	0	0	0	0	0	0	0	0	2	1	0	1	0	0	0	0	0	0	0	0	0	1	0	0
ENSG00000175701	224	161	185	185	170	211	119	201	148	85	365	98	174	157	156	176	226	243	223	260	364	145	309	212	137	190	79	218	257	167	228	219
ENSG00000261760	0	0	0	0	0	0	2	1	0	0	0	0	0	0	0	0	0	0	0	0	0	0	0	0	0	0	0	0	0	0	0	2
ENSG00000282304	0	0	0	0	0	0	0	0	0	0	0	0	0	0	0	0	0	0	0	0	0	0	0	0	0	0	0	0	0	0	0	0
ENSG00000282785	0	0	0	0	0	0	0	0	0	0	0	0	0	0	0	0	0	0	0	0	0	0	0	0	0	0	0	0	0	0	0	0
ENSG00000263881	0	0	0	0	0	0	0	0	0	0	0	0	0	0	0	0	0	0	0	0	0	0	0	0	0	0	0	0	0	0	0	0
ENSG00000283167	0	0	0	0	0	0	0	1	0	0	0	0	0	0	0	0	0	0	0	0	0	0	0	0	0	0	0	0	0	0	0	0
ENSG00000226991	0	0	0	0	0	0	0	0	0	0	0	0	0	0	0	0	0	0	0	0	0	0	0	0	0	0	0	0	0	0	0	0
ENSG00000230650	0	0	0	0	0	0	0	0	0	0	0	0	0	0	0	0	0	0	0	0	0	0	0	0	0	0	0	0	0	0	0	0
ENSG00000175772	0	0	0	0	0	0	0	0	0	1	0	1	0	0	0	0	0	0	0	0	0	0	1	0	0	0	0	1	0	0	0	0
ENSG00000226065	0	0	0	0	0	0	0	0	0	0	0	0	0	0	0	0	0	0	0	0	0	0	0	0	0	0	0	0	0	0	0	0
ENSG00000230596	0	0	0	0	0	0	0	0	0	0	0	0	0	0	0	0	0	0	0	0	0	0	0	0	0	0	0	0	0	0	0	0
ENSG00000273471	0	0	0	0	2	0	0	0	0	0	0	0	0	0	0	0	0	0	0	0	0	0	0	0	0	0	0	0	0	0	0	0
ENSG00000184115	0	0	0	0	1	0	1	0	0	0	0	0	0	0	0	0	0	0	0	0	0	0	0	0	0	0	0	0	0	0	0	0
ENSG00000257207	0	0	0	0	0	0	6	2	0	0	0	0	0	0	0	0	0	0	0	0	0	1	3	0	0	0	0	0	0	0	0	0
ENSG00000256671	0	0	0	0	0	0	0	0	0	0	0	0	0	0	0	0	0	0	0	0	0	0	0	0	0	0	0	0	0	0	0	0
ENSG00000227574	0	0	0	0	0	0	0	0	0	0	0	0	0	0	0	0	0	0	0	0	0	0	0	0	0	0	0	0	0	0	0	0
ENSG00000231583	0	0	0	0	0	0	0	0	0	0	0	0	0	0	0	0	0	0	0	0	0	0	0	0	0	0	0	0	0	0	0	0
ENSG00000183054	0	0	0	0	4	1	0	0	0	0	0	0	1	0	0	0	0	0	0	0	1	1	1	0	0	0	0	0	0	0	0	0
ENSG00000231536	0	0	0	0	2	1	1	2	0	0	0	0	0	1	0	1	0	0	0	0	0	0	0	0	0	0	0	0	0	0	0	0
ENSG00000216867	12	9	11	6	19	13	10	20	39	21	87	16	3	4	9	5	5	3	2	13	23	14	9	8	31	39	9	70	4	7	4	9
ENSG00000169679	5327	3712	4496	3550	4832	4868	3354	5137	3918	3279	9680	2396	2295	2178	2333	2643	3197	4695	4198	3338	7977	2923	5699	2654	4333	6514	1293	8582	1998	2165	2307	2497
ENSG00000235881	0	0	0	0	0	0	0	0	1	0	2	0	0	0	0	0	0	0	0	0	0	0	0	0	0	0	0	0	0	0	0	1
ENSG00000153093	6	10	20	7	10	7	8	15	3	4	7	3	297	279	357	337	9	8	5	17	9	3	14	12	2	1	6	9	210	258	302	309
ENSG00000236330	1	1	0	0	1	2	0	0	0	0	0	0	2	0	2	3	0	0	0	1	0	1	0	0	0	0	0	1	0	8	4	2
ENSG00000204581	0	1	0	0	0	2	4	3	0	1	6	0	41	60	52	57	0	0	0	0	5	1	3	0	0	3	4	0	7	51	24	42
ENSG00000153094	1052	1263	957	1264	593	662	444	650	1331	1214	8065	928	4739	4212	4523	5183	250	581	354	976	911	383	681	333	1526	2165	3132	5630	3216	4591	3495	4975
ENSG00000230499	0	3	3	1	9	11	17	13	31	19	43	6	2	5	2	4	2	1	1	4	18	10	8	8	19	16	6	34	3	2	7	6
ENSG00000172965	179	171	148	190	657	672	586	706	1065	870	2729	612	363	323	382	453	247	328	259	285	1444	593	1032	644	807	1277	429	1971	367	370	473	535
ENSG00000227992	6	2	2	4	18	23	20	28	29	42	83	16	10	4	6	11	0	3	0	6	40	10	24	15	29	51	8	68	4	4	2	12
ENSG00000271590	6	5	1	5	21	29	21	25	65	53	136	36	6	4	6	4	2	3	0	10	38	24	18	18	33	57	24	91	3	19	5	9
ENSG00000229118	2	1	3	0	9	7	4	5	4	4	21	2	0	1	1	2	2	0	0	1	8	3	2	4	5	6	2	18	0	1	3	6
ENSG00000270190	0	2	0	1	6	7	1	6	10	15	42	8	1	3	2	3	0	0	0	5	16	5	14	6	12	11	4	46	2	3	5	9
ENSG00000226928	0	1	2	0	8	5	6	10	24	12	45	5	2	7	4	3	0	0	0	3	12	3	6	9	19	12	6	36	1	1	6	6
ENSG00000266139	1	0	0	0	0	3	3	0	0	3	6	0	0	1	1	1	0	0	0	0	5	2	0	4	4	3	1	7	0	2	1	1
ENSG00000279191	7	4	10	7	31	29	53	44	81	56	220	31	14	16	12	16	4	2	6	12	63	47	52	65	83	91	35	204	4	24	21	32
ENSG00000223973	0	0	1	1	1	5	1	8	5	8	17	2	0	1	4	1	0	0	0	1	1	1	2	2	2	12	2	17	0	5	2	3
ENSG00000280878	0	0	1	0	1	0	0	0	0	0	2	0	0	1	0	0	0	0	0	0	2	1	1	0	0	0	1	1	1	2	0	0
ENSG00000240350	4	0	2	0	2	6	2	6	5	6	16	2	3	1	3	3	1	2	0	1	12	9	4	8	10	9	4	33	1	1	1	4
ENSG00000224959	3	4	4	5	11	8	7	20	12	14	51	10	1	8	3	7	7	0	1	12	23	8	18	17	20	21	7	66	0	5	9	16
ENSG00000271635	0	0	0	0	0	0	0	1	0	0	0	0	0	0	0	0	0	0	0	0	2	0	0	0	0	0	1	2	0	0	0	1
ENSG00000225744	1	2	0	1	1	3	0	3	1	0	1	1	1	0	2	0	0	1	2	0	2	0	0	0	0	1	0	0	2	1	0	0
ENSG00000237583	0	0	1	0	0	0	0	0	0	0	0	0	1	1	0	0	2	1	0	0	1	0	0	0	0	0	0	0	0	3	0	0
ENSG00000227842	1	1	2	1	0	0	0	0	1	0	0	0	0	1	0	0	0	0	0	2	0	1	0	0	0	0	0	3	0	2	1	0
ENSG00000228615	1	3	4	4	2	0	1	2	3	0	4	1	5	0	2	2	1	0	0	3	2	3	2	5	0	4	0	1	1	6	0	4
ENSG00000153107	3250	2401	3139	2399	2871	2719	2385	2941	2508	2098	7396	1346	3035	2488	3106	3123	1339	2186	1848	2652	5998	2355	4723	2212	2597	3874	1444	5362	2121	2739	2547	2843
ENSG00000266063	5	0	1	0	2	1	3	0	1	0	3	0	0	1	0	0	0	1	0	1	1	1	1	3	3	0	2	2	0	2	1	2
ENSG00000230958	5	0	4	3	1	1	2	2	0	1	5	0	6	0	0	1	0	3	0	0	4	3	0	3	4	0	6	1	0	2	4	2
ENSG00000236307	13	10	6	6	5	7	5	5	11	11	37	2	9	6	9	13	7	12	9	18	10	1	8	1	12	9	13	10	6	12	10	8
ENSG00000153208	20	34	33	32	488	586	419	542	37	47	304	49	10	16	16	33	10	19	15	29	859	339	536	347	42	62	67	178	7	14	16	27
ENSG00000240183	1	0	2	0	0	5	0	0	0	0	0	0	0	0	0	0	0	0	0	1	1	1	1	0	0	0	0	1	0	0	0	0
ENSG00000225496	0	0	0	0	1	0	0	0	0	0	0	0	0	0	0	0	0	0	0	0	3	0	3	3	0	0	0	2	0	0	0	0
ENSG00000232277	0	0	0	0	0	0	3	1	0	0	0	0	0	0	0	0	0	0	0	0	2	0	1	0	0	1	0	0	0	0	0	0
ENSG00000153214	957	707	831	652	1104	1114	867	1209	924	738	3198	539	917	854	1015	1030	245	279	245	509	1767	730	1124	748	794	1155	794	2193	561	857	878	983
ENSG00000144152	44	15	29	13	24	28	8	17	2	0	4	0	4	2	7	7	10	11	16	37	28	13	22	16	0	0	4	6	4	3	1	6
ENSG00000278962	4	4	5	1	0	1	2	1	0	0	0	0	0	1	0	5	1	3	1	4	3	2	1	2	0	0	0	0	0	1	0	0
ENSG00000144161	1402	948	1212	975	946	930	848	1045	809	706	2812	390	2023	1707	2442	2297	911	1055	1096	1335	2248	948	1628	908	709	1001	621	1591	1700	2092	2365	2368
ENSG00000188177	1319	1070	1145	1143	382	411	273	498	517	427	1900	230	386	406	261	460	300	381	271	522	410	202	310	192	303	345	604	1193	51	97	85	114
ENSG00000235258	8	6	19	10	4	5	2	6	7	4	22	1	3	4	3	7	1	1	1	11	1	1	5	2	1	5	7	10	1	2	0	1
ENSG00000236555	0	0	0	0	0	0	0	0	0	0	0	0	22	14	18	15	0	0	0	0	0	0	0	0	0	0	0	0	13	21	12	21
ENSG00000169629	145	128	118	120	152	138	112	153	77	88	350	65	254	265	323	316	58	93	77	117	290	126	183	131	84	150	115	243	225	268	265	319
ENSG00000283698	0	0	0	0	5	2	2	3	1	0	3	2	2	1	5	9	0	0	0	0	0	0	0	2	0	5	3	1	0	3	1	1
ENSG00000114999	2039	1615	1635	1558	1382	1402	1000	1476	1079	885	3664	694	1177	1121	1193	1425	662	904	796	1526	2256	975	1562	967	1092	1664	700	2769	780	1141	856	1279
ENSG00000125630	1090	813	1129	913	1319	1379	1092	1455	1944	1504	6473	1115	3180	2620	3222	3207	856	1635	1396	1756	2965	1297	2388	1119	1937	3200	1239	4405	2935	3125	3164	3348
ENSG00000207383	6	4	7	6	8	8	9	9	18	8	92	11	19	19	14	36	6	10	8	11	15	8	9	5	15	17	14	41	25	27	11	19
ENSG00000125611	0	0	0	0	0	0	1	0	333	253	1202	256	377	349	319	459	1	0	2	0	0	0	0	0	275	510	241	733	349	391	407	382
ENSG00000243389	0	1	0	0	0	2	0	0	5	3	21	2	0	2	3	3	0	3	1	1	0	0	0	0	0	1	4	6	2	2	1	2
ENSG00000228251	0	0	0	0	0	0	0	0	10	9	38	5	3	5	6	7	0	0	0	0	0	0	0	0	6	10	7	27	3	10	4	3
ENSG00000231747	1	2	2	1	0	0	0	3	10	1	11	1	0	2	2	2	2	3	6	1	1	0	0	1	3	6	5	1	1	2	0	1
ENSG00000237753	74	73	88	65	76	63	71	69	61	49	332	55	93	95	119	125	32	27	41	41	108	41	78	30	52	70	70	193	51	74	68	77
ENSG00000144136	2614	1867	2500	1900	2807	2818	2419	3104	4317	3728	22731	3142	3042	2818	3637	3929	1001	1401	1555	2298	5711	2298	4082	2257	4874	6995	6360	17348	2930	4637	3700	5113
ENSG00000144130	13	10	12	2	8	6	6	7	20	9	56	3	23	21	26	28	4	3	6	5	11	2	2	8	19	13	25	72	14	15	24	38
ENSG00000169607	1890	1298	1732	1335	1312	1500	967	1544	1545	1310	5024	1007	1074	1096	1284	1449	1311	1705	1494	1261	2848	1043	1797	989	1831	2653	653	4463	802	985	1038	1241

ENSG00000115008	1	0	2	6	5	8	7	10	6	11	45	10	9	11	22	17	1	1	1	3	13	7	9	8	15	16	5	63	6	11	11	11
ENSG00000125538	1	4	1	6	1	11	1	14	8	2	48	4	5	4	13	8	0	0	3	4	7	6	3	3	7	10	8	43	7	7	3	13
ENSG00000280228	2	3	5	3	6	8	8	6	4	5	42	4	8	6	13	8	0	0	0	1	2	1	5	4	4	9	8	61	2	8	5	11
ENSG00000180152	0	0	1	0	4	0	0	1	4	3	24	1	1	4	2	2	1	0	2	2	4	1	2	2	3	6	0	15	0	0	1	3
ENSG00000125571	1	0	2	1	1	4	0	1	0	2	11	1	6	5	3	4	1	0	2	1	0	2	0	4	1	4	0	10	0	3	0	6
ENSG00000227368	0	1	0	0	1	0	0	0	0	0	4	0	0	3	2	3	0	0	0	0	0	0	0	0	2	0	0	2	0	1	0	2
ENSG00000136688	0	2	0	2	1	0	0	0	0	0	0	0	0	0	0	3	0	0	0	2	0	0	0	0	0	0	0	1	0	0	0	0
ENSG00000236124	0	0	0	1	0	0	0	0	0	0	1	0	0	0	0	0	0	0	0	0	0	0	0	1	0	0	0	0	0	0	0	0
ENSG00000232090	0	0	0	0	0	0	0	0	0	0	0	0	0	0	0	0	0	0	0	0	0	0	0	0	0	0	0	0	0	0	0	0
ENSG00000136694	19	6	19	16	5	3	1	5	0	0	2	1	5	6	6	5	15	12	13	16	8	4	8	6	0	0	0	1	6	6	1	10
ENSG00000136696	0	0	0	0	0	0	0	0	0	0	0	0	0	0	0	0	0	0	0	0	0	0	0	0	0	0	0	0	0	0	0	0
ENSG00000136695	0	0	0	0	0	0	0	0	0	0	0	0	0	0	0	0	0	0	0	0	0	0	0	0	0	0	0	0	0	0	0	0
ENSG00000136697	0	0	0	0	0	0	0	0	0	0	0	0	0	0	0	0	0	0	0	0	0	0	0	0	0	0	0	0	0	0	0	0
ENSG00000201805	0	0	0	0	0	0	0	0	0	0	0	0	0	0	0	0	0	0	0	0	0	0	0	0	0	0	0	0	0	0	0	0
ENSG00000136689	0	0	0	0	1	0	0	0	0	0	1	0	10	11	21	25	0	1	0	0	0	0	0	0	0	0	0	0	0	0	4	2
ENSG00000125637	4204	2883	3879	2755	4481	4802	3086	4925	10925	9016	38421	7179	3535	3058	3433	4157	774	846	1127	1652	5569	2616	3815	2234	6193	10761	9018	21068	2193	2579	2120	2836
ENSG00000234174	18	17	17	18	13	25	15	21	30	14	68	15	15	12	10	8	9	1	2	7	23	16	19	16	15	21	24	91	7	8	12	13
ENSG00000189223	9	4	6	2	175	209	130	231	1164	1095	4728	760	1146	1073	1129	1459	14	3	10	10	226	129	169	117	731	1087	994	2271	622	1082	736	1247
ENSG00000125618	6	1	3	1	7	2	2	8	270	198	1012	219	251	240	218	256	1	2	3	2	8	1	3	0	184	233	202	551	71	168	128	217
ENSG00000231292	0	0	0	0	0	0	0	2	0	0	0	0	0	0	0	0	0	0	0	0	0	0	0	0	0	0	0	1	0	0	0	0
ENSG00000234997	0	0	0	0	0	0	0	1	0	0	1	1	0	0	0	0	0	0	0	0	0	0	0	0	1	0	0	0	0	0	0	0
ENSG00000272563	0	0	0	0	0	0	0	0	0	2	1	0	5	0	0	1	0	1	2	0	0	0	1	0	0	0	2	1	0	0	0	0
ENSG00000136682	1119	743	932	848	1141	1197	936	1366	709	535	2211	350	1057	860	1203	1133	805	892	725	1155	2218	928	1460	1027	655	885	421	1365	749	1167	1122	1251
ENSG00000184492	18	12	12	7	30	18	23	18	4	8	47	5	11	11	21	10	4	8	4	10	48	34	29	21	5	16	9	16	8	17	7	24
ENSG00000234148	0	0	0	0	1	2	1	0	0	0	2	1	0	0	0	0	0	0	0	0	0	2	0	0	0	0	4	0	0	0	0	0
ENSG00000231943	1	0	0	0	0	1	1	0	0	0	2	1	0	0	0	0	0	0	0	0	0	0	0	0	0	0	0	0	0	0	0	0
ENSG00000225398	2	4	2	2	1	3	3	1	2	0	8	0	2	3	1	3	0	0	1	9	2	0	3	2	1	3	1	6	0	0	0	0
ENSG00000226516	0	0	6	1	0	1	0	0	1	1	0	0	0	0	0	0	1	0	0	0	1	0	0	1	0	0	0	3	0	1	1	0
ENSG00000221055	14	12	16	11	18	19	11	11	1	2	10	1	0	4	1	6	4	0	1	4	12	10	16	7	0	0	1	3	0	0	2	0
ENSG00000146556	182	123	202	177	208	246	174	272	240	174	922	171	149	136	112	173	90	73	77	160	366	212	229	180	127	202	233	407	51	131	61	137
ENSG00000236397	81	61	97	77	156	152	140	193	28	28	119	26	42	29	42	70	42	39	37	78	215	100	151	99	20	42	15	83	31	60	24	70
ENSG00000279267	37	15	20	22	31	29	41	41	21	26	121	11	82	80	62	77	7	7	9	29	54	21	34	21	17	15	23	45	29	50	25	51
ENSG00000240356	198	125	170	151	114	137	102	149	105	60	327	60	307	262	283	300	218	198	211	194	214	97	246	125	70	83	65	177	255	266	340	281
ENSG00000144134	485	373	427	365	144	173	153	156	335	312	1515	225	399	416	360	485	118	204	141	341	238	133	165	119	187	334	348	709	204	391	199	403
ENSG00000144158	3	4	9	3	2	1	1	2	0	3	3	0	2	1	1	4	1	5	2	3	3	1	6	3	1	2	0	3	0	1	1	4
ENSG00000175509	0	0	0	0	3	2	1	0	0	0	0	0	0	0	0	0	1	0	0	0	1	0	0	0	0	0	0	0	0	0	0	0
ENSG00000233479	0	1	0	0	0	0	1	0	0	0	0	0	0	0	0	0	0	0	0	1	0	1	0	0	0	0	0	0	3	0	0	0
ENSG00000227359	2	3	2	0	11	11	5	6	1	4	10	2	4	2	1	3	0	4	0	0	21	8	5	10	5	2	1	10	4	5	9	4
ENSG00000202427	0	0	0	0	0	0	0	0	0	0	0	0	0	0	0	0	1	0	0	0	0	0	0	0	0	0	0	0	0	0	0	0
ENSG00000115084	785	469	568	532	1493	1506	1085	1544	563	520	2130	376	969	929	1084	1098	257	288	244	456	2437	1036	1662	1067	481	690	521	1337	713	1005	931	1080
ENSG00000265429	1	2	2	1	4	5	6	5	0	2	6	1	1	0	1	2	0	0	0	0	0	1	3	2	0	5	2	5	0	2	1	1
ENSG00000244063	21	12	25	19	2	3	2	3	11	14	50	2	19	16	12	26	21	11	11	17	12	3	4	6	8	5	12	22	9	20	15	17
ENSG00000228857	63	39	53	59	18	19	13	21	21	15	77	13	55	64	63	46	54	51	39	67	42	11	29	17	17	18	14	42	20	39	25	55
ENSG00000270019	53	37	64	41	24	34	10	33	40	26	207	31	61	37	56	55	25	27	13	57	41	25	45	40	22	43	28	96	23	51	48	56
ENSG00000115091	10790	6212	9492	6371	8020	7922	6591	8442	9469	6982	38752	5795	9047	8014	10821	10573	9165	9309	12427	10515	22895	8982	16233	8392	11007	15121	8104	24509	9578	10237	12011	12656
ENSG00000279957	7	4	7	2	0	2	1	1	7	7	17	3	2	1	5	3	3	0	1	5	6	4	5	6	16	8	9	24	6	4	4	9
ENSG00000234199	0	0	0	0	0	0	0	0	0	0	0	0	1	0	0	0	1	0	2	1	6	0	0	1	1	0	0	0	0	0	0	2
ENSG00000243179	0	0	1	0	4	0	3	0	0	0	3	0	0	0	0	0	0	0	0	2	2	1	0	0	1	2	1	2	0	1	3	1
ENSG00000212182	0	0	0	0	0	1	0	0	0	0	0	0	0	0	0	0	0	0	0	0	0	0	0	0	0	0	0	0	0	0	0	0
ENSG00000235242	0	0	0	0	0	0	0	0	0	0	0	0	0	0	0	0	0	0	0	0	0	0	0	0	0	0	0	0	0	0	0	0
ENSG00000231926	0	0	0	0	0	0	0	0	0	0	0	0	0	0	0	0	0	0	0	0	1	0	0	0	0	0	0	0	0	0	0	0
ENSG00000222923	0	0	0	0	0	0	0	0	0	0	0	0	0	0	0	0	0	0	0	0	0	0	0	0	0	0	0	0	0	0	0	0
ENSG00000175497	95	89	71	86	0	0	0	0	0	0	3	0	1	0	0	0	34	106	55	74	0	0	0	0	0	0	0	0	1	0	0	0
ENSG00000231538	0	0	0	0	0	0	0	0	0	0	0	0	0	0	0	0	0	0	0	0	0	0	0	0	0	0	0	0	0	0	0	0
ENSG00000235717	0	0	0	0	0	0	0	0	0	0	0	0	0	0	0	0	0	0	0	0	0	0	0	0	0	0	0	0	0	0	0	0
ENSG00000235026	24	16	28	25	0	0	0	0	0	0	0	0	0	0	0	0	15	33	31	28	0	0	0	0	0	0	0	0	0	0	0	0
ENSG00000230391	0	3	0	0	0	0	0	0	0	0	0	0	0	0	0	0	0	0	0	0	0	0	0	0	0	0	0	0	0	0	0	0
ENSG00000271667	0	0	0	0	0	0	0	0	0	0	0	0	0	0	0	0	0	0	0	0	0	0	0	0	0	0	0	0	0	0	0	0
ENSG00000224869	0	0	0	0	0	0	0	0	0	0	0	0	0	0	0	0	0	0	0	0	0	0	0	0	0	0	0	0	0	0	0	0
ENSG00000226341	0	0	0	0	0	0	0	0	0	0	0	0	0	0	0	0	0	0	0	0	0	0	0	0	0	0	0	0	0	0	0	0
ENSG00000235871	0	0	0	0	0	0	0	0	0	0	0	0	0	0	0	0	0	0	0	0	0	0	0	0	0	0	0	0	0	0	0	0
ENSG00000230158	0	0	0	0	0	0	0	0	0	0	0	0	0	0	0	0	0	0	0	0	0	0	0	0	0	0	0	0	0	0	0	0
ENSG00000230950	0	0	0	0	0	0	0	0	0	0	0	0	0	0	0	0	0	0	0	0	0	0	0	0	0	0	0	0	0	0	0	0
ENSG00000228898	0	0	0	0	0	0	0	0	0	0	0	0	0	0	0	0	0	0	0	0	0	0	0	0	0	0	0	0	0	0	0	0
ENSG00000239185	0	0	0	0	0	0	0	0	0	0	0	0	0	0	0	0	0	0	0	0	0	0	0	0	0	0	0	0	0	0	0	0
ENSG00000227291	0	0	0	0	0	0	0	0	0	0	0	0	0	0	1	0	0	0	0	0	0	0	0	0	0	1	0	0	0	0	1	0
ENSG00000238207	0	0	0	0	0	0	0	0	0	0	0	1	0	0	0	0	0	1	0	0	0	0	0	0	0	0	1	0	0	0	0	0
ENSG00000088205	4460	2822	3713	2937	3326	3553	3095	3842	3208	2348	9679	1665	4869	4354	5310	5315	4690	6466	5660	5112	9425	4094	6938	3903	3164	4554	2309	6901	5506	5601	6803	6511
ENSG00000236255	310	266	258	255	187	225	153	246	69	56	225	27	304	295	316	330	82	81	68	239	293	178	125	166	33	59	66	124	84	290	102	260
ENSG00000125631	15	8	17	14	17	20	6	12	5	2	17	4	14	18	10	10	0	5	3	9	18	10	13	15	7	10	7	22	2	16	16	16
ENSG00000125633	2938	2279	2412	2282	2573	2725	1965	2669	1426	1156	4320	872	2510	2399	2330	3083	940	1069	1071	1935	5114	2152	3454	2097	1235	1867	1097	3334	1202	2011	1312	2124
ENSG00000224967	4	2	5	3	5	4	5	2	4	2	6	1	2	1	3	2	2	2	2	2	11	3	2	4	2	4	6	10	0	5	1	8
ENSG00000235066	12	8	11	10	7	12	7	15	7	5	9	0	20	11	8	14	9	3	4	10	20	7	6	20	9	11	2	23	7	18	9	8
ENSG00000272895	1	5	1	2	0	4	0	3	0	0	2	2	1	1	3	0	0	0	0	4	6	1	2	3	5	2	0	5	0	4	5	2
ENSG00000279227	66	46	53	54	34	38	32	36	11	4	30	6	23	26	27	34	27	33	39	43	54	7	22	34	10	16	8	23	10	18	9	23
ENSG00000243510	0	0	1	0	0	0	0	0	0	0	1	0	1	0	0	0	0	0	0	1	0	0	0	0	0	0	1	3	0	0	0	0
ENSG00000125629	1309	838	806	819	2381	2177	1863	2625	1010	855	1737	412	927	1072	781	1215	733	848	607	667	2300	906	1450	888	468	611	422	1089	423	592	500	711
ENSG00000226856	49	38	45	33	18	13	14	17	8	6	18	4	19	32	19	25	31	25	37	45	29	12	21	12	5	8	7	9	7	15	17	21
ENSG00000258910	0	0	0	0	0	0	0	0	0	0	0	0	0	0	0	0	0	0	0	0	0	0	0	0	0	0	0	0	0	0	0	0
ENSG00000163064	0	0	0	0	0	0	0	0	0	0	0	1	0	0	0	0	0	0	0	0	0	0	0	0	0	0	0	0	0	0	0	0
ENSG00000019169	0	0	0	1	2	0	0	1	0	0	1	0	0	0	0	0	0	0	0	3	2	3	0	0	0	0	0	0	0	0	0	1
ENSG00000259094	0	0	0	0	0	0	0	0	0	0	0	0	0	0	0	0	0	0	0	0	0	0	0	0	0	0	0	0	0	0	0	0
ENSG00000144119	0	0	0	0	0	0	0	0	0	0	2	0	0	1	0	0	0	0	0	0	0	0	0	0	0	0	0	0	0	0	0	0
ENSG00000264833	0	0	0	0	0	0	0	0	0	0	0	0	0	0	0	0	0	0	0	0	0	0	0	0	0	0	0	0	0	0	0	0
ENSG00000115107	2	1	4	4	19	13	16	18	169	254	1555	261	165	254	162	296	0	2	0	3	10	11	7	5	200	439	168	990	100	207	79	157
ENSG00000229867	1	0	0	3	1	0	2	2	7	9	49	8	11	12	1	7	0	0	0	1	1	0	5	2	11	9	13	35	1	5	1	7
ENSG00000186132	183	117	142	147	113	93	80	106	59	49	320	46	137	125	142	168	165	207	196	211	222	94	139	100	66	91	83	178	129	99	133	132
ENSG00000155368	2729	2021	2030	2110	1929	2219	1414	2506	2169	1475	5755	1239	3264	3428	3320	4644	3330	4148	3326	3672	4254	1763	3038	2098	1866	2782	1028	4189	3743	3389	3868	4689
ENSG00000171227	0	0	0	0	0	1	3	0	1	3	0	0	0	0	0	0	0	0	0	0	0	0	5	0	0	0	2	2	0	1	0	0
ENSG00000080293	1	3	0	0	0	2	0	0	0	0	1	0	2	1	0	0	0	0	0	0	0	0	0	0	0	0	2	0	0	0	0	0
ENSG00000231013	0	0	0	0	0	0	0	1	0	0	0	0	0	0	0	0	0	0	0	0	0	0	0	0	0	0	0	0	0	0	4	0
ENSG00000163075	0	0	0	0	0	0	0	0	0	0	1	2	0	0	0	1	1	0	0	0	0	0	0	0	0	0	1	0	0	0	0	0
ENSG00000144120	390	299	251	302	221	240	141	226	242	168	755	149	509	536	525	612	246	401	227	314	312	142	284	124	213	296	146	461	496	547	441	576
ENSG00000229326	2	2	3	1	1	3	4	3	3	3	12	2	8	11	13	12	2	0	0	1	3	3	[illegible]	2	2	[illegible]	8	6	0	3	1	9
ENSG00000088179	1016	744	797	718	1041	1140	758	1060	2375	2021	11304	1551	1626	1639	1424	1964	461	753	496	899	2468	974	1641	1060	2340	3078	2957	7064	840	1408	929	1522
ENSG00000233741	0	0	0	0	0	0	0	0	1	0	3	0	0	0	0	1	0	0	0	0	2	0	1	0	0	0	2	0	0	2	0	1
ENSG00000115109	472	494	425	493	764	840	573	818	460	348	1679	292	466	527	548	779	155	296	225	413	1383	536	936	539	403	633	422	1111	315	535	471	660
ENSG00000224789	0	2	0	0	2	0	0	3	1	0	4	4	0	1	3	2	0	0	0	0	1	1	0	0	1	0	1	5	0	1	0	0
ENSG00000202046	0	0	0	0	0	0	0	0	0	0	0	0	0	0	0	0	0	0	0	0	1	0	0	0	1	1	0	3	0	0	1	0
ENSG00000236878	5	8	5	3	5	4	2	10	14	10	40	1	2	3	1	3	1	1	0	8	12	2	7	1	10	6	4	31	3	1	1	4
ENSG00000227788	7	5	2	9	8	4	3	10	19	7	40	5	5	12	3	6	2	1	1	10	8	0	7	7	12	5	6	26	1	4	6	8
ENSG00000233286	4	1	0	6	2	3	2	4	7	1	13	0	0	5	2	4	2	1	0	1	4	2	2	3	3	5	2	18	0	1	0	3
ENSG00000243104	2	0	4	0	0	2	5	5	11	0	26	2	0	2	4	2	1	0	0	0	2	2	0	1	4	6	4	11	1	4	0	4
ENSG00000234065	10	7	8	13	13	11	7	7	12	11	48	16	4	5	9	6	3	2	2	10	16	7	7	8	8	16	15	39	1	17	3	8
ENSG00000223549	6	9	17	12	17	15	16	34	19	14	102	11	15	15	10	11	1	1	2	6	26	14	10	10	18	16	14	44	2	13	3	13
ENSG00000226479	727	603	680	643	631	698	506	694	705	657	2790	529	1339	1316	1413	1675	253	404	423	693	1215	537	879	434	642	1037	528	1698	812	1160	822	1275
ENSG00000144118	241	308	300	325	1587	1520	1071	1654	958	789	3538	698	750	896	744	1248	91	172	195	217	2727	1127	1905	935	872	1282	749	2390	587	846	599	898
ENSG00000281676	0	0	0	0	1	1	1	0	0	1	3	0	1	0	0	0	0	0	0	0	5	2	0	0	1	1	0	4	0	0	0	0
ENSG00000235840	0	0	0	0	0	1	0	0	0	0	4	0	0	0	0	1	0	0	0	0	0	0	0	0	0	1	0	1	0	0	1	2
ENSG00000163083	0	0	0	0	3	0	1	0	0	1	1	1	0	0	0	2	0	0	0	0	3	7	3	1	3	2	1	0	0	0	0	0
ENSG00000280409	0	0	0	0	0	0	0	0	0	0	0	0	0	0	0	0	0	0	0	0	0	0	0	0	0	0	0	0	0	0	0	0
ENSG00000237614	0	0	0	0	0	0	0	0	0	0	0	0	0	0	0	0	0	0	0	0	0	0	0	0	0	0	0	0	0	0	0	0
ENSG00000232140	0	0	0	0	0	0	0	0	0	0	1	0	0	0	0	0	0	0	0	0	0	0	0	0	0	0	4	1	0	1	0	1
ENSG00000201006	0	0	0	0	0	0	0	0	0	0	0	0	0	0	0	0	0	0	0	0	0	0	0	0	0	0	0	0	0	0	0	0
ENSG00000229774	0	0	0	0	0	0	0	0	0	0	0	0	0	0	0	0	0	0	0	0	0	0	0	0	0	0	2	0	0	0	0	0
ENSG00000074047	0	0	0	0	5	2	4	4	0	0	2	0	0	0	0	1	0	0	0	0	3	1	3	3	0	0	0	0	0	0	0	1
ENSG00000271709	0	0	0	0	0	0	0	0	0	0	0	0	0	0	0	0	0	0	0	0	0	0	0	0	0	0	0	0	0	0	0	0
ENSG00000201584	0	0	0	0	0	0	0	0	0	0	0	0	0	0	0	0	0	0	0	0	0	0	0	0	0	0	0	0	0	0	0	0
ENSG00000234455	0	0	0	0	0	0	0	0	0	0	0	0	0	0	0	0	0	0	0	0	0	0	0	0	0	0	0	0	0	0	0	0
ENSG00000236145	0	0	0	0	0	0	0	0	0	0	0	0	0	0	0	0	0	0	0	0	0	0	0	0	0	0	0	0	0	0	0	0
ENSG00000115112	0	4	0	5	8	11	2	6	15	18	49	18	43	97	45	128	0	2	0	0	19	11	8	1	11	38	6	55	27	82	23	90
ENSG00000232723	0	0	0	0	0	0	0	0	0	0	0	0	0	0	0	0	0	0	0	0	0	0	0	0	0	0	0	0	0	0	0	0
ENSG00000074054	2954	2139	2396	2169	2963	3071	2249	3068	2240	1858	8264	1381	2488	2343	2340	2955	584	674	599	1358	4333	1856	2954	1618	1747	2919	1900	5510	1227	1860	1571	2133
ENSG00000265451	8	3	10	9	7	7	2	2	2	8	28	3	0	6	7	7	3	6	1	8	21	9	10	6	3	3	4	10	1	4	12	7
ENSG00000264229	1	1	0	3	0	2	2	0	1	1	5	1	0	2	1	4	3	0	4	3	6	7	5	0	1	1	5	6	7	12	4	3
ENSG00000222467	2	1	2	4	1	3	1	0	0	1	4	0	0	0	0	2	1	0	1	2	4	0	0	1	4	2	1	12	4	0	2	4
ENSG00000236859	201	168	191	158	159	176	168	198	212	162	739	143	182	205	184	272	91	91	84	92	287	113	195	108	171	230	146	447	104	167	165	234
ENSG00000230993	5	4	6	3	5	4	5	6	5	4	23	1	7	7	7	8	0	0	1	3	3	4	1	3	1	3	5	15	2	4	2	3
ENSG00000279721	3	2	4	2	0	2	1	5	2	2	1	0	11	6	1	2	0	0	0	0	1	0	1	0	0	3	0	3	0	3	0	1
ENSG00000213239	1	1	1	1	1	0	1	0	0	0	4	0	1	0	1	2	2	1	0	1	[illegible]	[illegible]	[illegible]	[illegible]	[illegible]	[illegible]	[illegible]	[illegible]	[illegible]	[illegible]	[illegible]	[illegible]
ENSG00000155438	1383	808	1040	824	1226	1153	979	1260	1343	1078	4732	942	1617	1437	2113	1947	2699	3452	2999	2732	4241	1520	2782	2095	1531	2136	998	2926	2689	2459	3264	3089
ENSG00000211460	3185	2168	2631	2179	3047	3115	2292	3127	2366	1761	7645	1382	3494	2824	3514	3716	2662	4373	3401	3069	7954	2889	5525	2728	2411	3530	1568	5064	3707	3555	3725	4137
ENSG00000270798	0	0	4	0	0	0	1	1	0	0	0	0	0	0	0	0	0	0	0	0	0	0	0	0	0	0	2	0	0	0	0	0
ENSG00000224655	0	0	1	1	0	0	0	1	0	0	0	0	0	0	0	2	0	0	1	0	2	1	0	0	0	0	1	0	1	0	0	0
ENSG00000237856	0	0	0	0	0	0	0	0	0	0	0	0	0	0	0	0	0	0	0	0	0	0	0	0	0	0	0	0	0	0	0	0
ENSG00000232740	0	0	0	0	0	0	0	0	0	0	0	0	0	0	0	0	0	0	0	0	0	0	0	0	0	0	0	0	0	0	0	0
ENSG00000226708	0	0	0	0	0	0	0	0	0	0	0	0	0	0	0	0	0	0	0	0	0	0	0	0	0	0	0	0	0	0	0	0
ENSG00000235640	0	0	0	0	3	6	10	7	0	0	0	0	0	0	0	0	0	0	0	0	4	5	0	2	0	0	0	0	0	0	0	0
ENSG00000227738	0	0	0	0	7	13	7	11	0	0	0	0	0	0	0	0	0	0	0	0	1	2	1	1	0	0	0	0	0	0	0	0
ENSG00000279874	0	0	0	0	0	0	0	0	0	0	0	0	0	0	0	0	0	0	0	0	0	0	0	0	0	0	0	0	0	0	0	0
ENSG00000223118	0	0	0	0	0	0	0	0	0	0	0	0	0	0	0	0	0	0	0	0	0	1	0	0	0	0	0	0	0	0	0	0
ENSG00000228400	0	0	0	0	1	0	0	0	0	0	0	0	0	0	0	0	0	0	0	0	0	0	0	0	0	0	0	0	0	0	0	0
ENSG00000155052	0	0	0	0	0	0	0	1	3	1	125	7	0	0	0	0	0	0	0	0	0	0	1	0	9	10	33	70	0	0	0	2
ENSG00000238046	0	0	0	0	0	0	0	0	0	0	0	0	0	0	0	0	0	0	0	0	0	0	0	0	0	0	0	0	0	0	0	0
ENSG00000270815	0	0	0	0	0	0	0	0	0	0	0	0	0	0	0	0	0	0	0	0	0	0	0	0	0	0	0	0	0	0	0	0
ENSG00000206726	0	0	0	0	0	0	0	0	0	0	0	0	0	0	0	0	0	0	0	0	0	0	0	0	0	0	0	0	0	0	0	0
ENSG00000235491	0	0	0	0	0	0	0	0	0	0	0	0	0	0	0	0	0	0	0	0	0	0	0	0	0	0	0	0	0	0	0	0
ENSG00000224410	0	0	0	0	0	0	0	0	0	0	0	0	0	0	0	0	0	0	0	0	0	0	0	0	0	0	0	0	0	0	0	0
ENSG00000226813	0	0	0	0	0	0	0	0	0	0	0	0	0	0	0	0	0	0	0	0	0	0	0	0	0	0	0	0	0	0	0	0
ENSG00000235774	0	0	0	0	0	0	0	0	0	0	0	0	1	0	0	0	0	0	0	0	0	0	0	0	0	0	0	0	0	0	0	0
ENSG00000236655	0	0	0	0	0	0	0	0	0	0	0	0	0	0	0	0	0	0	0	0	0	0	0	0	0	0	0	0	0	0	1	0
ENSG00000213236	1	0	2	0	0	2	0	2	0	1	9	0	1	2	1	4	2	2	3	0	3	1	1	0	0	0	1	4	0	2	1	2
ENSG00000136732	7382	6453	5636	6576	1676	1695	1162	1859	305	249	942	237	2418	2299	2595	2907	2396	5283	3363	4467	2636	1087	1931	959	166	298	173	607	1917	2041	2212	2388
ENSG00000235128	30	32	30	33	14	19	6	13	0	0	6	1	15	11	20	16	13	7	4	35	11	8	10	11	3	0	2	4	9	15	15	18
ENSG00000206963	2	0	0	2	0	1	0	0	0	0	0	0	0	0	0	0	0	0	0	0	0	0	0	0	0	0	0	0	0	0	0	0
ENSG00000237524	0	0	0	0	0	0	0	0	0	0	0	0	0	0	0	0	0	0	0	0	0	0	0	0	0	0	3	0	0	0	0	0
ENSG00000238788	0	0	0	0	0	0	0	0	0	0	0	0	0	0	0	0	0	0	0	0	0	0	0	0	0	0	0	0	0	0	0	0
ENSG00000260634	0	0	0	0	0	0	0	0	0	2	0	0	0	0	0	0	0	0	0	1	0	0	0	0	2	0	0	0	0	0	0	0
ENSG00000260163	0	1	0	0	0	1	0	0	0	1	9	0	1	0	0	0	1	0	0	0	1	0	0	0	0	0	3	0	0	0	0	1
ENSG00000136717	205	106	158	125	716	763	606	842	88	63	295	50	247	310	213	400	46	73	88	128	1059	506	820	443	32	50	73	125	98	118	83	135
ENSG00000237630	0	0	0	0	0	0	0	0	0	0	0	0	0	0	0	0	0	0	0	0	0	0	0	0	0	0	0	0	0	0	0	0
ENSG00000186684	0	0	0	0	2	8	3	5	1	1	7	0	0	1	0	0	0	0	1	0	1	1	1	1	0	1	0	4	0	0	0	1
ENSG00000226789	1	0	2	1	1	1	0	0	4	2	10	4	4	1	1	1	0	2	0	3	1	0	0	2	4	2	3	9	6	4	3	11
ENSG00000163161	2159	1492	1872	1582	2124	2202	1564	2246	1408	1165	5642	916	3340	2866	3734	4004	1021	1142	1209	1868	3545	1561	2411	1464	1181	1843	1323	3453	2567	3365	3012	3669
ENSG00000169967	3450	2444	2618	2286	2221	2169	1698	2253	1780	1512	8285	1272	3236	3170	3610	3806	1333	1740	1602	2746	4835	1862	3159	1997	2125	2926	2560	6257	2076	3221	2867	3566
ENSG00000200250	1	3	1	1	0	1	1	0	1	2	1	0	0	0	0	0	0	0	0	0	0	1	1	2	0	7	0	2	0	1	0	0
ENSG00000236682	36	28	39	40	25	31	10	19	18	19	102	12	36	42	36	57	50	56	63	57	62	13	35	32	27	25	24	67	33	29	44	32
ENSG00000115718	23	19	16	16	5	14	4	9	17	6	26	5	29	18	19	28	16	12	4	19	13	6	6	6	7	8	8	20	6	11	9	10
ENSG00000264075	4	0	0	1	0	0	0	0	0	0	0	0	0	0	0	0	0	0	0	0	0	0	2	0	1	0	1	2	0	0	1	0
ENSG00000163166	2888	2011	2491	2167	1714	1663	1315	1725	1301	1087	5399	959	3340	2844	3680	3806	1574	1831	1622	2117	3262	1355	2408	1181	1372	2211	1166	3842	2505	3060	2922	3566
ENSG00000231731	233	149	187	183	136	90	82	115	100	81	477	47	180	181	198	199	70	38	32	215	200	100	96	106	113	134	101	293	55	187	100	225
ENSG00000202429	7	1	1	0	0	4	0	1	1	1	11	2	3	9	8	4	0	0	0	1	0	0	1	1	2	3	2	3	2	3	2	7
ENSG00000169994	8191	7132	5747	7550	7	10	10	9	17	27	101	44	0	0	1	1	1930	2764	1230	5410	5	8	6	9	7	31	36	140	0	0	0	0
ENSG00000272789	263	239	181	220	1	0	0	1	0	0	3	2	0	0	0	0	67	51	33	238	0	0	0	1	0	1	9	10	0	0	0	0
ENSG00000072163	651	547	579	525	197	169	135	175	42	67	295	86	4	7	1	5	296	482	354	601	298	89	185	96	57	73	85	342	3	1	0	1
ENSG00000144230	7	11	7	5	0	0	1	1	2	4	22	4	0	0	0	0	1	0	2	28	2	0	0	0	3	6	10	52	0	0	0	0
ENSG00000136709	3273	2232	2773	2193	2103	2037	1478	2179	2201	1741	7179	1203	3790	3680	3704	4493	1491	1928	1787	2565	3960	1742	2973	1728	1916	2925	1619	5202	2746	3741	3007	4024
ENSG00000173349	0	0	0	0	0	0	0	0	0	0	0	0	0	0	0	0	0	0	0	0	0	0	0	0	0	0	0	0	0	0	0	0
ENSG00000201470	5	0	5	4	1	5	0	5	0	1	9	1	4	2	2	0	3	0	0	4	8	2	5	5	4	3	3	22	1	3	5	8
ENSG00000200390	0	0	0	0	0	0	0	0	0	0	0	0	0	0	0	0	0	0	0	0	0	0	0	0	0	0	0	0	0	0	0	0
ENSG00000274010	17	12	4	10	8	6	5	0	0	2	17	1	3	5	2	8	5	4	5	6	7	0	1	1	4	2	5	8	2	5	1	3
ENSG00000144231	3293	2438	2812	2737	2343	2313	1900	2543	2100	1608	6570	1251	4152	3954	4750	5116	1059	1677	1643	2364	4019	1639	2924	1427	1759	2523	1280	4386	3270	4173	3437	4295
ENSG00000202532	0	0	0	0	0	0	0	0	0	0	0	0	0	0	0	0	0	0	0	0	0	0	0	0	0	0	0	0	0	0	0	0
ENSG00000244563	0	0	0	0	0	0	0	0	0	0	0	0	0	0	0	0	0	0	0	0	0	0	0	0	0	0	0	0	0	0	0	0
ENSG00000273950	10	8	9	7	4	2	6	4	6	0	3	3	8	11	7	10	0	4	4	7	19	4	2	6	2	4	3	5	2	6	3	8
ENSG00000144233	1339	937	1175	1034	1062	1196	847	1076	826	681	3800	564	1995	1815	2217	2367	354	526	474	949	1826	773	1308	687	816	1377	862	2217	1588	1972	1476	2058
ENSG00000272667	49	34	60	42	20	20	21	38	48	31	146	37	26	41	46	52	26	30	25	46	40	22	33	22	25	42	29	81	40	40	48	49
ENSG00000204399	0	0	0	0	0	0	0	0	0	0	0	0	0	0	0	0	0	0	0	0	0	0	0	0	0	0	0	0	0	0	0	0
ENSG00000136715	1313	1100	1340	1109	1479	1543	1128	1563	1065	918	3611	674	1619	1483	1660	1890	258	374	391	652	2108	892	1499	639	969	1655	674	2881	930	1228	920	1297
ENSG00000234044	0	0	0	0	0	0	0	0	0	0	0	0	1	0	0	0	0	0	0	0	0	0	0	0	0	0	1	0	0	0	0	0
ENSG00000200432	0	0	0	0	0	0	0	0	0	0	1	0	0	0	0	0	0	0	0	0	0	0	0	0	0	0	0	0	0	0	0	0
ENSG00000136731	5395	4136	5107	3922	5189	5473	3728	5721	3176	2426	12039	2189	8131	7208	8479	9562	1511	1996	1964	3662	8412	3424	5673	2793	3111	5139	2465	8372	4949	6834	5550	7699
ENSG00000229173	2	4	2	3	5	0	3	4	2	1	1	0	3	1	5	0	1	0	0	2	2	3	0	1	4	5	0	4	0	3	4	7
ENSG00000232101	0	0	0	0	0	2	0	0	0	0	0	1	2	3	1	0	0	0	0	0	3	1	1	1	0	0	0	3	2	4	2	0
ENSG00000229758	0	0	6	2	0	1	0	1	4	2	6	1	4	3	4	10	1	0	2	0	3	1	2	1	2	11	8	11	4	3	0	2
ENSG00000185631	1	0	1	3	1	0	1	3	0	2	3	0	2	2	2	0	0	0	0	0	1	3	4	1	3	1	0	5	2	4	0	3
ENSG00000238180	0	2	2	2	0	1	2	0	0	1	2	0	0	0	0	1	0	0	0	0	0	0	0	1	2	1	0	1	0	0	0	0
ENSG00000136720	2602	2161	2594	2177	9150	9420	6074	10171	291	231	1102	235	1308	1376	1196	1897	675	1245	1114	1984	11442	5443	8574	4295	237	388	256	708	884	1104	793	1262
ENSG00000252742	0	0	0	0	0	0	1	2	0	0	0	0	1	0	0	0	0	0	0	0	1	0	2	3	0	0	0	0	0	1	0	0
ENSG00000227653	0	0	0	0	0	1	2	1	0	0	0	0	0	0	0	0	0	0	0	0	0	0	2	0	0	0	0	0	0	1	0	0
ENSG00000237532	0	0	0	0	0	0	1	0	0	0	1	0	0	0	0	0	0	0	0	0	0	0	1	0	0	0	0	0	0	0	0	0
ENSG00000238277	0	0	0	0	0	0	0	0	0	0	0	0	0	0	0	1	0	0	0	0	0	0	0	0	0	0	0	0	0	0	0	0
ENSG00000204460	0	0	0	0	0	0	0	0	0	0	0	0	0	0	0	0	0	0	0	0	0	0	0	0	0	0	0	0	0	0	0	0
ENSG00000233428	1	0	0	0	0	0	0	0	0	0	0	0	0	0	0	0	0	0	0	1	1	0	0	0	0	0	0	0	0	0	0	0
ENSG00000229536	2	3	2	1	0	0	0	0	0	5	1	0	0	0	0	0	3	1	1	5	0	0	0	0	2	0	2	3	0	0	1	0
ENSG00000214100	0	0	0	0	0	0	0	0	0	0	0	0	0	0	0	0	0	0	0	0	0	0	0	0	0	0	0	0	0	0	0	0
ENSG00000237574	0	0	0	0	0	0	0	0	0	0	0	0	0	0	0	0	0	0	0	0	0	0	0	0	0	1	0	0	0	0	0	0
ENSG00000234559	0	0	0	0	0	0	0	0	0	0	0	0	0	0	0	0	0	0	0	0	0	0	0	0	0	0	0	0	0	0	0	0
ENSG00000225449	0	0	0	2	0	0	0	0	143	117	617	85	0	0	1	0	0	0	1	2	0	0	0	0	88	103	126	279	0	0	0	0
ENSG00000222014	0	0	0	0	0	0	0	0	68	57	248	43	0	0	2	0	0	0	0	2	1	0	1	0	51	65	58	123	0	0	0	0
ENSG00000180178	1	5	0	4	0	0	0	0	276	292	641	150	504	525	481	679	1	5	0	3	0	0	0	0	169	272	197	531	114	471	205	495
ENSG00000250207	0	0	0	0	0	0	0	0	0	0	0	1	0	0	0	0	0	0	0	0	0	0	0	0	0	0	1	0	0	0	0	0
ENSG00000217289	2	0	2	1	0	1	0	2	0	1	3	1	4	5	1	5	0	0	1	0	0	0	0	1	1	0	2	2	0	1	4	3
ENSG00000231147	0	0	0	0	0	0	0	0	0	0	2	0	0	0	0	0	0	0	0	0	0	0	0	0	0	1	3	6	0	0	0	0
ENSG00000231240	0	0	0	1	0	0	0	0	65	57	160	43	132	132	130	205	0	1	0	4	0	0	0	0	29	61	49	110	37	108	69	133
ENSG00000248313	0	0	0	0	0	1	0	0	10	4	12	2	1	7	3	6	0	0	0	0	0	0	0	0	1	4	4	14	0	5	1	7
ENSG00000196604	2	2	2	5	0	0	0	0	61	74	167	41	196	226	190	241	0	1	1	1	1	0	0	0	50	99	62	217	57	189	106	259
ENSG00000228471	0	0	0	0	0	0	0	0	0	0	0	0	0	0	0	0	0	0	0	0	0	0	0	0	0	0	0	0	0	0	0	0
ENSG00000224087	0	0	0	0	0	0	0	0	0	0	0	1	0	1	2	1	0	0	0	0	0	0	0	0	1	3	0	2	0	1	2	0
ENSG00000252223	0	0	0	0	0	0	0	0	0	0	0	0	0	0	0	0	0	0	0	0	0	0	0	0	0	0	0	0	0	0	0	0
ENSG00000223760	0	0	1	0	0	0	0	0	20	19	53	13	1	0	3	0	0	0	0	0	0	0	0	0	10	13	16	41	0	0	0	0
ENSG00000152076	138	143	145	109	0	1	3	4	306	231	435	131	58	62	74	96	81	58	75	87	0	0	2	1	128	291	108	290	43	75	37	95
ENSG00000136699	2851	2177	2836	2322	2696	2736	1953	2751	2489	2153	7247	1554	2143	1854	2085	2442	915	1331	1312	2317	4520	2000	3246	1760	2080	3735	1236	4944	1580	2070	1659	2316
ENSG00000152082	2074	1514	1657	1704	1571	1646	1116	1626	1860	1613	4796	1288	1294	1247	1187	1594	1335	2268	1626	1916	2795	1086	2211	977	1505	2372	757	3227	1276	1209	1343	1491
ENSG00000152086	1	2	4	1	2	3	6	3	4	9	14	2	1	1	5	4	0	0	2	4	3	5	5	1	0	1	8	13	1	2	3	1
ENSG00000227632	3	2	2	0	2	1	2	6	0	2	0	0	0	0	0	1	0	0	0	0	7	2	9	0	0	0	1	2	0	0	0	0
ENSG00000230756	0	2	0	2	0	2	0	5	1	0	1	0	0	1	1	3	1	1	0	3	1	0	1	3	1	3	1	3	1	0	0	0
ENSG00000213225	1	0	0	1	1	2	1	1	1	0	1	0	0	1	1	0	0	0	0	1	2	0	2	0	0	0	2	2	1	1	3	0
ENSG00000234398	0	0	0	0	0	0	0	0	0	0	0	0	0	0	0	0	0	0	0	0	0	0	0	0	0	0	0	0	0	0	0	0
ENSG00000236595	0	0	0	0	0	0	0	0	0	0	0	0	0	0	0	0	0	0	0	0	0	0	0	0	0	0	0	0	0	0	0	0
ENSG00000237722	0	0	0	0	0	0	0	0	0	0	0	0	0	0	0	0	0	0	0	0	0	0	0	0	0	0	0	0	0	0	0	0
ENSG00000231954	0	0	0	0	0	0	0	0	0	0	0	0	0	0	0	0	0	0	0	0	0	0	0	0	0	0	0	0	0	0	0	0
ENSG00000236211	0	0	0	0	0	0	0	0	0	0	0	0	0	0	0	0	0	0	0	0	0	0	0	0	0	0	0	0	0	0	0	0
ENSG00000230496	0	0	0	0	0	0	0	0	0	0	0	0	0	0	0	0	0	0	0	0	0	0	0	0	0	0	0	0	0	0	0	0
ENSG00000232990	0	0	0	0	0	0	0	0	0	0	0	0	0	0	0	0	0	0	0	0	0	0	0	0	0	0	0	0	0	0	0	0
ENSG00000249997	0	0	0	0	0	0	0	0	0	0	0	0	0	0	0	0	0	0	0	0	0	0	0	0	0	0	0	0	0	0	0	0
ENSG00000230548	0	0	0	0	0	0	0	0	0	0	0	0	0	0	0	0	0	0	0	0	0	0	0	0	0	0	0	0	0	0	0	0
ENSG00000234934	0	0	0	0	0	0	0	0	0	0	0	0	0	0	0	0	0	0	0	0	0	0	0	0	0	0	0	0	0	0	0	0
ENSG00000248136	0	0	0	0	0	0	0	0	0	0	0	0	0	0	0	0	0	0	0	0	0	0	0	0	0	0	0	0	0	0	0	0
ENSG00000233550	0	0	0	0	0	0	0	0	0	0	0	0	0	0	0	0	0	0	0	0	0	0	0	0	0	0	0	0	0	0	0	0
ENSG00000226915	1	0	0	0	0	0	0	1	0	0	1	0	0	1	0	0	0	1	0	1	2	0	0	1	2	0	0	0	0	0	1	1
ENSG00000271435	0	0	0	0	0	0	0	0	0	0	0	0	0	0	0	0	0	0	0	0	0	1	0	0	0	0	0	0	0	0	0	0
ENSG00000224352	0	0	0	0	0	0	0	0	0	0	0	0	0	0	0	0	0	0	0	0	0	0	0	0	0	0	0	0	0	0	0	0
ENSG00000136710	816	564	681	509	785	795	551	841	625	494	1907	368	488	439	472	587	806	1052	933	813	1695	634	1185	602	537	784	450	1351	635	558	649	702
ENSG00000136718	1938	1401	1564	1457	1496	1365	992	1518	1844	1298	5483	1056	1946	1721	2222	2377	1773	2922	2436	2236	3185	1223	2438	972	1908	3040	984	3559	2432	2213	2430	2476
ENSG00000072135	475	509	455	497	2	0	0	0	3892	3210	13665	2515	989	983	817	1299	112	249	208	409	1	1	0	0	2905	4688	2425	8321	563	640	423	788
ENSG00000178162	0	0	0	0	0	0	0	0	0	4	13	1	0	0	2	1	0	0	0	0	0	0	0	0	4	3	1	6	0	1	0	0
ENSG00000230646	0	0	0	0	0	0	0	0	0	0	0	0	0	0	0	0	0	0	0	0	0	0	0	0	0	0	0	0	0	0	0	0
ENSG00000239402	0	0	0	0	0	0	0	0	0	0	0	0	0	0	0	1	0	0	0	0	0	0	0	0	0	0	0	0	0	0	0	1
ENSG00000196834	0	0	0	0	0	0	0	0	0	0	4	0	2	1	0	2	0	0	0	0	0	0	0	0	0	1	2	4	0	0	0	0
ENSG00000251973	0	0	0	0	0	0	0	0	0	0	0	0	0	0	0	0	0	0	0	0	0	0	0	0	0	0	0	0	0	0	0	0
ENSG00000225819	0	0	0	0	0	0	0	0	0	0	0	0	0	0	0	0	0	0	0	0	0	0	0	0	0	0	0	0	0	0	0	0
ENSG00000274711	0	0	0	0	0	0	0	0	0	0	0	0	0	0	0	0	0	0	0	0	0	0	0	0	0	0	0	0	0	0	0	0
ENSG00000152093	0	0	0	0	0	0	0	0	0	0	0	0	0	0	0	0	0	0	0	0	0	0	0	0	0	0	0	0	0	0	0	0
ENSG00000225341	0	0	0	0	0	0	0	0	0	0	0	0	0	0	0	0	0	0	0	0	0	0	0	0	0	0	0	0	0	0	0	0
ENSG00000184761	0	0	0	0	0	0	0	0	0	0	0	0	0	0	0	0	0	0	0	0	0	0	0	0	0	0	0	0	0	0	0	0
ENSG00000183292	0	0	0	0	0	0	0	0	4	0	10	1	0	0	0	0	0	0	0	0	0	0	0	0	2	0	2	2	0	0	0	0
ENSG00000232408	0	0	0	0	0	0	0	0	0	0	0	0	0	0	0	0	0	0	0	0	0	0	0	0	0	0	0	0	0	0	0	0
ENSG00000136698	0	0	0	0	0	0	0	0	0	0	0	0	0	0	0	0	0	0	0	0	0	0	0	0	0	0	0	0	0	0	0	0
ENSG00000277668	0	0	0	0	0	0	0	0	0	0	0	0	0	0	0	0	0	0	0	0	0	0	0	0	0	0	0	0	0	0	0	0
ENSG00000222038	0	0	0	0	0	0	0	0	2	3	1	0	0	0	0	0	0	0	0	0	0	0	0	1	0	0	0	0	0	0	2	0
ENSG00000251757	0	0	0	0	0	0	0	0	0	0	0	0	0	0	0	0	0	0	0	0	0	0	0	0	0	0	0	0	0	0	0	0
ENSG00000214081	0	0	0	0	0	0	0	0	1	0	0	0	0	0	0	0	0	0	0	0	0	0	0	0	0	1	1	1	0	0	0	0

ENSG00000282944	0	0	0	0	0	0	0	0	6	0	7	7	0	0	0	0	0	0	0	0	0	0	0	0	0	3	1	6	0	0	0	0
ENSG00000240253	0	0	0	0	0	0	0	0	1	1	2	0	0	0	0	0	0	0	0	0	0	0	0	0	0	0	0	0	0	0	0	0
ENSG00000223349	0	0	0	0	0	0	0	0	0	0	0	0	0	0	0	0	0	0	0	0	0	0	0	0	0	0	0	0	0	0	0	0
ENSG00000173302	0	0	0	0	0	0	0	0	0	0	0	0	0	0	0	0	0	0	0	0	0	0	0	0	0	0	0	0	0	0	0	0
ENSG00000229797	0	0	0	0	0	0	0	0	0	0	0	0	0	0	0	0	0	0	0	0	0	0	0	0	0	0	0	0	0	0	0	0
ENSG00000178171	0	0	0	1	0	0	0	0	0	1	0	0	0	0	0	0	0	0	0	0	0	0	0	0	1	0	1	1	0	0	0	0
ENSG00000232503	0	0	0	0	0	0	0	0	0	0	0	0	0	0	0	0	0	0	0	0	0	0	0	0	0	0	0	0	0	0	0	0
ENSG00000233221	0	0	0	0	0	0	0	0	0	0	1	0	2	0	1	0	0	0	0	0	0	0	0	0	3	0	1	0	0	0	0	1
ENSG00000136002	12	7	40	10	40	34	28	45	28	46	117	29	28	76	36	118	13	22	33	32	148	66	105	55	32	67	31	135	13	59	16	57
ENSG00000281516	0	0	0	0	0	0	0	0	0	0	0	0	0	0	0	0	0	0	0	0	0	0	0	0	0	0	0	0	0	0	0	0
ENSG00000281159	0	0	0	0	0	0	0	0	0	0	0	0	0	0	0	0	0	0	0	0	0	0	0	0	0	0	0	0	0	0	0	0
ENSG00000152102	4514	3267	3952	3613	3377	3661	2664	3674	2806	2297	10176	1738	5863	5377	6076	7188	1088	1701	1885	3004	6145	2512	4162	2158	2766	4437	2256	6924	4113	5240	4123	5483
ENSG00000115762	3917	2840	3256	2796	2016	2007	1350	2040	2757	2213	10662	1848	4648	3901	4450	5140	2054	3380	2722	3034	3742	1472	2619	1222	3030	4661	2397	7255	4044	4193	3940	4901
ENSG00000230450	0	0	0	0	0	0	0	0	0	0	0	1	0	0	1	0	0	0	0	0	0	0	1	0	0	0	0	0	1	0	0	0
ENSG00000225448	0	0	0	0	0	0	0	0	0	0	0	0	0	0	0	0	0	0	0	0	0	0	0	0	0	0	0	0	0	0	0	1
ENSG00000236956	0	0	0	0	0	0	0	0	0	0	0	0	0	0	0	0	0	0	0	0	0	0	0	0	0	0	0	0	0	0	0	0
ENSG00000248796	0	0	0	0	0	0	0	0	0	0	0	0	0	0	0	0	0	0	0	0	0	0	0	1	0	0	0	0	0	0	0	0
ENSG00000188219	1	3	0	2	1	2	1	3	24	36	65	15	7	20	16	19	0	0	1	2	2	1	3	0	26	36	21	78	4	13	4	14
ENSG00000212199	0	0	0	0	0	0	0	0	0	0	0	0	0	0	0	0	0	0	0	0	0	0	0	0	0	0	0	0	0	0	0	0
ENSG00000231431	0	0	0	0	0	0	0	0	4	6	12	1	0	0	0	2	0	0	0	0	0	0	0	0	1	4	7	12	0	0	0	1
ENSG00000236516	0	0	0	0	0	0	0	0	0	2	11	1	0	0	0	0	0	0	0	0	0	0	0	0	4	1	4	3	0	1	0	0
ENSG00000227817	0	0	0	0	0	0	0	2	0	0	0	0	0	0	0	0	0	0	0	0	0	0	0	0	0	0	0	0	0	0	0	0
ENSG00000224425	0	0	0	0	0	0	0	0	0	0	0	0	0	0	0	0	0	0	0	0	0	0	0	0	0	0	0	0	0	0	0	0
ENSG00000233087	0	0	0	1	2	1	0	0	2	0	1	0	0	0	0	3	0	1	1	1	1	0	1	0	0	0	0	0	0	0	0	2
ENSG00000283303	0	0	0	0	0	0	0	0	0	0	0	0	0	0	0	0	0	0	0	0	0	0	0	0	0	0	0	0	0	0	0	0
ENSG00000233285	0	0	0	0	0	0	0	0	0	0	0	0	0	0	0	0	0	0	0	0	0	0	0	0	0	0	0	0	0	0	0	0
ENSG00000227275	0	0	0	0	0	0	0	0	0	0	0	0	0	0	0	0	0	0	0	0	0	0	0	0	0	0	0	0	0	0	0	0
ENSG00000235080	0	0	0	0	0	0	0	0	0	0	0	0	0	0	0	0	0	0	0	0	1	0	0	0	0	0	0	0	0	0	0	0
ENSG00000231777	0	0	0	0	0	0	0	0	0	0	0	0	0	0	0	0	0	0	0	0	0	0	0	0	0	0	0	0	0	0	0	0
ENSG00000227661	0	0	0	0	0	0	0	0	0	0	0	0	0	0	0	0	0	0	0	0	0	0	0	0	0	0	0	0	0	0	0	0
ENSG00000236590	0	0	0	0	0	0	0	0	0	0	0	0	0	0	0	0	0	0	0	0	0	0	0	0	0	0	0	0	0	0	0	0
ENSG00000234480	0	0	0	0	0	0	0	0	0	0	0	0	0	0	0	0	0	0	0	0	0	0	0	0	0	0	0	0	0	0	0	0
ENSG00000237910	0	0	0	0	0	0	0	0	0	0	0	0	0	0	0	0	0	0	0	0	0	0	0	0	0	0	0	0	0	0	0	0
ENSG00000234939	0	0	0	0	0	0	0	0	0	0	0	0	0	0	0	0	0	0	0	0	0	0	0	0	0	0	0	0	0	0	0	0
ENSG00000234576	0	0	0	0	0	0	0	0	0	0	0	0	0	0	0	0	0	0	0	0	0	0	0	0	0	0	0	0	0	0	0	0
ENSG00000226831	0	0	0	0	0	0	0	0	0	0	0	0	0	0	0	0	0	0	0	0	0	0	0	0	0	0	0	0	0	0	0	0
ENSG00000276460	0	0	0	0	0	0	0	0	0	0	0	0	0	0	0	0	0	0	0	0	0	0	0	0	0	0	0	0	0	0	0	0
ENSG00000223631	6	3	2	3	0	0	1	0	0	0	1	0	0	0	0	0	17	28	13	14	0	0	0	0	0	0	0	0	0	0	0	0
ENSG00000214077	9	6	4	9	14	17	10	13	0	0	0	0	0	0	1	1	2	1	3	9	18	17	9	11	0	0	0	1	0	0	0	0
ENSG00000217950	0	4	0	0	0	1	1	0	2	0	0	1	1	0	0	1	1	0	0	0	0	0	0	0	0	2	0	1	1	0	0	1
ENSG00000232742	3	7	4	5	3	0	5	3	0	0	1	4	0	1	3	1	4	1	4	4	2	6	1	0	1	0	0	2	0	0	1	3
ENSG00000273073	8	2	6	4	2	2	2	1	4	6	20	2	0	0	0	3	3	2	0	5	2	0	0	2	4	2	4	4	0	3	2	1
ENSG00000173272	2014	1372	1541	1546	1598	1678	1137	1718	1328	1083	3561	899	1880	1763	1706	2337	890	1422	1195	1611	2647	1053	2073	928	1003	1548	607	2299	1443	1581	1434	1779
ENSG00000075886	62	35	62	39	59	58	44	79	92	82	302	69	56	54	77	81	20	11	24	55	81	60	51	46	63	97	53	194	27	85	46	81
ENSG00000152117	1689	1313	1497	1305	727	807	546	819	866	728	2875	542	814	764	809	1056	497	521	404	1090	1310	609	734	594	631	1039	559	1840	346	705	404	838
ENSG00000163040	673	546	618	566	0	0	0	0	690	587	1293	371	438	410	405	543	478	490	423	467	0	0	0	0	325	591	272	847	236	350	281	442
ENSG00000224679	0	1	0	0	0	0	0	0	2	3	4	0	0	0	2	0	0	0	0	0	0	0	0	0	0	0	2	0	0	0	1	0
ENSG00000204434	0	2	0	0	0	0	1	0	0	0	0	1	0	0	0	0	0	0	0	0	0	0	0	0	0	0	0	0	0	0	1	0
ENSG00000251956	0	0	0	0	0	0	0	0	0	0	0	0	0	0	0	0	0	0	0	0	0	0	0	0	0	0	0	0	0	0	0	0
ENSG00000273588	0	0	0	0	0	0	0	0	0	1	0	0	0	0	0	0	0	0	0	0	0	0	0	0	0	0	0	3	0	0	0	0
ENSG00000224559	0	0	0	0	0	0	0	0	2	4	2	1	0	0	0	0	0	0	0	1	0	0	0	0	1	2	0	3	0	0	0	0
ENSG00000270930	0	0	0	0	0	0	0	0	0	0	0	0	0	0	0	0	0	0	0	0	0	0	0	0	0	0	0	0	0	0	0	0
ENSG00000197927	11	22	16	16	161	136	121	157	122	125	348	69	10	18	16	32	5	12	10	22	259	122	176	110	97	136	77	259	6	17	7	16
ENSG00000213222	8	20	9	17	83	119	78	70	58	51	194	54	16	14	16	26	1	5	2	16	178	79	142	63	57	83	46	142	3	9	3	11
ENSG00000186825	0	1	0	1	0	0	2	0	1	0	1	0	0	0	0	0	0	0	0	0	0	0	0	0	0	0	0	0	0	0	0	1
ENSG00000274353	0	0	0	3	0	0	0	0	0	0	0	0	0	0	0	0	0	0	0	0	0	0	0	0	0	0	0	0	0	0	0	0
ENSG00000229203	0	0	4	1	0	0	0	0	0	0	0	1	3	0	0	0	0	0	0	0	0	0	0	0	0	0	0	0	0	0	0	0
ENSG00000232760	0	0	0	1	0	0	0	0	0	0	0	0	0	0	0	1	0	0	0	0	0	0	0	0	0	0	0	0	1	0	0	0
ENSG00000244337	0	0	0	0	0	0	0	0	0	0	0	0	0	0	0	0	0	0	0	0	0	0	0	0	0	0	0	0	0	0	0	0
ENSG00000235615	0	0	0	0	0	0	0	0	0	0	1	0	0	0	0	0	0	0	0	1	0	0	0	0	0	0	2	0	0	0	0	0
ENSG00000236485	0	0	0	0	0	0	0	0	0	0	0	0	0	0	0	0	0	0	0	0	0	0	0	0	0	0	0	0	0	0	0	0
ENSG00000226886	0	0	0	0	0	0	0	0	0	0	0	0	0	0	0	0	0	0	0	0	0	0	0	0	0	0	0	0	0	0	0	0
ENSG00000271179	0	0	0	0	0	0	0	0	0	0	0	0	0	0	0	0	0	0	0	0	0	0	0	0	0	0	0	0	0	0	0	0
ENSG00000225594	0	0	0	0	0	0	0	0	0	0	0	0	0	0	0	0	0	0	0	0	0	0	0	0	0	0	0	0	0	0	0	0
ENSG00000227745	0	0	0	0	0	0	0	0	0	0	0	0	0	0	0	0	0	0	0	0	0	0	0	0	0	0	0	0	0	0	0	0
ENSG00000163046	0	0	0	0	0	0	1	0	0	0	0	0	0	0	0	0	0	0	0	0	0	0	0	0	0	0	1	0	0	0	0	0
ENSG00000239108	0	0	0	0	0	0	0	0	0	0	0	0	0	0	0	0	0	0	0	0	0	0	0	0	0	0	0	0	0	0	0	0
ENSG00000221288	0	0	0	2	0	0	0	0	0	0	0	0	0	0	0	0	0	0	0	0	0	0	0	0	0	0	0	0	0	0	0	0
ENSG00000233786	0	0	0	0	0	0	1	3	0	0	0	0	0	0	0	0	0	0	0	0	0	0	0	0	0	0	0	0	0	1	0	0
ENSG00000230803	0	0	0	0	0	0	0	0	0	0	0	0	0	0	0	0	0	0	0	0	0	0	0	0	0	0	0	0	0	0	0	0
ENSG00000018607	0	0	0	0	0	0	0	0	0	0	0	0	0	0	0	0	0	0	0	0	0	0	0	0	0	0	0	0	0	0	0	0
ENSG00000272769	0	0	0	0	0	0	0	0	0	0	0	0	0	0	0	0	0	0	0	0	0	0	0	0	0	0	0	0	0	0	0	0
ENSG00000230992	0	0	0	0	0	0	0	0	0	0	0	0	0	0	0	0	0	0	0	0	0	0	0	0	0	0	0	0	0	0	0	0
ENSG00000252705	0	0	0	0	0	0	0	0	0	0	0	0	0	0	0	0	0	0	0	0	0	0	0	0	0	0	0	0	0	0	0	0
ENSG00000183840	0	0	0	0	0	0	0	0	0	0	1	0	0	0	0	0	0	0	0	0	0	0	0	0	0	0	0	0	0	0	0	0
ENSG00000232349	0	0	0	0	0	0	0	0	0	0	0	0	0	0	0	0	0	0	0	0	0	0	0	0	0	0	0	0	0	0	0	0
ENSG00000200718	0	0	0	0	0	0	0	0	0	0	0	0	0	0	0	0	0	0	0	0	0	0	0	0	0	0	0	0	0	0	0	0
ENSG00000150551	0	0	0	0	0	0	0	0	0	0	0	0	0	0	0	0	0	0	0	0	0	0	0	0	0	0	0	0	0	0	0	0
ENSG00000230065	0	0	0	0	0	0	0	0	0	0	0	0	0	0	0	0	0	0	0	0	0	0	0	0	0	0	0	0	0	0	0	0
ENSG00000176771	0	0	0	0	0	0	0	0	0	1	9	5	0	1	0	0	0	0	0	0	0	0	0	0	1	2	2	7	0	0	0	0
ENSG00000228721	0	0	0	0	0	0	0	0	0	0	0	0	0	0	0	0	0	0	0	0	0	0	0	0	0	0	0	0	0	0	0	0
ENSG00000233729	0	0	0	0	0	0	0	0	0	0	0	1	0	0	0	0	0	0	0	0	0	0	0	0	0	0	0	1	0	0	0	0
ENSG00000279512	0	0	0	0	0	0	0	0	0	0	0	0	0	0	0	0	0	0	0	0	0	0	0	0	0	0	0	0	0	0	0	0
ENSG00000226953	0	0	0	0	0	0	0	0	0	0	0	0	0	0	0	0	0	0	0	0	0	0	0	0	0	0	0	0	0	0	0	0
ENSG00000252688	0	0	0	0	0	0	0	0	0	0	0	0	0	0	0	0	0	0	0	0	0	0	0	0	0	0	0	0	0	0	0	0
ENSG00000222068	0	0	0	0	0	0	0	0	0	0	0	0	0	0	0	0	0	0	0	0	0	0	0	0	0	0	0	0	0	0	0	0
ENSG00000232474	0	0	0	0	0	0	0	0	0	0	0	0	0	0	0	0	0	0	0	0	0	0	0	0	0	0	0	0	0	0	0	0
ENSG00000223430	0	0	0	0	0	0	0	0	0	0	0	0	0	0	0	0	0	0	0	0	0	0	0	0	0	0	0	0	0	0	0	0
ENSG00000200708	0	0	0	0	0	0	0	0	0	0	0	0	0	0	0	0	0	0	0	0	0	0	0	0	0	0	0	0	0	0	0	0
ENSG00000152127	2489	2406	2189	2332	2918	3182	2112	3268	1758	1691	8811	1395	2649	2608	2478	3418	429	874	624	1410	4365	1901	2975	1522	1625	2789	3174	6863	1512	2107	1363	2142
ENSG00000263813	2	3	0	1	1	0	2	2	1	3	4	1	3	4	0	1	0	2	0	2	6	2	3	6	1	2	0	5	1	3	1	3
ENSG00000234060	2	2	0	0	3	1	0	0	1	0	2	0	1	3	0	0	0	0	0	0	2	0	1	1	3	1	0	12	0	2	0	0
ENSG00000152128	7	3	2	4	2	9	5	3	747	697	3265	494	2	1	0	5	2	0	1	4	8	1	6	0	523	809	421	1445	2	4	4	4
ENSG00000224043	48	29	24	34	18	22	10	13	27	28	105	22	31	39	29	57	28	30	25	38	24	21	18	17	24	27	23	59	30	38	32	49
ENSG00000234682	1	0	0	3	0	0	0	0	0	0	0	0	0	0	0	0	1	0	0	0	0	1	0	0	0	0	0	0	0	2	0	1
ENSG00000228098	0	2	0	0	0	0	0	1	0	0	0	0	0	0	0	0	0	0	0	0	0	0	0	0	0	1	0	1	0	0	0	0
ENSG00000153086	15	17	9	6	5	7	1	9	14	12	31	11	7	12	10	17	3	2	2	17	10	3	7	6	4	13	16	20	1	9	10	8
ENSG00000263783	0	0	0	2	0	0	0	0	0	1	2	0	0	0	0	0	0	0	0	0	1	0	0	0	0	0	0	0	0	0	0	0
ENSG00000082258	2103	1368	1614	1274	1740	1738	1392	1921	1347	1217	6214	982	2574	2448	2857	3125	1096	1179	1024	1724	4270	1811	2448	1809	1392	2000	1617	3788	1937	2846	2517	3385
ENSG00000176601	3	1	2	1	7	2	1	1	0	2	3	0	6	4	3	4	0	0	2	0	4	1	5	3	0	0	0	3	0	0	8	3
ENSG00000115839	1604	904	1356	1008	1449	1687	1232	1847	704	548	3083	349	4528	4157	4632	5431	830	899	1101	894	2754	1250	2260	1074	552	758	1076	1467	3136	3721	4023	4285
ENSG00000208308	0	0	0	1	0	2	0	1	0	1	1	0	2	5	1	3	0	0	0	1	1	4	2	1	1	0	1	2	1	2	4	4
ENSG00000121988	611	429	526	453	470	499	409	560	524	376	1404	271	1010	946	1029	1250	335	471	423	473	996	439	743	428	372	561	238	915	721	882	934	1065
ENSG00000235651	36	11	20	23	4	10	6	9	26	7	57	4	22	24	20	24	7	8	8	27	14	4	4	9	15	16	16	50	8	26	29	28
ENSG00000048991	3144	2469	3075	2559	3061	2897	2366	3123	2007	1656	7778	1153	4213	3763	4529	4903	766	1241	1209	1833	4598	1914	3585	1661	2001	3106	1781	5021	2791	3633	3133	3901
ENSG00000201308	2	1	0	1	2	0	0	0	0	1	4	0	0	1	3	0	0	2	0	1	1	0	0	3	0	0	1	3	1	0	1	3
ENSG00000207654	3	0	2	1	0	2	0	7	1	4	6	1	2	0	3	1	0	0	1	2	2	1	0	1	2	1	1	2	2	4	1	2
ENSG00000144224	6571	4103	5349	4220	4489	4601	3612	5077	4181	3394	15865	2629	8833	8791	8495	10844	3578	3636	3909	4599	9786	3919	6776	4092	3661	5300	4098	9970	7236	9096	8182	10646
ENSG00000115850	42	16	52	19	8	15	3	6	2	6	23	0	75	80	76	90	13	18	31	37	23	11	9	4	7	2	5	10	64	115	74	108
ENSG00000200664	0	0	1	0	0	0	0	0	0	0	0	0	0	0	0	0	0	0	0	0	0	0	0	0	0	0	0	0	0	0	0	0
ENSG00000226806	122	62	152	71	139	132	102	169	19	15	100	16	66	27	49	49	141	100	226	107	314	125	220	134	5	10	41	39	34	32	35	35
ENSG00000076003	7403	5294	5716	5048	5240	5832	4201	6255	6020	4549	13856	3290	15022	13032	10618	15339	6745	9451	8149	6231	13205	5008	9726	4544	5411	7693	2811	11284	10618	10657	9501	11493
ENSG00000214070	0	0	2	0	1	3	0	3	3	4	8	2	1	5	0	2	0	0	0	2	2	0	0	0	2	2	0	6	2	0	4	2
ENSG00000115866	5007	3659	4361	3557	4685	4719	3611	5315	3817	2754	8739	1942	9266	8352	9326	10625	3448	4479	3938	3347	9461	3392	6731	3876	2564	3701	1656	5301	7682	8070	8907	9442
ENSG00000231890	172	108	162	106	87	122	72	99	161	153	230	77	354	369	291	444	27	13	17	32	24	14	16	11	29	41	36	114	87	145	105	155
ENSG00000237262	8	2	9	3	0	1	0	0	0	1	0	0	11	9	4	10	0	0	0	0	0	0	0	0	0	0	0	0	1	1	4	1
ENSG00000121966	4806	3451	4384	3241	1813	1756	1512	2024	2365	1980	11081	1976	8556	8498	8776	10519	3307	5130	4733	3556	3370	1185	2782	1192	2054	2726	3388	7629	6168	6531	7402	7915
ENSG00000227347	1	2	4	0	3	3	1	5	6	3	9	1	0	7	3	5	0	9	3	2	2	2	3	5	2	4	0	12	2	4	7	9
ENSG00000279024	0	0	0	0	0	0	0	0	0	0	2	1	7	12	16	11	0	1	0	0	1	0	0	0	0	0	1	0	5	4	4	13
ENSG00000230037	1	0	0	0	0	1	0	0	0	0	0	0	0	0	0	0	0	0	0	0	0	0	0	1	0	0	0	1	0	0	0	0
ENSG00000251976	0	0	0	0	0	0	0	0	0	0	0	0	0	0	0	0	0	0	0	0	0	0	0	0	0	0	0	0	0	0	0	0
ENSG00000229568	0	0	0	0	0	0	0	0	0	0	0	0	0	0	0	0	0	0	0	0	0	0	0	0	0	0	0	0	0	0	1	0
ENSG00000144229	1	0	0	0	0	0	1	1	2	2	55	2	1	1	0	3	0	0	0	0	0	0	0	0	6	4	10	40	0	0	0	2
ENSG00000222394	0	0	0	0	0	0	0	0	0	0	0	0	0	0	0	0	0	0	0	0	0	0	0	0	0	0	0	1	0	0	0	0
ENSG00000150540	2	0	0	0	0	0	1	0	0	0	3	0	3	2	0	1	0	0	0	0	0	0	0	0	0	0	2	0	0	1	0	2
ENSG00000224231	0	0	0	0	0	0	0	0	0	0	4	0	0	5	1	6	1	0	0	0	0	0	0	0	0	0	0	0	0	1	1	3
ENSG00000233045	1	0	0	0	1	0	0	0	2	0	1	0	7	3	7	10	0	2	0	1	0	0	0	0	4	1	0	1	2	2	1	10
ENSG00000234645	4	1	0	0	1	0	0	1	0	0	7	1	3	2	4	6	0	1	0	2	2	1	1	1	0	2	0	5	2	4	0	3
ENSG00000237939	0	0	0	0	0	0	0	0	0	1	0	0	0	1	0	0	0	0	0	0	0	0	0	0	0	0	0	0	0	1	0	0
ENSG00000232915	2	2	2	0	0	0	0	0	6	3	11	1	8	11	11	17	0	1	0	0	0	0	0	0	3	2	3	15	4	13	3	12
ENSG00000228043	9	8	8	7	0	0	0	0	12	6	25	8	38	57	52	55	2	4	4	14	0	0	0	0	11	17	5	21	17	33	52	46
ENSG00000230569	1	1	1	4	0	0	0	0	4	3	6	0	12	8	10	7	0	0	0	0	0	0	0	0	0	1	0	8	3	23	7	8
ENSG00000144228	310	233	287	255	0	0	0	0	587	486	2470	470	1281	1262	1383	1533	206	288	248	343	0	0	0	0	773	1108	616	1848	1286	1527	1363	1682
ENSG00000241772	0	1	1	1	0	0	0	0	3	1	13	1	6	5	5	8	0	0	0	3	0	0	0	0	7	7	7	10	0	6	7	7
ENSG00000260059	0	1	1	0	0	0	0	0	8	1	6	0	4	7	3	10	0	0	0	0	0	0	0	0	0	4	2	5	2	7	9	7
ENSG00000237772	0	0	0	0	0	0	0	0	0	0	1	0	0	3	2	1	0	0	0	0	0	0	0	0	0	0	0	1	1	2	0	1
ENSG00000144227	0	0	0	0	0	0	0	0	0	0	0	0	0	0	0	0	0	0	0	0	0	0	0	0	0	0	0	0	0	0	0	0
ENSG00000222826	0	0	0	0	0	0	0	0	0	0	0	0	0	0	0	0	0	0	0	0	0	0	0	0	0	0	0	0	0	0	0	0
ENSG00000229104	0	0	0	0	0	0	0	0	0	0	0	0	0	0	0	0	0	0	0	0	0	0	0	0	0	0	0	0	0	0	0	0
ENSG00000232992	0	0	0	0	0	0	0	0	0	0	0	0	0	0	0	0	0	0	0	0	0	0	0	0	0	0	0	0	0	0	0	0
ENSG00000226939	2	1	2	3	0	0	0	0	0	0	0	0	13	6	5	18	0	3	2	3	1	1	0	0	0	0	0	0	1	10	6	13
ENSG00000229131	4	5	4	3	3	3	3	1	0	0	0	0	9	15	19	25	15	4	6	4	4	2	1	3	0	0	0	0	12	23	14	16
ENSG00000206901	0	0	2	0	0	0	0	0	0	0	0	0	0	0	0	0	0	0	0	0	0	0	0	0	0	0	0	0	0	1	3	0
ENSG00000235232	0	0	0	0	0	0	0	0	0	0	0	0	0	0	0	1	0	0	0	0	0	0	0	0	0	0	0	0	0	0	0	0
ENSG00000228374	1	0	0	0	0	0	0	0	0	0	0	0	0	0	0	0	0	0	0	0	0	0	0	0	0	0	0	0	0	0	0	0
ENSG00000223554	0	0	0	0	0	0	0	0	0	0	0	0	0	0	0	1	0	0	0	0	0	0	0	0	0	0	0	0	0	0	0	0
ENSG00000240977	0	0	0	0	0	0	0	0	0	0	0	0	0	0	0	0	0	0	0	0	0	0	0	0	0	0	0	0	0	0	0	0
ENSG00000224028	0	0	0	0	0	0	0	0	0	0	0	0	0	0	0	0	0	0	0	0	0	0	0	0	0	0	0	0	0	0	0	0
ENSG00000227149	0	0	0	0	0	0	0	0	0	0	0	0	0	0	0	0	0	0	0	0	0	0	0	0	0	0	0	0	0	0	0	0
ENSG00000231567	0	0	0	0	0	0	0	0	0	0	0	0	0	0	0	0	0	0	0	0	0	0	0	0	0	0	0	0	0	0	0	0
ENSG00000223921	0	0	0	0	0	0	0	0	0	0	0	0	0	0	0	0	0	0	0	0	0	0	0	0	0	0	0	0	0	0	0	0
ENSG00000168702	0	1	0	0	0	0	2	0	0	0	4	0	7	2	7	12	0	0	0	0	3	1	0	1	3	0	5	3	6	6	12	4
ENSG00000277306	0	0	0	0	0	0	0	0	0	0	0	0	0	0	0	0	0	0	0	0	0	0	0	0	0	0	0	0	0	0	0	0
ENSG00000283506	0	0	0	0	0	0	0	0	0	0	0	0	0	0	0	0	0	0	0	0	0	0	0	0	0	0	0	0	0	0	0	0
ENSG00000236440	0	0	0	0	0	0	0	0	0	0	0	0	0	0	0	0	0	0	0	0	0	0	0	0	0	0	0	0	0	1	0	0
ENSG00000238065	0	0	0	0	0	0	0	0	0	0	0	0	0	0	0	0	0	0	0	0	0	0	0	0	0	0	0	0	0	0	0	0
ENSG00000201892	0	0	0	0	0	0	0	0	0	0	0	0	0	0	0	0	0	0	0	0	0	0	0	0	0	0	0	0	0	0	0	0
ENSG00000252015	0	0	0	0	0	0	0	0	0	0	0	0	0	0	0	0	0	0	0	0	0	0	0	0	0	0	0	0	0	0	0	0
ENSG00000233329	0	0	0	0	0	0	0	0	0	0	0	0	0	0	0	0	0	0	0	0	0	0	0	0	0	0	0	0	0	0	0	0
ENSG00000244125	0	0	0	0	0	0	0	0	0	0	0	0	0	0	0	0	0	0	0	0	0	0	0	0	0	0	0	0	0	0	0	0
ENSG00000228134	0	0	0	0	0	0	0	0	0	0	0	0	0	0	0	0	0	0	0	0	0	0	0	0	0	0	0	0	0	0	0	0
ENSG00000229781	0	0	0	0	0	1	0	3	0	0	0	0	1	0	0	0	0	0	0	0	0	0	1	0	0	0	3	0	0	0	0	0
ENSG00000115919	0	1	0	0	40	31	24	26	1	0	12	0	18	14	11	15	0	0	2	3	32	13	20	11	0	0	0	4	5	2	5	5
ENSG00000270390	0	0	0	0	0	0	0	0	0	0	0	0	0	0	0	0	0	0	0	0	0	0	0	0	0	0	0	0	0	0	0	0
ENSG00000075884	3086	2082	2895	2007	2028	1962	1527	2245	1509	1041	4196	743	3498	3489	4091	4365	4824	5767	5725	4055	5322	1869	3745	2566	1318	1831	959	2904	3589	3697	5082	4839
ENSG00000224288	0	0	0	3	0	2	1	1	0	0	0	0	0	2	2	0	0	0	0	0	0	0	0	1	0	0	0	0	0	0	0	0
ENSG00000226839	0	0	0	0	0	0	0	0	0	0	0	0	0	0	0	0	0	0	0	0	0	0	0	0	0	0	0	0	0	0	0	0
ENSG00000224852	0	0	0	0	0	0	0	0	0	0	0	0	0	0	0	0	0	0	0	0	0	0	0	0	0	0	0	0	0	0	0	0
ENSG00000232772	0	0	0	0	0	0	0	0	0	0	0	0	0	0	0	1	0	0	0	0	0	0	0	0	0	0	0	0	0	0	0	0
ENSG00000236300	0	0	0	0	0	0	0	0	0	0	0	0	0	0	0	0	0	0	0	0	0	0	0	0	0	0	0	0	0	0	0	0
ENSG00000227557	0	0	0	0	0	0	0	0	0	0	0	0	0	0	0	0	0	0	0	0	0	0	0	0	0	0	0	0	0	0	0	0
ENSG00000225476	0	0	0	0	0	0	0	0	0	0	0	0	0	0	1	4	0	0	0	0	0	0	0	0	0	0	1	0	0	0	0	0
ENSG00000231766	0	0	0	0	0	0	0	1	0	0	0	0	0	0	0	0	0	0	0	0	0	0	0	0	0	0	0	0	0	0	0	0
ENSG00000257284	8	3	8	2	2	7	7	2	3	11	7	2	11	6	12	11	1	4	2	13	11	6	14	9	14	11	2	18	5	12	7	13
ENSG00000228655	35	16	21	20	38	33	28	38	35	14	89	15	41	63	59	37	17	9	11	53	69	39	43	47	54	44	21	98	17	76	45	78
ENSG00000257640	29	5	21	6	15	12	15	16	11	11	43	6	20	29	25	23	1	3	7	33	38	33	17	9	47	25	6	46	5	27	28	29
ENSG00000258268	23	7	21	4	10	10	8	12	10	17	36	1	29	15	23	33	4	3	1	16	31	9	15	18	23	19	3	31	2	27	18	22
ENSG00000231758	24	5	14	11	7	7	8	12	6	7	17	0	15	20	21	19	1	3	3	8	17	10	18	11	17	28	3	52	6	23	16	21
ENSG00000257277	10	6	16	6	7	8	6	11	5	6	19	8	13	14	15	17	3	2	2	15	21	8	7	13	7	10	10	32	2	14	14	18
ENSG00000257226	8	2	3	3	7	7	8	5	10	2	15	2	7	4	10	15	2	3	0	8	8	9	1	1	5	4	5	10	1	5	7	10
ENSG00000236356	0	0	0	0	0	0	0	0	0	0	0	0	1	0	0	0	0	0	0	0	0	0	0	0	0	0	0	0	0	0	0	0
ENSG00000200287	0	0	0	0	0	0	0	0	0	0	0	0	0	0	0	0	0	0	0	0	0	0	0	0	0	0	0	0	0	0	0	0
ENSG00000232377	6	6	2	3	3	6	5	3	3	0	11	1	2	0	0	2	2	2	1	1	5	8	1	2	1	0	2	5	2	4	0	2
ENSG00000121964	567	414	376	405	524	498	366	565	202	193	857	147	517	483	536	624	364	536	404	526	891	339	632	355	211	282	282	502	386	394	426	498
ENSG00000169554	5	11	13	12	1236	1296	951	1325	1168	1057	4101	694	201	272	239	340	4	4	4	4	2290	858	1538	826	1164	1523	1218	3262	60	219	153	301
ENSG00000283118	1	0	0	0	4	9	1	5	3	6	10	0	1	1	3	0	0	0	0	0	10	7	2	5	9	8	3	14	0	0	0	1
ENSG00000279166	0	0	0	1	17	14	12	17	15	23	23	6	2	4	3	5	0	0	0	0	42	25	20	22	46	37	10	83	0	2	9	6
ENSG00000238057	0	0	0	0	5	13	6	16	6	1	13	1	2	1	0	1	0	0	0	0	12	5	14	17	12	4	6	11	0	0	1	5
ENSG00000275372	0	0	0	0	0	0	0	0	0	0	0	0	0	0	0	0	0	0	0	0	0	0	0	0	0	0	0	0	0	0	0	0
ENSG00000273537	0	0	0	0	0	0	0	0	0	0	0	0	0	0	0	0	0	0	0	0	0	0	0	0	0	0	0	0	0	0	0	0
ENSG00000277444	0	0	0	0	0	0	0	0	0	0	0	0	0	0	0	0	0	0	0	0	0	0	0	0	0	0	0	0	0	0	0	0
ENSG00000232606	0	0	0	0	0	0	0	0	0	0	0	0	0	0	0	0	0	0	0	0	0	0	0	0	0	0	0	0	0	0	0	0
ENSG00000226674	0	0	0	0	0	0	1	0	0	1	8	0	0	1	1	2	0	0	0	1	1	0	1	0	0	0	1	0	2	1	0	1
ENSG00000234940	0	0	0	0	0	0	0	0	0	0	0	0	0	0	0	0	0	0	0	0	0	0	0	0	0	0	0	0	0	0	0	0
ENSG00000276706	0	0	0	0	0	0	0	0	0	0	0	0	0	0	1	0	0	0	0	0	0	0	0	0	0	0	0	0	0	0	0	0
ENSG00000235352	0	0	0	0	0	0	0	0	0	0	0	0	0	0	0	0	0	0	0	0	0	0	0	0	0	0	0	0	0	0	0	0
ENSG00000268580	0	0	0	0	0	0	0	0	0	0	0	0	0	0	0	0	0	0	0	0	0	0	0	0	0	0	0	0	0	0	2	0
ENSG00000235435	0	0	0	0	0	0	0	0	0	0	0	0	0	0	0	0	0	0	0	0	0	0	0	0	0	0	0	0	0	0	0	0
ENSG00000214064	0	0	0	0	0	0	0	0	0	0	0	0	0	0	0	0	0	0	0	0	0	0	0	0	0	0	0	0	0	0	0	0
ENSG00000225107	0	0	0	0	0	0	0	0	0	0	0	0	0	0	0	0	0	0	0	0	0	0	0	0	0	0	0	0	0	0	0	0
ENSG00000226218	0	0	0	0	0	0	0	0	0	0	0	0	0	0	0	0	0	0	0	0	0	0	0	0	0	0	0	0	0	0	0	0
ENSG00000234119	0	0	0	0	0	0	0	0	0	0	0	0	0	0	0	0	0	0	0	0	0	0	0	0	0	0	0	0	0	0	0	0
ENSG00000279876	0	0	0	0	0	0	0	0	0	0	0	0	0	0	0	0	0	0	0	0	0	0	0	0	0	0	0	0	0	0	0	0
ENSG00000233842	0	0	0	0	0	0	0	0	0	0	0	0	0	0	0	0	0	0	0	0	0	0	0	0	0	0	0	0	0	0	0	0
ENSG00000251905	0	0	0	0	0	0	0	0	0	0	0	0	0	0	0	0	0	0	0	0	0	0	0	0	0	0	0	0	0	0	0	0
ENSG00000230559	0	0	0	0	0	0	0	0	0	0	0	0	0	0	0	0	0	0	0	0	0	0	0	0	0	0	0	0	0	0	0	0
ENSG00000227134	0	0	0	0	0	0	0	0	0	0	0	0	0	0	1	0	0	0	0	0	0	0	0	0	0	0	0	0	0	0	0	0
ENSG00000198526	0	0	0	0	0	0	0	0	0	1	0	0	0	0	0	1	0	0	0	0	0	0	0	0	0	0	0	0	0	0	0	0
ENSG00000271583	0	0	0	0	0	0	0	0	0	0	0	0	0	0	0	0	0	0	0	0	0	0	0	0	0	0	0	0	0	0	0	0
ENSG00000231040	0	0	0	0	0	0	0	0	0	0	0	0	0	0	0	0	0	0	0	0	0	0	0	0	0	0	0	0	0	0	0	0
ENSG00000227902	0	0	0	0	0	0	0	0	0	0	0	0	0	0	0	0	0	0	0	0	0	0	0	0	0	0	0	0	0	0	0	1
ENSG00000207225	0	0	0	0	0	0	0	0	0	0	0	0	0	0	0	0	0	0	0	0	0	0	0	0	0	0	0	0	0	0	0	0
ENSG00000207161	0	0	0	0	0	0	0	0	0	0	0	0	0	0	0	0	0	0	0	0	0	0	0	0	0	0	0	0	0	0	0	0
ENSG00000202074	0	0	0	0	0	0	0	0	0	0	0	0	0	0	0	0	0	0	0	0	0	0	0	0	0	0	0	0	0	0	0	0
ENSG00000229143	0	0	0	0	0	0	0	0	0	0	0	0	0	0	0	0	0	0	0	0	0	0	0	0	0	0	0	0	0	0	0	0
ENSG00000121989	29	49	34	40	5	13	6	13	193	184	974	137	319	451	405	596	19	47	15	71	22	9	21	10	195	247	312	567	268	601	287	679
ENSG00000212389	0	0	0	0	0	0	0	0	0	3	5	0	0	3	4	0	0	0	0	0	0	0	0	0	3	2	0	3	0	0	2	3
ENSG00000223911	0	0	0	0	0	0	0	0	0	3	5	1	1	2	2	0	0	0	0	1	0	0	0	0	4	3	5	7	0	5	0	5
ENSG00000115947	1066	741	845	700	661	611	570	785	717	626	2700	449	1916	2135	2098	2484	1253	1479	1264	1314	1849	762	1274	935	735	1019	600	1746	2064	2345	2346	2750
ENSG00000204406	514	383	436	357	486	550	338	490	505	430	2164	282	681	732	624	909	150	139	162	346	668	304	422	298	419	546	682	1405	315	563	357	685
ENSG00000281469	15	7	11	9	20	15	13	11	16	19	69	12	30	38	28	44	1	3	4	9	17	7	10	15	15	17	15	48	7	27	10	35
ENSG00000281772	4	4	3	3	0	6	1	1	5	3	15	0	5	7	10	2	4	0	1	3	6	2	3	5	8	3	5	11	0	7	1	6
ENSG00000212181	1	0	0	0	0	0	0	0	1	2	0	0	0	0	1	2	0	0	0	0	1	0	1	0	0	4	1	2	0	2	3	1
ENSG00000224007	2	0	0	1	1	0	3	3	1	2	11	1	0	1	1	4	0	1	0	2	2	6	3	3	3	9	2	8	0	1	1	3
ENSG00000232238	0	0	0	0	0	0	0	0	0	0	0	0	0	0	0	0	0	0	0	0	0	0	0	0	0	0	0	0	0	0	0	0
ENSG00000135999	533	437	561	443	590	648	500	666	745	754	3620	563	501	538	637	742	377	523	480	732	1534	647	1145	605	879	1291	986	2389	441	714	631	797
ENSG00000222126	0	0	0	0	0	0	0	0	0	0	0	0	0	1	0	1	0	0	0	0	0	0	0	0	0	0	0	0	0	0	0	0
ENSG00000223962	0	0	0	0	0	0	0	0	0	0	1	0	1	0	0	3	0	0	0	0	0	0	0	0	0	0	0	0	0	0	0	1
ENSG00000231079	1	2	1	1	0	0	0	0	25	30	95	12	50	39	26	39	0	0	0	0	0	0	0	0	20	12	28	66	7	12	15	16
ENSG00000228848	0	0	0	0	0	0	0	0	13	7	24	1	6	5	9	6	0	0	0	0	0	0	0	0	3	3	0	12	0	6	3	3
ENSG00000226340	0	0	0	0	0	0	0	0	17	5	35	5	9	9	13	21	0	0	0	0	0	0	0	0	3	4	4	18	2	12	2	8
ENSG00000168280	79	141	73	178	0	0	0	0	1137	943	4677	664	297	356	330	515	18	85	22	124	1	0	0	0	999	1429	893	2702	194	428	225	483
ENSG00000150556	0	0	0	0	0	0	0	0	0	2	1	0	0	0	1	2	0	0	0	0	0	0	0	0	0	0	0	2	0	4	2	0
ENSG00000228236	0	0	0	0	0	0	0	0	0	0	0	0	0	0	0	0	0	0	0	0	1	0	0	0	1	0	0	0	0	0	1	0
ENSG00000224337	0	0	0	0	0	0	0	0	0	0	0	0	0	0	0	0	0	0	0	0	0	0	0	0	0	0	0	0	0	0	0	0
ENSG00000187123	0	0	0	0	0	0	0	0	0	0	0	0	2	0	0	0	0	0	0	0	0	0	0	0	0	0	0	0	0	0	0	0
ENSG00000223604	0	0	0	0	0	0	0	0	0	0	0	0	0	0	0	0	0	0	0	0	0	0	0	0	0	0	0	0	0	0	0	0
ENSG00000168288	2370	1622	1861	1511	2036	2058	1566	2252	2079	1577	7106	1212	1995	1893	1962	2107	2486	3758	3468	3559	5796	2430	4476	2704	2133	3093	1576	4404	2593	2447	2748	2823
ENSG00000231969	2	1	2	1	10	3	3	4	5	2	17	0	7	3	3	8	5	2	1	2	17	10	12	15	3	8	7	23	3	5	3	4
ENSG00000207270	0	0	0	0	0	0	0	0	0	0	1	0	1	0	0	3	0	0	0	0	0	0	0	0	0	0	0	1	0	0	1	0
ENSG00000162947	0	0	0	0	0	0	0	0	0	0	0	0	0	0	0	0	0	0	0	0	0	0	0	0	0	0	0	0	0	1	0	0
ENSG00000230645	0	0	0	0	0	0	0	0	0	0	0	0	0	0	0	0	0	0	0	0	0	0	0	0	0	0	0	0	0	0	0	0
ENSG00000231420	0	1	0	0	0	0	0	0	0	0	0	0	0	0	0	0	0	0	0	0	0	0	0	0	0	0	1	0	0	0	0	0
ENSG00000115963	3	0	0	0	2	0	0	0	256	228	3047	325	644	682	858	854	0	0	1	0	0	0	0	1	272	456	605	1482	785	944	1189	1177
ENSG00000236049	0	0	0	0	0	0	0	0	0	0	1	0	0	0	0	0	0	0	0	0	0	0	0	0	0	0	1	0	1	0	0	0
ENSG00000237220	0	0	0	0	0	0	0	0	0	0	0	0	0	0	0	0	0	0	0	0	0	0	0	0	0	0	0	0	0	0	0	0
ENSG00000232359	0	0	0	0	0	0	0	0	0	0	0	0	0	0	0	0	0	0	0	0	0	0	0	0	0	0	0	0	0	0	0	0
ENSG00000224048	0	0	0	0	0	0	0	0	0	0	0	0	0	0	0	0	0	0	0	0	0	0	0	0	0	0	0	0	0	0	0	0
ENSG00000228064	0	0	0	0	1	0	0	1	0	0	0	0	0	0	0	0	0	0	0	0	0	0	0	0	0	0	0	1	0	0	0	1
ENSG00000222031	0	0	0	0	0	1	3	2	0	0	4	0	1	1	0	1	0	0	0	0	1	1	1	0	0	1	0	1	0	0	1	0
ENSG00000236885	0	0	0	0	0	0	1	1	0	2	13	1	0	0	0	0	0	0	0	0	2	2	0	0	2	1	2	4	0	1	3	1
ENSG00000213201	0	0	0	0	0	0	0	0	0	0	1	0	0	0	0	0	0	0	0	0	0	0	0	0	1	0	0	0	0	0	0	0
ENSG00000184898	317	211	217	220	402	398	269	366	264	223	853	190	284	264	205	303	250	266	165	250	692	250	433	259	170	245	246	521	99	143	114	178
ENSG00000123609	1332	801	1099	863	2505	2642	2142	2871	1510	1123	5014	841	1698	1615	1948	2232	1777	2002	2057	1583	7056	2758	5081	3070	1396	1989	1017	3110	2066	2227	2597	2827
ENSG00000123610	0	0	0	0	0	0	0	0	0	0	0	0	32	43	73	53	0	0	0	0	0	0	0	0	0	0	1	0	35	41	60	58
ENSG00000264684	0	0	0	0	0	0	0	0	0	0	0	0	0	0	0	0	0	0	0	0	0	0	0	0	0	0	0	0	0	0	0	0
ENSG00000283569	0	0	0	0	0	0	0	0	0	0	0	0	0	0	0	0	0	0	0	0	0	0	0	0	0	0	0	0	0	0	0	0
ENSG00000242113	0	0	0	0	0	0	0	0	0	0	0	0	2	0	1	4	0	0	0	0	0	0	0	0	0	0	0	0	1	1	0	1
ENSG00000080345	5748	3850	4576	3707	2277	2336	1933	2522	5351	4369	16628	3211	5552	5091	5837	6291	2819	3906	3678	5412	5532	2214	3969	2279	5530	7649	3326	13115	4659	6416	6033	6948
ENSG00000183091	149	120	126	118	90	99	76	105	139	127	454	89	192	179	141	211	14	34	21	105	194	105	122	75	117	157	125	357	43	124	77	156
ENSG00000162980	1953	1331	1449	1391	1716	1739	1300	1805	1717	1451	6338	1112	1530	1387	1550	1766	1040	1926	1442	1971	3348	1273	2212	1344	1881	2554	1204	4254	1464	1798	1415	1863
ENSG00000283228	0	0	0	0	0	0	0	0	0	0	0	0	0	0	0	0	0	0	0	0	0	0	1	0	0	0	0	0	0	2	0	1
ENSG00000182389	23	23	19	31	236	291	172	305	32	44	113	26	26	43	21	73	6	9	3	34	286	159	223	165	21	42	31	77	7	49	22	79

ENSG00000225214	0	0	0	0	1	0	0	0	0	0	3	0	0	1	0	0	0	0	0	0	0	0	0	0	0	0	4	0	0	1	0	1
ENSG00000115145	725	509	561	531	476	605	390	635	669	534	3207	457	417	387	430	573	465	592	511	666	998	434	643	482	752	974	917	1932	412	503	482	589
ENSG00000234272	2	0	1	2	0	0	1	0	3	1	7	0	0	2	1	1	1	0	1	5	1	5	4	2	1	3	6	11	0	0	0	1
ENSG00000199884	0	0	0	0	0	0	0	0	0	0	0	0	0	0	0	0	0	0	0	0	0	0	0	0	0	0	0	0	0	0	0	0
ENSG00000157827	688	791	638	793	22	14	15	18	711	623	2500	439	1024	1153	913	1422	162	350	190	642	31	9	20	30	652	1015	581	1443	637	1187	683	1209
ENSG00000213197	1	1	3	3	0	0	0	0	3	3	6	3	3	9	5	5	1	0	1	4	0	0	0	0	4	5	0	18	1	4	5	9
ENSG00000270776	1	0	1	0	0	0	0	0	0	0	0	0	0	1	0	0	1	0	1	0	0	0	0	0	0	0	0	1	1	0	0	0
ENSG00000196504	7345	4851	5740	4552	5167	5032	4213	5482	3854	3005	13704	2289	5405	4774	6024	5970	5274	6850	6547	6336	12666	5037	9051	5266	4304	6016	3280	9474	4978	5687	6412	6737
ENSG00000177917	1541	1132	1334	1268	1375	1463	1260	1653	1257	1051	3203	718	920	1014	851	1261	1129	1707	1630	1553	3173	1267	2331	1381	1092	1506	681	2563	965	999	974	1227
ENSG00000234932	0	0	0	0	0	0	0	0	0	0	0	0	0	0	0	0	0	0	0	0	0	0	0	0	0	0	0	0	0	0	0	0
ENSG00000226213	0	0	0	0	0	0	0	0	0	0	0	0	0	0	0	0	0	0	0	0	0	0	0	0	0	0	0	0	0	0	0	0
ENSG00000214025	0	0	0	0	0	0	0	0	0	0	0	0	0	0	0	0	0	0	0	0	0	0	0	0	0	0	0	0	0	0	0	0
ENSG00000224612	0	0	0	0	0	0	0	0	0	0	0	0	0	0	0	0	0	0	0	0	0	0	0	0	0	0	0	0	0	0	0	0
ENSG00000226338	0	0	0	0	0	0	0	0	0	0	0	0	0	0	0	0	0	0	0	0	1	0	0	0	0	0	0	0	0	0	0	0
ENSG00000227708	0	0	0	0	0	0	0	0	4	1	5	1	0	0	0	0	0	0	0	0	0	0	0	0	2	6	1	2	0	0	0	0
ENSG00000214024	0	0	0	0	0	0	0	0	0	2	8	0	0	0	0	0	0	0	0	0	0	0	0	0	3	3	1	6	0	0	0	0
ENSG00000242575	0	0	0	0	0	0	0	0	43	39	93	39	2	11	8	3	0	0	0	0	0	0	0	0	29	57	18	95	4	12	9	6
ENSG00000227400	0	0	0	0	0	0	0	0	30	45	57	16	4	3	4	10	0	0	0	0	0	0	0	0	23	34	15	84	2	8	2	11
ENSG00000200377	0	0	0	0	0	0	0	0	1	1	2	0	2	2	1	0	0	0	0	0	0	0	0	0	0	0	0	1	0	3	5	0
ENSG00000177519	1	2	0	4	0	0	0	0	228	198	531	176	6	7	0	11	0	5	1	5	0	0	0	1	116	269	104	341	1	4	8	4
ENSG00000236917	0	0	0	0	0	0	0	0	0	0	0	0	0	0	0	0	0	0	0	0	0	0	0	0	0	0	0	0	0	0	1	0
ENSG00000144278	1	0	3	1	0	1	1	0	0	1	3	0	1	8	1	4	2	1	1	0	3	2	1	0	0	0	0	0	1	1	0	2
ENSG00000230251	0	0	0	0	0	0	0	0	0	0	0	0	0	0	0	0	0	0	0	0	0	0	0	0	0	0	0	0	0	0	0	0
ENSG00000224675	0	0	0	0	0	0	0	0	0	0	1	0	0	0	0	0	0	0	0	0	0	0	0	0	0	0	3	0	0	0	0	0
ENSG00000238004	0	0	0	0	0	0	0	0	0	0	0	0	0	0	0	0	0	0	0	0	0	0	0	0	0	0	0	0	0	0	0	0
ENSG00000235949	0	0	0	0	0	0	0	0	0	0	0	0	0	0	0	0	0	0	0	0	0	0	0	0	0	0	0	0	0	0	0	0
ENSG00000162989	0	0	0	0	0	0	1	0	0	0	1	0	0	1	0	1	0	0	0	1	0	0	0	0	0	0	0	0	0	0	0	0
ENSG00000228837	0	0	0	0	0	0	0	0	0	0	0	0	0	0	0	0	0	0	0	0	0	0	0	0	0	0	0	0	0	0	0	0
ENSG00000230991	0	0	0	0	0	0	0	0	0	1	2	0	0	0	0	0	0	0	0	0	0	0	0	0	0	0	2	0	0	0	0	0
ENSG00000223197	0	0	0	0	0	0	0	0	0	0	0	0	0	0	0	0	0	0	0	0	0	0	0	0	0	0	0	0	0	0	1	0
ENSG00000232982	0	0	0	0	0	0	0	0	0	0	0	0	0	0	0	0	0	0	0	0	0	0	0	0	0	0	0	0	0	0	0	0
ENSG00000229701	0	0	0	0	0	0	0	0	0	0	0	0	0	0	0	0	0	0	0	0	0	0	0	0	0	0	0	0	0	0	0	0
ENSG00000227682	0	0	0	0	0	0	0	0	0	0	0	0	0	0	0	0	0	0	0	0	0	1	0	0	0	0	1	0	0	0	0	0
ENSG00000233709	0	0	0	0	0	0	0	0	0	0	0	0	0	0	0	0	0	0	0	0	0	0	0	0	0	0	0	0	0	0	0	0
ENSG00000233039	0	0	0	0	0	0	0	0	0	0	0	0	0	0	0	0	0	0	0	0	0	0	0	0	0	0	0	0	0	0	0	0
ENSG00000233248	0	0	0	0	0	0	0	0	0	0	0	0	0	0	0	0	0	0	0	0	0	0	0	0	0	0	1	0	0	0	0	0
ENSG00000229935	0	0	0	0	0	0	0	0	0	0	0	0	0	0	0	0	0	0	0	0	0	0	0	0	0	0	0	0	0	0	0	0
ENSG00000206718	0	0	0	0	0	0	0	0	0	0	0	0	0	0	0	0	0	0	0	0	0	0	0	0	0	0	0	0	0	0	0	0
ENSG00000226383	0	0	0	0	0	0	0	0	0	0	0	0	0	0	0	1	0	0	0	1	0	0	0	0	0	0	0	0	0	0	1	0
ENSG00000235548	0	0	0	0	0	0	0	0	0	0	0	0	0	0	0	0	0	0	0	0	0	0	0	0	0	0	0	0	0	0	0	0
ENSG00000282440	0	0	0	0	0	0	0	0	0	0	0	0	0	0	0	0	0	0	0	0	0	0	0	0	0	0	0	0	0	0	1	0
ENSG00000153234	4	20	6	8	65	135	59	139	240	168	1329	174	135	109	271	207	0	2	0	8	111	61	53	46	189	250	245	537	69	96	123	120
ENSG00000115159	257	270	255	290	6	6	5	7	683	576	2209	397	20	11	9	21	109	259	199	336	8	2	6	5	643	836	509	1794	7	7	3	13
ENSG00000283436	0	0	0	0	0	0	0	0	0	5	238	20	1	0	0	0	0	0	0	0	0	0	0	0	9	9	46	100	0	1	0	0
ENSG00000238069	0	0	0	0	0	0	0	0	0	0	49	2	0	0	0	0	0	0	0	0	0	0	0	0	0	0	6	7	0	0	0	0
ENSG00000213194	0	0	0	0	1	0	0	0	0	0	0	0	0	0	0	0	0	1	0	0	0	0	0	0	0	0	0	0	0	0	0	0
ENSG00000136542	0	0	0	0	0	0	0	0	0	0	5	1	3	0	4	4	0	0	0	0	0	2	1	0	0	0	3	1	0	1	0	2
ENSG00000222404	0	0	0	0	0	0	0	0	0	0	0	0	0	0	0	0	0	0	0	0	0	0	0	0	0	0	0	0	0	0	0	0
ENSG00000136541	0	0	0	0	5	5	0	2	126	116	530	68	128	114	117	157	0	0	0	0	8	4	2	1	55	134	126	263	40	125	79	114
ENSG00000230562	14	4	7	8	25	22	21	35	15	12	70	17	17	7	16	24	30	29	29	26	97	45	53	28	26	34	19	37	12	26	24	27
ENSG00000115165	1	0	1	2	39	25	28	20	793	650	2999	497	1666	1687	2245	2272	6	4	2	3	84	20	70	20	782	1144	987	2265	1969	1995	2895	2386
ENSG00000123612	2	3	0	4	3	1	1	4	5	11	24	4	88	144	170	177	1	0	0	0	0	1	0	1	7	12	3	11	63	131	136	170
ENSG00000234584	2	2	1	3	3	0	1	0	1	2	3	0	0	0	2	1	0	1	2	2	2	2	2	1	1	2	2	2	0	4	1	2
ENSG00000115170	213	199	245	183	172	238	182	233	158	127	784	106	91	116	115	113	66	75	93	163	323	170	210	150	167	275	222	479	82	77	104	159
ENSG00000007001	2	2	4	4	2	3	3	10	1	3	11	0	1	1	0	4	2	6	6	3	3	1	2	1	0	1	2	5	0	1	2	4
ENSG00000270557	21	14	13	11	10	10	1	1	13	10	61	3	6	15	4	15	3	16	3	6	4	4	5	2	6	12	15	17	4	4	2	9
ENSG00000251980	1	0	0	0	0	0	0	0	0	0	0	0	0	0	0	0	0	0	0	0	0	0	0	0	0	0	0	0	0	0	0	0
ENSG00000271320	0	0	0	1	0	0	0	1	0	0	1	0	0	0	0	0	0	0	1	0	0	0	0	0	0	0	0	0	0	0	0	0
ENSG00000228937	0	0	0	0	0	0	0	0	0	0	0	0	0	0	0	0	0	0	0	0	1	0	0	0	0	0	0	0	0	0	0	0
ENSG00000212410	0	0	0	0	0	0	0	0	0	0	0	0	0	0	0	0	0	0	0	0	0	0	0	0	0	0	0	0	0	0	0	0
ENSG00000231158	0	0	0	0	0	0	0	0	0	0	0	0	0	0	0	0	0	0	0	0	0	0	0	0	0	0	0	0	0	0	0	0
ENSG00000237327	0	0	0	0	0	0	0	0	0	0	0	0	0	0	0	1	0	0	0	0	1	0	0	1	0	0	0	0	0	0	0	0
ENSG00000227480	0	0	0	0	0	2	0	1	0	0	0	0	0	0	0	0	0	0	0	1	0	0	2	0	0	0	0	0	0	0	0	0
ENSG00000153237	4	9	5	5	5	4	10	7	2	7	7	2	9	7	25	13	4	11	6	8	12	3	12	5	1	1	0	1	6	12	19	19
ENSG00000234890	1	0	0	1	0	0	0	0	0	0	0	0	0	0	0	0	0	0	0	0	0	0	0	0	0	0	0	0	0	0	0	0
ENSG00000243723	0	0	0	0	0	0	0	0	0	0	1	0	1	0	0	0	0	0	0	0	0	0	0	1	0	0	0	0	0	0	0	0
ENSG00000144283	2332	2240	1961	2415	2501	2655	1924	2952	1654	1451	4791	1095	2484	2362	2841	3226	693	1277	850	1785	3666	1583	2744	1318	1541	2603	763	4176	1676	2452	2098	2736
ENSG00000227331	5	2	4	4	4	8	3	3	2	1	11	0	5	4	2	1	1	1	2	7	11	4	6	5	6	5	3	16	0	7	1	11
ENSG00000281472	0	6	0	2	2	3	0	1	0	2	4	0	1	1	2	0	2	0	0	0	4	1	0	5	3	2	0	6	0	3	3	3
ENSG00000204380	38	34	26	36	41	29	18	31	15	16	57	11	17	25	36	37	7	12	5	36	39	27	24	27	36	31	21	70	5	43	33	53
ENSG00000228586	0	0	0	0	1	0	0	0	0	0	0	0	0	0	0	0	0	0	0	0	0	0	1	0	0	0	0	0	0	1	1	2
ENSG00000231675	0	0	0	0	0	0	0	0	0	0	0	0	0	0	0	0	0	0	0	0	0	0	0	0	0	0	0	0	0	0	0	0
ENSG00000163331	0	0	0	0	2	6	8	9	0	0	0	0	5	6	4	4	0	0	0	0	4	2	5	1	0	0	1	0	24	25	43	44
ENSG00000243792	0	0	0	0	0	0	0	1	0	0	0	0	0	0	0	1	0	0	0	0	0	0	0	0	0	0	0	0	0	2	1	1
ENSG00000251491	0	0	0	0	0	0	0	0	0	0	0	0	0	0	0	0	0	0	0	0	0	0	0	0	0	0	0	0	0	0	1	0
ENSG00000188668	0	0	0	0	0	0	0	0	0	0	0	0	0	0	0	0	0	0	0	0	0	0	0	0	0	0	0	0	0	0	0	0
ENSG00000222300	0	0	0	0	0	0	0	0	0	0	0	0	0	0	0	0	0	0	0	0	0	0	0	0	0	0	0	0	0	0	0	0
ENSG00000115183	585	783	566	675	370	441	248	430	193	191	780	172	76	89	52	110	140	243	198	487	191	88	154	98	44	116	108	481	23	64	27	83
ENSG00000213189	13	4	5	9	7	6	2	3	6	4	26	4	9	5	11	5	15	18	12	21	21	7	8	10	3	8	8	10	7	9	15	8
ENSG00000202029	1	0	0	0	0	0	0	0	0	0	0	0	0	0	0	0	0	0	0	0	1	0	0	0	0	0	0	0	0	0	0	0
ENSG00000226878	0	1	0	1	0	0	1	0	0	1	0	0	0	0	0	0	0	0	0	2	0	0	0	0	0	0	0	1	0	0	0	0
ENSG00000275141	0	0	0	0	2	0	1	0	0	0	0	0	1	0	0	0	0	0	0	0	0	1	0	0	0	0	0	0	0	0	0	0
ENSG00000196151	456	315	350	316	298	350	251	337	226	193	821	138	52	55	49	62	380	449	381	416	544	252	380	220	179	289	221	585	24	45	32	39
ENSG00000123636	176	134	161	156	937	976	713	1038	647	456	3061	320	533	610	609	805	88	106	105	249	1583	715	1161	677	443	561	822	1385	299	666	545	712
ENSG00000223642	0	0	1	0	3	7	9	6	3	5	11	1	3	4	5	2	0	0	0	0	10	11	7	17	7	2	5	11	2	9	3	3
ENSG00000270893	0	0	0	0	10	2	1	2	1	0	3	0	0	2	2	1	0	0	0	0	1	3	0	1	2	1	3	5	0	2	1	0
ENSG00000225369	0	0	0	0	0	0	0	0	0	0	0	0	0	0	0	0	0	0	0	0	0	0	0	0	0	0	0	0	0	0	0	0
ENSG00000207117	2	1	3	3	2	0	0	2	0	0	0	0	0	0	0	0	0	2	0	3	6	1	1	0	0	3	0	6	0	0	0	1
ENSG00000224152	36	36	31	24	31	24	28	26	15	10	75	14	5	12	8	9	6	10	12	29	60	29	43	31	15	16	14	37	3	4	3	10
ENSG00000226266	85	63	56	64	30	33	16	26	15	23	70	12	14	11	4	9	37	31	48	68	49	31	36	27	17	21	14	42	8	10	5	8
ENSG00000230783	10	4	6	1	2	5	1	6	3	3	17	3	0	2	0	3	3	3	0	4	3	3	5	1	5	2	6	7	0	0	1	2
ENSG00000136536	2521	1704	2029	1647	2360	2537	1803	2901	1920	1617	7386	1272	1760	1656	1879	2082	1955	2223	2323	2448	6237	2447	4032	2553	1771	2640	2003	4934	1678	1954	2126	2416
ENSG00000241399	11	8	4	5	37	44	25	54	7	7	41	0	23	15	6	9	2	2	4	9	66	25	32	28	15	19	10	38	7	7	9	18
ENSG00000248672	0	0	0	0	9	12	5	7	0	0	0	0	2	4	3	4	0	0	0	0	7	5	6	3	0	0	0	0	0	1	1	3
ENSG00000054219	0	0	0	0	347	345	267	404	2	5	19	5	621	579	603	708	0	0	0	1	458	207	313	193	10	6	11	12	292	459	361	507
ENSG00000227055	0	0	0	0	1	2	5	4	0	0	2	1	4	7	7	7	0	0	0	0	0	1	6	3	0	0	1	0	2	8	3	7
ENSG00000153246	0	0	0	0	3	8	1	19	0	0	0	0	0	0	1	1	0	0	0	0	25	7	12	10	1	1	3	2	0	0	0	0
ENSG00000115221	4	4	4	3	6	1	0	6	10	4	22	5	4	6	9	3	2	4	2	3	4	4	6	9	10	9	2	20	3	5	7	6
ENSG00000153250	1331	1022	1343	1008	2198	2302	1797	2454	3182	2542	12299	1702	3066	3085	3839	3918	451	535	737	1009	4005	1771	2990	1720	3278	4607	3456	8693	2990	4065	3193	4264
ENSG00000279844	0	0	0	0	0	0	0	0	0	0	2	0	0	0	0	0	0	0	0	0	0	0	0	0	0	0	0	0	0	0	0	0
ENSG00000244372	0	0	0	0	0	0	0	0	0	0	0	0	0	0	0	0	0	0	0	0	0	0	0	0	0	0	0	0	0	0	0	0
ENSG00000232337	0	3	0	0	0	0	0	0	2	3	3	1	0	1	0	0	0	0	0	0	0	0	0	0	2	2	4	4	0	0	0	0
ENSG00000224467	0	0	0	0	0	0	0	0	8	6	26	5	3	5	0	5	0	0	0	0	0	1	1	0	9	6	12	32	1	4	1	6
ENSG00000136560	851	581	731	612	699	805	572	885	2183	1912	14149	1792	1197	1164	1396	1639	888	1156	991	974	1703	692	1237	877	2120	2965	4775	8389	1269	1482	1774	1886
ENSG00000235724	97	62	97	87	24	17	12	28	34	42	257	23	9	8	23	15	56	50	72	91	34	13	24	24	26	48	65	155	0	7	11	14
ENSG00000227403	238	167	270	171	79	99	58	80	228	177	802	133	0	1	1	2	238	338	342	365	202	85	179	82	175	205	151	397	0	2	1	0
ENSG00000225813	1	0	0	2	0	0	0	0	0	0	2	0	0	0	0	0	1	0	0	0	0	0	0	0	0	0	0	2	0	0	0	3
ENSG00000115233	2704	1917	2153	1843	1937	1996	1528	2038	2225	1569	6767	1298	2277	2024	2214	2713	4749	5941	4398	4162	5589	2208	3756	2459	2236	3414	1249	4852	2846	2703	3324	3359
ENSG00000232320	1	1	3	2	0	1	2	1	2	1	4	0	0	1	1	2	0	0	1	2	1	1	1	1	0	2	3	8	0	0	0	3
ENSG00000136535	0	4	1	2	5	4	3	4	1	3	21	0	0	2	0	2	3	2	1	7	7	1	1	3	5	6	5	23	0	2	1	1
ENSG00000224076	0	0	0	0	0	0	0	0	0	0	0	0	0	0	0	0	0	0	0	0	0	0	0	0	0	0	0	0	0	0	0	0
ENSG00000251621	0	0	0	0	0	0	0	1	0	0	2	0	0	0	0	0	0	0	0	0	0	0	0	0	0	0	0	0	0	0	0	0
ENSG00000144290	0	0	0	0	3	3	0	1	308	225	885	176	5	10	11	16	0	0	0	0	14	5	7	5	230	261	142	358	4	4	7	13
ENSG00000280105	0	0	0	0	0	0	0	0	0	0	1	0	0	0	0	0	0	0	0	0	0	0	0	1	0	2	1	0	0	0	0	0
ENSG00000226121	17	6	10	9	4	10	3	6	2	2	8	4	8	2	2	7	1	1	6	3	14	0	12	2	9	6	3	17	3	4	2	4
ENSG00000238570	0	0	0	0	0	0	0	0	0	0	0	0	0	0	0	0	0	0	0	0	0	0	0	0	0	0	0	0	0	0	0	0
ENSG00000226946	0	0	0	0	0	0	0	0	0	0	0	0	0	0	0	0	0	0	0	0	0	0	0	0	0	0	0	0	0	0	0	0
ENSG00000235716	0	0	0	0	0	0	0	0	11	0	13	1	0	0	0	0	0	0	0	0	0	0	0	0	3	1	2	3	0	0	0	0
ENSG00000234732	0	0	0	0	0	0	0	0	1	0	0	0	0	0	0	0	0	0	0	0	0	0	0	0	0	0	0	0	0	0	0	0
ENSG00000197635	0	0	0	0	0	0	0	1	11722	9448	24605	5371	434	472	327	613	0	0	0	1	1	0	0	0	8984	13095	7483	17506	100	199	106	225
ENSG00000230918	0	0	0	0	0	0	0	0	77	76	219	34	4	0	0	2	0	0	0	0	0	0	0	0	67	83	57	124	2	0	0	1
ENSG00000231445	0	0	0	0	0	0	0	0	52	51	161	36	1	1	1	1	0	0	0	0	0	0	0	0	51	58	50	96	0	0	0	0
ENSG00000224674	0	0	0	0	0	0	0	0	0	0	2	1	0	1	0	0	0	0	0	0	0	0	0	0	1	0	1	0	0	0	0	0
ENSG00000233397	0	0	0	0	0	0	0	0	0	4	0	0	0	0	0	0	0	0	0	0	0	0	0	0	0	0	0	0	0	0	0	0
ENSG00000115263	0	0	0	0	0	0	0	0	0	0	1	0	0	4	0	0	0	0	0	0	0	0	0	0	0	0	0	2	0	0	0	0
ENSG00000236841	0	0	0	0	0	0	0	0	2	1	1	0	1	0	7	0	0	1	0	0	0	0	0	0	0	1	0	0	0	1	0	0
ENSG00000078098	1	0	0	1	0	0	0	1	0	0	2	0	0	1	4	3	0	0	0	0	1	0	0	0	0	1	0	0	1	5	8	1
ENSG00000115267	187	202	152	156	297	312	177	333	422	361	1579	251	1371	1351	1228	1551	46	104	70	159	312	127	192	111	327	593	372	983	812	1045	848	1112
ENSG00000115271	183	232	124	187	74	76	61	96	534	438	1493	317	591	693	556	862	128	305	154	240	136	50	87	50	388	468	318	919	491	621	614	769
ENSG00000184611	0	0	0	0	0	2	0	0	1	1	7	0	1	1	1	3	0	0	1	0	0	0	0	0	0	0	2	1	0	2	4	4
ENSG00000237750	0	0	0	0	0	0	0	0	0	0	0	0	0	0	0	0	0	0	0	0	0	0	0	0	0	0	0	0	0	0	0	0
ENSG00000230282	0	0	0	0	0	0	0	0	0	0	1	0	0	0	1	0	0	0	0	0	0	0	0	0	0	0	0	0	0	0	0	0
ENSG00000200902	0	0	0	0	0	0	0	0	0	0	0	0	0	0	0	0	0	0	0	0	0	0	0	0	0	0	0	0	0	0	0	0
ENSG00000182263	0	0	0	0	0	0	0	0	0	0	2	0	1	0	0	0	0	0	0	0	0	0	1	0	0	1	4	0	0	0	0	0
ENSG00000237844	0	0	0	0	0	0	0	0	0	0	0	0	0	0	0	0	0	0	0	0	0	0	0	0	0	0	0	0	0	0	0	0
ENSG00000226847	0	0	0	0	0	0	0	0	0	0	0	0	0	0	0	0	0	0	0	0	0	0	0	0	0	0	0	0	0	0	0	0
ENSG00000229641	0	0	0	0	0	0	0	0	0	0	0	0	0	0	0	0	0	0	0	0	0	0	0	0	0	0	0	0	0	0	0	0
ENSG00000115290	0	0	0	0	0	0	0	1	0	0	0	0	0	0	0	0	0	0	0	0	4	0	0	2	1	0	0	0	0	0	0	1
ENSG00000277998	1	4	0	5	2	0	1	0	2	0	1	0	0	0	0	2	4	3	6	1	4	0	1	0	3	3	1	1	2	0	0	1
ENSG00000082438	1	8	0	11	1	2	0	0	57	72	313	64	0	0	0	0	3	3	1	5	1	2	2	0	66	105	44	233	0	0	0	0
ENSG00000224331	0	0	0	0	0	0	0	0	0	0	0	0	0	0	0	0	0	0	0	0	1	0	0	0	0	1	0	1	0	0	0	0
ENSG00000206869	0	0	0	0	0	0	0	0	0	0	0	0	0	0	0	0	0	0	0	0	0	0	0	0	0	0	0	1	0	0	0	0
ENSG00000233255	0	0	0	0	0	0	0	0	0	0	0	0	0	0	0	0	0	0	0	0	0	0	0	0	0	0	0	2	0	0	0	0
ENSG00000169507	0	0	0	0	0	1	0	1	0	0	7	0	0	0	0	0	0	0	0	0	4	1	0	0	0	0	1	0	0	0	0	0
ENSG00000236283	0	1	0	0	3	2	1	3	1	4	53	3	0	0	0	0	0	0	0	0	0	5	5	4	0	7	8	17	0	0	0	0
ENSG00000153253	24	22	18	21	131	214	209	328	660	671	14887	1174	1	3	2	3	16	27	23	43	189	94	191	84	1028	1250	2287	5320	1	1	0	1
ENSG00000136531	7	0	7	3	0	5	3	5	300	182	1465	122	2	2	1	1	8	8	12	9	8	7	8	7	557	616	601	1071	0	3	3	11
ENSG00000233204	0	0	0	0	0	0	0	0	0	0	0	0	0	0	0	0	0	0	0	0	0	0	0	0	0	0	0	0	0	0	0	0
ENSG00000178662	0	0	0	0	4	8	2	4	0	0	2	0	0	1	0	0	0	0	0	0	11	3	8	5	0	1	2	0	0	0	0	0
ENSG00000115339	65	83	68	90	428	531	374	536	148	123	561	100	108	199	106	215	58	134	43	122	868	322	524	302	183	234	200	429	84	147	96	205
ENSG00000235192	4	3	2	4	47	45	27	70	8	2	31	2	2	1	1	2	2	1	1	8	49	24	48	35	10	9	8	22	1	2	1	4
ENSG00000229195	5	3	3	0	18	9	10	7	5	3	14	0	0	0	0	0	2	1	0	3	17	2	9	7	1	2	1	11	1	2	0	0
ENSG00000232411	3	1	3	6	12	12	6	16	0	1	16	1	0	0	0	0	2	0	0	11	14	5	5	3	5	1	3	13	0	0	0	0
ENSG00000123607	1463	1087	1085	1039	1310	1433	1024	1429	771	592	2564	432	653	643	598	741	637	820	835	994	2542	950	1645	1033	632	976	540	1771	436	680	604	800
ENSG00000224490	12	19	21	17	8	21	4	13	16	7	55	13	6	6	5	12	4	6	2	20	37	18	16	23	18	18	7	30	3	12	7	12
ENSG00000236107	0	0	0	0	0	2	0	0	1	2	7	0	0	0	0	0	0	0	0	0	0	0	0	0	0	7	0	5	0	0	1	0
ENSG00000144285	0	0	0	2	3	6	0	3	0	0	3	1	0	0	0	1	1	0	0	2	3	2	2	0	0	2	1	0	0	0	0	0
ENSG00000238813	0	0	0	0	0	0	0	0	0	0	0	0	0	0	0	0	0	0	0	0	0	0	0	0	0	0	0	0	0	0	0	0
ENSG00000222376	0	0	0	0	0	0	0	0	0	0	0	0	0	0	0	0	0	0	0	0	0	0	0	0	0	0	0	0	0	0	0	0
ENSG00000169432	0	0	3	0	0	0	0	0	300	264	1008	187	0	1	0	0	1	7	2	6	0	0	0	0	196	351	214	649	0	0	0	1
ENSG00000136546	0	1	0	1	0	4	3	3	0	0	1	0	0	0	0	0	0	1	2	1	3	2	1	1	0	0	0	0	0	1	0	0
ENSG00000283657	0	0	0	0	0	0	0	0	0	0	0	0	0	0	0	0	0	0	0	0	0	0	0	0	0	1	0	0	0	0	0	0
ENSG00000163092	0	0	0	0	14	19	29	32	0	0	0	0	0	0	0	0	0	0	0	0	80	20	86	27	0	0	0	0	0	0	0	0
ENSG00000225182	0	0	0	0	0	0	0	0	0	0	0	0	0	0	0	0	0	0	0	0	0	0	0	0	0	0	0	0	0	0	0	0
ENSG00000254552	0	0	0	0	0	0	0	0	0	0	0	0	0	0	0	0	0	0	0	0	0	0	1	0	0	0	0	0	0	0	0	0
ENSG00000228222	0	0	0	1	0	0	2	0	0	0	0	2	0	0	0	0	0	0	0	1	1	2	0	0	0	0	0	0	0	0	0	0
ENSG00000238357	0	0	0	0	0	0	0	0	0	0	0	0	0	0	0	0	0	0	0	0	0	0	0	0	0	0	0	0	0	0	0	0
ENSG00000214211	0	0	0	0	0	0	0	0	0	0	0	0	0	0	0	0	0	0	0	0	2	0	0	0	0	0	0	0	0	0	0	0
ENSG00000235335	0	1	2	0	1	0	0	0	0	1	0	0	0	0	0	0	1	1	5	3	2	4	2	2	0	0	0	0	0	0	0	0
ENSG00000172318	3	2	1	7	4	0	1	0	0	0	7	1	0	0	0	0	0	2	3	10	4	2	6	6	0	1	2	2	0	0	0	0
ENSG00000198648	2961	2391	2382	2283	2039	1957	1476	2065	1825	1383	5890	1080	1357	1354	1429	1722	992	1706	1485	1922	3636	1424	2927	1429	2043	2986	1241	4549	958	1224	1046	1416
ENSG00000238259	6	10	8	5	0	3	0	4	1	1	19	1	4	4	3	2	1	0	0	13	11	8	5	11	8	7	0	16	0	3	2	4
ENSG00000241074	0	0	1	1	0	1	0	0	0	0	1	0	0	0	0	0	0	0	0	0	3	0	4	1	0	0	0	1	3	0	0	0
ENSG00000172292	2563	2024	2337	1925	1776	1913	1375	1900	1095	1033	5293	794	870	790	1010	970	759	1317	1059	1854	3117	1258	2277	1063	1068	1708	1024	3278	682	976	813	1016
ENSG00000265694	2	1	2	0	0	1	0	0	0	0	6	0	0	0	2	0	0	0	6	1	0	3	2	2	2	0	0	12	0	2	1	2
ENSG00000199348	2	0	1	1	0	0	0	0	0	2	4	0	0	1	1	3	0	0	0	4	1	2	2	2	0	1	1	7	0	0	0	1
ENSG00000227617	4	7	0	7	3	1	3	3	0	0	15	3	1	4	1	1	2	2	0	2	3	2	1	3	7	1	0	5	0	3	3	1
ENSG00000163072	133	126	133	117	97	98	73	92	170	147	528	65	51	75	61	83	38	45	33	125	130	73	80	77	141	185	37	428	26	96	51	115
ENSG00000152253	1274	882	1142	973	687	787	621	825	819	599	1953	480	340	397	436	503	1421	1726	1631	1485	1941	712	1426	894	953	1203	249	2049	413	528	546	609
ENSG00000152254	0	0	0	0	1	0	0	0	0	0	2	0	0	0	0	0	0	0	0	0	0	0	0	0	0	0	0	0	0	0	0	0
ENSG00000073734	0	0	0	0	3	4	4	0	0	0	1	0	0	0	0	1	0	0	0	0	2	0	0	0	0	0	0	2	0	0	0	0
ENSG00000073737	0	0	0	0	10	8	4	5	0	0	0	0	0	0	0	0	0	0	0	0	12	4	2	3	0	0	0	0	0	0	0	0
ENSG00000235321	0	0	0	0	1	0	0	0	0	0	0	0	0	0	0	0	0	0	0	0	0	0	0	0	0	0	0	0	0	0	0	0
ENSG00000235995	0	0	0	0	0	0	0	0	0	0	0	0	0	0	0	0	0	0	0	0	0	0	0	0	0	0	0	0	0	0	0	0
ENSG00000081479	4	24	4	27	0	0	1	0	3383	2747	22723	4404	50	115	59	154	0	2	0	2	1	0	0	1	4348	7615	2260	21549	61	253	76	279
ENSG00000163093	221	155	180	178	115	115	86	137	107	101	433	79	153	143	174	197	66	97	98	160	196	74	119	72	93	124	101	234	132	168	134	164
ENSG00000251569	0	0	0	0	0	0	0	0	0	0	0	0	0	0	0	0	0	0	0	0	0	0	1	0	0	0	0	0	0	1	0	0
ENSG00000239474	6	12	8	1	9	5	6	6	10	6	34	1	13	5	4	13	4	5	8	19	8	5	11	1	7	10	5	12	5	12	7	16
ENSG00000138399	1380	889	1174	905	1142	1094	842	1162	1000	780	3047	551	740	702	739	871	1221	1442	1250	1420	2337	958	1321	1021	948	1366	662	1875	832	921	909	1034
ENSG00000229827	0	0	0	0	0	0	1	0	0	0	1	0	0	1	0	0	0	0	0	0	0	0	0	0	0	0	0	0	0	0	0	1
ENSG00000138398	5546	3464	4170	3697	3930	4098	3592	4520	3101	2214	9759	1762	3268	3009	3742	3824	4817	5094	4800	4917	10121	4200	7105	4289	2765	3894	2076	6728	3397	3629	4262	4398
ENSG00000154479	36	33	27	20	12	13	14	18	10	7	30	3	7	4	7	12	20	32	22	23	20	11	5	8	14	17	12	20	18	21	31	23
ENSG00000226072	0	0	0	0	1	0	0	0	0	0	0	0	0	2	0	0	0	0	0	1	0	1	0	0	0	1	0	1	0	0	0	0
ENSG00000144362	152	81	115	95	57	56	34	55	128	122	506	76	98	94	76	91	75	106	97	160	119	54	98	42	104	137	128	243	67	100	64	108
ENSG00000213160	1832	1399	1640	1453	1572	1699	1301	1758	875	797	2493	547	936	986	1043	1273	972	1624	1517	1764	3156	1155	2086	1134	836	1245	453	1860	958	1166	966	1273
ENSG00000206961	1	1	0	0	0	1	0	0	0	1	4	0	1	2	0	0	0	0	0	0	1	0	0	0	0	0	0	2	0	0	2	0
ENSG00000231550	27	32	26	20	17	16	9	17	4	5	18	9	16	9	11	7	9	8	5	29	30	16	12	11	8	7	3	18	1	12	5	13
ENSG00000214369	12	14	9	15	3	4	3	6	2	2	7	1	2	3	2	1	4	3	1	11	10	1	1	2	0	3	1	2	0	0	1	7
ENSG00000138385	6056	3683	4616	3945	4682	4533	3873	5034	3306	2276	9627	1725	3929	3482	4153	4488	8744	9814	7862	7234	11635	4514	8060	4904	3216	4451	2027	6251	5148	4580	5510	5871
ENSG00000138382	1566	1093	1197	1036	700	807	550	844	678	535	1876	390	1151	1002	1070	1185	2168	2691	2176	2149	1903	790	1575	1054	679	928	408	1299	1331	1170	1419	1453
ENSG00000144357	1525	1168	1341	1058	1110	1208	895	1233	1018	933	4490	680	880	892	880	1098	550	784	829	1298	2461	1078	1676	1031	1108	1748	1281	3430	678	1050	942	1097
ENSG00000227176	8	2	5	6	14	9	7	3	4	1	23	2	6	2	5	2	0	3	1	4	9	9	3	4	3	2	4	18	2	3	1	5
ENSG00000252807	0	0	0	0	0	0	0	0	0	0	0	0	0	0	0	0	0	0	0	0	0	0	0	0	0	0	0	0	0	0	0	0
ENSG00000071909	0	0	1	1	115	137	82	153	1	0	33	6	18	27	25	34	1	0	6	1	122	69	112	65	1	2	17	12	4	6	3	13
ENSG00000231898	0	0	1	0	1	2	0	2	0	0	0	0	1	1	0	0	0	0	0	0	2	1	2	0	0	0	2	1	0	0	0	0
ENSG00000214821	0	0	0	0	3	1	0	0	0	0	0	0	3	0	0	0	0	0	0	0	1	0	0	0	0	0	0	0	0	0	0	0
ENSG00000213981	1	1	0	0	0	0	0	0	0	0	2	1	0	1	0	1	0	0	0	0	0	0	2	0	0	0	3	2	0	0	0	1
ENSG00000275414	0	0	0	0	0	0	0	0	0	0	0	0	0	0	0	0	0	0	0	0	0	0	0	0	0	0	0	0	0	0	0	0
ENSG00000222033	0	0	0	0	0	0	0	1	1	0	0	0	1	2	1	1	0	0	0	0	0	0	0	0	0	0	1	0	0	1	1	3
ENSG00000204335	0	1	0	1	0	0	0	0	0	0	2	0	3	9	0	11	0	0	2	0	0	0	0	0	0	0	0	3	2	2	7	17
ENSG00000234350	0	6	1	2	0	0	0	0	2	2	7	0	2	0	3	6	0	0	0	0	0	0	0	0	1	1	0	10	1	1	0	4
ENSG00000128692	17	16	13	12	9	5	4	2	8	3	20	3	12	10	12	13	21	19	15	14	24	14	15	3	6	12	3	13	11	11	20	14
ENSG00000239467	1	15	0	13	0	0	0	0	6	11	31	6	8	23	4	33	0	7	0	11	0	0	0	0	3	6	3	21	4	10	1	12
ENSG00000222509	0	0	0	1	0	0	0	0	0	0	0	0	1	0	2	0	0	0	0	0	0	0	0	0	0	0	0	0	0	0	0	0
ENSG00000204334	0	3	2	1	0	0	0	0	3	2	4	2	2	8	1	6	0	0	1	1	0	0	0	0	0	1	4	5	0	4	2	5
ENSG00000128683	1	11	0	12	63	103	60	112	24	56	84	31	185	245	202	289	0	2	2	5	136	132	109	96	6	49	16	99	71	176	95	197
ENSG00000235934	1	1	0	0	3	0	1	1	1	2	2	0	17	11	4	12	0	0	0	0	1	0	0	0	0	2	0	3	1	11	1	8
ENSG00000115806	2516	1738	2069	1632	2091	2088	1539	2250	1641	1246	5728	1090	2488	2097	2428	2617	768	1168	1157	1667	3413	1379	2571	1199	1569	2454	1319	3916	2011	2283	1963	2455
ENSG00000198586	2291	1829	2015	1922	3598	3723	2926	3936	1954	1732	7567	1244	1653	1653	1754	2074	686	1242	1068	1692	7208	3039	5435	3014	2266	3190	2076	5581	1417	1741	1552	1956
ENSG00000123600	979	710	788	848	646	630	524	704	464	374	1999	304	1125	949	1002	1145	690	904	741	959	1126	429	826	416	414	649	360	987	773	974	787	1016
ENSG00000224553	0	0	2	0	0	0	1	0	1	0	1	0	0	1	0	0	1	0	0	0	2	1	0	0	1	0	0	1	0	1	1	2
ENSG00000115827	1352	827	1030	883	1257	1243	1029	1368	889	777	3706	582	1295	1176	1388	1484	614	741	677	1115	2588	1141	2029	1182	1001	1408	872	2390	1076	1342	1205	1458
ENSG00000228507	4	2	7	5	7	10	12	8	0	1	5	0	5	3	3	7	4	9	4	6	13	8	13	9	0	7	2	3	1	0	2	1
ENSG00000234061	4	1	3	0	2	0	2	3	1	0	1	0	1	0	0	0	1	1	0	0	9	0	5	2	1	0	0	0	0	0	0	3
ENSG00000233762	33	19	24	16	28	13	9	23	27	20	111	22	43	48	38	64	36	35	42	28	54	11	32	22	22	42	13	47	74	57	56	70
ENSG00000071967	929	783	833	816	1158	1167	904	1286	505	471	1642	321	975	1058	1025	1350	325	512	413	581	1817	722	1332	714	382	503	334	1090	704	1007	783	1124
ENSG00000233934	0	0	0	0	0	0	0	0	0	0	0	0	0	0	0	0	0	0	0	0	0	0	0	0	0	0	0	0	0	0	0	0
ENSG00000077380	940	761	797	582	2527	2506	1905	2585	1560	1275	5988	948	1786	1760	1803	2154	896	1143	835	871	5239	1974	3588	1806	1656	2101	1523	3775	1661	1741	1759	2025
ENSG00000115840	735	539	716	528	962	951	713	952	582	474	2108	394	591	524	546	711	559	909	788	831	1990	816	1479	803	616	905	493	1570	479	616	527	688
ENSG00000252779	0	1	1	0	7	2	1	0	2	1	1	1	0	1	3	1	1	0	5	1	3	2	3	1	2	1	0	1	0	0	1	1
ENSG00000128708	3188	1881	2433	1821	2542	2550	2017	3026	1579	1177	4303	943	1575	1317	1599	1715	3951	4200	4322	4056	7070	2898	5258	3697	1586	2237	860	3554	1937	1756	2235	2117
ENSG00000172878	419	318	351	336	279	306	233	281	466	357	1442	228	685	696	652	866	149	200	176	388	529	245	397	250	292	449	259	705	323	540	376	598
ENSG00000144355	8	21	16	31	8	2	4	6	9	13	36	13	220	202	267	309	3	24	7	21	8	6	3	2	12	19	10	27	249	334	306	359
ENSG00000115844	21	37	37	44	28	38	20	32	8	10	31	2	308	295	358	360	6	33	22	51	57	41	62	33	20	23	11	31	429	533	494	546
ENSG00000236651	2	0	1	2	1	3	0	5	0	0	1	1	2	7	11	8	0	2	5	0	7	0	0	2	1	0	0	0	6	11	14	9
ENSG00000232555	3	0	0	0	3	3	2	0	0	0	0	0	0	1	0	0	0	0	0	0	4	0	5	1	0	0	0	0	0	0	0	0
ENSG00000278924	0	0	0	0	1	1	0	1	0	0	0	0	0	0	1	0	0	0	0	0	0	2	0	0	0	0	3	0	0	0	0	0
ENSG00000091409	133	164	157	179	4909	4891	3964	5018	160	133	168	78	247	349	189	500	28	122	60	170	11164	4801	8086	4206	56	64	32	107	133	305	131	295
ENSG00000226963	0	0	0	0	0	3	2	1	4	1	0	0	0	0	1	0	0	1	0	0	5	10	5	2	0	0	2	0	0	0	0	0
ENSG00000232788	21	11	8	11	17	16	7	16	19	13	14	7	16	12	2	15	2	1	1	4	30	18	11	12	2	2	3	7	0	0	0	1
ENSG00000225205	65	68	70	60	157	154	143	215	138	133	166	65	72	70	63	66	19	13	10	15	192	103	123	91	46	40	21	74	23	28	9	48
ENSG00000152256	4293	3249	3667	3027	1129	1043	769	1256	2299	1864	3479	1066	1552	1413	1284	1696	877	1092	758	1109	1142	428	716	408	1063	1376	610	2321	448	692	585	829
ENSG00000199866	7	3	1	1	0	0	2	2	0	1	2	0	1	3	2	0	0	0	0	1	1	1	0	1	4	3	0	4	1	0	0	0
ENSG00000230104	1	1	1	0	1	0	0	0	0	0	0	0	0	0	0	1	0	0	0	2	0	0	0	2	0	1	0	0	0	4	0	0
ENSG00000228016	9	18	16	11	1	0	2	0	2	0	0	0	0	2	2	1	2	2	3	7	1	0	1	1	0	4	3	4	2	0	0	0
ENSG00000239041	1	0	2	0	0	0	0	0	0	0	0	0	0	0	0	0	0	0	0	1	0	0	0	0	1	0	0	0	0	0	0	0
ENSG00000091428	95	96	103	92	4	5	6	4	11	13	14	8	13	29	5	31	21	28	19	38	2	3	4	1	5	9	11	13	4	8	3	17
ENSG00000232292	0	2	0	0	0	0	0	0	0	0	0	0	0	0	0	0	0	0	0	0	0	0	0	0	0	0	0	0	0	0	0	0
ENSG00000091436	3047	2500	2671	2601	4	10	6	17	2744	2258	8173	1611	1981	2111	1928	2543	1049	1735	1556	2286	8	7	9	6	2492	3460	1770	5837	1399	1998	1477	2256
ENSG00000238133	26	14	21	22	2	5	3	3	19	13	52	5	9	7	5	10	3	8	6	33	1	0	1	0	15	22	16	46	1	17	1	18
ENSG00000231735	0	2	0	0	2	0	0	0	0	0	0	0	0	0	0	0	0	0	1	0	0	0	0	0	0	0	0	0	0	0	0	0
ENSG00000236391	11	12	11	9	12	11	16	29	5	5	16	1	3	6	7	2	3	9	12	10	24	13	26	14	3	11	7	12	3	2	2	5
ENSG00000144354	8809	6251	6936	5878	6796	7095	5081	7445	4692	3777	10410	2296	5180	4241	4847	5552	6105	6446	5527	5934	13166	5406	9227	5272	5213	6839	2348	8543	3640	4163	4003	5089
ENSG00000213148	0	0	0	0	0	0	0	0	0	0	0	0	0	0	1	0	0	0	0	0	0	0	0	0	0	0	0	0	0	1	1	1
ENSG00000236832	0	0	0	0	0	0	0	0	0	0	3	0	1	0	0	0	0	0	0	0	0	0	0	0	0	0	0	1	0	0	0	0
ENSG00000226131	0	0	0	2	0	0	0	0	0	0	0	0	0	0	0	0	1	0	0	0	1	0	0	0	0	1	1	0	0	1	0	1
ENSG00000237016	0	0	0	0	0	0	0	0	0	0	0	0	2	5	2	2	0	0	0	0	0	0	0	0	0	0	0	0	3	14	6	2
ENSG00000237617	0	0	0	0	0	0	0	0	0	0	0	0	2	2	2	2	0	0	0	0	0	0	0	0	0	0	0	1	0	0	3	5
ENSG00000270460	0	0	0	0	0	0	0	0	0	0	0	0	11	24	8	11	0	0	0	0	0	0	0	0	0	0	0	0	4	15	10	8
ENSG00000234415	0	0	0	0	0	0	0	0	0	0	0	0	0	0	0	1	0	0	0	0	0	0	1	0	0	0	0	0	0	0	0	0
ENSG00000224638	2	0	2	2	8	5	0	4	2	1	16	0	1	3	4	3	1	2	0	0	7	6	1	5	3	6	4	16	0	7	2	1
ENSG00000172845	4235	2750	3467	2635	3103	3103	2453	3416	3155	2616	11603	1911	2138	2173	2334	2747	2505	3485	3301	3597	7517	3102	5705	3005	3383	4802	2856	8637	1917	2315	2389	2721
ENSG00000271151	7	5	14	3	9	14	10	19	8	19	93	12	7	6	13	14	1	9	3	16	21	9	13	10	11	9	29	49	8	10	5	8

ENSG00000273258	0	0	0	0	0	0	1	0	0	0	0	0	0	0	0	0
ENSG00000260868	0	0	0	0	0	0	0	0	1	0	0	0	0	0	0	0
ENSG00000235414	0	0	0	0	0	0	0	0	0	0	0	0	0	0	0	0
ENSG00000138430	2101	1459	1631	1426	2763	2791	2296	3072	2586	1898	7574	1442	3465	3170	3808	4231
ENSG00000270799	3	2	4	4	6	2	5	3	3	3	2	1	2	5	2	3
ENSG00000243770	4	2	2	3	0	0	0	2	2	0	2	2	4	0	4	0
ENSG00000201088	0	0	0	0	0	0	0	0	0	0	0	0	0	0	0	0
ENSG00000227180	0	0	0	0	0	0	0	0	0	0	0	0	0	0	0	0
ENSG00000237804	0	0	0	0	0	1	0	0	0	0	1	0	0	0	0	0
ENSG00000231453	0	3	0	4	0	0	0	0	0	0	0	0	3	3	0	9
ENSG00000280414	1	1	0	3	0	0	0	0	0	0	1	1	4	3	4	11
ENSG00000217236	3	16	9	11	0	0	0	0	2	3	8	2	18	13	21	22
ENSG00000138433	707	506	579	490	612	598	527	698	713	464	2559	370	764	674	774	946
ENSG00000144306	375	290	338	251	480	487	399	614	514	418	2332	349	257	261	286	406
ENSG00000163328	385	258	338	324	63	87	45	88	308	262	2026	193	641	710	652	848
ENSG00000226853	16	9	16	10	9	4	4	9	4	2	19	2	12	10	9	11
ENSG00000179997	0	0	0	0	0	0	0	0	0	0	0	0	0	0	0	0
ENSG00000279884	0	0	1	1	1	0	1	0	3	1	0	0	2	1	1	2
ENSG00000236449	19	14	10	13	34	36	26	35	14	17	73	11	26	37	49	35
ENSG00000206965	1	1	1	1	3	9	6	8	2	3	10	1	2	4	3	2
ENSG00000115935	1824	1312	1678	1386	7987	7830	6487	8387	5858	4862	20706	3329	6181	5588	7521	7435
ENSG00000237798	3	3	5	0	15	10	8	11	11	7	32	4	6	11	10	12
ENSG00000235655	1383	900	1030	892	1348	1167	1035	1275	702	563	2059	422	165	164	158	179
ENSG00000138435	1	0	1	0	0	0	1	0	0	0	0	0	2	3	1	0
ENSG00000128656	712	552	579	630	8	7	11	9	10	6	165	10	33	49	38	52
ENSG00000212167	0	0	0	0	0	0	1	1	0	0	0	0	0	0	0	0
ENSG00000199827	0	0	0	0	0	0	1	0	0	0	0	0	0	0	0	0
ENSG00000227187	0	0	2	0	0	0	0	0	1	0	1	0	0	0	0	0
ENSG00000115966	2364	1681	1971	1519	2311	2264	1757	2533	1767	1488	5962	965	712	696	694	909
ENSG00000277948	22	10	8	17	8	16	6	18	8	7	34	3	11	4	9	7
ENSG00000215973	0	1	2	4	2	5	2	3	3	2	3	0	0	1	0	1
ENSG00000229750	7	5	3	3	5	5	3	8	3	1	3	0	3	0	5	5
ENSG00000154518	4563	3109	3656	3029	3983	4088	2755	4342	4308	2938	11004	2392	4423	4108	4771	5555
ENSG00000200121	0	0	0	0	0	0	0	0	0	0	0	0	0	0	0	0
ENSG00000233131	0	0	0	0	0	0	0	0	0	0	0	0	0	0	0	0
ENSG00000229066	0	0	0	0	0	0	0	0	0	0	0	0	1	0	1	0
ENSG00000223976	0	0	0	0	0	0	0	0	0	0	0	0	0	0	0	0
ENSG00000235047	0	1	0	0	0	0	0	0	0	0	0	0	0	0	0	0
ENSG00000229779	0	0	0	0	0	0	0	0	0	0	0	0	0	0	0	0
ENSG00000144320	2746	2055	2242	1996	2519	2515	1981	2804	1577	1358	4525	914	1548	1609	1539	2071
ENSG00000174279	0	4	0	0	0	0	0	0	0	0	0	0	0	0	0	0
ENSG00000128714	316	347	262	328	1	3	2	2	3	9	6	3	142	185	135	196
ENSG00000170178	15	12	17	18	18	12	17	19	2	2	9	8	4	8	6	6
ENSG00000128713	0	0	0	0	2	0	0	0	0	0	0	0	0	0	2	4
ENSG00000128710	0	0	0	0	1	1	0	0	0	0	17	1	1	1	0	2
ENSG00000237380	0	0	0	0	0	1	1	0	30	10	71	15	1	0	0	0
ENSG00000128709	0	0	0	0	0	2	0	1	2	5	28	6	0	0	3	0
ENSG00000175879	2	2	0	3	0	0	0	0	18	12	93	10	96	124	66	144
ENSG00000128652	0	0	0	0	0	0	0	0	20	14	115	10	0	2	2	0
ENSG00000207744	0	0	0	0	0	0	0	0	5	4	34	3	0	0	0	0
ENSG00000278500	0	0	0	0	0	0	0	0	13	7	83	7	0	0	0	0
ENSG00000170166	0	0	0	0	0	0	0	0	92	64	300	64	0	0	0	0
ENSG00000272729	0	0	0	0	0	0	0	0	5	7	31	6	0	2	0	2
ENSG00000224189	0	6	1	2	0	0	0	0	178	166	1009	133	29	84	38	93
ENSG00000226363	1	5	0	8	0	0	0	0	3	9	21	4	2	2	0	0
ENSG00000128645	0	0	0	0	0	0	0	0	7	4	27	3	4	8	4	7
ENSG00000279160	0	0	0	0	0	0	0	0	0	0	1	0	0	0	0	2
ENSG00000218175	10	5	3	4	5	8	7	9	9	7	23	9	9	4	1	6
ENSG00000230384	0	0	0	0	0	0	0	0	0	0	0	0	0	0	0	0
ENSG00000128654	1277	1029	999	1056	480	597	367	621	785	598	2614	498	1128	1085	1175	1392
ENSG00000237260	0	0	0	1	0	0	0	0	1	2	4	2	4	1	0	0
ENSG00000229434	0	0	0	0	0	0	0	0	7	5	23	0	12	14	6	10
ENSG00000236231	1	0	0	0	1	0	1	0	14	5	100	7	21	23	22	16
ENSG00000283203	0	0	0	0	0	0	0	1	0	1	10	0	1	1	4	2
ENSG00000272551	0	0	0	0	0	0	0	3	5	3	36	2	5	10	5	5
ENSG00000163364	0	0	0	0	0	0	0	0	67	71	435	57	47	42	42	59
ENSG00000224577	0	0	0	1	0	0	0	2	42	12	118	12	43	36	30	43
ENSG00000252027	0	0	0	0	0	0	0	0	1	1	7	0	2	6	1	4
ENSG00000230552	0	0	0	0	1	2	1	2	39	32	181	22	69	50	60	80
ENSG00000237053	0	0	0	0	4	0	0	1	5	3	16	3	20	15	13	25
ENSG00000223777	0	0	0	0	0	0	0	1	1	0	3	0	7	6	5	8
ENSG00000279205	0	0	0	0	0	1	0	0	19	2	43	9	36	39	23	40
ENSG00000227098	0	0	0	0	0	0	1	0	0	0	0	0	0	0	0	0
ENSG00000206866	0	0	0	0	0	0	0	0	0	0	0	0	0	0	0	0
ENSG00000236501	0	0	0	0	0	0	0	0	0	0	1	1	0	0	0	0
ENSG00000229337	1	2	0	2	0	1	0	0	1	0	1	0	0	2	0	0
ENSG00000226273	0	0	0	0	0	0	0	0	0	0	0	0	0	0	0	0
ENSG00000201102	0	0	0	0	0	0	0	0	0	0	1	0	0	0	1	1
ENSG00000213069	4	1	1	0	1	0	0	0	1	0	5	0	5	1	0	0
ENSG00000170144	32260	21283	26488	23358	25418	25284	20122	26613	28267	20111	89171	15840	28309	25768	28056	32677
ENSG00000116044	1245	677	1072	719	851	1013	658	900	906	787	5975	874	981	843	1063	1250
ENSG00000225808	34	28	25	36	24	31	16	35	9	5	37	2	3	5	13	10
ENSG00000265396	1	1	1	4	0	5	0	5	0	1	16	0	2	3	1	1
ENSG00000222043	11	8	14	4	5	10	4	8	5	4	23	6	7	6	11	6
ENSG00000213963	178	127	179	173	167	174	86	139	111	108	477	76	203	189	147	216
ENSG00000271825	10	7	9	10	11	8	10	13	8	9	31	2	16	16	13	32
ENSG00000271996	10	11	10	14	15	14	6	13	6	13	43	7	12	8	25	26
ENSG00000278418	1	1	3	2	0	1	1	2	5	3	8	1	1	3	1	6
ENSG00000280374	39	31	39	34	23	23	19	26	17	25	77	22	24	21	25	37
ENSG00000237178	2	0	2	0	2	3	4	1	3	0	13	0	2	6	2	3
ENSG00000238295	0	1	0	0	0	2	1	1	1	0	4	0	3	0	0	3
ENSG00000018510	3226	2326	2416	2263	4049	4410	3282	4522	2356	1981	8169	1500	3076	2866	3165	3533
ENSG00000196659	321	231	285	249	58	48	42	59	109	73	288	56	89	105	123	147
ENSG00000237655	0	0	0	0	0	0	0	0	0	0	0	0	0	1	0	0
ENSG00000197557	487	400	466	379	50	61	38	63	121	98	353	61	109	110	119	151
ENSG00000128655	16	9	19	14	13	11	15	11	3	1	3	1	0	2	2	4
ENSG00000229941	6	0	9	0	28	12	30	31	0	0	0	0	0	0	0	2
ENSG00000236664	0	0	0	0	0	0	0	0	0	0	0	0	0	0	0	0
ENSG00000213962	0	0	0	0	0	1	0	0	0	0	0	0	0	1	0	0
ENSG00000229744	0	0	0	0	0	0	0	0	0	0	0	0	0	0	0	0
ENSG00000206788	0	0	0	0	0	0	0	0	0	0	0	0	0	0	0	0
ENSG00000249909	0	0	0	0	0	0	0	0	0	0	0	0	0	0	0	0
ENSG00000227241	0	0	0	0	0	0	0	0	0	0	0	0	0	0	0	0
ENSG00000155636	396	276	324	260	281	354	251	346	349	268	1377	246	274	246	265	321
ENSG00000223096	0	0	0	0	0	0	0	1	0	0	0	0	0	0	0	0
ENSG00000079156	3	9	4	9	5	24	4	13	429	302	1375	276	53	89	40	75
ENSG00000223960	994	781	782	746	1193	1244	882	1371	609	554	2014	353	692	716	716	905
ENSG00000180228	1473	1076	1181	1058	1902	1940	1360	2043	723	571	2194	394	1998	1685	1756	2050
ENSG00000204311	62	68	79	62	133	136	118	181	28	8	79	11	92	77	98	107
ENSG00000238082	9	14	12	12	16	17	11	24	3	1	9	2	5	10	6	10
ENSG00000079150	438	312	262	249	162	180	152	164	190	139	480	84	260	229	241	317
ENSG00000116095	863	604	743	548	669	641	518	693	550	383	2352	317	520	533	600	643
ENSG00000237298	367	289	366	271	4	3	11	11	136	154	619	97	211	224	232	241
ENSG00000155657	65	49	23	44	108	142	121	157	41	40	222	38	508	563	434	605
ENSG00000283186	0	0	0	0	0	0	0	0	0	0	0	0	0	0	0	0
ENSG00000270956	32	25	50	24	1	0	0	0	11	7	34	10	44	27	19	24
ENSG00000270277	48	25	31	24	0	0	0	0	11	9	26	10	36	46	39	38
ENSG00000279598	25	13	22	18	2	0	1	4	7	11	20	3	12	28	16	21
ENSG00000271011	2	1	0	0	0	0	0	0	0	0	3	1	0	3	1	3
ENSG00000270574	28	7	27	16	0	0	0	0	8	11	31	6	3	20	10	14
ENSG00000271141	7	11	19	8	0	0	0	1	3	10	42	4	2	3	1	7
ENSG00000271401	2	6	8	5	0	0	1	1	0	5	14	3	3	4	1	3
ENSG00000267784	1	2	4	3	0	0	0	0	3	1	7	0	9	23	9	11
ENSG00000163492	2	0	0	1	97	117	94	140	142	78	406	59	800	805	840	935
ENSG00000238542	0	0	0	0	1	1	0	1	0	0	3	0	8	4	3	4
ENSG00000231541	0	0	0	0	0	1	0	0	1	0	0	0	0	1	4	7
ENSG00000187231	1248	1594	1011	1329	1798	1889	1335	2019	802	653	2604	514	706	825	614	948
ENSG00000237477	0	0	0	0	0	0	0	0	0	0	0	0	0	0	0	0
ENSG00000237964	0	0	0	0	0	0	0	0	0	0	0	0	0	0	0	0
ENSG00000144331	3	2	0	0	5	0	2	1	0	1	0	1	9	14	4	16
ENSG00000213126	0	0	0	0	0	0	0	0	0	0	0	0	0	0	0	0
ENSG00000221240	0	0	0	0	0	0	0	0	0	0	0	0	0	0	0	0
ENSG00000280636	0	0	0	0	0	0	0	2	1	1	0	0	1	2	1	0
ENSG00000202216	0	0	0	0	0	0	0	0	0	0	0	0	0	0	0	0
ENSG00000163510	1452	967	1177	946	1227	1249	1017	1431	909	634	2888	512	1285	1156	1440	1547
ENSG00000270289	0	0	0	0	0	0	0	0	0	0	0	0	0	0	0	0
ENSG00000225258	0	0	0	0	0	0	0	0	0	0	0	0	0	0	0	0
ENSG00000281131	0	0	0	0	0	0	0	0	0	1	1	0	0	0	0	0
ENSG00000279559	0	0	0	0	0	0	0	0	0	0	0	0	0	0	0	0
ENSG00000226564	3	1	1	1	0	2	2	3	1	2	1	0	5	1	2	1
ENSG00000236667	0	0	0	0	0	0	0	0	0	0	0	0	0	0	0	0
ENSG00000170035	1370	1238	1192	1204	1	0	0	0	938	840	3695	662	517	569	520	773
ENSG00000236153	0	0	0	1	0	0	0	0	0	0	0	0	1	0	1	0
ENSG00000238171	1	0	1	1	0	0	0	0	3	2	4	0	0	0	1	1
ENSG00000234663	53	29	39	19	162	171	197	180	56	34	89	13	86	81	85	90
ENSG00000266705	0	0	2	0	1	2	1	1	0	0	0	0	1	1	1	0
ENSG00000226681	1	0	3	0	1	3	2	6	0	0	0	0	1	0	3	0
ENSG00000115232	16094	13094	14351	12958	16937	16361	12075	16289	2280	2110	8317	1507	2858	2680	2785	3237
ENSG00000188452	2984	2144	2594	2191	3399	3227	2526	3361	650	539	2096	328	650	642	667	706
ENSG00000162992	0	3	1	0	0	0	0	0	0	0	0	0	0	0	0	0
ENSG00000234595	0	0	0	0	0	0	0	0	0	0	0	0	0	0	0	0
ENSG00000225570	0	0	0	0	0	0	0	0	0	0	0	0	0	0	0	0
ENSG00000223873	0	0	0	0	0	0	0	0	0	0	0	0	0	0	0	0
ENSG00000221023	0	0	0	0	0	0	0	0	0	0	1	0	0	0	0	0
ENSG00000260742	4	3	6	8	12	15	12	10	14	13	46	8	20	20	20	38
ENSG00000138434	5243	3996	4222	4066	5102	5085	3803	5324	1294	1174	4981	937	2294	2169	2463	2880
ENSG00000150722	79	63	102	53	40	37	25	29	1	0	2	0	25	19	19	31
ENSG00000213958	2	1	0	0	0	2	0	2	0	0	0	0	0	0	0	0
ENSG00000115252	0	2	1	0	31	35	35	46	0	1	4	0	1	3	4	6
ENSG00000242121	0	0	0	0	0	0	0	0	0	0	0	0	0	0	0	0
ENSG00000077232	2336	1811	1752	1714	2333	2458	1907	2735	2115	1716	7714	1336	3745	3413	3960	4401
ENSG00000232430	0	0	0	0	0	0	0	0	0	0	0	0	0	0	0	0
ENSG00000212404	0	0	0	0	0	0	0	0	0	0	0	0	0	0	0	0
ENSG00000162998	1	0	3	0	0	0	0	0	0	1	3	0	2	1	0	1
ENSG00000207178	0	0	0	0	0	0	0	0	0	0	0	0	0	0	0	0
ENSG00000061676	1151	1095	853	1061	0	0	0	0	244	328	1163	229	1308	1428	1326	1708
ENSG00000202141	1	0	0	0	0	0	0	0	0	0	2	0	2	1	1	1
ENSG00000221498	0	0	0	0	0	0	0	0	0	0	0	0	0	0	0	0
ENSG00000231203	0	0	0	0	0	0	0	0	0	0	0	0	0	0	0	0
ENSG00000162999	0	0	0	0	0	1	0	0	147	142	538	80	148	172	101	155
ENSG00000224643	0	0	0	0	0	0	0	0	0	2	1	0	1	1	0	0
ENSG00000163002	637	426	499	446	529	545	442	598	455	350	1197	243	571	602	645	700
ENSG00000272800	24	15	22	20	18	32	32	33	14	18	41	11	26	15	19	21
ENSG00000213120	1	0	0	0	0	0	0	0	0	0	0	0	0	0	0	0
ENSG00000177855	16	12	11	9	10	10	6	4	11	6	7	3	8	3	20	7
ENSG00000234172	0	0	0	0	0	0	0	0	0	0	0	0	0	0	0	0
ENSG00000266808	0	0	0	0	0	0	0	0	0	0	0	0	0	0	0	0
ENSG00000170396	0	0	0	0	0	0	0	0	0	0	3	0	11	7	9	4
ENSG00000237824	0	0	0	0	0	0	0	0	0	0	0	0	0	0	0	1
ENSG00000225406	0	0	0	0	0	0	0	0	0	0	0	0	0	0	0	0
ENSG00000212581	0	0	0	0	0	0	0	0	0	0	0	0	0	0	0	0
ENSG00000226747	1	1	0	0	0	0	0	0	0	1	0	0	0	0	1	0
ENSG00000188738	35	40	42	27	17	32	14	35	25	36	42	10	25	51	13	26
ENSG00000231646	6	2	2	1	1	3	3	2	3	3	5	2	3	5	3	2
ENSG00000224019	3	0	1	3	3	8	2	4	1	2	7	2	0	2	0	1
ENSG00000237877	25	20	19	14	9	13	10	21	10	10	36	9	14	12	18	13
ENSG00000226410	10	6	1	0	1	2	0	3	2	3	14	3	5	2	2	1
ENSG00000232568	1	2	3	0	0	1	1	0	0	0	0	0	0	0	0	0
ENSG00000259915	5	4	7	8	5	5	8	16	9	4	19	3	3	10	4	6
ENSG00000227692	5	0	5	2	3	3	4	4	5	4	5	2	7	5	2	3
ENSG00000065548	2540	1756	1995	1555	1921	2029	1579	2225	1967	1362	6182	1032	2206	1955	2606	2718
ENSG00000224323	1	0	0	2	2	1	1	1	0	1	1	3	1	2	1	2
ENSG00000281192	4	1	4	2	3	5	1	5	1	4	9	3	1	2	1	2
ENSG00000138448	1224	987	983	886	1200	1318	908	1244	580	560	2765	433	368	389	389	569
ENSG00000227227	13	6	5	8	10	3	2	6	1	2	16	2	3	2	1	2
ENSG00000144369	204	251	179	258	3	1	0	1	128	134	565	111	118	141	108	165
ENSG00000163012	0	0	0	0	0	0	0	0	0	0	0	0	0	0	0	0
ENSG00000226553	0	0	0	0	0	0	0	0	0	0	1	0	0	0	0	0
ENSG00000224063	0	0	0	1	5	2	2	3	0	0	3	0	2	3	1	10
ENSG00000222845	0	0	0	0	0	0	0	0	0	0	0	0	0	0	0	0
ENSG00000199626	0	0	0	0	0	0	0	0	0	0	0	0	0	0	0	0
ENSG00000064989	0	0	0	0	5	15	11	15	1	0	4	4	22	34	47	48
ENSG00000231739	0	0	0	0	0	0	0	0	0	0	0	0	0	0	0	0
ENSG00000003436	0	7	3	1	302	275	210	288	1	0	1	0	181	237	216	280
ENSG00000231689	0	0	0	0	0	0	0	2	0	0	2	0	0	1	0	0
ENSG00000235616	0	0	0	0	0	0	0	0	0	0	0	1	0	0	0	0
ENSG00000213115	0	0	0	0	0	0	0	0	0	0	0	0	0	0	0	0
ENSG00000144366	0	0	0	0	0	0	0	0	16	19	110	22	4	17	20	37
ENSG00000207951	0	0	0	0	0	0	0	0	0	0	0	0	0	0	0	0
ENSG00000223523	0	0	0	0	5	5	5	6	0	0	0	0	0	0	0	0
ENSG00000174325	0	0	0	0	0	0	0	0	0	0	0	0	0	0	0	0
ENSG00000168542	1	0	0	0	8	16	5	16	0	0	0	0	19	42	32	48
ENSG00000221502	0	0	0	0	0	0	0	0	0	0	0	0	0	0	0	0
ENSG00000283238	0	0	0	0	0	0	0	0	0	0	0	0	0	0	0	0
ENSG00000204262	29	28	38	22	31	31	36	44	482	270	1051	204	49	45	45	63
ENSG00000228073	0	0	0	0	0	0	0	0	0	0	0	0	0	1	0	0
ENSG00000264725	0	0	0	0	0	0	0	0	0	0	0	0	0	0	0	0
ENSG00000213601	1	0	0	0	0	0	0	0	0	0	0	0	1	0	1	2
ENSG00000115368	2157	1417	1511	1358	2163	2310	1796	2385	1636	1207	5064	999	2246	2250	2570	2882
ENSG00000233996	0	0	0	1	2	3	0	0	0	0	3	2	0	3	2	4
ENSG00000138449	702	620	607	615	1229	1109	893	1250	81	82	234	57	96	143	116	191
ENSG00000276828	0	0	0	0	0	0	0	0	0	0	0	0	0	0	0	0
ENSG00000138381	1153	758	905	735	819	872	747	894	1056	900	3591	656	809	847	1040	1105
ENSG00000151687	114	83	99	90	34	55	39	42	130	85	481	58	59	75	52	57
ENSG00000128694	386	278	285	289	250	313	212	283	369	309	1114	191	243	228	171	250
ENSG00000253559	34	21	32	23	15	17	14	9	17	17	61	11	23	15	18	25
ENSG00000273240	9	8	8	10	7	5	4	5	10	4	33	4	2	6	4	8
ENSG00000128699	1391	872	1046	825	1527	1453	1241	1617	1055	839	4088	657	861	806	1117	1132
ENSG00000064933	1606	1036	1137	1185	1255	1355	1001	1495	874	746	2988	495	1077	880	961	1087
ENSG00000187699	148	113	157	85	80	93	71	81	85	84	350	47	78	70	65	79
ENSG00000223824	0	0	0	0	0	0	0	0	0	0	0	0	0	0	0	0
ENSG00000204253	19	14	17	15	33	14	15	18	15	16	59	7	18	18	18	25
ENSG00000213110	0	0	0	0	0	0	0	0	0	0	0	0	0	0	0	0
ENSG00000138379	20	29	19	13	4	3	0	3	2	3	13	1	8	7	3	7
ENSG00000198130	959	644	772	655	1025	1079	768	1195	621	441	1869	306	483	436	518	569
ENSG00000151689	4	0	1	1	456	537	350	520	443	344	1869	323	338	350	365	505
ENSG00000151690	1659	1250	1585	1246	1213	1367	931	1405	2069	1676	9198	1332	727	686	652	897
ENSG00000272979	26	21	22	19	6	6	13	16	3	2	17	1	12	15	9	25
ENSG00000189362	882	685	713	602	469	546	386	554	516	407	1579	333	494	470	484	596
ENSG00000233654	49	23	24	13	11	11	5	6	35	30	119	21	5	4	4	6
ENSG00000251935	6	3	0	0	0	0	0	0	1	3	5	0	0	0	0	0
ENSG00000138386	1254	1097	1068	1020	1635	1771	1374	1826	1777	1546	6012	1058	380	525	422	666
ENSG00000228509	8	3	8	6	4	13	3	11	10	9	32	9	2	4	2	1
ENSG00000235852	16	16	15	10	9	19	7	8	7	9	36	3	16	16	23	23
ENSG00000115419	3593	2454	3140	2446	3796	3943	3189	4127	3172	2627	15080	2004	4137	3885	4594	4823
ENSG00000115415	2440	1771	1990	1628	2061	2277	1608	2288	1633	1380	7266	1288	5563	8642	6835	11313
ENSG00000229023	2	1	0	1	1	0	2	3	0	1	15	0	3	9	8	9
ENSG00000230686	0	0	0	0	0	0	0	0	0	0	1	0	0	0	1	0
ENSG00000231858	7	6	5	2	3	2	2	4	74	34	182	37	7	13	8	8
ENSG00000138378	13	13	11	9	37	51	40	47	1839	1352	6289	938	308	272	330	312
ENSG00000207402	0	0	0	0	0	0	0	0	1	2	3	0	0	0	0	0
ENSG00000280083	6	6	6	5	0	1	0	1	0	0	0	0	0	0	0	2
ENSG00000230611	2	0	0	1	0	1	0	0	1	0	1	0	0	1	0	0
ENSG00000227542	22	7	7	18	5	6	4	2	0	0	0	0	0	1	0	0
ENSG00000128641	2845	2443	2522	2437	2912	3083	2224	3351	24	28	51	24	546	581	641	755
ENSG00000252130	1	4	3	2	2	2	2	0	0	0	1	0	0	0	0	1
ENSG00000173559	900	627	901	656	1507	1718	1218	1709	649	564	3172	351	6740	6636	7407	8128
ENSG00000274845	1	0	0	0	0	0	0	0	0	0	0	0	0	2	0	0
ENSG00000225884	0	0	0	0	0	0	0	0	0	0	0	0	3	0	0	0
ENSG00000168497	0	0	0	0	11	6	6	7	0	0	0	0	0	0	0	0
ENSG00000233766	0	0	0	0	0	0	0	0	0	0	2	0	0	0	0	0
ENSG00000213946	0	0	0	0	0	0	0	0	0	0	0	0	0	0	0	0
ENSG00000144339	0	0	0	0	0	0	0	0	0	1	0	0	1	0	0	3

ENSG00000273258	0	0	0	0	0	0	0	0	0	0	0	0	0	0	0	0
ENSG00000260868	0	0	0	0	0	0	0	0	0	1	0	0	0	0	0	0
ENSG00000235414	0	0	0	0	0	0	0	0	0	0	0	0	0	1	0	0
ENSG00000138430	1674	2335	2104	1871	6198	2431	4700	2427	2730	3515	1545	5255	3413	3553	4160	4399
ENSG00000270799	1	0	2	20	10	2	2	5	7	3	1	4	3	3	13	4
ENSG00000243770	0	0	1	3	6	0	2	2	7	5	0	11	1	1	7	0
ENSG00000201088	0	0	0	0	0	0	0	0	0	0	0	0	0	0	0	0
ENSG00000227180	0	0	0	0	0	0	0	0	0	0	0	1	0	0	0	0
ENSG00000237804	0	0	0	0	0	1	0	0	0	0	0	2	1	0	1	0
ENSG00000231453	0	2	0	3	0	0	0	0	0	0	1	2	2	2	3	3
ENSG00000280414	1	1	2	8	0	0	0	0	0	2	1	3	3	11	4	4
ENSG00000217236	1	4	3	6	0	0	0	0	1	3	2	6	11	15	16	16
ENSG00000138433	651	723	678	572	1325	571	987	559	577	733	802	1601	752	771	969	986
ENSG00000144306	362	420	435	432	1096	459	728	430	503	632	563	1334	306	347	374	357
ENSG00000163328	102	164	100	191	54	23	43	21	282	351	930	1174	287	563	354	696
ENSG00000226853	17	15	28	14	27	9	16	12	4	5	2	12	11	14	16	13
ENSG00000179997	0	0	0	0	0	0	0	0	0	0	0	0	0	0	0	0
ENSG00000279884	0	0	0	0	2	0	0	0	0	0	0	2	1	1	1	6
ENSG00000236449	1	1	7	20	48	15	22	39	28	18	15	99	18	44	43	58
ENSG00000206965	2	0	2	5	6	3	5	0	5	3	9	7	0	1	6	4
ENSG00000115935	505	551	624	1142	11545	4885	9644	4460	5216	7602	4141	12412	4638	5627	5418	6066
ENSG00000237798	2	1	2	7	23	14	4	10	15	9	17	27	5	10	7	17
ENSG00000235655	1142	1725	1451	1012	3106	1119	2017	1012	747	848	427	1374	203	194	199	224
ENSG00000138435	0	0	0	0	0	1	1	2	0	0	0	0	1	0	2	1
ENSG00000128656	353	718	549	624	27	14	18	13	18	40	136	243	36	117	51	94
ENSG00000212167	0	0	0	0	0	0	0	0	0	0	0	0	0	0	0	0
ENSG00000199827	0	0	0	0	3	1	0	0	0	0	0	2	0	0	0	0
ENSG00000227187	0	0	0	0	1	1	0	1	3	0	0	3	0	0	0	0
ENSG00000115966	1815	2365	2054	1836	5148	2040	3484	2282	1642	2204	1412	4119	649	828	743	916
ENSG00000277948	4	1	0	19	20	15	7	6	6	14	5	22	2	4	4	9
ENSG00000215973	0	0	0	5	4	2	2	1	0	2	0	8	0	1	0	0
ENSG00000229750	3	1	4	1	11	1	3	2	0	8	3	7	0	2	2	3
ENSG00000154518	7069	8751	7881	5825	10795	3960	7054	4233	4063	5919	2135	7811	5869	5309	6761	6882
ENSG00000200121	0	0	0	0	0	0	0	0	0	0	0	0	0	0	0	0
ENSG00000233131	0	0	0	0	0	0	0	0	0	0	0	0	0	0	0	0
ENSG00000229066	0	0	0	0	0	0	0	0	0	0	0	0	0	0	0	0
ENSG00000223976	0	0	0	0	0	0	0	0	0	0	0	0	0	0	0	0
ENSG00000235047	0	0	0	0	0	0	0	0	0	0	0	0	0	0	0	0
ENSG00000229779	0	0	0	0	0	0	0	0	0	0	0	0	0	0	0	0
ENSG00000144320	1201	1558	1452	1827	3725	1578	2643	1581	1418	2120	992	3721	1089	1563	1183	1743
ENSG00000174279	0	0	0	1	0	0	0	0	0	1	3	7	0	0	0	0
ENSG00000128714	146	321	189	405	5	3	5	1	1	6	3	15	65	159	61	128
ENSG00000170178	7	12	8	18	35	21	27	18	1	9	2	18	1	2	2	6
ENSG00000128713	0	0	0	0	1	1	1	2	2	0	1	4	1	7	0	2
ENSG00000128710	0	0	0	1	3	0	3	1	3	2	6	11	0	2	0	2
ENSG00000237380	0	0	0	0	0	0	0	0	18	21	25	69	0	0	0	0
ENSG00000128709	0	0	0	0	1	0	0	0	1	5	11	20	0	0	0	0
ENSG00000175879	0	0	1	0	0	0	0	0	14	16	22	54	70	110	84	127
ENSG00000128652	0	0	0	0	0	0	0	0	32	34	35	83	1	0	2	2
ENSG00000207744	0	0	0	0	0	0	0	0	2	5	5	25	0	0	0	0
ENSG00000278500	0	0	0	0	0	0	0	0	2	19	37	58	0	0	0	0
ENSG00000170166	0	0	0	0	0	0	0	0	77	131	107	221	0	0	0	0
ENSG00000272729	0	0	0	0	0	0	0	0	2	10	20	36	0	3	0	3
ENSG00000224189	0	1	0	3	0	0	0	0	224	347	472	906	29	70	26	78
ENSG00000226363	0	7	0	4	0	0	0	0	6	3	9	21	0	0	0	1
ENSG00000128645	0	0	0	0	0	0	0	0	12	13	34	47	1	8	3	4
ENSG00000279160	0	0	0	0	0	0	0	1	0	0	3	0	0	0	0	0
ENSG00000218175	11	11	13	12	11	5	8	9	12	25	5	29	3	8	15	12
ENSG00000230384	0	0	0	0	0	0	0	0	0	0	0	0	0	0	0	0
ENSG00000128654	1340	2283	1660	1485	811	340	583	363	787	1090	664	1916	1143	1308	1461	1556
ENSG00000237260	1	0	0	2	0	0	0	0	0	6	2	3	2	3	1	0
ENSG00000229434	0	0	0	0	0	2	4	3	11	5	18	46	4	13	5	11
ENSG00000236231	0	0	0	0	0	1	0	1	20	29	62	117	1	21	25	30
ENSG00000283203	0	0	0	0	0	0	0	0	0	2	7	3	0	1	1	5
ENSG00000272551	0	0	0	0	0	2	0	0	11	5	26	34	2	10	6	13
ENSG00000163364	0	0	0	0	0	0	0	0	75	124	186	312	46	70	41	64
ENSG00000224577	0	0	0	0	0	0	1	1	20	28	59	100	21	37	25	31
ENSG00000252027	0	0	0	0	0	0	0	0	1	4	6	5	0	1	0	3
ENSG00000230552	0	0	0	0	5	2	0	2	45	33	82	154	11	35	15	30
ENSG00000237053	0	0	0	0	0	0	1	0	4	6	5	16	2	8	1	10
ENSG00000223777	0	0	0	0	0	0	0	1	1	0	1	2	2	1	3	0
ENSG00000279205	0	0	0	0	3	1	0	1	7	11	24	33	1	7	6	4
ENSG00000227098	0	0	0	0	0	0	0	0	0	0	0	0	0	0	0	0
ENSG00000206866	0	0	0	0	0	0	0	0	0	0	0	0	0	0	0	0
ENSG00000236501	0	0	0	0	0	0	0	0	0	0	1	0	0	0	0	0
ENSG00000229337	1	0	0	1	0	0	0	0	0	0	0	0	0	0	1	1
ENSG00000226273	0	0	0	0	0	0	0	0	0	0	0	0	0	0	0	0
ENSG00000201102	0	1	0	0	0	0	0	0	0	0	0	0	0	0	0	0
ENSG00000213069	0	0	0	1	1	0	0	0	1	1	0	2	0	0	2	1
ENSG00000170144	20812	21881	20886	22305	46687	19527	34698	17502	26033	37204	17579	59605	24732	27702	25084	31922
ENSG00000116044	890	905	801	1085	1531	842	1356	764	1097	1570	1705	4085	828	829	1346	1080
ENSG00000225808	9	10	4	16	15	6	7	18	6	8	6	33	2	6	1	9
ENSG00000265396	0	1	0	3	0	3	0	0	6	2	2	7	0	2	2	0
ENSG00000222043	6	9	5	14	11	11	8	8	8	11	9	25	5	12	5	9
ENSG00000213963	64	104	61	130	170	77	97	77	96	130	96	277	55	117	65	140
ENSG00000271825	0	2	3	7	7	6	5	3	9	10	5	17	1	10	5	8
ENSG00000271996	2	0	5	6	9	6	4	7	5	5	5	24	5	6	3	17
ENSG00000278418	0	0	0	2	1	0	0	1	2	1	1	4	0	1	0	2
ENSG00000280374	15	20	10	36	34	16	18	11	15	28	18	59	14	26	16	23
ENSG00000237178	0	0	0	4	7	1	0	1	3	1	2	3	5	2	1	1
ENSG00000238295	1	0	1	2	3	0	2	1	0	3	0	0	0	5	0	2
ENSG00000018510	1756	3027	2429	2774	9141	3702	6614	3298	2877	4232	1802	6532	2974	3319	3312	3760
ENSG00000196659	203	222	270	238	127	40	66	46	66	84	62	190	60	95	80	126
ENSG00000237655	0	0	0	0	0	0	0	0	0	0	0	0	0	0	0	0
ENSG00000197557	173	211	214	191	97	46	93	54	89	116	92	188	62	102	94	112
ENSG00000128655	4	17	18	18	62	19	44	16	0	0	6	1	0	2	1	4
ENSG00000229941	1	6	9	4	79	23	61	22	0	0	0	0	0	0	0	0
ENSG00000236664	0	0	0	0	0	0	0	0	0	0	0	0	0	0	0	0
ENSG00000213962	0	0	0	0	0	0	0	0	0	0	0	0	0	0	0	1
ENSG00000229744	0	0	0	0	0	0	0	0	0	0	0	0	0	0	0	0
ENSG00000206788	0	0	0	0	0	0	0	0	0	0	0	0	0	0	0	0
ENSG00000249909	0	0	0	0	0	0	0	0	0	0	0	0	0	0	0	0
ENSG00000227241	0	0	0	0	0	0	0	0	0	0	0	0	0	0	0	0
ENSG00000155636	295	387	338	459	747	327	486	340	362	489	324	834	269	343	383	392
ENSG00000223096	0	0	0	1	0	0	0	0	0	0	0	0	0	0	0	0
ENSG00000079156	0	1	0	2	0	0	3	1	390	561	299	1054	8	37	17	27
ENSG00000223960	331	412	330	759	1848	804	1232	795	511	551	348	1127	488	790	674	878
ENSG00000180228	1140	1599	1135	1304	3861	1538	2758	1527	655	920	490	1379	1490	1729	1745	1973
ENSG00000204311	22	30	26	78	158	98	136	108	13	12	25	37	53	73	55	90
ENSG00000238082	3	0	0	3	32	10	15	17	2	2	2	0	8	13	6	15
ENSG00000079150	432	618	381	488	366	148	239	177	165	222	83	218	246	258	301	304
ENSG00000116095	414	592	549	720	1363	599	1108	496	657	855	620	1684	502	621	764	723
ENSG00000237298	195	289	205	386	6	5	3	8	128	179	150	489	122	237	183	221
ENSG00000155657	14	12	1	36	141	68	122	61	35	24	63	128	145	477	281	516
ENSG00000283186	0	0	0	0	0	0	0	0	0	0	0	0	0	0	0	0
ENSG00000270956	11	15	19	19	0	0	0	1	10	12	23	28	8	26	25	26
ENSG00000270277	15	7	6	27	1	0	0	2	12	17	7	36	10	21	18	44
ENSG00000279598	7	4	3	38	0	0	0	0	14	23	5	38	5	20	27	20
ENSG00000271011	1	4	2	2	0	0	0	0	2	1	0	3	2	1	1	1
ENSG00000270574	8	17	17	33	2	1	1	0	12	16	7	18	4	9	21	29
ENSG00000271141	6	5	10	23	0	0	0	0	9	13	5	30	1	4	4	8
ENSG00000271401	2	2	3	15	0	0	0	0	2	1	1	10	6	3	1	5
ENSG00000267784	1	0	0	2	2	3	0	1	1	4	2	3	3	9	7	13
ENSG00000163492	0	0	0	1	200	101	188	71	86	87	84	203	546	733	793	843
ENSG00000238542	0	0	0	0	2	0	0	2	1	0	0	2	0	7	6	4
ENSG00000231541	0	0	0	0	0	0	1	1	0	1	0	1	1	1	3	8
ENSG00000187231	264	532	377	765	3217	1349	2210	1350	756	1128	640	2173	399	736	404	764
ENSG00000237477	0	0	0	0	0	0	0	0	0	0	0	0	0	0	0	0
ENSG00000237964	0	0	0	0	0	0	0	0	0	0	0	0	0	0	0	0
ENSG00000144331	0	0	0	0	5	3	9	0	0	0	0	0	7	7	5	8
ENSG00000213126	0	0	0	0	0	0	0	0	0	0	1	0	0	0	0	0
ENSG00000221240	0	0	0	0	0	0	0	0	0	0	0	0	0	0	0	0
ENSG00000280636	0	0	0	0	0	0	0	0	0	1	1	0	0	3	1	0
ENSG00000202216	0	0	0	0	0	0	0	0	0	0	0	0	0	0	0	0
ENSG00000163510	2049	2099	2062	1713	3545	1418	2399	1666	769	1238	726	1947	1403	1435	1720	1864
ENSG00000270289	0	0	0	0	0	0	0	0	0	0	0	0	0	0	0	0
ENSG00000225258	0	0	0	0	0	0	0	0	0	0	0	0	1	0	0	0
ENSG00000281131	0	0	0	0	0	0	0	0	0	0	0	0	0	0	0	0
ENSG00000279559	0	0	0	0	0	0	0	0	0	0	0	0	0	0	0	0
ENSG00000226564	0	2	4	2	6	2	5	1	1	0	1	3	0	2	0	1
ENSG00000236667	0	0	0	0	0	0	0	0	0	0	0	0	0	1	0	0
ENSG00000170035	663	1270	1048	1338	0	0	1	0	957	1475	822	2298	617	771	656	936
ENSG00000236153	0	0	2	2	0	0	0	0	1	0	0	0	0	0	0	0
ENSG00000238171	0	0	0	0	4	1	0	0	2	0	0	1	0	4	3	2
ENSG00000234663	20	23	21	27	406	147	323	207	16	10	11	16	60	63	87	93
ENSG00000266705	0	0	0	0	6	3	3	2	0	0	0	0	0	0	0	1
ENSG00000226681	0	0	0	0	6	1	6	3	0	0	0	0	0	1	0	0
ENSG00000115232	5952	9275	7390	13088	26304	10139	17619	9791	2613	3622	1175	4680	2706	3461	2861	3493
ENSG00000188452	1282	2090	1582	2592	5455	1973	3535	2087	651	802	274	1037	647	797	681	909
ENSG00000162992	1	0	1	0	0	0	0	0	0	0	1	1	0	0	0	0
ENSG00000234595	0	0	0	0	0	0	0	0	0	0	0	0	0	0	0	0
ENSG00000225570	0	0	0	0	0	0	0	0	0	0	0	0	0	0	0	0
ENSG00000223873	1	0	0	0	0	0	0	0	0	0	0	0	0	0	0	0
ENSG00000221023	0	0	0	0	0	0	2	0	0	0	0	0	0	0	0	0
ENSG00000260742	0	2	1	8	19	8	8	14	9	14	14	30	17	38	19	27
ENSG00000138434	1995	2874	2558	3375	9012	3665	6323	3454	1350	2078	1152	3751	1946	2753	2388	3133
ENSG00000150722	32	38	44	55	64	22	52	24	0	0	1	0	15	12	18	20
ENSG00000213958	0	0	0	0	0	0	1	1	0	0	0	0	0	0	0	0
ENSG00000115252	2	0	0	0	58	24	33	22	2	5	1	3	2	1	9	4
ENSG00000242121	0	0	0	0	0	0	0	0	0	0	0	0	0	0	0	0
ENSG00000077232	1970	3040	2146	2512	5742	2354	4056	2290	2363	3615	1897	5067	3411	4077	4312	4776
ENSG00000232430	0	0	0	0	0	0	0	0	0	0	0	0	0	0	0	0
ENSG00000212404	0	0	0	0	0	0	0	0	0	0	0	0	0	0	0	0
ENSG00000162998	0	0	0	0	0	0	0	0	0	1	0	2	0	3	0	3
ENSG00000207178	0	0	0	0	0	0	0	0	0	0	0	0	0	0	0	0
ENSG00000061676	460	829	549	1128	0	0	0	0	369	802	523	1688	915	1459	1091	1632
ENSG00000202141	0	0	0	0	0	0	0	0	3	0	0	0	0	2	3	0
ENSG00000221498	0	0	0	0	0	0	0	0	0	0	0	0	0	0	0	0
ENSG00000231203	0	0	0	0	0	0	0	0	0	0	0	0	0	1	0	0
ENSG00000162999	0	0	0	0	0	0	0	0	108	113	128	240	91	126	101	116
ENSG00000224643	0	0	0	0	0	0	0	0	0	0	0	0	0	0	0	1
ENSG00000163002	731	931	952	746	1543	534	1046	637	487	666	185	824	832	834	1018	968
ENSG00000272800	24	28	14	29	73	16	28	28	21	25	6	32	11	17	8	30
ENSG00000213120	0	0	0	1	0	0	0	2	0	0	0	0	0	0	0	0
ENSG00000177855	17	44	29	18	17	9	9	8	7	7	5	13	16	14	9	11
ENSG00000234172	0	0	0	0	0	0	0	0	0	0	0	0	0	0	0	0
ENSG00000266808	0	0	0	0	0	0	0	0	0	0	0	0	0	0	0	0
ENSG00000170396	0	0	0	0	0	0	0	0	0	0	1	1	6	6	15	9
ENSG00000237824	0	0	0	0	0	0	0	0	0	0	0	0	0	0	0	0
ENSG00000225406	0	0	0	0	0	0	0	0	0	0	0	0	0	0	0	0
ENSG00000212581	0	0	0	0	0	0	0	0	0	0	0	0	0	0	0	0
ENSG00000226747	0	3	0	2	0	0	0	0	2	0	0	3	0	0	0	0
ENSG00000188738	2	3	15	25	26	11	14	31	29	17	8	46	2	16	10	19
ENSG00000231646	0	1	2	3	5	0	0	4	2	3	3	1	0	0	1	0
ENSG00000224019	1	1	1	2	1	2	4	3	0	8	2	4	0	2	0	2
ENSG00000237877	12	8	7	18	23	8	13	13	6	4	8	35	2	10	5	12
ENSG00000226410	1	1	2	4	3	3	2	1	5	1	1	4	1	2	0	2
ENSG00000232568	0	0	1	2	2	0	2	1	0	1	0	0	0	0	0	0
ENSG00000259915	3	1	2	4	13	3	13	4	4	2	0	18	0	11	3	9
ENSG00000227692	3	8	2	6	2	5	4	5	5	4	1	8	0	1	6	7
ENSG00000065548	3871	4344	3696	3699	6389	2463	4189	3302	1794	2802	1314	4005	3122	3070	3947	3720
ENSG00000224323	0	1	0	0	3	5	4	2	3	4	0	7	1	1	1	5
ENSG00000281192	2	1	0	1	2	1	0	2	1	1	6	9	0	0	1	0
ENSG00000138448	350	497	371	862	2113	816	1298	870	609	776	715	1903	200	470	303	537
ENSG00000227227	1	3	0	4	7	6	9	4	0	3	5	14	2	1	1	4
ENSG00000144369	157	449	191	375	1	0	0	0	134	211	149	449	89	150	75	142
ENSG00000163012	0	0	0	0	0	0	0	0	1	0	0	0	0	0	0	0
ENSG00000226553	0	0	0	0	0	0	0	0	0	0	0	0	0	0	0	0
ENSG00000224063	0	0	0	0	10	3	3	1	0	0	1	0	0	5	3	4
ENSG00000222845	0	0	0	0	0	0	0	0	0	0	0	0	0	0	0	0
ENSG00000199626	0	0	0	0	0	0	0	0	0	0	0	0	0	0	0	0
ENSG00000064989	1	2	0	3	14	5	21	10	0	0	0	1	28	32	32	39
ENSG00000231739	0	0	0	0	0	0	0	0	0	0	0	0	0	0	0	0
ENSG00000003436	2	8	14	11	683	250	529	318	0	0	1	0	58	135	106	150
ENSG00000231689	0	0	0	0	0	0	0	0	0	0	3	0	0	0	0	0
ENSG00000235616	0	0	0	0	0	0	0	0	0	0	0	0	0	0	0	0
ENSG00000213115	0	0	0	0	0	0	0	0	0	0	0	0	0	0	0	0
ENSG00000144366	0	0	0	0	0	0	0	0	27	36	58	74	12	22	26	30
ENSG00000207951	0	0	0	0	0	0	0	0	0	0	0	0	0	0	0	0
ENSG00000223523	0	0	0	0	9	7	5	8	0	0	0	0	0	0	0	1
ENSG00000174325	0	0	0	0	0	0	1	0	0	0	0	0	0	0	0	0
ENSG00000168542	0	0	0	0	29	12	9	23	0	0	0	0	24	45	38	47
ENSG00000221502	0	0	0	0	0	0	0	0	0	0	0	0	0	0	0	0
ENSG00000283238	0	0	0	0	0	0	0	0	0	0	0	0	0	0	0	0
ENSG00000204262	22	26	21	59	84	29	63	23	363	453	184	668	31	50	49	50
ENSG00000228073	0	0	0	0	0	0	0	0	0	0	0	0	0	0	0	0
ENSG00000264725	0	0	0	0	0	0	0	0	0	0	0	0	0	0	0	0
ENSG00000213601	0	0	3	0	0	0	1	0	0	0	0	0	0	2	3	1
ENSG00000115368	2679	3405	2837	3088	5928	2330	3946	2627	1490	2238	1147	3414	2351	2870	3184	3324
ENSG00000233996	0	0	0	0	2	1	2	2	0	2	2	3	0	0	2	0
ENSG00000138449	687	1206	942	1253	2813	953	1899	993	46	43	31	84	58	83	55	111
ENSG00000276828	0	0	0	0	0	0	0	0	0	0	0	0	0	0	0	0
ENSG00000138381	1744	2070	1851	1655	2792	1054	2006	1186	1058	1495	860	2467	1419	1383	1769	1718
ENSG00000151687	40	61	41	134	100	54	48	44	119	118	126	268	24	61	25	76
ENSG00000128694	301	409	309	318	409	173	293	194	254	375	259	662	146	191	99	187
ENSG00000253559	5	21	14	27	23	19	12	7	12	28	20	33	13	24	22	29
ENSG00000273240	5	8	1	4	18	4	7	1	4	12	10	17	3	9	3	4
ENSG00000128699	903	1284	1341	1208	3278	1400	2319	1486	1040	1443	1168	2434	1017	1142	1427	1435
ENSG00000064933	1329	1488	1258	1512	2486	1083	1726	1142	804	1205	638	1916	785	944	871	1124
ENSG00000187699	35	30	36	149	160	74	97	83	51	56	92	177	21	85	46	119
ENSG00000223824	0	0	0	1	0	0	0	0	0	0	0	0	0	0	0	0
ENSG00000204253	28	34	16	29	44	27	30	20	10	18	4	21	20	22	26	31
ENSG00000213110	0	0	0	0	0	0	0	0	0	1	0	0	0	0	0	0
ENSG00000138379	11	26	17	45	1	2	1	4	0	3	3	0	1	9	4	7
ENSG00000198130	625	889	780	774	2026	844	1459	837	476	595	409	1157	486	615	668	753
ENSG00000151689	0	0	0	0	736	367	587	314	323	497	328	1037	407	549	516	712
ENSG00000151690	578	990	874	1293	2013	830	1536	812	2278	3354	2226	5779	539	779	563	842
ENSG00000272979	9	16	12	12	15	3	12	7	13	7	5	8	4	5	5	21
ENSG00000189362	484	706	514	576	1083	449	764	429	451	617	304	1194	317	479	369	470
ENSG00000233654	22	29	21	28	24	8	9	10	30	38	7	81	1	3	2	10
ENSG00000251935	0	2	0	6	0	0	1	0	4	2	2	3	0	0	0	0
ENSG00000138386	516	743	632	1184	2897	1204	2060	1294	1570	2278	1119	3884	345	623	407	655
ENSG00000228509	2	0	1	9	17	10	8	13	7	9	10	28	0	9	3	13
ENSG00000235852	9	9	6	12	19	15	13	17	9	5	9	23	15	23	13	38
ENSG00000115419	1881	2775	3144	3793	9085	3751	6683	3757	3841	5490	3746	9367	4256	5021	4992	5393
ENSG00000115415	716	1225	1251	1360	3536	1370	2845	1378	2205	3236	1483	5855	8223	19425	13349	22738
ENSG00000229023	0	0	1	2	3	2	2	5	1	3	3	7	3	31	22	36
ENSG00000230686	0	0	0	0	0	0	0	0	0	0	0	0	0	3	0	1
ENSG00000231858	1	7	3	3	2	1	13	4	39	83	57	115	9	14	9	19
ENSG00000138378	12	17	30	26	150	45	137	58	1540	2273	1430	3603	251	325	474	412
ENSG00000207402	0	1	0	0	1	0	0	1	4	4	1	2	0	0	2	1
ENSG00000280083	0	1	4	5	0	0	0	0	0	0	0	0	0	0	0	1
ENSG00000230611	0	0	1	0	0	0	0	1	0	0	0	0	0	0	0	0
ENSG00000227542	2	11	3	13	13	0	8	3	0	0	0	0	0	0	1	0
ENSG00000128641	1231	2375	1823	2674	4786	1882	3507	1725	22	42	2	60	195	302	227	384
ENSG00000252130	0	0	0	2	1	1	1	4	0	0	0	0	0	0	0	0
ENSG00000173559	447	553	621	940	2123	1054	1403	1082	603	686	985	1561	2548	5294	4378	6372
ENSG00000274845	0	0	0	1	0	0	0	0	0	0	0	0	0	1	0	2
ENSG00000225884	0	0	0	0	0	0	0	0	0	0	0	0	0	0	0	0
ENSG00000168497	0	0	0	0	7	0	14	5	0	0	0	0	0	0	1	1
ENSG00000233766	0	0	0	0	0	0	0	0	0	0	0	0	0	1	0	0
ENSG00000213946	0	0	0	0	0	0	0	0	0	0	0	0	0	0	0	0
ENSG00000144339	0	0	0	2	0	0	0	0	0	0	0	0	0	0	0	0

ENSG00000229395	0	0	0	0	0	0	0	0	0	0	0	0	0	0	0	0	0	0	0	0	0	0	0	0	0	0	0	0	0	0	0	0
ENSG00000232227	0	0	0	0	0	0	0	0	0	0	0	0	0	0	0	1	0	0	0	0	0	0	0	0	0	0	0	0	0	3	0	1
ENSG00000227418	0	0	0	0	0	0	0	0	0	0	1	0	0	0	0	0	0	0	0	0	0	0	0	0	0	0	0	1	0	0	0	0
ENSG00000226242	0	0	0	0	0	0	0	0	0	0	0	0	0	0	0	0	0	0	0	0	0	0	0	0	0	0	0	0	0	0	0	0
ENSG00000278406	0	0	0	0	0	0	0	0	0	0	0	0	0	0	0	0	0	0	0	0	0	0	0	0	0	0	0	0	0	0	0	0
ENSG00000232658	0	0	0	0	0	0	0	0	0	0	0	0	0	0	0	0	0	0	0	0	0	0	0	0	0	0	0	0	0	0	0	0
ENSG00000235218	0	0	0	0	0	0	0	0	0	0	0	0	0	0	0	0	0	0	0	0	0	0	0	0	0	0	1	0	0	0	0	0
ENSG00000224219	0	0	0	0	0	0	0	0	0	0	0	0	0	0	0	0	0	0	0	0	0	0	0	0	0	0	0	0	0	0	0	0
ENSG00000260142	0	0	0	0	42	46	30	41	0	0	0	0	0	0	1	0	0	0	0	0	56	37	37	43	0	0	0	0	0	0	0	0
ENSG00000224670	0	0	0	0	0	2	0	2	0	0	0	0	0	0	0	0	0	0	0	0	0	0	0	3	0	0	0	0	0	0	0	0
ENSG00000215043	0	0	0	0	0	0	0	0	0	0	1	0	0	0	0	0	0	0	0	0	0	0	0	0	0	0	0	0	0	0	0	0
ENSG00000236695	0	0	0	0	0	0	0	0	0	1	2	0	0	0	0	0	0	0	0	0	1	0	1	0	1	1	0	0	0	0	0	0
ENSG00000225539	0	0	0	0	0	0	0	0	0	0	13	2	0	1	0	0	0	0	0	0	0	0	0	0	1	1	1	1	1	0	0	1
ENSG00000225394	0	0	0	0	0	0	0	0	0	0	0	0	0	0	0	0	0	0	0	0	0	0	0	0	0	0	0	0	0	0	0	0
ENSG00000276123	0	0	0	0	0	0	0	0	0	0	0	0	0	0	0	0	0	0	0	0	0	0	0	0	0	0	0	0	0	0	0	0
ENSG00000252517	0	0	0	0	0	0	0	0	0	0	0	0	0	0	0	0	0	0	0	0	0	0	0	0	0	0	0	1	0	0	0	0
ENSG00000230173	1	0	1	0	0	1	0	1	1	1	1	0	0	1	0	0	0	0	0	0	0	1	0	0	0	1	0	0	1	0	0	1
ENSG00000235056	0	0	0	0	1	0	0	0	3	0	1	0	0	0	0	0	0	0	0	0	0	1	2	1	0	0	0	1	0	0	0	0
ENSG00000224099	0	1	1	1	1	7	1	2	9	1	3	1	1	2	4	0	1	0	0	1	1	0	2	1	2	10	1	8	1	0	0	2
ENSG00000271893	1	0	5	2	0	0	1	0	0	0	0	0	0	0	4	3	1	0	2	0	0	0	2	0	0	1	0	0	2	0	0	1
ENSG00000202206	0	0	0	0	0	0	0	2	0	0	0	0	1	0	0	0	0	0	0	0	0	0	0	0	0	0	0	0	0	0	0	0
ENSG00000223466	6	7	4	3	5	6	11	3	52	39	157	23	8	9	19	20	3	8	3	3	14	5	11	10	38	51	50	94	25	23	13	25
ENSG00000234919	0	0	0	0	0	0	0	0	0	0	3	0	0	0	0	1	0	0	1	0	0	0	0	2	0	0	1	1	0	1	0	0
ENSG00000196950	1660	1247	1177	1136	1568	1688	1160	1749	1860	1665	7255	1068	1651	1571	1713	1956	1100	1802	1168	1636	3312	1279	2250	1337	1988	2531	2126	4264	1310	1461	1475	1688
ENSG00000213107	0	0	0	0	0	0	0	0	2	0	9	0	1	0	0	0	0	0	0	0	0	0	0	0	0	0	1	3	0	0	0	0
ENSG00000201813	3	0	0	0	1	1	2	0	4	5	32	1	2	3	0	2	1	0	0	0	2	0	1	3	10	5	14	11	1	4	0	1
ENSG00000118997	5	8	8	5	8	6	7	4	6	5	65	6	18	11	20	24	2	4	1	9	17	6	9	10	20	8	16	28	14	15	29	23
ENSG00000203435	0	0	0	0	0	0	2	0	2	0	0	0	0	0	0	2	0	0	0	0	0	0	0	0	0	1	1	1	1	0	0	0
ENSG00000227878	0	0	0	0	0	0	0	0	0	0	0	1	0	0	0	0	0	0	0	0	0	0	0	0	0	0	0	0	0	0	0	0
ENSG00000081320	1385	850	1300	903	2173	2348	1794	2349	5301	4540	27605	3398	3520	2880	3705	3507	703	726	1009	884	5013	2038	3599	2157	5080	6913	9889	17548	2311	2832	3088	3242
ENSG00000272211	37	30	34	20	48	59	53	85	143	133	754	71	80	72	86	77	9	7	9	17	132	85	70	82	124	149	172	456	26	58	48	106
ENSG00000138411	0	0	2	1	150	206	124	192	8	7	38	4	5	19	4	25	0	2	0	1	176	100	124	82	3	0	7	12	8	13	7	8
ENSG00000229056	0	0	1	0	2	3	2	1	25	10	94	16	4	5	12	3	1	0	2	2	5	3	6	3	9	16	20	37	6	7	10	5
ENSG00000276200	0	0	0	0	0	2	0	0	0	0	1	2	0	0	0	0	0	0	0	0	0	0	0	1	0	0	0	0	0	0	0	0
ENSG00000282836	0	0	0	0	0	0	0	0	0	0	0	0	0	0	0	0	0	0	1	1	0	0	0	0	0	0	0	0	0	0	0	1
ENSG00000144395	522	322	485	354	966	958	794	1018	342	266	929	200	314	282	367	440	283	236	319	489	1976	832	1425	1008	347	489	144	807	209	381	279	494
ENSG00000252923	1	0	1	0	1	1	0	0	0	0	0	0	2	0	2	1	0	0	0	1	1	0	1	0	3	0	0	1	0	0	0	1
ENSG00000119041	2326	1484	1804	1480	2602	2631	1897	2591	1329	966	5206	792	2198	1964	2166	2464	1896	2210	2045	1995	5826	2293	3710	2360	1355	1820	1283	3303	1850	2034	2257	2509
ENSG00000187944	6	6	1	6	22	34	25	45	1	2	3	1	1	5	2	1	0	5	2	3	58	18	35	15	0	0	0	1	1	7	1	9
ENSG00000280176	1	0	1	2	2	2	0	0	0	0	1	0	0	0	2	0	1	0	0	0	4	3	2	0	0	1	0	0	0	1	2	2
ENSG00000197121	1588	1134	1128	1035	1161	1336	862	1376	361	308	1240	185	861	833	848	1049	356	367	328	977	1713	841	1124	909	253	361	279	702	350	704	527	802
ENSG00000065413	112	81	99	89	224	231	185	254	810	783	3893	538	251	222	283	305	22	36	44	63	488	214	348	236	916	1160	1148	2558	175	273	252	287
ENSG00000283224	0	0	0	0	0	0	0	0	0	0	0	0	0	0	0	0	0	0	0	0	0	0	0	0	0	0	0	0	0	0	0	0
ENSG00000225636	1	0	1	1	2	4	1	10	8	10	43	6	4	3	4	5	0	2	0	1	11	3	2	8	12	13	22	54	3	4	7	12
ENSG00000224442	2	2	0	0	3	0	5	3	20	15	61	6	11	4	5	2	0	0	0	0	13	7	9	6	15	11	15	55	4	9	1	6
ENSG00000231621	1	0	0	0	4	3	0	4	2	3	30	3	5	4	5	10	0	0	1	2	12	8	8	4	8	7	11	35	3	5	2	10
ENSG00000236977	12	8	18	11	33	19	22	48	100	79	401	46	25	28	21	20	2	0	3	8	66	31	38	45	108	145	110	388	10	25	24	21
ENSG00000230482	4	0	4	1	7	6	7	19	35	26	129	14	8	5	1	4	0	0	1	2	15	8	7	6	12	31	31	91	1	6	4	10
ENSG00000225979	0	0	0	0	0	0	0	0	0	1	7	0	0	0	3	0	0	0	0	1	0	0	0	0	1	0	1	3	0	0	0	1
ENSG00000213104	1	1	0	0	0	0	1	0	1	0	3	1	2	2	2	2	5	0	0	1	1	0	0	1	1	0	0	0	1	1	1	6
ENSG00000115524	9857	6901	8436	6588	12594	12863	9748	14124	9083	7305	33353	5709	8594	7774	8720	10146	5561	7809	7454	7265	25787	10297	18972	9963	8621	12958	8695	21672	7387	8066	8956	9324
ENSG00000202434	3	1	1	3	2	3	5	4	2	1	10	3	2	0	1	1	1	1	0	2	2	5	1	7	0	6	3	7	1	0	6	1
ENSG00000206836	0	1	0	0	0	0	0	0	0	0	1	1	0	0	1	0	0	0	0	0	1	0	0	0	0	0	0	0	0	1	0	0
ENSG00000115520	377	313	394	296	408	346	343	429	229	177	1464	164	379	284	452	417	420	449	467	365	972	353	723	402	369	499	382	962	368	393	517	440
ENSG00000144381	25500	15504	18372	15791	15028	14601	11875	15158	16093	10914	40043	8592	39858	34868	38259	43597	28917	38409	30533	25396	33395	12084	21443	11623	16342	22904	6643	28261	43215	39952	44347	46389
ENSG00000115541	5573	3012	4057	3386	3602	3669	2691	3968	4145	2656	10882	2260	7629	6620	8272	8833	8018	8338	8862	8432	8444	3468	6045	4603	4325	5767	1776	6806	10096	8500	10887	10324
ENSG00000270757	21	12	5	8	10	10	13	7	3	3	13	1	3	7	9	11	23	23	15	20	12	4	12	5	5	8	1	10	9	8	24	11
ENSG00000115540	628	380	524	382	887	878	739	942	475	409	1784	320	649	643	843	764	747	894	843	702	2653	1040	1872	1255	549	750	423	1247	877	843	1181	1144
ENSG00000231699	1	0	1	0	2	0	1	0	0	0	2	0	0	1	1	0	0	0	0	2	1	1	2	4	1	0	0	7	0	0	0	1
ENSG00000162944	29	36	36	18	7	5	4	16	14	8	34	7	61	63	64	79	23	13	8	38	13	11	7	3	9	11	19	24	32	49	46	72
ENSG00000222017	7	11	23	10	1	0	0	0	0	0	8	1	10	21	14	14	6	8	11	15	0	0	0	0	0	1	0	9	18	11	9	21
ENSG00000247626	635	398	564	496	543	594	416	592	629	511	2217	438	1110	814	1237	1101	295	415	418	640	1151	496	868	460	726	1068	455	1476	1074	1196	1154	1277
ENSG00000152430	33	28	27	18	1	1	0	1	0	1	11	0	12	13	18	22	5	8	11	19	2	1	1	0	1	1	2	7	7	13	11	21
ENSG00000115896	32	48	51	47	68	162	62	179	1	6	17	4	111	132	117	171	16	20	18	38	97	72	69	41	1	1	6	19	56	145	99	174
ENSG00000237035	0	0	0	0	0	0	0	0	0	0	0	0	0	0	0	0	0	0	0	0	0	0	0	0	0	0	0	0	0	0	0	0
ENSG00000236653	0	0	0	0	0	1	0	0	0	0	1	0	0	0	0	0	0	0	0	0	0	0	0	0	0	0	0	1	0	0	1	0
ENSG00000225421	3	1	4	0	5	3	0	6	0	0	0	0	1	1	2	3	0	0	1	2	1	1	2	4	0	0	0	0	1	0	1	2
ENSG00000231557	0	0	0	0	0	0	0	0	0	0	0	0	0	0	0	1	0	0	0	0	2	1	0	0	0	0	0	0	0	0	0	1
ENSG00000230407	0	0	0	0	0	0	0	0	0	0	0	0	0	0	0	0	0	0	0	0	1	0	0	0	0	0	0	0	0	0	1	0
ENSG00000238698	0	0	0	0	0	0	0	0	0	0	0	0	0	0	0	0	0	0	0	0	0	0	0	1	0	0	0	0	0	0	0	1
ENSG00000119042	385	468	433	480	2	2	1	1	527	464	1266	263	573	675	620	844	99	188	120	319	1	2	0	0	398	614	250	870	276	637	410	703
ENSG00000257045	2	1	2	1	0	0	0	0	3	0	6	0	3	2	5	3	2	0	0	0	1	0	0	0	0	3	0	6	0	0	0	3
ENSG00000225953	32	35	39	43	0	0	0	0	42	36	103	16	33	22	23	44	3	21	16	25	0	0	0	0	18	31	16	88	14	25	4	27
ENSG00000238217	3	0	1	0	0	0	0	0	0	0	1	0	0	0	0	0	0	1	0	0	0	0	0	0	0	0	0	0	0	0	0	0
ENSG00000213938	0	0	1	0	0	0	0	0	0	2	5	0	0	0	0	1	0	1	1	0	0	0	0	0	0	0	0	2	0	0	0	1
ENSG00000226124	54	61	58	53	0	1	0	0	28	30	80	10	47	61	49	60	36	109	59	74	0	0	3	0	27	35	13	58	27	59	27	55
ENSG00000232732	0	0	1	1	9	19	11	11	29	19	97	11	15	24	23	16	1	0	0	7	22	19	14	14	27	40	6	102	11	18	23	38
ENSG00000227004	0	0	0	0	2	0	0	0	0	0	0	0	0	0	1	0	0	0	0	0	0	0	0	1	0	0	0	0	0	1	0	0
ENSG00000240302	0	0	0	0	1	1	0	0	0	0	8	1	1	0	0	0	0	0	0	0	0	0	0	0	0	2	2	3	0	2	0	0
ENSG00000178074	846	618	646	639	624	628	433	693	471	413	1537	291	474	473	507	514	397	555	399	642	1240	446	750	451	550	762	277	1160	368	476	381	520
ENSG00000162971	498	380	409	358	544	552	369	589	488	399	1786	280	405	395	495	490	199	224	202	396	943	445	545	406	354	548	431	1074	303	457	454	554
ENSG00000162972	384	256	297	229	342	372	266	310	237	190	655	140	416	358	481	435	422	758	613	473	904	331	586	325	231	328	121	521	615	524	667	558
ENSG00000196141	4	21	2	20	3	3	3	6	477	350	1507	273	735	856	809	1157	2	20	8	23	1	1	3	3	548	782	357	1486	680	1169	723	1142
ENSG00000201649	0	0	0	0	0	0	0	0	0	0	6	0	0	0	0	0	0	0	0	0	0	0	1	0	1	0	6	5	0	3	0	1
ENSG00000155729	475	326	373	341	403	458	338	491	446	406	1756	265	350	372	351	483	147	170	162	289	605	277	460	222	362	513	454	1173	218	359	211	359
ENSG00000163535	2719	1934	2272	1951	1850	1860	1303	2049	1562	1252	3659	925	825	766	885	1102	2329	2785	2770	2205	3758	1338	2235	1501	1512	2274	481	3294	907	1024	1113	1168
ENSG00000138356	0	2	6	2	3	1	0	5	1	1	8	3	3	3	2	1	5	6	9	6	6	1	1	1	0	1	0	5	0	0	0	3
ENSG00000244301	2	2	1	0	1	0	1	0	0	0	0	0	0	0	0	0	2	0	0	0	0	0	0	0	0	0	1	0	0	0	0	0
ENSG00000280298	0	0	2	0	0	0	0	2	0	0	1	0	0	0	0	0	0	0	0	1	0	0	1	0	0	0	0	2	0	1	0	0
ENSG00000243478	0	0	3	2	1	0	4	4	0	0	0	0	0	0	0	0	0	0	0	1	0	0	0	0	0	0	0	0	0	0	0	0
ENSG00000237166	0	0	2	0	1	2	0	0	0	0	3	0	0	2	1	0	1	0	0	0	0	1	0	0	0	0	5	4	1	0	0	0
ENSG00000201737	0	0	0	0	0	0	0	0	0	0	0	0	0	0	0	0	0	0	0	0	0	0	0	0	0	0	0	0	0	0	0	0
ENSG00000230408	1	1	1	0	1	3	3	6	0	4	13	2	9	0	5	3	3	3	1	4	11	4	5	4	3	5	3	15	2	7	0	4
ENSG00000082153	3750	2217	3027	2263	5950	5833	4858	6492	5427	3855	17814	3059	4341	3844	5480	5135	4941	6342	5697	4388	18018	6521	12060	6958	5816	8019	3883	12092	6747	6173	8686	7785
ENSG00000207116	1	0	1	0	1	0	0	2	1	0	1	0	1	0	0	0	0	0	0	0	3	0	0	0	0	0	0	1	0	0	0	0
ENSG00000224346	1	4	0	0	0	1	2	3	0	0	2	0	0	1	0	0	0	1	0	0	3	1	2	0	0	0	0	1	0	0	1	0
ENSG00000013441	1560	994	1221	823	1907	2215	1772	2456	1112	963	5533	833	781	842	978	1090	1022	1078	1209	1495	4964	2288	3553	3003	1438	1897	1981	3609	1015	1311	1356	1612
ENSG00000252759	13	6	1	4	14	12	3	8	6	5	21	4	0	1	1	3	2	1	0	2	9	5	6	1	4	3	7	17	1	7	0	13
ENSG00000240344	814	567	604	496	842	1046	775	1128	831	648	2301	447	762	721	855	951	670	734	774	827	1811	854	1196	973	624	853	506	1432	876	954	1043	1031
ENSG00000201499	11	8	5	7	23	28	11	22	5	5	27	7	5	11	2	12	2	3	2	3	31	15	16	10	14	5	2	15	2	3	2	4
ENSG00000196290	835	524	714	531	840	848	606	932	701	583	2113	435	706	678	794	838	694	933	794	755	1742	673	1207	688	736	1055	410	1388	794	914	916	987
ENSG00000252916	7	5	4	6	2	5	7	6	7	1	11	2	6	6	8	3	5	1	1	4	14	3	7	9	3	6	2	7	4	16	4	8
ENSG00000115942	1616	1103	1299	1042	1871	1981	1453	2118	1566	1278	4861	871	1446	1220	1551	1600	612	869	805	1224	2888	1212	2140	1262	1638	2114	1102	3332	1111	1468	1255	1507
ENSG00000183308	4	7	6	5	24	28	16	26	6	7	37	11	9	6	7	2	0	3	0	9	34	29	54	23	10	19	6	22	1	8	6	7
ENSG00000155744	1958	1279	1510	1245	887	908	693	940	1408	1227	6469	988	1503	1302	1659	1547	683	776	749	1231	2032	878	1460	881	1796	2550	1545	4251	1180	1642	1504	1837
ENSG00000225695	14	11	4	9	4	2	4	14	8	4	30	7	10	10	4	5	5	9	1	12	13	6	3	3	11	13	11	34	5	13	10	10
ENSG00000226658	12	4	8	6	1	1	0	2	2	4	20	5	2	2	4	4	2	3	0	2	3	2	2	0	13	7	7	28	0	3	10	4
ENSG00000119013	1143	637	815	668	707	715	571	722	790	569	2663	490	709	624	817	838	1055	1075	1365	1281	1770	747	1452	1055	891	1152	630	1784	1004	832	1124	1141
ENSG00000252148	1	1	0	1	0	0	2	0	0	2	2	2	2	0	0	1	1	0	1	0	2	1	2	0	1	5	5	2	1	3	0	5
ENSG00000273975	0	2	4	2	0	0	0	0	3	6	8	1	1	3	0	4	0	1	0	1	1	0	1	0	0	0	2	7	1	1	0	2
ENSG00000232719	6	4	10	4	1	0	1	1	6	8	30	3	4	8	5	3	0	0	2	3	2	0	2	1	7	4	3	10	3	5	1	5
ENSG00000224672	0	0	0	1	1	0	0	0	2	0	6	0	0	2	1	1	0	0	0	0	0	0	0	0	1	0	0	0	0	1	0	1
ENSG00000003402	850	617	911	688	1732	1836	1261	2022	4600	3704	18494	2986	2085	2104	2346	2708	289	344	302	478	2519	1126	1580	939	3376	5126	3212	9219	1348	1998	1882	2384
ENSG00000232133	1	0	0	0	0	0	0	2	4	3	12	2	4	1	2	0	0	0	0	0	1	0	0	2	0	1	2	3	1	2	0	2
ENSG00000226312	24	17	9	10	14	20	7	25	80	99	377	38	52	33	48	42	6	6	1	15	40	24	11	31	82	108	61	268	19	39	57	47
ENSG00000238829	0	0	1	1	3	2	0	0	9	4	22	6	5	3	8	4	0	0	0	1	0	2	2	0	8	4	1	15	0	1	6	6
ENSG00000235579	0	0	0	0	3	2	1	0	0	2	8	2	4	1	2	1	0	0	0	0	1	2	0	0	1	3	1	6	0	3	0	3
ENSG00000234431	0	0	0	0	0	0	0	0	0	0	0	0	0	0	0	0	0	0	0	0	0	0	0	0	0	0	0	0	0	0	0	0
ENSG00000003400	146	56	94	63	1631	1659	1311	1752	409	331	1997	236	436	395	360	456	42	30	52	43	3322	1186	2254	1164	324	396	454	970	323	341	314	448
ENSG00000227348	1	0	0	0	2	4	5	10	0	0	11	1	3	1	5	6	0	0	0	0	16	10	9	3	1	0	0	9	0	5	1	6
ENSG00000225796	1	0	3	0	8	9	4	13	5	0	6	1	7	15	2	5	1	0	1	0	12	19	5	18	2	6	2	4	6	1	3	4
ENSG00000235318	2	0	0	0	2	1	2	2	0	0	1	0	2	4	0	0	0	0	0	1	6	2	1	2	0	0	1	5	0	1	3	0
ENSG00000064012	1161	627	989	678	1089	1105	782	1177	1500	1208	6518	1128	871	859	915	1121	670	832	943	950	2105	878	1492	861	1340	1768	1857	4206	754	1018	894	1174
ENSG00000155749	28	14	24	16	30	25	18	28	33	27	107	17	9	13	11	12	6	11	9	26	28	21	11	14	19	44	19	81	6	21	17	16
ENSG00000115993	623	388	562	421	765	759	561	779	929	760	4653	728	463	461	555	590	294	358	281	400	1087	443	794	451	1078	1562	1244	3160	442	594	573	706
ENSG00000082146	789	561	626	511	620	617	421	647	704	507	2129	398	436	456	356	531	599	873	634	657	1231	410	850	456	600	875	558	1499	415	500	361	511
ENSG00000213090	10	4	6	5	3	5	6	3	2	2	12	1	10	3	3	5	2	2	4	2	10	4	3	9	9	8	8	19	1	8	7	5
ENSG00000202008	0	0	0	0	1	0	0	0	0	1	1	0	0	0	0	0	0	0	0	1	0	0	0	0	0	1	0	0	0	2	1	0
ENSG00000155754	14	15	23	15	5	6	3	4	5	7	66	2	2	11	2	7	5	2	4	16	7	4	3	3	4	6	20	29	2	5	4	8
ENSG00000234842	3	0	0	0	0	0	0	0	0	0	0	0	0	0	0	0	0	0	0	0	0	0	0	0	0	0	0	0	0	0	0	0
ENSG00000224299	0	0	0	0	0	0	0	0	0	0	0	0	0	0	0	0	0	0	0	0	0	0	0	0	0	0	0	0	0	0	0	0
ENSG00000227736	0	0	0	1	0	0	0	0	0	0	0	0	0	0	0	0	0	0	0	0	0	0	0	0	0	0	0	0	0	0	0	0
ENSG00000224590	0	0	0	0	0	0	0	0	0	0	0	0	0	0	0	0	0	0	0	0	0	0	0	0	0	0	0	0	0	0	0	0
ENSG00000234731	0	0	0	0	0	0	0	1	0	0	0	0	0	0	0	0	0	0	0	0	0	0	0	0	0	0	0	0	0	0	0	0
ENSG00000249177	0	0	0	0	0	0	0	0	0	0	1	0	0	0	0	0	0	0	0	0	0	0	0	0	0	0	0	0	0	0	0	0
ENSG00000227841	0	0	0	1	0	0	0	0	0	0	0	0	0	0	0	0	0	0	0	0	0	0	0	0	0	0	0	3	0	0	0	0
ENSG00000155755	1326	930	1184	826	1216	1344	1058	1367	603	493	1917	369	627	585	716	851	1219	1568	1534	1534	2925	1080	2008	1201	563	840	349	1401	665	799	800	992
ENSG00000241790	0	0	0	0	0	0	0	0	0	0	0	0	0	0	0	0	0	0	0	0	0	0	0	0	0	0	0	0	0	0	0	0
ENSG00000082126	3	6	4	7	3	3	1	0	4	6	28	3	2	1	0	3	5	2	5	11	3	0	1	4	4	5	6	4	0	4	2	3
ENSG00000222972	0	0	0	0	0	0	0	0	0	0	0	0	0	0	0	0	0	0	0	0	0	0	0	0	0	0	0	0	0	0	0	0
ENSG00000003393	1134	793	942	872	634	702	511	714	463	494	2439	406	506	574	515	654	467	678	529	869	1017	407	772	357	491	784	748	1717	379	564	416	669
ENSG00000230799	17	2	5	3	6	10	4	8	1	3	13	3	1	4	4	5	0	1	0	8	5	2	3	8	2	6	6	19	1	0	1	4
ENSG00000138395	0	0	1	0	0	0	0	0	0	0	2	0	0	0	0	0	0	0	0	2	0	0	1	1	0	0	1	0	0	0	0	0
ENSG00000202137	0	0	0	0	0	0	0	0	0	0	0	0	0	0	0	0	0	0	0	0	0	0	0	0	0	0	0	0	0	0	0	0
ENSG00000231124	0	0	0	0	0	0	0	0	0	0	0	0	0	0	0	0	0	0	0	0	0	0	0	0	0	0	0	0	0	0	0	0
ENSG00000212184	0	0	0	0	0	0	0	0	0	0	0	0	0	0	0	0	0	0	0	0	0	0	0	0	0	0	0	0	0	0	0	0
ENSG00000273209	0	0	2	2	5	8	2	5	6	1	3	0	0	2	1	1	0	0	1	0	7	1	6	4	0	1	0	0	2	3	3	1
ENSG00000155760	57	147	65	135	252	245	155	242	123	107	327	91	115	163	146	216	15	37	11	48	299	146	220	120	83	119	198	281	50	103	72	140
ENSG00000182329	3	3	1	4	2	7	0	1	4	3	10	3	0	3	3	8	0	1	1	1	5	2	7	3	5	6	0	6	1	3	2	3
ENSG00000283310	0	0	0	0	0	0	0	0	0	0	0	0	0	0	0	0	0	0	0	0	0	0	0	0	0	0	0	0	0	0	0	0
ENSG00000222035	0	0	0	0	0	1	1	2	0	0	1	1	1	4	2	1	0	0	0	2	0	0	1	0	0	1	2	5	1	0	1	4
ENSG00000231903	0	0	0	1	0	0	0	0	1	0	1	0	0	1	1	0	3	6	2	1	0	1	1	0	0	2	1	1	0	0	0	2
ENSG00000227890	0	0	0	0	0	0	0	0	0	0	0	0	0	0	0	0	0	0	0	0	0	0	0	0	0	0	0	1	0	0	0	0
ENSG00000223825	7	5	2	0	0	1	0	0	2	1	3	1	4	2	3	0	1	1	0	1	1	0	1	2	0	1	0	5	1	3	0	2
ENSG00000116030	4104	2489	3057	2885	3238	3667	2511	3865	2951	2151	8771	1604	3042	2862	3048	3711	3751	4458	4309	3966	8236	3384	5938	3592	2884	3691	2024	5947	3473	3514	3963	4171
ENSG00000224463	3	2	1	1	3	1	1	0	3	3	9	2	0	1	0	0	0	1	0	2	3	1	2	4	2	2	1	7	3	1	4	0
ENSG00000055044	3569	2267	2757	2235	3284	3350	2475	3505	3969	2597	10309	1972	4878	4405	5600	5975	4514	5500	5032	4317	8860	3484	6141	3440	3432	4950	2103	7169	6389	6382	8109	7782
ENSG00000212534	8	1	1	0	1	3	3	4	4	5	7	2	10	3	5	3	1	0	2	7	4	5	6	8	3	7	3	16	2	4	10	17
ENSG00000212309	4	4	2	1	1	1	0	2	0	0	10	1	3	2	1	3	1	1	0	1	4	1	1	6	4	4	0	7	0	4	2	10
ENSG00000271852	3	3	3	0	3	4	1	5	3	2	8	0	3	2	5	4	0	1	1	1	7	4	2	5	3	2	0	8	6	1	11	4
ENSG00000238317	10	8	16	10	11	12	9	19	18	13	56	8	22	11	11	15	6	11	6	13	31	17	22	26	12	18	15	42	14	36	28	18
ENSG00000264275	0	0	1	2	1	0	0	0	6	4	29	9	1	1	0	0	0	0	0	1	0	0	0	0	4	5	6	9	1	1	1	2
ENSG00000226261	1	0	2	1	1	0	0	0	19	23	67	11	0	2	2	2	0	1	0	1	0	0	1	0	4	18	8	40	1	2	0	1
ENSG00000272966	0	0	1	0	1	0	1	2	22	11	39	7	0	2	0	2	0	0	0	2	0	0	0	0	5	9	8	23	1	1	1	1
ENSG00000242696	3	1	0	0	3	0	1	0	3	1	18	1	1	0	0	0	0	0	1	0	0	0	0	0	0	4	0	3	0	0	0	0
ENSG00000273456	20	20	32	41	12	7	7	8	9	9	48	4	7	9	11	14	17	11	8	17	17	5	10	5	8	12	8	28	11	8	14	11
ENSG00000204217	648	554	696	514	994	1095	687	1176	907	906	5090	647	1219	1094	1127	1398	173	222	226	409	1297	456	862	493	974	1481	1869	3175	840	1182	992	1189
ENSG00000270915	0	0	0	0	0	1	0	0	0	0	2	0	0	0	0	0	0	0	0	0	0	0	0	2	0	5	0	1	0	0	1	0
ENSG00000236047	5	3	4	2	2	9	2	2	3	2	18	2	5	7	2	2	0	0	2	4	9	3	0	2	0	4	9	13	1	5	7	5
ENSG00000223619	2	1	0	0	0	0	0	0	0	0	0	0	0	0	0	0	0	0	0	0	0	0	1	0	0	0	0	0	0	0	0	0
ENSG00000233888	0	0	2	0	0	0	0	0	0	0	0	0	0	0	0	0	0	0	0	0	1	0	0	1	0	0	0	0	0	1	0	0
ENSG00000235433	0	0	0	0	0	0	0	0	0	0	1	0	0	0	1	0	0	0	0	0	2	0	0	0	0	0	0	0	0	0	0	0
ENSG00000237542	2	0	0	0	1	0	0	0	0	0	0	0	0	0	0	0	0	0	0	0	0	0	1	0	0	0	0	0	0	1	0	0
ENSG00000233134	0	0	2	0	0	0	0	0	0	0	1	0	0	0	0	0	0	0	0	0	0	0	0	0	0	0	0	0	0	0	0	0
ENSG00000241221	0	0	0	0	0	0	0	0	0	0	1	0	0	0	0	0	0	0	0	0	0	0	0	0	0	0	0	0	0	0	0	0
ENSG00000227454	0	0	0	0	1	0	0	0	0	0	1	0	0	0	0	0	0	0	0	0	2	0	0	0	0	0	0	0	0	0	0	0
ENSG00000233311	2	0	2	0	0	0	0	0	0	0	1	0	0	0	0	0	0	0	2	1	0	0	0	0	0	0	0	0	0	0	0	0
ENSG00000138439	1097	862	1093	794	1442	1509	1173	1614	449	413	1908	262	954	963	1002	1191	201	322	428	496	2000	773	1366	699	384	543	453	889	814	1048	750	1065
ENSG00000163596	292	204	276	205	342	388	258	471	146	135	591	100	180	160	192	193	148	207	196	269	630	260	353	256	110	190	143	413	98	174	186	201
ENSG00000240761	0	0	0	0	0	0	0	0	0	0	0	0	0	0	0	0	0	0	0	0	0	0	0	0	0	0	0	0	0	0	0	0
ENSG00000233579	4	1	2	4	7	9	1	3	3	1	9	2	3	1	1	5	0	1	1	3	5	8	2	6	2	1	3	11	2	5	8	5
ENSG00000138442	1785	1072	1335	1091	1528	1504	1162	1574	1489	1173	4354	768	2373	2020	2337	2408	1814	2515	1944	1988	3563	1327	2428	1198	1430	2043	824	2852	2501	2366	2709	2747
ENSG00000138380	532	425	536	451	308	363	251	358	517	451	2234	341	285	275	293	397	175	257	246	390	471	202	320	205	307	452	500	1155	101	239	141	238
ENSG00000232479	10	4	7	7	2	5	1	7	2	3	9	2	2	0	2	0	7	8	7	9	8	1	6	7	3	2	5	11	3	2	2	4
ENSG00000270664	6	2	0	0	0	1	0	0	0	0	5	0	0	0	0	1	1	0	0	0	0	0	0	0	1	1	0	1	0	0	1	0
ENSG00000144426	545	367	398	431	329	371	257	381	543	450	2479	389	512	536	451	577	163	192	153	343	446	189	304	195	522	702	714	1654	185	347	294	409
ENSG00000232994	5	4	4	3	1	1	0	1	3	0	4	0	2	2	1	4	0	2	0	4	0	2	0	0	4	4	1	10	0	1	2	1
ENSG00000202059	1	1	2	1	0	0	0	0	0	1	0	1	1	1	0	0	0	0	1	0	2	1	0	2	1	0	2	5	2	3	0	0
ENSG00000228513	7	2	1	1	4	1	1	2	1	1	7	0	1	1	3	0	0	0	0	2	6	2	2	4	0	0	0	13	0	3	1	0
ENSG00000225124	0	0	0	0	0	0	0	1	0	0	2	0	0	4	0	1	0	0	0	1	0	0	0	0	2	0	0	1	0	1	0	0
ENSG00000204196	0	0	0	0	0	0	0	0	0	0	0	0	0	0	0	0	0	0	0	0	0	0	0	0	0	0	0	0	0	0	0	0
ENSG00000119004	371	275	311	244	306	359	292	396	303	233	1243	206	244	250	326	367	261	371	371	356	935	349	689	349	323	490	338	933	310	358	406	398
ENSG00000264041	0	0	0	0	0	0	0	1	2	0	0	0	1	0	2	0	0	0	0	0	0	1	0	0	0	1	1	2	0	1	0	4
ENSG00000238285	0	2	1	0	3	3	2	4	1	1	6	0	0	3	0	0	0	0	0	2	2	1	1	0	1	1	2	2	2	1	0	0
ENSG00000138443	3131	2445	2705	2422	2926	3150	2160	3261	2581	2354	8987	1613	1774	1711	1813	2266	645	931	989	1586	3497	1564	2537	1364	2164	3266	1919	5618	1031	1608	1175	1582
ENSG00000256458	2	1	2	0	2	3	3	0	1	0	6	2	0	0	1	0	0	0	0	1	1	2	5	6	2	1	4	6	0	1	1	6
ENSG00000173166	31	25	37	28	22	36	13	39	15	5	28	4	23	17	20	27	7	5	6	30	27	17	15	16	5	6	4	21	9	31	21	27
ENSG00000233459	0	0	0	0	0	0	0	1	0	0	0	0	0	0	0	0	0	0	0	0	0	0	0	0	0	0	0	0	0	0	0	0
ENSG00000233031	0	0	0	0	1	0	0	2	0	0	0	0	0	0	0	0	0	0	0	0	2	0	0	0	0	0	0	0	0	0	0	0
ENSG00000178562	1451	825	1398	866	828	947	750	1077	0	0	0	0	5	2	14	5	634	778	1129	1146	1345	648	1089	513	0	0	0	0	7	6	12	15
ENSG00000224791	0	0	0	0	2	2	2	1	0	0	0	0	0	0	0	0	1	0	2	0	5	0	1	0	0	0	0	0	0	0	0	0
ENSG00000213925	0	0	0	0	0	1	0	1	0	0	0	0	0	0	0	0	0	0	0	0	1	0	0	0	0	0	0	0	1	0	0	1
ENSG00000206970	0	0	0	0	0	0	0	0	0	0	0	0	0	0	0	0	0	0	0	0	0	0	0	0	0	0	0	0	0	0	0	0
ENSG00000163599	0	0	0	0	0	0	2	1	0	0	0	0	2	1	1	2	0	0	0	0	1	0	0	0	0	0	0	0	0	0	0	0
ENSG00000163600	10	7	15	2	0	2	0	2	381	318	1353	205	332	322	387	434	15	22	38	12	0	0	2	0	373	574	449	1052	567	653	916	780
ENSG00000235951	0	0	0	0	0	0	0	0	0	0	0	0	0	0	0	0	0	0	0	0	0	0	0	0	0	0	0	0	0	0	0	0
ENSG00000234714	0	0	0	0	0	0	0	0	0	0	0	0	0	2	0	0	0	0	0	0	0	0	0	0	0	0	0	0	2	2	2	0
ENSG00000237271	0	0	0	0	0	0	0	0	0	0	0	0	1	0	0	1	0	0	0	0	0	0	0	0	0	0	0	0	0	0	0	0
ENSG00000236681	0	0	0	0	0	0	0	0	0	0	0	0	0	0	0	1	0	0	0	0	0	0	0	0	0	0	0	0	0	0	0	0
ENSG00000225304	0	0	0	0	0	0	0	1	0	0	0	0	0	0	0	0	0	0	0	0	0	0	0	0	0	0	0	0	0	0	0	0
ENSG00000236634	0	0	0	0	0	0	0	0	0	0	0	0	0	0	3	0	0	0	0	0	0	0	0	0	0	0	0	0	0	0	0	1
ENSG00000237843	0	0	0	0	0	0	0	0	0	0	3	0	0	0	0	0	0	0	0	0	0	0	0	0	0	0	0	0	0	0	2	0
ENSG00000116117	0	2	0	0	7	4	3	6	140	134	500	101	299	328	433	414	0	0	1	1	2	2	6	2	103	206	100	451	207	343	321	384
ENSG00000223890	0	0	0	0	0	0	0	0	2	0	2	0	0	1	2	2	0	0	0	0	0	0	0	0	0	2	0	1	0	2	1	5
ENSG00000118257	1	0	0	0	1	0	0	3	2	4	47	6	1235	1232	1483	1582	0	0	0	0	1	2	1	0	5	18	35	56	498	918	855	1177
ENSG00000225216	0	0	0	0	0	0	0	0	0	0	1	0	10	4	8	11	0	0	0	0	0	0	0	0	0	0	0	2	1	5	6	11
ENSG00000201875	0	0	0	0	0	0	0	0	0	0	0	0	0	0	0	0	0	0	0	0	0	0	0	0	0	0	0	0	0	0	0	0
ENSG00000213739	0	0	0	0	0	0	0	0	0	0	1	0	0	0	0	0	0	0	0	0	0	0	0	0	0	0	0	0	0	0	0	0
ENSG00000225610	0	1	2	0	1	0	0	1	1	0	0	0	0	0	1	0	0	0	0	0	3	1	1	0	0	0	3	1	0	1	0	3
ENSG00000114933	735	510	599	510	1631	1634	1240	1758	855	817	3600	582	1186	1120	1072	1339	248	290	292	740	3172	1407	2009	1358	898	1338	1066	2619	614	1040	872	1243
ENSG00000252485	0	0	0	0	2	2	0	1	1	0	1	0	0	0	0	0	0	0	0	0	0	2	1	0	0	0	1	0	0	1	0	1
ENSG00000207386	0	0	1	2	2	2	4	0	1	0	1	2	0	0	0	0	0	0	0	2	2	3	0	2	0	4	0	1	0	0	1	1
ENSG00000237041	1	1	0	0	4	5	0	6	0	2	1	1	1	0	2	1	0	0	0	1	6	6	0	7	2	2	0	3	1	0	1	0
ENSG00000227946	20	18	23	7	299	270	218	245	47	57	274	31	22	31	22	40	2	16	11	15	454	197	323	116	43	76	68	129	22	31	30	30
ENSG00000023228	2663	1814	2239	1704	3537	3458	2998	3648	2319	1752	8487	1330	3793	3348	3939	4287	1684	2650	2209	2104	8978	3299	6748	2863	2704	3944	1900	6193	3575	4069	4283	4530
ENSG00000231955	0	0	0	0	0	0	0	0	0	0	0	0	0	0	0	0	0	0	0	0	0	0	0	0	0	0	0	0	0	0	0	0
ENSG00000237580	0	0	0	0	0	0	0	0	0	0	0	0	0	0	0	0	0	0	0	0	0	0	0	0	0	0	0	0	0	0	0	0
ENSG00000114942	7518	5061	4779	4604	4371	4321	2802	4539	9101	7150	28036	5546	9143	8421	8889	10929	9288	11527	8860	7736	11605	4320	7442	4657	8515	11653	5869	17866	11318	9840	13491	13012
ENSG00000277502	0	0	0	0	0	0	0	0	0	0	0	0	0	0	0	0	0	0	0	0	0	0	0	0	0	0	0	0	0	0	0	0
ENSG00000281780	13	6	4	14	12	8	6	9	3	11	23	2	9	9	17	8	4	5	7	4	21	12	12	8	8	12	5	23	3	11	6	10
ENSG00000207047	2	4	3	0	3	3	3	0	0	1	18	1	3	2	5	3	2	0	1	2	8	1	2	6	4	3	0	2	0	3	3	5
ENSG00000207406	0	0	0	0	0	0	0	0	0	0	0	0	0	0	0	0	0	0	0	0	0	0	0	0	0	0	0	0	0	0	0	0
ENSG00000183671	0	0	0	0	0	0	0	0	0	0	0	0	1	1	1	0	0	0	0	0	0	0	0	0	0	0	0	0	0	0	0	0
ENSG00000279220	0	0	0	0	0	0	0	0	3	8	33	8	0	0	0	2	0	1	0	0	0	0	0	0	5	3	8	11	0	0	0	2
ENSG00000222496	0	0	0	0	0	0	0	0	0	0	0	0	0	0	0	0	0	0	0	0	0	0	0	0	0	0	0	0	0	0	0	0
ENSG00000159186	0	0	0	0	0	0	0	0	0	0	0	0	0	0	0	0	0	0	0	0	0	0	0	0	0	0	0	0	0	1	0	0
ENSG00000224070	0	0	0	0	0	0	0	0	0	0	0	0	0	0	0	0	0	0	0	0	0	0	0	0	0	0	0	0	0	0	0	0
ENSG00000252716	0	0	0	0	0	0	0	0	0	0	0	0	0	0	0	0	0	0	0	0	0	0	0	0	0	0	0	0	0	0	0	0
ENSG00000204186	32	89	37	73	0	0	0	0	626	589	3177	465	855	1111	940	1273	4	13	5	26	0	0	0	0	510	768	1043	1805	330	711	557	867
ENSG00000279921	0	0	0	0	0	0	0	0	4	0	3	0	0	0	0	0	0	0	0	0	0	0	1	0	0	0	0	3	0	0	0	1
ENSG00000180105	0	0	0	0	0	1	0	0	0	0	0	0	0	0	0	0	0	0	0	0	0	0	0	0	0	0	0	0	0	0	0	0
ENSG00000234491	0	0	0	0	0	1	0	0	0	1	1	0	0	0	0	0	0	0	0	1	0	0	0	0	0	0	0	0	0	0	0	0
ENSG00000114948	0	5	0	2	0	0	0	0	0	1	6	4	5	11	1	7	0	0	0	1	0	0	0	0	0	3	0	8	0	4	0	7
ENSG00000228577	0	0	0	1	0	0	1	0	0	0	0	0	0	0	1	0	0	3	0	0	0	0	0	0	0	0	0	0	0	0	0	0
ENSG00000231653	0	0	0	0	0	0	0	0	0	0	0	0	0	0	0	0	0	0	0	0	0	0	0	0	0	0	0	0	0	0	0	0
ENSG00000235118	0	0	0	0	0	0	0	0	0	0	0	0	0	0	0	0	0	0	0	0	0	0	0	0	0	0	0	0	0	0	0	0
ENSG00000232125	0	0	0	0	0	0	0	0	0	0	1	0	0	0	1	0	0	0	0	0	0	1	0	0	0	1	3	1	0	0	0	2
ENSG00000213083	0	0	1	0	0	0	0	0	0	0	0	0	0	0	0	0	0	0	0	0	0	0	0	0	0	0	0	0	0	0	0	0
ENSG00000207319	0	0	0	0	0	0	0	0	0	0	0	0	0	0	0	0	0	0	0	0	0	0	0	0	0	0	0	0	0	0	0	0
ENSG00000138400	38	29	34	23	5	14	6	10	49	26	72	15	1	10	18	9	59	54	78	58	14	6	9	11	8	15	8	28	9	10	14	24
ENSG00000118246	903	549	699	554	795	830	648	864	936	693	3143	533	1124	949	1111	1184	1508	1779	1380	1297	2308	909	1352	970	840	1179	655	1911	1296	1294	1393	1599
ENSG00000263468	0	0	0	0	0	0	0	0	0	0	0	0	0	0	0	0	0	0	0	0	0	0	0	0	0	0	0	0	0	0	0	0
ENSG00000283469	0	0	0	0	0	0	0	0	0	0	0	0	0	0	0	0	0	0	0	0	0	0	0	0	0	0	0	0	0	0	0	0
ENSG00000260171	0	0	0	0	0	0	2	0	2	1	5	1	1	1	0	4	0	1	1	1	1	4	3	0	1	2	0	9	1	1	0	0
ENSG00000229321	0	0	0	2	0	1	0	1	0	0	7	0	0	1	0	0	0	3	0	0	1	0	0	0	0	0	0	4	0	0	1	0
ENSG00000144410	1	2	1	0	0	0	0	0	4	0	7	1	3	0	2	4	0	3	4	5	0	1	0	1	4	1	4	3	0	0	5	1
ENSG00000118263	594	452	559	433	461	429	377	477	532	412	1756	312	1322	1405	1211	1643	167	197	236	379	847	342	570	330	376	544	505	1192	496	895	578	978
ENSG00000253008	2	0	0	0	0	2	1	0	0	0	3	1	9	0	0	3	0	0	2	0	3	4	0	0	0	0	1	1	2	1	3	1
ENSG00000237892	19	8	7	7	8	15	8	11	5	1	33	11	9	14	12	19	7	1	3	6	20	14	19	8	9	12	7	29	12	10	6	19
ENSG00000277590	0	2	0	0	0	3	0	0	1	2	3	1	0	0	0	1	0	0	0	1	1	5	5	0	0	0	0	4	0	0	3	3
ENSG00000224342	0	0	0	1	1	0	0	0	0	0	0	0	0	2	3	1	0	1	0	0	0	0	2	0	0	0	0	0	2	0	0	0
ENSG00000234902	2	3	0	4	3	0	0	1	1	0	0	1	9	1	3	8	2	0	0	1	0	0	0	0	0	0	0	1	1	4	1	2
ENSG00000225916	0	0	0	0	0	0	0	0	0	1	0	0	2	3	0	1	0	0	0	0	0	0	0	0	0	0	0	0	0	1	1	0

ENSG00000240440	1	2	0	1	0	1	1	0	0	0	0	0	3	1	1	1	0	1	0	0	1	0	0	0	0	0	0	0	0	0	0	2
ENSG00000229647	2	4	0	7	0	1	0	2	0	0	0	0	1	4	2	8	2	0	2	3	1	1	0	1	0	0	0	0	2	1	1	0
ENSG00000223725	7	5	2	8	2	3	2	4	1	0	1	0	9	7	3	16	2	3	0	3	2	1	1	1	0	0	3	3	2	7	6	10
ENSG00000225064	0	0	0	0	0	0	0	0	0	0	0	0	0	0	0	0	0	0	0	1	0	0	0	0	0	0	0	0	0	0	0	0
ENSG00000221628	0	0	0	0	0	0	0	0	0	0	0	0	0	0	0	0	0	0	0	0	0	0	0	0	0	0	0	0	0	0	0	0
ENSG00000118260	2137	1459	1598	1314	2346	2462	1798	2516	1987	1742	7042	1277	1477	1406	1550	1827	1028	1272	1226	1439	4658	1958	3191	1726	2166	3141	1981	5094	1173	1662	1413	1720
ENSG00000144401	962	682	768	677	651	680	527	689	508	370	1093	229	873	770	838	1031	501	685	612	716	1325	528	911	492	465	563	246	865	646	727	683	780
ENSG00000223433	0	0	0	0	0	0	0	0	0	0	0	0	0	0	0	0	0	0	0	0	0	0	0	0	0	0	0	0	0	0	0	0
ENSG00000224137	0	0	0	0	0	0	0	0	0	0	0	0	3	10	13	5	0	0	0	0	0	0	0	0	1	0	0	0	0	5	7	8
ENSG00000199251	0	0	0	0	0	0	0	0	0	0	0	0	0	0	0	0	0	0	0	0	0	0	0	0	0	0	0	0	0	0	0	0
ENSG00000213082	0	0	0	0	0	0	0	0	0	0	1	1	0	0	0	0	0	0	0	0	0	0	1	1	0	0	0	0	0	0	0	0
ENSG00000200764	0	0	0	0	0	0	0	0	0	0	0	0	0	0	0	0	0	0	0	0	0	0	0	0	0	0	0	0	0	0	0	0
ENSG00000163249	372	358	352	337	711	709	467	689	328	275	1618	228	901	834	818	1095	105	190	181	273	1299	545	899	527	449	672	371	1124	472	913	596	965
ENSG00000272851	0	0	0	0	0	0	0	0	0	0	0	0	0	0	0	0	0	0	0	0	0	0	0	0	0	0	0	0	0	0	0	0
ENSG00000163251	159	216	136	230	266	286	183	282	214	142	639	137	234	245	216	282	17	69	26	102	535	228	388	210	154	339	117	533	139	216	130	255
ENSG00000178385	493	445	422	481	250	266	204	298	326	263	1898	261	522	476	394	623	87	165	121	260	361	168	243	152	337	511	567	1267	254	512	328	534
ENSG00000225111	2	1	3	0	0	0	1	2	2	0	10	0	2	4	2	1	0	0	0	0	5	1	1	2	0	2	1	4	1	3	1	1
ENSG00000212246	1	1	3	0	1	0	0	0	0	0	0	0	0	0	0	0	0	0	0	0	0	1	0	0	0	2	0	3	0	0	0	1
ENSG00000185078	0	0	0	1	0	0	0	0	0	0	0	0	0	0	0	0	0	0	0	0	0	0	0	0	0	0	0	0	0	0	0	0
ENSG00000224839	0	0	0	0	0	0	0	0	0	0	1	0	0	0	0	0	0	0	0	0	0	0	0	0	0	1	0	0	0	0	0	0
ENSG00000229150	0	0	0	0	0	0	0	0	0	0	0	0	0	0	0	0	0	0	0	0	0	0	0	0	0	0	0	0	0	0	0	0
ENSG00000118231	0	0	0	0	0	0	0	0	0	0	1	0	0	0	0	0	0	0	0	0	0	0	0	0	0	0	0	0	0	0	0	0
ENSG00000163254	0	0	0	0	0	0	0	0	0	0	0	0	1	0	0	0	0	0	0	0	0	0	0	0	0	0	0	0	0	0	0	0
ENSG00000224529	0	0	0	0	0	0	0	0	0	0	0	0	0	0	0	0	0	0	0	0	0	0	0	0	0	0	0	0	0	0	0	0
ENSG00000182187	0	0	0	0	0	0	0	0	0	0	0	0	0	0	0	0	0	0	0	0	0	0	0	0	0	0	0	0	0	0	0	0
ENSG00000168582	0	0	0	0	0	0	0	0	0	0	0	0	0	0	0	0	0	0	0	0	0	0	0	0	0	0	0	0	0	0	0	0
ENSG00000188674	0	0	0	0	0	0	0	0	0	0	0	0	0	0	0	0	0	0	0	0	0	0	0	0	0	0	0	0	0	0	0	0
ENSG00000233869	0	0	0	0	0	0	0	0	0	0	0	0	0	0	0	0	0	0	0	0	0	0	0	0	0	0	0	0	1	0	0	0
ENSG00000228111	0	0	0	0	0	0	0	0	0	0	0	0	0	0	0	0	0	0	0	0	0	0	0	0	0	0	0	0	0	0	0	0
ENSG00000138413	1411	834	917	869	3436	3027	2211	3194	808	632	2914	478	956	941	837	1225	794	1034	744	757	6265	2202	3711	1947	764	1053	586	1731	847	955	917	1099
ENSG00000231908	27	15	18	15	10	11	7	16	2	8	15	4	12	9	2	15	10	15	12	8	31	18	10	14	3	6	1	14	8	13	9	16
ENSG00000115020	4912	3511	3957	3479	3782	4189	2733	4303	1995	1816	9275	1355	3669	3351	3643	4329	994	1279	1270	2501	5338	2635	3592	2505	2240	3268	2256	6208	1915	3220	2332	3616
ENSG00000228380	3	2	3	1	2	3	3	1	1	0	2	0	1	1	4	1	2	1	0	0	10	1	4	1	1	2	1	4	1	3	1	4
ENSG00000144407	2	0	0	1	1	0	0	0	0	0	1	0	9	5	6	15	0	0	0	0	2	1	1	0	0	0	1	2	2	5	3	7
ENSG00000213081	0	0	0	0	0	0	0	0	0	0	1	0	1	0	0	0	0	0	0	0	0	0	0	0	0	0	0	0	0	0	5	2
ENSG00000227430	0	0	0	0	0	0	0	0	0	0	0	0	0	0	0	0	0	0	0	0	0	0	1	0	0	0	0	0	0	0	0	0
ENSG00000231896	0	0	0	0	0	0	0	0	0	0	0	0	0	0	0	2	0	0	0	0	0	0	0	0	0	0	0	1	0	1	0	0
ENSG00000232409	0	0	0	0	0	0	0	0	0	0	0	0	0	0	0	0	0	0	0	0	0	0	0	0	0	0	0	0	0	0	1	0
ENSG00000235864	0	0	0	0	0	0	0	0	0	0	0	0	0	0	0	0	0	0	0	0	0	0	0	0	0	0	0	0	0	0	0	0
ENSG00000232799	0	0	0	0	0	0	0	0	0	0	0	0	0	0	0	0	0	0	0	0	0	0	0	0	0	0	0	0	0	0	0	0
ENSG00000240286	0	0	0	0	0	0	0	0	0	0	0	0	0	0	0	0	0	0	0	0	0	0	0	0	0	0	0	0	0	0	0	0
ENSG00000229670	0	1	0	0	0	0	0	0	0	0	0	0	0	0	0	0	0	0	0	0	0	0	0	0	0	0	0	0	0	0	0	0
ENSG00000078018	0	6	0	5	0	0	2	1	43	21	76	18	1	7	4	8	0	0	0	4	4	0	2	2	21	32	15	49	0	7	1	4
ENSG00000144406	0	2	0	1	1	0	0	2	3	3	10	1	0	2	2	5	0	0	0	1	2	0	3	2	2	0	4	2	0	2	1	0
ENSG00000237194	0	0	0	0	0	0	0	0	0	0	0	0	0	0	0	0	0	0	0	0	0	0	0	0	0	0	0	0	0	0	0	1
ENSG00000197713	1372	880	1094	900	1235	1346	1067	1484	1235	990	4081	759	1571	1404	1680	1886	899	1208	993	1049	2707	1136	1926	1095	1263	1803	780	2854	1394	1625	1767	1941
ENSG00000144445	667	489	547	497	513	527	344	601	501	452	1903	330	703	661	608	787	173	204	160	404	952	375	498	412	483	584	494	1380	334	667	427	719
ENSG00000272807	65	60	38	57	75	73	59	55	72	67	278	61	84	65	55	74	22	20	14	59	114	61	76	65	69	90	65	181	25	73	39	87
ENSG00000229127	61	45	67	57	74	60	42	83	94	128	456	55	104	111	88	134	22	20	17	91	154	92	94	87	83	119	111	328	40	113	83	144
ENSG00000231063	1	3	1	1	3	1	1	1	2	5	8	1	7	4	3	2	0	0	0	3	4	4	1	1	1	5	3	8	2	4	3	3
ENSG00000231294	0	3	3	3	0	1	0	0	0	0	3	0	0	3	2	1	2	0	2	4	0	1	0	0	0	0	1	1	0	2	1	2
ENSG00000115361	4	3	1	1	0	0	0	0	7	8	43	9	3	1	3	1	1	0	2	0	1	0	0	0	9	21	4	38	2	3	1	0
ENSG00000279317	1	0	1	0	0	0	0	0	0	0	0	0	0	0	1	1	0	0	0	0	0	0	0	0	0	0	0	0	0	0	0	0
ENSG00000199203	0	0	0	0	0	0	0	0	0	0	0	0	0	0	0	0	0	0	0	0	0	0	0	0	0	0	1	0	0	0	0	0
ENSG00000168530	0	0	0	0	0	0	0	0	0	0	0	0	0	0	0	0	0	0	0	0	0	0	1	0	0	0	0	0	0	0	0	0
ENSG00000234281	21	12	21	14	18	30	17	28	23	10	90	9	14	22	23	37	4	4	6	23	31	28	20	27	28	29	23	59	10	21	28	23
ENSG00000115365	2104	1558	1740	1554	2664	2666	1871	2772	2144	1835	8367	1228	2051	1952	1959	2369	916	1608	1243	1419	4821	1858	3759	1830	1966	2713	1962	5141	1174	1619	1560	1903
ENSG00000021826	209	153	199	158	49	32	44	60	126	85	355	58	282	273	261	338	65	103	80	107	68	26	62	17	85	93	33	177	119	211	185	260
ENSG00000280837	1	0	0	0	0	0	0	0	0	0	0	0	0	0	1	0	0	0	0	1	0	0	0	2	0	0	0	0	0	0	1	0
ENSG00000271074	0	0	0	0	0	0	0	0	0	0	0	0	0	0	0	0	0	0	0	0	0	0	0	0	0	0	0	0	0	0	0	0
ENSG00000231017	0	0	0	0	0	0	0	0	0	0	0	0	0	0	0	0	0	0	0	0	0	0	0	0	0	0	0	0	0	0	0	0
ENSG00000178568	0	0	0	0	11	5	1	4	2	1	10	0	4	3	1	1	0	1	0	1	9	1	14	4	0	0	0	0	3	1	2	2
ENSG00000232631	0	0	0	0	0	0	0	0	0	0	0	0	0	0	0	0	0	0	0	0	0	0	0	0	0	0	0	0	0	0	0	0
ENSG00000236007	0	0	0	0	0	0	0	0	0	0	0	0	0	0	0	0	0	0	0	0	0	0	1	0	0	0	0	0	0	0	0	0
ENSG00000221782	0	0	0	0	0	0	0	0	0	0	0	0	0	0	0	0	0	0	0	0	0	0	0	0	0	0	0	0	0	0	0	0
ENSG00000273118	22	14	25	15	28	28	22	30	11	5	35	1	37	20	13	27	8	8	5	20	43	34	20	38	16	19	13	43	8	31	8	27
ENSG00000234308	5	7	2	3	3	3	1	7	3	3	10	0	10	8	7	6	2	1	0	4	10	3	8	8	4	1	1	13	6	23	8	13
ENSG00000231254	1	0	3	3	4	1	3	1	3	3	7	5	6	10	4	5	1	0	1	1	4	4	5	2	2	3	3	4	4	12	2	9
ENSG00000223373	4	2	4	3	6	7	1	13	6	2	15	2	13	9	7	8	0	0	1	3	13	2	8	3	8	5	6	30	8	21	10	18
ENSG00000283637	0	0	0	0	0	0	0	0	0	0	0	0	0	0	0	0	0	0	0	0	0	0	0	0	0	0	0	0	0	0	0	0
ENSG00000266354	0	0	0	0	0	0	0	0	0	0	0	0	0	0	0	0	0	0	0	0	0	0	0	0	0	0	0	0	0	0	0	0
ENSG00000030419	9504	6957	8022	6205	7909	7970	5938	8701	4963	4678	20416	3514	11209	8794	7509	9910	2610	3661	2951	6458	14368	6613	10811	6571	5495	7629	5355	14835	5348	7579	5696	7746
ENSG00000270659	64	58	80	47	43	34	26	56	46	39	130	16	256	263	247	278	37	52	48	66	97	33	63	23	50	38	33	80	150	236	147	234
ENSG00000236193	1	0	0	0	0	0	0	0	0	0	1	0	5	9	3	5	0	0	1	0	0	0	0	0	0	0	0	0	3	4	3	5
ENSG00000272519	0	0	1	0	0	0	0	0	0	8	10	0	26	25	23	21	0	0	0	0	0	0	0	0	0	0	1	2	8	10	4	15
ENSG00000196096	1	1	2	5	0	0	0	0	14	3	48	3	143	96	81	111	2	1	8	2	0	0	0	0	9	4	24	23	21	47	21	63
ENSG00000144451	293	211	246	219	0	1	1	7	316	238	888	173	466	422	439	502	152	218	214	242	2	2	1	1	206	296	194	577	308	359	366	431
ENSG00000265734	0	0	0	0	0	0	0	0	0	0	0	0	0	0	0	0	0	0	0	0	0	0	0	0	0	0	0	0	0	0	0	0
ENSG00000197585	0	0	0	0	2	1	0	0	0	0	7	0	0	2	0	0	0	0	0	0	0	0	1	0	0	0	2	1	0	0	0	0
ENSG00000213917	0	0	0	0	0	0	0	0	0	0	0	0	0	0	1	0	0	0	0	0	1	0	0	0	0	0	0	0	0	0	0	0
ENSG00000174453	0	0	0	0	0	1	0	0	0	0	2	0	0	0	0	1	0	0	0	0	1	0	0	0	1	1	8	1	0	1	0	0
ENSG00000224257	0	0	0	0	0	0	0	0	0	0	1	0	0	0	0	0	0	0	0	0	0	0	0	0	0	0	1	2	0	0	0	0
ENSG00000177261	0	0	0	0	0	0	0	0	0	0	0	0	0	0	0	0	0	0	0	0	0	0	0	0	0	0	0	0	0	0	0	0
ENSG00000138376	1401	921	1125	936	1292	1291	963	1339	1367	1136	4166	895	1003	968	1031	1137	852	1199	1146	1196	3263	1237	2351	1210	1537	2055	732	3961	758	941	894	1094
ENSG00000229267	16	22	19	13	49	57	53	65	68	63	211	32	1293	1292	1356	1657	9	19	21	19	140	56	127	59	86	99	48	172	1082	1319	1392	1438
ENSG00000207274	0	0	1	0	0	1	3	1	3	3	5	2	0	0	0	0	0	0	2	1	4	5	2	1	0	0	0	5	0	0	0	0
ENSG00000230076	1	1	0	3	8	2	1	3	8	2	14	3	0	2	0	6	1	2	1	0	1	6	6	4	1	5	3	8	1	3	2	2
ENSG00000144452	15	18	21	8	57	41	38	38	42	28	832	57	1243	1369	1017	1442	11	9	7	19	99	51	56	39	56	85	142	279	467	1379	940	1368
ENSG00000227769	0	1	5	3	6	8	4	7	6	4	38	3	394	439	351	506	3	1	0	4	8	5	2	7	23	17	10	27	111	343	163	316
ENSG00000237571	0	1	0	0	0	0	0	0	0	0	0	0	0	0	0	2	0	0	0	0	3	0	0	1	0	0	0	0	0	1	0	0
ENSG00000138363	2640	1842	2182	1886	3450	3515	2864	3695	2788	2049	8843	1715	27894	24360	28422	30952	3776	4608	3823	3239	9817	3644	7501	3724	2949	4163	1771	5876	25928	26005	29180	30563
ENSG00000115414	113	66	121	71	53	48	37	51	1447	1266	6070	947	1884	1855	1707	2391	32	72	68	250	69	59	72	38	981	2140	1787	4266	649	1338	943	1549
ENSG00000230695	0	0	0	0	0	0	0	0	0	1	3	1	0	6	1	1	1	0	0	0	0	0	0	0	0	3	2	0	0	2	1	2
ENSG00000225166	0	0	0	0	0	0	0	0	0	0	0	0	0	0	0	0	0	0	0	0	0	0	0	0	0	0	1	0	0	1	0	0
ENSG00000228618	0	0	0	0	0	0	0	0	0	0	0	0	0	0	0	0	0	0	0	0	0	0	0	0	0	0	0	0	0	0	0	0
ENSG00000234938	0	0	0	0	0	0	0	0	0	0	2	0	0	0	0	0	0	0	0	0	0	0	0	0	0	0	0	0	0	3	0	1
ENSG00000237525	0	0	0	0	0	0	0	0	0	0	1	0	0	0	0	0	0	0	0	0	0	0	0	0	0	0	1	0	0	0	0	0
ENSG00000227981	0	0	0	0	0	0	0	0	0	0	0	0	0	0	0	0	0	0	0	0	0	0	0	0	0	0	0	0	0	0	0	0
ENSG00000235770	1	1	0	0	0	1	1	0	1	1	6	0	4	4	0	0	0	0	1	1	1	0	1	0	0	1	1	1	0	0	1	2
ENSG00000230838	0	0	0	0	0	0	0	0	0	0	0	0	0	0	0	0	0	0	0	0	3	0	0	0	0	0	0	0	0	0	0	0
ENSG00000216721	0	0	0	0	0	0	0	0	0	0	0	0	0	0	0	0	0	0	0	0	0	0	0	0	0	0	0	1	0	0	0	0
ENSG00000227824	0	0	0	0	0	0	0	0	0	0	0	0	0	0	0	0	0	0	0	0	0	0	0	0	0	0	0	0	0	0	0	0
ENSG00000226276	1	0	0	1	0	1	9	3	0	4	4	2	9	5	10	17	1	0	0	4	5	6	4	3	0	4	0	2	4	6	4	13
ENSG00000118242	389	269	359	252	749	798	608	793	273	200	858	197	2464	2046	2625	2559	318	482	502	387	2068	759	1610	581	242	386	164	566	2838	3019	3060	3018
ENSG00000115425	166	131	139	126	258	289	198	326	248	183	720	151	795	788	881	1040	143	198	188	127	487	187	269	165	182	275	171	469	742	818	665	935
ENSG00000163449	88	52	52	81	77	108	60	111	11	9	95	10	31	43	38	74	18	37	25	47	70	42	36	28	11	9	27	38	19	25	11	34
ENSG00000079246	9550	6792	7479	6403	12999	13073	10211	14253	10359	7641	30506	5738	54293	48972	50258	62021	10685	15313	12527	9569	32946	11744	24253	11850	10738	15595	6523	23721	55392	54653	60082	63136
ENSG00000232970	5	1	2	1	3	4	4	7	0	4	9	4	23	24	27	24	0	0	5	5	3	14	5	4	3	7	1	28	3	21	23	31
ENSG00000236478	6	8	10	5	17	13	12	23	10	11	42	6	44	63	51	71	2	3	3	16	40	20	15	20	22	26	11	57	20	79	88	105
ENSG00000279348	103	56	59	50	170	139	100	145	238	251	1031	192	712	743	747	950	27	33	25	60	266	112	167	95	334	500	185	768	496	750	530	837
ENSG00000260804	102	92	80	72	177	222	146	193	612	504	1936	305	1134	1155	1211	1519	23	53	57	107	334	160	188	118	550	796	432	1185	886	1314	787	1345
ENSG00000144583	0	0	0	0	6	6	9	6	0	0	0	0	0	2	0	2	0	0	0	0	7	2	8	4	2	0	0	0	0	0	2	0
ENSG00000231092	0	0	0	0	1	1	0	0	0	0	0	0	0	0	0	0	0	0	0	0	0	0	0	1	0	0	0	0	0	0	0	0
ENSG00000233581	0	0	0	0	0	0	0	1	0	0	0	0	0	0	0	2	0	0	1	0	0	0	0	0	0	0	0	1	0	3	0	0
ENSG00000207303	0	0	0	0	0	0	1	1	0	0	0	0	0	0	0	0	0	0	0	0	0	0	0	1	0	0	0	0	0	0	0	0
ENSG00000235042	0	0	1	0	0	0	2	0	0	0	0	0	6	3	14	9	1	2	0	0	1	0	1	0	0	0	1	1	4	7	9	3
ENSG00000138375	709	454	589	474	1864	1829	1418	1993	601	456	1986	353	3681	3207	3362	3875	283	351	346	425	2620	1001	2015	867	462	750	470	1308	2348	2632	2386	2711
ENSG00000232485	5	6	12	5	11	7	9	17	11	8	38	8	69	107	66	98	11	10	13	14	14	5	8	13	15	12	3	10	52	55	73	75
ENSG00000241520	4	1	5	0	3	7	5	3	1	4	6	1	15	25	13	26	0	1	0	0	0	3	1	1	3	3	2	4	9	12	11	10
ENSG00000197756	14275	9551	10162	10189	11195	11294	7940	12302	16703	11794	40682	9191	89557	87118	97065	113441	16624	20756	19454	15705	26408	10199	19308	11616	13592	19239	7076	24245	106323	90417	132611	122828
ENSG00000224391	0	1	0	3	0	0	1	0	1	2	6	1	5	3	2	6	0	1	1	0	2	1	0	0	0	1	2	0	2	4	2	2
ENSG00000225794	2	1	7	1	0	1	1	1	1	4	10	3	5	15	1	4	2	6	2	2	1	2	0	2	0	1	1	1	2	3	1	0
ENSG00000235444	0	0	1	0	1	0	0	0	0	0	0	0	0	0	2	0	1	0	0	1	0	0	0	0	0	0	0	0	0	0	0	0
ENSG00000115457	11247	9584	8742	9221	17578	15562	9995	17108	9161	6562	5330	3627	2756	5137	2362	5895	1102	2365	1365	1954	11462	4484	8433	3366	1082	1981	255	1939	593	1041	619	1211
ENSG00000115461	2	3	2	7	86	101	57	120	51	61	301	48	22	82	21	100	0	2	0	1	37	18	24	22	62	115	234	305	2	13	6	14
ENSG00000236886	0	0	0	0	1	0	0	0	1	0	1	0	0	0	0	0	0	0	0	0	1	0	0	0	0	1	0	2	0	0	0	0
ENSG00000237930	0	0	0	0	0	0	0	0	0	0	0	0	0	0	0	0	0	0	0	0	0	0	0	0	0	0	0	0	0	0	0	0
ENSG00000229352	0	0	0	0	0	0	0	1	0	0	2	0	0	0	0	0	0	0	0	0	0	0	0	0	0	0	1	1	0	0	0	0
ENSG00000234588	0	0	0	0	0	0	0	0	0	0	1	0	0	0	0	0	0	0	0	0	0	0	0	0	0	0	1	0	0	0	0	0
ENSG00000231597	0	0	0	0	0	0	0	0	0	0	0	0	0	0	0	0	0	0	0	0	0	0	0	0	0	0	0	0	0	0	0	0
ENSG00000118245	0	0	0	0	0	0	0	0	0	0	0	0	0	0	0	0	0	0	0	0	0	0	0	0	0	0	1	2	0	0	0	0
ENSG00000278951	0	0	0	0	0	0	0	0	0	0	0	0	0	0	0	0	0	0	0	0	0	0	0	0	0	0	0	0	0	0	0	0
ENSG00000237479	0	0	0	0	0	0	0	0	0	0	1	0	0	0	1	0	0	0	0	0	0	0	0	0	0	0	0	1	0	0	0	0
ENSG00000227021	0	0	0	0	0	0	0	0	0	1	1	0	0	0	0	0	0	0	0	0	0	0	0	0	0	1	0	3	0	0	0	0
ENSG00000236295	0	0	0	0	0	0	0	0	1	0	0	0	0	2	0	0	0	0	0	0	0	0	0	0	0	0	1	2	0	0	0	0
ENSG00000223874	0	0	0	0	0	0	0	0	0	0	0	0	0	0	0	0	0	0	0	0	0	0	0	0	0	0	0	0	0	0	0	0
ENSG00000259855	0	0	0	0	0	0	0	0	0	0	0	0	0	0	0	0	0	0	0	0	0	0	0	0	0	0	0	0	0	0	0	0
ENSG00000251982	0	0	0	0	2	3	2	6	5	8	20	3	1	0	0	0	0	0	0	0	5	0	3	7	2	5	3	15	0	0	0	0
ENSG00000233143	0	0	0	0	11	14	13	20	28	20	79	9	0	0	0	0	0	0	0	0	38	17	25	27	10	14	12	31	0	0	0	0
ENSG00000278004	0	0	0	0	0	0	0	0	1	0	0	0	0	0	0	0	0	0	0	0	1	1	0	0	0	0	0	0	0	0	0	0
ENSG00000231672	1	1	3	2	19	23	13	42	30	19	114	13	0	1	0	0	0	0	0	0	18	13	15	10	14	10	12	24	0	0	0	0
ENSG00000224414	0	0	1	0	1	5	3	5	0	0	0	1	0	0	0	0	0	0	0	0	0	0	0	0	0	0	0	0	0	0	0	0
ENSG00000231035	0	0	0	0	0	0	0	0	0	0	0	0	0	1	0	0	0	0	0	0	0	0	0	0	0	0	0	0	0	0	0	0
ENSG00000079308	2	4	0	14	380	352	281	368	11	18	55	11	1	5	3	13	0	0	0	3	495	166	303	211	3	10	1	4	0	5	0	2
ENSG00000275458	0	0	0	0	0	1	2	0	0	0	0	0	0	0	0	0	0	0	0	0	0	0	0	1	0	0	0	0	0	0	0	0
ENSG00000223923	0	0	0	0	0	0	0	0	0	0	0	0	0	0	0	0	0	3	0	0	0	1	2	0	0	0	2	0	0	0	2	0
ENSG00000188282	0	14	2	8	17	21	11	28	0	0	0	0	4	19	4	10	0	2	0	2	12	4	5	13	0	0	0	0	0	1	1	3
ENSG00000229754	1	0	0	0	101	67	82	106	0	0	1	0	0	0	0	3	0	0	0	0	207	68	130	64	0	0	0	0	0	0	0	1
ENSG00000180871	0	1	4	0	0	0	0	0	1	3	96	5	2	3	1	1	0	0	0	2	1	0	0	0	3	4	14	28	0	4	0	4
ENSG00000163464	1	0	0	1	4	5	1	4	1	0	1	0	0	2	3	0	1	2	0	0	3	1	4	0	0	1	1	0	2	1	1	2
ENSG00000224159	1	0	0	1	1	0	0	0	0	0	5	0	0	0	0	0	0	0	0	0	1	0	0	1	0	4	0	0	0	0	0	0
ENSG00000163466	7535	4527	6592	4391	8352	7978	6119	8849	8692	5993	29840	5144	15959	13263	16886	17684	5909	7650	8474	5000	18625	6963	13674	6719	7883	11301	6223	18695	15250	14196	18697	17016
ENSG00000261338	20	12	27	10	60	43	34	40	83	43	299	38	79	63	73	98	12	15	21	19	99	39	78	33	57	104	66	177	72	70	58	86
ENSG00000179921	4	10	15	5	25	21	12	19	4	11	22	3	14	8	6	14	0	2	0	15	33	19	14	15	5	12	8	13	11	11	9	8
ENSG00000127837	2481	1695	2048	1847	2478	2566	1891	2681	2322	1704	6559	1296	5370	4707	5311	6188	1342	2270	1960	1966	4643	1940	3647	1625	1883	2788	1401	4240	4998	4896	5476	5496
ENSG00000127838	415	296	382	283	584	588	448	664	410	345	1286	235	1688	1671	1632	2064	206	311	358	297	1056	407	913	405	355	572	301	802	1227	1293	1278	1533
ENSG00000135926	259	138	236	154	255	260	222	326	271	246	1006	175	935	957	920	1283	72	92	86	155	404	142	379	145	198	282	274	716	436	535	398	629
ENSG00000237281	0	1	0	0	0	1	0	0	0	0	6	3	9	3	14	4	0	0	1	0	1	0	0	0	0	1	2	2	1	5	0	12
ENSG00000278524	0	0	3	0	0	0	0	0	0	0	0	0	0	0	1	5	0	0	0	0	0	0	0	0	0	0	0	0	0	1	0	0
ENSG00000230580	0	0	0	0	0	0	0	0	0	0	0	0	0	0	0	1	0	0	0	0	0	0	0	0	0	0	0	0	0	0	0	0
ENSG00000158428	0	0	0	0	6	13	9	21	0	0	2	0	15	8	12	17	0	0	0	0	18	15	28	13	0	1	0	3	3	2	7	8
ENSG00000225062	0	0	0	0	14	11	12	22	1	0	1	0	0	4	4	4	0	0	0	0	18	16	13	15	0	0	0	0	0	1	2	0
ENSG00000018280	24	10	15	15	20	21	20	24	14	5	24	8	54	35	41	55	5	3	14	7	31	16	28	13	3	2	4	11	21	21	28	28
ENSG00000144579	1304	922	1428	933	2165	2200	1691	2430	1528	1247	3577	952	2345	2460	2337	3049	408	581	719	881	3130	1296	2246	1309	1097	1738	668	2743	1681	2168	1593	2342
ENSG00000273361	17	8	16	10	30	41	26	42	10	5	17	7	22	15	29	37	5	5	10	18	24	16	20	21	3	2	1	14	14	16	11	28
ENSG00000199121	0	0	0	0	1	0	0	2	0	0	4	2	2	1	0	2	0	0	0	2	3	0	0	0	0	3	1	8	0	1	1	1
ENSG00000127831	24	11	19	10	9	1	5	9	6	8	5	2	208	184	189	244	2	2	0	2	10	6	10	11	3	6	8	20	104	155	62	157
ENSG00000135913	1212	854	1003	799	1015	1148	838	1199	766	718	2330	556	1791	1588	1680	1810	441	493	548	757	2197	916	1507	806	737	1102	474	1951	947	1439	1285	1618
ENSG00000222714	3	2	8	2	1	2	2	6	3	6	9	1	2	5	5	5	0	1	3	6	8	9	6	0	2	1	1	8	2	3	0	2
ENSG00000144580	3064	2056	2626	2144	2957	2895	2325	3178	2950	2418	8889	1914	6261	5481	6408	7034	1390	1975	1778	2316	5834	2377	4263	2162	2967	4319	1771	6494	5113	6315	5565	6746
ENSG00000207393	4	5	0	1	2	0	1	1	1	0	9	1	2	0	1	6	2	0	1	4	2	1	0	3	3	0	0	5	0	1	0	1
ENSG00000115556	110	121	123	157	101	121	90	135	127	109	527	76	385	295	368	379	46	46	41	130	175	114	120	78	120	205	114	267	257	380	288	442
ENSG00000273466	12	15	14	21	16	24	5	26	20	24	74	13	64	41	61	80	7	8	12	18	40	15	19	14	23	32	18	50	38	75	53	109
ENSG00000115568	814	669	870	706	975	987	759	1049	920	814	3622	628	1835	1535	1931	1978	205	272	299	580	1610	738	1198	673	828	1436	762	2348	1248	1778	1356	1815
ENSG00000074582	570	411	575	463	929	935	724	876	964	711	3028	587	1735	1630	1898	2187	424	566	511	764	1728	791	1298	806	701	1191	668	1694	1211	1825	1385	1882
ENSG00000163481	294	186	270	176	325	386	299	388	268	241	1124	215	616	479	713	673	254	320	352	265	898	330	648	304	299	431	300	687	707	690	900	779
ENSG00000163482	341	237	340	260	429	583	381	477	287	250	1141	150	860	720	995	992	95	104	107	199	754	401	456	368	167	322	303	568	485	914	656	935
ENSG00000135912	1086	927	1005	929	1275	1369	1017	1550	777	700	2111	492	2829	2519	2764	3148	389	405	358	813	1947	827	1304	729	576	1062	329	1419	1712	2508	1863	2655
ENSG00000135929	14	6	4	2	1	0	0	1	1	1	4	1	8	17	13	13	1	2	3	5	0	0	0	1	1	3	1	6	4	13	13	18
ENSG00000272555	0	0	0	0	0	0	0	0	0	0	0	0	0	0	0	0	0	0	0	0	0	0	0	0	0	0	0	0	0	0	0	0
ENSG00000115592	0	0	0	0	0	0	0	0	0	0	0	0	0	2	0	0	0	0	0	1	1	0	0	0	0	0	0	0	0	0	0	0
ENSG00000283639	0	0	0	0	0	0	0	0	0	0	0	0	0	0	0	0	0	0	0	0	0	0	0	0	0	0	0	0	0	0	0	0
ENSG00000232625	0	0	0	0	0	0	0	0	0	0	0	0	0	0	0	0	0	0	0	0	0	0	0	0	0	0	0	0	0	0	1	0
ENSG00000115596	0	1	0	0	0	0	0	1	10	11	22	18	7	11	3	11	0	0	0	0	0	0	0	0	4	7	2	20	1	8	5	10
ENSG00000135925	8	16	16	12	19	19	14	17	51	65	26	29	455	734	568	1012	3	4	1	6	10	5	11	4	32	78	9	30	484	910	497	1043
ENSG00000228135	0	0	0	0	0	1	0	2	0	6	2	2	13	23	16	27	0	0	0	0	1	0	1	1	2	1	0	3	6	10	11	14
ENSG00000200029	0	0	0	0	0	0	0	0	0	2	0	1	7	5	2	3	0	0	0	0	0	0	0	0	0	0	0	2	3	0	2	6
ENSG00000235024	3	1	1	1	4	5	4	2	0	0	0	0	4	1	2	4	0	0	1	0	1	2	0	0	0	0	0	0	0	0	2	4
ENSG00000224928	4	9	5	11	11	7	7	5	0	0	0	0	5	6	3	9	5	0	1	4	3	3	2	2	0	0	0	0	4	1	1	2
ENSG00000171450	25	50	50	51	30	25	13	27	0	0	1	0	1	15	5	20	9	19	12	20	24	15	33	14	0	0	1	0	3	12	6	10
ENSG00000236445	0	0	0	0	0	0	0	1	0	0	3	0	0	0	0	0	2	0	0	0	0	0	0	0	0	0	1	0	0	0	2	0
ENSG00000163497	6	12	4	11	22	23	9	21	0	3	0	0	0	4	2	5	0	7	5	8	36	22	27	18	0	0	0	0	2	4	1	2
ENSG00000163499	0	0	0	0	0	0	1	4	0	0	0	0	0	2	0	0	0	0	0	0	3	3	0	0	0	0	0	0	0	0	0	0
ENSG00000198973	0	0	0	0	0	0	0	0	0	0	0	0	0	0	0	0	0	0	0	0	0	0	0	0	0	0	0	0	0	0	0	0
ENSG00000224090	0	0	0	0	0	0	0	0	0	0	0	0	0	0	0	0	0	0	0	0	2	0	0	0	1	0	0	0	0	0	0	0
ENSG00000181378	1	3	0	0	0	1	0	1	0	1	3	0	1	0	1	1	0	0	0	2	0	0	0	0	0	0	1	3	1	0	1	1
ENSG00000163501	0	0	1	1	0	1	0	0	0	1	6	0	2	1	0	0	1	0	0	4	1	5	2	2	2	0	1	2	0	1	0	1
ENSG00000264755	0	0	0	0	0	0	0	0	0	0	2	0	0	0	0	0	0	0	0	0	0	0	0	1	0	0	1	0	0	0	0	0
ENSG00000272644	8	10	9	6	19	13	9	10	13	17	45	9	15	21	14	26	6	8	6	10	17	11	6	8	14	21	9	23	17	20	7	21
ENSG00000187736	53	33	65	36	57	39	43	64	61	55	144	37	70	82	78	102	35	52	36	56	112	45	86	37	52	88	48	144	88	135	109	130
ENSG00000280537	26	20	23	33	18	20	36	17	11	5	38	2	27	26	24	31	12	9	7	35	30	19	23	16	14	16	9	29	13	52	20	33
ENSG00000240317	0	1	0	1	0	0	0	1	0	1	6	0	0	0	0	0	0	0	0	0	0	1	0	0	0	1	0	4	1	2	0	0
ENSG00000213901	42	27	54	28	28	49	27	44	21	5	69	7	38	33	42	53	14	16	13	44	65	32	30	24	18	17	11	37	17	72	23	82
ENSG00000115649	1238	903	1119	962	1511	1483	1140	1625	1283	1109	4810	895	1902	1874	2067	2431	454	687	770	1006	2622	1077	2184	1001	1284	2126	1161	3581	1967	2267	2118	2511
ENSG00000144567	1383	981	1180	896	1544	1541	1028	1697	1239	1149	4751	926	1823	1739	1564	2106	432	561	584	768	1923	901	1631	781	960	1599	1225	3282	1320	1503	1225	1687
ENSG00000158552	604	470	520	381	435	404	349	446	388	311	1478	279	477	420	417	580	452	655	662	480	887	331	665	315	390	591	458	1138	478	519	545	561
ENSG00000115657	138	134	158	147	57	74	45	76	36	57	249	28	58	55	98	65	31	30	37	99	40	30	32	42	47	47	64	142	16	53	37	67
ENSG00000198925	967	850	871	709	893	1056	689	1053	604	540	3115	504	890	794	836	1053	206	262	239	433	839	376	525	330	531	881	885	1745	443	599	443	700
ENSG00000163516	3592	2689	2618	2691	2525	2675	1795	3017	1479	1063	4457	748	3492	2858	2949	3648	843	648	464	959	1327	677	935	645	688	1174	1261	2370	1153	1617	1182	1926
ENSG00000163521	117	125	123	76	54	82	46	72	81	75	278	45	92	84	63	100	40	61	45	77	76	33	67	30	50	67	68	142	45	69	62	80
ENSG00000115661	476	333	382	330	403	405	238	419	464	398	1754	340	968	807	838	1045	218	294	363	286	600	265	400	254	398	520	393	984	688	737	708	850
ENSG00000127824	377	333	487	350	775	1122	535	1172	949	762	2179	667	912	938	1194	1288	109	199	328	302	1005	544	625	399	572	1111	297	1869	514	553	682	761
ENSG00000243910	0	1	1	1	2	1	0	1	4	1	1	3	1	2	0	3	0	0	2	0	3	0	1	1	1	1	3	1	1	0	1	4
ENSG00000135924	995	642	974	700	947	1148	775	1223	299	196	2555	264	131	139	195	226	355	471	524	730	1210	582	967	520	222	323	935	1462	78	100	97	126
ENSG00000054356	5	15	4	6	42	55	27	83	1	2	2	0	3	2	0	7	1	0	0	1	2	1	2	1	0	0	0	0	0	1	0	0
ENSG00000207647	0	0	0	0	0	0	0	0	0	0	0	0	0	0	0	0	0	0	0	0	0	0	0	0	0	0	0	0	0	0	0	0
ENSG00000230432	3	3	0	0	13	16	5	29	0	1	0	0	0	1	1	0	0	0	0	0	0	0	0	0	0	0	0	0	0	0	0	0
ENSG00000182698	0	1	0	0	0	0	2	1	0	2	26	3	0	0	0	0	0	0	0	0	5	0	1	5	5	1	12	15	0	0	0	0
ENSG00000232789	0	1	0	0	0	0	0	0	0	0	0	0	0	0	0	0	0	0	0	0	1	0	0	0	0	1	0	8	0	0	0	0
ENSG00000123992	871	623	659	710	1350	1545	1038	1592	1213	923	4296	865	3051	2909	2867	3751	342	519	452	666	2067	915	1589	766	1022	1637	866	2643	2160	3036	2438	3432
ENSG00000229525	1	1	3	1	7	1	3	3	0	3	2	0	6	4	4	10	0	2	0	4	3	4	5	0	0	0	1	1	2	8	1	6
ENSG00000175084	0	1	0	0	0	1	1	1	0	0	0	0	0	5	0	3	0	0	0	0	0	0	0	0	0	0	0	0	0	2	0	0
ENSG00000234638	0	0	0	0	0	0	0	0	0	0	0	0	0	0	0	0	0	0	0	0	0	0	0	0	0	0	0	0	0	0	0	0
ENSG00000280323	0	0	0	0	1	0	0	0	0	0	0	0	0	0	0	0	0	0	0	0	0	0	0	1	0	0	0	0	0	0	0	0
ENSG00000072195	454	597	611	579	793	939	577	996	88	110	283	77	33	89	25	69	57	124	122	315	900	476	627	429	20	66	55	151	17	40	10	41
ENSG00000227432	0	2	0	3	3	5	3	6	0	0	0	0	1	2	0	0	0	0	1	0	4	1	1	0	0	1	0	1	0	2	0	0
ENSG00000268603	12	9	7	5	7	3	1	2	1	0	5	0	3	1	3	3	0	4	3	2	2	2	1	1	1	1	1	2	1	4	0	5
ENSG00000144591	583	474	450	429	639	755	521	719	411	335	1397	302	939	892	1053	1201	310	464	332	463	980	450	763	432	322	510	322	857	761	920	807	1103
ENSG00000072182	2	5	4	8	15	6	12	13	1	5	7	0	3	7	3	9	1	3	0	7	17	8	8	2	2	2	1	1	0	4	4	5
ENSG00000123989	427	359	300	344	394	477	312	508	318	284	1620	300	1259	1139	1446	1705	86	243	126	245	572	284	506	246	212	394	389	807	1060	1173	1153	1384
ENSG00000188760	26	45	37	41	24	27	15	26	49	64	387	45	151	131	178	216	10	14	22	44	37	19	27	17	31	57	45	121	83	126	138	180
ENSG00000268896	1	4	2	0	0	3	1	3	1	1	1	1	0	5	1	0	0	0	0	0	4	1	0	1	0	0	1	1	1	1	3	0
ENSG00000265252	0	0	0	1	0	1	0	0	0	0	0	0	0	1	0	3	0	0	0	0	0	0	0	2	0	0	0	0	0	0	0	0
ENSG00000124006	5	12	2	12	1715	2077	1210	1872	399	383	1763	335	85	140	99	193	0	4	1	18	1676	875	1156	656	126	261	410	798	26	105	52	136
ENSG00000269068	0	0	0	2	3	5	5	8	0	1	8	0	3	2	7	6	0	0	0	0	5	4	1	11	0	0	2	1	0	0	1	2
ENSG00000123999	1	0	0	0	26	37	19	49	7	4	16	2	0	0	0	0	0	0	0	0	35	21	23	20	0	3	5	16	0	0	1	1
ENSG00000222678	0	0	0	0	0	0	0	0	0	0	0	0	0	0	0	0	0	0	0	0	0	0	0	0	0	0	0	0	0	0	0	0
ENSG00000144589	527	411	458	487	737	749	485	776	582	495	2052	396	919	858	909	1161	195	174	198	357	931	381	634	411	381	740	526	1353	507	811	569	915
ENSG00000228973	0	0	0	0	0	0	0	0	2	0	0	0	0	0	0	0	0	0	0	1	0	0	0	0	0	0	0	0	0	0	0	0
ENSG00000114923	2	9	2	11	2	3	0	3	0	2	4	0	0	2	2	0	0	0	2	2	0	0	0	0	0	0	1	1	0	1	1	0
ENSG00000267919	0	0	0	0	0	0	0	0	0	0	0	0	0	0	0	0	0	0	0	0	0	0	0	0	0	0	0	0	0	0	0	0
ENSG00000231802	0	0	0	0	0	0	0	0	0	0	1	0	0	0	1	0	0	0	0	0	0	0	0	0	0	0	0	0	0	0	0	0
ENSG00000227308	0	1	0	0	0	0	0	0	0	0	1	0	0	0	0	0	0	1	0	2	0	0	0	0	0	1	1	2	0	0	1	3
ENSG00000223903	0	0	0	0	0	0	0	0	0	0	1	1	0	0	0	0	0	0	0	0	1	0	0	0	0	0	2	0	0	0	0	2
ENSG00000228909	0	0	0	0	0	0	0	0	0	0	0	0	0	0	0	0	0	0	0	0	0	0	0	0	0	0	0	0	0	0	0	0
ENSG00000266518	0	0	0	0	0	0	0	0	0	0	0	0	0	0	0	0	0	0	0	0	0	0	0	0	0	0	0	0	0	0	0	0
ENSG00000225911	0	0	0	0	0	0	0	0	0	0	0	0	0	0	0	0	0	0	0	0	0	0	0	0	0	0	0	0	0	0	0	0
ENSG00000239498	0	0	0	0	0	0	0	0	0	0	0	0	0	0	0	0	0	0	0	0	0	0	0	0	0	0	0	0	0	0	1	0
ENSG00000235337	0	0	0	0	0	0	0	0	0	0	0	0	0	0	0	0	0	0	0	0	0	0	0	0	0	0	0	0	0	0	0	0
ENSG00000236451	0	0	0	0	0	0	0	0	0	0	0	0	0	0	0	0	0	0	0	0	0	0	0	0	0	0	0	0	0	0	0	0
ENSG00000224819	0	0	0	0	0	0	0	0	0	0	0	0	0	0	0	0	0	0	0	0	0	0	0	0	0	0	0	0	0	0	0	0
ENSG00000232089	0	0	0	0	0	0	0	0	0	0	0	0	0	0	0	0	0	0	0	0	0	0	0	0	0	0	0	0	0	0	0	0
ENSG00000116106	0	0	0	0	338	366	297	350	0	0	2	0	0	0	0	1	0	0	0	1	552	222	484	222	0	2	0	0	0	1	0	0
ENSG00000226726	0	0	0	0	3	4	0	2	0	0	0	0	0	0	0	0	0	0	0	0	1	0	0	3	0	0	0	0	0	0	0	0
ENSG00000272944	0	0	0	0	0	3	3	2	0	0	0	0	0	0	0	0	0	0	0	0	10	1	3	3	0	0	0	0	0	0	0	0
ENSG00000230406	0	0	0	0	0	0	0	0	0	0	0	0	0	0	0	0	0	0	0	0	0	0	0	0	0	0	0	0	0	0	0	0
ENSG00000234446	0	0	0	0	0	0	0	0	0	0	1	0	0	0	0	0	0	0	0	0	0	0	0	0	0	0	1	0	0	0	0	0
ENSG00000233868	1	0	0	1	1	1	1	0	1	1	3	0	0	0	1	1	2	1	1	3	2	0	3	0	2	0	2	0	0	2	2	1
ENSG00000226666	0	1	1	0	0	1	0	0	0	0	1	0	0	0	0	1	0	0	0	0	0	0	2	0	0	0	0	0	1	1	0	0
ENSG00000226331	0	0	0	0	0	0	0	0	0	0	0	0	0	0	0	0	0	0	0	0	0	0	0	0	0	0	0	0	0	0	0	0
ENSG00000135903	0	0	0	0	0	0	0	0	0	0	0	0	0	2	0	0	0	0	0	0	0	0	0	0	0	0	0	0	0	0	0	0
ENSG00000163081	0	0	0	0	0	0	0	0	0	0	0	0	0	0	0	0	0	0	0	0	0	0	0	0	0	0	0	0	0	1	0	0
ENSG00000267034	4	24	4	11	0	0	0	0	0	0	0	0	1	1	2	5	0	2	1	8	0	0	0	0	0	0	0	0	0	2	2	3

ENSG00000237732	8	22	10	24	0	0	0	0	1	1	0	0	6	12	12	21	0	2	0	3	0	0	0	0	0	0	1	0	2	7	0	9
ENSG00000200822	0	0	0	0	0	0	0	0	0	0	0	0	0	0	0	0	0	0	0	0	0	0	0	0	0	0	0	0	0	0	0	0
ENSG00000163082	11	12	10	7	33	31	42	51	113	81	407	61	94	102	133	160	16	19	16	50	110	74	95	64	71	100	67	194	61	168	76	182
ENSG00000228670	0	0	0	0	0	0	0	0	0	0	0	0	0	0	0	0	0	0	0	0	0	0	0	0	0	0	0	0	0	0	0	0
ENSG00000202016	0	0	0	0	0	0	0	0	0	0	0	0	0	0	0	0	0	0	0	0	0	0	0	0	0	0	0	0	0	0	0	0
ENSG00000234285	0	0	0	0	0	0	0	0	0	0	0	0	0	0	0	1	0	0	0	0	0	0	0	0	0	0	0	0	0	0	0	0
ENSG00000261428	38	21	34	22	50	48	52	53	136	97	417	71	138	158	150	182	22	14	19	72	158	73	95	56	64	170	70	209	61	139	87	169
ENSG00000116120	2247	1672	1826	1591	1928	1923	1566	2080	2103	1471	5662	1105	6203	5419	6121	6989	3029	4943	3634	2379	4615	1640	3786	1801	1901	2904	968	4036	5334	5054	5665	5724
ENSG00000124003	2	0	0	0	0	0	0	0	0	0	1	0	0	0	0	1	0	0	1	0	0	0	0	0	0	0	0	0	0	0	1	2
ENSG00000233751	0	1	0	0	0	0	0	0	0	0	0	0	0	0	0	0	0	0	0	0	0	0	0	0	0	0	0	0	0	0	0	0
ENSG00000203531	0	0	0	1	0	0	0	0	0	0	2	0	0	0	0	2	0	0	0	0	0	0	0	0	0	0	0	0	0	0	1	1
ENSG00000235225	0	0	0	0	0	0	0	0	0	0	0	0	0	1	1	0	0	0	0	0	0	0	0	0	0	0	0	0	1	2	1	0
ENSG00000123983	4095	3158	3088	2984	1749	1794	1312	1830	923	860	3643	705	2657	3146	2829	3911	1579	2544	1888	2666	3327	1260	2264	1179	1375	2108	842	3091	1894	2899	1893	3185
ENSG00000224121	4	3	1	5	0	1	1	7	0	0	5	2	2	5	6	8	2	1	1	9	4	3	2	1	1	1	0	8	0	2	2	5
ENSG00000234193	11	9	12	5	3	9	1	8	3	3	6	0	6	5	12	1	3	5	3	16	3	3	1	4	3	1	2	4	3	5	2	3
ENSG00000278083	0	0	2	3	1	3	2	3	0	0	1	0	0	4	0	2	0	0	0	0	2	3	2	1	1	4	2	2	0	2	2	1
ENSG00000152049	1	0	0	1	0	1	0	0	0	1	18	0	1	1	0	0	0	0	0	1	2	0	0	0	0	0	1	2	0	0	0	0
ENSG00000243125	0	0	0	0	0	0	0	0	0	0	0	0	0	0	2	0	0	0	0	0	0	0	1	0	0	0	0	0	0	0	0	0
ENSG00000233299	0	0	0	0	0	0	0	0	0	0	0	0	0	0	0	2	0	0	0	0	0	0	0	0	0	0	0	0	0	0	0	0
ENSG00000231189	1	0	0	1	0	0	0	0	0	0	0	0	0	0	0	0	0	0	0	0	0	0	0	0	0	0	0	0	1	0	0	0
ENSG00000235610	2	0	1	0	0	0	0	0	0	0	0	0	0	0	0	1	0	0	0	0	0	0	0	0	0	0	0	0	0	0	0	1
ENSG00000171951	253	153	293	153	13	29	26	25	9	5	44	5	1647	1831	3129	2691	139	71	203	84	33	9	23	15	37	37	60	39	2718	3338	6091	4179
ENSG00000226539	0	1	1	0	0	0	0	1	1	1	13	2	3	3	1	3	1	1	0	0	0	0	0	0	2	1	5	0	3	3	3	6
ENSG00000152056	217	207	186	222	42	26	27	37	200	142	1105	141	756	684	835	851	150	236	177	230	31	24	31	21	197	348	152	644	582	651	635	608
ENSG00000085449	1477	1225	1314	1258	1342	1633	1137	1641	1441	1233	7167	1090	1660	1482	1561	1986	446	849	737	951	2114	976	1603	821	1423	2113	1855	4998	1218	1740	1407	1873
ENSG00000135900	764	476	579	519	482	524	383	532	520	385	1867	331	694	514	652	736	602	781	683	606	1065	423	814	361	531	713	470	1219	663	738	703	793
ENSG00000228802	5	3	3	4	4	3	0	1	0	1	10	1	3	2	6	3	0	0	1	0	4	2	3	4	4	4	1	4	0	0	4	6
ENSG00000135919	61	210	76	160	1137	1454	739	1529	1081	1008	1907	563	1404	1465	1390	1974	5	66	11	70	1631	629	921	594	570	903	208	1069	585	865	691	930
ENSG00000224826	0	0	0	0	0	0	0	0	0	0	1	0	0	0	0	0	0	0	0	0	0	0	0	0	0	0	4	2	0	0	0	0
ENSG00000228681	0	0	0	0	0	0	0	0	0	0	0	0	0	0	0	0	0	0	0	0	0	0	0	0	0	0	0	0	0	0	0	0
ENSG00000124019	0	0	0	0	270	255	180	306	4	7	11	0	285	233	250	322	0	0	1	4	558	186	390	141	9	11	8	10	235	313	306	316
ENSG00000274629	9	7	1	6	5	7	1	2	3	3	23	2	7	7	13	5	4	7	1	10	2	5	4	2	5	1	4	7	9	10	7	9
ENSG00000036257	2657	1894	2286	1917	2030	2049	1572	2239	1787	1473	6924	1121	3068	2662	3516	3438	791	1388	1444	2000	3736	1390	2784	1506	1958	2707	1590	4452	2660	3045	2844	3551
ENSG00000228446	9	4	1	5	1	7	5	1	3	0	16	3	11	6	12	7	3	1	3	3	12	6	5	4	6	9	5	15	3	15	14	13
ENSG00000283428	0	0	0	0	1	0	0	0	0	0	0	0	0	0	0	0	0	0	0	0	0	0	0	0	0	0	0	0	0	0	0	0
ENSG00000135905	1875	1142	2041	1154	2108	2251	1399	2322	8580	7109	42606	5921	5145	4444	5647	5665	1143	978	1906	1349	3498	1431	2391	1245	7966	11245	10355	25483	3030	4561	4304	5345
ENSG00000263828	0	0	0	0	2	0	3	1	1	9	10	3	5	1	4	4	0	0	0	1	2	0	0	2	11	5	4	20	0	4	5	5
ENSG00000144460	0	0	0	0	16	15	6	14	0	0	4	0	0	0	0	0	0	0	0	1	27	16	10	13	0	0	1	0	0	0	0	0
ENSG00000228206	0	0	0	0	0	0	0	0	0	0	0	0	0	0	0	0	0	0	0	0	0	0	0	0	0	0	0	0	0	0	0	0
ENSG00000273301	0	0	0	0	0	1	0	0	0	0	0	0	0	0	0	0	0	0	0	1	0	0	0	0	0	0	0	0	0	0	0	0
ENSG00000279015	0	0	0	0	0	0	0	0	0	0	0	0	0	0	0	0	0	0	0	0	0	0	0	0	0	0	0	0	0	0	0	0
ENSG00000230621	0	0	0	0	0	0	0	0	0	0	0	0	0	0	0	0	0	0	0	0	0	0	0	0	0	0	0	0	0	0	0	0
ENSG00000235070	0	0	0	0	0	0	0	0	0	0	0	0	0	0	0	0	0	0	0	0	0	0	0	0	0	0	0	0	0	0	0	0
ENSG00000263363	0	0	0	0	0	0	0	0	0	0	0	0	0	0	0	0	0	0	0	0	0	0	0	0	0	0	0	0	0	0	0	0
ENSG00000169047	690	582	675	650	286	304	197	276	246	211	865	194	51	104	41	105	204	274	211	462	357	170	274	131	141	324	165	580	18	56	14	74
ENSG00000272622	1	7	6	4	0	1	2	0	1	0	5	0	2	1	3	0	3	5	1	4	0	1	1	0	0	0	1	2	2	0	0	0
ENSG00000261379	2	2	1	0	0	0	0	0	0	0	0	1	0	0	0	0	2	1	1	6	0	0	0	0	0	0	0	1	0	0	0	0
ENSG00000144468	1288	969	1045	928	255	255	200	272	612	561	1962	381	1063	1008	896	1244	306	427	315	553	415	187	313	148	528	764	416	1296	522	708	565	824
ENSG00000212391	0	3	2	1	1	0	0	0	0	0	5	1	0	2	1	1	0	1	0	3	2	0	2	1	0	1	0	2	0	2	1	3
ENSG00000081052	19	7	22	16	10	9	8	9	3	8	64	8	54	64	43	81	1	6	4	10	16	8	1	7	11	20	21	77	23	52	26	59
ENSG00000169031	26	23	20	19	1	6	3	10	21	16	63	15	64	81	60	87	3	2	6	12	9	5	6	2	15	17	14	66	9	43	34	58
ENSG00000236432	19	23	16	20	12	10	6	12	18	13	82	9	32	31	41	51	11	11	15	16	10	4	5	4	7	16	15	31	23	31	23	25
ENSG00000168958	1646	1233	1349	1196	906	815	640	961	1357	1093	4637	829	2139	2071	2134	2606	1445	1801	1654	1613	1731	739	1171	742	1105	1590	1233	2900	2039	2313	2358	2633
ENSG00000168955	0	1	2	0	2	2	1	2	2	2	3	0	11	8	7	6	2	3	0	1	2	0	1	1	1	1	1	2	3	6	9	7
ENSG00000173744	1962	1531	1788	1562	1246	1304	963	1317	2406	2072	10011	1561	3523	3184	4339	4035	1120	1527	1344	1799	2586	1085	1738	1053	2463	3809	2509	6592	3245	4223	4536	4813
ENSG00000042304	0	0	0	0	0	0	0	0	0	0	0	0	0	1	0	0	0	0	0	0	0	0	0	0	0	0	0	0	0	2	0	0
ENSG00000236116	0	0	0	0	0	0	0	0	0	0	0	0	0	0	0	0	0	0	0	0	0	0	0	0	0	0	0	0	0	0	0	0
ENSG00000250599	0	0	0	0	0	0	0	0	0	0	5	0	0	0	0	0	0	0	0	0	1	0	0	0	1	0	0	0	0	0	0	1
ENSG00000135917	0	0	0	0	0	0	0	0	0	0	3	2	0	0	0	0	0	0	0	0	0	0	0	0	0	0	1	1	0	0	0	1
ENSG00000226718	0	0	0	0	0	0	0	0	0	0	0	0	1	1	0	0	0	0	0	0	0	0	0	0	0	0	0	0	1	1	3	1
ENSG00000229172	0	0	0	0	0	0	0	0	0	0	4	0	2	0	2	3	0	0	0	0	0	0	0	0	0	0	1	1	0	0	5	0
ENSG00000115009	4	1	2	3	0	0	0	0	0	0	4	0	170	112	179	180	1	0	2	1	0	0	0	0	0	0	2	0	120	117	196	143
ENSG00000183514	0	0	0	0	0	0	0	0	0	0	0	0	0	0	0	0	0	0	0	0	0	0	0	0	0	0	0	0	0	0	0	0
ENSG00000123977	0	0	0	0	0	0	0	0	0	1	0	0	6	0	5	2	0	0	1	0	0	0	0	0	0	0	7	0	1	1	9	2
ENSG00000272237	0	0	0	0	0	0	0	0	0	0	0	0	0	0	0	0	0	0	0	0	0	0	0	0	0	0	0	0	0	0	0	0
ENSG00000153820	0	0	0	0	0	0	0	0	0	0	0	0	0	0	0	0	0	0	0	0	0	0	0	0	0	0	0	0	0	1	0	0
ENSG00000200281	0	0	0	0	0	0	0	0	0	0	0	0	0	0	0	0	0	0	0	0	0	0	0	0	0	0	0	0	0	0	0	0
ENSG00000203387	0	1	0	0	0	0	0	0	0	0	0	0	0	0	0	0	0	0	0	0	0	0	0	0	0	0	1	0	0	0	0	0
ENSG00000232023	0	0	0	0	0	0	0	0	0	0	0	0	0	0	0	0	0	0	0	0	0	0	0	0	0	0	0	0	0	0	0	0
ENSG00000228226	0	0	0	0	0	0	0	0	0	0	0	0	0	0	0	0	0	0	0	0	0	0	0	0	0	0	0	0	0	0	0	0
ENSG00000237322	0	0	0	0	0	0	0	0	0	0	0	0	0	0	0	0	0	0	0	0	0	0	0	0	0	0	0	0	0	0	0	0
ENSG00000153823	0	0	0	0	5	0	2	2	0	0	0	0	0	0	1	0	0	0	1	0	0	0	2	0	0	0	0	0	0	0	0	0
ENSG00000226297	0	0	0	0	0	0	0	0	0	0	0	0	0	0	0	0	0	0	0	0	0	0	0	0	0	0	0	0	0	0	0	0
ENSG00000253006	0	0	0	0	0	0	0	0	0	0	0	0	0	0	0	0	0	0	0	0	0	0	0	0	0	0	0	0	0	0	0	0
ENSG00000187957	0	0	0	0	0	0	0	0	0	0	1	0	1	3	0	2	0	0	0	0	0	0	0	0	0	0	0	0	0	3	0	1
ENSG00000238782	0	0	0	0	0	0	0	0	0	0	0	0	0	0	0	0	0	0	0	0	0	0	0	0	0	0	0	0	0	0	0	0
ENSG00000235993	0	0	0	0	0	0	0	0	0	0	0	0	0	0	0	0	0	0	0	0	0	0	0	0	0	0	0	0	0	0	0	0
ENSG00000153827	6918	4613	6215	4464	3843	4086	2949	4235	5076	4476	22679	3677	9493	8143	9704	10090	2236	2966	3596	4671	6893	2861	5041	2540	5471	8235	5689	15528	7300	9025	8479	10355
ENSG00000222344	2	2	0	1	0	0	0	0	1	0	6	0	1	0	2	2	0	0	0	0	0	0	0	1	1	2	1	9	0	3	3	3
ENSG00000153832	225	138	195	148	20	15	7	27	19	23	87	19	318	252	288	248	176	172	117	137	37	10	19	12	40	37	41	91	201	172	185	220
ENSG00000252333	0	1	3	2	0	0	0	0	1	0	2	0	0	4	1	5	0	0	0	0	1	0	0	0	0	0	0	1	1	0	1	3
ENSG00000231534	2	3	1	0	3	0	0	1	2	0	1	0	1	1	3	1	2	0	0	2	1	0	0	0	1	0	0	8	1	3	2	1
ENSG00000206725	0	0	0	0	0	0	0	0	0	0	0	0	0	0	0	0	0	0	0	0	0	0	0	0	0	0	0	0	0	0	0	0
ENSG00000163053	627	591	482	575	267	343	217	362	3	3	14	1	13	7	8	27	330	490	329	491	368	172	273	164	3	0	3	13	3	8	6	15
ENSG00000199400	4	6	13	4	7	6	1	8	0	0	0	0	0	0	0	0	0	3	7	4	5	1	5	4	0	0	0	0	0	0	0	0
ENSG00000225963	62	54	72	31	21	20	22	16	105	99	906	92	110	123	129	168	10	19	15	43	23	11	13	11	72	82	152	411	75	136	61	164
ENSG00000283451	0	0	0	0	0	0	0	0	0	0	0	0	0	0	0	0	0	0	0	0	0	0	0	0	0	0	0	0	0	0	0	0
ENSG00000135899	377	265	321	229	131	122	94	139	601	462	4701	522	812	735	909	924	111	120	130	221	169	64	120	73	454	689	893	2248	542	865	707	1028
ENSG00000280755	0	0	0	0	0	0	0	0	0	0	0	0	0	0	0	0	0	0	0	0	0	0	0	0	0	0	0	0	0	0	0	0
ENSG00000079263	8	19	17	18	6	8	3	3	1256	856	5399	828	2367	2266	2455	2853	7	10	1	11	2	11	8	1	896	1182	853	2767	1693	2048	2017	2368
ENSG00000185404	1051	672	953	725	347	343	298	417	977	796	4365	637	952	1009	1051	1243	382	430	549	618	592	285	446	242	754	1160	1081	2347	735	1387	1038	1504
ENSG00000279964	1	0	1	1	1	3	0	2	1	2	5	3	5	0	5	3	0	0	0	1	5	1	5	0	0	1	0	6	3	0	3	1
ENSG00000067066	1043	608	888	616	421	432	319	524	1439	1275	6233	966	1356	1288	1518	1671	556	613	627	773	814	330	600	351	1336	1856	1362	3711	1019	1458	1401	1696
ENSG00000243650	7	5	8	6	6	12	3	6	24	28	81	15	7	9	6	11	4	7	4	15	10	6	5	10	22	22	24	59	3	10	8	19
ENSG00000250011	5	2	3	3	0	1	0	1	7	6	61	8	1	5	4	9	1	1	5	3	4	2	0	3	7	12	10	19	0	2	2	4
ENSG00000235419	1	1	4	1	2	1	0	0	12	14	42	10	3	4	4	3	1	3	2	6	3	0	0	1	9	12	6	28	4	8	6	8
ENSG00000183022	1	1	0	0	3	0	1	2	1	0	1	0	0	1	0	0	0	1	2	0	2	2	1	1	0	0	0	1	0	1	0	0
ENSG00000283202	0	0	0	0	0	0	0	0	0	0	0	0	0	0	0	0	0	0	0	0	0	0	0	0	0	0	0	0	0	0	0	0
ENSG00000199791	0	0	0	0	0	0	0	0	0	0	0	0	0	0	0	0	0	0	0	0	0	0	0	0	0	0	0	0	0	0	0	0
ENSG00000226125	3	3	2	2	1	0	0	1	0	0	5	0	2	2	0	1	0	0	0	2	0	0	0	2	0	0	0	1	0	0	0	0
ENSG00000135932	3274	2272	2895	2165	1530	1486	1085	1543	2586	2129	13558	1941	3934	3373	4483	4310	1318	2086	2201	2353	2872	1253	2106	1022	3246	4517	3567	8993	3912	4215	4741	4844
ENSG00000237758	7	5	19	11	4	3	8	5	9	6	32	4	8	9	8	11	7	5	4	6	17	3	6	2	7	11	23	32	4	10	12	14
ENSG00000201044	3	2	2	0	0	0	0	4	2	2	9	0	0	0	0	2	0	2	0	1	4	2	0	2	1	0	2	7	3	2	0	1
ENSG00000270891	0	0	0	0	0	0	0	0	0	0	0	0	0	0	0	0	0	0	0	0	0	0	0	0	0	0	0	0	0	0	0	0
ENSG00000135916	4685	3646	4688	3751	4930	5265	3473	5606	132	319	245	160	2756	2938	3209	4001	1777	2993	3604	3203	7302	3139	5845	2520	83	311	79	455	2406	3123	3181	3530
ENSG00000232520	1	6	6	4	0	0	2	1	0	0	0	0	12	3	3	5	0	0	5	4	1	0	0	0	0	0	0	0	2	0	0	0
ENSG00000135898	17	1	15	12	0	4	0	1	0	1	0	0	250	208	270	264	13	5	24	17	1	0	3	0	0	0	0	0	80	120	152	194
ENSG00000230385	0	0	0	0	0	0	0	0	0	0	0	0	0	0	0	0	0	0	0	0	0	0	0	0	0	0	0	0	0	0	0	0
ENSG00000283164	0	0	0	0	0	0	0	0	0	0	0	0	4	4	2	1	0	0	0	0	0	0	0	0	0	0	0	0	1	1	1	0
ENSG00000235013	0	0	0	0	0	0	0	1	0	0	0	0	4	0	3	5	0	0	2	0	0	0	0	0	0	0	0	0	0	1	1	4
ENSG00000238062	9	2	13	3	0	0	0	0	0	0	0	0	45	27	63	38	5	2	10	1	0	0	1	0	0	0	0	0	16	36	35	41
ENSG00000173699	0	0	0	0	0	0	0	0	0	0	0	0	6	3	2	2	0	0	0	1	0	0	0	0	0	0	0	0	0	0	1	2
ENSG00000204128	0	0	1	1	0	0	0	0	0	1	0	0	5	4	1	11	0	0	0	0	0	0	0	0	0	0	2	0	1	1	0	0
ENSG00000261829	1	1	0	1	0	0	0	0	0	0	0	0	0	0	2	0	0	0	0	0	0	0	0	0	0	0	0	0	0	0	0	0
ENSG00000173692	4995	3701	3963	3788	2061	2029	1472	2079	2248	1747	8094	1476	3377	3342	3315	4140	4653	7466	5765	4840	5293	2104	3775	1665	2809	4162	1778	6249	4270	4329	4413	5022
ENSG00000135914	30	20	27	18	6	9	14	10	4	8	31	11	204	169	241	190	13	5	9	30	38	23	24	21	16	18	7	49	383	309	438	329
ENSG00000201574	2	2	1	1	0	0	0	2	0	1	3	2	2	0	0	2	0	1	1	2	3	1	0	0	1	1	1	6	0	3	2	1
ENSG00000135931	438	320	392	307	84	78	62	92	146	127	574	95	384	290	452	442	172	251	238	321	137	80	119	51	122	163	127	380	255	344	355	369
ENSG00000237039	2	0	3	2	4	3	2	0	6	1	11	1	0	1	3	6	4	1	4	6	3	1	3	0	5	0	2	3	4	1	3	5
ENSG00000263641	1	0	0	0	0	0	0	0	0	0	2	1	0	0	0	0	1	1	0	1	0	1	2	0	0	1	1	0	0	0	3	1
ENSG00000224376	8	10	10	1	57	35	33	42	98	85	330	64	28	18	18	21	1	2	0	0	70	22	34	37	72	59	59	108	12	13	16	25
ENSG00000156966	4	9	3	7	4	10	9	9	66	55	338	40	6	14	4	10	0	1	0	0	14	7	11	5	67	93	61	171	1	1	2	3
ENSG00000172799	0	1	6	0	0	0	1	0	0	3	3	2	1	1	0	3	0	1	1	0	0	1	1	0	1	1	0	0	1	0	2	2
ENSG00000230047	0	1	0	0	0	0	0	0	1	1	1	0	2	0	3	0	0	0	0	0	1	0	0	1	0	2	0	0	0	3	2	2
ENSG00000233538	12	10	9	13	14	26	24	21	23	22	56	11	39	42	52	55	3	4	1	18	28	13	7	14	21	24	21	51	17	43	38	47
ENSG00000115053	42929	30270	36341	33624	19933	19466	17292	21460	37121	26360	90156	19554	77726	70890	76074	88566	32054	41440	35531	44578	42452	17883	32751	15556	31584	48842	16110	65334	73489	78583	76237	87964
ENSG00000206885	14	8	21	7	12	7	7	13	22	11	74	5	20	17	26	21	12	15	8	22	9	14	9	9	22	22	10	46	12	49	8	32
ENSG00000207280	21	8	16	19	13	13	11	11	23	24	67	6	16	17	28	14	13	5	2	19	12	5	10	15	19	23	14	44	11	21	18	27
ENSG00000202400	4	1	3	10	2	1	0	2	6	1	12	1	7	3	10	7	0	3	4	6	6	0	3	9	1	6	2	10	4	5	7	9
ENSG00000283312	78	65	74	56	23	21	14	28	45	41	36	12	58	58	62	57	18	18	18	37	17	15	13	10	6	5	0	16	17	28	22	26
ENSG00000283491	99	62	85	84	25	24	22	30	42	38	48	16	59	68	59	91	19	35	33	56	22	11	20	13	7	12	4	19	17	49	23	31
ENSG00000223198	19	15	26	18	4	12	5	7	10	15	11	8	26	15	16	18	3	6	3	13	5	0	3	3	0	4	0	8	3	2	9	3
ENSG00000181798	96	106	81	126	44	37	33	33	58	37	71	22	86	64	77	112	34	64	34	84	44	16	46	16	10	10	4	22	38	42	35	56
ENSG00000171596	1	1	2	0	0	2	0	0	2	2	0	0	2	0	1	3	0	0	0	0	0	0	0	0	0	0	0	0	1	0	0	0
ENSG00000231494	0	1	0	0	19	23	8	18	16	7	5	8	7	5	1	3	0	0	0	0	4	2	4	4	0	1	0	0	1	1	2	1
ENSG00000228884	1	1	1	0	0	1	0	0	0	0	0	0	0	0	1	1	0	0	0	0	0	0	0	0	0	0	0	0	0	0	0	0
ENSG00000177673	0	1	1	0	0	0	0	0	0	0	0	0	1	2	0	0	2	0	0	0	0	0	0	0	0	0	0	0	0	0	0	0
ENSG00000239202	0	0	1	0	0	0	0	0	0	0	0	0	0	0	0	1	0	2	0	0	0	0	0	0	0	0	0	1	0	0	0	3
ENSG00000187514	89720	59235	70400	70495	40293	39506	28528	42165	70109	47474	162635	35454	110607	97744	90277	113515	50324	57495	50565	53603	61797	24463	39875	20791	57888	81552	28514	106291	83652	87863	74042	97613
ENSG00000277986	0	0	0	0	0	0	0	0	0	0	0	0	0	0	0	0	0	0	0	0	0	0	0	0	0	0	0	0	0	0	0	0
ENSG00000156973	1075	735	806	748	236	263	170	261	404	312	1314	288	764	791	846	1000	760	1134	976	809	430	198	341	164	409	638	310	923	753	728	835	990
ENSG00000281072	8	6	9	4	1	3	1	0	1	3	18	1	2	4	1	6	1	5	2	2	1	2	0	1	1	3	3	8	0	5	1	7
ENSG00000144524	2331	1603	2242	1757	815	772	602	836	1048	853	3506	603	1464	1343	1540	1753	789	1076	1079	1458	1265	538	937	565	853	1493	709	2125	1080	1339	1295	1617
ENSG00000237641	29	22	25	25	17	11	9	13	15	16	54	4	16	17	8	21	6	4	9	28	17	11	7	9	7	18	11	30	6	27	8	22
ENSG00000261096	34	24	15	21	13	23	19	26	19	12	79	11	12	19	26	15	6	3	5	28	21	13	18	15	24	30	15	62	7	20	5	28
ENSG00000260622	0	0	0	0	0	0	0	0	0	0	0	0	0	0	0	0	0	0	0	0	0	0	0	0	0	0	0	0	0	0	0	0
ENSG00000222246	0	0	0	0	0	0	0	0	0	0	0	0	0	0	0	0	0	0	0	0	0	0	0	0	0	0	0	0	0	0	0	0
ENSG00000163273	35	43	17	34	0	0	0	0	38	29	1215	120	8	10	16	12	1	4	0	3	0	0	0	0	63	74	119	644	8	8	7	7
ENSG00000144535	756	576	634	628	336	394	259	448	719	672	3492	541	887	835	819	1011	214	223	204	499	490	288	355	224	600	912	645	2260	501	866	517	914
ENSG00000227033	0	2	1	1	0	0	0	0	0	1	6	0	3	1	2	1	2	1	0	5	0	2	0	1	2	4	1	4	0	4	2	2
ENSG00000207626	2	0	3	0	0	3	1	1	0	1	2	0	2	1	1	0	0	0	1	1	1	0	0	1	2	1	1	5	1	1	0	0
ENSG00000251485	6	3	7	4	12	9	10	26	21	28	152	25	11	25	33	32	2	4	7	29	28	10	12	14	9	35	32	79	14	46	13	48
ENSG00000230122	0	0	1	0	0	4	3	4	2	1	3	0	0	0	3	0	0	0	0	0	2	3	4	6	1	1	1	5	0	1	1	2
ENSG00000275157	0	0	0	0	0	0	0	0	0	0	0	0	0	0	0	0	0	0	0	0	0	0	0	0	0	0	0	0	0	0	0	0
ENSG00000163283	0	0	0	0	0	0	0	0	0	0	1	0	0	0	3	2	0	0	0	0	0	0	0	0	0	0	0	0	0	0	1	0
ENSG00000224516	0	0	0	0	0	0	0	0	0	0	0	0	0	0	0	0	0	0	0	0	0	0	0	0	0	0	0	0	0	0	0	0
ENSG00000244280	0	0	0	0	0	0	0	0	0	0	0	0	0	0	0	0	0	0	0	0	0	0	0	0	0	0	0	0	0	0	0	0
ENSG00000163286	0	0	0	0	0	0	0	0	0	0	0	0	0	0	0	0	0	0	0	1	0	0	0	0	0	0	0	0	0	0	0	0
ENSG00000204121	1	2	0	3	0	0	0	0	0	0	1	0	0	0	0	0	0	0	0	1	0	0	0	0	0	0	0	0	0	0	0	0
ENSG00000237087	0	0	0	0	0	0	0	0	0	0	0	0	0	0	0	0	0	0	0	0	0	0	0	0	0	0	0	0	0	0	0	0
ENSG00000223788	0	0	0	0	0	0	0	0	0	0	0	0	0	0	0	0	0	0	0	0	0	0	0	0	0	0	0	0	0	0	0	0
ENSG00000163295	2	0	0	0	0	0	0	0	0	0	0	0	0	0	0	0	0	0	0	0	0	0	0	1	0	0	0	0	0	0	0	0
ENSG00000171551	1	0	0	2	31	55	11	38	0	4	1	1	0	1	0	3	0	0	0	0	24	15	16	11	0	2	1	3	0	0	0	0
ENSG00000237412	0	3	0	0	0	0	0	0	1	0	0	0	0	0	5	0	0	0	0	1	0	0	0	0	0	0	1	1	0	0	1	0
ENSG00000135902	1	3	0	5	3	3	1	4	3	3	18	1	14	23	9	15	0	0	2	5	3	4	5	4	4	2	3	16	2	7	3	8
ENSG00000196811	46	19	25	26	16	18	17	24	20	24	90	10	78	69	51	83	15	6	13	34	24	22	16	18	16	25	18	74	20	54	57	62
ENSG00000221944	454	350	384	294	83	51	43	74	163	140	623	113	355	339	331	386	283	227	240	309	144	58	68	45	159	229	158	401	247	278	217	360
ENSG00000135930	1651	1145	1198	1274	1077	1073	722	1147	1871	1359	7042	1153	2075	1990	2161	2604	1119	1783	1396	1443	1877	869	1403	763	1562	2432	1578	4207	2171	2410	2285	2699
ENSG00000237126	1	10	14	3	9	4	4	5	17	15	111	19	21	19	16	24	3	7	7	15	8	5	8	4	17	36	46	54	20	24	15	18
ENSG00000115468	9	19	12	12	1	1	0	1	718	651	2340	453	21	21	21	41	1	6	6	6	0	1	0	0	654	1061	905	1900	6	20	10	27
ENSG00000244384	0	0	0	0	0	2	0	0	0	0	4	1	0	0	0	0	0	0	1	0	0	0	0	0	5	3	6	8	0	0	0	0
ENSG00000204120	3610	2618	2931	2703	1301	1346	956	1522	2225	1868	9052	1487	3019	2933	2988	3717	856	1160	1027	1979	2025	910	1433	789	2110	3309	2451	6538	1961	2824	2169	3007
ENSG00000213055	10	8	4	2	3	2	1	3	3	5	15	2	0	4	4	7	5	0	3	3	5	0	2	2	8	4	1	17	3	6	5	3
ENSG00000241409	2	2	1	4	2	2	3	4	6	6	11	1	1	6	7	6	0	0	1	5	0	4	5	3	10	7	6	16	0	6	5	3
ENSG00000115474	27	25	29	21	7	13	10	14	33	13	68	6	28	25	24	25	5	9	3	28	15	9	13	19	32	34	34	107	17	77	41	73
ENSG00000252452	1	1	7	1	0	0	1	4	0	2	5	1	2	1	1	0	1	0	0	1	4	0	1	0	0	9	3	3	0	2	0	5
ENSG00000199781	0	3	0	2	0	1	0	0	0	0	3	0	4	1	0	1	0	0	0	2	0	0	0	0	2	1	0	4	1	2	1	3
ENSG00000182600	38	27	28	26	17	12	12	16	3	9	51	10	69	54	59	94	10	9	8	23	23	9	14	7	5	7	17	23	21	28	31	46
ENSG00000066248	0	1	0	3	5	1	0	2	3	3	11	2	20	32	25	29	0	0	0	2	2	1	0	4	2	4	3	5	4	13	15	14
ENSG00000222001	0	0	0	0	0	0	0	0	0	0	0	0	0	0	0	0	0	0	0	0	0	0	0	0	0	0	0	0	0	0	0	0
ENSG00000115488	0	0	0	0	0	0	0	0	0	0	0	0	0	0	0	0	0	0	0	0	0	0	0	0	0	0	0	0	0	0	0	0
ENSG00000168918	953	858	882	840	3809	4092	2948	4311	2836	2292	11747	1909	4227	3615	3702	4524	212	239	248	688	3680	1710	2917	1483	2243	3739	2594	7104	2429	3108	2351	3146
ENSG00000263941	3	3	2	3	9	3	0	1	2	3	17	1	1	3	1	0	2	1	0	3	1	2	0	1	5	5	5	7	0	1	0	3
ENSG00000085978	914	580	740	596	460	408	374	489	420	400	1872	291	795	630	779	757	390	376	486	722	881	361	568	392	438	677	469	1320	634	903	743	948
ENSG00000252010	8	8	8	3	4	1	4	2	2	2	13	4	5	7	9	11	6	2	1	8	9	3	3	11	7	6	4	21	12	11	6	4
ENSG00000251791	7	13	14	2	2	5	5	4	7	10	13	4	5	3	5	3	1	3	5	20	8	8	2	7	2	8	1	16	2	12	15	13
ENSG00000130561	0	2	6	6	2	8	7	12	0	0	1	0	4	1	5	4	1	0	2	7	14	10	9	5	1	0	2	1	0	5	2	1
ENSG00000259793	1	4	1	0	0	1	0	3	0	0	0	0	2	0	1	1	2	0	0	0	0	1	1	1	0	0	0	0	0	0	0	0
ENSG00000077044	2846	2734	2513	2814	3188	3593	2554	3997	925	820	3361	587	1578	1488	1451	2026	496	708	615	1716	4110	2180	2844	1980	789	1296	921	2386	765	1310	854	1391
ENSG00000201013	1	1	0	0	0	1	1	2	0	4	2	2	1	0	0	1	0	1	0	1	2	3	0	0	3	0	0	6	1	1	0	5
ENSG00000085982	536	415	465	424	5	8	8	8	631	541	2403	395	472	470	495	593	141	181	216	445	9	7	6	4	394	585	530	1334	294	517	378	570
ENSG00000271639	3	2	4	6	0	0	0	0	2	6	8	3	1	2	3	4	0	3	0	3	0	0	0	0	1	10	8	23	0	1	2	7
ENSG00000228949	0	0	0	0	0	0	0	0	0	0	0	0	0	0	0	0	0	0	0	0	0	0	0	0	0	0	0	0	0	0	0	0
ENSG00000227846	0	0	0	0	0	0	0	0	0	0	0	0	0	0	0	0	0	0	0	0	0	0	0	0	0	0	0	0	0	0	0	0
ENSG00000242366	0	0	0	0	0	0	0	0	0	0	0	0	0	0	0	0	0	0	0	0	0	0	0	0	0	0	1	0	0	0	0	0
ENSG00000242515	0	0	0	0	0	0	0	0	0	0	0	0	0	0	0	0	0	0	0	0	0	0	0	0	0	1	0	0	0	0	1	0
ENSG00000234143	0	0	0	0	0	0	0	0	0	0	0	0	0	0	0	0	0	0	0	0	0	0	0	0	0	0	0	0	0	0	0	0
ENSG00000241119	0	0	0	0	0	0	0	0	0	0	0	0	0	0	0	0	0	0	0	0	0	0	0	0	0	0	0	0	0	0	0	0
ENSG00000244122	0	0	0	0	0	0	0	0	0	0	0	0	0	0	0	0	0	0	0	0	0	0	0	0	0	0	0	0	0	0	0	0
ENSG00000167165	0	0	0	0	0	0	0	0	0	0	1	0	0	0	0	0	0	0	0	0	0	0	0	0	0	1	0	0	3	0	0	0
ENSG00000224814	0	0	0	0	0	0	0	0	0	0	0	0	0	0	0	0	0	0	0	0	0	0	0	0	0	0	0	0	0	0	0	0
ENSG00000240224	0	0	0	0	0	0	0	0	0	0	0	0	0	0	0	0	0	0	0	0	0	0	0	0	0	0	0	0	0	0	0	0
ENSG00000244474	0	0	0	0	0	0	0	0	0	0	0	0	0	0	0	0	0	0	0	0	0	0	0	0	0	0	0	0	0	0	0	0
ENSG00000233445	0	0	0	0	0	0	0	0	0	0	0	0	0	0	0	0	0	0	0	0	0	0	0	0	0	0	0	0	0	0	0	0
ENSG00000243135	0	0	0	0	0	0	0	0	0	0	0	0	0	0	0	0	0	0	0	0	0	0	0	0	0	0	0	0	0	0	0	0
ENSG00000248114	0	0	0	0	0	0	0	0	0	0	0	0	0	0	0	0	0	0	0	0	0	0	0	0	0	0	0	0	0	0	0	0
ENSG00000227802	0	0	0	0	0	0	0	0	0	0	0	0	0	0	0	0	0	0	0	0	0	0	0	0	0	0	0	0	0	0	0	0
ENSG00000228445	0	0	0	0	0	0	0	0	0	0	0	0	0	0	0	0	0	0	0	0	0	0	0	0	0	0	0	0	0	0	0	0
ENSG00000248705	0	0	0	0	0	0	0	0	0	0	0	0	0	0	0	0	0	0	0	0	0	0	0	0	0	0	0	0	0	0	0	0
ENSG00000178836	0	0	0	0	0	0	0	0	0	0	0	0	0	0	0	0	0	0	0	0	0	0	0	0	0	0	0	0	0	0	0	0
ENSG00000241635	0	0	0	0	0	0	0	0	0	0	0	0	0	0	0	0	0	0	0	0	0	0	0	0	0	0	1	0	0	0	0	0
ENSG00000185038	4	1	8	1	7	10	4	7	1	2	10	4	4	0	0	1	1	0	1	3	16	5	5	7	12	3	1	9	0	2	1	3
ENSG00000123485	2999	2141	2540	2346	979	940	673	978	1869	1571	5349	1334	1431	1368	1661	2064	1256	1824	1671	1602	1547	694	1186	589	1907	2890	638	4959	1142	1587	1432	1817
ENSG00000224287	134	82	103	87	21	18	8	21	4	2	17	2	43	35	45	39	95	105	85	104	43	12	27	18	1	7	4	11	27	42	44	62
ENSG00000279809	0	0	0	0	0	0	0	0	0	0	0	0	0	0	0	0	0	0	0	0	0	0	0	0	0	0	0	0	0	0	0	0
ENSG00000144481	0	0	0	0	0	0	1	0	3	6	27	7	3	5	2	8	0	0	0	0	0	0	0	0	2	13	6	14	6	7	3	13
ENSG00000237581	0	0	0	0	0	0	0	0	0	0	0	0	0	0	0	0	0	0	0	0	0	0	0	0	0	0	0	0	0	0	0	0
ENSG00000072080	0	0	0	1	0	0	0	0	0	0	1	0	0	0	1	0	0	0	0	0	0	0	1	0	0	0	1	0	0	0	0	0
ENSG00000279218	0	0	0	0	0	0	0	0	0	0	0	0	0	0	0	0	0	0	0	0	0	0	0	0	0	0	0	0	0	0	0	0
ENSG00000226442	0	0	0	0	0	0	0	0	0	0	0	0	0	0	0	0	0	0	0	0	0	0	0	0	0	0	0	0	0	0	0	0
ENSG00000261298	0	0	0	0	0	0	0	0	0	1	1	0	0	0	0	0	0	0	0	0	0	0	0	0	0	0	0	3	0	0	0	0
ENSG00000237784	0	0	0	0	0	0	0	0	0	0	0	0	1	0	0	1	0	0	0	0	0	0	0	1	0	0	0	1	0	0	0	0
ENSG00000228162	0	0	1	0	0	0	0	0	0	0	4	0	0	0	0	0	0	0	0	0	0	0	0	0	0	0	0	0	0	1	0	1
ENSG00000231682	22	8	19	6	0	0	0	0	10	2	13	5	0	1	0	2	0	0	2	0	0	0	0	0	0	0	2	0	0	0	1	0
ENSG00000232690	0	0	0	0	0	0	0	0	0	0	2	0	0	0	0	0	0	0	0	0	0	0	0	0	0	0	0	0	0	0	0	0
ENSG00000188042	4143	3978	3843	4011	135	138	99	115	7838	7125	38853	6249	2969	2538	2624	3270	595	1302	979	1688	192	68	177	68	6498	10190	8016	21139	2511	3037	2056	3432
ENSG00000280744	0	0	0	0	0	0	0	0	0	0	0	0	0	0	0	0	0	0	0	0	0	0	0	0	0	0	0	0	0	0	0	0
ENSG00000278593	0	0	0	0	0	0	0	0	0	0	0	0	0	0	0	0	0	0	0	0	0	0	0	0	0	0	0	1	0	0	0	0
ENSG00000235726	1	0	0	0	0	0	0	0	1	0	3	0	0	0	0	0	0	0	0	0	0	0	0	0	0	0	0	1	0	1	0	0
ENSG00000130147	0	5	3	0	0	0	0	0	189	174	444	148	1	0	0	0	0	1	0	2	0	0	0	0	66	160	50	268	0	0	0	0
ENSG00000226542	0	0	0	0	0	0	0	0	0	0	0	0	0	0	0	0	0	0	0	0	0	0	0	0	0	0	0	0	0	0	0	0
ENSG00000231032	0	0	0	0	0	0	0	0	0	0	0	0	0	0	0	0	0	0	0	0	0	0	0	0	0	0	0	0	0	0	0	0
ENSG00000235293	0	0	0	0	0	0	0	0	0	0	0	0	0	0	0	0	0	0	0	0	0	0	0	0	0	0	0	0	0	0	0	0
ENSG00000157985	833	921	797	861	683	740	471	771	88	155	232	124	0	0	0	0	136	305	233	508	1039	402	766	371	52	116	39	245	0	0	1	0
ENSG00000235529	0	0	0	0	0	1	1	1	1	1	6	0	0	0	0	0	0	1	0	0	0	0	2	0	2	1	0	2	0	0	0	0
ENSG00000222007	1	4	3	3	1	2	2	2	0	0	1	0	0	0	0	0	0	1	1	2	5	4	4	4	0	1	0	5	0	0	0	0
ENSG00000228499	0	1	0	2	0	0	0	0	0	0	3	0	1	0	0	0	0	0	0	1	0	0	0	1	0	0	0	0	0	0	0	0
ENSG00000238812	0	0	0	0	0	1	0	0	0	0	0	0	0	0	0	0	0	0	0	1	0	0	0	0	0	0	0	0	0	0	0	0
ENSG00000264676	0	0	2	0	1	2	0	1	1	1	0	0	0	0	0	0	1	0	0	1	2	0	2	2	0	0	0	0	0	0	0	0
ENSG00000207049	0	0	0	0	0	0	0	0	0	0	0	0	0	0	0	0	0	0	0	0	0	0	0	0	0	0	0	0	0	0	0	0
ENSG00000168505	0	0	0	0	0	0	0	0	0	0	1	0	0	0	0	0	0	0	0	0	0	0	0	0	0	0	0	0	0	0	0	0
ENSG00000233611	0	0	0	0	0	0	0	0	0	0	0	0	0	0	0	0	0	0	0	0	0	0	0	1	0	0	0	0	0	0	0	0
ENSG00000182177	0	0	0	0	0	0	0	0	0	0	3	0	0	1	0	0	0	0	0	0	0	0	0	0	0	4	1	1	0	0	0	0
ENSG00000200745	0	0	0	0	0	0	0	0	0	0	0	0	0	0	0	0	0	0	0	0	0	0	0	0	0	0	0	0	0	0	0	0
ENSG00000132321	0	0	0	0	0	0	0	0	4	4	10	3	0	0	0	0	0	0	1	0	0	0	0	0	3	2	4	22	0	0	0	0
ENSG00000270540	0	0	0	0	0	0	0	0	0	0	6	0	1	0	0	0	0	0	0	0	0	0	0	0	0	0	2	1	0	0	0	0
ENSG00000232893	0	0	0	0	0	0	0	0	0	0	0	0	0	0	0	0	0	0	0	0	0	0	0	0	0	0	0	0	0	0	0	0
ENSG00000231546	0	0	0	0	0	0	0	0	0	0	0	0	0	0	0	0	0	0	0	1	0	0	0	0	0	0	0	0	0	0	0	0
ENSG00000144476	21	2	8	5	51	74	41	81	315	288	2031	237	0	0	0	0	14	29	13	12	125	50	86	66	180	284	346	785	0	1	1	5
ENSG00000232328	0	0	0	0	0	0	0	0	0	0	0	0	0	0	0	0	0	0	0	0	0	0	0	0	0	0	0	0	0	0	0	0
ENSG00000202341	1	5	0	0	0	0	0	0	0	0	0	0	1	1	0	0	0	0	0	0	0	0	0	0	0	0	0	0	0	0	0	0
ENSG00000124835	4	6	4	9	3	4	1	2	1	0	8	2	5	4	1	0	2	1	1	3	4	0	1	0	3	4	3	7	0	1	2	1
ENSG00000227252	62	53	41	56	4	13	6	7	24	9	62	13	21	25	18	21	20	13	19	31	15	7	5	5	12	14	13	22	10	17	17	17
ENSG00000198612	[illegible]	[illegible]	[illegible]	[illegible]	[illegible]	[illegible]	[illegible]	[illegible]	[illegible]	[illegible]	[illegible]	[illegible]	[illegible]	[illegible]	[illegible]	[illegible]	[illegible]	[illegible]	[illegible]	[illegible]	[illegible]	[illegible]	[illegible]	[illegible]	[illegible]	[illegible]	[illegible]	[illegible]	[illegible]	[illegible]	[illegible]	[illegible]
ENSG00000224844	[illegible]	[illegible]	[illegible]	[illegible]	[illegible]	[illegible]	[illegible]	[illegible]	[illegible]	[illegible]	[illegible]	[illegible]	[illegible]	[illegible]	[illegible]	[illegible]	[illegible]	[illegible]	[illegible]	[illegible]	[illegible]	[illegible]	[illegible]	[illegible]	[illegible]	[illegible]	[illegible]	[illegible]	[illegible]	[illegible]	[illegible]	[illegible]
ENSG00000224132	[illegible]	[illegible]	[illegible]	[illegible]	[illegible]	[illegible]	[illegible]	[illegible]	[illegible]	[illegible]	[illegible]	[illegible]	[illegible]	[illegible]	[illegible]	[illegible]	[illegible]	[illegible]	[illegible]	[illegible]	[illegible]	[illegible]	[illegible]	[illegible]	[illegible]	[illegible]	[illegible]	[illegible]	[illegible]	[illegible]	[illegible]	[illegible]
ENSG00000163359	[illegible]	[illegible]	[illegible]	[illegible]	[illegible]	[illegible]	[illegible]	[illegible]	[illegible]	[illegible]	[illegible]	[illegible]	[illegible]	[illegible]	[illegible]	[illegible]	[illegible]	[illegible]	[illegible]	[illegible]	[illegible]	[illegible]	[illegible]	[illegible]	[illegible]	[illegible]	[illegible]	[illegible]	[illegible]	[illegible]	[illegible]	[illegible]
ENSG00000222022	[illegible]	[illegible]	[illegible]	[illegible]	[illegible]	[illegible]	[illegible]	[illegible]	[illegible]	[illegible]	[illegible]	[illegible]	[illegible]	[illegible]	[illegible]	[illegible]	[illegible]	[illegible]	[illegible]	[illegible]	[illegible]	[illegible]	[illegible]	[illegible]	[illegible]	[illegible]	[illegible]	[illegible]	[illegible]	[illegible]	[illegible]	[illegible]
ENSG00000222032	[illegible]	[illegible]	[illegible]	[illegible]	[illegible]	[illegible]	[illegible]	[illegible]	[illegible]	[illegible]	[illegible]	[illegible]	[illegible]	[illegible]	[illegible]	[illegible]	[illegible]	[illegible]	[illegible]	[illegible]	[illegible]	[illegible]	[illegible]	[illegible]	[illegible]	[illegible]	[illegible]	[illegible]	[illegible]	[illegible]	[illegible]	[illegible]
ENSG00000115648	[illegible]	[illegible]	[illegible]	[illegible]	[illegible]	[illegible]	[illegible]	[illegible]	[illegible]	[illegible]	[illegible]	[illegible]	[illegible]	[illegible]	[illegible]	[illegible]	[illegible]	[illegible]	[illegible]	[illegible]	[illegible]	[illegible]	[illegible]	[illegible]	[illegible]	[illegible]	[illegible]	[illegible]	[illegible]	[illegible]	[illegible]	[illegible]
ENSG00000227847	[illegible]	[illegible]	[illegible]	[illegible]	[illegible]	[illegible]	[illegible]	[illegible]	[illegible]	[illegible]	[illegible]	[illegible]	[illegible]	[illegible]	[illegible]	[illegible]	[illegible]	[illegible]	[illegible]	[illegible]	[illegible]	[illegible]	[illegible]	[illegible]	[illegible]	[illegible]	[illegible]	[illegible]	[illegible]	[illegible]	[illegible]	[illegible]
ENSG00000222449	[illegible]	[illegible]	[illegible]	[illegible]	[illegible]	[illegible]	[illegible]	[illegible]	[illegible]	[illegible]	[illegible]	[illegible]	[illegible]	[illegible]	[illegible]	[illegible]	[illegible]	[illegible]	[illegible]	[illegible]	[illegible]	[illegible]	[illegible]	[illegible]	[illegible]	[illegible]	[illegible]	[illegible]	[illegible]	[illegible]	[illegible]	[illegible]
ENSG00000071677	[illegible]	[illegible]	[illegible]	[illegible]	[illegible]	[illegible]	[illegible]	[illegible]	[illegible]	[illegible]	[illegible]	[illegible]	[illegible]	[illegible]	[illegible]	[illegible]	[illegible]	[illegible]	[illegible]	[illegible]	[illegible]	[illegible]	[illegible]	[illegible]	[illegible]	[illegible]	[illegible]	[illegible]	[illegible]	[illegible]	[illegible]	[illegible]

ENSG00000124839	2	6	5	3	1	5	4	3	1	0	1	0	0	0	0	0	0	1	0	0	0	1	1	1	0	0	0	1	0	0	0	0
ENSG00000234949	4	2	0	0	2	0	0	1	0	0	0	0	0	0	0	0	0	0	0	0	1	0	1	0	0	0	0	0	0	0	0	0
ENSG00000227107	0	0	0	0	0	0	0	0	0	0	0	0	0	0	0	0	0	0	0	0	0	0	0	0	0	0	0	0	0	0	0	0
ENSG00000124831	6779	5696	5681	5940	3886	3877	2969	4270	6227	4672	21019	3614	6657	5894	7256	7936	2749	3966	2943	3876	6344	2461	4696	2350	5712	8863	3660	14339	4767	5232	5499	6066
ENSG00000177483	123	104	75	78	38	47	23	55	25	27	67	9	125	129	74	111	23	21	13	48	57	29	39	25	10	25	17	56	21	54	28	55
ENSG00000132329	0	0	0	0	0	0	0	0	1	1	0	3	1	2	0	4	0	0	0	0	2	0	0	1	1	4	0	7	0	3	1	2
ENSG00000263723	0	0	0	0	0	0	0	0	0	0	0	0	0	0	0	0	0	0	0	0	0	0	0	0	0	0	0	0	0	0	0	0
ENSG00000184182	1135	1002	1041	975	352	329	263	374	261	256	1366	164	343	340	400	450	470	622	556	528	742	272	544	209	257	494	274	770	420	530	533	538
ENSG00000258984	11	12	10	17	3	6	3	1	5	0	11	1	2	2	0	4	3	1	2	10	5	3	2	3	1	4	2	4	1	4	2	4
ENSG00000251971	0	0	1	0	0	0	0	0	0	1	3	0	0	1	2	0	0	0	0	3	1	0	0	0	1	1	0	1	0	3	2	0
ENSG00000132330	753	640	591	602	154	205	142	228	325	263	1095	174	780	649	681	885	151	178	132	438	283	167	177	137	285	467	209	811	521	921	670	992
ENSG00000144488	10	8	16	18	9	5	1	9	3	0	13	1	82	72	104	136	2	11	6	21	10	5	8	6	1	5	1	6	75	130	86	204
ENSG00000168427	1	0	1	0	9	3	9	18	0	0	12	2	0	2	22	5	0	0	0	0	17	9	9	8	0	1	0	0	0	1	24	5
ENSG00000279484	0	0	0	0	0	1	0	0	0	0	0	2	0	0	4	0	0	0	0	0	0	0	0	0	0	0	0	0	0	0	4	2
ENSG00000178752	261	394	297	415	1	1	0	1	24	17	57	23	84	65	65	99	21	70	38	134	1	0	3	1	12	28	13	48	29	40	18	47
ENSG00000132323	1046	760	876	783	584	659	467	677	1008	711	3373	633	878	866	1054	1147	482	672	768	919	1273	584	900	505	977	1349	807	2329	1056	1278	1229	1451
ENSG00000186235	2	1	0	0	0	0	0	0	0	1	1	0	0	0	0	0	0	1	0	0	0	3	0	0	0	1	1	1	0	1	0	0
ENSG00000225057	3	5	1	6	3	5	4	10	5	1	15	4	3	5	4	3	1	2	3	5	2	3	4	2	6	8	3	9	2	5	5	13
ENSG00000144485	528	684	437	655	357	355	231	350	833	674	2475	532	598	577	561	789	98	356	127	372	715	332	608	220	665	1164	722	1697	399	410	383	519
ENSG00000132326	664	629	652	665	451	470	285	483	568	473	1640	370	771	656	794	806	134	229	251	530	722	414	455	310	403	759	277	1061	743	852	707	883
ENSG00000283635	4	11	12	11	3	1	2	3	4	2	7	2	12	8	8	6	3	6	6	1	6	3	5	4	1	10	0	4	1	1	2	5
ENSG00000232306	0	0	0	0	0	0	0	0	0	1	0	0	0	0	0	0	0	0	0	0	0	0	0	0	0	0	0	0	0	0	0	0
ENSG00000204104	755	506	689	611	234	260	205	279	448	359	1878	289	485	487	558	651	313	405	426	625	441	238	323	210	415	656	452	1282	500	663	529	633
ENSG00000202099	0	0	0	0	0	0	0	0	0	0	1	0	1	0	1	0	0	0	0	0	0	0	0	0	0	0	0	1	0	0	1	0
ENSG00000065802	1619	1279	1290	1278	96	190	104	205	1019	846	3807	669	1341	1137	1236	1608	470	567	606	1095	369	186	237	135	739	1260	741	2031	717	1108	766	1358
ENSG00000229915	1	0	2	4	0	1	1	0	3	1	17	0	10	4	4	4	4	0	1	1	2	2	0	1	4	4	0	6	4	6	6	5
ENSG00000274346	0	1	0	2	0	0	0	0	1	0	7	1	0	1	0	5	0	0	0	0	2	1	0	3	0	6	0	9	0	5	1	3
ENSG00000225493	1	0	0	0	0	0	0	0	0	1	1	1	0	4	0	2	0	0	0	1	3	1	1	0	1	1	0	1	1	3	0	1
ENSG00000234279	0	0	0	0	0	0	0	0	0	0	0	0	0	0	0	0	0	0	0	0	0	0	0	0	0	0	0	0	0	0	0	0
ENSG00000224252	0	0	0	0	0	0	0	0	0	0	0	0	0	0	0	0	0	0	0	0	0	0	0	0	0	0	0	0	0	0	0	0
ENSG00000226992	0	0	0	0	0	0	0	0	0	0	0	0	0	0	0	0	0	0	0	0	0	0	0	0	0	0	0	0	0	0	0	0
ENSG00000233608	0	0	0	0	0	0	0	0	2	2	7	1	0	0	0	0	0	0	0	0	0	0	0	0	5	4	3	5	2	0	0	0
ENSG00000227744	0	1	0	0	0	0	0	0	0	2	0	0	0	0	0	0	0	0	0	0	0	0	0	0	0	0	0	0	0	0	0	0
ENSG00000068024	2713	2494	3232	2535	556	684	398	754	1294	1184	4219	739	1428	1338	1159	1783	720	813	903	1969	682	357	494	286	847	1484	1050	2956	586	982	646	1032
ENSG00000266109	7	4	1	1	0	2	0	0	1	0	2	1	0	0	0	0	2	0	0	1	1	0	0	2	2	0	2	0	0	0	2	0
ENSG00000264810	7	0	2	8	0	1	1	1	3	0	8	2	4	2	2	1	0	0	0	3	1	0	0	0	2	0	0	7	0	2	0	1
ENSG00000280260	6	13	12	7	4	2	3	5	6	6	11	1	6	5	3	4	4	4	3	8	7	2	0	1	3	2	0	19	2	6	3	11
ENSG00000265215	1	5	2	3	0	0	0	1	0	0	5	0	0	3	3	0	0	0	0	4	0	0	3	1	1	0	1	2	0	0	1	0
ENSG00000264292	18	20	19	13	2	2	0	5	2	3	8	1	2	4	2	1	0	2	1	5	3	0	0	1	1	1	1	5	0	0	4	2
ENSG00000222020	53	43	54	50	20	15	11	14	15	7	27	6	13	16	8	8	33	26	30	44	11	12	10	10	1	7	3	11	5	8	7	8
ENSG00000196758	80	63	100	85	0	0	0	0	0	0	0	0	0	0	0	0	34	57	37	84	0	0	0	0	0	0	0	0	0	0	0	0
ENSG00000227459	39	32	57	31	0	0	0	0	0	0	1	0	0	0	0	0	31	47	63	57	0	0	0	0	0	1	0	0	0	0	0	0
ENSG00000259889	0	0	0	0	0	0	0	0	0	0	0	0	0	0	0	0	0	0	0	0	0	0	0	0	0	0	0	0	0	0	0	0
ENSG00000220256	0	1	1	2	0	0	0	0	0	0	1	0	0	0	0	0	0	0	0	0	0	0	0	0	0	0	0	0	0	0	1	0
ENSG00000130414	2643	1846	2140	1791	844	897	661	983	1914	1395	6468	1082	2453	1886	2204	2587	1381	1649	1514	1761	1667	673	1027	684	1541	2371	1566	4090	1984	2480	2186	2732
ENSG00000264279	6	3	3	5	4	7	3	9	4	3	11	1	9	8	10	6	4	5	1	10	11	5	0	2	8	2	3	10	4	14	6	5
ENSG00000182083	3	0	0	0	0	0	0	0	0	0	0	0	0	0	0	0	4	0	0	1	0	0	0	0	0	0	0	0	0	0	2	0
ENSG00000221961	0	0	0	0	0	0	0	0	0	0	0	0	0	0	0	0	0	0	0	0	0	0	0	0	0	0	0	0	0	0	0	0
ENSG00000178586	0	0	0	0	0	0	0	0	0	0	0	0	0	0	0	0	0	0	0	0	0	0	0	0	0	0	0	0	0	0	0	0
ENSG00000231278	0	0	0	0	0	0	0	0	0	0	0	0	0	0	0	0	0	0	0	0	0	0	0	0	0	0	0	0	0	0	0	0
ENSG00000213048	0	0	1	0	0	0	0	0	0	0	2	1	0	0	0	0	0	0	0	1	1	0	0	0	0	2	0	0	0	0	0	0
ENSG00000172428	801	523	575	614	157	180	145	242	473	274	1721	316	361	281	398	489	1598	1740	1573	1397	608	244	424	305	446	628	389	1087	676	583	829	829
ENSG00000178602	0	0	0	1	0	0	0	0	0	0	0	0	0	0	0	0	0	0	0	1	0	0	0	0	0	0	0	0	0	0	0	0
ENSG00000227479	0	0	0	0	0	0	0	0	0	0	0	0	0	0	0	0	0	0	0	0	0	0	0	0	0	0	0	0	0	0	0	0
ENSG00000276366	0	0	0	0	0	0	0	0	0	0	0	0	0	0	0	0	0	0	0	0	0	0	0	0	0	0	1	0	0	0	0	0
ENSG00000063660	482	591	387	570	344	395	263	335	340	306	1589	299	22	39	15	40	101	241	101	337	536	232	352	166	332	504	421	1345	6	30	16	41
ENSG00000218416	9	10	5	6	8	11	3	24	6	8	60	1	0	1	2	0	1	2	2	6	13	14	13	10	10	15	5	45	0	0	2	6
ENSG00000207611	0	0	1	0	2	0	2	3	2	1	5	2	0	0	0	0	0	0	0	1	3	0	0	1	0	0	0	5	0	0	0	0
ENSG00000144504	617	477	642	538	203	259	147	260	803	715	2930	507	596	532	509	631	254	309	282	716	400	168	275	179	564	951	519	1833	255	506	299	543
ENSG00000188542	164	145	177	125	64	64	43	78	171	142	743	113	111	98	98	141	122	141	134	204	103	37	81	48	150	198	178	376	77	77	83	135
ENSG00000142327	1070	990	1174	988	632	646	417	597	1004	812	3949	692	1147	1110	854	1428	287	434	352	691	775	365	487	301	797	1286	722	2262	662	873	454	891
ENSG00000260942	167	128	161	118	24	28	32	28	124	79	437	73	82	93	99	96	50	49	70	128	46	15	29	16	69	122	79	199	52	85	59	105
ENSG00000142330	1385	992	1150	1126	413	491	313	526	644	492	2737	433	567	498	478	685	516	600	582	905	704	354	480	326	540	886	640	1559	452	652	415	763
ENSG00000178623	17	14	18	24	8	12	9	16	57	51	477	75	33	26	54	47	7	7	7	22	26	4	15	17	44	67	113	213	14	37	46	60
ENSG00000276949	0	0	0	0	0	0	0	0	0	0	0	0	0	0	0	0	0	0	0	0	0	0	0	0	0	0	0	0	0	0	0	0
ENSG00000185176	0	0	0	0	0	0	0	0	0	0	0	1	0	1	1	0	0	0	0	0	0	0	0	0	0	0	2	1	0	1	0	0
ENSG00000219159	0	2	0	0	0	0	0	0	2	5	14	0	3	2	4	5	0	0	0	0	0	0	0	0	0	3	3	8	1	1	0	0
ENSG00000184945	0	0	0	0	0	0	0	0	0	0	0	0	0	0	0	0	0	0	0	0	0	0	0	0	0	0	0	0	0	0	0	0
ENSG00000274977	0	0	0	0	0	0	0	0	0	0	0	0	0	0	0	0	0	0	0	0	0	0	0	0	0	0	0	0	0	0	0	0
ENSG00000130294	1	1	0	1	0	0	0	0	95	143	128	116	3	18	8	25	0	0	0	1	1	0	0	0	39	153	54	239	1	13	5	9
ENSG00000172482	0	0	0	0	0	0	0	0	0	0	0	0	0	0	0	0	0	0	0	1	0	0	1	0	0	2	0	0	0	1	0	0
ENSG00000172478	0	0	0	0	0	0	0	0	0	0	0	0	6	11	10	14	0	0	0	0	0	0	0	0	0	0	0	0	26	70	10	56
ENSG00000226321	0	2	2	5	1	0	0	0	0	0	1	0	2	1	1	6	0	0	0	0	0	0	0	0	0	0	0	0	0	1	0	0
ENSG00000233392	0	3	1	0	0	0	0	0	0	0	0	0	10	9	2	15	0	0	0	1	0	0	0	0	0	0	0	0	1	2	0	2
ENSG00000223991	0	0	1	0	0	0	0	0	0	0	0	0	0	0	0	0	0	0	0	0	0	0	2	0	0	0	0	0	0	0	0	0
ENSG00000162804	47	50	53	49	23	42	33	46	61	46	218	30	175	182	161	230	13	21	9	53	54	32	31	33	36	56	53	134	59	143	97	174
ENSG00000229996	0	3	4	1	0	0	0	0	0	0	0	0	0	0	0	1	0	1	2	0	0	0	0	0	0	0	0	0	0	0	0	0
ENSG00000225521	0	1	0	0	0	2	0	0	2	0	0	0	0	1	0	0	1	0	0	0	0	1	0	0	1	0	0	0	0	0	0	0
ENSG00000122085	969	631	850	701	318	308	230	382	730	583	2695	421	824	837	909	1057	429	522	588	707	702	275	450	304	495	784	619	1380	601	973	842	1075
ENSG00000115687	2711	2133	2172	2140	1170	1449	1085	1388	1514	1176	4578	888	3838	3069	3684	3980	546	731	718	1646	1852	968	1181	1001	1157	1966	803	3575	2356	3100	2557	3595
ENSG00000235861	7	6	2	4	0	1	0	1	1	3	9	1	4	2	1	4	0	0	2	3	1	2	2	0	1	1	0	1	0	2	2	5
ENSG00000115685	1975	1250	1558	1483	795	749	598	863	1300	1012	3921	753	2002	1881	2216	2450	1249	1645	1464	1344	1466	556	1054	444	1220	1794	796	2749	1893	2104	2139	2379
ENSG00000146205	11	13	6	8	2	4	3	6	64	47	111	27	129	137	101	185	2	2	1	6	4	1	0	0	20	41	28	76	62	91	45	83
ENSG00000115677	9057	6897	7725	7244	4424	4713	3356	4809	7219	5756	25942	4682	7407	6575	7429	8554	2739	4126	3529	5626	6626	2731	4792	2285	5826	9986	6264	18145	4691	5922	4996	6344
ENSG00000168385	6722	4525	5642	4752	5358	5436	4007	5689	7832	6547	29838	5206	9751	8761	10265	11813	4498	6084	5317	5426	10846	4151	7083	3891	7769	11171	7258	20538	9674	10814	11393	12551
ENSG00000223374	41	47	27	52	26	34	21	25	30	36	133	19	25	31	33	45	9	12	10	32	28	21	17	16	26	44	23	71	21	43	25	66
ENSG00000006607	1905	1464	1525	1448	661	663	453	706	815	714	2872	525	1054	986	1140	1289	427	771	493	1296	899	376	564	383	911	1345	655	2317	634	907	779	1076
ENSG00000263752	0	0	2	0	0	1	0	0	0	1	2	0	0	0	0	0	0	0	0	0	0	0	0	0	1	0	0	0	1	1	0	0
ENSG00000224232	2	2	2	4	0	0	1	1	1	0	9	1	1	1	1	1	1	0	3	6	0	0	0	2	1	4	3	3	0	5	2	4
ENSG00000115694	3735	2826	3126	2996	1034	1163	805	1215	2097	1920	8136	1587	1219	1242	1094	1636	1499	2100	1567	2769	1549	693	1125	605	1580	2740	1906	4863	843	1138	745	1380
ENSG00000234235	0	2	0	0	0	0	1	0	0	1	1	0	0	1	0	1	0	0	1	0	1	0	0	0	0	0	0	0	0	0	0	0
ENSG00000176720	59	52	54	60	16	7	4	4	16	17	76	11	10	16	12	7	20	24	29	53	29	9	4	11	9	4	19	26	8	10	8	18
ENSG00000273113	0	1	1	1	0	0	0	3	2	1	2	1	4	3	7	0	0	0	1	0	2	3	1	0	0	2	4	3	1	0	2	3
ENSG00000176946	1545	1045	1209	1082	539	559	386	568	784	575	2355	479	1667	1348	1608	1691	488	692	660	898	874	318	631	252	558	1048	503	1376	1430	1295	1352	1337
ENSG00000168397	1981	1744	2007	1739	1182	1374	1024	1408	1289	1050	4577	796	1612	1307	1316	1730	869	985	913	1389	1977	876	1316	880	904	1548	1100	2755	1021	1509	1075	1642
ENSG00000168393	1856	1366	1742	1450	658	607	484	653	1314	1044	3679	811	1088	1135	1044	1493	1132	1578	1592	1759	1216	536	936	485	1351	1944	493	2836	1216	1417	1089	1671
ENSG00000168395	1094	933	1204	996	721	854	622	875	1392	1012	3700	737	1148	1128	1122	1564	519	772	783	1261	1548	663	1143	576	938	1507	725	2372	738	1194	946	1279
ENSG00000235351	8	15	8	16	14	18	12	13	20	6	25	7	11	5	9	18	0	1	4	14	14	9	12	11	7	4	7	17	2	6	8	11
ENSG00000180902	983	830	863	947	344	487	313	488	143	198	205	93	418	408	299	500	198	269	228	784	494	269	288	272	15	70	30	132	95	183	97	239
ENSG00000215692	8	14	13	12	6	10	7	12	1	0	0	0	9	7	4	7	2	9	3	12	17	4	4	5	0	0	0	0	5	4	2	6
ENSG00000234793	0	0	1	0	0	0	0	0	0	0	1	0	0	0	1	1	0	1	0	1	0	2	0	0	0	0	0	0	0	1	0	0
ENSG00000154252	6	15	6	9	4	4	2	11	1	2	0	0	1	3	1	5	0	3	0	12	3	3	3	5	0	0	0	0	0	1	0	3
ENSG00000215023	6	4	0	8	0	2	0	0	0	0	0	1	0	0	0	0	1	0	1	3	3	1	0	0	0	0	0	0	0	0	0	0
ENSG00000224272	6	5	8	5	0	2	0	5	0	0	0	0	4	4	0	7	1	1	0	5	0	0	0	1	0	0	0	0	1	1	3	1
ENSG00000204099	3	3	4	0	0	0	0	2	0	0	0	0	1	3	1	1	0	1	0	0	0	2	1	1	0	0	0	0	0	2	1	1
ENSG00000235151	0	0	0	0	0	0	0	0	0	0	0	0	0	0	2	2	0	0	0	0	0	0	0	0	0	0	0	0	0	1	0	0
ENSG00000188389	60	57	73	53	0	0	0	0	0	0	0	0	1	8	1	6	22	26	52	63	0	0	0	0	0	0	0	0	0	2	2	2
ENSG00000188011	12	21	15	13	0	0	0	0	0	0	0	0	0	0	0	0	0	2	0	6	0	0	0	0	0	0	0	1	0	1	0	0
ENSG00000233806	135	158	161	145	73	73	63	85	5	6	0	0	77	72	35	76	36	50	39	130	92	41	53	38	0	2	1	1	21	44	36	72
ENSG00000216921	42	44	54	42	18	36	18	27	2	3	1	0	29	26	29	46	11	12	3	43	21	10	14	15	1	0	0	1	14	32	27	41
ENSG00000237940	306	278	332	338	65	85	48	92	7	5	4	0	148	131	99	103	41	38	36	142	52	34	32	51	1	1	0	2	23	60	18	83
ENSG00000280119	47	53	49	67	20	22	12	18	0	0	0	0	60	40	35	55	10	20	13	58	29	20	23	18	0	0	0	0	9	24	4	43
ENSG00000226423	0	0	0	0	0	0	0	0	0	0	0	0	3	4	7	6	0	0	1	0	1	0	2	1	0	0	0	0	1	18	10	16
ENSG00000232002	0	0	0	0	0	0	0	0	0	0	0	0	1	0	0	0	0	0	0	0	0	0	0	2	0	0	0	0	1	0	0	0
ENSG00000261186	13	13	5	6	4	3	2	6	7	2	15	3	3	7	5	7	7	4	5	10	13	2	5	3	2	4	7	6	0	6	1	2
ENSG00000220804	164	142	111	144	24	47	26	29	78	65	362	60	140	151	111	179	70	105	56	102	62	16	49	17	77	100	112	274	83	167	111	191
ENSG00000224160	1	0	0	0	1	0	1	0	0	0	8	0	3	1	0	0	1	0	0	2	0	0	0	0	0	0	0	5	2	0	0	1
ENSG00000244528	5	3	7	5	1	0	0	1	2	0	8	2	1	4	2	4	0	0	0	0	4	0	0	0	1	2	3	7	0	1	4	3
ENSG00000271153	4	1	2	14	0	1	2	1	1	0	8	1	6	4	3	6	0	1	1	4	1	0	2	0	1	2	0	3	3	7	4	7
ENSG00000223587	0	0	0	0	0	0	0	0	0	0	0	0	0	0	0	0	0	0	0	0	1	0	0	0	0	0	0	0	0	0	0	0
ENSG00000224918	0	0	0	0	0	0	0	0	0	0	0	0	0	0	0	0	0	0	0	0	0	0	0	0	0	0	0	0	0	0	0	0
ENSG00000224318	0	0	0	0	0	0	0	0	0	0	0	0	0	0	0	0	0	0	0	0	0	0	0	0	0	0	0	0	0	0	0	0
ENSG00000134121	0	0	0	0	0	0	0	0	0	0	2	0	0	0	0	0	0	0	0	0	0	0	0	0	1	0	2	1	0	0	1	1
ENSG00000252017	0	0	0	0	0	0	0	0	0	0	0	0	0	0	0	0	0	0	0	0	0	0	0	0	0	0	0	0	0	0	0	0
ENSG00000231660	0	0	0	0	0	0	0	0	0	0	0	0	0	0	0	0	0	0	0	0	0	0	0	0	0	0	0	0	0	0	0	0
ENSG00000234661	0	0	0	0	0	0	0	0	0	0	0	0	0	0	0	0	0	0	0	0	0	0	0	0	0	0	0	0	0	0	0	0
ENSG00000224957	0	3	1	0	0	0	0	0	0	0	0	0	0	0	0	1	0	0	0	0	0	0	0	0	0	0	0	0	0	0	0	0
ENSG00000238075	0	0	0	0	0	0	0	0	0	0	0	0	0	0	0	0	0	0	0	0	0	0	0	0	0	0	0	0	0	0	0	0
ENSG00000224239	4	2	2	2	1	2	0	0	0	0	0	0	0	0	0	0	0	0	0	1	0	0	0	0	0	0	0	0	0	0	0	0
ENSG00000235158	0	0	0	0	0	0	0	0	0	0	0	0	0	0	0	0	0	1	0	0	0	0	0	0	0	0	1	0	0	0	2	0
ENSG00000244169	0	0	0	0	0	0	0	0	0	0	0	0	0	0	0	0	0	0	0	0	0	0	0	0	0	0	0	0	0	0	0	0
ENSG00000134115	1	0	0	1	0	0	0	0	0	3	23	0	0	0	0	0	0	0	0	2	0	0	0	0	4	4	8	12	1	0	0	0
ENSG00000232563	0	0	0	0	0	0	0	0	0	1	0	0	0	0	0	0	0	0	0	0	0	0	0	0	0	0	0	0	0	0	0	0
ENSG00000184423	0	0	0	0	0	0	0	0	2	2	16	2	0	0	0	0	0	0	0	1	0	0	0	0	2	1	3	6	0	0	0	0
ENSG00000214074	0	0	0	0	0	0	0	0	7	3	10	0	0	0	0	0	0	0	0	0	0	0	0	0	4	5	6	3	0	0	0	0
ENSG00000214073	0	0	0	0	0	0	0	0	8	3	33	2	0	0	0	0	0	0	0	0	0	0	0	0	9	13	8	19	0	0	0	1
ENSG00000225044	0	0	0	0	0	0	0	0	5	6	22	1	0	0	0	1	0	0	0	0	0	0	0	0	6	11	4	11	0	0	0	0
ENSG00000223040	0	0	0	0	0	0	0	0	0	0	0	0	0	0	0	0	0	0	0	0	0	0	0	0	0	0	0	0	0	0	0	0
ENSG00000144619	2	0	0	1	2	2	0	1	1	2	0	2	25	15	31	19	0	0	0	0	1	3	3	0	0	1	0	2	4	9	15	5
ENSG00000227588	0	0	0	0	0	0	0	0	0	0	0	0	0	0	0	0	0	0	0	0	0	0	0	0	0	0	0	0	0	0	0	0
ENSG00000230398	0	0	0	0	0	0	0	0	0	0	0	0	0	0	0	0	0	0	0	0	0	0	0	0	0	0	0	0	0	0	0	0
ENSG00000225310	0	0	0	0	0	0	0	0	0	0	0	0	0	0	0	0	0	0	0	0	0	0	0	0	0	0	0	0	0	0	0	0
ENSG00000237990	0	0	0	0	0	0	0	0	0	0	2	0	0	0	1	0	0	0	0	0	0	0	1	0	0	0	0	0	0	0	0	0
ENSG00000091181	1	0	5	0	0	1	0	0	0	0	2	0	1	1	1	0	3	2	0	0	0	1	0	3	1	0	1	1	1	1	0	0
ENSG00000253049	0	0	0	0	0	0	0	0	0	0	0	0	0	0	0	0	0	0	0	0	0	0	0	0	0	0	0	0	0	0	0	0
ENSG00000072756	1295	893	1034	826	1010	1068	806	1058	683	589	2678	433	854	804	959	1053	983	1137	1076	1402	2224	966	1411	1011	772	1024	554	1598	903	1182	995	1373
ENSG00000281584	22	24	21	22	15	21	13	23	15	12	57	14	18	17	26	24	5	2	4	26	36	37	22	23	5	17	10	37	12	28	7	19
ENSG00000113851	1373	929	1107	888	1456	1431	1193	1507	821	721	3619	579	702	683	739	789	950	1016	1056	1370	4168	1740	2830	2082	766	1062	947	2159	687	906	843	1096
ENSG00000271870	110	93	115	72	130	93	68	109	99	65	304	64	94	58	86	106	79	54	64	127	210	97	87	102	86	98	66	159	51	114	50	96
ENSG00000223727	1	0	0	0	2	2	3	5	0	0	1	0	0	0	0	0	1	0	0	0	2	0	0	0	1	0	5	3	0	0	2	0
ENSG00000144455	269	186	208	189	115	111	83	138	89	86	446	75	173	125	129	172	127	180	154	213	193	98	173	73	101	113	118	283	137	173	142	177
ENSG00000175928	5	0	1	6	281	299	221	317	148	138	895	131	10	9	7	10	0	0	0	0	469	173	375	181	139	254	194	580	1	3	4	6
ENSG00000229241	7	3	2	3	1	1	0	1	0	3	1	3	4	2	0	5	1	0	0	2	0	0	0	0	7	4	1	4	1	2	1	3
ENSG00000170364	468	307	351	331	609	603	424	648	468	343	1542	291	407	361	432	464	502	731	619	628	1606	584	1001	582	443	596	326	959	542	596	527	748
ENSG00000234325	1	8	0	2	0	0	0	5	1	1	2	0	1	2	0	0	0	0	0	4	2	3	0	1	0	0	2	1	0	0	0	0
ENSG00000231249	14	7	14	8	6	13	4	2	6	6	84	13	20	13	13	17	4	7	6	12	6	0	2	2	7	17	28	39	4	8	6	14
ENSG00000150995	1081	831	941	856	1881	1889	1310	1962	1697	1498	11933	1467	2926	2863	3237	3549	291	436	343	726	2214	991	1560	860	1996	3206	4470	9634	2065	3145	2662	3613
ENSG00000235947	1	2	3	3	4	4	3	6	5	4	40	2	8	8	6	3	1	0	1	4	4	5	6	9	11	7	13	38	6	9	4	12
ENSG00000275981	0	0	0	0	1	1	2	0	1	0	18	0	5	4	3	1	0	0	0	0	2	0	1	3	5	3	0	8	1	1	0	6
ENSG00000235978	35	24	23	22	7	9	8	10	73	56	286	53	48	41	82	54	14	10	25	32	7	4	6	6	49	72	30	146	39	51	64	65
ENSG00000235831	10	4	13	8	8	9	7	17	97	81	372	78	230	169	300	242	11	11	14	22	12	3	7	4	73	139	89	182	116	123	158	149
ENSG00000134107	961	1133	723	1037	1449	1463	1053	1836	16470	13006	57410	10351	3634	3526	3656	4545	95	161	41	157	391	216	266	148	10074	17276	11395	29748	1312	2155	1370	2507
ENSG00000230182	0	0	0	0	0	0	0	0	0	0	1	0	0	0	0	0	0	0	0	0	0	0	0	0	0	0	0	1	0	0	0	0
ENSG00000224082	0	0	0	0	0	3	0	0	1	0	2	0	0	0	0	0	0	0	0	0	0	0	0	0	0	0	2	2	0	0	0	0
ENSG00000207283	0	0	0	0	0	0	1	0	0	0	1	0	0	0	0	0	0	0	0	0	0	0	0	0	0	0	0	0	0	0	0	0
ENSG00000237566	0	0	0	0	0	0	0	0	0	0	0	0	0	0	0	0	0	0	0	0	0	0	0	0	1	0	0	0	0	0	0	0
ENSG00000134108	1914	1371	1547	1241	1370	1377	971	1412	1323	1121	5840	887	1115	970	1179	1227	940	1453	1355	1493	3046	1196	2083	1143	1506	2343	1667	4091	1252	1431	1475	1577
ENSG00000233912	4	7	8	5	32	25	20	25	3	6	15	6	4	1	3	2	4	2	2	6	18	4	13	7	15	7	2	22	2	4	6	8
ENSG00000268509	48	37	47	34	6	4	2	2	0	1	9	2	4	4	4	2	3	5	12	14	8	4	2	0	4	0	2	5	1	6	1	4
ENSG00000134109	8780	5549	7175	5550	2700	2694	1884	2908	2638	2290	9621	1660	2068	1777	2146	2212	1492	1898	1888	2889	3645	1538	2561	1329	2278	3608	2084	6023	1327	1810	1636	1845
ENSG00000265180	0	0	0	0	0	0	0	0	0	0	0	0	0	0	0	0	0	0	0	0	0	0	0	0	0	0	0	0	0	0	0	0
ENSG00000241227	0	0	0	0	0	0	1	0	0	0	0	0	0	0	0	0	0	0	0	0	0	0	0	0	0	0	0	1	0	0	0	0
ENSG00000230944	0	3	0	0	0	0	0	0	0	0	0	0	0	0	0	0	0	0	0	0	0	0	0	0	0	0	0	0	0	0	0	0
ENSG00000229642	1	0	0	0	0	0	0	0	0	0	0	0	11	15	11	13	0	0	0	0	0	0	0	0	0	0	0	0	3	6	4	7
ENSG00000226022	0	0	0	0	0	0	0	0	0	0	0	0	0	0	0	2	0	0	0	0	0	0	0	0	0	0	0	0	0	1	0	0
ENSG00000232958	0	0	0	0	0	0	0	0	0	0	0	0	0	0	1	0	0	0	0	0	0	0	0	0	0	0	0	0	0	1	0	1
ENSG00000189229	1	1	0	1	341	307	258	368	0	0	0	0	6	10	7	7	0	0	0	0	682	260	398	267	0	0	0	0	2	8	12	15
ENSG00000226258	0	0	0	0	64	63	34	54	0	0	0	0	0	2	0	2	0	0	0	0	128	59	74	70	0	0	0	0	0	0	5	0
ENSG00000223719	0	0	0	0	36	41	44	48	0	0	1	0	0	0	0	0	0	0	0	0	42	32	25	27	0	0	0	0	0	0	0	0
ENSG00000196277	0	0	0	0	11	7	10	6	1	0	0	0	1	1	2	1	0	0	0	0	14	9	13	8	3	2	1	1	0	0	3	4
ENSG00000236152	0	0	0	0	1	0	0	0	1	0	0	0	0	0	0	0	0	0	1	0	0	0	0	0	0	0	0	0	1	0	0	0
ENSG00000237665	0	0	0	0	1	0	1	0	0	0	0	0	0	0	0	0	0	0	0	0	1	1	1	0	0	0	0	0	2	0	0	0
ENSG00000236202	0	0	0	0	0	0	0	0	0	0	3	0	0	1	1	1	0	0	0	0	0	0	1	0	0	0	0	0	0	0	0	5
ENSG00000270207	0	0	0	0	0	0	0	0	0	0	5	0	2	1	4	0	0	0	0	0	1	0	0	0	0	0	0	0	0	5	5	2
ENSG00000227110	0	0	0	2	0	0	0	0	0	0	0	0	1	3	0	2	0	0	0	0	0	0	1	0	0	0	0	0	0	1	2	0
ENSG00000228351	0	0	0	0	0	0	0	0	0	0	0	0	0	0	0	1	0	0	0	0	0	0	0	0	0	0	0	0	0	0	0	0
ENSG00000253081	0	0	0	0	0	0	0	0	0	0	0	0	0	0	0	0	0	0	0	0	0	0	0	0	0	0	0	0	0	0	0	0
ENSG00000231401	0	0	0	0	0	0	0	0	0	1	1	0	0	0	0	0	0	0	0	0	0	0	0	0	0	1	1	0	0	0	0	1
ENSG00000071282	0	0	0	0	2	1	0	0	0	0	3	1	112	122	156	184	0	0	0	0	0	0	1	3	0	2	0	1	52	56	64	58
ENSG00000224884	0	0	0	0	0	1	0	0	0	0	0	0	0	0	0	0	0	0	0	0	0	0	0	0	0	0	0	0	0	0	0	0
ENSG00000125046	4	6	5	4	10	22	24	16	1	2	14	1	9	10	9	20	3	3	0	8	19	9	13	20	2	3	2	7	2	3	2	8
ENSG00000215160	0	1	0	1	1	1	1	1	0	0	5	0	0	0	3	1	0	0	0	0	2	1	0	4	0	1	0	0	0	0	1	1
ENSG00000182533	13	8	9	8	11	8	6	10	11	7	13	1	10	4	13	10	3	2	0	13	12	6	3	13	4	10	1	20	3	9	5	7
ENSG00000180914	170	136	144	175	105	118	93	77	90	72	293	56	132	144	139	179	61	82	56	108	125	43	51	51	71	73	53	150	46	119	67	132
ENSG00000070950	2064	1592	1660	1326	1741	1707	1455	1787	1024	886	2734	627	1133	926	1223	1163	1723	2046	2066	1899	4360	1566	2862	1714	1087	1528	474	2358	1211	1392	1611	1590
ENSG00000281863	0	1	2	2	0	1	0	0	5	0	10	0	1	0	1	1	0	0	0	2	0	1	2	1	1	3	1	7	0	3	1	5
ENSG00000199815	0	0	0	0	0	0	0	0	0	0	0	0	0	0	0	0	0	0	0	0	0	0	0	0	0	0	0	0	0	0	0	0
ENSG00000196220	243	225	211	256	863	917	695	909	792	689	4965	631	35	37	16	39	69	126	70	239	1272	510	816	506	622	1078	1572	3349	23	30	18	32
ENSG00000224808	0	0	0	0	0	0	1	0	2	1	3	0	0	0	0	0	0	0	0	0	1	0	0	0	7	2	5	12	0	1	0	0
ENSG00000228723	0	4	1	2	3	4	1	9	11	9	26	4	0	0	0	0	0	0	0	5	13	6	6	4	9	15	10	48	0	2	0	1
ENSG00000227929	1	2	1	2	12	6	8	13	16	6	40	5	0	0	0	1	1	1	3	6	11	2	11	8	16	18	16	52	0	1	0	0
ENSG00000235830	0	0	0	0	1	0	1	1	0	0	1	0	0	0	0	0	0	0	0	3	0	0	1	1	0	0	0	1	0	0	0	0
ENSG00000254485	3	10	6	8	3	9	7	5	5	6	15	2	12	9	11	14	4	3	3	6	9	3	6	1	2	5	1	9	3	7	7	9
ENSG00000214041	2	0	3	2	0	2	0	0	3	1	7	1	1	2	0	1	1	1	0	4	2	4	1	0	0	2	2	7	0	0	0	2
ENSG00000206573	903	630	782	569	550	622	473	598	834	692	3093	498	455	486	563	632	342	354	349	690	991	437	704	510	693	960	586	1711	353	591	538	700
ENSG00000134077	1954	1252	1543	1340	1201	1270	971	1417	1336	1038	4358	820	971	923	1003	1204	1203	1600	1322	1467	2415	929	1734	925	1304	1786	968	2979	888	1129	971	1211
ENSG00000168137	6575	5301	5731	5107	5306	5684	4271	5965	4202	3500	13309	2368	3366	2870	3176	3755	1246	1511	1356	3163	7294	3398	5088	3201	3048	5039	2898	9072	1600	2963	2183	3423
ENSG00000156959	4	0	3	2	8	5	7	8	8	11	59	9	0	3	1	5	3	3	0	11	2	3	1	3	8	6	13	26	1	6	0	6
ENSG00000235338	0	0	0	0	0	0	0	0	0	0	0	0	0	0	0	0	0	0	0	0	0	0	0	0	0	0	0	0	0	0	0	0
ENSG00000163719	968	710	850	632	889	910	671	996	710	555	2841	486	688	652	785	859	310	464	492	562	1559	634	1162	573	569	1071	711	1678	577	689	685	808
ENSG00000144550	12	16	8	9	20	13	14	14	2	2	23	7	3	2	1	2	6	5	1	9	37	20	14	28	2	2	1	12	5	3	3	4
ENSG00000156983	1493	1038	1396	1119	1808	1872	1376	1818	1213	908	5004	851	812	779	987	976	437	564	613	967	3595	1404	2448	1280	1097	1801	1028	3250	829	908	847	1062
ENSG00000114026	477	273	451	287	1064	1140	926	1266	1770	1374	6869	1105	356	345	293	382	172	209	219	390	1752	739	1427	756	935	1600	1003	3073	263	327	299	371
ENSG00000134072	15	19	29	21	50	68	47	48	2092	1531	7355	1590	21	15	13	19	8	7	10	33	90	40	42	40	1216	2021	954	3510	8	20	16	17
ENSG00000171148	2234	1691	1987	1621	2490	2478	1758	2640	2282	1745	6907	1468	1207	1068	1281	1369	965	1325	1584	1499	4091	1638	3289	1410	1564	2216	1111	3741	1119	1225	1308	1332
ENSG00000241553	1226	721	1093	736	3081	2932	2183	3160	2409	1838	10486	1666	832	706	899	890	863	1213	1401	1228	6122	2472	4509	2091	2599	3760	1936	6252	1241	1356	1263	1372
ENSG00000250151	302	242	267	213	53	42	39	53	351	276	2156	274	121	115	171	176	78	52	61	162	64	21	32	26	212	317	436	872	63	158	76	166
ENSG00000214021	2466	2028	2630	1985	559	692	481	673	3459	2865	21155	2453	1501	1399	1661	1976	598	456	499	1740	774	486	437	422	1864	2812	4862	8520	589	1619	1000	2021
ENSG00000269886	10	8	11	4	1	2	1	1	13	4	18	0	4	1	3	4	2	2	2	2	3	3	3	3	6	6	3	18	1	4	1	9
ENSG00000156990	1247	976	977	899	1235	1160	851	1233	678	591	2128	478	1231	1011	1033	1283	495	804	591	882	1843	804	1532	724	496	916	412	1154	864	1015	851	1198
ENSG00000187288	0	0	0	0	0	1	0	0	0	0	2	0	3	0	0	1	1	0	1	0	0	0	0	0	0	0	0	0	0	0	1	0
ENSG00000171135	618	447	527	441	506	490	372	571	616	479	2001	378	531	542	648	689	398	520	560	571	952	377	749	323	484	849	412	1192	597	650	687	723
ENSG00000163701	84	51	102	59	921	948	652	905	13	12	35	1	8	3	4	6	32	26	54	95	1667	694	1216	652	3	2	12	8	7	8	3	6
ENSG00000163702	71	56	73	36	146	132	101	129	20	31	112	22	15	14	12	29	24	42	26	43	147	67	81	63	21	16	23	58	21	10	12	26
ENSG00000212327	1	0	0	0	3	1	2	2	0	0	0	0	0	0	0	0	0	0	0	0	2	1	3	3	0	0	0	0	0	0	0	0
ENSG00000163703	378	287	339	366	1180	1359	831	1390	375	290	1351	197	317	330	307	395	129	141	108	219	1076	431	557	436	201	297	319	625	143	240	148	274
ENSG00000269894	13	19	18	13	42	45	52	42	23	22	130	12	15	8	19	15	10	8	18	14	60	49	35	36	14	28	26	44	10	10	11	16
ENSG00000163704	164	165	181	134	189	229	154	244	86	72	343	65	327	292	418	380	42	62	65	123	343	134	197	133	47	76	84	224	292	331	349	467
ENSG00000230082	14	12	9	14	13	26	12	26	20	8	78	4	10	4	8	6	7	3	1	16	18	9	9	15	5	10	18	36	2	8	4	12
ENSG00000269982	85	58	91	84	75	91	60	102	77	68	406	54	66	38	48	53	24	15	23	55	137	46	81	42	48	93	111	258	28	55	36	62
ENSG00000125037	653	455	636	431	611	715	483	743	487	366	1873	398	565	505	600	640	660	953	1013	696	1687	694	1079	609	478	704	477	1268	765	672	1029	837
ENSG00000180385	540	367	573	368	372	413	295	450	571	491	1448	260	686	621	784	816	126	151	187	449	585	246	267	220	341	479	233	985	420	807	615	944
ENSG00000232901	54	18	46	34	23	33	35	33	29	22	107	13	28	24	38	26	6	10	13	34	41	14	23	19	12	31	7	52	8	41	24	47
ENSG00000206567	175	140	146	131	94	82	56	87	123	94	334	65	143	179	192	229	28	34	50	105	138	78	65	53	55	71	46	207	114	172	162	207
ENSG00000186162	205	129	220	164	146	147	134	190	122	97	425	86	106	122	181	144	96	164	158	181	278	118	243	111	126	242	89	283	126	128	206	156
ENSG00000144554	3660	2815	3562	2875	4531	4683	3930	5168	2378	1912	6003	1361	2335	2025	2390	2715	1314	1919	2018	2665	7767	3194	5723	3006	1897	2745	783	4956	1530	2224	2067	2616
ENSG00000201182	3	1	1	2	1	2	3	1	4	0	1	0	0	4	3	1	0	0	0	1	2	3	0	1	3	2	2	8	0	2	3	2
ENSG00000229217	1	0	3	1	3	2	3	8	2	2	7	3	4	0	0	0	3	1	2	4	12	1	5	1	1	1	0	6	2	3	1	3
ENSG00000163705	26	34	33	31	25	28	36	36	14	15	61	10	16	13	19	20	7	10	6	22	50	22	31	34	18	23	4	56	1	23	13	25
ENSG00000254999	2675	1764	2068	1977	2490	2628	1937	2705	1990	1429	7086	1306	1797	1745	1974	2279	2672	3189	3048	2357	5432	2263	4075	2279	1840	2794	1689	4762	2388	2226	3162	2876
ENSG00000134086	3894	2640	3565	2809	3334	3360	2573	3697	2277	1858	9181	1425	2709	2439	2960	3251	2236	2680	2805	2920	6507	2608	4799	2104	2026	3016	2207	5496	2637	3013	2835	3213
ENSG00000134070	5	3	5	7	4	7	0	6	4	9	20	0	140	96	165	124	0	1	4	9	2	0	4	1	9	6	6	12	123	136	184	135
ENSG00000157014	1100	819	1096	812	811	869	658	849	778	603	2849	478	762	636	883	888	332	484	604	890	1455	633	1004	491	778	1211	602	1855	741	948	798	980
ENSG00000272410	0	0	1	0	0	0	0	0	0	0	1	1	0	0	0	0	0	0	0	0	0	0	0	0	0	0	0	0	0	0	0	0
ENSG00000231177	55	36	61	39	47	56	37	62	45	42	210	33	59	54	65	56	14	13	20	42	81	49	52	40	57	63	62	154	29	85	68	88
ENSG00000157017	40	32	64	30	31	41	31	47	35	27	131	28	102	140	163	177	19	12	16	57	70	40	43	34	44	60	32	160	81	156	149	179
ENSG00000240288	72	41	62	44	28	46	33	51	46	42	221	38	94	70	107	114	36	24	19	49	62	37	43	41	51	91	32	141	57	124	94	131
ENSG00000276678	3	3	7	2	4	1	1	2	3	0	12	0	3	7	6	6	2	0	4	2	3	2	1	1	2	2	5	10	6	3	7	13
ENSG00000157020	2243	1670	2012	1665	1944	1982	1478	2128	1217	955	4169	786	1944	1637	1937	2286	626	1062	1121	1459	3161	1429	2426	1219	1106	1804	851	2863	1655	2089	1763	2149
ENSG00000157087	73	52	87	54	0	0	1	1	0	0	12	3	0	4	2	7	20	17	19	44	1	0	0	1	0	0	4	9	0	4	0	1
ENSG00000264534	0	0	0	0	0	0	0	2	0	0	0	0	0	0	0	0	0	0	0	0	0	0	0	0	0	0	0	0	0	0	0	0
ENSG00000216135	0	0	0	0	0	0	0	0	0	0	0	0	0	0	0	0	0	0	0	0	0	0	0	0	0	0	0	0	0	0	0	0
ENSG00000236999	0	1	0	0	0	0	0	0	0	0	0	0	0	0	0	1	0	0	0	0	0	0	0	0	0	0	0	0	0	0	0	0
ENSG00000224771	0	0	0	0	0	0	0	0	0	0	0	0	0	0	0	0	0	0	0	0	0	0	0	0	0	0	0	0	0	0	0	0
ENSG00000226567	0	0	0	0	0	0	0	0	0	0	2	0	0	0	0	0	0	0	0	0	0	0	0	0	0	0	0	0	0	0	0	0
ENSG00000230599	0	0	0	0	0	0	0	0	0	0	0	0	0	0	0	0	0	0	0	0	0	0	0	0	0	0	0	0	0	0	0	0
ENSG00000132164	0	0	0	0	0	0	0	0	0	0	0	0	0	0	0	0	0	0	0	0	0	0	0	0	0	4	0	0	0	0	0	0
ENSG00000157103	0	1	0	2	0	0	0	0	0	0	0	0	0	0	0	0	0	1	0	0	0	0	0	0	0	0	0	0	0	0	0	0
ENSG00000232287	0	0	0	0	0	0	0	0	0	0	0	0	0	0	0	0	0	0	0	0	0	0	0	0	0	0	0	0	0	0	0	2
ENSG00000196639	20	10	40	15	0	1	2	4	0	1	2	0	2	1	2	0	10	18	27	25	4	1	2	2	1	3	0	5	3	0	0	1
ENSG00000213964	0	0	0	0	0	0	0	0	0	0	0	0	0	0	0	0	0	0	0	0	0	0	1	0	0	0	0	0	0	0	0	0
ENSG00000197548	498	303	391	295	400	419	289	417	370	300	1364	226	312	330	320	392	263	418	333	339	618	304	534	257	335	490	333	915	288	385	424	381
ENSG00000226621	0	1	0	0	1	0	2	1	0	0	0	0	1	2	2	0	0	0	0	2	3	1	0	0	0	0	0	0	0	1	1	1

ENSG00000235240	0	0	3	0	2	0	0	1	2	0	3	1	0	0	1	0	0	0	0	0	1	3	1	2	0	1	0	3	1	0	0	0
ENSG00000270531	1	2	1	1	4	1	1	4	1	0	1	0	1	0	0	3	0	0	0	5	6	4	7	2	3	1	4	5	1	0	0	2
ENSG00000144560	2401	2043	2175	1959	1695	1758	1148	1848	1123	962	3889	768	1293	1130	1400	1331	502	780	645	909	2394	1020	1608	862	867	1356	841	2396	867	974	828	1095
ENSG00000271716	17	8	16	7	13	9	8	7	8	8	18	4	4	6	4	6	4	2	2	3	16	7	12	12	10	12	3	26	4	7	7	6
ENSG00000272483	7	3	12	8	3	7	7	8	6	3	22	4	3	5	2	4	0	2	0	8	10	4	6	8	5	14	3	27	2	5	2	12
ENSG00000144559	731	478	586	461	346	303	270	329	293	234	938	126	637	416	476	521	524	426	417	621	712	301	521	332	252	327	225	634	468	591	593	624
ENSG00000230342	0	0	1	0	0	0	0	0	4	2	4	3	1	0	3	2	0	0	0	0	1	0	0	0	4	3	1	8	0	4	1	1
ENSG00000224263	0	0	0	0	0	0	0	0	0	0	0	0	0	0	0	0	0	0	0	0	0	0	0	0	0	0	0	0	0	0	0	0
ENSG00000225275	0	1	0	0	0	0	0	0	0	0	0	0	0	0	0	0	0	1	0	0	0	0	0	0	0	0	0	0	0	0	0	0
ENSG00000231897	0	0	0	0	0	0	0	0	0	0	0	0	0	0	0	0	0	1	0	0	0	0	0	0	0	0	0	0	0	0	0	0
ENSG00000251038	0	0	0	0	0	0	0	0	0	0	0	0	0	0	0	0	0	0	0	0	0	0	0	0	0	0	0	0	0	0	0	0
ENSG00000263988	0	0	0	0	0	0	0	0	0	0	0	0	0	0	0	0	0	0	0	0	0	0	0	0	0	0	0	0	0	0	0	0
ENSG00000157152	1	21	3	22	1	0	0	0	2	4	9	1	0	0	0	0	1	6	2	11	0	0	2	0	3	8	3	11	0	0	0	0
ENSG00000226642	0	1	0	0	0	0	0	0	0	0	0	0	0	0	0	0	0	0	0	0	0	0	0	0	0	0	0	0	0	0	0	0
ENSG00000157150	0	0	0	1	0	0	0	0	0	0	0	0	0	0	0	0	0	0	0	0	0	0	0	0	0	0	0	0	0	0	0	0
ENSG00000233026	0	0	0	0	0	0	0	0	0	0	0	0	0	0	0	0	0	0	0	0	0	1	0	0	0	0	0	0	0	0	0	0
ENSG00000236062	0	0	0	0	0	0	0	0	0	0	0	0	0	0	0	0	0	0	0	0	0	0	0	1	0	0	0	0	0	0	0	0
ENSG00000132170	4	6	7	1	1	0	0	0	0	1	4	0	0	0	1	0	1	5	8	3	1	0	0	0	0	0	1	4	0	0	2	0
ENSG00000225026	0	0	0	0	0	0	0	0	1	0	0	0	0	0	0	0	0	0	0	0	0	0	0	0	0	0	0	0	0	2	0	0
ENSG00000154743	821	528	681	648	266	328	250	337	428	350	1503	240	663	608	697	715	625	833	715	978	574	262	459	273	383	571	306	889	522	745	720	859
ENSG00000251774	11	6	5	14	1	2	4	5	1	3	21	1	12	4	8	8	1	0	1	12	7	6	5	4	7	9	3	16	4	4	3	11
ENSG00000225526	53	57	67	42	21	21	13	28	34	14	84	16	46	49	46	43	15	24	17	60	40	22	15	8	28	38	20	55	18	67	32	51
ENSG00000075975	2121	1629	1789	1513	1037	1131	828	1173	904	802	3445	598	994	1009	1272	1326	748	1032	1080	1287	2069	810	1706	762	919	1502	845	2415	1036	1192	1293	1321
ENSG00000274514	15	1	4	5	1	6	5	9	1	4	18	3	7	0	1	2	6	0	1	6	4	2	1	4	3	3	10	10	1	4	1	1
ENSG00000132155	4500	3312	4260	3587	3426	3329	2711	3611	2510	2200	9188	1667	1602	1611	1767	2165	1433	1721	1865	3245	5829	2321	4046	2315	2285	3605	1789	6011	1417	2009	1642	2365
ENSG00000233252	0	0	0	0	0	0	0	0	0	0	0	0	0	0	0	0	0	0	0	0	0	0	0	0	0	0	0	0	0	0	0	0
ENSG00000088726	0	0	0	0	0	0	0	0	0	0	3	0	0	0	0	0	0	0	0	0	0	0	0	0	0	0	0	0	0	0	0	0
ENSG00000213943	5	1	0	3	0	0	1	1	0	0	0	0	0	0	0	0	1	0	2	1	3	0	1	0	0	0	0	0	0	0	0	0
ENSG00000144712	776	567	720	649	441	467	296	482	34	31	95	18	18	15	11	19	159	235	215	532	619	327	433	240	33	44	13	86	9	11	22	19
ENSG00000272263	11	9	21	4	10	5	7	5	5	3	21	4	4	5	7	2	8	4	2	11	27	8	16	10	4	17	2	18	3	12	6	11
ENSG00000144713	13988	9982	9959	9000	7341	7031	5121	7371	13003	9735	34736	7834	11495	11139	12527	14643	16176	18546	17523	17426	18526	7120	12770	9874	11143	15867	6431	21706	17411	15077	20302	20474
ENSG00000207496	0	0	0	0	0	0	0	0	0	0	0	0	0	0	0	0	0	0	0	0	0	0	0	0	0	0	0	0	0	0	0	0
ENSG00000250939	0	0	0	0	0	0	0	0	0	0	0	0	0	0	0	0	0	0	0	0	0	0	0	0	0	0	0	0	0	0	0	0
ENSG00000232746	0	0	0	0	0	3	1	0	0	0	0	0	0	0	0	0	0	0	0	0	0	1	0	0	0	0	1	0	0	0	0	0
ENSG00000144711	794	887	901	902	1307	1459	1008	1503	2199	1980	11190	1776	1254	1156	1110	1584	142	260	190	524	1352	557	920	503	1708	3054	2019	7267	682	1052	656	1243
ENSG00000231362	0	0	1	0	1	4	2	2	1	4	18	1	0	1	0	0	0	0	1	0	0	0	0	5	1	3	5	11	0	0	0	0
ENSG00000132182	12263	11742	11620	11169	11508	11889	7992	11776	12144	10686	33524	7888	13914	12003	12610	15980	2225	3846	2396	6636	15256	6287	9671	5357	8556	16606	4903	24098	8719	11684	8220	12771
ENSG00000238138	0	0	0	0	0	0	0	0	0	0	0	0	0	0	0	0	0	0	0	0	0	0	0	0	0	0	0	0	0	0	0	0
ENSG00000223398	0	1	0	1	0	0	0	0	0	0	0	0	0	0	0	0	0	0	0	0	0	0	0	0	0	0	0	0	0	0	0	0
ENSG00000244502	0	0	0	6	1	0	6	0	0	0	7	1	0	1	0	2	5	3	0	1	0	1	0	0	0	2	1	3	2	3	0	0
ENSG00000163517	122	144	137	123	71	118	56	120	100	118	962	117	24	36	19	55	26	74	73	83	95	38	63	36	85	120	253	649	20	34	20	38
ENSG00000163520	835	962	792	969	6	0	2	0	345	385	746	287	0	0	1	1	60	217	82	254	0	0	0	0	161	352	91	416	0	0	0	0
ENSG00000224514	0	0	0	0	0	0	0	0	0	0	0	0	0	0	0	0	0	0	0	0	0	0	0	0	0	1	0	0	0	0	0	0
ENSG00000154764	0	0	0	0	0	0	1	2	0	0	0	0	0	0	0	0	0	0	0	0	0	1	1	0	0	0	0	0	1	0	0	0
ENSG00000233121	9	13	14	12	0	1	0	0	0	0	0	2	0	0	1	0	8	6	9	15	0	0	0	0	0	2	0	0	0	0	0	0
ENSG00000275340	6	10	9	3	1	0	0	0	0	0	0	0	0	0	0	0	2	4	2	3	0	0	0	0	0	0	0	0	0	0	0	0
ENSG00000180438	1	2	4	5	0	0	0	0	0	0	2	0	0	0	0	0	0	0	0	2	1	0	0	0	0	0	0	1	0	0	0	0
ENSG00000224752	0	0	0	1	0	0	0	0	0	0	0	0	0	0	0	0	0	0	0	1	0	0	0	0	0	0	0	0	0	0	0	0
ENSG00000163528	893	587	654	582	312	338	218	349	495	323	1225	274	622	554	741	785	843	1379	1048	1021	754	274	543	244	424	739	202	852	791	819	993	938
ENSG00000170876	1771	1301	1587	1238	996	1046	733	1110	1240	1013	4082	747	1840	1730	2086	2410	689	904	945	1282	1818	791	1365	690	978	1651	1109	2602	1918	2401	2324	2612
ENSG00000268279	25	15	21	20	10	11	14	29	15	17	68	9	14	16	23	38	8	18	8	25	33	7	17	18	14	24	9	45	19	43	13	42
ENSG00000228242	104	63	104	83	57	42	41	56	73	68	842	98	72	82	75	79	16	12	26	105	106	63	70	69	75	98	231	368	29	86	59	117
ENSG00000154767	1385	962	1271	1063	894	935	629	966	937	766	8631	1145	1065	1057	1032	1340	363	529	468	866	1334	623	941	582	814	1198	2145	4614	674	902	658	1110
ENSG00000170860	2534	1686	2016	1712	1551	1702	1338	1833	1321	959	3358	731	1183	1108	1418	1447	2680	2946	3435	3488	4203	1787	3401	2462	1287	1880	605	2392	1690	1661	2221	2092
ENSG00000255021	0	0	0	0	0	0	0	0	0	0	0	0	0	0	0	0	0	0	0	0	1	0	1	0	0	0	0	0	0	0	0	0
ENSG00000251576	0	0	0	0	0	0	0	0	0	0	0	0	0	0	1	0	0	0	0	0	0	0	0	0	0	0	0	0	0	0	0	0
ENSG00000131389	3202	2803	2501	2890	2034	2103	1450	2194	1338	1106	4626	910	3027	2484	2677	3179	706	1105	619	2025	2695	1253	1834	1187	1318	2415	1832	4171	1633	2457	1685	2622
ENSG00000144596	8	11	8	11	2	3	3	3	2	3	8	0	355	321	344	444	1	2	0	2	7	3	3	6	1	6	4	6	68	105	47	141
ENSG00000207163	0	0	0	0	0	0	0	0	0	0	0	0	3	2	1	4	0	0	0	0	0	0	0	0	0	0	0	0	0	0	0	0
ENSG00000234123	0	0	0	0	0	0	0	0	0	0	0	0	0	0	0	0	0	0	0	0	0	0	0	0	0	0	0	0	0	0	0	0
ENSG00000283392	6	1	5	2	1	1	1	0	0	0	2	0	1	1	2	3	2	0	5	4	1	0	0	1	0	0	2	1	0	0	0	0
ENSG00000235629	1	1	3	4	1	1	2	0	5	3	8	1	2	3	3	4	2	1	2	2	5	0	2	2	2	2	0	6	0	2	0	0
ENSG00000154781	1107	676	830	619	576	523	454	599	508	428	1732	315	757	671	850	936	634	622	644	580	1132	475	879	404	440	578	476	1108	604	794	838	905
ENSG00000131379	36	18	33	19	16	10	5	21	3	8	26	2	28	37	32	30	16	10	8	37	24	7	17	8	3	8	6	13	14	45	30	27
ENSG00000230172	8	1	2	1	0	0	0	0	0	0	0	0	0	4	0	1	2	2	0	0	0	0	0	0	0	0	0	0	0	0	0	1
ENSG00000260902	0	0	0	0	0	0	0	0	0	1	1	0	1	0	6	6	0	0	0	0	0	0	0	0	0	0	0	0	4	2	2	5
ENSG00000154783	703	625	711	675	4	2	3	0	199	173	277	77	105	125	111	148	48	113	111	186	4	2	3	0	53	85	38	155	174	219	171	287
ENSG00000225733	5751	4153	5159	4032	2829	2919	2086	3001	2707	2278	10764	1862	2065	2101	2138	2716	2105	2665	2890	4003	5496	2336	3919	2108	2746	3851	2284	7127	1806	2502	1847	2720
ENSG00000177463	3971	3455	3348	3198	3289	3518	2659	3573	2548	2428	10475	1884	2401	2424	2352	2839	886	1087	940	2446	5520	2530	3524	2283	2484	3965	2329	7152	1297	2447	1537	2588
ENSG00000131368	2466	1887	2068	1884	1600	1688	1302	1811	1861	1449	5738	1100	1845	1601	1705	2281	1151	1611	1462	2361	2665	1304	1826	1206	1455	2185	1079	3430	1331	2114	1539	2418
ENSG00000131381	1171	940	1010	887	613	676	450	711	469	369	1447	326	599	584	516	734	287	379	354	663	794	342	515	318	390	585	426	1128	291	432	254	445
ENSG00000228874	0	0	0	0	0	0	0	0	1	1	2	0	2	4	1	5	0	0	0	0	0	0	0	0	0	0	1	2	0	3	3	2
ENSG00000227578	0	0	0	0	0	0	0	0	0	1	11	0	4	2	6	3	0	0	0	0	0	0	0	0	0	0	0	0	0	1	0	2
ENSG00000230524	3	1	5	3	0	0	1	0	6	7	58	8	24	45	44	59	2	1	1	4	0	0	0	0	2	3	9	28	20	43	25	35
ENSG00000131375	1582	1022	1237	1007	797	839	640	938	712	680	3514	572	834	785	854	944	901	1317	1236	1319	1758	724	1103	695	826	1214	964	2314	947	1137	1215	1236
ENSG00000224660	362	236	343	243	344	391	293	432	123	125	747	94	159	170	165	214	117	135	91	426	780	337	356	354	93	173	234	373	102	252	134	261
ENSG00000131370	185	165	170	204	358	394	328	420	0	6	9	1	392	421	490	598	66	141	87	194	497	211	444	160	2	7	7	12	256	407	274	486
ENSG00000201162	0	0	0	0	0	0	0	0	0	0	2	0	0	0	0	0	0	0	0	0	0	0	0	0	0	0	0	0	0	0	0	0
ENSG00000226427	3	2	1	3	0	0	0	0	0	0	1	0	0	0	0	0	0	0	1	1	0	0	1	0	0	0	1	1	0	1	0	1
ENSG00000206562	638	426	537	444	573	600	491	658	296	272	1200	209	387	327	409	473	669	790	747	677	1276	515	899	547	287	439	296	801	508	416	523	545
ENSG00000144597	1741	1216	1466	1266	779	824	672	854	804	622	3284	524	1132	985	1406	1358	722	1024	1078	1294	1545	573	1079	510	769	1197	760	2203	1076	1238	1238	1414
ENSG00000206926	4	8	7	7	1	4	0	6	4	2	9	3	2	1	3	1	1	2	2	5	5	4	5	1	3	1	0	5	1	5	0	3
ENSG00000249786	47	26	27	32	10	9	5	11	7	8	46	5	13	20	9	11	7	6	7	21	20	12	10	6	10	10	10	13	8	13	5	25
ENSG00000206561	165	151	163	125	43	38	30	48	59	46	333	32	28	29	34	50	32	42	25	113	47	18	24	21	28	56	70	146	10	47	17	67
ENSG00000263573	1	0	2	0	0	0	0	0	0	1	0	0	0	0	0	0	0	0	0	0	0	0	0	0	0	0	0	3	0	0	0	1
ENSG00000241064	0	1	1	1	0	0	0	1	0	0	6	1	0	1	0	0	1	1	0	1	1	0	0	0	0	0	0	3	0	0	0	0
ENSG00000131373	966	608	828	663	519	533	397	537	417	371	1653	319	692	612	654	799	884	1032	1101	909	1170	510	959	535	433	583	444	1208	812	728	1063	993
ENSG00000169814	55	77	53	46	161	175	101	191	67	40	300	47	92	79	79	101	77	96	100	79	264	96	154	107	78	84	128	202	83	92	81	114
ENSG00000206560	4272	2973	3847	2809	3809	3915	3165	4499	1778	1505	6222	985	2135	1900	2103	2487	2260	2489	3452	4194	9677	4515	7974	4280	1724	2491	1828	4275	1638	2226	1923	2455
ENSG00000264354	1	0	0	1	1	3	5	3	0	2	2	0	0	0	0	0	0	0	2	1	3	5	2	3	3	0	1	2	3	1	3	1
ENSG00000279465	6	0	5	1	9	2	1	3	7	0	1	1	5	0	2	4	0	0	3	12	14	5	5	8	1	3	3	7	1	5	3	1
ENSG00000270409	9	11	6	4	6	12	5	11	9	3	19	5	9	5	10	5	7	2	1	9	20	7	10	15	3	9	4	22	6	12	6	4
ENSG00000263740	14	6	5	8	1	9	9	7	5	0	14	3	1	4	3	3	10	2	8	10	14	6	8	12	0	7	2	12	4	5	9	12
ENSG00000207815	18	7	10	6	1	3	2	0	2	5	19	1	7	4	7	7	4	1	1	15	5	5	6	1	2	9	5	6	1	5	2	10
ENSG00000224728	96	66	66	76	17	26	19	23	39	32	119	17	39	45	38	46	9	21	24	96	39	12	15	9	13	31	27	64	20	38	33	47
ENSG00000131386	4	9	1	7	2	3	1	1	0	0	2	1	1	8	4	5	2	0	0	2	3	0	2	1	1	0	0	1	2	3	2	1
ENSG00000154813	471	263	372	248	364	420	313	411	348	277	1321	216	373	391	570	487	678	757	737	701	1092	408	670	489	491	643	296	912	702	631	945	792
ENSG00000154814	600	332	527	384	436	519	368	492	1358	1041	6234	897	627	540	765	713	529	678	733	587	1292	519	964	466	1216	1642	1559	3436	1072	938	1222	1165
ENSG00000270739	11	10	8	9	2	4	2	11	9	8	24	6	5	4	11	8	1	3	0	11	16	3	10	7	9	4	7	25	1	11	5	12
ENSG00000131378	1161	898	1139	1011	1813	1823	1349	2000	1792	1722	7615	1405	2953	2470	3201	3255	375	494	505	690	2519	974	1826	832	1881	3016	2190	5358	2195	2569	3030	3032
ENSG00000271964	9	12	13	5	15	17	9	26	61	59	279	28	20	19	19	17	3	2	2	18	30	16	19	16	54	63	73	173	22	32	27	30
ENSG00000272498	12	12	14	10	4	11	8	10	73	64	388	50	10	2	6	14	4	3	4	15	15	9	8	5	68	84	104	179	3	14	21	25
ENSG00000272529	9	11	15	5	8	15	1	5	73	61	307	44	6	3	12	9	1	5	5	10	11	11	14	7	46	68	88	181	6	20	17	21
ENSG00000269391	0	0	0	0	2	0	0	0	0	1	0	0	0	0	4	7	0	0	0	0	0	0	0	0	0	0	1	0	0	1	0	1
ENSG00000233570	0	0	0	0	0	0	0	0	0	0	0	0	0	0	0	1	0	0	0	0	0	0	0	0	0	0	0	0	0	0	0	0
ENSG00000092345	0	0	1	1	0	0	0	1	0	1	3	1	0	0	2	4	0	0	0	0	2	1	7	1	1	1	2	1	2	5	9	3
ENSG00000229271	0	0	0	0	0	0	0	0	0	0	0	0	0	0	0	0	0	0	0	0	0	0	0	0	0	0	0	0	0	0	0	0
ENSG00000224039	0	0	0	0	0	0	0	0	0	0	0	0	0	0	0	0	1	0	0	0	0	0	0	0	0	0	1	2	0	0	0	0
ENSG00000154822	1052	963	900	893	270	351	257	371	2658	2356	11395	1871	472	492	543	605	400	833	550	897	519	212	411	193	2530	3655	2625	7539	480	690	562	725
ENSG00000226441	1	4	1	1	0	1	2	0	7	5	21	2	1	2	5	6	0	0	1	1	1	2	1	1	4	10	4	31	0	1	5	5
ENSG00000131374	3290	2660	3015	2509	2871	2796	2250	3148	1545	1372	6053	1082	6586	5423	5744	6494	691	1178	1044	1836	3564	1563	2936	1406	1290	1922	1211	4144	3713	4663	3751	5007
ENSG00000231780	0	0	2	1	3	0	1	1	0	1	3	2	2	1	0	4	0	0	0	1	1	2	0	1	0	1	0	2	1	2	1	5
ENSG00000227309	9	3	2	1	1	3	3	3	1	0	3	2	6	1	6	4	0	0	0	2	7	2	3	3	5	3	3	10	1	7	5	7
ENSG00000271841	3	0	0	0	2	2	1	0	0	0	0	0	0	0	0	1	0	1	0	1	0	1	1	0	0	1	0	0	1	0	3	2
ENSG00000233133	4	2	0	2	1	2	2	1	2	1	7	3	2	3	3	1	1	0	2	4	10	1	4	2	1	5	1	3	0	4	1	10
ENSG00000213383	1	0	0	0	0	0	0	0	0	0	1	0	0	0	1	0	0	0	2	0	0	0	0	0	0	0	0	0	0	0	0	0
ENSG00000214286	0	0	0	0	0	0	0	0	0	0	0	0	0	0	0	0	0	1	0	1	0	0	0	0	0	0	0	0	0	0	0	0
ENSG00000226238	6	2	4	5	8	14	8	21	0	0	1	0	0	0	0	0	2	6	3	15	28	7	12	15	0	0	0	0	0	0	0	0
ENSG00000274840	63	36	62	37	127	132	124	158	0	0	0	2	0	0	0	0	31	36	33	52	318	121	240	141	0	0	0	0	0	0	0	0
ENSG00000182568	5345	3848	4804	4017	2015	2166	1440	2136	3243	2912	23888	2334	2	9	1	3	1678	2380	1816	3722	2882	1255	2104	1099	3146	4564	9768	14958	0	9	0	2
ENSG00000272477	4	7	9	3	2	3	5	2	4	7	26	0	0	0	0	0	2	2	2	12	2	6	4	5	9	1	11	49	0	0	0	0
ENSG00000228956	640	541	594	520	167	192	131	198	320	356	1888	229	0	0	0	0	177	293	219	584	221	130	158	147	235	313	643	971	0	0	0	0
ENSG00000236570	12	7	11	5	1	1	1	1	2	2	9	6	0	0	0	0	9	5	7	14	1	4	2	0	3	2	3	9	1	0	0	0
ENSG00000251761	0	0	0	0	0	0	0	0	0	0	0	0	0	0	0	0	0	0	0	0	0	0	0	0	0	0	0	0	0	0	0	0
ENSG00000183960	75	45	58	65	80	95	58	82	13	12	58	13	1	3	0	2	24	21	11	46	71	37	49	27	10	11	18	27	0	2	1	4
ENSG00000271898	0	0	0	0	0	0	0	0	0	0	0	0	0	0	0	0	0	0	0	0	0	0	0	0	0	0	0	0	0	0	0	0
ENSG00000163576	9	7	6	5	8	5	3	8	22	27	107	12	10	10	3	9	8	6	14	10	14	1	17	13	9	25	33	46	3	3	4	2
ENSG00000232953	1	1	0	3	1	1	0	5	2	1	6	4	1	1	1	4	1	1	1	3	3	2	0	1	0	0	5	8	0	3	0	1
ENSG00000144566	1482	924	1195	939	1062	1057	802	1116	1411	1162	7032	1000	905	856	1034	1075	691	898	989	914	2099	845	1610	755	1562	2124	2030	4526	878	1053	1044	1126
ENSG00000183977	38	24	15	25	22	21	23	36	28	19	154	16	9	16	13	19	17	8	3	15	38	22	18	15	34	44	35	89	7	15	15	17
ENSG00000201545	2	0	0	1	0	0	0	0	0	0	2	0	0	0	0	0	3	0	0	0	1	0	0	3	1	0	2	3	0	0	0	0
ENSG00000230697	2	0	2	3	1	0	0	3	3	5	11	0	0	0	0	1	1	0	1	1	2	0	0	0	3	0	4	3	0	0	0	0
ENSG00000229468	0	0	0	0	1	0	1	0	0	0	5	0	0	0	0	0	0	2	0	0	0	0	0	0	0	1	0	1	0	0	0	0
ENSG00000114166	600	427	596	405	746	796	611	819	4238	3592	31244	3680	102	144	135	186	186	139	206	284	1393	567	996	587	5558	7240	9358	19868	81	172	123	217
ENSG00000266745	0	0	1	0	1	1	0	0	2	3	15	1	0	0	0	1	0	0	0	0	0	1	0	0	6	5	6	20	0	0	0	0
ENSG00000129810	1327	945	1225	1056	815	834	647	831	934	732	2761	548	667	565	761	733	882	1044	991	1057	1577	562	1126	595	938	1298	517	2391	673	711	765	917
ENSG00000231304	18	7	12	11	8	8	4	13	27	15	164	22	10	13	6	11	1	3	2	20	10	3	11	5	27	34	75	117	6	14	7	21
ENSG00000199577	0	0	0	0	0	0	0	0	0	0	1	0	0	0	0	1	0	0	0	0	0	0	0	0	1	3	2	2	0	0	0	0
ENSG00000261734	2	0	0	0	0	2	0	0	0	0	0	0	0	4	2	1	1	0	0	0	0	0	0	1	0	2	1	5	0	3	0	0
ENSG00000206807	0	0	0	0	0	0	0	0	0	0	0	0	0	0	0	0	0	0	0	0	0	0	0	0	0	0	0	0	0	0	0	1
ENSG00000282987	0	0	0	0	0	0	0	0	0	0	2	0	0	0	0	0	0	0	0	0	0	0	0	0	0	0	0	0	0	0	0	0
ENSG00000236380	0	0	0	0	0	0	0	0	0	0	0	0	0	0	0	0	0	0	0	0	0	0	0	0	0	0	0	0	0	0	0	0
ENSG00000151789	0	3	4	1	0	0	0	1	0	0	1	0	0	0	0	0	3	6	7	2	0	0	0	0	0	0	1	0	0	0	6	0
ENSG00000225542	0	2	0	0	0	0	0	0	0	0	0	0	0	0	0	0	0	0	0	0	0	0	0	0	0	0	0	0	0	0	0	2
ENSG00000223351	0	0	0	0	0	0	0	0	0	0	0	0	0	0	0	0	0	0	0	0	0	0	0	0	0	0	0	0	0	0	0	0
ENSG00000132967	236	168	242	198	383	373	380	402	138	94	313	70	156	154	203	211	413	407	188	317	952	464	573	429	122	207	52	234	210	209	217	282
ENSG00000234723	0	0	0	0	0	0	1	0	0	0	0	0	0	0	0	0	0	0	0	0	0	0	0	0	0	0	0	0	0	0	0	0
ENSG00000271235	0	0	0	0	0	0	0	0	0	0	0	0	0	0	0	0	0	0	0	0	0	0	0	0	0	0	0	0	0	0	0	0
ENSG00000226183	0	0	0	0	0	0	0	0	0	0	0	0	0	0	0	0	0	0	0	0	0	0	0	0	0	0	0	0	0	0	0	0
ENSG00000231915	0	0	0	0	0	0	0	0	0	0	0	0	0	0	0	0	0	0	0	0	0	0	0	0	0	0	1	0	0	0	0	0
ENSG00000215016	0	0	0	0	0	0	0	0	1	0	2	0	0	0	0	0	0	0	0	0	0	0	0	0	0	0	1	0	0	0	0	0
ENSG00000233153	0	0	0	0	0	0	0	0	0	0	0	0	0	0	0	0	1	1	0	0	0	0	0	0	0	0	0	0	0	0	0	0
ENSG00000182247	562	542	505	569	484	546	367	595	14	37	68	20	290	297	294	344	207	492	327	539	683	299	578	286	7	34	14	67	326	477	395	508
ENSG00000206728	3	1	2	2	3	4	0	5	0	0	1	0	0	0	1	0	0	5	0	5	4	1	5	3	0	0	0	0	0	1	1	5
ENSG00000228171	1	1	2	2	1	2	4	3	0	1	0	0	2	0	2	0	0	1	0	0	1	0	1	1	0	0	0	2	0	1	1	1
ENSG00000252562	0	2	1	0	0	0	2	0	0	0	0	0	0	0	0	0	1	0	0	1	1	0	0	1	0	0	0	0	0	0	0	0
ENSG00000212269	0	0	0	0	0	0	0	0	0	0	0	0	0	0	0	0	0	0	0	2	0	1	0	0	0	0	0	0	0	0	0	0
ENSG00000223791	3	8	1	4	18	25	11	13	5	3	19	4	28	17	24	21	3	3	0	15	34	12	22	14	2	8	3	10	23	18	16	38
ENSG00000170142	2847	2051	2399	2234	4776	4720	3819	5102	2188	1737	6552	1346	3397	3148	3866	3957	2870	4488	3526	3586	12181	4312	8705	4735	1979	2835	1542	4162	4105	4164	5012	4946
ENSG00000231395	5	2	7	7	9	8	8	12	17	5	16	6	5	9	5	9	0	0	1	15	23	15	6	10	10	9	9	22	6	10	10	17
ENSG00000197885	156	140	161	135	129	131	101	163	123	113	520	71	100	120	122	161	178	269	150	222	253	112	151	87	162	212	160	387	128	188	135	185
ENSG00000174748	17650	12602	12048	11833	14450	14093	9904	14842	21501	16607	63533	13226	18081	16712	18188	21712	18990	28410	19011	16115	31931	11721	21381	11788	18953	26438	12182	37288	23278	20912	26584	26261
ENSG00000174738	637	544	537	532	324	315	283	333	658	544	3043	435	652	633	505	665	357	782	520	714	839	311	606	328	827	1180	918	2317	578	713	592	767
ENSG00000224074	0	0	1	1	0	0	0	0	0	0	0	0	0	0	0	0	0	0	0	0	1	0	0	0	0	0	1	0	0	0	1	0
ENSG00000242109	0	0	0	0	0	0	0	0	0	0	0	0	0	0	0	0	0	0	0	0	0	0	0	0	0	0	0	0	0	0	0	0
ENSG00000151090	0	1	1	0	0	0	0	0	0	0	0	0	0	0	0	0	1	0	2	1	0	0	1	0	0	0	0	2	0	0	0	0
ENSG00000229756	0	0	0	0	0	0	0	0	0	0	0	0	0	0	0	0	0	0	0	0	0	0	0	0	0	0	0	0	0	0	0	0
ENSG00000224822	0	0	0	0	0	0	0	0	0	0	0	0	0	0	0	0	0	0	0	0	0	0	0	0	0	0	0	0	0	0	0	0
ENSG00000228791	0	0	0	0	0	0	0	0	0	0	1	0	0	0	0	0	0	0	0	0	0	0	0	0	0	0	0	0	0	0	0	0
ENSG00000272554	0	0	0	0	0	0	0	0	0	0	0	0	0	0	0	0	0	0	0	0	0	0	0	0	0	0	0	0	0	0	0	0
ENSG00000265028	0	0	0	0	0	0	0	0	0	0	0	0	0	0	0	0	0	0	0	0	0	0	0	0	0	0	0	0	0	0	0	0
ENSG00000233544	0	0	0	0	0	0	0	0	0	0	0	0	0	0	0	1	0	0	0	0	0	0	0	0	0	0	0	0	0	0	0	0
ENSG00000237838	0	0	0	0	0	0	0	0	1	1	10	2	0	0	0	0	0	0	0	0	0	0	0	0	0	0	4	2	0	0	0	0
ENSG00000244404	0	0	0	0	0	0	0	0	0	0	0	0	0	0	0	0	0	0	0	0	0	0	0	0	0	0	0	0	0	0	0	0
ENSG00000077092	9	5	7	7	8	11	13	27	8	12	72	11	8	21	7	26	4	5	3	9	29	14	14	20	13	28	12	71	3	9	13	19
ENSG00000213877	0	0	0	0	0	0	0	0	0	0	0	0	0	0	0	0	0	0	0	0	0	0	0	0	0	0	0	0	0	0	0	0
ENSG00000229421	0	0	0	0	0	0	0	0	0	0	0	0	0	0	0	0	0	0	0	0	0	0	0	0	0	0	0	0	0	0	0	0
ENSG00000272166	0	0	0	0	0	1	0	0	0	0	0	0	0	0	0	0	0	0	0	0	0	0	0	0	0	0	0	0	0	0	0	0
ENSG00000077097	15645	10602	12577	10580	25663	25335	20211	27671	8535	6789	28516	4957	15618	13343	14427	16514	6384	9342	8460	8957	46274	17793	35283	16418	8242	12147	6142	18902	10663	12959	12691	14212
ENSG00000275227	14	9	14	9	23	24	18	40	12	16	57	3	18	12	16	19	2	4	2	13	42	30	26	28	11	17	13	19	4	22	6	9
ENSG00000238225	0	0	0	0	0	0	0	0	1	2	1	0	0	0	1	1	0	0	0	2	0	0	0	0	0	1	0	6	0	0	0	0
ENSG00000151092	2897	1928	1991	1899	2634	2357	1760	2910	1230	1010	4191	667	1860	1563	1609	1996	1468	1992	1433	1951	3033	1271	2095	1274	1075	1456	1025	2656	1551	1615	1795	1844
ENSG00000213872	58	35	24	24	43	31	24	51	16	13	78	7	19	9	16	19	9	6	1	32	40	17	16	3	15	13	29	64	6	11	11	13
ENSG00000213871	47	25	20	26	5	8	4	11	6	5	18	4	6	9	12	2	2	6	1	13	14	8	12	12	11	12	8	22	2	7	4	5
ENSG00000151093	602	454	478	487	349	343	255	392	276	219	834	147	248	258	228	317	593	800	610	576	634	278	452	325	199	346	197	481	291	317	325	385
ENSG00000230891	1	1	1	0	0	1	0	1	1	0	5	1	0	1	0	0	0	0	0	4	2	0	2	4	0	3	1	8	0	0	0	0
ENSG00000234158	0	0	0	0	0	0	0	5	3	0	3	0	0	0	0	0	0	0	0	0	1	0	0	2	0	1	1	0	0	0	0	0
ENSG00000237603	1	0	0	0	0	0	0	2	0	3	16	1	0	0	1	0	0	0	0	1	1	1	0	1	3	0	1	7	0	0	0	0
ENSG00000230465	0	0	0	1	0	0	0	0	0	0	0	0	0	0	0	0	0	0	0	0	0	0	0	0	0	0	0	0	0	0	0	0
ENSG00000225386	0	0	0	0	0	0	0	1	0	0	0	0	0	0	0	0	0	0	0	0	0	0	0	0	0	0	0	0	0	0	0	0
ENSG00000179796	0	0	0	0	0	0	0	0	0	1	0	0	0	0	0	0	0	0	0	0	0	1	0	0	0	0	0	0	0	0	0	0
ENSG00000234165	0	0	0	0	0	0	0	0	1	0	1	0	0	0	0	0	0	0	0	0	0	0	0	0	0	0	0	0	0	0	0	0
ENSG00000163491	54	32	55	45	23	17	17	32	11	17	94	7	6	5	5	11	20	25	51	42	41	31	23	28	17	25	14	46	2	10	6	13
ENSG00000224237	1	0	0	0	0	0	0	1	0	0	2	0	0	0	0	0	0	0	0	0	0	3	0	0	0	0	0	2	0	0	0	0
ENSG00000207251	0	0	0	0	0	0	0	0	0	0	0	0	0	0	0	0	0	0	0	0	0	0	0	0	0	1	0	3	0	0	0	0
ENSG00000033867	3197	2377	2294	2148	1943	2123	1342	2121	1956	1737	8419	1275	2756	2448	3126	2990	698	1161	868	1526	2976	1398	1971	1374	2624	3653	2078	5954	2243	2873	3128	3418
ENSG00000227523	6	6	4	3	4	0	2	5	7	4	11	3	4	0	3	6	0	0	5	3	1	2	3	3	5	5	6	31	0	4	7	8
ENSG00000230807	0	2	0	0	1	0	0	0	2	0	2	0	0	1	1	2	0	0	0	0	0	0	1	0	3	1	1	3	4	2	0	0
ENSG00000201033	0	0	0	0	0	0	0	0	0	0	0	0	0	0	0	0	0	0	0	0	0	0	0	0	0	3	0	0	0	0	0	0
ENSG00000232167	0	0	0	0	0	0	0	0	0	0	0	0	0	0	0	0	0	0	0	0	0	0	0	0	0	0	0	0	0	0	0	0
ENSG00000238912	0	0	0	0	0	0	0	0	0	0	0	0	0	0	0	0	0	0	0	0	0	0	0	0	0	0	0	0	0	0	0	0
ENSG00000223968	0	0	0	0	0	0	0	0	0	0	0	0	0	0	0	0	0	0	0	0	0	0	0	0	0	0	0	0	0	0	0	0
ENSG00000213846	0	2	3	2	33	37	30	41	25	15	112	16	20	31	28	43	4	2	2	4	63	31	30	19	24	15	32	44	11	17	19	35
ENSG00000271943	0	0	0	0	3	1	2	5	3	1	4	0	2	4	4	4	0	0	0	0	4	0	2	1	4	0	1	2	1	1	0	5
ENSG00000232766	0	0	0	0	0	0	0	0	0	0	0	0	0	0	0	0	0	0	0	0	0	0	0	0	0	1	3	1	0	0	0	0
ENSG00000272282	0	1	1	2	0	1	0	0	267	228	717	141	1	0	3	0	1	3	0	2	1	0	0	1	168	227	182	406	4	1	8	3
ENSG00000163508	98	118	97	105	110	144	96	132	2578	2581	8953	1810	389	403	810	604	25	78	42	87	365	161	347	132	3106	4824	2409	6861	726	874	1145	1060
ENSG00000225548	0	0	0	0	0	0	0	1	2	0	2	0	6	5	5	4	0	0	0	2	0	0	0	0	0	1	2	1	2	2	1	7
ENSG00000229243	0	0	0	0	0	0	0	1	0	0	0	0	2	5	0	1	0	0	0	0	0	0	0	0	1	1	0	0	0	0	0	0
ENSG00000235493	0	0	0	0	0	0	0	0	0	1	1	0	4	4	2	0	0	0	0	0	0	0	0	0	0	0	0	0	0	0	3	4
ENSG00000187118	752	565	608	538	322	393	280	420	1595	1242	3693	758	753	700	771	890	611	706	578	681	745	274	505	333	660	883	329	1259	659	688	676	922
ENSG00000163512	954	682	776	725	600	589	421	621	974	849	3132	593	457	451	544	607	573	715	650	914	1071	417	747	455	806	1156	698	2037	562	642	627	700
ENSG00000206559	22	19	32	27	17	22	7	16	7	12	51	12	8	6	11	12	18	26	13	24	23	7	11	7	8	10	19	29	7	8	10	11
ENSG00000283563	0	0	0	0	0	0	0	0	0	0	0	0	0	2	0	0	0	0	0	0	0	0	0	0	0	0	0	1	0	3	0	3
ENSG00000234048	1	0	0	0	0	0	0	0	0	0	0	0	0	0	0	0	0	0	0	0	1	0	0	0	0	0	0	2	0	0	0	0
ENSG00000230480	0	1	0	0	0	0	0	0	0	0	2	0	0	0	0	0	0	0	0	0	0	0	0	0	0	0	0	0	0	0	0	0
ENSG00000144642	0	3	0	1	2	4	0	1	20	20	54	10	64	123	54	132	1	1	0	1	4	1	0	2	7	8	12	14	7	40	12	31
ENSG00000215004	0	0	0	0	0	0	0	1	0	0	0	0	0	0	0	0	0	0	0	0	0	0	0	0	0	0	0	0	0	0	0	0
ENSG00000235904	0	0	0	0	0	0	0	0	0	1	4	0	3	0	0	2	0	0	0	0	0	0	0	0	0	1	2	2	0	3	0	1
ENSG00000226216	0	0	0	0	0	0	0	0	0	0	0	0	0	3	0	0	0	0	0	0	0	0	0	0	1	1	3	0	0	0	0	0
ENSG00000203506	0	0	0	1	0	0	0	0	3	0	2	0	8	4	3	4	0	0	0	0	0	0	0	0	0	0	1	1	1	6	2	1
ENSG00000227795	0	0	0	0	0	0	0	0	0	0	0	0	0	0	0	0	0	0	0	0	0	0	0	0	0	0	0	0	0	0	0	0
ENSG00000235593	0	0	0	0	0	0	0	0	0	0	0	0	1	0	0	0	0	0	0	0	0	0	0	0	0	0	0	0	0	6	0	0
ENSG00000199927	0	0	0	0	0	0	0	0	0	0	0	0	0	0	0	0	0	0	0	0	0	0	0	0	0	0	0	0	0	0	0	0
ENSG00000281710	0	0	0	0	0	0	0	0	0	0	0	0	0	0	0	0	0	0	0	0	0	0	0	0	0	0	0	0	0	0	0	0
ENSG00000227260	0	0	0	0	0	0	0	0	0	0	4	0	0	0	0	0	0	0	0	0	1	0	0	0	0	0	2	0	0	0	0	0
ENSG00000227549	0	0	0	0	0	0	0	0	0	0	0	0	0	0	0	0	0	0	0	0	0	0	0	0	0	0	0	0	0	0	0	0
ENSG00000163513	670	494	635	457	2010	2161	1580	2307	1588	1578	6389	1141	692	798	621	931	193	292	439	422	4164	1726	3219	1590	1317	2055	1996	4183	458	714	454	697
ENSG00000224618	8	3	2	0	6	4	0	12	7	6	22	4	1	5	3	2	1	0	4	4	22	17	8	15	9	7	6	19	0	3	5	4
ENSG00000261468	5	6	8	4	21	31	6	25	14	24	41	12	4	7	2	2	3	3	2	5	36	12	18	13	19	22	12	42	0	4	3	12
ENSG00000144644	4	2	4	2	11	19	9	16	1	0	9	1	0	0	0	0	1	4	0	3	13	9	8	4	0	0	3	2	0	1	0	0
ENSG00000265376	0	0	0	0	2	1	1	5	0	0	0	0	0	0	0	0	0	0	0	0	4	1	3	3	0	0	0	0	0	0	0	0
ENSG00000213854	1	1	1	1	20	34	11	27	0	0	0	0	0	0	0	0	0	0	1	0	37	20	29	33	0	0	0	0	0	0	0	0
ENSG00000230111	0	0	0	1	8	5	9	11	0	0	0	0	0	0	0	0	0	0	0	0	18	12	10	15	0	0	1	2	0	0	0	0
ENSG00000227339	1	0	0	1	0	0	0	0	0	0	1	0	0	0	1	1	0	0	0	0	1	1	0	0	0	2	0	0	0	0	0	0
ENSG00000163527	27300	21141	19617	18800	55868	55947	37956	57528	4148	3349	14430	2528	3528	3285	3637	4043	10317	18754	15389	22414	111475	45952	77946	41303	4090	6137	3381	10027	3229	3965	3980	4345
ENSG00000144645	6	7	15	4	122	92	93	120	0	4	16	4	6	5	5	12	8	7	12	30	261	159	139	170	13	9	2	11	4	10	12	13
ENSG00000232490	0	0	0	0	0	0	0	0	0	0	1	2	0	1	1	0	0	0	0	0	0	0	0	0	0	0	2	0	1	0	0	0
ENSG00000223965	0	0	0	0	0	0	0	0	0	0	1	0	0	0	0	0	0	0	0	0	0	0	0	0	0	0	0	1	0	1	1	0
ENSG00000197385	0	0	0	0	0	0	0	0	0	0	0	0	0	0	0	0	0	0	0	1	0	0	0	0	0	0	0	0	0	0	0	0
ENSG00000235670	0	0	0	0	0	0	0	0	0	0	1	0	0	0	0	0	0	0	0	0	0	0	0	0	0	0	0	0	0	0	0	0
ENSG00000201701	0	0	0	0	0	0	0	0	0	0	0	0	0	0	0	0	0	0	0	0	0	0	0	0	0	0	1	0	0	0	0	0
ENSG00000252398	0	0	0	0	0	0	0	0	0	0	0	0	0	0	0	0	0	0	0	0	0	0	0	0	0	0	0	0	0	0	0	0
ENSG00000251590	0	0	0	0	0	0	0	0	0	0	0	0	0	0	0	0	0	0	0	0	0	0	0	0	0	0	0	0	0	0	0	0
ENSG00000152642	774	664	717	735	328	424	268	382	356	315	1462	279	1167	1012	1202	1450	350	717	559	943	683	240	470	276	523	795	385	1304	892	1191	931	1277
ENSG00000236732	5	4	12	5	3	2	3	1	3	2	16	2	15	9	1	7	1	0	2	7	3	3	3	0	12	11	9	20	1	14	3	14
ENSG00000237433	0	1	0	0	0	0	0	0	0	0	0	0	0	0	0	1	1	1	0	0	0	0	0	1	0	0	0	0	0	0	0	2
ENSG00000261572	0	0	0	0	2	4	1	3	4	0	2	0	0	0	0	0	0	1	0	0	1	0	1	1	0	0	0	2	2	1	1	0
ENSG00000170293	102	124	79	126	123	151	64	137	53	35	122	37	57	67	50	78	43	91	42	100	149	62	100	63	60	66	22	101	36	53	35	55
ENSG00000270377	1	0	3	1	0	1	2	3	0	0	2	0	0	0	1	0	0	2	0	2	0	1	0	0	0	0	0	1	0	0	0	1
ENSG00000234737	2	2	1	4	2	1	3	7	1	3	5	2	1	2	0	2	0	0	0	3	1	4	3	0	2	4	1	10	1	2	1	0
ENSG00000153551	1	4	2	4	4	6	2	4	163	123	615	162	75	55	78	83	0	1	0	1	6	0	4	0	145	225	114	482	73	91	91	97
ENSG00000091317	2711	1766	1998	1810	4233	4271	3412	4612	1469	1302	6756	1056	2790	2616	3174	3355	1850	2572	2377	2508	10648	4239	7664	4026	2372	3285	1691	5205	3310	3759	3981	4459
ENSG00000276147	0	0	0	0	0	0	0	0	0	0	0	0	2	0	0	0	0	1	1	0	0	2	0	0	0	1	0	0	0	0	0	0
ENSG00000213849	0	0	0	1	0	3	0	0	0	0	2	0	1	0	1	0	0	0	0	0	1	0	0	0	0	0	0	0	0	0	4	0
ENSG00000144635	2024	1465	1593	1487	1591	1589	1230	1738	907	609	3233	538	1189	1136	1253	1363	1625	2450	2161	2046	3669	1353	2871	1402	1023	1477	662	2304	1368	1587	1600	1776
ENSG00000226955	0	0	0	0	0	0	0	0	0	0	0	0	0	0	0	0	0	0	0	0	0	0	0	0	0	0	0	0	0	0	0	0
ENSG00000231552	0	0	0	0	0	0	0	0	0	0	0	0	0	0	0	0	0	0	0	0	0	0	0	0	0	0	0	0	0	0	0	0
ENSG00000237676	0	1	1	0	0	0	0	0	0	0	0	0	0	0	0	0	0	0	1	0	1	0	0	0	0	0	0	0	0	0	0	0
ENSG00000182973	1666	1021	1377	1080	1018	1081	860	1217	872	749	2963	642	1237	1067	1279	1418	1429	2172	1994	1522	2528	933	1949	845	929	1290	673	2176	1442	1462	1735	1700
ENSG00000251224	2	2	2	1	0	3	4	0	3	1	10	0	0	0	1	1	1	1	0	3	1	1	4	3	0	1	0	9	1	0	2	1
ENSG00000213842	15	15	8	13	7	8	6	6	6	8	25	4	16	7	4	6	4	1	5	17	13	8	4	7	4	17	5	34	2	6	13	7
ENSG00000227337	1	0	0	0	1	1	0	1	0	1	3	0	0	0	1	0	0	1	0	1	2	0	0	2	0	0	0	2	0	0	0	0

ENSG00000206557	43	56	69	58	986	944	783	928	2	5	9	5	47	73	67	93	8	42	31	64	2057	808	1300	757	0	8	1	14	39	63	44	91
ENSG00000183813	145	163	139	130	128	134	103	149	0	0	0	0	628	695	791	949	94	143	161	196	304	116	219	84	0	0	3	0	1154	1247	1298	1261
ENSG00000170266	871	736	779	667	1813	1856	1167	1864	482	463	2050	347	1246	1199	1277	1658	339	471	427	493	2374	959	1711	787	470	762	610	1613	1257	1730	1570	1890
ENSG00000223899	2	2	5	3	0	1	1	5	9	4	33	3	7	7	8	10	3	1	3	4	4	1	2	2	12	10	12	33	4	31	12	29
ENSG00000235424	1	1	0	0	0	0	0	0	2	0	2	0	2	1	0	3	1	0	0	1	2	0	0	1	0	0	1	1	0	1	1	0
ENSG00000188167	204	128	153	135	100	148	98	141	100	99	439	78	120	128	148	160	29	30	31	126	182	90	154	82	125	179	132	361	127	196	197	235
ENSG00000275090	0	2	0	2	0	0	0	0	0	0	0	0	0	0	0	0	0	0	0	0	0	0	0	0	0	0	0	0	0	0	0	0
ENSG00000170275	1611	1499	1297	1483	3523	3944	2740	4298	1047	915	4375	793	1412	1416	1243	1696	523	1068	743	1096	5656	2404	4276	1853	1040	1603	1050	3331	978	1259	1082	1418
ENSG00000272149	0	0	0	0	0	0	0	0	0	0	0	0	0	0	0	0	0	0	0	0	0	0	0	0	0	0	0	0	0	0	0	0
ENSG00000173705	1	5	1	4	13	13	10	14	3	0	11	0	1	0	0	3	2	3	2	5	28	9	16	13	4	2	2	4	0	6	1	7
ENSG00000230119	0	0	0	0	0	0	0	0	0	0	0	0	0	0	0	0	0	0	0	0	0	0	0	0	0	0	0	0	0	0	0	0
ENSG00000153558	128	129	125	125	43	48	30	60	44	46	179	22	190	165	109	190	52	94	66	132	105	47	25	58	49	46	56	137	56	163	87	174
ENSG00000153560	3481	2540	2976	2682	3171	3297	2571	3451	2271	1940	9394	1498	2336	2186	2485	2844	1130	1433	1279	2469	5697	2467	3835	2235	2381	3506	2076	5681	2007	2729	2110	3018
ENSG00000252700	1	1	0	2	0	2	0	1	3	5	1	3	2	3	1	1	1	0	0	0	2	1	0	1	0	3	0	5	0	0	2	2
ENSG00000163539	2733	2000	2410	2062	3002	2998	2289	3296	1925	1658	7009	1179	1769	1664	1823	2073	1018	1532	1453	2267	5703	2433	4172	2559	1986	2977	1502	4970	1327	2082	1884	2295
ENSG00000238008	4	6	2	1	1	3	1	0	4	1	5	3	1	2	1	4	0	0	0	3	2	1	3	3	2	4	2	7	2	3	0	3
ENSG00000228112	0	0	0	0	0	0	0	0	0	0	0	0	0	0	0	0	0	0	0	0	0	0	0	0	0	0	1	0	0	0	0	0
ENSG00000227707	0	0	0	0	0	0	0	0	0	0	0	0	0	0	0	0	0	0	0	0	0	0	0	0	0	0	0	0	0	0	0	0
ENSG00000271324	1	1	2	2	4	3	3	1	4	1	21	3	1	1	1	0	0	0	0	1	1	1	0	0	7	2	0	8	1	3	1	3
ENSG00000271643	13	6	13	4	18	11	4	16	32	25	98	13	12	12	11	12	2	0	4	10	28	12	27	14	19	22	26	56	10	14	24	14
ENSG00000271020	5	3	5	6	5	7	10	13	5	9	78	12	10	4	11	7	0	2	5	6	13	4	13	5	5	24	13	38	1	4	3	10
ENSG00000170248	3704	2538	3109	2507	4202	4395	3269	4704	3131	2706	12093	2091	4246	3730	4167	4895	2748	3015	2811	3147	9423	3633	6595	3676	2834	4191	3132	7682	3611	4184	4210	4866
ENSG00000226320	0	0	0	0	0	0	0	0	0	0	0	0	0	0	0	0	0	0	0	0	0	0	0	0	0	0	0	0	0	0	0	0
ENSG00000236452	1	0	0	3	0	0	0	0	0	0	0	0	0	0	0	0	1	0	0	3	0	2	1	0	0	0	0	0	0	0	0	0
ENSG00000227498	0	0	0	0	0	0	0	0	0	0	0	0	0	0	0	0	0	0	0	0	0	0	0	0	0	0	0	0	0	0	0	0
ENSG00000235534	0	0	0	0	0	0	0	0	0	0	0	0	0	0	0	0	0	0	0	0	0	0	0	0	0	0	0	0	0	0	0	0
ENSG00000228767	0	0	0	0	0	0	0	0	0	0	0	0	0	0	0	0	0	0	0	0	0	0	0	0	0	0	0	0	0	0	0	0
ENSG00000212442	0	0	0	0	0	0	0	0	0	0	0	0	0	0	0	0	0	0	0	0	0	0	0	0	0	0	0	0	0	0	0	0
ENSG00000172995	2334	1784	1791	1781	8	6	2	1	1	0	0	0	0	0	0	0	612	544	432	791	4	1	2	1	0	1	0	1	0	0	0	0
ENSG00000230830	97	95	94	104	0	0	0	0	0	0	0	0	0	0	0	0	29	19	12	48	0	0	0	0	0	0	0	0	0	0	0	0
ENSG00000207625	1	0	0	0	0	0	0	0	0	0	1	0	0	0	0	0	0	0	1	0	0	0	0	0	0	0	0	0	0	0	0	0
ENSG00000226489	0	0	0	0	0	0	0	0	0	0	0	0	0	0	0	0	0	0	0	0	0	0	0	0	0	0	0	0	0	0	0	0
ENSG00000236158	0	0	0	0	0	0	0	0	0	0	0	0	0	0	0	0	0	0	0	0	0	0	0	0	0	0	0	0	0	0	0	0
ENSG00000144681	2	5	1	6	605	610	505	700	122	94	319	87	2	0	0	0	1	1	0	4	877	343	850	309	109	144	57	205	0	0	0	0
ENSG00000231382	0	0	0	1	0	0	0	0	0	0	0	0	0	0	0	0	0	0	0	0	0	0	0	0	0	0	1	0	0	0	0	0
ENSG00000201315	0	0	0	0	0	0	0	0	0	0	0	0	0	0	0	0	0	0	0	0	0	0	0	0	0	0	0	0	0	0	0	0
ENSG00000163673	1	1	1	4	0	0	0	0	0	0	0	0	0	0	1	0	0	0	0	0	0	0	0	2	0	0	0	0	0	0	1	1
ENSG00000230067	1	0	1	0	1	1	1	0	0	0	0	0	0	0	0	1	0	0	0	1	0	0	0	0	0	0	0	2	0	0	0	1
ENSG00000279727	0	0	0	0	5	4	4	1	0	4	6	0	2	2	1	2	0	0	0	0	0	0	2	0	1	1	0	3	2	3	1	2
ENSG00000281100	0	1	3	0	4	11	5	11	0	0	19	5	1	4	6	7	2	0	0	0	10	5	5	0	3	8	8	15	2	3	3	8
ENSG00000168016	131	48	178	47	986	1105	739	1239	479	503	4652	540	692	685	595	866	25	10	36	33	860	370	654	314	404	675	1186	2479	407	613	505	657
ENSG00000234073	0	0	0	0	2	0	0	3	0	1	14	1	0	2	3	2	0	0	0	0	0	0	0	0	0	1	1	10	1	3	1	4
ENSG00000210181	2	0	0	0	1	0	1	2	0	1	3	0	2	0	0	0	0	0	0	0	0	0	0	1	0	1	0	1	0	0	1	0
ENSG00000272334	6	2	5	2	4	1	6	12	7	5	17	0	5	8	1	10	0	2	3	5	12	1	2	5	0	2	1	6	3	2	6	3
ENSG00000236165	8	13	6	7	17	7	4	10	5	4	29	4	21	9	3	16	5	2	4	3	5	4	4	4	4	3	5	8	1	13	6	7
ENSG00000178567	3672	2484	3054	2527	1901	1905	1403	2033	1991	1654	8790	1405	1312	1245	1252	1639	1157	1070	1314	2174	2923	1321	2037	1379	1820	2304	2084	5223	759	1356	1040	1638
ENSG00000076242	2734	1875	2117	1767	2292	2281	1754	2424	1467	1193	4273	898	1605	1411	1422	1826	1923	2684	2341	1746	4974	1923	3720	1830	1355	1820	729	3241	1338	1589	1538	1769
ENSG00000224858	20	23	15	29	13	10	15	9	10	4	52	8	17	12	11	14	8	26	6	18	30	9	9	14	10	9	5	32	9	10	6	13
ENSG00000093167	1871	1423	1544	1468	1544	1644	1232	1789	784	643	2994	492	1046	954	1153	1297	842	1275	950	1438	3279	1540	2618	1379	777	1164	670	2275	939	1144	1110	1388
ENSG00000206645	7	1	6	3	3	3	2	4	1	4	7	0	2	3	2	1	1	1	0	5	11	10	8	3	1	2	3	6	2	1	6	0
ENSG00000225558	2	0	3	0	0	1	1	1	1	0	2	2	0	1	1	0	1	0	0	2	1	1	2	6	0	0	1	2	0	0	1	0
ENSG00000199594	0	1	0	0	0	0	3	0	0	1	0	0	0	0	0	0	3	0	0	1	1	0	2	1	0	2	0	3	0	1	2	1
ENSG00000224080	15	4	13	5	20	9	13	15	7	2	30	4	5	5	3	6	8	11	7	8	39	20	43	27	7	17	1	14	3	3	10	3
ENSG00000271993	36	26	26	25	7	24	19	15	13	16	80	13	14	15	11	15	7	11	12	15	12	12	5	8	11	20	16	40	9	7	3	10
ENSG00000231449	2	1	2	1	2	0	0	0	0	0	2	1	0	0	0	1	0	0	0	1	0	0	0	0	0	0	0	2	1	0	0	0
ENSG00000271653	3	0	5	0	1	0	0	0	0	0	0	1	0	0	0	0	0	0	1	0	0	0	0	0	0	0	0	1	0	0	0	0
ENSG00000270194	24	15	20	10	12	16	16	11	16	15	35	3	19	15	12	15	10	5	7	27	31	13	8	11	8	19	7	35	10	14	19	18
ENSG00000144674	3519	2499	2833	2518	2517	2829	2239	3194	1580	1256	5967	910	1696	1451	1642	1936	1462	1607	1568	2569	5369	2356	3595	2415	1514	2218	1645	4644	1113	1626	1547	1957
ENSG00000230409	13	9	17	15	16	15	16	25	11	6	33	7	11	8	8	13	9	17	13	15	36	26	25	17	6	10	13	24	9	8	12	10
ENSG00000198590	44	35	32	51	28	15	18	29	11	5	38	9	23	21	24	36	6	12	9	37	44	29	19	12	8	10	5	24	4	30	15	32
ENSG00000144668	11	5	18	14	23	13	23	18	13	41	82	17	2	2	3	8	1	4	4	10	25	10	20	13	13	26	19	72	2	11	3	9
ENSG00000239105	0	0	0	0	0	0	0	0	0	0	0	0	0	0	0	0	0	0	0	0	0	0	0	0	0	0	0	0	0	0	0	0
ENSG00000235257	47	56	67	41	67	96	60	110	0	3	12	1	19	25	9	24	23	50	23	59	107	54	58	32	1	0	2	3	12	11	7	7
ENSG00000235482	0	0	0	0	2	0	0	0	0	0	0	0	0	0	0	0	2	1	0	0	0	1	1	0	0	1	0	0	0	0	0	0
ENSG00000236411	1	1	2	1	7	5	3	4	2	0	0	2	0	1	0	2	0	1	2	1	5	2	5	2	0	0	0	0	0	0	0	0
ENSG00000144677	838	757	730	675	576	585	437	646	38	44	116	38	519	433	348	577	310	482	446	690	830	281	553	295	6	21	9	26	139	204	101	303
ENSG00000199075	1	0	0	2	0	3	0	1	0	0	0	0	0	1	1	1	0	0	0	1	0	0	0	3	0	0	0	0	0	0	0	0
ENSG00000136059	1454	1074	1195	1114	400	408	300	424	368	300	1327	229	128	137	152	195	756	774	605	1294	636	363	455	302	228	328	287	671	72	112	75	123
ENSG00000187091	164	111	125	116	66	91	73	102	29	33	125	22	94	79	53	95	38	34	36	52	65	29	47	29	19	34	27	87	25	41	42	55
ENSG00000200579	4	0	2	1	1	1	2	3	1	0	8	0	1	2	6	1	0	0	0	1	2	2	2	0	2	0	0	0	0	1	0	1
ENSG00000008226	67	39	51	48	18	32	24	32	57	48	161	26	60	47	45	69	16	18	20	57	42	40	17	35	37	67	33	176	20	43	33	55
ENSG00000231668	0	0	1	0	0	0	0	0	0	0	1	0	1	0	1	0	1	0	1	0	0	0	0	0	0	0	0	0	0	0	0	0
ENSG00000060971	1671	1120	1446	1145	926	867	630	950	2008	1576	7360	1314	901	721	916	1003	983	1377	1052	1285	1422	570	1006	505	1726	2681	1851	4583	862	910	973	1028
ENSG00000201965	0	0	0	0	0	0	0	0	0	0	0	0	0	0	0	0	0	0	0	0	0	0	0	0	0	0	0	0	0	0	0	0
ENSG00000172936	1266	890	1269	898	1493	1494	1062	1591	1357	1117	4955	881	1039	960	1208	1342	1006	1395	1466	1367	3138	1190	2275	1121	1297	2129	1194	3332	1361	1550	1343	1781
ENSG00000172939	5054	3642	4428	3697	3310	3311	2485	3681	3358	2688	9459	1903	3282	3016	3230	3732	1366	1983	1786	2804	4610	1923	3391	1676	2715	4162	1812	6191	2224	2874	2341	3258
ENSG00000172940	7	5	7	11	8	5	2	3	3	4	24	2	15	9	14	12	2	2	3	1	10	2	1	2	2	4	6	9	4	8	8	4
ENSG00000237982	0	0	0	0	1	0	0	1	1	0	0	0	0	0	0	2	1	0	0	2	0	0	0	0	0	0	0	1	0	0	0	0
ENSG00000144671	0	2	1	1	0	1	1	1	1	6	5	3	4	5	3	9	4	0	2	2	0	0	0	2	1	2	0	7	1	6	3	3
ENSG00000199514	0	0	0	0	0	0	0	0	0	0	0	0	0	0	0	1	0	0	0	0	0	0	0	0	0	0	0	0	0	0	0	0
ENSG00000230123	0	0	0	0	0	0	0	0	0	0	0	0	0	0	0	0	0	0	0	0	0	0	0	0	0	0	0	0	0	0	0	0
ENSG00000093217	742	570	695	644	623	691	515	793	482	382	1357	317	385	397	362	439	255	335	267	665	966	451	653	419	386	615	253	1173	288	430	259	403
ENSG00000229589	103	73	85	71	14	25	8	18	27	33	101	10	35	31	24	40	20	29	21	68	19	11	9	12	11	30	16	56	16	23	15	38
ENSG00000114739	2192	2070	1812	1932	440	492	323	520	1045	1003	3870	703	2003	2225	1751	2563	450	721	370	1157	490	222	304	180	845	1267	812	2471	912	1521	915	1633
ENSG00000157036	841	602	710	678	839	900	728	1040	651	546	2520	404	443	362	461	485	259	285	335	737	1657	734	1041	743	614	887	557	1704	314	549	465	613
ENSG00000207512	2	1	1	0	1	1	3	4	2	0	4	0	0	0	0	1	0	0	0	1	4	1	3	3	0	3	0	5	1	1	1	2
ENSG00000230683	12	6	9	6	4	5	5	14	6	5	33	5	9	9	9	9	3	1	3	13	13	11	3	18	5	12	5	20	2	12	8	14
ENSG00000232439	64	49	60	69	51	59	28	59	33	30	137	21	38	41	29	47	11	15	15	43	96	50	67	52	38	50	29	90	30	36	22	54
ENSG00000183873	6	27	6	18	5	10	5	9	20	25	55	17	31	84	33	90	0	5	4	10	11	4	5	1	7	14	6	46	24	32	18	57
ENSG00000185313	192	147	155	138	0	0	0	0	0	0	0	0	0	0	0	0	92	92	61	153	0	0	0	0	0	1	1	0	0	0	0	0
ENSG00000231243	0	0	0	0	0	0	0	0	0	0	0	0	0	0	0	0	0	0	0	0	0	0	0	0	0	0	0	0	0	0	0	0
ENSG00000168356	8	7	11	6	8	11	11	15	3	3	9	4	11	34	15	19	3	6	4	11	12	11	7	3	1	4	11	2	5	13	6	17
ENSG00000206708	0	0	0	0	0	0	0	0	0	0	0	0	0	0	0	0	0	0	0	0	0	0	0	0	0	0	0	0	0	0	0	0
ENSG00000114742	2144	1733	1584	1591	1718	1796	1380	1887	1126	890	4402	779	1848	1735	1789	2093	1102	1783	1266	1386	3437	1317	2359	1178	1103	1561	1188	2758	1558	1760	1765	2046
ENSG00000114745	858	715	759	676	831	991	686	935	128	92	810	118	460	440	539	587	305	491	430	590	1303	572	902	523	85	175	290	504	472	526	483	704
ENSG00000168026	143	143	137	139	38	48	30	51	5	3	44	6	56	68	47	58	34	49	27	114	41	19	35	18	3	4	17	30	15	37	39	62
ENSG00000144655	393	341	331	291	228	275	168	311	50	51	308	70	192	220	302	293	84	140	116	162	262	132	209	95	46	85	89	214	146	178	190	224
ENSG00000214305	0	0	0	0	0	0	0	0	0	0	0	0	0	0	0	0	0	0	0	0	0	1	0	0	0	0	0	0	0	0	0	0
ENSG00000168334	1	3	0	1	1	5	1	0	0	0	0	0	30	31	45	28	1	0	0	0	1	1	0	1	0	0	0	0	8	19	52	36
ENSG00000215034	0	0	0	0	0	0	0	0	0	0	0	0	0	0	0	0	0	0	0	0	0	0	0	0	0	0	0	0	0	0	0	0
ENSG00000168329	48	97	31	86	65	69	32	68	2	3	7	2	16	16	15	12	11	59	8	43	65	27	38	28	1	7	5	6	8	9	11	20
ENSG00000270367	0	0	1	0	0	0	0	0	1	1	0	1	0	0	2	0	1	0	0	2	0	0	0	0	0	0	1	2	2	2	2	1
ENSG00000179934	246	198	142	193	0	4	0	0	1	3	4	0	298	207	271	303	47	113	31	79	1	0	2	1	0	0	1	2	455	350	367	458
ENSG00000228168	326	257	225	295	1	1	2	6	1	0	4	0	442	314	407	472	67	123	32	100	1	0	0	4	1	1	1	4	667	508	563	730
ENSG00000238267	5	4	1	2	1	0	0	0	3	0	5	0	6	0	1	7	1	0	2	3	0	0	1	1	0	0	2	2	4	1	3	1
ENSG00000223668	17	14	23	15	4	6	8	5	2	0	8	1	3	3	0	3	12	9	16	22	10	2	4	1	1	0	0	4	2	3	7	8
ENSG00000144659	1913	1374	1455	1314	1278	1257	1015	1453	914	745	3951	659	596	531	720	704	806	1194	829	1412	1929	848	1662	854	852	1356	951	2366	715	828	874	960
ENSG00000168028	37739	27491	25522	24989	18626	18698	13686	19402	30080	23537	79319	17736	22143	20980	21796	26807	50493	76081	49248	41926	45127	17525	31602	18224	27033	36827	15011	48976	31726	28693	36456	34857
ENSG00000206760	0	0	0	0	0	0	0	0	0	0	0	0	0	0	0	0	0	0	0	0	0	0	0	0	0	0	0	0	0	0	0	0
ENSG00000202363	0	0	0	0	0	0	0	0	0	0	0	0	0	0	0	0	0	0	0	0	0	0	0	0	0	0	0	0	0	0	0	0
ENSG00000168314	0	1	0	4	0	1	0	4	0	0	12	1	0	0	0	0	0	1	1	2	0	0	0	1	0	1	1	3	0	0	0	2
ENSG00000230757	0	0	0	0	0	0	0	0	0	0	0	0	0	0	0	0	0	0	0	0	0	0	0	1	0	0	0	0	0	0	0	0
ENSG00000170011	19	27	7	6	7	5	7	8	20	45	46	8	9	5	7	10	2	5	1	8	6	3	2	6	12	18	7	18	2	3	1	9
ENSG00000201570	0	0	0	0	0	0	0	0	0	0	0	0	0	0	0	0	0	0	0	0	0	0	0	0	0	0	0	0	0	0	0	0
ENSG00000280739	52	51	44	36	15	23	16	31	16	15	93	14	47	42	34	42	21	24	24	38	32	17	28	11	16	9	16	49	13	26	27	26
ENSG00000265327	0	1	0	1	0	0	0	0	0	0	4	0	0	0	0	0	0	0	0	0	0	0	0	0	0	0	0	0	0	0	0	1
ENSG00000202517	1	1	0	1	0	0	2	0	2	0	0	1	1	2	3	2	0	1	0	0	0	1	0	0	0	0	0	0	2	0	1	0
ENSG00000114784	904	599	822	587	789	719	665	876	421	370	1732	309	472	472	609	584	773	949	1065	938	1793	642	1484	840	504	627	410	1170	629	645	781	895
ENSG00000223797	72	51	66	55	32	38	32	44	61	39	138	29	53	42	42	60	51	82	66	49	55	21	39	36	45	40	28	119	34	30	33	43
ENSG00000230274	0	0	1	0	0	0	0	0	0	0	2	0	0	0	0	0	0	2	0	0	0	1	0	0	0	0	0	0	1	0	0	0
ENSG00000168032	5	4	15	6	7	5	3	5	2	6	23	4	5	9	9	9	1	0	1	5	5	6	3	5	1	6	7	24	1	1	3	9
ENSG00000188846	15185	10374	10840	11317	8889	9003	6779	9511	22175	16092	68339	13320	21768	20386	22924	26654	23251	27881	21069	17230	23583	9147	16085	9887	18377	24792	14539	39317	26621	25059	29927	31440
ENSG00000177873	15	12	1	8	202	247	174	284	209	181	674	131	258	272	264	310	4	2	0	8	376	175	300	173	176	267	154	509	130	221	158	253
ENSG00000199906	0	0	0	0	7	4	5	6	9	7	25	6	7	3	7	19	0	0	0	0	3	4	4	5	13	10	5	24	7	14	1	7
ENSG00000177842	4	2	2	2	209	288	203	253	92	64	261	46	69	93	94	100	0	3	2	5	480	203	351	163	59	97	61	162	56	70	70	88
ENSG00000172888	689	500	626	497	1686	1776	1436	1803	1328	1113	5557	793	1433	1223	1490	1780	231	270	231	559	3147	1313	2107	1210	1034	1600	1479	3025	790	1421	1056	1455
ENSG00000234067	0	0	0	0	0	0	0	0	0	0	0	0	0	0	0	0	0	0	0	0	0	0	0	0	0	0	0	0	0	0	0	0
ENSG00000226845	0	0	0	0	0	0	0	0	0	0	0	0	0	0	0	0	0	0	0	0	0	0	0	0	0	0	0	0	0	0	1	1
ENSG00000226302	0	0	1	1	0	0	1	0	0	0	1	0	0	1	0	1	0	0	0	0	1	0	0	0	0	0	0	0	0	0	0	0
ENSG00000229072	0	0	0	0	0	0	0	0	0	0	0	0	0	0	0	0	0	0	0	0	0	0	0	0	0	0	0	0	0	0	0	0
ENSG00000234287	5	0	5	1	4	1	0	2	2	0	11	1	1	2	6	5	3	4	6	9	6	0	2	2	3	3	3	8	0	6	8	5
ENSG00000231873	0	0	0	0	0	0	0	0	0	0	0	0	0	0	0	0	0	0	0	0	0	0	0	0	0	0	0	0	0	0	0	0
ENSG00000233096	0	0	0	0	0	0	0	0	0	0	0	0	0	1	2	1	0	0	0	0	0	0	0	0	0	0	0	0	0	0	0	0
ENSG00000233919	0	0	0	0	0	0	0	0	0	0	0	0	0	0	0	0	0	0	0	0	0	0	0	0	0	0	0	0	0	0	0	0
ENSG00000275256	0	0	0	0	0	0	0	0	0	0	0	0	0	0	0	0	0	0	0	0	0	0	0	0	0	0	0	0	0	0	0	0
ENSG00000168036	2989	2427	2501	2284	3857	4245	2832	4324	4055	3426	16391	2831	2486	2458	2808	3328	1712	3019	2569	2778	9470	3871	6506	3213	3673	5368	3875	9896	2192	2901	2681	3238
ENSG00000168038	329	185	236	185	307	316	266	365	79	57	180	37	73	79	84	90	201	265	206	219	626	250	465	260	65	107	47	186	62	94	113	132
ENSG00000252409	0	1	0	0	0	1	0	1	0	0	1	0	3	0	0	1	0	1	0	1	1	0	0	0	0	0	0	0	0	0	1	0
ENSG00000222942	0	0	1	0	0	1	0	3	0	0	0	0	0	0	1	0	0	0	0	1	0	0	1	2	0	0	0	0	0	0	0	0
ENSG00000223513	1	1	0	0	1	3	2	2	1	0	2	0	0	1	0	1	0	0	0	4	1	2	1	1	0	3	0	1	0	0	0	2
ENSG00000251986	3	0	0	0	1	0	0	1	0	0	0	0	0	0	0	0	0	0	0	1	0	2	0	0	0	0	0	1	2	0	0	0
ENSG00000212145	1	0	0	0	0	1	0	2	0	0	0	0	0	0	0	0	0	0	0	0	0	1	0	1	0	0	0	0	0	0	1	1
ENSG00000234270	0	0	0	0	0	0	1	0	0	0	0	0	0	0	0	0	0	0	0	0	1	0	0	0	0	0	0	0	0	0	0	0
ENSG00000235339	0	0	0	0	0	0	0	0	0	0	0	0	0	0	1	0	0	0	0	0	1	0	0	0	0	0	0	0	0	0	0	0
ENSG00000182606	1096	1310	1041	1262	2433	2505	1803	2543	1361	1215	5673	1024	1788	1634	1630	2191	236	543	341	1010	3801	1636	2636	1441	1079	1821	1347	3595	934	1359	978	1528
ENSG00000229339	3	0	4	1	2	2	1	6	3	0	6	2	2	3	3	1	1	0	0	1	19	5	2	13	0	2	2	8	1	4	6	7
ENSG00000234562	2	8	1	3	7	7	9	8	1	2	11	4	4	4	3	2	0	0	0	4	18	6	8	9	6	5	9	26	1	4	7	9
ENSG00000222872	0	2	1	0	1	3	2	2	0	0	2	0	5	1	3	3	0	0	0	2	0	3	2	1	1	0	0	1	1	0	4	5
ENSG00000187094	0	0	0	0	0	0	0	0	0	0	0	0	0	0	0	0	0	0	0	0	0	0	0	0	0	0	0	0	0	0	0	0
ENSG00000281160	0	0	0	0	0	0	0	0	0	0	0	0	0	0	0	0	0	0	0	0	0	0	0	0	0	0	0	0	0	0	0	0
ENSG00000231280	0	0	0	0	0	0	0	0	0	0	0	0	6	5	6	6	0	0	0	0	0	0	0	0	0	0	0	0	6	3	5	3
ENSG00000229572	0	0	0	0	0	0	0	0	0	0	0	0	1	0	0	0	0	0	0	0	0	0	0	0	0	0	0	0	0	0	0	3
ENSG00000157093	0	0	0	0	0	0	0	0	0	0	3	0	10	12	13	8	0	0	0	0	0	0	0	0	0	0	3	0	20	16	23	23
ENSG00000235787	0	0	0	0	0	0	0	0	0	0	0	0	0	0	0	0	0	0	0	0	0	0	0	0	0	0	0	0	0	0	0	0
ENSG00000114812	7	6	6	4	2	6	1	2	17	18	19	6	18	41	39	57	2	0	3	0	6	3	2	1	5	7	6	14	38	62	47	85
ENSG00000232354	5	3	7	8	9	3	4	6	4	7	11	4	18	14	10	13	3	2	0	7	0	4	2	1	1	6	3	4	3	12	4	31
ENSG00000093183	1723	1231	1402	1263	939	998	676	999	1301	1127	5003	869	994	1003	1044	1308	607	827	747	1092	1467	644	1079	541	1219	1869	1144	3472	824	1195	843	1236
ENSG00000008324	558	353	465	380	447	486	384	523	652	418	1560	357	428	381	518	503	594	697	723	711	1164	481	850	631	611	788	335	1007	703	623	793	749
ENSG00000114857	11208	8192	8830	8393	8221	8818	6661	9345	6497	6003	24825	3903	5324	4988	4845	6456	2363	2155	1990	6941	12173	6107	6738	6143	4574	7012	5482	14707	1976	5287	2648	5818
ENSG00000230084	71	79	55	33	38	27	26	41	26	28	114	18	26	28	27	33	10	9	6	37	54	23	24	28	30	47	25	120	8	26	13	36
ENSG00000114853	2	0	1	1	90	134	76	114	0	0	1	0	3	7	2	5	0	0	0	0	143	68	100	46	0	4	0	1	0	0	2	3
ENSG00000240203	0	0	0	0	0	0	0	0	0	0	0	0	0	0	0	0	0	0	0	0	0	0	0	0	0	0	0	0	0	0	0	0
ENSG00000157119	0	0	0	0	0	0	0	0	0	0	0	0	0	0	0	0	0	0	0	0	0	0	0	0	0	0	0	0	0	0	0	0
ENSG00000244607	14	18	11	16	36	22	29	24	13	9	57	9	7	3	6	6	16	12	13	16	56	23	35	32	5	9	6	33	7	7	6	6
ENSG00000010282	0	0	0	1	0	0	0	0	0	0	0	0	0	0	1	0	0	0	0	1	3	0	3	0	0	0	0	4	0	0	0	0
ENSG00000230970	1	0	0	1	0	2	4	0	0	1	6	2	0	0	0	0	0	0	0	0	0	1	0	0	0	0	0	1	0	0	0	0
ENSG00000173811	3	3	7	4	9	8	5	8	1	1	15	2	0	0	0	0	1	2	4	11	22	7	5	6	2	5	5	9	1	1	0	0
ENSG00000280571	1	2	0	2	3	4	4	1	4	3	18	2	0	1	1	2	0	5	2	1	8	4	6	8	4	4	2	3	0	0	0	1
ENSG00000225611	1	0	1	1	2	4	3	5	0	1	2	0	2	0	0	1	3	0	0	1	3	5	3	2	0	1	0	1	0	0	0	0
ENSG00000181061	1591	1001	1424	1083	1266	1354	1051	1422	1031	833	4569	726	695	683	1017	821	1829	2522	1876	2275	3468	1459	2488	1692	1315	1844	839	2636	1093	993	1360	1285
ENSG00000235288	2	0	0	2	0	1	3	1	2	0	3	0	0	0	0	0	1	0	1	1	6	0	1	2	0	1	2	1	0	0	0	0
ENSG00000144648	9	14	11	11	266	298	211	313	11	14	107	6	3	9	5	6	2	4	7	15	427	162	261	176	7	7	12	39	1	3	2	3
ENSG00000273328	2	8	3	5	2	8	9	15	1	0	2	0	0	0	0	2	2	5	3	4	4	6	3	4	0	0	3	1	0	0	0	0
ENSG00000273291	4	2	4	3	0	3	1	0	0	0	4	0	0	0	1	0	2	4	1	2	1	0	0	0	0	0	0	0	0	0	0	1
ENSG00000240747	0	1	0	1	0	0	0	0	0	0	0	0	0	0	0	0	2	0	0	0	0	1	0	0	0	0	0	0	0	0	0	0
ENSG00000180432	2	2	5	2	32	46	42	50	0	0	11	1	0	0	0	0	1	0	0	5	35	21	27	22	0	1	0	1	0	0	0	0
ENSG00000236459	1	0	0	0	0	0	3	1	3	1	0	1	0	0	0	0	0	0	0	0	2	0	1	0	0	1	0	0	0	0	0	0
ENSG00000182983	0	3	0	3	0	2	0	0	1	0	1	0	0	0	0	2	0	0	1	1	0	0	0	0	0	1	0	0	0	1	0	1
ENSG00000206552	0	0	0	0	0	0	0	0	0	0	0	0	0	0	0	1	0	0	0	1	1	0	0	0	0	0	0	0	0	0	0	0
ENSG00000144649	30	37	33	43	6	14	8	15	0	1	8	1	5	9	2	4	15	31	14	34	12	7	9	9	1	0	0	3	1	6	4	7
ENSG00000232375	0	0	1	1	0	0	0	1	0	0	0	0	0	1	0	0	1	0	0	0	0	1	0	0	0	0	0	0	0	0	0	1
ENSG00000144647	851	654	705	701	499	492	327	489	255	246	810	182	421	451	459	589	280	578	348	656	713	282	547	274	233	413	166	630	407	521	389	552
ENSG00000227245	14	10	8	9	3	3	1	6	3	4	10	1	5	6	4	2	3	7	1	7	17	1	4	1	5	4	6	12	1	9	1	8
ENSG00000163788	1420	1086	1232	1039	939	1136	674	1086	729	646	3520	547	657	643	660	815	314	578	552	869	1562	762	1205	633	899	1380	849	2505	434	616	505	661
ENSG00000241939	3	0	4	0	2	0	2	2	2	2	9	1	0	6	2	0	1	0	0	3	3	8	2	2	2	5	8	12	5	1	4	1
ENSG00000271941	9	7	4	2	6	10	3	14	5	6	25	4	0	2	5	5	3	2	1	9	14	5	7	2	5	8	7	21	2	1	4	2
ENSG00000234617	7	5	12	0	11	3	1	8	0	2	17	2	4	3	2	9	0	1	1	14	9	4	4	2	8	2	5	13	1	6	3	5
ENSG00000160746	360	312	267	345	512	555	361	579	170	158	663	99	211	266	175	299	146	305	193	313	811	317	579	279	201	290	180	552	96	148	138	223
ENSG00000251811	0	1	2	0	0	1	1	0	1	0	0	0	0	1	1	0	0	0	0	0	1	0	1	1	1	1	2	1	0	0	0	1
ENSG00000223916	1	3	1	0	2	0	2	2	0	0	5	0	3	2	1	0	0	0	1	3	7	2	2	4	1	1	0	2	0	1	1	1
ENSG00000011198	464	311	397	342	294	340	273	361	183	130	686	129	238	247	250	319	480	646	570	580	991	329	591	383	184	288	147	533	288	333	336	355
ENSG00000235886	0	0	0	0	0	1	0	1	0	0	0	0	0	0	0	0	0	0	0	0	0	0	0	0	0	0	0	1	0	0	0	0
ENSG00000271192	0	1	0	1	0	0	0	0	0	0	0	0	0	0	0	0	0	0	0	3	0	0	0	0	0	0	0	0	0	0	0	0
ENSG00000272121	0	0	0	0	0	0	0	0	0	0	2	0	0	0	0	0	0	1	0	0	0	0	0	0	0	1	0	4	0	0	0	0
ENSG00000252980	0	0	0	0	0	0	0	0	0	0	2	0	0	0	0	0	0	0	0	0	0	0	0	0	0	0	0	0	0	0	0	0
ENSG00000207954	0	1	0	0	0	0	0	0	0	0	8	2	0	0	2	0	0	0	0	0	0	1	0	0	2	2	0	2	0	0	0	0
ENSG00000280435	0	3	0	5	0	0	0	0	13	18	71	10	5	9	9	1	0	2	0	3	0	0	0	0	3	12	7	25	1	1	0	3
ENSG00000261786	0	1	0	0	0	0	0	0	36	46	260	43	4	7	12	19	0	2	0	1	0	0	0	0	12	33	32	81	1	6	10	11
ENSG00000173769	0	0	2	0	0	1	0	1	0	0	1	0	0	0	0	0	0	1	0	1	0	0	0	0	0	0	0	0	0	0	0	1
ENSG00000271937	7	7	5	2	8	3	1	2	1	0	6	0	1	3	3	5	10	12	9	5	6	2	8	8	1	1	0	3	1	5	6	3
ENSG00000179152	1195	831	1062	822	623	694	459	716	844	692	2993	467	523	642	543	712	1288	1625	1414	1191	1390	615	1002	638	750	939	693	1941	501	538	558	688
ENSG00000225873	20	3	17	11	75	83	57	82	0	0	0	0	16	20	19	18	17	6	17	8	111	54	77	49	0	0	0	0	12	2	13	9
ENSG00000283036	9	5	6	8	21	18	5	16	0	0	0	0	7	12	5	8	3	0	2	2	16	19	12	18	0	0	0	0	0	3	4	3
ENSG00000185219	4596	3546	3999	3606	2821	3017	2200	3408	1506	1283	4952	829	2082	1932	1841	2388	939	997	963	2740	4041	1987	2796	1909	1176	1781	1214	3441	830	1467	992	1604
ENSG00000178917	390	328	368	294	258	300	230	323	157	127	540	70	189	152	184	193	119	101	105	267	453	217	314	187	122	194	144	356	74	143	111	151
ENSG00000214825	0	0	0	0	0	0	0	0	0	0	0	0	0	0	0	0	0	0	0	0	0	0	0	0	0	0	0	0	0	0	0	0
ENSG00000277509	0	0	0	0	0	0	0	0	0	0	0	0	0	0	0	0	0	0	0	0	0	0	0	0	0	0	0	0	0	0	0	0
ENSG00000196345	4	0	6	9	0	1	4	1	92	42	326	40	0	1	4	1	2	3	1	4	2	2	0	0	50	41	86	113	0	0	2	1
ENSG00000236869	2	1	2	0	11	3	1	5	18	11	78	4	5	4	7	5	2	0	0	1	7	2	5	4	11	21	16	58	2	4	3	7
ENSG00000144792	4	3	3	3	681	672	493	781	38	22	171	15	5	9	4	5	0	0	0	5	1352	524	823	534	23	33	33	73	0	9	15	11
ENSG00000214820	1	1	2	5	1	0	1	2	6	4	39	0	4	2	3	2	1	1	0	0	2	2	3	2	11	5	7	11	2	3	2	3
ENSG00000186448	865	683	695	618	927	1007	713	1055	709	682	3333	545	579	581	601	700	362	497	436	656	1702	769	1218	668	693	989	911	2056	388	576	456	601
ENSG00000233509	6	2	4	2	16	24	17	26	9	12	36	8	2	3	6	6	2	0	2	4	30	20	15	19	10	7	2	21	1	9	6	2
ENSG00000169981	20	15	23	22	52	76	40	50	171	142	909	130	75	72	101	104	7	9	16	27	72	33	51	25	228	336	210	621	101	109	167	146
ENSG00000272077	3	2	4	0	11	13	7	8	73	50	307	43	20	11	29	27	1	3	1	7	9	8	5	14	79	80	66	135	19	32	25	34
ENSG00000235805	0	0	0	0	0	0	0	0	0	0	3	0	0	0	0	0	0	0	0	0	0	0	0	1	0	0	0	0	0	0	0	0
ENSG00000196653	1	0	0	0	0	0	0	0	93	70	329	54	0	0	0	0	0	0	1	1	1	0	0	0	60	87	89	169	0	0	0	0
ENSG00000186446	0	0	1	0	117	99	91	126	94	77	343	70	84	88	113	90	2	2	0	2	212	113	200	83	72	131	104	224	48	51	91	67
ENSG00000163807	1693	975	1378	932	1896	1860	1586	1993	1578	1278	5092	974	1846	1618	1945	2179	1138	1491	1629	1682	4046	1705	3156	1571	1721	2341	1222	4003	1803	1725	2092	1971
ENSG00000163808	3937	2650	3466	2660	3004	3178	2403	3365	2142	1738	5133	1242	1377	1324	1479	1725	3174	3487	3585	3268	7317	2822	4857	2827	2100	3171	636	5009	1225	1554	1629	1816
ENSG00000169964	461	326	382	340	137	138	95	154	178	139	670	131	205	215	170	272	302	309	258	339	197	91	101	67	117	198	173	432	126	134	111	163
ENSG00000163810	0	1	2	3	1	2	3	0	1	3	12	3	1	0	1	0	0	0	0	1	7	8	3	10	2	6	2	10	0	3	5	1
ENSG00000235845	0	0	0	0	0	0	0	0	0	0	0	0	0	0	0	0	0	0	0	0	0	0	0	0	0	0	0	0	0	0	0	0
ENSG00000163812	1451	1031	1338	1160	1228	1296	871	1357	1104	814	4368	710	1080	855	1083	1236	623	1076	946	1166	2147	882	1509	791	1086	1738	1000	2815	891	1029	937	1188
ENSG00000227123	4	7	7	5	0	1	0	2	4	4	13	0	2	3	7	4	2	8	0	11	4	11	1	2	2	5	2	15	0	6	3	1
ENSG00000075914	1519	1030	1195	1197	1016	1093	764	1180	775	574	2454	442	1441	1248	1512	1746	1260	1806	1405	1276	2045	837	1420	777	694	1044	479	1497	1330	1384	1570	1543
ENSG00000163815	5	11	6	6	6	8	3	10	5	4	33	2	16	22	8	10	0	0	3	10	13	7	3	9	3	5	15	13	4	3	4	8
ENSG00000252410	0	0	1	0	0	0	2	2	0	0	0	1	1	0	0	1	0	0	0	0	0	0	0	1	0	0	0	0	0	1	0	0
ENSG00000163814	0	2	0	2	15	14	16	23	0	0	0	0	4	3	10	9	0	3	2	2	13	7	21	15	0	0	0	0	7	11	8	7
ENSG00000224094	0	1	1	1	2	0	0	2	2	4	5	0	0	0	1	1	1	0	0	2	3	0	1	0	0	3	1	2	3	1	2	1
ENSG00000249992	0	2	0	0	35	46	19	34	0	0	1	0	44	45	43	63	0	0	0	0	32	21	32	21	0	0	0	0	29	55	24	27
ENSG00000202268	0	0	0	0	0	0	0	0	0	0	0	0	0	0	0	0	0	0	0	0	0	0	0	0	0	0	0	0	0	0	0	0
ENSG00000011376	1575	1164	1342	1157	1371	1406	1039	1532	1026	804	3553	655	1071	857	1160	1162	399	624	659	985	2272	1036	1771	816	936	1562	705	2399	836	997	961	1067
ENSG00000232455	5	1	10	2	6	9	3	1	5	2	16	3	4	5	0	1	2	3	2	5	9	7	6	1	1	2	1	21	2	3	7	6
ENSG00000144791	2562	2336	2406	2286	2278	2343	1788	2375	1810	1491	6226	1200	1626	1446	1749	1865	518	771	704	1685	3682	1506	2432	1402	1200	2088	1242	4097	989	1395	1054	1590
ENSG00000230530	22	25	24	10	29	31	10	25	13	3	6	2	11	2	1	12	9	2	13	8	40	19	44	17	3	9	1	13	4	3	6	3
ENSG00000211456	2464	1552	2007	1603	4216	4398	3240	4733	2202	1791	7924	1301	2197	1822	2377	2400	2066	2542	2561	2515	10533	4450	6687	4714	2592	3749	1919	5447	2203	2337	2752	2731
ENSG00000244357	7	4	14	7	20	26	11	16	14	22	38	8	7	5	5	14	5	1	1	5	22	14	20	15	13	17	7	16	3	11	3	9
ENSG00000279017	0	1	0	1	1	0	0	2	0	0	2	0	0	0	0	0	0	0	1	0	2	3	1	2	2	2	1	9	0	2	0	3
ENSG00000163817	4	5	1	3	1	3	1	1	0	3	3	1	1	2	4	0	0	1	2	11	3	3	3	7	0	0	1	6	0	0	0	0
ENSG00000163818	1036	785	743	696	170	226	118	193	314	238	951	168	681	643	661	683	1072	1342	1137	1219	443	162	293	225	268	316	249	617	540	516	695	629
ENSG00000236930	0	1	1	0	0	0	0	0	0	0	0	0	0	0	0	0	0	0	0	0	0	0	0	0	0	0	0	0	0	1	1	1
ENSG00000173585	5	3	3	3	2	4	1	1	0	0	2	0	4	5	7	4	4	2	9	15	1	0	1	6	0	0	0	3	1	15	1	8
ENSG00000201635	3	0	0	0	0	1	0	0	0	0	0	0	0	0	0	0	0	0	0	0	0	0	0	0	0	0	0	1	0	0	0	0
ENSG00000163820	980	892	876	882	686	745	505	764	308	237	931	194	1164	1034	1053	1254	249	349	299	739	822	390	605	351	174	284	252	644	543	841	556	833
ENSG00000172215	33	34	31	32	12	18	15	22	17	8	39	5	15	22	13	14	7	3	16	40	32	27	9	20	11	14	10	36	7	18	12	22
ENSG00000173578	8	8	3	2	0	0	0	0	0	0	0	0	0	2	0	2	1	6	0	14	0	0	0	0	0	0	0	0	1	6	0	4
ENSG00000226933	1	3	0	0	0	0	0	0	0	0	1	0	0	1	0	0	1	0	1	5	0	0	0	0	0	0	1	1	2	1	0	1
ENSG00000229515	0	0	0	0	0	0	0	0	0	0	0	0	0	0	0	0	0	0	0	0	0	0	0	0	0	0	0	0	0	0	0	0
ENSG00000183625	1	4	1	1	0	0	0	0	2	3	22	6	1	1	1	1	2	0	0	2	0	0	0	0	0	1	1	5	2	3	1	2
ENSG00000163823	1	0	1	0	11	18	13	8	169	114	1260	122	15	19	15	26	0	0	1	1	25	9	9	8	98	106	155	316	11	14	14	25
ENSG00000236736	0	0	0	0	0	0	0	0	0	0	0	0	0	0	0	0	0	0	0	0	0	0	0	0	0	0	0	0	0	0	0	0
ENSG00000121807	4	4	5	4	0	0	0	4	1	0	4	2	0	0	0	0	2	3	6	2	2	1	1	0	0	0	3	0	0	0	0	0
ENSG00000223552	0	0	1	2	0	0	0	0	0	0	2	0	3	4	3	3	0	2	2	1	0	0	0	0	0	0	1	0	2	4	2	6
ENSG00000160791	1	0	0	0	5	7	5	9	21	11	181	12	0	2	0	3	1	0	2	0	5	4	4	3	23	19	55	87	0	2	0	2
ENSG00000121797	41	26	36	23	0	0	0	0	1	2	2	2	23	24	27	36	34	51	55	25	0	0	0	0	0	1	0	0	20	26	39	31
ENSG00000283646	0	0	0	0	0	0	1	0	0	0	0	0	6	9	8	7	0	0	0	0	1	1	1	0	0	0	0	0	0	0	0	5
ENSG00000012223	3	0	0	0	0	0	0	5	0	2	22	1	4	7	5	3	0	0	0	0	2	3	1	1	4	7	11	9	2	4	6	4
ENSG00000163825	0	0	0	0	0	0	0	0	0	0	0	0	2	0	0	1	0	0	0	0	0	0	0	0	0	0	0	1	0	1	1	1
ENSG00000163827	0	0	0	0	0	0	1	1	0	1	2	0	0	0	1	0	1	0	3	0	0	1	1	0	0	0	1	0	3	4	1	2
ENSG00000268324	2	7	0	6	0	0	0	1	0	0	0	0	0	1	0	0	3	2	1	1	0	0	0	0	0	0	0	0	0	0	1	3
ENSG00000241186	0	0	0	0	0	0	1	0	1	0	2	3	0	0	0	0	0	0	0	0	0	0	2	1	0	0	4	0	0	0	2	0
ENSG00000283473	0	0	0	0	0	0	0	0	0	0	0	0	0	0	0	0	0	0	0	0	0	0	0	0	0	0	0	0	0	0	0	0

ENSG00000251967	0	0	0	0	0	0	0	0	0	0	0	0	0	0	0	0	0	0	0	0	0	0	0	0	0	0	0	0	0	0	0	0
ENSG00000178038	35	62	29	50	1	0	1	4	4	0	6	0	10	7	2	16	8	12	5	27	1	0	1	0	0	0	2	0	2	3	2	1
ENSG00000181585	86	46	69	72	0	0	0	0	0	0	0	0	9	11	5	10	41	37	33	78	0	0	0	0	0	0	0	0	0	4	0	1
ENSG00000206549	1	1	2	1	0	3	1	2	0	0	0	0	3	3	2	5	1	1	1	1	1	0	1	2	0	0	0	0	4	0	3	3
ENSG00000261603	1	0	1	0	2	2	1	0	0	0	0	0	0	0	0	0	0	0	0	0	0	0	0	0	0	0	0	0	0	0	0	0
ENSG00000188086	0	0	0	0	1	3	0	1	0	0	0	0	0	0	0	0	0	0	0	0	3	0	0	0	0	0	0	0	0	0	0	0
ENSG00000238013	0	0	0	0	0	0	0	0	0	0	0	0	0	0	0	0	0	0	0	0	0	0	0	0	0	0	0	0	0	0	0	0
ENSG00000226074	0	0	0	0	1	0	1	1	0	0	0	0	0	0	0	0	0	0	0	0	0	0	0	0	0	0	0	0	0	0	0	0
ENSG00000178055	0	0	0	0	6	4	6	10	0	0	1	0	0	1	0	0	0	0	0	0	10	1	6	1	0	0	0	0	0	0	0	0
ENSG00000160808	0	0	0	0	0	0	0	0	0	0	0	0	0	0	0	0	0	0	0	0	0	0	0	0	0	0	0	0	0	0	0	0
ENSG00000160801	0	0	0	0	0	0	0	0	0	0	0	0	1	0	0	1	0	0	0	0	1	1	0	0	0	0	0	0	2	0	0	0
ENSG00000273682	3	3	3	0	5	4	3	6	8	6	18	3	5	4	2	4	0	4	0	4	11	4	7	5	5	5	6	21	3	10	4	11
ENSG00000160799	1377	780	1135	1012	1552	1420	1169	1640	1009	668	3215	616	1260	1090	1269	1435	1253	1443	1402	1231	3221	1371	2810	1151	820	1305	695	1938	1141	1067	1360	1367
ENSG00000160796	4942	4405	5538	4510	5127	6189	4500	6242	3822	3070	12563	2182	4361	3524	3891	4615	1455	1483	1582	5654	7951	4434	5309	4248	2442	4329	2657	7217	1492	2836	1626	3466
ENSG00000236409	8	10	4	8	10	11	7	20	7	10	27	4	18	12	9	12	5	6	12	12	3	2	3	11	1	10	5	21	2	6	3	7
ENSG00000181555	3441	2697	3369	2585	4280	4198	3290	4310	2479	2097	10102	1601	2273	1971	2450	2491	857	1091	1124	2207	7367	3131	5095	2891	2363	3802	2704	7082	1338	2073	1703	2424
ENSG00000275940	1	2	3	1	3	2	0	7	1	2	11	2	0	0	4	1	0	0	1	2	4	3	2	6	1	4	3	5	0	0	2	0
ENSG00000227398	125	118	106	112	67	51	31	56	21	20	133	14	29	24	34	39	17	33	28	58	79	29	53	20	35	46	39	79	11	24	26	37
ENSG00000088727	212	119	162	158	133	142	91	130	124	89	348	63	216	195	265	228	233	317	222	262	264	94	142	128	94	171	61	226	277	288	334	287
ENSG00000239128	1	0	0	0	0	1	1	1	2	0	0	0	1	1	0	3	0	0	0	1	0	0	0	0	0	0	0	3	1	1	1	0
ENSG00000114648	929	553	793	588	803	819	569	787	494	367	2296	361	878	682	1053	845	336	494	498	779	1467	663	1148	569	588	916	451	1428	865	928	1025	1156
ENSG00000280173	78	62	66	58	31	32	38	57	47	52	236	45	72	66	96	71	9	29	30	58	78	22	33	19	62	102	54	158	78	104	68	84
ENSG00000260236	39	58	52	59	52	51	32	54	55	56	239	27	32	28	26	62	16	15	21	54	78	33	37	42	41	62	65	122	9	39	21	55
ENSG00000076201	1342	1260	1419	1265	1440	1607	1158	1785	904	818	3838	618	797	772	858	1001	280	402	314	831	1994	981	1426	932	704	1336	845	2397	451	718	450	791
ENSG00000114650	2270	1906	2264	1976	2680	2737	1999	2721	1660	1367	6903	1158	1700	1471	1685	2001	562	983	844	1700	3641	1650	2632	1383	1304	2284	1440	3951	1311	1720	1330	1796
ENSG00000276925	9	2	8	3	1	7	6	3	4	2	27	1	4	6	1	2	2	3	3	10	12	4	5	5	2	14	10	25	1	5	4	11
ENSG00000163832	1058	800	907	797	961	1014	735	1039	840	623	2546	498	635	527	585	812	767	1187	986	1104	2014	833	1488	762	623	919	521	1631	644	664	650	784
ENSG00000271161	53	29	29	35	39	46	39	26	34	19	114	17	18	18	18	24	11	18	19	54	96	50	46	37	23	28	17	63	17	26	24	35
ENSG00000244732	0	0	0	0	0	0	0	0	0	0	0	0	0	0	0	0	0	0	0	0	0	0	0	0	0	0	0	0	0	0	0	0
ENSG00000114646	11	23	17	31	19	14	12	26	10	11	40	6	11	37	16	51	4	9	4	33	22	8	16	6	2	13	12	24	8	20	5	18
ENSG00000173473	8107	5448	7073	5593	12185	11559	9375	12099	6026	4721	18068	3403	8664	7332	8757	9122	1665	2352	2333	4267	17779	7193	13688	6564	5003	8209	3433	12086	5496	6498	5670	6934
ENSG00000214773	16	12	17	9	7	11	20	20	8	7	41	6	4	9	17	3	5	1	3	13	27	8	10	32	12	20	17	43	4	9	9	20
ENSG00000235695	5	4	1	0	1	3	2	5	4	0	10	1	1	1	3	2	2	0	3	2	6	1	0	4	4	4	0	8	1	3	0	4
ENSG00000132153	3629	2591	3188	2576	3980	3942	2862	3978	2370	1870	8950	1557	2877	2425	2803	3139	1292	1704	1700	2893	5819	2682	4377	2292	2165	3576	1953	6122	2308	2675	2296	3130
ENSG00000274420	0	1	3	2	5	0	0	0	0	0	0	0	1	1	0	2	1	0	0	6	7	0	0	2	0	0	7	5	0	0	0	2
ENSG00000221585	0	0	1	0	0	1	0	0	0	0	1	0	0	0	0	0	0	0	0	0	0	0	0	0	0	0	0	0	0	0	0	0
ENSG00000047849	5417	4531	4569	4618	3611	3926	2577	4132	5799	4591	20728	3494	5369	5089	4592	6371	876	1438	1030	2469	3706	1791	2495	1467	4475	7676	4529	13572	2818	3945	2660	4161
ENSG00000199591	2	0	1	0	0	1	0	0	2	0	4	0	1	0	0	0	0	0	0	1	1	1	0	0	0	3	0	9	0	4	1	0
ENSG00000224895	5	2	4	5	2	5	1	0	3	1	19	0	4	4	6	2	0	3	1	2	10	1	6	2	9	7	4	25	0	2	4	12
ENSG00000270285	1	4	4	1	1	0	1	4	3	3	14	3	1	2	1	3	0	1	0	2	2	1	1	0	2	2	3	17	0	1	2	2
ENSG00000265078	2	1	1	2	0	0	0	1	0	0	0	1	0	4	0	0	0	0	0	2	0	0	0	0	0	1	0	2	0	0	0	1
ENSG00000236768	0	0	0	0	0	1	0	1	1	1	0	0	0	0	1	0	0	1	0	0	0	0	0	0	0	1	0	1	0	2	1	0
ENSG00000164045	2949	2025	2577	2053	1695	1755	1454	1794	844	612	2290	521	2325	1861	2909	2488	1241	2089	2431	2410	3709	1482	2698	1259	910	1476	317	2023	2312	2542	2866	2875
ENSG00000235627	12	3	10	7	4	5	4	12	3	5	11	1	7	4	4	6	3	6	2	11	12	7	6	6	10	6	0	6	1	13	2	12
ENSG00000253056	3	6	7	5	5	6	4	1	1	1	9	1	4	1	1	0	0	3	1	2	4	2	3	2	1	5	3	2	6	0	6	5
ENSG00000231884	7	2	6	1	5	6	7	3	1	1	5	3	2	2	4	7	1	3	1	3	5	5	6	5	3	4	1	3	3	10	4	5
ENSG00000265483	0	0	0	0	0	0	0	0	0	0	0	0	0	0	0	0	0	0	0	0	0	0	0	0	2	0	1	0	0	0	0	0
ENSG00000164047	0	0	0	0	0	0	0	0	0	0	0	0	0	0	0	0	0	0	0	0	0	0	1	0	0	0	0	0	0	0	0	0
ENSG00000164048	1106	847	1032	887	629	768	492	796	1148	977	2756	620	407	375	372	512	460	417	397	799	853	393	651	403	623	837	520	1443	189	346	200	379
ENSG00000229759	18	13	22	18	21	25	11	24	53	43	125	32	12	15	16	9	5	2	2	18	15	7	9	6	27	40	25	68	1	8	3	19
ENSG00000199476	7	5	11	10	2	4	2	6	5	7	22	0	2	3	3	1	7	9	6	3	2	2	0	4	5	1	5	5	1	0	1	0
ENSG00000228638	105	88	134	80	108	115	100	136	120	82	474	100	74	90	92	122	54	89	88	111	219	81	195	97	125	223	110	296	79	106	87	102
ENSG00000172113	478	380	472	385	409	449	311	478	422	336	1555	286	331	342	426	497	375	467	398	434	830	381	659	324	419	664	385	1096	345	358	375	462
ENSG00000229453	2	3	5	1	1	2	0	0	0	0	0	0	0	0	1	0	0	0	1	2	2	0	0	0	0	0	1	3	0	0	0	2
ENSG00000252466	1	0	2	0	0	0	0	1	0	0	1	0	0	0	0	0	0	0	0	0	0	0	0	0	0	0	0	1	0	0	0	0
ENSG00000164049	10	10	14	4	5	4	8	13	2	2	13	2	2	0	0	1	7	4	7	14	25	6	7	6	2	2	2	6	0	0	0	0
ENSG00000277483	0	0	0	0	0	0	0	0	0	0	0	0	0	0	0	0	0	0	0	0	0	0	0	0	0	0	0	0	0	0	0	0
ENSG00000164050	1229	1221	1290	1287	1809	2114	1421	2131	623	607	5822	583	24	52	24	53	264	353	317	963	2411	1161	1211	1183	357	677	1310	1989	7	56	10	85
ENSG00000164051	397	296	375	294	556	523	435	558	438	305	1105	246	548	440	576	588	270	478	389	363	1110	477	1016	412	282	524	209	741	646	575	699	557
ENSG00000232112	2935	1880	2604	2253	2126	2014	1540	2388	2779	1764	9028	1630	1433	1410	1817	1877	4106	4277	4864	4160	5581	2246	4291	2829	2167	3232	1876	5111	2532	2021	3045	2768
ENSG00000244380	0	0	2	0	1	1	3	4	5	0	4	0	0	2	1	3	0	1	1	2	2	1	0	2	0	0	0	4	1	0	0	1
ENSG00000164053	65	41	59	47	40	52	35	30	42	33	142	38	30	27	39	28	13	34	31	50	65	32	46	23	22	59	25	96	30	24	31	26
ENSG00000282827	42	32	50	38	35	45	34	37	33	27	123	29	24	23	16	27	20	16	11	38	50	26	35	32	36	49	36	95	10	37	21	57
ENSG00000213689	0	0	0	0	0	0	0	0	0	0	0	0	0	0	0	0	0	0	0	0	0	0	0	0	0	0	0	0	0	0	0	0
ENSG00000164054	2594	1682	2326	1782	2555	2624	1810	2754	1825	1441	9922	1529	731	695	658	876	615	928	1062	1375	2605	1175	2233	1005	1465	2368	1983	5126	414	445	423	577
ENSG00000114268	2334	1853	1553	1837	1735	1682	1290	1952	2103	1632	640	710	1353	1235	793	1544	244	215	124	238	254	91	118	99	159	241	78	275	77	155	39	188
ENSG00000278799	0	0	0	0	0	0	0	1	1	0	0	0	0	0	0	0	0	0	0	1	0	0	0	0	0	0	0	0	0	0	0	0
ENSG00000145040	5	2	4	4	4	7	5	5	7	9	34	9	4	6	4	3	4	0	1	4	4	8	5	6	0	8	3	5	0	5	5	3
ENSG00000114270	223	241	285	304	395	577	400	602	217	232	879	168	116	131	115	177	78	82	80	216	345	231	216	216	53	103	94	269	25	92	43	90
ENSG00000010256	4021	2976	3561	3045	4174	4149	3084	4351	3157	2533	8072	1981	2815	2616	2701	3434	1719	2882	2864	3014	6587	2580	5234	2322	2417	4340	1529	5510	2448	2695	2292	3087
ENSG00000183396	16	8	14	16	11	8	14	21	1	5	5	3	1	1	1	2	3	2	6	19	21	4	8	6	2	6	2	3	0	1	0	1
ENSG00000225697	1480	1123	1359	1070	1631	1913	1417	1971	843	728	2748	554	299	331	281	420	373	295	276	664	1982	964	1187	889	453	762	577	1658	122	215	102	290
ENSG00000008300	2928	2606	2954	2655	712	800	566	961	1395	1246	3266	828	1466	1493	1302	1928	976	1258	1199	3498	756	388	489	356	1138	1997	700	2932	568	1202	659	1314
ENSG00000228350	24	23	14	34	13	17	10	13	19	23	37	10	33	24	19	32	3	3	1	27	11	4	11	4	13	27	6	31	6	21	15	16
ENSG00000271973	33	25	29	18	18	26	22	7	16	17	37	8	9	12	9	9	4	5	6	26	17	4	8	8	11	11	12	41	4	11	3	12
ENSG00000213672	1713	1404	1422	1263	822	858	554	943	1299	1065	2689	764	814	858	745	1083	330	458	352	768	784	297	506	291	710	1096	403	1779	465	598	404	715
ENSG00000068745	2883	2045	2378	1949	1863	1851	1378	1983	1208	925	5576	790	1361	1191	1364	1629	1965	2274	2109	1891	3997	1580	2712	1535	1028	1532	1441	2852	1223	1485	1393	1620
ENSG00000114302	80	80	73	67	809	858	692	862	1618	1227	6019	943	1824	1552	1806	1890	19	37	31	44	1622	597	1334	528	1561	2518	1439	4174	1709	1907	1823	1846
ENSG00000224424	6	3	6	3	7	12	7	8	38	17	91	17	26	17	20	20	1	3	0	2	8	6	3	2	13	26	22	58	5	3	10	15
ENSG00000178537	471	273	353	273	478	443	241	428	548	383	3125	393	244	196	317	314	297	333	401	376	852	323	682	317	663	978	715	1740	321	333	470	434
ENSG00000221883	42	35	51	42	18	8	5	16	26	29	139	23	10	11	17	25	18	25	19	35	22	9	10	9	35	21	37	106	17	12	6	17
ENSG00000177479	3477	2139	2938	2405	1669	1734	1342	1940	2456	1821	9280	1604	2321	1987	2665	2712	1634	2245	2045	2504	3538	1471	2692	1368	2307	3658	2036	6065	2107	2541	2385	2846
ENSG00000235236	3	1	2	4	1	1	3	7	9	11	30	2	5	6	3	7	6	2	1	7	10	5	3	2	8	8	10	21	2	8	11	14
ENSG00000223343	1	1	2	2	14	13	11	8	19	9	51	6	9	11	14	18	0	0	0	0	13	13	15	5	7	20	12	19	7	17	11	17
ENSG00000273211	0	0	0	0	0	0	0	0	0	0	0	0	0	0	0	0	0	0	0	0	0	0	0	0	0	0	0	0	0	0	0	0
ENSG00000178467	3	4	7	4	628	664	472	659	654	708	2822	537	1100	994	1009	1177	3	0	3	8	742	381	552	368	491	840	619	1645	677	917	742	1003
ENSG00000178252	4597	3457	3846	3702	5247	6074	4096	6085	3827	3327	14412	2537	2514	2539	2151	3270	1557	2112	1773	3470	7705	3824	5125	3619	2710	4523	3401	8122	1365	2386	1433	2796
ENSG00000178149	356	264	394	254	187	231	176	228	386	317	1130	253	404	346	399	483	197	258	208	250	381	217	313	186	321	464	224	651	260	293	292	389
ENSG00000199032	0	0	1	2	1	1	3	3	1	0	3	0	0	1	2	2	5	2	0	0	2	3	3	6	1	0	0	2	2	1	5	0
ENSG00000178057	2436	1720	2216	1868	790	768	493	809	600	491	2156	486	443	397	488	546	2931	4017	3545	2790	1459	558	1018	558	589	898	437	1406	462	428	634	584
ENSG00000207605	0	0	0	0	0	5	1	3	0	0	4	0	2	0	1	1	0	0	0	0	7	0	1	1	0	0	1	0	0	0	0	0
ENSG00000178035	8798	7173	7102	7063	3952	3993	3002	4412	7048	5316	17215	4133	7578	7334	7968	9707	6738	10839	8608	7407	7311	2959	6268	2440	5292	8252	3122	11631	7271	7598	8644	8668
ENSG00000272434	7	2	0	2	8	10	4	8	1	0	15	3	0	3	3	3	2	0	3	5	12	5	6	4	4	7	3	14	3	1	0	7
ENSG00000198218	3586	2644	3085	2660	2299	2327	1647	2318	1883	1509	6425	1230	1590	1491	1495	1805	1196	1384	1431	1950	3342	1455	2467	1281	1649	2469	1645	4450	1048	1320	1105	1444
ENSG00000241461	1	4	3	0	5	5	2	8	6	4	11	1	1	1	2	2	0	2	1	3	2	2	11	1	5	4	6	13	1	2	0	6
ENSG00000172053	2836	2200	2387	2027	3604	3823	2736	3802	4064	3320	13525	2566	4069	3743	4089	5144	2146	2441	2052	1951	6935	2727	5168	2185	3266	5137	2963	8671	3743	4265	4244	4819
ENSG00000172046	2192	1490	1774	1510	1910	2216	1470	2114	1512	1235	6510	1036	1657	1493	1620	1869	596	734	660	1259	3145	1467	2342	1387	1239	2057	1651	3854	1073	1464	1204	1506
ENSG00000172037	2	6	2	1	0	1	1	4	0	2	1	0	4	7	8	7	0	1	5	2	1	0	4	1	0	2	0	0	1	4	9	4
ENSG00000270441	86	84	56	82	31	36	35	25	37	46	139	24	99	94	64	115	4	15	5	21	38	12	28	16	29	30	40	94	20	37	24	28
ENSG00000177352	642	512	651	456	360	410	253	372	475	372	1633	326	284	296	314	399	336	471	498	531	517	246	482	211	372	533	295	891	203	276	222	298
ENSG00000185909	130	102	160	110	2	2	2	3	10	6	24	10	14	24	8	19	58	87	108	105	5	0	3	5	2	3	7	17	2	6	1	9
ENSG00000236980	0	0	0	2	0	0	0	0	0	0	0	0	0	0	0	0	0	0	0	1	0	0	0	0	0	0	0	0	0	0	0	1
ENSG00000173421	6	2	10	6	4	8	5	5	4	10	29	4	8	3	9	8	0	0	2	15	10	8	10	3	3	7	5	18	0	6	12	13
ENSG00000270538	0	0	0	2	2	1	0	0	2	0	4	0	1	0	0	0	0	0	0	0	0	0	0	0	0	0	2	1	0	0	0	0
ENSG00000225399	4	2	4	2	1	4	2	4	6	2	13	6	3	1	3	9	1	1	0	1	6	4	5	0	2	5	9	5	4	1	4	4
ENSG00000188315	365	267	407	328	384	429	313	411	350	283	1943	289	219	210	279	285	133	131	156	378	804	311	506	299	330	458	427	1019	166	302	234	321
ENSG00000199546	0	0	0	0	0	0	0	0	0	0	0	0	0	0	0	0	0	0	0	0	0	0	0	0	0	0	0	0	0	0	0	0
ENSG00000264633	0	0	0	0	0	0	0	0	0	0	0	0	0	0	0	0	0	0	0	0	0	0	0	0	0	0	0	0	0	0	0	0
ENSG00000114316	2425	1567	2051	1705	1587	1753	1252	1810	1249	952	5240	861	1104	885	1095	1106	745	1007	899	1297	2999	1292	2117	1125	1155	1856	1233	3500	717	971	852	1081
ENSG00000233276	2045	1497	1702	1445	1135	1174	786	1201	1105	834	4873	862	934	792	968	1175	2548	4190	3272	2603	2926	1125	2367	968	1065	1604	856	2852	1669	1303	1783	1477
ENSG00000067560	11315	8183	9502	8189	11892	11345	8201	12102	10534	7901	35267	6454	9161	7784	9181	10346	6446	8086	7557	7386	21085	7642	14251	7310	10473	15444	7829	25153	6937	7592	7403	8427
ENSG00000235908	8	15	20	10	12	15	16	22	10	17	42	9	14	8	10	7	1	3	3	12	11	9	7	6	7	10	9	36	6	11	6	10
ENSG00000145022	242	161	293	180	212	163	153	194	678	535	2960	451	246	261	255	329	115	215	246	292	303	163	276	119	593	894	741	1778	239	270	302	309
ENSG00000145020	51	46	68	53	26	51	27	33	63	40	205	29	50	39	41	37	13	14	13	33	34	22	14	26	23	42	44	121	8	27	13	36
ENSG00000283189	56	45	43	40	30	40	22	26	41	44	146	25	46	33	17	43	18	11	6	16	24	20	24	36	19	35	28	63	6	22	9	20
ENSG00000145029	0	2	2	2	3	2	0	0	1	0	8	1	1	2	0	0	0	0	0	0	2	0	0	0	0	0	2	5	0	1	0	0
ENSG00000173402	801	684	773	615	518	526	347	524	537	416	2248	361	661	587	548	779	174	290	236	493	784	364	536	295	578	915	523	1534	373	450	384	561
ENSG00000226913	28	28	25	35	3	1	0	1	0	1	3	2	4	4	4	8	20	8	19	31	5	2	0	1	2	2	5	5	4	2	1	5
ENSG00000164061	285	331	333	325	169	214	121	195	86	87	522	72	224	206	194	251	79	117	88	367	188	113	128	109	64	83	98	318	94	210	122	252
ENSG00000235120	0	0	1	0	0	0	0	0	0	0	0	0	1	0	2	0	0	0	0	1	0	0	0	0	0	1	0	0	0	0	0	1
ENSG00000164062	2982	2275	2282	2287	3876	3930	2766	3923	2692	2072	8377	1803	2908	2549	2942	3519	1206	2007	1713	1992	5842	2632	4728	2183	2343	4028	1606	5607	2588	2725	2709	2939
ENSG00000173531	477	494	588	468	485	582	416	550	136	136	741	77	48	58	77	82	191	193	161	370	560	281	374	327	90	126	232	380	39	80	53	101
ENSG00000259970	2	0	5	1	0	3	1	3	3	0	3	0	2	0	0	0	1	3	0	4	1	2	1	2	1	2	1	6	0	3	1	3
ENSG00000164068	1168	885	1012	982	801	918	648	945	742	667	3679	615	1027	864	939	1141	356	387	289	748	1102	550	813	522	650	1281	891	2463	582	907	614	1056
ENSG00000176020	8	2	6	2	2	1	3	3	3	4	12	1	4	3	2	0	0	0	1	3	8	2	5	1	2	2	6	11	2	4	4	1
ENSG00000173540	1191	805	1133	877	482	514	413	512	668	564	2273	467	377	343	395	465	396	576	484	869	887	402	694	363	595	996	415	1671	279	449	321	436
ENSG00000176095	970	682	928	600	922	931	696	958	813	674	3472	628	766	667	969	925	298	481	456	726	1594	699	1228	652	775	1322	900	2222	894	1000	904	1136
ENSG00000239576	1	2	2	6	0	0	1	1	2	4	7	1	3	1	3	2	0	2	2	1	6	3	1	1	1	5	8	14	0	2	4	3
ENSG00000244730	3	0	6	1	4	8	2	2	2	2	19	4	5	1	1	2	0	0	5	9	7	4	3	2	6	3	8	25	1	3	6	3
ENSG00000187492	2	3	5	2	14	8	8	4	2	2	6	0	2	2	0	5	1	1	2	6	13	6	8	8	0	5	7	0	0	5	0	2
ENSG00000185614	60	64	64	61	281	323	238	290	6	6	86	12	19	27	47	44	46	72	62	39	767	341	718	269	12	20	25	86	27	35	45	45
ENSG00000182179	74	62	83	82	1324	1384	900	1410	1045	932	5048	745	856	731	757	1005	24	30	25	60	1429	650	1170	551	611	845	1058	2394	386	705	425	756
ENSG00000183763	845	647	789	647	650	777	549	783	427	319	1143	241	416	384	534	544	384	394	380	685	1127	523	828	486	340	599	150	928	361	492	426	578
ENSG00000164076	140	291	103	260	0	0	0	0	5	4	5	5	175	148	152	173	47	170	50	264	0	0	0	0	3	7	0	4	52	105	91	95
ENSG00000264706	0	0	0	0	0	0	0	1	0	0	3	0	0	0	0	0	0	0	0	0	0	0	0	0	0	0	1	1	0	0	0	0
ENSG00000234667	0	0	0	0	0	0	0	0	0	0	0	0	7	4	6	7	0	0	0	1	1	0	0	0	0	0	0	0	2	5	3	14
ENSG00000164078	10	8	9	5	1	3	1	0	4	2	17	5	1023	930	1072	1234	3	11	7	5	5	0	3	2	7	4	7	16	560	995	600	1219
ENSG00000230698	0	0	0	0	1	0	0	1	0	0	1	0	2	3	11	4	0	0	0	0	2	1	0	0	0	0	0	0	4	5	7	9
ENSG00000228008	3	2	2	2	1	3	3	3	3	4	13	4	16	12	11	20	0	0	1	2	16	1	6	2	0	3	6	11	4	6	6	6
ENSG00000164077	293	199	264	196	295	273	225	332	251	183	966	157	230	187	309	260	119	233	202	234	602	238	451	216	296	411	191	599	323	308	312	359
ENSG00000004534	3392	2521	2675	2643	5159	5442	3807	5569	2368	1759	8575	1406	2269	2180	2269	2830	1260	1174	1006	2064	7046	3197	4571	3087	1911	2901	1940	5209	1324	2305	1541	2485
ENSG00000003756	3156	2285	2825	2406	3920	4325	3145	4681	2871	2412	12844	1992	1608	1590	1782	2128	968	950	1046	2091	6587	3080	4155	3202	2514	3473	3731	7594	1019	1909	1344	2166
ENSG00000281691	32	25	38	35	38	38	36	38	35	15	130	17	13	14	16	25	7	12	4	38	64	22	33	39	28	25	40	80	6	27	13	26
ENSG00000235016	88	44	59	65	108	118	104	114	109	86	480	48	36	35	36	68	24	16	24	71	204	90	116	105	63	104	92	273	22	53	26	53
ENSG00000001617	77	88	57	84	17	19	9	21	59	45	103	34	51	48	28	57	4	13	6	23	13	2	6	5	4	18	12	29	9	23	6	14
ENSG00000207922	0	0	0	0	0	0	0	0	0	0	0	0	0	0	0	0	0	0	0	0	0	0	0	1	0	0	0	0	0	0	0	0
ENSG00000114349	0	0	0	5	1	1	0	0	0	0	0	0	0	1	1	2	0	0	0	1	0	0	0	0	0	0	0	0	0	0	0	1
ENSG00000188338	21	49	19	56	0	0	0	0	0	4	5	2	0	3	0	1	7	7	1	17	1	0	0	0	0	0	3	6	0	0	2	1
ENSG00000114353	9960	6963	8264	7091	8203	7938	5543	7909	7675	6017	28006	4988	4502	4158	4593	5552	2405	3831	4224	5138	11823	4755	8805	3773	6734	11294	6236	18412	3886	4415	3859	4783
ENSG00000275334	2	0	1	1	1	6	1	1	0	0	0	0	1	0	1	2	0	0	0	3	3	2	1	0	0	0	0	1	3	1	1	2
ENSG00000213600	11	8	9	10	3	10	5	16	15	9	36	3	3	4	3	11	3	3	6	16	20	10	11	7	2	8	8	23	3	7	4	8
ENSG00000230454	45	36	50	41	74	78	87	98	34	31	118	27	32	36	23	43	8	5	8	45	116	57	62	78	17	16	44	70	8	21	7	18
ENSG00000232352	3	6	7	6	16	14	19	20	4	4	28	2	8	7	3	10	0	1	3	3	29	10	10	11	1	6	5	10	0	2	3	6
ENSG00000012171	24	36	43	48	82	102	65	101	24	20	139	11	47	56	27	50	5	10	14	27	114	80	63	58	5	16	33	55	8	34	13	26
ENSG00000179564	7	5	13	3	3	12	6	11	13	14	39	4	15	20	31	33	5	8	0	18	20	12	5	19	22	26	8	39	14	22	22	29
ENSG00000214706	1459	957	1338	1034	1107	1140	760	1127	1123	860	3327	710	2003	1688	2141	2291	907	1379	1271	1766	1936	839	1637	782	1153	1806	556	2188	2126	2242	2096	2306
ENSG00000186792	68	55	82	57	194	208	161	251	68	51	183	49	85	104	84	136	75	124	101	141	292	137	238	115	45	74	42	95	91	97	103	141
ENSG00000243477	287	229	234	285	279	268	212	303	195	173	584	127	188	164	141	206	115	120	84	194	293	150	214	140	91	168	158	352	87	114	66	127
ENSG00000114378	66	43	39	33	31	27	22	31	26	16	180	23	24	11	17	12	14	13	10	40	34	19	16	22	20	33	40	107	7	21	7	7
ENSG00000068001	271	189	259	217	564	514	348	519	173	118	596	128	212	180	197	251	278	389	372	330	1110	358	774	327	129	234	92	331	242	247	265	250
ENSG00000114383	698	428	597	496	451	446	283	435	374	287	1488	273	621	486	620	619	571	641	644	576	834	350	670	306	390	581	333	963	649	626	727	719
ENSG00000068028	835	649	760	671	2072	2113	1600	2469	2500	2141	7605	1703	696	625	711	920	328	449	540	622	3293	1564	2419	1381	1848	2703	1188	4291	530	690	543	870
ENSG00000281358	7	8	7	14	68	73	65	70	102	88	326	48	16	22	17	16	9	8	3	15	108	68	69	49	59	83	48	169	9	14	7	14
ENSG00000235058	1	0	0	0	0	0	0	1	0	0	0	0	0	0	0	1	0	0	0	0	0	0	0	0	0	0	0	0	0	1	0	0
ENSG00000004838	91	97	91	85	113	92	91	153	121	72	318	51	50	59	61	88	55	37	72	57	172	125	126	95	70	109	79	213	46	81	33	97
ENSG00000114388	948	655	761	678	536	599	460	641	791	590	2407	511	569	461	469	651	698	838	719	708	1079	451	822	421	591	899	551	1508	403	524	372	611
ENSG00000114395	315	188	283	211	264	285	188	252	279	214	942	177	170	189	206	240	311	473	468	353	508	255	434	209	260	412	180	591	263	255	267	267
ENSG00000272104	1	0	0	0	0	0	0	0	0	0	0	2	1	0	0	0	0	0	0	0	0	0	0	0	0	0	0	0	0	0	0	0
ENSG00000126062	417	264	361	254	425	430	312	522	342	332	1443	299	397	379	450	473	196	286	314	351	751	346	610	335	306	529	379	993	439	434	471	495
ENSG00000007402	56	170	76	145	37	53	26	62	22	20	79	22	29	24	27	35	15	35	10	65	29	20	16	31	10	23	14	44	3	17	5	18
ENSG00000271858	27	29	37	31	8	8	7	5	15	11	29	10	23	15	13	10	11	14	12	14	7	5	2	1	1	3	7	10	4	10	3	9
ENSG00000088543	219	158	197	204	158	184	114	179	1545	1129	3152	859	40	37	34	61	68	114	80	160	224	125	186	99	577	1104	395	1489	31	55	21	36
ENSG00000114735	967	837	1000	784	543	692	515	786	3579	2748	7125	1751	483	459	490	636	334	365	424	785	831	431	572	388	1295	2303	885	3510	261	473	313	581
ENSG00000280382	1	1	0	0	1	0	0	0	18	12	45	16	1	2	3	6	0	0	0	0	4	1	0	0	12	9	5	22	1	3	0	1
ENSG00000114737	137	92	125	90	46	67	43	60	6590	5297	18609	4305	1017	969	945	1356	64	58	62	94	100	54	67	41	4052	7360	2994	10832	556	729	441	840
ENSG00000114738	2458	1646	2034	1733	3351	3189	2205	3361	2465	1733	6556	1460	2378	2071	2362	2844	1335	2013	1822	1965	6164	2420	4775	2184	1815	3054	1010	4128	2511	2675	2328	3012
ENSG00000273356	28	33	24	23	14	19	10	17	5	5	45	6	8	8	5	8	6	14	7	17	37	11	9	13	7	15	19	35	2	10	4	9
ENSG00000272543	4	14	8	10	0	0	0	0	4	7	44	4	5	2	1	2	5	6	9	3	0	0	0	0	8	14	6	26	0	11	1	3
ENSG00000088538	692	747	723	796	16	23	17	20	864	919	5679	849	229	309	275	424	159	267	170	642	21	17	21	11	788	1402	1630	4482	120	358	154	379
ENSG00000228337	3	0	1	0	0	0	0	1	2	0	8	0	0	0	0	0	0	0	0	0	0	0	0	1	1	1	2	7	0	0	0	0
ENSG00000213587	1	0	0	1	0	0	0	0	2	2	6	0	0	0	2	0	0	0	0	5	0	0	0	0	0	2	3	10	0	0	1	0
ENSG00000235278	1	2	1	3	0	0	0	1	1	0	7	0	0	1	0	1	0	0	1	2	0	0	0	0	4	6	2	16	0	0	0	2
ENSG00000240959	0	0	0	0	0	0	0	0	0	1	2	0	0	0	0	0	0	0	0	0	0	0	0	0	4	2	0	10	0	1	3	1
ENSG00000145050	1189	893	1105	933	2791	2907	1843	3108	1802	1215	5420	1067	815	786	866	931	1547	1986	1779	1416	4761	1629	2430	1360	1656	2346	1103	3546	970	938	1070	1211
ENSG00000259956	2831	2136	2787	2114	2616	2538	1836	2618	1791	1490	7191	1295	1283	1150	1453	1515	880	1365	1428	2333	4039	1759	3177	1520	1667	2836	1447	4372	1156	1400	1141	1493
ENSG00000145041	3124	2053	2420	2057	2326	2482	1777	2543	2151	1652	6720	1312	2003	1684	2057	2265	929	1056	1091	1904	3818	1670	2797	1518	2067	3343	1456	4987	1527	1945	1590	2039
ENSG00000164080	3112	2566	2971	2604	2808	2979	2332	3055	1677	1343	7040	1125	1827	1578	1978	1992	750	998	881	2266	4419	2064	3016	1787	1483	2495	1772	4843	1194	1713	1467	2023
ENSG00000221015	0	0	0	0	0	0	1	0	0	0	0	0	0	0	0	1	0	0	0	1	0	4	2	1	2	1	0	9	0	0	0	0
ENSG00000164081	992	644	953	703	1016	1051	692	1030	640	530	1990	405	628	607	643	787	631	956	1006	947	2320	1039	1761	863	544	807	350	1171	588	594	740	677
ENSG00000164082	11	9	10	14	12	13	12	27	7	5	32	9	12	5	9	9	2	4	1	13	26	10	13	13	10	7	2	19	1	5	8	9
ENSG00000214686	0	0	0	0	0	0	0	0	0	0	0	0	0	2	0	1	0	0	0	0	0	0	0	0	0	0	0	1	0	0	0	0
ENSG00000224792	0	0	0	0	0	0	0	0	0	0	0	0	1	0	0	0	0	0	0	0	0	0	0	0	0	0	0	0	0	1	1	0
ENSG00000229972	0	0	0	0	0	0	0	0	0	0	0	0	1	1	1	2	0	0	0	0	0	0	0	0	0	0	2	0	0	3	0	0
ENSG00000184345	0	0	0	0	0	0	0	0	0	0	0	0	0	0	0	0	0	0	0	0	0	0	0	0	1	0	0	0	0	0	0	0
ENSG00000241789	0	0	0	0	0	0	0	0	0	0	0	0	0	0	0	0	0	0	0	0	0	0	0	0	0	0	0	0	0	0	0	0
ENSG00000235455	0	0	0	0	0	0	0	0	0	0	0	0	1	0	0	0	0	0	0	1	0	0	0	0	0	0	0	0	0	0	0	0
ENSG00000214681	0	0	0	0	0	0	0	0	0	0	0	0	0	0	0	0	0	0	0	0	0	0	0	0	0	0	0	0	0	0	0	0
ENSG00000173389	1	2	4	4	0	0	0	0	0	2	0	0	0	0	0	1	1	0	0	3	0	0	0	0	1	0	0	0	0	6	0	3
ENSG00000114767	1122	708	939	772	683	716	462	733	1044	780	3222	675	1787	1419	1801	1817	547	1007	905	1278	1273	560	1083	482	968	1759	634	2150	1910	1900	2052	2168
ENSG00000041880	489	304	571	372	806	798	617	820	474	374	1948	313	398	363	463	462	208	223	321	415	1115	502	862	528	347	526	375	1043	300	337	323	434
ENSG00000280422	2	0	7	4	6	7	3	9	6	15	60	2	0	1	1	3	2	1	0	2	12	4	5	7	3	6	9	17	0	4	3	2
ENSG00000180929	0	2	4	1	7	8	2	2	0	2	19	8	0	0	0	0	1	0	2	4	7	7	9	3	7	4	1	6	0	0	0	3
ENSG00000090097	723	580	651	549	675	655	440	731	578	453	2369	392	63	80	48	93	255	472	389	524	1044	443	716	457	420	770	563	1466	26	46	23	36
ENSG00000272762	0	0	0	1	0	0	0	1	1	0	0	4	0	0	0	0	0	0	1	0	0	0	0	0	0	0	0	2	0	0	0	0
ENSG00000114779	777	705	667	645	1577	1583	1081	1732	1972	1542	4992	1044	1198	997	919	1404	397	593	416	478	2402	918	1782	854	1368	2068	1084	3111	748	894	706	973
ENSG00000248487	161	126	108	122	244	245	140	258	254	169	585	171	175	157	173	220	56	86	95	126	309	139	247	126	147	249	119	428	153	180	101	157
ENSG00000114786	6	10	5	1	18	23	22	19	14	17	41	11	12	7	7	8	5	7	2	5	24	20	19	10	13	13	16	36	4	17	5	10
ENSG00000243989	125	84	91	83	101	105	74	94	163	141	494	122	119	109	107	182	44	68	43	102	175	69	110	72	113	183	91	337	64	98	75	129
ENSG00000162244	13272	9330	8658	8885	9687	9110	6852	9553	19197	14697	53400	12385	15022	14460	15377	18476	15465	19992	13553	14089	22455	8428	15255	10066	15409	22242	9638	30493	19884	16818	21844	22628
ENSG00000164086	1123	774	1020	809	987	912	665	998	597	441	1793	394	1072	923	1258	1263	416	599	652	817	1684	710	1128	644	571	837	429	1369	1152	1229	1218	1381
ENSG00000164087	1115	804	929	833	1008	1052	746	1068	572	443	1457	383	649	617	602	823	411	524	497	638	1550	636	1139	547	572	826	223	1207	518	570	571	678
ENSG00000242849	0	0	0	0	1	0	0	0	0	0	0	0	0	0	0	0	1	0	0	0	2	0	1	0	0	0	0	0	0	2	0	0
ENSG00000023330	1670	1028	1462	1169	1246	1263	1025	1282	917	690	3130	579	1184	940	1265	1306	514	631	775	911	1954	871	1546	738	809	1327	764	2041	1009	1095	1051	1257
ENSG00000280003	9	3	16	4	11	13	0	6	9	3	13	4	5	0	1	3	2	1	0	5	13	8	10	7	8	5	5	4	5	3	1	0
ENSG00000239732	7	9	5	8	13	13	19	9	3	3	16	1	4	8	5	3	4	1	3	4	24	6	13	12	2	3	1	11	3	4	4	4
ENSG00000173366	0	0	0	0	3	0	0	0	0	0	2	0	0	0	0	0	0	0	0	0	0	1	0	0	0	2	0	0	0	0	0	0
ENSG00000247596	1482	1086	1305	1102	1027	1006	660	1084	1492	1146	5035	998	950	860	923	1015	429	752	746	849	1802	800	1423	636	1331	1989	1197	3299	780	707	748	825
ENSG00000243224	91	82	88	80	29	28	13	29	43	38	104	25	47	33	48	51	47	44	50	59	34	18	11	18	19	34	21	57	16	12	9	17
ENSG00000164088	1563	1099	1340	1121	621	694	473	739	253	221	1032	195	160	160	121	173	613	803	740	993	939	458	757	432	240	330	348	737	42	47	44	76
ENSG00000164091	5760	3821	5342	4071	4409	4635	3359	4776	4984	3942	17458	3094	4512	3915	4672	4996	1660	2577	2943	3549	7901	3270	5938	2649	4876	7503	3904	11353	3790	4864	3580	4991
ENSG00000199150	0	1	0	0	0	0	0	1	1	1	7	0	0	1	0	1	0	0	0	1	6	3	4	2	2	3	0	9	0	1	0	0
ENSG00000168237	283	199	216	184	184	257	141	222	122	95	426	80	73	46	46	54	118	149	166	214	324	180	265	155	89	160	93	361	32	61	48	85
ENSG00000242797	15	14	22	12	25	18	19	19	17	8	61	5	9	15	6	8	5	3	8	22	23	18	14	15	6	17	12	39	6	6	13	11
ENSG00000207926	3	3	3	4	5	7	6	9	4	7	26	5	4	1	2	2	3	3	4	9	9	9	13	11	9	3	1	18	0	6	0	2
ENSG00000114841	536	618	537	547	462	645	397	596	664	637	3252	464	430	384	416	548	112	132	99	365	565	312	285	314	403	728	845	2067	149	424	216	475
ENSG00000239557	9	3	2	0	4	4	9	18	1	11	11	2	1	2	0	4	1	0	1	3	13	7	3	3	2	4	7	12	1	1	0	1
ENSG00000163930	4549	3038	4021	3264	3243	3265	2437	3501	2189	1774	7915	1493	1903	1677	1938	2308	1615	1938	2099	3102	4565	2176	3382	1786	1755	2940	1867	5189	1428	1765	1427	2057
ENSG00000010318	827	623	731	607	530	609	465	595	255	249	661	179	405	375	326	498	251	274	348	450	825	330	599	319	184	287	109	578	180	221	168	203
ENSG00000010319	298	199	314	232	121	93	82	113	17	25	46	18	102	90	132	122	131	195	117	322	218	82	193	83	5	24	13	28	56	70	37	62
ENSG00000114854	39	21	49	25	10	13	13	12	9	3	13	1	5	1	4	9	30	14	23	21	25	11	17	11	2	0	3	3	4	3	1	3
ENSG00000010322	4225	3650	3923	3601	4118	4377	2947	4479	2045	1783	8334	1430	1466	1374	1472	1786	1026	1032	879	2791	5519	2646	3231	2474	1402	2286	1966	4726	838	1439	889	1669
ENSG00000010327	5	5	5	8	12	19	24	18	1	0	5	0	0	2	11	1	1	2	1	5	30	11	18	13	3	2	2	5	0	0	2	2
ENSG00000168268	2306	2107	2251	1978	2898	2908	1991	2808	1199	1140	2718	773	986	900	839	1110	904	1319	1097	1842	4727	1936	3473	1587	986	1645	440	1853	670	867	568	928
ENSG00000168273	135	93	138	99	195	162	134	223	135	104	442	100	174	163	201	227	116	116	156	169	528	214	378	264	114	164	94	304	260	221	338	307
ENSG00000163939	5752	3711	5191	3635	4200	4379	3468	4558	3300	2707	12815	2148	2741	2570	3066	3276	1424	2030	2235	3431	7507	3239	5701	2786	3772	5742	2817	10116	1982	2796	2349	3049
ENSG00000252768	1	5	8	2	4	7	4	2	8	6	13	3	4	5	2	3	3	2	2	6	7	1	4	2	3	7	0	6	7	2	1	3
ENSG00000221518	10	1	7	4	3	7	3	5	3	9	10	5	3	2	5	0	0	0	1	8	4	3	11	3	4	2	5	10	2	2	0	8
ENSG00000163938	3331	2220	2793	2253	3675	3687	3087	4126	3278	2353	8999	1755	5835	5136	6703	6660	5321	6575	5325	5183	9944	3977	7178	4406	3002	4409	1750	5904	5683	5724	7484	7431
ENSG00000280904	2	3	0	0	0	5	1	3	1	0	2	0	2	3	5	1	0	0	0	3	1	2	2	2	1	0	0	2	2	3	0	2
ENSG00000252787	0	0	0	0	0	0	0	0	0	0	0	0	0	0	0	0	0	0	0	0	0	0	0	0	0	0	0	0	0	0	0	0
ENSG00000212493	14	11	7	13	29	17	8	25	13	28	56	6	28	25	29	22	8	5	7	14	28	16	13	18	10	16	15	36	6	21	21	36
ENSG00000238862	0	0	0	0	0	0	0	0	0	0	0	0	0	0	0	0	0	0	0	0	0	0	0	0	0	0	0	0	0	0	0	0
ENSG00000222345	18	13	16	10	22	16	17	18	9	17	49	6	25	33	23	25	12	13	9	20	34	13	18	23	29	25	11	23	15	30	16	29
ENSG00000212452	6	7	5	11	11	8	7	9	17	9	37	2	11	16	17	24	5	3	5	1	18	13	12	16	9	5	5	18	13	14	12	33
ENSG00000016864	1107	726	825	727	800	809	605	882	961	821	2883	576	769	770	804	944	462	641	540	721	1566	624	1160	629	796	1093	680	1723	702	831	677	920
ENSG00000114902	1741	1239	1465	1086	1223	1215	1088	1439	1231	746	3547	675	1082	887	1128	1277	2407	2963	2572	2104	3902	1454	3039	1673	1211	1725	755	2427	1377	1248	1823	1719
ENSG00000114904	1268	862	1164	967	1185	1265	1029	1366	861	637	2781	530	921	834	1119	1143	453	634	672	962	2609	1082	2017	1029	856	1274	595	1994	876	1130	1087	1184
ENSG00000275956	18	21	18	17	11	21	15	10	20	10	56	9	13	7	13	15	5	2	2	18	39	18	25	23	11	30	15	55	1	16	8	12
ENSG00000270941	2	3	8	2	4	5	5	6	3	4	21	1	4	3	2	4	2	1	2	8	18	7	9	3	2	5	3	17	0	3	5	5
ENSG00000271137	7	4	7	4	4	7	2	7	4	2	5	2	1	0	1	0	2	2	1	3	1	9	2	3	1	9	4	15	0	2	2	4
ENSG00000055957	1	1	1	1	1	1	0	0	1	0	1	0	3	2	8	4	0	0	0	0	0	0	1	1	0	0	0	0	1	0	0	1
ENSG00000162267	10	8	11	14	8	7	2	10	5	4	27	2	1	0	2	1	5	3	4	8	6	3	4	2	0	1	3	11	0	1	1	0
ENSG00000055955	8	22	10	21	27	18	42	25	24	21	95	8	8	2	3	10	12	7	2	17	27	29	11	15	4	21	29	66	3	3	2	11
ENSG00000243696	80	79	66	89	55	65	51	93	38	46	230	25	27	18	14	25	27	14	7	65	83	45	28	47	31	66	37	141	8	28	9	52
ENSG00000239799	0	1	3	1	3	0	1	0	2	1	6	3	1	1	1	0	0	1	0	3	1	0	1	3	4	1	7	12	0	1	2	1
ENSG00000272573	16	5	16	14	6	4	6	4	1	7	17	2	2	2	4	5	3	1	1	5	9	1	3	3	0	2	4	11	1	2	1	4
ENSG00000248592	81	90	86	104	78	86	63	90	48	41	151	35	51	47	37	60	26	30	13	90	86	42	58	48	24	67	38	132	14	40	21	60
ENSG00000213533	145	117	152	121	115	164	103	118	77	55	305	72	69	58	69	85	68	69	58	135	179	89	120	85	71	126	78	225	62	71	45	88
ENSG00000275789	1	2	0	0	0	1	1	0	0	1	3	0	1	0	0	0	1	0	0	3	6	0	3	0	0	2	1	2	1	1	0	2
ENSG00000279144	4	12	11	5	8	6	1	3	3	0	5	2	2	2	0	2	1	1	1	9	10	2	10	6	3	2	2	9	2	1	1	2
ENSG00000163935	1252	1050	1204	1130	748	850	554	861	875	646	3112	638	745	598	647	739	427	589	651	923	1377	627	1092	575	763	1295	653	2636	469	658	497	709
ENSG00000272305	29	32	33	41	21	11	12	22	17	18	71	4	21	21	22	37	3	13	10	23	35	22	18	12	20	39	20	47	7	21	11	21
ENSG00000280417	30	37	39	33	5	0	3	1	14	8	65	10	21	22	21	19	17	39	19	38	4	1	2	0	11	20	19	36	7	27	12	21

ENSG00000242142	4	6	5	2	0	1	0	2	1	5	14	0	3	2	4	2	0	0	0	1	0	0	0	0	0	1	0	6	0	4	0	6
ENSG00000163933	815	606	659	548	799	763	553	756	626	507	1976	397	665	661	758	854	616	1007	744	727	1357	565	961	523	514	815	391	1226	679	742	823	885
ENSG00000163932	273	240	270	246	230	249	166	216	371	352	1133	279	823	739	733	919	45	105	78	163	360	138	204	118	242	468	186	1059	483	595	458	654
ENSG00000163931	10389	7376	7027	6965	5989	5743	4231	5836	7018	5400	19361	4121	10078	9037	8861	11505	1881	3337	2809	4527	7821	3179	6017	2773	5822	9411	4389	12962	6790	7513	6376	8017
ENSG00000272886	1368	1014	1107	975	1206	1225	962	1403	1723	1275	5039	902	1541	1421	1715	1814	472	619	594	853	2291	927	1543	761	1484	2241	1165	3475	1264	1619	1371	1747
ENSG00000274967	4	2	2	6	1	3	4	0	6	4	6	4	1	1	3	3	0	0	1	1	1	3	5	6	5	3	2	6	0	0	2	0
ENSG00000157388	5	2	3	3	4	6	1	4	29	21	112	26	2	3	1	6	2	2	3	6	8	6	4	0	15	26	44	113	2	2	6	2
ENSG00000207109	1	0	1	2	0	1	2	0	1	0	3	1	0	2	0	0	0	1	0	4	0	0	2	0	0	1	2	1	0	0	1	0
ENSG00000212608	0	0	0	0	0	0	0	0	0	0	0	0	0	0	0	0	0	0	0	0	0	0	0	0	0	0	0	0	0	0	0	0
ENSG00000227224	0	0	0	0	0	0	0	0	0	0	0	0	0	0	0	0	0	0	0	0	0	0	0	0	0	0	0	0	0	0	0	0
ENSG00000251987	0	0	0	0	0	0	0	0	0	0	0	0	0	0	0	0	0	0	0	0	0	0	0	0	0	0	0	1	0	0	0	0
ENSG00000271916	0	0	0	0	0	0	0	0	0	1	2	0	0	0	0	0	0	0	0	0	0	0	0	0	1	0	0	4	0	0	0	0
ENSG00000016391	0	0	0	0	4	2	0	4	5	1	7	0	1	0	0	0	0	2	0	0	1	3	4	3	0	1	1	7	0	0	0	0
ENSG00000056736	71	32	66	44	147	147	87	144	126	99	516	83	97	90	77	115	22	30	33	74	263	110	191	108	107	133	141	316	66	84	88	86
ENSG00000271976	104	65	85	68	134	129	91	138	128	95	530	76	89	92	99	117	25	50	40	96	194	102	170	79	125	122	133	286	49	110	52	79
ENSG00000113812	938	593	730	681	1004	1180	883	1154	829	657	3532	552	781	745	848	878	444	616	609	742	2591	1092	1975	957	836	1090	913	2117	741	867	835	940
ENSG00000113811	778	524	522	580	390	473	301	499	651	439	2477	401	457	480	642	683	822	1164	923	975	916	447	714	428	690	924	632	1659	676	692	937	856
ENSG00000242983	0	0	0	0	0	0	0	0	0	0	0	0	0	0	0	0	0	0	0	0	0	0	0	0	0	0	0	0	0	0	0	0
ENSG00000157445	0	0	0	0	0	0	0	0	0	0	3	0	2	3	2	7	0	0	0	0	0	0	1	0	0	0	1	0	0	5	3	2
ENSG00000229806	0	1	0	0	0	0	0	0	0	1	1	0	0	0	0	0	1	0	0	0	0	0	0	0	0	0	0	0	0	1	0	0
ENSG00000265992	0	0	0	0	0	0	0	0	0	0	0	0	0	0	0	0	0	0	0	0	0	0	0	0	0	0	0	0	0	0	0	0
ENSG00000243715	0	0	0	0	0	0	0	0	0	0	0	0	0	0	0	0	0	0	2	0	0	0	0	0	0	0	0	1	0	0	2	1
ENSG00000144771	0	0	0	0	0	0	0	0	0	0	3	0	5	4	6	7	0	0	0	0	0	0	0	0	0	0	0	0	3	4	12	5
ENSG00000239991	0	0	0	0	0	0	0	0	0	0	0	0	0	0	0	0	0	0	0	2	0	0	0	0	0	1	0	1	0	0	0	0
ENSG00000243572	0	0	0	0	0	0	0	0	0	0	0	0	0	0	0	0	0	0	0	0	0	0	0	0	0	0	0	0	0	0	0	0
ENSG00000240708	0	0	0	0	0	0	0	0	0	0	0	0	0	0	0	0	0	0	0	0	0	0	0	0	0	0	0	0	0	0	0	0
ENSG00000242568	0	0	0	0	0	0	0	0	0	0	0	0	0	0	0	0	0	0	0	0	0	0	0	0	0	0	0	0	0	0	0	0
ENSG00000242775	0	0	0	0	0	0	0	0	0	0	1	0	0	0	0	0	0	0	0	0	0	0	0	0	0	0	0	0	0	0	0	0
ENSG00000114251	0	27	8	36	30	58	14	45	0	0	0	0	12	19	14	22	1	7	2	12	59	17	33	17	0	0	1	1	4	37	19	40
[illegible]																																

ENSG00000243422	2	1	4	4	6	6	5	7	7	8	38	9	0	0	0	0	1	1	5	1	8	4	8	2	7	6	13	15	0	0	0	0
ENSG00000282912	2	0	0	0	3	0	0	1	3	0	3	0	0	0	0	0	1	0	0	1	1	0	1	0	0	1	0	4	0	0	0	0
ENSG00000282511	0	3	3	1	11	12	11	17	4	7	21	7	0	0	0	0	0	0	2	2	14	2	10	10	6	11	7	29	0	0	0	0
ENSG00000241532	3	5	2	9	7	21	10	13	11	6	30	12	0	0	0	0	0	0	1	9	22	11	10	13	4	9	6	16	0	0	0	0
ENSG00000241622	5	6	4	0	8	12	5	7	8	5	26	6	0	0	0	0	5	0	1	1	8	4	7	3	4	3	7	19	0	0	0	0
ENSG00000172969	0	0	0	0	0	1	0	0	0	0	2	0	0	0	0	0	0	0	0	0	1	3	0	0	0	0	0	2	0	0	0	0
ENSG00000236138	0	0	0	0	4	6	2	4	0	0	3	0	2	0	1	2	0	0	0	0	18	2	6	9	1	0	2	1	0	0	2	0
ENSG00000242516	0	0	0	0	219	235	171	248	77	67	243	55	0	0	0	0	0	0	0	0	428	232	299	174	71	101	59	150	0	0	0	1
ENSG00000227124	0	0	0	0	1	6	0	3	10	5	23	9	17	11	18	24	0	0	0	0	1	2	2	1	1	7	4	20	4	12	13	8
ENSG00000266396	0	0	0	0	0	0	0	0	0	0	0	0	0	0	0	0	0	0	0	0	0	0	0	0	0	0	0	0	0	0	0	0
ENSG00000185008	0	0	0	0	164	155	151	191	0	0	0	0	1	1	1	2	0	0	0	0	142	74	117	52	0	0	2	0	0	0	0	1
ENSG00000222883	0	0	0	0	0	0	0	0	0	0	0	0	0	0	0	0	0	0	0	0	0	0	0	0	0	0	0	0	0	0	0	0
ENSG00000240809	11	4	5	3	3	0	1	0	2	1	6	1	1	3	0	2	0	1	2	5	1	2	3	1	3	0	2	7	1	2	1	2
ENSG00000199520	0	0	0	0	0	0	0	0	0	0	0	0	0	0	0	0	0	0	0	0	0	0	0	0	0	0	0	0	0	0	0	0
ENSG00000213450	0	0	0	0	0	0	0	0	0	0	0	0	0	0	0	0	0	0	0	0	0	0	1	1	0	0	0	0	0	0	0	0
ENSG00000206891	0	0	0	0	0	0	0	0	0	0	0	0	0	0	0	0	0	0	0	0	0	0	0	0	0	0	0	0	0	0	0	0
ENSG00000244461	0	0	0	0	0	0	0	0	0	0	0	0	0	0	0	0	0	0	0	0	0	0	0	0	0	0	0	0	0	0	0	0
ENSG00000240573	0	0	0	0	0	0	0	0	0	0	0	0	0	0	0	0	0	0	0	0	0	0	0	0	0	0	0	0	0	0	0	0
ENSG00000243957	0	0	0	0	0	0	0	0	0	0	0	0	0	0	0	0	0	0	0	0	0	0	0	0	0	0	0	0	0	0	0	0
ENSG00000222574	0	0	0	0	0	0	0	0	0	0	0	0	0	0	0	0	0	0	0	0	0	0	0	0	0	0	0	0	0	0	1	0
ENSG00000240241	0	0	0	0	0	0	0	0	0	0	1	0	0	0	0	0	0	0	0	0	0	0	0	0	0	0	3	0	0	2	0	0
ENSG00000239226	0	0	0	0	0	0	0	0	0	0	0	0	0	0	0	0	0	0	0	0	0	0	0	0	0	0	0	0	0	0	0	0
ENSG00000169855	0	1	0	1	0	0	0	0	1605	1369	6529	1032	27	34	23	35	0	0	0	1	0	0	0	0	1238	1922	1844	3958	8	40	17	29
ENSG00000240752	0	0	0	0	0	0	0	0	3	1	2	0	0	0	0	1	0	0	0	0	0	0	0	0	0	1	0	5	0	0	0	0
ENSG00000240964	0	0	0	0	0	0	0	0	4	0	12	1	0	0	0	0	0	0	0	0	0	0	0	0	4	4	2	7	0	0	2	1
ENSG00000265193	0	0	0	0	0	0	0	0	0	0	0	0	0	0	0	0	0	0	0	0	0	0	0	0	0	0	0	0	0	0	0	0
ENSG00000243877	0	0	0	0	0	0	0	0	0	0	0	0	0	0	0	0	0	0	0	0	0	0	0	0	0	0	0	0	0	0	0	0
ENSG00000271389	0	0	0	0	0	0	0	0	0	0	0	0	0	0	0	0	0	0	0	0	0	0	0	0	0	0	0	0	0	0	0	0
ENSG00000239699	0	0	0	0	0	0	0	0	0	0	0	0	0	0	0	0	0	0	0	0	0	0	0	0	0	0	0	1	0	1	0	0
ENSG00000242586	0	0	0	0	0	0	0	0	0	0	0	0	0	0	0	0	0	0	0	0	0	0	0	0	0	0	0	0	0	0	0	0
ENSG00000242828	0	0	0	0	0	0	3	0	0	0	0	0	0	0	0	0	0	0	0	0	1	0	0	0	0	0	0	0	0	0	0	0
ENSG00000242781	0	0	0	0	0	0	0	0	0	0	0	0	0	0	0	0	0	0	0	0	1	0	0	0	0	0	0	0	0	0	0	0
ENSG00000243694	0	0	0	0	0	0	0	0	0	0	0	0	0	0	0	0	0	0	0	0	0	0	0	0	0	0	0	0	0	0	0	0
ENSG00000242145	0	0	0	0	0	0	0	0	0	0	0	0	0	0	0	0	0	0	0	0	0	0	0	0	0	0	0	0	0	0	0	0
ENSG00000241593	1	1	3	3	0	2	2	3	3	4	8	2	1	1	0	2	0	0	0	0	0	0	0	0	1	2	4	3	0	0	0	0
ENSG00000244281	0	0	0	0	0	0	0	0	0	2	1	0	0	0	0	0	0	0	0	0	0	0	0	0	0	1	0	0	0	0	0	0
ENSG00000114480	4172	2972	2681	2609	4667	5039	3631	5685	3331	2723	7268	1906	4399	4371	3820	5041	631	744	557	653	4537	1821	3161	1652	1407	1941	1273	4038	1256	1620	1575	1875
ENSG00000222389	11	14	3	6	11	20	15	21	8	17	34	12	11	22	9	23	4	0	1	2	34	16	16	15	5	5	3	16	1	9	6	3
ENSG00000241319	0	0	3	1	4	6	2	4	5	1	0	1	1	7	1	4	0	0	0	1	6	5	1	4	4	0	0	6	0	0	2	1
ENSG00000240404	4	1	2	0	1	2	3	4	3	5	2	0	0	3	3	3	0	0	1	1	1	0	2	1	0	1	2	7	0	2	1	0
ENSG00000242190	1	5	4	7	1	2	1	1	0	0	6	0	0	0	1	2	2	6	0	4	0	0	2	1	0	0	3	2	0	1	1	0
ENSG00000242009	0	0	0	0	1	0	0	0	0	0	0	0	1	1	0	0	0	0	0	0	0	0	0	0	0	0	0	0	0	1	0	0
ENSG00000239440	0	0	0	0	0	0	0	0	1	0	2	0	0	3	1	2	0	0	0	0	0	0	0	0	0	0	0	0	3	0	0	0
ENSG00000241367	0	0	0	0	0	0	0	0	0	1	0	0	0	0	0	0	0	0	0	0	0	0	0	0	0	0	0	0	0	0	0	0
ENSG00000241095	0	0	0	0	1	0	0	0	1	0	1	0	3	0	0	2	0	0	1	0	1	2	1	0	0	1	0	1	0	0	0	0
ENSG00000242195	0	0	0	0	0	0	0	0	0	0	0	0	0	0	0	0	0	0	0	0	0	1	0	0	0	0	0	0	1	0	0	0
ENSG00000242641	0	0	0	0	0	0	0	0	0	0	0	0	0	0	0	0	0	0	0	0	0	0	0	0	0	0	0	0	0	0	0	0
ENSG00000241776	0	0	0	0	0	0	0	0	0	0	0	0	0	0	0	0	0	0	0	0	0	0	0	0	0	0	0	0	0	0	0	0
ENSG00000242339	0	0	0	0	0	0	0	0	0	0	0	0	0	0	0	0	0	0	0	0	0	0	0	0	0	0	0	0	0	0	0	0
ENSG00000175161	75	66	45	60	0	0	0	0	0	3	8	1	0	0	2	0	36	72	35	53	0	0	0	0	1	0	1	3	0	0	0	0
ENSG00000264084	0	0	0	0	0	0	0	0	0	0	0	0	0	0	0	0	0	0	0	0	0	0	0	0	0	0	0	0	2	0	0	0
ENSG00000241648	0	1	0	0	0	0	0	0	0	0	0	0	0	0	0	0	0	0	0	0	0	0	0	0	0	0	0	0	0	0	0	0
ENSG00000239519	1	5	0	3	0	0	0	0	0	0	1	0	0	0	0	0	0	1	1	7	0	0	0	0	0	0	0	0	0	0	0	0
ENSG00000243915	1	1	0	2	0	0	0	0	0	0	0	0	0	0	0	0	0	0	0	1	0	0	0	0	0	0	0	0	0	0	0	0
ENSG00000201410	0	0	0	0	0	0	0	0	0	0	0	0	0	0	0	0	0	0	0	0	0	0	0	0	0	0	0	0	0	0	0	0
ENSG00000251773	0	0	0	0	0	0	0	0	0	0	0	0	0	0	0	0	0	0	0	0	0	0	0	0	0	0	0	0	0	0	0	0
ENSG00000239767	0	0	0	0	0	0	0	0	0	0	0	0	0	0	0	0	0	0	0	0	0	0	0	0	0	0	0	0	0	0	0	0
ENSG00000222934	0	0	0	0	0	0	0	0	0	0	0	0	0	0	0	0	0	0	0	0	0	0	0	0	0	0	0	0	0	0	0	0
ENSG00000241328	0	0	0	0	0	0	0	0	0	0	0	0	0	0	0	0	0	0	0	0	0	0	0	0	0	0	0	0	0	0	0	0
ENSG00000277746	0	0	0	0	0	0	0	0	0	0	0	0	0	0	0	0	0	0	0	0	0	0	0	0	0	0	0	0	0	0	0	1
ENSG00000206538	0	0	0	1	277	292	205	278	7	9	38	5	0	0	0	0	0	0	0	0	493	226	409	200	17	41	22	58	1	0	0	0
ENSG00000241293	1	0	0	0	0	0	1	1	0	0	1	0	0	0	0	0	0	0	0	0	0	1	0	1	1	0	0	0	0	1	0	0
ENSG00000281392	0	0	0	0	67	70	54	100	0	0	0	0	0	0	0	0	0	0	0	0	134	60	65	66	0	0	0	0	1	0	1	0
ENSG00000264119	0	0	1	1	1	0	0	2	0	0	0	0	1	1	1	0	0	0	0	0	0	0	0	0	0	1	0	0	0	0	0	0
ENSG00000083937	1148	787	865	829	853	854	705	995	770	578	2632	446	1002	1012	1029	1342	1523	2122	1587	1667	2432	1001	1550	1136	804	1135	668	1912	1349	1403	1508	1766
ENSG00000064835	0	0	0	0	3	0	0	1	0	0	0	0	7	11	7	3	0	0	0	0	0	0	0	2	0	0	2	4	9	4	13	9
ENSG00000239829	0	0	0	0	0	0	0	1	0	0	0	0	0	0	0	0	0	0	0	0	0	0	0	0	0	0	0	0	0	0	0	0
ENSG00000240902	0	0	0	0	0	0	0	0	0	0	0	0	0	0	0	0	0	0	0	0	0	0	0	0	0	0	0	0	0	0	0	0
ENSG00000240255	0	0	0	0	0	0	0	0	0	0	0	0	0	0	0	0	0	0	0	0	0	0	0	0	0	0	0	0	0	0	0	0
ENSG00000239572	0	0	0	0	0	0	0	0	0	0	0	0	0	0	0	0	0	0	0	0	0	0	0	0	0	0	0	0	0	0	0	0
ENSG00000200703	0	0	0	0	0	0	0	0	0	0	0	0	0	0	0	0	0	0	0	0	0	0	0	0	0	0	0	0	0	0	0	0
ENSG00000221059	0	0	0	0	0	0	0	0	0	0	0	0	0	0	0	0	0	0	0	0	0	0	0	0	0	0	0	0	0	0	0	0
ENSG00000179097	0	0	0	0	0	0	0	0	1	0	1	0	0	0	1	0	0	0	0	1	0	0	0	0	0	0	0	0	0	0	0	3
ENSG00000163320	4845	3424	4020	3193	3399	3604	2572	3859	5036	4563	19214	3205	3182	3422	3508	4259	2616	3350	3197	3438	6541	2599	4507	2642	5357	7323	5216	12548	2334	3231	2939	3637
ENSG00000175105	1564	1094	1341	971	1186	1172	902	1423	1160	931	4466	700	557	610	560	658	500	545	626	799	2025	806	1335	883	920	1248	1073	2662	318	454	345	540
ENSG00000241535	11	7	13	11	16	17	7	10	7	13	47	13	1	8	14	11	2	2	2	13	20	13	18	22	13	20	13	37	4	12	10	14
ENSG00000179021	854	542	674	563	764	692	633	826	600	442	2010	314	542	575	581	672	722	985	945	802	2148	823	1480	759	579	832	587	1447	681	755	831	835
ENSG00000242159	0	0	0	0	0	0	0	0	0	0	1	0	0	0	0	0	0	0	0	0	0	0	0	0	0	0	0	0	0	0	0	0
ENSG00000283434	0	0	0	1	0	1	0	0	0	0	0	0	1	0	0	0	0	0	0	0	0	0	0	0	0	0	0	1	0	0	0	2
ENSG00000207316	0	0	0	0	0	0	0	0	0	0	0	0	0	0	0	0	0	0	0	0	0	0	0	0	0	0	0	0	0	0	0	0
ENSG00000242365	0	0	0	0	0	0	0	0	0	0	0	0	0	0	0	0	0	0	0	0	0	0	0	0	0	0	0	0	0	0	0	0
ENSG00000242705	0	0	0	0	0	0	0	0	0	0	0	0	0	0	0	0	0	0	0	0	0	0	0	0	0	0	0	0	0	0	0	0
ENSG00000241861	0	0	0	0	0	0	0	0	0	0	0	0	0	0	0	0	0	0	0	0	0	0	0	0	0	0	0	0	0	0	0	0
ENSG00000044524	336	314	334	337	0	0	0	0	559	478	2313	321	0	0	0	0	117	200	132	230	0	0	0	0	312	336	612	1023	0	0	0	0
ENSG00000240951	0	0	0	0	0	0	0	0	0	0	0	0	0	0	0	0	0	0	0	0	0	0	0	0	0	0	0	0	0	0	0	0
ENSG00000240309	0	0	0	0	0	0	0	0	0	0	0	0	0	0	0	0	0	0	0	0	0	0	0	0	0	0	0	0	0	0	0	0
ENSG00000212598	0	0	0	0	0	0	0	0	0	0	0	0	0	0	0	0	0	0	0	0	0	0	0	0	0	0	0	0	0	0	0	0
ENSG00000222490	0	0	0	0	0	0	0	0	0	0	0	0	0	0	0	0	0	0	0	0	0	0	0	0	0	0	0	0	0	0	0	0
ENSG00000189002	0	0	1	0	0	0	0	0	0	0	0	0	0	0	0	0	0	0	0	0	0	0	0	0	0	0	0	0	0	0	0	0
ENSG00000271024	0	0	0	0	0	0	0	0	0	0	0	0	0	0	0	0	0	0	0	0	0	0	0	0	0	0	0	0	0	1	0	0
ENSG00000283367	0	0	0	0	0	0	0	0	0	0	0	0	0	0	0	0	0	0	0	0	0	0	0	0	0	0	0	0	0	0	0	0
ENSG00000283544	1	0	0	0	0	0	0	0	11	3	32	3	0	0	0	0	0	0	0	0	0	0	0	0	6	8	7	6	0	0	0	0
ENSG00000251727	0	0	0	0	0	0	0	0	0	0	0	0	0	0	0	0	0	0	0	0	0	0	0	0	0	0	0	0	0	0	0	0
ENSG00000184500	175	168	143	161	919	1157	697	1150	24	24	81	26	6	23	7	45	118	192	144	207	1403	611	976	530	27	36	10	85	4	13	14	18
ENSG00000201339	0	0	0	0	0	0	1	3	1	0	0	0	0	0	0	0	0	0	0	1	1	0	0	5	0	0	0	0	0	0	0	0
ENSG00000200366	0	0	0	0	0	0	0	0	0	0	0	0	0	0	0	0	0	0	0	0	0	0	0	0	0	0	0	0	0	0	0	0
ENSG00000169379	712	391	553	426	403	495	422	534	374	272	1224	250	326	364	429	486	367	457	548	548	1022	458	800	466	476	673	284	1045	372	540	497	611
ENSG00000239614	4	2	6	2	3	1	1	1	0	0	11	4	0	2	0	1	0	4	2	5	3	2	0	5	7	8	4	10	2	3	3	3
ENSG00000178750	16	9	19	14	6	5	2	7	6	5	25	3	6	4	3	4	3	2	2	6	11	6	4	4	5	12	3	16	1	3	1	5
ENSG00000178700	679	519	578	515	271	315	192	319	492	375	1536	285	271	322	276	422	429	525	470	561	425	187	283	144	381	542	384	871	205	296	224	348
ENSG00000178694	469	362	340	273	376	348	289	398	341	289	1271	215	542	476	537	651	275	354	331	392	770	285	532	277	347	493	329	869	402	459	464	573
ENSG00000242457	7	7	9	2	10	11	11	11	14	13	47	3	5	9	8	9	5	0	2	6	24	9	7	10	8	14	13	33	0	11	8	9
ENSG00000178660	2	2	5	2	8	7	5	6	9	4	5	3	0	1	5	9	5	4	4	6	6	4	10	4	3	5	5	14	8	3	7	9
ENSG00000242911	0	0	0	0	0	0	0	0	0	0	0	0	0	0	0	0	0	0	0	0	0	0	0	0	0	0	0	0	0	0	0	0
ENSG00000234629	0	0	0	0	0	0	0	0	0	0	0	0	0	1	0	0	0	0	0	0	0	0	0	0	0	0	0	0	0	0	0	0
ENSG00000239589	0	0	0	0	0	0	0	0	0	0	0	0	0	0	0	0	0	0	0	0	0	0	0	0	0	0	0	0	0	0	0	0
ENSG00000244048	0	0	0	0	0	0	0	0	0	0	0	0	0	0	0	0	0	0	0	0	0	0	0	0	0	0	0	0	0	0	0	0
ENSG00000244681	0	0	0	0	0	0	0	1	0	0	0	0	3	1	0	0	0	0	0	0	0	0	0	0	0	0	1	0	0	0	0	1
ENSG00000271671	0	0	0	0	0	0	0	0	0	0	0	0	0	0	0	0	0	0	0	0	0	0	0	0	0	0	0	0	0	0	0	0
ENSG00000243547	9	3	7	7	4	9	3	10	7	6	18	2	3	7	3	6	5	5	14	8	7	1	11	2	3	8	5	11	4	8	5	7
ENSG00000278103	0	0	0	0	0	0	0	0	0	0	0	0	0	0	0	0	0	0	0	0	0	0	0	0	0	0	0	0	0	0	0	0
ENSG00000241815	0	0	0	0	0	0	0	0	0	0	0	0	0	0	0	0	0	0	0	0	0	0	0	0	0	0	0	0	0	0	0	0
ENSG00000199286	0	0	0	0	0	0	0	0	0	0	0	0	0	0	0	0	0	0	0	0	0	0	0	0	0	0	0	0	0	0	0	0
ENSG00000269028	3	1	4	1	2	4	1	4	3	0	6	0	1	0	1	2	1	0	1	3	6	2	5	4	3	1	0	4	0	0	2	3
ENSG00000218754	0	0	0	0	0	0	0	0	0	0	0	0	0	0	0	0	0	0	0	0	0	0	0	0	0	0	0	0	0	0	0	0
ENSG00000241018	0	0	0	0	0	0	0	0	0	0	0	0	0	0	0	0	0	0	0	0	0	0	0	0	0	0	0	0	0	0	0	0
ENSG00000176243	0	0	0	0	0	0	0	0	0	0	0	0	0	0	0	0	0	0	0	0	0	0	0	0	0	0	0	0	0	0	0	0
ENSG00000080224	0	0	0	0	0	0	0	0	45	39	67	28	0	0	1	0	0	0	0	0	0	0	0	0	44	47	8	70	0	0	0	0
ENSG00000251172	0	0	0	0	0	0	0	0	0	0	0	0	0	0	0	0	0	0	0	0	0	0	0	0	0	0	0	0	0	0	0	0
ENSG00000261292	0	1	0	1	0	0	0	0	0	2	5	4	0	0	0	1	0	0	0	0	0	0	0	0	0	2	1	0	0	0	0	0
ENSG00000113966	397	268	297	281	157	181	134	215	157	153	523	96	143	174	186	252	406	542	506	518	351	120	246	154	167	212	161	365	210	298	306	322
ENSG00000282527	0	0	0	0	0	0	0	1	0	0	0	0	0	0	0	0	0	0	0	0	0	0	0	2	0	0	0	0	0	0	0	0
ENSG00000080200	209	366	222	338	559	709	492	768	1425	1218	5376	970	352	475	415	600	41	122	61	291	786	378	579	348	1267	1949	1493	4136	213	504	317	560
ENSG00000279658	0	1	0	0	0	0	0	0	0	0	3	0	0	3	1	0	0	0	0	2	1	0	0	1	1	1	0	2	0	0	1	0
ENSG00000170854	1172	851	945	868	575	740	501	721	801	584	2728	484	1304	1146	1274	1371	968	1722	1373	1359	1348	579	1106	575	853	1328	561	1813	1261	1482	1346	1590
ENSG00000183185	0	0	0	0	0	0	0	0	0	0	1	0	0	1	0	0	1	0	0	0	0	0	1	0	0	0	1	0	0	0	1	3
ENSG00000250750	0	0	0	0	0	0	0	0	0	0	0	0	0	0	0	0	0	0	0	0	0	0	0	0	0	0	0	0	0	0	0	0
ENSG00000213439	0	0	0	0	0	0	0	0	0	0	0	0	0	0	0	0	0	0	0	0	0	0	0	0	0	0	0	0	0	0	0	0
ENSG00000196578	0	0	0	0	0	0	0	0	0	0	0	0	0	0	0	0	0	0	0	0	0	0	0	0	0	0	0	0	0	0	0	0
ENSG00000249225	0	0	0	0	0	0	0	0	0	0	0	0	0	0	0	0	0	0	0	0	0	0	0	0	0	0	0	0	0	0	0	0
ENSG00000251090	0	0	0	0	0	0	0	0	0	0	0	0	0	0	0	0	0	0	0	0	0	0	0	0	0	0	0	0	0	0	0	0
ENSG00000248365	0	0	0	0	0	0	0	0	0	0	0	0	0	0	0	0	0	0	0	0	0	0	0	0	0	0	0	0	0	0	0	0
ENSG00000231192	0	0	0	0	0	0	0	0	0	0	0	0	0	0	0	0	0	0	0	0	0	0	0	0	0	0	0	0	0	0	0	0
ENSG00000236032	0	0	0	0	0	0	0	0	0	0	0	0	0	0	0	0	0	0	0	0	0	0	0	0	0	0	0	0	0	0	0	0
ENSG00000233412	0	0	0	0	0	0	0	0	0	0	0	0	0	0	0	0	0	0	0	0	0	0	0	0	0	0	0	0	0	0	0	0
ENSG00000249321	0	0	0	0	0	0	0	0	0	0	0	0	0	0	0	0	0	0	0	0	0	0	0	0	0	0	0	0	0	0	0	0
ENSG00000249975	0	0	0	0	0	0	0	0	0	0	0	0	0	0	0	0	0	0	0	0	0	0	0	0	0	0	0	0	0	0	0	0
ENSG00000248507	0	0	0	0	0	0	0	0	0	0	0	0	0	0	0	0	0	0	0	0	0	0	0	0	0	0	0	0	0	0	0	0
ENSG00000251088	0	0	0	0	0	0	0	0	0	0	2	0	0	0	0	0	0	0	0	0	0	0	0	0	0	0	0	0	0	0	0	0
ENSG00000187900	0	0	0	0	0	0	0	0	0	0	0	0	0	0	0	0	0	0	0	0	0	0	0	0	0	0	0	0	0	0	0	2
ENSG00000230301	0	0	0	0	0	0	0	0	0	0	0	0	0	0	0	0	0	0	0	0	0	0	0	0	0	2	0	0	0	0	0	0
ENSG00000197938	0	0	0	0	0	0	0	0	0	0	0	0	0	0	0	0	0	0	0	0	0	0	0	0	0	0	0	0	0	1	0	0
ENSG00000232535	0	0	0	0	0	0	0	0	0	0	0	0	0	0	0	0	0	0	0	0	0	0	0	0	0	0	0	2	0	0	0	0
ENSG00000196098	0	0	0	0	0	0	0	0	0	0	0	0	1	0	0	0	0	0	0	0	0	0	0	0	0	0	0	0	0	0	0	0
ENSG00000206536	0	0	0	0	0	0	0	0	0	0	0	0	0	0	0	0	0	0	0	0	0	0	0	0	0	0	0	0	0	0	0	0
ENSG00000244653	0	0	0	0	0	0	0	0	0	0	0	0	0	0	0	0	0	0	0	0	0	1	0	0	2	0	0	0	0	0	0	0
ENSG00000232382	0	0	0	0	0	0	0	0	3	0	1	0	0	0	0	0	0	0	0	0	0	0	0	0	1	0	0	1	0	0	1	0
ENSG00000231861	2	0	1	2	4	2	2	1	16	17	75	7	2	5	5	2	0	0	0	1	7	5	1	3	4	21	20	36	1	2	2	4
ENSG00000080822	2051	1398	1534	1452	1334	1456	1060	1645	4298	3693	15412	2656	2223	2078	2513	2822	1870	2491	1886	2073	2864	1172	1887	1451	4701	6495	3982	10711	2701	2942	3585	3509
ENSG00000080819	1429	1200	1151	1235	1371	1494	1095	1440	2014	1703	8778	1562	1584	1431	1725	1779	451	861	670	1104	2209	942	1573	818	2124	3582	2340	6531	1470	1855	1570	1836
ENSG00000248839	3	0	0	0	0	0	0	0	16	8	46	10	2	2	5	3	1	0	0	0	0	0	0	0	6	8	10	29	4	6	2	9
ENSG00000250562	0	0	0	0	0	0	0	0	49	51	208	38	6	17	23	24	0	0	0	0	1	0	0	0	33	47	40	102	13	21	21	15
ENSG00000154165	0	0	0	0	0	0	0	0	3155	2693	15350	2734	1105	885	979	1212	0	0	1	1	0	0	0	0	3462	5385	3814	10537	1422	1179	1515	1482
ENSG00000240827	0	0	0	0	0	0	0	0	0	0	1	1	0	0	0	0	0	0	0	0	0	0	0	0	0	0	1	3	0	0	0	0
ENSG00000244153	0	0	0	0	0	0	0	0	0	0	2	0	0	0	0	0	0	0	0	3	0	0	0	0	0	1	0	1	0	0	1	0
ENSG00000239445	20	23	18	31	1	0	0	1	8	5	12	0	0	0	1	0	12	21	14	21	0	0	0	0	0	7	3	3	0	0	0	1
ENSG00000064225	219	132	179	177	28	29	19	34	66	64	189	35	2	5	0	4	60	102	45	133	33	8	19	22	67	100	46	192	0	0	0	4
ENSG00000249274	11	6	11	13	2	1	1	3	0	1	9	3	0	0	0	0	0	0	0	8	0	0	0	0	2	2	3	1	0	0	0	0
ENSG00000057019	857	716	712	744	584	739	447	755	376	370	1453	277	86	152	126	232	323	509	350	783	1190	463	760	447	305	479	339	998	72	143	86	194
ENSG00000206712	12	9	5	10	2	6	6	3	1	3	10	1	1	1	0	0	1	2	2	11	10	0	1	1	6	2	1	8	0	2	0	3
ENSG00000239462	1	1	4	2	0	2	0	1	0	3	4	0	0	0	0	0	1	2	2	2	1	4	1	0	0	3	0	0	1	0	0	0
ENSG00000207331	1	0	1	0	0	0	0	0	0	2	0	0	0	0	0	0	0	0	0	2	1	0	1	0	0	1	0	1	0	0	0	0
ENSG00000240476	0	0	0	0	0	1	1	0	0	0	0	0	1	1	0	1	0	0	0	1	2	0	0	0	0	0	0	0	0	0	0	0
ENSG00000275108	0	0	0	0	0	0	0	0	0	0	0	0	0	0	0	0	0	0	0	0	0	0	0	0	0	0	0	0	0	0	0	0
ENSG00000241305	0	0	0	0	0	0	0	0	0	0	0	0	0	0	0	0	0	0	0	0	0	0	0	0	0	0	0	0	0	0	0	0
ENSG00000240572	0	0	0	0	0	0	0	0	0	0	0	0	0	0	0	0	0	0	0	0	0	0	0	0	0	0	0	0	0	0	0	0
ENSG00000243296	0	0	0	0	0	2	0	0	0	0	0	0	0	0	0	0	0	0	0	0	0	0	0	0	0	0	0	0	0	0	0	0
ENSG00000243089	0	0	0	0	0	0	0	4	0	0	0	0	0	0	0	0	0	0	0	0	0	3	0	0	0	0	0	0	0	0	0	0
ENSG00000244464	0	0	0	0	0	0	0	0	0	0	4	0	0	0	0	0	0	0	0	0	2	0	0	0	0	0	0	0	0	0	0	0
ENSG00000144810	0	2	0	0	1	1	3	0	3	0	11	0	9	4	11	6	0	0	0	1	1	1	1	0	3	1	2	5	4	6	3	9
ENSG00000273374	29	31	30	24	11	17	9	15	51	35	99	28	50	51	63	57	9	4	7	16	14	8	12	4	20	37	13	52	10	35	27	42
ENSG00000184220	1471	1061	1176	1071	951	1031	772	1034	949	626	2142	493	1817	1634	1912	2238	2435	3231	2397	2441	2454	1043	1834	1188	880	1255	331	1575	2274	2110	2955	2694
ENSG00000168386	57	38	65	28	53	53	42	66	32	38	131	37	162	186	206	339	18	15	26	91	115	68	96	84	77	75	27	192	79	201	224	257
ENSG00000264897	0	0	0	0	0	0	0	0	0	0	2	0	1	0	0	0	0	0	0	1	0	0	0	0	0	0	0	0	0	0	0	0
ENSG00000235156	0	1	0	0	0	2	0	0	0	0	1	0	0	0	0	0	2	4	0	1	0	0	0	0	0	1	1	1	0	0	0	0
ENSG00000277000	0	0	0	0	0	0	0	0	0	0	0	0	0	0	0	0	0	1	0	0	0	0	0	0	0	0	0	1	0	0	0	0
ENSG00000243974	0	0	0	1	0	0	0	0	0	0	0	0	0	0	0	0	1	1	0	0	0	0	0	0	0	0	0	0	0	2	0	0
ENSG00000036054	922	631	669	598	907	992	769	1132	656	582	2928	404	595	580	588	783	652	949	801	866	2148	932	1531	1006	648	930	753	1779	631	697	776	850
ENSG00000114021	1548	1143	1222	1162	852	982	686	972	1225	966	4114	749	1063	983	1029	1261	1640	2672	1849	2147	2083	864	1457	912	984	1462	922	2416	911	1052	1167	1266
ENSG00000154174	2329	1512	1871	1500	3482	3706	2754	3770	3094	2468	10543	1867	3769	3293	3701	4161	1300	2230	1915	1987	6592	2564	5433	2516	3209	4629	2204	6999	3276	3348	3484	3957
ENSG00000206535	76	49	62	47	201	188	127	207	96	97	342	49	171	147	144	170	28	41	42	61	430	158	328	160	108	117	46	233	106	130	81	152
ENSG00000181458	0	0	0	0	18	13	8	26	31	26	22	5	195	220	225	313	0	0	0	0	4	0	0	1	2	2	3	1	67	86	83	138
ENSG00000144820	0	0	0	0	0	0	0	0	0	0	0	0	1	3	7	0	0	0	0	0	1	0	2	0	0	0	0	0	3	7	1	2
ENSG00000252989	0	0	0	0	0	0	0	0	0	0	0	0	0	0	0	0	0	0	0	0	0	0	0	0	0	0	0	0	0	0	0	0
ENSG00000243495	0	0	0	0	0	0	0	0	0	0	0	0	0	0	0	0	0	0	0	0	0	0	0	0	0	0	0	0	0	0	0	0
ENSG00000114354	1559	1162	1267	1125	1631	1706	1234	1906	1621	1244	5728	995	1494	1390	1647	1847	780	856	805	1221	3359	1335	2229	1224	1475	2236	1310	3683	1157	1482	1382	1833
ENSG00000154175	8	17	10	15	21	28	28	40	12	15	34	9	47	49	45	51	4	1	3	14	41	18	27	18	11	28	8	34	24	36	29	36
ENSG00000213431	0	0	0	0	0	0	0	0	0	0	0	0	0	0	0	0	0	0	0	0	0	0	0	0	0	0	0	0	0	0	0	0
ENSG00000201642	0	0	0	0	0	0	0	0	0	0	0	0	0	0	0	0	0	0	0	0	0	0	0	0	0	0	0	0	0	0	0	0
ENSG00000242573	0	0	0	0	0	0	0	0	0	0	0	0	0	0	0	0	0	0	0	0	0	0	0	0	0	0	0	0	0	0	0	0
ENSG00000081148	5	3	13	8	8	7	9	8	6	10	77	11	15	17	19	23	1	3	3	10	10	1	4	11	5	11	39	41	11	32	8	34
ENSG00000138468	1555	1046	1227	891	1627	1711	1291	1772	1003	951	4449	625	682	633	676	802	652	641	751	872	3440	1418	2253	1571	902	1216	1339	2925	368	559	540	631
ENSG00000241738	12	8	10	9	8	6	7	5	2	2	16	2	11	5	5	4	3	5	1	4	18	11	15	13	6	9	2	23	0	4	7	7
ENSG00000232830	0	0	4	0	0	3	0	3	2	0	13	0	1	2	3	3	0	0	0	0	1	3	2	1	0	3	4	14	0	0	0	5
ENSG00000282978	9	4	2	3	2	1	2	3	9	5	13	5	4	3	3	3	1	6	0	5	4	6	8	6	6	11	5	12	1	8	4	7
ENSG00000201554	0	0	0	0	1	1	0	0	0	0	3	0	0	0	1	1	0	0	0	1	0	0	0	1	4	2	0	5	0	0	0	0
ENSG00000175841	3	2	3	0	0	1	1	1	0	2	1	1	109	76	162	150	0	0	3	0	3	1	1	0	0	0	1	1	65	93	132	93
ENSG00000174173	1703	1017	1203	1037	592	521	476	623	713	506	2064	348	873	781	938	1000	2447	3039	2530	2386	1592	747	1071	798	694	1016	437	1410	1226	1070	1275	1250
ENSG00000081154	4912	3261	3831	3384	4307	4211	3243	4806	3975	3246	15048	2568	4057	3733	4421	4628	3515	4731	4175	4025	9250	3774	6495	3727	3933	5464	3491	9085	4004	4279	4520	4791
ENSG00000242299	56	73	44	50	36	36	30	42	68	59	258	57	45	52	59	65	50	39	37	49	63	40	47	43	54	78	44	130	50	59	52	66
ENSG00000243396	4	2	3	4	0	2	1	1	0	0	8	0	1	1	0	3	0	0	2	0	1	1	0	0	0	0	9	8	1	1	3	3
ENSG00000252348	2	0	1	0	0	0	0	0	0	0	2	0	0	1	0	0	0	0	3	1	0	0	1	1	1	0	0	1	0	2	0	0
ENSG00000212319	0	0	1	0	0	0	0	0	0	0	0	0	0	0	0	1	0	0	0	0	0	1	0	0	0	0	0	0	0	0	0	1
ENSG00000201511	1	0	0	0	0	1	0	0	1	3	3	0	1	0	1	0	0	0	0	1	0	0	0	0	0	3	1	1	0	0	2	1
ENSG00000066422	2291	1584	1771	1417	1051	1112	777	1241	1056	862	4611	675	1274	1149	1357	1460	1596	1958	1915	1819	2589	922	1735	1042	1192	1746	1190	3199	1073	1313	1328	1475
ENSG00000201121	5	0	7	2	0	0	6	3	2	1	7	2	2	2	0	0	3	1	0	1	1	0	5	1	3	0	1	5	1	3	1	0
ENSG00000256628	69	74	107	66	39	45	51	52	67	56	224	41	47	46	65	57	33	59	50	93	80	55	59	52	66	87	71	149	48	68	60	72
ENSG00000114391	12478	8031	8916	8125	8011	8167	5688	8732	13987	9454	42872	8186	12700	11845	13593	15612	16411	18400	17043	15054	21861	8715	16042	11227	11780	16126	9839	25122	16997	14466	20289	19325
ENSG00000244119	34	36	56	32	2	8	5	5	29	46	127	17	27	35	27	39	5	13	28	29	5	4	4	1	15	22	28	49	7	22	15	44
ENSG00000182504	2138	1390	1658	1314	1360	1496	1044	1456	832	652	3158	595	802	743	776	854	736	882	985	1192	2391	950	1572	976	974	1315	546	2443	522	710	670	769
ENSG00000144815	2060	1273	1746	1249	1632	1736	1131	1827	689	559	3631	440	835	785	894	1015	958	1206	1106	1188	2561	917	1821	863	891	1204	1167	2362	738	874	733	886
ENSG00000249474	0	0	0	0	0	0	0	0	0	0	0	0	0	0	0	0	0	0	0	0	0	0	0	0	0	0	0	0	0	0	0	0
ENSG00000144802	9	14	17	8	222	357	171	356	240	238	879	143	583	659	640	861	3	1	4	2	187	120	80	122	176	274	250	480	216	586	361	784
ENSG00000214407	0	0	0	0	9	23	11	26	0	0	1	0	0	0	3	1	0	0	0	0	4	4	6	3	0	0	0	0	0	0	1	0
ENSG00000202177	0	0	0	0	0	0	0	0	0	0	0	0	0	0	0	0	0	0	0	0	0	0	0	0	0	0	0	0	0	0	0	0
ENSG00000241280	0	0	0	0	2	13	3	9	0	0	0	0	0	1	2	1	0	0	0	0	4	2	2	1	0	0	0	0	0	0	2	0
ENSG00000170044	0	0	0	0	10	11	1	8	1	0	5	0	3	2	5	4	0	0	0	2	5	7	1	0	0	1	3	1	3	2	5	1
ENSG00000241754	0	0	0	0	0	0	0	0	0	0	0	0	0	0	0	0	0	0	0	0	0	0	0	0	0	0	0	0	0	0	0	0
ENSG00000201065	0	0	0	0	0	0	0	0	0	0	0	0	0	0	0	0	0	0	0	0	0	0	0	0	0	0	0	1	0	0	0	0
ENSG00000201922	0	0	0	0	0	0	0	0	0	0	0	0	0	0	0	0	0	0	0	0	0	0	0	0	0	0	0	0	0	0	0	0
ENSG00000236691	0	0	0	0	0	0	0	0	0	0	0	0	0	0	0	0	0	0	0	0	0	0	0	0	0	0	0	0	0	0	0	0
ENSG00000265328	0	0	0	0	0	0	0	0	0	0	0	0	0	0	0	0	0	0	0	0	0	0	0	0	0	0	0	0	0	0	0	0
ENSG00000240107	0	0	0	0	1	0	0	0	0	0	1	0	0	0	0	0	0	0	0	0	0	0	0	0	0	0	0	0	0	0	0	0
ENSG00000243195	0	0	0	0	0	0	0	0	0	0	0	0	0	0	0	0	0	0	0	0	0	0	0	0	0	0	0	0	0	0	0	0
ENSG00000214405	0	0	0	0	0	0	0	0	0	0	0	0	0	0	0	0	0	0	0	0	0	0	0	0	0	0	0	0	0	0	0	0
ENSG00000243635	0	0	0	0	0	4	2	2	0	0	0	0	0	1	0	0	0	1	0	0	2	1	0	0	0	0	0	0	0	0	0	0
ENSG00000170017	2382	1979	1813	1784	512	557	385	507	68	76	273	59	3029	2953	2901	3801	3417624	1284	905	1493	500	213	429	226	77	133	31	184	1864	2715	2310	3256
ENSG00000114423	841	664	781	562	599	676	471	870	1653	1273	6916	769	518	720	561	868	142	155	225	308	943	516	701	469	1334	1781	2017	3293	315	823	473	933
ENSG00000244652	0	0	0	0	0	0	0	0	0	0	0	0	0	0	0	0	0	0	0	0	0	0	0	0	0	0	0	0	0	0	0	0
ENSG00000200610	0	0	0	0	0	0	0	0	0	0	0	0	0	0	0	0	0	0	0	0	0	0	0	1	0	0	0	0	0	0	0	0
ENSG00000200361	2	1	2	0	1	0	1	1	0	0	0	0	0	0	0	0	0	0	0	0	1	0	0	0	0	0	0	0	0	0	0	0
ENSG00000242759	16	19	26	24	7	13	14	16	0	2	12	1	4	4	4	5	15	14	26	41	18	10	23	10	2	0	2	2	0	3	3	4
ENSG00000239997	0	1	3	0	0	2	0	1	0	0	0	0	0	1	0	0	0	0	0	1	1	1	0	0	0	0	0	0	0	0	0	0
ENSG00000241787	0	0	1	0	0	3	0	1	0	0	1	0	0	0	0	0	1	0	0	0	1	1	0	0	0	0	0	0	0	0	0	0
ENSG00000239632	0	0	0	0	0	0	0	0	0	0	0	0	0	0	0	0	0	0	0	1	0	0	0	0	0	0	0	2	0	0	0	0
ENSG00000242214	0	0	0	0	0	0	0	0	0	0	0	0	0	0	0	1	0	0	0	0	0	0	0	0	0	0	0	0	0	0	0	0
ENSG00000242836	1	1	1	1	0	0	0	1	0	0	0	0	0	0	0	0	0	0	0	0	0	0	0	0	0	0	0	0	0	0	0	0
ENSG00000249580	1	0	0	0	0	0	0	1	0	0	0	0	0	0	0	0	0	0	0	0	1	0	0	0	0	0	0	0	0	0	0	0
ENSG00000243658	1	2	1	1	2	1	0	1	0	0	0	0	0	0	0	1	2	0	1	0	0	0	1	2	0	0	0	0	0	1	0	0
ENSG00000240034	1	0	0	1	0	0	0	0	0	0	0	0	0	0	0	0	0	0	0	0	1	0	0	0	0	0	0	0	0	0	0	0
ENSG00000250630	0	0	0	0	0	0	1	0	0	0	0	0	0	0	0	2	0	0	0	0	0	0	1	0	0	0	0	0	0	0	0	0
ENSG00000239586	0	0	0	2	0	1	0	2	0	0	0	0	0	0	0	0	0	0	2	0	0	2	0	0	0	0	0	0	0	0	0	0
ENSG00000240796	2	0	0	0	0	0	0	0	0	0	0	0	0	0	0	0	2	0	0	0	0	0	0	0	0	0	0	0	0	0	0	0
ENSG00000252626	0	0	0	0	0	0	0	0	0	0	0	0	0	0	0	0	0	0	0	0	0	0	0	0	0	0	0	0	0	0	0	0
ENSG00000239455	0	0	1	0	0	1	1	0	0	0	0	0	0	0	0	1	0	0	0	1	0	1	1	1	0	0	0	0	0	0	0	0
ENSG00000243701	235	161	199	151	113	137	96	175	79	44	414	53	113	129	101	159	128	150	133	235	256	109	216	111	73	122	60	237	132	139	91	189
ENSG00000239828	18	11	2	15	1	0	1	1	3	0	12	1	1	2	0	1	1	2	3	6	3	0	0	0	0	1	2	3	0	1	1	3
ENSG00000241218	6	11	10	10	1	0	0	0	1	3	7	2	3	0	0	0	2	1	0	5	1	2	2	1	0	0	1	0	0	0	0	0
ENSG00000272597	1	3	1	3	2	0	0	0	0	0	2	0	1	0	0	0	1	0	0	2	0	0	0	0	0	1	0	1	0	0	1	0
ENSG00000138483	0	0	0	1	0	1	0	0	0	1	0	0	1	0	0	0	0	0	0	1	2	0	0	0	0	0	0	5	0	0	0	2
ENSG00000273125	0	0	0	0	0	1	0	1	1	0	6	1	7	3	3	5	0	0	0	0	4	1	2	3	0	0	0	2	1	0	2	1
ENSG00000114439	2887	2651	2576	2597	3397	3789	2740	3919	1989	1651	8953	1340	2508	2622	2601	3053	1282	1786	1446	2109	6180	2434	4875	2264	2100	3168	2545	6647	1831	2515	2143	2696
ENSG00000241469	0	0	0	2	0	0	0	0	0	0	0	0	2	1	0	4	0	5	0	1	1	2	2	0	0	0	0	0	0	1	0	0
ENSG00000240423	0	0	0	0	0	0	0	0	0	0	0	0	0	0	0	0	0	0	0	0	0	0	0	0	0	0	0	0	0	0	0	0
ENSG00000279277	22	29	24	23	19	18	19	37	10	10	52	11	2	5	6	8	11	6	5	39	55	15	24	20	16	16	4	29	0	10	3	10
ENSG00000196776	6509	5326	4723	4843	6616	6702	5474	7248	2766	2493	9013	1710	2344	2465	2280	3085	2228	4168	2804	4867	10706	4504	7606	4672	2344	3349	1825	5894	1427	2326	1614	2446
ENSG00000271856	410	302	421	285	371	412	336	423	13	10	19	3	579	596	722	831	310	532	440	633	842	366	586	386	12	11	4	2	559	752	731	948
ENSG00000114446	237	178	229	190	1	3	2	2	124	75	385	47	652	477	702	672	293	429	345	342	4	4	3	2	106	165	78	230	632	576	848	733
ENSG00000114455	23	24	32	23	2	4	7	13	0	0	1	0	12	15	16	18	14	19	7	17	6	0	2	3	0	2	0	2	1	9	4	12
ENSG00000243188	2	5	2	1	1	1	0	3	0	0	0	0	6	1	0	3	0	0	0	2	0	0	0	1	0	0	0	0	0	0	0	0
ENSG00000144821	50	60	62	67	156	168	116	194	34	35	71	14	11	21	12	21	28	38	30	84	211	101	139	88	23	42	18	44	5	12	9	36
ENSG00000241634	161	135	135	138	158	144	104	164	94	93	307	76	48	79	66	77	42	59	46	116	227	99	128	125	84	145	25	251	40	51	55	79
ENSG00000163507	2326	1556	2038	1651	2438	2388	1897	2566	1412	1162	4018	883	1162	1184	1472	1520	2301	2730	2704	2671	6270	2612	4518	3198	1600	2366	531	3627	1427	1808	2031	2235

ENSG00000202379	4	7	11	4	5	13	6	3	4	0	16	3	4	1	3	6	3	2	0	9	11	12	12	9	4	8	1	14	2	4	11	4
ENSG00000276763	7	5	8	4	18	15	12	13	12	14	22	7	7	6	9	16	2	2	3	13	39	20	23	21	10	19	6	24	6	17	5	8
ENSG00000280684	10	2	3	3	19	18	24	22	12	4	24	6	0	3	3	2	5	2	0	5	56	26	41	28	10	6	3	14	0	0	7	6
ENSG00000198919	1221	963	1086	911	444	534	365	559	729	563	2331	400	702	663	702	922	690	823	773	911	1028	436	765	405	552	818	489	1398	484	710	614	886
ENSG00000241777	0	0	0	0	0	0	0	0	0	0	0	0	0	0	0	0	0	0	0	0	0	0	0	0	0	0	0	0	0	0	0	0
ENSG00000163515	0	0	0	0	0	0	0	0	0	0	0	0	0	0	1	1	0	0	0	0	0	0	0	0	0	0	0	0	0	1	1	1
ENSG00000163519	50	15	38	11	117	100	107	92	13	2	14	0	58	49	73	81	95	74	100	74	450	149	411	201	4	0	0	1	120	111	169	160
ENSG00000138472	0	0	0	0	0	0	0	0	0	0	0	0	0	0	0	0	0	0	0	0	0	0	0	0	0	0	0	0	0	0	0	0
ENSG00000114487	1	0	0	0	0	0	1	0	0	0	1	0	0	3	0	0	0	0	0	0	1	1	1	0	0	0	0	1	0	0	0	1
ENSG00000221633	0	0	0	0	0	0	0	0	0	0	0	0	0	0	0	0	0	0	0	0	0	0	0	0	0	0	0	0	0	0	0	0
ENSG00000239314	0	0	0	0	0	0	0	0	0	0	0	0	0	0	0	0	0	0	0	0	0	0	0	0	0	0	0	0	0	0	0	0
ENSG00000241224	0	0	0	0	0	0	0	0	0	0	0	0	0	0	0	0	0	0	0	0	0	0	0	0	0	0	0	0	0	0	0	0
ENSG00000261364	0	0	0	0	0	0	0	0	0	0	0	0	0	0	0	0	0	0	0	0	0	0	0	0	0	0	0	0	0	0	0	0
ENSG00000214381	0	0	0	0	0	0	0	0	0	0	0	0	0	0	0	0	0	0	0	0	0	0	0	0	0	0	3	0	0	0	0	0
ENSG00000252889	0	0	0	0	0	0	0	0	0	0	0	0	0	0	0	0	0	0	0	0	0	0	0	0	0	0	0	0	0	0	0	1
ENSG00000239288	0	0	0	0	0	0	0	0	0	0	1	0	0	0	0	0	0	0	0	0	0	0	0	0	0	0	0	0	0	0	0	0
ENSG00000163530	0	0	0	0	0	0	0	0	0	0	0	0	0	0	0	0	0	0	0	0	0	0	0	0	0	0	0	2	0	0	0	0
ENSG00000277635	0	0	0	0	0	0	0	0	0	0	0	0	0	0	0	0	0	0	0	0	0	0	0	0	0	0	0	0	0	0	0	0
ENSG00000121570	0	1	0	0	0	0	0	0	0	0	0	0	0	0	0	0	0	0	0	0	0	0	1	0	0	0	1	1	0	0	0	0
ENSG00000241257	0	0	0	0	0	0	0	0	0	0	0	0	0	0	0	0	0	0	0	0	0	0	0	0	0	0	0	0	0	0	0	0
ENSG00000244561	0	0	0	0	0	0	0	0	0	0	0	0	0	0	0	0	0	0	0	0	0	0	0	0	0	0	0	0	0	0	0	0
ENSG00000230618	0	0	0	0	0	0	0	0	0	0	0	0	0	0	0	0	0	0	0	0	0	0	0	0	0	0	0	0	0	0	0	0
ENSG00000228980	0	0	0	0	0	0	0	0	0	0	1	0	0	0	0	0	0	0	0	0	0	0	0	0	0	0	1	0	0	1	0	0
ENSG00000244196	0	0	0	0	0	0	0	0	0	0	0	0	0	0	0	0	0	0	0	0	0	0	0	0	0	0	0	0	0	0	0	0
ENSG00000265956	0	0	0	0	0	0	0	0	0	0	0	0	0	0	0	0	0	0	0	0	0	0	0	0	0	0	0	0	0	0	0	0
ENSG00000242029	0	0	0	0	0	0	0	0	0	0	1	0	0	0	0	1	0	0	0	0	0	0	0	0	0	0	0	0	0	2	0	0
ENSG00000271249	0	0	0	0	0	0	0	0	0	0	0	0	1	0	0	0	0	0	0	0	0	0	0	0	0	0	0	0	0	0	0	0
ENSG00000214380	0	0	0	0	0	0	0	0	0	0	0	0	0	0	0	0	0	0	0	0	0	0	0	0	0	0	0	0	0	0	0	0
ENSG00000243945	0	0	0	0	0	0	0	0	0	0	0	0	0	0	0	0	0	0	0	0	0	0	0	0	0	0	0	0	0	0	0	0
ENSG00000240895	0	0	0	0	0	0	0	0	0	0	0	0	0	0	0	0	0	0	0	0	0	0	0	0	0	0	0	0	0	0	0	0
ENSG00000221206	0	0	0	0	0	0	0	0	0	0	0	0	0	0	0	0	0	0	0	0	0	0	0	0	0	0	0	0	0	0	0	0
ENSG00000244722	0	1	0	0	0	0	0	0	0	0	0	0	0	0	0	0	1	0	3	0	0	0	0	0	0	0	0	0	0	0	0	0
ENSG00000201426	0	0	0	0	0	0	0	0	0	0	0	0	0	0	0	0	0	0	0	0	0	0	0	0	0	0	0	0	0	0	0	0
ENSG00000206532	0	1	0	0	0	0	0	0	0	1	8	0	1	0	0	0	0	1	0	0	0	0	0	0	1	0	0	3	0	0	0	3
ENSG00000242242	0	0	1	1	0	0	0	0	0	2	6	3	1	1	2	0	0	0	0	1	1	0	0	0	2	2	4	2	0	0	0	0
ENSG00000177707	37	70	29	67	239	265	204	301	214	249	1180	178	88	109	126	168	7	58	31	60	569	247	508	207	384	550	424	1128	155	188	177	249
ENSG00000153283	1089	710	1017	637	3313	3058	2772	3446	5905	5068	25041	3763	2569	2404	2491	3152	764	749	825	588	7899	2789	6261	2894	5436	7204	8252	15290	2602	2594	3233	3204
ENSG00000239311	0	0	0	0	0	0	0	0	0	0	0	0	0	0	0	0	0	0	0	0	0	0	0	0	0	0	0	0	0	0	0	0
ENSG00000244142	0	0	0	0	0	0	0	0	0	0	0	0	0	0	0	0	0	0	0	0	0	0	0	0	0	0	0	0	0	0	0	0
ENSG00000240787	1	3	2	1	3	11	8	8	11	2	14	0	1	1	2	6	0	0	1	1	14	7	5	9	14	5	6	22	3	2	4	4
ENSG00000177494	13	10	16	8	46	30	34	53	56	55	277	38	26	35	25	38	1	1	3	12	67	21	32	35	73	63	86	239	10	42	43	48
ENSG00000244720	47	45	60	24	88	83	81	92	279	237	1259	160	85	89	86	123	8	4	10	30	128	71	76	72	191	227	359	591	31	108	61	104
ENSG00000240891	268	186	204	163	235	206	183	250	176	126	1539	160	442	403	326	480	89	95	105	169	426	180	308	151	259	378	567	980	293	462	269	470
ENSG00000240766	1	0	0	4	1	0	3	1	3	3	7	0	2	7	6	1	0	0	0	3	0	0	4	2	4	7	1	7	5	2	2	8
ENSG00000144824	19	35	29	38	289	377	255	388	147	215	352	115	892	986	936	1193	1	19	12	25	491	167	417	204	197	322	122	517	1114	1649	1238	1883
ENSG00000144827	1294	935	1044	943	1081	1234	831	1260	963	729	2736	482	1013	944	1054	1339	1538	2295	1729	1801	2941	1180	2143	1254	905	1172	613	1911	1224	1363	1424	1601
ENSG00000144834	0	1	1	3	3	0	0	0	0	0	1	0	0	0	0	0	1	0	0	2	2	0	0	0	0	0	0	1	1	0	1	0
ENSG00000239439	0	0	0	0	0	0	0	0	0	0	0	0	0	0	0	0	0	0	0	0	0	0	0	0	0	0	1	0	0	0	0	0
ENSG00000176040	0	0	1	0	0	0	0	0	0	0	5	0	0	0	0	0	0	0	0	0	0	0	0	0	0	0	3	1	0	0	0	0
ENSG00000114529	8	5	10	3	12	5	3	2	9	5	44	10	48	44	19	42	3	2	3	1	7	2	6	4	18	25	9	57	10	29	25	36
ENSG00000207940	0	0	0	0	0	1	0	0	0	0	0	0	0	0	0	0	0	0	0	0	0	0	0	0	0	0	0	0	0	1	0	0
ENSG00000174500	51	33	44	38	34	33	44	40	29	21	103	19	484	581	418	722	34	33	34	21	89	23	67	19	6	3	16	37	306	416	364	503
ENSG00000261488	0	0	1	0	0	0	0	0	1	1	2	2	0	0	0	1	0	0	0	0	0	0	0	0	2	1	5	4	0	0	0	0
ENSG00000172139	0	0	0	0	0	0	0	0	0	0	2	0	0	0	0	0	0	2	0	0	0	0	2	0	1	0	2	2	0	0	0	0
ENSG00000244144	0	0	0	0	0	0	0	0	0	0	1	0	0	0	0	1	0	0	0	0	0	1	0	0	0	0	0	0	0	0	0	0
ENSG00000239482	0	0	1	0	1	0	1	1	0	0	2	0	0	0	0	0	0	0	0	0	1	0	2	2	0	1	0	0	0	0	0	1
ENSG00000283669	0	0	0	0	0	0	0	0	0	0	3	0	0	0	0	0	0	0	0	0	0	0	1	0	0	0	2	1	0	0	0	0
ENSG00000091972	0	0	0	0	3	1	0	2	0	0	0	0	0	0	0	1	0	0	0	0	6	5	0	6	0	0	0	0	0	0	0	1
ENSG00000243081	0	0	0	0	0	0	0	0	0	0	1	0	0	0	0	0	0	0	0	0	0	0	0	0	0	0	0	0	0	0	0	1
ENSG00000186265	3	3	1	3	0	2	1	5	0	4	5	0	2	1	1	3	3	1	0	3	10	6	6	6	1	0	0	3	0	0	1	2
ENSG00000242525	2	0	1	0	0	0	1	1	3	0	0	0	0	1	0	2	0	0	0	0	1	1	2	0	2	0	1	1	0	0	1	2
ENSG00000279349	3	3	9	3	4	3	4	3	0	0	4	2	1	2	3	4	2	4	1	7	4	6	3	5	3	3	2	5	2	2	3	7
ENSG00000144848	2125	1408	1728	1438	1665	1680	1420	1760	1175	852	3468	650	1584	1372	1710	1783	2136	2700	2769	2110	4183	1578	3306	1692	1068	1705	703	2440	1737	1729	2218	2115
ENSG00000138459	639	410	513	419	763	839	641	854	486	423	1783	293	476	469	565	591	482	780	635	609	2004	720	1537	718	471	695	509	1227	428	593	644	694
ENSG00000091986	3	0	2	3	4	3	3	4	8	3	26	6	43	54	59	74	0	4	3	3	4	6	3	4	6	5	5	12	60	81	101	103
ENSG00000242308	0	0	0	0	0	0	0	0	0	0	0	0	0	0	0	0	0	0	0	0	0	0	0	0	0	0	0	0	0	0	0	0
ENSG00000240893	0	0	0	1	0	2	0	0	0	0	1	0	0	0	0	0	0	0	0	0	0	0	0	0	0	0	0	0	1	0	0	0
ENSG00000242770	0	0	0	0	0	0	0	0	0	0	0	0	0	0	0	0	0	0	0	0	0	0	0	0	0	0	1	0	0	1	0	0
ENSG00000206531	0	0	0	0	0	0	0	0	0	0	1	0	1	1	1	2	0	0	0	0	0	0	0	0	0	0	3	0	3	4	17	6
ENSG00000163606	9	4	7	0	0	0	0	0	21	13	103	9	72	53	132	96	5	6	5	7	0	0	0	6	5	8	6	14	151	104	267	154
ENSG00000272844	1	2	4	2	0	1	0	1	6	0	6	0	0	5	2	4	1	1	2	2	7	1	1	0	2	5	2	3	4	1	1	1
ENSG00000163607	755	522	570	563	440	447	359	474	424	357	1323	228	390	395	546	496	563	696	644	696	1106	455	801	442	412	512	296	801	530	624	674	704
ENSG00000163608	1969	1359	1574	1340	1490	1571	1127	1664	1534	1278	5616	929	1031	1098	1170	1387	1058	1381	1276	1606	3015	1335	2138	1337	1392	1976	1479	3453	941	1278	1094	1482
ENSG00000240057	21	13	15	13	12	6	5	18	12	18	53	8	22	22	29	25	4	13	13	9	11	3	4	3	8	13	15	43	12	14	24	21
ENSG00000243483	0	0	0	1	1	0	0	2	2	1	5	1	0	1	2	0	0	0	0	0	1	0	1	1	0	3	0	1	0	1	0	3
ENSG00000241219	6	3	1	2	0	0	0	0	3	1	12	0	2	4	4	1	3	1	0	1	3	2	2	1	7	4	4	7	1	5	7	0
ENSG00000243795	2	8	5	4	0	0	0	2	4	6	16	1	7	7	10	5	4	3	1	3	2	0	3	5	3	8	1	12	1	4	10	7
ENSG00000144857	2	2	1	1	0	1	0	2	3	0	1	0	1	6	3	5	0	0	0	3	1	0	0	0	2	1	5	5	2	4	1	2
ENSG00000206530	250	181	308	233	49	70	50	74	241	203	691	150	98	116	118	153	109	89	121	277	91	45	67	51	123	191	137	387	41	166	87	163
ENSG00000283241	0	0	0	0	0	0	0	0	0	0	0	0	0	0	0	0	0	0	0	0	0	0	0	0	0	0	0	0	0	0	0	0
ENSG00000240751	14	4	14	9	2	8	3	8	13	6	21	6	4	2	8	2	2	6	2	4	2	2	3	4	9	4	1	13	4	3	0	4
ENSG00000243849	10	6	11	6	3	4	0	3	4	3	9	4	1	4	2	4	0	4	1	5	6	3	2	2	4	3	2	11	0	3	4	4
ENSG00000275670	0	0	0	0	1	0	0	1	1	0	0	0	1	2	0	0	0	0	2	4	0	0	0	1	1	0	0	0	0	0	0	0
ENSG00000163611	1311	1055	1042	973	480	554	369	650	483	407	1257	342	520	469	613	694	322	486	445	696	824	352	593	365	373	532	275	1020	388	573	531	628
ENSG00000072858	585	516	623	449	147	191	115	222	532	491	1989	315	334	291	374	378	169	145	180	349	210	116	200	109	377	578	412	1030	175	288	215	317
ENSG00000239453	65	28	73	39	3	2	1	2	7	8	40	7	12	4	11	9	30	11	57	25	7	5	5	2	9	6	13	29	2	11	6	9
ENSG00000265253	1	2	1	0	0	0	0	0	0	3	0	0	1	2	2	2	4	0	1	0	2	1	0	0	2	1	0	0	1	1	2	2
ENSG00000241529	6	9	8	1	1	0	3	3	3	1	10	1	6	7	0	1	2	1	1	1	3	0	0	1	2	8	3	4	0	2	1	0
ENSG00000176542	1461	1117	1250	1033	1012	1064	709	1094	1158	1199	5967	835	592	635	575	780	357	562	478	1086	1507	658	1096	707	1364	2062	1580	4088	416	697	439	748
ENSG00000121579	6777	4393	5741	4656	5065	5384	4064	5636	4620	3765	13177	2577	5285	4888	6002	6414	6178	9015	8214	7411	13336	4967	9543	4766	4949	7258	2565	10234	6143	6549	6734	7412
ENSG00000239280	28	16	15	11	30	29	14	12	12	10	44	6	4	14	18	7	8	6	6	31	40	23	31	28	16	26	14	50	3	15	12	17
ENSG00000242659	2	9	14	6	5	6	3	12	4	4	26	6	5	3	7	9	0	4	2	4	8	5	5	2	10	6	6	16	9	8	14	7
ENSG00000114573	1560	1253	1309	1221	1109	1320	900	1389	970	895	4530	710	1651	1466	1786	1878	723	1207	1160	1202	2025	823	1661	789	1144	1707	1252	3313	1436	1733	1834	1946
ENSG00000178075	344	341	310	348	22	55	18	61	20	33	123	32	120	129	128	171	134	267	167	321	19	18	20	15	10	12	21	62	65	70	83	82
ENSG00000240776	0	0	1	0	0	0	0	0	0	0	1	0	0	0	1	0	0	0	0	0	0	2	0	0	0	0	0	0	0	0	0	0
ENSG00000241889	11	8	16	12	5	3	2	3	7	3	13	2	5	2	3	5	4	4	4	15	3	5	1	0	2	3	2	5	1	3	2	2
ENSG00000240375	0	1	0	0	0	0	0	0	0	0	2	0	0	0	0	1	0	0	0	1	0	0	0	0	0	0	0	0	0	2	2	1
ENSG00000273394	1	1	0	3	3	2	4	5	0	0	1	0	3	3	2	4	0	1	3	4	4	1	3	1	0	0	0	0	6	1	5	3
ENSG00000184307	1284	1285	1154	1206	564	712	449	645	50	77	265	62	702	739	621	888	403	554	441	1007	779	362	554	320	43	114	46	208	350	649	346	629
ENSG00000163617	255	235	222	213	98	103	53	96	161	162	475	85	201	249	211	315	136	124	130	211	92	59	57	57	73	94	171	308	82	187	149	215
ENSG00000270880	2	2	3	3	3	3	5	8	7	0	0	1	7	3	1	3	0	4	1	2	2	0	1	2	0	0	0	1	0	0	0	2
ENSG00000151576	1105	805	989	741	978	1012	785	1112	1109	970	3971	666	1514	1332	1688	1643	476	622	587	1041	1869	803	1313	662	1106	1559	778	2417	1215	1578	1300	1745
ENSG00000240912	0	0	0	0	0	0	0	0	0	0	1	0	0	0	0	0	0	0	0	0	0	0	0	0	0	0	0	0	0	0	0	0
ENSG00000151577	0	0	0	0	0	0	0	0	3	1	7	1	0	0	0	0	0	0	0	0	0	0	0	0	0	2	5	6	0	0	0	0
ENSG00000241490	0	0	0	0	1	1	1	0	31	39	174	29	9	3	6	6	0	0	0	0	0	0	0	0	32	41	37	52	1	1	4	10
ENSG00000240487	0	0	0	0	2	0	0	0	16	20	77	10	1	5	6	2	0	0	0	0	0	0	0	0	9	32	18	43	1	3	1	4
ENSG00000174255	6	2	1	5	0	1	0	0	491	496	1804	360	2	3	7	4	1	3	0	1	0	0	0	1	502	674	428	1067	0	2	4	7
ENSG00000181847	0	2	0	3	1	0	0	0	78	56	156	27	24	39	39	55	0	0	0	0	0	0	0	1	51	48	29	85	26	44	34	44
ENSG00000259976	0	0	0	0	0	0	0	0	0	0	0	0	0	0	0	0	0	0	0	0	0	0	0	0	0	0	0	0	0	0	0	0
ENSG00000181722	186	118	120	93	191	192	136	202	135	152	841	92	112	152	114	171	30	27	33	64	225	137	188	109	128	173	347	595	33	109	82	99
ENSG00000241560	4	0	3	3	3	6	2	3	0	5	9	2	31	19	22	26	2	0	0	0	5	3	5	8	2	2	8	17	11	22	16	11
ENSG00000242290	0	3	0	1	5	3	2	4	3	1	12	2	0	1	0	0	0	0	0	1	1	1	1	2	2	6	2	10	0	0	0	3
ENSG00000241295	0	0	1	1	1	1	0	0	0	0	0	0	0	0	0	0	0	0	0	0	0	1	3	0	0	1	0	2	0	0	0	0
ENSG00000264623	0	0	0	0	0	0	0	0	0	0	2	0	0	0	0	0	0	0	0	0	0	0	0	0	0	0	0	0	0	0	0	0
ENSG00000239946	1	0	3	1	4	1	4	3	1	3	7	0	2	0	0	3	2	0	0	4	3	8	3	3	3	3	5	5	1	2	0	0
ENSG00000240002	2	0	0	2	0	3	1	0	3	1	4	0	0	0	2	0	0	0	0	2	1	2	2	0	1	3	0	5	0	0	0	0
ENSG00000242767	5	0	4	0	1	5	2	4	3	2	9	0	0	1	1	2	0	3	1	1	15	5	11	9	5	3	3	17	0	1	0	0
ENSG00000279131	1	0	2	1	0	4	1	2	5	0	6	0	2	0	1	0	0	0	0	0	3	4	4	3	4	2	2	4	0	0	0	0
ENSG00000279380	2	5	3	1	4	5	10	3	2	1	25	5	4	3	3	17	0	0	0	2	10	2	9	6	9	10	9	28	2	2	4	6
ENSG00000242880	2	1	0	0	0	1	0	0	1	0	2	0	0	0	0	0	0	0	0	0	0	0	0	0	2	1	2	0	0	0	0	0
ENSG00000244157	0	0	0	0	1	0	1	0	0	0	0	0	0	0	0	0	0	0	0	0	0	0	0	0	0	0	1	0	0	0	0	0
ENSG00000172020	0	1	0	1	0	0	0	0	0	0	3	0	0	1	1	1	0	0	0	0	0	0	0	0	1	0	0	0	0	0	1	2
ENSG00000241596	0	0	0	0	0	0	0	0	0	0	0	0	0	0	1	0	0	0	0	0	0	0	0	0	0	0	0	0	1	0	0	0
ENSG00000185565	308	258	210	293	0	1	0	0	50	47	117	29	323	359	302	389	63	169	79	179	0	0	0	0	118	179	114	349	65	142	75	123
ENSG00000243359	2	1	2	1	0	0	0	0	0	1	1	1	0	0	0	1	0	0	0	1	0	0	0	0	0	2	0	2	0	0	0	0
ENSG00000240922	0	0	0	0	0	0	0	0	0	0	0	0	6	1	0	1	0	0	0	0	0	0	0	0	2	0	0	5	0	1	3	2
ENSG00000241397	1	0	0	0	0	0	0	0	0	0	0	0	0	0	0	0	0	1	0	0	0	0	0	0	0	0	0	0	0	0	0	0
ENSG00000241156	0	0	0	0	0	0	0	0	0	0	0	0	0	0	0	0	0	0	0	0	0	0	0	0	0	0	0	0	0	0	0	0
ENSG00000198406	6	7	6	4	11	13	12	19	12	3	32	6	9	5	9	9	19	12	18	8	42	15	23	17	14	7	10	18	13	11	27	15
ENSG00000243197	1	1	0	6	0	0	0	0	0	0	0	0	0	0	0	0	0	1	1	2	0	0	0	0	0	0	1	0	0	0	0	0
ENSG00000278072	0	0	0	0	0	0	0	0	0	0	0	0	0	0	0	0	0	0	0	0	0	0	0	0	0	0	0	0	0	0	0	0
ENSG00000265433	0	0	0	0	0	0	0	0	0	0	0	0	0	0	0	0	0	0	0	0	0	0	0	0	0	0	0	0	0	0	0	0
ENSG00000274732	0	0	0	0	0	0	0	0	0	0	0	0	0	0	0	0	0	0	0	0	0	0	0	0	0	0	0	0	0	0	0	0
ENSG00000242385	1	0	0	1	0	0	0	0	0	0	0	0	0	0	0	0	0	0	0	0	0	0	0	0	0	0	0	0	0	0	0	0
ENSG00000200372	0	0	0	0	0	0	0	0	0	0	0	0	0	0	0	0	0	0	0	1	0	1	0	0	0	0	0	0	0	0	0	0
ENSG00000243014	0	0	0	0	0	0	1	1	1	0	1	0	1	0	0	1	1	2	2	0	0	1	0	0	1	0	0	1	1	3	0	1
ENSG00000206889	0	0	0	0	0	0	0	0	0	0	0	0	0	0	0	0	0	0	0	0	0	0	0	0	0	0	0	0	0	0	0	0
ENSG00000239268	83	71	49	105	0	0	0	0	0	0	0	0	0	0	0	0	24	35	30	63	0	0	0	0	0	0	0	0	0	0	0	0
ENSG00000240393	1	1	1	1	0	0	0	0	0	0	0	0	0	0	0	0	0	0	0	0	0	0	0	0	0	0	0	0	0	0	0	0
ENSG00000241213	2	6	1	2	0	0	0	0	0	0	0	0	0	0	0	0	0	0	0	2	0	0	0	0	0	0	0	1	0	0	0	0
ENSG00000242816	1	1	0	0	0	0	0	0	0	0	0	0	0	0	0	0	0	0	0	4	0	0	0	0	0	0	0	0	0	0	0	0
ENSG00000243276	0	0	0	0	0	0	0	0	0	0	0	1	0	0	0	0	0	0	0	0	0	0	0	0	0	0	0	0	0	0	0	0
ENSG00000144847	1	2	1	2	5	1	1	1	0	1	3	0	2	3	1	0	0	1	0	2	5	0	2	0	1	0	5	2	0	1	0	0
ENSG00000239877	0	0	1	0	0	0	0	0	0	0	0	0	0	0	0	0	4	0	0	0	0	0	0	0	0	0	0	0	0	0	0	0
ENSG00000163424	0	0	1	0	2	0	1	0	0	0	2	0	0	0	0	0	0	0	0	0	1	0	0	0	0	0	0	0	0	0	0	0
ENSG00000251012	0	0	0	0	0	0	0	0	0	0	0	0	0	0	0	0	0	0	0	0	0	0	0	0	0	0	0	0	0	0	0	0
ENSG00000114638	1	1	0	1	3	1	2	6	1	0	0	1	0	0	1	3	0	0	0	1	5	8	1	2	1	1	0	5	0	0	1	1
ENSG00000121578	522	440	432	418	471	560	390	565	253	198	905	172	352	350	305	399	340	464	377	383	990	383	641	356	210	336	277	620	287	384	336	336
ENSG00000240254	1	8	2	1	4	0	2	0	0	1	2	2	2	2	3	2	1	2	0	3	2	3	1	3	2	3	1	6	0	1	2	1
ENSG00000031081	141	129	157	126	480	550	370	504	190	276	356	165	516	620	775	803	52	83	89	169	543	262	399	219	179	401	215	542	327	517	500	647
ENSG00000242829	0	3	0	5	8	4	5	4	2	2	4	3	6	3	5	5	0	0	0	2	7	8	7	4	0	2	2	5	3	6	7	4
ENSG00000241155	5	4	9	1	15	21	11	15	12	2	3	1	12	16	26	17	3	6	5	14	16	17	13	10	5	2	0	6	9	11	14	15
ENSG00000207310	0	0	0	0	1	0	0	0	0	0	2	0	0	0	1	0	0	0	0	0	0	0	1	0	0	0	0	0	0	0	0	0
ENSG00000176142	1192	759	948	798	714	847	604	871	503	432	1820	333	542	547	752	802	795	1116	1153	1138	1800	717	1227	673	530	691	499	1209	678	713	903	863
ENSG00000163389	972	628	909	686	1295	1278	1044	1423	794	624	2827	487	557	602	646	718	810	1165	1289	1348	2953	1232	2153	1319	738	1020	693	1556	603	924	921	1038
ENSG00000272662	7	7	7	8	3	9	4	4	7	1	28	2	7	0	3	5	2	7	2	6	4	0	1	3	0	10	6	11	2	7	4	3
ENSG00000113845	1721	1207	1516	1254	923	917	694	1066	1026	825	3774	702	1148	1069	1241	1418	1945	2507	2034	1740	2145	869	1664	771	1021	1505	796	2286	1310	1255	1605	1605
ENSG00000121594	0	5	1	2	6	1	1	1	4	1	12	4	4	12	19	28	0	3	1	8	1	0	0	9	5	2	1	8	2	3	19	14
ENSG00000272967	0	0	0	0	0	0	0	0	0	0	0	0	0	0	1	0	0	0	0	0	0	0	0	0	0	0	0	0	0	0	0	0
ENSG00000144843	4	7	1	4	0	1	0	3	0	0	1	0	5	3	3	9	1	4	1	6	0	0	0	0	0	0	1	3	0	2	11	5
ENSG00000144837	0	0	1	0	2	1	0	0	0	0	0	0	3	4	6	4	0	0	0	0	1	1	0	0	0	1	0	0	5	2	3	3
ENSG00000242052	1	0	0	0	2	0	0	1	0	0	0	0	0	0	0	0	0	0	0	0	1	0	0	0	0	0	0	3	0	1	0	0
ENSG00000121577	9	18	16	17	35	49	33	43	54	49	192	34	29	47	37	60	7	21	14	19	88	38	71	46	59	66	65	157	35	52	35	71
ENSG00000138495	917	636	743	651	399	444	274	445	415	292	1439	254	437	377	400	489	1208	1410	1658	1449	943	398	673	474	434	615	326	980	534	503	656	641
ENSG00000183833	0	0	0	0	0	0	0	0	0	0	0	0	1	3	5	3	0	0	0	0	0	0	0	0	0	0	0	0	0	1	0	0
ENSG00000239994	0	0	0	0	0	0	0	0	0	0	0	0	0	0	0	0	0	0	0	0	0	0	0	0	0	0	0	0	0	0	0	0
ENSG00000144852	4	1	0	2	1	1	0	0	0	0	1	1	1	0	0	0	0	0	2	3	2	1	0	0	0	1	1	0	0	1	0	0
ENSG00000240471	0	0	0	0	0	0	0	0	0	0	0	0	0	0	0	0	0	0	0	0	0	0	0	0	0	0	0	1	0	0	0	0
ENSG00000082701	4240	3054	3698	3077	3267	3143	2666	3491	2029	1651	8625	1321	1877	1837	1692	2199	1300	1596	1665	2568	6337	2563	4621	2215	1929	2985	2061	5690	1013	1485	1059	1584
ENSG00000239835	3	3	9	5	0	1	2	1	5	3	12	0	0	2	1	1	2	1	2	5	5	8	7	6	1	5	6	24	1	4	3	6
ENSG00000241546	6	3	2	3	2	1	1	3	0	2	4	1	0	2	0	2	0	1	0	4	6	3	1	4	3	6	0	3	0	1	1	1
ENSG00000242622	176	186	176	141	31	37	40	37	62	54	307	46	53	66	50	55	50	56	48	102	59	31	48	27	50	68	44	120	20	54	32	59
ENSG00000244308	1	3	3	3	0	0	0	1	0	0	6	0	3	3	3	3	0	1	0	2	0	0	0	1	1	0	0	0	1	0	1	1
ENSG00000244139	0	1	0	0	0	0	0	0	0	0	1	0	0	0	0	0	0	0	0	0	0	0	0	0	0	0	1	0	0	4	1	1
ENSG00000175697	4	9	8	3	0	1	1	3	1	0	2	1	1	1	1	1	1	3	5	11	7	3	2	6	0	5	0	4	1	5	1	0
ENSG00000206721	0	0	0	0	0	0	0	0	0	0	0	0	0	0	0	0	0	0	0	0	0	0	0	0	0	0	0	0	0	0	0	0
ENSG00000240882	1	1	1	1	0	2	0	0	1	0	2	1	0	0	0	1	3	5	4	8	2	4	2	6	1	2	1	2	0	1	1	1
ENSG00000163428	5042	4257	4035	4077	3813	3917	2973	4138	1999	1671	6363	1272	3882	3810	3954	4610	2574	4088	3343	5044	8209	3441	5557	3100	2370	3543	1373	5472	3405	4425	3390	4849
ENSG00000240661	1	0	0	2	3	0	1	0	6	6	10	4	0	1	0	0	0	1	0	1	1	1	0	0	3	3	3	8	0	0	0	0
ENSG00000282950	0	0	0	0	0	0	0	0	0	0	0	0	0	0	1	0	0	0	0	0	0	0	0	0	0	0	0	0	0	0	0	0
ENSG00000242613	0	0	0	0	0	0	0	0	0	0	0	0	0	0	0	0	0	0	0	0	0	0	0	0	0	1	0	0	0	0	0	0
ENSG00000163430	35	72	30	65	12	23	8	35	817	698	1879	555	10	12	17	21	9	47	8	62	9	7	8	5	522	712	321	1478	1	2	2	4
ENSG00000240774	0	0	0	0	0	0	0	0	0	1	0	0	0	0	0	0	0	0	0	0	0	0	0	0	0	1	0	3	0	0	0	0
ENSG00000244441	1	0	0	0	0	1	0	0	0	1	1	0	0	0	0	0	0	0	0	0	0	0	0	0	0	2	3	0	0	0	1	0
ENSG00000065518	3168	2218	2706	2363	1771	1752	1255	1950	1325	892	3846	813	1505	1343	1429	1756	3128	3019	3585	3921	3758	1487	2801	1852	1124	1600	774	2540	1722	1528	1705	2064
ENSG00000113924	10	15	14	3	5	3	3	4	6	1	19	1	0	2	0	2	0	3	4	19	1	1	0	2	8	2	4	4	0	5	1	1
ENSG00000144840	1297	940	1105	950	441	554	365	617	629	498	2122	366	508	438	513	598	905	1320	989	1057	781	315	525	312	664	912	387	1391	446	473	443	594
ENSG00000240452	1	0	0	1	0	0	0	1	0	0	0	0	0	1	0	0	0	0	0	3	0	0	0	0	1	0	3	6	0	0	0	0
ENSG00000243257	0	0	0	1	0	1	0	0	0	0	1	0	0	1	1	0	0	0	1	0	0	1	0	0	0	1	2	1	0	0	0	0
ENSG00000153767	1037	785	910	781	532	534	443	613	425	333	1420	198	597	526	690	693	468	693	595	645	972	363	805	365	456	597	299	1025	515	553	537	565
ENSG00000213371	0	1	0	0	0	0	0	0	1	0	2	0	0	0	1	2	0	1	0	0	0	0	0	0	0	0	0	0	0	0	0	1
ENSG00000243593	0	0	0	0	0	0	0	0	0	0	0	0	0	0	0	0	0	0	0	0	0	0	0	0	0	0	0	0	0	0	0	0
ENSG00000145087	147	122	102	83	20	19	13	12	10	8	53	11	112	95	99	116	71	85	111	122	39	19	20	33	33	27	5	42	67	76	105	102
ENSG00000270782	1	0	0	0	0	0	0	0	0	0	0	0	0	0	0	0	0	0	0	0	0	0	0	0	0	0	0	0	0	0	0	0
ENSG00000264477	0	0	0	0	0	0	0	0	0	0	0	0	0	0	0	0	0	0	0	0	0	0	0	0	0	0	0	0	0	0	0	0
ENSG00000243813	0	0	0	0	0	0	0	0	0	0	0	0	0	0	0	0	0	0	0	0	0	0	0	0	0	0	0	0	1	0	2	0
ENSG00000242103	1	0	0	1	0	0	0	0	0	0	0	0	0	0	0	0	0	0	0	0	0	0	0	0	0	0	0	0	0	0	0	0
ENSG00000051341	3469	2498	3035	2454	2335	2519	1792	2697	1682	1338	4823	1072	1899	1605	2208	2166	953	1015	1140	2147	4269	1910	2647	2049	1602	2262	606	4483	1007	1787	1549	2137
ENSG00000242445	4	4	6	4	5	5	8	3	10	0	16	3	7	5	5	7	1	4	0	7	16	7	7	10	4	8	0	29	0	8	14	13
ENSG00000186103	0	0	0	0	0	0	0	0	0	0	0	0	0	0	0	0	0	0	0	0	0	0	1	0	0	0	0	0	0	2	0	0
ENSG00000163833	9	5	6	7	7	4	4	12	9	3	49	8	7	6	4	4	5	2	3	9	13	7	6	4	7	15	11	19	1	4	0	5
ENSG00000180353	8232	5116	7523	6009	8374	8474	6535	9418	6812	4673	29690	4130	6837	5969	6831	7907	2688	3300	4239	4580	14714	6540	12918	5607	5438	7745	7049	16469	4641	5532	5490	6772
ENSG00000243544	17	6	18	8	14	10	14	7	24	19	136	17	9	13	10	13	6	7	3	18	12	10	11	12	26	23	25	42	5	12	10	18
ENSG00000222057	10	7	9	6	6	6	8	6	9	11	89	3	13	6	9	6	1	0	2	8	9	7	10	13	23	17	13	27	2	6	3	8
ENSG00000173230	4640	3416	3896	3382	3197	3713	2945	4166	2289	2004	10410	1443	2835	2639	2983	3452	1412	1244	1277	2549	6946	2814	4620	2731	1744	2605	2925	6553	1240	2090	2188	2763
ENSG00000173226	1434	923	1135	972	1485	1444	1120	1483	1468	1074	4587	794	1763	1460	2082	2042	1513	1941	1648	1524	3384	1420	2517	1496	1193	1728	1027	3211	1593	1728	2314	2239
ENSG00000145088	762	534	639	590	397	389	310	364	98	69	284	52	195	200	401	316	728	890	1023	1149	1011	383	769	449	75	123	47	175	285	268	439	422
ENSG00000163406	947	739	726	785	346	242	237	321	112	114	338	50	121	123	163	177	202	264	184	526	464	208	239	173	40	89	41	183	45	114	87	162
ENSG00000145103	16	9	11	6	4	2	4	3	0	3	4	2	68	69	82	88	2	7	4	11	5	4	1	1	1	0	1	4	31	40	52	40
ENSG00000202388	0	0	0	0	0	0	0	0	0	0	0	0	0	0	0	1	0	0	0	0	0	0	0	0	0	0	0	0	0	0	0	0
ENSG00000114013	7	1	3	2	0	1	0	2	145	139	565	106	1526	1313	1937	1828	1	2	0	4	0	0	0	0	125	256	121	385	1371	1538	1925	2047
ENSG00000036828	1	12	8	3	0	0	0	0	0	0	0	0	10	17	21	23	2	2	1	4	0	0	0	0	0	0	0	0	18	11	15	37
ENSG00000283187	0	0	0	0	0	0	0	0	0	0	0	0	0	0	0	0	0	0	0	0	0	0	0	0	0	0	0	0	0	0	0	0
ENSG00000240236	0	0	0	0	0	0	0	0	0	0	0	0	0	0	1	0	0	0	0	0	0	0	0	0	0	0	0	0	0	0	0	0
ENSG00000121552	14	20	19	12	1	1	0	0	0	0	4	0	365	277	412	307	27	18	25	16	0	0	0	0	0	0	0	4	505	424	695	498
ENSG00000160124	1005	594	806	599	626	545	505	583	491	345	1286	214	695	606	800	704	973	941	1081	1209	1231	494	996	755	431	534	206	771	820	764	939	841
ENSG00000114023	4535	3218	3819	3342	1974	1831	1501	2305	1635	1015	1855	660	1764	1601	1483	2059	2700	2898	2409	2335	1555	605	1020	817	533	805	355	992	1235	1074	1271	1348
ENSG00000196981	331	219	300	197	130	176	124	175	305	263	1328	231	208	180	222	269	207	230	238	326	340	167	256	157	300	333	349	726	113	187	186	262
ENSG00000272758	89	49	70	61	52	60	45	48	85	84	398	53	46	38	46	84	22	30	17	77	92	57	58	58	75	113	86	224	27	57	44	53
ENSG00000242531	3	7	10	10	5	8	5	9	4	8	59	8	5	4	6	7	1	2	3	13	10	5	3	3	10	5	10	37	3	4	3	5
ENSG00000114030	1857	1362	1641	1389	1815	1928	1467	1988	1444	1137	6745	943	1608	1464	1794	2004	923	1286	1145	1689	4020	1508	2774	1483	1648	2474	1640	4221	1446	1937	1815	2106
ENSG00000275348	3	4	2	0	0	1	2	2	0	1	16	0	2	4	2	1	0	1	1	2	1	3	1	4	1	7	0	6	1	0	4	4
ENSG00000138496	674	449	581	473	730	741	568	817	754	583	3196	560	609	607	687	906	343	378	434	435	1147	452	795	439	843	1175	694	2266	677	1154	874	1529
ENSG00000163840	1176	832	1039	833	1338	1437	1088	1468	1961	1574	8536	1314	1701	1678	1713	2053	274	451	469	662	1929	789	1522	671	2091	2870	1675	5493	1227	1918	1336	1999
ENSG00000173200	351	242	273	212	408	384	320	421	672	644	3421	420	41	49	40	77	105	82	84	241	562	205	330	250	430	581	825	1443	28	35	23	53
ENSG00000243918	0	0	0	0	0	0	0	0	0	0	0	0	0	1	0	0	0	0	0	0	0	0	0	0	0	0	0	0	0	0	0	0
ENSG00000173193	1444	969	1251	984	2690	2915	2031	2983	2813	2272	11892	1976	1787	1887	1919	2284	288	401	382	777	4137	1752	2716	1545	2584	3880	2369	7826	1439	2580	1961	2859
ENSG00000169087	430	279	316	317	247	254	186	219	136	141	945	119	249	221	230	251	313	363	293	408	554	227	384	235	170	197	293	645	223	306	316	368
ENSG00000138463	192	135	185	166	108	125	82	124	26	18	239	26	56	54	55	45	86	132	99	143	252	102	187	89	34	47	85	141	34	60	30	48
ENSG00000273033	157	175	138	181	61	77	48	81	18	21	263	26	59	85	45	95	20	30	14	94	100	50	66	38	21	24	106	144	23	57	15	44
ENSG00000082684	1	0	0	1	6	10	2	7	0	4	31	1	8	10	8	17	1	0	0	0	0	1	0	0	0	2	15	20	0	8	8	15
ENSG00000065485	683	604	537	647	1700	1855	1237	2047	270	221	736	201	753	683	601	854	292	499	350	632	2170	955	1643	881	233	420	114	604	473	565	440	662
ENSG00000121542	350	264	243	231	286	297	194	325	313	244	955	176	303	274	283	330	343	493	329	397	508	223	362	242	199	276	212	590	236	269	266	317
ENSG00000273454	0	2	4	2	5	3	7	5	5	1	7	1	4	3	2	6	1	0	1	1	5	2	2	3	0	4	0	7	3	3	2	7
ENSG00000173175	2	9	8	13	19	16	11	16	10	0	31	3	15	17	11	13	2	6	2	28	34	19	19	20	0	9	4	7	7	13	10	17
ENSG00000272678	0	0	0	0	0	0	0	0	0	0	0	0	0	0	0	0	0	0	0	0	0	0	0	0	0	0	0	0	0	0	0	0
ENSG00000206527	1858	1399	1493	1308	1414	1361	1058	1409	1247	1065	4030	775	1037	989	1108	1327	894	1331	1172	1483	2760	1051	2001	915	1216	1847	778	2761	1004	1232	1130	1341
ENSG00000239523	44	27	37	37	29	39	17	37	29	17	85	19	60	50	56	89	30	36	35	28	67	24	40	27	29	44	15	42	41	44	73	44
ENSG00000065534	60	78	79	80	1175	1452	1072	1495	60	44	231	27	87	96	66	124	31	33	27	71	1625	806	1410	734	45	89	38	141	47	102	90	99
ENSG00000250174	0	0	0	0	5	1	0	0	0	0	0	0	0	2	0	1	0	0	0	0	4	0	1	1	0	0	0	0	0	0	0	1
ENSG00000273123	0	0	0	0	5	1	1	2	0	0	0	0	0	0	0	0	0	0	0	0	2	1	0	4	0	0	0	0	0	0	0	0
ENSG00000207002	0	0	0	0	0	0	1	0	0	0	0	0	0	0	0	0	0	0	0	0	0	0	0	1	0	0	0	0	0	0	0	0
ENSG00000175455	5586	4733	4581	4562	4697	5294	3784	5406	3300	3113	10761	2238	3214	3387	3285	4468	2589	2690	2487	5731	8331	3965	5018	4104	2639	3921	1966	7339	1321	3298	2125	3948
ENSG00000065371	0	0	0	0	0	0	0	1	0	0	0	0	0	0	1	1	0	0	0	1	0	0	1	0	1	0	0	0	0	0	0	0
ENSG00000160145	10	23	23	21	183	157	116	184	14	20	7	6	370	339	345	414	4	12	16	20	207	109	123	116	2	9	0	14	231	323	274	349
ENSG00000266383	0	0	0	0	1	4	1	0	0	0	0	0	1	4	4	1	0	0	0	0	0	0	0	1	0	0	0	0	1	2	0	3
ENSG00000240622	0	0	0	0	0	1	1	0	0	0	0	0	12	4	7	8	0	0	0	0	0	1	0	0	0	0	0	0	2	9	4	3
ENSG00000276656	0	0	0	0	0	0	0	0	0	0	0	0	0	0	0	0	0	0	0	0	0	0	0	0	0	0	0	0	0	0	0	0
ENSG00000252751	0	0	0	0	0	0	0	0	0	0	0	0	0	1	1	0	0	0	0	0	0	1	0	0	0	0	0	0	1	0	0	2
ENSG00000260391	0	0	0	0	0	0	0	0	0	0	0	0	0	0	0	0	0	0	0	0	0	0	0	0	0	0	0	0	0	0	0	0
ENSG00000114491	1283	852	1085	978	1479	1532	1170	1701	1172	911	3749	747	1432	1302	1610	1687	1453	2358	2022	1659	3663	1519	2707	1443	1169	1680	776	2420	1641	1788	2036	2108
ENSG00000265981	3	7	4	1	7	3	0	5	2	2	14	4	4	1	0	6	0	0	2	7	2	4	2	8	1	4	3	3	2	3	0	1
ENSG00000242199	14	3	3	4	4	3	8	7	6	4	18	1	2	4	4	7	4	2	2	10	13	10	13	11	4	8	2	12	1	5	1	2
ENSG00000082781	12	16	19	20	43	38	43	74	16	19	59	11	43	61	43	77	5	7	15	19	93	79	102	73	7	10	28	31	26	73	41	59
ENSG00000244286	1	0	1	0	0	0	0	1	0	0	0	0	0	2	0	0	0	0	0	0	0	3	0	0	0	2	1	1	0	0	0	0
ENSG00000280042	0	0	0	1	1	1	2	1	0	0	1	1	0	1	0	0	0	0	0	1	3	5	0	1	0	0	0	3	0	2	0	2
ENSG00000276626	0	0	0	0	0	0	0	0	0	0	0	0	0	0	0	0	0	0	0	0	0	0	0	0	0	0	0	0	0	0	0	0
ENSG00000243986	0	0	0	0	0	1	0	0	0	0	0	0	0	0	0	0	0	0	0	0	0	0	0	0	1	0	0	0	0	1	0	1
ENSG00000173702	0	1	0	0	1	0	0	2	0	2	1	0	90	98	175	118	0	0	0	0	1	0	0	0	0	0	3	2	59	108	107	75
ENSG00000173706	396	697	368	660	1513	1610	957	1454	2618	2769	8836	1940	1900	1941	1869	2427	52	229	65	353	1914	756	1235	641	1531	3363	2009	5733	1103	2017	1086	2260
ENSG00000221955	8	4	10	9	8	11	4	5	55	36	93	26	28	40	43	32	0	0	1	1	11	5	6	6	11	29	9	12	20	20	18	39
ENSG00000199327	0	0	0	0	0	0	0	0	0	0	0	0	0	0	0	0	0	0	0	0	0	0	0	0	0	0	0	0	0	0	1	0
ENSG00000264986	0	0	0	0	0	1	0	0	0	0	0	0	0	0	0	0	0	0	0	0	0	0	0	0	0	0	0	0	0	0	0	0

ENSG00000163848	1938	1321	1529	1260	1785	1844	1297	1954	1615	1389	5475	992	1153	1058	1182	1270	945	1191	1197	1455	3602	1450	2451	1521	1589	2333	1408	4128	881	1036	1006	1080
ENSG00000229048	7	7	5	8	3	1	0	1	0	1	3	1	3	5	1	2	1	2	1	1	4	0	4	1	1	1	1	12	0	1	1	2
ENSG00000243508	8	5	2	0	3	5	1	2	4	2	7	0	3	7	2	2	2	2	0	4	13	2	6	4	16	9	3	40	0	5	9	6
ENSG00000252953	0	0	0	0	0	0	0	0	0	0	0	0	0	0	0	0	0	0	0	0	0	0	0	0	0	0	0	0	0	0	0	0
ENSG00000114520	1540	885	1185	980	953	1066	792	1100	1144	879	4533	703	971	846	921	1114	1668	2214	2163	1734	2760	1142	1891	1160	1213	1779	1059	3274	1028	1046	1434	1260
ENSG00000201800	2	1	2	2	4	0	1	1	3	0	11	1	3	1	3	0	0	0	0	2	3	3	0	2	1	1	4	7	0	0	1	0
ENSG00000252965	1	1	1	1	1	3	3	1	2	0	8	3	1	1	4	1	4	1	0	2	7	1	5	2	0	7	1	4	1	1	1	1
ENSG00000144909	1177	794	908	736	1002	1000	749	1082	547	480	1925	361	647	665	778	881	527	748	723	942	2126	833	1710	814	734	1079	383	1536	720	922	808	1023
ENSG00000243072	0	0	0	0	0	0	0	0	0	0	0	0	0	0	0	0	0	0	0	0	0	0	0	0	0	0	0	1	0	0	0	0
ENSG00000242001	0	0	0	0	0	0	0	0	0	0	0	0	0	0	0	0	0	0	0	0	0	0	0	0	0	0	0	0	0	0	0	0
ENSG00000234458	0	0	0	0	0	0	0	0	0	0	0	0	0	0	0	0	0	0	0	0	0	0	0	0	0	1	0	0	0	0	1	0
ENSG00000243429	2	0	0	2	1	0	0	1	3	0	0	0	1	1	0	1	2	0	0	0	0	2	0	0	0	0	0	0	0	0	1	0
ENSG00000228963	0	0	0	0	0	0	0	0	0	0	0	0	0	0	0	0	0	0	0	0	0	0	0	0	0	0	0	0	0	0	0	0
ENSG00000239978	0	0	0	0	0	1	0	2	0	0	0	0	0	0	0	0	0	0	0	0	2	0	0	0	0	0	0	0	0	0	0	0
ENSG00000179170	0	0	0	0	0	0	1	0	0	0	0	0	0	0	0	0	0	0	0	0	1	0	0	0	0	0	0	0	0	0	0	0
ENSG00000239432	0	0	0	0	0	0	0	0	0	0	0	0	0	0	0	0	0	0	0	0	0	0	0	0	0	0	0	0	0	0	0	0
ENSG00000272840	0	3	2	0	1	0	0	1	0	0	0	0	0	1	0	3	0	1	0	1	1	1	0	0	0	0	0	1	1	0	2	0
ENSG00000239804	1	1	0	3	0	0	0	1	0	0	1	0	1	1	0	0	0	0	0	0	0	0	0	1	0	0	0	0	0	0	0	1
ENSG00000221737	0	3	0	0	0	0	1	0	0	0	0	0	1	1	0	0	0	1	0	0	0	0	0	0	0	0	0	0	0	2	0	2
ENSG00000242229	0	3	1	4	1	2	0	0	3	0	1	0	3	4	0	3	0	0	0	0	0	0	1	0	0	0	0	0	0	3	2	1
ENSG00000241346	0	0	0	1	0	0	0	0	0	0	0	0	0	1	0	3	0	0	0	0	0	0	0	0	0	0	3	0	0	1	0	1
ENSG00000241288	14	24	12	20	14	18	17	32	23	21	36	10	15	26	7	37	2	7	1	17	14	8	15	6	10	3	10	27	5	16	11	13
ENSG00000241278	1	0	4	4	4	2	2	3	2	2	1	0	0	4	2	3	0	0	0	3	1	5	2	3	2	0	5	5	2	2	0	4
ENSG00000248787	0	0	2	0	2	1	1	4	0	1	3	0	1	3	1	0	0	0	0	0	2	1	2	3	1	0	0	3	0	0	1	0
ENSG00000171084	133	114	117	121	159	161	127	173	40	24	93	15	111	102	82	124	61	112	68	134	235	117	173	87	22	34	21	53	49	79	46	78
ENSG00000189366	38	28	33	30	32	39	25	42	9	11	64	7	21	42	13	25	29	63	28	50	49	19	40	20	5	9	18	31	16	22	20	33
ENSG00000241439	0	1	4	3	1	0	0	0	0	0	3	0	0	0	0	0	0	0	0	0	0	1	0	0	1	0	0	1	0	0	1	0
ENSG00000114547	64	41	41	58	36	34	22	32	9	7	24	7	14	17	16	29	15	32	24	52	54	15	29	20	6	10	1	18	2	25	10	21
ENSG00000114544	910	750	741	714	488	578	346	550	494	465	2073	338	697	684	671	981	431	695	509	655	775	339	551	264	437	729	560	1415	557	763	686	792
ENSG00000248607	1	3	2	2	0	0	0	0	0	0	2	1	6	0	3	0	0	1	0	0	2	0	1	0	2	0	4	2	0	0	3	1
ENSG00000250012	5	23	22	14	1	2	3	11	7	1	20	2	4	5	7	16	14	12	8	14	6	4	5	3	2	10	3	10	2	5	11	7
ENSG00000144908	3	6	4	8	0	0	1	0	1	0	7	0	1	4	2	1	0	6	3	0	1	1	0	0	0	5	3	4	0	1	0	5
ENSG00000250218	0	0	0	0	0	0	0	0	0	1	0	0	0	0	0	0	0	0	0	1	0	0	0	0	0	0	0	0	0	0	0	0
ENSG00000272020	0	0	0	0	0	0	0	0	0	0	0	0	0	0	0	0	0	0	0	0	0	0	0	0	0	0	0	0	0	0	0	0
ENSG00000246022	0	0	0	0	0	0	0	0	0	0	0	0	0	0	0	0	0	0	0	0	0	0	0	0	0	0	0	0	0	0	0	0
ENSG00000250934	0	0	0	0	0	0	0	0	0	0	0	0	0	0	0	0	0	0	0	0	0	0	0	0	0	1	0	0	0	0	0	0
ENSG00000251448	0	0	0	0	0	0	0	0	0	0	0	0	0	0	0	0	0	0	0	0	0	0	0	0	0	0	0	0	0	0	0	0
ENSG00000163884	3	1	0	2	0	1	0	1	5	3	5	7	0	5	2	6	0	0	0	2	0	0	0	0	6	3	2	14	2	3	2	3
ENSG00000249833	0	0	0	0	0	0	0	0	0	0	0	0	1	0	0	0	0	0	0	0	0	0	0	0	0	0	0	0	1	0	0	0
ENSG00000163885	3	1	1	1	20	13	13	24	7	5	27	12	4	5	4	12	0	0	1	5	32	16	25	24	2	4	5	24	3	15	4	17
ENSG00000279328	4	2	4	5	6	15	4	14	7	3	27	3	7	8	6	10	1	0	0	3	23	13	20	20	3	2	5	14	2	15	6	8
ENSG00000070476	1057	821	1042	908	1418	1603	1025	1541	782	686	3363	588	631	632	614	832	253	330	363	854	2402	1205	1551	1051	590	1039	849	2136	345	670	400	697
ENSG00000159650	3	0	7	3	293	312	175	319	0	0	0	0	1	2	0	4	1	2	2	2	360	190	268	128	0	0	0	1	1	0	0	2
ENSG00000180767	0	2	0	0	277	291	147	299	0	0	0	0	0	0	0	1	0	0	0	0	377	160	311	139	0	0	0	0	0	0	0	0
ENSG00000180697	0	0	1	0	4	2	2	6	0	0	0	0	0	0	0	0	0	0	0	1	10	3	4	9	0	0	0	0	0	0	0	0
ENSG00000283374	3	3	3	0	2	6	0	1	0	0	0	0	1	0	1	0	0	1	0	3	3	1	0	0	0	0	0	0	0	0	0	0
ENSG00000206483	0	0	0	0	0	0	0	0	0	0	0	0	0	0	0	0	0	0	0	0	0	0	0	0	0	0	0	0	0	0	0	0
ENSG00000197763	992	859	725	831	755	827	550	850	145	134	428	104	176	201	115	223	305	666	372	773	1272	567	913	537	117	170	73	322	86	135	53	115
ENSG00000248557	41	62	45	46	39	35	38	49	9	8	24	15	5	3	9	17	8	22	7	43	76	38	31	54	9	9	9	13	1	6	3	8
ENSG00000198284	3	2	2	0	0	0	1	2	0	0	0	0	0	0	0	0	0	0	1	1	1	0	2	2	0	0	0	0	0	0	0	0
ENSG00000248850	1	0	2	2	0	0	0	0	0	0	0	0	0	0	0	0	1	0	0	0	0	0	0	0	0	0	0	0	0	0	0	0
ENSG00000159685	801	594	603	691	307	298	192	296	221	170	556	138	420	439	381	517	535	755	542	601	485	185	348	162	163	225	92	385	360	400	405	462
ENSG00000249558	4	5	6	2	2	0	5	4	2	1	6	0	5	3	5	4	1	4	1	3	5	1	1	4	4	2	0	10	0	4	0	4
ENSG00000280053	1	2	0	4	0	0	0	0	5	3	7	3	1	2	4	6	1	0	1	2	0	4	1	1	1	4	6	1	3	2	1	6
ENSG00000114554	1875	2008	1927	1904	1460	1614	916	1641	5615	4847	21155	4705	1801	1649	1601	2332	473	735	530	1706	2141	1017	1283	888	3058	6465	1801	9284	1186	1867	1082	1923
ENSG00000214324	30	17	32	17	36	38	37	34	0	0	0	0	0	0	0	0	10	9	3	26	26	30	16	36	1	0	0	0	0	0	0	0
ENSG00000212556	51	51	62	28	89	88	64	72	0	0	0	0	0	0	0	1	31	40	31	49	131	53	66	49	0	0	0	0	0	0	1	0
ENSG00000243016	31	26	19	22	74	63	37	55	0	0	0	0	1	4	0	0	7	4	2	19	75	26	40	21	0	0	0	0	0	0	0	0
ENSG00000201288	1	0	0	0	2	3	3	4	0	0	0	0	0	0	0	2	0	0	0	0	4	2	0	6	0	0	0	0	0	0	0	0
ENSG00000239620	5	1	0	3	2	21	13	9	0	0	0	0	0	0	1	0	2	4	2	0	10	5	17	13	0	0	0	0	0	0	0	0
ENSG00000244215	0	0	0	0	0	0	0	0	0	0	0	0	1	0	0	0	0	0	0	0	0	0	0	0	0	0	0	0	0	0	0	0
ENSG00000239921	0	0	0	1	1	0	1	0	0	1	4	0	0	1	0	1	0	0	0	0	2	0	0	0	1	0	1	1	0	0	0	0
ENSG00000240562	0	0	0	0	0	0	0	0	0	0	0	0	0	0	0	0	0	0	0	0	0	0	0	0	0	0	0	0	0	0	0	0
ENSG00000282975	0	1	0	0	1	1	0	0	2	1	5	1	0	1	0	0	0	0	0	0	0	0	0	0	4	2	0	2	0	0	0	0
ENSG00000282860	2	0	0	1	2	4	4	3	2	8	19	4	0	3	2	4	0	2	0	0	13	3	2	4	4	10	2	26	0	2	3	1
ENSG00000163870	842	581	709	593	750	681	505	734	734	619	2309	539	472	473	575	611	415	607	425	632	1304	551	1009	464	633	994	485	1569	457	579	526	634
ENSG00000275067	2	0	0	3	0	0	0	0	0	0	0	0	2	2	0	0	1	0	0	0	1	1	0	0	0	0	0	2	0	0	0	0
ENSG00000276083	0	1	2	0	0	2	2	1	0	2	2	0	1	0	1	2	0	0	0	3	4	1	1	5	0	1	0	3	0	4	1	1
ENSG00000073111	7484	5784	6815	5567	9269	9435	7207	9871	6583	5164	13888	3672	7677	6565	6308	8336	1740	2913	3128	4560	14316	6049	11649	5403	4229	7463	2232	10557	4924	5657	4833	6153
ENSG00000114631	23	22	18	33	36	17	11	18	148	206	274	149	10	14	6	16	13	14	10	24	42	25	39	31	62	174	49	240	9	9	9	8
ENSG00000114626	716	555	631	577	387	400	272	405	530	519	3330	502	185	227	164	298	233	237	215	351	567	278	442	280	353	565	1083	1574	113	197	148	217
ENSG00000074416	8	13	10	20	2721	2677	1858	2703	0	0	9	0	2	2	0	10	4	20	7	23	3511	1501	2735	1270	1	2	0	1	1	2	0	0
ENSG00000187715	0	0	2	0	2	1	0	2	2	2	14	1	2	0	1	2	0	0	0	3	2	1	0	0	0	1	3	4	0	0	0	0
ENSG00000058262	4920	3535	4643	3675	5677	5909	4238	6106	3564	2965	16106	2670	3244	2987	3696	4252	2362	4473	3662	4634	10731	4251	7912	3702	3697	6004	3514	10379	3430	4246	3926	4737
ENSG00000175792	2169	1499	1831	1513	1908	2011	1473	2227	2073	1366	5651	1163	3805	3290	4359	4557	2336	3565	2753	2031	4267	1716	3372	1484	1939	2741	857	3643	4372	4126	5443	4956
ENSG00000222627	1	1	1	3	2	3	1	3	3	4	7	1	2	2	4	2	0	1	1	0	1	5	3	1	0	2	4	7	0	6	2	2
ENSG00000239608	8	4	9	8	11	7	7	12	4	2	30	4	4	7	6	7	5	4	0	14	10	4	4	9	5	10	8	18	4	8	5	12
ENSG00000212344	0	0	2	0	0	0	0	0	0	0	1	0	0	0	0	0	0	0	1	0	0	0	0	0	0	0	0	0	0	0	2	1
ENSG00000132394	487	442	423	396	645	640	411	649	501	355	1161	285	523	476	432	621	166	324	202	372	893	397	733	314	324	535	240	860	397	509	386	487
ENSG00000179407	0	0	1	1	0	1	0	1	0	0	2	0	0	2	1	2	0	0	0	1	0	2	0	2	0	2	0	3	0	0	0	1
ENSG00000242049	0	0	0	0	0	0	0	1	0	0	1	0	1	0	0	0	0	0	0	0	1	0	0	0	1	0	0	0	0	0	0	1
ENSG00000179348	7	49	7	71	1745	1824	1346	1940	4	3	5	0	245	218	252	349	0	21	5	36	3196	1576	2481	1353	1	2	1	0	138	226	163	294
ENSG00000244300	0	1	2	2	2	7	1	5	1	3	1	1	188	250	179	291	0	0	0	1	4	4	2	1	0	2	0	2	175	247	133	272
ENSG00000239405	0	0	0	1	0	0	0	0	0	0	0	0	0	2	0	1	0	0	0	0	0	0	0	0	0	0	0	1	1	0	0	0
ENSG00000198685	0	2	0	0	0	0	0	0	0	0	0	0	1	0	1	0	0	0	0	0	4	0	0	2	0	1	1	0	0	0	0	1
ENSG00000163902	5784	4033	4762	3737	5518	5815	3862	6017	2664	2202	8005	1827	3541	3241	4040	4327	4789	7529	6887	4910	11965	4357	8724	3867	2901	4362	1803	6530	4234	4121	5106	4881
ENSG00000277250	1	1	5	2	1	2	1	0	0	0	2	1	1	0	0	0	2	2	1	11	2	1	0	0	2	0	2	2	0	1	0	0
ENSG00000242551	16	9	14	17	6	1	1	8	0	4	10	1	5	5	3	5	8	4	12	16	4	1	7	1	0	3	2	3	3	0	3	2
ENSG00000207130	0	0	0	0	0	0	0	0	0	0	1	0	0	0	0	0	0	0	0	0	0	0	0	0	0	0	0	0	0	0	0	0
ENSG00000075785	4745	3245	3963	3305	4757	4559	3567	4875	3537	2730	13295	2337	4369	3993	3984	4941	2783	3153	2715	3258	8699	3423	5712	2894	3387	5583	3237	9352	3524	3870	3625	4445
ENSG00000239503	14	9	8	13	9	10	11	13	12	10	29	4	11	11	5	5	2	4	3	17	28	9	12	12	11	15	13	57	6	12	16	7
ENSG00000242992	10	5	4	4	2	2	2	1	7	1	14	3	6	3	1	2	0	3	0	4	10	6	3	3	6	11	6	23	2	2	8	11
ENSG00000244232	3	2	4	0	1	1	0	2	0	2	5	2	2	0	1	3	0	1	1	6	9	4	3	0	1	8	6	6	1	2	2	3
ENSG00000239483	9	6	4	3	3	5	8	6	7	5	19	0	0	4	6	5	2	1	3	6	7	3	7	7	9	13	4	18	1	4	2	6
ENSG00000244065	1	0	0	0	0	5	0	0	0	0	2	0	1	1	0	2	0	0	0	0	0	1	1	0	0	2	3	3	0	0	1	0
ENSG00000243022	0	0	0	1	1	0	0	1	0	0	1	0	0	0	0	0	0	0	0	0	0	0	1	3	1	0	0	0	0	1	4	1
ENSG00000242062	0	0	0	1	0	0	0	0	0	1	2	0	0	1	1	0	0	0	1	0	1	1	0	0	0	0	3	1	0	0	0	1
ENSG00000261159	39	40	57	33	35	37	20	22	19	21	95	10	18	16	22	26	25	22	45	103	63	24	42	35	23	23	21	33	15	26	21	43
ENSG00000231305	75	45	117	81	47	63	50	45	30	24	139	20	17	19	30	45	36	32	53	178	104	71	79	61	25	44	50	83	14	38	28	64
ENSG00000250796	3	4	3	1	7	5	7	6	5	1	9	0	1	5	2	7	3	4	5	8	7	2	4	4	3	1	3	7	2	4	2	5
ENSG00000177646	1366	1092	1335	1086	1194	1230	983	1254	1033	847	3259	694	880	787	919	1064	638	1013	882	1290	2018	890	1468	778	939	1497	685	2482	727	1073	909	1046
ENSG00000114656	19	12	10	15	2	0	0	5	0	1	7	6	21	21	13	16	2	12	6	4	2	0	2	4	2	8	12	27	8	4	13	10
ENSG00000187695	31	30	54	24	40	47	34	47	27	27	74	14	18	26	28	40	8	22	14	62	83	39	47	41	14	17	28	64	10	42	26	34
ENSG00000279507	7	6	12	5	18	11	12	11	6	6	14	7	5	2	2	5	4	3	1	9	19	12	12	10	2	3	8	14	0	5	7	7
ENSG00000114654	0	0	0	0	0	2	0	0	0	6	6	0	8	12	12	15	1	0	0	1	0	0	1	0	2	6	10	18	5	7	7	3
ENSG00000249505	0	0	1	0	0	0	0	1	0	0	0	0	0	0	0	0	0	0	0	0	0	0	0	0	0	0	0	0	0	0	0	0
ENSG00000169704	0	0	0	0	0	0	0	0	0	0	0	0	0	0	0	0	0	0	0	0	0	0	0	0	0	0	0	0	0	0	0	0
ENSG00000172780	269	229	286	248	89	92	59	121	75	111	517	82	113	85	111	131	56	75	91	121	117	49	58	37	76	198	116	320	77	89	101	102
ENSG00000261796	4	1	4	8	3	0	0	0	0	2	2	1	0	0	2	1	1	1	0	3	2	0	1	0	0	0	0	2	1	2	0	2
ENSG00000273174	13	9	23	3	9	8	5	11	7	5	55	4	16	8	9	26	4	2	5	9	14	5	11	8	13	8	12	20	7	16	5	8
ENSG00000240682	1020	604	749	676	721	653	533	757	584	419	1984	347	760	644	784	811	868	1009	918	672	1398	557	1128	460	609	883	470	1305	857	759	893	852
ENSG00000273437	3	5	16	4	8	3	4	6	11	7	31	3	9	5	11	14	3	3	1	3	8	4	8	6	4	9	9	16	2	8	6	15
ENSG00000169714	8232	5238	6108	5060	6031	5768	4906	6485	7500	5478	23223	4205	10063	8597	9814	11395	10283	13126	11924	9527	18218	6646	12333	6839	7057	10260	5125	15378	11382	10554	13455	13319
ENSG00000241673	0	0	0	0	0	0	0	0	0	0	0	0	0	0	0	0	0	0	0	0	0	0	0	0	0	0	0	0	0	0	0	0
ENSG00000181789	2739	2092	2418	2170	3060	3218	2328	3318	2237	1748	8318	1414	2118	2077	2182	2519	1111	1528	1355	1746	4627	1872	3556	1602	1781	3028	2082	5540	1552	1849	1821	2168
ENSG00000278658	1	0	0	0	0	3	2	0	1	0	2	0	0	0	1	0	0	0	1	0	1	0	0	1	0	0	0	0	0	0	0	1
ENSG00000183624	2157	1484	2018	1759	1498	1648	1123	1711	1832	1378	6092	1193	989	949	984	1337	1044	1416	1601	1406	2240	957	1890	824	1450	2295	1317	3792	726	849	805	936
ENSG00000184897	4695	4768	3884	4764	2366	2451	1474	1993	3380	3247	9837	2302	2289	2755	2194	3132	890	1796	1367	2244	2602	1096	1727	692	2297	4032	1765	6156	1222	1803	1186	1619
ENSG00000206417	21	10	13	15	237	282	233	294	160	134	925	125	31	21	19	36	10	4	6	9	372	165	264	184	117	164	116	428	10	21	14	17
ENSG00000248916	0	0	1	0	8	8	8	10	17	7	41	5	0	1	0	2	1	1	0	0	13	5	6	8	6	5	6	29	0	0	0	1
ENSG00000270492	0	0	0	0	0	0	0	3	2	5	19	2	0	0	4	0	0	0	0	0	1	1	0	2	0	1	1	5	0	0	0	0
ENSG00000270773	0	0	0	0	2	0	2	1	0	0	9	0	0	0	0	0	0	2	0	0	0	0	1	2	0	1	0	3	0	0	0	0
ENSG00000244932	134	88	134	89	100	119	110	129	112	84	513	55	100	97	89	115	45	24	30	103	168	79	99	80	88	140	85	234	29	83	41	85
ENSG00000251474	677	507	600	573	540	590	416	665	569	455	2376	367	505	435	473	578	205	184	177	491	985	510	523	465	401	593	541	1196	220	380	233	473
ENSG00000207088	9	15	10	9	16	12	27	18	8	14	44	8	18	15	12	10	8	1	1	17	32	23	24	25	8	8	11	25	4	8	9	8
ENSG00000172771	150	129	157	150	28	37	21	32	34	26	91	15	31	59	30	70	43	27	27	102	33	19	24	10	14	32	14	49	13	32	16	32
ENSG00000129071	1605	1206	1388	1113	1102	1130	888	1256	1041	863	3370	647	877	814	924	1080	1629	1796	1756	1512	2697	959	1791	1114	1011	1426	715	2349	958	1023	1157	1305
ENSG00000163913	1824	1365	1647	1548	982	1057	767	1097	620	626	1764	408	943	798	874	1069	491	624	571	1250	1350	644	1001	554	480	767	231	1300	409	755	557	908
ENSG00000163914	1	0	0	1	0	0	0	0	0	0	2	1	0	0	0	0	1	0	0	1	1	0	0	0	0	0	0	0	0	0	0	0
ENSG00000178804	0	0	0	0	2	3	0	2	0	0	0	0	0	0	0	0	0	0	0	0	2	0	0	1	0	0	0	0	0	0	0	0
ENSG00000004399	386	545	460	654	3696	3842	2695	4065	33	64	38	25	383	639	362	784	79	207	154	571	4457	1957	3016	1693	9	53	4	50	115	375	117	375
ENSG00000239437	0	0	1	0	2	4	2	4	0	0	0	0	0	2	0	0	0	0	0	1	4	3	1	2	0	0	0	0	0	0	0	0
ENSG00000248659	0	0	0	0	1	4	0	1	0	0	1	0	0	3	0	0	0	0	0	0	0	0	2	0	0	0	0	1	0	0	0	0
ENSG00000172765	582	417	487	393	296	314	251	357	397	308	1367	232	312	303	256	380	215	288	230	343	473	237	407	212	348	495	345	1004	194	251	191	264
ENSG00000202412	2	5	0	1	1	0	2	0	1	3	11	1	2	0	0	1	1	0	0	1	1	0	2	5	1	1	3	7	1	1	0	0
ENSG00000283509	4	2	1	0	2	2	0	1	1	1	5	1	2	1	1	0	1	0	0	4	3	0	2	0	2	7	3	18	0	0	0	1
ENSG00000203644	163	113	117	101	69	74	70	102	115	56	300	63	73	63	70	82	111	170	146	153	182	72	122	65	74	129	56	204	61	72	60	78
ENSG00000271270	160	96	139	103	196	179	134	240	304	219	932	142	62	50	58	123	77	111	96	162	261	116	203	95	205	250	186	463	23	48	27	36
ENSG00000250643	0	0	0	0	42	47	38	42	0	0	2	0	0	0	0	1	0	0	0	0	75	28	48	25	0	0	1	0	0	4	1	1
ENSG00000170893	0	0	0	0	19	20	18	21	0	0	0	1	0	0	1	0	0	0	0	0	33	14	19	30	0	0	0	0	0	4	1	2
ENSG00000248459	0	0	0	0	15	3	7	20	0	1	1	0	0	0	0	4	0	0	0	0	17	6	11	6	0	0	0	0	0	3	0	1
ENSG00000249869	0	0	0	0	0	0	0	0	0	0	0	0	0	0	0	0	0	0	0	0	0	0	0	0	0	0	0	0	0	0	0	0
ENSG00000180770	0	0	0	0	0	0	0	0	0	0	0	0	0	0	0	0	0	0	0	0	0	0	0	0	0	0	0	0	0	0	0	0
ENSG00000251178	0	0	0	0	0	0	0	0	0	0	0	0	0	0	0	0	0	0	0	0	1	1	2	1	0	0	2	2	0	0	0	0
ENSG00000251610	0	0	0	0	0	0	0	0	0	0	0	0	0	0	0	1	0	0	0	0	0	1	1	0	0	0	0	0	0	1	1	0
ENSG00000251287	8	11	9	8	124	156	105	149	12	9	31	7	36	47	32	47	6	5	9	17	165	82	104	78	12	8	7	17	22	38	42	41
ENSG00000248243	0	5	0	1	41	13	17	29	0	0	5	0	42	26	24	49	0	1	0	1	36	11	35	20	1	4	2	7	17	14	24	17
ENSG00000253540	46	40	61	50	137	162	100	123	42	44	130	26	127	113	119	159	43	28	17	128	162	89	105	85	21	44	18	58	36	93	70	122
ENSG00000249846	10	8	3	4	43	51	48	67	27	32	107	21	8	26	11	23	4	4	7	6	107	35	51	46	6	10	21	29	7	25	16	27
ENSG00000248618	0	0	0	0	1	0	0	1	0	0	0	0	0	0	0	0	2	0	0	0	0	0	0	2	0	0	0	0	0	0	0	0
ENSG00000250580	0	0	0	0	0	0	0	0	0	0	0	0	0	0	0	0	0	0	0	0	0	0	0	0	0	0	0	0	0	0	0	0
ENSG00000228252	0	3	2	0	37	45	21	29	3	0	6	1	14	7	10	18	0	0	0	3	20	9	8	11	0	0	5	2	1	10	2	4
ENSG00000249253	0	0	0	0	0	0	0	0	0	0	0	0	0	0	0	0	0	0	0	0	0	0	0	0	0	0	0	0	0	0	0	0
ENSG00000172752	0	0	0	0	0	0	0	0	0	0	0	0	0	0	0	0	0	0	0	0	0	0	0	1	0	0	0	0	0	0	2	0
ENSG00000206384	14	7	13	12	4	4	4	8	1	9	14	2	7	11	1	2	7	2	3	9	19	5	4	6	7	7	10	13	1	7	3	7
ENSG00000196455	1841	1309	1498	1259	999	1031	724	1137	912	702	3538	615	895	720	781	872	630	877	828	1082	1779	662	1226	604	895	1441	856	2441	715	754	599	854
ENSG00000222783	0	0	0	0	0	0	0	0	0	0	0	0	0	0	0	0	0	0	0	0	0	0	0	0	0	0	0	0	0	0	0	0
ENSG00000232829	0	0	0	0	1	0	0	0	0	0	0	0	1	0	0	0	0	0	0	0	0	0	0	0	0	0	0	0	0	0	0	0
ENSG00000017260	4252	3403	3609	3316	3516	3813	3076	4385	1902	1643	7503	1239	3751	3758	4048	4838	1786	2477	2304	3405	9485	4234	7739	3978	1909	2831	1670	4731	3255	4101	4409	4882
ENSG00000280127	2	4	2	4	20	18	9	8	1	0	1	0	10	8	11	8	0	0	0	3	17	18	17	16	0	0	0	1	1	4	7	7
ENSG00000250592	4	4	3	1	0	0	0	5	0	0	0	0	0	1	0	3	1	0	0	0	0	3	6	4	8	4	1	10	0	3	1	0
ENSG00000252894	2	2	2	0	0	0	0	0	0	0	0	3	1	1	2	0	0	0	1	1	3	0	1	2	1	1	0	1	0	2	2	4
ENSG00000249098	5	3	6	0	3	1	1	4	0	1	9	1	1	3	3	3	0	1	3	6	9	2	3	3	12	8	2	12	0	7	6	4
ENSG00000251579	2	3	1	4	0	2	0	4	0	3	0	3	3	1	4	0	0	0	3	0	4	0	1	2	2	0	0	11	0	1	1	5
ENSG00000034533	375	231	318	258	200	196	202	245	196	164	713	154	176	129	187	203	347	440	456	379	545	186	407	224	257	272	186	589	149	199	172	186
ENSG00000114670	73	48	81	50	7	14	5	11	14	6	19	3	31	32	23	48	81	77	118	76	23	22	13	9	8	12	8	19	25	40	32	28
ENSG00000250129	3	2	1	0	2	4	0	0	0	0	0	0	1	4	0	1	0	0	0	4	2	0	2	1	0	1	0	2	0	1	0	0
ENSG00000201550	0	0	0	0	0	0	0	0	0	0	0	0	0	0	1	0	0	0	0	0	0	0	0	0	0	0	0	0	0	0	0	0
ENSG00000250608	4	6	2	2	7	3	4	5	5	7	62	7	10	4	12	16	1	3	4	2	4	3	2	2	6	4	16	32	5	12	3	3
ENSG00000246082	0	2	0	4	2	2	3	1	0	5	29	0	3	6	6	5	0	3	0	2	1	2	2	0	0	13	6	8	0	3	1	1
ENSG00000198585	1	1	2	3	3	13	6	3	745	563	3301	535	857	881	871	1065	0	1	0	3	4	9	7	2	601	931	727	1943	535	799	699	932
ENSG00000261167	72	53	58	42	131	104	68	113	130	78	376	79	109	115	94	129	18	56	47	72	206	82	162	110	110	158	80	287	62	102	63	117
ENSG00000114686	3728	2219	2537	2254	2548	2572	1982	2747	2546	1855	6986	1441	3694	3554	3919	4412	5304	7683	5827	5100	7744	2883	5265	3052	2783	3736	1322	4691	4798	4577	5686	5623
ENSG00000249020	4	2	5	2	3	7	3	5	2	1	11	3	2	6	8	15	2	2	0	8	13	4	4	6	1	4	0	3	0	3	4	8
ENSG00000248468	6	5	6	5	0	0	1	2	4	0	3	2	2	2	2	0	16	13	18	16	2	3	3	7	4	0	1	2	0	1	2	6
ENSG00000248530	0	0	0	0	0	0	0	0	0	0	1	0	0	0	2	0	0	0	0	0	0	1	0	0	0	0	0	1	0	0	1	0
ENSG00000196353	1	0	0	0	1	1	1	1	1	3	5	0	3	4	0	2	2	4	0	3	1	5	0	2	2	0	1	3	1	0	0	1
ENSG00000251447	0	0	0	0	1	0	0	0	0	0	0	0	0	0	0	0	0	0	0	1	0	0	0	0	0	0	0	0	0	0	0	0
ENSG00000265859	0	0	0	0	0	0	0	0	0	0	0	0	0	0	0	0	0	0	0	0	0	0	0	0	0	0	0	0	0	0	0	0
ENSG00000240854	0	0	0	0	1	0	0	0	0	0	2	0	1	0	1	0	0	0	0	0	2	0	1	0	0	0	0	0	0	0	0	0
ENSG00000242899	0	0	0	1	0	0	1	0	0	0	0	0	1	0	0	0	0	0	0	0	0	0	0	0	0	0	0	0	0	0	1	0
ENSG00000014257	0	1	1	0	2	3	1	1	0	0	0	1	145	103	124	144	0	0	0	0	5	2	1	0	5	0	0	1	68	83	78	117
ENSG00000240890	0	0	0	0	0	0	0	0	0	0	2	0	2	0	0	0	0	0	0	0	0	0	0	0	0	2	0	1	0	0	0	0
ENSG00000213264	0	0	0	0	0	0	0	0	0	1	0	0	0	0	0	0	0	0	0	0	0	0	0	0	0	0	0	1	0	0	0	1
ENSG00000138246	2816	2113	2048	1977	1483	1633	1234	1865	1560	1257	5426	931	1471	1404	1321	1701	1120	1592	1159	2054	2842	1251	2094	1088	1591	2163	1209	3792	953	1378	976	1500
ENSG00000240303	30	22	41	30	43	48	27	36	27	36	159	35	36	45	38	49	14	7	11	27	30	33	41	26	25	29	37	86	10	33	18	27
ENSG00000274810	21	24	26	19	15	19	14	23	13	13	50	4	10	8	9	4	5	3	6	16	20	4	11	11	5	8	6	12	1	5	2	6
ENSG00000129048	8	14	16	8	9	9	7	14	13	13	40	1	4	17	7	11	4	0	2	8	25	10	11	8	14	7	10	32	0	6	10	8
ENSG00000251614	0	0	6	0	1	3	6	2	3	0	10	2	5	7	2	3	0	0	0	4	4	3	4	2	5	9	0	12	2	7	6	12
ENSG00000081307	1789	1459	1362	1352	1526	1581	1246	1886	1287	1051	4264	764	1324	1217	1400	1532	1043	1510	1161	1570	2820	1173	2054	1199	1087	1702	979	2784	1089	1304	1295	1570
ENSG00000113971	242	225	219	245	506	641	419	652	305	249	1214	188	85	117	109	123	60	41	59	158	596	348	379	303	176	228	422	600	24	76	57	130
ENSG00000248724	2	1	0	0	8	5	5	5	0	1	10	0	0	1	0	1	0	0	0	0	6	2	2	2	1	3	1	7	0	1	6	5
ENSG00000249725	0	0	0	0	0	0	0	0	0	0	0	0	0	0	0	0	0	0	0	0	0	0	0	0	0	0	0	0	0	0	0	0
ENSG00000144868	1	0	1	0	0	0	0	0	43	48	186	34	0	0	0	0	0	0	0	0	0	0	0	0	23	25	52	114	0	0	0	0
ENSG00000251011	0	0	0	0	0	0	0	0	0	0	1	0	0	0	0	0	0	0	0	0	0	0	0	0	0	0	0	0	0	0	0	0
ENSG00000170819	4	6	6	3	0	0	1	0	7	6	18	4	0	0	2	0	32	11	29	9	2	0	0	0	2	3	1	6	0	0	2	0
ENSG00000249820	0	0	0	0	0	0	0	0	2	1	11	3	0	0	0	0	0	0	0	0	0	0	0	0	0	4	0	2	0	0	0	0
ENSG00000249993	0	0	0	0	0	0	0	0	0	3	10	1	0	0	0	0	0	0	1	0	0	0	0	0	0	0	3	0	0	0	1	0
ENSG00000214301	0	0	0	0	0	0	0	0	0	0	0	0	0	0	0	0	0	0	0	0	0	0	0	0	0	0	0	0	0	0	0	0
ENSG00000272832	0	0	0	0	0	0	0	0	0	0	1	0	0	0	0	2	0	0	0	0	1	0	0	1	0	0	0	0	0	0	0	0
ENSG00000250983	0	0	0	0	1	0	0	0	2	1	1	0	0	0	0	0	0	0	0	0	0	0	0	1	0	0	3	0	0	0	1	1
ENSG00000201827	0	0	1	0	0	0	0	0	0	0	1	0	2	0	0	0	0	0	0	1	0	0	0	0	0	0	0	0	0	0	0	0
ENSG00000091527	6045	4469	5213	4390	5607	5550	4008	5781	4158	3295	14107	2509	6850	6077	7018	7294	2100	3207	3293	4468	9723	3846	7119	3657	4087	6291	3112	9017	5488	6467	5855	6436
ENSG00000163781	4678	3290	3944	3072	4370	4536	3384	5004	3980	3202	11104	2431	3386	3122	3420	3913	3281	3862	3835	4202	10238	3888	6765	3968	3691	5424	1937	8953	2994	3603	3678	4232
ENSG00000242337	9	5	3	14	10	5	8	6	12	22	77	9	2	9	4	14	3	4	5	5	9	3	4	7	4	9	10	36	4	4	4	8
ENSG00000252641	2	2	7	6	1	0	1	3	2	2	23	2	5	1	1	0	0	0	0	5	0	0	0	1	3	3	2	6	0	1	0	2
ENSG00000091513	6	6	11	9	8	11	5	15	16	20	57	8	8	10	4	9	1	8	2	4	3	3	3	1	5	10	31	20	3	4	2	2
ENSG00000244062	0	0	0	1	2	0	1	0	0	0	2	0	0	0	0	0	0	0	0	0	0	0	0	0	0	0	0	0	0	0	0	0
ENSG00000144867	1438	936	1124	1040	1213	1301	1055	1391	1110	883	3932	775	1320	1068	1514	1579	1959	2990	2362	1912	3756	1454	2925	1298	1271	1865	792	2645	1870	1934	2320	2053
ENSG00000154917	73	149	89	147	56	28	25	55	33	44	122	41	31	35	29	54	37	96	35	150	86	40	55	21	26	52	25	98	16	19	14	29
ENSG00000221972	0	0	0	0	0	0	0	0	0	0	0	0	0	0	0	0	0	0	0	0	0	0	0	0	0	0	0	0	0	0	0	0
ENSG00000174640	0	0	0	0	1	0	0	4	7	15	7	2	0	1	1	0	0	0	0	0	4	2	4	0	4	10	0	16	0	2	3	8
ENSG00000243832	0	0	0	0	0	0	0	0	0	0	0	0	3	1	0	1	0	0	0	0	0	0	0	0	0	0	0	0	0	2	0	0
ENSG00000163785	1203	986	986	926	1227	1372	896	1423	280	310	1303	221	563	521	671	802	622	1046	782	1003	2286	915	1458	840	315	522	358	930	585	743	674	761
ENSG00000231845	9	5	1	4	2	4	2	5	1	1	8	0	3	2	2	5	0	1	0	4	10	7	3	3	2	6	4	1	4	1	3	6
ENSG00000240006	0	0	0	0	0	0	0	0	0	0	0	0	0	0	0	0	0	0	0	0	0	0	0	0	0	0	0	0	0	0	0	0
ENSG00000260633	0	0	0	0	0	5	2	1	0	0	0	0	0	0	0	0	0	0	0	0	2	0	3	2	0	0	0	0	0	0	0	0
ENSG00000214289	0	0	0	0	0	0	1	0	0	0	0	0	0	0	0	0	0	0	0	0	2	0	0	0	0	0	0	0	0	0	0	0
ENSG00000114019	10	18	20	8	119	125	62	133	0	0	1	3	0	1	1	0	6	7	8	20	142	49	95	45	0	1	0	1	0	0	0	0
ENSG00000277723	0	0	0	0	0	0	0	0	0	0	0	0	0	0	0	0	0	0	0	0	0	0	0	0	0	0	0	0	0	0	0	0
ENSG00000248377	0	0	0	0	0	0	0	0	0	0	0	0	0	0	0	0	0	0	0	0	0	0	0	0	0	0	0	0	0	0	0	0
ENSG00000214288	0	0	0	0	0	0	0	0	0	0	0	0	0	0	0	0	0	0	0	0	0	0	0	0	0	0	0	0	0	0	0	0
ENSG00000129055	743	480	600	528	672	758	548	742	563	400	1968	335	775	775	839	945	876	1128	1116	1007	1903	765	1340	914	547	778	353	1127	940	959	1157	1164
ENSG00000182923	2029	1512	1614	1650	1341	1468	1237	1660	808	597	3015	473	678	616	626	767	1316	1760	1569	1636	3064	1227	2267	1283	705	1096	793	2022	536	731	668	822
ENSG00000249691	7	3	5	2	5	1	2	1	0	1	3	1	1	2	0	0	0	0	3	7	3	5	1	3	1	2	2	6	1	2	1	2
ENSG00000154928	12	22	9	14	130	140	102	179	133	187	511	152	29	37	11	65	8	5	2	13	215	80	134	78	198	395	172	969	12	55	18	66
ENSG00000174611	12	6	9	11	2	4	3	5	1	3	18	2	4	3	1	2	3	3	1	22	9	6	2	5	3	4	4	7	1	0	1	2
ENSG00000253087	1	0	0	0	0	0	0	0	0	0	0	0	0	1	0	0	0	0	0	0	0	0	0	0	0	0	0	0	0	0	0	0
ENSG00000240086	0	0	0	0	0	0	0	0	0	0	0	0	0	0	0	0	0	0	0	0	0	0	0	0	0	0	0	0	0	0	0	0
ENSG00000242222	0	0	0	0	0	0	0	0	0	0	0	0	0	0	0	0	0	0	0	0	0	0	0	0	0	0	0	0	0	0	0	1
ENSG00000241905	0	0	0	0	0	0	0	0	0	0	0	0	0	1	0	0	0	0	0	0	0	0	0	1	0	0	1	0	0	1	0	1
ENSG00000073711	51	25	35	31	23	23	28	34	70	63	262	56	11	21	22	30	8	33	5	43	41	26	39	28	65	94	68	206	6	37	20	37
ENSG00000227267	0	2	0	0	0	0	0	0	1	0	0	0	1	0	1	0	0	0	0	0	0	1	0	0	0	0	0	2	0	0	1	0
ENSG00000273455	0	0	0	0	0	1	0	2	0	0	8	1	0	1	3	3	0	0	0	0	1	4	7	0	0	0	4	1	1	3	3	4
ENSG00000174579	4931	3282	4393	3316	1963	1805	1546	1965	1722	1545	6311	1167	1816	1653	1790	2094	1721	2077	2258	3168	3421	1337	2669	1189	1775	2636	1397	4435	1153	1578	1251	1617
ENSG00000241438	8	3	7	1	0	2	1	3	4	4	15	2	5	1	0	8	2	0	0	6	7	7	2	4	8	15	4	11	1	8	3	7
ENSG00000244740	0	0	0	0	0	0	0	0	0	0	0	0	0	0	0	0	0	0	0	0	0	0	0	0	0	0	0	0	0	0	0	0
ENSG00000114054	3024	2260	2284	2409	2341	2293	1733	2482	1058	744	3105	600	1436	1178	1415	1660	1673	2643	2125	2212	4093	1654	3202	1347	1033	1587	634	2328	1228	1309	1384	1476
ENSG00000118007	1825	1496	1600	1547	1746	1908	1395	2039	1916	1678	5639	1274	738	831	870	992	1459	2417	2050	2499	4616	1872	3471	1788	1875	2990	1226	4782	828	1129	1091	1302
ENSG00000200571	0	1	0	0	1	1	1	0	2	2	5	0	0	0	0	0	0	2	0	2	1	3	0	3	1	2	0	5	0	0	0	0
ENSG00000244101	0	0	3	0	0	1	1	2	1	0	0	0	0	0	1	0	0	0	0	0	2	1	0	0	0	0	0	8	0	1	0	0
ENSG00000252914	3	3	0	2	2	3	1	1	2	0	5	1	0	2	0	1	0	0	1	5	3	1	5	3	5	4	2	13	0	9	0	3
ENSG00000240695	16	9	22	8	19	19	25	25	17	13	46	9	2	12	5	9	12	8	4	25	38	19	15	19	16	19	14	77	5	24	11	14
ENSG00000261758	6	6	6	3	9	4	7	9	5	6	24	4	8	11	7	13	5	3	4	14	27	8	5	14	2	8	10	16	5	12	10	12
ENSG00000242968	1	1	2	3	1	0	0	0	0	0	6	1	0	2	1	1	2	10	9	3	0	0	1	1	1	4	4	9	0	0	1	0
ENSG00000168917	153	119	159	88	245	278	211	340	44	34	174	31	134	135	176	204	86	134	148	186	592	267	424	250	33	36	40	102	133	161	216	236
ENSG00000273486	70	61	85	68	47	43	51	68	81	55	218	40	54	52	46	53	18	25	28	67	92	58	48	53	32	57	48	102	16	35	16	46
ENSG00000239213	204	124	190	132	79	90	63	90	95	76	254	53	144	98	145	174	110	129	154	160	126	68	103	61	63	105	52	186	90	83	105	156
ENSG00000158092	1019	677	851	648	1032	1034	841	1103	599	450	1864	340	652	617	748	804	1085	1185	1202	1006	2528	955	1736	1083	636	817	480	1264	652	712	839	824
ENSG00000214283	9	5	7	4	1	6	5	4	3	4	9	0	0	5	5	4	1	0	1	5	6	2	0	4	3	7	2	20	1	2	0	5
ENSG00000174564	32	14	24	18	29	29	18	29	16	9	61	8	10	6	10	13	17	23	35	43	58	20	27	13	10	13	16	37	9	16	16	13
ENSG00000249407	2	2	1	1	1	1	3	2	0	0	4	1	2	1	10	1	2	0	1	6	6	2	2	0	6	0	3	5	0	1	2	0
ENSG00000243886	0	0	0	1	0	0	0	0	0	0	0	0	9	13	8	7	0	0	0	0	0	0	0	0	0	0	0	0	3	5	4	11
ENSG00000242070	0	0	0	0	0	0	0	0	0	0	0	0	0	0	0	0	0	0	0	0	0	0	0	0	0	0	0	0	0	0	0	0
ENSG00000168875	0	1	0	0	0	0	0	0	0	1	0	0	0	0	0	0	0	0	0	0	0	0	2	0	0	0	0	0	0	0	0	0
ENSG00000239513	0	0	0	0	0	0	0	0	0	0	0	0	0	0	0	0	0	0	0	0	0	1	0	0	0	0	0	0	0	0	0	0
ENSG00000261146	0	0	0	0	0	0	0	0	0	0	0	0	0	0	0	0	0	0	0	0	0	0	0	0	0	0	0	0	0	0	0	0
ENSG00000241478	1	0	0	0	1	0	0	2	1	0	0	0	0	0	0	0	1	0	0	0	1	0	0	0	0	0	0	0	1	0	0	0
ENSG00000066405	0	0	1	1	66	85	68	93	0	0	0	0	0	0	0	0	0	0	0	1	96	60	39	45	0	0	0	0	0	0	0	3
ENSG00000272609	0	0	0	0	5	5	3	2	0	0	0	0	0	0	0	0	0	0	0	1	5	1	2	2	0	0	0	0	0	0	0	0
ENSG00000158163	2	8	5	6	480	516	345	579	15	7	20	4	3	11	12	25	2	0	4	3	716	364	427	318	4	9	6	33	3	23	22	34
ENSG00000240668	0	1	1	0	6	6	4	10	0	0	0	0	0	0	1	0	0	0	0	0	5	10	4	3	0	0	1	0	0	0	0	1
ENSG00000118017	0	2	0	0	3	1	0	1	0	0	1	0	0	0	0	0	0	0	0	1	1	0	0	0	0	0	1	0	1	0	0	0

ENSG00000138231	828	595	614	708	787	750	596	823	698	541	2118	413	828	826	936	1055	604	845	743	826	1835	690	1310	627	583	1045	422	1490	987	1148	1113	1389
ENSG00000114098	1444	1035	1146	966	1903	1812	1445	2021	857	709	3166	536	1117	1010	1201	1459	1210	1533	1389	1348	5003	1957	3496	2055	962	1382	849	2208	1203	1369	1523	1698
ENSG00000181322	101	80	67	75	112	129	84	144	66	45	216	20	86	65	63	76	21	19	15	91	168	90	89	93	43	81	66	148	22	76	56	109
ENSG00000158186	0	1	1	0	3	4	2	10	2	5	48	6	0	0	0	0	0	1	0	0	1	2	1	1	3	9	46	60	0	0	0	0
ENSG00000158220	563	510	516	539	56	81	64	64	105	101	241	54	145	140	104	165	223	286	185	683	89	38	52	31	47	121	43	232	45	131	55	164
ENSG00000280399	121	89	107	111	30	45	22	31	28	22	54	9	14	26	17	30	25	27	44	110	51	24	26	27	22	23	5	54	6	43	17	33
ENSG00000114107	1776	1124	1416	1179	1156	1235	950	1236	351	224	925	202	318	327	329	457	1993	2083	2081	2061	2530	928	1698	1163	365	486	152	669	263	374	414	519
ENSG00000158234	62	39	61	48	670	655	542	722	364	286	1185	228	200	263	240	335	118	120	128	146	2107	799	1404	827	432	524	261	785	458	506	422	558
ENSG00000174715	4	3	8	4	5	9	4	9	4	0	7	2	0	2	0	5	1	0	1	3	5	1	4	4	8	0	1	13	1	2	1	4
ENSG00000051382	906	767	909	722	1125	1133	820	1243	261	261	1095	196	727	746	794	837	305	500	495	675	1715	673	1188	645	346	477	274	965	555	695	637	747
ENSG00000239207	3	2	2	2	3	3	1	3	2	1	4	0	2	0	4	4	2	2	0	4	9	3	2	2	4	3	0	2	1	5	5	1
ENSG00000242321	0	0	2	0	3	0	0	4	0	0	0	0	2	2	1	0	1	0	0	1	3	1	2	0	0	0	0	6	2	1	0	1
ENSG00000241429	1	3	4	1	2	9	4	9	1	1	9	0	1	3	3	4	0	1	0	9	17	6	3	4	1	2	4	6	1	4	3	4
ENSG00000242244	0	1	0	0	0	0	1	0	0	1	0	0	0	0	0	0	0	0	0	0	0	1	0	0	0	0	0	0	0	0	0	0
ENSG00000244578	0	1	0	5	4	4	3	8	0	0	0	0	1	1	3	2	0	2	1	3	10	4	3	4	0	0	0	0	2	5	0	5
ENSG00000183770	0	0	0	0	2	1	5	0	0	0	2	0	1	0	4	4	0	0	0	1	2	6	3	0	0	0	0	0	1	1	0	2
ENSG00000206262	1	6	0	3	19	17	19	26	0	1	5	0	4	10	6	11	0	0	0	1	16	12	5	6	0	0	0	0	1	12	5	6
ENSG00000206260	0	0	0	0	0	0	0	1	0	0	0	0	0	2	0	1	0	0	0	0	1	1	1	1	0	0	0	0	0	0	0	1
ENSG00000175110	1117	701	836	666	918	975	806	1085	491	376	1565	310	906	816	1047	1027	1366	1694	1562	1240	2536	1040	1955	1225	459	638	315	962	1272	1139	1457	1362
ENSG00000184814	0	0	0	0	0	0	0	0	0	0	0	0	0	0	0	0	0	0	0	0	1	0	0	0	0	0	0	0	0	0	0	0
ENSG00000233701	0	0	0	0	0	0	0	0	0	0	0	0	0	2	0	0	0	0	0	0	0	1	2	0	0	0	0	0	0	0	0	3
ENSG00000251137	0	0	0	0	0	0	0	0	0	0	0	0	0	0	0	0	0	0	0	0	0	0	0	0	0	0	0	0	0	0	0	0
ENSG00000232416	0	0	0	0	5	3	2	3	0	0	1	0	0	0	0	0	2	0	0	0	2	0	6	4	0	0	0	0	0	0	0	2
ENSG00000281473	0	0	0	0	0	1	1	0	0	0	0	0	0	0	0	0	0	0	0	0	0	0	0	1	0	0	1	0	0	0	0	0
ENSG00000276304	0	0	0	0	0	0	0	0	0	0	0	0	0	0	0	0	0	0	0	0	0	0	0	0	0	0	0	0	0	0	0	0
ENSG00000272656	5	9	15	7	12	10	9	6	5	3	13	2	2	5	5	8	2	1	3	9	13	15	10	7	5	2	6	4	2	9	3	5
ENSG00000184432	3704	2559	3064	2509	3466	3659	2614	3786	2332	1768	8973	1466	3546	3383	3599	4535	2346	3328	3113	2944	7869	2972	5973	2591	2428	3587	2216	6044	3579	3841	4086	4314
ENSG00000283418	5	7	7	3	5	6	2	6	1	2	16	2	11	5	5	8	1	0	0	9	6	13	1	2	1	5	6	7	3	10	1	10
ENSG00000248932	33	31	26	13	44	31	28	43	35	36	138	24	37	44	32	73	17	19	25	19	36	21	35	16	24	43	38	74	34	35	37	58
ENSG00000114113	1	3	3	1	0	1	6	4	4	6	21	5	5	11	6	6	0	0	0	3	2	3	1	4	1	1	6	10	0	9	2	3
ENSG00000248790	0	0	0	0	2	0	0	0	1	0	6	2	1	1	0	3	0	0	0	0	0	0	0	0	0	0	3	2	0	0	2	1
ENSG00000178631	1	4	4	4	12	7	4	5	10	19	50	7	14	16	7	9	1	5	2	4	7	5	3	6	8	3	15	19	5	2	13	7
ENSG00000114115	1	0	1	1	2	1	2	2	1	2	5	0	1	1	2	2	0	1	0	3	0	1	1	1	0	3	1	1	1	1	0	1
ENSG00000163864	0	1	2	0	608	602	437	611	108	68	231	53	1	2	0	1	2	1	0	1	963	340	630	297	63	82	45	132	0	0	0	4
ENSG00000214280	0	2	0	1	4	5	5	5	2	1	12	3	2	3	1	4	0	0	0	4	8	2	3	1	0	0	4	5	0	1	0	0
ENSG00000201600	0	1	0	0	0	0	1	0	0	0	0	0	0	1	0	0	0	0	0	0	1	1	1	2	0	0	0	0	0	0	0	0
ENSG00000244692	0	0	0	0	1	2	0	3	0	0	4	0	0	1	2	1	0	0	0	0	2	1	0	1	3	0	0	4	0	1	0	0
ENSG00000261763	0	0	0	0	0	0	0	0	0	0	1	0	0	0	0	0	0	0	0	0	1	0	1	0	0	0	0	0	0	0	0	0
ENSG00000250543	0	0	0	0	0	0	0	0	0	0	0	0	0	0	0	0	0	0	0	0	0	0	0	0	0	0	0	0	0	0	0	0
ENSG00000251471	0	0	0	0	0	0	0	0	0	0	0	0	0	0	0	0	0	0	0	1	0	0	0	0	0	0	0	0	0	0	0	0
ENSG00000250161	0	0	0	0	0	0	0	0	0	0	0	0	0	0	0	0	0	0	0	0	0	0	0	0	0	0	0	0	0	0	0	0
ENSG00000158258	3	4	6	4	2	0	1	2	4	4	3	3	3	6	7	2	1	0	1	0	5	4	2	0	2	2	0	2	0	3	3	3
ENSG00000249290	0	1	0	0	0	0	0	0	0	1	0	0	0	0	0	0	0	0	0	0	1	0	0	0	0	0	0	0	0	0	0	0
ENSG00000251058	0	0	0	0	0	0	0	0	0	0	0	0	0	0	0	0	0	0	0	0	0	0	0	0	0	0	0	0	0	0	0	0
ENSG00000250433	2	3	3	3	2	2	5	0	2	0	0	0	9	5	4	7	2	2	2	5	0	1	4	1	0	0	0	0	4	3	2	4
ENSG00000249305	0	0	0	0	0	0	0	0	0	0	0	0	0	0	0	0	0	0	0	0	0	0	0	0	0	0	0	0	0	0	0	0
ENSG00000155890	0	0	0	1	0	0	0	0	0	0	0	0	0	0	0	0	0	1	0	0	0	0	0	0	1	0	0	0	0	1	1	0
ENSG00000261826	1	0	0	0	1	1	0	0	0	0	0	0	0	2	0	0	0	0	0	0	0	0	0	0	0	0	0	1	0	0	1	0
ENSG00000224085	0	0	0	1	1	0	0	0	0	0	0	0	1	0	0	0	0	0	0	1	0	0	1	0	0	0	0	0	1	1	0	0
ENSG00000114120	6158	4331	4480	4284	4348	4833	3348	5189	3285	3116	11873	2166	6032	5353	5754	6867	2431	3005	2181	4270	6695	2914	4156	3018	2505	3592	3085	7058	3190	4728	4449	5789
ENSG00000248773	17	11	13	13	9	13	7	12	17	8	34	8	17	9	10	9	8	2	1	14	10	7	4	8	8	11	4	15	14	12	8	21
ENSG00000175093	294	264	255	250	280	399	234	322	1	2	3	0	23	49	49	66	110	204	197	295	478	219	389	172	0	2	0	4	22	39	36	59
ENSG00000251270	3	10	7	5	5	3	3	2	0	0	0	0	0	0	0	0	1	0	0	6	13	9	7	6	0	0	0	0	0	0	2	1
ENSG00000155893	1892	1592	1787	1493	1002	991	708	1083	602	447	978	319	548	603	461	720	807	1030	808	1145	889	391	517	332	331	489	172	656	267	537	327	534
ENSG00000241400	1	6	11	5	2	4	1	8	2	2	4	1	0	2	0	2	0	0	1	11	5	3	1	0	1	0	0	1	1	2	2	4
ENSG00000249417	21	32	21	11	9	16	7	4	4	5	10	1	10	9	11	10	5	10	1	17	4	4	0	4	4	8	2	11	1	14	2	7
ENSG00000249540	4	4	2	6	2	0	2	1	0	0	1	0	0	0	6	2	1	0	0	1	0	0	0	3	2	1	0	0	0	1	1	0
ENSG00000177311	21	13	15	16	8	8	6	5	1328	1040	5708	869	390	398	343	433	7	15	9	30	3	12	5	3	1189	1730	1718	3813	282	429	298	458
ENSG00000215606	0	1	0	0	0	0	0	0	0	0	0	1	0	1	0	0	0	0	0	0	0	0	0	0	0	1	1	0	0	0	0	0
ENSG00000155903	1373	933	1040	915	1022	1286	784	1245	1593	1432	5351	819	1404	1384	1377	1705	475	551	613	904	2148	997	1293	973	1773	2250	1416	3972	1015	1565	1245	1704
ENSG00000280530	3	0	0	0	0	0	0	0	2	0	1	0	1	2	2	3	1	0	2	0	0	1	1	0	0	3	0	1	0	0	0	1
ENSG00000250170	1	2	2	2	1	3	1	2	1	1	16	3	2	2	5	1	2	0	1	12	8	3	4	6	4	8	3	17	1	0	6	6
ENSG00000249986	6	0	2	1	2	9	4	6	7	9	33	3	2	5	5	11	0	1	3	4	3	8	6	3	8	10	12	31	3	6	4	10
ENSG00000242104	0	0	0	0	0	0	0	0	0	0	0	0	0	0	0	0	0	0	0	0	0	0	0	0	0	0	4	0	0	1	0	0
ENSG00000241899	0	0	0	0	0	0	0	0	0	0	0	0	0	0	0	0	0	0	0	0	0	0	0	0	0	0	0	0	0	0	0	0
ENSG00000241526	0	0	0	0	0	0	0	0	0	0	0	0	0	0	0	0	0	0	0	0	0	0	0	0	0	0	0	0	0	0	0	0
ENSG00000114125	1193	755	998	842	693	756	534	796	879	651	2887	479	1146	1034	1233	1457	945	1360	1369	1229	1291	561	1042	546	707	1109	620	1578	1191	1215	1426	1350
ENSG00000114124	3	6	3	1	1	1	0	4	3	1	9	0	1	3	1	1	0	3	1	6	3	0	1	2	4	2	1	3	0	1	1	1
ENSG00000228398	0	0	0	0	0	0	0	0	0	0	0	0	0	0	0	0	0	0	0	0	0	0	0	0	0	0	0	0	0	0	1	0
ENSG00000069849	3363	2374	2963	2229	1970	1988	1511	2171	1711	1402	5488	958	2892	2461	2975	3150	1756	2581	2449	2633	4466	1682	3252	1602	1983	2746	1152	3855	2760	2826	3010	3270
ENSG00000200389	5	4	7	4	0	1	3	0	1	1	5	0	1	1	1	0	3	0	1	1	3	0	1	0	0	1	1	4	2	2	0	0
ENSG00000244124	54	61	66	50	26	36	19	27	27	19	106	7	19	23	14	22	10	10	11	47	62	16	30	17	12	39	17	56	5	18	17	31
ENSG00000279147	3	3	5	1	3	9	7	4	4	7	18	1	5	7	5	1	1	1	6	7	11	15	6	4	5	3	5	3	1	3	2	4
ENSG00000114126	6153	3825	5909	3919	6767	5859	5824	6421	2101	1804	8482	1383	4923	4084	4279	4943	2931	3311	3692	3715	17382	5852	14590	5917	1663	2061	1996	4757	3015	3449	2814	3914
ENSG00000233597	11	9	7	8	6	4	5	4	7	3	9	5	12	6	7	9	6	2	1	0	10	6	6	9	1	1	6	10	0	1	4	3
ENSG00000206604	1	1	0	4	0	0	0	1	1	0	2	0	1	1	0	0	0	0	1	0	1	0	1	2	0	1	0	5	0	0	0	1
ENSG00000175066	1868	1438	1678	1438	795	861	645	867	951	803	4340	672	1406	1270	1374	1554	621	781	685	1165	1233	593	1033	480	781	1216	1119	2731	875	1237	804	1269
ENSG00000114127	2514	1811	1976	1642	2238	2372	1830	2495	1753	1534	8309	1209	2228	2095	2095	2657	638	742	768	1181	4005	1657	2881	1646	1854	2613	2228	5723	1216	1921	1569	2080
ENSG00000251804	8	2	0	0	2	3	0	0	2	1	7	1	0	0	1	3	0	0	0	2	0	0	0	0	1	1	0	4	0	1	1	0
ENSG00000202125	1	0	2	1	0	0	0	0	0	1	1	0	2	0	0	0	0	0	2	0	0	2	3	0	0	0	1	2	0	1	0	1
ENSG00000175054	2774	2042	2281	1911	1267	1339	1009	1477	1288	1148	5470	848	2936	2532	2748	3148	1091	1280	1095	2151	2698	1124	1721	1069	1593	2213	1238	3875	1652	2874	2220	3222
ENSG00000242479	0	0	0	0	0	0	0	0	0	0	0	0	0	0	0	0	0	0	0	0	0	0	0	0	0	0	0	0	0	0	0	0
ENSG00000244327	28	17	24	24	11	17	14	20	29	21	84	11	25	30	41	32	11	7	3	30	42	18	12	12	12	20	12	43	9	43	25	30
ENSG00000242390	2	1	5	6	2	1	0	0	1	0	3	0	3	2	2	3	0	1	0	1	0	0	0	0	1	0	0	0	1	1	0	1
ENSG00000120756	1510	1075	1128	1073	999	1032	700	1124	210	204	519	154	585	663	610	802	1484	2078	1664	1489	2271	790	1384	802	250	371	84	439	763	841	805	1007
ENSG00000239641	1	0	2	2	0	0	0	0	1	1	0	0	1	0	1	1	0	0	1	1	0	0	0	2	0	0	0	1	0	0	0	1
ENSG00000199319	1	0	0	1	2	1	0	0	1	0	0	0	0	0	0	2	0	0	0	0	3	1	0	1	0	0	0	0	0	0	1	0
ENSG00000144935	11	12	17	13	208	226	145	225	95	83	331	53	149	198	139	247	2	16	12	30	321	166	275	176	39	60	116	189	42	124	74	189
ENSG00000251787	0	2	0	0	1	5	0	1	0	0	3	0	1	1	0	3	0	0	0	0	2	1	1	0	0	0	1	0	0	2	0	5
ENSG00000163710	194	231	153	245	216	210	161	228	5	6	10	3	451	517	460	690	178	392	191	339	403	186	332	157	0	6	3	5	387	553	433	610
ENSG00000240950	1	3	2	1	2	1	1	0	0	0	0	0	4	6	4	7	0	0	3	4	7	6	0	0	0	0	0	0	1	6	2	4
ENSG00000243818	0	0	0	0	0	0	0	0	0	0	0	0	0	0	0	0	0	0	0	0	0	0	0	0	0	0	0	0	0	1	0	2
ENSG00000188582	1	7	7	8	0	0	0	0	1	0	3	1	2	7	5	6	2	4	1	5	0	0	0	0	0	0	0	0	2	7	2	6
ENSG00000241592	2	0	0	2	0	0	0	0	0	0	0	0	0	0	1	0	0	0	0	0	0	0	0	0	0	0	0	0	0	0	0	0
ENSG00000241570	1	1	1	2	0	0	0	0	0	0	1	1	3	6	0	3	0	4	0	3	0	0	0	0	0	2	0	0	0	2	1	1
ENSG00000163714	11587	7407	9219	7506	6527	6791	5346	7215	5856	4556	18189	3283	8841	7572	9511	9438	7829	8573	7994	10064	15295	6064	10056	6270	5634	7842	3723	12113	6988	8687	8684	9900
ENSG00000268129	8	4	4	4	1	7	0	4	1	0	5	4	8	10	6	7	4	12	3	6	3	0	3	0	3	1	3	4	12	3	10	4
ENSG00000243733	0	0	0	0	0	0	0	0	0	0	1	0	0	0	0	0	0	0	0	0	0	0	0	0	0	0	0	0	0	0	0	0
ENSG00000175040	1786	1786	1639	1925	216	291	189	299	1482	1206	8356	1230	107	127	162	200	432	873	648	1313	342	184	255	159	1642	2646	1774	4151	142	228	110	205
ENSG00000241679	5	6	9	19	0	1	1	2	15	15	104	12	1	0	0	2	1	1	4	24	1	0	0	0	9	17	20	47	0	0	0	0
ENSG00000244171	5	3	4	2	1	5	3	5	3	2	7	2	2	1	1	4	2	1	0	2	2	2	2	0	0	3	1	4	1	0	1	2
ENSG00000181804	38	23	32	21	45	64	50	73	42	50	175	31	36	65	56	57	29	69	38	43	88	36	75	25	27	49	48	88	32	48	36	47
ENSG00000240888	1	1	0	0	1	0	0	0	1	0	0	0	0	0	0	1	0	0	0	1	0	0	0	1	2	1	0	4	0	0	1	0
ENSG00000240012	0	0	0	0	1	0	0	1	0	1	3	1	0	0	0	0	0	1	0	0	0	0	0	0	1	0	0	4	0	0	0	0
ENSG00000244493	0	0	0	0	0	0	0	0	0	1	9	0	0	0	0	0	0	0	0	0	1	0	0	0	1	2	6	1	0	0	0	2
ENSG00000243033	0	0	1	0	0	1	0	1	0	0	2	0	0	0	0	0	0	0	1	0	0	1	0	1	1	0	0	0	0	0	0	0
ENSG00000243759	1	0	1	1	1	3	1	1	0	1	0	0	0	1	0	1	1	0	0	1	1	2	0	0	0	0	1	1	1	2	1	0
ENSG00000248374	0	0	0	0	1	0	1	0	0	0	3	0	0	0	0	0	1	0	0	0	0	0	0	0	0	3	0	1	1	0	1	0
ENSG00000235110	0	0	0	0	0	0	0	0	0	0	0	0	0	0	0	0	0	0	0	0	0	0	0	0	0	0	0	0	0	0	0	0
ENSG00000181744	969	785	711	738	1310	1382	990	1418	864	658	2780	518	687	702	681	959	346	594	518	682	2021	784	1444	741	756	1097	666	1820	503	716	534	718
ENSG00000280397	0	3	0	0	3	4	0	1	0	0	3	0	3	0	0	1	0	1	0	1	4	0	0	2	2	0	2	2	1	0	2	1
ENSG00000244024	0	0	0	0	0	0	0	0	0	0	0	0	0	0	0	0	0	0	0	0	0	0	0	0	0	0	0	0	0	0	0	0
ENSG00000241695	0	0	0	0	0	0	0	0	0	0	0	0	0	0	0	0	0	0	0	0	0	0	0	0	0	0	0	0	0	0	0	0
ENSG00000239797	0	0	0	2	0	0	0	0	0	0	0	1	0	0	0	0	1	0	0	0	0	0	0	0	0	0	0	0	0	0	1	0
ENSG00000244358	0	0	0	0	0	0	0	0	0	0	0	0	0	0	0	0	0	0	0	0	0	0	0	0	0	0	0	0	0	0	0	0
ENSG00000240095	0	0	0	0	0	0	0	0	0	0	0	0	0	0	0	0	0	0	0	0	0	0	0	0	0	0	0	0	0	0	0	0
ENSG00000261051	0	0	0	0	0	0	0	0	4	4	7	3	0	0	0	0	0	0	0	0	0	0	0	0	0	3	5	8	0	0	0	0
ENSG00000243415	0	0	0	0	0	0	0	0	3	2	1	0	0	0	0	0	0	0	0	0	0	0	0	0	6	5	0	1	0	0	0	0
ENSG00000240032	0	0	0	0	0	0	0	0	1	0	3	2	0	0	0	0	0	0	0	0	0	0	0	0	0	0	0	6	0	0	0	0
ENSG00000152952	0	0	0	0	0	0	1	0	645	540	1513	386	0	1	0	0	0	0	0	0	0	0	0	1	558	819	273	1268	0	1	0	0
ENSG00000114698	0	0	0	0	0	0	0	0	116	106	430	85	0	0	0	0	0	0	0	0	0	0	0	0	115	118	116	346	0	0	0	0
ENSG00000243596	0	0	0	0	0	0	0	0	0	0	0	0	0	0	0	0	0	0	0	0	0	0	0	0	0	0	0	0	0	0	0	0
ENSG00000241358	0	0	0	0	0	0	0	0	0	0	0	0	0	0	0	0	0	0	0	0	0	0	0	0	0	0	0	0	0	0	0	0
ENSG00000163746	1	0	0	2	0	4	0	2	0	1	8	0	0	0	0	3	1	0	0	0	1	0	0	3	1	0	4	2	0	0	0	1
ENSG00000279320	4	8	5	4	26	19	14	21	16	13	43	6	8	10	2	6	1	3	3	6	25	11	17	6	13	12	22	31	6	11	5	8
ENSG00000188313	618	577	554	513	822	969	732	1034	672	545	2540	466	964	1062	1082	1375	766	1135	875	926	2373	1031	1752	1120	619	943	565	1699	1092	1360	1274	1724
ENSG00000199812	0	0	0	0	0	0	0	0	0	0	0	0	0	0	0	0	0	0	0	0	0	0	0	0	0	0	0	0	0	0	0	0
ENSG00000231213	0	0	0	0	1	1	0	1	0	0	0	0	0	0	0	0	0	0	0	0	5	5	2	1	0	0	0	0	0	0	0	0
ENSG00000241457	0	0	0	0	0	0	0	0	0	0	0	0	0	0	0	0	0	0	0	0	0	0	0	0	0	0	0	0	0	0	0	0
ENSG00000243620	0	0	0	0	0	0	0	0	0	1	2	1	0	0	0	0	0	0	0	0	0	0	0	0	0	0	0	0	0	0	0	0
ENSG00000242671	0	0	0	0	0	0	0	0	0	0	0	0	0	0	0	0	0	0	0	0	0	0	0	0	0	0	0	0	0	0	0	0
ENSG00000207156	0	0	0	0	0	0	0	0	0	0	0	0	0	0	0	0	0	0	0	0	0	0	0	0	0	0	0	0	0	0	0	0
ENSG00000239412	0	0	0	0	0	0	0	0	0	0	2	0	0	0	0	0	0	0	0	0	0	0	0	0	0	0	0	0	0	0	0	0
ENSG00000174963	0	0	0	0	0	0	0	0	0	0	6	2	1	0	0	0	0	0	0	0	0	0	0	0	0	0	0	0	0	0	0	0
ENSG00000241202	0	0	0	0	0	0	0	0	0	0	0	0	0	0	0	0	0	0	0	0	0	0	0	0	0	0	0	0	0	0	0	0
ENSG00000152977	0	0	0	0	0	0	0	0	0	0	3	1	0	0	0	0	0	0	0	2	0	0	0	0	0	1	0	4	0	0	0	0
ENSG00000240804	0	0	0	0	0	0	0	0	0	0	0	0	0	0	0	0	0	0	0	0	0	0	0	0	0	0	0	1	0	0	0	0
ENSG00000239661	0	0	0	0	0	0	0	0	0	0	0	0	0	0	0	0	0	0	0	0	0	0	0	0	0	0	0	0	0	0	0	0
ENSG00000239922	0	0	0	0	0	0	0	0	0	0	3	0	0	0	1	0	0	0	0	0	0	1	0	0	0	0	0	1	0	0	0	0
ENSG00000241131	0	0	0	0	0	0	0	0	0	0	0	0	0	1	0	0	0	0	0	0	0	0	0	0	0	0	1	1	0	0	0	0
ENSG00000241792	0	0	0	0	0	0	0	0	0	0	0	0	0	0	0	0	0	0	0	0	0	0	0	0	0	0	0	0	0	0	0	0
ENSG00000244650	0	0	0	0	0	0	0	0	0	0	0	0	0	0	0	0	0	0	0	0	0	0	0	0	0	0	0	0	0	0	0	0
ENSG00000205412	0	0	0	0	0	0	0	0	0	0	0	0	0	1	0	0	0	0	0	0	0	0	0	0	0	0	0	0	0	0	0	0
ENSG00000243347	0	0	0	0	0	0	0	0	0	0	0	0	0	0	0	0	0	0	0	0	0	0	0	0	0	0	0	0	0	0	0	0
ENSG00000242440	0	0	0	0	0	0	0	0	0	0	1	0	0	0	0	0	0	0	0	0	0	0	0	0	0	0	2	1	0	0	0	0
ENSG00000241993	0	0	0	0	0	0	0	0	0	0	0	0	0	0	0	0	0	1	0	0	0	0	0	0	0	0	0	0	0	0	0	0
ENSG00000144891	0	0	0	0	0	0	0	0	0	0	0	0	0	0	0	0	0	0	0	0	0	0	0	0	0	0	0	0	0	0	0	0
ENSG00000153002	0	0	0	0	7	3	4	4	0	0	1	0	2	3	0	0	0	0	0	0	15	1	4	4	0	0	1	3	0	0	0	0
ENSG00000244089	0	0	0	0	0	0	0	0	0	0	0	0	0	0	0	0	0	0	0	0	0	0	0	0	0	0	0	0	0	0	0	0
ENSG00000240521	0	0	0	0	6	6	3	3	0	0	2	0	0	0	0	0	0	0	1	0	1	1	2	3	0	0	2	0	0	0	0	0
ENSG00000163751	0	2	0	0	1194	1102	989	1270	0	0	0	0	0	0	0	0	0	14	7	13	2013	879	1487	982	0	0	1	1	0	0	0	0
ENSG00000241627	1	0	1	1	4	4	3	1	0	2	9	1	0	3	0	0	1	0	1	4	8	2	2	2	0	3	0	4	0	1	3	1
ENSG00000163754	738	518	581	531	1304	1275	995	1387	1715	1183	6850	989	1450	1207	1337	1530	460	803	639	724	2817	1085	2202	1052	2000	2561	1607	4247	1423	1482	1398	1573
ENSG00000071794	7732	5115	5910	4596	7828	7666	6584	8507	3607	2872	10466	2027	4365	4059	4090	5004	4288	6046	5247	5275	16885	6437	12864	6573	3324	4394	2249	7187	3386	3886	4003	4573
ENSG00000239718	0	0	0	0	0	0	0	0	0	0	0	0	0	2	0	0	1	0	1	0	0	0	0	1	0	0	0	0	0	0	0	0
ENSG00000201031	0	0	0	0	0	0	0	0	0	0	0	0	0	0	0	0	0	0	0	0	0	0	0	0	0	0	0	0	0	0	0	0
ENSG00000240511	0	0	0	1	0	0	0	0	0	0	0	2	0	0	0	0	0	0	0	0	0	0	0	0	1	0	0	0	0	0	0	0
ENSG00000163755	1462	1264	1193	1251	1467	1540	1193	1693	1400	1254	5564	914	1011	933	996	1250	866	1196	903	976	2634	988	1856	976	1267	1815	1164	3443	638	881	783	1012
ENSG00000047457	8	15	5	4	15	10	15	10	11	7	50	11	12	9	3	8	5	0	7	4	16	5	8	11	18	10	18	60	6	9	8	17
ENSG00000240216	1	0	0	0	2	4	6	2	0	0	5	1	2	0	1	2	0	0	0	1	6	1	8	3	0	0	0	0	0	0	0	4
ENSG00000244468	0	0	0	1	0	0	0	0	0	0	0	0	0	1	0	0	0	0	0	0	0	0	0	0	0	0	0	0	0	0	0	1
ENSG00000163762	0	0	0	0	0	0	0	0	0	0	2	0	0	0	2	0	0	1	0	0	0	0	0	0	0	0	1	0	0	0	2	0
ENSG00000243822	0	0	0	0	0	0	0	0	0	0	0	0	0	0	0	0	0	0	0	0	0	0	0	0	0	0	0	0	0	0	0	0
ENSG00000169908	0	0	0	0	0	0	0	0	0	0	0	0	0	1	0	1	0	0	0	0	0	0	0	0	0	0	0	0	0	0	0	0
ENSG00000240541	0	0	0	0	0	0	0	0	0	0	0	0	0	0	0	0	0	0	0	0	0	0	0	0	0	0	0	0	0	0	0	0
ENSG00000243885	0	0	0	0	0	0	0	0	0	0	0	0	0	0	1	0	0	0	0	0	0	0	0	0	0	0	1	0	0	0	0	0
ENSG00000240246	0	0	0	0	0	0	0	0	0	0	0	0	0	0	0	0	0	0	0	0	0	0	0	0	0	0	0	0	0	0	0	0
ENSG00000241929	0	0	0	0	0	0	0	0	0	0	0	0	0	0	0	0	0	0	0	0	0	0	0	0	0	0	0	0	0	0	0	0
ENSG00000282502	0	0	0	0	0	0	0	0	0	0	0	0	0	0	0	0	0	0	0	0	0	0	0	0	0	0	0	0	0	0	0	0
ENSG00000239408	0	0	0	0	0	0	0	0	0	0	0	0	0	0	0	0	0	0	0	0	0	0	0	0	0	0	0	0	0	0	0	0
ENSG00000169903	0	0	0	0	0	0	0	0	0	0	0	0	0	0	0	0	0	0	0	0	0	0	0	0	0	0	0	0	0	0	0	0
ENSG00000244503	0	0	0	0	0	0	0	0	0	0	0	1	0	0	0	0	0	0	0	0	0	0	0	0	0	0	0	0	0	0	0	0
ENSG00000018408	14	18	8	11	4	7	1	4	78	48	338	52	91	111	94	136	13	5	6	21	16	8	1	4	74	73	100	269	44	95	50	129
ENSG00000252581	0	0	0	0	0	0	0	0	0	0	1	1	1	1	0	0	0	0	0	0	0	0	0	0	0	0	0	0	0	0	2	0
ENSG00000241985	0	0	0	0	0	0	0	0	3	1	12	0	2	1	0	0	0	0	0	2	1	0	0	0	5	6	4	9	0	0	0	2
ENSG00000241313	0	0	0	0	0	0	0	0	13	4	34	2	4	4	3	11	0	0	0	0	0	0	0	1	3	6	3	8	6	5	2	7
ENSG00000114744	823	543	687	561	961	1034	806	1045	838	677	3122	477	1460	1297	1552	1720	900	1123	958	845	2486	1002	1673	1010	962	1284	778	1979	1474	1487	1650	1742
ENSG00000206199	3	5	3	2	4	3	3	3	1	0	17	1	3	10	5	5	0	1	1	6	5	4	6	4	6	3	4	6	5	5	8	7
ENSG00000251854	0	0	0	0	0	0	0	0	0	0	0	0	0	0	0	0	0	0	0	0	0	0	0	0	0	0	0	0	0	0	0	0
ENSG00000082996	583	409	446	369	663	716	571	880	614	519	2426	422	955	900	954	1197	657	889	767	675	1703	723	1297	831	719	1022	731	1738	836	1027	1064	1202
ENSG00000252172	5	3	1	3	2	1	0	2	1	1	9	0	3	9	1	11	0	1	0	1	5	4	7	4	4	2	4	6	1	3	8	5
ENSG00000239351	4	3	8	4	3	5	3	6	4	5	22	0	2	5	8	1	2	5	3	8	5	8	2	5	3	4	3	23	0	5	5	3
ENSG00000070087	1077	1277	986	1259	1620	1780	1048	1888	233	401	1206	287	301	575	382	683	402	1042	566	1078	1873	866	1429	750	261	559	412	1218	168	463	213	500
ENSG00000242791	0	4	0	0	4	5	1	3	1	2	8	4	1	2	0	2	1	1	1	4	0	0	0	1	1	2	2	1	0	1	0	0
ENSG00000224831	16	7	7	6	2	7	6	6	10	1	15	5	6	3	3	3	11	11	4	8	8	6	11	8	6	5	9	13	4	8	14	7
ENSG00000182000	0	1	0	0	0	0	0	0	0	0	2	0	0	2	0	0	0	0	0	1	1	0	0	0	0	0	0	0	0	0	3	1
ENSG00000241947	1	0	0	0	0	1	0	0	2	0	1	0	0	0	0	0	0	0	1	1	2	0	0	0	0	0	2	4	0	0	0	0
ENSG00000243944	0	0	1	0	1	0	0	0	0	0	1	0	2	0	0	1	0	0	1	0	0	0	0	0	0	0	0	0	0	0	0	0
ENSG00000240477	1	0	1	0	0	0	0	0	0	0	2	0	0	0	0	0	0	1	0	0	0	0	0	0	0	0	0	1	1	0	0	0
ENSG00000243321	0	0	0	0	0	0	0	0	0	0	0	0	0	0	0	0	0	0	0	0	0	0	0	0	0	0	0	0	0	0	0	0
ENSG00000241143	0	0	0	0	0	0	0	0	0	0	1	0	0	0	0	0	0	0	0	0	0	0	0	0	0	0	0	0	0	0	0	0
ENSG00000241048	0	0	0	1	0	0	0	0	0	0	0	0	0	0	0	0	0	0	0	0	0	0	0	0	0	0	0	0	0	0	0	0
ENSG00000244541	0	0	0	0	0	0	0	0	0	0	0	0	0	0	2	0	0	0	0	0	0	0	0	0	0	0	0	0	2	0	0	0
ENSG00000278234	0	0	0	0	0	0	0	0	0	0	0	0	0	0	0	0	0	0	0	0	0	0	0	0	0	0	0	0	0	0	0	0
ENSG00000243550	0	0	0	0	0	0	0	0	0	0	0	0	0	0	0	0	0	0	0	0	0	0	0	0	0	0	0	0	0	0	0	0
ENSG00000196428	664	612	603	541	859	836	504	798	156	170	731	126	1067	1049	1184	1190	158	223	242	461	1146	536	821	454	203	367	185	672	520	884	647	1023
ENSG00000120742	4333	2878	3444	3023	3357	3393	2518	3729	2441	2051	7295	1450	4283	4031	4403	5238	1860	2762	2531	3270	6328	2608	4652	2481	2602	3686	1557	5132	3901	4554	4124	5012
ENSG00000144895	2871	1819	1886	1762	2023	2035	1519	2115	2738	2150	8958	1647	3096	3091	2916	3734	2748	3790	2871	2814	5253	1955	3278	2195	2473	3655	2003	5845	3283	3480	3794	4194
ENSG00000198843	1977	1200	1501	1077	1904	1759	1342	1927	1652	1285	6627	1036	1298	1258	1455	1528	2647	3380	3032	2318	5455	2200	3669	2264	2022	2674	1841	4549	2128	1882	2403	2264
ENSG00000271711	8	9	8	12	3	2	3	7	6	4	17	6	4	7	4	6	4	0	1	4	18	11	12	8	5	11	11	19	6	7	4	11
ENSG00000163645	7	4	8	2	10	7	4	7	9	10	58	7	3	1	2	4	5	5	1	8	15	4	8	6	3	9	17	35	0	5	7	2
ENSG00000240137	21	13	23	14	18	37	20	27	45	45	255	38	14	16	9	17	8	8	9	10	61	25	28	18	47	44	63	153	10	9	13	12
ENSG00000244668	2	7	2	5	4	4	2	0	13	7	49	4	0	2	3	1	0	3	3	3	2	5	1	2	11	18	15	30	1	2	1	9
ENSG00000181788	1565	1292	1443	1267	981	978	680	908	601	542	1980	429	1616	1303	1851	1596	698	1157	1005	1044	1523	620	1208	461	591	859	439	1278	1340	1475	1546	1432
ENSG00000244265	4	9	9	10	6	4	7	7	5	8	29	3	12	9	16	13	3	5	3	5	13	4	6	2	6	4	8	20	4	7	11	6
ENSG00000280141	0	0	0	0	0	0	0	0	0	0	0	0	0	0	0	0	0	0	0	0	0	0	0	0	0	0	0	0	0	0	0	0
ENSG00000239265	3	8	6	9	2	3	0	5	1	0	12	2	2	1	1	2	3	4	1	7	5	1	8	5	2	0	3	7	0	1	0	0
ENSG00000214237	1	0	0	0	0	0	0	1	0	0	0	0	2	0	1	0	0	0	1	1	1	0	2	0	0	0	1	1	1	2	1	1
ENSG00000260234	0	0	0	0	0	0	0	0	0	0	0	0	1	0	0	0	0	0	0	0	0	0	1	0	0	3	0	0	0	1	0	0
ENSG00000243273	0	0	0	0	0	0	0	0	0	0	0	0	0	0	0	0	1	0	0	0	0	0	0	0	0	0	0	0	1	0	1	0
ENSG00000244382	0	0	0	0	0	0	0	0	0	0	0	0	0	0	0	0	0	0	0	0	0	0	0	0	0	0	0	0	0	0	0	0
ENSG00000163646	0	0	0	0	0	0	0	0	0	0	0	0	0	0	1	0	0	0	0	1	0	0	0	0	1	0	0	0	0	1	1	3
ENSG00000276055	0	0	0	0	0	0	0	0	0	0	0	0	0	0	0	0	0	0	0	0	0	0	0	0	0	0	0	0	0	0	0	0
ENSG00000144893	1004	1128	949	1098	2052	1949	1532	2140	687	579	3565	570	102	136	88	193	228	401	312	912	3769	1701	2490	1611	1015	1421	1194	3573	77	168	93	167
ENSG00000174946	15	9	9	11	53	86	56	108	547	395	6390	670	192	129	180	201	13	15	23	35	134	92	76	99	547	684	1123	3164	166	109	181	123
ENSG00000174944	21	18	19	19	39	47	48	51	72	37	767	65	1	0	2	2	10	4	8	42	151	64	64	105	72	82	87	371	6	5	7	8
ENSG00000239570	6	5	5	6	17	11	12	17	5	17	52	5	0	1	2	2	1	0	1	6	23	12	15	30	17	16	14	46	0	3	2	3
ENSG00000138271	8	4	4	8	26	27	15	23	3	8	48	3	1	0	2	1	1	1	1	15	56	32	19	34	21	27	15	46	0	2	6	5
ENSG00000181631	5	6	4	3	14	13	14	19	2	2	13	0	1	0	1	0	1	3	0	8	32	27	15	23	10	20	11	39	0	1	0	4
ENSG00000169313	5	5	5	3	32	26	19	49	3	2	22	0	5	5	1	5	2	3	0	11	76	31	41	37	16	9	9	40	1	2	3	3
ENSG00000152580	3	4	1	0	13	15	15	19	2	1	24	0	8	9	10	7	1	0	0	14	39	16	13	41	14	20	10	48	8	5	10	6
ENSG00000242810	0	0	0	0	0	0	1	1	0	0	1	1	0	0	0	0	0	0	0	0	1	0	2	0	0	0	0	0	0	0	0	0
ENSG00000242119	0	0	0	0	0	0	0	0	0	0	0	0	0	0	0	0	0	0	0	0	0	0	0	0	0	0	0	2	0	0	0	0
ENSG00000264330	0	0	0	0	0	0	0	0	0	0	0	0	0	0	0	0	0	0	0	0	0	0	0	0	0	0	0	0	0	0	0	0
ENSG00000241151	0	0	0	0	0	0	0	1	0	0	0	0	0	0	0	0	0	0	0	0	0	0	0	0	0	0	0	0	0	0	0	0
ENSG00000197953	0	0	0	0	1	0	0	0	0	0	0	0	2	0	0	0	0	0	0	0	0	0	0	0	0	0	0	0	0	0	1	0
ENSG00000242908	0	0	0	0	0	0	1	0	0	0	0	0	0	1	0	1	0	0	0	0	0	0	1	0	0	0	0	0	0	0	0	1
ENSG00000242097	0	0	0	0	0	2	0	0	0	0	0	0	0	0	0	0	0	0	0	0	0	0	0	0	0	0	0	0	0	0	0	0
ENSG00000240602	0	0	0	0	0	0	0	1	0	0	0	0	0	0	0	0	0	0	0	0	0	0	0	0	0	0	0	0	0	0	0	1
ENSG00000250271	0	0	0	0	0	0	0	0	0	0	0	0	0	0	0	1	0	0	0	0	1	0	0	0	0	0	0	0	0	0	0	1
ENSG00000114771	0	0	0	0	0	0	0	0	0	0	1	0	1	0	0	0	0	0	0	0	0	0	0	0	1	0	0	0	0	0	0	0
ENSG00000198829	1	1	0	0	75	38	64	70	0	0	2	0	117	53	145	91	0	2	2	0	80	15	95	19	0	1	3	0	88	31	158	47
ENSG00000239941	0	0	0	0	1	3	0	0	0	0	0	0	4	5	2	4	0	0	0	0	0	0	1	0	1	0	0	0	1	0	0	0
ENSG00000152601	7477	5382	6614	5413	4808	4664	4091	5047	8558	6989	35447	5678	6196	5497	6393	6577	5088	7152	7190	8573	11902	4712	9379	4972	10208	14203	7988	25388	5403	6537	6631	7537
ENSG00000229619	66	33	62	35	12	21	20	28	87	68	395	48	43	35	57	62	24	20	45	68	40	14	31	19	101	120	78	253	43	57	33	66
ENSG00000221962	13	2	8	5	6	8	4	2	9	9	40	2	14	8	10	8	0	0	1	4	17	10	16	16	24	21	10	44	12	19	12	11
ENSG00000201217	40	28	23	23	19	16	15	19	31	26	160	13	20	24	25	31	5	7	3	38	36	26	15	9	26	60	44	115	6	29	16	33
ENSG00000243305	0	0	0	0	0	0	0	0	0	0	0	0	0	0	0	0	0	0	0	0	0	0	0	0	0	0	0	0	0	0	0	0
ENSG00000244545	0	0	0	0	1	0	0	0	0	0	0	0	0	0	0	0	0	0	0	0	0	0	0	0	0	0	0	0	0	0	1	1
ENSG00000242561	0	0	0	0	1	0	1	0	0	0	0	0	0	0	0	0	0	0	0	0	4	3	2	3	0	0	0	0	0	1	1	0
ENSG00000169860	40	109	41	105	1128	1419	826	1369	256	212	1145	208	628	733	550	987	22	57	17	147	1963	908	1451	862	222	325	236	604	271	557	234	584
ENSG00000241810	0	0	0	0	0	0	0	0	0	0	0	0	0	0	0	0	0	0	0	0	0	0	0	0	0	0	0	0	0	0	0	0
ENSG00000241671	0	0	0	0	0	0	0	0	0	0	1	0	0	0	0	0	0	0	0	0	0	0	0	0	0	0	0	0	0	0	0	0
ENSG00000244268	1	2	4	1	8	8	5	7	0	0	16	2	0	0	2	3	0	0	0	1	10	2	10	1	0	0	1	2	0	1	0	0
ENSG00000181467	1206	993	1003	941	1949	2172	1556	2259	710	655	4178	641	1202	1088	1025	1297	438	819	689	858	3331	1536	2649	1277	711	1107	1163	2319	808	984	858	1019
ENSG00000265813	0	0	0	0	0	0	0	0	0	0	4	0	0	0	0	0	0	0	0	0	0	0	0	0	0	0	0	1	0	0	0	0
ENSG00000241220	0	0	0	0	1	0	0	0	0	0	0	0	0	0	0	0	0	0	0	0	0	0	0	0	0	0	0	0	0	0	0	0
ENSG00000241912	1	0	0	0	0	0	0	0	0	0	0	0	0	0	1	0	0	0	0	0	0	2	0	0	0	0	0	1	0	0	0	0
ENSG00000238755	0	0	0	0	5	1	1	2	0	0	0	0	0	0	1	0	0	0	0	0	1	1	0	1	0	0	2	1	0	0	0	0
ENSG00000243012	0	0	0	0	0	0	0	0	0	0	0	0	0	0	0	0	0	0	0	0	0	0	0	0	0	0	1	1	0	0	0	0
ENSG00000207323	0	0	0	0	0	0	0	0	0	0	0	0	0	0	0	0	0	0	0	0	0	0	0	0	0	0	0	0	0	0	0	0
ENSG00000237787	0	0	0	0	1	0	1	0	0	0	0	0	0	0	0	0	0	0	0	0	0	0	0	0	0	0	0	0	0	0	0	0
ENSG00000200162	0	0	0	0	0	0	0	0	0	0	0	0	0	0	0	0	0	0	0	0	0	0	0	0	0	0	0	0	0	0	0	0
ENSG00000243486	0	0	0	0	0	0	0	0	0	0	0	0	0	0	0	0	0	0	0	0	0	0	0	0	0	0	0	0	0	0	0	0
ENSG00000201398	0	0	0	0	0	0	0	0	0	0	0	0	0	0	0	0	0	0	0	0	0	0	0	0	0	0	0	0	0	0	0	0
ENSG00000240068	0	0	0	0	0	0	0	0	0	0	0	0	0	0	0	0	0	0	0	0	0	0	0	0	0	0	0	0	0	0	0	0
ENSG00000243069	7	8	5	12	3	10	3	14	9	10	11	4	0	1	0	0	2	7	2	9	7	4	10	5	4	4	4	2	1	0	0	0
ENSG00000114790	221	307	248	273	146	260	166	244	230	173	684	141	22	10	16	13	44	95	50	197	188	106	155	96	246	332	160	646	6	20	17	20
ENSG00000174953	2756	1895	2297	1807	2566	2736	2112	3053	2066	1624	8006	1244	2713	2427	2942	3246	1228	1769	1734	2256	5400	2182	4007	2312	2161	3064	1886	5179	2240	3034	2494	3226
ENSG00000271922	0	0	0	0	0	0	0	0	0	0	0	0	0	0	0	0	0	0	0	0	0	0	0	0	0	0	0	0	0	0	0	0
ENSG00000279305	0	0	0	0	0	0	0	0	0	0	0	0	0	0	0	0	0	0	0	0	0	0	0	0	0	0	0	0	0	0	0	0
ENSG00000174948	0	0	0	0	0	0	0	0	0	0	0	0	0	0	0	0	0	0	0	0	0	0	0	0	0	0	0	1	0	0	0	0
ENSG00000240048	0	0	0	0	0	0	0	0	0	0	1	0	1	0	0	0	0	0	0	0	0	0	0	0	0	0	0	2	0	0	0	0

ENSG00000243861	0	0	0	0	0	0	0	0	0	0	0	0	0	0	0	0	0	0	0	0	0	0	0	0	0	0	0	0	0	0	0	0
ENSG00000243154	0	0	0	0	0	0	0	0	0	0	0	0	0	0	0	0	0	0	0	0	0	0	0	0	0	0	2	0	0	0	0	0
ENSG00000242151	0	0	0	0	0	0	0	0	0	0	0	0	0	0	0	0	0	0	0	0	0	0	0	0	0	0	0	0	0	0	0	0
ENSG00000240601	0	0	0	0	0	0	0	0	0	0	0	0	0	0	0	0	0	0	0	0	0	0	0	0	0	0	0	0	0	0	0	0
ENSG00000243969	0	0	0	0	0	0	0	0	0	0	0	0	0	0	0	0	0	0	0	0	2	0	0	1	0	0	0	0	0	0	0	0
ENSG00000243953	0	0	0	0	0	0	0	0	0	0	0	0	0	0	0	0	0	0	0	0	0	0	0	0	0	0	0	0	0	0	0	0
ENSG00000196549	0	1	3	1	224	312	214	303	0	0	6	0	0	0	0	0	0	3	1	1	221	100	162	101	0	1	1	3	0	0	0	0
ENSG00000240666	0	0	0	0	2	1	1	0	0	0	0	0	0	0	0	0	0	0	0	0	0	0	0	2	0	0	0	0	0	0	0	0
ENSG00000241336	0	0	0	0	4	7	4	7	0	0	0	0	0	0	0	0	0	0	0	0	7	7	3	4	0	0	0	0	0	0	0	0
ENSG00000240045	0	2	0	1	1	0	0	0	0	0	0	0	1	0	0	0	0	0	0	0	0	0	0	0	0	0	0	0	0	0	0	0
ENSG00000242790	0	0	0	0	0	0	0	0	0	0	0	0	0	0	0	0	0	0	0	0	0	0	0	0	0	0	0	0	0	0	0	0
ENSG00000239443	0	0	0	0	0	0	0	1	0	0	0	0	0	0	0	0	0	0	0	0	0	0	0	1	0	0	0	0	0	0	0	0
ENSG00000114805	355	167	513	198	1223	1309	937	1311	1	0	4	1	2	0	1	4	269	338	754	511	2425	1045	1945	963	0	1	0	1	0	5	1	5
ENSG00000248772	0	0	0	0	0	0	0	0	0	0	0	0	0	0	0	0	0	0	0	0	0	0	0	0	0	0	0	0	0	0	0	0
ENSG00000243518	1	0	0	0	0	0	0	0	0	0	0	0	0	0	0	0	0	0	0	0	1	0	0	0	0	0	0	0	0	0	0	0
ENSG00000242152	0	0	0	0	0	1	0	3	0	0	0	0	0	0	0	0	0	0	1	0	0	0	2	0	0	0	0	0	0	0	0	0
ENSG00000272096	0	0	0	0	0	0	1	1	0	0	0	0	0	0	0	0	0	1	0	0	1	1	0	0	0	0	0	0	0	0	0	0
ENSG00000239508	2	1	1	0	1	4	3	2	0	0	1	0	0	0	0	0	0	0	1	1	4	5	2	5	0	0	0	0	0	0	0	0
ENSG00000242925	0	0	0	0	2	5	1	2	0	0	0	0	0	0	0	0	0	0	0	0	6	6	2	2	0	0	0	0	0	0	0	0
ENSG00000239212	0	0	0	1	0	0	0	0	0	0	0	0	0	0	0	0	0	1	0	0	0	0	0	0	0	0	0	0	0	0	0	0
ENSG00000214919	1	0	0	0	0	0	0	0	0	0	0	0	0	0	0	0	0	0	0	0	0	0	0	0	0	1	2	0	0	0	0	0
ENSG00000241307	2	0	3	0	1	0	0	0	0	0	1	0	0	1	3	1	0	1	0	4	2	1	2	0	1	1	0	2	3	0	0	3
ENSG00000174928	242	131	153	146	118	108	47	98	45	43	183	23	105	126	117	133	118	170	143	182	176	55	92	52	37	55	57	100	88	111	77	121
ENSG00000169359	1353	923	1069	891	776	864	530	800	587	563	2549	370	1237	1026	1282	1396	495	627	621	849	1232	510	802	476	575	851	781	1647	798	1025	1027	1280
ENSG00000163655	4905	3738	4038	3334	3850	4032	3189	4389	3356	2819	10794	2127	4529	4307	4325	5313	2122	3183	2613	3464	7389	2774	5638	2602	3356	5079	2075	8506	3715	4291	3643	4577
ENSG00000240489	16	16	11	13	10	15	17	19	21	16	60	14	26	26	39	33	12	13	24	16	29	14	19	4	18	32	19	44	32	38	30	23
ENSG00000174930	0	0	0	0	0	1	0	1	0	1	0	0	0	0	0	0	0	0	0	0	0	0	0	0	0	0	0	0	0	0	0	0
ENSG00000169282	68	41	48	32	79	93	58	107	37	33	125	10	47	36	81	60	14	22	16	40	206	108	121	80	55	84	39	126	34	54	60	62
ENSG00000242017	0	0	0	0	0	0	0	0	0	0	2	0	1	0	1	0	4	0	0	0	0	0	1	0	0	0	1	1	0	2	1	3
ENSG00000242537	0	0	0	0	0	0	0	0	0	0	1	1	0	0	0	0	0	0	0	0	0	0	1	0	0	0	0	0	0	0	0	0
ENSG00000227709	0	0	0	0	0	0	0	0	0	0	0	0	0	0	0	0	0	0	0	0	0	0	0	0	0	0	0	0	0	0	0	0
ENSG00000240596	0	0	0	0	0	0	0	0	0	0	0	0	0	0	0	0	0	0	0	0	0	0	0	0	0	0	0	0	0	0	0	0
ENSG00000242370	0	0	0	0	0	0	0	0	0	0	0	0	0	0	0	0	0	0	0	0	0	0	0	0	0	0	0	0	0	0	0	0
ENSG00000272990	12	10	20	9	25	21	18	30	8	9	30	7	16	30	9	26	2	7	1	18	50	17	24	19	10	13	11	27	9	10	10	19
ENSG00000114850	4353	3084	3202	2883	3320	3242	2517	3685	2687	1913	9558	1537	4152	3654	4594	4564	5309	6951	4597	5200	9030	3238	6059	3507	3136	4183	2540	7233	5156	4828	5836	5707
ENSG00000243926	32	32	17	35	27	22	15	29	17	23	62	11	31	25	15	34	22	27	18	25	33	12	26	19	14	22	14	31	29	20	28	30
ENSG00000163659	488	423	390	424	355	392	250	408	742	619	3357	537	1080	1142	1123	1398	339	482	368	414	670	284	454	284	878	1280	1219	2731	1192	1368	1456	1683
ENSG00000174912	5	3	4	2	2	1	0	0	2	4	2	0	8	8	9	10	3	2	4	7	0	0	0	0	3	6	4	11	6	6	8	7
ENSG00000240875	2	0	0	0	0	0	0	0	3	0	0	0	46	66	55	73	2	4	0	1	0	0	0	0	0	0	0	1	22	20	18	33
ENSG00000230457	4	0	2	5	1	1	2	2	1	2	1	0	10	12	7	6	3	0	1	2	3	0	2	1	0	0	1	2	5	7	3	13
ENSG00000197980	0	0	0	0	40	35	17	35	2	1	6	1	45	33	60	64	0	1	0	1	54	25	30	27	0	1	3	4	23	41	72	52
ENSG00000241879	0	0	0	0	0	0	0	0	0	0	0	0	0	0	0	0	0	0	0	0	0	0	0	0	0	0	0	0	0	0	0	0
ENSG00000239706	0	0	0	0	0	1	0	0	0	0	0	0	1	0	0	1	0	0	0	0	2	0	0	0	0	0	0	0	0	1	1	1
ENSG00000222499	0	0	0	0	0	0	0	0	0	0	0	0	0	0	0	0	0	0	0	0	0	0	0	0	0	0	0	0	0	0	0	0
ENSG00000243629	0	2	0	1	0	0	0	0	1	5	8	2	1	0	4	4	0	2	0	0	0	0	0	0	0	2	0	6	3	0	3	1
ENSG00000241544	2	1	0	0	2	1	0	0	0	1	0	0	0	0	0	0	0	0	0	0	0	0	0	0	0	1	0	1	0	0	0	1
ENSG00000241135	0	1	1	0	0	0	0	0	1	0	2	0	2	0	0	1	0	0	0	0	0	0	1	0	1	0	1	0	0	0	0	2
ENSG00000163660	4080	2886	3393	2836	3135	3227	2356	3288	3192	2706	13378	2123	3362	3261	3632	4346	1954	1800	1819	2957	6072	2495	3572	2791	2979	4420	3277	8159	2086	3578	2801	4155
ENSG00000201778	0	0	0	0	0	0	0	0	0	0	0	0	0	0	0	0	0	0	0	0	0	0	0	0	0	0	0	0	0	0	0	0
ENSG00000244515	18	21	17	12	7	9	10	6	3	5	13	0	13	9	11	8	6	3	9	15	15	1	7	4	5	7	9	10	7	4	8	4
ENSG00000241770	7	2	2	2	3	1	4	1	2	2	8	0	2	4	3	1	2	3	0	2	1	0	3	2	3	5	1	2	2	2	1	7
ENSG00000243176	14	8	18	8	28	40	21	49	20	19	101	11	38	27	34	51	4	10	8	4	57	23	47	28	22	25	22	53	9	18	24	24
ENSG00000270981	0	0	0	0	0	2	0	1	0	2	2	0	0	0	2	4	0	0	0	0	1	1	0	1	0	0	0	5	0	0	1	1
ENSG00000197415	2	4	6	0	10	6	7	10	4	8	34	10	3	10	9	6	0	4	4	1	12	8	8	4	13	10	5	20	0	6	7	11
ENSG00000163661	1	0	1	3	4	4	1	6	8	7	21	3	1	4	0	2	1	1	0	0	2	2	1	4	1	1	1	6	0	0	0	0
ENSG00000174899	0	2	0	0	0	0	0	0	0	0	0	0	0	0	0	0	0	0	0	0	0	0	1	0	0	0	0	0	0	1	0	0
ENSG00000251751	0	0	0	0	0	0	0	0	0	0	0	0	0	0	0	0	0	0	0	0	0	0	0	0	0	0	0	0	0	0	0	0
ENSG00000239716	0	0	0	0	0	0	0	0	0	0	0	0	0	0	0	0	0	0	0	0	0	0	0	0	0	0	0	0	0	0	0	0
ENSG00000242536	0	0	0	0	0	0	0	0	0	0	0	0	0	0	0	0	0	0	0	0	0	0	0	0	0	0	0	0	0	0	0	0
ENSG00000168779	66	91	85	92	0	0	0	1	15	13	59	14	22	27	20	48	32	37	32	68	3	1	2	0	5	19	7	31	11	18	11	18
ENSG00000174891	1345	1064	1049	994	1240	1243	993	1408	926	741	2665	492	1459	1372	1403	1658	987	1314	972	1081	2575	1048	1844	998	936	1184	532	1989	1125	1269	1300	1497
ENSG00000241723	0	0	1	0	0	0	0	0	0	0	1	0	1	1	0	1	1	2	0	1	1	1	1	0	1	2	0	4	0	2	2	3
ENSG00000277143	0	1	1	0	2	1	1	4	0	0	2	3	0	3	0	3	0	1	0	3	6	2	0	8	1	3	0	4	0	4	1	2
ENSG00000243314	1	3	2	2	1	1	1	1	1	2	2	0	7	1	1	2	0	0	0	2	7	2	0	3	4	4	0	8	0	1	3	4
ENSG00000243150	0	0	0	0	0	1	0	0	0	1	2	1	0	0	0	0	0	1	0	1	0	0	2	1	0	0	0	0	0	1	0	0
ENSG00000178053	0	0	0	0	1	0	0	2	246	189	838	168	137	123	118	209	0	0	0	0	1	0	0	1	159	200	117	486	87	152	145	179
ENSG00000243025	0	0	0	0	0	0	0	0	0	0	3	0	0	1	0	0	0	0	0	0	0	0	0	0	0	0	0	1	1	1	0	0
ENSG00000243165	0	0	0	0	0	0	0	0	0	0	0	0	0	0	0	0	0	0	0	0	0	0	0	0	0	0	0	0	0	0	0	0
ENSG00000168827	1940	1142	1689	1234	1973	2058	1628	2178	1238	950	5118	737	2986	2413	3362	3237	864	1339	1333	1712	4471	1796	3317	1656	1589	2305	1110	3433	3050	3530	3558	3806
ENSG00000079257	10	2	11	4	165	180	131	191	144	132	440	98	151	157	147	223	5	2	6	7	381	166	259	195	106	159	107	264	201	146	200	196
ENSG00000272087	10	8	17	4	12	20	8	18	15	12	90	15	32	32	42	35	8	9	11	21	52	11	29	19	23	43	13	62	36	62	39	56
ENSG00000272440	5	0	4	1	1	4	2	3	1	4	8	5	7	4	8	6	4	2	1	2	4	6	3	5	3	4	1	18	5	5	10	16
ENSG00000118849	8	7	8	9	6	6	5	2	5	0	18	2	12	12	11	10	4	1	0	13	10	5	14	14	13	17	5	34	3	10	12	11
ENSG00000118855	656	385	573	370	319	362	304	361	438	317	2237	335	724	638	667	821	360	503	472	593	872	351	618	316	531	714	650	1494	841	899	849	967
ENSG00000240207	18	11	15	17	13	10	7	15	6	4	55	1	13	10	5	9	3	4	2	17	11	3	7	6	22	16	24	38	2	4	11	6
ENSG00000243675	0	1	1	0	0	0	0	3	0	0	3	0	1	0	3	0	0	0	0	2	0	0	0	1	1	0	2	2	0	0	0	1
ENSG00000271778	7	6	2	4	1	3	1	1	3	3	8	0	6	4	6	1	0	3	0	0	2	0	0	1	0	4	3	3	5	2	0	0
ENSG00000272247	4	2	3	4	0	0	1	1	0	0	4	1	1	1	2	1	2	3	1	7	0	0	0	0	0	0	0	4	0	0	1	3
ENSG00000232374	6	1	1	2	1	2	0	1	0	1	2	1	3	0	0	0	0	0	1	0	3	0	1	1	1	0	1	4	0	0	0	0
ENSG00000279311	1	0	2	0	4	1	2	6	0	0	7	0	0	0	0	0	0	0	0	0	2	0	1	0	0	0	1	1	0	0	0	0
ENSG00000214216	0	0	0	0	0	2	0	0	0	0	0	0	0	0	0	0	0	0	0	0	0	0	0	0	0	0	0	0	0	0	0	0
ENSG00000283154	0	0	0	0	0	0	0	0	0	0	0	0	0	0	0	0	0	0	0	0	0	0	0	0	0	0	0	0	0	0	0	0
ENSG00000283245	0	0	0	0	0	0	0	0	0	0	0	0	0	0	0	0	0	0	0	0	0	0	0	0	0	0	0	0	1	0	0	0
ENSG00000263634	0	0	0	0	0	0	0	0	0	0	0	0	0	0	0	0	0	0	0	0	0	0	0	0	0	0	0	0	0	0	0	0
ENSG00000244249	0	0	0	0	0	0	0	0	0	0	0	0	0	0	0	0	0	0	0	0	0	0	0	0	0	0	0	0	0	0	0	0
ENSG00000243116	0	0	0	0	0	0	0	0	0	0	1	0	0	0	0	0	0	0	0	0	0	0	0	0	0	0	3	2	0	0	0	0
ENSG00000241211	0	3	0	2	0	0	0	0	3	5	6	0	0	2	0	1	0	0	0	1	0	0	0	0	10	14	2	30	2	4	0	7
ENSG00000252763	0	0	1	0	0	0	0	0	1	0	6	0	0	5	0	0	0	0	0	0	0	0	0	0	4	2	0	9	0	0	0	1
ENSG00000151967	21	18	18	29	0	0	0	0	147	135	888	113	61	83	58	101	10	35	12	38	0	0	0	0	197	260	144	609	59	112	62	103
ENSG00000244040	0	1	0	0	0	0	0	0	0	0	3	1	1	6	1	2	0	0	0	0	0	0	0	0	1	0	0	3	1	1	0	2
ENSG00000168811	0	4	0	0	0	0	0	0	1	0	4	2	19	19	13	14	0	1	0	5	0	0	0	0	2	1	0	4	9	19	17	28
ENSG00000242107	0	0	0	0	0	0	0	0	0	0	0	0	0	0	0	0	0	0	0	0	0	0	0	0	0	0	0	0	0	0	0	0
ENSG00000184100	2	5	4	4	5	2	3	6	2	2	7	4	0	3	1	4	2	5	5	7	9	3	4	1	1	6	1	7	0	3	1	1
ENSG00000180044	0	0	0	0	0	0	0	0	11	18	35	14	68	74	66	103	0	0	0	1	0	0	0	0	7	20	6	30	68	149	55	117
ENSG00000248710	0	0	0	1	0	0	0	0	0	0	6	1	0	0	0	0	0	0	0	1	0	0	0	0	1	6	5	6	0	0	0	0
ENSG00000068885	567	349	537	322	487	511	366	527	752	653	2342	404	765	732	771	931	330	329	341	477	728	354	573	378	530	704	536	1457	514	795	601	936
ENSG00000243687	1	1	3	0	1	2	0	0	3	1	4	0	3	3	3	2	0	0	0	1	3	1	0	1	2	3	2	13	0	3	2	5
ENSG00000113810	9809	6901	8174	6730	8255	8891	7128	9423	7784	6087	22472	4734	6349	6327	7189	8513	3352	3796	3820	6016	15206	6424	10986	6545	8298	11949	3220	19320	4426	6852	5719	7241
ENSG00000207779	0	0	0	0	0	0	0	0	0	0	1	0	0	0	0	0	0	0	0	0	1	2	0	1	2	0	0	3	0	0	0	1
ENSG00000198987	0	1	1	0	0	0	0	0	0	1	1	0	0	1	0	0	0	0	0	0	0	5	1	0	0	0	0	0	1	0	0	0
ENSG00000213186	362	260	338	258	92	95	58	96	629	550	1803	333	1183	1158	1171	1591	139	198	182	281	140	80	79	85	646	838	382	1273	862	1165	846	1230
ENSG00000244009	1	4	0	0	2	0	1	1	0	0	0	1	0	1	0	0	1	1	0	1	2	0	1	0	0	1	2	0	0	2	0	1
ENSG00000238427	0	0	0	0	0	0	0	2	0	0	0	0	0	0	0	0	0	0	0	0	0	0	0	0	0	0	0	0	0	0	0	0
ENSG00000186432	2796	1933	2181	1943	2274	2406	1729	2439	1838	1586	8089	1255	4117	3596	4552	4459	953	1514	1506	2115	4599	1838	3383	1761	2186	3267	2043	5623	3551	4336	4170	4731
ENSG00000238741	14	5	7	7	17	7	8	25	9	9	22	0	9	12	5	7	24	5	4	11	46	14	25	28	13	5	8	27	11	13	14	20
ENSG00000229320	214	164	180	200	75	90	62	95	95	92	345	81	177	186	207	212	159	143	122	197	137	71	94	57	91	137	74	200	150	211	181	230
ENSG00000242285	0	0	0	0	0	0	0	0	0	1	0	0	0	0	2	2	0	0	0	1	0	0	0	0	1	0	0	0	1	5	3	2
ENSG00000179674	0	0	0	0	0	0	0	0	0	0	0	0	67	60	87	84	0	0	0	0	0	0	0	0	0	0	0	0	74	83	122	105
ENSG00000207084	0	0	0	0	0	0	0	0	0	0	0	0	0	0	0	1	0	0	0	0	0	0	0	0	0	0	0	0	1	1	3	1
ENSG00000260572	19	13	19	25	4	8	8	5	27	38	170	15	28	31	29	27	21	19	13	18	3	6	12	5	24	34	31	69	27	29	33	42
ENSG00000163590	1315	1311	1068	1408	521	526	428	602	657	706	3624	599	1089	1083	964	1400	260	491	307	956	726	307	558	282	762	1308	985	2931	409	782	503	799
ENSG00000169255	0	0	0	0	0	0	0	0	0	0	0	0	1	0	0	0	0	2	0	0	0	0	0	0	0	0	0	1	0	0	0	0
ENSG00000169251	1781	1132	1186	1102	1414	1540	1115	1602	1362	1135	4622	835	2715	2294	3006	2964	2193	2971	2173	2268	3878	1508	2649	1845	1210	1966	1099	3430	3308	3190	4256	3725
ENSG00000271052	0	1	0	0	0	1	1	1	0	0	3	0	0	2	2	2	1	1	0	4	0	2	0	0	0	1	2	1	1	0	0	0
ENSG00000240138	0	0	0	0	0	0	0	0	0	0	0	0	0	0	0	0	0	0	0	0	0	0	0	0	0	0	0	0	0	0	0	0
ENSG00000243838	0	0	0	0	0	0	0	0	0	0	0	0	1	1	0	0	0	0	0	0	0	0	0	0	0	0	0	0	0	0	0	0
ENSG00000196542	0	0	0	0	0	0	0	0	0	0	0	0	0	1	0	0	0	0	0	0	0	0	0	0	0	3	0	0	0	4	4	5
ENSG00000240567	0	0	0	0	1	1	0	1	0	0	3	0	0	0	0	0	0	0	0	0	0	0	3	0	0	1	0	2	0	2	0	0
ENSG00000269888	5	6	7	5	7	3	2	4	16	5	31	5	0	3	10	6	10	3	18	4	9	3	8	9	5	6	8	9	8	6	12	8
ENSG00000234851	69	68	70	39	71	77	56	71	117	92	410	78	112	93	118	136	113	120	117	94	157	66	85	88	92	99	80	257	133	114	159	178
ENSG00000182447	0	0	0	0	0	1	0	0	0	0	0	0	0	0	0	0	0	0	0	0	0	0	0	0	0	0	0	0	1	0	0	0
ENSG00000240354	0	0	0	0	0	0	0	0	0	0	0	0	0	0	0	0	0	0	0	0	0	0	0	0	0	0	0	0	0	0	0	0
ENSG00000241874	0	0	0	0	0	0	0	0	0	0	0	0	0	0	0	0	0	0	0	0	0	0	0	0	0	0	0	0	0	0	0	1
ENSG00000243044	0	0	0	0	0	0	0	0	0	0	0	0	0	0	0	0	0	0	0	0	0	0	0	0	0	0	0	0	0	0	0	0
ENSG00000241168	0	0	0	0	0	0	0	0	0	0	0	0	0	0	0	0	0	0	0	0	0	0	0	0	0	0	0	0	0	0	0	0
ENSG00000244571	0	0	0	0	0	0	0	0	0	0	0	0	0	0	0	0	0	0	0	0	0	0	0	0	0	0	0	0	0	0	0	0
ENSG00000241369	0	0	0	0	0	0	0	0	0	0	3	0	0	0	0	1	0	0	0	0	0	0	0	1	0	0	0	1	0	0	1	0
ENSG00000238924	0	0	0	0	0	0	0	0	0	0	0	0	0	0	0	0	0	0	0	0	0	0	0	0	0	0	0	0	0	0	0	0
ENSG00000240792	0	0	0	0	0	0	0	0	0	0	0	0	0	0	0	0	0	0	0	0	0	0	0	0	0	0	0	0	0	0	0	0
ENSG00000214210	0	0	0	0	0	0	0	0	0	0	0	0	0	0	0	0	0	0	0	0	0	0	0	0	0	0	0	0	0	0	0	0
ENSG00000221251	0	0	0	0	0	0	0	0	0	0	0	0	0	0	0	0	0	0	0	0	0	0	0	0	0	0	0	0	0	0	0	0
ENSG00000241636	0	0	0	0	0	0	0	0	0	0	0	0	0	0	0	0	0	0	0	0	0	0	0	0	0	0	0	0	0	0	0	0
ENSG00000241767	0	0	0	0	0	0	0	0	0	0	5	0	0	1	0	1	0	0	0	0	0	0	0	0	0	1	0	1	0	0	0	0
ENSG00000090402	0	0	0	0	0	0	0	0	0	0	1	0	0	0	0	0	0	0	1	0	0	0	0	0	0	0	1	0	0	0	0	0
ENSG00000200052	0	0	0	0	0	0	0	0	0	0	0	0	0	0	0	0	0	0	0	0	0	0	0	0	0	0	0	0	0	0	0	0
ENSG00000239205	0	0	0	0	0	0	0	0	0	0	2	0	0	0	0	0	0	0	0	0	0	0	0	0	0	0	0	1	0	0	0	0
ENSG00000121871	0	0	0	0	1	0	0	0	0	0	0	0	0	0	0	0	0	0	0	0	0	0	0	0	0	0	1	0	0	0	0	0
ENSG00000244128	1	0	0	0	0	0	0	0	0	0	2	0	2	0	0	1	0	0	0	0	0	0	0	0	0	0	0	0	0	1	0	0
ENSG00000114200	874	457	612	452	2	6	2	6	245	189	783	132	1270	1355	1137	1807	659	683	631	560	5	0	3	1	231	284	255	584	973	1193	1164	1481
ENSG00000242178	0	0	0	0	1	0	2	1	0	0	0	0	0	0	1	1	0	0	0	0	13	12	17	16	0	0	0	0	1	1	6	4
ENSG00000240084	0	0	0	0	0	0	0	0	0	0	0	0	0	0	0	1	0	0	0	0	2	4	0	0	0	0	0	0	0	0	0	1
ENSG00000240824	0	0	0	0	0	0	0	1	0	0	0	0	0	0	0	0	0	0	0	0	4	0	4	1	0	0	0	0	0	0	0	0
ENSG00000242456	0	0	0	0	2	2	1	2	0	0	0	0	0	0	1	0	0	0	0	0	9	0	6	2	0	0	0	0	0	0	0	3
ENSG00000242326	0	0	0	0	0	0	0	0	0	0	0	0	0	0	3	1	0	0	0	0	0	0	4	0	0	0	0	0	0	0	1	0
ENSG00000244429	0	0	0	0	0	0	0	0	0	0	0	0	0	0	0	0	0	0	0	0	0	0	0	0	0	0	0	0	0	0	0	0
ENSG00000244321	0	0	0	0	0	0	0	0	0	0	0	0	0	0	2	1	0	0	0	0	0	0	0	0	0	0	0	0	0	1	2	0
ENSG00000274149	0	0	0	0	0	0	0	0	0	0	0	0	0	0	0	0	0	0	0	0	0	0	0	0	0	0	0	0	0	0	0	0
ENSG00000251759	0	0	0	0	0	0	0	0	0	0	0	0	0	0	0	0	0	0	0	0	0	0	0	0	0	0	0	0	0	0	0	0
ENSG00000224962	0	0	0	1	0	0	0	0	0	0	0	0	0	0	0	0	0	0	0	0	0	0	0	0	0	0	0	0	0	0	0	0
ENSG00000240458	0	0	0	0	0	0	0	0	0	0	0	0	0	0	0	0	0	0	0	0	0	0	0	0	0	0	0	0	0	0	0	0
ENSG00000231516	0	0	0	0	0	0	0	0	0	0	0	0	0	0	0	0	0	0	0	0	0	0	0	0	0	0	0	0	0	0	0	0
ENSG00000240033	0	0	0	0	0	0	0	0	0	0	0	0	2	2	6	4	0	0	0	0	0	0	0	0	0	0	0	0	0	1	0	0
ENSG00000239227	0	0	1	0	0	0	0	0	0	0	0	0	0	0	0	1	0	1	0	0	0	0	0	2	0	0	0	2	0	0	0	0
ENSG00000220343	0	0	0	0	0	0	0	0	0	0	0	0	0	1	7	7	0	0	0	0	0	0	0	0	0	0	0	0	0	0	0	0
ENSG00000169064	2	3	1	0	0	1	1	0	3	0	54	2	1	4	10	11	0	1	0	2	1	1	0	0	1	1	12	11	1	5	4	3
ENSG00000241669	0	0	0	0	0	0	0	0	0	0	0	0	0	0	0	0	0	0	0	0	0	0	0	0	0	0	0	0	0	0	0	0
ENSG00000114204	0	0	0	0	0	0	0	0	0	1	2	0	2	0	1	7	0	0	0	1	1	0	0	0	2	3	0	0	0	1	0	2
ENSG00000174776	1	0	1	0	3	1	1	2	1	0	2	2	5	8	9	2	0	0	0	0	2	1	2	0	0	0	0	2	0	1	2	6
ENSG00000114209	1281	933	903	862	1101	1198	859	1316	1063	759	3719	603	1263	1282	1404	1559	1477	1881	1455	1822	3441	1518	2399	1825	1260	1531	799	2688	1559	1560	2100	1942
ENSG00000241120	19	12	12	15	9	13	9	12	7	6	26	12	10	7	11	13	7	3	0	10	35	20	10	22	10	11	2	29	2	19	6	12
ENSG00000163536	54	41	45	52	132	152	136	171	45	30	178	28	121	166	165	214	68	102	84	102	391	150	363	190	30	37	42	92	114	150	195	225
ENSG00000244227	0	0	0	0	1	0	1	2	0	0	1	0	0	0	0	1	0	0	0	0	5	1	4	4	0	0	1	0	0	1	0	1
ENSG00000244706	0	0	0	0	0	0	2	0	0	0	0	0	0	0	0	0	0	0	0	0	1	0	0	0	0	0	0	0	0	0	0	0
ENSG00000239238	0	0	0	0	0	0	0	0	0	0	0	0	0	0	0	0	0	0	0	0	0	0	0	0	0	0	0	0	0	0	0	0
ENSG00000242036	0	0	0	0	0	0	0	0	0	0	0	0	0	0	0	0	0	0	0	0	0	0	0	0	0	0	0	0	0	0	0	0
ENSG00000241203	0	0	0	0	0	0	0	0	0	0	0	0	0	0	0	0	0	0	0	0	0	0	0	0	0	0	0	0	0	0	0	0
ENSG00000173905	0	0	0	0	4625	6121	4074	6924	2259	1837	10099	1587	1256	1226	1244	1577	0	0	0	0	13012	7667	10183	8236	2299	3488	2052	6146	1002	1296	1032	1352
ENSG00000206120	1	0	0	0	0	0	0	0	12	17	96	11	0	0	0	0	0	0	0	0	0	0	0	0	16	25	37	76	0	1	0	0
ENSG00000241882	0	0	0	0	0	0	0	0	0	0	0	0	0	0	0	0	0	0	0	0	0	0	0	0	0	0	1	2	0	0	0	0
ENSG00000222357	0	0	0	0	0	0	0	0	0	0	0	0	0	0	0	0	0	0	0	0	0	0	0	0	0	0	0	1	0	0	0	0
ENSG00000207717	0	0	0	0	0	0	0	0	0	0	0	0	0	0	0	0	0	0	0	0	0	0	0	0	0	0	0	0	0	0	0	0
ENSG00000226765	0	0	0	0	0	0	0	0	1	0	2	1	0	0	0	0	0	0	0	0	0	0	0	0	0	0	0	2	0	0	0	0
ENSG00000231688	0	0	0	0	0	0	0	0	0	0	2	0	0	0	0	0	0	0	0	0	0	0	0	0	0	1	5	4	0	0	0	0
ENSG00000242268	0	0	0	0	0	0	0	0	1	0	3	0	0	0	0	0	0	0	0	0	0	0	0	0	0	0	3	7	0	0	0	0
ENSG00000272797	0	0	0	0	0	0	0	0	0	0	0	0	0	0	0	0	0	0	0	0	0	0	0	0	0	0	1	1	0	0	0	0
ENSG00000273177	0	0	0	0	0	0	0	0	0	0	0	0	0	0	0	0	0	0	0	0	0	0	0	0	0	0	0	0	0	0	0	0
ENSG00000241383	0	0	0	0	0	0	0	0	0	0	0	0	0	0	0	0	0	0	0	0	0	0	0	0	0	0	0	0	0	0	0	0
ENSG00000085276	0	2	0	2	15	11	4	15	3	15	11	1	0	0	0	1	0	0	0	2	24	6	13	8	10	14	9	18	0	1	0	0
ENSG00000241479	0	1	0	0	0	0	0	0	0	0	1	0	0	1	0	0	0	0	0	0	0	0	0	0	0	0	0	0	0	1	1	1
ENSG00000213178	16	9	13	6	12	12	5	7	9	14	34	12	9	13	20	18	24	22	13	24	44	16	17	8	20	17	7	15	13	16	26	21
ENSG00000281137	0	0	0	0	0	0	0	0	0	0	0	0	0	0	0	0	0	0	0	0	0	0	0	0	0	0	0	0	0	0	0	0
ENSG00000242795	0	0	0	0	0	0	0	0	0	0	0	0	0	0	0	0	0	0	0	0	0	0	0	0	0	0	0	0	0	0	0	0
ENSG00000244381	0	0	0	0	0	0	0	0	0	0	0	0	0	0	0	0	0	0	0	0	0	0	0	0	0	0	0	0	0	0	0	0
ENSG00000206777	0	0	0	0	0	0	0	0	0	0	0	0	0	0	0	0	0	0	0	0	0	0	0	0	0	0	0	0	0	0	0	0
ENSG00000270141	1	1	3	0	5	2	2	2	0	0	5	0	4	1	0	1	0	0	5	0	7	5	2	5	0	0	2	6	2	0	3	3
ENSG00000277925	0	0	0	0	0	0	0	0	0	0	0	0	0	0	0	0	0	0	0	0	0	0	0	0	0	0	0	0	0	0	0	0
ENSG00000184378	18	12	18	16	29	22	14	31	3	0	2	0	11	18	27	20	9	8	27	11	47	28	28	32	0	0	1	3	21	16	17	22
ENSG00000269889	7	1	3	0	8	2	1	4	0	0	0	0	3	3	3	3	2	1	2	3	14	5	8	4	3	2	0	3	3	2	2	3
ENSG00000085274	657	467	525	447	590	629	482	651	715	615	2677	450	540	561	665	694	602	703	646	754	1475	653	1172	730	707	985	649	1706	601	728	767	751
ENSG00000269984	77	61	75	49	99	118	87	100	69	69	285	60	50	45	69	75	26	29	20	87	183	111	114	81	43	69	58	145	25	76	65	90
ENSG00000171757	369	290	314	349	603	587	429	641	412	312	1047	205	96	74	75	92	230	311	231	357	1119	494	730	420	344	413	164	649	42	79	36	80
ENSG00000270135	0	0	0	0	0	0	0	0	0	0	0	0	0	0	0	0	0	0	0	0	0	0	0	0	0	0	0	0	0	0	0	0
ENSG00000270096	0	0	0	0	0	0	0	0	0	0	0	0	0	0	0	0	0	0	0	0	0	0	0	0	0	0	0	0	0	0	0	0
ENSG00000188306	21	10	16	14	42	28	18	30	12	14	22	0	4	5	0	9	11	11	8	16	69	23	38	27	4	9	2	9	7	1	1	5
ENSG00000114248	5	2	5	1	7	8	4	7	3	3	2	1	2	0	0	1	0	0	0	5	10	1	5	4	0	4	1	6	0	2	0	1
ENSG00000240128	0	0	0	0	0	0	0	0	0	0	0	0	0	0	0	0	0	0	0	0	0	0	0	0	0	0	0	0	0	0	0	0
ENSG00000187033	0	1	0	0	0	0	0	0	0	0	2	0	0	0	1	0	0	0	0	1	0	0	0	0	0	2	0	3	0	0	2	0
ENSG00000244039	0	0	0	0	0	0	0	0	0	0	0	0	0	0	0	0	0	0	0	0	0	0	0	0	0	0	0	0	0	0	0	0
ENSG00000239219	5	7	8	4	2	2	2	1	0	0	9	1	1	0	0	1	2	4	3	6	2	1	1	4	1	1	1	5	0	1	0	2
ENSG00000244193	0	0	0	0	0	0	0	0	0	0	0	0	0	0	0	0	0	0	0	0	0	0	0	0	0	0	0	0	0	0	0	0
ENSG00000008952	2245	1510	1706	1510	2179	2249	1894	2493	1924	1535	7184	1137	2092	2135	2400	2843	2534	3213	2615	2644	5907	2391	4165	2446	1979	2649	1984	4639	2442	2594	3016	3247
ENSG00000240373	2	2	3	8	5	8	0	2	7	7	20	1	4	5	8	2	0	0	0	2	16	7	6	6	5	9	8	15	3	5	5	7
ENSG00000173890	177	161	216	178	13	8	4	14	227	246	551	140	335	341	338	416	92	244	150	210	9	7	7	13	235	300	135	506	396	386	385	457
ENSG00000201342	0	0	0	1	0	0	0	0	0	0	0	0	0	0	0	1	0	0	0	1	0	0	0	0	0	1	0	1	0	0	0	1
ENSG00000242583	0	1	0	1	0	0	0	0	1	0	1	0	0	0	1	4	0	0	0	2	0	0	0	0	0	0	0	2	1	1	0	0
ENSG00000173889	3233	2664	2714	2532	2025	2214	1497	2253	1859	1735	9271	1299	3007	2705	2962	3290	848	907	988	2288	3738	1712	2227	1638	1868	2770	2731	6019	1536	2652	2043	2935
ENSG00000268220	0	0	0	0	0	0	0	0	0	0	0	0	0	0	0	0	0	0	0	0	0	0	0	0	0	0	0	0	0	0	0	0
ENSG00000199536	0	3	7	3	5	6	4	2	3	0	9	3	4	2	2	6	0	0	1	4	11	5	4	7	7	10	6	15	0	4	8	7
ENSG00000163558	1054	868	905	884	1226	1287	854	1325	938	802	3249	533	1784	1755	1765	2186	367	622	474	708	1905	759	1359	692	1007	1398	980	2318	1282	1636	1391	1861
ENSG00000200118	0	1	0	0	3	0	0	0	0	1	0	0	1	1	3	1	0	2	0	0	1	0	1	1	0	2	2	1	0	1	2	3
ENSG00000239628	0	0	0	0	0	0	0	0	0	0	0	1	0	0	0	0	0	0	0	0	0	0	1	0	0	0	0	0	0	0	0	0
ENSG00000136603	255	198	298	205	300	266	190	277	831	731	4625	539	886	742	1180	1037	97	156	277	331	591	290	450	296	1016	1275	2113	2952	682	1006	1208	1128
ENSG00000242578	0	0	1	0	0	0	1	0	2	4	13	1	0	0	0	1	1	0	0	0	0	0	0	0	0	0	1	6	0	0	0	0
ENSG00000013297	0	0	0	0	0	0	1	0	1	3	16	6	0	0	0	2	0	0	0	0	1	1	0	0	5	2	5	7	1	0	0	2
ENSG00000283474	0	0	0	0	0	0	0	0	0	0	0	0	0	0	0	0	0	0	0	0	0	0	0	0	0	0	0	0	0	0	0	0
ENSG00000013293	0	0	0	0	0	0	0	0	0	1	1	0	0	0	0	0	0	0	0	1	0	0	0	0	0	0	2	0	0	0	0	0
ENSG00000241416	0	0	0	0	0	0	0	0	0	0	0	0	0	0	0	0	0	0	0	0	0	0	0	0	0	0	0	0	0	0	0	0
ENSG00000213174	0	0	0	0	0	0	0	0	0	0	0	0	0	0	0	0	0	0	0	0	0	0	0	0	0	0	0	0	0	0	0	0
ENSG00000244738	0	0	0	0	0	0	0	0	0	0	0	0	0	0	0	0	0	0	0	0	0	0	0	0	0	0	0	0	0	0	0	0
ENSG00000239739	0	0	0	0	0	0	0	0	0	0	0	0	0	0	0	0	0	0	0	0	0	0	0	0	0	0	0	0	0	0	0	0
ENSG00000163584	610	459	364	401	286	245	228	340	1120	758	2109	610	455	456	547	536	567	780	622	936	733	377	660	483	1099	1536	402	1826	659	645	748	699
ENSG00000163577	267	215	250	226	441	423	321	482	308	237	1417	202	226	252	260	335	184	213	180	273	799	329	515	290	441	701	395	1209	268	343	352	381
ENSG00000240704	0	0	0	0	0	0	0	0	0	0	0	0	1	1	0	0	0	0	0	0	0	0	0	0	0	0	0	0	0	1	0	0
ENSG00000199488	0	0	0	0	0	0	0	0	2	0	0	1	3	0	0	4	1	0	1	0	1	0	0	0	2	2	2	1	1	7	0	8
ENSG00000163581	0	0	0	0	0	0	0	0	5	3	13	3	5	3	1	1	0	0	0	0	0	0	0	0	18	10	2	18	0	2	0	0
ENSG00000154310	141	295	137	280	3096	3424	2492	3364	2398	2210	12832	1872	2117	2330	2436	2974	19	86	37	151	4997	2181	3827	1973	2370	3649	3442	8646	1111	1749	1358	2113
ENSG00000207963	0	0	0	0	0	1	2	0	0	0	7	0	0	5	2	0	0	0	0	0	1	1	2	1	0	4	1	8	1	2	2	2
ENSG00000222506	0	0	0	0	0	1	1	4	4	2	11	0	0	1	1	3	0	0	0	0	4	0	2	1	1	3	2	8	1	0	1	0
ENSG00000279673	2	3	4	10	63	42	39	57	48	34	222	39	54	50	73	80	1	7	4	15	106	38	86	47	44	56	36	99	60	58	57	82
ENSG00000240497	0	0	0	0	4	0	0	2	4	3	5	1	3	7	11	11	1	0	1	0	9	3	8	6	1	3	1	5	9	8	16	14
ENSG00000207114	0	0	0	0	0	0	0	0	0	0	0	0	0	0	0	0	0	0	0	0	0	0	0	0	0	0	0	0	0	0	0	0
ENSG00000234594	0	0	0	0	0	0	0	0	0	0	0	0	0	0	0	0	0	0	0	0	0	0	0	0	0	0	0	0	0	0	0	0
ENSG00000075651	0	4	6	1	0	0	0	0	286	277	1131	182	802	715	697	872	0	4	0	0	0	0	0	0	194	331	216	694	224	309	308	364
ENSG00000186329	0	0	0	0	0	0	0	0	0	1	1	0	2	0	0	0	0	0	0	0	0	0	0	0	0	0	1	0	0	0	0	0
ENSG00000234717	0	0	0	0	0	0	0	0	0	0	0	0	0	0	0	0	0	0	0	0	0	0	0	0	0	0	0	0	0	0	0	0
ENSG00000235943	0	0	0	0	0	0	0	0	0	0	0	0	0	0	0	0	0	0	0	0	0	0	0	0	0	0	0	0	0	0	0	0
ENSG00000075420	417	411	413	409	641	676	436	698	63	115	249	71	578	550	561	733	131	194	141	363	794	374	569	282	65	151	57	197	308	498	387	583
ENSG00000237483	3	0	0	0	0	1	0	5	0	1	2	0	0	4	1	0	0	0	0	3	1	1	2	1	0	0	0	0	0	2	0	0
ENSG00000243398	0	1	3	1	1	0	1	5	0	0	1	0	2	0	0	3	0	1	0	3	0	2	2	2	0	0	0	0	1	6	2	3
ENSG00000279862	3	2	1	1	2	2	0	1	0	0	0	1	2	1	2	0	1	0	0	6	2	2	3	3	1	2	0	3	4	3	4	6
ENSG00000236686	0	0	0	0	0	0	2	0	0	0	0	0	0	0	0	0	0	0	0	0	1	0	0	0	0	0	0	0	0	1	0	0
ENSG00000121853	0	0	0	0	0	0	0	0	0	1	2	0	0	0	0	0	0	0	0	0	0	0	0	0	2	1	6	5	0	0	0	2
ENSG00000121858	106	121	111	107	112	126	101	147	1400	1143	4679	766	911	1052	1216	1496	83	171	107	102	245	81	168	89	1081	1721	1351	2850	961	1098	1457	1599
ENSG00000224611	0	0	0	0	0	1	0	0	3	10	19	5	3	3	5	3	0	0	0	0	0	0	0	0	3	4	5	17	0	7	3	9
ENSG00000223387	3	11	5	9	0	0	0	1	0	1	2	0	4	3	7	16	1	0	0	5	0	0	0	0	2	4	0	2	2	9	9	7
ENSG00000224426	4	3	1	0	0	0	1	0	1	0	3	0	0	1	0	2	1	0	1	1	0	0	0	0	0	0	0	1	0	1	1	1
ENSG00000144959	228	203	257	199	261	302	182	309	212	187	967	176	263	247	402	400	213	354	325	324	688	258	491	211	308	477	204	708	304	408	467	484
ENSG00000231384	1	1	0	0	4	1	3	1	3	3	12	1	1	2	0	2	0	5	0	2	1	0	1	1	2	1	2	13	1	0	3	2
ENSG00000252082	0	0	0	1	0	1	0	1	0	0	3	0	1	0	2	2	1	0	0	0	0	3	3	2	3	0	2	4	0	1	3	0
ENSG00000114346	4253	2813	3574	2732	2825	2883	2018	2939	1445	1281	4520	931	1433	1457	1726	1891	4067	4972	4464	4104	6894	2504	4345	2828	1983	2761	566	4076	1563	1842	2047	2231
ENSG00000200355	10	10	7	6	1	0	3	1	6	1	1	1	1	1	3	8	0	0	0	2	4	1	1	1	7	3	2	9	3	3	4	1
ENSG00000201458	4	12	3	4	4	4	1	3	0	1	9	2	3	4	4	1	3	1	0	13	3	3	4	5	6	6	2	20	2	8	6	6
ENSG00000227440	12	5	8	13	19	10	10	20	10	5	16	3	7	6	10	14	7	8	9	5	12	6	16	6	6	9	3	11	7	13	6	11
ENSG00000237501	1	1	2	0	6	0	0	3	1	0	3	0	1	0	0	1	0	0	0	0	1	0	1	1	0	2	0	2	1	1	0	0
ENSG00000144962	1	4	1	0	0	2	0	3	0	0	1	0	1	0	0	0	0	0	1	2	3	0	0	4	0	0	0	0	0	0	0	0
ENSG00000237473	0	1	1	1	1	0	0	0	0	0	0	0	0	0	0	0	0	0	0	0	1	0	2	0	0	0	0	0	0	0	0	0
ENSG00000169760	1	1	0	3	2	3	1	3	2	4	11	2	0	0	0	0	1	1	0	0	1	0	0	1	3	4	5	19	0	0	0	0
ENSG00000237645	0	0	0	0	0	0	0	0	0	0	0	0	0	0	0	0	0	0	0	0	0	0	0	0	0	0	0	0	0	0	0	0
ENSG00000228213	0	0	0	0	0	0	0	0	0	0	0	0	0	0	0	0	0	0	0	0	0	0	0	0	0	0	0	0	0	0	0	0
ENSG00000252982	0	0	0	0	0	0	0	0	0	0	0	0	0	0	0	0	0	0	0	0	0	0	0	0	0	0	0	0	0	0	0	0
ENSG00000232601	0	0	0	0	0	0	0	0	0	0	0	0	0	0	0	0	0	2	0	0	0	0	0	0	0	0	1	0	0	0	0	0
ENSG00000213169	0	0	0	0	1	2	0	0	1	0	0	0	0	1	0	0	0	0	0	0	0	0	0	0	0	0	0	0	0	0	0	1
ENSG00000177694	7	5	4	6	13	4	8	5	29	24	57	7	47	43	45	75	4	4	4	5	27	11	17	8	36	33	12	83	13	49	19	32

ENSG00000253020	0	0	0	0	0	0	0	0	0	0	0	0	0	0	0	0	0	0	0	0	0	0	0	0	1	0	0	1	0	0	0	0
ENSG00000230497	0	0	0	0	0	0	0	0	0	0	0	1	0	0	0	0	0	0	0	0	0	0	0	0	0	0	0	0	0	0	0	0
ENSG00000230292	0	1	0	0	0	0	0	0	0	0	0	0	0	0	0	0	0	0	0	0	1	0	0	0	0	0	0	0	0	0	0	0
ENSG00000226779	0	0	0	0	0	0	0	0	0	0	0	0	113	69	103	118	0	0	0	0	0	0	0	0	0	0	0	0	28	79	31	80
ENSG00000264974	0	0	0	0	0	0	0	0	0	0	0	0	0	0	0	0	0	0	0	0	0	0	0	0	0	0	0	0	0	0	0	0
ENSG00000199792	0	0	0	0	0	0	0	0	0	0	0	0	0	0	0	0	0	0	0	0	0	0	0	0	0	0	0	0	0	0	0	0
ENSG00000201648	0	0	0	0	0	0	0	0	0	0	0	0	0	0	0	0	0	0	0	0	0	0	0	0	0	0	0	0	0	0	0	0
ENSG00000236241	0	0	0	0	0	0	0	0	0	0	0	0	1	0	0	0	0	0	0	0	0	0	0	0	0	0	0	0	0	0	2	0
ENSG00000214192	2	1	0	0	0	0	0	2	0	0	2	0	0	2	0	0	0	0	0	1	1	0	0	0	0	1	1	0	2	0	1	1
ENSG00000201452	0	0	0	0	0	0	0	0	0	0	0	0	0	0	0	0	0	0	0	0	0	0	0	0	0	0	0	0	0	0	0	0
ENSG00000225552	0	0	0	0	0	0	0	0	0	0	0	0	0	0	0	0	0	0	0	0	0	0	0	0	0	0	0	0	0	0	0	0
ENSG00000260217	0	0	0	0	0	0	0	0	0	0	0	0	0	0	0	0	0	0	0	0	0	0	0	0	0	0	0	0	0	0	0	0
ENSG00000230362	0	0	0	0	0	0	0	0	0	0	0	0	0	0	0	0	0	0	0	0	0	0	0	0	0	0	0	0	0	0	0	0
ENSG00000236357	0	0	0	0	0	0	0	0	0	0	0	0	0	0	0	0	0	0	0	0	0	0	0	0	0	0	0	0	0	0	0	1
ENSG00000271413	0	0	0	0	0	0	0	0	0	0	0	0	0	0	0	0	0	0	0	0	0	0	0	0	0	0	0	0	0	0	0	0
ENSG00000238026	0	0	0	0	0	0	0	0	0	0	0	0	0	0	0	0	0	0	0	0	0	0	0	0	0	0	0	0	0	0	0	0
ENSG00000283333	0	0	0	0	0	0	0	0	0	0	0	0	0	0	0	0	0	0	0	0	0	0	0	0	0	0	0	0	0	0	0	0
ENSG00000223715	0	0	0	0	0	1	1	0	0	0	0	0	2	0	0	0	0	0	0	0	2	0	1	0	0	0	0	0	0	0	0	0
ENSG00000232461	0	0	0	0	1	0	0	0	0	0	0	0	0	0	0	1	0	0	0	0	0	0	2	0	0	0	0	0	0	0	0	0
ENSG00000228308	0	0	0	0	0	0	0	0	0	0	0	0	0	0	0	0	0	1	0	0	0	0	0	0	0	0	1	0	0	0	0	2
ENSG00000231888	1	0	1	0	5	3	0	4	0	3	11	0	1	0	0	0	1	0	0	0	6	2	2	1	1	3	0	7	2	0	1	1
ENSG00000177565	6916	4518	5557	4443	7215	7025	5085	7132	4346	3654	15251	2486	5185	4435	4675	5374	3623	4563	4069	5707	13824	6029	9616	6124	4631	6368	3286	10606	3360	3856	3856	4368
ENSG00000231310	14	7	7	4	7	8	9	6	1	2	9	2	3	9	1	2	4	2	3	20	16	9	8	11	6	8	4	18	0	6	2	1
ENSG00000201343	1	3	3	1	1	1	1	6	0	3	10	1	2	1	3	4	2	1	1	7	5	2	7	3	2	7	1	9	0	2	3	5
ENSG00000200882	1	0	1	4	2	3	2	0	1	1	4	0	1	3	5	1	0	0	0	7	4	1	0	2	3	1	1	7	0	1	1	5
ENSG00000203645	1	0	0	0	0	0	0	1	0	1	0	0	1	0	0	0	0	0	0	0	2	0	0	0	0	1	0	1	0	0	0	1
ENSG00000236893	0	0	0	0	0	0	0	0	0	0	0	0	0	0	0	0	0	0	0	0	0	0	0	0	0	0	0	1	0	0	0	0
ENSG00000226782	0	0	0	0	1	0	0	0	0	0	1	0	0	0	0	0	0	0	0	0	0	0	0	0	0	0	1	0	0	1	1	0
ENSG00000228221	0	0	0	0	112	91	85	110	0	1	14	0	0	1	0	0	0	0	0	0	129	64	139	51	1	0	0	6	0	1	0	0
ENSG00000252028	1	0	0	0	0	0	0	0	0	0	0	0	0	0	0	0	0	0	0	0	0	0	0	0	0	0	0	0	0	0	0	0
ENSG00000236385	0	0	0	0	0	0	0	0	0	0	0	0	0	0	0	0	0	0	0	0	0	0	0	0	0	0	0	1	0	0	0	0
ENSG00000200288	0	0	0	0	0	0	0	0	0	0	0	0	0	0	0	0	0	0	0	0	0	0	0	0	0	0	0	0	0	0	0	0
ENSG00000228561	0	0	0	0	136	123	124	158	0	1	34	2	0	0	0	0	0	0	0	0	280	126	241	147	5	2	0	8	0	0	1	0
ENSG00000277241	0	0	0	0	12	14	11	10	0	0	2	0	0	0	0	0	0	0	0	0	23	10	7	8	0	0	0	1	0	0	0	0
ENSG00000231574	0	0	0	0	1	0	1	0	0	0	1	1	0	0	0	0	0	0	0	0	3	2	0	0	1	0	0	0	0	0	0	0
ENSG00000225790	0	0	0	0	0	1	0	0	0	0	0	0	0	0	0	0	0	0	0	0	0	0	0	0	0	0	0	0	0	0	0	0
ENSG00000270321	0	0	0	0	0	0	0	0	0	0	0	0	0	0	0	0	0	0	0	0	1	1	0	0	0	0	0	0	0	0	0	0
ENSG00000199858	0	0	0	0	0	0	0	0	0	0	0	0	0	0	0	0	0	0	0	0	0	0	0	0	0	0	0	0	0	0	0	0
ENSG00000223930	0	0	0	0	6	1	4	6	23	13	221	15	0	0	0	0	0	0	0	0	7	0	2	1	29	32	50	63	0	0	0	0
ENSG00000197584	0	0	1	1	0	0	0	0	0	0	3	1	6	6	8	5	2	3	0	0	0	0	0	0	0	0	1	2	4	5	3	5
ENSG00000275163	0	0	0	0	0	0	0	0	0	0	0	0	0	0	0	0	0	0	0	0	0	0	0	0	0	0	0	0	0	0	0	0
ENSG00000223941	0	0	0	0	0	0	0	0	0	0	0	0	0	0	0	0	0	0	0	0	1	0	0	0	0	0	0	0	0	0	0	0
ENSG00000271131	0	0	0	0	0	0	0	0	0	0	0	0	2	0	1	2	0	0	0	0	0	0	0	0	0	0	0	0	0	0	0	0
ENSG00000237978	1	1	0	1	0	0	0	0	0	1	5	0	32	34	26	47	1	0	0	1	0	0	1	0	0	0	1	4	27	35	44	38
ENSG00000172667	1442	1244	1223	1184	422	407	325	482	688	551	18405	1660	1592	1262	1521	1496	333	510	461	834	943	366	624	361	779	1081	5023	7815	1000	1224	1083	1371
ENSG00000200616	0	0	0	0	0	0	0	0	0	0	0	0	0	0	0	0	0	0	0	0	0	0	0	0	0	0	0	0	0	0	0	0
ENSG00000229102	0	0	0	0	0	0	0	0	0	0	0	0	0	0	0	0	0	0	0	0	1	0	0	0	0	0	0	0	0	0	0	1
ENSG00000121879	1851	1235	1432	1217	1139	1207	932	1187	752	666	3004	512	2051	1837	2098	2274	873	1359	1112	1366	2327	979	1528	980	837	1157	863	2159	1473	1875	1808	2321
ENSG00000201957	1	0	2	1	7	4	4	7	6	2	18	3	1	3	7	7	0	0	0	2	10	4	2	2	2	4	5	6	2	5	2	2
ENSG00000171121	4	3	1	6	489	475	375	550	16	13	84	8	32	28	28	62	4	4	6	8	1005	414	698	384	17	13	10	52	11	46	39	43
ENSG00000240429	0	0	1	0	117	166	99	145	19	10	71	14	23	14	34	35	0	0	0	0	241	117	157	129	12	21	13	40	17	37	45	38
ENSG00000121864	1385	1001	1202	913	1356	1290	999	1371	1003	814	2700	550	1463	1230	1584	1642	942	1437	1277	1478	2919	1159	2075	1123	932	1437	596	2000	1530	1630	1671	1803
ENSG00000260743	16	13	14	4	10	7	8	8	15	12	31	1	27	16	25	28	9	7	6	10	10	6	6	7	14	19	1	22	26	23	25	25
ENSG00000171109	1681	1098	1307	1101	1111	1238	936	1286	706	604	2896	400	881	716	901	944	1333	1484	1260	1347	2419	969	1576	1068	775	1057	752	1838	732	816	953	997
ENSG00000114450	1797	1682	1337	1587	1805	1842	1337	1869	1337	1137	4028	872	1289	1570	1203	1854	718	1773	928	1611	2911	1132	1997	933	1466	2207	683	3412	1226	1816	1215	1983
ENSG00000242539	0	0	0	0	0	0	0	0	0	0	0	0	0	0	0	0	0	0	0	0	0	0	0	0	0	0	0	0	0	0	0	0
ENSG00000272699	8	4	10	4	4	2	3	4	2	3	15	2	4	3	0	3	1	3	1	11	3	6	4	6	5	9	5	10	1	7	5	6
ENSG00000239255	0	0	0	0	0	0	0	0	0	0	0	0	0	0	0	0	0	0	0	0	0	0	0	0	0	0	0	0	0	0	0	0
ENSG00000181260	0	0	0	0	0	0	0	0	0	0	1	0	1	0	1	0	0	2	0	0	0	0	1	0	0	1	0	0	0	0	0	0
ENSG00000136518	3277	2162	2521	2222	2839	2766	2073	2959	2274	1687	6953	1411	3336	2961	3568	4011	3579	4770	4134	3567	7274	2814	5212	2910	2207	3311	1202	4826	3748	3678	4275	4474
ENSG00000272910	8	19	20	12	20	17	8	21	20	13	60	12	16	11	11	18	5	4	6	22	16	11	11	15	9	27	16	36	10	22	5	16
ENSG00000272660	5	4	3	7	5	8	6	12	10	6	23	3	6	4	15	11	2	0	0	1	21	6	15	5	5	3	5	18	3	7	7	7
ENSG00000136522	1197	685	986	706	1044	932	737	1051	551	461	1715	294	1046	926	1120	1131	1653	1764	1995	1781	3166	1338	2565	1852	686	955	393	1294	1548	1402	1964	1677
ENSG00000136521	1377	899	1060	868	943	1002	672	1012	1179	903	4071	651	1529	1428	1663	1925	1697	2116	1786	1274	2382	912	1710	1084	1198	1532	984	2573	1758	1651	2405	2216
ENSG00000239096	4	4	3	4	0	1	5	4	1	2	10	1	2	2	0	1	0	1	0	4	2	4	1	4	0	2	2	4	0	2	0	4
ENSG00000243977	0	1	0	0	0	0	1	0	0	0	0	0	0	2	0	0	1	0	1	0	0	0	2	0	0	1	1	0	0	0	0	1
ENSG00000058056	1532	1356	1472	1441	2484	2373	1916	2547	1294	1147	4709	927	1296	1196	1249	1615	364	672	535	1072	3910	1609	2851	1434	1369	2147	840	3903	731	1210	891	1373
ENSG00000114757	129	77	89	77	81	89	61	82	119	77	286	42	139	162	147	183	43	49	59	83	123	78	61	43	93	139	132	260	74	118	62	106
ENSG00000270178	4	1	7	0	7	3	3	2	3	3	3	0	8	2	3	5	1	0	2	2	5	3	3	7	0	0	1	5	2	2	0	5
ENSG00000243799	0	0	1	0	0	3	2	2	0	0	1	0	1	1	0	2	0	0	0	0	2	1	2	1	1	1	0	0	0	2	0	3
ENSG00000244302	0	0	0	0	2	3	0	0	2	5	6	2	3	0	3	2	0	0	0	0	1	0	0	0	1	1	0	3	0	1	0	2
ENSG00000282327	0	0	0	0	0	0	0	0	0	0	0	0	0	0	0	0	0	0	0	0	0	0	0	0	0	0	0	0	0	0	0	0
ENSG00000213158	0	0	0	0	0	0	0	0	0	0	0	0	0	2	0	0	0	0	0	0	0	0	0	0	0	0	0	0	0	2	0	3
ENSG00000239880	0	0	0	0	0	0	0	0	0	0	0	0	0	0	0	0	0	0	0	0	0	0	0	0	0	0	0	0	0	0	0	0
ENSG00000241696	0	0	0	0	0	0	0	0	0	0	0	0	0	0	0	0	0	0	0	0	0	0	0	0	0	0	0	0	0	0	0	0
ENSG00000201810	0	0	0	0	0	0	0	0	0	0	0	0	0	0	0	0	0	0	0	0	0	0	0	0	0	0	0	0	0	0	0	0
ENSG00000239774	1	1	4	2	0	0	0	0	2	0	5	0	2	3	0	0	3	0	2	4	0	0	0	0	3	1	0	4	3	0	3	0
ENSG00000199986	0	0	0	0	0	0	0	0	0	0	1	0	0	0	0	0	0	0	0	0	0	0	0	0	0	0	0	0	0	0	0	0
ENSG00000241946	0	2	0	0	0	0	0	0	0	0	0	0	0	0	0	0	0	0	0	0	0	0	0	0	0	0	0	0	0	0	1	0
ENSG00000163728	3271	2376	2565	2207	0	0	0	0	1627	1390	7588	1135	1782	1625	1577	2085	1261	1180	1299	2294	1	0	0	0	1518	2184	2029	4294	927	1694	1266	1830
ENSG00000145075	540	358	453	372	0	0	0	0	403	408	1972	265	346	335	366	412	133	138	128	443	0	1	1	0	403	553	467	981	164	402	172	370
ENSG00000243187	0	0	0	0	0	0	0	0	4	1	3	0	0	1	0	0	1	0	0	0	0	0	0	0	0	0	0	1	0	0	0	0
ENSG00000242811	0	0	0	0	0	0	0	0	0	0	0	0	0	0	0	0	0	0	0	0	0	0	0	0	0	0	0	0	0	0	0	0
ENSG00000242068	0	0	0	0	0	0	0	0	0	0	0	0	0	0	0	0	0	0	0	0	0	0	0	0	0	0	0	0	0	0	0	0
ENSG00000241473	0	0	0	0	0	0	0	0	0	0	0	0	0	0	0	0	0	0	0	0	0	0	0	0	0	0	0	0	0	0	0	0
ENSG00000239516	0	0	0	0	0	0	0	0	0	0	0	0	0	0	1	0	0	0	0	0	0	0	0	0	0	0	0	0	0	0	0	0
ENSG00000114416	5387	3403	3902	3611	4262	4829	3478	5039	2994	2298	10239	1704	4824	4316	4764	5580	4685	5350	4440	3864	10579	4028	6813	4159	2817	4188	2431	7074	4314	4338	5441	5498
ENSG00000213157	0	0	0	0	0	0	0	0	0	0	0	0	1	0	0	0	0	0	0	0	0	0	0	0	0	0	0	0	0	0	0	0
ENSG00000205981	804	520	639	514	495	595	402	601	518	409	1591	319	722	739	864	1013	1053	1241	1104	1031	1386	606	1039	625	546	683	381	1020	1000	911	1297	1229
ENSG00000242808	10	9	10	7	1	2	3	3	54	57	173	26	6	5	8	9	7	9	6	9	7	2	5	2	28	45	40	67	2	5	7	7
ENSG00000242140	0	0	0	0	1	3	0	0	0	0	0	0	0	0	0	0	1	0	0	0	0	0	0	0	0	0	0	0	0	0	0	0
ENSG00000206932	3	1	1	1	5	3	3	5	0	0	2	0	0	0	2	4	2	0	3	2	6	3	6	0	0	1	1	1	1	0	5	5
ENSG00000237806	0	0	1	0	0	0	0	0	0	1	2	1	0	0	0	0	0	0	0	0	0	0	1	0	0	0	0	0	0	1	0	0
ENSG00000241231	0	0	0	0	0	0	0	0	0	0	0	0	0	0	0	0	0	0	0	0	0	0	0	0	0	0	0	0	0	0	0	0
ENSG00000235294	0	0	0	0	0	0	0	0	0	0	0	0	0	0	0	0	0	0	0	0	0	0	0	0	0	0	0	0	0	0	0	1
ENSG00000239381	1	2	0	0	0	0	0	0	0	1	0	0	1	0	0	0	0	0	0	1	1	1	0	0	0	0	0	2	0	1	0	2
ENSG00000276074	0	0	0	0	0	0	0	0	0	0	0	0	0	0	0	0	0	0	0	0	0	0	0	0	0	0	0	0	0	0	0	0
ENSG00000276690	0	0	0	0	0	0	0	0	0	0	0	0	0	0	0	0	0	0	0	0	0	0	0	0	0	0	0	0	0	0	0	0
ENSG00000181449	1	4	0	0	0	0	0	0	598	466	1631	317	5	8	10	11	0	3	0	0	0	0	0	0	484	844	237	1148	12	16	8	28
ENSG00000278026	0	0	0	0	0	0	0	0	0	0	0	0	0	0	0	0	0	0	0	0	0	0	0	0	0	0	0	0	0	0	0	0
ENSG00000266317	0	0	0	0	0	0	0	0	0	0	0	0	0	0	0	0	0	0	0	0	0	0	0	0	0	0	0	0	0	0	0	0
ENSG00000239805	0	0	0	0	0	0	0	0	0	0	0	0	0	0	0	0	0	0	0	0	0	0	0	0	0	0	0	0	0	0	0	0
ENSG00000213155	0	0	0	0	0	0	0	0	0	0	1	0	0	0	0	0	0	0	0	0	0	0	0	0	0	0	1	0	0	0	0	0
ENSG00000242512	0	0	0	0	0	0	0	0	0	0	8	4	3	2	1	2	0	0	0	0	0	1	0	0	1	0	3	3	3	2	1	6
ENSG00000252257	0	0	0	0	0	0	0	0	0	0	0	0	0	0	0	0	0	0	0	0	0	0	0	0	0	0	0	0	0	0	0	0
ENSG00000240478	0	0	0	0	0	0	0	0	0	0	0	0	0	0	0	0	0	0	0	0	0	0	0	0	0	0	0	0	0	0	0	0
ENSG00000242012	0	0	0	0	0	0	0	0	0	0	0	0	0	0	0	0	0	0	0	0	0	0	0	0	0	0	0	0	0	0	0	0
ENSG00000241098	0	0	0	0	0	0	0	0	0	0	0	0	0	0	0	0	0	0	0	0	1	0	0	0	0	0	0	0	0	0	0	0
ENSG00000244199	0	0	0	0	0	0	0	0	0	0	0	0	0	0	0	0	0	0	0	0	0	0	0	0	0	0	0	0	0	0	0	0
ENSG00000244247	0	0	0	0	4	1	1	4	0	0	0	0	0	0	0	0	0	0	0	0	0	0	0	0	0	0	0	0	0	0	0	0
ENSG00000228766	1	0	0	0	2	0	0	0	1	0	4	0	0	2	1	4	1	1	0	1	1	0	1	0	0	0	0	3	2	2	3	3
ENSG00000238837	0	0	0	0	0	0	0	0	0	0	0	0	0	0	1	0	0	0	0	0	0	0	0	0	0	1	0	0	0	0	0	0
ENSG00000243378	0	0	0	0	0	0	0	0	0	0	0	0	0	0	0	0	0	0	0	0	0	0	0	0	0	0	0	0	1	0	0	0
ENSG00000260377	0	0	0	0	0	0	0	0	0	0	3	0	0	0	2	0	0	0	0	0	0	0	0	1	0	0	6	0	1	1	0	0
ENSG00000240063	3	2	2	6	0	0	0	0	0	0	4	0	2	4	8	7	2	4	4	5	0	0	0	0	0	1	4	0	2	5	1	4
ENSG00000058063	5320	3755	4446	3518	1	8	6	4	3192	2750	11741	1957	6194	5512	6285	6822	2211	3190	2668	3414	6	1	6	1	3203	4269	2834	7640	4744	5972	5642	7045
ENSG00000270554	6	3	4	4	0	0	0	0	11	4	16	0	9	8	13	6	2	0	0	2	0	0	0	0	11	8	9	19	4	8	7	10
ENSG00000273261	28	23	23	14	0	0	0	1	26	18	70	6	43	19	26	42	5	4	6	11	3	0	0	0	28	20	20	46	11	42	19	34
ENSG00000043093	1908	1228	1503	1198	1855	1891	1577	2043	1572	1228	6129	932	1908	1979	2002	2367	1703	2041	1778	2284	5054	1936	3449	2347	1832	2340	1439	4253	2279	2795	2766	3276
ENSG00000244346	14	11	9	5	18	9	6	10	18	8	40	5	7	7	7	6	8	6	2	14	19	14	18	18	10	13	19	62	5	17	10	13
ENSG00000078070	908	643	812	653	1692	1696	1286	1856	972	785	3041	533	1239	1052	1211	1367	624	803	775	675	2944	1166	2321	1059	754	1044	596	1791	1076	1194	1407	1561
ENSG00000243368	38	20	13	20	18	27	9	22	16	11	61	11	12	18	15	16	7	4	1	21	33	15	13	13	10	9	14	22	12	21	6	22
ENSG00000078081	416	499	409	528	82	98	42	97	58	46	243	55	822	779	717	1038	73	284	148	363	69	31	47	20	28	61	59	138	417	587	415	651
ENSG00000053524	77	103	75	76	224	245	158	214	111	91	443	73	88	117	104	116	25	36	23	119	343	152	189	182	82	150	109	363	31	80	66	98
ENSG00000176597	31	15	38	12	29	18	20	51	17	14	103	6	27	33	33	32	11	6	23	28	82	42	42	52	27	28	26	84	16	36	37	46
ENSG00000240024	87	72	84	89	499	562	411	610	344	261	1255	196	249	313	347	495	52	133	99	89	1161	531	945	465	330	414	346	753	412	449	484	541
ENSG00000201229	2	0	0	0	0	1	0	1	1	0	4	1	0	1	0	1	0	0	0	0	6	0	0	2	0	1	0	4	0	0	0	2
ENSG00000253092	1	3	7	3	8	8	7	8	13	3	29	4	5	7	4	7	1	2	0	2	15	8	9	9	3	7	5	11	4	7	4	5
ENSG00000199363	0	0	0	0	0	0	0	0	0	0	0	0	0	0	0	0	0	0	0	0	0	0	0	0	0	0	0	0	0	0	0	0
ENSG00000251730	0	0	0	0	0	0	0	2	2	0	4	0	1	0	0	1	0	2	0	1	0	3	0	0	0	1	2	4	0	1	0	0
ENSG00000271682	0	0	0	0	0	1	0	0	0	0	0	0	0	0	0	0	0	0	0	0	0	0	3	0	0	0	0	0	0	0	0	0
ENSG00000172578	1383	985	1068	908	3175	3346	2426	3427	653	445	2644	339	3560	3234	3255	4179	424	548	604	782	4746	1938	3377	1645	416	410	719	1003	1597	2069	1728	2337
ENSG00000242522	3	2	1	0	10	7	3	1	1	1	9	1	7	4	2	3	1	0	1	0	16	6	7	6	5	1	2	4	5	4	5	6
ENSG00000243584	0	0	0	0	0	0	3	0	0	1	0	0	1	0	0	1	0	0	0	0	0	0	0	0	0	0	2	1	0	0	0	0
ENSG00000114796	1846	1313	1173	1223	634	657	473	744	485	476	4461	476	954	873	561	1008	312	374	244	394	842	347	534	294	472	709	1863	2659	334	457	289	436
ENSG00000163872	4502	3782	3974	3742	4147	4089	2973	4392	2453	2068	6871	1425	3116	3135	2977	3800	726	1021	851	1838	4754	2136	3406	1741	1456	2327	1509	4348	1277	2221	1506	2328
ENSG00000233885	110	89	101	83	135	131	120	182	86	58	228	40	71	68	71	101	21	27	17	47	174	82	96	102	33	65	66	161	26	75	50	79
ENSG00000180834	120	123	109	102	51	52	36	40	37	27	125	16	50	42	44	53	63	155	93	184	109	50	73	56	26	69	34	107	32	49	56	54
ENSG00000175193	1984	1418	1632	1473	1240	1270	896	1436	917	673	2619	546	1465	1287	1627	1776	1955	2717	2519	1849	3370	1252	2570	1292	870	1250	566	1708	1866	1704	2188	2203
ENSG00000234371	0	0	1	0	1	0	0	1	0	0	0	0	0	1	0	0	1	2	2	3	2	0	1	2	0	0	0	1	0	2	0	1
ENSG00000263932	0	0	0	0	1	0	0	0	0	0	0	0	1	0	0	0	0	0	0	0	0	0	0	0	0	0	0	0	0	0	0	3
ENSG00000233441	0	0	0	0	0	0	0	0	0	0	2	0	0	0	1	0	0	0	0	0	0	0	1	0	0	0	0	0	0	0	0	0
ENSG00000114770	2203	1979	2012	1919	1320	1469	978	1466	1667	1432	7153	1082	1116	1140	1225	1608	648	700	585	1560	1928	862	1125	858	1058	1820	1445	3539	759	1698	1045	1879
ENSG00000223882	8	7	1	6	1	5	4	3	3	3	12	1	3	1	2	3	0	3	2	6	7	3	4	3	2	6	7	11	0	5	8	6
ENSG00000223529	0	0	0	2	0	0	0	0	0	0	0	0	0	0	0	1	0	1	0	1	0	1	0	0	0	2	1	0	0	0	0	0
ENSG00000186090	0	0	0	0	0	0	0	0	0	0	1	0	0	0	0	0	0	0	0	0	0	0	0	0	0	0	0	0	0	0	0	0
ENSG00000178084	0	1	0	0	0	0	0	0	0	0	0	0	1	0	0	0	0	0	0	0	0	0	0	0	0	0	0	0	0	1	0	2
ENSG00000233576	1	2	1	1	1	0	0	0	0	1	2	0	0	1	0	3	0	0	0	0	0	0	0	0	0	0	0	0	0	0	0	0
ENSG00000207351	0	2	0	0	0	0	0	0	0	0	1	0	0	1	0	0	0	0	0	0	0	0	0	0	0	0	1	1	1	0	0	0
ENSG00000238020	1	0	0	0	0	0	0	0	0	0	1	0	0	1	0	1	0	0	0	1	0	0	0	0	0	0	2	0	0	0	0	0
ENSG00000186038	0	3	1	0	0	2	0	1	0	0	0	3	0	0	0	0	0	0	0	0	0	0	1	1	1	0	0	1	0	0	0	0
ENSG00000205955	1	1	2	1	2	1	2	1	0	0	3	0	5	1	3	1	0	2	0	3	0	1	0	0	0	2	2	4	1	0	1	5
ENSG00000273181	6	5	5	7	2	5	5	2	5	2	13	1	14	10	2	8	3	9	8	3	4	0	7	1	7	4	5	6	2	8	3	7
ENSG00000272721	15	10	8	24	9	12	6	3	7	2	16	1	25	18	14	12	6	19	14	8	15	1	10	3	7	6	2	10	8	13	17	10
ENSG00000145191	2220	1274	1844	1454	2089	2057	1529	2086	1363	1116	5103	890	2591	2052	2096	3026	1133	1331	1001	1692	2376	1602	2546	1365	1077	2001	959	3089	2219	2714	2677	3025
ENSG00000228205	13	9	5	3	2	8	1	4	4	3	12	5	3	7	2	7	4	5	3	11	7	0	1	2	6	2	5	11	3	7	7	6
ENSG00000161202	2868	2252	2997	2300	2773	3002	2058	2958	1773	1631	8474	1361	1554	1610	1663	2169	723	885	1068	1582	4047	1738	2942	1520	1435	2503	1954	5027	1251	1826	1385	2119
ENSG00000161203	6720	4878	6133	5286	6275	6441	4590	6795	4174	3257	14760	2841	4172	3788	4033	4845	2227	3881	4044	4640	10447	4345	8929	3703	3731	6289	3410	9489	3867	4540	4023	4781
ENSG00000161204	1982	1510	1724	1649	1058	1174	801	1143	837	732	3030	552	1049	935	1093	1310	895	926	836	1533	1631	791	1236	612	590	1056	690	1900	784	1150	955	1332
ENSG00000145198	77	180	136	169	24	54	22	61	5	8	18	7	24	22	29	44	14	41	30	78	33	18	22	25	9	19	7	18	14	51	34	59
ENSG00000221120	0	0	0	0	0	0	0	0	0	0	0	0	0	0	0	0	0	0	0	0	0	0	0	0	0	0	0	0	0	0	0	0
ENSG00000214160	1010	788	773	827	975	976	755	991	757	588	2454	491	1383	1155	1323	1386	973	1625	967	1014	1969	698	1469	671	669	1108	423	1484	1277	1320	1244	1435
ENSG00000145194	377	350	261	332	346	353	208	371	166	117	508	92	692	562	615	709	280	692	335	418	524	227	390	169	132	254	74	300	823	671	857	698
ENSG00000163888	67	58	82	54	97	97	79	109	9	11	53	25	16	22	13	30	10	31	26	45	157	82	132	59	12	17	10	43	12	21	18	21
ENSG00000175166	8290	5474	6766	6372	6477	6952	5091	6999	3863	2964	13603	2527	6524	5550	6061	7283	2645	4568	4328	6330	11418	4886	8567	3952	4128	6807	2977	9693	5561	6606	5799	7206
ENSG00000114867	9269	7160	8826	7761	13200	14003	10142	14386	11301	8437	38764	7203	18392	16024	18467	21071	2570	4020	3667	7336	20246	9007	14078	7008	10052	17909	7590	26377	13363	16981	12707	17938
ENSG00000212158	1	0	2	1	3	1	0	1	0	2	5	0	2	1	1	2	0	0	0	2	0	5	3	2	0	1	3	3	1	1	3	7
ENSG00000175182	409	320	361	369	46	35	40	45	41	29	201	34	23	31	29	24	97	143	94	255	49	25	34	21	42	67	73	167	10	37	25	44
ENSG00000114859	377	391	425	308	276	271	224	312	133	152	560	101	322	315	388	383	108	138	108	415	430	190	306	219	101	156	134	358	185	289	236	361
ENSG00000163882	2054	1529	1596	1451	1796	1929	1296	1941	1338	974	3381	768	1524	1478	1518	1953	2057	2871	2470	2045	2631	1041	1796	1039	963	1484	645	2129	1582	1602	1578	1857
ENSG00000090534	3	1	1	2	4	0	7	2	3	0	2	2	1	1	2	1	0	0	0	3	5	1	3	1	1	0	5	6	0	3	0	1
ENSG00000090539	1	2	3	1	2	2	3	2	1	3	5	1	0	6	3	3	2	0	1	1	0	2	1	3	0	0	2	2	0	1	0	3
ENSG00000224153	0	0	0	0	1	0	0	0	0	0	0	0	0	0	0	0	0	0	0	0	0	1	0	0	0	0	0	0	0	0	0	0
ENSG00000283625	0	0	0	0	0	0	0	0	0	0	0	0	0	0	0	0	0	0	0	0	0	0	0	0	0	0	0	0	0	0	0	0
ENSG00000227509	0	0	0	0	0	0	0	0	0	0	0	0	0	0	0	0	0	0	0	0	0	0	0	0	0	0	0	0	0	0	0	0
ENSG00000230215	0	0	0	0	0	0	0	0	0	1	0	0	0	0	0	0	0	0	0	0	0	0	0	0	0	0	0	0	0	0	2	0
ENSG00000182580	0	1	0	0	0	0	0	0	13	23	42	18	0	0	0	2	0	0	0	0	0	0	0	0	3	1	18	18	0	2	1	4
ENSG00000177383	438	459	342	434	836	886	614	987	1041	849	3253	675	1578	1653	1628	2192	181	329	261	345	1415	650	1205	581	733	1178	682	2009	1319	1640	1383	1775
ENSG00000272970	0	3	1	3	4	0	2	2	1	0	5	1	4	1	4	6	1	0	2	0	5	1	6	0	0	0	1	1	4	2	0	5
ENSG00000273403	0	0	1	0	1	0	1	2	0	0	0	0	1	0	0	0	1	0	0	0	0	0	2	0	0	2	0	0	0	1	0	3
ENSG00000272922	0	0	0	3	0	0	2	2	0	0	0	0	0	0	2	0	0	0	0	0	0	0	0	0	0	2	1	0	0	0	0	3
ENSG00000229433	0	0	0	0	1	0	0	0	0	0	1	0	0	1	0	0	0	0	0	0	0	0	0	0	0	0	1	5	0	0	2	0
ENSG00000156931	1177	883	971	799	1516	1596	1259	1773	558	458	2653	378	779	739	789	956	650	865	781	1038	3150	1264	2198	1351	501	766	817	1662	584	904	779	1062
ENSG00000187068	1	0	0	0	1	0	0	0	20	27	52	27	0	0	0	0	0	0	0	0	0	0	0	0	13	48	14	119	0	0	0	2
ENSG00000223358	0	0	0	0	0	0	0	0	0	0	0	0	0	0	0	0	0	0	0	0	0	0	0	0	0	1	0	1	0	0	0	0
ENSG00000113790	0	0	0	0	380	408	288	393	113	98	439	66	27	39	32	41	0	0	0	0	645	277	528	240	103	139	121	241	20	21	12	32
ENSG00000229704	0	0	0	0	6	2	2	1	0	0	2	0	0	0	0	0	0	0	0	0	9	5	3	1	0	0	2	2	0	0	0	0
ENSG00000264614	0	0	0	0	1	0	0	0	0	0	0	0	0	0	0	0	0	0	0	0	1	2	0	1	0	0	0	0	0	0	0	0
ENSG00000073803	143	130	98	151	244	242	224	303	134	91	370	63	349	370	373	449	26	33	26	96	202	106	105	131	54	79	80	209	288	370	287	463
ENSG00000229638	46	18	30	23	30	27	20	21	38	24	103	15	29	23	32	38	52	58	32	31	51	23	31	18	30	30	15	56	25	24	33	43
ENSG00000163900	612	554	528	440	811	876	708	936	324	261	1131	216	756	738	875	987	333	582	422	488	1579	610	1239	595	284	420	308	750	662	840	783	951
ENSG00000163898	0	1	1	2	6	7	7	10	0	2	9	5	6	7	2	10	0	0	1	6	4	2	4	1	1	0	3	0	1	4	3	8
ENSG00000163904	1357	836	1114	950	1467	1391	1203	1554	652	557	2873	453	1540	1459	1597	1956	645	807	820	953	2786	1104	2213	1048	602	992	769	1735	1284	1550	1460	1737
ENSG00000236489	6	4	5	3	4	4	3	10	2	2	12	2	3	3	3	4	1	1	2	14	7	6	2	5	3	7	2	15	1	2	5	4
ENSG00000073792	4	5	1	7	1067	1067	873	1175	9	8	29	5	16	33	16	37	0	3	0	11	1294	495	942	489	7	21	16	42	2	16	7	13
ENSG00000163915	0	0	0	0	9	12	4	13	0	1	5	5	0	0	0	2	0	0	0	0	11	8	10	8	0	0	2	1	0	0	0	0
ENSG00000265470	0	0	0	0	0	0	0	0	0	0	0	0	0	0	0	0	0	0	0	0	5	0	0	0	0	0	0	0	0	0	0	0
ENSG00000136527	8140	5302	6622	5188	7368	7597	5652	7893	7322	5769	20193	4199	5989	5823	6841	7430	5834	8726	8661	8212	19301	7551	12885	7253	7205	10405	3824	14887	7096	8289	8476	9610
ENSG00000171658	1	0	1	2	1	0	2	0	0	0	4	0	3	3	7	3	0	7	2	1	0	0	0	1	0	0	0	2	2	3	7	6
ENSG00000244405	659	579	615	553	565	823	302	719	0	8	2	2	649	871	701	1087	114	180	280	274	885	494	432	418	1	8	4	4	212	530	368	671
ENSG00000277118	2	1	1	1	2	2	2	3	0	0	0	2	0	3	1	1	0	0	0	1	3	4	2	0	0	0	0	0	0	4	1	0
ENSG00000234197	2	5	1	3	9	9	1	3	0	0	1	0	2	4	3	0	1	0	0	2	23	6	3	6	0	0	0	1	0	1	2	3
ENSG00000058866	195	237	214	237	4	7	5	11	0	0	0	0	91	109	106	176	162	348	238	368	15	13	7	6	0	1	0	0	91	180	119	210
ENSG00000234548	0	0	0	0	0	0	0	0	0	0	0	0	0	0	0	0	0	0	0	0	0	0	0	0	0	0	0	0	0	0	0	0
ENSG00000224406	0	0	0	0	0	0	1	0	0	0	0	0	1	0	0	0	0	0	0	0	0	0	0	0	0	0	1	0	0	0	0	0
ENSG00000226859	0	0	0	0	0	0	0	0	0	0	4	0	0	0	0	0	0	0	0	0	0	0	0	0	0	3	5	0	0	1	1	0
ENSG00000213139	119	95	101	87	59	92	59	86	82	76	283	43	88	82	81	116	50	30	37	111	162	79	71	75	55	61	71	168	42	112	58	135
ENSG00000113838	676	518	568	502	588	596	397	618	400	347	1364	288	412	376	427	523	312	444	400	511	1047	423	700	404	423	556	337	961	374	543	414	585
ENSG00000090520	1250	765	1021	818	2063	2116	1528	2288	1128	880	4675	773	1203	1129	1470	1586	899	1158	1317	1246	4477	1791	2570	1472	1200	1928	991	2984	1507	1809	1996	2016
ENSG00000197099	2	2	3	2	21	32	18	37	3	3	19	1	37	24	27	28	0	2	4	4	14	14	9	12	4	7	6	7	8	11	9	22
ENSG00000283149	0	0	0	0	0	0	0	0	0	0	0	1	0	0	0	0	0	0	0	0	0	0	0	0	0	0	0	0	0	0	0	0
ENSG00000283390	6	3	9	6	2	2	1	8	3	2	11	4	4	6	5	9	13	5	4	6	8	0	9	8	3	5	0	7	4	6	2	12
ENSG00000145192	4	2	2	2	9	8	11	9	0	0	0	0	1	1	2	3	8	4	1	13	14	4	6	2	0	0	0	0	0	1	0	0
ENSG00000275696	0	0	0	0	0	0	0	0	0	0	0	0	0	0	0	0	0	0	0	0	0	0	0	0	0	0	0	0	0	0	0	0
ENSG00000090512	0	0	1	0	0	0	0	0	0	0	0	0	0	0	0	0	0	0	0	0	0	0	0	0	0	0	0	0	0	0	0	0
ENSG00000113905	0	0	0	0	1	0	0	2	0	0	1	0	3	1	0	2	0	1	0	0	0	0	0	0	0	0	0	0	1	1	2	1
ENSG00000225175	0	0	0	0	0	0	0	0	0	0	0	0	0	0	0	0	0	0	0	0	0	0	0	0	0	0	1	0	0	0	0	0
ENSG00000226724	0	0	0	0	0	1	0	1	0	0	0	0	3	0	1	0	0	0	0	0	0	0	0	0	0	0	0	0	0	0	0	0
ENSG00000113889	0	2	2	0	9	14	12	18	4	5	5	3	12	18	10	17	0	0	0	2	4	2	7	2	1	3	7	3	2	6	0	5
ENSG00000226652	0	0	2	0	4	2	4	2	0	0	5	0	0	7	4	4	0	0	0	0	3	0	2	4	1	0	4	0	1	2	2	2
ENSG00000207505	0	0	0	0	3	0	2	0	1	0	2	1	4	0	0	0	0	0	0	0	0	0	1	1	0	0	0	0	0	1	0	2
ENSG00000230951	3	1	2	1	3	8	2	8	0	2	4	3	2	5	4	6	0	1	0	1	12	5	4	5	0	2	1	2	2	2	2	8
ENSG00000263826	211	152	216	226	246	296	151	244	338	253	1269	182	149	142	176	209	110	88	79	262	311	132	157	153	209	367	206	659	100	217	162	237
ENSG00000156976	14215	9659	10439	9907	9791	10640	8255	11474	9840	7526	34659	5775	14192	14410	17266	19057	10459	11544	9025	11349	22844	10318	15753	11156	8720	12346	8571	19304	12593	14890	16809	18788
ENSG00000238942	0	0	0	0	0	0	0	0	0	0	0	0	0	0	0	0	0	0	0	0	0	0	0	0	0	0	0	0	0	0	0	0
ENSG00000200418	0	0	0	0	0	0	0	0	0	0	0	0	0	0	0	0	0	0	0	0	0	0	0	0	0	0	0	0	0	0	0	0
ENSG00000221420	0	0	0	0	0	0	0	0	0	0	0	0	0	0	0	0	0	0	0	0	0	0	0	0	0	0	0	0	0	0	0	0
ENSG00000200320	0	0	0	0	0	0	0	0	0	0	0	0	0	0	0	0	0	0	0	0	0	0	0	0	0	0	0	0	0	0	0	0
ENSG00000263776	0	0	0	0	0	0	0	0	0	0	0	0	0	0	0	0	0	0	0	0	0	0	0	0	0	0	0	0	0	0	0	0
ENSG00000163918	2300	1506	1907	1531	1314	1374	1199	1644	1366	1042	3269	753	1983	1800	2010	2441	2336	2803	2809	2092	3786	1419	2883	1541	1235	1597	528	2403	2055	2172	2486	2663
ENSG00000231724	4	20	5	12	4	13	6	6	0	1	6	1	11	18	13	15	6	1	4	15	16	7	6	4	0	0	0	0	3	11	2	15
ENSG00000232233	64	79	60	91	51	41	33	40	9	7	21	9	62	83	46	97	31	40	28	92	54	29	35	24	1	10	3	10	24	67	24	69
ENSG00000231982	53	55	58	66	20	38	17	40	11	12	26	7	62	66	39	64	31	44	16	96	46	20	28	16	4	18	7	15	39	55	16	57
ENSG00000181092	0	0	0	0	0	0	1	1	0	0	0	0	0	0	0	0	0	0	0	0	0	0	1	0	0	0	0	0	0	0	0	0
ENSG00000226482	0	0	0	0	2	0	1	4	0	0	0	0	0	0	0	0	0	0	0	0	1	0	3	0	0	0	0	0	0	0	0	0
ENSG00000223803	3	2	0	2	1	5	0	2	2	0	2	2	1	1	3	4	4	2	5	0	7	4	0	4	1	2	0	1	0	3	3	1
ENSG00000233565	0	0	0	0	0	0	0	0	0	0	0	0	0	0	0	0	0	0	0	0	0	0	0	0	0	0	0	0	0	0	0	0
ENSG00000073849	2131	1688	1809	1690	1831	1753	1412	1976	38	49	163	37	1953	1895	1536	2220	418	732	548	1114	2429	962	1793	800	36	64	20	162	945	1295	735	1210
ENSG00000163923	767	560	562	519	113	114	78	136	336	197	951	205	510	456	467	562	946	1073	1022	918	211	86	136	109	309	388	136	608	489	435	533	563
ENSG00000198491	0	0	0	0	0	0	0	0	0	0	2	0	0	0	0	0	0	0	0	0	2	0	0	0	0	0	0	0	0	0	0	1
ENSG00000175077	0	0	0	0	0	1	0	0	0	0	2	0	0	0	0	0	0	0	0	0	0	0	0	0	0	0	0	5	0	0	1	0
ENSG00000127241	0	0	0	0	0	0	0	0	0	0	1	0	0	0	0	2	0	0	0	0	0	0	0	0	0	0	1	2	0	0	1	0
ENSG00000283175	0	0	0	0	0	0	0	0	0	0	2	0	0	0	0	0	0	0	0	0	0	0	0	0	0	0	2	0	0	0	0	0
ENSG00000136514	0	0	0	0	0	0	1	0	209	160	905	145	210	193	211	272	0	0	0	0	0	0	0	0	183	216	217	554	232	249	265	287
ENSG00000280270	0	0	0	0	0	0	0	0	1	0	3	0	0	0	0	0	0	0	0	0	0	0	0	0	0	0	0	3	0	0	0	0
ENSG00000239093	0	0	0	0	0	0	0	0	0	0	0	0	0	0	0	0	0	0	0	0	0	0	0	0	0	0	0	0	0	0	0	0
ENSG00000228952	0	0	0	0	0	0	0	0	0	0	1	0	0	0	0	0	0	0	0	0	0	0	0	0	0	0	0	0	0	0	0	0
ENSG00000157005	0	0	0	0	0	0	0	0	0	0	0	0	0	0	0	0	0	0	0	0	0	0	0	0	0	0	0	3	0	0	0	0
ENSG00000198471	0	0	0	2	0	0	0	0	0	2	0	0	0	0	1	0	0	0	0	1	1	0	1	0	0	0	0	0	1	0	0	1
ENSG00000228804	2	1	0	0	10	14	11	19	0	0	1	0	0	0	3	1	0	0	0	0	6	5	6	7	1	0	2	2	0	2	3	0
ENSG00000113916	48	92	47	87	1274	1365	1071	1485	118	95	520	89	181	220	169	279	9	24	16	24	1137	513	986	470	94	174	167	413	63	141	68	174
ENSG00000223401	2	5	2	1	16	31	19	33	24	9	66	7	18	18	12	22	0	0	1	1	36	19	13	11	14	24	23	68	7	20	9	26
ENSG00000224049	0	0	0	0	0	0	0	0	0	0	0	0	0	0	0	0	0	0	0	0	0	0	0	0	0	0	0	0	0	0	1	0
ENSG00000236864	0	0	0	0	0	1	0	0	0	0	0	0	0	0	0	0	0	0	0	0	0	1	0	0	0	0	0	0	0	0	0	0
ENSG00000224187	0	0	1	0	2	1	0	0	0	0	3	0	0	0	0	0	0	0	0	0	5	0	1	1	0	1	0	1	0	1	1	1
ENSG00000225058	0	0	0	0	0	0	0	0	2	0	0	0	0	0	0	0	0	0	0	0	3	0	0	0	0	0	0	1	0	1	0	1
ENSG00000236412	0	0	0	0	1	0	0	0	0	0	0	0	0	1	0	0	0	0	0	0	0	0	0	0	0	1	0	0	0	0	0	0
ENSG00000234238	0	0	0	0	0	0	0	0	0	0	0	0	0	0	0	0	0	0	0	0	1	1	0	0	0	0	0	0	0	0	0	0
ENSG00000270959	10	6	30	12	48	75	53	65	52	47	249	41	14	23	20	32	4	12	12	23	81	32	66	29	32	56	64	157	11	36	31	44
ENSG00000145012	2672	1912	2273	1952	2114	2283	1305	2316	533	500	2432	388	1455	1554	1609	1882	435	588	677	1365	2534	1307	1568	912	520	793	598	1839	711	1215	1179	1536
ENSG00000279891	16	2	8	5	15	11	8	17	5	3	20	4	10	7	3	10	4	1	4	11	30	23	13	20	6	9	3	27	4	9	13	16
ENSG00000224563	0	0	5	0	0	1	0	1	0	0	2	2	0	1	1	0	0	0	0	2	1	0	0	1	0	0	0	1	0	0	1	0
ENSG00000207651	0	0	0	0	0	0	0	0	0	0	0	0	0	0	0	0	0	0	0	0	1	0	0	0	0	0	0	1	0	0	0	0

ENSG00000234076	0	0	0	0	0	0	0	0	0	0	1	0	0	0	1	0	0	0	0	0	0	0	0	0	0	0	1	0	0	0	0	0
ENSG00000188001	1	0	0	4	1	0	2	2	34	22	208	19	153	132	172	206	1	0	2	1	4	1	11	8	50	43	47	106	183	196	221	251
ENSG00000236182	0	0	0	0	0	0	0	0	0	0	0	0	1	4	5	4	0	0	0	0	0	0	0	0	0	0	0	0	0	2	1	3
ENSG00000230115	0	0	0	0	0	0	0	0	1	0	1	0	0	5	1	1	0	0	0	0	1	0	1	0	0	0	0	0	1	0	0	1
ENSG00000073282	0	0	0	0	148	116	78	113	0	0	4	0	1249	1116	1177	1414	0	0	0	0	103	46	67	45	2	1	3	4	865	1182	1088	1375
ENSG00000227971	0	0	0	0	0	1	0	2	0	0	1	0	2	1	1	1	0	0	0	0	0	0	0	0	0	0	0	0	2	5	3	5
ENSG00000216058	0	0	0	0	0	0	0	0	0	0	0	0	0	1	1	0	0	0	0	0	0	1	0	0	0	0	0	0	2	6	2	4
ENSG00000090530	0	0	0	0	0	0	0	0	1	2	2	0	71	149	118	199	0	0	0	0	0	1	0	1	1	1	2	9	87	218	145	319
ENSG00000230077	0	0	0	0	0	0	0	0	0	0	0	0	0	0	1	0	0	0	0	0	0	0	0	0	0	0	0	0	0	0	0	3
ENSG00000225764	0	0	0	0	0	0	0	0	0	0	1	0	1	2	0	0	0	0	0	0	0	0	0	0	0	0	1	1	0	0	0	0
ENSG00000212489	0	0	0	0	0	0	0	0	0	0	0	0	0	0	0	0	0	0	0	0	0	0	0	0	0	0	0	0	0	0	0	0
ENSG00000238077	0	0	0	0	0	0	0	0	0	0	0	0	0	0	0	0	0	0	0	0	0	1	0	0	0	0	0	0	0	0	0	0
ENSG00000163347	0	0	0	0	0	0	0	0	0	1	21	3	1007	1015	1244	1395	0	0	0	0	0	0	0	2	0	0	0	10	1161	1770	1279	1825
ENSG00000113946	0	0	0	0	4	12	3	8	0	0	0	0	91	73	139	94	0	0	0	0	6	8	7	6	0	0	0	1	52	47	84	69
ENSG00000198398	0	0	0	0	0	0	0	0	0	0	0	0	0	0	0	0	0	0	0	0	0	0	0	0	0	0	0	0	0	1	0	0
ENSG00000196083	542	420	451	380	1227	1685	1030	1806	127	121	510	97	241	257	296	346	282	442	546	538	2944	1636	2251	1340	184	233	146	507	309	328	397	406
ENSG00000231047	0	2	1	3	2	2	10	6	3	0	3	0	0	1	7	0	0	0	0	3	6	6	8	7	0	0	0	5	0	2	0	1
ENSG00000223117	4	0	4	1	8	10	7	12	3	2	8	0	1	1	3	4	1	0	0	1	9	7	4	10	0	0	1	7	1	5	4	2
ENSG00000273370	0	0	0	0	0	0	0	0	0	0	0	0	0	0	0	0	0	0	0	0	0	0	0	0	0	0	0	0	0	0	0	0
ENSG00000228730	0	0	0	0	0	0	0	0	0	0	0	0	0	0	0	0	0	0	0	0	0	0	0	0	0	0	0	0	0	0	0	0
ENSG00000232610	0	0	0	0	0	0	0	0	0	0	0	0	0	0	0	0	0	0	0	0	0	0	0	0	0	0	0	0	0	0	0	0
ENSG00000214147	0	0	0	0	0	0	0	0	2	0	0	0	0	0	0	0	0	0	0	0	0	0	0	0	0	0	0	0	0	0	0	0
ENSG00000205835	0	0	0	0	0	0	0	0	0	0	0	0	3	1	0	1	0	0	0	0	0	0	0	0	0	0	0	0	0	1	0	1
ENSG00000188729	0	0	0	0	0	1	0	0	0	0	0	0	0	0	0	0	0	0	0	0	0	0	0	0	0	0	0	0	0	0	0	0
ENSG00000233308	0	0	0	0	0	0	0	0	0	0	0	0	1	1	0	1	0	0	0	0	0	0	0	0	0	0	0	0	0	1	0	1
ENSG00000188958	0	0	0	0	0	0	0	0	4	0	8	0	0	3	4	5	0	0	0	0	0	0	0	0	1	0	2	8	1	3	3	7
ENSG00000152492	0	1	0	1	0	0	0	0	1727	1497	6794	1175	3447	3347	3926	4240	0	0	0	0	0	0	0	0	1634	2466	1529	4546	2759	3265	3330	3894
ENSG00000253548	0	0	0	0	0	0	0	0	0	0	0	0	0	0	0	0	0	0	0	0	0	0	0	0	0	0	0	0	0	0	0	0
ENSG00000223812	0	0	0	0	0	0	0	0	0	0	0	0	0	0	0	0	0	0	0	0	0	0	0	0	0	0	0	0	0	0	0	0
ENSG00000201860	0	0	0	0	0	0	0	0	0	0	0	0	0	0	0	0	0	0	0	0	0	0	0	0	0	0	0	0	0	0	0	0
ENSG00000237653	0	0	0	0	0	0	0	0	0	0	0	0	0	0	0	0	0	0	0	0	0	0	0	0	0	0	0	0	0	0	0	0
ENSG00000232353	0	0	0	0	0	0	0	0	0	0	3	0	0	0	0	0	0	0	0	0	0	0	0	0	0	0	0	0	0	0	0	0
ENSG00000222583	0	0	0	0	0	0	0	0	0	0	0	0	0	0	0	0	0	0	0	0	0	0	0	0	0	0	0	0	0	0	0	0
ENSG00000114279	0	4	0	0	0	1	0	1	5	7	15	9	4	9	6	5	0	2	0	4	0	0	0	1	5	8	5	6	1	0	2	2
ENSG00000231383	0	0	0	0	0	0	0	0	0	0	0	0	0	0	0	0	0	0	0	0	0	0	0	0	0	0	0	0	0	0	0	0
ENSG00000230126	0	0	0	0	0	0	0	0	0	0	0	0	0	0	0	0	0	0	0	0	0	0	0	0	0	0	0	0	0	0	0	0
ENSG00000226709	0	0	0	0	0	0	0	0	0	0	0	0	0	0	0	0	0	0	0	0	0	0	0	0	0	0	0	0	1	0	0	0
ENSG00000238902	0	0	0	0	0	0	0	0	0	0	1	0	0	0	0	0	0	0	0	0	1	0	0	0	0	0	0	0	0	0	0	0
ENSG00000200184	0	0	0	0	0	0	0	0	0	0	0	0	0	0	0	0	0	0	0	0	0	0	0	0	0	0	0	0	0	0	0	1
ENSG00000180611	45	82	54	91	145	152	99	157	628	552	2639	473	205	244	224	329	31	85	51	106	341	110	243	114	538	916	716	1778	202	310	193	335
ENSG00000232130	0	0	0	0	0	0	0	0	0	0	0	0	0	0	0	0	0	0	0	0	0	0	0	0	0	0	0	0	0	0	0	0
ENSG00000236229	0	0	0	0	1	0	1	0	0	0	0	0	0	0	0	0	0	0	0	0	0	0	0	0	0	0	0	0	0	0	0	1
ENSG00000127252	0	0	1	0	0	0	0	0	0	0	0	0	0	0	0	0	0	0	0	0	0	0	0	0	0	0	0	0	0	0	0	0
ENSG00000187527	1	1	0	3	2	3	1	3	0	0	4	0	1	0	1	3	0	0	0	2	2	0	3	1	1	0	1	2	2	0	1	1
ENSG00000236508	0	0	0	0	0	0	0	0	0	0	0	0	0	0	0	0	0	0	0	0	0	0	0	0	0	0	0	0	0	0	0	0
ENSG00000127249	93	76	72	80	69	53	46	74	87	49	186	42	74	77	80	100	17	27	11	28	61	35	38	24	28	44	51	139	41	69	44	52
ENSG00000225473	0	0	3	1	1	1	3	2	2	1	1	1	2	3	2	2	0	0	0	1	0	0	1	2	0	1	1	1	0	0	0	2
ENSG00000236297	18	13	32	14	24	24	30	48	27	26	106	10	9	10	8	11	12	12	11	10	37	19	45	13	24	39	18	53	15	14	11	22
ENSG00000198836	2714	1912	2309	1983	3189	3301	2621	3682	2515	1885	8497	1567	5455	4366	5206	5589	1787	2354	2257	2473	7755	3057	5775	3144	2613	3764	1862	5849	4393	4780	5526	5736
ENSG00000224855	22	10	7	10	8	14	9	9	6	10	33	11	16	14	17	18	4	2	2	13	12	16	24	12	17	22	9	44	4	17	16	26
ENSG00000207370	2	6	8	9	3	8	9	7	6	5	20	4	7	14	12	7	0	1	0	6	9	5	6	10	6	4	9	22	6	16	12	10
ENSG00000243991	0	0	0	0	0	0	0	0	0	0	0	0	0	0	1	0	0	1	0	0	0	0	0	0	0	0	0	0	0	0	1	0
ENSG00000223986	0	0	0	0	0	0	0	0	0	0	0	0	0	0	3	0	0	0	0	0	0	0	0	0	0	0	0	0	0	0	0	0
ENSG00000229155	0	0	0	0	0	0	0	2	0	0	0	0	0	0	0	0	0	0	0	0	0	0	0	0	0	0	0	0	0	0	0	0
ENSG00000214146	0	0	0	0	5	3	1	2	0	0	0	0	0	0	0	0	0	0	0	1	1	4	4	5	0	0	0	0	0	0	0	0
ENSG00000238043	0	0	0	0	0	2	0	0	0	0	0	0	0	0	0	0	0	0	0	0	1	0	2	0	0	0	0	0	0	0	0	0
ENSG00000224403	0	0	0	0	0	0	0	0	0	0	0	0	0	0	0	0	0	0	0	0	0	0	1	0	0	0	0	0	0	0	0	0
ENSG00000230102	0	3	1	0	2	4	4	3	0	0	0	0	0	3	1	2	0	1	0	4	5	0	5	2	0	0	0	0	0	1	0	1
ENSG00000276407	0	1	0	0	0	1	0	0	0	0	0	0	0	0	0	0	0	0	1	0	0	0	0	0	0	0	0	0	0	0	0	0
ENSG00000229964	0	0	0	0	0	0	0	0	0	0	0	0	0	0	0	0	0	0	0	0	1	0	0	0	0	0	0	0	0	0	0	0
ENSG00000232874	4	18	22	22	23	21	11	16	0	0	0	0	5	7	14	7	1	6	4	19	23	10	5	4	0	0	0	0	0	5	1	0
ENSG00000114315	163	213	216	211	48	64	49	55	0	0	0	0	454	576	614	772	85	263	221	276	102	40	58	37	0	0	2	0	570	735	549	769
ENSG00000242201	0	0	0	0	0	0	0	0	0	0	0	0	0	0	0	0	0	0	0	0	0	0	0	0	0	0	0	0	0	0	0	0
ENSG00000225742	0	0	1	0	22	25	15	28	0	0	0	0	7	7	6	10	3	4	1	1	47	20	21	20	0	0	0	0	3	4	8	9
ENSG00000238097	0	2	1	0	4	1	1	3	0	0	0	0	6	2	6	2	0	2	3	2	9	3	2	3	0	0	0	0	0	2	7	5
ENSG00000228271	0	1	1	0	1	2	0	1	0	0	0	0	0	0	0	0	0	0	0	0	0	0	0	1	0	0	0	0	1	2	1	1
ENSG00000214145	6	4	4	5	9	12	7	15	0	0	1	0	11	8	10	14	2	0	3	5	12	16	3	1	0	1	0	0	16	15	14	19
ENSG00000178772	0	0	0	0	0	0	0	0	0	0	0	0	0	0	0	0	0	0	0	0	0	0	0	0	0	0	0	0	0	0	0	0
ENSG00000172061	0	0	0	0	1	0	0	0	0	0	0	0	0	0	0	0	0	0	0	0	1	1	1	0	0	0	0	0	0	0	0	1
ENSG00000178732	22	26	31	28	246	289	164	262	4	4	6	0	3	4	1	2	4	7	14	31	362	165	293	149	4	10	2	3	0	0	2	0
ENSG00000133657	5504	4142	4519	4050	4495	4667	3558	4811	3310	2940	13158	2169	8039	7371	8486	9295	2274	3199	2825	5286	9838	4116	6662	4125	3969	5822	2903	9849	6428	9005	7446	10108
ENSG00000271483	0	1	1	0	2	2	3	1	1	1	3	0	5	2	2	1	2	1	0	5	10	6	2	3	2	7	1	16	1	5	5	2
ENSG00000233058	32	25	36	37	240	264	216	266	5	6	9	2	285	244	293	343	8	13	11	30	297	141	191	166	1	1	1	10	96	179	109	196
ENSG00000244675	14	2	12	2	102	110	81	112	1	0	6	1	115	114	73	138	1	0	9	7	111	69	62	61	1	0	1	7	27	77	31	70
ENSG00000252053	0	0	0	0	0	0	0	0	0	0	0	0	0	0	1	0	0	0	0	0	0	0	0	0	0	0	0	0	0	0	0	0
ENSG00000232873	0	0	0	0	0	0	0	0	0	0	0	0	0	0	0	0	0	0	0	2	0	0	0	0	0	0	0	0	0	0	0	0
ENSG00000231770	74	70	84	93	88	74	59	52	97	62	397	84	99	100	118	144	70	99	90	110	107	52	72	53	64	84	89	214	94	99	172	169
ENSG00000145014	920	663	849	619	1218	1247	941	1243	249	225	723	153	265	312	248	374	264	256	279	703	1899	839	1113	754	178	233	144	436	114	271	145	251
ENSG00000229334	13	14	28	7	12	22	18	30	10	16	46	5	6	8	10	19	10	9	8	11	42	14	21	27	6	10	6	25	8	16	15	18
ENSG00000272707	2	6	6	4	1	1	6	6	2	1	7	2	1	6	2	4	1	4	3	1	4	3	5	2	3	1	3	6	1	3	2	0
ENSG00000041802	1296	892	1227	861	1906	1820	1459	1889	1146	943	4410	699	1805	1541	2098	2083	720	1040	955	1413	3976	1559	2773	1339	1227	1868	1041	2873	1958	2289	2021	2507
ENSG00000185112	3	3	1	0	0	5	0	1	99	111	905	138	315	405	381	632	1	2	1	2	3	0	3	2	120	183	461	499	418	624	505	595
ENSG00000233987	0	0	0	0	0	1	0	0	0	0	0	0	0	0	0	0	0	0	0	0	0	0	0	0	0	0	0	0	0	0	0	0
ENSG00000237222	8	5	1	1	9	2	3	5	0	0	2	2	1	2	1	1	2	3	0	4	8	3	7	4	0	0	0	3	1	1	1	1
ENSG00000234805	0	0	0	0	0	0	0	1	0	0	0	1	0	0	0	0	0	0	0	0	0	0	0	0	0	0	0	0	0	0	0	0
ENSG00000230401	0	0	0	0	0	0	1	1	0	0	9	0	0	0	0	0	0	0	0	0	1	1	1	0	0	0	0	1	0	0	0	2
ENSG00000238031	0	0	0	0	0	0	0	0	0	0	4	0	0	0	0	0	0	0	0	0	0	0	1	0	0	0	0	1	0	0	0	0
ENSG00000173950	578	420	485	413	1115	1108	895	1140	538	411	1912	376	855	835	815	1062	193	359	272	487	1916	845	1444	750	538	850	482	1384	722	982	695	1007
ENSG00000233303	8	2	0	4	3	9	2	6	3	0	6	0	5	4	5	2	0	0	0	5	10	4	9	4	5	2	1	12	3	10	11	13
ENSG00000265333	0	0	1	2	1	5	3	6	0	1	4	0	1	0	0	4	0	0	1	1	7	7	3	4	2	1	0	8	2	2	6	4
ENSG00000244314	0	0	3	0	9	5	8	6	0	2	8	0	3	3	5	4	0	1	0	1	13	8	10	6	4	7	3	4	2	2	5	7
ENSG00000230266	4	2	5	3	22	33	34	40	2	4	14	1	8	6	6	5	3	0	3	3	66	26	38	49	10	0	6	17	3	14	9	13
ENSG00000206600	0	0	0	0	1	0	1	1	0	2	1	0	2	3	0	0	0	0	0	0	0	0	0	0	2	1	1	1	0	0	0	1
ENSG00000114331	3199	2284	2616	2280	2000	2260	1551	2306	1773	1708	8552	1429	2385	2238	2354	2666	1001	1387	1508	2144	4364	1834	3149	1782	2219	3137	2295	6561	1667	2303	2067	2381
ENSG00000229325	78	54	72	52	42	45	45	50	42	42	227	25	21	29	33	27	10	16	11	35	75	44	53	35	53	51	54	134	5	29	18	28
ENSG00000227433	2	2	4	2	0	1	0	1	0	2	4	0	0	1	3	2	1	0	0	2	2	2	1	2	4	2	2	12	0	0	3	2
ENSG00000207368	2	2	2	0	0	6	3	0	3	3	11	1	2	0	2	1	0	0	1	2	8	3	1	2	3	3	7	9	1	2	4	2
ENSG00000184203	1226	833	988	769	864	889	665	928	1037	864	4488	674	1377	1208	1483	1601	779	1366	1117	876	2155	803	1470	755	1045	1480	1356	2921	1522	1510	1968	1829
ENSG00000252620	11	11	11	5	4	7	2	7	9	6	34	4	6	9	7	6	0	3	1	15	9	2	5	3	2	11	11	19	5	4	4	3
ENSG00000223711	3	5	2	1	0	1	1	2	3	1	17	1	0	2	5	2	1	2	3	1	2	0	2	0	0	0	6	16	4	0	1	2
ENSG00000265882	0	0	0	0	0	0	0	0	0	0	0	0	0	0	0	0	0	1	0	0	0	1	0	0	0	0	0	0	0	0	1	0
ENSG00000236844	2	1	0	0	2	0	0	1	0	0	6	0	1	0	0	0	0	0	1	0	0	0	0	0	0	1	1	0	0	1	0	0
ENSG00000236425	0	1	0	0	0	1	0	0	1	0	0	0	0	0	0	0	0	0	0	0	0	0	0	0	0	0	0	0	1	0	0	0
ENSG00000189058	0	1	0	0	0	0	2	0	11	1	61	10	61	98	110	114	0	0	0	0	0	0	0	0	4	4	15	18	26	73	49	81
ENSG00000224769	15	8	12	25	1	4	4	3	14	17	123	12	1	2	0	1	2	11	2	20	5	1	3	2	13	12	47	51	0	0	0	0
ENSG00000282206	26	21	16	45	21	20	14	20	15	11	28	7	14	15	20	18	6	2	4	6	18	8	9	6	5	5	8	15	5	15	8	22
ENSG00000271662	35	42	52	44	15	25	8	28	28	32	122	14	9	13	12	22	4	14	5	41	29	14	17	15	14	38	35	57	4	11	3	8
ENSG00000229178	25	24	15	21	11	14	11	16	14	13	33	7	13	9	7	7	12	10	16	23	22	4	15	17	11	17	14	27	2	5	2	7
ENSG00000242086	1134	1003	1063	1038	1093	1266	1005	1316	1667	1331	6639	987	432	426	443	568	292	325	279	1004	1953	933	1352	935	1122	1768	1631	4283	212	608	301	633
ENSG00000215837	0	0	0	0	0	0	0	0	0	0	0	0	0	0	0	0	0	0	0	0	0	0	0	0	0	0	0	0	0	0	0	0
ENSG00000207650	45	34	35	31	33	28	36	38	74	68	288	41	16	20	15	22	8	6	6	23	54	28	25	28	46	64	73	124	9	17	9	14
ENSG00000283426	0	0	0	0	0	0	0	0	0	0	0	0	0	0	0	0	0	0	0	0	0	0	0	0	0	0	0	0	0	0	0	0
ENSG00000176945	21	29	22	26	25	31	31	48	23	26	198	19	35	47	31	45	2	13	8	31	56	23	42	36	22	32	48	76	13	57	16	63
ENSG00000145113	12	11	16	5	2	2	3	13	9	5	35	4	6	3	3	8	1	0	4	11	13	13	2	12	5	7	4	25	1	5	5	6
ENSG00000223783	0	0	0	0	0	2	1	0	0	0	1	0	0	1	0	1	0	0	0	1	3	2	2	2	0	0	6	1	2	1	0	0
ENSG00000235836	0	0	0	0	0	0	0	1	0	0	0	0	0	0	0	0	0	0	0	0	1	0	0	0	0	0	0	0	0	0	0	0
ENSG00000061938	1008	1108	1072	1172	1344	1724	1079	1527	1635	1229	5567	891	1166	1190	1130	1380	202	291	230	805	2042	1078	1330	1099	1283	2108	1713	3769	608	1161	694	1270
ENSG00000276489	0	0	0	0	0	0	0	1	0	0	1	0	0	0	0	0	0	0	0	0	1	0	0	0	0	0	2	0	0	0	0	2
ENSG00000224614	19	24	32	26	41	42	39	50	45	19	116	9	14	19	25	19	11	4	0	23	67	21	36	40	33	46	38	108	13	16	18	33
ENSG00000226155	0	1	5	0	0	1	1	2	3	1	7	2	1	1	1	2	1	0	0	1	0	2	0	0	2	2	6	5	0	4	0	4
ENSG00000273009	1	1	7	2	0	1	2	3	0	2	5	0	1	1	0	0	0	0	0	5	0	0	0	0	0	2	2	6	0	0	0	3
ENSG00000239122	6	3	2	9	2	6	2	2	0	1	8	0	3	3	2	6	2	1	1	5	3	3	3	2	2	0	0	8	0	2	0	3
ENSG00000231443	61	55	71	57	33	37	28	50	28	26	100	17	46	56	34	48	9	13	9	40	26	11	16	19	10	20	18	32	8	25	15	34
ENSG00000260261	289	240	278	217	183	234	148	212	115	122	455	67	215	167	161	211	32	43	44	143	210	93	85	101	80	113	85	233	47	121	62	141
ENSG00000185485	819	643	704	718	493	524	436	535	652	553	2488	437	248	251	222	276	195	285	171	546	711	400	514	388	505	831	580	1695	131	255	155	277
ENSG00000231464	5	10	4	11	3	5	5	5	5	11	11	0	0	0	0	0	0	0	1	8	5	0	2	1	0	0	2	5	0	0	0	2
ENSG00000072274	6824	4595	6674	4706	51731	48175	37300	51786	8840	6787	19034	4725	23516	19478	21965	25235	5572	8909	8746	9898	110465	43063	75609	35292	7979	11171	4075	16877	18726	20902	20118	23718
ENSG00000228413	4	5	0	3	6	10	6	7	3	0	16	7	0	4	6	5	1	5	0	3	12	7	13	7	1	4	0	3	1	4	0	3
ENSG00000252174	8	1	4	1	16	18	11	12	2	0	1	1	3	12	1	3	8	3	1	9	11	9	11	11	5	2	3	10	1	15	7	5
ENSG00000224652	0	0	0	0	2	1	0	1	0	0	3	0	228	172	209	269	0	0	0	0	1	1	0	0	0	0	2	0	140	160	149	188
ENSG00000163958	0	0	0	2	1	3	2	2	0	0	1	0	0	0	1	0	0	1	0	0	0	0	0	0	0	0	0	0	0	0	0	0
ENSG00000222335	0	0	0	0	0	0	0	0	0	0	1	0	0	0	0	0	0	0	0	1	0	0	0	0	0	0	1	0	0	0	0	0
ENSG00000163959	34	34	34	26	45	37	35	56	29	24	144	23	29	18	25	29	21	12	7	41	91	59	76	51	26	35	55	97	19	17	10	43
ENSG00000161217	983	678	899	703	1046	1185	911	1222	963	736	4042	632	501	484	592	608	493	637	585	831	2316	1046	1781	865	1015	1481	1061	2799	477	559	568	634
ENSG00000272741	3	0	3	5	0	1	0	0	0	0	4	0	1	1	0	0	0	1	1	1	1	0	0	0	2	3	0	4	0	0	0	0
ENSG00000228028	2	5	5	8	2	6	2	8	3	4	14	6	5	4	1	0	3	0	0	2	7	3	6	6	5	7	1	4	1	1	3	1
ENSG00000213123	380	310	329	308	62	68	39	88	211	143	564	91	55	54	51	69	404	594	549	509	111	65	85	54	131	181	85	321	67	55	74	103
ENSG00000237418	10	6	8	5	2	1	1	6	16	13	33	7	0	3	4	3	3	3	0	12	6	2	1	3	8	12	12	20	4	1	1	3
ENSG00000273331	8	6	9	6	1	5	5	7	8	5	33	6	1	3	3	3	7	0	1	6	6	5	9	6	1	7	3	20	2	5	0	2
ENSG00000235897	18	10	15	13	5	16	1	6	18	12	35	9	15	14	9	25	7	6	8	12	13	7	7	2	2	2	11	23	2	10	2	12
ENSG00000145107	3	1	2	0	3	0	3	0	2	3	10	0	1	5	1	11	1	0	0	4	5	1	3	0	1	0	9	1	4	1	1	0
ENSG00000212146	0	0	0	0	0	0	0	0	0	0	0	0	16	14	26	46	0	0	0	0	0	0	0	1	0	1	0	0	8	15	18	27
ENSG00000163960	3852	2841	3294	2883	2639	2620	2118	2847	1844	1431	6575	1072	3095	2832	2744	3520	808	1020	921	1906	4053	1671	2907	1428	1576	2159	1648	4079	1585	2241	1808	2483
ENSG00000206644	5	1	0	0	0	0	0	1	1	1	1	1	0	0	1	0	0	0	0	0	1	0	0	0	0	0	0	1	0	0	2	0
ENSG00000241868	2	6	1	1	0	2	0	5	3	0	7	0	3	0	2	6	0	0	0	2	4	2	1	3	1	5	0	2	0	7	1	2
ENSG00000243339	7	0	3	4	0	2	5	6	1	5	8	5	2	3	6	1	2	5	1	2	4	6	5	0	3	5	2	10	0	5	1	2
ENSG00000226345	0	1	3	3	2	1	0	1	0	0	4	0	1	2	1	0	1	0	0	1	3	1	4	3	0	4	0	7	0	0	0	1
ENSG00000225822	11	5	9	4	9	5	7	9	10	12	35	5	16	7	15	12	7	2	8	8	22	7	7	6	3	10	12	17	1	8	9	7
ENSG00000225240	3	1	0	0	3	0	0	6	0	0	5	1	0	0	1	0	1	0	0	1	3	3	0	0	0	1	0	0	0	0	0	1
ENSG00000163961	9729	5790	6063	5460	15611	15341	11313	16198	1047	880	3977	743	2338	2260	1961	2870	3158	2874	1801	3481	25118	10232	17138	8793	928	1339	718	3037	1126	1762	1218	1770
ENSG00000273013	24	25	30	21	102	102	75	116	7	7	36	3	6	11	17	18	9	9	2	11	105	44	63	52	6	4	3	18	3	14	1	10
ENSG00000214097	9	3	5	8	8	4	1	1	5	1	4	1	0	1	0	0	2	1	4	4	13	6	5	2	2	0	0	0	0	0	0	1
ENSG00000213358	0	1	1	0	1	0	0	1	0	0	0	0	3	0	0	0	1	1	1	0	0	0	3	0	0	0	4	1	0	1	0	0
ENSG00000225770	3	1	3	1	0	0	0	1	0	0	2	1	1	0	1	1	0	0	0	0	0	0	0	0	0	0	1	2	0	0	1	2
ENSG00000185798	534	329	469	369	241	299	223	324	219	171	797	175	352	272	377	399	378	441	451	466	567	253	518	245	211	303	138	552	346	386	482	478
ENSG00000174013	2560	1746	2077	1688	1171	1195	1055	1288	1164	943	3935	720	1989	1802	2088	2122	1073	1613	1459	1890	2241	985	1794	868	1262	1767	772	2798	1661	1872	1573	1905
ENSG00000232065	6	6	5	3	6	6	7	4	3	0	3	2	3	5	0	5	3	2	3	3	9	3	1	11	0	0	2	0	2	5	0	4
ENSG00000174004	1670	1114	1709	974	7053	6731	4823	6693	1174	965	2600	677	2310	1876	1982	2406	562	890	886	1455	9946	3899	6959	3340	989	1590	257	1710	1799	1976	1401	1898
ENSG00000163964	1696	1208	1404	1175	480	503	376	554	1028	890	3225	649	828	803	701	951	1050	1712	1471	1338	999	425	750	392	953	1391	728	2138	657	709	678	758
ENSG00000174007	230	149	222	160	49	78	54	77	100	78	289	51	140	138	203	191	133	117	188	159	158	69	134	66	84	119	86	189	161	158	184	174
ENSG00000201441	1	1	5	0	0	0	1	1	1	3	3	0	1	0	0	0	0	1	2	0	1	0	0	1	1	1	0	2	0	5	0	2
ENSG00000180370	11939	7514	9526	7356	5712	5764	4216	5811	4107	3364	14913	2425	6508	5488	6565	6871	2297	3340	3363	4862	9543	3732	6427	3350	4298	6484	3468	10307	4467	5596	4773	5549
ENSG00000272359	15	9	22	13	5	8	4	11	4	15	36	2	8	10	16	9	3	16	5	7	11	6	2	1	9	8	8	20	7	8	9	11
ENSG00000206892	2	0	0	1	1	0	2	0	1	2	0	0	0	2	1	1	0	0	2	1	1	0	1	0	0	0	1	2	0	1	1	0
ENSG00000119231	2757	2081	2357	1956	1797	1825	1362	1881	1145	986	5388	822	2072	1836	2253	2382	964	1233	1234	1792	3455	1322	2439	1365	1270	1830	1313	3477	1502	1967	1908	2167
ENSG00000230732	78	55	82	57	37	41	35	39	39	47	207	16	65	42	49	50	16	14	13	82	84	34	39	38	37	61	43	126	18	44	40	48
ENSG00000114503	4294	2945	3236	3133	2329	2613	1666	2680	2184	1699	7122	1260	3042	2924	3105	4038	3818	5555	4002	4097	4564	1796	3081	1771	1927	2959	1697	4577	2521	3157	2703	3609
ENSG00000225578	16	15	9	5	16	9	6	20	15	11	24	4	9	9	16	16	7	3	2	15	29	9	20	4	5	4	9	14	2	13	4	24
ENSG00000270170	448	288	358	337	312	338	180	347	395	284	1270	239	598	587	648	786	364	418	373	354	676	238	512	255	371	499	279	809	756	705	761	762
ENSG00000119227	355	337	322	281	37	65	26	54	86	68	264	48	21	21	11	19	52	86	37	164	48	20	38	17	39	70	60	182	2	13	2	16
ENSG00000233487	26	18	21	18	8	14	3	10	0	0	0	3	0	1	0	0	3	3	4	19	19	7	5	7	0	0	0	1	3	1	0	0
ENSG00000163975	820	830	816	846	455	543	358	546	492	517	995	362	340	440	293	545	241	302	210	657	613	327	475	316	290	581	126	955	120	297	93	292
ENSG00000228109	433	404	413	448	223	276	225	261	92	66	125	38	105	109	58	126	188	159	179	285	326	149	221	144	20	47	12	91	29	53	25	56
ENSG00000075711	2890	2126	2386	1966	2244	2341	1741	2482	1191	1116	4648	790	2018	1846	2054	2415	1052	1500	1390	2089	5634	2265	3852	2175	1249	1898	1032	3330	1419	1974	1587	2230
ENSG00000265850	0	1	0	1	0	1	0	0	0	0	1	0	0	1	1	0	0	0	0	0	3	1	1	1	0	0	0	0	0	0	0	0
ENSG00000227375	1	4	2	1	9	4	4	6	0	1	3	0	2	2	3	7	0	0	2	6	14	4	3	1	0	0	2	3	1	5	3	1
ENSG00000235126	11	18	26	25	0	2	0	0	0	0	0	0	0	0	0	0	9	16	17	19	1	0	0	0	0	0	0	0	0	0	0	2
ENSG00000229912	0	0	0	0	0	1	0	0	0	0	0	0	0	1	0	0	0	0	0	0	0	3	0	0	0	0	0	0	0	0	1	0
ENSG00000237167	0	0	0	0	0	0	0	0	0	0	0	0	0	0	0	1	0	0	0	0	0	1	0	0	0	0	0	0	0	0	0	0
ENSG00000272989	4	6	5	5	55	43	46	59	89	63	337	62	10	12	5	14	1	6	13	7	73	34	46	27	65	118	67	208	14	16	8	14
ENSG00000161267	986	615	794	590	1878	1987	1323	2009	2533	1928	7712	1492	1309	1188	1403	1707	425	479	496	876	2992	1354	2068	1292	1817	2915	1544	4880	1169	1548	1258	1670
ENSG00000214135	986	700	884	719	702	714	592	795	774	634	2400	465	424	407	374	490	339	347	337	634	1032	435	595	468	558	854	497	1539	202	432	210	522
ENSG00000213519	0	0	4	2	3	2	1	5	2	0	6	1	3	6	2	2	0	2	0	2	3	1	2	1	0	3	1	2	0	1	0	1
ENSG00000249626	83	71	99	75	27	32	31	45	70	57	285	19	41	19	23	55	21	13	29	49	30	13	14	16	24	34	50	90	10	24	12	35
ENSG00000273308	64	70	66	67	22	33	30	47	31	28	140	18	24	29	19	30	6	10	13	56	33	23	17	20	15	19	41	73	4	19	8	27
ENSG00000236833	7	1	7	6	7	7	4	7	4	1	3	0	0	0	0	1	0	0	2	9	17	10	13	4	0	0	4	5	0	1	0	1
ENSG00000145016	1694	1370	1726	1312	835	984	636	991	930	836	4204	734	738	670	754	902	429	566	565	1124	1194	527	803	475	831	1562	1043	3150	514	722	536	844
ENSG00000122068	3214	2317	2765	2286	3758	3944	2898	4186	1874	1585	9235	1557	2116	2154	2387	2701	2567	3786	3830	3190	10062	3919	7101	3894	2315	3569	2352	6447	2650	3020	3237	3460
ENSG00000273375	6	0	2	1	9	6	2	5	2	0	6	4	4	8	2	2	1	0	0	2	3	5	6	1	1	5	1	3	3	3	5	4
ENSG00000186001	2090	1611	1755	1578	1307	1459	1071	1569	1343	1195	5278	884	1103	1169	1122	1343	545	562	574	1070	2156	926	1504	818	989	1762	1337	3428	751	1144	832	1196
ENSG00000234136	7	6	3	6	6	5	0	4	3	2	10	2	6	6	3	5	0	2	3	3	8	5	9	3	3	8	4	16	0	5	1	7
ENSG00000234742	20	13	16	14	6	17	14	9	6	8	43	6	9	10	12	8	5	2	3	10	17	11	10	10	4	16	6	30	3	8	6	16
ENSG00000114473	961	546	757	533	187	219	117	254	59	74	260	43	231	214	261	306	720	673	761	546	290	111	213	106	44	60	71	184	240	249	263	291
ENSG00000199306	5	2	1	4	0	3	3	1	1	0	4	2	0	2	0	0	0	0	0	0	0	1	1	2	1	0	0	2	0	1	1	0
ENSG00000182899	6742	4774	4365	4631	4162	4596	2746	4655	8215	5684	23279	4811	8878	8372	9461	11443	6635	8111	7088	6531	10363	4148	7521	4880	6471	9377	5058	14356	11642	10205	13806	13425
ENSG00000185621	921	611	749	629	125	118	97	148	93	120	244	66	631	486	650	643	274	331	286	565	218	130	164	128	45	102	73	222	308	501	477	614
ENSG00000201622	1	1	0	0	0	0	0	0	0	0	0	1	1	0	1	0	0	0	0	0	0	0	0	0	0	0	0	0	0	0	0	0
ENSG00000232832	4	7	15	9	0	0	0	0	2	0	3	7	5	5	4	13	3	4	2	6	1	2	0	0	0	1	0	4	1	6	4	10
ENSG00000226435	157	147	119	131	0	3	2	0	57	65	166	53	58	67	75	91	58	91	62	187	1	1	0	1	32	40	35	143	39	85	73	108
ENSG00000212297	13	4	10	13	0	0	0	0	2	4	5	1	7	7	2	3	4	2	1	12	0	0	0	0	3	3	0	8	1	4	2	5
ENSG00000232783	0	0	0	1	0	0	0	0	0	0	0	0	0	0	0	0	0	1	0	0	2	0	1	2	0	0	0	0	0	0	0	0
ENSG00000213113	0	0	0	1	0	0	0	0	0	0	1	0	0	0	0	0	0	0	0	0	0	0	0	0	0	0	0	0	0	0	0	0
ENSG00000236246	0	0	0	0	0	0	0	0	0	0	0	0	0	0	0	0	0	0	0	0	0	0	1	0	0	0	0	0	0	0	0	0
ENSG00000236438	5	5	9	3	2	9	3	10	9	9	27	3	89	76	88	91	1	0	0	1	9	6	7	8	7	13	9	19	35	72	37	77
ENSG00000222792	0	0	0	0	0	0	0	0	0	0	0	0	0	0	0	0	0	0	0	0	0	0	0	0	0	0	0	0	0	0	1	1
ENSG00000230304	0	0	0	0	0	0	0	0	0	3	0	3	0	0	1	0	0	0	0	0	0	0	0	0	0	2	1	2	0	0	1	0
ENSG00000226008	0	0	0	0	0	0	0	0	0	0	1	0	0	0	0	0	0	0	0	0	0	0	0	0	0	0	0	0	0	0	0	0
ENSG00000248302	0	0	0	0	5	3	8	9	0	0	0	0	0	0	5	0	0	0	0	0	9	6	2	2	0	0	0	0	0	0	0	0
ENSG00000272602	35	17	18	17	368	328	273	456	0	0	0	0	5	4	1	4	18	10	12	10	820	299	586	277	0	0	1	1	1	4	3	2
ENSG00000250238	1	0	0	0	0	0	0	0	0	0	0	0	0	0	0	0	0	0	0	0	0	0	0	0	0	0	0	0	0	0	0	0
ENSG00000250312	6	1	8	5	329	336	305	384	0	0	1	0	207	206	210	224	2	2	5	3	703	266	526	286	0	0	0	0	146	210	246	254
ENSG00000275426	1	0	0	0	8	8	4	14	0	0	0	0	18	19	20	18	1	0	0	0	20	8	13	7	0	0	0	0	4	18	6	18
ENSG00000250892	0	0	0	0	30	20	34	30	0	0	0	0	16	14	23	17	0	0	0	0	52	13	26	16	0	0	0	0	4	9	18	33
ENSG00000198155	0	2	1	0	81	93	59	94	0	0	0	0	51	51	62	55	4	0	1	1	84	37	86	38	0	0	0	0	20	32	37	41
ENSG00000251188	0	0	0	5	10	2	3	10	0	0	0	0	2	1	1	2	0	0	0	0	7	5	1	1	0	0	0	0	0	3	0	2
ENSG00000251689	0	0	0	0	1	3	1	3	0	0	0	0	4	0	2	1	0	0	0	0	0	4	1	0	0	0	0	2	0	0	0	0
ENSG00000186777	0	0	0	0	18	8	6	17	0	0	0	0	2	2	2	7	0	0	0	0	5	2	4	4	0	1	1	0	1	6	2	3
ENSG00000251535	0	0	0	1	0	0	0	0	0	0	0	0	3	0	3	2	0	0	0	0	0	0	0	0	0	0	0	0	0	4	0	0
ENSG00000131127	30	16	36	21	0	1	0	0	0	0	2	0	771	746	835	868	5	3	14	9	0	0	0	0	0	1	0	1	443	722	634	769
ENSG00000248564	0	0	3	0	0	0	0	0	0	0	0	0	1	2	13	5	0	0	0	1	0	0	0	0	0	0	0	0	2	6	6	5
ENSG00000250321	4	2	1	3	0	0	1	2	3	0	8	1	3	7	3	10	6	7	9	0	5	1	5	1	2	2	3	1	1	2	5	3
ENSG00000207642	1	0	0	0	0	0	0	0	0	0	0	0	2	1	1	2	0	0	0	0	0	0	0	0	0	0	0	0	1	4	2	2
ENSG00000281016	5	5	2	3	9	11	5	27	10	12	67	3	16	17	22	16	0	0	1	4	18	12	10	10	20	26	22	52	5	14	20	27
ENSG00000249025	0	0	0	1	1	0	0	0	0	0	0	0	0	0	0	1	0	0	0	0	0	0	0	0	0	0	0	0	0	0	0	0
ENSG00000272885	1	1	3	2	12	12	12	17	5	3	13	1	10	13	14	12	0	0	0	1	10	2	15	4	1	4	3	13	4	9	11	8
ENSG00000251595	10	10	10	11	47	51	35	63	51	43	153	22	108	63	92	106	3	4	12	8	106	48	68	44	44	47	52	131	81	104	121	124
ENSG00000182903	774	398	665	477	730	799	556	909	690	575	3001	447	1233	1031	1280	1268	418	368	519	543	1521	622	933	795	690	1007	878	2021	758	969	1192	1178
ENSG00000174227	1409	922	1387	1015	1150	1330	858	1373	1206	1024	5174	808	1742	1507	1678	1897	517	437	495	1034	2084	962	1356	869	997	1682	1275	3083	1031	1749	1219	2002
ENSG00000273238	4	1	4	5	0	0	0	0	0	0	1	0	0	0	0	0	2	0	0	1	0	0	1	0	0	0	0	0	0	0	0	0
ENSG00000283183	44	21	71	21	0	0	0	0	0	0	0	0	0	0	0	0	5	21	19	12	0	0	0	0	0	0	0	1	0	0	0	0
ENSG00000133256	443	353	444	360	375	360	288	379	10	5	40	6	7	4	14	5	219	275	277	373	435	202	377	176	14	14	16	23	1	11	6	10
ENSG00000242686	39	48	63	72	13	21	29	33	0	0	0	0	0	0	0	0	6	5	11	40	24	11	11	13	0	0	0	0	0	0	0	0
ENSG00000272927	55	45	70	39	73	95	67	98	0	0	0	0	0	0	0	0	14	15	21	41	79	44	73	63	0	0	0	0	0	0	0	0
ENSG00000169020	972	637	864	615	718	681	434	746	967	593	2776	520	1000	880	1138	1220	1952	1654	2287	1658	2120	784	1546	1188	853	1275	648	1964	1656	1365	1977	2040
ENSG00000215375	290	353	338	330	126	127	92	144	235	182	835	144	211	170	163	255	169	131	119	355	225	116	154	120	119	172	230	536	105	162	90	176
ENSG00000169026	12	15	16	23	10	11	12	16	23	7	75	10	7	13	2	9	5	5	5	27	14	10	5	7	12	29	37	63	4	11	4	7
ENSG00000185619	2416	1782	2111	1837	1551	1699	1200	1638	1688	1433	6646	997	1782	1761	1761	2243	794	995	1058	1839	2571	1105	1582	1045	1228	1876	1675	3843	1039	1713	1063	2061
ENSG00000248416	16	10	21	6	4	10	15	18	24	9	42	5	8	3	1	6	2	1	2	15	8	4	4	17	10	15	13	33	0	6	6	10
ENSG00000272588	20	20	26	16	20	36	22	45	37	26	142	12	8	8	14	18	3	5	0	25	32	12	20	29	24	44	36	108	4	17	6	20
ENSG00000249592	217	197	246	160	154	142	127	178	210	151	716	130	132	125	107	181	74	59	58	140	163	89	75	71	150	256	163	419	63	121	65	136
ENSG00000233799	13	15	13	8	15	29	14	21	16	11	68	10	7	13	7	12	5	0	3	17	25	14	5	19	10	20	13	45	5	9	11	16
ENSG00000168993	393	259	440	313	2	1	0	0	1	3	4	1	17	25	26	32	42	47	62	80	1	2	2	1	2	1	0	4	22	29	19	43
ENSG00000260262	1	1	0	0	1	0	0	2	0	0	10	0	0	0	1	1	0	0	1	2	0	0	0	0	1	1	3	10	0	1	0	1
ENSG00000178950	2832	2467	2792	2676	1577	1982	1336	2048	2243	1675	11184	1725	2370	2079	1979	2539	644	834	785	1774	2463	1183	1617	1121	1820	3153	2614	7063	1599	2233	1490	2474
ENSG00000127419	254	218	332	251	215	267	202	272	379	362	2290	367	235	211	178	286	115	83	122	238	329	205	263	190	294	546	586	1453	151	294	141	310
ENSG00000145214	1153	1043	1073	964	475	650	398	646	936	877	5187	850	781	633	609	793	299	319	254	763	710	410	460	418	899	1508	1089	3112	422	614	375	695
ENSG00000145217	150	145	211	161	87	120	73	109	53	66	387	60	81	91	78	138	49	62	35	187	174	109	129	101	50	68	96	247	26	105	48	101
ENSG00000127415	279	358	359	377	262	343	223	290	217	212	1580	209	241	249	202	316	95	91	64	272	369	228	229	156	105	206	410	704	67	223	59	207
ENSG00000127418	540	546	579	537	825	739	553	897	681	584	3483	568	604	675	554	964	122	269	256	434	1146	424	731	403	471	950	1018	1862	318	550	242	605
ENSG00000250259	0	0	0	0	1	0	0	2	4	0	5	0	2	0	0	0	0	0	0	0	0	0	0	0	3	1	13	10	0	0	0	1
ENSG00000178222	1113	690	897	774	0	0	0	0	2	0	0	0	44	51	33	54	633	671	599	843	0	1	0	0	0	0	0	2	21	34	16	40
ENSG00000251639	29	32	36	28	0	0	0	1	0	0	0	0	2	0	2	2	14	18	4	19	0	0	0	0	0	0	0	0	0	1	0	0
ENSG00000251652	45	42	24	17	0	0	0	0	0	0	0	0	3	4	2	3	14	11	22	28	0	0	0	0	0	0	0	0	0	0	1	4
ENSG00000215367	79	47	80	35	0	0	0	0	0	0	0	0	6	2	3	4	18	13	18	63	0	0	0	0	0	0	0	0	3	3	3	4
ENSG00000212458	17	15	20	10	0	0	0	0	0	0	0	0	2	1	1	0	3	4	1	16	0	0	0	0	0	0	0	0	0	0	0	0
ENSG00000227189	49	54	96	68	0	0	0	0	0	0	0	0	9	6	3	5	21	9	13	61	0	0	0	0	0	0	0	0	0	7	1	4
ENSG00000159674	3251	2128	2329	2151	578	730	469	743	12	10	16	4	760	889	513	1044	857	859	641	1056	594	308	367	221	6	12	6	34	162	246	153	283
ENSG00000273179	41	40	40	37	13	13	15	20	0	0	0	0	21	18	16	32	12	11	10	36	11	4	5	3	0	0	0	0	0	8	6	10
ENSG00000280927	191	149	182	169	75	101	69	106	104	88	481	57	62	68	91	106	70	72	73	232	164	62	77	87	75	117	101	246	54	131	57	126
ENSG00000159692	5153	3790	4683	3979	3154	3236	2364	3153	2566	2094	9783	1725	3308	2975	2827	3768	1847	2668	2383	3505	5217	2145	3588	1897	2302	3807	2321	6438	2425	3102	2124	3315
ENSG00000196810	766	550	752	583	322	360	251	361	353	298	1050	194	462	537	471	634	185	239	271	471	528	226	338	192	288	382	272	786	266	437	275	448
ENSG00000090316	1582	1119	1499	1137	1149	1256	841	1262	1301	991	5407	924	1687	1470	1664	2030	1098	1271	1300	1274	2476	1063	1801	861	1328	2024	1276	3594	1742	2089	1784	2221
ENSG00000163945	1004	1016	1131	1110	833	934	701	1029	473	376	1878	258	752	609	551	802	212	195	211	668	1099	563	631	543	285	532	476	1114	346	577	336	648

ENSG00000254094	10	17	12	21	6	8	2	8	5	2	12	1	3	3	4	3	3	3	2	9	5	8	2	8	3	5	1	18	0	1	6	4
ENSG00000253399	11	6	5	10	2	12	2	13	4	1	12	0	0	1	1	4	7	0	2	4	14	4	3	6	4	5	1	12	2	5	1	4
ENSG00000179979	171	130	222	147	281	327	259	302	171	136	768	100	269	215	207	249	48	31	43	155	376	188	200	204	101	158	224	439	99	216	93	206
ENSG00000235608	0	0	0	0	0	0	0	0	0	0	0	0	0	0	0	0	0	0	0	0	0	0	0	0	0	0	0	0	0	0	0	0
ENSG00000272783	0	0	0	0	0	0	0	0	0	0	0	0	0	0	0	0	0	0	0	0	0	0	0	0	0	0	0	0	0	0	0	0
ENSG00000244459	7	6	3	5	33	48	28	47	7	6	48	11	4	10	3	12	0	4	4	0	73	18	33	19	3	9	12	14	2	6	4	1
ENSG00000174137	26	28	41	48	15	31	19	21	16	19	73	10	19	24	19	41	18	28	29	36	41	25	40	24	7	8	11	43	8	19	9	19
ENSG00000207009	0	1	1	1	0	1	0	0	1	0	0	0	2	0	0	1	0	0	0	0	1	0	0	2	0	0	0	3	0	0	1	0
ENSG00000163950	3925	2721	3366	2497	1497	1418	1284	1560	2314	1865	7553	1488	3475	3023	3610	3944	3689	4865	4906	4046	4059	1511	2828	1581	2547	3554	1379	6226	3626	3602	4151	4427
ENSG00000270195	114	118	103	90	50	66	51	51	72	62	269	58	69	73	75	97	42	44	53	56	56	26	33	30	39	78	36	138	33	53	31	86
ENSG00000168936	738	654	712	703	791	835	571	786	897	783	3806	698	806	725	706	1014	253	330	283	521	1249	601	1050	476	854	1411	794	2124	510	778	454	796
ENSG00000013810	5522	4123	5019	4536	3252	3193	2228	3345	3774	3025	11920	2645	3359	3114	3699	4247	1371	1842	1888	3168	4322	1907	3093	1671	3567	5690	1524	9409	2654	3486	2711	3855
ENSG00000068078	429	544	326	499	95	119	90	137	258	367	3344	781	640	886	525	1157	70	116	55	191	222	95	123	105	252	662	385	3040	600	1337	438	1424
ENSG00000168924	3893	2859	3227	3111	1713	1577	1242	1610	1746	1311	7549	1143	3695	3056	3648	4101	1303	1832	1432	2899	2588	1218	1861	1107	1775	3053	1478	4697	3162	3713	2851	4093
ENSG00000109685	12708	9977	11624	10450	6160	6253	4782	6689	7563	6128	21878	4798	6728	6083	6348	7864	2763	3377	3322	6960	8559	3641	6017	3389	6275	10354	3304	18118	3936	6003	3947	6758
ENSG00000249784	12	13	14	11	9	11	6	5	3	3	11	2	10	4	5	13	9	1	5	13	12	9	12	12	8	5	1	25	11	7	1	12
ENSG00000185049	1857	1309	1443	1338	866	961	680	973	960	710	2540	502	1498	1413	1405	1797	499	661	648	1064	1305	591	914	545	665	1118	514	1795	1022	1190	992	1330
ENSG00000243449	569	388	508	479	99	116	75	89	226	204	732	177	209	226	221	211	520	914	757	796	204	77	145	59	191	339	192	547	264	258	294	238
ENSG00000185818	1033	1052	926	1136	669	710	434	721	816	862	2474	649	695	745	684	1062	363	520	418	1036	1209	557	730	445	454	1062	399	1431	238	454	342	586
ENSG00000130997	49	51	60	49	7	9	5	10	53	41	148	24	51	36	38	78	14	28	17	54	15	6	5	5	26	33	36	76	28	69	32	67
ENSG00000249006	0	0	1	2	0	1	0	0	3	1	2	0	0	0	0	1	0	1	0	0	0	0	0	0	1	2	0	0	1	2	1	2
ENSG00000250623	0	0	1	3	1	0	0	0	0	0	4	0	1	0	0	1	0	0	0	2	0	0	0	0	1	0	0	3	0	1	1	3
ENSG00000214367	1309	874	1084	944	631	715	551	741	940	817	2705	611	968	940	981	1139	629	845	851	1058	1278	552	896	539	743	1101	524	2041	657	981	792	1158
ENSG00000249047	12	9	3	5	1	4	0	2	3	1	9	2	7	5	4	5	4	3	1	3	5	4	1	1	4	1	3	3	0	3	5	7
ENSG00000123933	750	608	710	621	1074	1157	873	1292	350	386	4702	486	488	516	314	695	287	426	385	528	2299	1154	1866	1002	347	527	1919	2333	326	356	284	444
ENSG00000159733	324	341	286	376	8	9	7	5	21	20	38	17	96	102	88	125	90	144	80	280	13	3	5	1	3	6	15	18	12	48	30	36
ENSG00000251148	8	8	4	8	1	0	2	1	0	0	0	0	1	0	0	2	0	4	3	5	1	1	0	0	0	0	0	0	0	1	0	0
ENSG00000239247	3	1	3	3	0	0	0	0	0	0	1	0	0	2	0	3	0	0	0	4	0	0	2	0	0	1	0	0	0	0	0	2
ENSG00000249077	1	3	3	2	0	0	0	0	0	0	0	0	0	1	3	3	0	0	0	3	0	0	0	0	0	1	0	1	0	0	0	0
ENSG00000206113	2	10	20	11	3	1	4	4	0	1	7	3	42	38	43	49	0	2	6	10	3	1	1	2	0	2	1	2	17	28	20	40
ENSG00000063978	2599	1829	2325	1796	1944	2007	1453	2010	1849	1398	6025	1199	2155	1888	2405	2544	1265	1573	1706	1638	3629	1375	2442	1182	1785	2880	1240	4440	1970	2257	2251	2513
ENSG00000248399	5	4	6	4	1	0	0	2	0	0	1	0	9	5	4	7	0	3	0	2	0	2	0	3	1	0	1	2	2	7	5	6
ENSG00000251229	60	34	27	28	21	22	24	38	42	24	124	17	23	27	16	31	9	6	5	50	79	18	36	27	30	42	10	77	16	26	12	57
ENSG00000125386	1170	910	1231	1056	1083	1137	855	1258	1082	815	4306	656	803	776	949	985	259	282	312	773	1745	777	1187	700	764	1268	968	2899	566	821	702	946
ENSG00000248155	2	0	3	1	2	0	0	1	4	0	6	0	1	1	0	2	1	1	0	0	5	3	2	2	1	4	0	3	0	1	4	4
ENSG00000206738	1	0	0	0	2	0	0	2	0	1	3	0	0	1	0	0	0	0	0	0	2	1	0	3	2	0	3	6	0	3	0	2
ENSG00000168884	892	623	736	690	601	628	439	588	687	551	2572	528	893	925	898	1127	435	597	566	770	925	412	629	322	633	1081	513	1638	893	958	827	1089
ENSG00000087266	1200	977	1254	1001	1222	1354	962	1352	1550	1324	8385	1285	1522	1450	1699	1820	483	509	476	1301	2328	968	1506	1024	1550	2702	1790	4873	1037	1535	1195	1801
ENSG00000087274	4621	3587	4340	3746	2738	2889	2017	2940	5169	4365	21048	3288	2851	2747	2837	3583	992	1438	1513	2420	3748	1678	2693	1404	4219	6882	4898	12931	2059	3001	1863	3146
ENSG00000109736	1137	814	1104	876	906	934	634	1045	1449	1223	4566	894	779	636	636	888	466	468	393	796	1101	595	831	575	822	1378	801	2136	290	457	340	559
ENSG00000249673	455	343	445	341	145	147	115	182	315	290	1550	248	231	177	221	216	174	195	216	352	267	136	200	130	276	448	331	1111	134	273	201	273
ENSG00000087269	3014	2066	2651	2236	1672	1720	1441	1844	2413	1943	7957	1472	4374	3802	4246	4893	1307	1892	1720	3011	3613	1459	2833	1272	2227	3691	1643	5814	3663	4430	3683	4656
ENSG00000125388	273	248	328	266	165	186	137	186	75	76	405	50	98	87	81	132	83	132	123	273	298	115	215	113	79	94	76	210	64	122	93	107
ENSG00000199335	0	0	0	0	2	2	2	2	0	0	0	0	0	0	0	0	0	0	0	0	3	1	0	0	0	0	0	0	0	0	0	0
ENSG00000251075	0	0	0	0	2	0	0	0	1	1	0	1	0	0	0	0	0	0	1	1	2	0	0	0	0	0	0	2	0	0	0	0
ENSG00000278778	0	0	0	0	0	1	0	0	0	0	0	0	0	0	0	0	0	0	0	0	1	0	0	1	0	0	0	0	0	0	0	0
ENSG00000274693	0	0	0	0	0	0	0	0	0	0	0	0	0	0	0	0	0	0	0	1	1	0	0	0	0	0	0	0	0	0	0	0
ENSG00000197386	3812	3362	3919	3469	2544	2851	2010	2959	2641	2407	11161	1835	2969	2814	2514	3503	697	1035	1106	2268	3544	1611	2558	1416	2190	3864	2486	7737	1500	2451	1491	2822
ENSG00000188981	8	5	7	8	7	13	5	23	7	9	81	2	22	16	21	36	1	4	7	19	10	14	8	9	6	13	13	40	13	18	31	29
ENSG00000159788	1512	1683	1649	1788	1375	1529	909	1504	282	332	1787	301	147	238	171	330	235	413	438	1031	1661	844	1043	747	150	290	394	1095	46	165	73	206
ENSG00000248840	4	6	9	4	10	3	5	11	2	1	9	0	1	1	0	1	1	0	1	8	9	8	5	8	0	4	0	7	0	1	0	1
ENSG00000248843	3	4	4	4	3	2	3	5	1	1	1	0	0	1	1	2	0	0	0	3	2	8	3	7	0	3	0	4	0	0	1	0
ENSG00000109758	0	5	2	3	8	15	2	16	0	2	15	6	0	0	2	1	0	0	0	1	15	7	10	8	0	4	0	3	1	0	1	0
ENSG00000175920	1	3	3	5	16	18	17	30	138	89	485	126	42	59	67	67	5	2	0	3	32	34	23	26	99	154	56	327	47	71	76	78
ENSG00000261643	34	31	30	28	13	12	20	20	18	19	92	12	20	15	27	18	8	7	4	22	36	15	15	11	18	24	25	61	8	17	21	33
ENSG00000163956	1867	1344	1514	1509	2180	2267	1404	2304	1607	1387	6442	1208	1591	1493	1360	1994	575	903	757	1460	2919	1253	2095	1017	1283	2396	1288	4035	1371	1726	1161	1711
ENSG00000280230	1	0	0	0	0	0	0	0	0	0	0	0	0	0	0	0	1	0	0	0	0	1	0	0	0	0	1	0	0	0	0	1
ENSG00000270090	0	0	1	0	0	0	0	0	1	3	5	0	0	0	0	0	0	0	0	0	0	0	0	0	1	4	1	3	0	0	0	0
ENSG00000216560	0	0	0	0	0	0	0	0	0	0	0	0	0	0	0	0	0	0	0	0	0	0	0	0	0	0	0	0	0	0	0	0
ENSG00000250681	0	0	0	0	0	0	0	0	0	0	0	0	0	0	0	0	0	0	0	0	0	0	0	0	0	0	0	0	0	0	0	0
ENSG00000250632	0	0	0	0	0	0	0	0	0	0	0	0	0	0	0	0	0	0	0	0	0	0	0	0	0	0	0	0	0	0	0	0
ENSG00000250986	0	0	0	0	0	0	0	0	0	0	0	0	0	0	0	0	0	0	0	0	0	0	0	0	0	0	0	1	0	0	0	0
ENSG00000184160	0	0	0	0	0	0	0	0	0	4	2	0	0	0	0	0	0	0	0	0	0	0	0	0	0	0	0	2	0	0	0	0
ENSG00000251166	0	0	0	0	0	0	0	0	0	0	0	0	0	0	0	0	0	0	0	0	0	0	0	0	0	0	0	0	0	0	0	0
ENSG00000234386	0	0	0	0	0	0	0	0	0	0	0	0	0	0	0	0	0	0	0	0	1	0	0	0	0	0	0	0	0	0	0	0
ENSG00000249522	0	0	0	0	0	0	0	0	0	0	0	0	0	0	0	0	0	0	0	0	0	0	0	0	0	0	0	0	0	0	0	0
ENSG00000253917	3	1	1	2	0	0	1	0	0	0	0	0	2	0	0	0	0	1	1	0	0	0	0	0	0	0	0	0	0	0	0	0
ENSG00000271977	5	1	0	3	0	0	0	0	0	0	2	0	0	0	0	0	0	1	0	1	1	0	0	0	0	0	0	0	0	0	0	0
ENSG00000251271	24	13	34	21	11	10	4	7	3	0	4	2	6	5	1	12	9	12	2	7	14	1	7	2	3	2	1	5	3	10	1	3
ENSG00000251669	164	134	151	111	64	87	58	86	97	68	231	47	215	181	167	259	111	169	135	196	141	54	121	69	96	119	34	153	142	232	133	187
ENSG00000250476	0	1	1	1	0	0	0	0	0	1	0	0	0	0	0	2	0	0	0	0	0	0	0	0	0	0	0	0	0	0	0	0
ENSG00000243894	0	3	2	1	2	1	2	1	0	0	0	0	0	1	0	0	0	0	0	3	2	0	1	0	0	0	0	0	0	0	0	0
ENSG00000250940	2	3	3	3	1	4	1	4	0	0	0	0	0	1	0	0	0	2	0	4	1	0	0	0	0	0	0	1	1	1	0	0
ENSG00000248669	0	0	0	0	0	0	0	0	0	0	0	0	0	0	0	0	0	0	0	0	0	0	0	0	0	0	0	0	0	0	0	0
ENSG00000198787	0	0	0	0	0	0	0	0	0	0	0	0	0	0	0	0	0	0	0	0	0	0	0	0	0	0	0	0	0	0	0	0
ENSG00000250381	0	0	0	0	0	0	0	0	0	0	0	0	0	0	0	0	0	0	0	0	0	0	0	0	0	0	0	0	0	0	0	0
ENSG00000250710	0	0	0	0	0	0	0	0	0	0	0	0	0	0	0	0	0	0	0	0	0	0	0	0	0	0	0	0	0	0	0	0
ENSG00000249844	0	0	0	0	0	0	0	0	0	0	0	0	0	0	0	0	0	1	0	0	0	0	0	0	0	0	0	0	0	0	0	0
ENSG00000163982	0	0	2	0	0	0	0	0	0	0	0	0	0	0	0	0	0	0	0	0	0	0	0	0	0	0	0	0	0	0	0	0
ENSG00000132406	445	335	377	335	425	456	323	415	375	298	1530	234	615	548	696	703	500	589	564	526	1224	445	779	505	341	489	372	965	596	618	675	812
ENSG00000145220	1743	1072	1469	1299	2189	2135	1905	2370	1304	822	2714	567	3247	2520	3623	3492	3040	3722	2990	2439	5599	2109	4402	2173	1260	1699	401	2000	4604	3917	5301	4683
ENSG00000168826	298	161	277	190	125	128	97	127	94	76	373	68	180	110	188	183	147	187	206	201	268	109	200	116	70	165	101	280	147	153	155	152
ENSG00000248516	2	0	1	0	0	0	0	1	0	2	1	1	2	1	2	0	0	1	0	0	3	0	1	0	0	0	0	0	0	0	2	0
ENSG00000168824	11	22	10	13	329	372	224	351	265	273	931	218	5	0	8	7	0	11	3	9	473	184	266	150	154	238	236	634	2	8	10	11
ENSG00000168818	884	564	742	622	482	549	418	574	532	451	2199	369	871	738	993	1039	445	615	598	770	947	435	766	420	577	775	553	1549	876	1115	993	1216
ENSG00000248221	3	3	7	1	0	0	3	3	0	2	8	0	3	3	2	1	0	0	1	2	2	4	2	4	3	3	2	11	3	2	10	5
ENSG00000237170	3	1	2	0	1	1	0	2	1	2	7	1	5	5	0	0	2	1	1	2	2	2	1	1	3	7	1	14	0	6	4	1
ENSG00000247708	188	152	177	128	68	84	76	91	109	100	403	77	286	284	361	350	69	92	72	172	108	76	124	63	90	126	76	234	217	310	322	364
ENSG00000280310	11	6	4	9	2	2	1	0	4	1	3	3	36	23	32	30	5	5	5	14	4	0	4	1	2	2	3	11	14	18	12	24
ENSG00000273396	0	2	1	0	0	0	1	0	3	1	2	3	6	6	1	7	0	0	0	1	0	0	0	1	0	1	2	3	0	1	1	11
ENSG00000163132	56	109	63	94	91	96	64	91	177	152	778	119	288	322	379	381	23	43	35	57	190	68	138	62	206	393	210	572	316	384	410	419
ENSG00000251587	0	0	0	0	0	0	0	0	0	0	0	0	0	0	0	0	0	0	0	0	0	0	0	0	0	0	0	0	0	0	0	0
ENSG00000200761	0	0	0	0	0	0	0	0	0	0	0	0	0	0	0	0	0	0	0	0	0	0	0	0	0	0	0	0	0	0	0	0
ENSG00000170891	0	1	0	0	0	0	0	0	0	3	4	0	0	0	0	0	0	0	0	1	0	0	0	0	0	0	2	2	0	0	0	0
ENSG00000152953	19	13	10	28	0	0	0	0	4	3	12	2	0	0	0	1	0	5	3	19	1	0	0	0	3	1	7	15	0	0	0	0
ENSG00000252266	0	0	0	0	0	0	0	0	0	0	0	0	0	0	0	0	0	0	0	0	0	0	0	0	0	0	0	0	0	0	0	0
ENSG00000201496	0	0	0	0	0	0	0	0	0	0	0	0	0	0	0	0	0	0	1	0	0	0	0	0	0	0	0	0	0	0	0	0
ENSG00000082929	0	0	0	0	0	0	0	0	1	2	8	0	0	0	0	0	0	0	0	0	0	0	0	0	1	2	9	12	0	0	0	0
ENSG00000173040	0	0	0	0	0	0	0	0	58	52	198	35	0	0	0	0	0	0	0	0	0	0	0	0	96	113	95	278	0	0	0	0
ENSG00000072840	0	0	0	0	0	0	0	0	142	156	576	116	6	3	1	7	0	0	0	0	0	0	0	1	191	346	294	924	1	4	0	2
ENSG00000072832	1	1	3	0	5	8	11	18	62	52	190	41	242	255	188	335	1	0	1	1	17	7	28	5	72	106	106	322	72	128	56	141
ENSG00000181215	0	2	3	0	0	0	0	0	23	14	67	12	80	90	95	104	0	0	0	2	0	0	0	0	4	13	12	27	48	87	35	82
ENSG00000263631	0	0	0	0	0	0	0	0	0	0	0	0	0	0	0	0	0	0	0	0	0	0	0	0	0	0	0	0	0	1	0	0
ENSG00000152969	1537	1037	1318	1112	0	0	0	1	1065	740	2356	533	2964	2406	2688	3158	472	752	502	939	0	0	0	0	729	1144	332	1330	1526	1552	1529	1754
ENSG00000249896	43	23	42	31	0	0	0	0	1	0	2	0	26	28	41	56	11	7	10	24	0	0	0	0	0	0	2	4	8	13	11	14
ENSG00000251408	37	24	28	22	0	0	0	0	12	7	40	7	50	46	40	55	1	10	6	30	0	0	0	0	6	6	4	10	11	19	9	37
ENSG00000109501	409	364	423	383	76	88	48	97	77	106	554	107	135	147	149	193	86	163	163	311	144	71	112	55	78	186	112	385	105	126	102	150
ENSG00000074211	0	1	0	1	0	0	2	0	5	11	16	18	1	2	0	3	0	0	0	1	1	1	0	0	2	5	0	23	0	2	0	0
ENSG00000013288	1333	1102	1456	1109	748	845	501	846	602	529	2872	470	658	621	663	841	324	405	396	861	1012	430	708	398	445	799	660	1711	446	667	476	762
ENSG00000179010	5501	3860	4383	4338	3149	3465	2384	3603	3751	2877	13499	2251	5351	5163	5375	6774	4992	6590	5218	4356	6911	2804	5173	2299	3166	4841	3098	7787	5531	5547	5998	6413
ENSG00000251580	23	18	30	8	7	12	11	13	13	12	39	4	14	12	10	8	10	25	19	27	35	19	22	8	10	18	16	23	16	13	28	21
ENSG00000170846	902	698	859	785	425	438	346	430	935	705	2817	504	887	776	792	971	529	848	734	835	851	340	613	292	741	1137	631	1691	672	856	754	934
ENSG00000246526	52	53	57	40	18	38	26	45	274	204	948	142	143	137	131	177	40	36	35	47	119	30	74	38	188	215	224	442	115	162	126	127
ENSG00000279859	0	1	0	0	3	0	1	2	0	3	30	8	2	2	1	2	1	1	0	3	0	1	2	1	3	11	1	13	3	3	1	1
ENSG00000163993	21	14	9	15	7	4	8	8	12	6	48	7	111	97	120	157	15	16	17	24	14	7	8	3	16	11	14	28	130	98	130	128
ENSG00000282742	0	0	1	1	0	1	3	2	4	8	19	7	8	2	0	3	0	0	0	5	5	4	2	1	0	6	7	23	1	5	2	4
ENSG00000178988	1748	1173	1384	1254	1306	1295	1081	1470	1703	1243	6115	1047	1821	1656	2224	2399	1977	2369	1978	1664	3664	1453	2587	1406	1488	2415	1657	3963	2935	2656	3411	3278
ENSG00000186222	434	336	414	375	153	177	106	143	317	308	1371	252	406	319	445	360	119	209	227	320	311	127	187	79	263	449	255	714	403	443	422	383
ENSG00000170871	2087	1464	1731	1404	972	1066	718	1034	727	620	4009	593	837	815	810	1051	708	974	798	1378	2018	814	1375	828	853	1242	1084	2774	530	936	605	961
ENSG00000132405	1696	1191	1349	1221	1099	1187	854	1234	927	789	3335	601	1104	1025	1070	1244	569	868	726	1102	2000	1034	1584	767	778	1278	794	2377	776	901	794	966
ENSG00000187904	8	10	7	12	6	4	3	10	13	4	23	3	5	6	1	4	2	1	1	3	8	5	15	5	5	7	5	21	2	2	7	10
ENSG00000202392	5	2	4	4	3	0	3	5	6	7	21	0	5	2	0	3	2	0	0	11	8	3	2	4	4	7	4	8	0	7	1	3
ENSG00000245748	11	7	7	18	7	13	11	10	4	8	28	2	9	2	7	5	2	1	1	9	10	6	5	10	8	14	6	14	1	5	6	8
ENSG00000173013	103	85	112	106	1	4	4	5	6	10	60	11	7	3	10	13	35	42	37	76	6	5	6	1	17	31	22	70	9	8	12	14
ENSG00000173011	643	566	630	493	364	407	285	444	432	380	2365	375	562	479	627	697	186	260	282	414	743	309	500	237	498	873	720	1646	544	627	500	654
ENSG00000109519	1713	1063	1413	1176	753	785	538	850	1047	780	3103	537	2246	1891	2728	2439	2168	3189	2656	2181	1799	702	1312	712	1054	1628	585	2024	3268	2887	3898	3427
ENSG00000245468	1	2	3	7	5	4	1	2	0	2	10	1	1	2	0	1	5	2	3	6	6	2	2	1	0	3	4	8	0	2	1	1
ENSG00000200867	0	0	0	0	0	0	0	0	0	0	0	0	0	0	0	0	0	0	0	0	0	0	0	0	0	0	0	0	0	0	0	0
ENSG00000184985	6	1	4	8	11	11	5	11	0	0	0	1	3	3	0	1	0	0	3	4	9	2	2	6	0	0	0	2	0	3	1	3
ENSG00000264658	0	0	0	0	0	0	0	0	0	0	0	0	0	0	0	0	0	0	0	0	0	0	0	0	0	0	0	0	0	0	0	0
ENSG00000178597	0	0	0	0	0	0	0	0	0	0	0	0	0	0	0	0	0	0	0	0	0	0	0	0	0	0	0	0	0	0	0	0
ENSG00000266690	0	0	0	0	0	0	0	0	0	0	0	0	0	0	0	0	0	0	0	0	0	0	0	0	0	0	0	0	0	0	0	0
ENSG00000272620	6	10	5	19	0	0	0	0	0	0	0	0	8	5	11	12	0	2	2	16	0	0	0	0	0	0	0	0	3	11	8	14
ENSG00000196526	654	810	729	836	1	1	1	6	0	2	10	3	883	1065	923	1343	135	361	214	588	2	1	1	1	0	2	5	7	440	719	428	853
ENSG00000228919	2	2	2	0	0	1	0	0	0	0	0	0	5	5	0	4	0	0	0	1	0	0	0	0	0	0	0	0	0	5	1	5
ENSG00000163995	31	40	28	46	15	26	7	23	5	6	29	4	16	31	22	41	15	33	11	39	4	16	9	1	4	8	7	24	7	14	8	18
ENSG00000207807	0	0	0	0	0	0	0	0	0	0	0	0	0	0	0	0	0	0	0	0	0	0	0	0	0	0	0	0	0	0	0	0
ENSG00000273267	2	1	1	0	1	0	3	0	0	0	0	0	1	0	0	0	0	0	0	2	0	1	0	1	0	0	1	0	0	1	0	1
ENSG00000251460	0	0	0	0	0	0	0	0	0	0	0	0	0	0	0	2	0	0	0	1	0	0	0	0	0	0	0	0	0	0	0	0
ENSG00000250471	2	5	0	2	1	2	2	3	3	2	6	0	5	6	3	1	4	1	2	4	6	1	1	3	2	2	2	3	2	3	0	1
ENSG00000125089	80	44	98	56	1071	1115	865	1157	104	98	1067	155	144	188	154	244	62	62	101	114	1607	679	1056	681	110	109	384	915	76	127	67	126
ENSG00000281359	0	1	0	0	11	10	8	13	1	3	14	0	0	3	0	0	0	0	1	0	21	12	13	7	2	1	2	8	1	1	0	2
ENSG00000170801	0	0	0	0	0	2	0	3	1	0	1	2	0	0	0	0	0	0	0	1	2	0	0	0	0	0	1	1	0	0	0	0
ENSG00000249145	0	0	0	0	0	0	0	0	0	0	0	0	0	0	0	0	0	0	0	0	0	0	0	0	0	0	0	0	0	0	0	0
ENSG00000248986	0	0	0	0	0	0	0	0	0	0	1	0	0	0	0	0	0	0	0	0	0	0	0	0	0	0	0	0	0	0	0	0
ENSG00000251615	21	5	13	12	31	23	17	22	29	23	168	18	6	18	3	31	5	15	12	28	43	17	22	11	13	24	70	94	6	8	9	19
ENSG00000087008	541	375	437	392	341	326	220	355	255	230	842	156	337	329	290	417	131	207	235	323	389	186	285	208	154	329	260	622	242	343	228	338
ENSG00000155275	501	352	511	407	295	335	222	327	302	256	1121	209	457	415	462	502	222	243	303	468	533	274	370	243	245	420	271	830	344	538	421	560
ENSG00000251186	4	2	3	2	4	1	1	4	1	2	8	1	1	7	5	3	2	1	3	4	8	0	2	7	4	5	2	10	1	6	8	11
ENSG00000205959	11	7	5	11	28	38	21	36	18	18	56	9	48	47	28	38	4	1	2	7	28	18	16	21	3	15	11	31	8	14	1	14
ENSG00000155269	0	0	0	0	1	0	0	0	1	1	1	0	0	1	0	0	0	0	0	0	0	0	0	0	0	0	0	0	0	0	0	0
ENSG00000109625	0	0	0	0	0	1	0	0	0	0	0	0	0	0	0	1	0	0	0	1	0	0	0	0	0	0	0	0	0	0	0	0
ENSG00000250915	0	0	0	0	0	0	0	0	0	0	0	0	0	0	0	0	0	0	0	0	0	0	0	0	0	0	0	0	0	0	0	0
ENSG00000215612	0	0	0	0	0	0	0	0	0	0	0	0	0	0	0	0	0	0	0	0	0	0	0	0	0	0	0	0	0	0	0	0
ENSG00000258507	0	0	0	0	0	0	0	0	0	0	0	0	0	0	0	0	0	0	0	0	0	0	0	0	0	0	0	0	0	0	0	0
ENSG00000197468	0	0	0	0	0	0	0	0	0	0	0	0	0	0	0	0	0	0	0	0	0	0	0	0	0	0	0	0	0	0	0	0
ENSG00000249272	0	0	0	0	0	0	0	0	0	0	0	0	0	0	0	0	0	0	0	0	0	0	0	0	0	0	0	0	0	0	0	0
ENSG00000250804	0	0	0	0	0	0	0	0	0	0	0	0	0	0	0	0	0	0	0	0	0	0	0	0	0	0	0	0	0	0	0	0
ENSG00000249347	0	0	0	0	0	0	0	0	0	0	0	0	0	0	0	0	0	0	0	0	0	0	0	0	0	0	0	0	0	0	0	0
ENSG00000271057	0	1	0	0	0	0	0	0	0	0	0	0	0	0	0	0	0	0	0	0	0	0	0	0	0	3	0	0	0	0	0	1
ENSG00000251313	0	0	1	2	0	1	0	1	0	0	1	0	0	0	0	0	0	0	0	1	0	0	1	4	0	0	0	1	0	0	0	2
ENSG00000250342	3	0	0	0	2	1	0	0	0	0	0	0	0	0	0	0	0	0	0	0	0	0	0	0	0	0	0	0	0	0	0	0
ENSG00000249767	7	11	14	14	6	2	5	2	2	2	7	1	2	7	1	2	1	1	0	6	5	5	1	4	1	1	0	8	0	0	1	1
ENSG00000163612	1	3	0	2	0	3	1	4	2	0	1	0	0	1	2	1	1	1	0	4	0	0	1	0	0	0	0	0	2	0	0	0
ENSG00000250268	2	0	0	0	0	0	0	0	0	0	1	1	0	0	0	0	0	0	0	1	0	0	0	0	0	0	0	0	0	0	0	0
ENSG00000229924	0	0	0	0	0	1	0	0	0	1	0	1	0	0	0	0	0	0	0	0	1	0	0	0	0	0	1	0	0	0	0	0
ENSG00000231396	0	0	0	0	0	0	0	0	0	0	0	0	0	0	0	0	0	0	0	0	0	0	0	0	0	0	0	0	0	0	0	0
ENSG00000233136	0	0	0	0	0	0	0	0	0	0	0	0	0	0	0	0	0	0	0	0	0	0	0	0	0	0	0	0	0	0	0	0
ENSG00000227551	0	0	0	0	0	0	0	0	0	0	0	0	0	0	0	0	0	0	0	0	0	0	0	0	0	0	0	0	0	0	0	0
ENSG00000232399	0	0	0	0	0	0	0	0	0	0	0	0	0	0	0	0	0	0	0	0	0	0	0	0	0	0	0	0	0	0	0	0
ENSG00000249482	0	0	0	0	0	0	0	0	0	0	0	0	1	0	0	0	0	0	1	0	0	0	0	0	0	0	0	0	0	0	0	0
ENSG00000223569	0	0	0	0	0	0	0	0	0	0	0	0	0	0	0	0	0	0	0	0	0	0	0	0	0	0	0	0	0	0	0	0
ENSG00000250231	0	0	0	0	0	0	0	0	0	0	0	0	0	0	0	0	0	0	0	0	0	0	0	0	0	0	0	0	0	0	0	0
ENSG00000249104	0	0	0	0	0	0	0	0	0	0	0	0	0	0	0	0	0	0	0	0	0	0	0	0	0	0	0	0	0	0	0	0
ENSG00000250844	0	0	0	0	0	0	0	0	0	0	0	0	0	0	0	0	0	0	0	0	0	0	0	0	0	0	0	0	0	0	0	0
ENSG00000248920	0	0	0	0	0	0	0	0	0	0	0	0	0	0	0	0	0	0	0	0	0	0	0	0	0	0	0	0	0	0	0	0
ENSG00000250745	0	0	0	0	0	0	0	0	0	0	0	0	0	0	0	0	0	0	0	0	0	0	0	0	0	0	0	0	0	0	0	0
ENSG00000249811	0	0	0	0	0	0	0	0	0	0	0	0	0	0	0	0	0	0	0	0	0	0	0	0	0	0	0	0	0	0	0	0
ENSG00000248933	0	0	0	0	0	0	0	0	0	0	0	0	0	0	0	0	0	0	0	0	0	0	0	0	0	0	0	0	0	0	0	0
ENSG00000250913	0	0	0	0	0	0	0	0	0	0	0	0	0	0	0	0	0	0	0	0	0	0	0	0	0	0	0	0	0	0	0	0
ENSG00000232264	0	0	0	0	0	0	0	0	0	0	0	0	0	0	0	0	0	0	0	0	0	0	0	0	0	0	0	0	0	0	0	0
ENSG00000230430	0	0	0	0	0	0	0	0	0	0	0	0	0	0	0	0	0	0	0	0	0	0	0	0	0	0	0	0	0	0	0	0
ENSG00000229579	0	0	0	0	0	0	0	0	0	0	0	0	0	0	0	0	0	0	0	0	0	0	0	0	0	0	0	0	0	0	0	0
ENSG00000227140	0	0	0	0	0	0	0	0	0	0	0	0	0	0	0	0	0	0	0	0	0	0	0	0	0	0	0	0	0	0	0	0
ENSG00000235780	0	0	0	0	0	0	0	0	0	0	0	0	0	0	0	0	0	0	0	0	0	0	0	0	0	0	0	0	0	0	0	0
ENSG00000231051	0	0	0	0	0	0	0	0	0	0	0	0	0	0	0	0	0	0	0	0	0	0	0	0	0	0	0	0	0	0	0	0
ENSG00000231637	0	0	0	0	0	0	0	0	0	0	0	0	0	0	0	0	0	0	0	0	0	0	0	0	0	0	0	0	0	0	0	0
ENSG00000251694	0	0	0	0	0	0	0	0	0	0	0	0	0	0	0	0	0	0	0	0	0	0	0	0	0	0	0	0	0	0	0	0
ENSG00000228856	0	0	0	0	0	0	0	0	0	0	0	0	0	0	0	0	0	0	0	0	0	0	0	0	0	0	0	0	0	0	0	0
ENSG00000205946	0	0	0	0	0	0	0	0	0	0	0	0	0	0	0	0	0	0	0	0	0	0	0	0	0	0	0	0	0	0	0	0
ENSG00000219492	0	0	0	0	0	0	0	0	0	0	0	0	0	0	0	0	0	0	0	0	0	0	0	0	0	0	0	0	0	0	0	0
ENSG00000188438	0	0	0	0	0	0	0	0	0	0	0	0	0	0	0	0	0	0	0	0	0	0	0	0	0	0	0	0	0	0	0	0
ENSG00000232773	0	0	0	0	0	0	0	0	0	0	0	0	0	0	0	0	0	0	0	0	0	0	0	0	0	0	0	0	0	0	0	0
ENSG00000186146	0	0	0	0	0	0	0	0	0	0	0	0	0	0	0	0	0	0	0	0	1	0	0	0	0	0	0	0	0	0	0	0
ENSG00000248364	0	0	0	0	0	0	1	0	0	0	0	0	0	0	0	0	0	0	0	0	0	0	0	0	0	0	0	0	0	0	0	0
ENSG00000250884	0	0	0	0	0	0	0	0	0	0	0	0	0	0	0	0	0	0	0	0	0	0	0	0	0	0	0	0	0	0	0	0
ENSG00000251624	0	0	0	0	0	0	0	0	0	0	0	0	0	0	0	0	0	0	0	0	0	0	0	0	0	0	0	0	0	0	0	0
ENSG00000249866	0	0	1	0	0	0	0	0	0	0	0	0	0	0	0	0	0	0	0	1	1	0	0	0	0	0	0	0	0	0	0	0
ENSG00000249443	0	0	0	0	0	0	0	0	0	0	0	0	0	0	0	0	0	0	0	0	0	0	0	0	0	0	0	0	0	0	0	0
ENSG00000242034	12	6	12	7	7	9	8	14	1	1	0	0	0	5	1	0	2	4	0	19	12	15	9	7	2	2	0	2	0	0	0	5
ENSG00000221275	15	13	22	11	4	6	6	15	0	0	0	0	0	0	0	0	3	0	3	24	17	6	5	13	1	0	1	1	0	0	0	1
ENSG00000244004	26	25	22	17	9	16	9	17	0	0	0	1	0	2	0	1	4	6	6	19	18	16	13	19	1	0	1	3	0	1	2	3
ENSG00000249799	10	8	9	6	1	1	4	3	0	0	1	0	0	1	0	1	1	4	0	16	9	2	3	6	0	0	0	0	0	0	0	0
ENSG00000250942	39	32	28	35	20	29	17	33	0	0	0	0	0	0	2	0	10	14	10	61	71	29	25	38	0	0	0	1	0	0	0	0
ENSG00000186234	0	0	0	1	0	0	0	2	0	0	0	1	0	0	0	0	0	1	0	4	0	1	0	1	0	0	0	0	0	0	0	0
ENSG00000251087	3	4	1	0	0	0	1	0	0	0	0	0	0	0	0	0	1	0	2	2	0	0	0	0	0	0	0	0	0	0	0	0
ENSG00000203436	0	1	2	0	0	0	0	0	0	1	2	0	0	0	3	1	0	0	2	0	0	0	0	0	1	0	0	0	0	1	0	1
ENSG00000249563	0	0	0	0	0	0	0	0	0	0	0	0	0	0	0	0	0	0	0	0	0	0	0	0	0	0	0	0	0	0	0	0
ENSG00000250425	0	0	0	0	0	0	0	0	0	0	0	0	0	0	0	0	0	0	0	0	0	0	0	0	0	0	1	0	0	0	0	0
ENSG00000109667	35	40	59	49	4	4	2	6	0	0	5	2	11	9	8	9	17	45	25	54	8	3	3	4	0	0	2	1	1	5	3	2
ENSG00000169676	0	0	0	0	0	0	0	0	0	0	0	0	0	0	0	0	0	0	0	0	0	0	0	0	0	0	0	0	0	0	0	0
ENSG00000249219	0	1	0	0	0	0	0	0	0	0	0	0	0	0	0	0	0	0	0	0	0	0	0	0	0	0	0	0	0	0	0	0
ENSG00000250413	1	0	3	0	0	0	0	0	0	0	0	0	0	0	0	1	0	0	0	1	0	0	0	2	0	0	0	0	0	0	2	0
ENSG00000261490	96	77	90	68	90	72	82	98	114	91	575	80	98	70	88	110	43	36	50	148	194	73	86	88	88	155	127	323	55	128	61	127
ENSG00000071127	18371	13821	17496	14619	7474	7229	5377	7745	7658	5819	35133	5526	9124	7947	9862	10672	4723	8422	8989	13083	12684	5053	9894	4486	7506	11937	6618	21901	7483	8978	8168	9966
ENSG00000264931	2	2	3	4	0	1	1	1	5	0	5	0	1	2	1	1	2	2	2	8	4	1	1	2	5	5	2	9	4	0	2	2
ENSG00000250393	0	0	0	0	0	0	0	0	0	0	0	0	0	0	1	0	0	2	0	1	0	0	0	0	0	0	0	1	0	0	0	3
ENSG00000251296	0	0	2	0	0	0	0	0	0	0	0	0	0	0	0	0	0	0	0	1	0	1	0	0	0	0	0	0	0	0	0	2
ENSG00000248777	0	0	0	0	0	0	0	0	0	0	0	0	0	0	0	0	0	0	0	0	0	0	0	0	0	0	0	1	0	0	1	0
ENSG00000271544	0	0	0	0	0	0	0	0	0	0	0	0	0	0	0	0	0	0	0	0	0	0	0	0	0	0	0	0	0	0	0	0
ENSG00000250040	0	0	2	0	0	0	0	2	0	1	0	0	0	0	0	0	0	0	0	0	0	2	0	0	0	0	0	1	0	1	2	4
ENSG00000250573	0	0	0	0	0	0	0	0	0	0	4	0	0	0	0	0	0	0	0	0	0	0	0	0	0	0	0	0	0	0	0	1
ENSG00000250074	0	0	0	0	0	0	0	1	0	0	0	0	0	0	0	0	0	0	0	0	1	0	0	0	0	0	0	1	0	0	0	2
ENSG00000250505	0	0	0	0	0	0	0	0	0	0	2	0	0	0	0	0	0	0	0	0	0	1	0	1	0	0	0	2	0	2	0	2
ENSG00000251278	0	0	0	2	0	0	0	0	0	0	0	0	0	0	0	0	0	0	0	0	0	0	0	0	0	0	0	0	0	0	0	0
ENSG00000251338	1	1	0	0	0	0	0	0	1	0	0	0	0	0	0	0	0	0	0	0	0	0	0	0	0	0	0	0	0	1	2	1
ENSG00000250613	2	0	0	0	0	0	0	0	0	0	0	0	0	0	1	1	0	2	0	1	0	1	0	0	0	0	0	1	0	1	1	0
ENSG00000178163	3581	2811	3311	2810	378	422	283	487	350	361	1353	243	1120	1251	1125	1489	1663	2036	1872	3142	788	302	548	297	333	520	397	955	656	1151	855	1334
ENSG00000248419	0	0	0	0	1	0	0	2	0	0	0	0	0	0	0	0	0	0	0	2	0	0	0	0	0	0	0	0	0	0	0	0
ENSG00000109684	0	0	0	0	319	325	249	355	14	8	99	6	3	2	4	5	1	0	0	0	446	206	294	183	2	9	20	29	0	0	1	0
ENSG00000249334	0	0	0	0	45	28	37	36	1	0	2	0	0	0	0	1	0	2	0	0	114	32	98	37	0	0	1	0	0	0	0	0
ENSG00000283083	0	0	0	0	0	0	3	1	0	0	0	0	1	0	0	0	0	0	0	0	0	0	1	0	0	0	0	0	0	0	0	0
ENSG00000207716	1	0	0	0	5	2	1	2	0	0	0	0	0	1	0	0	0	0	1	0	2	4	1	5	0	0	0	0	0	0	0	0
ENSG00000250896	4	8	2	6	2	9	2	7	3	0	5	2	2	2	3	4	0	0	1	0	9	4	11	3	0	3	0	4	0	2	2	3
ENSG00000002587	1	2	4	1	55	63	37	71	0	0	1	0	5	4	0	2	0	0	0	3	58	25	35	31	0	0	0	0	0	1	1	1
ENSG00000251152	0	4	0	1	12	12	11	6	0	0	0	0	0	0	0	0	0	0	0	0	19	6	3	4	0	0	0	0	0	0	0	0
ENSG00000249631	1	0	1	0	13	14	11	12	0	0	0	0	0	0	0	0	0	0	0	0	9	0	5	10	0	0	0	0	0	3	1	0
ENSG00000248300	0	0	0	1	3	0	1	1	0	0	0	0	0	0	0	0	0	0	0	0	1	1	0	1	0	0	0	0	0	0	0	0
ENSG00000248262	0	0	0	0	0	0	0	0	0	0	0	0	0	0	0	0	0	0	0	0	0	0	0	0	0	0	0	0	0	0	0	0
ENSG00000251210	0	0	0	0	0	0	0	0	0	0	3	0	0	0	0	0	0	0	0	0	0	0	0	0	0	0	0	0	0	1	0	0
ENSG00000212282	0	0	0	0	0	0	0	0	0	0	0	0	0	0	0	0	0	0	0	0	0	0	0	0	0	0	0	0	0	0	0	0
ENSG00000249995	0	0	0	0	0	0	0	0	0	0	0	0	0	0	0	1	0	0	0	0	0	0	0	0	0	0	0	0	0	0	0	0
ENSG00000249780	0	0	0	0	0	0	0	0	1	0	0	0	0	0	0	0	0	0	0	0	0	0	0	0	0	0	0	0	0	0	0	0
ENSG00000250371	3	0	1	2	1	2	0	2	7	2	3	0	3	5	1	8	0	0	0	3	2	0	2	2	3	6	0	7	0	3	1	9
ENSG00000250098	0	0	0	0	0	0	0	0	0	0	1	0	1	1	0	1	1	0	0	0	1	0	1	0	0	4	1	3	0	2	0	3
ENSG00000283271	0	0	0	0	0	0	0	0	0	0	3	0	0	0	0	0	0	0	0	1	0	0	0	0	0	0	0	0	0	0	0	0
ENSG00000205940	4	4	3	2	1	4	4	2	0	4	17	2	17	5	6	5	1	1	3	7	10	11	5	11	10	9	3	26	3	19	6	20
ENSG00000157869	934	605	675	597	347	331	246	369	531	460	1729	370	596	523	543	678	749	985	704	786	820	309	561	346	491	815	421	1285	577	613	680	796
ENSG00000281202	0	0	0	0	0	0	0	0	5	3	10	0	8	6	10	5	0	0	0	0	0	1	0	0	1	3	0	4	2	1	3	1
ENSG00000109705	20	13	13	13	1	1	0	0	15	9	37	14	17	31	20	28	1	6	6	12	1	0	0	0	7	17	4	32	2	7	5	10
ENSG00000246095	20	13	28	16	0	1	0	1	4	7	9	4	14	18	16	31	13	15	13	20	0	0	0	0	1	10	4	13	7	9	11	14
ENSG00000038219	2941	2134	2693	2044	3022	3284	2597	3484	3389	2711	14190	2079	5278	4935	5462	6303	898	733	833	1800	4988	2339	3497	2088	2947	4463	3739	9273	2690	4574	3353	4979
ENSG00000281620	0	0	0	0	0	0	0	0	0	0	0	0	0	0	0	0	0	0	0	0	0	0	0	0	0	0	0	0	0	0	0	0
ENSG00000266240	3	1	2	1	0	3	1	1	2	0	2	1	3	0	3	4	1	1	1	3	1	4	0	0	0	0	0	0	3	0	0	0
ENSG00000249148	2	0	10	0	2	0	1	1	1	1	4	0	4	0	1	4	0	0	0	6	2	0	0	1	0	0	0	3	0	0	5	2
ENSG00000242209	0	1	0	0	0	0	0	0	0	0	0	0	0	0	0	0	0	0	0	0	0	0	0	0	0	0	0	1	0	0	0	0
ENSG00000250634	0	0	0	0	1	2	2	11	0	0	0	0	0	0	0	0	0	0	0	0	4	3	4	2	0	0	0	0	0	0	0	0
ENSG00000250497	0	0	0	0	0	0	0	0	0	0	0	0	0	0	0	0	0	0	0	0	0	1	0	0	0	0	0	1	0	0	0	0
ENSG00000224569	0	0	0	0	1	2	2	5	0	0	0	0	0	0	0	0	0	0	0	0	0	0	5	1	0	0	0	0	0	0	0	0
ENSG00000248698	0	0	0	0	2	2	0	0	0	0	2	0	0	0	0	1	0	1	0	0	2	1	0	2	2	0	0	0	0	0	0	0
ENSG00000251317	0	0	0	0	0	0	0	0	0	0	0	0	0	0	0	0	0	0	0	0	0	0	0	0	0	0	0	0	0	0	0	0
ENSG00000251679	0	0	0	0	0	0	0	0	0	0	0	0	1	0	0	0	0	0	0	0	0	0	0	0	0	0	0	0	0	0	1	0
ENSG00000249988	0	0	0	1	0	0	0	0	0	0	0	0	0	0	0	0	0	0	0	0	0	0	0	0	0	0	0	0	0	0	0	0

ENSG00000251412	0	0	0	0	1	0	0	0	0	0	0	0	0	0	0	0	0	0	0	0	0	0	0	0	0	0	0	0	0	0	0	0
ENSG00000248425	0	0	0	0	0	0	0	0	0	0	0	0	0	0	0	0	0	0	0	0	0	0	0	0	0	0	0	0	0	1	0	0
ENSG00000248360	0	1	1	1	63	40	63	68	0	0	0	0	0	6	4	4	0	0	1	0	238	75	156	76	0	1	0	2	0	0	0	0
ENSG00000251252	0	0	0	0	0	0	0	1	0	0	0	0	0	0	0	0	0	0	0	0	1	0	0	0	0	0	0	0	0	0	0	0
ENSG00000202449	0	0	0	0	1	0	0	0	0	0	0	0	0	0	0	0	0	0	0	0	3	2	0	0	0	0	0	0	0	0	0	0
ENSG00000247624	1	2	2	1	0	4	0	7	0	1	10	2	0	1	0	2	1	0	0	4	2	4	1	0	2	3	0	16	0	1	2	1
ENSG00000137449	152	173	126	145	281	278	222	303	47	61	458	46	68	120	88	114	33	46	44	124	514	237	392	249	83	102	179	282	45	75	45	83
ENSG00000249252	3	3	10	1	4	8	3	1	0	2	13	1	1	0	2	0	0	0	0	1	8	7	9	7	0	0	2	7	0	0	1	2
ENSG00000199420	0	0	0	0	2	0	0	0	1	0	0	0	0	0	0	0	0	0	0	0	0	0	0	0	0	0	0	0	0	0	0	0
ENSG00000163145	1	1	3	2	1	2	3	1	0	1	3	0	186	139	153	215	0	0	1	0	2	0	0	2	0	1	2	4	145	133	135	183
ENSG00000251379	1	0	0	0	1	3	0	1	0	0	0	1	1	1	0	3	0	0	0	0	4	0	0	0	1	0	0	0	0	1	1	0
ENSG00000048342	28	23	56	29	27	38	27	39	43	26	118	19	31	47	38	37	10	9	10	45	56	30	25	51	45	68	41	142	8	52	43	49
ENSG00000249930	1	0	1	1	0	0	0	0	1	1	3	0	2	0	0	2	0	0	0	0	2	0	0	0	0	0	0	1	1	1	0	0
ENSG00000273133	0	4	2	2	0	2	3	2	0	3	8	3	2	6	3	1	2	5	0	0	2	2	4	1	1	0	1	7	1	3	1	1
ENSG00000118564	1227	815	947	779	980	1049	747	1065	1018	905	4052	776	751	828	827	1024	1071	1200	1047	1005	2268	891	1468	1008	922	1226	849	2670	751	937	892	1148
ENSG00000237765	263	181	259	165	169	165	165	190	298	280	1400	219	287	348	387	398	260	222	350	255	362	164	302	201	288	404	285	893	402	453	583	606
ENSG00000109743	84	66	97	65	0	1	0	0	4	3	15	7	8	6	14	9	68	123	123	101	5	0	0	0	7	14	7	17	8	12	1	10
ENSG00000214846	0	1	0	5	0	0	0	0	1	0	4	2	0	0	0	1	0	0	2	5	0	1	0	0	0	0	0	0	0	0	0	1
ENSG00000248188	0	0	0	0	0	0	0	0	0	0	0	0	0	0	0	0	0	0	0	0	0	0	0	0	0	0	0	0	0	0	0	0
ENSG00000004468	165	132	157	132	374	483	372	507	2733	2034	6893	1772	190	301	223	359	23	58	64	57	1288	543	1058	486	199	473	21	569	76	198	116	227
ENSG00000251644	0	0	0	0	0	0	0	0	0	0	0	0	0	0	0	0	0	0	0	0	0	0	0	0	0	0	0	0	0	0	0	0
ENSG00000137440	0	0	0	0	0	0	0	0	0	1	0	0	0	0	0	0	0	0	0	0	0	0	0	0	0	0	0	0	0	0	0	0
ENSG00000137441	11	7	7	5	24	29	22	48	391	377	672	226	0	2	0	2	0	0	0	0	10	7	6	6	13	21	47	39	0	0	0	2
ENSG00000007062	2	0	4	4	16	8	5	10	2	1	6	1	4	1	3	3	2	2	3	0	17	7	25	6	0	0	6	3	0	1	3	1
ENSG00000169762	1775	1303	1487	1255	1053	1050	862	1075	741	747	3224	504	751	738	808	904	761	1118	1010	1255	1982	798	1319	795	651	881	660	1820	645	928	809	984
ENSG00000249234	18	4	9	7	5	6	2	15	4	2	19	3	5	5	6	4	8	3	5	9	15	10	6	13	1	5	6	8	0	6	2	6
ENSG00000263327	54	47	66	41	21	28	21	26	38	32	166	16	24	24	27	40	19	30	23	53	44	32	29	24	13	30	49	66	15	39	19	37
ENSG00000242358	0	0	0	0	0	0	0	0	0	0	0	0	0	0	0	0	0	0	0	0	0	0	0	0	0	0	0	0	0	0	0	0
ENSG00000248851	0	0	0	0	0	0	0	0	0	0	0	0	0	0	0	0	0	0	0	0	0	1	0	0	0	0	0	0	0	0	0	0
ENSG00000249506	0	0	3	0	0	0	0	0	0	0	0	0	0	0	0	0	0	0	0	0	0	0	0	0	0	0	0	1	0	1	0	0
ENSG00000248138	0	0	0	0	0	0	0	0	0	1	1	1	0	0	0	0	0	0	0	0	0	0	0	0	1	0	2	0	0	0	0	0
ENSG00000169744	0	0	0	1	0	0	0	0	12	11	58	12	1	0	1	1	0	0	0	0	1	0	0	0	28	18	33	63	0	0	0	0
ENSG00000249998	0	0	0	0	0	0	0	0	0	0	0	0	0	0	0	0	0	0	0	0	0	0	0	0	0	0	0	0	0	0	0	0
ENSG00000251212	0	0	0	0	0	0	0	0	0	0	0	0	0	0	0	0	0	0	0	0	0	0	0	0	0	0	0	0	0	0	0	0
ENSG00000250819	0	0	0	0	0	0	0	0	0	0	0	0	0	0	0	0	0	0	0	0	0	0	0	0	0	0	0	0	0	0	0	0
ENSG00000206780	0	0	0	0	0	1	0	0	0	0	0	0	0	0	0	0	0	0	0	0	0	0	0	0	0	0	0	0	0	0	0	0
ENSG00000215278	0	0	0	0	0	0	0	0	0	0	2	0	0	0	0	0	0	0	0	0	0	0	0	0	0	0	0	0	0	0	0	0
ENSG00000151552	452	430	369	473	610	650	422	656	577	411	1652	324	448	436	423	555	210	405	299	437	973	418	756	385	440	733	333	1136	368	405	389	430
ENSG00000249581	0	2	0	0	0	1	0	0	0	0	1	0	0	0	0	0	0	0	0	0	0	1	0	1	0	0	0	0	0	1	0	0
ENSG00000249488	1	2	0	0	0	0	0	0	0	0	3	0	0	0	0	1	0	0	0	0	0	0	1	0	0	0	0	0	0	0	0	0
ENSG00000283219	2	0	0	0	0	0	0	0	0	0	0	0	0	0	0	0	0	0	0	0	0	0	0	0	0	0	0	0	0	0	1	0
ENSG00000002549	1586	1119	1393	1187	1629	1634	1295	1779	1780	1316	5230	1081	3008	2762	3019	3520	1536	2688	2183	1599	3562	1360	2898	1183	1656	2460	1050	3794	3599	3536	4020	3962
ENSG00000249502	12	11	14	12	3	5	7	9	1	3	15	4	24	6	10	12	7	5	10	10	10	2	5	4	4	4	5	10	7	9	9	24
ENSG00000118579	4685	3279	3933	3487	2265	2343	1762	2538	2058	1646	7850	1287	2065	1830	1943	2430	2703	3335	2940	3727	3955	1568	2519	1521	1703	2569	1802	4403	1550	2081	1788	2386
ENSG00000047662	63	57	43	68	18	17	11	24	29	16	53	8	28	26	12	27	11	7	8	41	31	11	24	17	9	15	15	35	7	19	14	15
ENSG00000163257	2180	1683	2139	1722	1716	1757	1274	1891	2156	1791	7607	1300	2548	2225	2456	3046	694	931	881	1551	3082	1261	2093	1168	1730	2594	1432	4963	1446	2404	1782	2722
ENSG00000109805	3864	2548	3203	2520	1878	2106	1640	2181	2364	1817	5492	1350	2095	2159	2256	2898	4173	4826	4586	3730	5976	2193	3901	2487	2455	3517	764	5589	2589	3137	3331	3869
ENSG00000178177	1525	1280	1050	1075	726	791	544	760	633	586	2054	406	923	995	866	1211	647	902	676	1144	1397	581	846	627	736	1041	446	1876	524	833	749	1007
ENSG00000235413	11	2	8	8	8	6	7	7	7	14	35	7	5	9	5	12	0	2	3	1	10	6	12	11	12	18	8	48	3	7	10	19
ENSG00000251048	0	0	0	0	0	0	0	0	0	0	0	0	0	0	0	0	0	0	0	0	0	0	0	0	0	0	0	0	0	0	0	0
ENSG00000279918	0	0	0	0	0	0	0	0	0	0	0	0	0	0	0	0	0	0	0	0	0	0	0	0	0	0	0	0	0	0	0	0
ENSG00000248238	0	0	0	0	0	0	0	0	0	0	0	0	0	0	0	0	0	0	0	0	0	0	0	0	0	0	1	0	0	0	0	0
ENSG00000279379	0	0	0	0	0	0	0	0	0	0	0	0	0	0	0	0	0	0	0	0	0	0	0	0	0	0	0	0	0	0	0	0
ENSG00000248515	0	0	0	0	0	0	0	0	0	0	0	0	0	0	0	0	0	0	0	0	0	0	0	0	0	0	0	1	0	0	0	0
ENSG00000249441	0	0	0	0	0	0	0	0	0	0	0	0	0	0	0	0	0	0	0	0	0	0	0	0	0	0	0	0	0	0	0	0
ENSG00000241612	1	0	0	0	0	1	0	0	0	1	0	0	0	1	0	0	0	0	0	0	0	0	0	0	0	1	0	0	0	1	0	0
ENSG00000250611	0	0	0	0	0	0	0	0	0	0	0	0	0	0	0	0	0	0	0	0	0	0	0	0	0	0	0	0	0	0	0	0
ENSG00000145147	5	14	20	20	0	0	0	0	0	3	3	1	0	0	0	0	2	14	3	17	0	0	0	0	0	4	6	9	0	0	0	0
ENSG00000248228	0	0	0	0	0	0	0	0	0	0	0	0	0	0	0	0	0	0	0	0	0	0	0	0	0	0	0	0	0	0	0	0
ENSG00000207732	0	0	0	0	0	0	0	0	0	0	0	0	0	0	0	0	0	0	0	0	0	0	0	0	0	0	0	0	0	0	0	0
ENSG00000163138	468	327	325	343	167	191	142	202	236	204	793	123	512	453	521	670	363	518	351	493	275	125	200	175	218	346	183	528	428	550	477	635
ENSG00000185774	89	51	74	77	31	30	23	55	81	75	266	37	98	132	102	131	32	46	40	106	79	40	37	21	75	108	58	165	69	117	69	129
ENSG00000272995	4	3	2	1	0	0	0	1	2	0	0	0	1	2	1	2	2	2	0	7	1	0	0	0	0	0	1	0	2	0	0	0
ENSG00000250243	0	0	0	0	0	0	0	0	0	0	0	0	0	0	0	0	0	0	0	0	0	0	0	0	0	0	0	0	0	0	0	0
ENSG00000283281	0	0	0	0	0	0	0	0	0	0	0	0	0	0	0	0	0	0	0	0	0	0	0	0	0	0	0	0	0	0	0	0
ENSG00000250092	0	0	0	0	0	0	0	0	0	0	1	0	0	0	0	0	0	0	0	0	0	0	0	0	0	0	0	0	0	0	0	0
ENSG00000242768	0	0	0	0	0	0	0	0	0	0	0	0	0	0	0	0	0	0	0	0	0	0	0	0	0	0	0	0	0	0	0	0
ENSG00000248343	0	0	0	0	0	0	0	0	0	0	0	0	0	0	0	0	0	0	0	0	0	0	0	0	0	0	0	0	0	0	0	0
ENSG00000280650	0	0	0	0	0	0	0	0	0	0	0	0	0	0	1	0	0	0	0	0	0	0	0	0	0	0	0	2	0	0	0	0
ENSG00000250039	4	4	3	7	6	2	2	6	1	0	10	0	0	0	0	1	0	4	0	3	2	1	5	3	1	5	4	3	0	0	0	0
ENSG00000239001	0	0	0	0	0	0	0	0	0	0	0	0	0	0	0	0	0	0	0	0	0	0	0	0	0	0	0	0	0	0	0	0
ENSG00000152990	1374	1269	1234	1317	1198	1240	863	1239	1027	887	7407	982	2	5	0	5	627	1164	836	1584	2161	919	1497	852	1136	1508	1603	4774	0	4	2	1
ENSG00000244538	0	0	0	0	0	0	0	0	0	0	0	0	0	0	0	0	0	0	0	0	0	0	0	0	0	2	0	0	1	0	0	1
ENSG00000251516	0	1	0	0	0	0	0	0	0	0	3	0	0	0	0	0	0	1	2	0	0	0	0	0	0	0	1	0	0	0	0	0
ENSG00000249948	0	0	0	0	0	0	0	0	0	0	0	0	0	0	0	0	0	0	0	0	0	0	0	0	0	0	0	0	0	0	0	0
ENSG00000237350	9	5	3	1	3	9	4	3	5	3	13	2	1	1	5	5	7	3	6	1	14	3	5	4	0	4	1	11	1	4	8	5
ENSG00000248837	0	0	0	0	0	0	0	0	0	0	1	0	4	3	2	1	0	0	0	0	0	0	0	0	0	0	0	0	2	4	1	3
ENSG00000249645	0	0	0	0	0	0	0	0	0	0	0	0	0	1	0	2	0	0	0	0	0	0	0	0	0	0	0	0	0	0	0	0
ENSG00000249547	0	0	0	0	0	0	0	0	0	0	0	0	0	0	0	0	0	0	0	0	0	0	0	0	0	0	0	0	0	0	0	0
ENSG00000251220	0	0	0	0	0	0	0	0	0	0	0	0	0	0	0	0	0	0	0	0	0	0	0	0	0	0	0	0	0	0	0	0
ENSG00000250137	0	0	0	0	0	0	0	0	0	0	1	0	0	0	0	0	0	0	0	0	0	0	0	1	0	0	0	0	0	0	0	0
ENSG00000251292	0	0	0	0	0	0	0	0	0	0	0	0	0	0	0	0	0	0	0	0	0	0	0	0	0	0	0	0	0	0	0	0
ENSG00000109819	0	2	1	3	7	2	3	4	4	1	2	0	0	1	0	4	0	1	0	6	0	1	3	0	2	0	6	0	0	3	0	0
ENSG00000249453	0	0	0	1	0	0	0	1	0	0	2	0	0	3	1	0	0	0	0	0	0	0	0	0	0	0	2	0	0	0	0	0
ENSG00000109606	12026	8162	9012	7962	4582	4885	3631	5232	6496	5221	19796	4067	8263	7580	8501	9228	9180	11018	9953	11792	12106	4833	7749	4607	6583	9766	4097	14495	7469	8704	8513	10283
ENSG00000207697	26	36	27	36	14	23	21	18	29	32	64	7	41	27	16	43	5	2	4	25	14	9	11	9	18	28	16	43	12	25	12	25
ENSG00000243005	5	2	4	1	1	2	0	3	4	2	3	1	5	5	4	1	0	8	0	8	4	3	1	6	0	4	0	12	2	7	15	5
ENSG00000249256	0	0	0	0	0	0	0	0	0	0	1	0	1	1	0	0	0	0	0	1	0	0	0	0	0	1	0	0	1	0	0	0
ENSG00000261121	0	0	1	2	0	0	0	0	0	0	2	0	4	3	0	1	0	0	0	0	0	0	0	0	0	2	1	2	0	0	0	0
ENSG00000251638	0	0	0	0	0	0	0	0	0	0	0	0	0	0	0	0	0	0	0	0	0	0	0	0	0	0	0	0	0	0	0	0
ENSG00000248444	0	0	0	0	0	0	0	0	1	0	0	0	0	0	0	0	0	0	0	0	0	0	0	0	0	0	0	1	0	0	0	0
ENSG00000109610	0	0	0	0	0	0	0	0	0	0	0	0	0	0	0	0	0	0	0	0	0	0	0	0	0	0	0	0	0	0	0	0
ENSG00000181982	2	3	0	0	0	0	1	0	0	0	2	1	1	0	0	0	2	1	0	0	0	1	0	0	0	0	1	2	0	0	2	1
ENSG00000153012	0	1	1	0	2	3	0	0	1	2	7	3	0	0	0	0	0	0	0	1	3	0	0	0	5	4	8	7	0	0	0	0
ENSG00000109618	1018	773	798	756	792	778	615	865	492	412	1760	278	742	651	640	819	634	760	662	764	1887	772	1215	729	440	590	507	1080	428	536	515	649
ENSG00000281501	80	68	68	42	75	51	45	63	131	83	318	61	81	95	93	105	27	43	49	69	92	42	57	44	84	100	73	228	69	75	96	107
ENSG00000281028	7	6	2	2	0	0	0	1	1	0	0	0	1	2	5	2	5	3	3	5	5	0	2	1	2	2	0	0	6	2	1	5
ENSG00000271172	10	14	13	8	4	4	2	7	10	5	21	4	7	7	9	11	2	6	2	6	5	0	9	2	2	4	4	10	2	8	4	5
ENSG00000038210	1312	846	904	778	1211	1126	918	1251	895	735	2187	505	1450	1456	1106	1768	773	1198	693	926	2806	1159	2031	1013	924	1213	326	1743	1126	1384	916	1337
ENSG00000278135	0	1	1	0	3	0	3	1	2	0	6	2	0	0	1	2	0	0	0	1	4	3	2	6	2	3	0	6	1	2	0	8
ENSG00000168228	674	476	627	426	489	485	354	467	292	253	1027	197	526	503	674	702	462	533	529	540	1178	450	733	432	290	431	188	714	563	604	637	684
ENSG00000053900	1812	1222	1439	1136	1260	1316	1120	1497	984	776	3716	513	1322	1214	1225	1500	1272	1520	1397	1665	2849	1179	1896	1336	897	1328	798	2304	1058	1548	1380	1738
ENSG00000249320	0	0	0	0	0	0	0	1	0	0	2	0	0	1	0	2	0	0	0	0	1	0	0	1	0	1	0	0	0	0	0	1
ENSG00000248608	0	0	0	0	0	0	0	0	0	0	1	0	0	0	0	0	0	0	0	0	1	0	0	0	0	1	1	1	0	0	0	0
ENSG00000238632	0	0	0	0	0	0	0	0	0	0	0	0	0	0	0	0	0	0	0	0	0	0	0	0	0	0	0	0	0	0	0	0
ENSG00000248545	0	0	0	0	0	0	0	0	0	0	0	0	0	0	0	0	0	0	0	0	0	0	0	0	0	0	0	0	0	0	0	0
ENSG00000228154	0	0	0	0	0	0	0	0	0	0	0	0	0	0	0	0	0	0	0	0	0	0	0	0	0	0	0	0	0	0	0	0
ENSG00000157765	1	1	0	1	0	1	0	1	0	0	3	0	1	0	0	0	0	1	1	0	0	0	0	0	0	1	0	1	0	0	0	0
ENSG00000242640	0	0	0	0	0	0	0	0	0	0	0	0	0	0	0	0	0	0	0	0	0	0	0	0	0	0	0	0	0	0	0	0
ENSG00000249277	0	0	0	0	0	0	0	0	0	0	0	0	0	0	0	0	0	0	0	0	0	0	0	0	0	0	0	0	0	0	0	0
ENSG00000250050	1	0	0	0	0	0	0	0	0	0	4	0	2	1	0	0	0	0	0	0	0	0	0	0	0	0	0	0	0	1	1	0
ENSG00000248133	0	0	0	0	0	0	0	0	0	0	1	0	0	0	0	0	0	0	0	0	0	0	0	0	0	0	0	0	0	0	0	0
ENSG00000251353	0	0	0	0	0	0	0	0	0	0	0	0	1	0	0	0	0	0	0	0	0	0	0	0	0	0	0	0	0	0	0	0
ENSG00000248654	1	1	0	0	0	0	0	0	0	0	0	0	2	1	0	1	0	0	0	0	0	0	1	0	0	0	0	0	0	1	0	0
ENSG00000091490	47	163	87	143	0	0	0	1	524	410	1557	322	1323	1596	1148	1863	24	177	53	160	1	0	0	0	392	645	336	1140	953	1347	982	1418
ENSG00000250541	0	3	0	0	0	0	0	0	0	2	4	0	6	2	4	6	1	0	0	5	0	0	0	0	0	2	2	9	2	6	7	4
ENSG00000250317	504	378	410	385	212	191	150	201	208	156	833	153	339	324	340	403	668	1020	615	677	517	197	398	225	205	332	208	533	374	344	363	418
ENSG00000251009	0	0	0	0	0	0	0	0	0	0	1	0	0	3	4	1	0	0	0	0	0	0	0	0	0	0	0	2	0	3	1	1
ENSG00000283314	0	0	0	0	0	0	0	0	0	0	0	0	2	0	0	0	0	0	0	0	0	0	0	0	0	0	1	0	0	0	0	0
ENSG00000248452	0	0	0	0	0	0	0	0	0	0	0	0	0	0	0	3	0	0	0	0	0	0	0	0	0	0	0	0	0	0	1	0
ENSG00000168214	5767	4724	4611	4548	3943	3926	2881	4308	2182	1855	7130	1220	4871	4867	4721	5884	2155	3078	2335	3211	5038	2023	3365	1893	1780	2510	1966	4960	2970	3745	3086	4073
ENSG00000163394	0	0	0	0	0	0	0	0	0	0	0	0	0	0	0	0	0	0	0	0	0	0	0	0	0	0	0	0	0	0	0	1
ENSG00000109680	480	255	427	255	90	92	68	124	115	91	338	53	166	207	204	278	307	420	462	397	194	85	155	95	97	109	104	227	123	214	232	187
ENSG00000200999	5	1	2	2	1	0	0	0	0	0	0	0	0	1	1	0	0	0	2	1	0	0	0	0	0	0	0	1	0	3	1	0
ENSG00000109689	1719	1311	1627	1432	675	741	586	727	791	675	3522	554	824	952	986	1231	852	1077	963	1395	1717	708	1321	631	935	1323	878	2448	953	1282	1028	1419
ENSG00000240005	8	7	6	5	4	1	1	1	1	1	6	4	4	2	3	3	3	3	5	9	1	2	2	0	3	2	4	3	2	1	3	2
ENSG00000248340	3	3	5	6	0	4	2	2	0	3	4	0	1	3	0	1	2	0	0	6	1	3	5	5	7	7	3	7	0	2	4	4
ENSG00000251080	0	0	0	0	0	0	0	0	0	0	0	0	0	0	0	0	0	0	0	0	0	0	0	0	0	1	0	0	0	0	0	0
ENSG00000205830	0	0	0	0	0	0	0	0	0	0	0	0	0	0	0	0	0	0	0	0	0	0	0	0	0	0	0	0	0	0	0	0
ENSG00000249699	0	0	0	0	0	0	0	0	0	0	0	0	0	0	0	0	0	0	0	0	0	0	0	0	0	0	0	0	0	0	0	0
ENSG00000222206	0	0	0	0	0	0	0	0	0	0	0	0	0	0	0	0	0	0	0	0	0	0	0	0	0	0	0	0	0	0	0	0
ENSG00000251325	0	0	0	0	0	0	0	0	0	0	0	0	0	0	0	0	0	0	0	0	0	0	0	0	0	0	0	0	0	0	0	0
ENSG00000250858	0	0	0	0	0	0	0	0	0	0	0	0	0	0	0	0	0	0	0	0	0	0	0	0	0	0	0	0	0	0	0	0
ENSG00000251410	2	0	0	0	0	1	0	0	0	0	0	0	0	1	1	0	0	0	0	0	0	0	0	0	0	0	0	0	0	0	0	0
ENSG00000250038	0	0	0	0	0	0	0	0	0	0	0	0	0	0	0	0	0	0	0	0	0	0	0	0	0	0	0	0	0	0	0	0
ENSG00000250064	0	0	0	0	0	0	0	0	0	0	1	0	0	0	0	2	0	0	0	0	0	0	0	0	0	0	0	0	0	1	0	0
ENSG00000243548	0	0	0	0	0	0	0	0	0	0	0	0	0	0	0	0	0	0	0	0	0	0	0	0	0	0	0	0	0	0	0	0
ENSG00000283275	0	0	0	0	0	0	0	0	0	0	0	0	0	0	0	0	0	0	0	0	0	0	0	0	0	0	0	0	0	0	1	0
ENSG00000250927	0	0	0	0	0	0	0	0	0	0	0	0	0	0	0	0	0	0	0	0	0	0	0	0	0	0	0	0	0	0	0	0
ENSG00000250190	0	0	0	0	0	0	0	0	0	0	0	0	0	0	0	0	0	0	0	0	0	0	0	0	0	0	0	0	0	0	0	0
ENSG00000276507	0	0	0	0	0	0	0	0	0	0	0	0	0	0	0	0	0	0	0	0	0	0	0	0	0	0	0	0	0	0	0	0
ENSG00000249228	0	0	0	0	0	0	0	0	0	0	0	0	0	0	0	0	0	0	0	0	0	0	0	0	0	0	0	0	0	0	0	0
ENSG00000248176	0	0	0	0	0	0	0	0	0	0	0	0	0	0	0	0	0	0	0	0	0	0	0	0	0	0	0	0	0	0	0	0
ENSG00000248338	0	0	0	0	0	0	0	0	0	0	1	0	0	0	0	0	0	0	0	0	0	0	0	0	0	0	0	0	0	0	0	0
ENSG00000251373	0	0	0	0	0	0	0	0	0	0	1	0	0	0	0	0	0	0	0	0	0	0	0	0	0	0	0	0	0	0	0	0
ENSG00000251113	0	0	0	0	0	0	0	0	0	0	0	0	0	0	0	0	0	0	0	0	0	0	0	0	0	0	0	0	0	0	0	0
ENSG00000283666	0	0	0	0	0	0	0	0	0	0	0	0	0	0	0	0	0	0	0	0	0	0	0	0	0	0	0	0	0	0	0	0
ENSG00000249542	0	0	0	0	0	0	0	0	0	0	0	0	0	0	0	0	0	0	0	0	0	0	0	0	0	0	0	0	0	1	0	0
ENSG00000249564	0	0	0	0	0	0	0	0	0	0	0	0	0	0	0	0	0	0	0	0	0	0	0	0	0	0	0	0	0	0	0	0
ENSG00000244031	0	0	0	0	0	0	0	0	0	0	0	0	0	0	0	0	0	0	0	0	0	0	0	0	0	0	0	0	0	0	0	0
ENSG00000248281	0	0	0	0	0	0	0	0	0	0	0	0	0	0	0	0	0	0	0	0	0	0	0	0	0	0	0	0	0	0	0	0
ENSG00000169851	0	1	1	0	1	0	0	0	527	419	1755	351	0	3	8	2	0	1	0	0	0	0	0	0	563	782	480	1357	0	7	5	10
ENSG00000249678	0	0	0	0	0	0	0	0	2	5	7	1	0	0	0	0	0	0	0	0	0	0	0	0	2	6	2	3	0	0	0	0
ENSG00000250624	0	0	0	0	0	0	0	0	0	0	0	0	0	0	0	0	0	0	0	0	0	0	0	0	0	2	5	1	0	0	0	0
ENSG00000251182	0	0	0	0	0	0	0	0	0	0	0	0	0	0	0	0	0	0	0	0	0	0	0	0	0	0	0	0	0	0	0	0
ENSG00000251434	0	0	0	0	0	0	0	0	0	0	0	0	0	0	0	1	0	0	0	0	0	0	0	0	0	0	1	0	0	0	0	0
ENSG00000249882	0	0	0	0	0	0	0	0	0	0	0	0	0	0	0	0	0	0	1	0	0	0	0	0	0	0	0	0	0	0	0	0
ENSG00000251129	0	0	0	0	0	0	0	0	0	0	3	0	5	2	3	9	0	0	0	0	1	0	1	1	0	0	1	0	1	8	3	1
ENSG00000251329	0	0	0	0	0	0	0	0	0	0	0	0	0	0	0	0	0	0	0	0	0	0	0	0	0	0	0	0	0	0	0	0
ENSG00000250076	0	0	0	0	0	0	0	0	0	0	0	0	0	1	0	0	0	0	0	0	0	0	0	0	0	0	0	0	0	0	0	0
ENSG00000249452	0	0	0	0	0	0	0	0	0	0	0	0	0	0	0	0	0	0	0	0	0	0	0	0	0	0	0	0	0	0	0	0
ENSG00000249766	0	0	0	0	0	0	0	0	0	0	0	0	0	0	0	0	0	0	0	0	0	0	0	0	0	0	0	0	0	0	0	0
ENSG00000248417	0	0	0	0	0	0	0	0	0	0	0	0	0	0	0	0	0	0	0	0	0	0	0	0	0	0	0	0	0	0	0	0
ENSG00000250954	0	0	0	0	0	0	0	0	0	0	8	0	2	4	3	3	0	0	0	0	0	0	0	0	0	0	3	3	0	5	0	8
ENSG00000250723	0	0	0	0	0	0	0	0	0	0	1	0	0	0	4	0	0	0	0	0	0	0	0	0	0	0	1	0	2	0	2	3
ENSG00000239983	0	0	0	0	0	0	0	0	0	0	0	0	0	1	0	0	0	0	0	0	0	0	0	0	0	0	0	0	0	0	1	1
ENSG00000248685	0	0	0	0	0	0	0	0	0	0	1	0	0	0	0	0	0	0	0	0	0	0	0	0	0	0	0	0	0	0	0	0
ENSG00000250597	0	0	0	0	0	0	0	0	0	0	0	0	0	0	0	0	0	0	0	0	0	0	0	0	0	0	0	0	0	0	1	0
ENSG00000275250	0	0	0	0	0	0	0	0	0	0	0	0	0	0	0	0	0	0	0	0	0	0	0	0	0	0	0	0	0	0	0	0
ENSG00000238694	0	0	0	0	0	0	0	0	0	0	0	0	0	0	0	0	0	0	0	0	0	0	0	0	0	0	0	0	0	0	0	0
ENSG00000250416	0	0	0	0	0	0	0	0	0	0	1	0	0	0	0	0	0	0	0	0	0	0	0	0	0	0	0	0	0	0	0	0
ENSG00000206665	0	0	0	0	0	0	0	0	0	0	0	0	0	0	0	0	0	0	0	0	0	0	0	0	0	0	0	0	0	0	0	0
ENSG00000047365	34	25	21	27	631	841	599	895	297	288	2643	365	1456	1315	1376	1667	16	33	42	59	1587	737	1227	723	323	490	270	1332	1086	1724	1444	1775
ENSG00000248466	0	0	0	0	2	2	0	1	0	0	4	0	2	3	1	3	0	0	0	0	4	0	2	1	1	5	0	6	0	5	3	3
ENSG00000247193	0	0	0	1	3	1	2	2	0	0	2	0	18	12	15	19	0	0	0	0	5	0	3	5	0	0	1	3	10	17	26	20
ENSG00000197057	0	0	0	0	4	4	2	4	2	0	26	1	38	47	41	60	0	2	0	0	5	2	8	2	0	1	1	8	59	85	83	93
ENSG00000251438	0	0	0	0	0	0	0	0	0	0	0	0	0	0	0	0	0	0	0	0	0	0	0	0	0	0	0	0	0	0	1	0
ENSG00000283207	0	0	0	0	0	0	0	0	0	0	0	0	0	0	0	0	0	0	0	0	0	0	0	0	0	0	0	0	0	0	0	0
ENSG00000248215	0	0	0	0	0	0	0	0	0	0	0	0	0	0	0	0	0	0	0	0	0	0	0	0	0	0	0	0	0	0	2	0
ENSG00000251588	0	0	0	0	0	0	0	0	0	0	0	0	0	0	0	0	0	0	0	0	0	0	0	0	0	0	0	0	0	0	0	0
ENSG00000261761	0	0	0	0	0	0	0	0	0	0	0	0	0	0	0	0	0	0	0	0	0	0	0	0	0	0	0	0	0	0	0	0
ENSG00000247810	0	0	0	0	0	0	0	0	0	0	1	0	0	0	0	0	0	0	0	0	0	0	0	0	0	0	0	0	0	0	0	0
ENSG00000264319	0	0	0	0	0	0	0	0	0	0	0	0	0	0	0	0	0	0	0	0	0	0	0	0	0	0	1	0	0	0	0	0
ENSG00000174145	1	2	3	0	0	0	0	0	0	0	0	0	0	0	0	0	1	0	0	2	0	1	0	0	0	0	0	1	0	0	0	0
ENSG00000154274	23	10	14	13	10	13	7	14	29	12	110	17	11	28	13	13	5	3	4	23	24	7	7	16	22	42	24	116	7	23	8	19
ENSG00000248936	12	2	10	8	3	5	4	2	11	5	80	4	4	8	5	2	1	0	1	3	7	3	4	10	11	12	17	44	2	10	5	4
ENSG00000181826	114	93	125	120	109	109	103	137	133	119	722	107	102	109	94	139	20	39	41	90	205	78	153	74	136	214	220	557	60	93	65	85
ENSG00000207075	0	0	0	0	0	0	0	0	0	0	0	0	0	0	0	0	0	0	0	0	0	0	0	0	0	0	0	0	0	0	0	0
ENSG00000239532	0	0	0	1	0	0	0	0	0	0	4	1	0	0	0	0	0	0	0	2	0	0	0	0	1	0	1	0	0	0	0	0
ENSG00000169299	1897	1331	1431	1378	1955	2100	1662	2313	1376	1035	4078	815	1946	1628	2140	2293	1145	2173	1819	1669	4228	1627	3180	1503	1507	2106	971	3251	1907	2167	2318	2239
ENSG00000279386	31	25	32	32	25	38	21	29	26	33	89	23	72	61	68	58	6	13	9	15	44	21	36	27	22	36	21	78	27	32	37	52
ENSG00000249863	95	77	55	83	75	89	61	85	77	68	245	35	172	166	145	144	24	20	22	57	103	49	78	37	91	104	49	132	75	99	59	103
ENSG00000065882	3388	2939	2773	3047	1321	1432	931	1528	1879	1617	5664	1200	1488	1653	1589	2052	877	1718	1403	2012	1779	730	1520	745	1631	2529	1176	4096	886	1305	1020	1428
ENSG00000250254	2	0	2	2	4	1	0	0	0	1	9	3	1	1	2	3	0	0	0	0	0	1	2	0	5	6	5	13	1	2	0	5
ENSG00000251332	12	5	4	7	2	2	3	2	6	4	10	4	2	1	2	1	2	1	3	8	3	2	1	2	5	6	1	7	1	3	2	5
ENSG00000249649	0	0	1	1	0	2	0	0	2	0	3	0	1	1	1	4	0	0	1	3	1	0	0	0	1	1	0	0	0	2	2	0
ENSG00000251642	0	0	0	0	0	0	0	0	0	0	0	0	0	0	0	0	0	0	0	0	0	0	0	0	0	0	0	0	0	0	0	0
ENSG00000248227	0	0	0	0	0	0	0	0	0	0	1	0	0	0	0	0	0	0	0	0	0	0	0	0	0	0	0	0	0	0	0	2
ENSG00000280015	0	0	0	0	0	0	0	0	0	0	0	0	0	0	0	0	0	0	0	0	0	0	0	0	0	0	0	0	0	0	0	0
ENSG00000249534	0	0	0	0	0	0	0	0	0	0	3	0	0	0	0	2	0	0	0	0	0	0	0	0	0	0	0	0	0	0	0	1
ENSG00000249667	0	0	0	0	0	0	0	0	0	0	0	0	0	0	0	2	0	0	0	0	0	0	0	0	0	0	0	0	0	0	0	0
ENSG00000251635	0	0	0	0	0	0	0	0	0	0	0	0	0	0	0	0	0	0	0	0	0	0	0	0	0	0	0	0	0	0	0	0
ENSG00000231160	0	1	0	3	14	14	7	26	19	15	61	16	14	18	10	21	0	0	0	4	12	2	8	4	17	13	26	26	9	14	9	21
ENSG00000271958	0	0	0	0	0	0	0	0	0	0	0	0	0	0	0	0	0	0	0	0	0	0	0	0	0	0	0	0	0	0	0	0
ENSG00000109787	1	11	1	15	185	230	143	217	351	371	1276	254	331	369	309	491	0	3	1	9	190	94	123	71	410	614	309	882	219	320	186	351
ENSG00000174123	0	0	0	0	0	0	0	0	0	0	0	0	2	2	13	5	0	0	0	0	1	0	0	0	0	1	0	0	2	4	5	4
ENSG00000174125	4	0	6	1	0	0	0	0	16	10	54	7	261	281	291	367	3	3	3	7	0	0	0	0	16	24	6	29	188	250	266	300
ENSG00000174130	4	2	7	0	0	5	3	6	5	2	25	4	68	79	52	121	3	12	8	11	13	2	3	2	3	8	4	15	22	82	52	97
ENSG00000197712	0	0	0	0	1	0	0	0	4	2	10	3	5	10	6	10	1	0	0	0	0	1	0	1	1	6	1	8	5	7	5	4
ENSG00000207944	0	0	0	0	0	0	0	0	0	0	0	0	0	0	0	0	0	0	0	0	0	0	0	0	0	0	0	0	0	0	0	0
ENSG00000121895	93	31	83	37	149	161	149	164	358	261	981	163	408	410	512	482	125	78	98	57	336	112	248	137	235	336	196	509	392	386	563	525
ENSG00000109790	442	365	352	374	1891	1901	1420	2018	1341	1210	4787	877	2632	2451	2943	3123	180	322	225	317	3164	1208	2217	1103	1361	2030	1077	3225	2269	2651	2768	2949
ENSG00000249207	11	5	2	3	14	12	5	10	15	16	67	10	15	17	20	17	2	1	2	4	22	22	17	9	25	19	11	40	7	15	7	23
ENSG00000249685	4	8	13	6	28	23	14	14	28	21	116	19	22	25	26	26	1	1	2	9	43	16	45	16	23	48	26	66	10	38	15	26
ENSG00000157796	1264	859	1022	813	235	293	175	320	415	396	1761	320	684	688	721	933	720	772	768	906	557	233	301	224	325	450	564	1064	473	706	707	901
ENSG00000035928	3842	2747	3428	2685	3163	3297	2838	3654	3121	2434	8996	1888	4139	3652	4394	4719	2475	3696	3652	2829	7801	2865	6401	2679	2673	3841	1901	7049	3424	3533	4494	4176
ENSG00000206675	4	6	3	1	3	4	5	3	3	3	11	2	5	5	1	4	0	2	4	2	10	2	2	6	5	5	1	17	3	8	1	4
ENSG00000222592	0	0	0	0	0	0	0	0	0	0	1	0	0	0	0	0	0	0	0	0	0	0	0	0	0	0	0	0	0	0	0	0
ENSG00000134962	12	5	10	5	7	11	6	8	13	3	34	6	21	20	20	28	3	2	1	16	21	13	7	15	23	14	8	33	22	31	31	41
ENSG00000264621	0	0	0	0	0	0	0	0	0	0	0	0	0	0	0	0	0	0	0	0	0	0	0	0	0	0	0	0	0	0	0	0
ENSG00000238797	0	0	0	0	0	0	0	0	0	0	0	0	0	0	0	0	0	0	0	0	0	0	0	0	0	0	0	0	0	0	0	0
ENSG00000163682	14771	9345	9790	9288	9523	9686	6624	10494	21488	15463	60205	12369	18252	16753	19826	22941	12896	13659	13639	14045	21568	8657	15562	10529	18313	24718	12748	36632	21440	19094	25747	24905
ENSG00000121897	435	328	343	279	311	399	285	374	320	283	1142	192	574	486	511	610	284	355	290	410	792	336	559	300	324	415	228	644	468	524	508	561
ENSG00000224097	4	11	4	4	6	4	11	10	10	13	33	6	5	2	8	8	0	7	5	6	27	1	14	4	11	9	9	20	2	7	11	5
ENSG00000109814	957	634	737	685	1086	1156	783	1249	1128	913	3709	714	1836	1627	2148	2145	283	495	409	575	1593	697	1033	550	1057	1454	843	2462	1510	1829	1892	1903
ENSG00000249348	15	5	8	10	18	26	15	17	50	42	191	26	39	41	35	32	4	0	4	5	19	9	21	13	37	44	41	88	14	19	16	29
ENSG00000163683	931	832	812	678	92	133	58	126	149	207	1283	166	2070	1807	2111	2409	366	563	472	395	128	42	108	43	179	371	542	975	1205	1427	1303	1414
ENSG00000252796	0	0	0	1	0	0	0	0	0	0	1	0	1	1	1	0	2	0	0	0	0	0	0	0	0	0	1	1	0	2	0	2
ENSG00000255458	35	18	28	14	3	4	4	8	3	8	23	1	5	7	9	6	11	7	13	21	4	4	6	0	2	2	6	17	0	5	9	5
ENSG00000078140	4553	3023	3667	3176	3863	3872	2947	4073	2588	2223	9255	1593	4736	4179	5175	5361	2908	3452	3375	4011	7748	3122	5428	2946	3030	4196	1953	6370	4670	5065	4797	5675
ENSG00000252975	11	6	3	1	5	5	4	9	9	5	17	0	4	4	7	3	3	5	2	4	6	3	4	6	5	9	3	21	2	3	6	10
ENSG00000249019	6	6	6	4	7	3	11	14	14	7	34	4	7	9	6	5	6	0	3	3	27	11	10	21	18	12	13	56	4	20	13	12
ENSG00000243260	15	6	13	13	11	7	7	16	17	3	27	4	5	14	5	9	0	3	4	5	12	8	2	10	14	16	9	26	3	19	20	14
ENSG00000180610	21	17	15	18	16	18	13	22	20	13	63	10	13	14	19	14	8	2	2	30	31	15	17	26	20	23	20	74	7	20	22	33
ENSG00000121892	8434	5421	6894	5435	6572	6666	5082	7022	5953	4857	21107	3790	7804	6898	7927	8438	4038	5774	5360	6174	13675	5216	9624	5221	6516	9545	4952	15955	6387	8068	7676	8768
ENSG00000271278	5	0	0	3	4	5	7	4	9	6	11	0	4	2	3	3	3	0	0	4	12	6	11	5	11	5	6	20	2	4	7	15
ENSG00000250568	5	3	0	3	1	0	2	4	3	4	25	0	2	1	4	5	1	0	4	2	7	6	6	4	1	8	1	10	0	2	3	7
ENSG00000231707	9	3	10	9	8	7	3	6	14	10	28	1	12	12	6	6	3	2	1	9	22	15	10	20	9	20	13	44	6	9	14	13
ENSG00000249064	0	4	0	0	0	0	0	0	6	1	7	1	1	0	0	1	0	0	0	0	0	1	0	0	0	0	0	9	0	0	0	0
ENSG00000205794	10	5	7	5	3	1	2	1	7	5	32	8	0	6	5	2	1	3	0	1	6	4	5	2	8	5	7	21	5	3	5	7
ENSG00000078177	3172	2188	2456	2071	1725	1853	1435	1888	2327	2148	10705	1689	1420	1390	1398	1775	1013	1341	1212	1932	3946	1635	2830	1464	2436	3434	2179	7431	941	1348	1246	1572
ENSG00000200455	2	3	3	4	3	2	1	0	3	0	9	0	1	0	0	4	0	0	3	4	1	2	3	0	1	1	7	9	0	1	0	0
ENSG00000201863	2	2	5	4	0	0	0	0	1	2	8	0	1	1	0	4	0	0	0	4	3	2	3	4	2	3	1	11	0	1	1	1
ENSG00000248977	1	1	1	2	0	0	1	0	1	1	5	0	1	0	1	3	0	0	0	2	2	0	0	5	1	2	2	2	0	0	2	1
ENSG00000260296	72	65	53	56	58	60	51	68	77	57	249	33	19	19	16	27	14	10	5	37	92	44	49	38	30	75	67	135	9	23	9	35
ENSG00000168421	2177	1778	2227	1720	1763	1546	1511	1745	591	451	2187	362	1400	1197	1498	1622	1530	2771	2364	1846	4014	1452	3650	1378	523	745	519	1368	1662	1514	1918	1815
ENSG00000250338	0	0	0	1	2	0	0	3	1	4	5	2	1	1	0	0	0	0	1	1	0	0	3	1	0	0	2	1	0	0	0	0
ENSG00000249241	4	2	4	2	0	0	0	0	0	0	5	0	1	3	3	2	6	1	9	1	2	2	3	2	0	0	1	1	1	3	2	4
ENSG00000174343	44	26	35	30	6	22	11	23	2	0	14	1	4	6	9	9	58	86	81	83	43	19	46	17	4	3	7	4	6	6	6	8
ENSG00000239010	0	0	0	0	0	0	0	0	0	0	0	0	0	0	0	0	0	0	0	0	0	0	0	0	0	0	0	0	0	0	0	0
ENSG00000163694	1	0	1	0	0	0	0	0	0	2	7	0	34	46	50	57	0	0	0	3	1	0	1	0	5	1	2	5	24	37	32	42
ENSG00000250893	0	0	0	0	0	0	0	0	0	0	0	0	0	0	0	0	0	0	0	0	0	0	1	0	0	0	0	0	0	0	0	0
ENSG00000242197	0	0	0	0	0	0	0	0	0	0	1	0	0	0	0	0	0	0	0	0	0	0	0	0	0	0	0	0	0	0	0	0
ENSG00000263642	0	0	0	0	0	0	0	0	0	0	0	0	0	0	0	0	0	0	0	0	0	0	0	0	0	0	0	0	0	0	0	0
ENSG00000179299	2	0	0	0	0	0	1	0	0	0	1	0	1	0	0	0	0	0	0	1	1	0	0	0	0	0	0	0	0	0	0	0
ENSG00000248780	0	0	0	0	0	0	0	0	0	0	0	0	0	0	0	0	0	0	0	1	0	0	0	0	0	0	0	0	0	0	0	0
ENSG00000163697	771	827	604	824	185	266	123	238	31	52	78	33	59	106	48	111	164	476	230	651	153	100	126	90	16	30	22	79	40	109	63	134
ENSG00000250906	0	3	7	1	0	0	1	3	0	2	0	0	0	0	0	0	0	0	0	2	0	0	0	0	0	0	0	0	0	1	0	1
ENSG00000207195	0	0	0	0	0	1	0	1	0	0	2	0	0	1	0	0	0	0	0	0	0	0	0	0	0	0	0	0	0	0	0	0
ENSG00000199790	0	1	0	0	0	0	0	0	0	0	0	2	0	0	0	1	1	0	0	0	0	0	0	0	0	0	0	0	0	0	0	1
ENSG00000207198	1	0	0	0	0	0	0	0	0	0	0	0	0	0	0	0	0	0	0	1	0	0	0	0	0	0	0	0	0	0	0	0
ENSG00000251173	0	0	0	0	0	0	0	0	0	0	0	0	0	0	0	0	0	0	0	0	0	0	0	0	0	0	0	0	0	0	0	0
ENSG00000154277	0	0	0	0	0	0	0	0	31	50	73	42	0	0	0	0	0	0	0	0	0	0	0	0	43	177	45	186	0	0	0	0

ENSG00000207481	0	0	0	0	0	0	0	0	0	0	0	0	0	0	0	0	0	0	0	0	0	0	0	0	0	0	0	0	0	0	0	0
ENSG00000064042	0	6	0	3	0	0	0	0	0	0	7	1	0	0	1	2	0	5	1	4	0	0	0	0	1	1	2	0	0	2	2	2
ENSG00000239627	0	0	0	0	0	0	0	0	0	0	0	0	0	0	0	0	0	0	0	0	0	0	0	0	0	0	0	0	0	0	0	0
ENSG00000249216	0	0	0	0	0	0	0	0	0	0	0	0	0	0	0	0	0	0	0	0	0	0	0	0	0	0	0	0	0	0	0	0
ENSG00000249081	0	0	0	0	0	0	0	1	0	0	0	0	0	0	0	0	0	0	0	0	0	0	0	0	0	0	0	1	0	0	0	0
ENSG00000109132	0	0	0	0	0	0	0	0	0	0	0	0	0	0	0	0	0	0	2	0	0	0	0	0	0	0	1	0	0	0	0	0
ENSG00000250467	0	0	0	0	0	0	0	0	0	0	0	0	0	0	0	0	0	0	0	0	0	0	0	0	0	0	0	0	0	0	0	0
ENSG00000249122	0	0	0	0	0	0	0	0	0	0	0	0	0	0	0	0	0	0	0	0	0	0	0	0	0	0	0	0	0	0	0	0
ENSG00000200338	0	0	0	0	0	0	0	0	0	0	0	0	0	0	0	0	0	0	0	0	0	0	0	0	0	0	0	0	0	0	0	0
ENSG00000249745	0	0	0	0	0	0	0	0	0	0	0	0	0	0	0	0	0	0	0	0	0	0	0	0	1	0	0	0	0	0	0	0
ENSG00000245870	0	0	0	0	0	0	0	0	0	0	1	0	0	0	0	0	0	0	0	0	0	0	0	0	0	1	0	0	0	0	0	0
ENSG00000249771	0	0	0	0	0	0	0	0	0	0	0	0	0	0	0	1	0	0	0	0	0	0	0	0	0	0	2	0	0	0	0	0
ENSG00000249887	0	0	0	0	0	0	0	0	0	0	0	0	0	0	0	0	0	0	0	1	0	0	0	0	0	0	0	0	0	0	0	0
ENSG00000109133	3588	2392	2826	2454	1846	1955	1483	2062	1547	1239	6663	951	4585	3612	4798	4517	2480	3421	2526	3471	4646	1779	2813	1644	2083	2910	1378	4663	4236	4848	5101	5332
ENSG00000182308	75	33	33	45	41	16	15	16	11	12	75	9	32	31	43	38	30	25	31	76	42	30	27	20	15	37	19	44	14	75	45	76
ENSG00000272862	6	1	2	4	0	3	0	0	1	1	5	0	5	0	0	12	2	1	3	3	3	0	4	0	0	2	0	4	1	0	0	1
ENSG00000014824	2037	1395	1729	1439	945	1040	854	1195	1588	1251	5556	854	1886	1753	2058	2380	1649	2317	2114	1816	2640	1051	1943	1010	1464	2119	1330	3520	2023	2209	2426	2756
ENSG00000249212	9	6	11	9	2	3	3	3	4	4	15	0	6	9	5	6	2	3	1	9	8	3	3	10	5	9	8	22	3	5	15	4
ENSG00000188848	982	961	875	1089	402	495	349	550	720	790	2791	552	778	892	715	1176	273	488	371	857	657	312	510	287	731	1139	501	1575	396	800	449	841
ENSG00000250781	0	0	0	0	0	0	0	1	0	0	1	0	0	0	0	0	0	0	0	1	0	1	0	0	0	0	1	0	0	0	0	0
ENSG00000178343	0	2	0	5	0	1	0	0	111	114	363	96	15	32	15	23	0	1	0	2	1	0	0	0	130	206	53	347	4	11	13	23
ENSG00000124406	1559	1405	1099	1302	3347	3283	2613	3520	1643	1435	6866	1023	1699	1772	1732	2272	643	1058	560	1340	7282	3064	4824	3182	1343	1819	1688	3364	849	1554	1171	1686
ENSG00000240167	1	1	1	0	1	2	1	2	0	1	6	0	0	1	2	2	0	0	0	3	4	2	2	3	1	2	1	3	0	0	4	2
ENSG00000248283	5	10	4	5	25	20	9	19	8	2	11	2	7	7	1	12	3	2	0	12	40	20	18	15	2	5	8	24	4	4	6	7
ENSG00000260519	0	0	0	0	0	0	1	2	0	0	4	0	0	0	0	0	0	0	0	0	3	0	2	5	0	1	0	0	1	0	1	4
ENSG00000251517	2	0	0	0	3	2	9	3	1	0	6	0	4	2	7	15	0	0	1	0	2	3	1	3	2	1	1	1	6	4	6	9
ENSG00000252595	0	0	0	0	0	0	0	0	0	0	0	0	0	0	0	0	0	0	0	0	0	0	0	0	0	0	0	0	0	0	0	0
ENSG00000215203	0	0	0	0	0	0	0	0	0	0	0	0	1	0	1	1	0	0	0	0	0	0	0	0	0	0	1	0	0	1	0	1
ENSG00000248939	0	0	0	0	0	0	0	0	0	0	0	0	0	0	0	0	0	0	0	0	0	0	0	0	0	0	0	0	0	0	1	0
ENSG00000250657	0	0	0	0	0	0	0	0	0	0	0	0	0	0	0	0	0	0	0	0	0	0	0	0	0	0	0	0	0	0	0	0
ENSG00000277096	0	0	0	0	0	0	0	0	0	0	0	0	0	0	0	0	0	0	0	0	0	0	0	0	0	0	0	0	0	0	0	0
ENSG00000213851	0	0	1	0	0	0	0	0	0	0	0	0	0	0	0	0	0	0	1	0	0	0	0	0	0	0	0	0	0	0	0	0
ENSG00000248143	0	0	0	0	0	0	0	0	0	0	0	0	0	0	0	0	0	0	0	0	0	0	0	0	0	0	0	0	0	0	0	0
ENSG00000251501	0	0	0	0	0	0	0	0	0	0	0	0	0	0	0	0	0	0	0	0	0	0	0	0	0	0	0	0	0	0	0	0
ENSG00000239464	0	0	0	0	0	0	0	0	0	0	0	0	0	0	0	0	0	0	0	0	0	0	0	0	0	0	0	0	0	0	0	0
ENSG00000239679	0	0	0	0	0	0	0	0	0	0	0	0	0	0	0	0	0	0	0	0	0	0	0	0	0	0	0	0	0	0	0	0
ENSG00000124399	2	0	0	0	0	1	0	1	0	0	1	0	1	0	0	0	0	0	1	0	1	0	2	2	0	0	0	0	0	0	0	0
ENSG00000250863	0	0	0	0	0	0	0	0	0	0	0	0	0	0	0	0	0	0	0	0	0	0	0	0	0	0	0	0	0	0	0	0
ENSG00000251350	0	0	0	0	0	0	0	0	54	43	131	35	27	59	31	59	0	0	0	0	0	1	0	0	48	65	21	116	15	16	10	13
ENSG00000183783	0	0	0	0	0	0	0	0	0	0	0	0	0	0	0	0	0	0	0	0	0	0	0	0	0	0	0	0	0	0	0	0
ENSG00000251159	0	0	0	0	0	0	0	0	2	0	0	0	0	0	0	0	0	0	0	0	0	0	0	0	0	0	0	0	0	0	0	0
ENSG00000177752	5	0	1	2	0	3	1	1	2	5	6	3	2	1	2	0	0	1	0	6	4	3	0	0	1	3	2	1	0	0	0	0
ENSG00000151806	1705	1239	1333	1168	1390	1453	1136	1597	1097	869	3425	613	3137	2734	3175	3427	689	1018	960	1196	3050	1228	2206	1233	852	1293	793	2032	2289	2972	2420	3167
ENSG00000163281	1502	975	1014	937	899	879	660	968	664	535	2356	425	670	640	633	801	1172	1655	1373	1597	1921	739	1187	898	656	920	595	1595	566	769	710	873
ENSG00000273369	45	37	40	40	50	49	47	38	36	27	110	27	70	56	53	82	18	17	12	52	92	40	43	53	24	44	25	81	32	61	25	60
ENSG00000272936	0	0	0	0	0	0	0	0	0	0	0	0	0	0	0	0	0	0	0	0	0	0	0	0	0	0	0	0	0	0	0	0
ENSG00000249729	0	0	0	0	0	0	0	0	0	0	0	0	0	0	0	1	0	0	0	0	0	0	0	0	0	0	0	0	0	0	0	0
ENSG00000248987	0	0	0	0	0	0	0	0	0	0	1	0	0	0	0	0	0	0	0	1	0	0	0	0	0	0	0	0	0	0	0	0
ENSG00000275959	0	0	0	0	0	0	0	0	0	0	0	0	0	0	0	0	0	0	0	0	0	0	0	0	0	0	0	0	0	0	0	0
ENSG00000248744	0	0	0	0	0	0	0	0	0	0	0	0	0	0	0	0	0	0	0	0	0	0	0	0	0	0	0	0	0	0	0	0
ENSG00000248320	0	0	0	0	0	0	0	0	0	1	0	0	0	0	0	0	0	0	0	0	0	0	0	0	0	0	0	0	0	0	0	0
ENSG00000224560	0	0	0	0	0	0	0	0	0	0	1	0	0	0	0	0	0	0	0	0	0	0	0	0	0	0	0	0	0	0	0	0
ENSG00000200720	0	0	0	0	0	0	0	0	0	0	0	0	0	0	0	0	0	0	0	0	0	0	0	0	0	0	0	0	0	0	0	0
ENSG00000222257	0	0	0	0	0	0	0	0	0	0	0	0	0	0	0	0	0	0	0	0	0	0	0	0	0	0	0	0	0	0	0	0
ENSG00000163285	0	0	0	0	1	0	0	0	0	0	1	0	0	0	0	0	0	2	0	0	1	0	0	0	0	0	0	0	0	0	0	0
ENSG00000260878	0	0	0	0	0	0	0	0	0	0	1	0	0	0	0	0	0	0	0	0	0	0	0	0	0	0	0	0	0	0	0	0
ENSG00000151834	0	0	0	0	0	0	0	0	11	14	8	6	0	1	0	0	0	0	0	0	0	0	0	0	2	5	4	7	0	0	1	0
ENSG00000249330	0	0	0	0	0	0	0	0	0	0	0	1	0	0	0	0	0	0	0	0	0	0	0	0	0	0	0	0	0	0	0	0
ENSG00000252243	0	0	0	0	0	0	0	0	0	0	0	0	0	0	0	0	0	0	0	0	0	0	0	0	0	0	0	0	0	0	0	0
ENSG00000249936	5	0	4	0	6	7	4	2	2	1	6	1	1	0	1	1	1	2	2	3	12	8	7	4	3	4	1	2	0	3	2	1
ENSG00000170516	0	0	0	0	0	0	0	0	0	0	0	0	0	0	0	0	0	0	0	0	0	0	0	0	0	0	0	0	0	0	0	0
ENSG00000109158	0	0	0	0	0	1	1	0	0	1	0	0	0	0	0	0	1	0	0	0	0	0	0	1	0	1	0	1	0	0	1	0
ENSG00000163288	1	0	0	0	0	0	0	0	0	1	1	0	0	0	0	0	0	0	0	0	0	0	0	0	0	1	0	0	0	0	0	0
ENSG00000260918	1	1	3	2	0	0	2	1	0	0	0	0	0	2	1	0	0	2	2	3	0	0	0	3	0	0	0	0	0	1	2	2
ENSG00000169019	748	524	595	470	556	595	427	616	368	293	1687	275	641	612	637	810	1222	1492	1312	1178	1472	611	1030	829	463	543	372	1075	800	792	844	930
ENSG00000282904	3	2	0	0	0	0	0	0	0	0	0	0	0	0	1	0	2	0	1	1	2	0	0	0	0	1	0	0	0	0	0	0
ENSG00000145246	95	76	96	64	241	293	185	369	299	321	2795	363	107	147	150	177	64	88	136	133	339	148	255	152	335	570	740	1665	62	141	112	170
ENSG00000242431	0	0	0	1	0	0	0	2	0	0	5	3	0	1	1	0	0	0	0	1	0	0	0	0	2	1	2	8	0	1	1	1
ENSG00000248254	1	0	0	1	3	3	1	6	9	4	26	7	0	2	4	3	2	0	2	5	6	3	3	1	9	2	13	18	0	2	0	2
ENSG00000145244	12	9	3	13	22	17	11	22	14	20	31	15	33	30	20	22	2	3	2	10	16	11	15	15	5	12	4	18	8	22	11	22
ENSG00000277388	0	0	0	0	0	0	1	0	0	0	0	0	0	0	0	0	0	0	0	1	0	0	0	0	0	0	0	0	0	0	0	0
ENSG00000242262	1	0	1	0	1	0	0	0	1	0	1	1	0	0	0	1	0	1	2	0	0	1	0	0	0	4	0	1	0	0	0	0
ENSG00000282917	2	1	6	1	8	2	4	2	15	8	15	2	6	9	9	9	0	2	1	4	10	3	4	4	6	8	1	7	6	4	7	7
ENSG00000259959	246	177	203	161	201	192	158	202	183	127	437	80	224	189	164	234	63	91	71	175	319	139	202	163	121	179	79	251	121	217	131	230
ENSG00000170448	886	669	801	688	723	739	667	869	419	379	1512	269	1280	1098	1455	1385	440	669	638	921	1676	665	1269	718	446	640	370	1015	975	1267	1301	1533
ENSG00000163293	4	8	9	5	9	13	11	4	12	7	36	10	30	29	44	54	2	5	8	5	22	12	20	13	14	11	6	32	29	50	50	66
ENSG00000198515	0	5	1	4	1	1	0	0	3	1	12	1	8	9	15	8	0	2	3	1	1	1	4	1	7	7	0	14	7	6	17	17
ENSG00000074966	0	0	0	0	188	223	188	200	1333	1068	4306	715	79	105	83	114	1	1	0	1	451	190	251	174	483	807	750	2266	88	159	95	159
ENSG00000200269	0	0	0	0	0	0	0	0	7	2	34	9	0	1	1	0	0	0	0	1	6	3	3	4	3	3	2	8	0	0	0	2
ENSG00000283499	0	0	0	0	0	1	2	1	3	5	22	3	0	0	1	2	0	0	0	0	1	0	1	1	3	1	1	10	0	1	0	4
ENSG00000135605	11	9	10	15	983	991	783	1043	127	77	208	50	360	282	341	400	4	6	1	8	1715	765	1130	758	44	75	31	138	266	369	292	442
ENSG00000202014	0	0	0	0	0	3	5	2	1	0	1	2	4	2	0	3	0	0	0	0	3	3	1	12	0	0	0	4	1	3	1	3
ENSG00000109171	1682	1191	1455	1212	1799	1814	1335	1859	1139	912	4357	728	1301	1143	1353	1532	469	792	742	1101	2790	1117	2026	1086	1310	2141	993	3265	1044	1331	1182	1453
ENSG00000145248	12	13	3	9	0	4	1	0	2	2	5	2	0	4	2	2	1	3	1	4	1	2	2	1	5	0	3	10	0	2	2	2
ENSG00000182223	1	1	1	0	2	1	2	0	0	0	4	1	1	1	0	1	0	0	0	5	3	2	0	1	0	3	0	1	0	0	0	0
ENSG00000075539	5477	4249	5090	4041	3218	3454	2388	3553	2709	2413	11144	1992	3167	3122	3328	3744	1500	1816	2098	3120	5456	2520	3768	2326	3046	4349	2773	8826	2057	3118	2715	3381
ENSG00000251722	1	0	0	0	0	1	0	0	0	0	0	0	0	0	0	0	0	0	0	3	0	0	0	1	0	1	0	2	0	0	2	1
ENSG00000109180	2606	1676	2049	1668	2691	2789	2154	2931	2190	1751	7733	1218	2588	2530	2831	3192	1672	2134	2230	2325	6090	2380	4196	2432	1962	2788	1856	4826	2328	2960	3085	3379
ENSG00000248256	12	3	12	14	12	15	16	16	9	8	36	4	8	5	8	11	4	6	3	14	21	16	21	15	12	16	12	24	1	11	9	18
ENSG00000145247	1549	1113	1449	1125	856	1001	641	982	860	639	3138	552	1630	1506	1708	2034	2044	2564	2659	2006	2329	932	1575	1002	875	1180	794	1881	2310	1944	2629	2485
ENSG00000252913	1	0	0	0	0	0	0	1	0	0	0	0	0	0	0	0	0	0	0	0	0	0	0	0	0	0	0	0	0	0	2	0
ENSG00000251334	0	0	0	0	0	0	0	0	0	0	0	0	0	0	0	0	0	0	0	0	0	0	0	0	0	0	0	0	0	0	0	0
ENSG00000109182	0	0	0	0	0	0	0	0	0	0	1	0	0	0	2	0	0	0	0	0	0	0	0	0	0	0	0	0	0	0	2	0
ENSG00000225455	0	0	0	0	0	0	0	0	0	0	0	0	0	0	0	0	0	0	0	0	0	0	0	0	0	0	0	0	0	0	0	0
ENSG00000249828	0	0	0	0	0	0	0	0	0	0	0	0	0	0	0	0	0	0	0	0	0	0	0	0	0	0	0	0	0	0	0	0
ENSG00000249079	0	0	0	0	0	0	0	0	2	0	5	0	0	0	0	0	0	0	0	0	0	0	0	0	0	0	0	1	0	0	0	0
ENSG00000280043	0	0	0	0	0	0	0	0	2	0	6	0	0	0	0	0	0	0	0	0	0	2	0	0	1	0	0	3	0	0	0	0
ENSG00000250016	0	0	0	0	0	0	0	0	0	0	0	0	0	0	0	0	0	0	0	0	0	0	0	0	0	0	0	0	0	0	0	0
ENSG00000237961	0	0	0	0	0	0	0	0	2	2	8	0	0	0	0	0	0	0	0	0	0	0	1	0	0	1	0	3	0	0	1	0
ENSG00000268736	0	0	0	0	0	0	0	0	0	0	0	0	0	0	0	0	0	0	0	0	0	0	0	0	0	0	0	0	0	0	0	0
ENSG00000248946	0	0	0	0	0	0	0	0	0	0	0	0	0	0	0	0	0	0	0	0	0	0	0	0	0	0	0	0	0	0	0	0
ENSG00000248583	0	0	0	0	0	0	0	0	1	0	12	0	0	0	0	0	0	0	0	0	0	0	0	0	3	0	0	7	0	0	0	0
ENSG00000251056	0	0	0	0	0	0	0	0	3	2	3	0	1	0	0	0	0	0	0	0	0	0	0	0	0	0	0	3	0	0	0	0
ENSG00000250769	0	0	0	0	0	0	0	0	0	0	0	0	0	0	0	0	0	0	0	0	0	0	0	0	0	0	0	0	0	0	0	0
ENSG00000248532	0	0	0	0	0	0	0	0	0	0	0	0	0	0	0	0	0	0	0	0	0	0	0	0	0	0	0	0	0	0	0	0
ENSG00000269848	0	0	0	0	0	0	0	0	0	0	0	0	0	0	0	0	0	0	0	0	0	0	0	0	0	0	0	0	0	0	0	0
ENSG00000268967	0	0	0	0	0	0	0	0	0	0	0	0	0	0	0	0	0	0	0	0	0	0	0	0	0	0	0	0	0	0	0	0
ENSG00000268391	0	0	0	0	0	0	0	0	0	0	0	0	0	0	0	0	0	0	0	0	0	0	0	0	0	0	0	0	0	0	0	0
ENSG00000232471	0	0	0	0	0	0	0	0	1	3	5	1	0	0	0	0	0	0	0	0	0	0	0	0	0	0	1	3	0	0	0	0
ENSG00000251630	0	0	0	0	0	0	0	0	0	0	77	3	0	0	0	0	0	0	0	0	0	0	0	0	0	0	0	0	0	0	0	0
ENSG00000251539	0	0	1	0	0	0	0	0	0	0	0	0	0	0	0	0	0	0	0	0	0	0	0	0	0	0	0	1	0	0	0	0
ENSG00000250753	0	0	0	0	0	0	0	0	0	0	1	1	0	0	0	0	0	0	0	0	0	0	0	0	0	1	1	0	0	0	0	0
ENSG00000249337	0	0	0	0	0	0	0	0	0	0	0	0	0	0	0	0	0	0	0	0	0	0	0	0	0	0	0	0	0	0	0	0
ENSG00000109184	1098	761	945	791	1370	1543	1154	1545	1192	935	3384	630	932	872	854	1037	692	800	712	960	2755	1169	1878	1336	961	1271	813	2276	579	799	665	881
ENSG00000250473	10	3	3	3	5	12	3	5	2	4	12	2	6	2	3	5	2	6	1	11	11	5	10	9	7	6	2	17	0	5	3	8
ENSG00000272576	2	4	1	1	6	6	8	6	9	4	22	4	1	3	5	2	2	4	5	6	15	5	12	5	4	3	5	9	3	7	0	6
ENSG00000243929	0	0	0	0	0	0	0	0	0	0	0	0	0	0	0	0	0	0	0	0	0	0	0	0	0	0	0	0	0	0	0	0
ENSG00000188993	13	25	19	15	4	4	4	10	7	6	31	7	0	2	1	2	11	6	14	31	9	1	6	3	6	3	13	9	0	7	0	6
ENSG00000163069	645	558	596	614	391	460	376	535	554	507	2490	429	229	340	278	482	368	496	524	630	776	300	615	290	619	827	669	1595	245	451	329	482
ENSG00000251040	1	0	0	0	0	0	0	0	0	0	0	0	0	0	0	0	0	0	0	0	0	0	0	0	0	0	0	0	0	0	0	0
ENSG00000163071	0	0	0	0	1	0	0	0	0	0	0	0	0	0	0	0	0	0	0	0	0	0	0	0	0	0	0	6	0	1	0	0
ENSG00000251286	0	0	0	0	0	0	0	0	0	0	0	0	0	0	0	0	0	0	0	0	0	0	0	0	0	0	1	0	0	0	0	0
ENSG00000199924	0	0	0	0	0	0	0	0	0	0	0	0	0	0	0	0	0	0	0	0	0	0	0	0	0	0	0	0	0	0	0	0
ENSG00000109189	650	521	610	551	961	955	776	1053	423	319	2056	281	192	173	181	241	253	519	441	673	2012	782	1249	740	517	684	528	1280	186	314	227	279
ENSG00000248866	26	25	25	39	8	19	4	22	46	29	144	33	15	10	10	11	19	26	28	50	23	15	11	18	29	43	36	105	8	10	14	11
ENSG00000260120	0	0	0	2	0	0	0	0	0	0	1	0	0	0	0	0	0	0	0	1	0	1	0	0	1	0	0	0	0	0	0	0
ENSG00000226950	1301	877	1131	998	785	684	577	809	1003	748	3107	493	613	535	781	820	2402	2906	3012	2539	2105	772	1523	846	1040	1422	513	1616	1057	1024	1367	1384
[truncated: 6,993,636 more chars]
