# Supplementary material for: SAM-Competitive EZH2-Inhibitors Induce Platinum Resistance by EZH2-Independent Induction of ABC-Transporters
Source: Cancers (Basel). 2023 Jun 3;15(11):3043. doi: 10.3390/cancers15113043 (PMC10252553; doi:10.3390/cancers15113043)
Supplement: Supplementary file 1 [file cancers-15-03043-s001.zip › cancers-2406836-supplementary.pdf]

Supplementary Tables

**Supplementary Table S1 Origin, RRID and seeding densities for cell viability assay of used cell lines.** Cell lines were obtained from German Collection of Microorganisms and Cell Cultures (DSMZ) or American Type Culture Collection (ATCC).

| Cell Line | Source | Cat # | Date of Acquisition | RRID | Density |
|---|---|---|---|---|---|
| Jurkat | DSMZ | ACC 282 | 2019/06/13 | RRID:CVCL_0065 | 10k |
| SUP-T11 | DSMZ | ACC 605 | 2018 | RRID:CVCL_2210 | 15k |
| Loucy | ATCC | CRL-2629 | 2019 | RRID:CVCL_1380 | 10k |
| DERL-2 | DSMZ | ACC 531 | 2017/01/18 | RRID:CVCL_2016 | 75k |
| HH | DSMZ | ACC 707 | 2020 | RRID:CVCL_1280 | 5k |
| SR786 | | | 2019/01/30 | RRID:CVCL_1711 | 20k |
| Daudi | DSMZ | ACC 78 | 2020/09/09 | RRID:CVCL_0008 | 7k |
| Oci-Ly1 | DSMZ | ACC 722 | 2020/09/09 | RRID:CVCL_1879 | 20k |

**Supplementary Table S2 Primer sequences for EZH2 knockdown.**

| shRNA | Sigma-Aldrich TRC-Clone ID | Target Sequence |
|---|---|---|
| HH EZH2 KD1 | TRCN0000040077 | CCCAACATAGATGGACCAAAT |
| HH EZH2 KD2 | TRCN0000353069 | TATGATGGTTAACGGTGATCA |
| HH mock shRNA | SHC002 | CAACAAGATGAAGAGCACCAA |
| Jurkat EZH2 KD1 | TRCN0000040075 | CCAACACAAGTCATCCCATTA |
| Jurkat EZH2 KD2 | TRCN0000353069 | TATGATGGTTAACGGTGATCA |
| Jurkat mock shRNA | SHC002 | CAACAAGATGAAGAGCACCAA |

**Supplementary Table S3 RT-qPCR primer sequences.**

| Target | 5'-forward Primer-3' | 5'-reverse Primer-3' |
|---|---|---|
| ABCG1 | CGCTTTCTCGGTCGGCA | CTGGGCTTCCGTGAGGTTAT |
| ABCG2 | TCAGGAGGCCTTGGGATACT | AGGCTCTATGATCTCTGTGGC |
| EZH1 | AACCCAACACTTCCCCTTGC | ACTGAACAGGTTGGACACGA |
| EZH2 | GCAATTATTCTTTTCATGCAACACC | TTGGTGGGGTCTTTATCCGC |
| SREBF1 | AGGGCGGGCGCAGAT | GGTTGTTGATAAGCTGAAGCATGT |
| SREBF2 | GGCTGAAGAATAGGAGTTGCC | AACGGTCATTCACCCAGGTC |
| TBP | AGGAGCCAAGAGTGAAGAACAG | AGGAGAACAATTCTGGGTTTGA |

**Supplementary Table S4 Mutation and expression status of used cell lines.** Data was acquired from Cosmic [accessed 2022/06/03] for Jurkat, Loucy, HH, SR786, and Daudi, from DepMap [accessed 2022/09/20] for SUPT11 and DERL-2, and from Morin *et al.* and Wright *et al.* for Oci-Ly1 [35, 50-53].

| **Background** | | **Jurkat** | **SUPT11** | **Loucy** |
|---|---|---|---|---|
| T-cell origin, immature | | Cortical T-ALL | ETP-ALL | ETP-ALL |
| PRC2 Complex | EZH2 | wt,<br>high expression | wt,<br>wt expression | wt,<br>silenced |
| | EZH1 | p.R557H,<br>low expression | wt,<br>wt expression | wt,<br>wt expression |
| | H3K27me3 | wt trimethylation | wt trimethylation | no trimethylation |
| | EED | wt | wt | wt |
| | SUZ12 | p.R516H<br>p.Q737Tfs*20 | wt | wt |
| | RbAp48 | p.A294T | wt | wt |
| DNA Damage Response | Tp53 | p.R196* | p.R213* | p.V272M |
| | p21 | wt | wt | wt |
| | ATM | wt | wt | wt |
| | ATR | p.R1631H<br>p.I1264M | wt | wt |
| | MDM2 | wt | wt | wt |
| Ras/Raf | SRC | wt | wt | wt |
| | Nras | wt | wt | wt |
| | Nras1 | wt | wt | wt |
| | Raf1 | p.A237T | wt | wt |
| | Araf | wt | p.A475P | wt |
| | Braf | p.A728V | wt | wt |
| Apoptosis Suppression | Casp2 | wt | wt | wt |
| | Casp3 | p.E190D | wt | wt |
| | Casp6 | wt | wt | wt |
| | Casp7 | wt | wt | wt |
| | Casp8 | p.L107R | wt | wt |
| | Casp9 | wt | wt | wt |
| | Casp10 | p.V33Sfs*12 | wt | wt |
| | LMNA | p.R166W<br>p.Q293*<br>p.R388H<br>p.R482Q | wt | wt |
| | LMNB1 | wt | wt | wt |
| | LMNB2 | wt | wt | wt |
| | ICAD | wt | wt | wt |
| | PARP1 | wt | wt | wt |
| | Pak2 | p.S508N | wt | p.S508N |
| ABC Transporters | ABCG1 | p.D587N<br>p.D587N | wt | wt |
| | ABCG2 | wt | wt | wt |
| | ABCB1 | wt | wt | wt |
| | ABCC1 | wt | wt | wt |
| | ABCC2 | p.H823Y | wt | wt |
| | ABCC3 | p.Q614Rfs*34 | wt | wt |
| | ABCC4 | wt | wt | wt |
| | ABCC5 | wt | wt | wt |
| | ABCC6 | wt | wt | wt |
| | ABCC10 | wt | wt | wt |
| SREBF | SREBF1 | wt | wt | wt |
| | SREBF2 | p.R896C<br>p.E328Kfs*26 | wt | wt |

| **Background** | | **DERL-2** | **HH** | **SR786** |
|---|---|---|---|---|
| | | | | |
| T-cell origin, mature | | HSTCL | CTCL | ALK+ ALCL |
| PRC2 Complex | EZH2 | wt,<br>high expression | wt,<br>wt expression | wt,<br>wt expression |
| | EZH1 | wt,<br>high expression | wt,<br>high expression | wt,<br>high expression |
| | H3K27me3 | no trimethylation | wt trimethylation | wt trimethylation |
| | EED | wt | wt | wt |
| | SUZ12 | wt | wt | wt |
| | RbAp48 | wt | wt | wt |
| DNA Damage Response | Tp53 | wt | c.560-1G>A | p.P152L |
| | p21 | wt | wt | wt |
| | ATM | wt | wt | wt |
| | ATR | wt | wt | wt |
| | MDM2 | wt | wt | wt |
| Ras/Raf | SRC | wt | wt | wt |
| | Nras | wt | wt | wt |
| | Nras1 | wt | wt | wt |
| | Raf1 | wt | wt | wt |
| | Araf | wt | wt | wt |
| | Braf | wt | wt | wt |
| Apoptosis Suppression | Casp2 | wt | wt | wt |
| | Casp3 | wt | wt | wt |
| | Casp6 | wt | wt | wt |
| | Casp7 | wt | wt | wt |
| | Casp8 | wt | wt | wt |
| | Casp9 | wt | wt | wt |
| | Casp10 | wt | wt | wt |
| | LMNA | wt | wt | wt |
| | LMNB1 | wt | wt | wt |
| | LMNB2 | wt | wt | wt |
| | ICAD | wt | wt | wt |
| | PARP1 | wt | wt | wt |
| | Pak2 | wt | wt | wt |
| ABC Transporters | ABCG1 | wt | wt | wt |
| | ABCG2 | wt | wt | wt |
| | ABCB1 | wt | wt | wt |
| | ABCC1 | wt | wt | wt |
| | ABCC2 | wt | wt | wt |
| | ABCC3 | wt | wt | wt |
| | ABCC4 | wt | wt | wt |
| | ABCC5 | wt | wt | wt |
| | ABCC6 | wt | wt | wt |
| | ABCC10 | wt | wt | wt |
| SREBF | SREBF1 | wt | wt | wt |
| | SREBF2 | wt | p.P870R<br>p.D582N | wt |

| **Background** | | **Oci-Ly1** | **Daudi** |
|---|---|---|---|
| B-cell origin, mature | | GC-DLBCL | Burkitt Lymphoma |
| PRC2 Complex | EZH2 | p.Y641N, high expression | wt, wt expression |
| | EZH1 | wt, wt expression | wt, high expression |
| | H3K27me3 | high trimethylation | wt trimethylation |
| | EED | wt | wt |
| | SUZ12 | wt | wt |
| | RbAp48 | wt | wt |
| DNA Damage Response | Tp53 | p.R158H | p.G266E |
| | p21 | wt | wt |
| | ATM | wt | wt |

| | | | |
|---|---|---|---|
| | ATR | wt | p.F2427L |
| | MDM2 | wt | wt |
| Ras/Raf | SRC | wt | wt |
| | Nras | wt | wt |
| | Nras1 | wt | wt |
| | Raf1 | wt | wt |
| | Araf | wt | wt |
| | Braf | wt | wt |
| Apoptosis Suppression | Casp2 | wt | wt |
| | Casp3 | wt | wt |
| | Casp6 | wt | wt |
| | Casp7 | wt | wt |
| | Casp8 | wt | wt |
| | Casp9 | wt | wt |
| | Casp10 | wt | wt |
| | LMNA | wt | wt |
| | LMNB1 | wt | wt |
| | LMNB2 | wt | wt |
| | ICAD | wt | wt |
| | PARP1 | wt | wt |
| | Pak2 | wt | wt |
| ABC Transporters | ABCG1 | wt | wt |
| | ABCG2 | wt | wt |
| | ABCB1 | wt | wt |
| | ABCC1 | wt | wt |
| | ABCC2 | wt | wt |
| | ABCC3 | wt | wt |
| | ABCC4 | E936Q | wt |
| | ABCC5 | wt | p.S523T<br>p.V476F |
| | ABCC6 | wt | wt |
| | ABCC10 | wt | wt |
| SREBF | SREBF1 | wt | wt |
| | SREBF2 | wt | wt |

Abbreviations: T-ALL – T-cell acute lymphoblastic leukemia: ETP-ALL – early T-cell precursor acute lymphoblastic leukemia; HSTCL – hepatosplenic T-cell lymphoma; CTCL – cutaneous T-cell lymphoma; ALK+ ALCL – anaplastic large cell lymphoma, ALK-positive; GC-DLBCL – diffuse large B-cell lymphoma, germinal center-like.

Supplementary Figures

Supplementary Figure S1

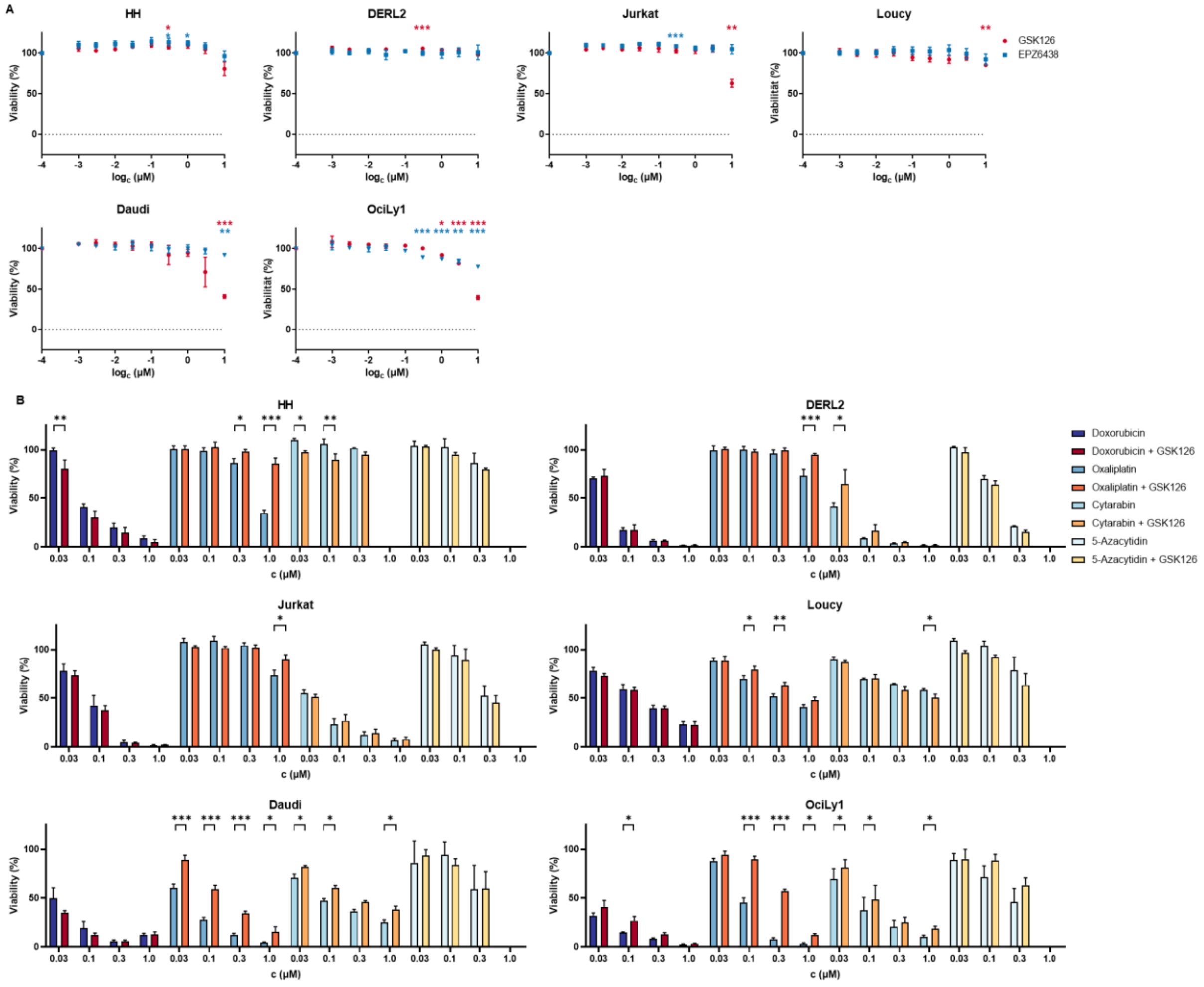


**Supplementary Figure S1 SAM-competitive EZH2 inhibitors are not sufficient as single agent but modify chemotherapy sensitivity. A** Both GSK126 and EPZ6438 were evaluated for reduction of cell viability at clinically relevant concentrations after 72 h of incubation in cell viability assays. Significant differences between the untreated control and under GSK126 (red) or EPZ6438 (blue) incubation are indicated by the respective coloured asterisks. Each point represents mean ± SEM, n=3. **B** In a screening approach, sensitivity towards the respective chemotherapeutic as single agent or in combination with GSK126 was evaluated. Each bar represents the mean ± SEM, n=3 (* p≤0.05; ** p≤0.005; *** p≤0.001).

Supplementary Figure S2

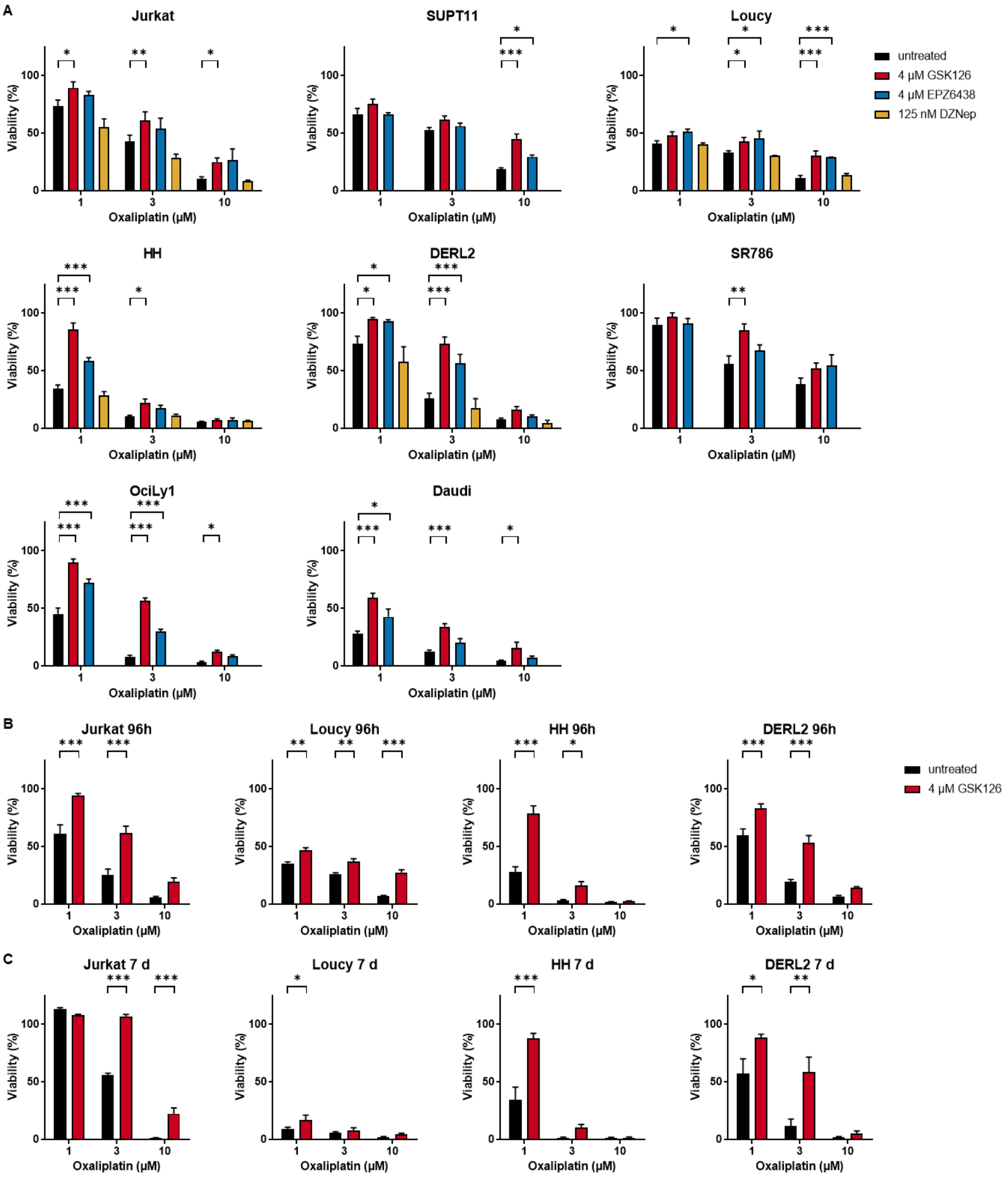


**Supplementary Figure S2 SAM-competitive EZH2 inhibitors increase oxaliplatin resistance significantly. A** Both GSK126 and EPZ6438 were able to induce oxaliplatin resistance in a broad panel of cell lines after 72 h of incubation in oxaliplatin cell viability assays. Oxaliplatin resistance persisted in a representative subset of cell lines after (**B**) 96 h and (**C**) 7 d of combinational incubation with GSK126. Each bar represents the mean ± SEM, n=3 (* p≤0.05; ** p≤0.005; *** p≤0.001).

Supplementary Figure S3

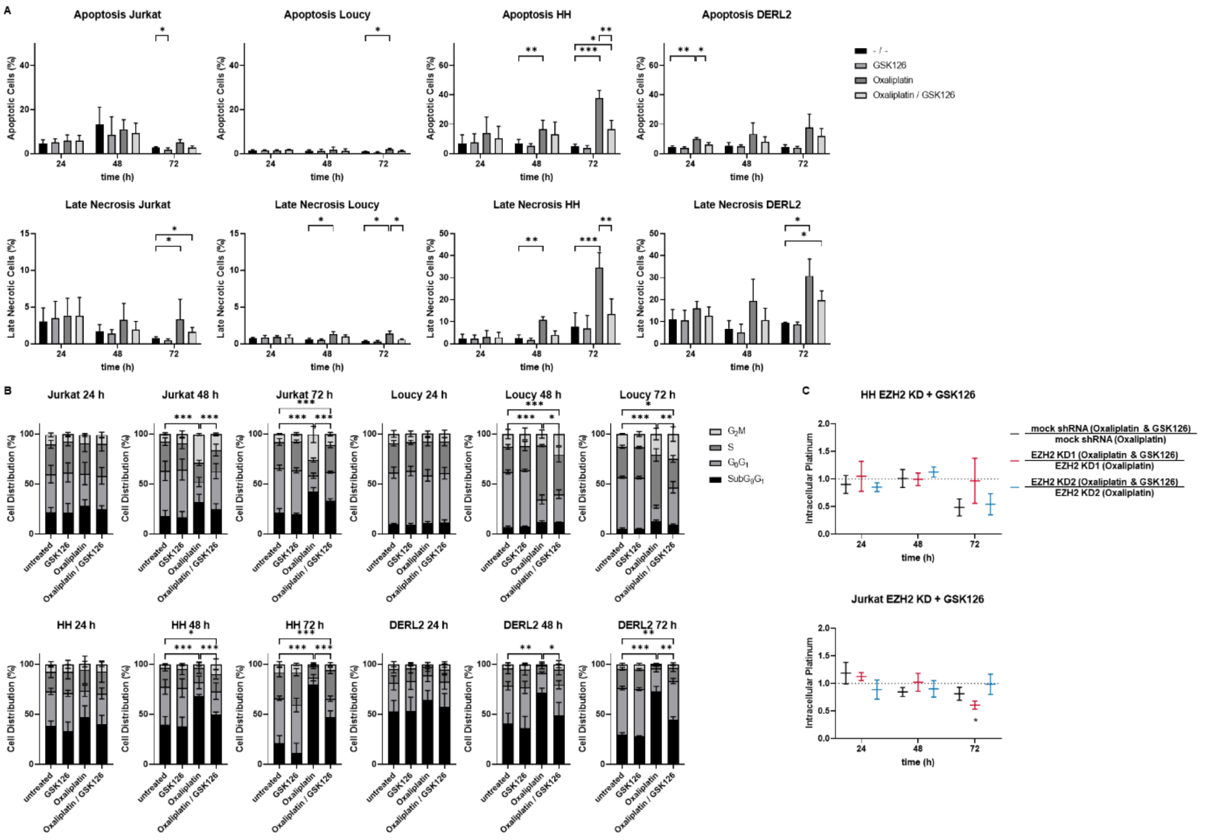


**Supplementary Figure S3 Mechanistic changes under pharmacological EZH2 inhibition or EZH2 knockdown.** Pharmacological EZH2 Inhibition reduces the oxaliplatin induced (**A**) apoptosis and necrosis as well as (**B**) cell cycle deregulation. Each bar represents the mean ± SD, n=3. **C** In ICP-MS, the intracellular platinum content was reduced by pharmacological EZH2 inhibition even under EZH2 knockdown. The values were normed to the respective shRNA cell line oxaliplatin monotherapy. Each line represents the mean ± SEM, n=3 (* $p \leq 0.05$; ** $p \leq 0.005$; *** $p \leq 0.001$).

Supplementary Figure S4

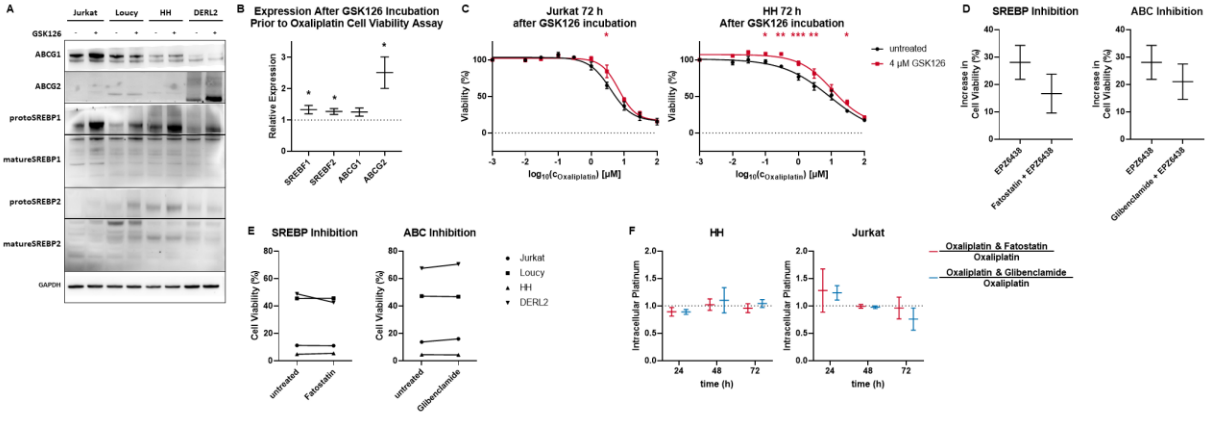


**Supplementary Figure S4 RNA sequencing validation on protein level, sequential pharmacological EZH2 inhibition and SREBP and ABC inhibition validation.** **A** Proteins found to be differentially expressed by pharmacological EZH2 inhibition were validated by westernblot. **B** Pharmacological EZH2 inhibition by GSK126 was confirmed by upregulation of SRE binding proteins and ABC transporters after 72 h of treatment. Each line represents the mean of the exemplary cell lines Jurkat and HH ± SEM, n=3 per cell line. C Oxaliplatin cell viability assay after pharmacological EZH2 inhibition by GSK126 treatment again was able to induce oxaliplatin resistance after 72 h of sequential incubation. Each point represents the mean ± SEM, n=3. **D** In oxaliplatin viability assay, reduction of oxaliplatin resistance by inhibition of SRE binding proteins or ABC transporters was confirmed by pharmacological EZH2 inhibition by EPZ6438 in combined analysis. For comparison the oxaliplatin concentration of the maximal induced resistance by pharmacological EZH2 inhibition of each respective cell line was chosen. Each line represents the mean of the exemplary cell lines Jurkat, Loucy and HH ± SEM, n=3 per cell line. **E** In oxaliplatin cell viability assays, neither Fatostatin nor Glibenclamide were able to alter oxaliplatin sensitivity. For comparison the oxaliplatin concentration of the maximal induced resistance by pharmacological EZH2 inhibition of each respective cell line was chosen. Each point represents the mean of n=3. **F** The intracellular platinum content, measured by ICP-MS, was not altered by Fatostatin or Glibenclamide. For comparison, values of combination approaches were normed to the respective oxaliplatin monotherapy control. Each line represents the mean ± SEM, n=3.

Supplementary Figure S5

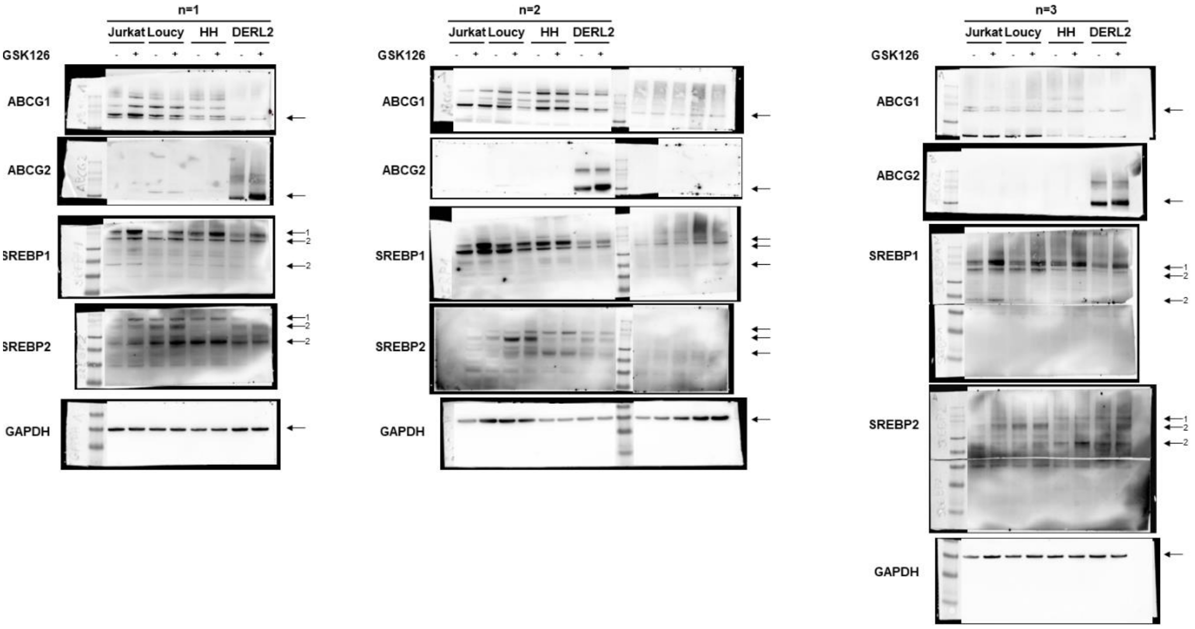


**Supplementary Figure S5 Original westernblot data of RNA sequencing data validation.** Arrows indicate the correct protein band. Probes that were not related to the validation are not labelled. Arrow specifications: 1 – protoSREBP1/2. 2 – matureSREBP1/2.

Supplementary Figure S6

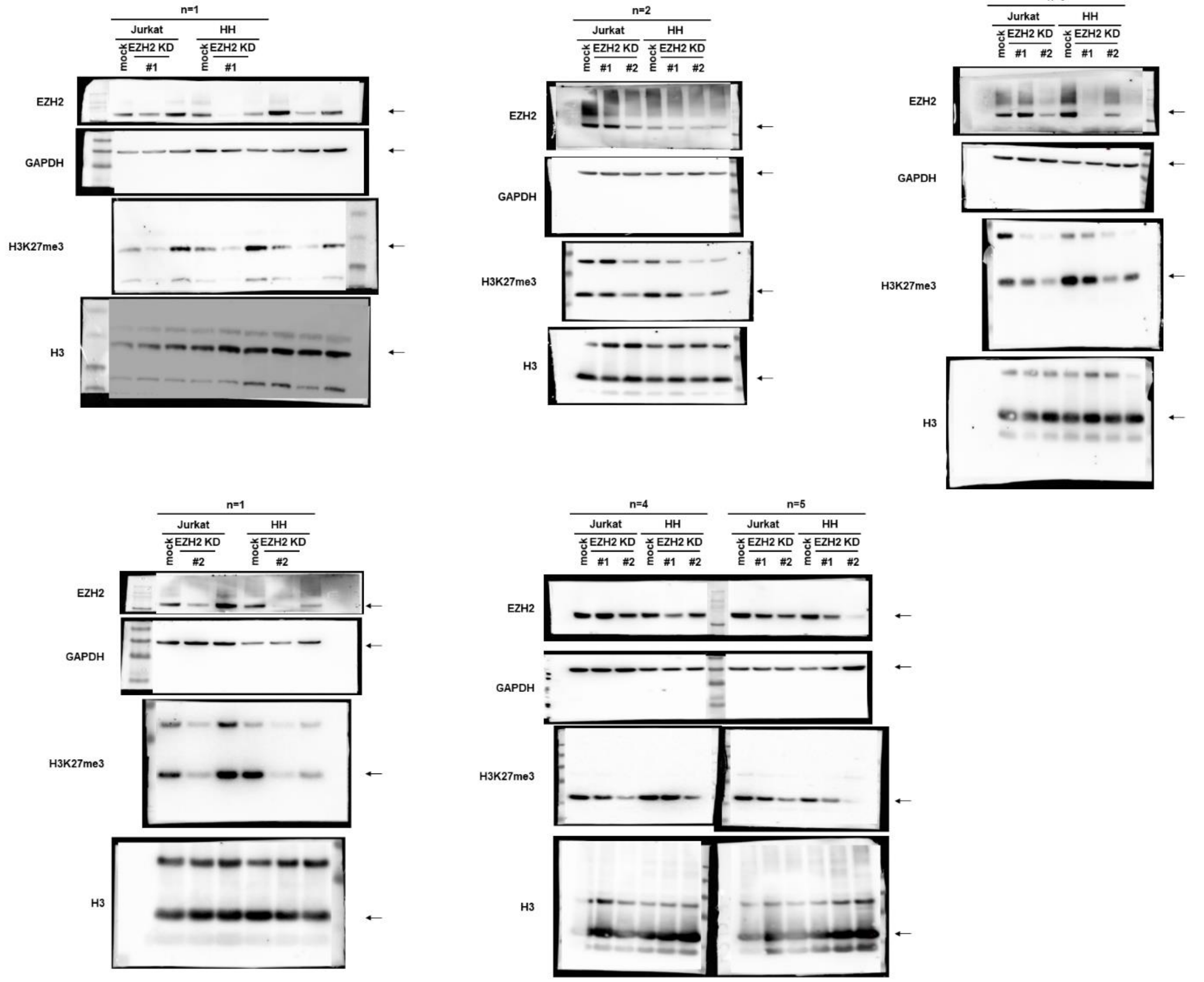


**Supplementary Figure S6 Original westernblot data of RNA sequencing data validation.** Arrows indicate the correct protein band. Probes of EZH2 knockdown cell lines that were not further used are not labelled.

Supplementary Figure S7

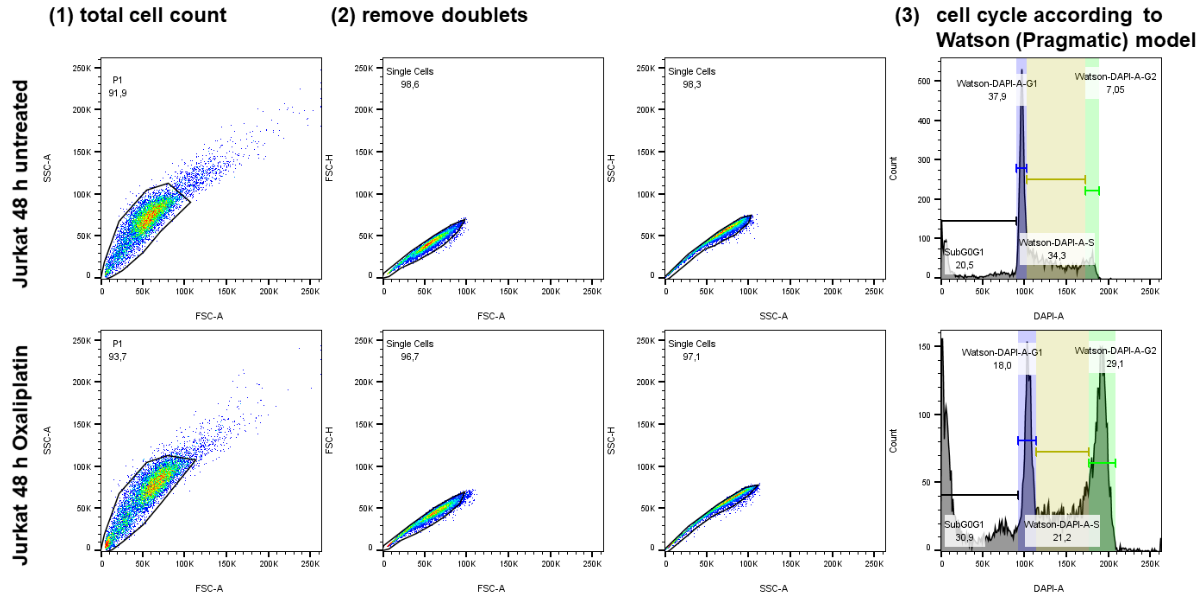


**Supplementary Figure S7 Exemplary gating strategy for cell cycle analysis.** The data is shown for the samples Jurkat untreated and Jurkat oxaliplatin treated, each after 48 h of incubation.

Supplementary Figure S8

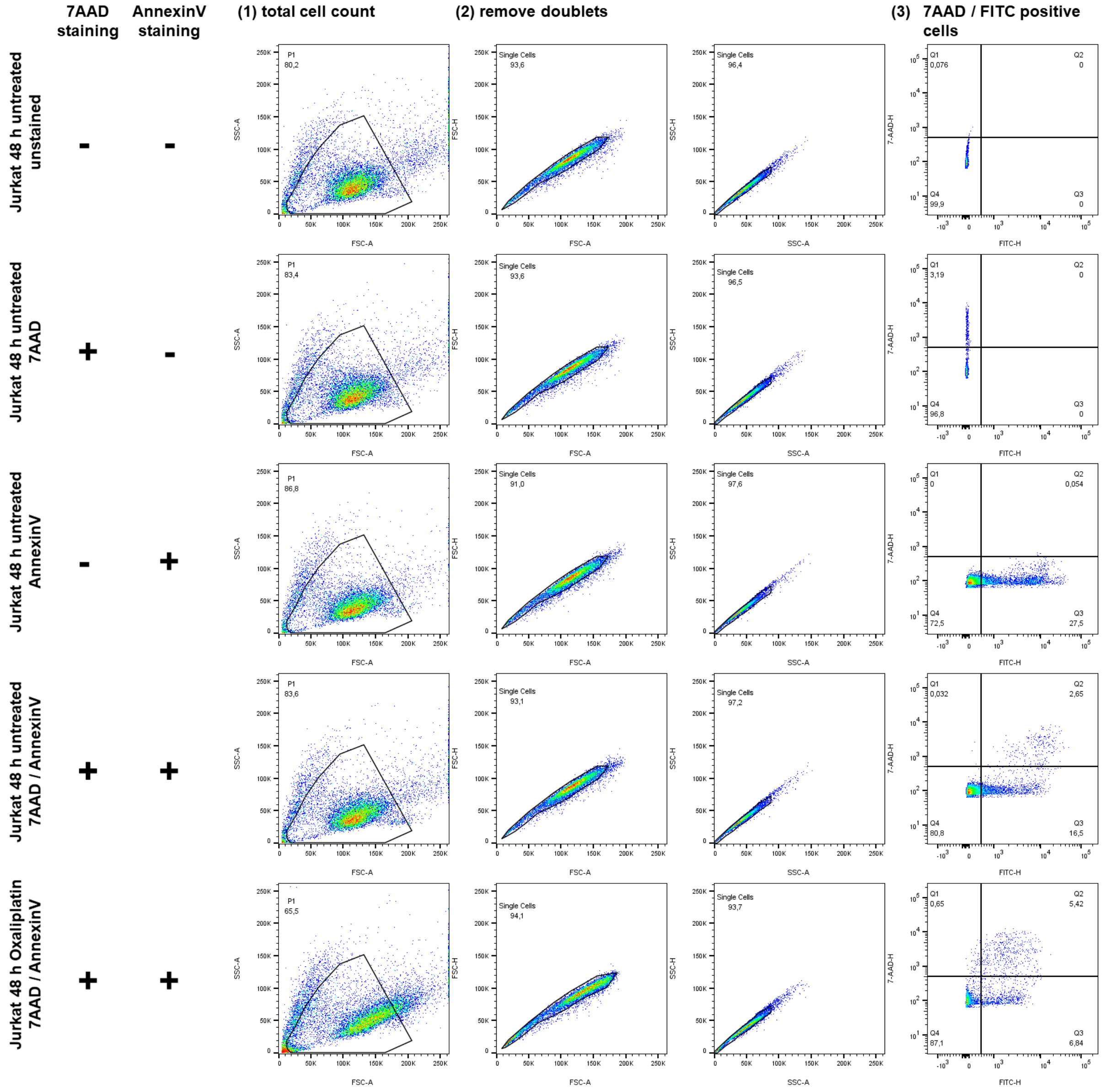


**Supplementary Figure S8 Exemplary gating strategy for cell cycle analysis.** The data is shown for the samples Jurkat untreated and Jurkat oxaliplatin treated, each after 48 h of incubation. 7AAD- and AnnexinV-positivity was examined per cell line by evaluating unstained control, 7AAD-stained control, and AnnexinV-stained control. The according gates were used for each sample of the respective cell line of the experiment.

Supplementary References

35. Morin, R.D.; Johnson, N.A.; Severson, T.M.; Mungall, A.J.; An, J.; Goya, R.; Paul, J.E.; Boyle, M.; Woolcock, B.W.; Kuchenbauer, F.; et al. Somatic mutations altering EZH2 (Tyr641) in follicular and diffuse large B-cell lymphomas of germinal-center origin. Nat. Genet. 2010, 42, 181–185.

50. Tate, J.G.; Bamford, S.; Jubb, H.C.; Sondka, Z.; Beare, D.M.; Bindal, N.; Boutselakis, H.; Cole, C.G.; Creatore, C.; Dawson, E.; et al. COSMIC: The Catalogue of Somatic Mutations in Cancer. Nucleic Acids Res. 2019, 47, D941–D947.

51. Barretina, J.; Caponigro, G.; Stransky, N.; Venkatesan, K.; Margolin, A.A.; Kim, S.; Wilson, C.J.; Lehár, J.; Kryukov, G.V.; Sonkin, D.; et al. The Cancer Cell Line Encyclopedia enables predictive modelling of anticancer drug sensitivity. Nature 2012, 483, 603–607.

52. Morin, R.; Mungall, K.; Pleasance, E.; Mungall, A.; Goya, R.; Huff, R.; Scott, D.W.; Ding, J.; Roth, A.; Chiu, R.; et al. Mutational and structural analysis of diffuse large B-cell lymphoma using whole-genome sequencing. Blood 2013, 122, 1256–1265.

53. Wright, G.W.; Huang, D.W.; Phelan, J.D.; Coulibaly, Z.A.; Roulland, S.; Young, R.M.; Wang, J.Q.; Schmitz, R.; Morin, R.D.; Tang, J.; et al. A Probabilistic Classification Tool for Genetic Subtypes of Diffuse Large B Cell Lymphoma with Therapeutic Implications. Cancer Cell 2020, 37, 551–568.e14.
